# Systematic review with meta-analysis of the epidemiological evidence relating smoking to COPD, chronic bronchitis and emphysema

**DOI:** 10.1186/1471-2466-11-36

**Published:** 2011-06-14

**Authors:** Barbara A Forey, Alison J Thornton, Peter N Lee

**Affiliations:** 1 P N Lee Statistics and Computing Ltd, Sutton, Surrey, UK; 2 Independent consultant, Exeter, Devon, UK

## Abstract

**Background:**

Smoking is a known cause of the outcomes COPD, chronic bronchitis (CB) and emphysema, but no previous systematic review exists. We summarize evidence for various smoking indices.

**Methods:**

Based on MEDLINE searches and other sources we obtained papers published to 2006 describing epidemiological studies relating incidence or prevalence of these outcomes to smoking. Studies in children or adolescents, or in populations at high respiratory disease risk or with co-existing diseases were excluded. Study-specific data were extracted on design, exposures and outcomes considered, and confounder adjustment. For each outcome RRs/ORs and 95% CIs were extracted for ever, current and ex smoking and various dose response indices, and meta-analyses and meta-regressions conducted to determine how relationships were modified by various study and RR characteristics.

**Results:**

Of 218 studies identified, 133 provide data for COPD, 101 for CB and 28 for emphysema. RR estimates are markedly heterogeneous. Based on random-effects meta-analyses of most-adjusted RR/ORs, estimates are elevated for ever smoking (COPD 2.89, CI 2.63-3.17, n = 129 RRs; CB 2.69, 2.50-2.90, n = 114; emphysema 4.51, 3.38-6.02, n = 28), current smoking (COPD 3.51, 3.08-3.99; CB 3.41, 3.13-3.72; emphysema 4.87, 2.83-8.41) and ex smoking (COPD 2.35, 2.11-2.63; CB 1.63, 1.50-1.78; emphysema 3.52, 2.51-4.94). For COPD, RRs are higher for males, for studies conducted in North America, for cigarette smoking rather than any product smoking, and where the unexposed base is never smoking any product, and are markedly lower when asthma is included in the COPD definition. Variations by sex, continent, smoking product and unexposed group are in the same direction for CB, but less clearly demonstrated. For all outcomes RRs are higher when based on mortality, and for COPD are markedly lower when based on lung function. For all outcomes, risk increases with amount smoked and pack-years. Limited data show risk decreases with increasing starting age for COPD and CB and with increasing quitting duration for COPD. No clear relationship is seen with duration of smoking.

**Conclusions:**

The results confirm and quantify the causal relationships with smoking.

## Background

It has been known for many years that smoking causes chronic obstructive pulmonary disease (COPD). In 1984, the US Surgeon General [1] concluded that, in the United States, 80 to 90% of morbidity from COPD is attributable to cigarette smoking. However, we know of no previous systematic review quantifying this relationship by meta-analysis, and we attempt to rectify this omission. It is recognized [1] that COPD comprises three separate, often interconnected disease processes: (1) airway thickening and narrowing with expiratory airflow obstruction; (2) chronic mucus hypersecretion, resulting in chronic cough and phlegm production; and (3) emphysema, an abnormal dilation of distal airspaces combined with destruction of alveolar walls. The present review considers all three processes by summarizing the epidemiological evidence relating smoking separately to the incidence or prevalence of COPD, chronic bronchitis (CB) and emphysema. Elsewhere [2], we systematically review evidence on the relationship between smoking and decline in forced expiratory volume in one second (FEV_1_).

Because COPD is rarely seen in children or adolescents, we restrict attention to adults. We also limit attention to studies of the general population, so do not, for example, consider studies in subjects suffering from alpha-1 antitrypsin deficiency or exposed to particular respiratory hazards. To provide a broad description of the relationship, we do not concentrate on one primary analysis, but quantify the relationship of each of the three outcomes studied (COPD, CB, emphysema) to each of a range of indices of smoking, investigating how these relationships vary according to characteristics such as sex, age, location, study design, period considered, definition of outcome, definition of exposure and extent of confounder adjustment.

## Methods

Full details of the methods used are described in Additional file 1, and are summarized below.

### Inclusion and exclusion criteria

Attention was restricted to epidemiological studies published before 2007 on COPD, CB or emphysema, providing relative risk (RR) estimates for one or more defined "major indices" (ever, current or ex smoking compared with never smoking) or "dose-related indices" (amount smoked, age of starting to smoke, pack-years smoked, duration of smoking or duration of quitting). Throughout this paper, we use the term RR to include its various estimators, including the odds ratio and the hazard ratio.

Studies were excluded if in children or adolescents, or in subjects at especially high risk of respiratory disease (e.g. workers in risky occupations), selected as having co-existing diseases or conditions, or from atypical populations likely to have a highly unusual prevalence of smoking or disease. Also excluded were uncontrolled case studies, and studies of disease exacerbation or undiagnosed disease, of symptom-free subjects, or where the only results were adjusted for symptoms or precursors of disease.

### Definition of the outcomes

#### COPD

The term COPD is quite recent, so studies with outcomes described otherwise were also included. These could be based on International Classification of Diseases (ICD) codes, on lung function criteria, on a combination of lung function criteria and symptoms, or on combinations of diagnosed conditions (such as CB or emphysema, or CB, emphysema or asthma), where diagnoses were extracted from medical records or reported in questionnaires. Unacceptable outcomes included CB or emphysema separately, acute or unspecified bronchitis, non-specific respiratory disease, or outcomes based only on symptoms and not on lung function. The range of ICD codes had to cover both CB and emphysema, and could also cover asthma, acute and unqualified bronchitis, bronchiectasis and some other defined lung conditions. Broader-ranging definitions (e.g. respiratory disease) were not accepted. Acceptable lung function criteria included those of the Global Initiative for Chronic Obstructive Lung Disease (GOLD) [3,4], the British Thoracic Society (BTS) [5], the European Respiratory Society (ERS) [6] and the American Thoracic Society (ATS) [7-9]. Use of a bronchodilator was not a requirement.

#### CB

Where based on the ICD, the range had to include the code(s) for CB and could also include codes for acute or unspecified bronchitis. Acceptable outcomes could also be based on medical records, in-study diagnosis, self-report of physician diagnosis or of history of the disease, or on symptoms. The British Medical Research Council (MRC) criterion of daily productive cough for at least three consecutive months for more than two successive years [10,11] was recognized as a set of symptoms defining CB. Diagnoses or symptoms called "bronchitis" were accepted where the context clearly indicated it was chronic. Diagnoses based on symptoms not referred to as CB were also accepted, provided the definition included both chronic cough and phlegm.

#### Emphysema

The outcome could be based on the ICD code for emphysema, on medical records, in-study diagnosis, or on self-report of physician diagnosis or history of the disease.

#### Choice of outcome

Where a study provided data for multiple acceptable definitions of an outcome, results were entered only for one. Additional file 1 gives the rules specifying choice of outcome, and, for studies providing a choice, lists definitions selected and rejected. It also gives, for all studies, the description of the disease and the source of the diagnosis for all outcomes where data were entered.

### Literature searching

Searching was carried out in phases. Initially, 1407 potentially relevant papers, published up to 2002, were derived by AJT from an unpublished project which used the MeSH terms chronic bronchitis and symptoms, emphysema, lung function, genetic determinants, mortality, adults and smoking. Subsequently, additional Medline searches were conducted in 2006 by AJT and in 2008 by BAF, using the MeSH term "Pulmonary disease, chronic obstructive". Papers were also sought from in-house files on smoking and health, and references cited in papers obtained. Publications before 2007 were considered, with no restriction on language or on peer-reviewed journals. Reasons for rejection were recorded.

### Identification of studies

Relevant papers were allocated to studies, noting multiple papers on the same study, and papers reporting on multiple studies. Each study was given a unique reference code (REF) of up to 6 characters (e.g. DICKIN or CHEN3), based on the principal author's name, and distinguishing multiple studies by the same author. Occasionally, an original study was split into separate studies (e.g. where follow-up periods differed by sex).

Some studies were noted as having overlaps or links with other studies. To minimize problems in meta-analysis arising from double-counting of cases, these links were divided into three types, as shown in Additional file 2. The first involved no such double-counting, while the second included studies with minor overlap, which could not be disentangled, and which it was decided to ignore. The third type contains sets of studies which probably or definitely overlap. Here the set member containing the most valuable data (e.g. largest study size or longest follow-up) was called the 'principal study', other members being 'subsidiary studies' only considered in meta-analyses where the required RR was unavailable from the principal study.

### Data recorded

For each study, relevant information was entered onto a study database and a linked RR database. The study database contains a record for each study, describing relevant publications, sexes considered, age range, location, timing, length of follow-up, whether principal or subsidiary, overlaps or links with other studies, study design, populations studied, major study weaknesses, outcome definitions, numbers of cases and subjects, types of controls and matching factors used in case-control studies, confounding variables, and availability of results for each smoking index. The RR database holds the detailed results, typically containing multiple records for each study. Each record is linked to the relevant study and refers to a specific RR, recording the comparison made and the results. This record includes the outcome, the sex and the analysis type (prevalence or incidence). Smoking exposure is defined by status (ever, current or ex), product (any, cigarettes, cigarettes only) and similar information about the unexposed base. For dose-related indices, the level of exposure is recorded. The source of the RR is also recorded, as are details on adjustment variables. Results recorded include numbers of exposed and unexposed cases, and, for unadjusted results, numbers of exposed and unexposed members of the comparison group. The RR itself and its lower and upper 95% confidence limits (LCL and UCL) are always recorded, with the odds ratio chosen if available for a prevalence analysis and the relative risk (or hazard ratio if provided) for an incidence analysis. These may be as reported, or derived by various means (see below), with the method of derivation noted.

### Identifying which RRs to enter

For each outcome RRs were entered relating to defined combinations of smoking index (major or dose-related), confounders adjusted for, and sex, as described below.

#### The major smoking indices

The intention was to enter RRs comparing current smokers, ever smokers or ex smokers with never smokers. Near-equivalent definitions were accepted when stricter definitions were unavailable, so that never smokers could include occasional smokers (or exceptionally, light smokers), while current smokers could include, and ex-smokers exclude, those who quit smoking up to two years ago. If available, results were entered for five comparisons: any product vs. never any product, cigarettes vs. never any product, cigarettes only vs. never any product, cigarettes vs. never cigarettes, and cigarettes only vs. never cigarettes. Here "cigarettes" ignores whether other products (i.e. pipes and cigars) are smoked, while "cigarettes only" excludes mixed smokers.

#### Dose-related smoking indices

RRs were entered for five measures: amount smoked, age of starting, pack-years (cigarettes smoked per day times years of smoking, divided by 20), duration of smoking and duration of quitting. RRs were expressed relative to never smokers (or near equivalent), if available, or relative to non smokers otherwise. For duration of quitting, RRs were also expressed relative to current smokers. Further RRs were entered, restricted to smokers, and expressed relative to the level expected to have the lowest risk (e.g. lowest amount smoked, or longest time quit).

#### Confounders adjusted for

For prospective studies, results were entered adjusted for age and the greatest number of potential confounding variables for which results were available, and also adjusted for age only or age and the smallest number of confounders. Unadjusted results were only entered if no age-adjusted results were available. For other study types, results were entered adjusted for the greatest number of confounders, and also unadjusted (or adjusted for the smallest number of confounders). These alternative RRs are subsequently referred to as "most-adjusted" and "least-adjusted". For dose-related RRs restricted to smokers, results with "most adjustment" but without adjustment for other aspects of smoking were also entered if available.

#### Sex

Results were entered for males and females separately when available, with combined sex results only entered where sex-specific results were not available.

### Derivation of RRs

Adjusted RRs and their 95% CIs were entered as provided, when available. Unadjusted RRs and CIs were calculated from their 2 × 2 table, using standard methods (e.g. [12]), noting any discrepancies between calculated values and those provided by the author. Sometimes the 2 × 2 table was constructed by summing over groups (e.g. adding current and ex smokers to obtain ever smokers) or from a percentage distribution. Various other methods were used as required to provide estimates of the RR and CI. The more commonly used methods are summarized below, fuller details being given in Additional file 1.

*Correction for zero cell*. If the 2 × 2 table has a zero cell, 0.5 was added to each cell, and the standard formulae applied. *Combining independent RRs*. RRs were combined over ℓ strata (e.g. from a 2 × 2 × ℓ table) using fixed-effect meta-analysis [13], giving an estimate adjusted for the stratifying variable. *Combining non-independent RRs*. The Hamling *et al *method [14] was used (e.g. to derive an adjusted RR for ever smokers from available adjusted RRs for current and ex smokers, each relative to never smokers, or to combine adjusted RRs for several diseases, each relative to a single control or disease-free group). *Estimating CI from crude numbers*. If an adjusted RR lacked a CI or p-value but the corresponding 2 × 2 table was available, the CI was estimated assuming that the ratio UCL/LCL was the same as for the equivalent unadjusted RR.

### Data entry and checking

Master copies of all the papers in the study file were read closely, with relevant information highlighted to facilitate checking. Where multiple papers are available for a study, a principal publication was identified, although details described only in other publications were also recorded. Preliminary calculations and data entry were carried out by one author and checked by another, and automated checks of completeness and consistency were also conducted. RR/CIs underwent validation checks ([15]).

### Selecting RRs for the meta-analyses

All meta-analyses are restricted to records with available RR and CI values. The process of selecting RRs for inclusion in a meta-analysis must try to include all relevant data and to avoid double-counting. For a given analysis (e.g. of current cigarette smoking), several definitions of RR may be acceptable (e.g. cigarette smoking, or cigarette only smoking), so, for studies with multiple RRs, the one to be used is determined by an order of preference defined for the meta-analysis. Orders of preference may be required for smoking status, smoking product, the unexposed base, and extent of confounder adjustment. As the definitions of RR available may differ by sex (e.g. a study may provide RRs for any product smoking for males, but for cigarette smoking for females), the most appropriate RR is chosen within each sex. Sexes combined results are only considered where sex-specific results are not available. Similarly RRs from a subsidiary study are only used where eligible RRs are unavailable from the principal study. When multiple orders of preference are involved, the sequence of implementation may affect the selection, so preferences for the most important aspects, usually concerning smoking, are implemented first.

### Carrying out the meta-analyses

Fixed-effect and random-effects meta-analyses were conducted using the methods of Fleiss and Gross [13], with heterogeneity quantified by H, the ratio of the heterogeneity chisquared to its degrees of freedom. For all meta-analyses, Egger's test of publication bias [16] was also conducted.

A series of meta-analyses was conducted for each of the three main outcomes. For each meta-analysis conducted, combined estimates were made first for all the RRs selected, then for RRs subdivided by level of various characteristics, testing for heterogeneity between levels. These characteristics may include sex, continent, national cigarette type (blended, Virginia), start year of study, publication year, study type, lowest age included, highest age included, presence of study weakness, outcome subtype, how asthma was taken into account, use of a bronchodilator, study size (number of cases), analysis type (prevalence, onset), smoking product (any, cigarettes, cigarettes only), unexposed base (never any product, never cigarettes), smoking results available (ever smoking, current smoking, both), number of adjustment variables, whether the RR was adjusted for sex, age or for other factors, and how the RR and CI were derived. In this univariate approach, differences in fixed-effect estimates by level of a characteristic were tested for significance using an F-test which compared variation between and within levels of the characteristic considered. Additional file 1 fully defines the levels of each characteristic considered, and which characteristics are considered in each meta-analysis. It also details all the meta-analyses conducted, and describes the layout and notation used in the meta-analyses and associated forest and funnel plots.

For each selected outcome and exposure, separate meta-analyses were conducted based on most-adjusted and least-adjusted RRs.

For the major smoking indices, four broad types of meta-analysis were conducted: A ever smoking, B current smoking, C ever smoking (but using current smoking RRs if ever smoking RRs are not available) and D ex smoking. In each type, RRs for the "main analysis" were selected in the following order of preference: firstly for smoking of any product vs. never smoked any product, then for smoking of cigarettes (or of cigarettes only) vs. never smoked any product, and then for smoking of cigarettes vs. never smoked cigarettes, accepting RRs vs. near-equivalents to never smokers only when RRs vs. never smokers were unavailable. A variant analysis used a different order of preference, so that RRs for cigarette smoking were preferred. In type C meta-analyses, a further variant analysis preferred RRs for current smoking to those for ever smoking. Other variant analyses restricted attention to specific subtypes of outcome (e.g. for COPD, whether the definition was based on mortality, on lung function criteria only, or on other definitions).

For the dose-related indices, meta-analyses were conducted for: E amount smoked, F age of starting to smoke, G pack-years, H duration of smoking, I duration of quitting compared to never smokers (or long-term ex smokers), and J duration of quitting compared to current smokers (or short-term quitters). For any measure, a study typically provides a set of non-independent RRs for each dose-category, expressed relative to a common base. To avoid double-counting only one was included in any one meta-analysis. Two approaches were adopted. The first involves specifying a scheme with a number of levels of exposure ("key values"), then carrying out meta-analyses for each level in turn. For an RR to be allocated to a key value, its dose-category has to include that key value and no other. Schemes with a few, widely spaced, key values tend to involve RRs from more studies, whereas schemes with more key values, closely spaced, involve RRs from fewer studies, but ones with dose categories more closely clustered around the key value. The key value schemes used were: 5, 20 & 45 and 1, 10, 20, 30, 40 & 999 for amount smoked; 26, 18 & 14 for age of starting to smoke; 5, 20 & 45 and 1, 10, 20, 30 & 999 for pack-years; 12, 7 & 3 and 20, 12 & 3 for duration of quitting vs. never; and 3, 7 & 12 and 3, 12 & 20 for duration of quitting vs. current (with 999 indicating an open-ended category). The second approach involves meta-analysing RRs comparing the highest vs. lowest categories of exposure. Though this approach generally includes RRs from all studies, whereas the key-value approach does not, the highest and lowest categories compared may vary markedly by study.

### Meta-regression analyses of the major smoking indices

For COPD and CB meta-regression analyses were also carried out using the sets of RRs selected for the main meta-analyses for ever smoking and for current smoking. Following preliminary meta-regressions (not shown), a "basic model" was fitted which included eight categorical variables (sex, continent, outcome subtype, how asthma was taken into account, smoking product, unexposed base group, adjustment for age, and adjustment for factors other than age or sex) and also midpoint age, a continuous variable estimated from the age range of the population. The significance of each of these variables was estimated by an F-test based on the increase in deviance resulting from its exclusion from the basic model. A list of secondary variables was also defined (national cigarette type, publication year, study type, presence of a study weakness, use of a bronchodilator, study size, smoking results available for the study, method of derivation of the RR and CI and analysis type), with the significance of adding each characteristic to the basic model estimated by an F-test based on the increase in deviance. Alternative formulations of some basic variables were also tested; see also Additional file 1.

### Additional analyses

For each outcome, and for ever smoking and current smoking, pairs of corresponding RR and CI estimates within the same study for males and for females were used to carry out meta-analyses of the sex ratio. Pairs of corresponding least-adjusted or most-adjusted RRs were also identified. Unlike the sex-specific pairs, these pairs were non-independent and the variance of their ratio cannot readily be calculated. Here the numbers of pairs where the most-adjusted/least-adjusted ratio exceeded or did not exceed 1 were compared by the sign test, with separate meta-analyses also conducted for the least-adjusted and most-adjusted members. Similar methods were also used to compare non-independent pairs of RRs for current smokers of cigarettes only and for current smokers of cigarettes ignoring other products.

### Software

All data entry and most statistical analysis were carried out using ROELEE version 3.1 (available from P.N.Lee Statistics and Computing Ltd, 17 Cedar Road, Sutton, Surrey SM2 5DA, UK). Some analyses were conducted using Excel 2003.

## Results

### Studies identified

Some 218 relevant studies were identified, based on information from 298 papers.

For the 2,150 papers rejected, reasons are summarized in Table 1, with further details of the searching, including a flow diagram, shown in Additional file 1. Many papers had multiple reasons for rejection, the counts in Table 1 relating only to the first listed reason which applied. A Reference Manager file is available on request which, for each rejected publication, gives its reference and the reasons for rejection.

**Table 1:** Reasons for rejection of publications identified

Reason^a^	Number of publications	
IMMEDIATE REJECTS	63	
Title of publication indicates it is irrelevant (abstract/paper unavailable)		60
Publication could not be obtained		3
PUBLICATION DOES NOT PROVIDE ORIGINAL DATA	430	
Results the same as or superseded by another publication		16
Review (including guideline, handout, lecture, bibliography, meta-analysis)		329
Editorial		31
Comment, letter, interview or news article		47
Publication is a theoretical modelling exercise		7
STUDY POPULATION INAPPOPRIATE	343	
Study of children or adolescents		30
Animal study		5
Study in population at high risk of respiratory disease, such as risky occupations		71
Study of alpha-1antitrypsin deficient subjects		39
Study of subjects with other coexisting diseases or conditions		105
Study of atypical populations		7
Subjects selected on smoking habits		61
Study of symptom-free or symptom-restricted populations		25
STUDY DESIGN INAPPROPRIATE	323	
Not a case-control, prospective or cross-sectional study		84
Study of cases only		216
Control group not appropriate		9
Selection of subjects not clear		14
OUTCOME INAPPROPRIATE	566	
Outcome not relevant		557
Study of undiagnosed disease		4
Study of disease exacerbation		5
USEFUL RESULTS BY SMOKING UNAVAILABLE	425	
Never smokers not considered		36
No relevant results by smoking		304
Comparisons with never smokers and ex-smokers combined		17
Study of smokers of unusual cigarettes (e.g. chuttas)		2
Relative risks not calculable		61
Relative risks adjusted for symptoms or precursors of disease		5
Total rejected	2150	


^a^Where publications had more than one reason for rejection, the publication is counted under the first relevant reason listed

Table 2 presents selected details of the 218 studies while Table 3 gives the distribution of their major characteristics. Additional file 2 gives fuller descriptions of the studies, including overlapping and linked studies, medical and other exclusions, detailed definitions of disease outcomes, and fuller distributions.

**Table 2:** Selected details of the 218 studies of COPD, CB and/or emphysema

Study REF [refs]	Study type^a^	Country	Years^b^	Population^c^	Outcome(s)^d^	Study group (if Subsid)^e^	Weakness^f^
ALDERS [26,27]	CCh	UK/England	1977-82		CB		No
ALESSA [28]	CCp	Italy	1992-93		COPD		Yes
AMIGO [29]	CCh	Chile	2001-03		COPD		No
ANDER1 [30]	CS	Canada	1963		COPD, CB		No
ANDER2 [31]	CS	USA	(ca 1964?)		EM		No
ANDER3 [32,33]	CCp	Poland	(ca 1997?)		COPD		Yes
AUERBA [34,35]	CS	USA	1963-70		EM		No
BANG [36]	CS	USA	1982-84	hispanic	CB		No
BECK1 [37]	CS	USA	1972-73		CB		No
BECK2 [37]	P	USA	1972-73/1978		CB		No
BEDNAR [38]	CS	Poland	2000-02		COPD		No
BEST [39,40]	P	Canada	1955-56/1962	military veterans	COPD, CB, EM		No
BJORNS [41]	CS	Sweden	1990		CB		No
BROGGE [42]	CCm	Norway	2003		COPD	JOHANN	Yes
BROWN [43]	CS	UK/England	1956		CB		No
CERVER [44]	CS	Italy	1998-00		CB		No
CHAPMA [45]	CS	USA	1976	parents	CB	HOUSE	No
CHEN1 [46]	P	China	1972-78/1993	workers at 11 factories	COPD		No
CHEN2 [47]	CS	Canada	1994-95	household members	COPD		No
CHEN3 [48]	CS	Canada	2000-01	household members	COPD		Yes
CHENG [49]	CS	China	1992		COPD		No
CLEMEN [50]	P	Belgium	1960-*/1975	Air Force personnel	COPD		No
COATES [51]	CS	USA	1962	Post Office employees	CB		No
COCCI [52]	CCp	Italy	(ca 2000?)		COPD		Yes
COLLEG [53,54]	CS	UK/GB	(ca 1960?)		CB		No
DEAN1 [55,56]	CCp	UK/England	1969-73		COPD		Yes
DEAN2 [57]	CS	UK/GB	1972		CB		No
DEANE [58]	CS	USA	1963	telephone company employees	CB		No
DEJONG [59]	CS	USA	(ca 2003?)		COPD		Yes
DEMARC [60-62]	CS	Multi-Europe	1991-93		COPD, CB		No
DETORR [63]	CS	Spain	2001-03	patients	COPD		Yes
DICKIN [64]	CS	UK/England	(ca 1997?)		COPD		No
DOLL1 [65-67]	P	UK	1951/1991	doctors	COPD, CB		No
DOLL2 [66,68]	P	UK	1951/1973	doctors	COPD, CB		No
DONTA1 [69]	CS	Greece	1960		COPD	JACOBS	Yes
DONTA2 [69]	P	Greece	1960/1970		CB, EM	JACOBS	Yes
DOPICO [70]	CS	USA	(ca 1982?)	employed^g^	CB		No
EHRLIC [71]	CS	South Africa	1998	household members	CB		No
EKBERG [72]	CS	Sweden	1974-92		COPD		No
ENRIGH [73]	CS	USA	1989-90	health insurance members	CB, EM		No
ENSTRO [74]	P	USA	1960/1998	household members	COPD	HAMMO2	No
FERRI1 [54,75-77]	CS	USA	1961	household members	COPD, CB	FERRI2	No
FERRI2 [75,78]	CS	USA	1967	household members, long term residents	COPD		No
FERRI3 [79]	CS	USA	1973	household members, long term residents	COPD	FERRI2	No
FIDAN [80]	CS	Turkey	2000-01	coffeehouse or shop workers	COPD		No
FINKLE [81]	CS	USA	1969-70	military recruits	CB		No
FLETCH [82]	CS	UK	1956-57	employed^g^	CB		No
FORAST [83]	CS	USA	1993-94		COPD		Yes
FOXMAN [84]	CS	USA	(ca 1981?)		CB		No
FUKUCH [85]	CS	Japan	2000	household members	COPD		No
GEIJER [86]	P	Netherlands	1998/2003		COPD		No
GODTFR [18,87]	P	Denmark	1964-93/1997		COPD		No
GOLDBE [88]	CS	USA	1970	parents	CB		No
GULSVI [89-91]	CS	Norway	1972-74		COPD, EM		No
HAENSZ [92]	CS	Norway	1964	mixed^h^	CB		No
HAMMO2 [93-97]	P	USA	1959-60/1965	household members	COPD, EM		No
HARDIE [98]	CS	Norway	1998-99		COPD, CB, EM		No
HARIKK [99]	P	USA	1962-*/(ca 2000?)		COPD		No
HARRIS [100]	CS	Nigeria	(ca 1992?)	soldiers	CB		No
HAWTHO [101]	P	UK/Scotland	1965-75/1977	mixed^h^	COPD, CB	TANG	Yes
HAYES [102]	CS	USA	1970	parents	CB		No
HEDMAN [103]	CS	Finland	1996		COPD		No
HIGGI2 [104]	CS	UK/Wales	1956		CB	PETO	No
HIGGI3 [105]	CS	UK/Scotland	1956		CB		No
HIGGI4 [106,107]	P	USA	1962-79/1987		COPD		Yes
HIGGI6 [108,109]	CS	USA	1962-65		CB	HIGGI4	No
HIRAYA [110,111]	P	Japan	1965/1982		CB, EM		No
HO [112,113]	CS	Hong Kong	1991	long term residents	COPD, CB, EM		No
HOLLA2 [114]	CS	USA	1962	telephone company employees	CB		No
HOLLNA [115,116]	CS	Denmark	1976-77		CB		No
HOUSE [117]	CS	USA	1970	parents	CB		No
HOZAWA [118-120]	CS	USA	1987-89		COPD, EM		No
HRUBEC [121]	CS	USA	(ca 1972?)	veterans and twins	CB		No
HUCHON [122]	CS	France	(ca 2001?)	household members	CB		No
HUHTI1 [123]	CS	Finland	1961		COPD, CB, EM		No
HUHTI2 [124]	CS	Finland	1971	long term residents	COPD, CB	HUHTI1	No
HUHTI3 [125]	CS	Finland	1968-70		COPD, CB		No
ITABAS [126]	CCp	Japan	(ca 1989?)		COPD		Yes
JACOBS [127]	P	Multi-Europe	1957-64/(ca 1989?)		COPD		No
JAENDI [128]	CS	Spain	2001-02	patients	COPD		Yes
JENSEN [129]	CS	Denmark	(ca 1996?)	patients	CB		Yes
JINDA2 [130]	CS	India	(ca 2004?)	household members	CB		No
JOHANN [131-134]	CS	Norway	1996-97	long term residents	COPD		No
JOSHI [135]	CS	India	(ca 1974?)	employed^g^	CB		No
JOUSI1 [136]	CS	Finland	1972-77		CB		No
KACHEL [137]	CS	Poland	(ca 2002?)	workers at five factories	COPD		No
KAHN [138-144]	P	USA	1954-57/1980	military veterans	COPD, CB, EM		No
KAHN2 [138]	P	USA	1954-57/1962	military veterans	COPD, CB, EM	KAHN	No
KARAKA [145]	NCCp	Greece	*/1996		COPD	VINEIS	No
KATANC [146]	CS	USA	1997-98	mixed^h^	COPD		No
KATO [147]	CS	Japan	1985		CB		No
KHOURY [148]	CS	USA	(1970s)	mixed^h^	COPD		Yes
KIM [149]	CS	Korea	2001-02	household members	COPD		No
KIRAZ [150]	CS	Turkey	1999	mixed^h^	COPD, CB		No
KLAYTO [151]	CS	USA	(ca 1974?)	employed^g^	COPD		Yes
KOJIMA [152]	CS	Japan	2001-02		COPD		No
KOTAN1 [153]	CS	Finland	1995-96		CB		No
KOTAN2 [154]	CS	Finland	1996-97		COPD	KOTAN1	No
KRZYZA [155]	P	Poland	1968/1981		COPD		No
KUBIK [156]	CS	Czechoslovakia	1972		CB		Yes
KULLER [157]	P	USA	1972-74/1980	volunteers	COPD		No
LAI [158]	CS	China	2001-03		COPD		No
LAM1 [159]	P	China	1976/1996	employed^g^	COPD		No
LAM2 [160,161]	CS	China	1987	military veterans	COPD		No
LAM3 [160-162]	P	China	1987/2005	military veterans	COPD		No
LAMBER [163,164]	CS	UK	1965	household members	CB	TODD	No
LANGE [165-169]	P	Denmark	1976-78/1989		COPD	GODTFR	No
LANGE2 [170]	CS	Denmark	1991-94		CB	VESTBO	No
LANGHA [19]	CS	Norway	1995-97		CB		No
LAVECC [171]	CS	Italy	1983	household members	CB, EM		No
LEBOWI [172-177]	CS	USA	1972-73	household members	COPD, CB, EM		No
LEE [164,178]	P	UK	1964-65/1977	siblings of migrants	COPD		No
LIAW [179]	P	Taiwan	1982-86/1993	volunteers	COPD	WEN	No
LINDBE [180]	P	Sweden	1996/2003	long term residents	COPD		No
LINDST [181]	CS	Europe	(ca 1998?)		COPD, CB		No
LIU1 [182]	CCd	China	1986-91		COPD		No
LIU2 [183,184]	CS	China	2002-03		COPD		No
LUNDB1 [185,186]	CS	Sweden	1996-97	long term residents	COPD		No
LUNDB2 [187-189]	CCp	Sweden	1986		CB	LUNDB1	Yes
MADOR [190]	CCh	USA	(ca 2002?)	military veterans	COPD		No
MAGNUS [191]	CS	Iceland	1993		CB		No
MANFRE [192]	CS	Canada	1978-79		CB		No
MANNI1 [193-198]	CS	USA	1988-94		COPD		No
MANNI2 [199,200]	CS	USA	1971-75	household members	COPD		No
MANNI3 [199]	P	USA	1971-75/1992	household members	COPD		No
MARAN1 [201]	CS	Thailand	1998		COPD		Yes
MARAN2 [201]	P	Thailand	1998/1999		COPD		Yes
MARCUS [202]	P	USA	1965-68/1984	Japanese ancestry	COPD		No
MATHES [203]	CCp	Australia	(ca 2005?)		COPD		No
MELLST [204]	CS	Sweden	1971-77		CB		No
MENEZ1 [205]	CS	Brazil	1990	household members	CB		No
MENEZ2 [206,207]	CS	Brazil	2003	household members	COPD		No
MENEZ3 [206]	CS	Chile	2003	household members	COPD		No
MENEZ4 [206]	CS	Mexico	2003	household members	COPD		No
MENEZ5 [206]	CS	Uruguay	2003	household members	COPD		No
MENEZ6 [206]	CS	Venezuela	2003	household members	COPD		No
MEREN [208]	CS	Estonia	1995-96		CB		No
MILLER [209]	CS	USA	1978	household members	CB, EM		No
MILNE [210,211]	CS	UK/Scotland	1968-70		CB		No
MOLLER [212]	CCp	Germany	(ca 1999?)		CB		Yes
MONTNE [213-215]	CS	Sweden	1992		COPD		No
MUELLE [216]	CS	USA	1967	household members	COPD, CB		No
NAWA [217]	CS	Japan	1998-00	mixed^h^	EM		No
NEJJAR [218]	CS	France	1991	household members	CB		No
NIEPSU [219]	CS	Poland	2001		COPD		Yes
NIHLEN [220,221]	P	Sweden	1992/2000	long term residents	COPD		No
NILSSO [222-224]	P	Sweden	1963/1996		COPD		No
OGILVI [225]	CCp	UK/England	1955-56	household members	CB		No
OMORI [226]	CS	Japan	(ca 2004?)		EM		Yes
OSWAL1 [227]	CCh	UK/England	1951-53	mixed^h^	CB		No
OSWAL2 [228]	CS	UK/England	1954-55	civil servants	CB		Yes
PANDEY [229,230]	CS	Nepal	1979-80		CB		No
PEAT [231,232]	P	Australia	1966-75/1984		COPD		No
PELKON [233]	P	Finland	1959/2000		COPD, CB	JACOBS	No
PEREZP [234]	CCm	Mexico	1992-94		COPD, CB		No
PETO [235]	P	UK/England and Wales	1954-61/1981	mixed^h^	COPD		Yes
PRATT [236]	CS	USA	(ca 1978?)	military veterans	EM		Yes
PRICE [237,238]	CS	UK	(ca 2004?)		COPD		No
REID [239]	CS	USA	1962-63	mixed^h^	CB		No
RENWIC [240]	CS	UK/England	1992-94		COPD		No
RICCIO [241]	CS	Italy	2002	patients	COPD		Yes
RIMING [242,243]	CS	UK/England	1970	volunteers	CB		No
RYDER [244]	CS	UK/Wales	(ca 1969?)		EM		No
SARGEA [245,246]	CS	UK/England	1993-96		COPD	VINEIS	No
SAWICK [247,248]	CS	Poland	1968		COPD, CB		No
SCHWAR [249,250]	CS	USA	1976-80	household members	CB		No
SHAHAB [251]	CS	England	2001		COPD		No
SHARP [252,253]	CS	USA	1960-61	employed^g^	CB		No
SHIMUR [254]	CCh	Japan	(ca 1994?)		CB		Yes
SHIN [255]	CS	Korea	1999-00	household members	COPD		No
SICHLE [256]	CS	Greece	2000-01		COPD		No
SILVA [257-260]	P	USA	1972-*/(ca 1992?)	household members	COPD, CB, EM		No
SOBRAD [261-264]	CS	Spain	1996-97		CB		No
SPEIZE [265]	P	USA	1974-77/1986	household members	COPD		No
STERLI [266,267]	CCp	USA	1986-87		COPD		Yes
STJERN [268]	CS	Sweden	1981		CB		No
STROM [269]	CS	Sweden	1982-83	long term residents	COPD		No
SUADIC [270]	CS	Denmark	1985-86	employed^g^	CB		No
SUTINE [271]	CS	Finland	1971-72		EM		No
TAGER [272]	CS	USA	1973-74		COPD		No
TAGER2 [273]	CS	USA	1973-74	household members	CB		Yes
TANG [274]	P	UK	1967-82/*	mixed^h^	COPD		No
THUN [96,97,275,276]	P	USA	1982/1988	household members	COPD		No
TODD [164,178]	P	UK	1965-66/1977	household members	COPD		No
TROISI [277]	P	USA	1980/1990	nurses	CB		No
TRUPIN [278,279]	CS	USA	2001	telephone subscribers	COPD		No
TSUSHI [280]	CS	Japan	2003-04	volunteers	COPD		No
TVERDA [281]	P	Norway	1972-78/1988		COPD		Yes
URRUTI [282]	CS	Spain	(ca 2004?)		CB	DEMARC	No
VESTBO [168,283]	CS	Denmark	1976-78		COPD		No
VIEGI1 [284]	CS	Italy	1980-82	household members	CB		No
VIEGI2 [285]	CS	Italy	1988-91		COPD		No
VIKGRE [286-288]	P	Sweden	1994-95/2001	long term residents	EM		No
VINEIS [289]	P	Europe	1993-98/(ca 2003?)	volunteers	COPD		No
VOLLM1 [290]	CS	USA	1971-72	volunteers	COPD	VOLLM2	Yes
VOLLM2 [290]	P	USA	1971-72/1982	volunteers	COPD		Yes
VONHER [291]	CS	Finland	1978-80		COPD		No
WAGEN2 [292-294]	CCp	Netherlands	2001	employed^g^	CB		No
WALD [295]	P	UK/England	1975-82/1993	professional/business men	COPD	TANG	Yes
WANG2 [296]	CS	Japan	1996-98	volunteers	EM		No
WATSON [297]	CCp	UK	(ca 2000?)		COPD		No
WEISS [298]	CS	USA	1961	volunteers	COPD, EM		No
WEN [299]	P	Taiwan	1982-92/2000	mixed^h^	COPD, CB, EM		No
WIG [300]	CS	India	(ca 1963?)	household members	CB		Yes
WILHEL [301]	CS	Sweden	1967		CB		No
WILSO1 [302]	CS	Australia	2000	household members	COPD		No
WILSO2 [303]	CS	Australia	1998	household members	CB, EM		No
WOJTYN [304,305]	CS	Poland	1968-73		COPD, CB	SAWICK	No
WOODS [306]	CS	Australia	(ca 1998?)		CB		No
WOOLF [307]	CS	Canada	(ca 1970-73?)	employed^g^	CB		No
XIAO [308]	CCp	China	(ca 2003?)		COPD		No
XU [309]	CS	China	2000-01	long term residents	COPD		No
YAMAGU [20,310]	CS	China	1986	long term residents	COPD, CB		No
YUAN [311]	P	China	1986-89/1993		COPD		No
ZIELI1 [312-314]	CS	Poland	1999	volunteers	COPD		No
ZIELI2 [315-317]	CS	Poland	2000-03	volunteers	COPD		No
ZIETKO [318]	CCp	Poland	(ca 2003?)		COPD		Yes
ZOIA [319]	CS	Italy	(ca 1993?)		CB		No

^a^CS = cross-sectional, P = prospective, CC = case control, NCC = nested case-control, h = hospital or clinic controls, p = population controls, d = decedent controls, m = mixed controls.

^b^* = unknown. Values in brackets are approximate, based on one year before first publication. For prospective studies, baseline year(s)/final follow-up year. Results refer to the full follow-up period except:

DOLL1, DOLL2 CB, to 1961

HIRAYA Emphysema (except by amount smoked), to 1978

KAHN Current smoking by amount smoked, to 1970

NILSSO Smoking of any product, to 1979

THUN Ex and ever smoking, to 1986

^c ^Unless shown otherwise in this column, the study specified no major inclusion criteria.

^d ^COPD = chronic obstructive lung disease, CB = chronic bronchitis, EM = emphysema

^e ^For subsidiary studies, this column shows the relevant principal study.

^f ^Weakness identified, including studies where the base for comparison of the major smoking indices was not strictly never smokers:

ALESSA Small clinical study, not stated how subjects selected

ANDER3 Small clinical study, not clear how controls were selected

BROGGE More cases than controls were drawn from hospital sample (with hospitalisation for COPD in last 5 years) and their average age was 3.5 years older

CHEN3 Analysis combines current smokers with those who gave up in last 5 years, and omits those who started smoking before age 13 or after age 22

COCCI Small clinical study, not stated how cases and controls were selected

DEAN1 Cases occurred in 1969-72 while information on controls was collected in 1973. Cases were population sample but controls were household members only

DEJONG Non-representative convenience sample particularly aimed at smokers

DETORR Subjects were volunteers, invited from all smokers attending wards or clinics, so likely to have concomitant disease

DONTA1 Inclusion of various lung diseases other than COPD in study endpoint, exclusion of subjects who died, emigrated or made dramatic changes to their smoking habits during follow-up

DONTA2 Exclusion of subjects who died, emigrated or made dramatic changes to their smoking habits during follow-up

FORAST Cases without symptoms in the last year were excluded

HAWTHO Base for comparison includes smokers of up to 5 cigarettes per day

HIGGI4 Because of inadequate detail in the report and use of differing age groups in different tables, estimates are rather speculative

ITABAS Small clinical study, not stated how cases and controls were selected

JAENDI Study population were those who visited their primary care physician, so may have been less healthy than the general population. Some attempts were made to contact patients who did not visit their physician during the study period, but it is unclear if they were then included in the study. It is not clear why only 7% of subjects were age 65+

JENSEN All subjects were participants in smoking cessation programme

KHOURY 13% of sample were 1st degree relatives of COPD cases, and a further 3% were 1st degree relatives of lung cancer cases

KLAYTO Base for comparison includes smokers of up to 5 pack-years

KUBIK Base for comparison includes smokers of up to 3 cigarettes per day

LUNDB2 A few subjects were analysed as controls (as determined at the start of the study) even if diagnosed with CB or asthma at the second phase of the study when the diagnosis category of the cases was determined

MARAN1 Base for comparison includes smokers of up to 0.5 pack-years

MARAN2 Base for comparison includes smokers of up to 0.5 pack-years

MOLLER Small clinical study, not clear how subjects were selected

NIEPSU Numbers of smokers not given for subset of participants undergoing spirometry (74%), therefore estimated using same proportions as whole study sample

OMORI Different diagnostic techniques used for smokers

OSWAL2 Base for comparison includes smokers of up to 5 cigarettes per day or who had smoked for less than 5 years

PETO Three of the samples were drawn from mining areas with over 60% miners or other dusty jobs, implying about 40% of the overall sample were occupationally exposed.

PRATT Study contained small number of subjects who were cotton or tobacco farmers or who worked in tobacco factory

RICCIO Subjects were recruited through a respiratory clinic but it is not stated whether they all had respiratory conditions. The definition of a smoker seems implausible

SHIMUR Small autopsy study, not clear how subjects were selected

STERLI All decedents proxy vs. none of living sample. Living sample 1 year later than decedents

TAGER2 Age distribution for both men and women in study sample was significantly different from general population from which sample was drawn. Subjects who smoked but did not inhale were excluded

TVERDA Includes acute bronchitis

VOLLM1 Study population consists of volunteers who responded to extensive media advertising and cohort is biased towards those with respiratory disease, and analysis restricted to those with follow-up data. Subjects with abnormal baseline FEV were not invited to some phases so may have different follow-up rate

VOLLM2 Study population consists of volunteers who responded to extensive media advertising and cohort is biased towards those with respiratory disease, and analysis restricted to those with follow-up data. Subjects with abnormal baseline FEV were not invited to some phases so may have different follow-up rate

WALD Includes ICD9: 416 (chronic pulmonary heart disease) and 519 (other diseases of respiratory system)

WIG Urban area is not a typical sample, as socio-economic status is above average

ZIETKO Small clinical study, not clear how controls were selected

Note that weakness is in respect of the current review, and is not a criticism of the original study which may have been designed with different objectives.

^g ^Study conducted in employed or occupational group:

DOPICO outdoor workers for city and power company

FLETCH men-postmen, women-clerical workers

JOSHI employees at machine tool factory and woollen hosiery mill

KLAYTO employees at two research facilities

LAM1 employees in a machine factory

SHARP clerical and light assembly workers at power company

SUADIC armed forces, customs service, railway, telephone, post, banking and construction companies

WAGEN2 heterogeneous population of employees from different companies and organisations

WOOLF employees of large commercial firms

^h ^Study conducted in mixed groups:

HAENSZ nationwide sample plus siblings of migrants to USA still resident in Norway

HAWTHO occupational groups (from industry, not otherwise specified) and census-identified sample

KATANC whites from Medicare, blacks from general population

KHOURY subjects were relatives of COPD cases (cases having been identified through Johns Hopkins Hospital respiratory laboratory), relatives of lung cancer and non-pulmonary patients, or community-based samples (neighbours and teachers)

KIRAZ rural group using biomass cookers and urban group using fuel oil

NAWA healthy workers/retired persons

OSWAL1 cases were general clinic patients, and civil servants referred after repeated sickness due to bronchitis

PETO occupational groups (transport and clerical workers) and census-identified sample

REID general and migrants from UK and Norway

TANG businessmen/professionals, civil servants, general population from socially deprived area, industrial workers

WEN community cohort were volunteers invited for screening and comprised 25% of population in study areas; other cohort were civil servants and teachers in government employee insurance scheme

**Table 3:** Distribution of the main characteristics of the 218 studies of COPD, CB and/or emphysema

		Disease studied (principal studies)			Study type (principal studies)^a^			Subsidiary	All
					
Characteristic	Level	COPD	CB	EMP	CC	Prosp	Cross-sec	studies	studies
Study status	Principal	116	87	26	20	39	134	-	193
	Subsidiary	(17)	(14)	(2)	-	-	-	25	25
Study type	Case/control	14	7	0	20	-	-	2	22
	Prospective	35	9	7	-	39	-	8	47
	Cross-sectional	67	71	19	-	-	134	14	148
	Nested case/control	0	0	0	-	-	-	1	1
Study sex	Both	89	62	18	17	20	108	20	165
	Male	23	20	8	2	17	23	5	47
	Female	4	5	0	1	2	3	0	6
Lowest age^b^	< 20	26	22	10	5	4	42	3	54
	20-29	28	25	2	3	10	32	9	54
	30-39	23	12	4	6	15	11	6	38
	40-49	26	17	4	5	7	29	5	46
	50+	13	11	6	1	3	20	2	26
Highest age^b^	< 50	3	6	0	0	2	6	1	9
	50-59	4	8	0	0	4	8	1	13
	60-69	20	21	6	2	9	29	5	45
	70-79	19	12	1	4	6	19	8	37
	80+	70	39	19	14	18	71	10	113
	Unknown	0	1	0	0	0	1	0	1
Region	USA/Canada	31	28	12	2	14	40	7	63
	S/C America	7	2	0	2	0	6	0	8
	UK	11	13	1	5	6	12	5	28
	Western Europe	8	9	1	4	2	11	1	18
	Scandinavia	18	16	5	0	6	26	7	39
	Other Europe	12	4	0	2	1	11	4	18
	Asia	22	10	6	4	7	21	1	33
	Other	3	4	1	1	1	5	0	7
	Multicountry	4	1	0	0	2	2	0	4
Start year of study	< 1960	7	10	3	2	7	4	3	16
	1960-1969	19	15	4	1	12	17	8	38
	1970-79	15	19	5	1	9	23	6	39
	1980-89	12	10	4	2	5	13	2	22
	1990-99	23	16	6	2	6	29	3	40
	> 1999	25	1	0	2	0	24	1	27
	Unknown	15	16	4	10	0	24	2	36
Major study weakness	Present	19	5	2	9	4	13	6	32
Smoking results available	Current v never	89	70	15	14	34	98	23	169
	Ex v never	82	65	12	12	29	93	19	153
	Ever v never	95	71	20	16	29	113	18	176
	Amount smoked	39	42	10	7	23	47	14	91
	Age started	13	3	1	5	7	5	3	20
	Pack years	42	13	3	10	5	43	5	63
	Duration of smoking	6	4	3	2	4	6	2	14
	Duration of quitting	8	7	2	3	8	7	4	22
Outcomes	COPD only	93	-	-	13	28	52	11	104
	CB only	-	63	-	6	2	55	7	70
	Emp only	-	-	9	0	1	8	0	9
	More than one	23	24	17	1	8	19	7	35
	COPD	116	-	-	14	35	67	17	133
	CB	-	87	-	7	9	71	14	101
	Emp	-	-	26	0	7	19	2	28
All studies		116	87	26	20	39	134	25	218


^a ^CC = case control, Prosp = prospective, Cross-sec = cross-sectional

^b ^At start of study.

Of the 218 studies, 193 are classified as principal, 20 (10.4%) of these being case-control studies, 39 (22.7%) prospective, and 134 (69.4%) cross-sectional. The other 25 studies are classified as subsidiary. Ninety-three principal studies are of COPD only, 63 of CB only, nine of emphysema only, with 28 providing results for multiple outcomes. In total, information is available on COPD for 133 studies (116 principal), CB for 101 (87 principal) and emphysema for 28 (26 principal). Of the principal studies, only 9 (10.3%) are prospective for CB, compared with 35 (30.2%) for COPD and 7 (26.9%) for emphysema. There are no case-control studies for emphysema.

Of the 193 principal studies, 145 (75.1%) provide results for both sexes, 42 (21.8%) for males only, and six (3.1%) for females only. Ninety-six (49.7%) of the studies include subjects under 30 (at baseline for prospective studies), while only 24 (12.4%) are restricted to subjects aged 50 or more. Subjects aged 80 or more are included by 103 (53.6%), while only 20 (10.3%) are restricted to subjects aged 60 or less. Fifty-six (29.0%) principal studies were conducted in USA or Canada, with 32 (16.6%) in Scandinavia, 32 (16.6%) in Asia, 23 (11.9%) in the UK, 17 (8.8%) in Western Europe, 14 (7.3%) in other parts of Europe, eight (4.1%) in South or Central America and seven (3.6%) elsewhere. Four (2.1%) were carried out in more than one of these areas. Of the 159 principal studies where the start year is given, 76 (47.7%) started before 1980. For 26 (13.5%) of the 193 studies a major study weakness is noted. Most commonly this is a failure to clarify, or to state at all, how study subjects were selected (studies ALESSA, ANDER3, COCCI, ITABAS, MOLLER, SHIMUR, ZIETKO). Other more commonly occurring weaknesses include use of unrepresentative samples which oversampled smokers (DEJONG, DETORR, JENSEN), those with respiratory disease (VOLLM1, VOLLM2) or those with occupational exposure (PETO, PRATT), and the use of controls that systematically differ from cases and controls in various ways (BROGGE, DEAN1, LUNDB2, STERLI). These weaknesses are described more fully in the footnotes to Table 2.

Most principal studies provide some results compared to never smokers, 146 (75.6%) for current smokers, 134 (69.4%) for ex smokers and 158 (81.8%) for ever smokers. Dose-response data are commonly available by amount smoked (77 studies, 39.9%) and by pack-years (58, 30.1%), but less so by age of starting to smoke (17, 8.8%), duration of smoking (12, 6.2%) or duration of quitting (18, 9.3%).

Of the 116 principal studies of COPD, outcome is based on ICD codes in 29 (25.0%), and lung function only in 59 (50.9%). The GOLD criteria are used in 27 (23.2%) studies, with MRC, ATS, ERS or BTS criteria used in 12 (10.3%). In 69 (59.5%) studies the subjects' asthma status is ignored, in 18 (15.5%) all asthmatics subjects are excluded, and in 14 (12.1%) the disease definition includes asthma. Only 19 (16.4%) of the 116 principal studies mention conducting spirometry after use of a bronchodilator. The outcome is based on prevalence in 79 (68.1%) principal studies, mortality in 28 (24.1%) and incidence in 10 (8.6%). In the principal studies, the median number of subjects is 2,033, and of cases 131 (range 13 to 32,822).

Of the 87 principal studies of CB, the outcome is based on symptoms (not lung function) in 59 (67.8%), and on ICD in only six (6.9%). Other studies use self-report, a doctor diagnosis, or other definitions. The MRC questionnaire is used in 21 (24.1%). Asthmatics are excluded totally from six (6.9%) studies, with asthmatics excluded only from the controls in three (3.4%). The outcome is based on prevalence in 78 (89.7%) of the principal studies, mortality in six (6.9%) and incidence in three (3.4%). The median number of subjects is 2,826, and of cases 193.5 (range 2 to 4,769).

Of the 26 principal studies of emphysema, the outcome is based on visual comparison in 10 (38.5%), on diagnosis in seven (26.9%), on ICD codes in five (19.2%) and on other sources including self-report in four (15.4%). Asthmatics are excluded in two (7.7%) studies. The outcome is based on prevalence in 19 (73.1%) of the studies, on mortality in five (19.2%) and on incidence in two (7.7%). The median number of subjects is 2,433, and of cases 96.5 (range 2 to 1384).

### Relative risks

A total of 3,538 RRs are entered, 1,578 for COPD, 1,689 for CB and 271 for emphysema, the number recorded per study varying from 1 to 211. Some 675 relate to subsidiary studies. Table 4 summarizes the distribution of various characteristics of the RRs by outcome, by study type for the principal studies, and overall. For fuller distributions of the RRs, referred to as necessary below, see Additional file 3.

**Table 4:** Distribution of the main characteristics of the relative risks (principal studies only^a^)

RRs	Characteristic	Level	Outcome			
			
			COPD	CB	Emphysema	Total
All RRs	Total	Total	1,342	1,311	210	2,863
	Study type	Case/control	194	162	0	356
		Prospective	414	86	66	566
		Cross-sectional	734	1,063	144	1,941
	Sex	Both	289	215	35	539
		Male	660	552	114	1,326
		Female	393	544	61	998
	Adjusted for any variable	No	605	677	93	1,375
		Yes	737	634	117	1,488
	Adjusted for age	No	624	758	99	1,481
		Yes	718	553	111	1,382
	Adjusted for other confounders^b^	No	1,051	1,135	187	2,373
		Yes	291	176	23	490
	Derivation	Original or 2 × 2^c^	409	478	86	973
		Other derived	933	833	124	1890
RRs for major smoking indices	Total	Total	551	547	105	1,203
	Exposed group: smoking status	Ever	185	186	45	416
		Current	214	205	36	455
		Ex	152	156	24	332
	Exposed group: smoking product	Any product	166	211	29	406
		Cigarettes (+/- other)	314	269	62	645
		Cigarettes only	71	67	14	152
	Unexposed	Never any product	283	335	53	671
		Never cigarettes	261	206	52	519
		Other^d^	7	6	0	13
RRs for all dose-response indices	Total	Total	791	764	105	1,660
	Exposed group: smoking status	Ever	221	138	19	378
		Current	415	492	83	990
		Ex	155	134	3	292
	Exposed group: smoking product	Any product	64	176	8	248
		Cigarettes (+/- other)	525	399	69	993
		Cigarettes only	202	189	28	419
	Unexposed	Never (any or cigs)	389	387	60	836
		Low^e ^(any or cigs)	318	295	38	651
		Current (any or cigs)	33	32	0	65
		Other^f^/not applicable^g^	51	50	7	108
RRs for amount smoked	Total	Total	335	474	65	874
	Exposed group: smoking status	Ever	3	51	9	63
		Current	323	413	55	791
		Ex	9	10	1	20
Sets for amount smoked		vs. never (any or cigs)	66	94	14	174
		vs. low amount	65	97	16	178
		vs. non^h^	2	9	0	11
		Non categorical	9	11	3	23
RRs for age started	Total	Total	78	50	6	134
	Exposed group: smoking status	Ever	6	38	0	44
		Current	65	12	6	83
		Ex	7	0	0	7
Sets for age started		vs. never (any or cigs)	12	4	2	18
		vs. later start	21	9	2	32
		vs. non^h^	1	0	0	1
		Non categorical	6	0	0	6
RRs for pack-years	Total	Total	225	88	15	328
	Exposed group: smoking status	Ever	208	48	5	261
		Current	14	30	10	54
		Ex	3	10	0	13
Sets for pack-years		vs. never (any or cigs)	34	15	3	52
		vs. low pack-years	41	15	3	59
		Non categorical	25	9	2	36
RRs for duration of smoking	Total	Total	17	39	17	73
	Exposed group: smoking status	Ever	4	1	5	10
		Current	13	37	12	62
		Ex	0	1	0	1
Sets for duration of smoking		vs. never (any or cigs)	2	6	3	11
		vs. low duration	2	6	2	10
		Non categorical	5	5	0	10
RRs for duration of quitting	Total	Total	136	113	2	251
Sets for duration of quitting		vs. never (any or cigs)	13	13	0	26
		vs. current	12	11	0	23
		vs. long term ex	14	11	0	25
		vs. recent ex	14	11	0	25
		Non categorical	2	3	2	7

^a ^The number of additional RRs recorded for subsidiary studies was 236 for COPD, 378 for CB and 61 for emphysema.

^b ^Variables other than sex, age and aspects of smoking

^c ^Calculated directly from 2 × 2 or 2 × 2 × ℓ table

^d ^Never or low amount (any or cigs)

^e ^Low in terms of the measure of exposure (e.g. low amount for amount smoked, later starting for age started)

^f ^Never or low amount (any or cigs); or never and ex smokers combined

^g ^Dose-response RR not for categorical data

^h ^Never and ex smokers combined

Of the 2,863 RRs in principal studies, 67.8% relate to cross-sectional, 19.8% to prospective, and 12.4% to case-control studies. 81.2% of RRs are sex-specific. About half the RRs (52.0%) are adjusted for one or more variables. Of 1,488 adjusted RRs, age is adjusted for in 1,382 (92.9%) but only 490 (32.9%) are adjusted for variables other than age, sex or other smoking aspects. 34.0% of the RRs are given directly or calculated from a 2 × 2 or 2 × 2 × ℓ table, the rest being derived.

Of the 3,538 RRs, 1,439 are for major smoking indices, and 2,099 for dose-related indices (including 236 and 439 respectively in subsidiary studies). Of the 1,203 RRs in principal studies for major indices, 34.6% are for ever smoking, 37.8% current smoking and 27.6% ex smoking. 53.6% are for cigarette smoking ignoring other products, 33.8% any product smoking, and 12.6% cigarettes only. The unexposed group is typically never any product (55.8%) or never cigarettes (43.1%).

The distribution of smoking status for the 1,660 RRs in principal studies for dose-related indices differs considerably, with 22.8% for ever smoking, 59.6% current smoking and 17.6% ex smoking. Again, most (59.8%) RRs relate to cigarette smoking ignoring other products. The unexposed group is never smoking (any product or cigarettes) for 50.4% of these RRs, low smoking for 39.2%, and current smoking for 3.9%. 52.7% of RRs are for amount smoked, 8.1% age of starting, 19.8% pack-years, 4.4% years duration, and 15.1% years quit (about half compared to never smokers or long-term quitters, the rest compared to current smokers or short-term quitters). Based on RRs with an unexposed base of never smoking, there are 174 sets of categorical data for amount smoked, 18 for age of starting, 52 for pack-years, 11 for duration of smoking, and 26 for duration of quitting. For emphysema, there are few dose-related data other than for amount smoked

None of the RRs included in the meta-analyses and meta-regressions show more than minor failures of the validation tests used, attributable to rounding errors or small imprecisions or uncertainties in estimating the RRs and CIs. Additional File 3 provides further detail.

### The meta-analyses and meta-regressions

The main findings are summarized in the following sections, with tables and forest plots. Fuller results of the meta-analyses for the major smoking variables are given in Additional file 4 for COPD, Additional file 5 for CB and Additional file 6 for emphysema. Similar results for the dose-related smoking variables are given in Additional file 7 for COPD, Additional file 8 for CB and Additional file 9 for emphysema. An Excel file, available as Additional file 10, allows the user readily to view selected results from all these meta-analyses. Detailed meta-regression outputs are given in Additional file 11. For dose-related indices, Additional file 12 presents within-study plots of the dose-response relationships, while Additional file 13 gives results that were originally presented in a form unsuitable for meta-analysis. The interested reader should first refer to Additional file 1, which describes the content and structure of all these Additional files.

#### A. Risk from ever smoking

**Figure 1:**
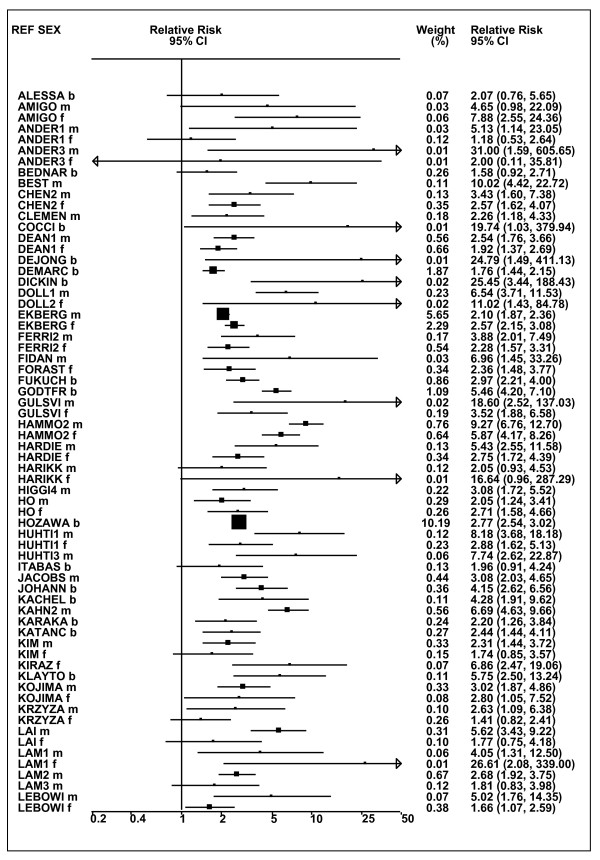
**Forest plot of ever smoking of any product and COPD-part 1**. Table 5 presents the results of a main meta-analysis for COPD based on 129 relative risk (RR) and 95% confidence interval (CI) estimates for ever smoking of any product (or cigarettes if any product not available). The individual study estimates are shown numerically and graphically on a logarithmic scale in Figures 1 and 2. The weights (inverse-variance) are also shown numerically, expressed as a percentage of the overall weight. The studies are sorted in order of sex within study reference (REF). In the graphical representation individual RRs are indicated by a solid square, with the area of the square proportional to the weight. Arrows indicate where the CI extends outside the range allocated. Where the RR value falls outside the range, the size of the plotting symbol indicates the weight but the position is not true to the scale.

**Figure 2:**
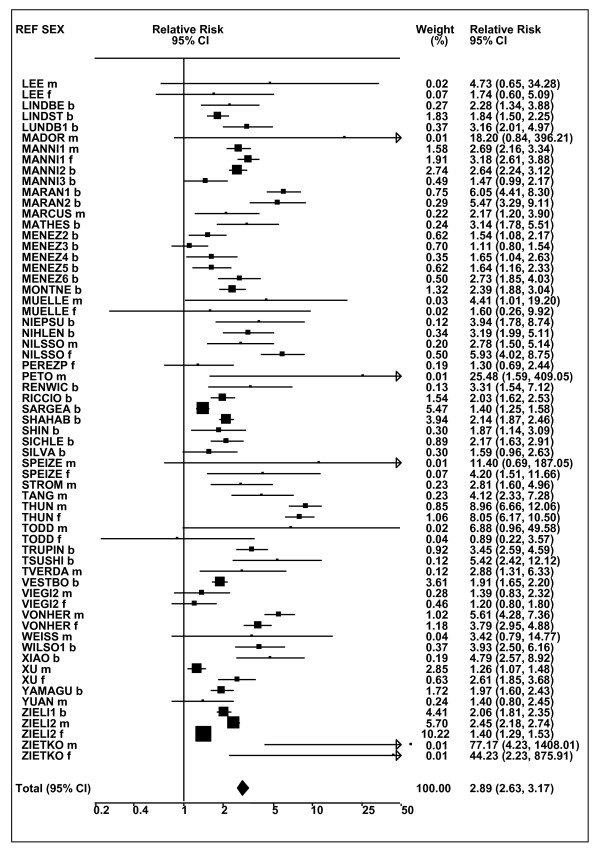
**Forest plot of ever smoking of any product and COPD-part 2**. This is a continuation of Figure 1, presenting the remaining individual study data included in the main meta-analysis for COPD shown in Table 5. Also shown are the combined random-effects estimates. These are represented by a diamond of standard height, with the width indicating the 95% CI.

Figures 1 and 2 (COPD), 3 and 4 (CB) and 5 (emphysema) present the results of the main meta-analyses for ever smoking any product (or cigarette smoking from studies without RRs for any product), based on most-adjusted RRs. Additional results subdivided by level of certain characteristics are shown in Table 5. From these findings, various observations can be made.

**Figure 3:**
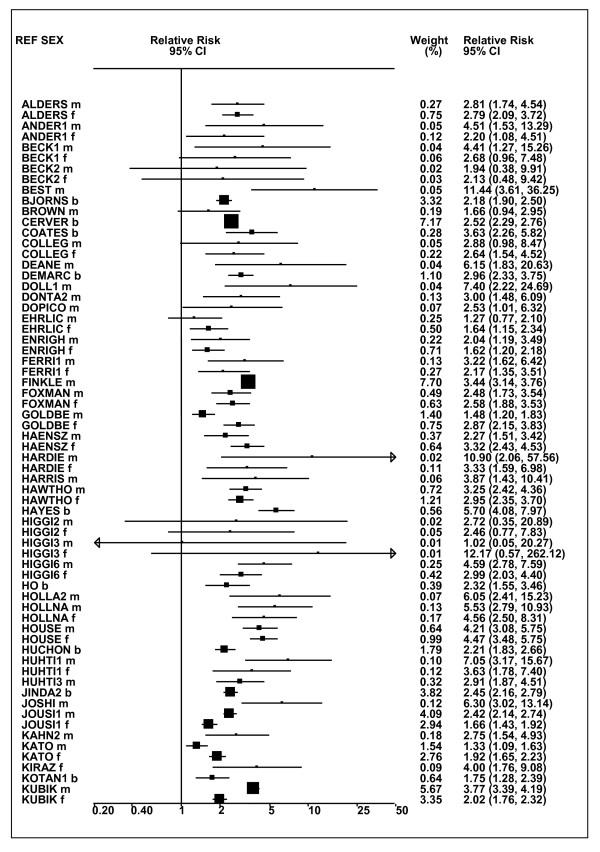
**Forest plot of ever smoking of any product and CB-part 1**. Table 5 presents the results of a main meta-analysis for CB based on 114 relative risk (RR) and 95% confidence interval (CI) estimates for ever smoking of any product (or cigarettes if any product not available). The individual study estimates are shown numerically and graphically on a logarithmic scale in Figures 3 and 4. The weights (inverse-variance) are also shown numerically, expressed as a percentage of the overall weight. The studies are sorted in order of sex within study reference (REF). In the graphical representation individual RRs are indicated by a solid square, with the area of the square proportional to the weight. Arrows indicate where the CI extends outside the range allocated.

**Figure 4:**
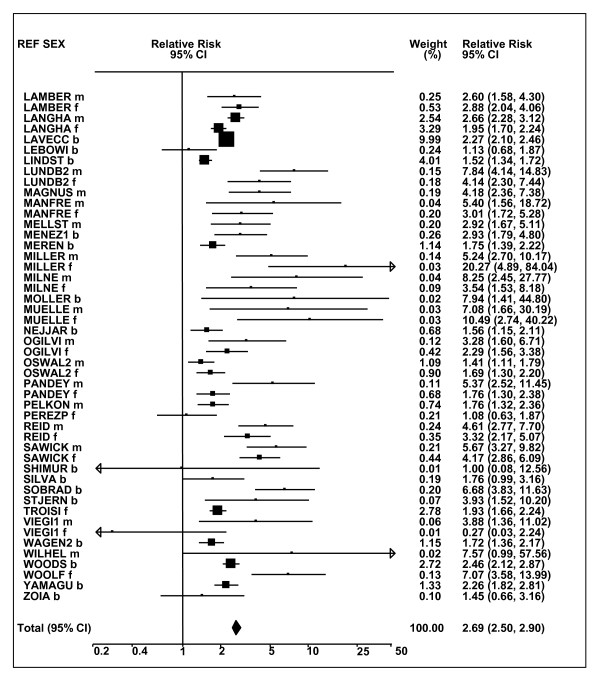
**Forest plot of ever smoking of any product and CB-part 2**. This is a continuation of Figure 3, presenting the remaining individual study data included in the main meta-analysis for CB shown in Table 5. Also shown are the combined random-effects estimates. These are represented by a diamond of standard height, with the width indicating the 95% CI.

**Figure 5:**
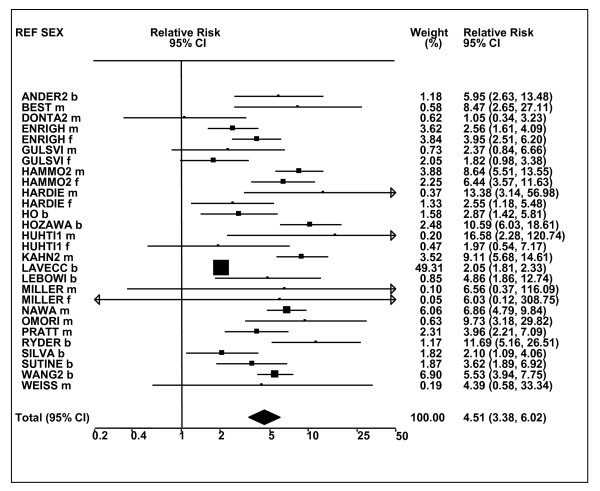
**Forest plot of ever smoking of any product and emphysema**. Table 5 presents the results of a main meta-analysis for emphysema based on 28 relative risk (RR) and 95% confidence interval (CI) estimates for ever smoking of any product (or cigarettes if any product not available). The individual study estimates are shown numerically and graphically on a logarithmic scale. The weights (inverse-variance) are also shown numerically, expressed as a percentage of the overall weight. The studies are sorted in order of sex within study reference (REF). In the graphical representation individual RRs are indicated by a solid square, with the area of the square proportional to the weight. Arrows indicate where the CI extends outside the range allocated. Also shown are the combined random-effects estimates. These are represented by a diamond of standard height, with the width indicating the 95% CI.

**Table 5:** Main meta-analyses for ever smoking of any product (or cigarettes if any product not available)^a^

Characteristic	Level	Statistic^b^	COPD	CB	Emphysema
All	All	n	129	114	28
		F	2.34 (2.27-2.40)	2.42 (2.36-2.48)	3.22 (2.95-3.52)
		R	2.89 (2.63-3.17)	2.69 (2.50-2.90)	4.51 (3.38-6.02)
		H, P_H_	8.11, < 0.001	5.81, < 0.001	6.42, < 0.001
Sex	Male	n	49	51	13
		F	2.69 (2.55-2.84)	2.87 (2.74-3.00)	5.71 (4.74-6.87)
		R	3.60 (2.98-4.34)	3.18 (2.77-3.65)	5.42 (3.69-7.96)
	Female	n	35	39	6
		F	2.16 (2.04-2.28)	2.22 (2.11-2.32)	3.44 (2.60-4.55)
		R	2.73 (2.17-3.43)	2.57 (2.28-2.89)	3.25 (2.07-5.10)
	Combined	n	45	24	9
		F	2.26 (2.17-2.35)	2.24 (2.15-2.33)	2.62 (2.36-2.92)
		R	2.51 (2.23-2.82)	2.29 (2.05-2.57)	4.47 (2.72-7.34)
	Between levels	P_B_	NS	< 0.001	< 0.05
Continent	N America	n	35	38	14
		F	3.21 (3.04-3.39)	2.91 (2.75-3.07)	5.33 (4.49-6.33)
		R	3.48 (2.88-4.20)	3.17 (2.70-3.71)	5.32 (3.86-7.32)
	Europe	n	59	60	10
		F	2.09 (2.01-2.17)	2.34 (2.27-2.41)	2.19 (1.95-2.46)
		R	2.79 (2.46-3.16)	2.62 (2.39-2.89)	3.13 (2.01-4.87)
	Asia	n	23	9	4
		F	2.26 (2.08-2.45)	2.08 (1.92-2.24)	5.76 (4.59-7.23)
		R	2.73 (2.15-3.46)	2.21 (1.78-2.75)	5.59 (3.93-7.95)
	Other or multicountry	n	12	7	0
		F	1.91 (1.71-2.14)	2.34 (2.09-2.61)	
		R	2.13 (1.66-2.73)	2.09 (1.59-2.74)	
	Between levels	P_B_	< 0.001	< 0.05	< 0.001
Publication year	Before 1980	n	25	49	13
		F	3.82 (3.40-4.29)	3.02 (2.86-3.18)	5.81 (4.74-7.12)
		R	3.79 (2.82-5.10)	3.22 (2.82-3.68)	5.37 (3.72-7.75)
	1980-89	n	10	28	5
		F	2.13 (1.80-2.50)	2.44 (2.33-2.56)	2.10 (1.86-2.37)
		R	2.34 (1.79-3.05)	2.72 (2.33-3.17)	2.39 (1.49-3.82)
	1990-99	n	19	17	3
		F	3.75 (3.36-4.18)	2.09 (1.96-2.22)	3.14 (2.34-4.22)
		R	3.31 (2.39-4.58)	2.24 (1.91-2.63)	3.14 (2.34-4.22)
	2000 or later	n	75	20	7
		F	2.19 (2.13-2.26)	2.19 (2.09-2.29)	5.76 (4.72-7.04)
		R	2.62 (2.36-2.90)	2.19 (1.93-2.48)	5.58 (3.63-5.58)
	Between levels	P_B_	< 0.001	< 0.001	< 0.001
Study type	Case-control	n	16	10	0
		F	2.51 (2.10-2.99)	2.35 (2.04-2.69)	
		R	2.95 (2.14-4.06)	2.72 (1.85-3.77)	
	Prospective	n	37	11	6
		F	4.49 (4.12-4.89)	2.30 (2.08-2.55)	6.12 (4.77-7.85)
		R	3.71 (2.94-4.69)	2.60 (2.04-3.30)	4.95 (2.72-8.99)
	Cross-sectional	n	76	93	22
		F	2.16 (2.10-2.22)	2.43 (2.36-2.49)	2.93 (2.67-3.22)
		R	2.54 (2.32-2.80)	2.70 (2.49-2.93)	4.36 (3.19-5.96)
	Between levels	P_B_	< 0.001	NS	< 0.05
Outcome subtype	Mortality	n	29	3	4
		F	4.50 (4.09-4.94)	4.10 (2.54-6.60)	8.24 (6.24-10.87)
		R	3.95 (3.00-5.21)	5.49 (2.14-14.11)	8.24 (6.24-10.87)
	Lung function (COPD) or symptoms (CB)	n	60	83	0
		F	2.16 (2.09-2.23)	2.51 (2.44-2.58)	
		R	2.35 (2.13-2.59)	2.78 (2.55-3.03)	
	Other	n	40	28	24
		F	2.31 (2.19-2.45)	2.11 (2.00-2.22)	2.89 (2.63-3.18)
		R	3.10 (2.56-3.76)	2.31 (2.01-2.65)	4.00 (2.97-5.37)
	Between levels	P_B_	< 0.001	< 0.05	< 0.01
How asthma taken into account	Ignored	n	76	103	26
		F	2.60 (2.52-2.70)	2.44 (2.38-2.50)	3.22 (2.94-3.52)
		R	2.95 (2.62-3.32)	2.69 (2.50-2.91)	4.61 (3.43-6.19)
	Excluded	n	21	7	2
		F	1.83 (1.73-1.94)	1.91 (1.71-2.13)	3.22 (1.45-7.11)
		R	2.35 (1.92-2.87)	2.13 (1.61-2.80)	3.20 (0.36-28.37)
	Included in outcome definition	n	19	-	-
		F	1.76 (1.61-1.92)		
		R	2.55 (2.00-3.26)		
	Other	n	13	4	0
		F	3.68 (3.20-4.24)	5.41 (3.58-8.18)	
		R	3.62 (2.80-4.69)	5.37 (3.13-9.21)	
	Between levels	P_B_	< 0.001	< 0.1	NS
Number of cases	1-50	n	23	17	6
		F	4.06 (3.12-5.29)	2.70 (1.98-3.69)	2.89 (1.78-4.67)
		R	4.45 (3.19-6.21)	2.70 (1.98-3.69)	2.89 (1.78-4.67)
	51-100	n	29	13	9
		F	2.42 (2.12-2.75)	3.71 (2.91-4.72)	2.87 (2.15-3.83)
		R	2.55 (2.09-3.12)	4.15 (2.97-5.80)	3.19 (2.10-4.85)
	101-200	n	30	19	5
		F	2.39 (2.18-2.63)	3.07 (2.66-3.53)	7.30 (5.31-10.03)
		R	2.69 (2.24-3.24)	3.48 (2.64-4.58)	7.48 (4.73-11.85)
	201+	n	47	65	8
		F	2.31 (2.24-2.38)	2.38 (2.32-2.44)	3.03 (2.74-3.24)
		R	2.91 (2.54-3.32)	2.51 (2.31-2.72)	4.96 (2.99-8.22)
	Between levels	P_B_	NS	NS	NS
Analysis type	Prevalence	n	93	105	22
		F	2.17 (2.11-2.23)	2.43 (2.37-2.50)	2.93 (2.67-3.22)
		R	2.57 (2.35-2.81)	2.71 (2.51-2.93)	4.36 (3.19-5.96)
	Onset	n	36	9	6
		F	4.55 (4.17-4.96)	2.02 (1.78-2.28)	6.12 (4.77-7.85)
		R	3.77 (2.98-4.78)	2.37 (1.80-3.13)	4.95 (2.72-8.99)
	Between levels	P_B_	< 0.001	NS	< 0.05
Smoking product	Any	n	48	61	11
		F	2.60 (2.47-2.74)	2.59 (2.51-2.68)	2.51 (2.24-2.80)
		R	2.99 (2.61-3.44)	2.89 (2.63-3.19)	4.16 (2.55-6.78)
	Cigarettes (ignoring other products)	n	73	51	15
		F	2.08 (2.01-2.15)	2.18 (2.10-2.26)	4.96 (4.26-5.78)
		R	2.48 (2.23-2.76)	2.44 (2.17-2.73)	4.58 (3.33-6.30)
	Cigarettes only	n	8	2	2
		F	6.38 (5.61-7.24)	3.03 (2.29-4.01)	6.69 (3.43-13.05)
		R	6.42 (4.22-9.78)	5.04 (1.29-19.70)	6.69 (3.43-13.05)
	Between levels	P_B_	< 0.001	< 0.05	< 0.01
Unexposed base^c^	Never any product	n	57	70	14
		F	2.95 (2.82-3.09)	2.61 (2.52-2.69)	2.75 (2.47-3.06)
		R	3.44 (2.95-4.01)	2.90 (2.65-3.18)	4.76 (3.02-7.50)
	Never cigarettes	n	72	44	14
		F	2.05 (1.98-2.12)	2.16 (2.07-2.24)	4.62 (3.93-5.43)
		R	2.47 (2.22-2.74)	2.42 (2.15-2.73)	4.28 (3.08-5.96)
	Between levels	P_B_	< 0.001	< 0.01	< 0.05
RR adjusted for age	Yes	n	69	63	15
		F	2.46 (2.35-2.56)	2.17 (2.10-2.24)	2.70 (2.43-3.00)
		R	2.97 (2.56-3.45)	2.46 (2.26-2.69)	4.44 (2.90-6.82)
	No	n	60	51	13
		F	2.26 (2.18-2.34)	2.78 (2.67-2.88)	4.91 (4.17-5.79)
		R	2.77 (2.46-3.12)	3.03 (2.69-3.41)	4.67 (3.45-6.33)
	Between levels	P_B_	NS	< 0.001	< 0.05
RR adjusted for factors other than age or sex	Yes	n	34	30	6
		F	2.09 (1.98-2.21)	2.28 (2.20-2.37)	2.11 (1.87-2.38)
		R	2.64 (2.23-3.13)	2.41 (2.14-2.71)	2.11 (1.87-2.38)
	No	n	95	84	22
		F	2.43 (2.35-2.51)	2.53 (2.45-2.61)	5.26 (4.62-6.00)
		R	3.00 (2.68-3.36)	2.86 (2.60-3.15)	5.02 (3.88-6.50)
	Between levels	P_B_	< 0.1	< 0.1	< 0.001

^a ^Within each study, results are selected in the following order or preference, within each sex, for: unexposed group-never any product, never cigarettes, other; smoking product-any, cigarettes (ignoring other products), cigarettes only; overlapping studies-principal, subsidiary; and then for single sex results in preference to combined sex results. Results adjusted for the most potential confounders are selected.

^b ^n = number of estimates combined, F = fixed-effect meta-analysis RR (95% CI), R = random-effects meta-analysis RR (95% CI), H = heterogeneity chisquared per degree of freedom, P_H _= probability value for heterogeneity expressed as p < 0.001, p < 0.05, p < 0.1 or NS (p ≥ 0.1), P_B _= probability value for between levels (see methods) similarly expressed.

^c ^Includes acceptable near-equivalent estimate (see methods) if estimate for strictly defined never smoker base not available (COPD: 3 for never cigarettes, CB: 2 for never any product and 4 for never cigarettes).

First, the RRs for all three outcomes are markedly heterogeneous, with H at least 5 for all three diseases (p < 0.001). Individual RRs vary up to 77.17 for COPD (study ZIETKO for males), 20.27 for CB (MILLER/females) and 16.58 for emphysema (HUHTI/males). Based on random-effects estimates, a positive association is seen, strongest for emphysema (RR 4.51, 95% CI 3.38-6.02, based on 28 RRs), but also clearly evident for COPD (2.89, 2.63-3.17, n = 129) and CB (2.69, 2.50-2.90, n = 114). Although the strength of association varies markedly by study, the consistency of direction is clear, with only one of the 129 COPD RRs, one of the 114 CB RRs, and none of the 28 emphysema RRs below 1.0.

These estimates are little affected by preferring RRs for ever smoking cigarettes to those for ever smoking any product, the random-effects estimates changing to 2.92 (2.65-3.20) for COPD, 2.70 (2.50-2.91) for CB and 4.57 (3.40-6.15) for emphysema. This is partly due to many studies providing only one type of RR, so that for COPD, for example, 117 of the 129 RRs are common to both meta-analyses. Nor are they affected by preferring least-adjusted, rather than most-adjusted RRs, with the estimates now 2.85 (2.59-3.15, n = 133) for COPD, 2.73 (2.52-2.95, n = 119) for CB and 4.16 (3.03-5.71, n = 30) for emphysema, slightly more RRs being included as some studies have sex-specific unadjusted RRs but only sexes combined adjusted RRs.

Returning to the main meta-analysis (most-adjusted and preferring ever smoking any product), there is also large variation between RRs in the weight they contribute to the analysis. For COPD, of a total weight of 5,116 for the 129 RRs (mean 39.7), the largest weight is 523 for study ZIELI2 for females, with six other RRs having weights of over 200. For CB, of the total of 6,146 for the 114 RRs (mean 53.9), the largest weight is 614 for study LAVECC for sexes combined, with eight other RRs having weights over 200. For emphysema, where the total weight is much lower, 489 (mean 17.5 for the 28 RRs), the weight of 241 for LAVECC for the sexes combined RR contributes almost a half.

In investigating sources of heterogeneity, variation was studied firstly using a univariate approach, the results for the characteristics considered in Table 5 being summarized below.

##### Sex

RRs for males are highest for all three outcomes, with the variation by sex significant for CB (p < 0.001) and emphysema (p < 0.05).

##### Continent

There is significant variation by continent for COPD (p < 0.001), CB (p < 0.05) and emphysema (p < 0.001). For COPD and CB, RRs are higher for North America than for Europe, Asia or other countries (including multicentre results). For emphysema, RRs are again relatively low for Europe, though RRs for North America and Asia are similar.

##### Publication year

For all three outcomes, there is significant (p < 0.001) variation by publication year. Though there is some indication that RRs are relatively high for studies published before 1980, the pattern is erratic for both COPD (high for pre-1980 and 1990-99, low for 1980-89 and 2000 or later) and emphysema (high for pre-1980 and 2000 or later, low for 1980-89 and 1990-99).

##### Study type

For COPD, there is marked variation (p < 0.001), with RRs higher for prospective studies than for other study designs. For CB, no variation is evident, most RRs coming from cross-sectional studies. For emphysema, where no results come from case-control studies, RRs are again higher in prospective studies, particularly for the fixed-effect estimates (p < 0.05).

##### Outcome subtype

For all three outcomes, the estimates are substantially higher when based on mortality, although for CB and emphysema few RRs are so based. For COPD, the random-effects estimates of 3.95 (3.00-5.21, n = 29) based on mortality, and 2.35 (2.13-2.59, n = 60) based on lung function, differ substantially.

##### How asthma was taken into account

For COPD, the random-effect estimates tend to be lower when asthmatics are excluded (2.35, 1.92-2.87, n = 21) or when asthma is included as part of the definition (2.55, 2.00-3.26, n = 19), than when it is ignored (2.95, 2.62-3.32, n = 76) or is taken into account in other ways or it is unclear whether the definition of the outcome includes asthma or not (3.62, 2.80-4.69, n = 13). For CB and emphysema, the great majority of RRs come from studies where the comparison is made irrespective of asthma.

##### Study size

There is no convincing evidence that RRs vary according to the number of cases of the outcome that are studied.

##### Analysis type

For COPD, RRs based on onset are clearly higher than those based on prevalence (p < 0.001). A similar tendency is seen for emphysema (p < 0.05), though only six RRs are based on onset. For CB, where again nearly all RRs are based on prevalence, no difference is seen by analysis type.

##### Smoking product

For COPD, a clear difference is seen by definition of smoking product (p < 0.001), with random-effects estimates of 6.42 (4.22-9.78, n = 8) for cigarette only smoking, 2.48 (2.23-2.76, n = 73) for cigarettes ignoring other products, and 2.99 (2.61-3.44, n = 48) for any product. For CB and emphysema, RRs based on cigarette only smoking are few, and the pattern less clear.

##### Unexposed base group

For COPD, RRs are lower when the comparison group is never cigarettes (so that smokers of other products only may be included in the denominator) than when it is never any product (p < 0.001). For CB, there is a smaller difference in the same direction (p < 0.01). For emphysema, however, fixed-effect estimates are lower when the comparison group is never any product (p < 0.05), but this difference is reversed when random-effects estimates are used.

##### Age-adjustment

For COPD, there is no difference in RRs based on age-adjusted or age-unadjusted RRs. For CB (p < 0.001) and emphysema (p < 0.05), however, adjusted RRs are lower.

##### Adjustment for factors other than age or sex

For all three outcomes, there is a tendency for RRs adjusted for other factors to be lower than those that are not so adjusted (p < 0.1 for COPD and CB, p < 0.001 for emphysema).

Variation by other characteristics (see Additional file 10) was also studied. For no outcome is there any clear evidence that RRs varied by the type of tobacco (blended or Virginia) typically used in the country where the study was conducted, by the lowest, or highest, age of subjects included in the study, by presence of the study weaknesses defined in Table 2, by whether the outcome was assessed using a bronchodilator (only relevant to COPD), or by whether the RR was directly available, derived from 2 × 2 tables provided, or using more complex methods. Differences are seen by start year of the study, but, like publication year, they do not follow any clear pattern over time. For emphysema, estimates are higher (p < 0.001) for studies providing RRs only for ever smoking than studies providing RRs for both ever smoking and current smoking, with random-effects estimates, respectively, 5.51 (4.08-7.43, n = 11) and 3.77 (2.63-5.42, n = 17). Sexes combined RRs tend to be lower if adjusted for sex for COPD (p < 0.05) and emphysema (p < 0.001), but not for CB. RRs adjusted for more factors tend to be lower for COPD (p < 0.01), CB (p < 0.01) and emphysema (p < 0.001). This is unsurprising given the results already noted for adjustment for age and for factors other than age or sex.

For COPD, the relationship to the characteristics was also studied separately for three subtypes of outcome-based on mortality (31 RRs), on lung function (62 RRs) and on other definitions (42 RRs). The tendency for RRs to be higher for North American studies is clearest when outcome is based on mortality, also evident when based on lung function only, but not evident when based on other definitions. The relationship of risk to study type cannot usefully be studied as nearly all relevant mortality studies are prospective, and nearly all other studies are cross-sectional. Similarly most data from mortality studies are of onset, whereas most data from other studies are of prevalence. The higher RRs noted in the overall results for smoking of cigarettes only are also evident solely in the mortality studies, as no RRs for this exposure are included for the other COPD subtypes. There is, however, a consistent tendency for all subtypes for RRs to be higher when the comparison group is never smoking of any product than when it is never smoking of cigarettes.

As only three CB RRs based on mortality are included, the relationship to the characteristics for CB is only studied separately for two subtypes-outcomes based on symptoms (83 RRs), and other than on mortality or symptoms (28 RRs). Tendencies noted in Table 5 for RRs to be higher in males than females, lower if adjusted for age than if unadjusted, and lower if the unexposed base group is never cigarettes than if it is never any product, are apparent for both subtypes.

For emphysema, the relationship to the characteristics separated by subtype of outcome cannot usefully be studied due to limited numbers, with four of the 28 RRs being based on mortality, and 24 based on other definitions.

In an attempt to evaluate the independent role of the characteristics, meta-regression analyses were conducted for COPD and CB, the results from the basic model being summarized in Table 6. There are too few data points for emphysema for useful meta-regression analysis, especially since almost half the total weight comes from one study (LAVECC).

**Table 6:** Meta-regression results for ever smoking of any product (or cigarettes if any product not available)^a^

		COPD		CB	
			
Characteristic	Level	Estimate^b ^(SE^c^)	p^d^	Estimate^b ^(SE^c^)	p^d^
Constant		+1.149 (0.141)		+1.316 (0.266)	
Sex	Male	Base	< 0.05	Base	< 0.1
	Female	-0.212 (0.044)		-0.171 (0.036)	
	Combined	-0.006 (0.039)		-0.121 (0.077)	
Continent	N America	Base	< 0.05	Base	< 0.01
	Europe	-0.200 (0.040)		-0.278 (0.039)	
	Asia	-0.295 (0.067)		-0.281 (0.060)	
	Other	-0.355 (0.072)		-0.177 (0.074)	
Outcome subtype	Mortality	Base	< 0.01	Base	NS
	Lung function (COPD) or symptoms (CB)	-0.404 (0.092)		-0.212 (0.249)	
	Other	-0.114 (0.085)		-0.302 (0.248)	
How asthma taken into account	Ignored	Base	< 0.001	Base	< 0.05
	Excluded	-0.143 (0.043)		-0.109 (0.066)	
	Included in outcome definition	-0.461 (0.060)		No data	
	Other	+0.283 (0.093)		+0.996 (0.214)	
Smoking product	Any	Base	< 0.01	Base	NS
	Cigarettes (ignoring other products)	+0.428 (0.128)		-0.060 (0.128)	
	Cigarettes only	+0.589 (0.107)		+0.449 (0.149)	
Unexposed group	Never any product	Base	< 0.05	Base	NS
	Never cigarettes	-0.603 (0.123)		-0.111 (0.126)	
RR adjusted for age	Yes	Base	NS	Base	< 0.01
	No	+0.046 (0.045)		+0.214 (0.032)	
RR adjusted for factor other than age or sex	Yes	Base	< 0.1	Base	NS
	No	+0.195 (0.052)		-0.113 (0.070)	
Midpoint age	Per 10 years	+0.003 (0.002)	NS	+0.003 (0.001)	NS

^a ^Based on the same data as for Table 5. See that table for further definition of RRs selected for analysis, and numbers of estimates of each characteristic level.

^b ^Estimates are of log RR. For a particular entry, the predicted RR for a given estimate is calculated by adding the constant to the values for each level applicable to the estimate (taking the value for the base level as zero) and taking the exponential of the result.

^c ^SE = standard error.

^d ^The p value is estimated from the drop in deviance from removing the characteristic from the model using an F-test. It is expressed as p < 0.001, p < 0.01, p < 0.05, p < 0.1 or NS (p ≥ 0.1).

For COPD the deviance reduces from 1,038.1 on 128 degrees of freedom to 421.8 on 112 degrees of freedom on fitting the basic model, substantially reducing, but not eliminating, the heterogeneity. The results in Table 6 demonstrate an independent role of six characteristics noted in the univariate analyses: sex (lower RRs for females), continent (higher for North America), smoking product (higher for cigarette smokers than smokers of any product), the unexposed base (higher for never any product than never cigarettes), and particularly the outcome subtype (lower when based on lung function), and the way asthma is taken into account (lowest when asthma is included in the COPD definition). Effects of adjustment and of age are not clearly seen, however. For none of the secondary characteristics do their inclusion into the model significantly improve the fit (at p < 0.05). This includes study type and analysis type, which are highly significant (p < 0.001) in the univariate analyses shown in Table 5. Both these are highly correlated with outcome subtype-thus where mortality is the outcome, the study type will nearly always be prospective, and the analysis type will nearly always be onset.

Inspection of standardized residuals from the basic model for COPD reveals two estimates where the observed RR differ markedly from the RR fitted by the model. The largest residual of -3.49 is for males in study XU, where an observed RR of 1.26 compares with a fitted RR of 2.20. The corresponding RRs for females are 2.61 observed and 1.78 fitted, with a residual of +1.12. For study GODTFR, sexes combined, the observed RR of 5.46 compares with a fitted value of 2.79, with a residual of +2.58. Other residuals are all less than +/- 2.20.

For CB the deviance reduces from 657.1 on 113 degrees of freedom to 433.3 on 103 degrees of freedom on fitting the basic model, again substantially reducing, but not eliminating, the heterogeneity. Though the direction of differences by level of the various characteristics is quite similar to that for COPD, the effects of individual characteristics are less clear, with significant differences (at p < 0.05) only for continent, how asthma was taken into account, and age-adjustment. No secondary characteristics help to improve the model fit (at p < 0.05), except for publication year, where a tendency is seen for earlier published studies to provide higher RRs.

The largest standardized residual from the basic model for CB, -2.74, is for males in study GOLDBE, where the observed RR of 1.48 compares to a fitted RR of 2.69, corresponding RRs for females being 2.87 observed and 2.27 fitted, with a residual of +0.79. Another large residual, -2.53, is for females in study JOUSI1, where the observed RR of 1.66 compares to a fitted value of 2.43, with the corresponding RRs for males being 2.42 observed and 2.88 fitted, with a residual of -1.36. Other residuals are all less than +/- 2.20.

#### B. Risk from current smoking

**Figure 6:**
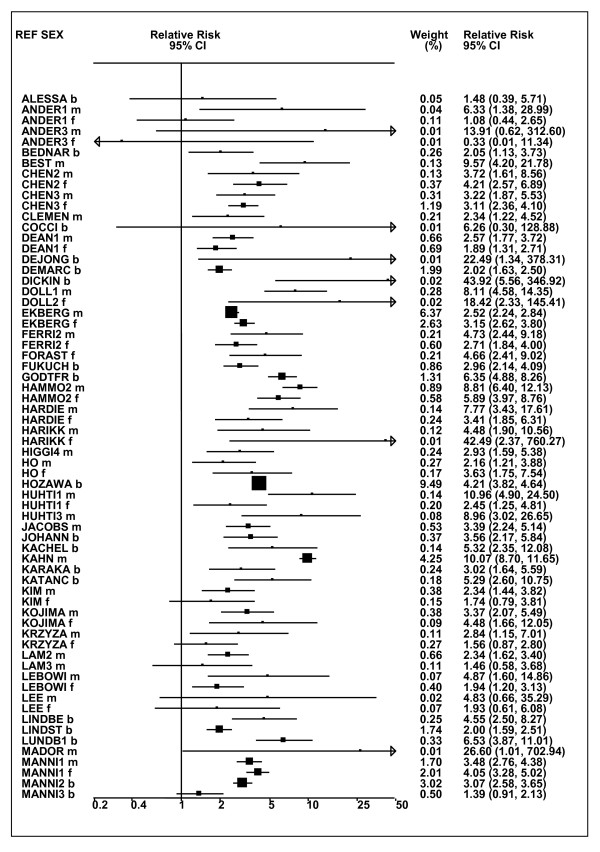
**Forest plot of current smoking of any product and COPD-part 1**. Table 7 presents the results of a main meta-analysis for COPD based on 120 relative risk (RR) and 95% confidence interval (CI) estimates for current smoking of any product (or cigarettes if any product not available). The individual study estimates are shown numerically and graphically on a logarithmic scale in Figures 6 and 7. The weights (inverse-variance) are also shown numerically, expressed as a percentage of the overall weight. The studies are sorted in order of sex within study reference (REF). In the graphical representation individual RRs are indicated by a solid square, with the area of the square proportional to the weight. Arrows indicate where the CI extends outside the range allocated.

**Figure 7:**
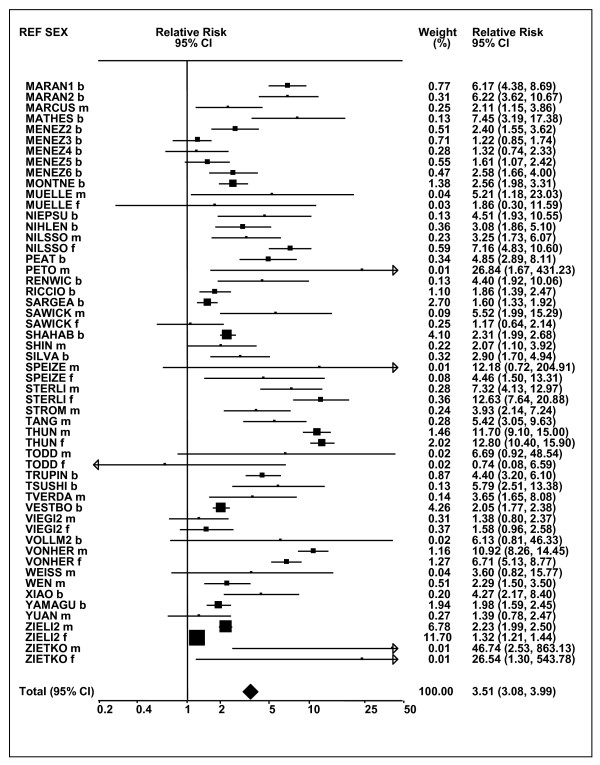
**Forest plot of current smoking of any product and COPD-part 2**. This is a continuation of Figure 6, presenting the remaining individual study data included in the main meta-analysis for COPD shown in Table 7. Also shown are the combined random-effects estimates. These are represented by a diamond of standard height, with the width indicating the 95% CI.

Figures 6 and 7 (COPD), 8 and 9 (CB) and 10 (emphysema) present the results of the main meta-analyses for current smoking of any product. As before, RRs for smoking of cigarettes are used if RRs for any product smoking are not available, and RRs are most-adjusted. Some results by levels of characteristics studied are shown in Table 7.

**Figure 8:**
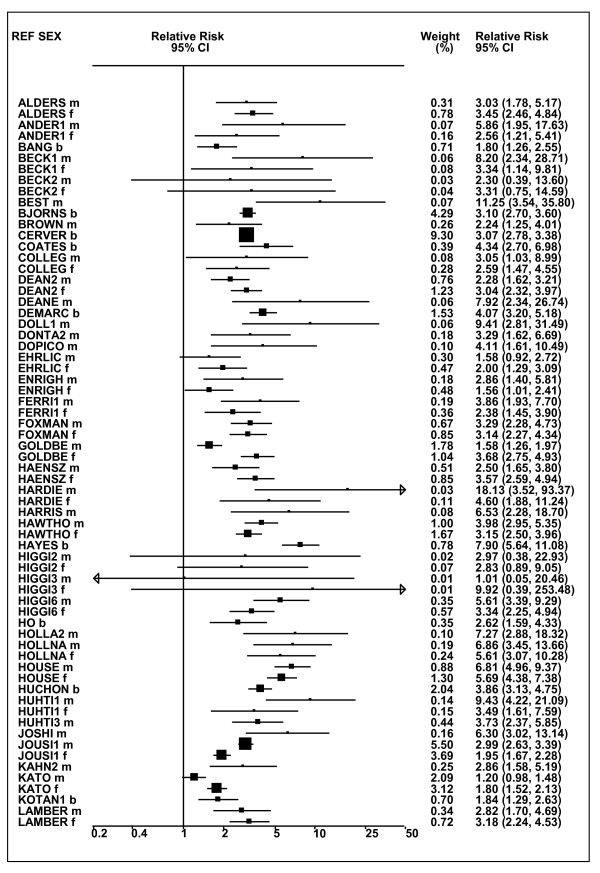
**Forest plot of current smoking of any product and CB-part 1**. Table 7 presents the results of a main meta-analysis for CB based on 113 relative risk (RR) and 95% confidence interval (CI) estimates for current smoking of any product (or cigarettes if any product not available). The individual study estimates are shown numerically and graphically on a logarithmic scale in Figures 8 and 9. The weights (inverse-variance) are also shown numerically, expressed as a percentage of the overall weight. The studies are sorted in order of sex within study reference (REF). In the graphical representation individual RRs are indicated by a solid square, with the area of the square proportional to the weight. Arrows indicate where the CI extends outside the range allocated.

**Figure 9:**
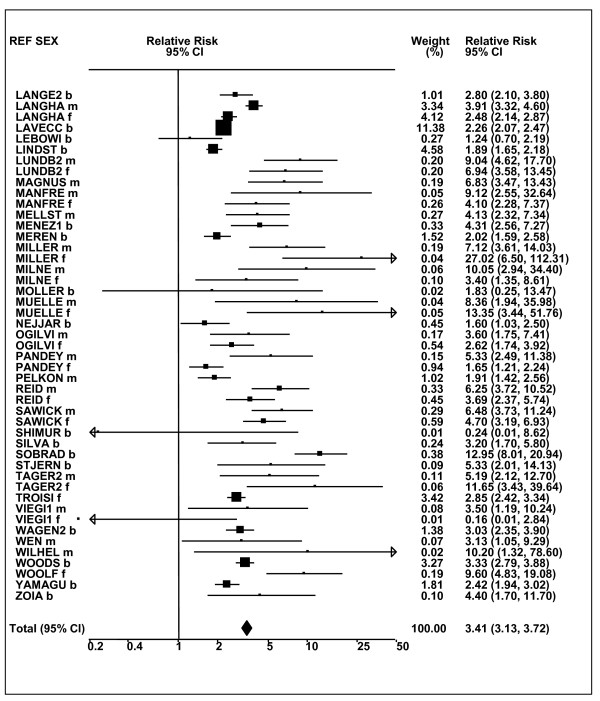
**Forest plot of current smoking of any product and CB-part 2**. This is a continuation of Figure 8, presenting the remaining individual study data included in the main meta-analysis for CB shown in Table 7. Also shown are the combined random-effects estimates. These are represented by a diamond of standard height, with the width indicating the 95% CI.

**Figure 10:**
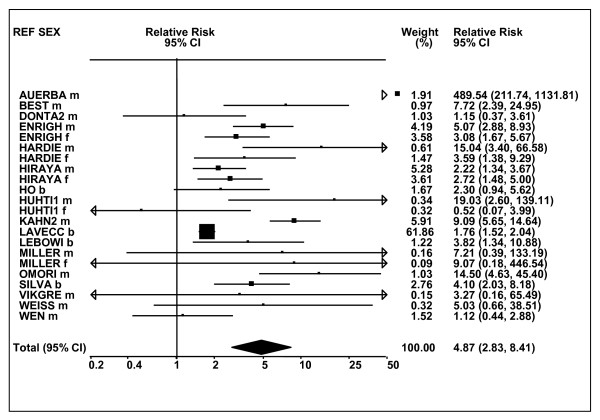
**Forest plot of current smoking of any product and emphysema**. Table 7 presents the results of a main meta-analysis for emphysema based on 22 relative risk (RR) and 95% confidence interval (CI) estimates for current smoking of any product (or cigarettes if any product not available). The individual study estimates are shown numerically and graphically on a logarithmic scale. The weights (inverse-variance) are also shown numerically, expressed as a percentage of the overall weight. The studies are sorted in order of sex within study reference (REF). In the graphical representation individual RRs are indicated by a solid square, with the area of the square proportional to the weight. Arrows indicate where the CI extends outside the range allocated. Where the RR value falls outside the range, the size of the plotting symbol indicates the weight but the position is not true to the scale. Also shown are the combined random-effects estimates. These are represented by a diamond of standard height, with the width indicating the 95% CI.

**Table 7:** Main meta-analyses for current smoking of any product (or cigarettes, if any product not available)^a^

Characteristic	Level	Statistic^b^	COPD	CB	Emphysema
All	All	N	120	113	22
		F	3.00 (2.91-3.09)	2.82 (2.74-2.90)	2.61 (2.33-2.93)
		R	3.51 (3.08-3.99)	3.41 (3.13-3.72)	4.87 (2.83-8.41)
		H, P_H_	13.81, < 0.001	5.80, < 0.001	11.54, < 0.001
Sex	Male	N	48	51	13
		F	3.80 (3.60-4.02)	3.08 (2.90-3.28)	6.71 (5.29-8.53)
		R	4.11 (3.28-5.15)	4.07 (3.44-4.83)	7.66 (3.00-19.61)
	Female	n	31	37	5
		F	2.53 (2.39-2.68)	2.75 (2.60-2.90)	2.85 (1.94-4.18)
		R	3.28 (2.35-4.58)	3.23 (2.80-3.72)	2.85 (1.94-4.18)
	Combined	n	41	25	4
		F	2.81 (2.69-2.95)	2.74 (2.62-2.86)	1.86 (1.62-2.14)
		R	3.04 (2.60-3.55)	2.98 (2.57-3.47)	2.54 (1.54-4.20)
	Between levels	P_B_	< 0.05	NS	< 0.05
Continent	N America	n	39	40	10
		F	5.02 (4.76-5.29)	3.44 (3.20-3.69)	8.13 (6.32-10.45)
		R	4.56 (3.69-5.62)	4.11 (3.41-4.97)	8.99 (3.34-24.26)
	Europe	n	55	58	7
		F	2.31 (2.22-2.41)	2.79 (2.69-2.89)	1.83 (1.58-2.11)
		R	3.31 (2.80-3.92)	3.28 (2.96-3.62)	2.88 (1.36-6.09)
	Asia	n	17	9	5
		F	2.73 (2.45-3.05)	1.83 (1.65-2.02)	2.52 (1.83-3.47)
		R	2.86 (2.27-3.60)	2.26 (1.67-3.05)	2.74 (1.50-4.99)
	Other or multicountry	n	9	6	0
		F	2.15 (1.89-2.44)	3.32 (2.94-3.75)	
		R	2.37 (1.75-3.21)	3.11 (2.33-4.16)	
	Between levels	P_B_	< 0.001	< 0.001	< 0.01
Publication year	Before 1980	n	24	50	7
		F	5.64 (5.12-6.21)	3.59 (3.36-3.84)	15.01 (10.59-21.28)
		R	3.81 (2.64-5.50)	3.96 (3.41-4.59)	10.21 (2.09-49.96)
	1980-89	n	11	26	6
		F	2.18 (1.84-2.59)	2.29 (2.16-2.44)	1.83 (1.59-2.10)
		R	2.47 (1.87-3.27)	3.25 (2.67-3.96)	1.83 (1.59-2.10)
	1990-99	n	18	17	3
		F	6.20 (5.59-6.88)	2.74 (2.56-2.93)	3.65 (2.50-5.31)
		R	4.91 (3.37-7.16)	3.04 (2.51-3.68)	3.57 (2.30-5.55)
	2000 or later	n	67	20	6
		F	2.60 (2.52-2.69)	2.86 (2.72-3.01)	4.04 (2.65-6.16)
		R	3.13 (2.73-3.60)	2.94 (2.50-3.47)	4.57 (2.05-10.20)
	Between levels	P_B_	< 0.001	< 0.001	< 0.01
Study type	Case-control	n	14	9	0
		F	3.72 (3.09-4.48)	3.39 (2.90-3.96)	
		R	4.69 (2.83-7.77)	3.66 (2.77-4.83)	
	Prospective	n	38	12	8
		F	6.46 (6.01-6.95)	2.96 (2.67-3.29)	3.61 (2.81-4.64)
		R	4.34 (3.38-5.56)	3.12 (2.56-3.78)	3.12 (1.74-5.60)
	Cross-sectional	n	68	92	14
		F	2.53 (2.45-2.62)	2.78 (2.70-2.87)	2.39 (2.10-2.73)
		R	3.00 (2.63-3.41)	3.42 (3.10-3.77)	6.60 (2.74-15.92)
	Between levels	P_B_	< 0.001	NS	NS
Outcome subtype	Mortality	n	31	4	5
		F	6.57 (6.09-7.10)	4.16 (2.67-6.48)	3.79 (2.87-5.00)
		R	4.57 (3.39-6.15)	4.96 (2.43-10.12)	3.42 (1.60-7.35)
	Lung function (COPD) or symptoms (CB)	n	56	81	0
		F	2.41 (2.32-2.50)	3.06 (2.95-3.17)	
		R	2.79 (2.42-3.20)	3.63 (3.29-3.99)	
	Other	n	33	28	17
		F	3.45 (3.21-3.70)	2.24 (2.12-2.37)	2.42 (2.13-2.74)
		R	3.77 (3.01-4.72)	2.69 (2.26-3.21)	5.56 (2.65-11.68)
	Between levels	P_B_	< 0.001	< 0.001	NS
How asthma taken into account	Ignored	n	72	103	19
		F	3.78 (3.64-3.92)	2.80 (2.72-2.89)	2.59 (2.30-2.91)
		R	3.60 (3.08-4.20)	3.38 (3.08-3.70)	5.02 (2.80-9.00)
	Excluded	n	17	6	3
		F	1.79 (1.69-1.90)	2.85 (2.53-3.21)	4.03 (1.85-8.77)
		R	3.05 (2.34-3.97)	3.30 (2.39-4.54)	3.90 (0.57-26.55)
	Included in outcome definition	n	18	-	-
		F	2.11 (1.88-2.37)		
		R	2.73 (2.10-3.55)		
	Other	n	13	4	0
		F	4.00 (3.42-4.67)	6.93 (4.39-10.92)	
		R	4.03 (2.96-5.48)	5.70 (2.63-12.37)	
	Between levels	P_B_	< 0.001	NS	NS
Number of cases	1-50	n	21	18	8
		F	4.62 (3.42-6.24)	3.85 (2.81-5.28)	2.09 (1.31-3.34)
		R	4.90 (3.34-7.18)	3.85 (2.81-5.28)	2.09 (1.31-3.34)
	51-100	n	23	14	6
		F	3.08 (2.64-3.60)	4.92 (3.84-6.30)	4.83 (3.06-7.61)
		R	3.21 (2.48-4.16)	5.28 (3.88-7.03)	5.14 (2.53-10.45)
	101-200	n	29	19	1
		F	2.61 (2.35-2.89)	3.93 (3.41-4.52)	14.50 (4.63-45.41)
		R	2.92 (2.39-3.57)	4.71 (3.37-6.57)	14.50 (4.63-45.41)
	201+	n	47	62	7
		F	3.02 (2.92-3.12)	2.74 (2.66-2.83)	2.48 (2.19-2.81)
		R	3.66 (3.02-4.43)	3.06 (2.78-3.27)	6.62 (2.44-18.00)
	Between levels	P_B_	NS	< 0.05	NS
Analysis type	Prevalence	n	84	103	14
		F	2.57 (2.48-2.65)	2.82 (2.74-2.91)	2.39 (2.10-2.73)
		R	3.14 (2.78-3.56)	3.44 (3.14-3.77)	6.60 (2.74-15.92)
	Onset	n	36	10	8
		F	6.58 (6.11-7.09)	2.75 (2.42-3.13)	3.61 (2.81-4.64)
		R	4.40 (3.41-5.69)	3.00 (2.31-3.89)	3.12 (1.74-5.60)
	Between levels	P_B_	< 0.001	NS	NS
Smoking product	Any	n	46	57	5
		F	3.09 (2.92-3.26)	2.84 (2.72-2.96)	2.05 (1.78-2.36)
		R	3.67 (3.05-4.40)	3.54 (3.17-3.97)	3.84 (1.20-12.22)
	Cigarettes (ignoring other products)	n	66	52	16
		F	2.67 (2.57-2.77)	2.79 (2.67-2.91)	4.37 (3.54-5.40)
		R	3.06 (2.60-3.60)	3.27 (2.84-3.76)	5.11 (2.45-10.65)
	Cigarettes only	n	8	4	1
		F	8.51 (7.55-9.59)	3.27 (2.55-4.20)	7.72 (2.39-24.94)
		R	7.47 (4.63-12.05)	3.36 (2.24-5.05)	7.72 (2.39-24.94)
	Between levels	P_B_	< 0.001	NS	NS
Unexposed base^c^	Never any product	n	58	69	7
		F	4.07 (3.89-4.26)	2.89 (2.77-3.01)	2.41 (2.11-2.77)
		R	4.27 (3.51-5.19)	3.56 (3.20-3.96)	8.93 (1.83-43.50)
	Never cigarettes	n	62	44	15
		F	2.34 (2.25-2.44)	2.74 (2.63-2.87)	3.18 (2.56-3.96)
		R	2.87 (2.48-3.31)	3.21 (2.77-3.72)	3.30 (2.39-4.56)
	Between levels	P_B_	< 0.001	NS	NS
RR adjusted for age	Yes	n	63	65	13
		F	3.47 (3.30-3.65)	2.57 (2.48-2.67)	2.52 (2.23-2.85)
		R	3.66 (3.07-4.36)	3.05 (2.74-3.39)	5.77 (2.77-12.02)
	No	n	57	48	9
		F	2.76 (2.66-2.87)	3.33 (3.17-3.51)	3.57 (2.49-5.14)
		R	3.34 (2.76-4.04)	4.10 (3.57-4.71)	3.35 (1.90-5.91)
	Between levels	P_B_	< 0.1	< 0.001	NS
RR adjusted for factors other than age or sex	Yes	n	27	29	6
		F	2.77 (2.61-2.94)	2.77 (2.65-2.88)	1.87 (1.62-2.15)
		R	3.17 (2.65-3.80)	2.97 (2.57-3.44)	2.62 (1.65-4.17)
	No	n	93	84	16
		F	3.09 (2.98-3.20)	2.87 (2.75-3.00)	5.27 (4.30-6.46)
		R	3.66 (3.11-4.31)	3.68 (3.29-4.12)	5.57 (2.67-11.62)
	Between levels	P_B_	NS	NS	< 0.05

^a ^Within each study, results are selected in the following order or preference, within each sex, for: unexposed group-never any product, never cigarettes, other; smoking product-any, cigarettes (ignoring other products), cigarettes only; overlapping studies-principal, subsidiary; and then for single sex results in preference to combined sex results. Results adjusted for the most potential confounders are selected.

^b ^n = number of estimates combined, F = fixed-effect meta-analysis RR (95% CI), R = random-effects meta-analysis RR (95% CI), H = heterogeneity chisquared per degree of freedom, P_H _= probability value for heterogeneity expressed as p < 0.001, p < 0.05, p < 0.1 or NS (p ≥ 0.1), P_B _= probability value for between levels (see methods) similarly expressed.

^c ^Includes acceptable near-equivalent estimate (see methods) if estimate for strictly defined never smoker base not available (COPD: 3 for never cigarettes, CB: 2 for never any product and 4 for never cigarettes).

As for ever smoking, the RRs for COPD, CB and emphysema are heterogeneous (p < 0.001), with the largest seen being 43.92 for COPD (DICKIN/sexes combined), 27.02 for CB (MILLER/females), and a remarkable 489.54, with lower 95% CL 211.74, for emphysema (AUERBA/males). The random-effects estimates (COPD 3.51, 95% CI 3.08-3.99, n = 120; CB, 3.41, 3.13-3.72, n = 113; emphysema 4.87, 2.83-8.41, n = 22) are all clearly positive, and somewhat larger than the corresponding estimates for ever smoking. Similarly to ever smoking, the individual RRs are virtually all above 1.0, though varying substantially. The estimates are also little affected by preferring RRs for current smoking of cigarettes to those for current smoking of any product, the random-effects estimates changing to 3.59 (3.15-4.09) for COPD, 3.45 (3.17-3.77) for CB and 5.00 (2.87-8.72) for emphysema. The estimates are again little affected by preferring least, rather than most, adjusted RRs, with the estimates now 3.41 (3.00-3.87) for COPD, 3.43 (3.12-3.77) for CB and 4.32 (2.40-7.78) for emphysema.

For the main meta-analysis, the studies contributing the most to the total weight are the same as for the corresponding meta-analysis for ever smoking: ZIELI2/females for COPD (11.7% of the total of 4,226), and LAVECC/sexes combined for CB (11.4% of 4,326) and emphysema (61.9% of 287).

For the characteristics considered in Table 7 the pattern of variation seems quite similar to that for ever smoking in Table 5. Thus, as for ever smoking, RRs tend to be higher for males and for North American studies for all three outcomes, higher for prospective studies for COPD, and higher when based on mortality for COPD and CB, with no obvious variation by study size, and an erratic pattern for publication year. RRs also show a similar pattern by how asthma is taken into account for COPD to that seen for ever smoking, and are again higher when based on onset for COPD, higher for cigarette only smoking for COPD, higher when the unexposed group is never smoked any product for COPD, and lower for RRs unadjusted for age for CB. As for ever smoking, variation in RRs by other characteristics (not shown in Table 7) was also studied. For most of these there seems little evidence of any difference. For COPD, there is a tendency (p < 0.001) for estimates to be higher for studies providing RRs only for current smoking than for studies providing RRs for both ever smoking and current smoking, with random-effects estimates, respectively, 4.52 (2.69-7.59, n = 10) and 3.40 (3.00-3.87, n = 110), but no such differences are seen for CB and emphysema. Compared to the results for ever smoking, there seems less clear evidence of an effect of adjustment, except as already noted for adjustment for age for CB (Table 7).

For COPD, the relationship to the characteristics was also studied separately for outcomes based on mortality (33 RRs), based only on lung function (58 RRs) and based on other definitions (36 RRs). As for ever smoking, risk is higher in North American studies when the outcome is based on mortality or lung function, but not when based on other definitions. Also as for ever smoking, and for reasons stated in the previous section, variation cannot usefully be studied by study type, or by analysis type (onset or prevalence), or in relation to smoking of cigarettes only. Again RRs are consistently higher for all the outcome subtypes when the comparison group is never smoking of any product than when it is never smoking of cigarettes.

As only four CB RRs based on mortality are included, the relationship to the characteristics for CB is only studied separately for outcomes based on symptoms (81 RRs) and based other than on mortality or symptoms (28 RRs). The tendency noted in Table 7 for RRs to be higher for North American studies is only evident when outcome is based on symptoms, but the tendency for RRs to be lower if adjusted for confounders seems evident in both groups.

As is the case for ever smoking, the relationship to the characteristics by outcome subtype cannot usefully be studied for emphysema due to limited numbers, with only four of 28 RRs based on mortality.

Also as for ever smoking, meta-regression analyses are conducted for COPD and CB, the results from the basic model being summarized in Table 8.

**Table 8:** Meta-regression analyses for current smoking of any product (or cigarettes if any product not available)^a^

		COPD		CB	
			
Characteristic	Level	Estimate^b ^(SE^c^)	p^d^	Estimate^b ^(SE^c^)	p^d^
Constant		+1.011 (0.156)		+1.335 (0.258)	
Sex	Male	Base	< 0.05	Base	NS
	Female	-0.218 (0.045)		-0.097 (0.043)	
	Combined	-0.007 (0.045)		+0.039 (0.082)	
Continent	N America	Base	< 0.001	Base	< 0.001
	Europe	-0.347 (0.051)		-0.353 (0.052)	
	Asia	-0.499 (0.079)		-0.493 (0.070)	
	Other	-0.510 (0.081)		-0.144 (0.086)	
Disease definition	Mortality	Base	< 0.001	Base	< 0.01
	Lung function (COPD) or symptoms (CB)	-0.435 (0.071)		-0.104 (0.232)	
	Other	+0.049 (0.076)		-0.406 (0.232)	
How asthma taken into account	Ignored	Base	< 0.001	Base	< 0.05
	Excluded	-0.334 (0.051)		+0.159 (0.076)	
	Included in outcome definition	-0.721 (0.072)		No data	
	Other	-0.055 (0.103)		+1.225 (0.237)	
Smoking product	Any	Base	< 0.05	Base	NS
	Cigarettes (ignoring other products)	+0.255 (0.084)		-0.023 (0.136)	
	Cigarettes only	+0.505 (0.099)		+0.410 (0.136)	
Unexposed group	Never any product	Base	< 0.01	Base	NS
	Never cigarettes	-0.446 (0.077)		-0.127 (0.130)	
RR adjusted for age	Yes	Base	NS	Base	< 0.01
	No	+0.076 (0.050)		+0.219 (0.039)	
RR adjusted for factor other than age or sex	Yes	Base	NS	Base	NS
	No	+0.148 (0.057)		-0.007 (0.072)	
Midpoint age	Per 10 years	+0.012 (0.002)	< 0.05	+0.003 (0.002)	NS

^a ^Based on the same data as for Table 7. See that table for further definition of RRs selected for analysis, and numbers of estimates of each characteristic level.

^b ^Estimates are of log RR. For a particular entry, the predicted RR for a given estimate is calculated by adding the constant to the values for each level applicable to the estimate (taking the value for the base level as zero) and taking the exponential of the result.

^c ^SE = standard error.

^d ^The p value is estimated from the drop in deviance from removing the characteristic from the model using an F-test. It is expressed as p < 0.001, p < 0.01, p < 0.05, p < 0.1 or NS (p ≥ 0.1).

For COPD the deviance reduces from 1,643.4 on 119 degrees of freedom to 433.3 on 103 degrees of freedom on fitting the basic model. The results in Table 8 confirm the independent role of the six characteristics noted for ever smoking: sex, continent, smoking product, the unexposed group, outcome subtype, and the way asthma is taken into account. A significant effect (p < 0.05) of age is also seen. No secondary variable significantly improves the fit (at p < 0.05). The largest standardized residual from the basic model, +2.30, is for males in study VONHER where the observed RR of 10.92 compares to a fitted RR of 5.58, corresponding RRs for females being 6.71 observed and 4.49 fitted, with a residual of +1.44.

For CB the deviance reduces from 649.2 on 112 degrees of freedom to 407.7 on 97 degrees of freedom on fitting the basic model. The characteristics which significantly (at p < 0.05) contribute to the model are continent, outcome subtype, whether the RR is adjusted for age, and how asthma is taken into account. As for ever smoking, the largest standardized residuals are for males in study GOLDBE (-3.44) and females in study JOUSI1 (-2.88).

#### C. Risk from ever or current smoking

In an attempt to incorporate data from all the studies (except those with only dose-related data), additional analyses were carried out. The first set of analyses uses results for ever smoking if available from a study, but if not results for current smoking. Conversely, the second set prefers results for current smoking to results for ever smoking where both are available. The meta-analysis RRs are shown in Table 9. The RRs are intermediate between those for ever smoking (Table 5) and those for current smoking (Table 7). For example for COPD, random-effects estimates are 2.89 (95%CI 2.63-3.17) specifically for ever smoking, 3.00 (2.71-3.32) preferring ever smoking, 3.46 (3.07-3.90) preferring current smoking, and 3.51 (3.08-3.99) specifically for current smoking. As so many of the RRs are common between the specific ever smoking analyses in Table 5 and the analyses preferring ever smoking in Table 9 the pattern of RRs by level of the characteristics studied tends to be quite similar. The same is true for the specific current smoking analyses and the analyses preferring current smoking in Table 9. Results for ever or current smoking by level of selected characteristics are therefore only presented in the Additional files.

**Table 9:** Main meta-analyses for current or ever smoking of any product (or cigarettes, if not available)a

Preference	Statistic^b^	COPD	CB	Emphysema
Ever smoking to current smoking	n	138	121	33
	F	2.47 (2.40-2.53)	2.42 (2.36-2.48)	3.32 (3.04-3.61)
	R	3.00 (2.71-3.32)	2.70 (2.51-2.90)	4.83 (3.46-6.73)
	H, P_H_	10.61, < 0.001	5.61, < 0.001	9.95, < 0.001
Current smoking to ever smoking	n	139	121	33
	F	2.87 (2.79-2.95)	2.82 (2.75-2.90)	3.44 (3.13-3.78)
	R	3.46 (3.07-3.90)	3.27 (3.02-3.54)	5.05 (3.51-7.25)
	H, P_H_	13.15, < 0.001	6.53, < 0.001	10.52, < 0.001

^a ^Within each study, results are selected in the following order of preference, within each sex, for: smoking status-ever, current or current, ever according to analysis; unexposed group-never any product, never cigarettes, other; smoking product-any, cigarettes (ignoring other products), cigarettes only; overlapping studies-principal, subsidiary; and then for single sex results in preference to combined sex results. Results adjusted for the most potential confounders are selected.

^b ^n = number of estimates combined, F = fixed-effect meta-analysis RR (95% CI), R = random-effects meta-analysis RR (95% CI), H = heterogeneity chisquared per degree of freedom.

#### D. Risk from ex smoking

**Figure 11:**
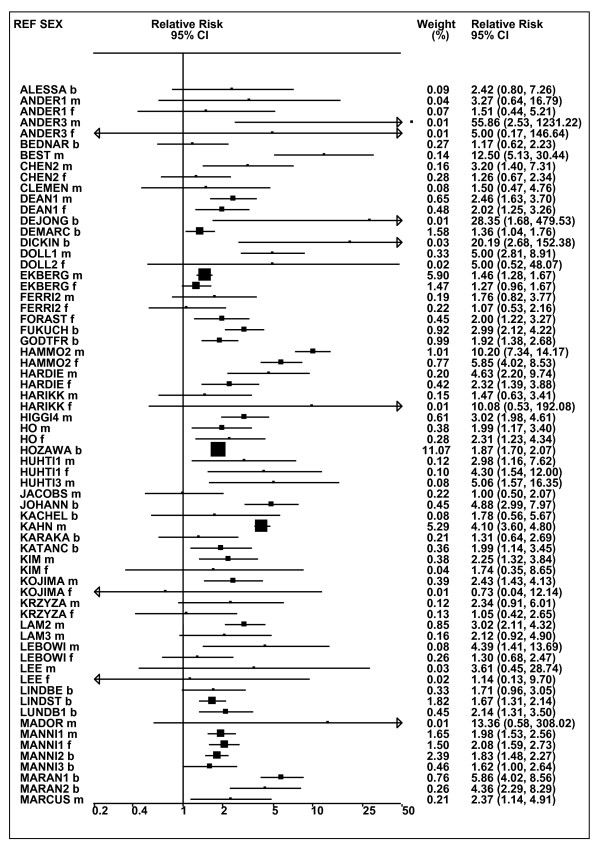
**Forest plot of ex smoking of any product and COPD-part 1**. Table 10 presents the results of a main meta-analysis for COPD based on 110 relative risk (RR) and 95% confidence interval (CI) estimates for ex smoking of any product (or cigarettes if any product not available). The individual study estimates are shown numerically and graphically on a logarithmic scale in Figures 11 and 12. The weights (inverse-variance) are also shown numerically, expressed as a percentage of the overall weight. The studies are sorted in order of sex within study reference (REF). In the graphical representation individual RRs are indicated by a solid square, with the area of the square proportional to the weight. Arrows indicate where the CI extends outside the range allocated. Where the RR value falls outside the range, the size of the plotting symbol indicates the weight but the position is not true to the scale.

**Figure 12:**
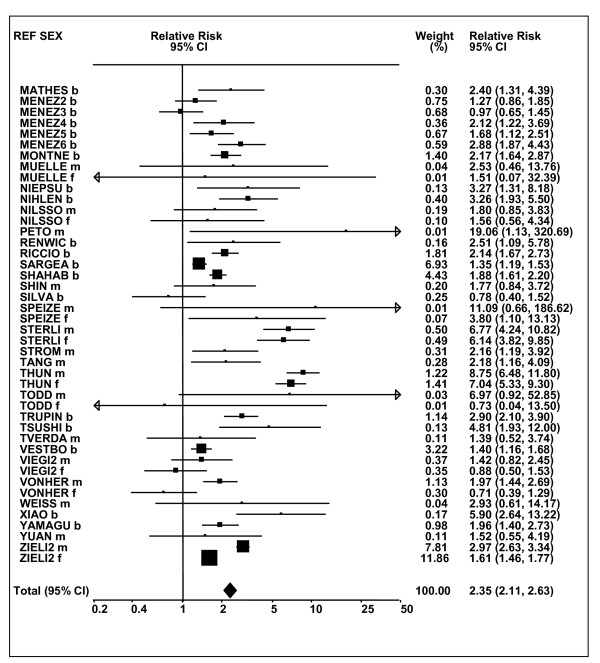
**Forest plot of ex smoking of any product and COPD-part 2**. This is a continuation of Figure 11, presenting the remaining individual study data included in the main meta-analysis for COPD shown in Table 10. Also shown are the combined random-effects estimates. These are represented by a diamond of standard height, with the width indicating the 95% CI.

Figures 11 and 12 (COPD), 13 and 14 (CB) and 15 (emphysema) present the results of the main meta-analyses for ex smoking of any product (or cigarettes if any product was not available), based on most-adjusted RRs. Some results by levels of characteristics are shown in Table 10.

**Figure 13:**
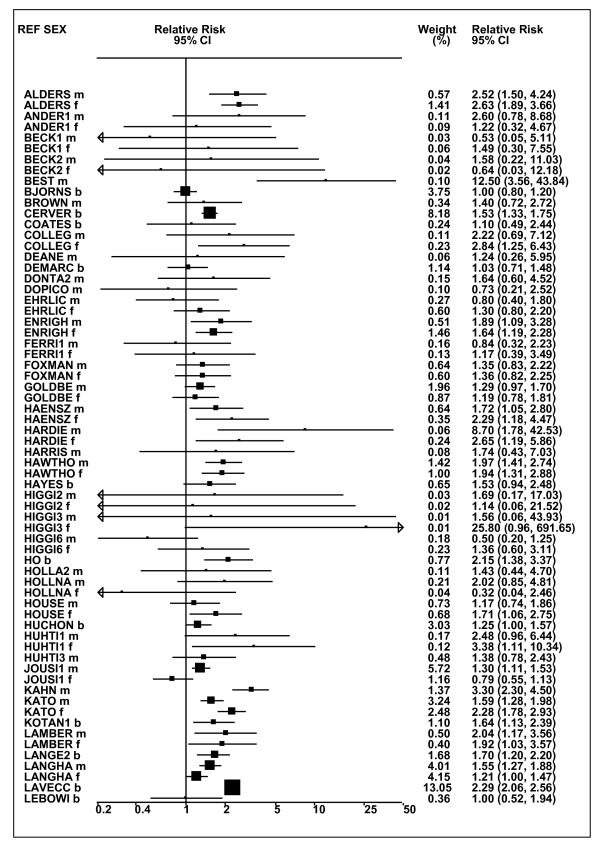
**Forest plot of ex smoking of any product and CB-part 1**. Table 10 presents the results of a main meta-analysis for CB based on 105 relative risk (RR) and 95% confidence interval (CI) estimates for ex smoking of any product (or cigarettes if any product not available). The individual study estimates are shown numerically and graphically on a logarithmic scale in Figures 13 and 14. The weights (inverse-variance) are also shown numerically, expressed as a percentage of the overall weight. The studies are sorted in order of sex within study reference (REF). In the graphical representation individual RRs are indicated by a solid square, with the area of the square proportional to the weight. Arrows indicate where the CI extends outside the range allocated. Where the RR value falls outside the range, the size of the plotting symbol indicates the weight but the position is not true to the scale.

**Figure 14:**
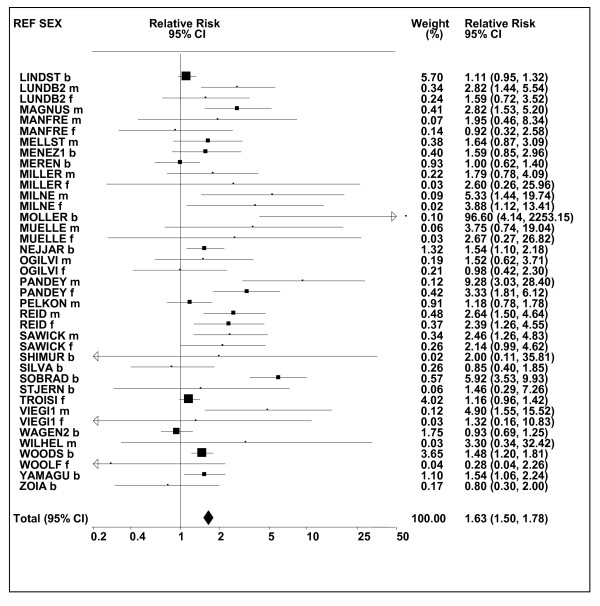
**Forest plot of ex smoking of any product and CB-part 2**. This is a continuation of Figure 13, presenting the remaining individual study data included in the main meta-analysis for CB shown in Table 10. Also shown are the combined random-effects estimates. These are represented by a diamond of standard height, with the width indicating the 95% CI.

**Figure 15:**
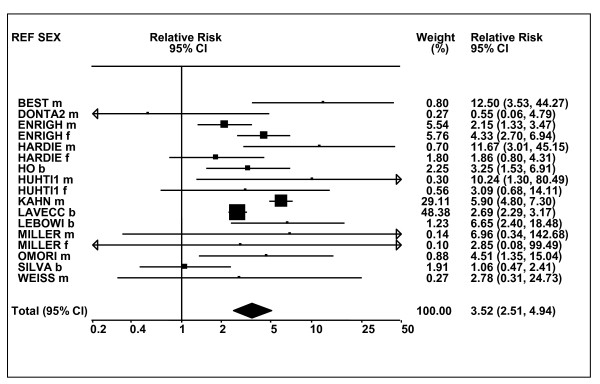
**Forest plot of ex smoking of any product and emphysema**. Table 10 presents the results of a main meta-analysis for emphysema based on 17 relative risk (RR) and 95% confidence interval (CI) estimates for ex smoking of any product (or cigarettes if any product not available). The individual study estimates are shown numerically and graphically on a logarithmic scale. The weights (inverse-variance) are also shown numerically, expressed as a percentage of the overall weight. The studies are sorted in order of sex within study reference (REF). In the graphical representation individual RRs are indicated by a solid square, with the area of the square proportional to the weight. Arrows indicate where the CI extends outside the range allocated. Also shown are the combined random-effects estimates. These are represented by a diamond of standard height, with the width indicating the 95% CI.

**Table 10:** Main meta-analyses for ex smoking of any product (or cigarettes, if any product not available)^a^

Factor	Level	Statistic^b^	COPD	CB	Emphysema
All	All	N	110	105	17
		F	2.12 (2.05-2.19)	1.56 (1.50-1.62)	3.50 (3.13-3.92)
		R	2.35 (2.11-2.63)	1.63 (1.50-1.78)	3.52 (2.51-4.94)
		H, P_H_	7.43, < 0.001	3.14, < 0.001	3.87, < 0.001
Sex	Male	n	44	46	9
		F	2.80 (2.64-2.97)	1.63 (1.52-1.76)	5.12 (4.26-6.15)
		R	2.87 (2.35-3.50)	1.80 (1.57-2.06)	4.70 (2.66-8.30)
	Female	n	28	35	4
		F	1.92 (1.78-2.06)	1.52 (1.40-1.65)	3.50 (2.36-5.19)
		R	2.02 (1.53-1.68)	1.64 (1.40-1.93)	3.50 (2.36-5.19)
	Combined	n	38	24	4
		F	1.83 (1.74-1.92)	1.54 (1.46-1.63)	2.68 (2.30-3.12)
		R	2.07 (1.83-2.35)	1.44 (1.22-1.71)	2.68 (1.58-4.54)
	Between levels	P_B_	< 0.001	NS	< 0.05
Continent	N America	n	36	37	9
		F	2.77 (2.61-2.93)	1.45 (1.32-1.60)	4.70 (3.97-5.57)
		R	2.98 (2.35-3.78)	1.47 (1.25-1.73)	3.82 (2.30-6.34)
	Europe	n	50	55	6
		F	1.81 (1.73-1.89)	1.57 (1.50-1.65)	2.71 (2.32-3.17)
		R	1.99 (1.76-2.25)	1.71 (1.51-1.94)	3.00 (1.70-5.28)
	Asia	n	16	7	2
		F	2.84 (2.91-3.25)	1.94 (1.69-2.23)	3.56 (1.88-6.75)
		R	2.79 (2.23-3.48)	2.17 (1.63-2.88)	3.56 (1.88-6.75)
	Other or multicountry	n	8	6	0
		F	1.52 (1.31-1.76)	1.34 (1.14-1.57)	
		R	1.58 (1.22-2.05)	1.33 (1.13-1.57)	
	Between levels	P_B_	< 0.001	NS	< 0.05
Study type	Case-control	n	11	9	0
		F	3.38 (2.78-4.10)	1.65 (1.38-1.98)	
		R	3.45 (2.26-5.28)	1.87 (1.18-2.96)	
	Prospective	n	35	10	4
		F	3.92 (3.60-4.26)	1.59 (1.40-1.81)	5.32 (4.36-6.49)
		R	2.88 (2.23-3.73)	1.79 (1.24-2.60)	2.99 (0.91-9.84)
	Cross-sectional	n	64	86	13
		F	1.85 (1.78-1.92)	1.55 (1.49-1.62)	2.87 (2.50-3.30)
		R	1.99 (1.81-2.19)	1.60 (1.45-1.75)	3.16 (2.50-4.00)
	Between levels	P_B_	< 0.001	NS	< 0.01
Outcome subtype	Mortality	n	29	2	2
		F	4.41 (4.04-4.80)	3.61 (2.61-4.99)	6.02 (4.90-7.40)
		R	3.55 (2.75-4.57)	5.57 (1.56-19.92)	6.56 (3.93-10.94)
	Lung function (COPD) or symptoms (CB)	n	51	76	-
		F	1.86 (1.78-1.93)	1.39 (1.32-1.46)	
		R	1.92 (1.72-2.13)	1.50 (1.36-1.64)	
	Other	n	30	27	15
		F	1.87 (1.74-2.02)	1.90 (1.78-2.04)	2.78 (2.43-3.18)
		R	2.18 (1.80-2.64)	1.84 (1.58-2.14)	2.95 (2.23-3.91)
	Between levels	P_B_	< 0.001	< 0.001	< 0.001
Smoking product	Any	n	43	53	4
		F	1.76 (1.65-1.88)	1.64 (1.55-1.74)	2.72 (2.31-3.19)
		R	2.09 (1.78-2.45)	1.67 (1.45-1.93)	2.72 (2.31-3.19)
	Cigarettes (ignoring other products)	n	59	49	12
		F	2.07 (1.99-2.15)	1.46 (1.38-1.54)	4.42 (3.76-5.18)
		R	2.19 (1.94-2.47)	1.53 (1.38-1.70)	3.33 (2.17-5.10)
	Cigarettes only	n	8	3	1
		F	6.79 (5.87-7.86)	2.80 (2.13-3.67)	12.50 (3.53-44.27)
		R	5.78 (3.81-8.77)	3.28 (1.84-5.84)	12.50 (3.53-44.27)
	Between levels	P_B_	< 0.001	< 0.05	< 0.1
Unexposed base^c^	Never any product	n	54	65	6
		F	2.41 (2.28-2.54)	1.69 (1.60-1.79)	3.67 (3.23-4.16)
		R	2.60 (2.12-3.17)	1.75 (1.53-2.01)	4.70 (2.58-8.53)
	Never cigarettes	n	56	40	11
		F	1.96 (1.88-2.04)	1.44 (1.36-1.52)	2.93 (2.28-3.76)
		R	2.15 (1.92-2.42)	1.50 (1.35-1.66)	2.99 (1.96-4.56)
	Between levels	P_B_	< 0.05	< 0.05	NS

^a ^Within each study, results are selected in the following order or preference, within each sex, for: unexposed group-never any product, never cigarettes, other; smoking product-any, cigarettes (ignoring other products), cigarettes only; overlapping studies-principal, subsidiary; and then for single sex results in preference to combined sex results. Results adjusted for the most potential confounders are selected.

^b ^n = number of estimates combined, F = fixed-effect meta-analysis RR (95% CI), R = random-effects meta-analysis RR (95% CI), H = heterogeneity chisquared per degree of freedom, P_H _= probability value for heterogeneity expressed as p < 0.001, p < 0.05, p < 0.1 or NS (p ≥ 0.1), P_B _= probability value for between levels (see methods) similarly expressed.

^c ^Includes acceptable near-equivalent estimate (see methods) if estimate for strictly defined never smoker base not available (COPD: 3 for never cigarettes, CB: 2 for never any product and 4 for never cigarettes).

Again the RRs are markedly heterogeneous (p < 0.001 for all three outcomes), ranging up to 55.86 for COPD (ANDER3/males), 96.60 for CB (MOLLER/combined sexes) and 12.50 for emphysema (BEST/males). The random-effects estimates (COPD 2.35, 95% CI 2.11-2.63, n = 110; CB 1.63, 1.50-1.78, n = 105; emphysema 3.52, 2.51-4.94, n = 17), though all clearly positive, are smaller than the corresponding estimates for current smoking. Individual RRs are only occasionally below 1.0 and never significantly so. Estimates are little affected by preferring RRs for ever smoking cigarettes to those for ever smoking any product, the random-effects estimates changing to 2.37 (2.12-2.64) for COPD and unchanged for CB and for emphysema. They are little changed by preferring least-adjusted RRs, with the estimates now 2.37 (2.13-2.63) for COPD, 1.72 (1.55-1.92) for CB and 3.68 (2.70-5.00) for emphysema.

For the main meta-analysis, the studies contributing the greatest to the total weight are ZIELI2/females for COPD (11.9% of the total of 3,510), and LAVECC/sexes combined for CB (13.1% of 2,493) and emphysema (48.4% of 300).

For the characteristics considered in Table 10, the pattern of variation is quite similar to that for current smoking seen in Table 7. Thus, for COPD, RRs are, for both current and ex smoking, higher in males, lower in European studies, lower in cross-sectional studies, higher where the outcome is mortality, higher for cigarette only smoking and higher for never any product as the unexposed base. For CB, RRs are higher for mortality for both current and ex smoking, but the differences by continent seen for current smoking are not evident for ex smoking. The same is true for differences by age-adjustment (not shown in Table 10). The small numbers of emphysema RRs for ex smoking (17) preclude reliable study of variation by level of the characteristics of interest. Further details of variations in RRs by level of the characteristics for all three outcomes, overall and (for COPD and CB) by outcome subtype are given in the Additional files.

#### E. Risk by amount smoked

Table 11 summarizes the results of meta-analyses using RRs categorized by amount smoked. These are based on those 33 studies for COPD, 44 for CB and 10 for emphysema which provided data that could be used in the meta-analyses. For all three outcomes, results are shown for one of the sets of "key values" (see methods) and for the comparison of the highest and lowest exposures. For all three outcomes, a clear increase is seen for RRs for categories containing 5, but not 20, cigarettes/day, with the meta-analysis RR increasing monotonically with increasing amount smoked. Random-effects estimates for categories containing 45, but not 20 cigarettes/day, are 9.50 (7.38-12.22, n = 26) for COPD, 7.37 (5.86-9.28, n = 35) for CB and 7.19 (2.74-18.87, n = 6) for emphysema. The key value analyses do not use results for all the dose-response data available, as a number of the studies use broad dose-response categories (such as 1-20 or 20+ cigs/day) which span more than one of the key values. The "highest vs. lowest" analyses in Table 11 include results from all the dose response relationships which can be meta-analysed, and emphasise the positive relationship, with random-effects estimates of 2.32 (1.90-2.83, n = 44) for COPD, 2.42 (2.10-2.79, n = 62) for CB, and 2.73 (1.23-6.04) for emphysema. Fuller results are given in the Additional files. These results, which also include some meta-analyses by level of selected characteristics, show that the increase with amount smoked is also clearly evident using an alternative set of key values (1, 10, 20, 30, 40, 999), though numbers of available RRs are quite sparse for the higher values, and using least-adjusted rather than most-adjusted RRs. The additional files also include available results for some other studies which present dose response data in a form that cannot readily be included in the meta-analyses (e.g. comparison of mean or median consumption in cases and non-cases). These results do not appear inconsistent with those summarized in Table 11.

**Table 11:** Meta-analyses for amount smoked^a^

Amount smoked	Statistic^b^	COPD	CB	Emphysema
Number of sets^c^		42	57	11
About 5 cigs/day^d, g^	N	40	53	9
	F	2.58 (2.39-2.78)	2.04 (1.91-2.16)	2.21 (1.88-2.59)
	R	2.89 (2.41-3.45)	2.32 (2.07-2.60)	4.24 (1.88-9.55)
	H, P_H_	3.58, < 0.001	2.08, < 0.001	7.82, < 0.001
About 20 cigs/day^e, g^	N	23	33	6
	F	6.24 (5.79-6.73)	3.64 (3.33-3.98)	7.10 (4.83-10.44)
	R	6.21 (4.72-8.17)	4.43 (3.68-5.32)	5.07 (2.04-12.61)
	H, P_H_	7.83, < 0.001	3.32, < 0.001	3.56, < 0.01
About 45 cigs/day^f, g^	N	26	35	6
	F	9.83 (8.85-10.92)	6.00 (5.48-6.57)	12.39 (7.49-20.50)
	R	9.50 (7.38-12.22)	7.37 (5.86-9.28)	7.19 (2.74-18.87)
	H, P_H_	3.28, < 0.001	4.66, < 0.001	2.23, < 0.05
Highest v lowest	N	44	62	11
	F	2.22 (2.08-2.37)	1.99 (1.87-2.12)	1.41 (1.17-1.70)
	R	2.32 (1.90-2.83)	2.42 (2.10-2.79)	2.73 (1.23-6.04)
	H, P_H_	6.02, < 0.001	3.65, < 0.001	7.50, < 0.001

^a ^Within each study, results are selected in the following order of preference, within each sex, for: smoking status-current, ever; unexposed group (where relevant)-never any product, never cigarettes; smoking product-cigarettes (ignoring other products), cigarettes only, any product; overlapping studies-principal, subsidiary; and then for single sex results in preference to combined sex results. Results adjusted for the most potential confounders are selected.

^b ^n = number of estimates combined, F = fixed-effect meta-analysis RR (95% CI), R = random-effects meta-analysis RR (95% CI), H = heterogeneity chisquared per degree of freedom, P_H _= probability value for heterogeneity expressed as p < 0.001, p < 0.05, p < 0.1 or NS (p ≥ 0.1).

^c ^Number of sets of RRs available for the key value analysis, where base for comparison is never smoked.

^d ^Category for which results are provided includes 5 cigs/day but does not include 20 cigs/day.

^e ^Category for which results are provided includes 20 cigs/day but does not include 5 or 45 cigs/day.

^f ^Category for which results are provided includes 45 cigs/day but does not include 20 cigs/day.

^g ^Base for comparison is never smoked.

#### F. Risk by age of starting to smoke

There is rather limited evidence available for age of starting, with only 10 studies for COPD, three for CB and one for emphysema providing data usable in meta-analyses. Table 12 summarizes the meta-analysis results. Random-effects RRs for earliest compared to latest starting are significantly elevated for COPD (1.49, 1.26-1.76, n = 14) and CB (2.08, 1.29-3.35, n = 6), but not for emphysema (1.14, 0.70-1.88, n = 2). The increase in risk with earlier starting seen for COPD is consistent with the results of the key value analyses, with random-effects estimates rising to 3.12 (2.07-4.70, n = 8) for categories containing 14, but not 18 years.

**Table 12:** Meta-analyses for age started to smoke^a^

Age started	Statistic^b^	COPD	CB	Emphysema
Number of sets^c^		10	2	2
About age 26 years^d, g^	N	6	Insufficient data	Insufficient data
	F	1.74 (1.29-2.34)		
	R	1.91 (1.25-1.91)		
	H, P_H_	1.48, NS		
About age 18 years^e, g^	N	6	Insufficient data	Insufficient data
	F	1.96 (1.60-2.41)		
	R	2.11 (1.08-4.11)		
	H, P_H_	7.43, < 0.001		
About age 14 years^f, g^	N	8	Insufficient data	Insufficient data
	F	3.34 (2.74-2.08)		
	R	3.12 (2.07-4.70)		
	H, P_H_	2.88, < 0.01		
Earliest v latest	N	14	6	2
	F	1.41 (1.30-1.52)	1.99 (1.42-2.79)	1.14 (0.70-1.88)
	R	1.49 (1.26-1.76)	2.08 (1.29-3.35)	1.14 (0.70-1.88)
	H, P_H_	1.29, NS	1.54, NS	0.01, NS

^a ^Within each study, results are selected in the following order of preference, within each sex, for: smoking status-current, ever; unexposed group (where relevant)-never any product, never cigarettes; smoking product-cigarettes (ignoring other products), cigarettes only, any product; overlapping studies-principal, subsidiary; and then for single sex results in preference to combined sex results. Results adjusted for the most potential confounders are selected.

^b ^n = number of estimates combined, F = fixed-effect meta-analysis RR (95% CI), R = random-effects meta-analysis RR (95% CI), H = heterogeneity chisquared per degree of freedom, P_H _= probability value for heterogeneity expressed as p < 0.001, p < 0.05, p < 0.1 or NS (p ≥ 0.1).

^c ^Number of sets of RRs available for the key value analysis, where base for comparison is never smoked.

^d ^Category for which results are provided includes 26 years but does not include 18 years.

^e ^Category for which results are provided includes 18 years but does not include 14 or 26 years.

^f ^Category for which results are provided includes 14 years but does not include 18 years.

^g ^Base for comparison is never smoked

#### G. Risk by pack-years

Table 13 summarizes the results for pack-years, based on 24 studies for COPD, eight for CB and two for emphysema. Key value analysis was not attempted for emphysema, due to the limited data. For COPD and CB, a clear dose-response is seen, with meta-analysis RRs increased for categories containing 5, but not 20 pack-years, and increasing monotonically with increasing pack-years. Random-effects estimates for categories containing 45, but not 20, pack-years are 3.69 (2.79-4.86, n = 15) for COPD, and 7.04 (5.06-9.79, n = 36) for CB. The "highest vs. lowest" analyses confirm the existence of a dose-response relationship for all three outcomes, with random-effects estimates of 2.80 (2.37-3.30, n = 31) for COPD, 3.09 (2.33-4.10, n = 11) for CB, and 2.42 (1.25-4.70) for emphysema. Fuller results are given in the Additional files. As for amount smoked, these results show that the dose-related increase can be clearly seen using alternative key values (1, 10, 20, 30, 999), and using least-adjusted rather than most-adjusted RRs. The additional file also summarizes results for quite a number of other studies presenting dose response data in a form that cannot readily be meta-analysed. Many of these reported a significantly increased risk with increasing pack-years.

**Table 13:** Meta-analyses for pack-years^a^

Pack-years	Statistic^b^	COPD	CB	Emphysema
Number of sets^c^		28	11	3
About 5^d, g^	N	23	10	Insufficient data
	F	1.13 (1.06-1.20)	2.11 (1.74-2.55)	
	R	1.25 (1.09-1.44)	1.74 (1.17-2.58)	
	H, P_H_	2.06, < 0.01	2.85, < 0.01	
About 20^e, g^	N	11	8	Insufficient data
	F	1.68 (1.58-1.79)	4.54 (3.69-5.58)	
	R	2.53 (1.87-3.43)	4.54 (3.69-5.58)	
	H, P_H_	4.44, < 0.001	0.63, NS	
About 45^e, g^	N	15	8	Insufficient data
	F	3.14 (2.97-3.32)	7.33 (5.98-8.97)	
	R	3.69 (2.79-4.86)	7.04 (5.06-9.79)	
	H, P_H_	6.34, < 0.001	1.82, < 0.1	
Highest v lowest	N	31	11	3
	F	2.82 (2.69-2.97)	2.52 (2.25-2.82)	1.86 (1.40-2.47)
	R	2.80 (2.37-3.30)	3.09 (2.33-4.10)	2.42 (1.25-4.70)
	H, P_H_	4.09, < 0.001	2.23, < 0.05	1.79, NS

^a ^Within each study, results are selected in the following order of preference, within each sex, for: smoking status-current, ever; unexposed group (where relevant)-never any product, never cigarettes; smoking product-cigarettes (ignoring other products), cigarettes only, any product; overlapping studies-principal, subsidiary; and then for single sex results in preference to combined sex results. Results adjusted for the most potential confounders are selected.

^b ^n = number of estimates combined, F = fixed-effect meta-analysis RR (95% CI), R = random-effects meta-analysis RR (95% CI), H = heterogeneity chisquared per degree of freedom, P_H _= probability value for heterogeneity expressed as p < 0.001, p < 0.05, p < 0.1 or NS (p ≥ 0.1).

^c ^Number of sets of RRs available for the key value analysis, where base for comparison is never smoked.

^d ^Category for which results are provided includes 5 pack-years but does not include 20 pack-years.

^e ^Category for which results are provided includes 20 pack-years but does not include 5 or 45 pack-years.

^f ^Category for which results are provided includes 45 pack-years but does not include 20 pack-years.

^g ^Base for comparison is never smoked.

#### H. Risk by duration of smoking

Evidence for duration of smoking that can be used in meta-analyses is only available for three studies for COPD, three for CB and two for emphysema. Table 14 summarizes the results of the meta-analyses, which for CB and emphysema are based on heterogeneous data. Random-effects RRs for longest compared to shortest duration show no clear pattern for COPD (1.12, 0.63-1.98, n = 3), CB (2.25, 0.68-7.42, n = 4), or emphysema (7.67, 0.15-390.65, n = 2).

**Table 14:** Meta-analyses for duration of smoking^a, b^

Duration of smoking	Statistic^c^	COPD	CB	Emphysema
Longest vs. shortest	N	3	4	2
	F	1.12 (0.63-1.98)	2.73 (1.52-4.92)	7.82 (2.00-30.58)
	R	1.12 (0.63-1.98)	2.25 (0.68-7.42)	7.67 (0.15-390.65)
	H, P_H_	0.76, NS	3.60, < 0.05	8.31, < 0.01

^a ^Within each study, results are selected in the following order of preference, within each sex, for: smoking status-current, ever; never cigarettes; smoking product-cigarettes (ignoring other products), cigarettes only, any product; overlapping studies-principal, subsidiary; and then for single sex results in preference to combined sex results. Results adjusted for the most potential confounders are selected.

^b ^The number of sets of RRs available for key value analysis, where base for comparison is never smoked, is 3 for COPD, 4 for CB and 3 for emphysema, and no key value analysis was carried out.

^c ^n = number of estimates combined, F = fixed-effect meta-analysis RR (95% CI), R = random-effects meta-analysis RR (95% CI), H = heterogeneity chisquared per degree of freedom, P_H _= probability value for heterogeneity expressed as p < 0.001, p < 0.05, p < 0.1 or NS (p ≥ 0.1).

#### I. Risk by duration of quitting (vs. never smoking)

The number of studies providing usable data for duration of quitting compared to never smoking is seven for COPD, and seven for CB, but none for emphysema. As shown in Table 15, there is some evidence of higher risks in short-term quitters for COPD, with the shortest vs. longest random-effects meta-analysis estimate 2.21 (1.24-3.94, n = 10) and a tendency for estimates to be lower for the longer-term quitters in the key value analysis, though the trend is not monotonic. For CB, evidence of higher risks in short-term quitters is less convincing, with the shortest vs. longest estimate of 1.25 (0.99-1.59, n = 11) not significant, and RRs varying little by key value. The results are limited by the variability of the categories used by different studies to classify duration of quitting. This makes it difficult to find a key scheme which includes sufficient numbers of studies across the range. For instance, for COPD, the key scheme shown in Table 15 includes only three RRs at the two shorter levels, whereas an alternative set of key values (20, 12 and 3 years, shown in the Additional files) incorporates only three RRs at the two longer levels.

**Table 15:** Meta-analyses for duration of quitting (vs. never smoked)^a^

Duration of quitting	Statistic^b^	COPD	CB
Number of sets^c^		10	11
About 12 years^d, g^	n	10	9
	F	3.45 (2.96-4.01)	1.40 (1.21-2.62)
	R	2.12 (1.06-4.26)	2.20 (1.33-3.65)
	H, P_H_	12.74, < 0.001	8.04, < 0.001
About 7 years^e, g^	n	3	5
	F	8.15 (5.88-11.28)	1.83 (1.49-2.25)
	R	4.94 (1.21-20.07)	2.36 (1.29-4.32)
	H, P_H_	14.80, < 0.001	6.52, < 0.001
About 3 years^f, g^	n	3	7
	F	3.58 (2.44-5.25)	2.16 (1.82-2.57)
	R	4.08 (0.80-20.77)	2.42 (1.73-3.38)
	H, P_H_	16.66, < 0.001	2.79, < 0.05
Shortest v longest	n	10	11
	F	2.20 (1.76-2.76)	1.29 (1.06-1.56)
	R	2.21 (1.24-3.94)	1.25 (0.99-1.59)
	H, P_H_	4.88, < 0.001	1.29, NS

^a ^Within each study, results are selected in the following order of preference, within each sex, for: smoking status-current, ever; unexposed group (where relevant)-never any product, never cigarettes; smoking product-cigarettes (ignoring other products), cigarettes only, any product; overlapping studies-principal, subsidiary; and then for single sex results in preference to combined sex results. Results adjusted for the most potential confounders are selected.

^b ^n = number of estimates combined, F = fixed-effect meta-analysis RR (95% CI), R = random-effects meta-analysis RR (95% CI), H = heterogeneity chisquared per degree of freedom, P_H _= probability value for heterogeneity expressed as p < 0.001, p < 0.05, p < 0.1 or NS (p ≥ 0.1).

^c ^Number of sets of RRs available for the key value analysis, where base for comparison is never smoked. No data available for emphysema.

^d ^Category for which results are provided includes quit 12 years ago but does not include quit 7 years ago.

^e ^Category for which results are provided includes quit 7 years ago but does not include quit 3 or 12 years ago.

^f ^Category for which results are provided includes quit 3 years ago but does not include quit 7 years ago.

^g ^Base for comparison is never smoked.

#### J. Risk by duration of quitting (vs. current smoking)

For duration of quitting compared to current smoking, data are available from one less study than for duration of quitting compared to never smoking for COPD, but from the same studies for CB. The longest vs. shortest analysis shown in Table 16 is the inverse of the shortest vs. longest analysis in Table 15. The key value analyses are based on a limited number of RRs but are consistent with the association declining with longer-term quitting. For categories including 12, but not 7, years quitting random-effects meta-analysis RRs relative to current smoking are 0.52 (0.37-0.71, n = 9) for COPD and 0.65 (0.41-1.04, n = 9) for CB.

**Table 16:** Meta-analyses for duration of quitting (vs. current smoking)^a^

Duration of quitting	Statistic^b^	COPD	CB
Number of sets^c^		9	11
About 3 years^d, g^	n	2	7
	F	0.77 (0.51-1.15)	1.07 (0.91-1.25)
	R	0.77 (0.51-1.15)	1.00 (0.70-1.43)
	H, P_H_	0.28, NS	3.85, < 0.001
About 7 years^e, g^	n	2	5
	F	1.03 (0.62-1.70)	1.00 (0.84-1.19)
	R	1.03 (0.62-1.70)	0.87 (0.47-1.61)
	H, P_H_	0.53, NS	9.89, < 0.001
About 12 years^e, g^	n	9	9
	F	0.52 (0.43-0.63)	0.61 (0.53-0.69)
	R	0.52 (0.37-0.71)	0.65 (0.41-1.04)
	H, P_H_	1.93, < 0.1	9.11, < 0.001
Longest v shortest	n	10	11
	F	0.43 (0.34-0.53)	0.78 (0.64-0.95)
	R	0.45 (0.24-0.84)	0.80 (0.63-1.02)
	H, P_H_	5.78, < 0.001	1.28, NS

^a ^Within each study, results are selected in the following order of preference, within each sex, for: smoking product-cigarettes (ignoring other products), cigarettes only, any product; overlapping studies-principal, subsidiary; and then for single sex results in preference to combined sex results.

^b ^n = number of estimates combined, F = fixed-effect meta-analysis RR (95% CI), R = random-effects meta-analysis RR (95% CI), H = heterogeneity chisquared per degree of freedom, P_H _= probability value for heterogeneity expressed as p < 0.001, p < 0.05, p < 0.1 or NS (p ≥ 0.1).

^c ^Number of sets of RRs available for the key value analysis, where base for comparison is current smoking. No data available for emphysema.

^d ^Category for which results are provided includes quit 3 years ago but does not include quit 7 years ago.

^e ^Category for which results are provided includes quit 7 years ago but does not include quit 3 or 12 years ago.

^f ^Category for which results are provided includes quit 12 years ago but does not include quit 7 years ago.

^g ^Base for comparison is current smoking.

### Further analyses based on within-study differences

Some studies provide independent RRs for males and females for the same definition of outcome and exposure. Random-effects meta-analysis of the male/female sex ratio for current and ever smoking for each outcome confirm the impression already gained from the analyses shown in Tables 5 to 8 that RRs are somewhat higher for males, though again the difference is not always statistically significant. For ever smoking, the meta-analysis RRs of the sex ratio are 1.28 (1.02-1.60) for COPD, 1.16 (0.97-1.38) for CB and 1.44 (0.72-2.87) for emphysema, based on, respectively, 31, 35 and 6 RRs. For current smoking the estimates are 1.25 (1.00-1.58, n = 29) for COPD, 1.17 (0.96-1.42, n = 35) for CB, and 1.98 (0.75-5.22, n = 5) for emphysema.

Some studies also provide separate non-independent least-adjusted and most-adjusted RRs for the same definition of exposure. There is little evidence that adjustment reduces the RR for ever or current smoking. For ever smoking, using the same preferences as in the main meta-analyses (Figures 1, 2, 3, 4 and 5), the most-adjusted estimate is lower than the least-adjusted estimate for 14 of the 30 (46.7%) pairs for COPD, for 18 of the 41 (43.9%) pairs for CB, and for 2 of the 5 (40.0%) pairs for emphysema. For current smoking the corresponding numbers are 11/26 (42.3%) for COPD, 16/36 (44.4%) for CB and 2/3 (66.7%) for emphysema. In no case do the percentages differ from 50% (at p < 0.05), and in each case the random-effects meta-analysis estimate based on the least-adjusted pair members is similar to the corresponding estimate based on the most-adjusted pair members (data not shown).

After excluding studies with no pipe or cigar smokers, some studies allow comparison of RRs of the risk of current smoking vs. never smoking for cigarette smokers ignoring other products with equivalent RRs for cigarette only smokers. These estimates are non-independent. For 7 of the 9 pairs of RRs for COPD, for all 6 of the pairs for CB (p < 0.05) and for both the pairs for emphysema the RR is lower for cigarette smokers ignoring other products. However the RR ratio is never markedly different from 1, ranging from 0.78 to 1.13 for COPD, from 0.84 to 0.99 for CB, and from 0.86 to 0.96 for emphysema.

RRs for a dose-related index of smoking may be adjusted for other such indices. However, this is only at all common for age of starting to smoke, where adjustment for amount smoked is carried out in five of the 10 studies providing data for COPD, and in one of the three providing data for CB. It is not possible to assess the effect of adjustment for amount smoked, as three of the six relevant studies provide the adjusted RR and no other RR, and the other three provide only adjusted and totally unadjusted RRs.

**Figure 16:**
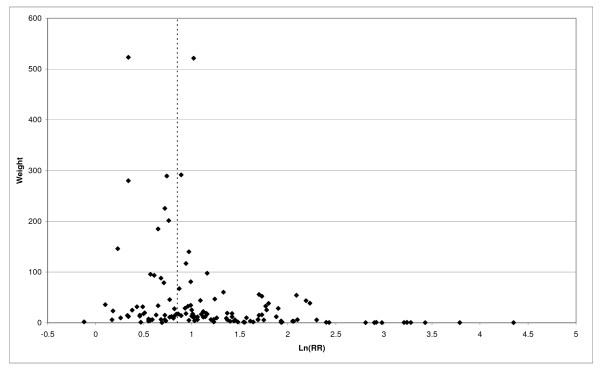
**Funnel plot for ever smoking and COPD**. Funnel plot of the 129 relative risk estimates for ever smoking and COPD included in the main meta-analysis in Table 5 against their weight (inverse-variance of log RR). The dotted vertical line indicates the fixed-effect meta-analysis estimate.

For all three outcomes, Egger's test [16] shows significant evidence of publication bias for both ever smoking (COPD p < 0.001, CB p < 0.05, emphysema p < 0.01) and current smoking (COPD p < 0.05, CB p < 0.001, emphysema p < 0.05). Figures 16 (COPD), 17 (CB) and 18 (emphysema) show funnel plots for ever smoking. All the plots give an impression of there being more lower-weight RRs above the mean and more higher-weight RRs below the mean.

**Figure 17:**
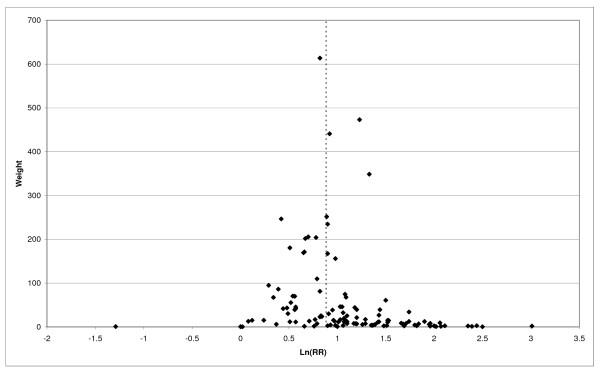
**Funnel plot for ever smoking and CB**. Funnel plot of the 114 relative risk estimates for ever smoking and CB included in the main meta-analysis in Table 5 against their weight (inverse-variance of log RR). The dotted vertical line indicates the fixed-effect meta-analysis estimate.

**Figure 18:**
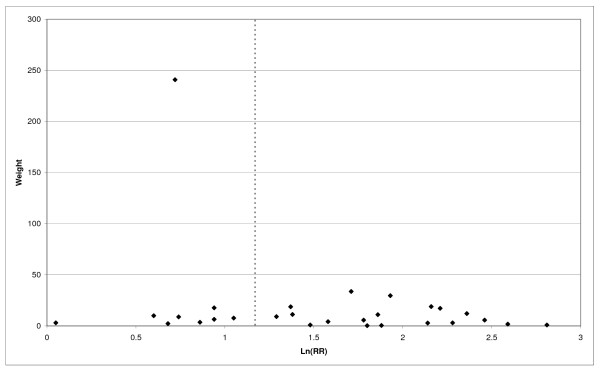
**Funnel plot for ever smoking and emphysema**. Funnel plot of the 28 relative risk estimates for ever smoking and emphysema included in the main meta-analysis in Table 5 against their weight (inverse-variance of log RR). The dotted vertical line indicates the fixed-effect meta-analysis estimate.

## Discussion

### Evidence of a relationship

The meta-analyses carried out demonstrate a clear relationship of smoking to all three outcomes considered-COPD, CB and emphysema. This is evident for ever, current and ex smoking, and for outcomes based on mortality, lung function, symptom prevalence or other methods. That this relationship is causal is supported by the evidence of a dose-response, risk increasing with amount smoked and pack-years for all three outcomes, and (based on more limited data) decreasing with increasing age of starting to smoke for COPD and CB, and with increasing duration of quitting for COPD. It is also supported by the similarity of results based on most-adjusted and least-adjusted RRs, and by within-study comparisons showing that additional confounder adjustment little affected estimates for the same exposure definition.

### Heterogeneity

The studies are remarkably consistent in reporting an increased risk in ever smokers. Only two of the 271 RRs for the three outcomes combined considered in Figures 1, 2 and 3 are less than 1.0. However, studies also vary markedly in the magnitude of the estimated RR, as illustrated by the high values of H seen in the meta-analyses of the major smoking indices, which often exceed 5 and sometimes exceed 10. (H values of 5 and 10 are the same as I^2 ^values [17] of 80% and 90%). This is unsurprising given the many sources of variation involved, including sex, location, timing, study design and populations, definition of outcome and exposure, type of product smoked, and extent of confounder adjustment.

Using univariate and multivariate (meta-regression) methods, we investigated variation in risk by a number of characteristics of the study and the RR. For each outcome no characteristic on its own explains a major part of the variation, and substantial excess heterogeneity remains even after fitting multivariate models. However, differences in the strength of the association with smoking by level of some characteristics are apparent, these differences being quite similar for each outcome and each major smoking index. RRs tend to be higher for North American studies, for males, and for cigarette smoking than smoking of any product. For COPD RRs are substantially higher for studies of mortality or onset, especially those where the definition of COPD excludes asthma, and lower where the definition is lung function based. Studies of mortality are less common for CB or emphysema, but also give relatively high estimates. Effects of some other characteristics, such as study timing and study type, though significant in some univariate analyses, are not significant with the multivariate approach. As some characteristics are correlated (e.g. mortality studies are often prospective, US studies are more often prospective than elsewhere, and studies using lung function criteria are commonly cross-sectional) it is not straightforward to identify underlying effects. However, we feel that the main meta-regression models for ever smoking (Table 6) and current smoking (Table 8) for COPD and CB are useful in explaining some of the heterogeneity, their usefulness being confirmed by the fact that adding in further characteristics did not significantly improve prediction. Particularly for COPD, the meta-regressions show there are many characteristics that independently modify the risk estimates. Meta-regressions were not tried for emphysema, where there were fewer RRs available, or for ex smoking, where the relationship was weaker than for ever or current smoking. Sources of variation are discussed further in the following paragraphs.

### Sex

If possible, sex-specific results are included in the meta-analyses, with combined sex results included only if not. Though variation by sex was not significant in all the main analyses, risk estimates generally tended to be higher for males than females. This is supported by additional analyses comparing RRs within-study for the same outcome and exposure definition. The higher RRs for males do not necessarily indicate any greater susceptibility, and seem more likely to reflect increased smoking. We note that some publications (e.g. [18-20]) have suggested that women may have a greater susceptibility than men to the effects of smoking on COPD or lung function, but others (e.g. [21-23]) have suggested the opposite. A detailed overall assessment of this aspect is beyond the scope of this paper.

### Age

In the meta-regressions a continuous variable was included that indicated the midpoint of the age group to which the RR applied. The fitted coefficient was always positive, but significant (at p < 0.05) only for current smoking for COPD. Note that for each study only RRs for the whole age range were entered, though the availability of age-specific data was recorded. Proper assessment of the relationship of age to the RRs for the different outcomes would require entry and analysis of these further data. For the present, the data can only be regarded as indicating that RRs for studies in older populations may be greater than those in younger populations.

### Location

The meta-regressions showed significant variation in risk by continent, mainly due to higher RRs for North American studies. Similar differences are seen in the univariate analyses for emphysema, and also for ex smoking (except for CB). This difference is not readily explained, but it could relate to differences in diagnosis not fully accounted for by the model, in amount smoked, or in type of product smoked. However a variable accounting for the predominant long-term use of blended cigarettes in some countries (including the US), and of flue-cured Virginia cigarettes in others (including the UK and Canada), did not significantly predict risk.

### Study timing

In the univariate analyses of ever and current smoking RRs varied significantly by when the study was published, but the pattern was erratic, with no trend. Study timing did not, however, add predictive power to the multivariate models. This suggests that differences between the periods studied are correlated with differences in other study characteristics. The term COPD has only been widely used in the last 25 years or so, and definitions based on lung-function have been changing, so there may well be differences by time in the nature of outcomes we classified as COPD. There have also been changes in the nature of the product smoked, with reducing tar deliveries of cigarettes and declining use of pipes and cigars[24].

### Definition of the disease outcome

For all RRs meta-analysed, the outcome had to be CB specifically, emphysema specifically or COPD generally. Thus each RR applied only to one outcome. The term COPD is quite recent, so data from some earlier studies which might legitimately have been included may have been excluded or entered against the wrong outcome. Some early studies described their outcomes as CB. If they supported their definitions by ICD codes incorporating all the core components of COPD, we reclassified the outcome as COPD. However, where ICD codes were not given, we left the outcome as CB, though we suspect that sometimes the outcome might better have been COPD.

For COPD, the definitions allowed vary considerably, and the cases may not represent a homogeneous set. Thus population-based cross-sectional studies using lung function criteria alone probably include cases with less severe disease than studies in hospitals or using mortality records. Most prospective studies of incidence made no attempt to trace deaths, so may have omitted more rapidly progressive cases. We have not studied variation in risk in those few studies presenting results by severity of disease. Similar considerations apply to CB and emphysema, though less strongly, partly because there were fewer studies of mortality.

For COPD RRs are higher when the definition was based on mortality than when based on lung function or other criteria. Compared to RRs based on lung function, the meta-regressions indicated that RRs based on mortality are about 1.5 times higher for both ever and current smoking. The tendency for RRs based on mortality to be higher is also seen for CB and emphysema, but based on fewer studies.

For COPD RRs also clearly vary by how asthma was taken into account. For most studies, co-existing asthma was ignored (i.e. diagnosis was made independent of asthma, and both cases and non-cases could include asthmatics). However there were some (mainly mortality) studies where asthma is part of the outcome definition (e.g. COPD = CB, emphysema or asthma). Here, usually only the underlying cause of death is considered, so the possibility of a CB or emphysema case also being recorded as having asthma does not arise. RRs are much lower for these studies. For others, asthmatics had been totally excluded, and RRs tend to be intermediate.

### Study type

For COPD particularly, the univariate analyses show a tendency for RRs to be higher for prospective studies than for other designs. Study type did not contribute in multivariate analyses, probably reflecting its strong correlation with disease outcome definition, prospective studies tending to present mortality results, but other study types tending to use lung function, symptoms or other criteria.

### Aspects of smoking

For COPD the meta-regressions show significant variation by smoking product, with RRs highest for smokers of cigarettes only, lowest for smokers of any product, and intermediate for smokers of cigarettes (ignoring other products). As the estimates for cigarette only smokers depended largely on just two large studies (HAMMO2, THUN), we further investigated the difference between smokers of cigarettes only and smokers of cigarettes by within-study comparisons. This confirmed the tendency for cigarette only smokers to have higher risks. Though we have not considered data for smoking of pipes and cigars only, the results are consistent with a greater effect of smoking cigarettes than other products. Smokers of any product include some who smoke no cigarettes at all, while smokers of cigarettes include some who smoke cigarettes and pipes/cigars and who are likely to smoke less cigarettes per day than smokers of cigarettes only. For CB and emphysema there are few RRs for cigarette only smokers, but these also suggest higher risks for this group.

For COPD, the results show a higher RR where the unexposed group is never any product than when it is never cigarettes. This is consistent with the absolute risk being higher where the unexposed group includes some smokers (of pipes/cigars), than where it does not. However, this pattern is not seen for CB and emphysema.

We investigated the dose-response relationship by meta-analyses for five exposure measures-amount smoked, age of starting, pack-years, duration of smoking, duration of quitting (both vs. never smokers and vs. current smokers).

Meta-analysis of RRs expressed relative to never smokers or relative to current smokers is hampered by the different categories used by different studies to define level of exposure, so we also analyzed RRs comparing extreme levels of exposure within smokers, an approach allowing all studies to be included (including those only presenting analyses for smokers). For all three outcomes, risk increases with amount smoked and pack-years. For COPD and CB earlier starters have significantly higher risks, and risk also tended to decrease with longer-term quitting. Data are too few for emphysema to make inferences for age of starting and duration of quitting. The only measure showing no dose-relationship is duration of smoking but data are very limited. Note that all the outcomes are chronic diseases and disease presence may affect smoking habits. Depending on when smoking habits are recorded, this may bias downwards associations with these dose-related measures.

### Derivation of RRs

About a third of RRs used in meta-analyses are available from the source or can be derived directly from cross-tables of exposure by outcome. Otherwise more complex methods had to be used to derive the required RR. It was reassuring that whether or not the RR was derived did not add predictive power to the main meta-regression model, suggesting that use of derived RRs caused no material bias.

### Effect of studies with high RRs or large weight

The statistical analyses investigated the role of various characteristics on the estimated risk of the three outcomes in relation to smoking, but did not formally test the effect of exclusion of specific studies with extreme RRs or large weights. For ever and current smoking, we have noted the highest RRs and those contributing most to the total weight. For COPD and CB, where each analysis involves over 100 most-adjusted RRs, no single RR contributes more than 12% of the total weight, and the distribution of RRs and of standardized residuals from the meta-regression models did not suggest any single RR had an undue influence. For emphysema, the situation is different. There are fewer RRs, only 28 for ever smoking and 22 for current smoking, and one study (LAVECC) contributes substantially to the overall weight (49% for ever, 62% for current) while having a relatively low RR (2.05 for ever, 1.76 for current). Furthermore, study AUERBA, which does not provide an RR for ever smoking, has a strikingly large RR of 489.54 for current smoking.

We therefore investigated the effect of exclusion of these studies on the combined current smoking RR, where the problem is most severe (Table 17). It can be seen that exclusion of AUERBA substantially reduces the random-effects estimate, while exclusion of LAVECC substantially increases the fixed-effects estimate. Both exclusions, particularly AUERBA, reduce the heterogeneity substantially.

**Table 17:** Investigating the effect of excluding a study with a very large weight, (LAVECC) and/or a study with a very high RR (AUERBA) on the meta-analysis, estimate for current smoking for emphysema

Studies included	Fixed-effects RR (95% CI)	Random-effects RR (95% CI)	H^a^
All 22 studies	2.61 (2.33-2.93)	4.87 (2.83-8.41)	11.54
Exclude AUERBA	2.36 (2.10-2.65)	3.62 (2.50-5.24)	4.48
Exclude LAVECC	4.95 (4.10-5.97)	5.20 (2.85-9.49)	8.50
Exclude both	3.89 (3.21-4.71)	3.85 (2.71-5.47)	2.55

^a ^H is the heterogeneity chisquared per degree of freedom.

Why should the estimates vary so much? LAVECC was a large national health survey in Italy, in which 437/22, 376 (2.0%) male and female current smokers of any product and 595/44, 172 (1.3%) male and female never smokers of any product reported they had emphysema or respiratory insufficiency, with no independent check on the diagnosis. AUERBA involved an examination of whole-lung sections prepared from lungs removed at autopsy, with 816/839 (97.3%) male current cigarette smokers and 20/176 (11.4%) male never smokers of any product diagnosed as having minimal, slight, moderate, advanced or far advanced emphysema. These percentages differ widely between the two studies and reflect differences in what is considered emphysema. Someone interviewed in a survey would be unaware of lower grades of emphysema. For AUERBA it is possible to derive RRs for higher grades of emphysema. For instance, restricting attention to advanced or far advanced emphysema reduces the rate in the male smokers to 134/839 (16.0%), and in never smokers to zero, so still indicating an extremely high RR.

We also compared the results reported by AUERBA with those reported in the other autopsy studies (ANDER2, PRATT, RYDER and SUTINE), although only results for ever smoking are available in those studies, PRATT being of males and the other studies of both sexes combined. Among never smokers of any product, rates of emphysema (ANDER2 30/51 = 58.8%, PRATT 15/97 = 15.5%, RYDER 21/73 = 28.8%, SUTINE 28/73 = 38.4%) are all much higher than reported by LAVECC and also higher than reported by AUERBA. Among ever smokers of any product (cigarettes only for ANDER2), rates of emphysema (ANDER2 = 89.5%, PRATT = 42.0%, RYDER = 75.5%, SUTINE = 69.2%) are again much higher than reported by LAVECC but clearly lower than reported by AUERBA. While it is clear that emphysema rates based on autopsy studies are much higher than those based on surveys, (and also than those based on mortality studies, data not shown), the very high RR seen in AUERBA is due to a far greater discrimination between smokers and never smokers than seen in other autopsy studies. These results emphasise the problem of heterogeneity in deriving combined estimates.

### Representativeness

We excluded studies of populations with a co-existing medical condition, with clearly atypical smoking habits (e.g. cocaine users or residents of a homeless shelter), or with clearly atypical risk (e.g. alpha-1 antitrypsin deficiency). Thus most studies include subjects broadly representative of the general population. Some studies had eligibility criteria such as long-term residence, household residence (excluding residents of institutions or military personnel) or telephone subscribers, criteria that may have resulted in under-representing subjects with lower SES or more mobile lifestyles. A few studies involved patients attending their physician or clinics, who may have been less healthy than average. It seems unlikely that any of these effects would have materially affected the relationship between smoking and COPD.

Studies of subjects with a high occupational risk for respiratory disease were excluded. The classification of high risk was based on our educated judgment, and not formally tested. Low occupational risk studies included in this report involved armed forces personnel, doctors, nurses, teachers, civil servants, professional and businessmen, coffeehouse and shop workers, postal, telephone, transport and clerical workers, and outdoor workers, as well as persons working in specific factories, research facilities, or unspecified industry.

Some studies included were originally designed along clinical or experimental rather than epidemiological lines, and subject selection was unclear. These studies are generally small, and any non-representativeness would little affect our results.

### Other sources of bias

It is well known that researchers are more likely to wish to publish, and editors more likely to accept for publication, studies finding a statistically significant association between exposure and disease. The published literature may therefore overstate any true association or produce a false-positive relationship. There is some formal evidence of publication bias, with Egger's test suggesting bias in a number of the meta-analyses (see Figures 10 to 12). While some small studies showing no association may never have been published, large studies are likely to publish, and it is these which contribute most to the meta-analyses. We have not attempted to quantify bias, as formal methods are all based on assumptions which cannot be tested, but it seems doubtful whether publication bias is a serious issue.

Another possible source of bias is misclassification of smoking status. Random misclassification would dilute the association, as would any tendency for cases to deny or understate their smoking more than for the general population. Any tendency for current smokers to claim to be ex smokers, as might happen in a study conducted in a clinical setting or where patients have been advised to stop smoking, would tend to inflate the risk for ex smoking. Not only may misclassification rates vary by aspects of the study design and the way questions are asked, they may also vary by sex, age or other demographic variables.

The meta-analyses were conducted by combining direct estimates of the RR (from prospective studies) with ORs (from case-control and cross-sectional studies and occasionally from prospective studies). ORs somewhat overestimate relative risks where the disease is not rare [25], but here the overestimation is of little practical importance. Based on unadjusted data from prospective studies, where one could calculate both the relative risk and the OR, we estimate that the median bias from using the OR would have been only 1.01 for COPD and emphysema, and 1.04 for chronic bronchitis.

### Limitations

This review has various limitations, many unavoidable. Lack of access to individual subject data limits the ability to carry out meta-analyses using similar exposure indices and confounder adjustment throughout, but obtaining such data was not feasible given many studies were conducted years ago. Obtaining a reliable definition of outcome and exposure is often hindered by incomplete information in the source papers. This review is also to some extent limited by restricting attention only to stratification by sex, and not attempting to record RRs subdivided by age or other characteristics. We also limited attention to specific indices of smoking, for example not entering data on pipe or cigar smoking, filter/plain smoking, or tar level. However we have recorded the availability of such extra information, and further work incorporating such data may give more insights. The procedures conducted for this review were extremely time-consuming and it was impractical to bring the literature included fully up-to-date. However consideration of data from 218 studies published between 1953 and 2006 should give a reliable enough picture.

## Conclusions

After excluding studies conducted in children or adolescents, or in populations at high respiratory disease risk or with co-existing diseases, we identified, from papers published between 1953 and 2006, 218 studies which relate one or more of a defined set of smoking indices to COPD, CB and emphysema. One hundred and thirty-three of the studies provide relevant data for COPD, 101 for CB and 28 for emphysema.

One major conclusion is that for each outcome the RRs for a given smoking index were markedly heterogeneous.

Another conclusion is that estimates are clearly elevated for all three outcomes. Individual study RRs virtually all exceed 1.0, and based on random-effects meta-analyses of most-adjusted RRs, estimates are elevated for ever smoking (COPD 2.89, CI 2.63-3.17, n = 129 RRs; CB 2.69, 2.50-2.90, n = 114; emphysema 4.51, 3.38-6.02, n = 28), current smoking (COPD 3.51, 3.08-3.99, n = 120; CB 3.41, 3.13-3.72, n = 113; emphysema 4.87, 2.83-8.41, n = 22) and ex smoking (COPD 2.35, 2.11-2.63, n = 110; CB 1.63, 1.50-1.78, n = 105); emphysema 3.52, 2.51-4.94, n = 17). The consistency and strength of the relationships are consistent with a causal relationship. A causal relationship is supported by the fact that estimates are not materially affected by adjustment for confounding variables, and by the evidence of a dose-response relationship, with risk increasing with amount smoked and pack-years for all three outcomes and (based on more limited data) risk decreasing with increasing starting age for COPD and CB and with increasing quitting duration for COPD.

Our review also provides evidence that various characteristics of the study and RR affect risk estimates. For COPD, RRs are higher for males, for studies conducted in North America, for cigarette smoking rather than any product smoking, where the unexposed base is never smoking any product, and are markedly lower when asthma is included in the COPD definition. Variations by sex, continent, smoking product and unexposed group are in the same direction for CB, but less clearly demonstrated. For all outcomes RRs are higher when based on mortality, and for COPD are markedly lower when based on lung function.

This comprehensive review provides further insight into the relationship of smoking to COPD, CB and emphysema.

## Abbreviations

ATS: American Thoracic Society; BTS: British Thoracic Society; CB: Chronic Bronchitis; CI: Confidence Interval; COPD: Chronic Obstructive Lung Disease; ERS: European Respiratory Society; GOLD: Global Initiative for Chronic Obstructive Lung Disease; ICD: International Classification of Diseases; LCL: Lower Confidence Limit; MRC: Medical Research Council; REF: 6 character Reference code used to identify a study; RR: Relative Risk; UCL: Upper Confidence Limit

## Competing interests

PNL, founder of P.N.Lee Statistics and Computing Ltd., is an independent consultant in statistics and an advisor in the fields of epidemiology and toxicology to a number of tobacco, pharmaceutical and chemical companies.This includes Philip Morris International, Inc., the sponsor of this study. BAF is an employee of, and AJT a consultant to, P.N.Lee Statistics and Computing Ltd. All authors read and approved the final manuscript.

## Authors' contributions

BAF and PNL were responsible for planning the study. Final literature searches were carried out by BAF with PNL's assistance, with some earlier searches conducted by AJT. Data entry was either carried out by AJT and checked by BAF, or carried out by BAF and checked by PNL. Where appropriate, difficulties in interpreting published data or in the appropriate methods for derivation of RRs were discussed by BAF and PNL. The statistical analyses were conducted by BAF along lines discussed and agreed with PNL. PNL drafted the paper, which was then critically reviewed by BAF and AJT.

## Pre-publication history

The pre-publication history for this paper can be accessed here:

http://www.biomedcentral.com/1471-2466/11/36/prepub

## Supplementary Material

Additional file 1 **Methods**. .DOC file giving a fuller version of the Methods section than in the paper [320-324]. Particular topics described in more detail include the following:
• the rules for preferring one outcome definition to another where a study provides multiple qualifying alternatives, and giving the outcomes selected and alternatives not used for these studies. It also gives details of core and allied conditions for each of the three outcomes, and the definitions of COPD based on published criteria of lung function.
• the literature searching, including a flow chart.
• the methods by which RRs and CIs were derived, where required, from the data presented in the source papers.
• the statistical analyses conducted. It does not include any results itself, but describes the content and structure of additional files 4 to 13 that do provide detailed statistical results. Click here for file

Additional file 2 **Studies**. .DOC file concerning the 218 studies included on the database. This describes which studies provided data for which outcome and gives details of the overlapping and linked studies, as well as fuller distributions of study characteristics than those given in the paper and also details of study populations and exclusions. For each of the three outcomes, a study by study description of the full definition of the outcome and source of diagnostic information is given. Click here for file

Additional file 3 **RRs**. .DOC file concerning the RRs included on the database. This gives the numbers of RRs per study as well as fuller distributions than those given in the paper of the characteristics of the RRs for the major smoking indices and the dose-response indices, and of the characteristics of the sets of RRs for the dose-response indices. It also lists which studies provide RRs for which indices, and gives details of the results of checking RRs for apparent errors. Click here for file

Additional file 4 **MetaMajorCOPD**. .RTF file giving the full results of the meta-analyses for the major smoking variables-ever smoking, current smoking, ever or current smoking, and ex smoking-for COPD. Click here for file

Additional file 5 **MetaMajorCB**. .RTF file giving the full results of the meta-analyses for the major smoking variables for CB. Click here for file

Additional file 6 **MetaMajorEMP**. .RTF file giving the full results of the meta-analyses for the major smoking variables for EMP. Click here for file

Additional file 7 **MetaDoseCOPD**. .RTF file giving the full results of the meta-analyses for the dose-related smoking variables-amount smoked, age of starting to smoke, pack-years, duration of smoking, duration of quitting vs. never smoking, and duration of quitting vs. current smoking-for COPD. Click here for file

Additional file 8 **MetaDoseCB**. .RTF file giving the full results of the meta-analyses for the dose-related smoking variables for CB. Click here for file

Additional file 9 **MetaDoseEMP**. .RTF file giving the full results of the meta-analyses for the dose-related smoking variables for EMP. Click here for file

Additional file 10 **MetaSumm**. .XLS file allowing the user readily to view selected results from the full meta-analysis output in additional files 4 to 9. Click here for file

Additional file 11 **MetaRegressionTables**. .DOC file giving the full results of the meta-regressions carried out for ever smoking and current smoking. Click here for file

Additional file 12 **DoseSetsList**. .XLS file listing and plotting the study-specific sets of dose-response data. Click here for file

Additional file 13 **DoseNotMetaData**. .DOC file summarizing the results of the dose-related data that could not be included in the dose-related meta-analyses. Click here for file
