# Supplementary material for: Systematic review with meta-analysis of the epidemiological evidence relating smoking to COPD, chronic bronchitis and emphysema
Source: BMC Pulm Med. 2011 Jun 14;11:36. doi: 10.1186/1471-2466-11-36 (PMC3128042; doi:10.1186/1471-2466-11-36)
Supplement: Additional file 1 — Methods. .DOC file giving a fuller version of the Methods section than in the paper [320-324]. Particular topics described in more detail include the following: • the rules for preferring one outcome definition to another where a study provides multiple qualifying alternatives, and giving the outcomes selected and alternatives not used for these studies. It also gives details of core and allied conditions for each of the three outcomes, and the definitions of COPD based on published criteria of lung function. • the literature searching, including a flow chart. • the methods by which RRs and CIs were derived, where required, from the data presented in the source papers. • the statistical analyses conducted. It does not include any results itself, but describes the content and structure of additional files 4 to 13 that do provide detailed statistical results. [file 1471-2466-11-36-S1.DOC]

**Systematic review with meta-analysis of the epidemiological evidence relating smoking to COPD, chronic bronchitis and emphysema**

Barbara A Forey, Alison J Thornton and Peter N Lee

**Additional file 1: Methods**

Contents

[Inclusion and exclusion criteria 5](#__RefHeading___Toc293476391)

[Definition of the outcomes 5](#__RefHeading___Toc293476392)

[COPD 5](#__RefHeading___Toc293476393)

[CB 7](#__RefHeading___Toc293476394)

[Emphysema 8](#__RefHeading___Toc293476395)

[Choice of outcome 8](#__RefHeading___Toc293476396)

[*COPD* 8](#__RefHeading___Toc293476397)

[*Exceptions for COPD* 10](#__RefHeading___Toc293476398)

[*CB* 11](#__RefHeading___Toc293476399)

[*Emphysema* 12](#__RefHeading___Toc293476400)

[Literature searching 13](#__RefHeading___Toc293476401)

[Diagram of literature searching 15](#__RefHeading___Toc293476402)

[Identification of studies 16](#__RefHeading___Toc293476403)

[Data recorded 17](#__RefHeading___Toc293476404)

[Identifying which RRs to enter 18](#__RefHeading___Toc293476405)

[The major smoking indices 18](#__RefHeading___Toc293476406)

[Dose-related smoking indices 19](#__RefHeading___Toc293476407)

[Confounders adjusted for 21](#__RefHeading___Toc293476408)

[Strata 22](#__RefHeading___Toc293476409)

[Derivation of RRs 22](#__RefHeading___Toc293476410)

[Correction for zero cell 23](#__RefHeading___Toc293476411)

[Combining independent RRs 24](#__RefHeading___Toc293476412)

[Combining non-independent RRs 24](#__RefHeading___Toc293476413)

[Ratio of rates 25](#__RefHeading___Toc293476414)

[CI estimated from crude numbers 25](#__RefHeading___Toc293476415)

[Converting CI from a different confidence level 25](#__RefHeading___Toc293476416)

[Inverting from a different denominator 25](#__RefHeading___Toc293476417)

[Using symmetry of the CI 26](#__RefHeading___Toc293476418)

[Using SMRs, or expected values 26](#__RefHeading___Toc293476419)

[Data entry and checking 26](#__RefHeading___Toc293476420)

[Meta-analyses 27](#__RefHeading___Toc293476421)

[Overview 27](#__RefHeading___Toc293476422)

[Selecting RRs for the meta-analyses 27](#__RefHeading___Toc293476423)

[Carrying out the meta-analyses 28](#__RefHeading___Toc293476424)

[The major smoking indices 29](#__RefHeading___Toc293476425)

[The dose-related smoking indices 29](#__RefHeading___Toc293476426)

[The meta-analysis tables 31](#__RefHeading___Toc293476427)

[*Numbering system for the tables* 31](#__RefHeading___Toc293476428)

[*The full tables* 32](#__RefHeading___Toc293476429)

[*Notation* 35](#__RefHeading___Toc293476430)

[*Characteristics considered* 38](#__RefHeading___Toc293476431)

[*The summary tables* 41](#__RefHeading___Toc293476432)

[Forest plots 42](#__RefHeading___Toc293476433)

[Funnel plots 42](#__RefHeading___Toc293476434)

[Meta-regression 42](#__RefHeading___Toc293476435)

[Other files showing dose-related data 45](#__RefHeading___Toc293476436)

[Additional analyses 46](#__RefHeading___Toc293476437)

[Software 46](#__RefHeading___Toc293476438)

[Table 1 Core and allied conditions for COPD, CB and emphysema in successive revisions of the ICD 47](#__RefHeading___Toc293476439)

[Table 2 Definitions of COPD based on published criteria of lung function 48](#__RefHeading___Toc293476440)

[Table 3 Outcomes selected and alternatives not used for studies with multiple qualifying outcomes 49](#__RefHeading___Toc293476441)

[3A COPD 49](#__RefHeading___Toc293476442)

[3B CB 52](#__RefHeading___Toc293476443)

[3C Emphysema 54](#__RefHeading___Toc293476444)

[Table 4 Meta-analysis tables 55](#__RefHeading___Toc293476445)

[4A Main and variant analyses for major smoking indices 55](#__RefHeading___Toc293476446)

[4B Analyses for dose-related indices 56](#__RefHeading___Toc293476447)

[Table 5 Abbreviations used in listings 57](#__RefHeading___Toc293476448)

References – see main paper

# Inclusion and exclusion criteria

Attention was restricted to publications before 2007, to epidemiological case-control, prospective or cross-sectional studies on the prevalence or incidence of, or mortality from, COPD, CB or emphysema, and to studies where relative risk (RR) estimates were available or could be derived (or a statement on their statistical significance provided) for one or more defined “major indices” (ever, current or ex smoking compared with never smoking) or “dose-related indices” (amount smoked, age of starting to smoke, pack-years smoked, duration of smoking or duration of quitting). Note that in this paper the term RR is used generically to include various estimators of it, including the odds ratio and the hazard ratio.

There were a number of exclusion criteria. The study should not be conducted in a population at especially high risk of respiratory disease (e.g. workers in risky occupations), in children or adolescents, in subjects selected as having co-existing diseases or conditions, or in subjects from atypical populations where the prevalence of smoking or disease was likely to be highly unusual. Uncontrolled case studies were not included, as RRs could not be calculated. Also excluded were studies of disease exacerbation or of undiagnosed disease, studies restricted to symptom-free subjects, and studies where the only available results were adjusted for symptoms or precursors of disease.

# Definition of the outcomes

## COPD

While the inclusion criteria refer to COPD, this term is relatively recent, and studies using other descriptions of this outcome were also included. Depending on the publication, the outcome COPD might be based on appropriate combinations of International Classification of Diseases (ICD) codes, on relevant lung function criteria, on a combination of lung function criteria and symptoms (but not on symptoms only), or on relevant combinations of diagnosed conditions, such as CB or emphysema, or CB, emphysema or asthma. Diagnoses might be extracted from medical records, or reported in questionnaires.

Where a study defined the outcome based on a range of ICD codes, the range had to include specific “core codes”, and could also include one or more defined “allied codes”. The core and allied conditions according to the various revisions of the ICD are shown here in Table 1. The core codes include CB and emphysema, and the allied codes include asthma and bronchiectasis, in every revision. Thus broader-ranging disease definitions (e.g. respiratory disease) were not accepted. Studies were included if the set of codes used was satisfactory, even though the author did not refer to it as COPD, perhaps using terms such as CB, or CB and emphysema. Exceptionally, studies were accepted when relatively rarely occurring conditions not defined as core or allied were part of the outcome considered by the authors, or when rare core conditions were excluded, e.g. the diagnosis used in study WALD based on ICD 9: 416, 491, 492, 496 and 519, which included the rarely occurring code 416, and the diagnosis used in study TVERDA, which omitted code 496 for cases coded under the 9^th^ revision, as the 8^th^ revision was used for most of its follow-up period.

Diagnoses of COPD based solely on lung function measured by spirometry were accepted. This includes studies using criteria published by the Global Initiative for Chronic Obstructive Lung Disease (GOLD) [3,4], the British Thoracic Society (BTS) [5], the European Respiratory Society (ERS) [6] and the American Thoracic Society (ATS) [7-9] (Table 2). Studies were accepted where spirometry was conducted without use of a bronchodilator, even where the published criteria specified its use. Other similar lung function criteria used in individual studies were also accepted, irrespective of whether named COPD by the original authors.

Studies which did not base their criteria for COPD on ICD codes or on lung function only were also accepted if the outcome used was based on: lung function and symptoms; CB or emphysema (and/or asthma); CB, emphysema or COPD (and/or asthma); COPD otherwise defined (e.g. a reported diagnosis plus lung function criteria); or COPD not otherwise specified. The British Medical Research Council (MRC) criterion of daily productive cough for at least 3 consecutive months for more than two successive years [10,11] was recognized as a set of symptoms defining CB.

Outcomes not accepted for COPD included: CB or emphysema separately; outcomes based on symptoms only and not lung function; acute or unspecified bronchitis; and non-specific respiratory disease.

## CB

Where a study defined the outcome based on a range of ICD codes, the range had to include the specific “core codes” for CB and could also include one or more “allied codes” indicating acute bronchitis or bronchitis unspecified as acute or chronic (Table 1).

Diagnoses were also accepted if based on medical records, diagnosis in the course of the study, self-report of physician diagnosis, self-report of has/had disease, or based on symptoms. Diagnoses or symptoms referred to as CB by the original author were preferred, but “bronchitis” was acceptable if the context clearly indicates that it is chronic. Diagnoses based on symptoms not referred to as CB were also accepted, but the definition had to include at least chronic cough and phlegm (so that “cough and/or phlegm” was not acceptable). Results from analyses where controls but not cases could have asthma were not accepted.

## Emphysema

Where the ICD was used, the definition had to be based on the specific “core code” for emphysema (Table 1). There were no “allied codes”. Diagnoses were also accepted based on medical records, diagnosis in the course of the study, self-report of physician diagnosis or self-report of has/had disease.

## Choice of outcome

For each of the three diseases (COPD, CB and Emphysema) some studies provide results for more than one acceptable outcome definition. Results are entered on the database for only a single definition (as shown in Tables 5 and 6 of Additional File 2), with the choice generally determined according to the following orders of preference. Studies for which a choice existed are listed in Table 3, which gives the outcome definition for which data were entered and the alternatives which were not used.

### *COPD*

Where a study provided results for more than one definition of COPD, one was chosen using the order of preference as follows:

1. Results based on ICD codes if available.
   1. If the actual codes are defined in the paper, then prefer the definition with the fewest allied conditions provided all the Core conditions are included (see Table 1 for a definition of Core and Allied conditions). Note that the outcome could be included as COPD according to the ICD codes even if named “chronic bronchitis” by the original author;
   2. If the codes are unspecified by the authors then prefer the outcome the authors refer to as COPD (or COLD) to the outcome the authors refer to as “CB or Emphysema”;
   3. Prefer results for mortality to results for incidence;
   4. Prefer results for underlying cause to results for any mention (underlying or contributory) on the death certificate.
2. Based on lung function only.
   1. Prefer the definition nearest to GOLD stage I (= post-bronchodilator FEV1/FVC <70%);
   2. Prefer a definition of any disease to one of severe disease;
   3. Prefer criteria based on FEV1/FVC rather than on FEV1 vs predicted;
   4. Prefer results where subjects with restrictive airflow are included in the base (comparison) group to results where they are excluded.
3. Based on lung function and symptoms. (Note this includes GOLD stage 0)
4. Based on medical records or diagnosis in the course of this study.
   1. Prefer COPD as named by original author (irrespective of further definition);
   2. Otherwise the following preference applies.
      1. COPD, CB and/or emphysema;
      2. CB and/or emphysema;
      3. COPD, CB, emphysema and/or asthma;
      4. CB, emphysema and/or asthma.
5. Based on self-report of physician diagnosis, with sub-preferences as in 4.
6. Based on self-report of has/had disease (not specified as physician diagnosis), with sub-preferences as in 4.
7. Other (e.g. lung function and diagnosis).

### *Exceptions for COPD*

Although results were generally selected according to these rules, there were some exceptions.

KAHN The outcome (mortality, underlying cause COPD (ICD 7:501-502,527.1 from 1954-68; 501-502, 527.1, 527.2 from 1969-80) included one allied condition (527.2) and had results available for the longest follow-up ([141]), and was selected for the major smoking indices. The alternative outcome (mortality, underlying cause bronchitis/emphysema 500-502,527.1) included a different allied condition (500) and was selected for the dose-related indices despite the shorter follow-up ([139]). Further alternatives were not selected, in line with the rules above, because they included both underlying and contributory mortality ([140]), or included the additional allied condition asthma (214) ([142]).

KARAKA The outcome selected was : COPD (ERS criteria=FEV1/VC<88% pred M <89% pred F ) and/or chronic bronchitis (chronic cough and chronic phlegm for 3 months for at least 2 years), and/or physician diagnosed CB, emphysema or COPD. The alternative available was : self-reported history of COPD, chronic bronchitis, emphysema or respiratory symptoms (such as breathlessness, chronic cough and chronic phlegm for 3 months for at least 2 years), which would have included subjects with breathlessness only.

MANNI1 The outcome selected is : Low lung function (FEV1/FVC <0.7 and FEV1 <80%) predicted (i.e. GOLD II). This was preferred to GOLD I COPD as reported by Hyman and Reid [196] because the analyses in that paper excluded subjects who reported asthma, and were restricted to subjects age 30+ and attending at least 3 survey waves; also to airflow limitation (FEV1/FVC <lower limit of normal LLN) (subdivided into diagnosed, with asthma diagnosed or undiagnosed) from Coultas et al. [194] because that analysis was limited to whites age 45+.

NIHLEN The outcome selected is : self-report of physician diagnosis of chronic bronchitis and/or emphysema and/or COPD, chosen in preference to prevalence of COPD by spirometry, because that was available only in a very small subset of subjects (164 from a specific municipality [221], out of 4000 in the main study)

SARGEA The outcome selected was that termed obstructive airways disease (OAD) by the original authors, defined as physician-diagnosed bronchitis, emphysema or asthma, or FEV1 <80% predicted and FEV1/FVC<70%. This was chosen in preference to self-reported OAD; and to self-reported diagnosis of or medication for OAD.

### *CB*

The order of preference for CB was as follows:

1. Results based on ICD codes if available.
   1. If the actual codes are defined in the paper, then prefer the definition with the fewest allied conditions provided all the Core conditions are included;
   2. If the codes are unspecified by the authors then use the outcome the authors refer to as CB;
   3. Prefer results for mortality to results for incidence;
   4. Prefer results for underlying cause to results for any mention (underlying or contributory) on the death certificate.
2. Based on medical records or diagnosis in the course of this study
   1. Prefer CB as named by the original author, although “bronchitis” is acceptable if the context clearly indicates that it is chronic;
   2. Prefer results irrespective of co-existing asthma to results excluding all asthmatics. (Note that results from analyses where asthma status is treated differently for the CB cases and non-cases are not acceptable.)
3. Based on self-report of physician diagnosis, with sub-preferences as in 2.
4. Based on self-report of has/had disease (not specified as physician diagnosis), with sub-preferences as in 2.
5. Based on symptoms.
   1. Prefer symptoms defined as CB by the original author;
   2. Otherwise use a symptom definition as close as possible to cough and phlegm for at least 3 months for at least 2 years. The definition must include at least chronic cough and phlegm – note that “cough and/or phlegm” is not acceptable.
6. Other.

There were no exceptions in the application of these rules for CB.

### *Emphysema*

The order of preference for emphysema was as follows:

1. Results based on ICD codes if available.
   1. If the actual codes are defined in the paper, then use the Core condition;
   2. If the codes are unspecified by the authors then use the outcome the authors refer to as emphysema;
   3. Prefer results for mortality to results for incidence;
   4. Prefer results for underlying cause to results for any mention (underlying or contributory) on the death certificate.
2. Based on medical records or diagnosis in the course of this study, preferring results for any grade, type or anatomical location of emphysema to those for a specified grade, type or location.
3. Based on self-report of physician diagnosis, with preferences as in 2.
4. Based on self-report of has/had disease (not specified as physician diagnosis), with preferences as in 2.
5. Other.

There were no exceptions in the application of these rules for emphysema.

# Literature searching

In an earlier project (supported by Philip Morris, but unpublished), 915 papers were identified from a Medline search, using the MeSH terms chronic bronchitis and symptoms, emphysema, lung function, genetic determinants, mortality, adults and smoking, conducted in September 2001. The reference lists of those papers were searched (by AJT), relevant papers obtained and the process repeated, with a further 492 papers identified in all. These papers, published in the period 1954-2002, were obtained.

Further Medline searches were conducted in August 2006 (by AJT), and were repeated in April 2008 (by BAF) with publication date limited to the end of 2006 (but with no limit on the start date). The search strategy used was:

(“Smoking/adverse effects”[Mesh] OR “Smoking/epidemiology”[Mesh] OR “Smoking/mortality”[Mesh]) AND “Pulmonary Disease, Chronic Obstructive”[Mesh] AND “humans”[Mesh]

Note that in the Mesh Database, the Mesh term “Pulmonary Disease, Chronic Obstructive” is described as having subcategories of COPD including chronic bronchitis and pulmonary emphysema.

With the exception of some papers identified through the 2008 Medline search (which were rejected on the basis of the online abstract if available, or the title), all papers were obtained (if possible) and examined. Although no other systematic literature searching was undertaken, some additional references were identified from the reference lists of the examined papers, and from the extensive files on smoking and health accumulated by P N Lee Statistics and Computing.

The Diagram shows the numbers of papers considered at each stage. Finally, a total of 2448 papers were considered, of which 298 were accepted and 2150 rejected. References to the included papers are given in the main paper. Table 1 of the paper summarises the reasons for rejection, and a database (in Reference Manager format) of the 2150 rejected papers, including a brief note on reason for rejection, is available on request.

## Diagram of literature searching

^*^ See Table 1 of paper for further detail on reasons for rejection

Medline search September 2001

915

Searched for secondary references

492

Medline search August 2006

duplicates eliminated

304

Medline search

April 2008

duplicates eliminated

Total 1831 potentially relevant papers

617 rejected^*^ on basis of abstract or title :

No abstract, inappropriate title 58

No original data280

Inappropriate population 97

Inappropriate design154

Inappropriate outcome 9

No relevant smoking results 19

Informal searching

298 accepted

1528 rejected^*^ :

No original data150

Inappropriate population246

Inappropriate design169

Inappropriate outcome557

No relevant smoking results406

Total 2150 rejections

80

40

5 unobtainable

1826 papers obtained

# Identification of studies

Relevant papers were classified into distinct studies, identifying multiple papers referring to the same study (with one being designated as the principal paper), and multiple studies reported within a paper. Studies were named by a unique alphanumeric reference code (REF) of up to 6 characters (e.g. DICKIN or CHEN3), based on the name of the principal author and distinguishing multiple studies with the same author, and they were then entered on a database (see also next section). On occasion, an original study was split and entered as separate studies (e.g. the UK Doctors study was entered separately as DOLL1 for males and DOLL2 for females as the follow-up periods differed markedly).

Some sets of studies were noted on the database as having overlaps or links with other studies. In theory, RRs being meta-analysed should come from independent studies involving distinct cases, as cases featuring in multiple studies may be double-counted. In practice, avoiding such double-counting is difficult, and not necessarily the best option for various reasons, including loss of power. For the purposes of analysis, the study links were divided into three types, as shown in Table 2 of Additional file 2. The first type includes studies which, although linked, do not overlap. Here, the subjects may be included in more than one study, but the cases are distinct, so that RRs from the two studies are independent. In particular, this arises where a study provides baseline results for prevalence of disease, the disease-free subjects then forming the at-risk population for subsequent analyses of incidence or mortality. The second type includes studies with a modest degree of overlap, which cannot be disentangled and which it was decided to ignore. The third type contains sets of studies which probably or definitely do overlap. Here the member of the set containing the most appropriate data (e.g. largest study size, widest geographical coverage or longest follow-up) was identified as the ‘principal study’ and the other members as ‘subsidiary studies’, from which RRs are only included in meta-analyses where equivalent results are not available for the principal study. Note that the allocation as a principal or subsidiary study did not take study quality into account.

# Data recorded

For each study, relevant information was entered onto a study database and a linked RR database.

The study database contains a single record for each study, describing the relevant publications, sexes considered, age range, location, timing, length of follow-up, whether principal or subsidiary, details of overlaps or links with other studies, study design (case-control, prospective or cross-sectional), type of population studied, major study weaknesses identified, details of the definition of the outcome(s), number of cases, number of subjects, types of controls and matching factors used in case-control studies, confounding variables considered, and availability of results for each of the smoking indices. Fuller details of the study database structure are available on request.

The RR database holds the detailed results, typically containing multiple records for each study. Each record refers to a specific RR, describing the comparison made and the actual results, as well as the study REF to link it to the relevant study. The descriptive information includes the outcome, the sex for the RR (males, females, combined) and, for prospective studies, the analysis type (prevalence or incidence). The smoking exposure is defined by smoking status (ever, current or ex) and smoking product (any, cigarettes irrespective of other products, or cigarettes only), together with similar information about the unexposed base. For the dose-related indices, the level of exposure is recorded. The source of the RR (publication, table and page numbers) is also recorded, as are details on adjustment variables, including, for the dose-related indices, adjustment for other aspects of smoking. The results recorded include numbers of exposed and unexposed cases, and, for unadjusted results, numbers of exposed and unexposed controls or disease-free subjects for prevalence analyses, or numbers or person-years at risk for incidence analyses. The RR estimate itself and its lower and upper 95% confidence limits (LCL and UCL) are always recorded, with the odds ratio [OR] chosen if available for a prevalence analysis. For an incidence analysis, relative risks (or hazard ratios if provided) were chosen in preference to ORs, which were used only where RRs were not available (typically when estimated from a multiple logistic regression), and this is noted in the database. Data may be as provided in the source, or derived by various means, with the method of derivation noted, possible methods of derivation being described below. Fuller details of the RR database structure are also available on request.

# Identifying which RRs to enter

For each of the three outcomes (COPD, CB and emphysema) RRs were entered relating to defined combinations of smoking index (major or dose-related), confounders adjusted for, and strata, as described below.

## The major smoking indices

The intention was to enter RRs comparing current smokers, ever smokers or ex smokers with never smokers. However various near equivalents were accepted, depending on the definition used in the source. Thus, a smoker may be defined as a regular or daily smoker, with never smokers including occasional smokers, and current smokers may include, and ex smokers exclude, those who stopped smoking recently, with a maximum of two years accepted. Where no results were available using a more strictly defined never smoker base, the definition of never smokers could include those smoking up to 5 cigarettes per day or up to 5 pack-years. If available, results were entered for five comparisons: any product vs never any product, cigarettes vs. never any product, cigarettes only vs. never any product, cigarettes vs. never cigarettes, and cigarettes only vs. never cigarettes. Here “cigarettes” is irrespective of whether other products (i.e. pipes and cigars) are also smoked, while “cigarettes only” excludes mixed smokers of other products. RRs with an unexposed base which includes smokers who have only ever smoked other products were accepted (i.e. never cigarettes). The availability of results referring specifically to smoking of pipes, cigars or other combinations, or to specific types of cigarettes (hand-rolled, filter/plain) was noted on the database, but RRs were not entered.

## Dose-related smoking indices

Results were entered for five measures of smoking-related dose: amount smoked, age of starting to smoke, pack-years (which is defined as daily number of cigarettes smoked multiplied by years of smoking, divided by 20), duration of smoking, and duration of quitting. RRs were expressed relative to never smokers (or near equivalent), if available or relative to nonsmokers otherwise. For duration of quitting, RRs were also expressed relative to current smokers. The availability of results for other aspects of smoking-related dose, such as inhalation, or tar or nicotine level, was noted on the database, but RRs were not entered.

For amount smoked and pack-years, exposure was often measured as numbers of cigarettes, but results including smoking of other products expressed as cigarette-equivalents, or as grams of tobacco were also accepted. Results originally presented as lifetime cigarettes, lifetime packs or cigarette years were converted to pack-years.

RRs are often available for each of a set of dose categories (e.g. 1-10, 11-20, 21-30 etc cigarettes/day) compared with a common base of never smokers, and were entered provided the set included at least two dose categories. Near equivalents for never smoking were accepted as described previously, and, failing that, a base of non-smoking (or near equivalent) was accepted (e.g. current smokers of 1-10, 11-20 etc compared with never smokers and ex smokers combined).

Further RRs were entered, restricted to smokers, so allowing adjustment for other smoking variables (e.g. adjusted for amount smoked when studying age of starting to smoke), which is not possible in an analysis with never smokers as the base group. For amount smoked, pack-years and duration of smoking, where risk was expected to rise as values increased, these were expressed relative to smokers with a low value as the common base (e.g. comparing 11-20, 21-30, 31+ cigs/day with 1-10 cigs/day). For age of starting to smoke, and duration of quitting, where risk was expected to rise as values decreased, the RRs were expressed relative to smokers with a high value as the common base. For duration of quitting, additional RRs were entered relative to a base of current smokers. Whatever base was used, sets of RRs were entered for all the combinations of smoking status and product described in the previous section, except that duration of quitting refers only to ex smokers.

The foregoing comments assume the dose categories cover the full range of possible values for the dose measure. Where this was not so, only results for the highest exposure compared with a never and/or a low exposure base were entered (e.g. 40+ cigarettes/day compared with either never smokers, or with 1-10 cigarettes/day). Otherwise, data for incomplete sets of dose categories were not entered on the database.

Sometimes, the only available results had a base group combining never smokers with a low exposure group, beyond the limit we defined as acceptable. For example, where the combined group of never smokers and smokers of up to 10 cigs/day was compared with smokers of 11-20, 21-30, 31+ cigs/day. These results were entered compared with the base as given, but no attempt was made to enter data relative to 11-20 cigs/day.

Where results for the dose-related smoking indices were provided, but not in the form of categorical data, a comment was recorded in the database. This includes results from regression analyses expressed as risk per unit dose, differences in mean dose between subjects with and without disease, and general statements that risk was or was not related to level of exposure.

## Confounders adjusted for

For case-control and cross-sectional studies, results were entered adjusted for the greatest number of potential confounding variables for which results were available, and also unadjusted (or adjusted for the smallest number of confounders). For prospective studies, only age-adjusted results were considered, with results entered adjusted for age and the greatest number of confounders, and for age only or age and the smallest number of confounders; unadjusted results were entered only if no age-adjusted results were available. The alternative RRs are subsequently referred to as “most-adjusted” and “least-adjusted”.

For dose-related RRs restricted to smokers in which the adjusting variables for the most-adjusted RR included another aspect of smoking, an additional RR was entered (if available) with the same adjusting variables, but without adjustment for the other aspect of smoking. Similarly, if a RR was available with the same adjusting variables as the least-adjusted, but additionally adjusted for another aspect of smoking, then that RR was also entered.

## Strata

Results were entered for males and females separately when available. Combined sex results were only entered when the equivalent results (i.e. for the same outcome, smoking index, and confounders) were not available. Results were not entered stratified by other variables, such as age or socioeconomic group, but their availability was noted on the database.

# Derivation of RRs

Adjusted RRs and their 95% CIs were entered as given when available. Unadjusted RRs were calculated from their 2 × 2 table, if available using standard methods (e.g. [12]) and otherwise they were entered as given. If the numbers of cases are denoted by *a_i_* and the numbers of controls (or the disease-free population in a cross-sectional study) by *b_i_*, where the subscript *i* = 0 refers to the unexposed group and *i* = 1 refers to the exposed group, then the RR and its 95% confidence limits LCL and UCL (as estimated by the OR) are calculated by:

RR = (*a*_1_ *b*_0_) / (*a*_0_ *b*_1_) (1)

LCL = RR / *φ* (2)

UCL = RR *φ* (3)

where *φ*, a factor based on the variance of the RR, is given by:

ln( *φ* ) = N_95_$\sqrt{((1/a_{0})+(1/a_{1})+(1/b_{0})+(1/b_{1}))}$ (4)

Here N_95_ denotes the inverse standard normal value for 95% confidence (i.e. very close to 1.96).

For an incidence analysis, b_i_ denotes the at-risk population, and the formulae to calculate the RR and its CI are the same, except that

ln( *φ* ) = N_95_$\sqrt{((1/a_{0})+(1/a_{1})-(1/b_{0})-(1/b_{1}))}$ (5)

If both a 2 × 2 table and an unadjusted RR/CI were provided by the author, then the RR/CI calculated as above was used, any discrepancy from that originally given being noted in the database.

The 2 × 2 table may be constructed by summing groups (e.g. adding current and ex smokers to obtain ever smokers, or adding over other stratifying factors), or from a percentage distribution.

A variety of other methods were used to provide estimates of the RR and CI in other circumstances. The main methods are described below. Calculations were mainly carried out using Excel spreadsheets.

## Correction for zero cell

If the 2 × 2 table has one cell with value zero, the unadjusted RR and CI cannot be calculated by the usual formulae. The method used was to add a correction of 0.5 to each of the four cells, and then apply the formulae.

## Combining independent RRs

Combining RRs over strata was carried out using fixed-effect meta-analysis [13]. The resulting estimate is adjusted for the stratifying variable. When this combined RR is subsequently used in a fixed-effects meta-analysis, the end result will be exactly the same as if all the original RRs had been included. This method is also appropriate for combining RRs for individual disease groups, provided they are independent estimates (i.e. each disease group has a separate control group).

When combining strata from data available as a 2 × 2 × ℓ table (i.e. ℓ levels of the stratifying variable), adjacent levels were combined if necessary to avoid any empty cells, or if that was not possible, then the correction for zero cell (as described above) was carried out within strata.

## Combining non-independent RRs

When non-independent RRs were to be combined, for instance where adjusted RRs are available for current and ex smokers, each versus never smokers, then the method of Hamling *et al* [14] was used to provide a combined estimate for ever smokers. This method starts from a source table giving adjusted RRs and CIs for *n* exposed groups relative to a single non-exposed base group. The hypothetical underlying 2 × (*n* + 1) table of numbers of ‘adjusted cases and controls’ is estimated, these then being summed to give the required groups for the numerator and denominator, and the resulting 2 × 2 table used with the usual formulae to estimate the adjusted RR and CI. A variation of the method allows non-independent disease groups to be combined. Thus when RRs for several disease groups were available, each relative to a single shared control/disease-free group, the disease groups can be combined, or one disease group (e.g. COPD) can be compared with a combination of another disease group (e.g. other respiratory symptoms) and the control group.

## Ratio of rates

Results from prospective studies are often presented as mortality rates rather than as RRs. If they are presented separately for the exposed and unexposed groups (R1 and R0), then the RR is calculated by:

RR = R1 / R0 (6)

## CI estimated from crude numbers

When an adjusted RR was presented originally without a CI or p-value, but the corresponding 2 × 2 table (or numbers of exposed and unexposed cases from a prospective study) was available, then the original RR was used and its CI estimated by assuming its width is the same as the width of the interval for the equivalent unadjusted RR. In fact, the estimated interval will be narrower than the true one (since adjustment widens the interval [15]), and thus this method will increase the weight that the estimate is given when entered into a meta-analysis. However this will usually have a very small effect, the only alternative being to omit the RR altogether from all meta-analyses. The same method is used for a RR calculated as a ratio of rates if no CIs were presented for the rates.

## Converting CI from a different confidence level

Where the RR and CI were originally presented with a different confidence level *c* the 95% CIs were calculated from the c% CI using formulae (2) and (3) with:

ln ( *φ* ) = N_95_ (ln (UCL*c*) – ln (LCL*c*) ) / 2N*c* (7)

## Inverting from a different denominator

If the RR and CI were originally presented with the exposed and unexposed groups reversed from those required, then the required values were calculated by:

RR = 1 / RR*O*  (8)

LCL = 1 / UCL*O*  (9)

UCL = 1 / LCL*O*  (10)

where the subscript *O* indicates the values as originally presented.

If RR/CIs for separate levels of an exposure variable are given relative to a common base level, then RR/CIs each relative to another of the levels can be estimated by the method described above for “Combining non-independent RRs”.

## Using symmetry of the CI

When only two of the RR, LCL and UCL are given, then the third can be calculated to give a CI symmetrical about the RR. For instance if UCL is missing, then formula (3) is used with:

*φ* = RR / LCL, i.e. (11)

UCL = RR^2^ / LCL (12)

## Using SMRs, or expected values

When the observed numbers of cases were given together with SMRs or expected values relative to a standard (e.g. national) population, then the ‘ratio of two standardised ratios’ was calculated as described by Altman *et al* [320] using the program CIA (Confidence Interval Analysis) [12].

# Data entry and checking

Master copies of all the papers in the study file were read closely. The information to be entered for a study was identified and highlighted on the papers (with notes made as necessary) to facilitate later checking. Where more than one paper was available for the study, a principal publication to provide most of the information was selected, though details of interest described only in other publications were also identified. Occasionally, descriptions of some study aspects conflicted between papers. Where necessary, the most likely version was determined by discussion between the authors of this report, sometimes after attempts to contact the original authors, with notes on the problem being recorded. Preliminary calculations and data entry were carried out by AJT and checked by BAF, or carried out by BAF and checked by PNL, and an automatic program investigated the completeness and consistency of the data. RR/CIs underwent validation checks ([15]).

Detailed instructions used for data extraction and entry onto the databases, along with full details of the automated checks carried out, are available on request.

# Meta-analyses

## Overview

A series of meta-analyses was conducted for various smoking indices for each of the three main outcomes (COPD, CB or emphysema). Each meta-analyses was repeated, based on most-adjusted RRs and on least-adjusted RRs. For each meta-analysis conducted, combined estimates were made first for all the RRs selected, then for RRs subdivided by level of various characteristics, testing for heterogeneity between levels. Results are presented in Tables (both full and summary) and plots. Further detail is given in the following sections.

## Selecting RRs for the meta-analyses

All meta-analyses are restricted to records where values are available for both the RR and the CI. The process of selecting those RRs to include in an analysis is quite complex as it must simultaneously address the two main objectives of inclusion of all relevant data and avoidance of double-counting.

When defining relevant data for an analysis (e.g. of current cigarette smoking), a single specific value of a smoking index may be chosen, and studies with no such RRs are excluded. However on occasion, a number of values of a smoking index may be acceptable in an analysis, (e.g. any product smoking, cigarette smoking or cigarette only smoking), and if a study has more than one acceptable RR, the one to be used in the meta-analysis has to be determined by an order of preference defined specifically for that meta-analysis. Similarly orders of preference may be required for the unexposed base, or for most-adjusted or least-adjusted RRs. When multiple orders of preference are specified, the sequence of implementation may affect the selection, so preferences for the most important aspects of the analysis, usually concerning smoking, are implemented first. As smoking results may differ between the sexes (e.g. a study may provide RRs for smoking of any product for males but RRs for smoking of cigarettes for females, or may provide unadjusted results for separate sexes, but adjusted results only for sexes combined), care is taken to ensure that the most appropriate RR is chosen within each sex stratum, with a preference for single sex to sexes-combined results implemented later. Finally, RRs from a subsidiary study are retained only where no eligible RRs are available from its principal study.

## Carrying out the meta-analyses

The method used to carry out meta-analyses of selected RRs is that described by Fleiss and Gross [13]. Both fixed-effect and random-effects meta-analysis have been conducted to produce combined estimates. Fixed-effect meta-analysis assumes a common underlying RR estimate and only takes into account within-study variability, whereas random-effects meta-analysis also takes into account between-study variability. Where there is no evidence of heterogeneity, the two analyses give the same results. Heterogeneity has been quantified by H, the ratio of the heterogeneity chisquared to its degrees of freedom. If required, the statistic I^2^ [17] can be calculated directly from H using the formula I^2^ = 100 (H-1)/H.

For all the meta-analyses conducted, a test of publication bias using Egger’s test [16] was also included.

## The major smoking indices

For the major smoking indices, four broad types of meta-analysis were conducted: A ever smoking, B current smoking, C ever smoking (but with current smoking used if ever smoking not available) and D ex smoking. In what is referred to as the main analysis, smoking of any product is preferred by selecting RRs in the following order of preference:

(1) smoking of any product vs. never smoked any product,

(2) smoking of cigarettes vs. never smoked any product,

(3) smoking of cigarettes only vs. never smoked any product,

(4) smoking of cigarettes vs. never smoked cigarettes,

(5) smoking of cigarettes only vs. never smoked cigarettes,

(6-10) as options 1-5 except “never smoked” is replaced by “never smoked or near equivalent”.

A variant analysis prefers cigarette smoking (by reordering these preference as 4, 5, 2, 3, 1, 9, 10, 7, 8, 6). In meta-analyses of type C (ever smoking), a further variant analysis reverses the preference so current smoking results are preferred to those for ever smoking. Other variant analyses restrict attention to specific subtypes of outcome (e.g. for COPD, whether the definition is based on mortality, on a basis of lung function criteria only, or on other definitions).

## The dose-related smoking indices

For the dose-related indices, meta-analyses were conducted for: E amount smoked, F age of starting to smoke, G pack-years, H duration of smoking, I duration of quitting compared to never smokers (or long-term ex smokers), and J duration of quitting compared to current smokers (or short-term quitters). For any measure of exposure, the data for a study typically consist of an RR for each of a set of dose-categories expressed relative to a common base. Within each set, the RRs are not independent, and to avoid “double-counting” only one RR was included in any one meta-analysis. Two approaches were adopted. The first involves defining a number of levels of exposure, then carrying out meta-analyses for each level in turn, based on the RR from each sex within study that compares that level of exposure with never smokers, if such an RR is available. The second approach involves conducting a meta-analysis of RRs for the highest compared with the lowest categories of exposure available for each study.

For the first approach, the levels were defined by a scheme of “key values”. A category was allocated to the level whose key value it included, providing it did not also include another key value. Thus categories which included no key value, or more than one, were excluded. For example, if key values for number smoked were defined as 5, 20 and 45 cigs/day, and a study provided RRs for 1-9, 10-19, 20-29, 30-39 and 40+ cigs/day relative to never smokers, the RR for 1-9 cigs/day would be allocated to level 1, that for 20-29 cigs/day would be allocated to level 2, and that for 40+ cigs/day to level 3, with the RRs for 10-19 and 30-39 cigs/day not being used; while if another study provided RRs for 1-19 and 20+, the RR for 1-19 would be allocated to level 1 with that for 20+ unused. Note that a scheme with a few key values, widely spaced, is likely to involve RRs from more studies, whereas a scheme with more key values, closely spaced, will involve RRs from fewer studies, but ones with dose categories more closely clustered around the key value. For most of the measures two schemes were used; for amount smoked and pack-years, one scheme had broader and the other closer spaced key values; for duration of quitting one scheme focused more on shorter-term and the other on longer-term quitting. The key values used (with 999 indicating an open-ended category) for amount smoked were 5, 20 & 45 and 1, 10, 20, 30, 40 & 999, for age of starting to smoke were 26, 18 & 14, for pack-years were 5, 20 & 45 and 1, 10, 20, 30 & 999, for duration of quitting vs. never were 12, 7 & 3 and 20, 12 & 3 and for duration of quitting vs. current were 3, 7 & 12 and 3, 12 & 20. No key value analysis was attempted for duration of smoking. Note also that though the second approach will generally include RRs from all studies, whereas the first approach will not, the “highest” and “lowest” categories being compared under the second approach may vary considerably between studies.

## The meta-analysis tables

For each meta-analysis, a full output is available in .rtf format, and a summary is also available in .xls format. The full output comes in eight Sections, and the summary tables include Section 3 and Section 6. Further detail is given below.

### *Numbering system for the tables*

Tables are numbered by a four part code such as Table 1 – C – 7 – 5.

The first part identifies the outcome (1 = COPD, 2 = CB, 3 = Emphysema).

The second part identifies the smoking index (A = ever smoking, B = current smoking, C = current smoking or ever if current not available, D = ex smoking, E = amount smoked, F = age started, G = pack-years, H = duration of smoking, I = duration of quitting (vs never), J = duration of quitting (vs current)).

The third part of the code identifies the analysis carried out. This varies by smoking index and is explained more fully in Table 4A for the major smoking indices and in Table 4B for the dose-related indices. For analyses of the major smoking indices Tables with a third code of 1 are the main analyses, with codes of 2 or greater indicating variant analyses, as described above. For analyses of the dose-related smoking indices, the codes discriminate analyses for different key values or for “highest” vs “lowest” comparisons.

The fourth part of the code identifies the section of output. Sections 1 to 3 relate to the “most-adjusted” analysis, and Sections 4 to 6 to the least-adjusted analysis, with Sections 7 and 8 providing further detail. The contents of each section are described below under “full tables”.

### *The full tables*

The full detailed output for the major smoking indices is shown in Additional files 4, 5 and 6, and for the dose-related indices in Additional files 7, 8 and 9. Each table comes in eight sections preceded by a cover page. All the pages for the meta-analysis have the same first three codes in the Table number and the same main heading (describing the analysis), with the section number blank for the cover page and 1 to 8 for the specific section. For sections 1 to 3, from each study, the RR(s) adjusted for the most potential confounders are chosen, referred to as the ‘most-adjusted analysis’. Note that some of the chosen RRs may be unadjusted (e.g if a study has only unadjusted RRs, or if none of the RRs for a particular study chosen by the earlier stages of the preferencing method are adjusted). Sections 4 to 6 concern ‘least-adjusted’ data, i.e. with RRs adjusted for the least potential confounders chosen from each study, recalling that, as described in the main paper age-adjusted data if available have already been selected in preference to unadjusted data for prospective studies.

The content of each section is as follows:

| Cover page : | This shows  (i) restrictions on the data included,  (ii) the order of preference for selecting RRs to be included, and  (iii) a short description of the contents of the table |
| --- | --- |
|  |  |
| Section 1 : | For all the most-adjusted RRs selected, a listing of their relevant characteristics. This includes the values of certain variables used to select the RR and used as ‘characteristics’ in Section 3, as well as the two key identifiers of the RR: the study 6-character reference (REF) and the number of the RR within that study (NRR). It also may indicate where RRs differ from those in another table – for example, where Table 1-A-1-1 presents analyses preferring smoking of any product and Table 1-A-2-1 an analysis preferring smoking of cigarettes, a character "x" in a column headed "Cmp1A1" in the output for Table 1-A-2-1 indicates those studies where the RRs in the two tables actually differ. Abbreviations used are shown in Table 5. |
|  |  |
| Section 2 : | For each most-adjusted RR selected, the output shows in the first part of the section the sex, the number of potential confounding variables adjusted for, the numbers of cases (and the numbers of controls for unadjusted RRs) where available, the RR with its 95% confidence interval (CI), and in the second part of the section Y_s_, W_s_, Q_s_ and P_s_ (as defined in *Notation* below). Where multiple independent RRs are available for a study (typically different sexes), combined results are also shown for the study. Note that the 2 × 2 table is headed “exposed/non-exposed” × “case/control”. Exposed and non-exposed are as defined in the cover page. Control will be numbers at risk or person-years for prospective studies or disease-free for cross-sectional studies. Unadjusted RRs calculated using the “correction for zero cell” method are indicated by a tilde (~). Section 2 ends with the results of a meta-analysis of the overall data, identical to that shown at the start of section 3 and described below. |
|  |  |
| Section 3 : | This gives the results of fixed-effect and random-effects meta-analyses of the most-adjusted data. For the overall data and for data subdivided by sex, and for data subdivided by levels of various other characteristics, the output indicates, for each level, the number of RRs combined (N), the number of studies from which these RRs come (NS), the combined weight for the studies combined (Wt) as well as the RRs and CLs limits themselves (RR, RRl, RRu) and coded P values testing for heterogeneity and for variation between levels of the characteristic. P values are coded as described in *Notation* below. For the first analysis, of the overall data not subdivided by levels of any characteristic, coded P values for Egger’s test of publication bias (Asymm P)[16] are also given. For the data subdivided by characteristic level, the tests for variation between factor levels are shown at the foot of the total column. Levels of the characteristics are as described in *Characteristics considered* below. |
|  |  |
| Sections 4 to 6 : | As for Sections 1 to 3 but for least-adjusted data. A column headed X indicates, in the section 4 output, by entries of x against specific RRs, those that differ from the corresponding most-adjusted RRs. Typically, an x will not be entered where a study only has one relevant RR available, adjusted or unadjusted but not both. |
|  |  |
| Section 7 : | This lists the studies excluded from consideration, together with information on the stage at which they were excluded. Stage 1 refers to studies partially entered on the database but rejected from all analyses. The other stages refers back to the various restriction and selection stages described in the cover page. A study is excluded when no RR can be found to satisfy the criteria required. |
|  |  |
| Section 8 : | This lists linked and potentially overlapping studies for which data have been included (about which more information is given in Table 2 of Additional File 2), and also any results which would have been included in preference except that they had incomplete data (typically a RR with no CI). |

Note that the main results are given in Sections 3 and 6, while Sections 1, 2, 4, 5, 7 and 8 mainly provide detailed information only required when one wants to see the individual RRs or to check the program is correctly selecting the data.

### *Notation*

The notation used in some of the output (particularly sections 2, 3, 5, 6) is the same, where relevant, as that used by Fleiss and Gross[13]. Thus, we have:

N the number of RRs being combined

NS the number of studies from which the RRs are taken, (except when the analysis is subdivided into levels of characteristics, NS in the Total column is the sum of the values in the individual columns, i.e. the number of study × characteristic levels from which the RRs are taken)

s the individual RR estimate being combined (s = 1, …N)

Y_s_ the logarithm of the RR estimate s

W_s_ the associated weight, calculated as the inverse of the variance of the logarithm of the RR

Wt the total weight for all the RRs being combined

Fixed RR the fixed-effect RR estimate, calculated by
exp ((∑ W_s_Y_s_)/( ∑ W_s_)) = exp ($\overline{Y}$) summation being over s = 1, … N

Fixed RRl the lower 95% confidence limit (CL) of the fixed-effect RR estimate, calculated by exp($\overline{Y}$-1.96/$\sqrt{\sum W_{s}}$)

Fixed RRu the upper 95% CL of the fixed-effect RR estimate, calculated by exp($\overline{Y}$+1.96/$\sqrt{\sum W_{s}}$)

Fixed P the probability value associated with the fixed-effect RR estimate, given in coded form as +++, --- p<0.001; ++, -- p<0.01; +, - p<0.05; (+), (-) p<0.1; N.S. (not significant) p>0.1. Plus signs indicate the RR is significantly greater than 1.0, minus signs that it is significantly less

Q_s_ the study’s contribution to the heterogeneity estimate, calculated by $W_{s}(Y_{s}-\overline{Y})^{2}$. Where N is large, this can be regarded approximately as a chisquared on 1 d.f.

P_s_ the associated probability value, used to indicate outliers, coded as for Fixed P

Het Chi (or Q in Fleiss and Gross notation) the heterogeneity chisquared on N-1 d.f., calculated by ∑ Q_s_. If Q < N-1, the random-effects and fixed-effect estimates are the same, but if Q > N-1 they differ.

Het df the degrees of freedom corresponding to Het Chi (= N-1)

(Note that Het Chi divided by Het df is referred to as H in the tables in the paper)

Het P the probability value associated with Het Chi and Het df, coded as for Fixed P

Random RR,

Random RRl,

Random RRu The random-effects RR estimate and its lower and upper 95% CLs. The method for deriving this, originally described by DerSimonian and Laird[321], is most conveniently given by Fleiss and Gross[13]

Random P the probability value associated with the random-effects RR estimate, coded as for Fixed P

Asymm P the probability value associated with Egger's test of publication bias,[16] coded as *** p<0.001; ** p<0.01; * p<0.05; (*) p<0.1; N.S. (not significant) p>0.1.. Only presented for analyses not subdivided by levels of different characteristics

Between Chi where the meta-analysis is subdivided by levels of a characteristic, this is the chisquared value for the difference between the fixed-effect RR estimates for the levels of the characteristics

Between df the degrees of freedom corresponding to Between Chi, equal to the number of levels of the characteristic minus 1

Between P the probability value associated with Between Chi and Between df, coded as for Fixed P

Btwn (F) P the coded probability value associated with the residual variation. This is an alternative test for the difference between characteristic levels, and may be the more appropriate test when there is substantial unexplained heterogeneity. The residual heterogeneity chisquared is calculated by subtracting the Between Chi value for the characteristic from the total Het Chi, and the residual df is calculated by subtracting the Between df from the total Het df. The ratio of the heterogeneity chisquared per degree of freedom for the characteristic to that for the residual is then tested according to the F distribution.

(Note that Btwn (F) P is referred to as P_B_ in the tables in the paper)

### *Characteristics considered*

The meta-analysis output in Sections 3 and 6 first gives overall results for all the RRs selected. Results of an analysis subdivided by the characteristic **sex** are then shown with RRs compared for combined sex results and those specifically for males and females (recalling that sexes-combined results are only entered on the database when equivalent sex-specific results are not available).

Further analyses may show results for the various characteristics described below. The most-adjusted analysis of the first table for each major smoking index gives results for the full list of characteristics, but the least-adjusted and variant analyses, and the analyses of dose-related indices, may include a shorter list of characteristics, or no characteristics.

**Continent** The levels are: NAmer (=North America); Europe; Asia; and oth/mult (=other or multi-continent).

**National cigarette tobacco type** The levels are blended (=Belgium, Brazil, Chile, Croatia, Czechoslovakia, Denmark, Estonia, Finland, France, Germany, Greece, Hong Kong, Iceland, Italy, Japan, Korea, Mexico, Netherlands, Norway, Poland, Serbia, Spain, Sweden, Switzerland, Thailand, Turkey, Uruguay, Venezuela), virginia (=Australia, Canada, India, Ireland, Nepal, New Zealand, Nigeria, South Africa, United Kingdom), mixed (=Taiwan, or multi-country studies from both the Virginia and Blended groups), and unknown (=China). This classification was based on data supplied by PMI on 2^nd^ September 2009, using a criteria of at least 75% market share and assuming that the cigarette type had not varied over time.

**Start year of study** The levels are: <1970; 1970-79; 1980-89;1990-1999, 2000+; and unknown.

**Publicat**i**on year** The levels are: <1980; 1980-1989, 1990-1999; and 2000+. This refers to the principal publication for the study.

**Study type** The levels are: CC (=Case-control, including nested CC); Pr (=Prospective); and CS (=Cross-sectional).

**Lowest age in RR** The levels are: <25/unlim (=under 25 or no lower limit); 25-39; 40+; and unknown.

**Highest age in RR** The levels are: <65; 65-74; 75-84; 85+/unlim (=85 or more or no upper limit); and unknown.

**Study weakness** The levels are yes; and no (see Table 2 footnote f in main paper, excluding those where the weakness refers only to the base for comparison for the major smoking indices)

**COPD/CB/Emp subtype** For COPD, the levels are: mort (=COPD mortality); LF (=COPD defined in terms of lung function criteria only); and other. For CB, the levels are: mort (=CB mortality); sympt (=CB defined in terms of symptoms only); and other. For emphysma, the levels are: mort (=emphysema mortality); and other. **Asthma analysis type (COPD/CB/Emp)** This refers to how the original study handled subjects with asthma. The first two levels for all outcomes are inc-irres (=asthmatics included, analysis irrespective of asthma status) and excl-all (=all subjects with asthma excluded). For COPD, the other levels are defn-incl (=the definition of the outcome includes asthma); and other (=studies where cases may have co-existing asthma but controls are asthma-free; studies where it is unclear if the definition includes asthma or not; and studies where subjects can have only one diagnosis and the controls but not the cases may have asthma). For CB, the other level is excl-cntr (=studies where cases may have co-existing asthma but controls are asthma-free). There are no other levels for emphysema.

**Bronchodilator/reversibility** The levels are no/unkn (=spirometry conducted without bronchodilator use, or unknown), and yes/revs (spirometry was postbronchodilator, or criteria involved reversibility). This characteristic was defined only for outcome COPD defined in terms of lung function only.

**Number of COPD/CB/Emp cases** This refers to the number of cases in the whole study, rather than that used to calculate the specific RR. The levels are: 1-50; 51-100; 101-200; 201+.

**Analysis type** The levels are: prevlnce (=prevalence); and onset.

**Smoking product** The levels are: any; cigs (=cigarettes irrespective of smoking of other products); cigsonly (=cigarettes only).

**Smoking status** For Tables for smoking index C only, the levels are: ever; and current.

**Unexposed group** The levels are nev any (=never smoked any product); nev cig (=never smoked cigarettes); nev+ any (=never smoked any product or smoked a low amount); and nev+ cig (=never smoked cigarettes or smoked a low amount). As there are usually very few RRs in the latter two levels, analysis is repeated with levels “nev any” combined with “nev+ any”, and with “nev cig” combined with “nev+ cig”.

**Smoking results reported in study (COPD/CB/Emp)** The levels are ever (=that the study reported ever smoking but not current for that outcome), current (=that the study reported current smoking but not ever), or both (=that the study reported both current and ever smoking). For ever smoking (A), only the first and third levels can occur, and similarly for current smoking (B), only the second and third are possible.

**Number of adjustment variables** This refers to the adjustment variables used in the specific RR included in the meta-analysis. The levels are: 0; 1; 2+.

**RR adjustment** This refers to the adjustment variables used for the RR. The variables considered, each with levels yes or no, are: sex (analysis here is restricted to combined sex RRs only); age; and any other variables.

**Derivation of RR/CI** The levels are: Orig/2×2 (=as given by original author, calculated from a 2×2 table (which may have been estimated from a % distribution), or adjusted calculation from a 2×2×ℓ table); and other (=other methods, including recalculation due to a discrepancy between a 2 × 2 table and an original RR/CI. See *Derivations of RRs* above.

### *The summary tables*

The summary tables are given in Additional file 10. They include only Sections 3 and 6 from the full tables. Each analysis is presented on a separate worksheet, which includes a copy of the cover sheet, and sections 3 and 6 from the full tables (as described above).

A sheet “OverallSumm” brings together the overall results (not subdivided by any characteristic), with most-adjusted and least-adjusted results displayed side-by-side. The reader can overtype the table numbers in the cells shaded green to bring together particular tables of interest. Similarly in the sheet “CharacteristicSumm”, the reader can choose a characteristic of interest, and bring together results from selected tables.

## Forest plots

For each RR included, referenced by the study REF and sex, the RR is shown as a rectangle, the area of which is proportional to its weight. The CI is indicated by a horizontal line. The RRs and CIs are plotted on a logarithmic scale so that the RR is centred in the CI. Where the lower or upper CL is outside the range shown, this is indicated by an arrow on the end of the line. Rarely, when the RR itself is greater than the range, its rectangle is shown to the right of the line so that its area is apparent, but its position is not true to the scale. Also shown are the values of each RR and CI and the weight as a percentage of the total. Results from the random-effects meta-analysis are shown at the bottom of the plot. The combined estimate is presented as a diamond with the width corresponding to the CI and the RR as the centre of the diamond.

## Funnel plots

The logarithm of the RR is plotted against its weight. A dotted vertical line corresponds to the fixed-effect RR estimate.

# Meta-regression

Meta-regression analyses were also carried out using the sets of RRs selected for the main meta-analyses for ever smoking and for current smoking. Full results are presented in Additional file 11. Due to the sparsity of the data for emphysema, and the fact that a large proportion of the weight came from a single study, the results for emphysema are given only in the Additional file, while the meta-regressions for COPD and CB are also included in the main paper The meta-regression output comes in six tables, Tables 1-A-6, 1-B-6, 2-A-6, 2-B-6, 3-A-6 and 3-B-6. As before, the first part of the code relates to outcome (1 = COPD, 2 = CB, 3 = Emphysema) and the second to smoking index (A = ever smoking, B = current smoking). The third part (= 6) distinguishes it from the values of 1 to 5 used for the other meta-analysis output. All the analyses are based on the most-adjusted RRs for smoking of any product (or cigarettes if all products not available), as used in the main meta-analysis. Thus, for example, the data used in Table 1-A-6 is that listed and analysed in sections 1 to 3 of Table 1-A-1.

For each table, the first page of output, labelled “Fixed model”, shows a basic model including those characteristics selected as likely to be relevant. These characteristics were selected based on general considerations, the univariate results from the meta-analyses, preliminary meta-regression analyses using alternative lists of characteristics (not shown), and the desire to avoid inclusion of highly correlated variables in the same model. The basic list of characteristics comprised sex, continent, outcome subtype, how asthma was taken into account, smoking product, unexposed base group, whether the RR was adjusted for age, whether the RR was adjusted for factors other than age or sex (all with levels as defined in *Characteristics considered*), and midpoint age (calculated as an average of the high and low values of the age range, treating <15 or no lower limit as 15 years).

This first page starts by giving the deviance and degrees of freedom (DF) of the model. This can be compared with the deviance (Heterogeneity chisquared) for the corresponding model in the main meta-analysis (e.g. for COPD ever smoking 1-A, compare 1038.04 on 128 DF with no characteristics included in the model [shown in Table 1 - A - 1 - 3] with 421.765 on 112 DF with all the characteristics included). For each level of each characteristic, the output then shows the estimates, standard errors and p-values; as in Tables 6 and 8 of the main paper. The p values are coded as +++, --- p<0.001, ++, -- p<0.01, +, - p<0.05, (+), (-) p<0.1, and N.S. (not significant) p≥0.1, with the signs indicating the direction of difference. The output also shows the RR and 95% CI estimated from the weighted least-squares means and SEs, equivalent to using the SAS OM (observed marginals) technique[322].

The next part of the output is headed “Test by removing variables one at a time”. Each characteristic in the basic list is removed in turn, indicated on the output by e.g. “Omitting continent”, with the output shown for the model omitting the given characteristic laid out similarly to before. The drop in deviance (Drop Dev) compared to the fixed model including all the basic characteristics is also shown, together with its p-value using an F-test, coded as above. Note that when omitting characteristics, the deviance increases, so that the drop shown is negative.

The next part of the output shows the results of tests for reducing smoking product from three to two levels (applies to all outcomes), or reducing outcome subtype from three to two levels (for COPD to lung function or other, and for CB to symptoms or other).

This is then followed by the results of tests for “adding extra variables one at a time”, using a list of secondary variables (national cigarette type, publication year, study type, presence of a study weakness, use of a bronchodilator, study size, which smoking results were available for the study, how the RR and CI were derived and analysis type). These generally have the same levels as defined earlier although where appropriate, to avoid small numbers of estimates, levels may be combined, e.g. mixed and unknown are combined for national cigarette tobacco type. The drop in deviance compared to the fixed model is that shown at the start of the output for each variable, with the fitted estimates for the introduced characteristic at the end.

Finally, fitted values and residuals are shown for the fixed model. The output shows, for each RR, the number of the RR on the relative risk database (preceded by #), the study REF (see Table 2 of the main paper), the number of the RR within the study (NRR), the sex (m, f or b = both), the logarithm of the RR (LOGRR), the fitted value (FITVAL), its standard error (SEFITV) and the standardized residual (STDRES).

# Other files showing dose-related data

In addition to the meta-analyses described above, dose-related data are also shown in two other files.

All the sets of categorical data eligible for the “key-value” meta-analyses are listed in the Excel file Additional File 12. For those sets of categorical data actually selected for the key value analyses (i.e. highest preference), the RRs are also plotted. Note that all the RRs from the set are plotted, whereas only some of the set may have been included in the key value analysis. The worksheet “Intro” in that file explains the layout of the data listings and the plots.

Dose-related data not eligible for either the “key-value” or the “highest vs lowest” meta-analyses are shown in Additional File 13.

# Additional analyses

For the three outcomes, and for ever smoking and current smoking, pairs of corresponding RR and CI estimates within the same study for males and for females, were identified and used to carry out meta-analyses of the male/female sex ratio. Pairs of corresponding RRs within the same study were also identified that were either least-adjusted or most-adjusted. Unlike the pairs of sex-specific results, these pairs were non-independent and the variance of their ratio could not readily be calculated. Instead the numbers of pairs where the ratio exceeded or did not exceed 1 were counted and compared by the sign test, and also meta-analyses were conducted separately for the least-adjusted and most-adjusted members of the pairs. Similar methods were also used to compare non-independent pairs of RRs for current smokers of cigarettes only and for current smokers of cigarettes irrespective of other products, each expressed relative to never smokers.

# Software

All data entry and most statistical analysis were carried out using ROELEE version 3.1 (available from P.N.Lee Statistics and Computing Ltd, 17 Cedar Road, Sutton, Surrey SM2 5DA, UK). Some additional analyses were carried out using Excel 2003.

# Table 1 Core and allied conditions for COPD, CB and emphysema in successive revisions of the ICD

| ICD revision | Code | Description | Status* |  |  |
| --- | --- | --- | --- | --- | --- |
|  |  |  | COPD | CB | Emp |
| 7^th^ | 241 | Asthma | Allied |  |  |
|  | 500 | Acute bronchitis | Allied | Allied |  |
|  | 501 | Bronchitis, unqualified | Allied | Allied |  |
|  | **502** | **Chronic bronchitis** | **Core** | **Core** |  |
|  | 526 | Bronchiectasis | Allied |  |  |
|  | **527.1** | **Emphysema without mention of bronchitis** | **Core** |  | **Core** |
|  | 527.2 | Other (diseases of lung and pleural cavity†) | Allied |  |  |
|  |  |  |  |  |  |
| 8^th^ | 466 | Acute bronchitis and bronchiolitis | Allied | Allied× |  |
|  | 490 | Bronchitis, unqualified | Allied | Allied× |  |
|  | **491** | **Chronic bronchitis** | **Core** | **Core** |  |
|  | **492** | **Emphysema** | **Core** |  | **Core** |
|  | 493 | Asthma | Allied |  |  |
|  | 518 | Bronchiectasis | Allied |  |  |
|  | 519‡ | Other diseases of respiratory system | Allied |  |  |
|  |  |  |  |  |  |
| 9^th^ | 416 | Chronic pulmonary heart disease | Allied |  |  |
|  | 466 | Acute bronchitis and bronchiolitis | Allied | Allied× |  |
|  | 490 | Bronchitis, not specified as acute or chronic | Allied | Allied× |  |
|  | **491** | **Chronic bronchitis** | **Core** | **Core** |  |
|  | **492** | **Emphysema** | **Core** |  | **Core** |
|  | 493 | Asthma | Allied |  |  |
|  | 494 | Bronchiectasis | Allied |  |  |
|  | 495 | Extrinsic allergic alveolitis | Allied |  |  |
|  | **496** | **Chronic airway obstruction, not elsewhere classified** | **Core^§^** |  |  |
|  | 519 | Other diseases of respiratory system | Allied |  |  |
|  |  |  |  |  |  |
| 10^th^ | I26 | Pulmonary embolism | Allied× |  |  |
|  | I27 | Other pulmonary heart diseases | Allied× |  |  |
|  | I28 | Other disease of pulmonary vessels | Allied× |  |  |
|  | J20 | Acute bronchitis | Allied× | Allied× |  |
|  | J40 | Bronchitis, not specified as acute or chronic | Allied | Allied× |  |
|  | **J41** | **Simple and mucopurulent chronic bronchitis** | **Core** | **Core** |  |
|  | **J42** | **Unspecified chronic bronchitis** | **Core** | **Core** |  |
|  | **J43** | **Emphysema** | **Core** |  | **Core** |
|  | **J44** | **Other COPD** | **Core** |  |  |
|  | J45 | Asthma | Allied× |  |  |
|  | J46 | Status asthmaticus | Allied× |  |  |
|  | J47 | Bronchiectasis | Allied× |  |  |

*, × Allied codes marked × were not in fact used in the selected outcome from any study.

† Code 527 also has 527.0 “Pulmonary collapse”

‡ In the ICD as originally published[323], only subcodes .0 “pulmonary collapse”, .1 “acute oedema of lung”, .2 “other disease of lung” and .9 “other” were defined. Subsequently (probably in 1976[324]) codes 519.7 “non-specific lung disease” and 519.8 “obstructive airway disease” were introduced. However in study MARCUS[202], code 519.3 is used, defined there as “chronic obstructive disease, not elsewhere classified”.

§ Exceptionally, study TVERDA was included despite lacking the core condition 496 in ICD9. The study follow-up period ran from 1972 to 1988 and the 9^th^ revision of the ICD was introduced in Norway in 1986. As code 496 only gradually came to be used for COPD, it is unlikely that omission of this code for the final 3 years of the study would have had much impact.

# Table 2 Definitions of COPD based on published criteria of lung function

| Criteria | Ref | Definition of categories | Comments |
| --- | --- | --- | --- |
| GOLD 2001 | [3] | |  |  | FEV1/FVC (%) | FEV1  (% pred) | Other | | --- | --- | --- | --- | --- | | 0 | At risk | ≥70% |  | Chronic symptoms* | | I | Mild | <70% | ≥ 80% | With or without chronic symptoms* | | II | Moderate | <70% | 30-79% | With or without chronic symptoms† | | III | Severe | (a) <70% | <30% |  | |  |  | or (b) |  | respiratory failure | |  |  | or (c) |  | clinical signs of right heart failure | | FEV1 is postbronchodilator.  * cough, sputum production  † cough, sputum production, dyspnea |
|  |  |  |  |
| GOLD 2006 | [4] | |  |  | FEV1/FVC (%) | FEV1 (% pred) | Other | | --- | --- | --- | --- | --- | | I | Mild | <70% | ≥ 80% |  | | II | Moderate | <70% | 50-79% |  | | III | Severe | <70% | 30-49% |  | | IV | Very severe | (a) <70% | <30% |  | |  | or (b) | <50% | and chronic respiratory failure | | FEV1 is postbronchodilator.  The main change from the earlier GOLD criteria is that grade 0 was eliminated and grade II split. |
|  |  |  |  |
| BTS | [5] | |  |  | FEV1/VC (%) | FEV1 (% pred) | | --- | --- | --- | --- | | I | Mild | <70% | 60-80% | | II | Moderate | <70% | 40-59% | | III | Severe | <70% | <40% | | Mild COPD is described as “presymptomatic within the community and usually unknown to the doctor”. Moderate COPD patients “usually have presented to their GP with intermittent chest problems and may be finding work difficult”. Severe COPD patients “are likely to have significant symptoms and to have intermittent admissions to hospital”. |
|  |  |  |  |
| ERS | [6] | |  |  | FEV1/VC  (%) | FEV1 (% pred) | | --- | --- | --- | --- | | I | Mild | <88% for men or <89% for women | ≥70% | | II | Moderate | <88% for men or <89% for women | 40-59% | | III | Severe | <88% for men or <89% for women | <40% | | The FEV_1_/FVC criterion is equivalent to >1.64 residual standard deviation (RSD) below the predicted value. Some authors restate the criteria slightly differently (e.g. Wilson *et al* [302] specify the limits as 88.2% and 89.3%, while Zieliñski and Bednarek [312] give 85%) |
|  |  |  |  |
| ATS | [7-9] | FEV1/FVC ratio below the normal range and :   |  |  | FEV1 (% pred) | | --- | --- | --- | | I | Mild | ≥50% | | II | Moderate | 35-49% | | III | Severe | <35% | | Although defining the normal range as a fixed % is not recommended, it is given as <70% by e.g. Wilson *et al*[302] and Mannino *et al*[193] |

# Table 3 Outcomes selected and alternatives not used for studies with multiple qualifying outcomes

## 3A COPD

| Ref | Type | Selected COPD | Alternative COPD |
| --- | --- | --- | --- |
| CLEMEN | Prev | Reduced FEV1 and/or VC (<70% predicted at least once and average below normal) | Severity of COPD: extra loss of FEV1 and/or VC |
| DOLL1 | Mort | COLD : ICD 7 or ICD 9 (underlying cause, codes unspecified but includes chronic bronchitis and emphysema) | Chronic bronchitis, emphysema and pulmonary heart disease (ICD7, codes unspecified) from [67] |
| EKBERG | Prev | COPD (GOLD criteria FEV1/FVC<70%) | Severity of COPD: GOLD 0 and GOLD II, III, IV |
| HUHTI3 | Prev | Chronic obstructive lung disease (FEV% < 60) | Chronic non-specific lung disease (chronic bronchitis [=chronic phlegm], chronic obstructive lung disease [as left] and/or asthma) |
| JOHANN | Prev | COPD (post-bronchodilator FEV1/FVC<0.7 =GOLD I-IV) (Phase 3 of the original study) | Incident cases of COPD (as left) during follow-up from an earlier study phase (Phase 2) [133] |
|  |  |  | Prevalence of airflow limitation (=GOLD stage II) from [132] (Phase 2) |
|  |  |  | Prevalence of COLD (=chronic cough; phlegm when coughing; breathlessness and/or wheezing; and FEV1/FVC <0.7) at Phase 2 from [134] |
|  |  |  | COPD defined from pre-bronchodilator from [131] |
| KAHN | Mort | Underlying COPD (ICD 7:501-502,527.1 from 1954-68; 501-502, 527.1, 527.2 from 1969-80) | Br/Emp/asthma (500-502,527.1,241) from [142];  COPD (501-502,527.1,527.2 later) (underlying or contributory) from [140];  Br/Emp (500-502,527.1) (some dose-related data entered for COPD) from [139] |
| KARAKA | Prev | COPD (ERS criteria=FEV1/VC<88% pred M <89% pred F ) and/or chronic bronchitis (chronic cough and chronic phlegm for 3 months for at least 2 years, and/or physician diagnosed CB, emphysema or COPD | self-reported history of COPD, chronic bronchitis, emphysema or respiratory symptoms (such as breathlessness, chronic cough and chronic phlegm for 3 months for at least 2 years) |
| KIM | Prev | COPD (FEV1/FVC <70%) | Physician-diagnosed CB, emphysema or COPD, ATS and ERS criteria for age 45+, BTS criteria for age 18+ from supplement;  also by GOLD stage |
| KOTAN2 | Prev | Post-bronchodilator FEV1/FVC <0.7 | Results also available using BTS criteria. Results reallocating COPD cases with concomitant physician-diagnosed asthma and <10 pack-years as non-cases were rejected |
| KULLER | Mort | COPD (ICD9 490-496) underlying cause of death | COPD (ICD9: 490-496) as contributory cause, or any mention |
| LANGE | Mort | COPD underlying or contributing mortality (490-2) | Incidence of GOLD-COPD at 5,15 or 25-year follow-up from [168,169] (with severity by GOLD stages).  Mortality (underlying) available but either unadjusted or with adjustment for FEV1, and so rejected (this being a prospective study) |
| LEBOWI | Prev | Predicted FEV1/FVC <80% | OAD (=physician-confirmed asthma, CB, emphysema LEBOWI1981);  FEV1 <74% predicted (LEBOWI1977C)  Also AOD grades 1 and 2 in [175] |
| LINDBE | Inc | COPD (GOLD modified no bronchodilator) | Severity of COPD: GOLD II or higher |
| LUNDB1 | Prev | COPD (GOLD postbronchodilator FEV1/FVC<70%) | BTS criteria, FEV1 <80% predicted |
| MADOR | Prev | COPD (ATS, FEV1 <70%) | Severity of COPD: FEV1 <35% |
| MANNI1 | Prev | Low lung function (FEV1/FVC <0.7 and FEV1 <80%) predicted (i.e. GOLD II) | Airflow obstruction (as left) but excluding subjects with restrictive lung disease (FEV1/FVC ≥ 0.7 and FVC <80% predicted) from base from [198]; |
|  |  |  | GOLD I COPD from [196] but excluding subjects who reported asthma, and restricted to age 30+ and attending at least 3 survey waves; |
|  |  |  | OLD (=self-report of diagnosis of current asthma or bronchitis, or ever emphysema) from [193]; |
|  |  |  | airflow limitation (FEV1/FVC <lower limit of normal LLN) subdivided into diagnosed, with asthma diagnosed or undiagnosed from [194] but limited to whites age 45+; |
|  |  |  | Severity of COPD : GOLD moderate or severe from [195] |
| MANNI2 | Prev | COPD (FEV1/FVC <0.7) | FEV1 <65% predicted from [200]  Severity of COPD : GOLD moderate or severe |
| MANNI3 | Mort | COPD (ICD 9: 490-496) as underlying cause | Any mention of COPD on death certificate |
| MUELLE | Prev | Chronic airway obstruction (FEV1/FVC < 60%) | Chronic non-specific lung disease (=CAO [as left], CB [phlegm from chest at least 3m each year] or asthma [physician diagnosed attacks of breathlessness with wheeze and FEV1/FVC>60%]) |
| NIHLEN | Inc | Self-report of physician diagnosis of Chronic bronchitis and/or emphysema and/or COPD | Prevalence of COPD by spirometry in small subset from [221] |
| NILSSO | Mort | Bronchitis and emphysema (ICD 8: 490-492) | Bronchitis, emphysema and asthma combined from [223] |
| PEAT | Prev | Chronic airflow limitation (FEV1/FVC <65% or FEV1 <65% predicted in at least two surveys. If age >75 CAL started before age 75 or also FEV1 <65% predicted) | CAL (as left)with CB symptoms |
| PELKON | Inc | Incident FEV1/FVC <70% persisting at all subsequent examinations, or COPD mortality (ICD codes not specified) | Results also available for COPD (as left) and CB (=cough with phlegm for at least 3m each year) combined. |
| RICCIO | Prev | Bronchial obstruction (GOLD criteria) | ATS and ERS criteria |
| SARGEA | Prev | Obstructive airways disease (physician-diagnosed bronchitis, emphysema or asthma, or FEV1 <80% predicted and FEV1/FVC<70%) | Self-reported OAD;  self-reported diagnosis of or medication for OAD (from [246])  [undiagnosed OAD also available as separate endpoint but not acceptable] |
| SHAHAB | Prev | ATS/ERS FEV1/FVC <0.7 | Severity of COPD: mild, moderate, sever/very severe |
| THUN | Mort | COPD (ICD 9: 490-492, 496) | Chronic bronchitis/emphysema as combined endpoint at baseline for pipe smokers only from [275] |
| TRUPIN | Prev | COPD (physician diagnosis of chronic bronchitis, emphysema or COPD) | COPD or emphysema (CB excluded) |
| TSUSHI | Prev | Post-beta2 stimulant FEV1/FVC <70% | Results also available for GOLD Stage 0+ |
| TVERDA | Mort | Asthma, bronchitis and emphysema (underlying, ICD 8: 466, 490-493; ICD 9: 466, 490-493) | Asthma, bronchitis and emphysema as underlying or contributory cause of death |
| VESTBO | Prev | COPD (GOLD stage 1+ FEV1/FVC <0.7) | Severity of COPD: GOLD stages 0, I, II, III |
| VIEGI2 | Prev | COPD ('clinical' criteria FEV1/FVC<70%) | ERS and ATS criteria |
| VONHER | Prev | Examining physician’s assessment of CB (cough and phlegm for at least 3 months for 2 consecutive years excluding other explanations) or emphysema, | COPD severity : results available with cases subdivided on Airway Obstruction FEV1/FVC >80% none, 70-79% mild, 50-69% moderate, <50% severe |
| WILSO1 | Prev | COPD (GOLD FEV1/FVC <70.0%) | ERS 1RSD, ERS, ATS, BTS |
| ZIELI1 | Prev | Bronchial obstruction (ERS: FEV1/FVC<85% pred) | Results also available excluding Restrictive or Mixed airflow pattern (i.e. FEV1/FVC>100% and FVC<80%, or 85%<FEV1/FVC<=99%, FVC<80% and FEV1>=70%) from the disease-free group;  Severity of COPD : ERS moderate (FEV1 50-69% of normal) or severe (FEV1 <50% of normal) |
| ZIELI2 | Prev | Airflow limitation (Gold: FEV1/FVC <0.7) | Results also available excluding Restricted airflow (i.e. FEV1/FVC>70% and FVC<80% in [316], or FEV1/ FVC>60% and FVC<80%N in [317]) from the disease-free group.  Severity of COPD : ERS moderate (FEV1 50-69% of normal) or severe (FEV1 <50% of normal |

## 3B CB

| Ref | Type | Selected CB | Alternative CB |
| --- | --- | --- | --- |
| COLLEG | Prev | Chronic bronchitis (diagnosed by physician using standard questionnaire) | Standard diagnosis of chronic bronchitis (morning phlegm in winter, cough and phlegm lasting 3 weeks during past 2 years, and breathlessness on the level) |
| DEANE | Prev | Chronic bronchitis (persistent cough and phlegm) | Persistent cough with phlegm with period of increased cough and phlegm in last 3 years. Persistent cough with phlegm with grade 2 shortness of breath |
| DEMARC | Prev | Chronic bronchitis (regular cough with phlegm for at least 3 months every year) | CB (cough and phlegm from the chest usually in winter and on most days for 3 months each year) and no self-reported diagnosis of asthma |
| FLETCH | Prev | Chronic bronchitis (production of phlegm on rising on most days for at least 3 months each year) | Production of phlegm all day on most days for at least 3 months each year |
| FOXMAN | Prev | Chronic bronchitis (phlegm most days 3 months in last year) | Restricted activities due to chronic bronchitis, bed days due to chronic bronchitis, chronic bronchitis and has ever visited physician for it |
| HAENSZ | Prev | Chronic bronchitis (termed MRC but symptoms unspecified) | Persistent cough and phlegm |
| HIGGI2 | Prev | Chronic bronchitis (persistent phlegm for at least 3 months of year + at least 1 chest illness with increased cough and sputum during past 3 years) | Persistent cough and sputum |
| HIGGI3 | Prev | Chronic bronchitis (persistent phlegm and 1+ bronchitic chest illness in past 3 years) | Persistent cough and sputum |
| HO | Prev | Chronic bronchitis (self-reported) | Coughing up phlegm for 3 consecutive months for 2 years from [113] |
| KOTAN1 | Prev | Chronic bronchitis (physician-diagnosed) | Adjusted chronic bronchitis (physician diagnosed chronic bronchitis or chronic productive cough but not physician diagnosed asthma) |
| LAMBER | Prev | Chronic bronchitis (cough and phlegm lasting 3+ months, breathlessness on walking and period of increased cough and phlegm lasting 3+ weeks in past 3 years) | Persistent cough and phlegm |
| LANGHA | Prev | Chronic bronchitis (cough and phlegm for 3+ months for the past 2 years) | Persistent cough with phlegm |
| LEBOWI | Prev | Chronic bronchitis (physician diagnosed) | chronic cough and phlegm. |
| LUNDB2 | Prev | Self-reported respiratory disease/symptoms confirmed at examination as chronic bronchitis (cough/sputum most days for at least 3 months for at least 2 years, or impaired lung function of obstructive type and FEV1 <80% predicted with history typical of chronic bronchitis) | With/without asthma; self-reported physician diagnosed CB (analysed as cross-sectional study, males only [188], or sexes combined without CI [189];  cough and phlegm (sexes combined without CI [189] |
| MEREN | Prev | Chronic productive cough (phlegm when coughing or phlegm on chest at least 3 months in 2 successive years) | Prevalence of physician-diagnosed and self-reported chronic bronchitis also given, but due to lack of information on numbers of subjects, no RRs can be calculated |
| MILLER | Prev | Chronic bronchitis (examining physician's impression of chronic bronchitis) | Chronic bronchitis defined as persistent sputum on most days for at least 3 consecutive months, previous diagnosis of chronic bronchitis |
| MILNE | Prev | Persistent cough and phlegm [MRC questionnaire] | Persistent cough and phlegm with chest illness with extra phlegm lasting 3 weeks or more in previous 3 years |
| OSWAL2 | Prev | Bronchitis (habitual cough and phlegm with disability due to exacerbations and/or breathlessness in previous 5 years) | Severity of disease (CB) briefly discussed in text |
| REID | Prev | Chronic bronchitis (persistent cough and phlegm (most days for at least 3 months each year), with shortness of breath while walking with other people at ordinary pace, and at least 1 period of increased cough and phlegm lasting 3+ weeks during previous 3 years) | Persistent cough and phlegm |
| STJERN | Prev | Chronic bronchitis (cough/phlegm most days for 3 months for at least 2 years) | Obstructive CB (CB and FEV1 <80% predicted) but only including occupationally exposed subjects |
| VIEGI1 | Prev | Chronic bronchitis (confirmed by physician) | Chronic cough and phlegm |

## 3C Emphysema

| Ref | Type | Selected emphysema | Alternative emphysema |
| --- | --- | --- | --- |
| ANDER2 | Prev | Emphysema (visual comparison with pre-set standard, Grade 1+)  [Grades are defined as 1-6, grade 4 corresponds to about 50% parenchymal destruction] | Severity (grade 4+, and grades 1-2, 3-4 and 5-6) and type (panlobular and centrilobular) of emphysema. |
| AUERBA | Prev | Emphysema (visual comparison against standard (Grade 1+))  [Grades are defined as 1-9, grade 1=barely discernible, grade 3=moderate, grade 6=advanced] | Severity of emphysema: (grades  1-1.75; 2-2.75;3-3.75; 4-4.75; 5-6.75; 7-9) |
| NAWA | Prev | Emphysematous changes (visual comparison) | Anatomical type of emphysema (localized, distal, proximal, mixed) |
| PRATT | Prev | Centrilobular emphysema (visual comparison with standard point-counting technique, including “trace” only) | Severity of emphysema (>25% centrilobular emphysema involvement) |
| SUTINE | Prev | Emphysema (air spaces >0.1 cm, grade 5+)  [Grades are defined as 0 to 100, grade 5=lower limit of mild, grade 30 = lower limit of moderate, grade 60=lower limit of severe] | Severity and anatomical type of emphysema (mild grades 5-20; moderate grades 30-50; severe grade 60+; centrilobular, panlobar, paracicatricial) |
| WANG2 | Prev | Emphysema (low-attenuation areas with disrupted vasculature but without discernible surrounding walls in the pulmonary parenchyma)  [Graded on four level scale – grade 1 involvement of 1-25% of the lung parenchyma; grade II 25-50%, grade III 50-75%, grade IV >75%] | Severity and anatomical type of emphysema (grades 1-4, upper lung, lower lung, diffuse, irregular) |

# Table 4 Meta-analysis tables

## 4A Main and variant analyses for major smoking indices

| Major smoking  index (code) | Smoking product preference |  | Table number | | |  |
| --- | --- | --- | --- | --- | --- | --- |
|  |  |  | COPD | CB | Emp | |
|  |  |  |  |  |  | |
| Ever smoking (A) | Any product^a,b^ |  | 1-A-1 | 2-A-1 | 3-A-1 | |
|  | Cigarettes^c^ |  | 1-A-2 | 2-A-2 | 3-A-2 | |
|  | Any product^b^ (based on mortality) |  | 1-A-3 | 2-A-3 |  | |
|  | Any product^b^ (based on lung function only) |  | 1-A-4 |  |  | |
|  | Any product^b^ (based on other than mortality/lung function) |  | 1-A-5 |  |  | |
|  | Any product^b^ (based on symptoms only) |  |  | 2-A-4 |  | |
|  | Any product^b^ (based on other than mortality/symptoms) |  |  | 2-A-5 |  | |
|  |  |  |  |  |  | |
| Current smoking (B) | Any product^a,b^ |  | 1-B-1 | 2-B-1 | 3-B-1 | |
|  | Cigarettes^c^ |  | 1-B-2 | 2-B-2 | 3-B-2 | |
|  | Any product^b^ (based on mortality) |  | 1-B-3 | 2-B-3 |  | |
|  | Any product^b^ (based on lung function only) |  | 1-B-4 |  |  | |
|  | Any product^b^ (based on other than mortality/lung function) |  | 1-B-5 |  |  | |
|  | Any product^b^ (based on symptoms only) |  |  | 2-B-4 |  | |
|  | Any product^b^ (based on other than mortality/symptoms) |  |  | 2-B-5 |  | |
|  |  |  |  |  |  | |
| Ever smoking (or current if ever not available) (C) | Any product^a,b^ |  | 1-C-1 | 2-C-1 | 3-C-1 | |
|  | Cigarettes^c^ |  | 1-C-2 | 2-C-2 | 3-C-2 | |
|  |  |  |  |  |  | |
| Current smoking (or ever if current not available) (C) | Any product^a,b^ |  | 1-C-6 | 2-C-6 | 3-C-6 | |
|  | Cigarettes^c^ |  | 1-C-7 | 2-C-7 | 3-C-7 | |
|  |  |  |  |  |  | |
| Ex smoking (D) | Any product^a.b^ |  | 1-D-1 | 2-D-1 | 3-D-1 | |
|  | Cigarettes^c^ |  | 1-D-2 | 2-D-2 | 3-D-2 | |
|  |  |  |  |  |  | |
| ^a^ Main analysis  ^b^ or cigarettes if all product not available, see *The major smoking indices* for detail  ^c^ or all product if cigarettes not available, see *The major smoking indices* for detail | | | | | | |

## 4B Analyses for dose-related indices

| Dose-related index (code) | Table type | Key value | Maximum range | Table number | | |
| --- | --- | --- | --- | --- | --- | --- |
|  |  |  |  | COPD | CB | Emp |
| Amount smoked (E) | Key value scheme 1 | 5 | 1-19 | 1-E-1 | 2-E-1 | 3-E-1 |
|  |  | 20 | 6-44 | 1-E-2 | 2-E-2 | 3-E-2 |
|  |  | 45 | 21+ | 1-E-3 | 2-E-3 | 3-E-3 |
|  | Key value scheme 2 | 1 | 1-9 | 1-E-4 | 2-E-4 |  |
|  |  | 10 | 2-19 | 1-E-5 | 2-E-5 |  |
|  |  | 20 | 11-29 | 1-E-6 | 2-E-6 |  |
|  |  | 30 | 21-39 | 1-E-7 | 2-E-7 |  |
|  |  | 40 | 31-98 | 1-E-8 | 2-E-8 |  |
|  |  | 999* | 41+ | 1-E-9 | 2-E-9 |  |
|  | Highest vs lowest |  |  | 1-E-10 | 2-E-10 | 3-E-10 |
| Age started (F) | Key value | 26 | 19+ | 1-F-1 |  |  |
|  |  | 18 | 15-25 | 1-F-2 |  |  |
|  |  | 14 | 1-17 | 1-F-3 |  |  |
|  | Youngest vs oldest |  |  | 1-F-4 | 2-F-4 | 3-F-4 |
| Pack-years (G) | Key value scheme 1 | 5 | 1-19 | 1-G-1 | 2-G-1 |  |
|  |  | 20 | 6-44 | 1-G-2 | 2-G-2 |  |
|  |  | 45 | 21+ | 1-G-3 | 2-G-3 |  |
|  | Key value scheme 2 | 1 | 1-9 | 1-G-4 |  |  |
|  |  | 10 | 2-19 | 1-G-5 |  |  |
|  |  | 20 | 11-29 | 1-G-6 |  |  |
|  |  | 30 | 21-39 | 1-G-7 |  |  |
|  |  | 999* | 41+ | 1-G-8 |  |  |
|  | Highest vs lowest |  |  | 1-G-9 | 2-G-9 | 3-G-9 |
| Duration of smoking (H) | Highest vs lowest |  |  | 1-H-1 | 2-H-1 | 3-H-1 |
| Duration of quitting (vs never) (I) | Key value scheme 1 | 12 | 8+ | 1-I-1 | 2-I-1 |  |
|  |  | 7 | 4-11 | 1-I-2 | 2-I-2 |  |
|  |  | 3 | 1-6 | 1-I-3 | 2-I-3 |  |
|  | Key value scheme 2 | 20 | 13+ | 1-I-4 | 2-I-4 |  |
|  |  | 12 | 4-19 | 1-I-5 | 2-I-5 |  |
|  |  | 3 | 1-11 | 1-I-6 | 2-I-6 |  |
|  | Most recent vs longest |  |  | 1-I-7 | 2-I-7 |  |
| Duration of quitting (vs current) (J) | Key value scheme 1 | 3 | 1-6 | 1-J-1 | 2-J-1 |  |
|  |  | 7 | 4-11 | 1-J-2 | 2-J-2 |  |
|  |  | 12 | 8+ | 1-J-3 | 2-J-3 |  |
|  | Key value scheme 2 | 3 | 1-11 | 1-J-4 | 2-J-4 |  |
|  |  | 12 | 4-19 | 1-J-5 | 2-J-5 |  |
|  |  | 20 | 13+ | 1-J-6 | 2-J-6 |  |
|  | Longest-term vs most recent |  |  | 1-J-7 | 2-J-7 |  |
| * 999 represents an open-ended category | | | | | | |

# Table 5 Abbreviations used in listings

| Variable name | Meaning | Level abbreviation^a^ | Level meaning |
| --- | --- | --- | --- |
| REF | study 6-character reference |  |  |
| NRR | RR number within study |  |  |
| Cmp | Comparison with another table (e.g. Cmp1A1 for comparison with Table 1-A-1) | x | The RR does not appear in the comparison table |
| X | In section 4 (least-adjusted analysis), comparison with most-adjusted analysis (section 1 of same table) | x | The RR does not appear in the most-adjusted analysis |
| SEX | Sex of the RR | m | Male |
|  |  | f | Female |
|  |  | b | Both |
| AGEL, AGEH | Lower and higher limits of age range of RR |  |  |
| REGION |  | Am:USA | USA |
|  |  | AM:Canada | Canada |
|  |  | Am:Sth/Cent | South and Central America |
|  |  | Eu:UK | UK |
|  |  | Eu:West | Western Europe |
|  |  | Eu:Scand | Scandinavia |
|  |  | Eu:SE/Balkn | SE Europe and the Balkans |
|  |  | Asia:MidE/S | Middle East and South Asia |
|  |  | Asia:SE/Pac | SE Asia and the Pacific |
|  |  | Asia:FarE | Far East Asia |
|  |  | Aus/NZ | Australia and New Zealand |
|  |  | Africa | Africa |
|  |  | Multi | More than one of the above regions |
| BEGYR | Start year of study | * | Unknown |
| PUBYR | Year of principal publication |  |  |
| STTYP | Study type | CC | case-control |
|  |  | nCC | nested case-control |
|  |  | Pr | Prospective |
|  |  | CS | cross-sectional |
| ONSET | Analysis type | Prev | Prevalence |
|  |  | Inc | Incidence |
| DISEAS | Outcome | COPD:mort | COPD mortality |
|  |  | COPD:ICD | COPD defined in terms of ICD codes (except if mortality) |
|  |  | COPD:LF | COPD based on lung function only |
|  |  | COPD:LF/symp | COPD based on lung function and symptoms |
|  |  | COPD/CB/EM | COPD based on diagnosis or medical record of COPD, CB and/or emphysema |
|  |  | CB/EM | COPD based on diagnosis or medical record of CB and/or emphysema |
|  |  | CB/EM/Ast | COPD based on diagnosis or medical record of CB, emphysema and/or asthma |
|  |  | COPD:oth | other definition of COPD |
|  |  | COPD undef | COPD not further defined |
|  |  | CB:mortality | CB mortality |
|  |  | CB:diagnosed | CB based on diagnosis or medical records |
|  |  | CB:self-rep | CB based on self-report (except if self-report of physician diagnosis) |
|  |  | CB:symptoms | CB based on symptoms |
|  |  | CB:other | other definition of CB |
|  |  | Emp:mort | emphysema mortality |
|  |  | Emp:diagnosd | emphysema based on diagnosis or medical records |
|  |  | Emp:self-rep | emphysema based on self-report (except if self-report of physician diagnosis) |
|  |  | Emp:other | other definition of emphysema |
| ADJ | Number of adjustment variables in RR |  |  |
| SMOKSTA | Smoking status of exposed group |  |  |
| PRODUCT | Smoking product of exposed group | Any | Smoked any product |
|  |  | Cigs | Smoked cigarettes (irrespective of cigarette type, and irrespective of whether also smoked other product) |
|  |  | Cigs only | Smoked cigarettes only |
|  |  | MCigs | Smoked manufactured cigarettes |
|  |  | MCigs only | Smoked manufactured cigarettes only |
| UNEXP | Definition of unexposed group | Nev any | Never smoked any product |
|  |  | Nev cigs | Never smoked cigarettes |
|  |  | N/L any | Never smoked any product or only smoked a low amount |
|  |  | N/L cigs | Never smoked cigarettes or only smoked a low amount of cigarettes |
|  |  | Cur any | Current smoker of any product |
|  |  | Cur cigs | Current smoker of cigarettes |
| BASE-HI^b^ | For “highest vs lowest” analysis, the upper limit of exposure in the “lowest” group (for age started this is shown as BASE-LO as higher age of starting corresponds to lower exposure)^c^ |  |  |
| LOW, HI | Range of exposure in the exposed group ^c^ | + | no upper limit |

^a^ Only abbreviated levels are shown, except that unabbreviated levels are also shown where necessary to define an “other” level.

^b^ For age started smoking and years quit (vs never) this is shown as BASE-LO as higher age of starting or more years quit correspond to lower exposure

^c^ units are in terms of the measure of exposure (e.g number of cigarettes for amount smoked, years of age for age start)
