# Supplementary material for: Systematic review with meta-analysis of the epidemiological evidence relating smoking to COPD, chronic bronchitis and emphysema
Source: BMC Pulm Med. 2011 Jun 14;11:36. doi: 10.1186/1471-2466-11-36 (PMC3128042; doi:10.1186/1471-2466-11-36)
Supplement: Additional file 2 — Studies. .DOC file concerning the 218 studies included on the database. This describes which studies provided data for which outcome and gives details of the overlapping and linked studies, as well as fuller distributions of study characteristics than those given in the paper and also details of study populations and exclusions. For each of the three outcomes, a study by study description of the full definition of the outcome and source of diagnostic information is given. [file 1471-2466-11-36-S2.DOC]

# Systematic review with meta-analysis of the epidemiological evidence relating smoking to COPD, chronic bronchitis and emphysema

Barbara A Forey, Alison J Thornton and Peter N Lee

**Additional File 2 : Studies**

**Contents**

[Systematic review with meta-analysis of the epidemiological evidence relating smoking to COPD, chronic bronchitis and emphysema 1](#__RefHeading___Toc293476810)

[Table 1 The 218 studies 4](#__RefHeading___Toc293476811)

[Table 2 Overlapping and linked studies 8](#__RefHeading___Toc293476812)

[Table 3 Study characteristics 11](#__RefHeading___Toc293476813)

[Table 3 (continued – COPD studies) 14](#__RefHeading___Toc293476814)

[Table 3 (continued – CB studies) 19](#__RefHeading___Toc293476815)

[Table 3 (continued – Emphysema studies) 23](#__RefHeading___Toc293476816)

[Table 4 Study populations and exclusions 26](#__RefHeading___Toc293476817)

[Table 5 Disease outcomes - summary 34](#__RefHeading___Toc293476818)

[Table 6 Disease outcomes – detailed 37](#__RefHeading___Toc293476819)

[Table 6A : COPD based on ICD 37](#__RefHeading___Toc293476820)

[Table 6B : COPD based on LF (lung function) only 38](#__RefHeading___Toc293476821)

[Table 6C : COPD based on LF and symptoms 40](#__RefHeading___Toc293476822)

[Table 6D : COPD not further defined 41](#__RefHeading___Toc293476823)

[Table 6E : COPD defined as COPD, CB or Emphysema 41](#__RefHeading___Toc293476824)

[Table 6F : COPD defined as CB or Emphysema 42](#__RefHeading___Toc293476825)

[Table 6G : COPD defined as CB, Emphysema or AST 42](#__RefHeading___Toc293476826)

[Table 6H : COPD other 43](#__RefHeading___Toc293476827)

[Table 6I : CB based on ICD 44](#__RefHeading___Toc293476828)

[Table 6J : CB based on diagnosis 44](#__RefHeading___Toc293476829)

[Table 6K : CB based on self-report 45](#__RefHeading___Toc293476830)

[Table 6L : CB based on symptoms 45](#__RefHeading___Toc293476831)

[Table 6M : CB other 48](#__RefHeading___Toc293476832)

[Table 6N : Emphysema based on ICD 49](#__RefHeading___Toc293476833)

[Table 6O : Emphysema based on visual comparison 50](#__RefHeading___Toc293476834)

[Table 6P : Emphysema based on diagnosis 50](#__RefHeading___Toc293476835)

[Table 6Q : Emphysema based on self-report 51](#__RefHeading___Toc293476836)

[Table 6R : Emphysema other 51](#__RefHeading___Toc293476837)

[Grading systems used throughout Table 6: 52](#__RefHeading___Toc293476838)

References – see main paper

## Table 1 The 218 studies

| **REF** | **Brief description of study** | **Original study name** | **Outcomes included** | | |
| --- | --- | --- | --- | --- | --- |
|  |  |  | **COPD** | **CB** | **EMP** |
| ALDERS | English hospital inpatient CC 1977-82 |  | 0 | x | 0 |
| ALESSA | Roman outpatients CC 1992-93 |  | x | 0 | 0 |
| AMIGO | Santiago clinic CC 2001-03 |  | x | 0 | 0 |
| ANDER1 | British Columbian (Chilliwack) CS 1963 | Chilliwack Respiratory Survey | x | x | 0 |
| ANDER2 | Florida autopsy CS study ca 1964? |  | 0 | 0 | x |
| ANDER3 | Southern Polish plasma sample CC ca 1997? |  | x | 0 | 0 |
| AUERBA | New Jersey/New York CS autopsy study 1963-70 | New Jersey Lung study | 0 | 0 | x |
| BANG | HHANES US hispanic CS 1982-84 | HHANES | 0 | x | 0 |
| BECK1 | Lebanon US baseline CS 1972 |  | 0 | x | 0 |
| BECK2 | Lebanon US follow-up PS 1972-78 |  | 0 | x | 0 |
| BEDNAR | Warsaw sleep apnea CS 2000-2002 |  | x | 0 | 0 |
| BEST | Canadian veteran PS 1955-62 | Canadian Veterans | x | x | x |
| BJORNS | Swedish ECRHS young adults 1990 | ECRHS, first stage | 0 | x | 0 |
| BROGGE | Bergen hospital+cohort CC 2003 |  | x | 0 | 0 |
| BROWN | Birmingham elderly men CS 1956 |  | 0 | x | 0 |
| CERVER | Italian ISAYA young adult CS 1998-2000 | ISAYA study | 0 | x | 0 |
| CHAPMA | Utah sulfur oxide exposed parents CS 1976 |  | 0 | x | 0 |
| CHEN1 | Shanghai factory PS 1972-1993 |  | x | 0 | 0 |
| CHEN2 | Canadian NPHS middle-aged CS 1994/5 | National Population Health Survey 1st cycle | x | 0 | 0 |
| CHEN3 | Canadian (CCHS) CS 2000-1 | Canadian Community Health Survey | x | 0 | 0 |
| CHENG | China (Beijing, Hubei, Liaoning) CS 1992 |  | x | 0 | 0 |
| CLEMEN | Belgian Airmen PS 1960-75 | Belgian Air Force study | x | 0 | 0 |
| COATES | Detroit middle-aged post office employees CS 1962 | Post office employees | 0 | x | 0 |
| COCCI | Italian (Pisa?) hospital patients CC ca 2000? |  | x | 0 | 0 |
| COLLEG | UK GP patients CS ca 1960? | College of General Practitioners Study | 0 | x | 0 |
| DEAN1 | Cleveland mortality risk factors CC 1969-1973 |  | x | 0 | 0 |
| DEAN2 | UK (GB) adults CS 1972 |  | 0 | x | 0 |
| DEANE | California telephone co employees CS 1963 |  | 0 | x | 0 |
| DEJONG | Kalamazoo COPD screening/intervention CS ca 2003? |  | x | 0 | 0 |
| DEMARC | Multi-country (ECRHS) young adults CS 1991-93 | ECRHS study, second stage | x | x | 0 |
| DETORR | Spain high-risk smoker screening CS 2001-03 |  | x | 0 | 0 |
| DICKIN | Barton elderly patients CS ca 1997? |  | x | 0 | 0 |
| DOLL1 | UK male doctors PS 1951-91 | British male doctors study | x | x | 0 |
| DOLL2 | UK female doctors PS 1951-73 | British female doctors study | x | x | 0 |
| DONTA1 | Cretan male villagers CS baseline 1960 | part of Seven Countries Study | x | 0 | 0 |
| DONTA2 | Cretan male villagers PS 1960-70 | part of Seven Countries Study | 0 | x | x |
| DOPICO | Minnesota/Wisconsin grain handlers CS ca 1982? |  | 0 | x | 0 |
| EHRLIC | S African household CS 1998 | South African Demographic and Health Survey | 0 | x | 0 |
| EKBERG | Malmo cardiovascular risk baseline 1974-1992 | Malmo Preventive Program | x | 0 | 0 |
| ENRIGH | US Cardiovascular Health elderly CS 1989-90 | Cardiovascular Health study | 0 | x | x |
| ENSTRO | Californian households (CPS I) PS 1960-98 | CPS I | x | 0 | 0 |
| FERRI1 | Berlin (US) air pollution CS 1961 | Berlin study | x | x | 0 |
| FERRI2 | Berlin (US) air pollution CS 1967 | Berlin study | x | 0 | 0 |
| FERRI3 | Berlin (US) air pollution CS 1973 | Berlin study | x | 0 | 0 |
| FIDAN | Izmir coffeehouse workers CS 2000-1 |  | x | 0 | 0 |
| FINKLE | Chicago military recruits CS 1969-70 |  | 0 | x | 0 |
| FLETCH | UK middle-aged post office workers CS 1956-7 |  | 0 | x | 0 |
| FORAST | Sonoma female occupational CS 1993-4 | SPPARCS study | x | 0 | 0 |
| FOXMAN | US HIE adults CS ca 1981? | Rand Health Insurance Experiment | 0 | x | 0 |
| FUKUCH | Japanese (NICE) CS 2000 | NICE study | x | 0 | 0 |
| GEIJER | IJsselstein PS 1998-2003 | IJsselstein cohort study | x | 0 | 0 |
| GODTFR | Copenhagen and Glostrup CCHS/GPS/CMS PS 1964-97 | Copenhagen City Heart/ Glostrup/ Copenhagen Male studies | x | 0 | 0 |
| GOLDBE | New York parents CS 1970 |  | 0 | x | 0 |
| GULSVI | Oslo two-phase CS 1972-74 |  | x | 0 | x |
| HAENSZ | Norwegian population/sibling adults CS 1964 |  | 0 | x | 0 |
| HAMMO2 | US households (CPS I) PS 1959-65 | CPS I | x | 0 | x |
| HARDIE | Bergen elderly CS 1998-99 |  | x | x | x |
| HARIKK | Baltimore long-term adults PS 1962-ca 2000? | Baltimore Longitudinal Study of Aging | x | 0 | 0 |
| HARRIS | Nigerian soldiers CS ca 1992? |  | 0 | x | 0 |
| HAWTHO | Paisley/Tiree/Renfrew male occupational PS 1965-77 |  | x | x | 0 |
| HAYES | US Rocky Mountains parents CS 1970 |  | 0 | x | 0 |
| HEDMAN | Southern Finland asthma/aspirin CS 1996 |  | x | 0 | 0 |
| HIGGI2 | UK Vale of Glamorgan adults CS 1956 | Vale of Glamorgan study | 0 | x | 0 |
| HIGGI3 | UK Annandale middle-aged adults CS 1956 | Annandale study | 0 | x | 0 |
| HIGGI4 | Tecumseh PS 1962-87 | Tecumseh Community Health Study | x | 0 | 0 |
| HIGGI6 | Tecumseh CS 1962-65 | Tecumseh Community Health Study | 0 | x | 0 |
| HIRAYA | Japanese adults PS 1965-82 | Six prefecture study | 0 | x | x |
| HO | Hong Kong elderly CS 1991 |  | x | x | x |
| HOLLA2 | US middle-aged male telephone employees CS 1962 |  | 0 | x | 0 |
| HOLLNA | Glostrup 40 year old CS 1976-7 | Glostrup Population Study | 0 | x | 0 |
| HOUSE | US sulfur oxide exposure parents CS 1970 | Salt Lake Basin study | 0 | x | 0 |
| HOZAWA | US 4 centre ARIC baseline CS 1987-89 | ARIC study | x | 0 | x |
| HRUBEC | US Veterans Twin Registry adults CS ca 1972? |  | 0 | x | 0 |
| HUCHON | French adults CS ca 2001? |  | 0 | x | 0 |
| HUHTI1 | Harjavalta middle-aged CS 1961 | Harjavalta study baseline | x | x | x |
| HUHTI2 | Harjavalta middle-aged CS 1971 | Harjavalta study follow-up | x | x | 0 |
| HUHTI3 | Hankasalmi rural men CS 1968-70 |  | x | x | 0 |
| ITABAS | Sendai elderly CC ca 1989? |  | x | 0 | 0 |
| JACOBS | Seven Countries middle-aged men PS 1957-89? | Seven Countries study | x | 0 | 0 |
| JAENDI | Toledo general practice CS 2001-02 |  | x | 0 | 0 |
| JENSEN | Danish bronchial reactivity adults CS ca 1996? |  | 0 | x | 0 |
| JINDA2 | Indian urban/rural adults CS ca 2004? |  | 0 | x | 0 |
| JOHANN | Hordaland community PS 1985-1997 | Hordaland County study | x | 0 | 0 |
| JOSHI | Indian male factory workers CS ca 1974? |  | 0 | x | 0 |
| JOUSI1 | North Karelia/Kuopio CS 1972 + 1977 | North Karelia/Kuopio study | 0 | x | 0 |
| KACHEL | Bielsko-Biala factory workers CS ca 2002? |  | x | 0 | 0 |
| KAHN | US Veterans PS 1954-80 | US Veterans - Dorn study | x | x | x |
| KAHN2 | US Veterans PS 1954-62 | US Veterans - Dorn Study | x | x | x |
| KARAKA | EPIC (Athens) air pollution NCC up to 1996 | EPIC study (nested case-control) | x | 0 | 0 |
| KATANC | Pittsbgh/Tennessee elderly periodontal CS 1997-98 | Health ABC study | x | 0 | 0 |
| KATO | Japanese baseline cancer CS 1985 |  | 0 | x | 0 |
| KHOURY | Baltimore familial component study (1970s) | Johns Hopkins familial component study | x | 0 | 0 |
| KIM | Korean NHANES II CS 2001-2 | Korean NHANES II | x | 0 | 0 |
| KIRAZ | Kayseri biomass fumes CS 1999 |  | x | x | 0 |
| KLAYTO | San Francisco MM research workers CS ca 1974? |  | x | 0 | 0 |
| KOJIMA | Aichi Health Centre CS 2001-02 |  | x | 0 | 0 |
| KOTAN1 | Finnish outdoor workers/skiers CS 1995-96 |  | 0 | x | 0 |
| KOTAN2 | North Finland clinical CS 1996-97 |  | x | 0 | 0 |
| KRZYZA | Polish (Cracow) population PS 1968-1981 | Cracow study | x | 0 | 0 |
| KUBIK | Kolin lung cancer adults CS 1972 |  | 0 | x | 0 |
| KULLER | US MRFIT all screenees 1972-74 with 6 yr follow-up | MRFIT study (all screenees) | x | 0 | 0 |
| LAI | Hong Kong CS 2001-2003 |  | x | 0 | 0 |
| LAM1 | Xi'an factory workers PS 1976-96 |  | x | 0 | 0 |
| LAM2 | Xi'an retired military men CS 1987 (baseline) |  | x | 0 | 0 |
| LAM3 | Xi'an retired military men PR 1987-2005(follow-up) |  | x | 0 | 0 |
| LAMBER | English/Welsh/Scottish adults CS 1965 |  | 0 | x | 0 |
| LANGE | Copenhagen PS 1976-78 to 1989 | Copenhagen City Heart Study (prospective) | x | 0 | 0 |
| LANGE2 | Danish elderly population CS 1991-4 | Copenhagen City Heart Study 3rd round | 0 | x | 0 |
| LANGHA | Norwegian HUNT adults CS 1995-97 | HUNT study | 0 | x | 0 |
| LAVECC | Italian National Health Survey CS 1983 | second National Health Survey | 0 | x | x |
| LEBOWI | Tucson white adults PS (baseline) 1972-3 | Tucson study | x | x | x |
| LEE | Great Britain migrants' siblings PS 1964 to 1977 | British sibling sample, USA-UK-Norway Migrant stdy | x | 0 | 0 |
| LIAW | Taiwan multicentre screening PS 1982-1993 |  | x | 0 | 0 |
| LINDBE | Norrbotten OLIN PS 1996-2003 | OLIN study | x | 0 | 0 |
| LINDST | Sweden/Finland FinEsS CS ca 1998? | FinEsS study | x | x | 0 |
| LIU1 | China retrospective mortality CC study 1986-91 | Million deaths study | x | 0 | 0 |
| LIU2 | Guangdong urban/rural CS 2002-03 |  | x | 0 | 0 |
| LUNDB1 | North Sweden OLIN CS 1996 | OLIN study | x | 0 | 0 |
| LUNDB2 | Swedish OLIN adults CC 1986 | OLIN Study 1986 | 0 | x | 0 |
| MADOR | New York veterans exercise CC (ca 2002?) |  | x | 0 | 0 |
| MAGNUS | Icelandic male CS 1993 |  | 0 | x | 0 |
| MANFRE | Canadian Rural Adults CS 1978-9 |  | 0 | x | 0 |
| MANNI1 | US (NHANES III) adults CS 1988-94 | NHANES III Survey | x | 0 | 0 |
| MANNI2 | US (NHANES I) adults CS 1971-75 | NHANES I Survey (baseline prevalence) | x | 0 | 0 |
| MANNI3 | US (NHANES I) adults PS 1971-92 | NHANES I Survey (22 years follow-up) | x | 0 | 0 |
| MARAN1 | Bangkok elderly baseline 1998 |  | x | 0 | 0 |
| MARAN2 | Bangkok elderly PS 1998-9 |  | x | 0 | 0 |
| MARCUS | Oahu (HHP) Japanese men PS 1965-84 | Honolulu Heart Program/Japan-Hawaii Cancer Study | x | 0 | 0 |
| MATHES | Melbourne genotyping CS (ca 2005?) |  | x | 0 | 0 |
| MELLST | Goteborg 70 year old men CS 1971-72 and 1976-77 | 70 Year Old People in Goteborg | 0 | x | 0 |
| MENEZ1 | Brazilian Urban Adults CS 1990 | Pelotas study | 0 | x | 0 |
| MENEZ2 | Sao Paulo PLATINO CS 2003 | PLATINO study | x | 0 | 0 |
| MENEZ3 | Santiago PLATINO CS 2003 | PLATINO study | x | 0 | 0 |
| MENEZ4 | Mexico City PLATINO CS 2003 | PLATINO study | x | 0 | 0 |
| MENEZ5 | Montevideo PLATINO CS 2003 | PLATINO study | x | 0 | 0 |
| MENEZ6 | Caracas PLATINO CS 2003 | PLATINO study | x | 0 | 0 |
| MEREN | Estonian postal CS 1995-6 |  | 0 | x | 0 |
| MILLER | Michigan PBB contaminants adults CS 1978 |  | 0 | x | x |
| MILNE | Edinburgh Elderly adults baseline CS 1968-70 | Edinburgh Elderly study: baseline | 0 | x | 0 |
| MOLLER | German adults CC ca 1999? |  | 0 | x | 0 |
| MONTNE | Malmo postal survey baseline 1992 |  | x | 0 | 0 |
| MUELLE | Glenwood Springs household CS 1967 |  | x | x | 0 |
| NAWA | Japanese workers/retired men CS 1998-2000 |  | 0 | 0 | x |
| NEJJAR | French elderly CS ca 1991? | PAQUID cohort | 0 | x | 0 |
| NIEPSU | Zabrze adults CS 2001 |  | x | 0 | 0 |
| NIHLEN | Malmo family history PS 1992-2000 |  | x | 0 | 0 |
| NILSSO | Swedish smoking habits PS 1963-1996 | 1963 smoking habit survey | x | 0 | 0 |
| OGILVI | Newcastle adults CC 1955-56 |  | 0 | x | 0 |
| OMORI | Japanese male screening CS ca 2004? |  | 0 | 0 | x |
| OSWAL1 | UK adult patients/civil servants CC 1951-3 |  | 0 | x | 0 |
| OSWAL2 | London adult civil servants CS 1954-5 |  | 0 | x | 0 |
| PANDEY | Nepalese rural adults CS 1979-80 |  | 0 | x | 0 |
| PEAT | Busselton LS 1966-84 | Busselton study | x | 0 | 0 |
| PELKON | Finland rural PS 1959-2000 | Seven Countries study (Finland centres) | x | x | 0 |
| PEREZP | Mexico City women CC 1992-4 |  | x | x | 0 |
| PETO | UK male mortality PS 1954-81 | Leigh/Staveley/Rhondda/Vale of Glamorgan/London studies (follow-up) | x | 0 | 0 |
| PRATT | US autopsy CS ca 1978? |  | 0 | 0 | x |
| PRICE | UK (Aberdeen) & USA (Denver) CS ca 2004? |  | x | 0 | 0 |
| REID | US migrants/native born CS 1962-3 |  | 0 | x | 0 |
| RENWIC | Manchester white adults CS 1992-4 |  | x | 0 | 0 |
| RICCIO | Chieti hospital CS 2002 |  | x | 0 | 0 |
| RIMING | UK radiography volunteers CS 1970 |  | 0 | x | 0 |
| RYDER | Welsh? autopsy CS ca 1969? |  | 0 | 0 | x |
| SARGEA | UK EPIC-Norfolk diet CS 1993-96 | EPIC study | x | 0 | 0 |
| SAWICK | Polish (Cracow) population CS baseline 1968 | Cracow study | x | x | 0 |
| SCHWAR | US national adults CS 1976-80 | NHANES II Survey | 0 | x | 0 |
| SHAHAB | England nationwide health CS 2001 | Health Survey for England | x | 0 | 0 |
| SHARP | Chicago electric company male workers 1960-1 | Western Electric Company study | 0 | x | 0 |
| SHIMUR | Japanese autopsy CC ca 1994? |  | 0 | x | 0 |
| SHIN | Ansan City residents CS 1999-2000 |  | x | 0 | 0 |
| SICHLE | Northern Greece CS 2000-01 |  | x | 0 | 0 |
| SILVA | Tucson white adults PS 1972-ca 1992? | Tucson study | x | x | x |
| SOBRAD | Spanish IBERPOC CS 1996-97 | IBERPOC study | 0 | x | 0 |
| SPEIZE | US 6 cities PS 1974-86 | Six cities study | x | 0 | 0 |
| STERLI | US (NMFS/NHIS) adult decedents CC 1986/87 | 1986 NMFS (National Mortality Followback Survey)/ 1987 NHIS (National Health Interview Survey) | x | 0 | 0 |
| STJERN | Swedish sulphite exposed adults CS 1981 |  | 0 | x | 0 |
| STROM | Malmo male risk factors CS 1982-3 | Men born in 1914 | x | 0 | 0 |
| SUADIC | Copenhagen middle-aged men CS 1985-6 | Copenhagen Male study | 0 | x | 0 |
| SUTINE | Finnish necropsy CS 1971-72 |  | 0 | 0 | x |
| TAGER | East Boston family (1 deg relatives) CS 1973-74 |  | x | 0 | 0 |
| TAGER2 | East Boston adults CS 1973-74 |  | 0 | x | 0 |
| TANG | Pooled analysis 4 UK PS 1967-82, max 19y follow-up | BUPA/Whitehall/Paisley-Renfrew/UKHD prevention | x | 0 | 0 |
| THUN | US (CPS II) household PS 1982-88 | CPS II | x | 0 | 0 |
| TODD | Great Britain PS 1965 followed to 1977 | British pop sample, USA-UK-Norway Migrant study | x | 0 | 0 |
| TROISI | US female nurses PS 1980-90 | Nurses' Health Study | 0 | x | 0 |
| TRUPIN | US telephone survey CS 2001 |  | x | 0 | 0 |
| TSUSHI | Nagano lung cancer screening CS 2003-2004 |  | x | 0 | 0 |
| TVERDA | Norwegian mortality PS 1972-88 |  | x | 0 | 0 |
| URRUTI | Spanish ECRHS young adults CS ca 2004? | ECRHS study (based on Spanish data only) | 0 | x | 0 |
| VESTBO | Copenhagen baseline 1976-78 | Copenhagen City Heart Study (baseline) | x | 0 | 0 |
| VIEGI1 | Northern Italian low pollution CS 1980-82 |  | 0 | x | 0 |
| VIEGI2 | Po delta CS 1988-91 |  | x | 0 | 0 |
| VIKGRE | Swedish elderly men PS 1994-2001 | Men born in 1933 in Goteborg - follow-up | 0 | 0 | x |
| VINEIS | EPIC multicentre PS 1993-ca 2003? | EPIC study | x | 0 | 0 |
| VOLLM1 | Portland screening clinic volunteers CS 1971-2 | Portland/screening centre cohort study - baseline | x | 0 | 0 |
| VOLLM2 | Portland screening clinic volunteers PS 1971-82 | Portland/screening centre cohort study - follow-up | x | 0 | 0 |
| VONHER | Finnish (MFHS) CS 1978-80 | Mini-Finland Health Survey | x | 0 | 0 |
| WAGEN2 | Dutch employees CC 2001 | Maastricht Cohort Study | 0 | x | 0 |
| WALD | London professional/businessmen PS 1975-93 | BUPA study | x | 0 | 0 |
| WANG2 | Japanese cancer screening CS 1996-98 |  | 0 | 0 | x |
| WATSON | Southampton GP CC study (ca 2000?) |  | x | 0 | 0 |
| WEISS | Philadelphia lung cancer project men CS 1961 | Philadelphia Pulmonary Neoplasm Research Project | x | 0 | x |
| WEN | Taiwan community & civil service PS 1982-2000 |  | x | x | x |
| WIG | Indian urban/rural adults CS ca 1963? |  | 0 | x | 0 |
| WILHEL | Swedish 54 year old men CS 1967 | Men born in 1913 in Goteborg | 0 | x | 0 |
| WILSO1 | Northwest Adelaide Health Study Baseline 2000 | Northwest Adelaide Health Study | x | 0 | 0 |
| WILSO2 | South Australian adults CS 1998 | South Australian Health Omnibus Survey | 0 | x | x |
| WOJTYN | Polish (Cracow) population CS 1968,1973 combined | Cracow study | x | x | 0 |
| WOODS | Melbourne and Riverina young adults CS ca 1998? |  | 0 | x | 0 |
| WOOLF | Canadian employed women CS ca 1970-1973? |  | 0 | x | 0 |
| XIAO | Beijing Hospital CC ca 2003? |  | x | 0 | 0 |
| XU | Nanjing urban/rural CS 2000-01 |  | x | 0 | 0 |
| YAMAGU | Beijing air pollution CS 1986 | Beijing Respiratory Health study | x | x | 0 |
| YUAN | Shanghai middle-aged men PS 1986-93 |  | x | 0 | 0 |
| ZIELI1 | Polish 12 cities screening CS 1999 | Know the Age of Your Lung Study 1 | x | 0 | 0 |
| ZIELI2 | Poland nationwide screening CS 2000-2003 | Know the Age of Your Lung Study 2 | x | 0 | 0 |
| ZIETKO | Bialystok nitric oxide CC ca 2003? |  | x | 0 | 0 |
| ZOIA | Lombardy dietary CS ca 1993? |  | 0 | x | 0 |

## Table 2 Overlapping and linked studies

| **N^a^** | **Type^b^** | **REF** | **REFGP^b^** | **Princ/Subsid^b^** | **COPD^c^** | **CB^c^** | **Emp^c^** | **Comment** |
| --- | --- | --- | --- | --- | --- | --- | --- | --- |
| 1 | B | AMIGO | AMIGO | Principal | i | - | - | Possible overlap between study AMIGO (2001-03 clinic cases of COPD with outpatient controls) and MENEZ3 (household survey 2003) both in Santiago |
| 1 | B | MENEZ3 | MENEZ3 | Principal | p | - | - |  |
| 2 | A | BECK1 | BECK1 | Principal | - | p | - | BECK1 is cross-sectional analysis of Lebanon survey at baseline, BECK2 is incidence analysis based on 6 years of follow-up in those disease-free at baseline |
| 2 | A | BECK2 | BECK2 | Principal | - | i | - |  |
| 3 | C | DEMARC | DEMARC | Principal | p | p | - | DEMARC is multinational study, treated as principal. URRUTI is Spanish sample only but gives data for cigarettes per day. Study DEMEER based on Dutch sample only but rejected as unexposed group unsatisfactory. |
| 3 | C | URRUTI | DEMARC | Subsidiary | - | p | - |  |
| 4 | B | EKBERG | EKBERG | Principal | p | - | - | Study EKBERG includes persons attending screening in Malmo and born approx 1913-65, so may overlap with NIHLEN (random sample of persons living in Malmo area and born approx 1933-72) and with STROM (50% of all men living in Malmo and born 1914) |
| 4 | A/B | MONTNE | MONTNE | Principal | p | - | - | Study MONTNE includes a random sample of persons living in Malmo area and born approx 1933-72, so may overlap with EKBERG (persons attending screening in Malmo and born approx 1913-65). Disease-free subjects from this study were followed up in study NIHLEN |
| 4 | A/B | NIHLEN | NIHLEN | Principal | i | - | - | Study NIHLEN includes a random sample of persons living in Malmo area and born approx 1933-72, so may overlap with EKBERG (persons attending screening in Malmo and born approx 1913-65). NIHLEN is a follow-up of the disease-free subjects from study MONTNE. |
| 4 | B | STROM | STROM | Principal | p | - | - | Study STROM includes 50% of all men living in Malmo and born 1914, so may overlap with EKBERG (persons attending screening in Malmo and born approx 1913-65) |
| 5 | B | ENRIGH | ENRIGH | Principal | - | p | p | HOZAWA was conducted in 1987-89 in four communities, including Washington county MD and Forsyth County NC. ENRIGH also included those two counties but as it started in 1989 and included only age 65+ any overlap will be minimal. HARIKK was conducted in Washington/ Baltimore MD and continuously recruited subjects since 1958 |
| 5 | B | HARIKK | HARIKK | Principal | i | - | - |  |
| 5 | B | HOZAWA | HOZAWA | Principal | p | - | p |  |
| 6 | C | FERRI2 | FERRIS | Principal | p | - | - | Study FERRI1 consists of original sample surveyed in 1961. Study FERRI2 is follow-up survey in 1967 which also included newly recruited second sample. Study FERRI3 is further follow-up cross-sectional survey in 1973. Study FERRI2 chosen as principal because based on largest sample. |
| 6 | C | FERRI1 | FERRIS | Subsidiary | p | p | - |  |
| 6 | C | FERRI3 | FERRIS | Subsidiary | p | - | - |  |
| 7 | C | GODTFR | GODTFR | Principal | i | - | - | Study GODTFR is pooled analysis of 3 prospective studies, with outcome COPD hospitalization and excluding subjects with previous hospitalization. Study VESTBO is baseline prevalence of GOLD-COPD in the Copenhagen City Heart Study, SUADIC is cross-sectional analysis of MRC-CB at 15-year follow-up in the Copenhagen Male Study, and HOLLNA is baseline prevalence of CB in the Glostrup Study. All have been marked as principal although some overlap with GODTFR is likely. Study LANGE is follow-up to COPD-related mortality from the CCHS - this is marked as subsidiary to GODTFR, and some overlap with VESTBO is likely. LANGE2 is a cross-sectional analysis of CB at 15-year follow-up in the CCHS - this is marked as subsidiary to VESTBO, and some overlap with GODTFR is likely |
| 7 | A/C | LANGE | GODTFR | Subsidiary | m | - | - |  |
| 7 | A/C | HOLLNA | HOLLNA | Principal | - | p | - |  |
| 7 | A/C | SUADIC | SUADIC | Principal | - | p | - |  |
| 7 | A/C | VESTBO | VESTBO | Principal | p | - | - |  |
| 7 | A/C | LANGE2 | VESTBO | Subsidiary | - | p | - |  |
| 8 | C | HAMMO2 | HAMMO2 | Principal | m | - | m | HAMMO2 is based in 25 states, ENSTRO is based on subset of subjects in one state only but follow-up is longer |
| 8 | C | ENSTRO | HAMMO2 | Subsidiary | m | - | - |  |
| 9 | B | HARDIE | HARDIE | Principal | p | p | p | Half of subjects in study BROGGE were drawn from survivors of study JOHANN (ongoing community cohort study up to age 82 in county Hordaland incl Bergen since 1985) and the remainder from hospital records in Bergen. Also possible overlap with study HARDIE (conducted in Bergen in 1998-9 age 70+) |
| 9 | B/C | JOHANN | JOHANN | Principal | p | - | - |  |
| 9 | B/C | BROGGE | JOHANN | Subsidiary | p | - | - |  |
| 10 | C | HIGGI4 | HIGGI4 | Principal | m | - | - | HIGGI6 is a baseline prevalence study, with follow-up mortality in all subjects entered as HIGGI4. Prevalence at an interim follow-up with additional community sample (METZNE1983) has been mentioned as alternative outcome in HIGGI6 |
| 10 | C | HIGGI6 | HIGGI4 | Subsidiary | - | p | - |  |
| 11 | C | HOUSE | HOUSE | Principal | - | p | - | HOUSE is baseline (1970) cross-sectional analysis of Salt Lake Basin communities, study CHAPMA presents results of 1976 survey in same communities. HOUSE includes higher number of subjects, so is treated as principal study. |
| 11 | C | CHAPMA | HOUSE | Subsidiary | - | p | - | . |
| 12 | C | HUHTI1 | HUHTI1 | Principal | p | p | p | Studies HUHTI1 and HUHTI2 are cross-sectional surveys conducted on same subjects 10 years apart |
| 12 | C | HUHTI2 | HUHTI1 | Subsidiary | p | p | - |  |
| 13 | C | JACOBS | JACOBS | Principal | m | - | - | JACOBS is multinational study. Subjects from one centre (Crete/Greece) are also included in studies DONTA1 (baseline prevalence of COPD) and DONTA2 (10-year incidenc), and subjects from West and East Finland centres are included in study PELKON |
| 13 | C | DONTA1 | JACOBS | Subsidiary | p | - | - |  |
| 13 | C | DONTA2 | JACOBS | Subsidiary | - | i | i |  |
| 13 | C | PELKON | JACOBS | Subsidiary | i | i | - |  |
| 14 | C | KAHN | KAHN | Principal | m | m | m | Study KAHN is based on 26 years follow-up but gives minimal results for CB and EM. Study KAHN2 is based on only 8.5 years follow-up but gives detailed results for CB and EM |
| 14 | C | KAHN2 | KAHN | Subsidiary | m | m | m |  |
| 15 | C | KOTAN1 | KOTAN1 | Principal | - | p | - | Study KOTAN1 was a postal cross-sectional survey. Study KOTAN2 comprises a random subsample from part of the study area invited to clinical study |
| 15 | C | KOTAN2 | KOTAN1 | Subsidiary | p | - | - |  |
| 16 | A | KRZYZA | KRZYZA | Principal | i | - | - | Study SAWICK is baseline analysis using outcomes FEV1/FVC <60% and MRC-CB. Follow-up study KRZYZA excludes subjects with baseline FEV1/FVC <70% (so no overlap with SAWICK) but analyses outcome FEV1/FVC <65% at 13 years. Results entered as study WOJTYN defines outcomes as FEV1/FVC<70% and MRC-CB both at baseline and at 5-year follow-up, and is marked as subsidiary study |
| 16 | A/C | SAWICK | SAWICK | Principal | p | p | - |  |
| 16 | A/C | WOJTYN | SAWICK | Subsidiary | p | p | - |  |
| 17 | A | LAM2 | LAM2 | Principal | p | - | - | Study LAM3 is prospective follow-up of disease-free subjects from LAM2 |
| 17 | A | LAM3 | LAM3 | Principal | m | - | - |  |
| 18 | B | LEBOWI | LEBOWI | Principal | p | p | p | Study LEBOWI is baseline study of subjects who were prospectively followed-up in study SILVA. As the outcomes studied are not identical, some cases may be included in both studies. Results from paper LEBOWI1977 omitted due to considerable inconsistencies in data presented |
| 18 | B | SILVA | SILVA | Principal | i | i | i |  |
| 19 | A | LINDBE | LINDBE | Principal | i | - | - | Study LUNDB2 was the first wave (1986) of the OLIN study, analysed as CC with a subset of symptom-free subjects as controls for CB cases. The full cohort but males only was also analysed cross-sectionally (data not entered but mentioned as alternative CB outcome in LUNDB2), and also served as a control group in a study of miners (study HEDLUN, subsequently rejected). A subset of the original full cohort was contacted again in 1996 and analysed for both prevalent COPD (study LUNDB1) and subsequent incidence (study LINDBE). LUNDB2 is marked as subsidiary to LUNDB1 |
| 19 | A/C | LUNDB1 | LUNDBA | Principal | p | - | - |  |
| 19 | A/C | LUNDB2 | LUNDBA | Subsidiary | - | p | - |  |
| 20 | A | MANNI2 | MANNI2 | Principal | p | - | - | Study MANNI2 is baseline prevalence, MANNI3 is follow-up of COPD-free subjects |
| 20 | A | MANNI3 | MANNI3 | Principal | m | - | - |  |
| 21 | A | MARAN1 | MARAN1 | Principal | p | - | - | Study MARAN1 includes prevalence at baseline and study MARAN2 includes incidence in 1 year follow-up of those disease-free at baseline |
| 21 | A | MARAN2 | MARAN2 | Principal | i | - | - |  |
| 22 | B | OSWAL2 | OSWAL2 | Principal | - | p | - | 36% of cases in CC study OSWAL1 were civil servants, and some overlap with OSWAL2, a cross-sectional study in civil servants, cannot be ruled out |
| 22 | B | OSWAL1 | OSWALD | Principal | - | p | - |  |
| 23 | C | PETO | PETO | Principal | m | - | - | Study PETO includes 5 samples, one of which is also reported as a subsidiary study (HIGGI2) |
| 23 | C | HIGGI2 | PETO | Subsidiary | - | p | - |  |
| 24 | A | TAGER | TAGER | Principal | p | - | - | Study TAGER refers to 1st degree relatives and is based on a random sample of subjects aged 45-54 years while study TAGER2 refers to cluster sample of households and includes subjects aged 5+ years |
| 24 | A | TAGER2 | TAGER2 | Principal | - | p | - |  |
| 25 | C | TANG | TANG | Principal | m | - | - | Study TANG is marked as principal study and is a pooled analysis of 4 samples, 2 of which are also entered as subsidiaries. Study HAWTHO includes 3 samples one of which (survey 3) was drawn from Paisley in 1974-5 thus also included in TANG (Renfrew and Paisley 1972-76). Study WALD (the BUPA study) is also included in the TANG analysis. |
| 25 | C | HAWTHO | TANG | Subsidiary | m | p | - |  |
| 25 | C | WALD | TANG | Subsidiary | m | - | - |  |
| 26 | C | TODD | TODD | Principal | m | - | - | Study LAMBER is baseline study of prevalent CB. Study TODD is 12-year follow-up of CNSLD mortality (not excluding subjects with baseline CB) |
| 26 | C | LAMBER | TODD | Subsidiary | - | p | - |  |
| 27 | C | VINEIS | VINEIS | Principal | m | - | - | VINEIS is the multi-national EPIC study, conducted in 10 countries and is marked as principal. SARGEA (UK) and KARAKA (Greece) were analyses using the data from one country only and are marked as subsidiary |
| 27 | C | KARAKA | VINEIS | Subsidiary | p | - | - |  |
| 27 | C | SARGEA | VINEIS | Subsidiary | p | - | - |  |
| 28 | C | VOLLM2 | VOLLME | Principal | m | - | - | Study VOLLM1 is baseline, and VOLLM2 is prospective follow-up of all subjects |
| 28 | C | VOLLM1 | VOLLME | Subsidiary | p | - | - |  |
| 29 | C | WEN | WEN | Principal | m | m | m | Study WEN includes two cohorts (community and civil service/teachers) followed until 2000, with results available only for males. Study LIAW reports both sexes for the community cohort only, with follow-up to 1993, and is marked as subsidiary |
| 29 | C | LIAW | WEN | Subsidiary | m | - | - |  |
| 30 | B | WILSO1 | WILSO1 | Principal | p | - | - | WILSO1 was conducted in northwest Adelaide among persons age 18+ in 2000, WILSO2 in metropolitan Adelaide and country towns among persons age 15+ in 1998, so some overlap cannot be ruled out |
| 30 | B | WILSO2 | WILSO2 | Principal | - | p | p |  |

^a^ N identifies linked studies (the numbering is based on alphabetical order of the first principal study in each set).

^b^ If the studies are independent (Type A), or the overlap between studies is deemed minor (Type B), then each study is marked as principal and has a separate value of REFGP. Where the overlap is deemed major (Type C), the studies share a common value of REFGP, and only one study within REFGP is marked as principal. The principal study is chosen on the basis of factors such as largest study size, widest geographical coverage or longest follow-up, and not on assessment of study quality. A/B indicates a type A link with at least one study in the set and a type B with another, and similarly for A/C and B/C – see Comment for details.

^c^ Availability of each outcome is indicated by m=mortality, i=incidence and p=prevalence

## Table 3 Study characteristics

| **All studies^a^** |  | **Study Type^b^** | | | | |
| --- | --- | --- | --- | --- | --- | --- |
| Variable^c^ | Levels^c^ | **CC** | **Prosp** | **CrossSec** | **Subsid** | **Total** |
|  |  |  |  |  |  |  |
| **Total** |  | 20 | 39 | 134 | 25 | 218 |
|  |  |  |  |  |  |  |
| **Study type** | **case/control** | 20 | 0 | 0 | 2 | 22 |
|  | **prospective** | 0 | 39 | 0 | 8 | 47 |
|  | **cross-sectional** | 0 | 0 | 134 | 14 | 148 |
|  | **nested case/control** | 0 | 0 | 0 | 1 | 1 |
|  |  |  |  |  |  |  |
| **Study sex** | **both** | 17 | 20 | 108 | 20 | 165 |
|  | **male** | 2 | 17 | 23 | 5 | 47 |
|  | **female** | 1 | 2 | 3 | 0 | 6 |
|  |  |  |  |  |  |  |
| **Lowest age in study** | **no limit** | 4 | 0 | 5 | 0 | 9 |
|  | **<15** | 0 | 1 | 4 | 0 | 5 |
|  | **15 or adult NOS** | 1 | 0 | 21 | 2 | 24 |
|  | **16-19** | 0 | 3 | 12 | 1 | 16 |
|  | **20-29** | 3 | 10 | 32 | 9 | 54 |
|  | **30-39** | 6 | 15 | 11 | 6 | 38 |
|  | **40-49** | 5 | 7 | 29 | 5 | 46 |
|  | **50-59** | 0 | 0 | 8 | 1 | 9 |
|  | **60-69** | 1 | 3 | 8 | 1 | 13 |
|  | **70+** | 0 | 0 | 4 | 0 | 4 |
|  |  |  |  |  |  |  |
| **Highest age in study (at baseline for prospective study)** | **unknown** | 0 | 0 | 1 | 0 | 1 |
|  | **<50** | 0 | 2 | 6 | 1 | 9 |
|  | **50-59** | 0 | 4 | 8 | 1 | 13 |
|  | **60-69** | 2 | 9 | 29 | 5 | 45 |
|  | **70-79** | 4 | 6 | 19 | 8 | 37 |
|  | **80-89** | 2 | 1 | 5 | 2 | 10 |
|  | **90-98** | 0 | 2 | 4 | 1 | 7 |
|  | **no limit** | 12 | 15 | 62 | 7 | 96 |
|  |  |  |  |  |  |  |
| **Highest age in study at final follow-up (prospective study)** | **60-69** | - | 6 | - | 0 | 6 |
|  | **70-79** | - | 2 | - | 2 | 4 |
|  | **80-89** | - | 9 | - | 3 | 12 |
|  | **90-98** | - | 3 | - | 0 | 3 |
|  | **no limit** | - | 19 | - | 4 | 23 |
|  |  |  |  |  |  |  |
| **Region** | **USA** | 2 | 13 | 35 | 7 | 57 |
|  | **Canada** | 0 | 1 | 5 | 0 | 6 |
|  | **S/C America** | 2 | 0 | 6 | 0 | 8 |
|  | **UK** | 5 | 6 | 12 | 5 | 28 |
|  | **Western Europe** | 4 | 2 | 11 | 1 | 18 |
|  | **Scandinavia** | 0 | 6 | 26 | 7 | 39 |
|  | **E Europe** | 2 | 1 | 8 | 1 | 12 |
|  | **SE Europe/Balkans** | 0 | 0 | 3 | 3 | 6 |
|  | **Middle East/S Asia** | 0 | 0 | 4 | 0 | 4 |
|  | **SE Asia/Pacific** | 0 | 1 | 1 | 0 | 2 |
|  | **Far East** | 4 | 6 | 16 | 1 | 27 |
|  | **Australia/NZ** | 1 | 1 | 3 | 0 | 5 |
|  | **Africa** | 0 | 0 | 2 | 0 | 2 |
|  | **multi** | 0 | 2 | 2 | 0 | 4 |
|  |  |  |  |  |  |  |
| **National cigarette tobacco type** | **Blended** | 12 | 25 | 100 | 19 | 156 |
|  | **Virginia** | 6 | 8 | 26 | 5 | 45 |
|  | **Mixed** | 0 | 2 | 2 | 1 | 5 |
|  | **Unknown** | 2 | 4 | 6 | 0 | 12 |
|  |  |  |  |  |  |  |
|  |  |  |  |  |  |  |
| **Start year of study** | **unknown** | 10 | 0 | 24 | 2 | 36 |
|  | **<1960** | 2 | 7 | 4 | 3 | 16 |
|  | **1960-69** | 1 | 12 | 17 | 8 | 38 |
|  | **1970-79** | 1 | 9 | 23 | 6 | 39 |
|  | **1980-89** | 2 | 5 | 13 | 2 | 22 |
|  | **1990-99** | 2 | 6 | 29 | 3 | 40 |
|  | **>1999** | 2 | 0 | 24 | 1 | 27 |
|  |  |  |  |  |  |  |
| **End year of study (of baseline for prospective study)** | **unknown** | 10 | 3 | 24 | 1 | 38 |
|  | **<1960** | 2 | 4 | 4 | 3 | 13 |
|  | **1960-69** | 0 | 9 | 13 | 6 | 28 |
|  | **1970-79** | 1 | 10 | 23 | 7 | 41 |
|  | **1980-89** | 2 | 5 | 13 | 3 | 23 |
|  | **1990-99** | 3 | 8 | 30 | 4 | 45 |
|  | **>1999** | 2 | 0 | 27 | 1 | 30 |
|  |  |  |  |  |  |  |
| **Final follow up year (prospective study)** | **unknown** | - | 5 | - | 0 | 5 |
|  | **1960-69** | - | 2 | - | 1 | 3 |
|  | **1970-79** | - | 5 | - | 2 | 7 |
|  | **1980-89** | - | 12 | - | 1 | 13 |
|  | **1990-99** | - | 9 | - | 4 | 13 |
|  | **>1999** | - | 6 | - | 1 | 7 |
|  |  |  |  |  |  |  |
| **Principal publication year** | **<1960** | 2 | 0 | 4 | 1 | 7 |
|  | **1960-69** | 0 | 3 | 12 | 1 | 16 |
|  | **1970-79** | 1 | 2 | 23 | 5 | 31 |
|  | **1980-89** | 1 | 11 | 16 | 6 | 34 |
|  | **1990-99** | 6 | 10 | 19 | 4 | 39 |
|  | **>1999** | 10 | 13 | 60 | 8 | 91 |
|  |  |  |  |  |  |  |
| **Type of Population** | **general** | 16 | 13 | 61 | 14 | 104 |
|  | **household** | 1 | 6 | 26 | 3 | 36 |
|  | **long-term resident** | 0 | 3 | 6 | 1 | 10 |
|  | **household + long-term resident** | 0 | 0 | 1 | 1 | 2 |
|  | **phone subscriber** | 0 | 0 | 1 | 0 | 1 |
|  | **employed** | 1 | 5 | 13 | 1 | 20 |
|  | **health insurance member** | 0 | 0 | 1 | 0 | 1 |
|  | **military veterans** | 1 | 3 | 2 | 1 | 7 |
|  | **military veteran + twin** | 0 | 0 | 1 | 0 | 1 |
|  | **other military** | 0 | 1 | 2 | 0 | 3 |
|  | **mixed** | 1 | 3 | 6 | 1 | 11 |
|  | **specfic race** | 0 | 1 | 1 | 0 | 2 |
|  | **volunteer** | 0 | 3 | 6 | 2 | 11 |
|  | **parent** | 0 | 0 | 3 | 1 | 4 |
|  | **siblings of migrants** | 0 | 1 | 0 | 0 | 1 |
|  | **clinic patients** | 0 | 0 | 4 | 0 | 4 |
|  |  |  |  |  |  |  |
|  |  |  |  |  |  |  |
| **Type of controls (for CC studies)** | **population** | 13 | - | - | 2 | 15 |
|  | **hospital** | 5 | - | - | 0 | 5 |
|  | **decedents** | 1 | - | - | 0 | 1 |
|  | **mixed** | 1 | - | - | 1 | 2 |
|  |  |  |  |  |  |  |
| **Type of population - controls (differences from case population)** | **same as cases** | 9 | - | - | 2 | 11 |
|  | **no respiratory symptoms** | 1 | - | - | 0 | 1 |
|  | **no respiratory disease** | 2 | - | - | 0 | 2 |
|  | **no respiratory symptoms/disease** | 1 | - | - | 1 | 2 |
|  | **normal LF** | 2 | - | - | 0 | 2 |
|  | **normal LF, no respiratory symptoms** | 1 | - | - | 0 | 1 |
|  | **normal LF, no major disease** | 2 | - | - | 0 | 2 |
|  | **household members** | 2 | - | - | 0 | 2 |
|  |  |  |  |  |  |  |
| **Any proxy use** |  | 3 | 1 | 6 | 0 | 10 |
|  |  |  |  |  |  |  |
| **Any major study weakness** |  | 9 | 4 | 13 | 6 | 32 |
|  |  |  |  |  |  |  |
| **Matching factors: (CC studies):** |  |  |  |  |  |  |
| **sex** |  | 5 | - | - | 1 | 6 |
| **age** |  | 8 | - | - | 1 | 9 |
| **race** |  | 20 | - | - | 3 | 23 |
| **location (within study area)** |  | 2 | - | - | 0 | 2 |
| **socioeconomic status** |  | 1 | - | - | 0 | 1 |
| **hospital admission (ward, date etc)** |  | 1 | - | - | 0 | 1 |
|  |  |  |  |  |  |  |
| **Results available by :** |  |  |  |  |  |  |
| **current vs never** |  | 14 | 34 | 98 | 23 | 169 |
| **ex vs never** |  | 12 | 29 | 93 | 19 | 153 |
| **ever vs never** |  | 16 | 29 | 113 | 18 | 176 |
| **current vs non** |  | 14 | 28 | 101 | 17 | 160 |
| **amount smoked** |  | 7 | 23 | 47 | 14 | 91 |
| **age started smoking** |  | 5 | 7 | 5 | 3 | 20 |
| **pack-years** |  | 10 | 5 | 43 | 5 | 63 |
| **duration of smoking** |  | 2 | 4 | 6 | 2 | 14 |
| **duration of quitting (vs never)** |  | 3 | 8 | 7 | 4 | 22 |
| **inhalation level** |  | 2 | 5 | 6 | 2 | 15 |
| **filter plain** |  | 2 | 3 | 4 | 3 | 12 |
| **tar level** |  | 1 | 2 | 0 | 1 | 4 |
| **nicotine level** |  | 0 | 1 | 0 | 0 | 1 |
| **other aspects of smoking** |  | 4 | 16 | 27 | 9 | 56 |
|  |  |  |  |  |  |  |
| **Outcomes available in study** |  |  |  |  |  |  |
| **COPD** |  | 14 | 35 | 67 | 17 | 133 |
| **CB** |  | 7 | 9 | 71 | 14 | 101 |
| **Emphysema** |  | 0 | 7 | 19 | 2 | 28 |
|  |  |  |  |  |  |  |
| **COPD only** |  | 13 | 28 | 52 | 11 | 104 |
| **CB only** |  | 6 | 2 | 55 | 7 | 70 |
| **Emp only** |  | 0 | 1 | 8 | 0 | 9 |
| **COPD and CB** |  | 1 | 2 | 8 | 5 | 16 |
| **COPD and Emp** |  | 0 | 1 | 3 | 0 | 4 |
| **CB and Emp** |  | 0 | 1 | 4 | 1 | 6 |
| **COPD, CB and EMP** |  | 0 | 4 | 4 | 1 | 9 |
|  |  |  |  |  |  |  |
| **Results also available for other disease definitions^d^** |  | 0 | 9 | 38 | 9 | 56 |
| **Results also available by severity of COPD^d^** |  | 1 | 2 | 10 | 1 | 14 |
|  |  |  |  |  |  |  |
| **Results by stratifying factors available (other than sex)** |  | 5 | 14 | 62 | 9 | 90 |
|  |  |  |  |  |  |  |
| **Number of relative risks** |  |  |  |  |  |  |
|  | **1-2** | 2 | 5 | 16 | 3 | 26 |
|  | **3-4** | 7 | 10 | 26 | 4 | 47 |
|  | **5-6** | 2 | 5 | 20 | 2 | 29 |
|  | **7-8** | 3 | 1 | 13 | 1 | 18 |
|  | **9-10** | 1 | 4 | 9 | 1 | 15 |
|  | **11-20** | 2 | 2 | 29 | 5 | 38 |
|  | **21-50** | 1 | 9 | 15 | 6 | 31 |
|  | **51-100** | 1 | 3 | 4 | 1 | 9 |
|  | **>100** | 1 | 0 | 2 | 2 | 5 |

## Table 3 (continued – COPD studies)

| **COPD studies^a^** |  | **Study Type^b^** | | | | |
| --- | --- | --- | --- | --- | --- | --- |
| Variable^c^ | Levels^c^ | **CC** | **Prosp** | **CrossSec** | **Subsid** | **Total** |
|  |  |  |  |  |  |  |
| **Total** |  | 14 | 35 | 67 | 17 | 133 |
|  |  |  |  |  |  |  |
| **Study type** | **case/control** | 14 | 0 | 0 | 1 | 15 |
|  | **prospective** | 0 | 35 | 0 | 7 | 42 |
|  | **cross-sectional** | 0 | 0 | 67 | 8 | 75 |
|  | **nested case/control** | 0 | 0 | 0 | 1 | 1 |
|  |  |  |  |  |  |  |
| **Study sex** | **both** | 11 | 18 | 60 | 13 | 102 |
|  | **male** | 2 | 16 | 5 | 4 | 27 |
|  | **female** | 1 | 1 | 2 | 0 | 4 |
|  |  |  |  |  |  |  |
| **Lowest age in study** | **no limit** | 4 | 0 | 5 | 0 | 9 |
|  | **<15** | 0 | 0 | 1 | 0 | 1 |
|  | **15 or adult NOS** | 1 | 0 | 5 | 1 | 7 |
|  | **16-19** | 0 | 3 | 7 | 1 | 11 |
|  | **20-29** | 1 | 10 | 17 | 5 | 33 |
|  | **30-39** | 3 | 14 | 6 | 4 | 27 |
|  | **40-49** | 4 | 6 | 16 | 5 | 31 |
|  | **50-59** | 0 | 0 | 3 | 1 | 4 |
|  | **60-69** | 1 | 2 | 4 | 0 | 7 |
|  | **70+** | 0 | 0 | 3 | 0 | 3 |
|  |  |  |  |  |  |  |
| **Highest age in study (at baseline for prospective study)** | **<50** | 0 | 2 | 1 | 0 | 3 |
|  | **50-59** | 0 | 3 | 1 | 1 | 5 |
|  | **60-69** | 1 | 8 | 11 | 2 | 22 |
|  | **70-79** | 2 | 6 | 11 | 6 | 25 |
|  | **80-89** | 1 | 1 | 4 | 2 | 8 |
|  | **90-98** | 0 | 2 | 1 | 1 | 4 |
|  | **no limit** | 10 | 13 | 38 | 5 | 66 |
|  |  |  |  |  |  |  |
| **Highest age in study at final follow-up (prospective study)** | **60-69** | - | 4 | - | 0 | 4 |
|  | **70-79** | - | 2 | - | 2 | 4 |
|  | **80-89** | - | 9 | - | 2 | 11 |
|  | **90-98** | - | 3 | - | 0 | 3 |
|  | **no limit** | - | 17 | - | 4 | 21 |
|  |  |  |  |  |  |  |
| **Region** | **USA** | 2 | 11 | 14 | 5 | 32 |
|  | **Canada** | 0 | 1 | 3 | 0 | 4 |
|  | **S/C America** | 2 | 0 | 5 | 0 | 7 |
|  | **UK** | 2 | 6 | 3 | 3 | 14 |
|  | **Western Europe** | 2 | 2 | 4 | 0 | 8 |
|  | **Scandinavia** | 0 | 5 | 13 | 5 | 23 |
|  | **E Europe** | 2 | 1 | 6 | 1 | 10 |
|  | **SE Europe/Balkans** | 0 | 0 | 3 | 2 | 5 |
|  | **SE Asia/Pacific** | 0 | 1 | 1 | 0 | 2 |
|  | **Far East** | 3 | 5 | 12 | 1 | 21 |
|  | **Australia/NZ** | 1 | 1 | 1 | 0 | 3 |
|  | **multi** | 0 | 2 | 2 | 0 | 4 |
|  |  |  |  |  |  |  |
| **Start year of study** | **unknown** | 8 | 0 | 7 | 1 | 16 |
|  | **<1960** | 0 | 7 | 0 | 2 | 9 |
|  | **1960-69** | 1 | 11 | 7 | 5 | 24 |
|  | **1970-79** | 0 | 8 | 7 | 5 | 20 |
|  | **1980-89** | 2 | 4 | 6 | 1 | 13 |
|  | **1990-99** | 2 | 5 | 16 | 2 | 25 |
|  | **>1999** | 1 | 0 | 24 | 1 | 26 |
|  |  |  |  |  |  |  |
| **End year of study (of baseline for prospective study)** | **unknown** | 8 | 3 | 7 | 0 | 18 |
|  | **<1960** | 0 | 4 | 0 | 2 | 6 |
|  | **1960-69** | 0 | 8 | 6 | 3 | 17 |
|  | **1970-79** | 1 | 9 | 6 | 6 | 22 |
|  | **1980-89** | 1 | 4 | 5 | 2 | 12 |
|  | **1990-99** | 3 | 7 | 18 | 3 | 31 |
|  | **>1999** | 1 | 0 | 25 | 1 | 27 |
|  |  |  |  |  |  |  |
| **Final follow up year (prospective study)** | **unknown** | - | 5 | - | 0 | 5 |
|  | **1960-69** | - | 2 | - | 1 | 3 |
|  | **1970-79** | - | 4 | - | 1 | 5 |
|  | **1980-89** | - | 11 | - | 1 | 12 |
|  | **1990-99** | - | 8 | - | 4 | 12 |
|  | **>1999** | - | 5 | - | 1 | 6 |
|  |  |  |  |  |  |  |
| **Principal publication year** | **1960-69** | 0 | 3 | 3 | 1 | 7 |
|  | **1970-79** | 1 | 2 | 8 | 3 | 14 |
|  | **1980-89** | 0 | 9 | 2 | 4 | 15 |
|  | **1990-99** | 5 | 9 | 8 | 3 | 25 |
|  | **>1999** | 8 | 12 | 46 | 6 | 72 |
|  |  |  |  |  |  |  |
| **Type of Population** | **general** | 13 | 11 | 31 | 8 | 63 |
|  | **household** | 0 | 6 | 14 | 2 | 22 |
|  | **long-term resident** | 0 | 2 | 6 | 1 | 9 |
|  | **household + long-term resident** | 0 | 0 | 1 | 1 | 2 |
|  | **phone subscriber** | 0 | 0 | 1 | 0 | 1 |
|  | **employed** | 0 | 4 | 3 | 1 | 8 |
|  | **military veterans** | 1 | 3 | 1 | 1 | 6 |
|  | **other military** | 0 | 1 | 0 | 0 | 1 |
|  | **mixed** | 0 | 3 | 3 | 1 | 7 |
|  | **specfic race** | 0 | 1 | 0 | 0 | 1 |
|  | **volunteer** | 0 | 3 | 4 | 2 | 9 |
|  | **siblings of migrants** | 0 | 1 | 0 | 0 | 1 |
|  | **clinic patients** | 0 | 0 | 3 | 0 | 3 |
|  |  |  |  |  |  |  |
| **Type of controls (for CC studies)** | **population** | 10 | - | - | 1 | 11 |
|  | **hospital** | 2 | - | - | 0 | 2 |
|  | **decedents** | 1 | - | - | 0 | 1 |
|  | **mixed** | 1 | - | - | 1 | 2 |
|  |  |  |  |  |  |  |
| **Type of population - controls (differences from case population)** | **same as cases** | 7 | - | - | 2 | 9 |
|  | **no respiratory symptoms/disease** | 1 | - | - | 0 | 1 |
|  | **normal LF** | 2 | - | - | 0 | 2 |
|  | **normal LF, no respiratory symptoms** | 1 | - | - | 0 | 1 |
|  | **normal LF, no major disease** | 1 | - | - | 0 | 1 |
|  | **household members** | 2 | - | - | 0 | 2 |
|  |  |  |  |  |  |  |
| **Any proxy use** |  | 3 | 1 | 0 | 0 | 4 |
|  |  |  |  |  |  |  |
| **Any major study weakness** |  | 7 | 4 | 8 | 4 | 23 |
|  |  |  |  |  |  |  |
| **Matching factors (for CC studies) :** |  |  |  |  |  |  |
| **sex** |  | 2 | - | - | 1 | 3 |
| **age** |  | 5 | - | - | 1 | 6 |
| **location (within study area)** |  | 1 | - | - | 0 | 1 |
|  |  |  |  |  |  |  |
| **Results also available by severity of COPD** |  | 1 | 2 | 10 | 1 | 14 |
|  |  |  |  |  |  |  |
| **Disease definition (subtype)** | **mortality** | 3 | 25 | 0 | 6 | 34 |
|  | **lung function** | 7 | 5 | 47 | 4 | 63 |
|  | **other** | 4 | 5 | 20 | 7 | 36 |
|  |  |  |  |  |  |  |
| **Disease definition** | **ICD** | 3 | 26 | 0 | 6 | 35 |
|  | **LF only** | 7 | 5 | 47 | 4 | 63 |
|  | **LF and symptoms** | 2 | 1 | 1 | 0 | 4 |
|  | **CB or EM** | 0 | 0 | 6 | 1 | 7 |
|  | **COPD CB or EM** | 1 | 1 | 2 | 1 | 5 |
|  | **CB, EM or AST** | 0 | 0 | 2 | 0 | 2 |
|  | **other COPD** | 0 | 2 | 7 | 5 | 14 |
|  | **COPD undefined** | 1 | 0 | 2 | 0 | 3 |
|  |  |  |  |  |  |  |
| **Diagnostic criteria** | **GOLD** | 2 | 2 | 23 | 2 | 29 |
|  | **MRC** | 0 | 0 | 1 | 1 | 2 |
|  | **ATS** | 3 | 1 | 2 | 0 | 6 |
|  | **ERS** | 2 | 0 | 1 | 0 | 3 |
|  | **ICD** | 3 | 21 | 0 | 5 | 29 |
|  | **Mixed** | 0 | 1 | 3 | 4 | 8 |
|  | **unspecified** | 3 | 9 | 32 | 5 | 49 |
|  | **Thoracic Society of Thailand** | 0 | 1 | 1 | 0 | 2 |
|  | **Van Schayck** | 0 | 0 | 1 | 0 | 1 |
|  | **Enright** | 0 | 0 | 1 | 0 | 1 |
|  | **Chinese Soc Resp Dis** | 0 | 0 | 1 | 0 | 1 |
|  | **BTS** | 1 | 0 | 1 | 0 | 2 |
|  |  |  |  |  |  |  |
| **ICD7 codes** | **501-502,527.1,(527.2 later)** | - | 1 | - | 0 | 1 |
|  | **500-502,527.1** | - | 2 | - | 1 | 3 |
|  | **241,500-502,527.1** | - | 0 | - | 1 | 1 |
|  | **502,526,527.1** | - | 2 | - | 0 | 2 |
|  | **unspecified** | - | 2 | - | 1 | 3 |
|  |  |  |  |  |  |  |
| **ICD8 codes** | **490-492** | 0 | 2 | - | 1 | 3 |
|  | **490-492,519** | 0 | 1 | - | 0 | 1 |
|  | **490-493** | 1 | 0 | - | 2 | 3 |
|  | **466, 490-493** | 0 | 1 | - | 0 | 1 |
|  | **490-493,518,519.3** | 0 | 1 | - | 0 | 1 |
|  | **491-492,519** | 0 | 1 | - | 0 | 1 |
|  | **unspecified** | 0 | 1 | - | 1 | 2 |
|  |  |  |  |  |  |  |
| **ICD 9 codes** | **491,492,496** | 0 | 1 | - | 0 | 1 |
|  | **490-492,496** | 1 | 1 | - | 0 | 2 |
|  | **490-492,496,416.7** | 1 | 0 | - | 0 | 1 |
|  | **490-496** | 0 | 7 | - | 1 | 8 |
|  | **416,491,492,496,519** | 0 | 0 | - | 1 | 1 |
|  | **466, 490-493** | 0 | 1 | - | 0 | 1 |
|  | **unspecified** | 0 | 2 | - | 2 | 4 |
|  |  |  |  |  |  |  |
| **ICD 10 codes** | **J40-J44** | - | 1 | - | 0 | 1 |
|  | **unspecified** | - | 2 | - | 1 | 3 |
|  |  |  |  |  |  |  |
| **Analysis type for asthma** | **include irrespective of asthma** | 3 | 16 | 50 | 9 | 78 |
|  | **excluding all asthmatics** | 5 | 3 | 10 | 1 | 19 |
|  | **cases include but controls exclude asthmatics** | 4 | 0 | 0 | 0 | 4 |
|  | **COPD definition includes asthma** | 1 | 9 | 4 | 6 | 20 |
|  | **unkn if COPD defn includes asthma** | 1 | 6 | 2 | 1 | 10 |
|  | **cases exclude but controls include asthmatics (cannot have both diagnoses)** | 0 | 1 | 1 | 0 | 2 |
|  |  |  |  |  |  |  |
| **Lung function criteria** | **FEV1/FVC <70%** | 2 | 2 | 30 | 3 | 37 |
|  | **FEV1/FVC<70% and FEV1<80% predicted** | 1 | 0 | 2 | 1 | 4 |
|  | **FEV1/FVC<88%M, 89%F** | 1 | 0 | 1 | 1 | 3 |
|  | **FEV1/FVC<normal (ATS value nk)** | 2 | 0 | 1 | 0 | 3 |
|  | **FEV1<65% predicted or FEV/FVC<65%** | 0 | 1 | 0 | 0 | 1 |
|  | **FEV1% <60** | 0 | 0 | 1 | 1 | 2 |
|  | **FEV1/FVC <80%** | 0 | 0 | 1 | 0 | 1 |
|  | **FEV1 <75% predicted** | 1 | 0 | 0 | 0 | 1 |
|  | **FEV1/or VC <70%predicted** | 0 | 1 | 0 | 0 | 1 |
|  | **FEV1 <65% predicted** | 0 | 1 | 1 | 0 | 2 |
|  | **FEV1/FVC<70% + reversibility<15%** | 0 | 1 | 1 | 0 | 2 |
|  | **FEV1/FVC<88% + reversibility<12%** | 0 | 0 | 1 | 0 | 1 |
|  | **FEV1<5th%ile + reversibility<9%** | 0 | 0 | 1 | 0 | 1 |
|  | **FEV1/FVC <65%** | 0 | 0 | 1 | 0 | 1 |
|  | **FEV1 or MMEF <2SD below predicted** | 0 | 0 | 1 | 0 | 1 |
|  | **FEV1 or DLCO <80% predicted** | 0 | 1 | 0 | 0 | 1 |
|  | **FEV1/FVC <68%** | 0 | 0 | 2 | 0 | 2 |
|  | **FEV1/FVC <60%** | 0 | 0 | 5 | 2 | 7 |
|  | **GOLD2+ (FEV1/FVC<70%, FEV1<80%)** | 1 | 1 | 2 | 1 | 5 |
|  | **FEV1 < 85% predicted** | 0 | 0 | 1 | 0 | 1 |
|  | **ATS NOS** | 1 | 0 | 0 | 0 | 1 |
|  | **FEV1/FVC<75%** | 0 | 0 | 1 | 0 | 1 |
|  | **FEV1/FVC<70%, FEV1<80% predicted, reversibility <12** | 1 | 0 | 0 | 0 | 1 |
|  | **FEV1 > 2SE below predicted** | 0 | 0 | 1 | 0 | 1 |
|  | **unspecified** | 0 | 0 | 1 | 0 | 1 |
|  |  |  |  |  |  |  |
| **Spirometry with bronchodilator** | **post-bronchodilator** | 0 | 1 | 11 | 2 | 14 |
|  | **without/pre/unknown** | 9 | 6 | 39 | 7 | 61 |
|  | **pre + post-bronchodilator** | 1 | 1 | 5 | 0 | 7 |
|  |  |  |  |  |  |  |
| **Source of diagnosis** | **Hospital/GP records** | 1 | 2 | 0 | 0 | 3 |
|  | **Questionnaire: physician diagnosis** | 0 | 1 | 7 | 1 | 9 |
|  | **Questionnaire: other** | 0 | 0 | 3 | 0 | 3 |
|  | **Spirometry** | 8 | 5 | 47 | 4 | 64 |
|  | **Death certificate/ registry** | 2 | 21 | 0 | 6 | 29 |
|  | **Mixed** | 3 | 6 | 10 | 6 | 25 |
|  |  |  |  |  |  |  |
| **Questionnaire** | **none** | 14 | 32 | 48 | 11 | 105 |
|  | **MRC** | - | 0 | 3 | 4 | 7 |
|  | **NHLI** | - | 1 | 0 | 0 | 1 |
|  | **ATS** | - | 1 | 2 | 0 | 3 |
|  | **FinEsS** | - | 0 | 1 | 0 | 1 |
|  | **Harvard** | - | 0 | 1 | 0 | 1 |
|  | **OLIN** | - | 1 | 1 | 0 | 2 |
|  | **NHS** | - | 0 | 1 | 0 | 1 |
|  | **MRC/ATS** | - | 0 | 1 | 0 | 1 |
|  | **ATS/NHLBI** | - | 0 | 1 | 0 | 1 |
|  | **unnamed/ unspecified** | - | 0 | 8 | 2 | 10 |
|  |  |  |  |  |  |  |
| **Type of outcome** | **prevalence** | 10 | 2 | 67 | 10 | 89 |
|  | **mortality** | 3 | 25 | 0 | 6 | 34 |
|  | **incidence** | 1 | 8 | 0 | 1 | 10 |
|  |  |  |  |  |  |  |
| **Number of COPD cases** | **unknown** | 1 | 0 | 0 | 0 | 1 |
|  | **1-100** | 10 | 19 | 19 | 7 | 55 |
|  | **101-200** | 1 | 8 | 18 | 3 | 30 |
|  | **201-500** | 0 | 4 | 17 | 4 | 25 |
|  | **501-1000** | 1 | 2 | 3 | 1 | 7 |
|  | **>1000** | 1 | 2 | 10 | 2 | 15 |
|  | Median | 64.00 | 91.00 | 178.00 | 195.00 | 134.00 |
|  | Range | 19:32822 | 13:3483 | 34:21506 | 24:2243 | 13:32822 |
|  | Median (based on principal studies only) |  |  |  |  | 131.00 |
|  | Range |  |  |  |  | 13:32822 |
|  |  |  |  |  |  |  |
| **Total number of subjects** | **unknown** | 1 | 0 | 0 | 0 | 1 |
|  | **1-100** | 5 | 0 | 0 | 0 | 5 |
|  | **101-200** | 2 | 0 | 0 | 0 | 2 |
|  | **201-500** | 4 | 1 | 10 | 2 | 17 |
|  | **501-1000** | 0 | 5 | 10 | 4 | 19 |
|  | **>1000** | 2 | 29 | 47 | 11 | 89 |
|  | Median | 200.00 | 7633.00 | 1727.00 | 1530.00 | 2032.50 |
|  | Range | 29:261535 | 399:786387 | 207:105380 | 336:248195 | 29:786387 |
|  | Median (based on principal studies only) |  |  |  | - | 2033.00 |
|  | Range |  |  |  | - | 29:786387 |
|  |  |  |  |  |  |  |
| **Total number of adjustment factors used** | **none** | 9 | 10 | 33 | 6 | 58 |
|  | **1** | 1 | 10 | 12 | 7 | 30 |
|  | **2** | 0 | 5 | 3 | 1 | 9 |
|  | **3** | 2 | 4 | 5 | 1 | 12 |
|  | **4** | 1 | 1 | 5 | 0 | 7 |
|  | **5** | 0 | 1 | 2 | 1 | 4 |
|  | **6** | 1 | 2 | 3 | 0 | 6 |
|  | **7** | 0 | 2 | 1 | 1 | 4 |
|  | **8** | 0 | 0 | 1 | 0 | 1 |
|  | **9** | 0 | 0 | 1 | 0 | 1 |
|  | **13** | 0 | 0 | 1 | 0 | 1 |
|  |  |  |  |  |  |  |
| **Adjusted for :^e^** |  |  |  |  |  |  |
| **sex** |  | 0 | 3 | 15 | 2 | 20 |
| **age** |  | 5 | 25 | 30 | 11 | 71 |
| **race** |  | 0 | 0 | 3 | 0 | 3 |
| **location within study (1)** |  | 1 | 3 | 6 | 3 | 13 |
| **location within study (2)** |  | 1 | 0 | 0 | 0 | 1 |
| **aspects of study design** |  | 0 | 5 | 0 | 0 | 5 |
| **family (parent/sibling) medical history** |  | 1 | 1 | 5 | 0 | 7 |
| **household composition** |  | 0 | 1 | 1 | 0 | 2 |
| **SES/education (1)** |  | 2 | 1 | 9 | 2 | 14 |
| **SES/education (2)** |  | 0 | 0 | 2 | 0 | 2 |
| **occupation (1)** |  | 1 | 1 | 7 | 0 | 9 |
| **occupation (4)** |  | 0 | 0 | 0 | 1 | 1 |
| **cooking/heating/aircon/ventilation (1)** |  | 1 | 0 | 1 | 0 | 2 |
| **cooking/heating/ aircon/ ventilation (3)** |  | 0 | 0 | 1 | 0 | 1 |
| **housing quality** |  | 0 | 0 | 1 | 0 | 1 |
| **exposure to allergens (4)** |  | 0 | 1 | 0 | 0 | 1 |
| **other medical history/symptoms (1)** |  | 0 | 0 | 4 | 0 | 4 |
| **other medical history/symptoms (2)** |  | 0 | 1 | 0 | 0 | 1 |
| **other medical history/symptoms (3)** |  | 0 | 3 | 1 | 0 | 4 |
| **obesity/BMI** |  | 1 | 2 | 6 | 0 | 9 |
| **exercise** |  | 0 | 1 | 1 | 0 | 2 |
| **diet/alcohol (1)** |  | 1 | 3 | 1 | 1 | 6 |
| **diet/alcohol (2)** |  | 0 | 0 | 2 | 0 | 2 |
| **diet/alcohol (7)** |  | 0 | 0 | 1 | 0 | 1 |
| **ETS exposure (1)** |  | 1 | 0 | 1 | 0 | 2 |
| **other aspects of smoking (1)** |  | 1 | 4 | 1 | 1 | 7 |
| **other aspects of smoking (2)** |  | 0 | 1 | 0 | 0 | 1 |
| **other aspects of smoking (5)** |  | 0 | 0 | 1 | 0 | 1 |
|  |  |  |  |  |  |  |
| **Other confounders considered but rejected** |  | 0 | 0 | 2 | 1 | 3 |
|  |  |  |  |  |  |  |
| **Smoking results available for COPD :** |  |  |  |  |  |  |
| **current vs never** |  | 9 | 30 | 50 | 15 | 104 |
| **ex vs never** |  | 7 | 27 | 48 | 11 | 93 |
| **ever vs never** |  | 11 | 27 | 57 | 11 | 106 |
| **current vs non (as well as curr vs never)** |  | 8 | 25 | 48 | 11 | 92 |
| **current vs non (and not curr vs never)** |  | 1 | 2 | 3 | 0 | 6 |
| **amount smoked** |  | 3 | 19 | 17 | 7 | 46 |
| **age started smoking** |  | 3 | 6 | 4 | 2 | 15 |
| **pack-years** |  | 9 | 3 | 30 | 5 | 47 |
| **duration of smoking** |  | 2 | 1 | 3 | 1 | 7 |
| **duration of quitting (vs never)** |  | 2 | 4 | 2 | 2 | 10 |
| **duration of quitting (vs current)** |  | 1 | 3 | 2 | 2 | 8 |

## Table 3 (continued – CB studies)

| **CB studies^a^** |  | **Study Type^b^** | | | | |
| --- | --- | --- | --- | --- | --- | --- |
| Variable^c^ | Levels^c^ | **CC** | **Prosp** | **CrossSec** | **Subsid** | **Total** |
|  |  |  |  |  |  |  |
| **Total** |  | 7 | 9 | 71 | 14 | 101 |
|  |  |  |  |  |  |  |
| **Study type** | **case/control** | 7 | 0 | 0 | 1 | 8 |
|  | **prospective** | 0 | 9 | 0 | 4 | 13 |
|  | **cross-sectional** | 0 | 0 | 71 | 9 | 80 |
|  |  |  |  |  |  |  |
| **Study sex** | **both** | 6 | 3 | 53 | 11 | 73 |
|  | **male** | 0 | 4 | 16 | 3 | 23 |
|  | **female** | 1 | 2 | 2 | 0 | 5 |
|  |  |  |  |  |  |  |
| **Lowest age in study** | **<15** | 0 | 1 | 2 | 0 | 3 |
|  | **15 or adult NOS** | 0 | 0 | 14 | 1 | 15 |
|  | **16-19** | 0 | 0 | 5 | 1 | 6 |
|  | **20-29** | 2 | 3 | 20 | 5 | 30 |
|  | **30-39** | 3 | 4 | 5 | 3 | 15 |
|  | **40-49** | 2 | 1 | 14 | 2 | 19 |
|  | **50-59** | 0 | 0 | 4 | 1 | 5 |
|  | **60-69** | 0 | 0 | 4 | 1 | 5 |
|  | **70+** | 0 | 0 | 3 | 0 | 3 |
|  |  |  |  |  |  |  |
| **Highest age in study (at baseline for prospective study)** | **unknown** | 0 | 0 | 1 | 0 | 1 |
|  | **<50** | 0 | 0 | 6 | 1 | 7 |
|  | **50-59** | 0 | 1 | 7 | 1 | 9 |
|  | **60-69** | 1 | 0 | 20 | 4 | 25 |
|  | **70-79** | 2 | 0 | 10 | 5 | 17 |
|  | **80-89** | 1 | 1 | 1 | 1 | 4 |
|  | **90-98** | 0 | 1 | 2 | 0 | 3 |
|  | **no limit** | 3 | 6 | 24 | 2 | 35 |
|  |  |  |  |  |  |  |
| **Highest age in study at final follow-up (prospective study)** | **60-69** | - | 1 | - | 0 | 1 |
|  | **70-79** | - | 0 | - | 2 | 2 |
|  | **80-89** | - | 0 | - | 2 | 2 |
|  | **90-98** | - | 1 | - | 0 | 1 |
|  | **no limit** | - | 7 | - | 0 | 7 |
|  |  |  |  |  |  |  |
| **Region** | **USA** | 0 | 4 | 20 | 4 | 28 |
|  | **Canada** | 0 | 1 | 3 | 0 | 4 |
|  | **S/C America** | 1 | 0 | 1 | 0 | 2 |
|  | **UK** | 3 | 2 | 8 | 3 | 16 |
|  | **Western Europe** | 2 | 0 | 7 | 1 | 10 |
|  | **Scandinavia** | 0 | 0 | 16 | 4 | 20 |
|  | **E Europe** | 0 | 0 | 3 | 1 | 4 |
|  | **SE Europe/Balkans** | 0 | 0 | 1 | 1 | 2 |
|  | **Middle East/S Asia** | 0 | 0 | 4 | 0 | 4 |
|  | **Far East** | 1 | 2 | 3 | 0 | 6 |
|  | **Australia/NZ** | 0 | 0 | 2 | 0 | 2 |
|  | **Africa** | 0 | 0 | 2 | 0 | 2 |
|  | **multi** | 0 | 0 | 1 | 0 | 1 |
|  |  |  |  |  |  |  |
| **Start year of study** | **unknown** | 2 | 0 | 14 | 1 | 17 |
|  | **<1960** | 2 | 4 | 4 | 3 | 13 |
|  | **1960-69** | 0 | 1 | 14 | 6 | 21 |
|  | **1970-79** | 1 | 2 | 16 | 2 | 21 |
|  | **1980-89** | 0 | 2 | 8 | 1 | 11 |
|  | **1990-99** | 1 | 0 | 15 | 1 | 17 |
|  | **>1999** | 1 | 0 | 0 | 0 | 1 |
|  |  |  |  |  |  |  |
| **End year of study (of baseline for prospective study)** | **unknown** | 2 | 1 | 14 | 1 | 18 |
|  | **<1960** | 2 | 4 | 4 | 3 | 13 |
|  | **1960-69** | 0 | 1 | 11 | 4 | 16 |
|  | **1970-79** | 0 | 1 | 17 | 4 | 22 |
|  | **1980-89** | 1 | 1 | 9 | 1 | 12 |
|  | **1990-99** | 1 | 1 | 15 | 1 | 18 |
|  | **>1999** | 1 | 0 | 1 | 0 | 2 |
|  |  |  |  |  |  |  |
| **Final follow up year (prospective study)** | **unknown** | - | 1 | - | 0 | 1 |
|  | **1960-69** | - | 1 | - | 1 | 2 |
|  | **1970-79** | - | 2 | - | 2 | 4 |
|  | **1980-89** | - | 2 | - | 0 | 2 |
|  | **1990-99** | - | 2 | - | 0 | 2 |
|  | **>1999** | - | 1 | - | 1 | 2 |
|  |  |  |  |  |  |  |
| **Principal publication year** | **<1960** | 2 | 0 | 4 | 1 | 7 |
|  | **1960-69** | 0 | 2 | 10 | 1 | 13 |
|  | **1970-79** | 0 | 0 | 16 | 4 | 20 |
|  | **1980-89** | 1 | 3 | 14 | 4 | 22 |
|  | **1990-99** | 2 | 2 | 12 | 1 | 17 |
|  | **>1999** | 2 | 2 | 15 | 3 | 22 |
|  |  |  |  |  |  |  |
| **Type of Population** | **general** | 4 | 2 | 32 | 8 | 46 |
|  | **household** | 1 | 1 | 14 | 2 | 18 |
|  | **long-term resident** | 0 | 0 | 2 | 1 | 3 |
|  | **employed** | 1 | 3 | 10 | 0 | 14 |
|  | **health insurance member** | 0 | 0 | 1 | 0 | 1 |
|  | **military veterans** | 0 | 2 | 0 | 1 | 3 |
|  | **military veteran + twin** | 0 | 0 | 1 | 0 | 1 |
|  | **other military** | 0 | 0 | 2 | 0 | 2 |
|  | **mixed** | 1 | 1 | 3 | 1 | 6 |
|  | **specfic race** | 0 | 0 | 1 | 0 | 1 |
|  | **volunteer** | 0 | 0 | 1 | 0 | 1 |
|  | **parent** | 0 | 0 | 3 | 1 | 4 |
|  | **clinic patients** | 0 | 0 | 1 | 0 | 1 |
|  |  |  |  |  |  |  |
| **Type of controls (for CC studies)** | **population** | 3 | - | - | 1 | 4 |
|  | **hospital** | 3 | - | - | 0 | 3 |
|  | **mixed** | 1 | - | - | 0 | 1 |
|  |  |  |  |  |  |  |
| **Type of population - controls (differences from case population)** | **same as cases** | 3 | - | - | 0 | 3 |
|  | **no respiratory symptoms** | 1 | - | - | 0 | 1 |
|  | **no respiratory disease** | 2 | - | - | 0 | 2 |
|  | **no respiratory symptoms/disease** | 0 | - | - | 1 | 1 |
|  | **normal LF, no major disease** | 1 | - | - | 0 | 1 |
|  |  |  |  |  |  |  |
| **Any proxy use** |  | 0 | 0 | 3 | 0 | 3 |
|  |  |  |  |  |  |  |
| **Any major study weakness** |  | 2 | 0 | 3 | 2 | 7 |
|  |  |  |  |  |  |  |
| **Matching factors (for CC studies) :** |  |  |  |  |  |  |
| **sex** |  | 3 | - | - | 0 | 3 |
| **age** |  | 3 | - | - | 0 | 3 |
| **location (within study area)** |  | 1 | - | - | 0 | 1 |
| **socioeconomic status** |  | 1 | - | - | 0 | 1 |
| **hospital admission (ward, date etc)** |  | 1 | - | - | 0 | 1 |
|  |  |  |  |  |  |  |
| **Disease definition** | **CB based on ICD** | 0 | 6 | 0 | 1 | 7 |
|  | **CB based on symptoms not LF** | 5 | 1 | 53 | 10 | 69 |
|  | **CB self-reported** | 0 | 0 | 3 | 0 | 3 |
|  | **CB diagnosed** | 1 | 1 | 11 | 1 | 14 |
|  | **CB other** | 1 | 1 | 4 | 2 | 8 |
|  |  |  |  |  |  |  |
| **Diagnostic criteria** | **MRC** | 2 | 1 | 18 | 2 | 23 |
|  | **ATS** | 0 | 1 | 3 | 0 | 4 |
|  | **ICD** | 0 | 5 | 0 | 1 | 6 |
|  | **Mixed** | 1 | 0 | 0 | 1 | 2 |
|  | **unspecified** | 4 | 2 | 47 | 10 | 63 |
|  | **Rose** | 0 | 0 | 1 | 0 | 1 |
|  | **Fletcher** | 0 | 0 | 2 | 0 | 2 |
|  |  |  |  |  |  |  |
| **ICD7 codes** | **500-502** | - | 3 | - | 1 | 4 |
|  | **unspecified** | - | 2 | - | 1 | 3 |
|  |  |  |  |  |  |  |
| **ICD8 codes** | **unspecified** | - | - | - | 1 | 1 |
|  |  |  |  |  |  |  |
| **ICD 9 codes** | **unspecified** | - | 0 | - | 1 | 1 |
|  | **491** | - | 1 | - | 0 | 1 |
|  |  |  |  |  |  |  |
| **ICD 10 codes** | **unspecified** | - | - | - | 1 | 1 |
|  |  |  |  |  |  |  |
| **Analysis type for asthma** | **include irrespective of asthma** | 2 | 8 | 68 | 10 | 88 |
|  | **excluding all asthmatics** | 2 | 1 | 3 | 3 | 9 |
|  | **cases include but controls exclude asthmatics** | 3 | 0 | 0 | 1 | 4 |
|  |  |  |  |  |  |  |
| **Lung function criteria** | **FEV1 <80% predicted** | - | 1 | 0 | - | 1 |
|  | **FEV1/FVC <88% predicted** | - | 0 | 1 | - | 1 |
|  |  |  |  |  |  |  |
| **Spirometry with bronchodilator** | **without/pre/ unknown** | - | 1 | 1 | - | 2 |
|  |  |  |  |  |  |  |
| **Source of diagnosis** | **Hospital/GP records** | 3 | 0 | 0 | 0 | 3 |
|  | **Questionnaire: physician diagnosis** | 0 | 1 | 8 | 0 | 9 |
|  | **Questionnaire: other** | 2 | 2 | 53 | 9 | 66 |
|  | **Death certificate/registry** | 0 | 6 | 0 | 1 | 7 |
|  | **Other** | 0 | 0 | 4 | 0 | 4 |
|  | **Mixed** | 1 | 0 | 6 | 4 | 11 |
|  | **Unspecified** | 1 | 0 | 0 | 0 | 1 |
|  |  |  |  |  |  |  |
| **CB: Questionnaire** | **none** | 4 | 6 | 7 | 1 | 18 |
|  | **MRC** | 0 | 0 | 23 | 9 | 32 |
|  | **ECRHS** | 0 | 0 | 3 | 1 | 4 |
|  | **NHLI** | 0 | 1 | 1 | 0 | 2 |
|  | **ATS** | 1 | 0 | 4 | 0 | 5 |
|  | **NHLBI** | 0 | 0 | 2 | 0 | 2 |
|  | **WHO** | 0 | 0 | 1 | 0 | 1 |
|  | **Rose** | 0 | 0 | 1 | 0 | 1 |
|  | **FinEsS** | 0 | 0 | 2 | 0 | 2 |
|  | **ECSC** | 0 | 0 | 1 | 0 | 1 |
|  | **Harvard** | 0 | 0 | 1 | 0 | 1 |
|  | **NHANES** | 0 | 0 | 2 | 0 | 2 |
|  | **OLIN** | 0 | 0 | 1 | 0 | 1 |
|  | **NHS** | 0 | 1 | 0 | 0 | 1 |
|  | **MRC/ATS** | 0 | 0 | 1 | 0 | 1 |
|  | **MRC/ECRHS** | 0 | 0 | 2 | 0 | 2 |
|  | **Health Omnibus Survey** | 0 | 0 | 1 | 0 | 1 |
|  | **CECA** | 0 | 0 | 1 | 0 | 1 |
|  | **unnamed/ unspecified** | 2 | 1 | 17 | 3 | 23 |
|  |  |  |  |  |  |  |
| **Type of outcome** | **prevalence** | 7 | 0 | 71 | 11 | 89 |
|  | **mortality** | 0 | 6 | 0 | 1 | 7 |
|  | **incidence** | 0 | 3 | 0 | 2 | 5 |
|  |  |  |  |  |  |  |
| **Number of cases** | **unknown** | 0 | 1 | 2 | 0 | 3 |
|  | **1-100** | 2 | 5 | 17 | 2 | 26 |
|  | **101-200** | 1 | 2 | 16 | 4 | 23 |
|  | **201-500** | 3 | 0 | 18 | 6 | 27 |
|  | **501-1000** | 1 | 1 | 7 | 1 | 10 |
|  | **>1000** | 0 | 0 | 11 | 1 | 12 |
|  | Median | 300.00 | 85.00 | 209.00 | 254.00 | 198.50 |
|  | Range | 6:973 | 2:798 | 8:4769 | 40:1119 | 2:4769 |
|  | Median (based on principal studies only) |  |  |  |  | 193.50 |
|  | Range |  |  |  |  | 2:4769 |
|  |  |  |  |  |  |  |
| **Total number of subjects** | **1-100** | 2 | 0 | 1 | 0 | 3 |
|  | **101-200** | 0 | 0 | 1 | 0 | 1 |
|  | **201-500** | 1 | 0 | 10 | 1 | 12 |
|  | **501-1000** | 2 | 1 | 9 | 2 | 14 |
|  | **>1000** | 2 | 8 | 50 | 11 | 71 |
|  | Median | 600.00 | 53091.00 | 2435.00 | 2857.00 | 2826.00 |
|  | Range | 10:5255 | 901:265118 | 85:72284 | 460:248195 | 10:265118 |
|  | Median (based on principal studies only) |  |  |  |  | 2826.00 |
|  | Range |  |  |  |  | 10:265118 |
|  |  |  |  |  |  |  |
| **Total number of adjustment factors used** | **none** | 5 | 1 | 24 | 3 | 33 |
|  | **1** | 2 | 6 | 21 | 8 | 37 |
|  | **2** | 0 | 1 | 7 | 0 | 8 |
|  | **3** | 0 | 0 | 4 | 1 | 5 |
|  | **4** | 0 | 0 | 4 | 0 | 4 |
|  | **5** | 0 | 0 | 2 | 1 | 3 |
|  | **6** | 0 | 1 | 5 | 1 | 7 |
|  | **7** | 0 | 0 | 3 | 0 | 3 |
|  | **8** | 0 | 0 | 1 | 0 | 1 |
|  |  |  |  |  |  |  |
| **Adjusted for :^e^** |  |  |  |  |  |  |
| **sex** |  | 0 | 1 | 18 | 1 | 20 |
| **age** |  | 1 | 8 | 38 | 9 | 56 |
| **race** |  | 0 | 0 | 2 | 0 | 2 |
| **location within study (1)** |  | 0 | 0 | 13 | 3 | 16 |
| **location within study (2)** |  | 0 | 0 | 2 | 0 | 2 |
| **aspects of study design (1)** |  | 0 | 1 | 0 | 0 | 1 |
| **aspects of study design (3)** |  | 0 | 0 | 1 | 0 | 1 |
| **family (parent/sibling) medical history (1)** |  | 0 | 0 | 3 | 0 | 3 |
| **family (parent/sibling) medical history (2)** |  | 0 | 0 | 1 | 0 | 1 |
| **SES/education** |  | 0 | 0 | 13 | 0 | 13 |
| **occupation (1)** |  | 0 | 0 | 7 | 1 | 8 |
| **occupation (4)** |  | 0 | 0 | 0 | 1 | 1 |
| **cooking/heating/aircon/ventilation** |  | 0 | 0 | 4 | 0 | 4 |
| **housing quality** |  | 0 | 0 | 1 | 0 | 1 |
| **exposure to allergens** |  | 0 | 0 | 1 | 0 | 1 |
| **other medical history/symptoms (1)** |  | 1 | 0 | 7 | 0 | 8 |
| **other medical history/symptoms (2)** |  | 0 | 0 | 1 | 1 | 2 |
| **other medical history/symptoms (4)** |  | 0 | 1 | 0 | 0 | 1 |
| **obesity/BMI** |  | 0 | 0 | 2 | 0 | 2 |
| **diet/alcohol (1)** |  | 0 | 0 | 0 | 1 | 1 |
| **diet/alcohol (2)** |  | 0 | 0 | 2 | 0 | 2 |
| **diet/alcohol (3)** |  | 0 | 0 | 1 | 0 | 1 |
| **ETS exposure** |  | 0 | 0 | 1 | 0 | 1 |
| **other aspects of smoking** |  | 0 | 0 | 4 | 1 | 5 |
|  |  |  |  |  |  |  |
| **Other confounders considered but rejected** |  | 0 | 0 | 4 | 1 | 5 |
|  |  |  |  |  |  |  |
| **Smoking results available for CB:** |  |  |  |  |  |  |
| **for current vs never** |  | 5 | 8 | 57 | 14 | 84 |
| **for ex vs never** |  | 5 | 6 | 54 | 13 | 78 |
| **for ever vs never** |  | 6 | 5 | 60 | 12 | 83 |
| **for current vs non (as well as curr vs never)** |  | 4 | 3 | 53 | 11 | 71 |
| **for current vs non (and not curr vs never)** |  | 1 | 0 | 5 | 0 | 6 |
| **amount smoked** |  | 4 | 5 | 35 | 9 | 53 |
| **age started smoking** |  | 2 | 0 | 1 | 1 | 4 |
| **pack-years** |  | 2 | 0 | 11 | 1 | 14 |
| **duration of smoking** |  | 0 | 2 | 2 | 0 | 4 |
| **duration of quitting (vs never)** |  | 1 | 2 | 4 | 1 | 8 |
| **duration of quitting (vs current)** |  | 1 | 2 | 3 | 1 | 7 |

## Table 3 (continued – Emphysema studies)

| **Emphysema studies^a^** |  | **Study Type^b^** | | | | |
| --- | --- | --- | --- | --- | --- | --- |
| Variable^c^ | Levels^c^ | **CC** | **Prosp** | **CrossSec** | **Subsid** | **Total** |
|  |  |  |  |  |  |  |
| **Total** |  | - | 7 | 19 | 2 | 28 |
|  |  |  |  |  |  |  |
| **Study type** | **prospective** | - | 7 | 0 | 2 | 9 |
|  | **cross-sectional** | - | 0 | 19 | 0 | 19 |
|  |  |  |  |  |  |  |
| **Study sex** | **both** | - | 3 | 15 | 0 | 18 |
|  | **male** | - | 4 | 4 | 2 | 10 |
|  |  |  |  |  |  |  |
| **Lowest age in study** | **<15** | - | 0 | 1 | 0 | 1 |
|  | **15 or adult NOS** | - | 0 | 8 | 0 | 8 |
|  | **16-19** | - | 0 | 1 | 0 | 1 |
|  | **20-29** | - | 1 | 1 | 1 | 3 |
|  | **30-39** | - | 4 | 0 | 1 | 5 |
|  | **40-49** | - | 1 | 3 | 0 | 4 |
|  | **50-59** | - | 0 | 2 | 0 | 2 |
|  | **60-69** | - | 1 | 1 | 0 | 2 |
|  | **70+** | - | 0 | 2 | 0 | 2 |
|  |  |  |  |  |  |  |
| **Highest age in study (at baseline for prospective study)** | **60-69** | - | 1 | 5 | 0 | 6 |
|  | **70-79** | - | 0 | 1 | 1 | 2 |
|  | **80-89** | - | 1 | 0 | 1 | 2 |
|  | **90-98** | - | 1 | 3 | 0 | 4 |
|  | **no limit** | - | 4 | 10 | 0 | 14 |
|  |  |  |  |  |  |  |
| **Highest age in study at final follow-up (prospective study)** | **60-69** | - | 1 | - | 0 | 1 |
|  | **80-89** | - | 0 | - | 2 | 2 |
|  | **90-98** | - | 1 | - | 0 | 1 |
|  | **no limit** | - | 5 | - | 0 | 5 |
|  |  |  |  |  |  |  |
| **Region** | **USA** | - | 3 | 8 | 1 | 12 |
|  | **Canada** | - | 1 | 0 | 0 | 1 |
|  | **UK** | - | 0 | 1 | 0 | 1 |
|  | **Western Europe** | - | 0 | 1 | 0 | 1 |
|  | **Scandinavia** | - | 1 | 4 | 0 | 5 |
|  | **SE Europe/Balkans** | - | 0 | 0 | 1 | 1 |
|  | **Far East** | - | 2 | 4 | 0 | 6 |
|  | **Australia/NZ** | - | 0 | 1 | 0 | 1 |
|  |  |  |  |  |  |  |
| **Start year of study** | **unknown** | - | 0 | 4 | 0 | 4 |
|  | **<1960** | - | 3 | 0 | 1 | 4 |
|  | **1960-69** | - | 1 | 3 | 1 | 5 |
|  | **1970-79** | - | 1 | 4 | 0 | 5 |
|  | **1980-89** | - | 1 | 3 | 0 | 4 |
|  | **1990-99** | - | 1 | 5 | 0 | 6 |
|  |  |  |  |  |  |  |
| **End year of study (of baseline for prospective study)** | **unknown** | - | 1 | 4 | 0 | 5 |
|  | **<1960** | - | 2 | 0 | 1 | 3 |
|  | **1960-69** | - | 2 | 2 | 1 | 5 |
|  | **1970-79** | - | 0 | 5 | 0 | 5 |
|  | **1980-89** | - | 0 | 2 | 0 | 2 |
|  | **1990-99** | - | 2 | 5 | 0 | 7 |
|  | **>1999** | - | 0 | 1 | 0 | 1 |
|  |  |  |  |  |  |  |
| **Final follow up year (prospective study)** | **unknown** | - | 1 | - | 0 | 1 |
|  | **1960-69** | - | 2 | - | 1 | 3 |
|  | **1970-79** | - | 0 | - | 1 | 1 |
|  | **1980-89** | - | 2 | - | 0 | 2 |
|  | **>1999** | - | 2 | - | 0 | 2 |
|  |  |  |  |  |  |  |
| **Principal publication year** | **1960-69** | - | 3 | 3 | 1 | 7 |
|  | **1970-79** | - | 0 | 5 | 0 | 5 |
|  | **1980-89** | - | 1 | 3 | 1 | 5 |
|  | **1990-99** | - | 0 | 2 | 0 | 2 |
|  | **>1999** | - | 3 | 6 | 0 | 9 |
|  |  |  |  |  |  |  |
| **Type of Population** | **general** | - | 1 | 9 | 1 | 11 |
|  | **household** | - | 2 | 4 | 0 | 6 |
|  | **long-term resident** | - | 1 | 1 | 0 | 2 |
|  | **health insurance member** | - | 0 | 1 | 0 | 1 |
|  | **military veterans** | - | 2 | 1 | 1 | 4 |
|  | **mixed** | - | 1 | 1 | 0 | 2 |
|  | **volunteer** | - | 0 | 2 | 0 | 2 |
|  |  |  |  |  |  |  |
| **Any proxy use** |  | - | 1 | 4 | 0 | 5 |
|  |  |  |  |  |  |  |
| **Any major study weakness** |  | - | 0 | 2 | 1 | 3 |
|  |  |  |  |  |  |  |
| **Emphysema definition** | **EM based on visual comparison** | - | 1 | 9 | 0 | 10 |
|  | **EM diagnosed** | - | 0 | 7 | 1 | 8 |
|  | **EM deaths (with ICD code)** | - | 5 | 0 | 1 | 6 |
|  | **EM self-reported** | - | 0 | 2 | 0 | 2 |
|  | **other EM** | - | 1 | 1 | 0 | 2 |
|  |  |  |  |  |  |  |
| **Diagnostic criteria** | **ICD** | - | 4 | 0 | 1 | 5 |
|  | **unspecified** | - | 3 | 18 | 1 | 22 |
|  | **Japanese Respiratory Society** | - | 0 | 1 | 0 | 1 |
|  |  |  |  |  |  |  |
| **ICD7 codes** | **527.1** | - | 4 | - | 1 | 5 |
|  |  |  |  |  |  |  |
| **ICD 9 codes** | **492** | - | 1 | - | - | 1 |
|  |  |  |  |  |  |  |
| **Analysis type for asthma** | **include irrespective of asthma** | - | 6 | 18 | 1 | 25 |
|  | **excluding all asthmatics** | - | 1 | 1 | 1 | 3 |
|  |  |  |  |  |  |  |
| **Lung function criteria** | **FEV1/FVC <70%** | - | 0 | 1 | - | 1 |
|  | **FEV1 or DLCO <80% predicted** | - | 1 | 0 | - | 1 |
|  |  |  |  |  |  |  |
| **Spirometry with bronchodilator** | **without/pre/ unknown** | - | 1 | 1 | - | 2 |
|  |  |  |  |  |  |  |
| **Source of diagnosis** | **Questionnaire: physician diagnosis** | - | 1 | 7 | 0 | 8 |
|  | **Questionnaire: other** | - | 0 | 2 | 0 | 2 |
|  | **X-ray** | - | 0 | 1 | 0 | 1 |
|  | **CT scan** | - | 1 | 3 | 0 | 4 |
|  | **Death certificate/registry** | - | 5 | 0 | 1 | 6 |
|  | **Autopsy** | - | 0 | 5 | 0 | 5 |
|  | **Mixed** | - | 0 | 1 | 1 | 2 |
|  |  |  |  |  |  |  |
| **Questionnaire** | **none** | - | 6 | 10 | 1 | 17 |
|  | **MRC** | - | 0 | 1 | 1 | 2 |
|  | **NHLI** | - | 1 | 1 | 0 | 2 |
|  | **ATS** | - | 0 | 2 | 0 | 2 |
|  | **Health Omnibus Survey** | - | 0 | 1 | 0 | 1 |
|  | **unnamed/unspecified** | - | 0 | 4 | 0 | 4 |
|  |  |  |  |  |  |  |
| **Type of outcome** | **prevalence** | - | 0 | 19 | 0 | 19 |
|  | **mortality** | - | 5 | 0 | 1 | 6 |
|  | **incidence** | - | 2 | 0 | 1 | 3 |
|  |  |  |  |  |  |  |
| **Number of cases** | **1-100** | - | 4 | 9 | 1 | 14 |
|  | **101-200** | - | 0 | 5 | 0 | 5 |
|  | **201-500** | - | 3 | 2 | 1 | 6 |
|  | **501-1000** | - | 0 | 2 | 0 | 2 |
|  | **>1000** | - | 0 | 1 | 0 | 1 |
|  | Median | - | 57.00 | 101.00 | 220.50 | 96.50 |
|  | Range | - | 2:473 | 6:1384 | 15:426 | 2:1384 |
|  | Median (based on principal studies only) |  |  |  |  | 96.50 |
|  | Range |  |  |  |  | 2:1384 |
|  |  |  |  |  |  |  |
| **Total number of subjects** | **1-100** | - | 1 | 0 | 0 | 1 |
|  | **101-200** | - | 0 | 3 | 0 | 3 |
|  | **201-500** | - | 0 | 1 | 1 | 2 |
|  | **501-1000** | - | 0 | 3 | 0 | 3 |
|  | **>1000** | - | 6 | 12 | 1 | 19 |
|  | Median | - | 78000.00 | 1476.00 | 124327.50 | 2433.00 |
|  | Range | - | 66:1003229 | 164:72284 | 460:248195 | 66:1003229 |
|  | Median (based on principal studies only) |  |  |  |  | 2433.00 |
|  | Range |  |  |  |  | 66:1003229 |
|  |  |  |  |  |  |  |
| **Total number of adjustment factors used** | **none** | - | 1 | 8 | 1 | 10 |
|  | **1** | - | 4 | 7 | 1 | 12 |
|  | **2** | - | 1 | 1 | 0 | 2 |
|  | **3** | - | 0 | 2 | 0 | 2 |
|  | **6** | - | 1 | 1 | 0 | 2 |
|  |  |  |  |  |  |  |
| **Adjusted for :^e^** |  |  |  |  |  |  |
| **sex** |  | - | 1 | 4 | 0 | 5 |
| **age** |  | - | 6 | 10 | 1 | 17 |
| **location within study** |  | - | 0 | 2 | 0 | 2 |
| **aspects of study design** |  | - | 1 | 0 | 0 | 1 |
| **SES/education** |  | - | 0 | 3 | 0 | 3 |
| **other medical history/symptoms (4)** |  | - | 1 | 0 | 0 | 1 |
| **diet/alcohol (2)** |  | - | 0 | 1 | 0 | 1 |
|  |  |  |  |  |  |  |
| **Smoking results available for Emphysema:** |  |  |  |  |  |  |
| **for current vs never** |  | - | 5 | 10 | 2 | 17 |
| **for ex vs never** |  | - | 3 | 9 | 2 | 14 |
| **for ever vs never** |  | - | 3 | 17 | 2 | 22 |
| **for current vs non (as well as curr vs never)** |  | - | 1 | 8 | 2 | 11 |
| **amount smoked** |  | - | 4 | 6 | 2 | 12 |
| **age started smoking** |  | - | 1 | 0 | 0 | 1 |
| **pack-years** |  | - | 1 | 2 | 0 | 3 |
| **duration of smoking** |  | - | 2 | 1 | 0 | 3 |
| **duration of quitting (vs never)** |  | - | 1 | 1 | 0 | 2 |
| **duration of quitting (vs current)** |  | - | 0 | 0 | 0 | 0 |

^a^ All studies are counted in the first section of the Table. In subsequent sections, studies with at least one relative risk for each of the disease outcomes are counted; outcome-specific fields (including description of the outcome definition and availability of results for the major and dose-related smoking indices) are included only in those sections. The availability of results for other disease definitions and stratified results, as well as the number of relative risks per study, are included only in the first section

^b^ Principal studies are categorized as CC (case-control, including nested CC), Prosp (prospective) or CrossSec (cross-sectional). Subsidiary studies are categorized separately.

^c^ For presence/absence variables, the “Levels” column is left blank and counts for only the “presence” level are shown. “Not applicable” levels are not shown (e.g. for variables specific to case-control studies)

^d^ "Results also available for other disease definitions" refers to all disease definitions qualifying for this project but not entered, with the exception of different levels of severity of COPD which are counted separately (see following variable “Results also available by severity of COPD”)

^e^ Factors adjusted for – by 1 variable except where shown in brackets.

## Table 4 Study populations and exclusions

| **REF** | **Population** | **Medical exclusions^a^** | **Other exclusions** |
| --- | --- | --- | --- |
|  |  |  |  |
| ALDERS | all (general population) |  |  |
| ALESSA | all (general population) | Pulmonary embolism, metabolic acidosis, cancer, acute inflammatory, immunological, renal or peripheral vascular disease, thrombosis, hypertension, diabetes, antiplatelet/anticoagulant drugs in last 15d, hematocrit>50% |  |
| AMIGO | all (general population) | History of tuberculosis, silicosis, cancer, asthma, other respiratory disease |  |
| ANDER1 | all (general population) |  |  |
| ANDER2 | all (general population) |  |  |
| ANDER3 | all (general population) |  |  |
| AUERBA | all (general population) |  | Snuff users |
| BANG | Hispanics |  |  |
| BECK1 | all (general population) | Died during subsequent 6-year follow-up | Non whites, never smokers who started smoking pipe/cigar only during subsequent 6-year follow-up |
| BECK2 | all (general population) | Died during follow-up | Non-whites, baseline smokers who started smoking pipe/cigars only during follow-up |
| BEDNAR | all (general population) | Asthma | Non-Caucasian |
| BEST | military veterans | Long-term hospitalization | Additionally includes a few non-veterans who received Canadian war veteran pensions |
| BJORNS | all (general population) |  |  |
| BROGGE | all (general population) | AATD, previous chronic lung disease, dementia, HIV, hepatitis | Non-Caucasian |
| BROWN | all (general population) |  |  |
| CERVER | all (general population) |  |  |
| CHAPMA | parents |  | Living >2 miles from air monitoring station |
| CHEN1 | workers at 11 factories |  |  |
| CHEN2 | household members |  | Residents of Indian reserves and remote areas of Quebec and Ontario |
| CHEN3 | household members | COPD, heart disease or rheumatoid arthritis diagnosed before the age of 25 years | Residents of Indian reserves and remote areas |
| CHENG | all (general population) | Cardiopulmonary disease (identified by electrocardiogram and chest slides |  |
| CLEMEN | Air Force personnel |  | Subjects with fewer than 3 measurements of lung function during study period |
| COATES | Post Office employees |  |  |
| COCCI | all (general population) | **a** |  |
| COLLEG | all (general population) |  |  |
| DEAN1 | all (general population) |  |  |
| DEAN2 | all (general population) |  |  |
| DEANE | telephone company employees |  |  |
| DEJONG | all (general population) |  |  |
| DEMARC | all (general population) |  |  |
| DETORR | clinic or GP patients^b^ | Positive bronchodilator response |  |
| DICKIN | all (general population) | Died within 12 month study period. Asthma or other restrictive disease |  |
| DOLL1 | doctors |  |  |
| DOLL2 | doctors |  |  |
| DONTA1 | all (general population) | Died during subsequent follow-up period | Emigrated or made dramatic changes to smoking habits during subsequent follow-up period |
| DONTA2 | all (general population) | Baseline COLD (i.e. CB, EM, asthma or other lung disease). Died during follow-up | Emigrated or made dramatic changes to smoking habits during follow-up |
| DOPICO | outdoor workers for City and power company |  | Spending <50% of work day on functions outdoors |
| EHRLIC | household members |  |  |
| EKBERG | all (general population) | Subjects with self-reported current/previous cancer, stroke, angina pectoris, heart infarction or diabetes at baseline |  |
| ENRIGH | health insurance members | Terminal illness, inability to walk, communicate or give informed consent | Institutionalization, likely to move from area in next 3 years |
| ENSTRO | household members |  | Subjects in institutions or military service, migrant workers and similar subjects who would be difficult to trace, households in which no-one was aged at least 45 years |
| FERRI1 | household members |  | Regularly work away from home |
| FERRI2 | households+long term residents |  | Regularly work away from home |
| FERRI3 | households+long term residents |  | Regularly work away from home |
| FIDAN | coffeehouse or shop workers |  | Restricted to coffeehouse and small shop workers, but excluding current employees in hair salons, barbershops, and bakeries, ex-employees in shoe-making, painting, mining, textile and other occupations with possible respiratory risks |
| FINKLE | military recruits |  | Recruits from states other than Illinois and Indiana |
| FLETCH | men - postmen, women - clerical workers |  |  |
| FORAST | all (general population) | Asthma diagnosed before age 18; asthma (diagnosis as adult) or CB/EM without symptoms in last year | Subjects for whom a proxy interview was obtained |
| FOXMAN | all (general population) | Disabled subjects eligible for Medicare | Subjects in military or various institutions, high income families |
| FUKUCH | household members |  |  |
| GEIJER | all (general population) | Previous lung disease diagnosis or GOLD II at baseline, died or severe illness during followup (5yrs) |  |
| GODTFR | all (general population) |  |  |
| GOLDBE | parents |  | Non-white subjects |
| GULSVI | all (general population) |  |  |
| HAENSZ | nationwide sample plus siblings of migrants to USA still resident in Norway |  |  |
| HAMMO2 | household members |  | Subjects in institutions or military service, migrant workers and similar subjects who would be difficult to trace, households in which no-one was aged at least 45 years |
| HARDIE | all (general population) |  |  |
| HARIKK | all (general population) | Baseline FEV1 <65% predicted, asthma, acute bronchitis, bronchiectasis, baseline diagnosis of COPD (with or without abnormal spirometry) | Non-whites |
| HARRIS | soldiers | Proven pulmonary tuberculosis or bronchial asthma |  |
| HAWTHO | Occupational groups (from industry, not otherwise specified) and census-identified sample |  |  |
| HAYES | parents |  | Prolonged occupational exposure to coal dust, cutting oils, asbestos, mine dust, smelter fumes or raw cotton dust |
| HEDMAN | all (general population) |  |  |
| HIGGI2 | all (general population) | TB, bedridden from stroke, incapable of participating | Subjects who had worked in mining or other dusty occupation |
| HIGGI3 | all (general population) | Recent cardiac infarction |  |
| HIGGI4 | all (general population) |  |  |
| HIGGI6 | all (general population) |  |  |
| HIRAYA | all (general population) |  |  |
| HO | long-term residents |  |  |
| HOLLA2 | telephone company employees | Sickness + absence from work on day of survey |  |
| HOLLNA | all (general population) |  |  |
| HOUSE | parents |  | Lived in community <2 years, occupational exposure to irritating fumes, dusts or aerosols |
| HOZAWA | all (general population) | history of CHD or stroke | race other than white or African American |
| HRUBEC | military veterans and twins |  | Non-whites |
| HUCHON | household members |  |  |
| HUHTI1 | all (general population) | TB and "other" respiratory diseases |  |
| HUHTI2 | long-term residents | TB and other respiratory diseases, excluding asthma and emphysema | Started or restarted smoking in last 10 years |
| HUHTI3 | all (general population) | TB and other respiratory diseases, but not emphysema or asthma |  |
| ITABAS | all (general population) | Asthma |  |
| JACOBS | all (general population) |  |  |
| JAENDI | clinic or GP patients^b^ |  |  |
| JENSEN | clinic or GP patients^b^ | Airway infection within previous 2 weeks, asthma with at least 20% increase in FEV1 after terbutaline inhalation |  |
| JINDA2 | household members |  |  |
| JOHANN | long-term residents |  |  |
| JOSHI | employees at machine tool factory and woollen hosiery mill |  |  |
| JOUSI1 | all (general population) | Reported history of MI or stroke before survey |  |
| KACHEL | workers at 5 factories |  |  |
| KAHN | military veterans |  | Non-whites |
| KAHN2 | military veterans |  | Non-whites |
| KARAKA | all (general population) | History of heart disease, asthma or episodic wheezing in childhood or adulthood, or other respiratory diseases (e.g. bronchiectasis, TB) |  |
| KATANC | whites from Medicare, blacks from general population | Difficulty in walking quarter of mile, climbing 10 steps without resting or performing basic activities of daily living, need of assistive walking device, active treatment for cancer in previous 3 years | Planning to move out of study area in next 3 years |
| KATO | all (general population) |  |  |
| KHOURY | Relatives of COPD cases (cases having been identified through Johns Hopkins Hospital respiratory laboratory), relatives of lung cancer and non-pulmonary patients, or community-based samples (neighbours and teachers) |  | Non-whites |
| KIM | household members |  |  |
| KIRAZ | rural group using biomass cookers and urban group using fuel oil |  |  |
| KLAYTO | employees at 2 research facilities | Subjects with MZ phenotype |  |
| KOJIMA | all (general population) | Asthma and TB |  |
| KOTAN1 | all (general population) |  |  |
| KOTAN2 | all (general population) |  |  |
| KRZYZA | all (general population) | Died during study period |  |
| KUBIK | all (general population) |  |  |
| KULLER | self-selected volunteers |  |  |
| LAI | all (general population) |  |  |
| LAM1 | employees in a machine factory |  |  |
| LAM2 | military veterans |  |  |
| LAM3 | military veterans | COPD at baseline |  |
| LAMBER | household members |  | Subjects with unknown daily smoking amount, and occasional smokers |
| LANGE | all (general population) |  |  |
| LANGE2 | all (general population) |  |  |
| LANGHA | all (general population) |  |  |
| LAVECC | household members |  |  |
| LEBOWI | household members |  | Non-whites and Mexican-Americans, subjects with <6 months full time employment history and men with occupational exposure for COPD endpoint only |
| LEE | siblings of migrants |  | Restricted to siblings of migrants to USA |
| LIAW | self-selected volunteers | Previous history cancer or major diseases (eg cardiovascular disease, diabetes mellitus, hypertension) |  |
| LINDBE | long-term residents | Died during follow-up | Subjects born outside the years 1919-20, 1934-5 and 1949-50 |
| LINDST | all (general population) |  |  |
| LIU1 | all (general population) |  |  |
| LIU2 | all (general population) | neurological disease, serious arteriosclerosis, chest or eye surgery in last 2 months, detached retina, acute respiratory infection |  |
| LUNDB1 | long-term residents |  | Subjects born outside the years 1919-20, 1934-5 and 1949-50 |
| LUNDB2 | all (general population) | **a** |  |
| MADOR | military veterans | Significant underlying disease**^a^** |  |
| MAGNUS | all (general population) |  | Restricted to subjects aged 50 and 80 years |
| MANFRE | all (general population) |  | Exposure to grain in farming |
| MANNI1 | all (general population) | Recent chest/abdominal surgery, heart attack, angina or congestive heart failure | Subjects who classified their race as neither black nor white |
| MANNI2 | household members |  |  |
| MANNI3 | household members | Subjects with COPD (GOLD) |  |
| MARAN1 | all (general population) | Upper respiratory tract infection on day of study |  |
| MARAN2 | all (general population) | Upper respiratory tract infection on day of study, died during study period |  |
| MARCUS | Japanese ancestry |  |  |
| MATHES | all (general population) | **a** |  |
| MELLST | all (general population) |  |  |
| MENEZ1 | household members |  |  |
| MENEZ2 | household members | Mental disease,thoracic/abdominal surgery,MI,eye surgery, hospitalization for any cardiac problem, current TB, pregnancy, heart rate >120 beats/minute |  |
| MENEZ3 | household members | Mental disease,thoracic/abdominal surgery,MI,eye surgery, hospitalization for any cardiac problem, current TB, pregnancy, heart rate >120 beats/minute |  |
| MENEZ4 | household members | Mental disease,thoracic/abdominal surgery,MI,eye surgery, hospitalization for any cardiac problem, current TB, pregnancy, heart rate >120 beats/minute |  |
| MENEZ5 | household members | Mental disease,thoracic/abdominal surgery,MI,eye surgery, hospitalization for any cardiac problem, current TB, pregnancy, heart rate >120 beats/minute |  |
| MENEZ6 | household members | Mental disease,thoracic/abdominal surgery,MI,eye surgery, hospitalization for any cardiac problem, current TB, pregnancy, heart rate >120 beats/minute |  |
| MEREN | all (general population) |  |  |
| MILLER | household members |  | Nonwhite subjects |
| MILNE | all (general population) |  |  |
| MOLLER | all (general population) | **a** |  |
| MONTNE | all (general population) |  |  |
| MUELLE | household members |  |  |
| NAWA | healthy workers/retired persons |  |  |
| NEJJAR | household members |  |  |
| NIEPSU | all (general population) |  |  |
| NIHLEN | long-term residents | Died during follow-up | Moved away during follow-up |
| NILSSO | all (general population) |  |  |
| OGILVI | household members |  |  |
| OMORI | all (general population) | Asthma or other pulmonary diseases |  |
| OSWAL1 | general clinic patients, and civil servants referred after repeated sickness due to bronchitis | **a** |  |
| OSWAL2 | civil servants | Absent from work due to sickness | Away on leave or temporary duty |
| PANDEY | all (general population) |  | Working away from home |
| PEAT | all (general population) | History of asthma, died during study period |  |
| PELKON | all (general population) | Subjects with asthma, TB, bronchiectasis, pulmonary fibrosis and thorax deformity were excluded from CB analysis |  |
| PEREZP | all (general population) | Asthma, bronchiectasis or any respiratory diagnosis other than CB or CAO. Radiographic abnormalities |  |
| PETO | transport and clerical workers, and census-identified sample |  |  |
| PRATT | military veterans |  | Subjects who worked in cotton textile industry |
| PRICE | all (general population) | Non-obstructive lung disease (eg sarcoidosis, TB, lung cancer), acute symptoms suggestive of unstable heart disease |  |
| REID | general group, and migrants from UK and Norway |  | Non-whites from native-born sample |
| RENWIC | all (general population) | History of ischaemic heart disease, currently receiving beta blockers, anticholinergic medications or oral steroids, evidence of myocardial ischaemia on ECG, housebound, confused or unsuitable for other reasons according to GP | Non-white subjects |
| RICCIO | clinic or GP patients^b^ | Asthma, bronchiectasis, interstitial lung disease, acute sinusitis, pregnancy, gastro-oesophageal reflux, positive reversibility test |  |
| RIMING | self-selected volunteers | Disease or abnormality detected on chest x-ray thought likely to be cause of sputum e.g. certain tuberculous disease, lung cancer and bronchiectasis |  |
| RYDER | all (general population) |  | Coal miners |
| SARGEA | all (general population) |  |  |
| SAWICK | all (general population) |  |  |
| SCHWAR | household members |  | Subjects in the military services or who resided in institutions |
| SHAHAB | all (general population) |  |  |
| SHARP | clerical and light assembly workers at power company |  |  |
| SHIMUR | all (general population) | **a** |  |
| SHIN | household members |  |  |
| SICHLE | all (general population) | Lung injury or operation, cardiac disease, TB, asthma or pulmonary neoplasms |  |
| SILVA | household members | Subjects with CB/EM at baseline, or those who underwent heart or lung surgery before or during study period, subjects with concurrent CB and emp for CB analyses only | Non-whites and Mexican-Americans |
| SOBRAD | all (general population) |  |  |
| SPEIZE | household members |  | Non-white subjects |
| STERLI | all (general population) |  | Non-civilian and institutionalised subjects from living sample |
| STJERN | all (general population) |  | Employees at sulphite pulp factory |
| STROM | long-term residents |  |  |
| SUADIC | Armed Forces, Customs service, railway, telephone, post, banking and construction companies |  |  |
| SUTINE | all (general population) |  |  |
| TAGER | all (general population) |  |  |
| TAGER2 | household members |  |  |
| TANG | businessmen/professionals, civil servants, general population from socially deprived area, industrial workers |  |  |
| THUN | household members |  | Subjects in institutions or military service, migrant workers or similar who would be difficult to trace, households where all members were aged <45 years |
| TODD | household members |  |  |
| TROISI | nurses | Physician's diagnosis of chronic bronchitis or emphysema before 1980, or diagnosis of cancer except nonmelanoma skin cancer, cardiovascular disease or diabetes at each biennial examination, or any diagnosis of asthma |  |
| TRUPIN | telephone subscribers |  |  |
| TSUSHI | self-selected volunteers | asthma, bronchiectasis, panbronchiolitis, TB |  |
| TVERDA | all (general population) |  | Users of cigars only or other combinations of smoking products, or missing smoking data |
| URRUTI | all (general population) | Asthma related symptoms (woken in night due to lack of air or asthma attack in last 12 months or taking asthma medication) |  |
| VESTBO | all (general population) | Self-reported asthma (at baseline or follow-up) |  |
| VIEGI1 | household members |  |  |
| VIEGI2 | all (general population) |  |  |
| VIKGRE | long-term residents | Presence of any pulmonary disease requiring medical attention, history of congestive heart failure or unstable angina pectoris at baseline, presence of any other severe disease at baseline or follow-up, died during follow-up (although no deaths were from COPD), emphysematous lesions at baseline | Quit smoking before examination or moved away during study period |
| VINEIS | self-selected volunteers |  |  |
| VOLLM1 | self-selected volunteers |  |  |
| VOLLM2 | self-selected volunteers |  |  |
| VONHER | all (general population) |  |  |
| WAGEN2 | heterogeneous population of employees from different companies and organisations | Asthma**^a^** | Subjects who worked less than 16 hours/week, temporary workers |
| WALD | professional/business men |  | Restricted to men with NHS numbers |
| WANG2 | self-selected volunteers |  |  |
| WATSON | all (general population) | History of IHD, diabetes, gastrointestinal disease**^a^** | Smoked <=10 pack years |
| WEISS | self-selected volunteers |  |  |
| WEN | community cohort were volunteers invited for screening and comprised 25% of population in study areas; other cohort were civil servants and teachers in government employee insurance scheme | Previous history of major disease (eg cardiovascular diseases, diabetes mellitus, or hypertension) for community cohort only |  |
| WIG | household members |  |  |
| WILHEL | all (general population) | Participants in 1963 survey who died before 1967 |  |
| WILSO1 | household members |  |  |
| WILSO2 | household members |  | Residents of hotels, motels, hospitals, nursing homes and other institutions |
| WOJTYN | all (general population) |  | Subjects who changed smoking habit during study |
| WOODS | all (general population) |  |  |
| WOOLF | employees of large commercial firms | Asthma, heart disease, obesity or chest wall deformities |  |
| XIAO | all (general population) |  |  |
| XU | long-term residents |  |  |
| YAMAGU | long-term residents |  | 50 "unreliable" subjects |
| YUAN | all (general population) | History of cancer |  |
| ZIELI1 | self-selected volunteers |  |  |
| ZIELI2 | self-selected volunteers | History of asthma, bronchiectasis, TB |  |
| ZIETKO | all (general population) | Patients treated with inhaled or systemic corticosteroids during the past 3 months**^a^** |  |
| ZOIA | all (general population) |  | Did not consent to 7-day dietary assessment (65% of original sample) |

^a^ For case-control studies, the criteria shown apply to the cases. Additional criteria for the controls, if any, are as follows:

| **COCCI** | Normal LF. No controls reported history of lung disease or occupational exposure |
| --- | --- |
| **LUNDB2** | No self-reported asthma, CB or respiratory symptoms suggestive of CB or asthma |
| **MADOR** | Normal LF |
| **MATHES** | Normal LF and no respiratory symptoms |
| **MOLLER** | Normal LF, no respiratory or cardiovascular disease |
| **OSWAL1** | No pulmonary disease |
| **SHIMUR** | No respiratory disease |
| **WAGEN2** | No respiratory symptoms |
| **WATSON** | Normal LF and no lung disease in GP records |
| **ZIETKO** | No chronic or recurrent respiratory symptoms, no past history of significant pulmonary disease |

^b^ Studies with population "clinic or GP patient" have also been marked as having a study weakness (see Table 2 of paper)

## Table 5 Disease outcomes - summary

Study REFs are listed in columns corresponding to the sections in Table 6, where more detail is given.

| **COPD** | | | | | | | |
| --- | --- | --- | --- | --- | --- | --- | --- |
| **A** | **B** | **C** | **D** | **E** | **F** | **G** | **H** |
| **ICD^a^** | **LF only** | **LF+ symptoms** | **COPD undefined** | **COPD, CB, Emp** | **CB, Emp** | **CB, Emp, Asthma** | **Other** |
| BEST | ANDER3 | ALESSA | HO | AMIGO | CHEN2 | HARDIE | ANDER1 |
| CHEN1 | BEDNAR | COCCI | LAM2 | CHEN3 | FORAST | HUHTI1 | DONTA1 |
| DEAN1 | BROGGE | KIRAZ | XIAO | KARAKA | LINDST |  | FERRI1 |
| DOLL1 | CHENG | SILVA |  | NIHLEN | MONTNE |  | FERRI2 |
| DOLL2 | CLEMEN |  |  | TRUPIN | VOLLM1 |  | FERRI3 |
| ENSTRO | DEJONG |  |  |  | VONHER |  | FIDAN |
| GODTFR**^a^** | DEMARC |  |  |  | XU |  | GULSVI |
| HAMMO2 | DETORR |  |  |  |  |  | HARIKK |
| HAWTHO | DICKIN |  |  |  |  |  | HEDMAN |
| HIGGI4 | EKBERG |  |  |  |  |  | MARAN1 |
| JACOBS | FUKUCH |  |  |  |  |  | MARAN2 |
| KAHN | GEIJER |  |  |  |  |  | PELKON |
| KAHN2 | HOZAWA |  |  |  |  |  | SARGEA |
| KULLER | HUHTI2 |  |  |  |  |  | SICHLE |
| LAM1 | HUHTI3 |  |  |  |  |  |  |
| LAM3 | ITABAS |  |  |  |  |  |  |
| LANGE | JAENDI |  |  |  |  |  |  |
| LEE | JOHANN |  |  |  |  |  |  |
| LIAW | KACHEL |  |  |  |  |  |  |
| LIU1 | KATANC |  |  |  |  |  |  |
| MANNI3 | KHOURY |  |  |  |  |  |  |
| MARCUS | KIM |  |  |  |  |  |  |
| NILSSO | KLAYTO |  |  |  |  |  |  |
| PETO | KOJIMA |  |  |  |  |  |  |
| SPEIZE | KOTAN2 |  |  |  |  |  |  |
| STERLI | KRZYZA |  |  |  |  |  |  |
| TANG | LAI |  |  |  |  |  |  |
| THUN | LEBOWI |  |  |  |  |  |  |
| TODD | LINDBE |  |  |  |  |  |  |
| TVERDA | LIU2 |  |  |  |  |  |  |
| VINEIS | LUNDB1 |  |  |  |  |  |  |
| VOLLM2 | MADOR |  |  |  |  |  |  |
| WALD | MANNI1 |  |  |  |  |  |  |
| WEN | MANNI2 |  |  |  |  |  |  |
| YUAN | MATHES |  |  |  |  |  |  |
|  | MENEZ2 |  |  |  |  |  |  |
|  | MENEZ3 |  |  |  |  |  |  |
|  | MENEZ4 |  |  |  |  |  |  |
|  | MENEZ5 |  |  |  |  |  |  |
|  | MENEZ6 |  |  |  |  |  |  |
|  | MUELLE |  |  |  |  |  |  |
|  | NIEPSU |  |  |  |  |  |  |
|  | PEAT |  |  |  |  |  |  |
|  | PEREZP |  |  |  |  |  |  |
|  | PRICE |  |  |  |  |  |  |
|  | RENWIC |  |  |  |  |  |  |
|  | RICCIO |  |  |  |  |  |  |
|  | SAWICK |  |  |  |  |  |  |
|  | SHAHAB |  |  |  |  |  |  |
|  | SHIN |  |  |  |  |  |  |
|  | STROM |  |  |  |  |  |  |
|  | TAGER |  |  |  |  |  |  |
|  | TSUSHI |  |  |  |  |  |  |
|  | VESTBO |  |  |  |  |  |  |
|  | VIEGI2 |  |  |  |  |  |  |
|  | WATSON |  |  |  |  |  |  |
|  | WEISS |  |  |  |  |  |  |
|  | WILSO1 |  |  |  |  |  |  |
|  | WOJTYN |  |  |  |  |  |  |
|  | YAMAGU |  |  |  |  |  |  |
|  | ZIELI1 |  |  |  |  |  |  |
|  | ZIELI2 |  |  |  |  |  |  |
|  | ZIETKO |  |  |  |  |  |  |

| **CB** | | | | |
| --- | --- | --- | --- | --- |
| **I** | **J** | **K** | **L** | **M** |
| **ICD^a^** | **Diagnosis** | **Self-report** | **Symptoms** | **Other** |
| BEST | ALDERS | HO | ANDER1 | JENSEN |
| DOLL1 | BANG | KATO | BECK1 | KIRAZ |
| DOLL2 | BROWN | LAVECC | BECK2 | LUNDB2 |
| HIRAYA | COLLEG |  | BJORNS | MELLST |
| KAHN | DONTA2 |  | CERVER | PELKON |
| KAHN2 | ENRIGH |  | CHAPMA | SHIMUR |
| WEN | HARDIE |  | COATES | SILVA |
|  | KOTAN1 |  | DEAN2 | WIG |
|  | LEBOWI |  | DEANE |  |
|  | MILLER |  | DEMARC |  |
|  | SCHWAR |  | DOPICO |  |
|  | TROISI |  | EHRLIC |  |
|  | VIEGI1 |  | FERRI1 |  |
|  | WILSO2 |  | FINKLE |  |
|  |  |  | FLETCH |  |
|  |  |  | FOXMAN |  |
|  |  |  | GOLDBE |  |
|  |  |  | HAENSZ |  |
|  |  |  | HARRIS |  |
|  |  |  | HAWTHO |  |
|  |  |  | HAYES |  |
|  |  |  | HIGGI2 |  |
|  |  |  | HIGGI3 |  |
|  |  |  | HIGGI6 |  |
|  |  |  | HOLLA2 |  |
|  |  |  | HOLLNA |  |
|  |  |  | HOUSE |  |
|  |  |  | HRUBEC |  |
|  |  |  | HUCHON |  |
|  |  |  | HUHTI1 |  |
|  |  |  | HUHTI2 |  |
|  |  |  | HUHTI3 |  |
|  |  |  | JINDA2 |  |
|  |  |  | JOSHI |  |
|  |  |  | JOUSI1 |  |
|  |  |  | KUBIK |  |
|  |  |  | LAMBER |  |
|  |  |  | LANGE2 |  |
|  |  |  | LANGHA |  |
|  |  |  | LINDST |  |
|  |  |  | MAGNUS |  |
|  |  |  | MANFRE |  |
|  |  |  | MENEZ1 |  |
|  |  |  | MEREN |  |
|  |  |  | MILNE |  |
|  |  |  | MOLLER |  |
|  |  |  | MUELLE |  |
|  |  |  | NEJJAR |  |
|  |  |  | OGILVI |  |
|  |  |  | OSWAL1 |  |
|  |  |  | OSWAL2 |  |
|  |  |  | PANDEY |  |
|  |  |  | PEREZP |  |
|  |  |  | REID |  |
|  |  |  | RIMING |  |
|  |  |  | SAWICK |  |
|  |  |  | SHARP |  |
|  |  |  | SOBRAD |  |
|  |  |  | STJERN |  |
|  |  |  | SUADIC |  |
|  |  |  | TAGER2 |  |
|  |  |  | URRUTI |  |
|  |  |  | WAGEN2 |  |
|  |  |  | WILHEL |  |
|  |  |  | WOJTYN |  |
|  |  |  | WOODS |  |
|  |  |  | WOOLF |  |
|  |  |  | YAMAGU |  |
|  |  |  | ZOIA |  |

| **Emphysema** | | | | |
| --- | --- | --- | --- | --- |
| **N** | **O** | **P** | **Q** | **R** |
| **ICD^a^** | **Visual Comparison** | **Diagnosis** | **Self-report** | **Other** |
| BEST | ANDER2 | DONTA2 | HO | HUHTI1 |
| HAMMO2 | AUERBA | ENRIGH | LAVECC | SILVA |
| HIRAYA | NAWA | GULSVI |  |  |
| KAHN | OMORI | HARDIE |  |  |
| KAHN2 | PRATT | HOZAWA |  |  |
| WEN | RYDER | LEBOWI |  |  |
|  | SUTINE | MILLER |  |  |
|  | VIKGRE | WILSO2 |  |  |
|  | WANG2 |  |  |  |
|  | WEISS |  |  |  |

^a^ Outcomes based on ICD codes are mortality studies, with the exception of GODTFR (COPD) which is incidence.

## Table 6 Disease outcomes – detailed

Sections in this table correspond to the columns in Table 5, and footnotes are given at the end of each section.

## Table 6A : COPD based on ICD

| **REF** | **Outcome type** | **ICD7^a^** | **ICD8 ^a^** | **ICD9 ^a^** | **ICD10 ^a^** | **Defn incl asthma** | **DIAGSC ^a^** | **Description of disease** | **Source of diagnostic information** |
| --- | --- | --- | --- | --- | --- | --- | --- | --- | --- |
|  |  |  |  |  |  |  |  |  |  |
| BEST | mortality | 3 | - | - | - | No | c | Bronchitis/emphysema mortality (ICD7 500-02,527.1) | Death records of Dept of Veterans Affairs |
| CHEN1 | mortality | - | - | 6 | - | Yes | Mixed | COPD : ICD 9 490-496 | Death certificates (supplemented if necessary by hospital records and inquiry of family members and factory medical staff) |
| DEAN1 | mortality | - | 6 | - | - | Yes | c | Chronic bronchitis (sic): ICD 8 490-493 | Death certificates |
| DOLL1 | mortality | 9 | - | 9 | - | Unkn | c | COLD: ICD 7 or ICD 9 (underlying cause, codes unspecified but includes chronic bronchitis and emphysema) | Death certificates |
| DOLL2 | mortality | 9 | - | - | - | Unkn | c | Chronic bronchitis and emphysema: ICD 7 (codes unspecified) | Death certificates |
| ENSTRO | mortality | 5 | 6 | 6 | - | Yes | c | COPD (ICD 7: 241, 500-2, 527.1; ICD 8: 490-3; ICD 9: 490-6) | Death certificates |
| GODTFR | incidence | - | 2 | - | 2 | No | r | First hospitalisation for COPD (ICD 8: 490-492 or ICD 10: J40-J44) | Hospital admission and discharge diagnoses |
| HAMMO2 | mortality | 3 | - | - | - | No | c | COPD: ICD 7 500-502, 527.1 | Death certificates |
| HAWTHO | mortality | - | 6 | - | - | Yes | c | Chronic bronchitis (sic) (ICD 8: 490-493) | Mortality registry |
| HIGGI4 | mortality | - | - | 9 | - | Unkn | c | Underlying or contributing COPD (ICD 9: codes unspecified. Probably CB,EM,COPD and asthma as shown in Table 3 of HIG GIN1989) | Death certificates |
| JACOBS | mortality | - | 9 | - | - | Unkn | Mixed | COPD (ICD 8: codes unspecified) | Death certificates, medical records, interview with physicians and relatives of deceased |
| KAHN | mortality | 2 | - | - | - | No | c | Underlying COPD (ICD 7:501-502,527.1 from 1954-68; 501-502, 527.1, 527.2 from 1969-80). ICD code 500 (acute bronchitis) was additionally included only in dose-related analyses of current smoking by amount smoked | Death certificates |
| KAHN2 | mortality | 3 | - | - | - | No | c | Underlying COPD (ICD 7: 500-502, 527.1) | Death certificates |
| KULLER | mortality | - | - | 6 | - | Yes | c | COPD (ICD9 490-496) underlying cause of death | Death certificates |
| LAM1 | mortality | - | - | 6 | - | Yes | Mixed | COPD (ICD 9: 490-496) | Medical records, death certificates |
| LAM3 | mortality | - | - | - | 9 | Unkn | r | COPD (ICD 10, codes unspecified) | Hospital records |
| LANGE | mortality | - | 2 | - | - | No | c | COPD underlying or contributing mortality (490-2) | Death register |
| LEE | mortality | 6 | - | - | - | No | c | CNSLD (ICD 7 502=CB, 526=Bronchiectasis, 527.1= emphysema) | Death certificate |
| LIAW | mortality | - | - | 9 | - | Unkn | c | ICD9-COPD (codes unspecified) | Computerized national mortality register |
| LIU1 | mortality | - | - | 3 | - | No | Mixed | COPD (ICD 9):ICD 9 (490, 491, 492, 496 and 416.7) | Local administrative records, supplemented by medical records and discussions with health workers and family |
| MANNI3 | mortality | - | - | 6 | - | Yes | c | COPD (ICD 9: 490-496) as underlying cause | Death certificates |
| MARCUS | mortality | - | 8 | - | - | Yes | c | COPD (ICD 8: 490-493, 518, 519.3) underlying or contributory. (ICD8 519.3 is defined by MARCUS as chronic obstructive disease not elsewhere classified, although this code is not listed in the ICD8 Manual) | Hospital records, death certificates |
| NILSSO | mortality | - | 2 | - | - | No | c | Bronchitis and emphysema (ICD 8: 490-492) | Death certificates |
| PETO | mortality | - | 4 | - | - | No | c | COPD (ICD 8: 490-492,519) | Death certificates |
| SPEIZE | mortality | - | - | 6 | - | Yes | c | COPD (ICD 9: 490-496) | Death certificates |
| STERLI | mortality | - | - | 2 | - | No | c | COPD (ICD 9: 490-492, 496) | Death certificates |
| TANG | mortality | - | a | - | - | No | c | chronic obstructive lung disease ICD-8 491-492,519 | Death certificates |
| THUN | mortality | - | - | 2 | - | No | c | COPD (ICD 9: 490-492, 496) | Death certificates |
| TODD | mortality | 6 | - | - | - | No | c | CNSLD (ICD 7 502=CB, 526=Bronchiectasis, 527.1= emphysema) | Death certificates |
| TVERDA^b^ | mortality | - | 7 | a | - | Yes | c | Asthma, bronchitis and emphysema (underlying, ICD 8, 466, 490-493; ICD 9: 466, 490-493) | Death certificates |
| VINEIS | mortality | - | - | - | 9 | Unkn | c | COPD or emphysema (ICD 10, no codes specified) | Cancer and mortality registries |
| VOLLM2 | mortality | - | - | 6 | - | Yes | c | Underlying and contributory COPD (ICD 9: 490-496) | Death certificates |
| WALD | mortality | - | - | 8 | - | No | c | COPD (ICD 9: 416,491,492,496,519) | Mortality register |
| WEN | mortality | - | - | 1 | - | No | c | CB (ICD9 491), Emphysema (ICD9 492) or CAO (ICD9 496) | Computerized national mortality register |
| YUAN | mortality | - | - | 6 | - | Yes | c | COPD (ICD 9: 490-496) | Death certificates |

^a^ Codes are explained at the end of Table 6 (although the information is generally repeated in text form in columns "Description of disease" and " Source of diagnostic information "; the levels are the same for all 3 outcomes).

^b^ For study TVERDA, this COPD outcome was accepted despite lacking the core condition ICD9-496. As the study follow-up period was 1972 to 1988, and Norway adopted the 9th revision in 1986, the 8th revision was in force for the majority of the study. As code 496 only gradually came to be used for COPD, it is unlikely that omission of this code for the final 3 years of the study would have had much impact.

## Table 6B : COPD based on LF (lung function) only

| **REF** | **Outcome type** | **Diagnostic Criteria^a^** | **LUNGFN^b^** | **SPBRON^c^** | **Description of disease** | **Source of diagnostic information** |
| --- | --- | --- | --- | --- | --- | --- |
|  |  |  |  |  |  |  |
| ANDER3 | prevalence | ATS | q | no/unkn | COPD (ATS criteria NOS) | Spirometry |
| BEDNAR | prevalence | BTS | 2 | no/unkn | COPD/airflow limitition (ERS FEV1/FVC<70%) | Spirometry |
| BROGGE | prevalence | GOLD | n | post | COPD (GOLD II postbronc'r FEV1/FVC<70% FEV1<80%pr) | Spirometry |
| CHENG | prevalence | unspec | 2 | no/unkn | FEV1/FVC < 70% | Spirometry |
| CLEMEN | prevalence | unspec | a | no/unkn | Reduced FEV1 and/or VC (<70% predicted at least once and average below normal) Endpoint not originally defined as COPD, but based on FEV1 and/or VC value <70% predicted, with average over the study period also below normal | Spirometry |
| DEJONG | prevalence | GOLD | 2 | no/unkn | Obstructive lung disease (FEV1/FVC <70%) | Spirometry |
| DEMARC | prevalence | GOLD | 2 | no/unkn | COPD (FEV1/FVC <70% = GOLD 2001 I-IV) | Spirometry |
| DETORR | prevalence | GOLD | 2 | post | Post-bronchodilator FEV1/FVC <0.7 (Patients with documented positive bronchodilator response were excluded)) | Spirometry |
| DICKIN | prevalence | Van Schayck | f | revers | COPD (FEV1 <5th centile + reversibility <9%) | Spirometry |
| EKBERG | prevalence | GOLD | 2 | no/unkn | COPD (GOLD criteria FEV1/FVC<70%) | Spirometry |
| FUKUCH | prevalence | GOLD | 2 | no/unkn | Airflow limitation (FEV1/FVC <70%) | Spirometry |
| GEIJER | incidence | GOLD | n | post | Incident GOLD II FEV1/FVC<70% FEV1<80%pred) | Spirometry |
| HOZAWA | prevalence | unspec | p | no/unkn | FEV1 <85% predicted | Spirometry |
| HUHTI2 | prevalence | unspec | 7 | no/unkn | Chronic airflow obstruction (FEV1% < 60) | Spirometry |
| HUHTI3 | prevalence | unspec | 7 | no/unkn | Chronic obstructive lung disease (FEV% < 60) | Spirometry |
| ITABAS | prevalence | ATS^d^ | 5 | no/unkn | COPD (abnormal expiratory flow for several months) | Spirometry |
| JAENDI | prevalence | unspec | n | post | post-bronchodilator FEV1/FVC<70% and FEV1<80%pred | Spirometry |
| JOHANN | prevalence | GOLD | 2 | post | COPD (post-bronchodilator FEV1/FVC<0.7 =GOLD I-IV) | Spirometry |
| KACHEL | prevalence | unspec | n | no/unkn | Airflow limitation (FEV1/FVC <0.70, FEV1 <80%pred) | Spirometry |
| KATANC | prevalence | ATS | 5 | no/unkn | Obstructive airway disease (ATS criteria: reduced FEV1/FVC and FEV1 <100% predicted) | Spirometry |
| KHOURY | prevalence | unspec | k | no/unkn | Airways obstruction (FEV1/FVC <68%) | Spirometry |
| KIM | prevalence | GOLD | 2 | no/unkn | COPD (FEV1/FVC <70%) | Spirometry |
| KLAYTO | prevalence | unspec | h | no/unkn | COPD (FEV1 or MMEF rate <2 SD below predicted) | Spirometry |
| KOJIMA | prevalence | GOLD | 2 | no/unkn | COPD (FEV1/FVC <70%) | Spirometry |
| KOTAN2 | prevalence | GOLD | 2 | post | Post-bronchodilator FEV1/FVC <0.7 | Spirometry |
| KRZYZA | incidence | unspec | b | no/unkn | COPD (FEV1 <65% predicted) | Spirometry |
| LAI | prevalence | GOLD | 2 | no/unkn | prebronchodilator FEV1/FVC <70% | Spirometry |
| LEBOWI | prevalence | unspec | 8 | no/unkn | Predicted FEV1/FVC <80% | Spirometry |
| LINDBE | incidence | GOLD | 2 | no/unkn^e^ | COPD (GOLD modified no bronchodilator)^e^ | Spirometry |
| LIU2 | prevalence | Chinese | 2 | post | post-bronchodilator FEV1/FVC <70% | Spirometry |
| LUNDB1 | prevalence | GOLD | 2 | revers^e^ | COPD (GOLD postbronchodilator FEV1/FVC<70%)^e^ | Spirometry |
| MADOR | prevalence | ATS | 5 | no/unkn | COPD (ATS, FEV1 <70%) | Spirometry |
| MANNI1 | prevalence | unspec | 3 | no/unkn | Low lung function (FEV1/FVC <0.7 and FEV1 <80%) predicted | Spirometry |
| MANNI2 | prevalence | GOLD | 2 | no/unkn | COPD (FEV1/FVC <0.7) | Spirometry |
| MATHES | prevalence | GOLD | n | no/unkn | FEV1/FVC <0.7 and FEV1 <80% predicted | Spirometry |
| MENEZ2 | prevalence | GOLD | 2 | post | COPD (postbronchodilator FEV1/FVC <0.7) | Spirometry |
| MENEZ3 | prevalence | GOLD | 2 | post | COPD (postbronchodilator FEV1/FVC <0.7) | Spirometry |
| MENEZ4 | prevalence | GOLD | 2 | post | COPD (postbronchodilator FEV1/FVC <0.7) | Spirometry |
| MENEZ5 | prevalence | GOLD | 2 | post | COPD (postbronchodilator FEV1/FVC <0.7) | Spirometry |
| MENEZ6 | prevalence | GOLD | 2 | post | COPD (postbronchodilator FEV1/FVC <0.7) | Spirometry |
| MUELLE | prevalence | unspec | l | no/unkn | Chronic airway obstruction (FEV1/FVC < 60%) | Spirometry |
| NIEPSU | prevalence | GOLD | 2 | no/unkn | COPD (GOLD 2001 criteria) | Spirometry |
| PEAT | prevalence | unspec | 6 | no/unkn | Chronic airflow limitation (FEV1/FVC <65% or FEV1 <65% predicted in at least two surveys. If age >75 CAL started before age 75 or also FEV1 <65% predicted) | spirometry |
| PEREZP | prevalence | unspec | 9 | no/unkn | Chronic airway obstruction (FEV1 <75% predicted) | Spirometry |
| PRICE | prevalence | GOLD | 2 | post | Postbronchodilator FEV1/FVC <0.70 | Spirometry |
| RENWIC | prevalence | Enright | g | no/unkn | Chronic airways obstruction (FEV1/FVC <65% for subjects aged <65 years, predicted and lower limit of normal FEV1/FVC calculated for those aged 65+ years by Enright formula) | Spirometry |
| RICCIO | prevalence | GOLD^f^ | 2 | no/unkn | Bronchial obstruction (GOLD criteria)^f^ | Spirometry |
| SAWICK | prevalence | unspec | l | no/unkn | FEV1/FVC <60% | Spirometry |
| SHAHAB | prevalence | Mixed | 2 | no/unkn | ATS/ERS FEV1/FVC <0.7 | Spirometry |
| SHIN | prevalence | ATS | r | no/unkn | Airway obstruction (FEV1/FVC <75%) | Spirometry |
| STROM | prevalence | unspec | 2 | no/unkn | Airflow limitation (FEV1/VC <70%) | Spirometry |
| TAGER | prevalence | unspec | b | no/unkn | Obstructive airways disease (FEV1 <65% predicted) | Spirometry |
| TSUSHI | prevalence | GOLD | 2 | post | Post-beta2 stimulant FEV1/FVC <70% | Spirometry |
| VESTBO | prevalence | GOLD | 2 | no/unkn | COPD (GOLD stage 1+ FEV1/FVC <0.7) | Spirometry |
| VIEGI2 | prevalence | unspec | 2 | no/unkn | COPD ('clinical' criteria FEV1/FVC<70%) | Spirometry |
| WATSON | prevalence | BTS | 3 | no/unkn | BTS (FEV1<=80%pred and FEV1/FVC <=70%pred) | Spirometry |
| WEISS | prevalence | unspec | u | no/unkn | COPD (FEV1 > 2 SE below mean predicted for height) | Spirometry |
| WILSO1 | prevalence | GOLD | 2 | revers | COPD (GOLD FEV1/FVC <70.0%) | Spirometry |
| WOJTYN | prevalence | unspec | 2 | no/unkn | FEV1/FVC<70% at both baseline and 5-year follow-up | Spirometry |
| YAMAGU | prevalence | unspec | k | no/unkn | Airways obstruction (FEV1/FVC <68%) | Spirometry |
| ZIELI1 | prevalence | ERS | 4 | no/unkn | Bronchial obstruction (ERS: FEV1/FVC<85% pred) | Spirometry |
| ZIELI2 | prevalence | GOLD | 2 | no/unkn | Airflow limitation (Gold: FEV1/FVC <0.7) | Spirometry |
| ZIETKO | prevalence | ERS | t | revers | COPD (ERS criteria: FEV1/FVC <70%, FEV1 <80% predicted, <12% reversibility) | Spirometry |

^a^ As named by original author.

^b^ Codes are explained at the end of Table 6 (although the information is generally repeated in text form in columns "Description of disease" and " Source of diagnostic information "; the levels are the same for all 3 outcomes).

^c^ Field SPBRON refers to spirometry used post-bronchodilator, no (=without bronchodilator) or unknown, and reversibility criteria.

^d^ Reference given for definition of disease status relates to ATS document, so it is assumed these criteria were used.

^e^ Although Lundbäck (2003) [185] states that GOLD criteria were applied to values after reversibility test, Lindberg (2006) [180] (describing both baseline and follow-up) states that no reversibility test was performed.

^f^ Bronchodilator was used and reversibility is mentioned but it is not clear how it relates to use of the GOLD criteria.

## Table 6C : COPD based on LF and symptoms

| **REF** | **Outcome type** | **Diagnostic Criteria^a^** | **DIAGSC^b^** | **Questionnaire** | **LUNGFN** | **SPBRON** | **Description of disease** | **Source of diagnostic information** |
| --- | --- | --- | --- | --- | --- | --- | --- | --- |
|  |  |  |  |  |  |  |  |  |
| ALESSA | prevalence | unspec | Mixed | - | 2 | no/unkn | COPD (attending outpatients with CB and irreversible AO, clinically confirmed as COPD and FEV1/FVC < 70% predicted) | Clinical history, physical examination, x-ray, ECG, spirometry |
| COCCI | prevalence | ERS | s | - | 4 | no/unkn | COPD (hospitalized for COPD and FEV1/VC <88% predicted) | Not stated, but spirometry results available |
| KIRAZ | prevalence | unspec | Mixed | MRC/ATS | e | revers | COPD (chronic bronchitis, decreased respiratory sounds or increased expiration time on physical examination, FEV1/FVC <88% predicted and reversibility <12%) | Questionnaire: ATS/MRC, physical examination, spirometry |
| SILVA | incidence | ATS^c^ | Mixed | NHLI | i | no/unkn | COPD (chronic bronchitis (cough/phlegm for 3 months for 2 consecutive years) and/or physician-diagnosed emphysema, and FEV1 or DLCO <80% predicted) | Questionnaire, spirometry |

^a^ As named by original author.

^b^ Codes are explained at the end of Table 6 (although the information is generally repeated in text form in columns "Description of disease" and " Source of diagnostic information "; the levels are the same for all 3 outcomes).

^c^ ATS criteria refers to chronic bronchitis part only.

## Table 6D : COPD not further defined

| **REF** | **Outcome type** | **Diagnostic Criteria^a^** | **DIAGSC^b^** | **Defn incl asthma** | **Description of disease** | **Source of diagnostic information** |
| --- | --- | --- | --- | --- | --- | --- |
|  |  |  |  |  |  |  |
| HO | prevalence | unspec | Mixed | Unkn | COPD (physician diagnosis) | Questionnaire and physician diagnosis |
| LAM2 | prevalence | unspec | q | Unkn | Chronic obstructive pulmonary disease (criteria unspecified) | Questionnaire |
| XIAO | prevalence | unspec | Mixed | Unkn | Hospital patients with COPD (unspecified criteria) | Spirometry, x-ray, CT scan, blood gases |

^a^ As named by original author.

^b^ Codes are explained at the end of Table 6 (although the information is generally repeated in text form in columns "Description of disease" and " Source of diagnostic information "; the levels are the same for all 3 outcomes).

## Table 6E : COPD defined as COPD, CB or Emphysema

| **REF** | **Outcome type** | **Diagnostic Criteria^a^** | **DIAGSC^b^** | **Questionnaire** | **LUNGFN^b^** | **SPBRON^c^** | **Description of disease** | **Source of diagnostic information** |
| --- | --- | --- | --- | --- | --- | --- | --- | --- |
|  |  |  |  |  |  |  |  |  |
| AMIGO | incidence | GOLD^d^ | r | - | 2 | no/unkn | Clinical diagnosis of COPD in last 2 years, GOLD 2001, (said to be equivalent to ICD J44.8-44.9 which includes chronic bronchitis and pulmonary emphysema)^d^ | Clinical diagnosis (unspecified) |
| CHEN3 | prevalence | unspec | q | unnamed/unsp | - |  | COPD (either emphysema, CB or COPD) starting from age 25 | Questionnaire |
| KARAKA | prevalence | Mixed | Mixed | unnamed/unsp | 4 | no/unkn | COPD (ERS criteria=FEV1/VC<88% pred M, 89% pred F) and/or chronic bronchitis (chronic cough and chronic phlegm for 3 months for at least 2 years) and/or physician diagnosed CB, emphysema or COPD) | Questionnaire, spirometry, physician diagnosis |
| NIHLEN | incidence | unspec | d | OLIN | - |  | Chronic bronchitis and/or emphysema and/or COPD | Questionnaire: physician diagnosed |
| TRUPIN | prevalence | unspec | d | unnamed/unsp | - |  | COPD (physician diagnosis of chronic bronchitis, emphysema or COPD) | Questionnaire |

^a^ As named by original author.

^b^ Codes are explained at the end of Table 6 (although the information is generally repeated in text form in columns "Description of disease" and " Source of diagnostic information "; the levels are the same for all 3 outcomes).

^c^ Field SPBRON refers to spirometry used post-bronchodilator, no (=without bronchodilator) or unknown, and reversibility criteria.

^d^ Disease definition is unclear as it mentions both clinical diagnosis and GOLD 2001. It also states that ICD J44.8-44.9 include chronic bronchitis and pulmonary emphysema which they do not.

## Table 6F : COPD defined as CB or Emphysema

| **REF** | **Outcome type** | **Diagnostic Criteria^a^** | **DIAGSC^b^** | **Questionnaire** | **Description of disease** | **Source of diagnostic information** |
| --- | --- | --- | --- | --- | --- | --- |
|  |  |  |  |  |  |  |
| CHEN2 | prevalence | unspec | d | unnamed/unsp | COPD (physician diagnosis of chronic bronchitis or emphysema) | Questionnaire |
| FORAST | prevalence | unspec | d | ATS/NHLBI | Chronic bronchitis/emphysema^c^ | Questionnaire: physician-diagnosed in last year^c^ |
| LINDST | prevalence | unspec | d | FinEsS | Physician diagnosed chronic bronchitis/emphysema | Questionnaire: FinEsS, physician diagnosis |
| MONTNE | prevalence | unspec | q | OLIN | Self-reported chronic bronchitis and/or emphysema | Questionnaire |
| VOLLM1 | prevalence | unspec | d | MRC | Chronic bronchitis and/or emphysema | Questionnaire: physician diagnosed |
| VONHER | prevalence | unspec | Mixed | unnamed/unsp | Examining physician's assessment of CB (=cough and phlegm for at least 3 months for 2 consecutive years excluding other explanations) or emphysema | Screening (questionnaire, spirometry, x-ray) confirmed by clinical examination by physician. (Spirometry only used for severity, not for case status) |
| XU | prevalence | MRC | d | unnamed/unsp | COPD (physician diagnosed chronic bronchitis or emphysema | Questionnaire (verified from medical records) |

^a^ As named by original author.

^b^ Codes are explained at the end of Table 6 (although the information is generally repeated in text form in columns "Description of disease" and " Source of diagnostic information "; the levels are the same for all 3 outcomes).

^c^ The outcome categories as defined originally (adult asthma diagnosed, CB/EM, asthma-like symptoms without diagnosis) were stated to be mutually exclusive, so it is assumed that no subject had both asthma and CB/EM. It is stated that CB/EM diagnosis was in last year, but it seems more likely that this means ever-diagnosed and disease active in last year. Results used refer to CB/EM versus all others. However no information is available on subjects with a history of inactive respiratory disease (diagnosis but no symptoms in last year, 68 subjects) or with childhood asthma (diagnosed before age 18, 18 subjects) so those subjects are omitted.

## Table 6G : COPD defined as CB, Emphysema or AST

| **REF** | **Outcome type** | **Diagnostic Criteria^a^** | **Defn incl asthma** | **DIAGSC^b^** | **Questionnaire** | **Description of disease** | **Source of diagnostic information** |
| --- | --- | --- | --- | --- | --- | --- | --- |
|  |  |  |  |  |  |  |  |
| HARDIE | prevalence | unspec | Yes | d | ATS | Chronic bronchitis, emphysema or asthma (physician-diagnosed) | Questionnaire: ATS |
| HUHTI1 | prevalence | unspec | Yes | Mixed | MRC | Chronic non-specific lung disease (CB (phlegm most days for 3 months for 2 years), emphysema (FEV/FVC <60% or FEV/FVC <70% and x-ray changes) and/or asthma) | Spirometry, x-ray and questionnaire |

^a^ As named by original author.

^b^ Codes are explained at the end of Table 6 (although the information is generally repeated in text form in columns "Description of disease" and " Source of diagnostic information "; the levels are the same for all 3 outcomes).

## Table 6H : COPD other

| **REF** | **Outcome type** | **Diagnostic Criteria^a^** | **Defn incl asthma** | **DIAGSC^b^** | **Questionnaire** | **LUNGFN^b^** | **SPBRON^c^** | **Description of disease** | **Source of diagnostic information** |
| --- | --- | --- | --- | --- | --- | --- | --- | --- | --- |
|  |  |  |  |  |  |  |  |  |  |
| ANDER1 | prevalence | unspec | Yes^d^ | Mixed | Harvard | l | no/unkn | Obstructive lung disease (wheezing most days or nights, dyspnea walking at own pace on level, or FEV1/FVC <60%) or asthma | Questionnaire: Harvard, and spirometry |
| DONTA1 | prevalence | MRC | Yes | Mixed | MRC | - |  | Chronic bronchitis and/or emphysema, or other lung diseases (5% of subjects) | Questionnaire, physical examination |
| FERRI1 | prevalence | Mixed | Yes | Mixed | MRC | l | no/unkn | Chronic non-specific respiratory disease (chronic bronchitis (phlegm for 3+ months for previous 3 years), asthma (physician-diagnosed) or chronic obstructive pulmonary disease (wheezing/whistling in chest most days/nights, breathlessness while walking at own pace, and/or FEV1/FVC <60%)) | Questionnaire, spirometry |
| FERRI2 | prevalence | Mixed | Yes | Mixed | MRC | l | no/unkn | Chronic non-specific respiratory disease (chronic bronchitis (phlegm for 3+ months for previous 3 years), asthma (physician-diagnosed) or chronic obstructive pulmonary disease (wheezing/whistling in chest most days/nights, breathlessness while walking at own pace, and/or FEV1/FVC <60%)) | Questionnaire, spirometry |
| FERRI3 | prevalence | Mixed | Yes | Mixed | MRC | l | no/unkn | Chronic non-specific respiratory disease (chronic bronchitis (phlegm for 3+ months for previous 3 years), asthma (physician-diagnosed) or chronic obstructive pulmonary disease (wheezing/whistling in chest most days/nights, breathlessness while walking at own pace, and/or FEV1/FVC <60%)) | Questionnaire, spirometry |
| FIDAN | prevalence | unspec | No | Mixed | ATS | 2 | no/unkn | Airway disease (chronic bronchitis and/or pathologic signs in auscultation and at least two respiratory symptoms and/or FEV1/FVC <70%) | Questionnaire: ATS, physical examination, spirometry |
| GULSVI | prevalence | unspec | No | Mixed | unnamed/unsp | j | no/unkn | COLD (chronic bronchitis with obstruction, emphysema, asthmatic bronchitis, obstructive lung disease further classification not possible) | Questionnaire, physical examination, lung function, chest x-ray |
| HARIKK | incidence | Mixed | No | Mixed | ATS | 2 | no/unkn | COPD (emphysema, chronic bronchitis, chronic airways obstruction and FEV1/FVC <70%) | Questionnaire, physical examination, spirometry, chest x-ray |
| HEDMAN | prevalence | unspec | No | d | NHS | - |  | COPD : Doctor diagnosed COPD | Questionnaire: physician-diagnosed |
| MARAN1 | prevalence | Thai | No | Mixed | - | d | revers | COPD (FEV1/FVC <70%, reversibility of <15%), no x-ray abnormalities | Spirometry, x-ray |
| MARAN2 | incidence | Thai | No | Mixed | - | d | revers | COPD (FEV1/FVC <70%, reversibility of <15%), no x-ray abnormalities | Spirometry, x-ray |
| PELKON | incidence | Mixed | Unkn | Mixed | - | 2 | no/unkn | Incident FEV1/FVC <70% persisting at all subsequent examinations, or COPD mortality (ICD codes not specified) | Spirometry at 5-yearly examination or death cert |
| SARGEA | prevalence | unspec | Yes | Mixed | unnamed/unsp | 3 | no/unkn | Obstructive airways disease (physician-diagnosed bronchitis, emphysema or asthma, or FEV1 <80% predicted and FEV1/FVC<70%) | Questionnaire: physician diagnosed, spirometry |
| SICHLE | prevalence | Mixed | No | Mixed | MRC | 3 | no/unkn | COPD (BTS criteria from spirometry and/or MRC criteria for chronic bronchitis from questionnaire) | Spirometry, questionnaire: MRC |

^a^ As named by original author.

^b^ Codes are explained at the end of Table 6 (although the information is generally repeated in text form in columns "Description of disease" and " Source of diagnostic information "; the levels are the same for all 3 outcomes).

^c^ Field SPBRON refers to spirometry used post-bronchodilator, no (=without bronchodilator) or unknown, and reversibility criteria.

^d^ Cases of pure asthma (i.e. without OLD) were very rare, 0.4% of males and 0.7% of females.

## Table 6I : CB based on ICD

| **REF** | **Outcome type** | **ICD7^a^** | **ICD8^a^** | **ICD9^a^** | **ICD10^a^** | **DIAGSC^a^** | **Description of disease** | **Source of diagnostic information** |
| --- | --- | --- | --- | --- | --- | --- | --- | --- |
|  |  |  |  |  |  |  |  |  |
| BEST | mortality | 7 | - | - | - | c | Bronchitis mortality (ICD7 500-502) | Death records of Dept of Veterans Affairs |
| DOLL1 | mortality | 9 | - | - | - | c | Chronic bronchitis (underlying cause, ICD codes unspecified) | Death certificates |
| DOLL2 | mortality | 9 | - | - | - | c | Chronic bronchitis (underlying cause, ICD codes unspecified) | Death certificates |
| HIRAYA | mortality | 7 | - | - | - | c | Bronchitis (ICD 500-502) | Death certificates |
| KAHN | mortality | 7 | - | - | - | c | Chronic bronchitis deaths (underlying) (ICD7 500-502) | Death certificates |
| KAHN2 | mortality | 7 | - | - | - | c | Underlying chronic bronchitis deaths (ICD 7: 500-502) | Death certificates |
| WEN | mortality | - | - | b | - | c | Chronic bronchitis (ICD 9: 491) | Death certificates |

^a^  Codes are explained at the end of Table 6 (although the information is generally repeated in text form in columns "Description of disease" and " Source of diagnostic information "; the levels are the same for all 3 outcomes).

## Table 6J : CB based on diagnosis

| **REF** | **Outcome type** | **Diagnostic Criteria^a^** | **DIAGSC^b^** | **Questionnaire** | **Description of disease** | **Source of diagnostic information** |
| --- | --- | --- | --- | --- | --- | --- |
|  |  |  |  |  |  |  |
| ALDERS | prevalence | unspec | r | - | Chronic bronchitis: Hospital diagnosis | Hospital records (final diagnosis if available) |
| BANG | prevalence | unspec | d | NHANES^c^ | Chronic bronchitis: Self reported physician- diagnosed CB | Questionnaire (NHANES?): physician-diagnosed |
| BROWN | prevalence | unspec | Mixed | - | Chronic bronchitis : Medical diagnosis of CB | Physical examination, x-ray |
| COLLEG | prevalence | unspec | Other | - | Chronic bronchitis (diagnosed by physician using standard questionnaire) | Physician diagnosis |
| DONTA2 | incidence | MRC | Mixed | MRC | Clinical diagnosis of chronic bronchitis (using MRC criteria) | Questionnaire, physical examination, spirometry |
| ENRIGH | prevalence | unspec | d | ATS | Chronic bronchitis (physician-diagnosed) | Questionnaire: ATS |
| HARDIE | prevalence | unspec | d | ATS | Current physician-diagnosed chronic bronchitis | Questionnaire: ATS |
| KOTAN1 | prevalence | unspec | d | FinEsS | Chronic bronchitis (physician-diagnosed) | Questionnaire: FinEsS |
| LEBOWI | prevalence | unspec | d | NHLI | Chronic bronchitis (physician diagnosed) | Questionnaire: NHLI |
| MILLER | prevalence | unspec | Other | - | Chronic bronchitis (examining physician's impression of chronic bronchitis) | Physical examination; physician diagnosis, criteria unspecified |
| SCHWAR | prevalence | unspec | d | NHANES | Bronchitis (ever diagnosed by physician and still has condition) | Questionnaire: physician diagnosed |
| TROISI | incidence | unspec | d | NHS | Chronic bronchitis (CB based on physician diagnosis) | Questionnaire: physician diagnosed |
| VIEGI1 | prevalence | unspec | d | NHLBI | Chronic bronchitis (confirmed by physician) | Questionnaire: physician diagnosed |
| WILSO2 | prevalence | unspec | d | HealthOmnibus | Bronchitis (physician-diagnosed) | Questionnaire: Health Omnibus Survey |

^a^ As named by original author.

^b^ Codes are explained at the end of Table 6 (although the information is generally repeated in text form in columns "Description of disease" and " Source of diagnostic information "; the levels are the same for all 3 outcomes).

^c^ Assumed that HHANES used same questionnaire as NHANES studies.

## Table 6K : CB based on self-report

| **REF** | **Outcome type** | **Diagnostic Criteria^a^** | **DIAGSC^b^** | **Questionnaire** | **Description of disease** | **Source of diagnostic information** |
| --- | --- | --- | --- | --- | --- | --- |
|  |  |  |  |  |  |  |
| HO | prevalence | unspec | q | unnamed/unsp | Chronic bronchitis (self-reported) | Questionnaire (unnamed) |
| KATO | prevalence | unspec | q | unnamed/unsp | History of chronic bronchitis (criteria unspecified) | Questionnaire (unspecified) |
| LAVECC | prevalence | unspec | q | unnamed/unsp | Chronic bronchitis (self-reported) | Questionnaire |

^a^ As named by original author.

^b^ Codes are explained at the end of Table 6 (although the information is generally repeated in text form in columns "Description of disease" and " Source of diagnostic information "; the levels are the same for all 3 outcomes).

## Table 6L : CB based on symptoms

| **REF** | **Outcome type** | **Diagnostic Criteria^a^** | **DIAGSC^b^** | **Questionnaire** | **Description of disease** | **Source of diagnostic information** |
| --- | --- | --- | --- | --- | --- | --- |
|  |  |  |  |  |  |  |
| ANDER1 | prevalence | unspec | q | Harvard | Chronic bronchitis (phlegm on 4 days/week for 3 months/year for 3 years) | Questionnaire: Harvard |
| BECK1 | prevalence | MRC | q | unnamed/unsp | Chronic bronchitis (cough and phlegm for 3 months for at least 2 years) | Questionnaire |
| BECK2 | incidence | MRC | q | unnamed/unsp | Chronic bronchitis (cough and phlegm for 3 months for at least 2 years) | Questionnaire |
| BJORNS | prevalence | unspec | q | MRC/ECRHS | Bronchitis symptoms (phlegm and long-term cough and/or morning cough in recent years) | Questionnaire: ECRHS/MRC |
| CERVER | prevalence | MRC | q | ECRHS | Chronic bronchitis (cough and phlegm most days for at least 2 years) | Questionnaire: ECRHS |
| CHAPMA | prevalence | unspec | q | MRC | Persistent cough and phlegm (for at least 3 months every year) | Questionnaire: MRC |
| COATES | prevalence | MRC | q | MRC | Chronic bronchitis (combined chronic cough and phlegm on most days for at least 3 months every year) | Questionnaire: MRC |
| DEAN2 | prevalence | unspec | q | MRC | Bronchitis syndrome (shortness of breath, prolonged cough and prolonged phlegm) | Questionnaire: MRC |
| DEANE | prevalence | unspec | q | MRC | Chronic bronchitis (persistent cough and phlegm) | Questionnaire: MRC |
| DEMARC | prevalence | unspec | q | MRC/ECRHS | Chronic bronchitis (regular cough with phlegm for at least 3 months every year) | Questionnaire: MRC/ECRHS |
| DOPICO | prevalence | unspec | q | ATS | Chronic bronchitis (phlegm usually daily or twice a day 4 days/week, for 3 months of year for 2 or more years) | Questionnaire: ATS |
| EHRLIC | prevalence | MRC | q | unnamed/unsp | Chronic bronchitis (cough with phlegm for 3 months for at least 2 years) | Questionnaire |
| FERRI1 | prevalence | unspec | q | MRC | Chronic bronchitis (phlegm on 4+ days per week for 3 months per year for 3 years) | Questionnaire: MRC |
| FINKLE | prevalence | MRC | q | MRC | Chronic bronchitis (Cough and phlegm for 3+ months each year) | Questionnaire: MRC |
| FLETCH | prevalence | unspec | q | unnamed/unsp | Chronic bronchitis (production of phlegm on rising on most days for at least 3 months each year) | Questionnaire (unnamed) |
| FOXMAN | prevalence | unspec | q | WHO | Chronic bronchitis (phlegm most days 3 months in last year) | Questionnaire: WHO |
| GOLDBE | prevalence | MRC | q | MRC | Chronic bronchitis (cough and phlegm for 3+ months each year) | Questionnaire: MRC |
| HAENSZ | prevalence | MRC | q | MRC | Chronic bronchitis (termed MRC but symptoms unspecified) | Questionnaire: MRC |
| HARRIS | prevalence | unspec | q | MRC | Chronic bronchitis: MRC questionnaire but criteria unspecified | Questionnaire: MRC |
| HAWTHO | prevalence | MRC | q | MRC | Bronchitis syndrome (MRC but exact criteria not given) | Questionnaire: MRC |
| HAYES | prevalence | MRC | q | MRC | Chronic bronchitis (Cough and phlegm for 3+ months each year) | Questionnaire: MRC |
| HIGGI2 | prevalence | unspec | q | unnamed/unsp | Chronic bronchitis (persistent phlegm for at least 3 months of year + at least 1 chest illness with increased cough and sputum during past 3 years) | Questionnaire |
| HIGGI3 | prevalence | unspec | q | unnamed/unsp | Chronic bronchitis (persistent phlegm and 1+ bronchitic chest illness in past 3 years) | Questionnaire |
| HIGGI6 | prevalence | unspec | Mixed | unnamed/unsp | Chronic bronchitis (cough and phlegm for at least 3 months each year) | Questionnaire (unnamed), physician examination |
| HOLLA2 | prevalence | unspec | q | MRC | Persistent cough and phlegm (assumed to be defined as cough and phlegm, either morning or day, in winter for 3 months each year | Questionnaire: MRC |
| HOLLNA | prevalence | MRC | q | unnamed/unsp | Chronic bronchitis (cough and/or phlegm for 3 months for the past 2 years) | Questionnaire (unnamed) |
| HOUSE | prevalence | MRC | q | MRC | Chronic bronchitis (cough and phlegm most days for at least 3 months every year) | Questionnaire: MRC |
| HRUBEC | prevalence | MRC | q | MRC | Bronchitis (regular or extended periods of cough, and phlegm from chest for more than 3 months) | Questionnaire: MRC |
| HUCHON | prevalence | unspec | q | ECSC | Chronic bronchitis (cough + phlegm for 3 months for at least 2 years) | Questionnaire: ECSC |
| HUHTI1 | prevalence | MRC | q | MRC | Chronic bronchitis (production of phlegm on most days for at least 3 months of year for at least 2 consecutive years) | Questionnaire: MRC |
| HUHTI2 | prevalence | unspec | q | MRC | Chronic bronchitis (production of phlegm on most days for at least 3 months of year) | Questionnaire: MRC |
| HUHTI3 | prevalence | unspec | q | MRC | Chronic bronchitis (Production of phlegm on most days for at least 3 months of year) | Questionnaire: MRC |
| JINDA2 | prevalence | unspec | q | unnamed/unsp | Chronic bronchitis (cough with phlegm for 3 months for at least 2 years) | Questionnaire (unnamed) |
| JOSHI | prevalence | MRC | q | MRC | Chronic bronchitis (phlegm for 3+ months/year for at least 2 years) | Questionnaire: MRC |
| JOUSI1 | prevalence | Rose | q | Rose | Chronic bronchitis symptoms grade 2 (cough with phlegm most days or nights for 3 months each year) | Questionnaire: Rose |
| KUBIK | prevalence | MRC | q | unnamed/unsp | Chronic bronchitis (cough with phlegm for 3 months for at least 2 years) | Questionnaire (unspecified) |
| LAMBER | prevalence | unspec | q | MRC | Chronic bronchitis (cough and phlegm lasting 3+ months, breathlessness on walking and period of increased cough and phlegm lasting 3+ weeks in past 3 years) | Questionnaire: MRC |
| LANGE2 | prevalence | unspec | q | unnamed/unsp | Chronic bronchitis (phlegm for at least 3 months per year for at least 2 consecutive years) | Questionnaire (unnamed) |
| LANGHA | prevalence | ATS | q | unnamed/unsp | Chronic bronchitis (cough and phlegm for 3+ months for the past 2 years) | Questionnaire (unspecified) |
| LINDST | prevalence | unspec | q | FinEsS | Chronic productive cough (cough with phlegm most days for at least 3 months for at least 2 successive years) | Questionnaire: FinEsS |
| MAGNUS | prevalence | ATS | q | ECRHS | Chronic bronchitis (phlegm from chest for 3 months for 2 years) | Questionnaire: ECRHS |
| MANFRE | prevalence | unspec | q | NHLBI | Chronic bronchitis (cough and/or phlegm most days at least 3 months every year) | Questionnaire: NHLBI |
| MENEZ1 | prevalence | unspec | q | ATS | Chronic bronchitis (cough and phlegm most days for 3 months for at least 2 years) | Questionnaire: ATS |
| MEREN | prevalence | unspec | q | OLIN | Chronic productive cough (phlegm when coughing or phlegm on chest at least 3 months in 2 successive years) | Questionnaire: OLIN |
| MILNE | prevalence | unspec | q | MRC | Persistent cough and phlegm | Questionnaire: MRC |
| MOLLER | prevalence | MRC | u | - | Chronic obstructive bronchitis (cough and phlegm for 3 months during 2 previous years) | Unspecified |
| MUELLE | prevalence | unspec | q | MRC | Chronic bronchitis (phlegm from chest for 3+ months every year) | Questionnaire: MRC |
| NEJJAR | prevalence | Fletcher | q | unnamed/unsp | Chronic bronchitis (phlegm most days for 3 months for previous 2 years) | Questionnaire (unspecified) |
| OGILVI | prevalence | unspec | Mixed | unnamed/unsp | Chronic bronchitis (daily cough with phlegm for 3 months for at least 2 years) without other causative respiratory disease | Questionnaire, confirmed by physical examination |
| OSWAL1 | prevalence | unspec | r | - | Chronic bronchitis (cough/phlegm/breathlessness with some degree of disability persisting for at least 1 year) | Hospital diagnosis |
| OSWAL2 | prevalence | unspec | q | unnamed/unsp | Bronchitis (habitual cough and phlegm with disability due to exacerbations and/or breathlessness in previous 5 years) | Questionnaire (unnamed) |
| PANDEY | prevalence | MRC | Mixed | - | Chronic bronchitis (MRC criteria: cough and sputum most days for at least 3 consecutive months of 2 successive years) | Questionnaire: MRC, usually confirmed at field clinic |
| PEREZP | prevalence | unspec | q | ATS | Chronic bronchitis (cough or phlegm on most days for more than 3 months of year for at least 2 consecutive years) | Questionnaire: ATS |
| REID | prevalence | unspec | q | unnamed/unsp | Chronic bronchitis (persistent cough and phlegm (most days for at least 3 months each year), with shortness of breath while walking with other people at ordinary pace, and at least 1 period of increased cough and phlegm lasting 3+ weeks during previous 3 years) | Questionnaire (un-named) |
| RIMING | prevalence | unspec | q | unnamed/unsp | Chronic bronchitis (coughed up phlegm for 3 months for at least 2 years) | Questionnaire |
| SAWICK | prevalence | unspec | q | MRC | Chronic bronchitis (cough and phlegm most days for 3 consecutive months for at least 2 years) | Questionnaire: MRC |
| SHARP | prevalence | unspec | q | MRC | Persistent cough and phlegm for at least 3 months per year | Questionnaire: MRC |
| SOBRAD | prevalence | unspec | q | CECA | Chronic bronchitis (cough and phlegm for 3+ months per year for at least 2 consecutive years) | Questionnaire: CECA |
| STJERN | prevalence | ATS | q | MRC | Chronic bronchitis (excessive mucous secretion and chronic recurrent cough for 3 months for at least 2 years, excluding other causes) | Questionnaire: MRC |
| SUADIC | prevalence | MRC | q | MRC | Chronic bronchitis (cough and phlegm for 3 months for at least 2 years) | Questionnaire: MRC |
| TAGER2 | prevalence | unspec | q | MRC | Chronic bronchitis (cough and phlegm for 3 months per year for 2+ years) | Questionnaire: MRC |
| URRUTI | prevalence | unspec | q | ECRHS | Chronic bronchitis (cough and phlegm for 3 months for last 2 years), and no asthma-related symptoms (woken in night due to lack of air or asthma attack in last 12 months, or taking asthma medication) | Questionnaire: ECRHS |
| WAGEN2 | prevalence | MRC | q | unnamed/unsp | Chronic bronchitis (cough and phlegm most days for 3 months during 2 previous years) | Questionnaire (not specified) |
| WILHEL | prevalence | MRC | Other | - | Chronic bronchitis (cough and phlegm for 3 months each year) | Physical examination |
| WOJTYN | prevalence | unspec | q | MRC | Chronic bronchitis (cough and phlegm most days for at least 3 consecutive months for 2 or more years) at both baseline and 5-year follow-up | Questionnaire: MRC |
| WOODS | prevalence | unspec | q | ECRHS | Chronic bronchitis (cough with phlegm for 3 months for at least 2 successive years) | Questionnaire: ECRHS |
| WOOLF | prevalence | unspec | Mixed | - | Chronic bronchitis (chronic cough and phlegm) | History and physical examination |
| YAMAGU | prevalence | MRC | q | MRC | Chronic bronchitis (cough and phlegm most days for 3 months each year) | Questionnaire: MRC |
| ZOIA | prevalence | unspec | q | unnamed/unsp | Chronic bronchitis (chronic cough and phlegm) | Questionnaire (unspecified) |

^a^ As named by original author.

^b^ Codes are explained at the end of Table 6 (although the information is generally repeated in text form in columns "Description of disease" and " Source of diagnostic information "; the levels are the same for all 3 outcomes).

## Table 6M : CB other

| **REF** | **Outcome type** | **Diagnostic Criteria^a^** | **DIAGSC^b^** | **Questionnaire** | **LUNGFN^b^** | **SPBRON^c^** | **Description of disease** | **Source of diagnostic information** |
| --- | --- | --- | --- | --- | --- | --- | --- | --- |
|  |  |  |  |  |  |  |  |  |
| JENSEN | prevalence | unspec | Mixed | MRC^d^ | - |  | Bronchitis (physician diagnosis of bronchitis, asthmatic bronchitis or asthma, <20% increase in FEV1 after terbutaline inhalation and/or daily cough and/or expectoration for at least 3 months a year) | Questionnaire (MRC?): physician diagnosis/other |
| KIRAZ | prevalence | unspec | Mixed | MRC/ATS | v | no/unkn | Chronic bronchitis (Cough and sputum most days for at least 3 months for at least 2 years plus chest rales, decreased respiratory sounds or increased expiration time and FEV1/FVC <88% predicted, with or without COPD (reversibility of <12%))^e^ | Questionnaire: ATS/MRC, physical examination, spirometry |
| LUNDB2 | prevalence | unspec | Mixed | MRC | - |  | Self-reported respiratory disease/symptoms confirmed at examination as chronic bronchitis (cough/sputum most days for at least 3 months for at least 2 years, or impaired lung function of obstructive type and FEV1 <80% predicted with history typical of chronic bronchitis) | Questionnaire: MRC, spirometry, physical examination |
| MELLST | prevalence | Fletcher | Other | - | - |  | Chronic bronchitis (Phlegm most days for 3+ months each year and/or diagnosis of asthmatic bronchitis in previous 10 years) | Physical examination |
| PELKON | incidence | Mixed | Mixed | MRC | - |  | Incident chronic bronchitis (cough with phlegm for at least 3 months each year), or CB mortality (ICD codes not specified) | Questionnaire: MRC or death certificate |
| SHIMUR | prevalence | Mixed # | r | ATS/MRC | - |  | Chronic bronchitis (ATS/MRC criteria), chronic obstructive mucopurulent bronchitis, with phlegm for at least 5 years and chronic airflow limitation, death from chronic respiratory failure | Medical records |
| SILVA | incidence | ATS | q | NHLI | c | no/unkn | Chronic bronchitis (cough and phlegm most days for 3 months for at least 2 consecutive years and FEV1 <80% predicted) and no physician diagnosis of emphysema | Spirometry, questionnaire: NHLI |
| WIG | prevalence | unspec | Mixed | unnamed/unsp | - |  | Chronic bronchitis (criteria unspecified) | Questionnaire (unnamed), physical examination, x-ray |

^a^ As named by original author.

^b^ Codes are explained at the end of Table 6 (although the information is generally repeated in text form in columns "Description of disease" and " Source of diagnostic information "; the levels are the same for all 3 outcomes).

^c^ Field SPBRON refers to spirometry used post-bronchodilator, no (=without bronchodilator) or unknown, and reversibility criteria.

^d^ Questionnaire used stated to be British Committee on Research into Chronic Bronchitis, assumed to be MRC.

^e^ CB cases appear to have been split according to the presence of COPD, although this is not made clear. Therefore, both groups combined to form CB endpoint.

## Table 6N : Emphysema based on ICD

| **REF** | **Outcome type** | **ICD7^a^** | **ICD8^a^** | **ICD9^a^** | **ICD10^a^** | **DIAGSC^a^** | **Description of disease** | **Source of diagnostic information** |
| --- | --- | --- | --- | --- | --- | --- | --- | --- |
|  |  |  |  |  |  |  |  |  |
| BEST | mortality | 8 | - | - | - | c | Emphysema mortality (ICD7 527.1) | Death records of Dept of Veterans Affairs |
| HAMMO2 | mortality | 8 | - | - | - | c | Emphysema (ICD 7 Code 527.1, modified to include bronchitis and emphysema as joint cause) | Death certificates |
| HIRAYA | mortality | 8 | - | - | - | c | Emphysema (ICD 527.1) | Death certificates |
| KAHN | mortality | 8 | - | - | - | c | Emphysema deaths (underlying) (ICD7 527.1) | Death certificates |
| KAHN2 | mortality | 8 | - | - | - | c | Underlying emphysema deaths (ICD 7: 527.1) | Death certificates |
| WEN | mortality | - | - | c | - | c | Emphysema (ICD 9: 492) | Death certificates |

^a^ Codes are explained at the end of Table 6 (although the information is generally repeated in text form in columns "Description of disease" and " Source of diagnostic information "; the levels are the same for all 3 outcomes).

## Table 6O : Emphysema based on visual comparison

| **REF** | **Outcome type** | **Diagnostic Criteria^a^** | **DIAGSC^b^** | **Questionnaire** | **Description of disease** | **Source of diagnostic information** |
| --- | --- | --- | --- | --- | --- | --- |
|  |  |  |  |  |  |  |
| ANDER2 | prevalence | unspec | a | - | Emphysema(visual comparison with pre-set standard, Grade 1+ vs none) | Autopsy |
| AUERBA | prevalence | unspec | a | - | Emphysema (visual comparison against standard (Grade 1+)) | Autopsy |
| NAWA | prevalence | unspec | t | - | Emphysematous changes (visual comparison) | Low dose spiral CT scan |
| OMORI | prevalence | Japanese | t | - | Emphysema (visual comparison with pre-defined grading system) | Low dose CT scan plus high resolution CT at 3 locations in current and ex-smokers only |
| PRATT | prevalence | unspec | a | - | Centrilobular emphysema (visual comparison with standard point-counting technique, including trace only) | Autopsy |
| RYDER | prevalence | unspec | a | - | Emphysema (airspace of 0.1cm diameter or greater) | Autopsy |
| SUTINE | prevalence | unspec | a | - | Emphysema (air spaces >0.1 cm, grade 5+) | Autopsy |
| VIKGRE | incidence | unspec | t | - | Emphysema (areas of low attentuation and/or presence of stretched narrow vessels) | CT scan |
| WANG2 | prevalence | unspec | t | - | Emphysema (low-attenuation areas with disrupted vasculature but without discernible surrounding walls in the pulmonary parenchyma) | CT scan |
| WEISS | prevalence | unspec | x | - | Emphysema (low flat diaphragm with little difference demonstrable photofluorographically between inspiration and expiration films, increased general radiolucency of the lungs and widening of the interspaces) | X-ray |

^a^ As named by original author.

^b^ Codes are explained at the end of Table 6 (although the information is generally repeated in text form in columns "Description of disease" and " Source of diagnostic information "; the levels are the same for all 3 outcomes).

## Table 6P : Emphysema based on diagnosis

| **REF** | **Outcome type** | **Diagnostic Criteria^a^** | **DIAGSC^b^** | **Questionnaire** | **Description of disease** | **Source of diagnostic information** |
| --- | --- | --- | --- | --- | --- | --- |
|  |  |  |  |  |  |  |
| DONTA2 | incidence | unspec | Mixed | MRC | Clinical diagnosis of pulmonary emphysema | Questionnaire, physical examination, spirometry |
| ENRIGH | prevalence | unspec | d | ATS | Emphysema: physician diagnosed | Questionnaire: ATS |
| GULSVI | prevalence | unspec | d | unnamed/unsp | Emphysema (physician diagnosis/hospitalisation) | Questionnaire (unspecified) |
| HARDIE | prevalence | unspec | d | ATS | Ever diagnosed with emphysema by physician | Questionnaire: ATS |
| HOZAWA | prevalence | unspec | d | unnamed/unsp | Emphysema (physician-diagnosed) | Questionnaire (unnamed) |
| LEBOWI | prevalence | unspec | d | NHLI | Emphysema (physician diagnosed) | Questionnaire: NHLI |
| MILLER | prevalence | unspec | d | MRC | Emphysema (ever diagnosed by physician) | Questionnaire (physician diagnosis) |
| WILSO2 | prevalence | unspec | d | HealthOmnibus | Emphysema (physician-diagnosed) | Questionnaire: Health Omnibus Survey |

^a^ As named by original author.

^b^ Codes are explained at the end of Table 6 (although the information is generally repeated in text form in columns "Description of disease" and " Source of diagnostic information "; the levels are the same for all 3 outcomes).

## Table 6Q : Emphysema based on self-report

| **REF** | **Outcome type** | **Diagnostic Criteria^a^** | **DIAGSC^b^** | **Questionnaire** | **Description of disease** | **Source of diagnostic information** |
| --- | --- | --- | --- | --- | --- | --- |
|  |  |  |  |  |  |  |
| HO | prevalence | unspec | q | unnamed/unsp | Emphysema (self-reported) | Questionnaire (unnamed) |
| LAVECC | prevalence | unspec | q | unnamed/unsp | Emphysema or respiratory insufficiency (self- reported) | Questionnaire (unspecified) |

^a^ As named by original author.

^b^ Codes are explained at the end of Table 6 (although the information is generally repeated in text form in columns "Description of disease" and " Source of diagnostic information "; the levels are the same for all 3 outcomes).

## Table 6R : Emphysema other

| **REF** | **Outcome type** | **Diagnostic Criteria^a^** | **DIAGSC^b^** | **Questionnaire** | **LUNGFN^b^** | **SPBRON^c^** | **Description of disease** | **Source of diagnostic information** |
| --- | --- | --- | --- | --- | --- | --- | --- | --- |
|  |  |  |  |  |  |  |  |  |
| HUHTI1 | prevalence | unspec | Mixed | - | 2 | no/unkn | Emphysema (FEV1/FVC <60% or FEV1/FVC 60-69% plus 2 or more of following chest x-ray changes: deficiency of peripheral vascular pattern, prominent main pulmonary artery and hilar blood vessels, narrow vertically situated heart, low position or poor mobility of diaphragm, large retrosternal space) | Spirometry, x-ray |
| SILVA | incidence | unspec | d | NHLI | i | no/unkn | Emphysema (physician-diagnosis, and FEV1 or DLCO <80% predicted) | Spirometry, questionnaire: NHLI |

^a^ As named by original author.

^b^ Codes are explained at the end of Table 6 (although the information is generally repeated in text form in columns "Description of disease" and " Source of diagnostic information "; the levels are the same for all 3 outcomes).

^c^ Field SPBRON refers to spirometry used post-bronchodilator, no (=without bronchodilator) or unknown, and reversibility criteria.

| Grading systems used throughout Table 6: | | |
| --- | --- | --- |
| DIAGSC | r | Hospital/GP records |
|  | d | Questionnaire: physician diagnosis |
|  | q | Questionnaire: other |
|  | s | Spirometry |
|  | x | X-ray |
|  | t | CT scan |
|  | c | Death certificate/registry |
|  | a | Autopsy |
|  | o | Other |
|  | m | Mixed |
|  | u | Unspecified |
| ICD7 | 1 | 502,527.1 |
|  | 2 | 501-502,527.1,(527.2 later) |
|  | 3 | 500-502,527.1 |
|  | 4 | 501,502,526,527.1 |
|  | 5 | 241,500-502,527.1 |
|  | 6 | 502,526,527.1 |
|  | 7 | 500-502 |
|  | 8 | 527.1 |
|  | 9 | unspecified |
|  | a | 502.0, 527.1 |
| ICD8 | 1 | 491-492 |
|  | 2 | 490-492 |
|  | 3 | 490-492,518 |
|  | 4 | 490-492,519 |
|  | 5 | 491-493 |
|  | 6 | 490-493 |
|  | 7 | 466, 490-493 |
|  | 8 | 490-493,518,519.3 |
|  | 9 | unspecified |
|  | a | 491-492,519 |
| ICD9 | 1 | 491,492,496 |
|  | 2 | 490-492,496 |
|  | 3 | 490-492,496,416.7 |
|  | 4 | 491-493 |
|  | 5 | 490-493 |
|  | 6 | 490-496 |
|  | 7 | 490-492,494,496 |
|  | 8 | 416,491,492,496,519 |
|  | 9 | unspecified |
|  | a | 466, 490-493 |
|  | b | 491 |
|  | c | 492 |
| ICD10 | 2 | J40-J44 |
|  | 3 | J43-J44 |
|  | 9 | unspecified |

| LUNGFUN | 2 | FEV1/FVC <70% |
| --- | --- | --- |
|  | 3 | FEV1/FVC<70% and FEV1<80% predicted |
|  | 4 | FEV1/FVC<88%M, 89%F |
|  | 5 | FEV1/FVC<normal (ATS value nk) |
|  | 6 | FEV1<65% predicted or FEV/FVC<65% |
|  | 7 | FEV1% <60 |
|  | 8 | FEV1/FVC <80% |
|  | 9 | FEV1 <75% predicted |
|  | a | FEV1/or VC <70% predicted |
|  | b | FEV1 <65% predicted |
|  | c | FEV1 <80% predicted |
|  | d | FEV1/FVC<70% + reversibility <15% |
|  | e | FEV1/FVC<88%+ reversibility <12% |
|  | f | FEV1<5^th^ percentile + reversibility <9% |
|  | g | FEV1/FVC <65% |
|  | h | FEV1 or MMEF <2SD below predicted |
|  | i | FEV1 or DLCO <80% predicted |
|  | j | unspecified |
|  | k | FEV1/FVC <68% |
|  | l | FEV1/FVC <60% |
|  | m | FEV1/FVC<70% + FEV1<80% predicted + reversibility <10% |
|  | n | GOLD2+ (FEV1/FVC<70%,FEV1<80%) |
|  | o | FEV1/FVC<70% + FEV1<70% predicted + reversibility <12% |
|  | p | FEV1 < 85% predicted |
|  | q | ATS NOS |
|  | r | FEV1/FVC<75% |
|  | s | FEV1/FVC<85% |
|  | t | FEV1/FVC<70%, FEV1<80% predicted, reversibility <12% |
|  | u | FEV1 > 2SE below predicted |
|  | v | FEV1/FVC <88% predicted |
