# Supplementary material for: Systematic review with meta-analysis of the epidemiological evidence relating smoking to COPD, chronic bronchitis and emphysema
Source: BMC Pulm Med. 2011 Jun 14;11:36. doi: 10.1186/1471-2466-11-36 (PMC3128042; doi:10.1186/1471-2466-11-36)
Supplement: Additional file 3 — RRs. .DOC file concerning the RRs included on the database. This gives the numbers of RRs per study as well as fuller distributions than those given in the paper of the characteristics of the RRs for the major smoking indices and the dose-response indices, and of the characteristics of the sets of RRs for the dose-response indices. It also lists which studies provide RRs for which indices, and gives details of the results of checking RRs for apparent errors. [file 1471-2466-11-36-S3.DOC]

**Systematic review with meta-analysis of the epidemiological evidence relating smoking to COPD, chronic bronchitis and emphysema**

Barbara A Forey, Alison J Thornton and Peter N Lee

**Additional File 3 : RRs**

Contents

[Table 1 Numbers of relative risks per study 3](#__RefHeading___Toc293476587)

[Table 2 Characteristics of the relative risks for the major smoking indices 4](#__RefHeading___Toc293476588)

[Table 2 (continued) COPD 6](#__RefHeading___Toc293476589)

[Table 2 (continued) CB 8](#__RefHeading___Toc293476590)

[Table 2 (continued) Emphysema 10](#__RefHeading___Toc293476591)

[Table 3 Characteristics of the relative risks for the dose-response indices 12](#__RefHeading___Toc293476592)

[Table 3 (continued) COPD 15](#__RefHeading___Toc293476593)

[Table 3 (continued) CB 17](#__RefHeading___Toc293476594)

[Table 3 (continued) Emphysema 19](#__RefHeading___Toc293476595)

[Table 4 Characteristics of the sets of relative risks for the dose-response indices 21](#__RefHeading___Toc293476596)

[Table 4 (continued) COPD 24](#__RefHeading___Toc293476597)

[Table 4 (continued) CB 27](#__RefHeading___Toc293476619)

[Table 4 (continued) Emphysema 29](#__RefHeading___Toc293476642)

[Table 5 Studies with RRs for the major smoking indices 30](#__RefHeading___Toc293476651)

[Table 6 Studies with RRs for the dose-response indices 33](#__RefHeading___Toc293476677)

[Table 7 Relative risks with apparent errors 37](#__RefHeading___Toc293476685)

[Table 7A RR non-central in CI 37](#__RefHeading___Toc293476686)

[Table 7B Number of cases less than minimum implied by CI 38](#__RefHeading___Toc293476689)

[Table 7C Number in any cell of 2x2 table less than minimum implied by CI 40](#__RefHeading___Toc293476690)

[Table 7D Other 41](#__RefHeading___Toc293476691)

References – see main paper

## Table 1 Numbers of relative risks per study

|  |  | **Study Type** | | | | |
| --- | --- | --- | --- | --- | --- | --- |
|  |  | **CC** | **Prosp** | **CrossSec** | **Subsid** | **Total** |
|  |  |  |  |  |  |  |
| **Number of relative risks (overall)** |  |  |  |  |  |  |
|  | **1-2** | 2 | 5 | 16 | 3 | 26 |
|  | **3-4** | 7 | 10 | 26 | 4 | 47 |
|  | **5-6** | 2 | 5 | 20 | 2 | 29 |
|  | **7-8** | 3 | 1 | 13 | 1 | 18 |
|  | **9-10** | 1 | 4 | 9 | 1 | 15 |
|  | **11-20** | 2 | 2 | 29 | 5 | 38 |
|  | **21-50** | 1 | 9 | 15 | 6 | 31 |
|  | **51-100** | 1 | 3 | 4 | 1 | 9 |
|  | **>100** | 1 | 0 | 2 | 2 | 5 |
|  | Total | 20 | 39 | 134 | 25 | 218 |
|  | Median | 6.00 | 6.00 | 8.00 | 12.00 | 8.00 |
|  | Range | 1:118 | 1:56 | 1:211 | 1:149 | 1:211 |
|  |  |  |  |  |  |  |
| **Number of relative risks for COPD** |  |  |  |  |  |  |
|  | **0** | 6 | 4 | 67 | 8 | 85 |
|  | **1-2** | 1 | 4 | 12 | 3 | 20 |
|  | **3-4** | 6 | 11 | 13 | 4 | 34 |
|  | **5-6** | 2 | 6 | 10 | 1 | 19 |
|  | **7-8** | 2 | 2 | 8 | 1 | 13 |
|  | **9-10** | 1 | 2 | 2 | 1 | 6 |
|  | **11-20** | 1 | 3 | 14 | 3 | 21 |
|  | **21-50** | 0 | 5 | 6 | 2 | 13 |
|  | **51-100** | 0 | 2 | 2 | 2 | 6 |
|  | **>100** | 1 | 0 | 0 | 0 | 1 |
|  | Total^a^ | 14 | 35 | 67 | 17 | 133 |
|  | Median^a^ | 5.00 | 6.00 | 6.00 | 7.00 | 6.00 |
|  | Range^a^ | 2:118 | 1:56 | 1:86 | 1:58 | 1:118 |
|  |  |  |  |  |  |  |
| **Number of relative risks for CB** |  |  |  |  |  |  |
|  | **0** | 13 | 30 | 63 | 11 | 117 |
|  | **1-2** | 1 | 3 | 7 | 2 | 13 |
|  | **3-4** | 3 | 1 | 10 | 1 | 15 |
|  | **5-6** | 0 | 1 | 14 | 0 | 15 |
|  | **7-8** | 0 | 0 | 6 | 1 | 7 |
|  | **9-10** | 0 | 0 | 7 | 1 | 8 |
|  | **11-20** | 1 | 2 | 14 | 4 | 21 |
|  | **21-50** | 1 | 2 | 9 | 3 | 15 |
|  | **51-100** | 1 | 0 | 3 | 2 | 6 |
|  | **>100** | 0 | 0 | 1 | 0 | 1 |
|  | Total^a^ | 7 | 9 | 71 | 14 | 101 |
|  | Median^a^ | 4.00 | 6.00 | 8.00 | 16.00 | 9.00 |
|  | Range^a^ | 1:91 | 1:23 | 1:152 | 2:91 | 1:152 |
|  |  |  |  |  |  |  |
| **Number of relative risks for Emphysema** |  |  |  |  |  |  |
|  | **0** | 20 | 32 | 115 | 23 | 190 |
|  | **1-2** | 0 | 2 | 4 | 0 | 6 |
|  | **3-4** | 0 | 1 | 4 | 0 | 5 |
|  | **5-6** | 0 | 0 | 7 | 0 | 7 |
|  | **9-10** | 0 | 1 | 1 | 1 | 3 |
|  | **11-20** | 0 | 2 | 1 | 0 | 3 |
|  | **21-50** | 0 | 1 | 2 | 0 | 3 |
|  | **51-100** | 0 | 0 | 0 | 1 | 1 |
|  | Total^a^ | 0 | 7 | 19 | 2 | 28 |
|  | Median^a^ | - | 9.00 | 6.00 | 30.50 | 6.00 |
|  | Range^a^ | - | 1:22 | 1:39 | 10:51 | 1:51 |

^a^ Excluding zero category

## Table 2 Characteristics of the relative risks for the major smoking indices

| **All RRs^a^** |  | **Study Type^b^** | | | | |
| --- | --- | --- | --- | --- | --- | --- |
| **Variable^c^** | **Levels^c^** | **CC** | **Prosp** | **CrossSec** | **Subsid** | **Total** |
|  |  |  |  |  |  |  |
| **Total** |  | 91 | 200 | 912 | 236 | 1439 |
|  |  |  |  |  |  |  |
| **Sex** | **both** | 24 | 28 | 200 | 12 | 264 |
|  | **male** | 37 | 118 | 354 | 113 | 622 |
|  | **female** | 30 | 54 | 358 | 111 | 553 |
|  |  |  |  |  |  |  |
| **Onset** | **prevalence** | 91 | 6 | 912 | 179 | 1188 |
|  | **onset** | 0 | 194 | 0 | 57 | 251 |
|  |  |  |  |  |  |  |
| **Exposed group: smoking status** | **ever** | 38 | 62 | 316 | 71 | 487 |
|  | **current** | 30 | 82 | 343 | 91 | 546 |
|  | **ex** | 23 | 56 | 253 | 74 | 406 |
|  |  |  |  |  |  |  |
| **Exposed group: smoking product** | **all** | 27 | 58 | 321 | 69 | 475 |
|  | **cigarettes (+/- other)** | 48 | 98 | 499 | 121 | 766 |
|  | **cigarettes only** | 16 | 44 | 92 | 46 | 198 |
|  |  |  |  |  |  |  |
| **Exposed group:cigarette type** | **all** | 46 | 141 | 589 | 167 | 943 |
|  | **manufactured only** | 18 | 1 | 2 | 0 | 21 |
|  |  |  |  |  |  |  |
| **Unexposed group** | **never anything** | 52 | 138 | 481 | 126 | 797 |
|  | **never cigarettes** | 39 | 59 | 421 | 101 | 620 |
|  | **never/low anything^d^** | 0 | 0 | 2 | 0 | 2 |
|  | **never/low cigarettes^d^** | 0 | 3 | 8 | 9 | 20 |
|  |  |  |  |  |  |  |
| **N adjusted for** | **0** | 64 | 60 | 511 | 79 | 714 |
|  | **1** | 17 | 84 | 271 | 148 | 520 |
|  | **2** | 0 | 31 | 27 | 0 | 58 |
|  | **3** | 8 | 8 | 22 | 0 | 38 |
|  | **4** | 0 | 0 | 22 | 3 | 25 |
|  | **5** | 0 | 0 | 12 | 6 | 18 |
|  | **6** | 2 | 12 | 36 | 0 | 50 |
|  | **7** | 0 | 5 | 3 | 0 | 8 |
|  | **8** | 0 | 0 | 6 | 0 | 6 |
|  | **13** | 0 | 0 | 2 | 0 | 2 |
|  |  |  |  |  |  |  |
| **Adjusted for :** |  |  |  |  |  |  |
| **sex** |  | 0 | 17 | 86 | 5 | 108 |
| **age** |  | 24 | 140 | 362 | 149 | 675 |
|  |  |  |  |  |  |  |
| **Adjusted for other confounders** | **None** | 78 | 144 | 761 | 221 | 1204 |
|  | **1** | 3 | 39 | 64 | 6 | 112 |
|  | **2** | 8 | 0 | 24 | 0 | 32 |
|  | **3** | 0 | 0 | 16 | 6 | 22 |
|  | **4** | 0 | 9 | 19 | 3 | 31 |
|  | **5** | 2 | 3 | 20 | 0 | 25 |
|  | **6** | 0 | 5 | 3 | 0 | 8 |
|  | **8** | 0 | 0 | 3 | 0 | 3 |
|  | **12** | 0 | 0 | 2 | 0 | 2 |
|  |  |  |  |  |  |  |
| **Unadjusted RRs only :** |  |  |  |  |  |  |
| **Number of cases available** |  | 64 | 60 | 509 | 79 | 712 |
| **Number of controls/at risk/disease free available** |  | 64 | 60 | 490 | 79 | 693 |
| **Whole 2x2 table available** |  | 64 | 60 | 490 | 79 | 693 |
|  |  |  |  |  |  |  |
| **Adjusted RRs only :** |  |  |  |  |  |  |
| **Number of cases available** |  | 23 | 103 | 359 | 143 | 628 |
|  |  |  |  |  |  |  |
| **Relative risk value** | **missing** | 0 | 0 | 3 | 1 | 4 |
|  | **<0.5** | 2 | 4 | 4 | 1 | 11 |
|  | **0.5-<1** | 7 | 9 | 22 | 4 | 42 |
|  | **1 exactly** | 1 | 1 | 3 | 0 | 5 |
|  | **>1-<1.5** | 4 | 21 | 88 | 37 | 150 |
|  | **1.5-<2** | 7 | 19 | 137 | 32 | 195 |
|  | **2-<3** | 32 | 26 | 238 | 62 | 358 |
|  | **3-<4** | 10 | 20 | 160 | 28 | 218 |
|  | **4-<5** | 4 | 16 | 61 | 19 | 100 |
|  | **5-<10** | 11 | 60 | 139 | 31 | 241 |
|  | **10-<20** | 5 | 20 | 45 | 15 | 85 |
|  | **20+** | 8 | 4 | 12 | 6 | 30 |
|  | N | 91 | 200 | 909 | 235 | 1435 |
|  | Median | 2.78 | 4.00 | 2.84 | 2.75 | 2.88 |
|  | Range | 0.24:96.60 | 0.24:42.49 | 0.16:489.54 | 0.50:57.04 | 0.16:489.54 |
|  |  |  |  |  |  |  |
| **CI available** |  | 91 | 197 | 905 | 235 | 1428 |
|  |  |  |  |  |  |  |
| **Derivation of RR^e^** | **original** | 8 | 35 | 33 | 10 | 86 |
|  | **RR/CI from numbers** | 20 | 16 | 226 | 31 | 293 |
|  | **RR/CI recalc from numbers** | 0 | 2 | 4 | 0 | 6 |
|  | **combined smoking levels/sum** | 29 | 31 | 195 | 37 | 292 |
|  | **combined disease levels/sum** | 2 | 0 | 34 | 4 | 40 |
|  | **other combined/sum** | 0 | 3 | 183 | 35 | 221 |
|  | **RR/CI calc using 0.5 for 0** | 16 | 13 | 22 | 5 | 56 |
|  | **significant** | 0 | 0 | 7 | 0 | 7 |
|  | **combined smoking levels (Hamling *et al* [14])** | 0 | 20 | 40 | 6 | 66 |
|  | **combined disease levels (Hamling *et al* [14])** | 0 | 0 | 1 | 2 | 3 |
|  | **other combined (Hamling *et al* [14])** | 0 | 0 | 6 | 1 | 7 |
|  | **adj from orig RRs (mini-meta)** | 0 | 0 | 5 | 0 | 5 |
|  | **combined (Hamling *et al* [14]) then adj minimeta** | 0 | 0 | 2 | 0 | 2 |
|  | **other** | 0 | 33 | 154 | 78 | 265 |
|  | **RR orig CI est from numbers** | 1 | 5 | 0 | 0 | 6 |
|  | **other (CI est from numbers)** | 15 | 42 | 0 | 27 | 84 |
|  |  |  |  |  |  |  |

## Table 2 (continued) COPD

| **COPD RRs^a^** |  | **Study Type^b^** | | | | |
| --- | --- | --- | --- | --- | --- | --- |
| **Variable^c^** | **Levels^c^** | **CC** | **Prosp** | **CrossSec** | **Subsid** | **Total** |
|  |  |  |  |  |  |  |
| **Total** |  | 52 | 162 | 337 | 78 | 629 |
|  |  |  |  |  |  |  |
| **Sex** | **both** | 12 | 22 | 100 | 7 | 141 |
|  | **male** | 24 | 97 | 119 | 45 | 285 |
|  | **female** | 16 | 43 | 118 | 26 | 203 |
|  |  |  |  |  |  |  |
| **Onset** | **prevalence** | 52 | 6 | 337 | 48 | 443 |
|  | **onset** | 0 | 156 | 0 | 30 | 186 |
|  |  |  |  |  |  |  |
| **Exposed group: smoking status** | **ever** | 20 | 50 | 115 | 22 | 207 |
|  | **current** | 19 | 66 | 129 | 34 | 248 |
|  | **ex** | 13 | 46 | 93 | 22 | 174 |
|  |  |  |  |  |  |  |
| **Exposed group: smoking product** | **all** | 16 | 56 | 94 | 25 | 191 |
|  | **cigarettes (+/- other)** | 26 | 70 | 218 | 39 | 353 |
|  | **cigarettes only** | 10 | 36 | 25 | 14 | 85 |
|  |  |  |  |  |  |  |
| **Exposed group:cigarette type** | **all** | 24 | 105 | 243 | 53 | 425 |
|  | **manufactured only** | 12 | 1 | 0 | 0 | 13 |
|  |  |  |  |  |  |  |
| **Unexposed group** | **never anything** | 28 | 117 | 138 | 42 | 325 |
|  | **never cigarettes** | 24 | 42 | 195 | 33 | 294 |
|  | **never/low cigarettes^d^** | 0 | 3 | 4 | 3 | 10 |
|  |  |  |  |  |  |  |
| **N adjusted for** | **none** | 36 | 53 | 198 | 22 | 309 |
|  | **1** | 6 | 61 | 92 | 51 | 210 |
|  | **2** | 0 | 29 | 12 | 0 | 41 |
|  | **3** | 8 | 8 | 3 | 0 | 19 |
|  | **4** | 0 | 0 | 12 | 1 | 13 |
|  | **5** | 0 | 0 | 6 | 4 | 10 |
|  | **6** | 2 | 6 | 9 | 0 | 17 |
|  | **7** | 0 | 5 | 0 | 0 | 5 |
|  | **8** | 0 | 0 | 3 | 0 | 3 |
|  | **13** | 0 | 0 | 2 | 0 | 2 |
|  |  |  |  |  |  |  |
| **Adjusted for :** |  |  |  |  |  |  |
| **sex** |  | 0 | 11 | 23 | 3 | 37 |
| **age** |  | 16 | 109 | 129 | 56 | 310 |
|  |  |  |  |  |  |  |
| **Adjusted for other confounders** | **None** | 42 | 114 | 289 | 73 | 518 |
|  | **1** | 0 | 37 | 13 | 0 | 50 |
|  | **2** | 8 | 0 | 11 | 0 | 19 |
|  | **3** | 0 | 0 | 10 | 4 | 14 |
|  | **4** | 0 | 3 | 3 | 1 | 7 |
|  | **5** | 2 | 3 | 6 | 0 | 11 |
|  | **6** | 0 | 5 | 0 | 0 | 5 |
|  | **8** | 0 | 0 | 3 | 0 | 3 |
|  | **12** | 0 | 0 | 2 | 0 | 2 |
|  |  |  |  |  |  |  |
| **Unadjusted RRs only :** |  |  |  |  |  |  |
| **Number of cases available** |  | 36 | 53 | 197 | 22 | 308 |
| **Number of controls/at risk/disease free available** |  | 36 | 53 | 178 | 22 | 289 |
| **Whole 2x2 table available** |  | 36 | 53 | 178 | 22 | 289 |
|  |  |  |  |  |  |  |
| **Adjusted RRs only :** |  |  |  |  |  |  |
| **Number of cases available** |  | 12 | 85 | 137 | 49 | 283 |
|  |  |  |  |  |  |  |
|  |  |  |  |  |  |  |
| **Relative risk value** | **missing** | 0 | 0 | 3 | 0 | 3 |
|  | **<0.5** | 1 | 4 | 0 | 0 | 5 |
|  | **0.5-<1** | 2 | 7 | 8 | 2 | 19 |
|  | **1 exactly** | 0 | 1 | 0 | 0 | 1 |
|  | **>1-<1.5** | 3 | 17 | 29 | 13 | 62 |
|  | **1.5-<2** | 3 | 14 | 60 | 13 | 90 |
|  | **2-<3** | 15 | 20 | 102 | 14 | 151 |
|  | **3-<4** | 3 | 15 | 44 | 4 | 66 |
|  | **4-<5** | 3 | 15 | 30 | 10 | 58 |
|  | **5-<10** | 10 | 50 | 45 | 12 | 117 |
|  | **10-<20** | 5 | 15 | 10 | 7 | 37 |
|  | **20+** | 7 | 4 | 6 | 3 | 20 |
|  | N | 52 | 162 | 334 | 78 | 626 |
|  | Median | 3.31 | 4.12 | 2.64 | 2.30 | 2.88 |
|  | Range | 0.33:77.17 | 0.24:42.49 | 0.70:43.92 | 0.78:36.86 | 0.24:77.17 |
|  |  |  |  |  |  |  |
| **CI available** |  | 52 | 162 | 332 | 78 | 624 |
|  |  |  |  |  |  |  |
| **Derivation of RR^e^** | **original** | 8 | 25 | 11 | 8 | 52 |
|  | **RR/CI from numbers** | 12 | 14 | 87 | 8 | 121 |
|  | **RR/CI recalc from numbers** | 0 | 2 | 2 | 0 | 4 |
|  | **combined smoking levels/sum** | 9 | 25 | 53 | 5 | 92 |
|  | **combined disease levels/sum** | 1 | 0 | 13 | 4 | 18 |
|  | **other combined/sum** | 0 | 3 | 58 | 2 | 63 |
|  | **RR/CI calc using 0.5 for 0** | 14 | 11 | 8 | 3 | 36 |
|  | **significant** | 0 | 0 | 5 | 0 | 5 |
|  | **combined smoking levels (Hamling *et al* [14])** | 0 | 18 | 15 | 6 | 39 |
|  | **combined disease levels (Hamling *et al* [14])** | 0 | 0 | 1 | 2 | 3 |
|  | **other combined (Hamling *et al* [14])** | 0 | 0 | 6 | 1 | 7 |
|  | **other** | 0 | 27 | 78 | 30 | 135 |
|  | **RR orig CI est from numbers** | 0 | 1 | 0 | 0 | 1 |
|  | **other (CI est from numbers)** | 8 | 36 | 0 | 9 | 53 |
|  |  |  |  |  |  |  |

## Table 2 (continued) CB

| **CB RRs^a^** |  | **Study Type^b^** | | | | |
| --- | --- | --- | --- | --- | --- | --- |
| **Variable^c^** | **Levels^c^** | **CC** | **Prosp** | **CrossSec** | **Subsid** | **Total** |
|  |  |  |  |  |  |  |
| **Sex** | **both** | 12 | 3 | 82 | 5 | 102 |
|  | **male** | 13 | 13 | 199 | 56 | 281 |
|  | **female** | 14 | 7 | 204 | 85 | 310 |
|  |  |  |  |  |  |  |
| **Onset** | **prevalence** | 39 | 0 | 485 | 131 | 655 |
|  | **onset** | 0 | 23 | 0 | 15 | 38 |
|  |  |  |  |  |  |  |
| **Exposed group: smoking status** | **ever** | 18 | 6 | 162 | 45 | 231 |
|  | **current** | 11 | 10 | 184 | 53 | 258 |
|  | **ex** | 10 | 7 | 139 | 48 | 204 |
|  |  |  |  |  |  |  |
| **Exposed group: smoking product** | **all** | 11 | 1 | 199 | 41 | 252 |
|  | **cigarettes (+/- other)** | 22 | 17 | 230 | 76 | 345 |
|  | **cigarettes only** | 6 | 5 | 56 | 29 | 96 |
|  |  |  |  |  |  |  |
| **Exposed group:cigarette type** | **all** | 22 | 22 | 284 | 105 | 433 |
|  | **manufactured only** | 6 | 0 | 2 | 0 | 8 |
|  |  |  |  |  |  |  |
| **Unexposed group** | **never anything** | 24 | 14 | 297 | 75 | 410 |
|  | **never cigarettes** | 15 | 9 | 182 | 65 | 271 |
|  | **never/low anything^d^** | 0 | 0 | 2 | 0 | 2 |
|  | **never/low cigarettes^d^** | 0 | 0 | 4 | 6 | 10 |
|  |  |  |  |  |  |  |
| **N adjusted for** | **none** | 28 | 6 | 260 | 54 | 348 |
|  | **1** | 11 | 13 | 155 | 88 | 267 |
|  | **2** | 0 | 1 | 12 | 0 | 13 |
|  | **3** | 0 | 0 | 13 | 0 | 13 |
|  | **4** | 0 | 0 | 10 | 2 | 12 |
|  | **5** | 0 | 0 | 6 | 2 | 8 |
|  | **6** | 0 | 3 | 23 | 0 | 26 |
|  | **7** | 0 | 0 | 3 | 0 | 3 |
|  | **8** | 0 | 0 | 3 | 0 | 3 |
|  |  |  |  |  |  |  |
| **Adjusted for :** |  |  |  |  |  |  |
| **sex** |  | 0 | 3 | 50 | 2 | 55 |
| **age** |  | 8 | 17 | 202 | 84 | 311 |
|  |  |  |  |  |  |  |
| **Adjusted for other confounders** | **None** | 36 | 19 | 398 | 136 | 589 |
|  | **1** | 3 | 1 | 39 | 6 | 49 |
|  | **2** | 0 | 0 | 13 | 0 | 13 |
|  | **3** | 0 | 0 | 6 | 2 | 8 |
|  | **4** | 0 | 3 | 12 | 2 | 17 |
|  | **5** | 0 | 0 | 14 | 0 | 14 |
|  | **6** | 0 | 0 | 3 | 0 | 3 |
|  |  |  |  |  |  |  |
| **Unadjusted RRs only :** |  |  |  |  |  |  |
| **Number of cases available** |  | 28 | 6 | 259 | 54 | 347 |
| **Number of controls/at risk/disease free available** |  | 28 | 6 | 259 | 54 | 347 |
| **Whole 2x2 table available** |  | 28 | 6 | 259 | 54 | 347 |
|  |  |  |  |  |  |  |
| **Adjusted RRs only :** |  |  |  |  |  |  |
| **Number of cases available** |  | 11 | 9 | 188 | 85 | 293 |
|  |  |  |  |  |  |  |
| **Relative risk value** | **missing** | 0 | 0 | 0 | 1 | 1 |
|  | **<0.5** | 1 | 0 | 4 | 1 | 6 |
|  | **0.5-<1** | 5 | 2 | 6 | 1 | 14 |
|  | **1 exactly** | 1 | 0 | 3 | 0 | 4 |
|  | **>1-<1.5** | 1 | 2 | 54 | 22 | 79 |
|  | **1.5-<2** | 4 | 5 | 65 | 19 | 93 |
|  | **2-<3** | 17 | 3 | 124 | 48 | 192 |
|  | **3-<4** | 7 | 4 | 102 | 24 | 137 |
|  | **4-<5** | 1 | 0 | 27 | 9 | 37 |
|  | **5-<10** | 1 | 3 | 74 | 16 | 94 |
|  | **10-<20** | 0 | 4 | 22 | 2 | 28 |
|  | **20+** | 1 | 0 | 4 | 3 | 8 |
|  | N | 39 | 23 | 485 | 145 | 692 |
|  | Median | 2.62 | 2.85 | 2.91 | 2.75 | 2.82 |
|  | Range | 0.24:96.60 | 0.64:12.50 | 0.16:30.80 | 0.50:57.04 | 0.16:96.60 |
|  |  |  |  |  |  |  |
| **CI available** |  | 39 | 20 | 483 | 145 | 687 |
|  |  |  |  |  |  |  |
| **Derivation of RR^e^** | **original** | 0 | 6 | 19 | 2 | 27 |
|  | **RR/CI from numbers** | 8 | 2 | 114 | 22 | 146 |
|  | **RR/CI recalc from numbers** | 0 | 0 | 2 | 0 | 2 |
|  | **combined smoking levels/sum** | 20 | 6 | 114 | 30 | 170 |
|  | **combined disease levels/sum** | 1 | 0 | 20 | 0 | 21 |
|  | **other combined/sum** | 0 | 0 | 110 | 33 | 143 |
|  | **RR/CI calc using 0.5 for 0** | 2 | 1 | 7 | 2 | 12 |
|  | **significant** | 0 | 0 | 2 | 0 | 2 |
|  | **combined smoking levels (Hamling *et al* [14])** | 0 | 1 | 22 | 0 | 23 |
|  | **adj from orig RRs (mini-meta)** | 0 | 0 | 5 | 0 | 5 |
|  | **combined (Hamling *et al* [14]) then adj minimeta** | 0 | 0 | 2 | 0 | 2 |
|  | **other** | 0 | 4 | 68 | 48 | 120 |
|  | **RR orig CI est from numbers** | 1 | 2 | 0 | 0 | 3 |
|  | **other (CI est from numbers)** | 7 | 1 | 0 | 9 | 17 |
|  |  |  |  |  |  |  |

## Table 2 (continued) Emphysema

| **Emphysema RRs^a^** |  | **Study Type^b^** | | | | |
| --- | --- | --- | --- | --- | --- | --- |
| **Variable^c^** | **Levels^c^** | **CC** | **Prosp** | **CrossSec** | **Subsid** | **Total** |
|  |  |  |  |  |  |  |
| **Total** |  | - | 15 | 90 | 12 | 117 |
|  |  |  |  |  |  |  |
| **Sex** | **both** | - | 3 | 18 | 0 | 21 |
|  | **male** | - | 8 | 36 | 12 | 56 |
|  | **female** | - | 4 | 36 | 0 | 40 |
|  |  |  |  |  |  |  |
| **Onset** | **prevalence** | - | 0 | 90 | 0 | 90 |
|  | **onset** | - | 15 | 0 | 12 | 27 |
|  |  |  |  |  |  |  |
| **Exposed group: smoking status** | **ever** | - | 6 | 39 | 4 | 49 |
|  | **current** | - | 6 | 30 | 4 | 40 |
|  | **ex** | - | 3 | 21 | 4 | 28 |
|  |  |  |  |  |  |  |
| **Exposed group: smoking product** | **all** | - | 1 | 28 | 3 | 32 |
|  | **cigarettes (+/- other)** | - | 11 | 51 | 6 | 68 |
|  | **cigarettes only** | - | 3 | 11 | 3 | 17 |
|  |  |  |  |  |  |  |
| **Exposed group:cigarette type** | **all** | - | 14 | 62 | 9 | 85 |
|  |  |  |  |  |  |  |
| **Unexposed group** | **never anything** | - | 7 | 46 | 9 | 62 |
|  | **never cigarettes** | - | 8 | 44 | 3 | 55 |
|  |  |  |  |  |  |  |
| **N adjusted for** | **none** | - | 1 | 53 | 3 | 57 |
|  | **1** | - | 10 | 24 | 9 | 43 |
|  | **2** | - | 1 | 3 | 0 | 4 |
|  | **3** | - | 0 | 6 | 0 | 6 |
|  | **6** | - | 3 | 4 | 0 | 7 |
|  |  |  |  |  |  |  |
| **Adjusted for :** |  |  |  |  |  |  |
| **sex** |  | - | 3 | 13 | 0 | 16 |
| **age** |  | - | 14 | 31 | 9 | 54 |
|  |  |  |  |  |  |  |
| **Adjusted for other confounders** | **None** | - | 11 | 74 | 12 | 97 |
|  | **1** | - | 1 | 12 | 0 | 13 |
|  | **4** | - | 3 | 4 | 0 | 7 |
|  |  |  |  |  |  |  |
| **Unadjusted RRs only :** |  |  |  |  |  |  |
| **Number of cases available** |  | - | 1 | 53 | 3 | 57 |
| **Number of controls/at risk/disease free available** |  | - | 1 | 53 | 3 | 57 |
| **Whole 2x2 table available** |  | - | 1 | 53 | 3 | 57 |
|  |  |  |  |  |  |  |
| **Adjusted RRs only :** |  |  |  |  |  |  |
| **Number of cases available** |  | - | 9 | 34 | 9 | 52 |
|  |  |  |  |  |  |  |
| **Relative risk value** |  |  |  |  |  |  |
|  | **0.5-<1** | - | 0 | 8 | 1 | 9 |
|  | **>1-<1.5** | - | 2 | 5 | 2 | 9 |
|  | **1.5-<2** | - | 0 | 12 | 0 | 12 |
|  | **2-<3** | - | 3 | 12 | 0 | 15 |
|  | **3-<4** | - | 1 | 14 | 0 | 15 |
|  | **4-<5** | - | 1 | 4 | 0 | 5 |
|  | **5-<10** | - | 7 | 20 | 3 | 30 |
|  | **10-<20** | - | 1 | 13 | 6 | 20 |
|  | **20+** | - | 0 | 2 | 0 | 2 |
|  | N | 0 | 15 | 90 | 12 | 117 |
|  | Median | - | 5.90 | 3.59 | 10.06 | 3.95 |
|  | Range | - | 1.06:12.50 | 0.52:489.54 | 0.55:14.17 | 0.52:489.54 |
|  |  |  |  |  |  |  |
| **CI available** | **present** | - | 15 | 90 | 12 | 117 |
|  |  |  |  |  |  |  |
| **Derivation of RR^e^** | **original** | - | 4 | 3 | 0 | 7 |
|  | **RR/CI from numbers** | - | 0 | 25 | 1 | 26 |
|  | **combined smoking levels/sum** | - | 0 | 28 | 2 | 30 |
|  | **combined disease levels/sum** | - | 0 | 1 | 0 | 1 |
|  | **other combined/sum** | - | 0 | 15 | 0 | 15 |
|  | **RR/CI calc using 0.5 for 0** | - | 1 | 7 | 0 | 8 |
|  | **combined smoking levels (Hamling *et al* [14])** | - | 1 | 3 | 0 | 4 |
|  | **other** | - | 2 | 8 | 0 | 10 |
|  | **RR orig CI est from numbers** | - | 2 | 0 | 0 | 2 |
|  | **other (CI est from numbers)** | - | 5 | 0 | 9 | 14 |

^a^ All RRs are counted in the first section of the Table. In subsequent sections, RRs for each of the disease outcomes are counted.

^b^ Principal studies are categorized as CC (case-control, including nested CC), Prosp (prospective) or CrossSec (cross-sectional). Subsidiary studies are categorized separately.

^c^ For presence/absence variables, the “Levels” column is left blank and counts for only the “presence” level are shown. “Not applicable” levels are not shown (e.g. cigarette type where the exposed group is any product)

^d^ See footnote f to Table 2 in main paper.

^e^ See Additional file 1 (*Derivation of RRs*) for further explanation.

## Table 3 Characteristics of the relative risks for the dose-response indices

| **All RRs^a^** |  | **Study Type^b^** | | | | |
| --- | --- | --- | --- | --- | --- | --- |
| **Variable^c^** | **Levels^c^** | **CC** | **Prosp** | **CrossSec** | **Subsid** | **Total** |
|  |  |  |  |  |  |  |
| **Total** |  | 265 | 366 | 1029 | 439 | 2099 |
|  |  |  |  |  |  |  |
| **Sex** | **both** | 25 | 6 | 256 | 26 | 313 |
|  | **male** | 117 | 280 | 420 | 249 | 1066 |
|  | **female** | 123 | 80 | 353 | 164 | 720 |
|  |  |  |  |  |  |  |
| **Onset** | **prevalence** | 265 | 3 | 1029 | 261 | 1558 |
|  | **onset** | 0 | 363 | 0 | 178 | 541 |
|  |  |  |  |  |  |  |
| **Exposed group: smoking status** | **ever** | 108 | 20 | 250 | 92 | 470 |
|  | **current** | 74 | 244 | 672 | 282 | 1272 |
|  | **ex** | 83 | 102 | 107 | 65 | 357 |
|  |  |  |  |  |  |  |
| **Exposed group: smoking product** | **all** | 50 | 16 | 182 | 47 | 295 |
|  | **cigarettes (+/- other)** | 99 | 209 | 685 | 278 | 1271 |
|  | **cigarettes only** | 116 | 141 | 162 | 114 | 533 |
|  |  |  |  |  |  |  |
| **Exposed group:cigarette type** | **all** | 99 | 350 | 814 | 392 | 1655 |
|  | **manufactured only** | 116 | 0 | 33 | 0 | 149 |
|  |  |  |  |  |  |  |
| **Exposed group: dose response** | **level 1** | 42 | 63 | 211 | 81 | 397 |
|  | **level 2** | 87 | 137 | 422 | 156 | 802 |
|  | **level 3** | 83 | 100 | 255 | 120 | 558 |
|  | **level 4** | 24 | 40 | 44 | 58 | 166 |
|  | **level 5** | 7 | 15 | 29 | 15 | 66 |
|  | **level 6** | 0 | 5 | 10 | 4 | 19 |
|  | **level 7** | 0 | 0 | 4 | 0 | 4 |
|  | **per unit dose regression** | 0 | 0 | 21 | 0 | 21 |
|  | **dose response other** | 18 | 4 | 28 | 3 | 53 |
|  | **dose response partial (vs no)** | 4 | 2 | 5 | 2 | 13 |
|  |  |  |  |  |  |  |
| **Measure of exposure** | **cigarettes/day** | 88 | 186 | 600 | 335 | 1209 |
|  | **years of age started** | 65 | 36 | 33 | 11 | 145 |
|  | **pack-years** | 28 | 23 | 277 | 62 | 390 |
|  | **years duration smoking** | 3 | 35 | 35 | 5 | 78 |
|  | **years since quit (v never/long-term)** | 41 | 47 | 46 | 13 | 147 |
|  | **years since quit (v current/recent)** | 40 | 39 | 38 | 13 | 130 |
|  |  |  |  |  |  |  |
| **Unexposed group** | **never anything** | 87 | 102 | 272 | 148 | 609 |
|  | **never cigarettes** | 26 | 77 | 272 | 110 | 485 |
|  | **never/low^d^ cigarettes** | 0 | 0 | 4 | 4 | 8 |
|  | **low^d^ any** | 21 | 8 | 66 | 17 | 112 |
|  | **low^d^ cigs** | 83 | 153 | 320 | 148 | 704 |
|  | **non any** | 0 | 0 | 14 | 0 | 14 |
|  | **non cigs** | 6 | 5 | 8 | 0 | 19 |
|  | **current anything** | 6 | 0 | 8 | 8 | 22 |
|  | **current cigarettes** | 18 | 17 | 16 | 1 | 52 |
|  |  |  |  |  |  |  |
| **N adjusted for** | **none** | 167 | 58 | 515 | 101 | 841 |
|  | **1** | 68 | 197 | 389 | 308 | 962 |
|  | **2** | 6 | 91 | 34 | 24 | 155 |
|  | **3** | 22 | 16 | 14 | 6 | 58 |
|  | **4** | 0 | 0 | 14 | 0 | 14 |
|  | **5** | 0 | 1 | 10 | 0 | 11 |
|  | **6** | 2 | 3 | 37 | 0 | 42 |
|  | **7** | 0 | 0 | 1 | 0 | 1 |
|  | **8** | 0 | 0 | 3 | 0 | 3 |
|  | **9** | 0 | 0 | 2 | 0 | 2 |
|  | **13** | 0 | 0 | 10 | 0 | 10 |
| **Adjusted for** |  |  |  |  |  |  |
| **sex** |  | 0 | 3 | 63 | 20 | 86 |
| **age** |  | 98 | 308 | 450 | 328 | 1184 |
|  |  |  |  |  |  |  |
| **Adjusted for other aspects of smoking** | **1** | 6 | 20 | 31 | 6 | 63 |
|  | **4** | 0 | 0 | 2 | 0 | 2 |
|  |  |  |  |  |  |  |
| **Adjusted for other confounders** | **None** | 241 | 262 | 887 | 419 | 1809 |
|  | **1** | 0 | 100 | 61 | 20 | 181 |
|  | **2** | 22 | 0 | 17 | 0 | 39 |
|  | **3** | 0 | 0 | 13 | 0 | 13 |
|  | **4** | 0 | 1 | 12 | 0 | 13 |
|  | **5** | 2 | 3 | 24 | 0 | 29 |
|  | **6** | 0 | 0 | 3 | 0 | 3 |
|  | **7** | 0 | 0 | 2 | 0 | 2 |
|  | **12** | 0 | 0 | 10 | 0 | 10 |
|  |  |  |  |  |  |  |
| **Unadjusted RRs only** |  |  |  |  |  |  |
| **Number of cases available** |  | 149 | 44 | 486 | 98 | 777 |
| **Number of controls/at risk/disease free available** |  | 149 | 44 | 455 | 98 | 746 |
| **Whole 2x2 table available** |  | 149 | 44 | 455 | 98 | 746 |
|  |  |  |  |  |  |  |
| **Adjusted RRs only** |  |  |  |  |  |  |
| **Number of cases available** |  | 88 | 262 | 451 | 306 | 1107 |
|  |  |  |  |  |  |  |
| **Relative risk value** | **missing** | 18 | 5 | 39 | 5 | 67 |
|  | **<0.5** | 8 | 24 | 12 | 7 | 51 |
|  | **0.5-<1** | 47 | 52 | 70 | 15 | 184 |
|  | **1 exactly** | 0 | 2 | 3 | 1 | 6 |
|  | **>1-<1.5** | 51 | 38 | 141 | 42 | 272 |
|  | **1.5-<2** | 35 | 39 | 149 | 36 | 259 |
|  | **2-<3** | 56 | 49 | 207 | 85 | 397 |
|  | **3-<4** | 28 | 37 | 87 | 68 | 220 |
|  | **4-<5** | 6 | 25 | 68 | 57 | 156 |
|  | **5-<10** | 10 | 54 | 126 | 67 | 257 |
|  | **10-<20** | 6 | 32 | 91 | 49 | 178 |
|  | **20+** | 0 | 9 | 36 | 7 | 52 |
|  | N | 247 | 361 | 990 | 434 | 2032 |
|  | Median | 1.76 | 2.50 | 2.53 | 3.33 | 2.57 |
|  | Range | 0.39:18.53 | 0.07:32.00 | 0.26:4124.26 | 0.14:60.54 | 0.07:4124.26 |
|  |  |  |  |  |  |  |
| **CI available** |  | 247 | 342 | 988 | 422 | 1999 |
|  |  |  |  |  |  |  |
| **Derivation of RR^e^** | **original** | 12 | 44 | 84 | 25 | 165 |
|  | **RR/CI from numbers** | 106 | 35 | 354 | 100 | 595 |
|  | **RR/CI recalc from numbers** | 2 | 3 | 1 | 0 | 6 |
|  | **combined smoking levels/sum** | 35 | 2 | 33 | 0 | 70 |
|  | **combined disease levels/sum** | 0 | 0 | 59 | 2 | 61 |
|  | **other combined/sum** | 6 | 0 | 185 | 37 | 228 |
|  | **RR/CI calc using 0.5 for 0** | 0 | 15 | 13 | 3 | 31 |
|  | **non-significant** | 2 | 1 | 3 | 4 | 10 |
|  | **significant** | 10 | 0 | 6 | 1 | 17 |
|  | **read from graph/chart** | 0 | 7 | 0 | 0 | 7 |
|  | **RR original, CI from P-value** | 0 | 1 | 0 | 0 | 1 |
|  | **combined smoking levels (Hamling *et al* [14])** | 0 | 30 | 29 | 16 | 75 |
|  | **combined disease levels (Hamling *et al* [14])** | 0 | 0 | 3 | 0 | 3 |
|  | **other combined (Hamling *et al* [14])** | 0 | 0 | 6 | 0 | 6 |
|  | **adj from orig RRs (mini-meta)** | 0 | 0 | 2 | 0 | 2 |
|  | **combined (Hamling *et al*  [14]) then adj minimeta** | 0 | 0 | 1 | 0 | 1 |
|  | **other** | 10 | 120 | 250 | 133 | 513 |
|  | **RR orig CI est from numbers** | 5 | 20 | 0 | 0 | 25 |
|  | **other (CI est from numbers)** | 77 | 88 | 0 | 118 | 283 |

## Table 3 (continued) COPD

| **COPD RRs^a^** |  | **Study Type^b^** | | | | |
| --- | --- | --- | --- | --- | --- | --- |
| **Variable^c^** | **Levels^c^** | **CC** | **Prosp** | **CrossSec** | **Subsid** | **Total** |
| **Total** |  | 142 | 252 | 397 | 158 | 949 |
|  |  |  |  |  |  |  |
| **Sex** | **both** | 18 | 6 | 131 | 21 | 176 |
|  | **male** | 76 | 202 | 142 | 96 | 516 |
|  | **female** | 48 | 44 | 124 | 41 | 257 |
|  |  |  |  |  |  |  |
| **Onset** | **prevalence** | 142 | 3 | 397 | 72 | 614 |
|  | **onset** | 0 | 249 | 0 | 86 | 335 |
|  |  |  |  |  |  |  |
| **Exposed group: smoking status** | **ever** | 26 | 12 | 183 | 39 | 260 |
|  | **current** | 53 | 167 | 195 | 91 | 506 |
|  | **ex** | 63 | 73 | 19 | 28 | 183 |
|  |  |  |  |  |  |  |
| **Exposed group: smoking product** | **all** | 20 | 12 | 32 | 12 | 76 |
|  | **cigarettes (+/- other)** | 42 | 146 | 337 | 105 | 630 |
|  | **cigarettes only** | 80 | 94 | 28 | 41 | 243 |
|  |  |  |  |  |  |  |
| **Exposed group:cigarette type** | **all** | 42 | 240 | 365 | 146 | 793 |
|  | **manufactured only** | 80 | 0 | 0 | 0 | 80 |
|  |  |  |  |  |  |  |
| **Exposed group: dose response** | **level 1** | 22 | 41 | 79 | 23 | 165 |
|  | **level 2** | 48 | 95 | 157 | 50 | 350 |
|  | **level 3** | 46 | 68 | 90 | 38 | 242 |
|  | **level 4** | 6 | 27 | 24 | 26 | 83 |
|  | **level 5** | 0 | 11 | 17 | 12 | 40 |
|  | **level 6** | 0 | 5 | 7 | 4 | 16 |
|  | **level 7** | 0 | 0 | 1 | 0 | 1 |
|  | **per unit dose regression** | 0 | 0 | 12 | 0 | 12 |
|  | **dose response other** | 16 | 3 | 7 | 3 | 29 |
|  | **dose response partial (vs no)** | 4 | 2 | 3 | 2 | 11 |
|  |  |  |  |  |  |  |
| **Measure of exposure** | **cigarettes/day** | 27 | 143 | 165 | 102 | 437 |
|  | **years of age started** | 27 | 30 | 21 | 7 | 85 |
|  | **pack-years** | 24 | 13 | 188 | 30 | 255 |
|  | **years duration smoking** | 3 | 9 | 5 | 5 | 22 |
|  | **years since quit (v never/long-term)** | 31 | 32 | 9 | 7 | 79 |
|  | **years since quit (v current/recent)** | 30 | 25 | 9 | 7 | 71 |
|  |  |  |  |  |  |  |
| **Unexposed group** | **never anything** | 38 | 75 | 83 | 39 | 235 |
|  | **never cigarettes** | 9 | 42 | 142 | 42 | 235 |
|  | **never/low^d^ cigarettes** | 0 | 0 | 2 | 4 | 6 |
|  | **low^d^ any** | 8 | 6 | 13 | 4 | 31 |
|  | **low^d^ cigs** | 47 | 112 | 132 | 61 | 352 |
|  | **non cigs** | 6 | 5 | 0 | 0 | 11 |
|  | **current anything** | 6 | 0 | 2 | 4 | 12 |
|  | **current cigarettes** | 12 | 9 | 4 | 1 | 26 |
|  |  |  |  |  |  |  |
| **N adjusted for** | **none** | 80 | 57 | 181 | 28 | 346 |
|  | **1** | 32 | 84 | 153 | 108 | 377 |
|  | **2** | 6 | 91 | 7 | 20 | 124 |
|  | **3** | 22 | 16 | 9 | 2 | 49 |
|  | **4** | 0 | 0 | 11 | 0 | 11 |
|  | **5** | 0 | 1 | 5 | 0 | 6 |
|  | **6** | 2 | 3 | 19 | 0 | 24 |
|  | **9** | 0 | 0 | 2 | 0 | 2 |
|  | **13** | 0 | 0 | 10 | 0 | 10 |
|  |  |  |  |  |  |  |
| **Adjusted for** |  |  |  |  |  |  |
| **sex** |  | 0 | 3 | 33 | 20 | 56 |
| **age** |  | 62 | 195 | 207 | 130 | 594 |
|  |  |  |  |  |  |  |
| **Adjusted for other aspects of smoking** | **None** | 136 | 232 | 392 | 156 | 916 |
|  | **1** | 6 | 20 | 3 | 2 | 31 |
|  | **4** | 0 | 0 | 2 | 0 | 2 |
|  |  |  |  |  |  |  |
| **Adjusted for other confounders** | **None** | 118 | 148 | 340 | 156 | 762 |
|  | **1** | 0 | 100 | 6 | 2 | 108 |
|  | **2** | 22 | 0 | 14 | 0 | 36 |
|  | **3** | 0 | 0 | 8 | 0 | 8 |
|  | **4** | 0 | 1 | 3 | 0 | 4 |
|  | **5** | 2 | 3 | 14 | 0 | 19 |
|  | **7** | 0 | 0 | 2 | 0 | 2 |
|  | **12** | 0 | 0 | 10 | 0 | 10 |
|  |  |  |  |  |  |  |
| **Unadjusted RRs only** |  |  |  |  |  |  |
| **Number of cases available** |  | 64 | 44 | 168 | 25 | 301 |
| **Number of controls/at risk/disease free available** |  | 64 | 44 | 137 | 25 | 270 |
| **Whole 2x2 table available** |  | 64 | 44 | 137 | 25 | 270 |
|  |  |  |  |  |  |  |
| **Adjusted RRs only** |  |  |  |  |  |  |
| **Number of cases available** |  | 52 | 190 | 181 | 106 | 529 |
|  |  |  |  |  |  |  |
| **Relative risk value** | **missing** | 16 | 3 | 12 | 3 | 34 |
|  | **<0.5** | 6 | 15 | 7 | 4 | 32 |
|  | **0.5-<1** | 27 | 38 | 20 | 9 | 94 |
|  | **1 exactly** | 0 | 0 | 2 | 1 | 3 |
|  | **>1-<1.5** | 35 | 23 | 67 | 10 | 135 |
|  | **1.5-<2** | 13 | 25 | 53 | 17 | 108 |
|  | **2-<3** | 28 | 34 | 81 | 29 | 172 |
|  | **3-<4** | 7 | 24 | 39 | 21 | 91 |
|  | **4-<5** | 1 | 20 | 28 | 15 | 64 |
|  | **5-<10** | 3 | 43 | 47 | 24 | 117 |
|  | **10-<20** | 6 | 19 | 37 | 22 | 84 |
|  | **20+** | 0 | 8 | 4 | 3 | 15 |
|  | N | 126 | 249 | 385 | 155 | 915 |
|  | Median | 1.35 | 2.62 | 2.56 | 3.33 | 2.46 |
|  | Range | 0.39:18.53 | 0.07:32.00 | 0.30:65.73 | 0.14:55.36 | 0.07:65.73 |
|  |  |  |  |  |  |  |
| **CI available** | **present** | 126 | 235 | 383 | 151 | 895 |
|  |  |  |  |  |  |  |
| **Derivation of RR^e^** | **original** | 10 | 27 | 41 | 24 | 102 |
|  | **RR/CI from numbers** | 29 | 35 | 110 | 19 | 193 |
|  | **RR/CI recalc from numbers** | 2 | 3 | 0 | 0 | 5 |
|  | **combined smoking levels/sum** | 30 | 2 | 5 | 0 | 37 |
|  | **combined disease levels/sum** | 0 | 0 | 13 | 0 | 13 |
|  | **other combined/sum** | 3 | 0 | 47 | 0 | 50 |
|  | **RR/CI calc using 0.5 for 0** | 0 | 13 | 4 | 2 | 19 |
|  | **non-significant** | 2 | 1 | 3 | 4 | 10 |
|  | **significant** | 10 | 0 | 4 | 1 | 15 |
|  | **RR original, CI from P-value** | 0 | 1 | 0 | 0 | 1 |
|  | **combined smoking levels (Hamling *et al* [14])** | 0 | 24 | 20 | 16 | 60 |
|  | **combined disease levels (Hamling *et al* [14])** | 0 | 0 | 3 | 0 | 3 |
|  | **other combined (Hamling *et al* [14])** | 0 | 0 | 6 | 0 | 6 |
|  | **other** | 10 | 88 | 141 | 50 | 289 |
|  | **RR orig CI est from numbers** | 5 | 8 | 0 | 0 | 13 |
|  | **other (CI est from numbers)** | 41 | 50 | 0 | 42 | 133 |

## Table 3 (continued) CB

| **CB RRs^a^** |  | **Study Type^b^** | | | | |
| --- | --- | --- | --- | --- | --- | --- |
| **Variable^c^** | **Levels^c^** | **CC** | **Prosp** | **CrossSec** | **Subsid** | **Total** |
| **Total** |  | 123 | 63 | 578 | 232 | 996 |
|  |  |  |  |  |  |  |
| **Sex** | **both** | 7 | 0 | 111 | 5 | 123 |
|  | **male** | 41 | 43 | 243 | 104 | 431 |
|  | **female** | 75 | 20 | 224 | 123 | 442 |
|  |  |  |  |  |  |  |
| **Onset** | **prevalence** | 123 | 0 | 578 | 189 | 890 |
|  | **onset** | 0 | 63 | 0 | 43 | 106 |
|  |  |  |  |  |  |  |
| **Exposed group: smoking status** | **ever** | 82 | 0 | 56 | 39 | 177 |
|  | **current** | 21 | 35 | 436 | 170 | 662 |
|  | **ex** | 20 | 28 | 86 | 23 | 157 |
|  |  |  |  |  |  |  |
| **Exposed group: smoking product** | **all** | 30 | 1 | 145 | 35 | 211 |
|  | **cigarettes (+/- other)** | 57 | 29 | 313 | 145 | 544 |
|  | **cigarettes only** | 36 | 33 | 120 | 52 | 241 |
|  |  |  |  |  |  |  |
| **Exposed group:cigarette type** | **all** | 57 | 62 | 400 | 197 | 716 |
|  | **manufactured only** | 36 | 0 | 33 | 0 | 69 |
|  |  |  |  |  |  |  |
| **Exposed group: dose response** | **level 1** | 20 | 10 | 122 | 51 | 203 |
|  | **level 2** | 39 | 21 | 241 | 92 | 393 |
|  | **level 3** | 37 | 20 | 151 | 68 | 276 |
|  | **level 4** | 18 | 10 | 20 | 18 | 66 |
|  | **level 5** | 7 | 2 | 12 | 3 | 24 |
|  | **level 6** | 0 | 0 | 3 | 0 | 3 |
|  | **level 7** | 0 | 0 | 3 | 0 | 3 |
|  | **per unit dose regression** | 0 | 0 | 9 | 0 | 9 |
|  | **dose response other** | 2 | 0 | 15 | 0 | 17 |
|  | **dose response partial (vs no)** | 0 | 0 | 2 | 0 | 2 |
|  |  |  |  |  |  |  |
| **Measure of exposure** | **cigarettes/day** | 61 | 21 | 392 | 184 | 658 |
|  | **years of age started** | 38 | 0 | 12 | 4 | 54 |
|  | **pack-years** | 4 | 0 | 84 | 32 | 120 |
|  | **years duration smoking** | 0 | 14 | 25 | 0 | 39 |
|  | **years since quit (v never/long-term)** | 10 | 14 | 36 | 6 | 66 |
|  | **years since quit (v current/recent)** | 10 | 14 | 29 | 6 | 59 |
|  |  |  |  |  |  |  |
| **Unexposed group** | **no section** | 2 | 0 | 24 | 0 | 26 |
|  | **never anything** | 49 | 15 | 165 | 85 | 314 |
|  | **never cigarettes** | 17 | 13 | 128 | 64 | 222 |
|  | **never/low^d^ cigarettes** | 0 | 0 | 2 | 0 | 2 |
|  | **low^d^ any** | 13 | 1 | 51 | 13 | 78 |
|  | **low^d^ cigs** | 36 | 26 | 168 | 66 | 296 |
|  | **non any** | 0 | 0 | 14 | 0 | 14 |
|  | **non cigs** | 0 | 0 | 8 | 0 | 8 |
|  | **current anything** | 0 | 0 | 6 | 4 | 10 |
|  | **current cigarettes** | 6 | 8 | 12 | 0 | 26 |
|  |  |  |  |  |  |  |
| **N adjusted for** | **none** | 87 | 0 | 296 | 66 | 449 |
|  | **1** | 36 | 63 | 224 | 158 | 481 |
|  | **2** | 0 | 0 | 26 | 4 | 30 |
|  | **3** | 0 | 0 | 5 | 4 | 9 |
|  | **4** | 0 | 0 | 3 | 0 | 3 |
|  | **5** | 0 | 0 | 5 | 0 | 5 |
|  | **6** | 0 | 0 | 15 | 0 | 15 |
|  | **7** | 0 | 0 | 1 | 0 | 1 |
|  | **8** | 0 | 0 | 3 | 0 | 3 |
|  |  |  |  |  |  |  |
| **Adjusted for** |  |  |  |  |  |  |
| **sex** |  | 0 | 0 | 26 | 0 | 26 |
| **age** |  | 36 | 63 | 227 | 156 | 482 |
|  |  |  |  |  |  |  |
| **Adjusted for other aspects of smoking** | **None** | 123 | 63 | 550 | 228 | 964 |
|  | **1** | 0 | 0 | 28 | 4 | 32 |
|  |  |  |  |  |  |  |
| **Adjusted for other confounders** | **None** | 123 | 63 | 496 | 214 | 896 |
|  | **1** | 0 | 0 | 55 | 18 | 73 |
|  | **2** | 0 | 0 | 3 | 0 | 3 |
|  | **3** | 0 | 0 | 5 | 0 | 5 |
|  | **4** | 0 | 0 | 6 | 0 | 6 |
|  | **5** | 0 | 0 | 10 | 0 | 10 |
|  | **6** | 0 | 0 | 3 | 0 | 3 |
|  |  |  |  |  |  |  |
| **Unadjusted RRs only** |  |  |  |  |  |  |
| **Number of cases available** |  | 85 | - | 282 | 66 | 433 |
| **Number of controls/at risk/disease free available** |  | 85 | - | 282 | 66 | 433 |
| **Whole 2x2 table available** |  | 85 | - | 282 | 66 | 433 |
|  |  |  |  |  |  |  |
| **Adjusted RRs only** |  |  |  |  |  |  |
| **Number of cases available** |  | 36 | 34 | 258 | 158 | 486 |
|  |  |  |  |  |  |  |
| **Relative risk value** | **missing** | 2 | 0 | 21 | 2 | 25 |
|  | **<0.5** | 2 | 9 | 3 | 2 | 16 |
|  | **0.5-<1** | 20 | 11 | 38 | 6 | 75 |
|  | **1 exactly** | 0 | 2 | 1 | 0 | 3 |
|  | **>1-<1.5** | 16 | 5 | 73 | 32 | 126 |
|  | **1.5-<2** | 22 | 8 | 91 | 17 | 138 |
|  | **2-<3** | 28 | 7 | 125 | 51 | 211 |
|  | **3-<4** | 21 | 5 | 46 | 36 | 108 |
|  | **4-<5** | 5 | 1 | 39 | 35 | 80 |
|  | **5-<10** | 7 | 6 | 75 | 38 | 126 |
|  | **10-<20** | 0 | 8 | 46 | 11 | 65 |
|  | **20+** | 0 | 1 | 20 | 2 | 23 |
|  | N | 121 | 63 | 557 | 230 | 971 |
|  | Median | 2.16 | 1.75 | 2.49 | 3.25 | 2.54 |
|  | Range | 0.40:7.58 | 0.08:21.20 | 0.26:172.67 | 0.42:60.54 | 0.08:172.67 |
|  |  |  |  |  |  |  |
| **CI available** | **present** | 121 | 62 | 557 | 222 | 962 |
|  |  |  |  |  |  |  |
| **Derivation of RR^e^** | **original** | 2 | 6 | 35 | 1 | 44 |
|  | **RR/CI from numbers** | 77 | 0 | 209 | 74 | 360 |
|  | **RR/CI recalc from numbers** | 0 | 0 | 1 | 0 | 1 |
|  | **combined smoking levels/sum** | 5 | 0 | 26 | 0 | 31 |
|  | **combined disease levels/sum** | 0 | 0 | 46 | 2 | 48 |
|  | **other combined/sum** | 3 | 0 | 134 | 37 | 174 |
|  | **RR/CI calc using 0.5 for 0** | 0 | 0 | 5 | 1 | 6 |
|  | **significant** | 0 | 0 | 2 | 0 | 2 |
|  | **read from graph/chart** | 0 | 7 | 0 | 0 | 7 |
|  | **combined smoking levels (Hamling *et al* [14])** | 0 | 4 | 8 | 0 | 12 |
|  | **adj from orig RRs (mini-meta)** | 0 | 0 | 2 | 0 | 2 |
|  | **combined (Hamling *et al* [14]) then adj minimeta** | 0 | 0 | 1 | 0 | 1 |
|  | **other** | 0 | 18 | 109 | 83 | 210 |
|  | **RR orig CI est from numbers** | 0 | 6 | 0 | 0 | 6 |
|  | **other (CI est from numbers)** | 36 | 22 | 0 | 34 | 92 |

## Table 3 (continued) Emphysema

| **Emphysema RRs^a^** |  | **Study Type^b^** | | | | |
| --- | --- | --- | --- | --- | --- | --- |
| **Variable^c^** | **Levels^c^** | **CC** | **Prosp** | **CrossSec** | **Subsid** | **Total** |
| **Total** |  | - | 51 | 54 | 49 | 154 |
|  |  |  |  |  |  |  |
| **Sex** | **both** | - | 0 | 14 | 0 | 14 |
|  | **male** | - | 35 | 35 | 49 | 119 |
|  | **female** | - | 16 | 5 | 0 | 21 |
|  |  |  |  |  |  |  |
| **Onset** | **prevalence** | - | 0 | 54 | 0 | 54 |
|  | **onset** | - | 51 | 0 | 49 | 100 |
|  |  |  |  |  |  |  |
| **Exposed group: smoking status** | **ever** | - | 8 | 11 | 14 | 33 |
|  | **current** | - | 42 | 41 | 21 | 104 |
|  | **ex** | - | 1 | 2 | 14 | 17 |
|  |  |  |  |  |  |  |
| **Exposed group: smoking product** | **all** | - | 3 | 5 | 0 | 8 |
|  | **cigarettes (+/- other)** | - | 34 | 35 | 28 | 97 |
|  | **cigarettes only** | - | 14 | 14 | 21 | 49 |
|  |  |  |  |  |  |  |
| **Exposed group:cigarette type** | **all** | - | 48 | 49 | 49 | 146 |
|  |  |  |  |  |  |  |
| **Exposed group: dose response** | **level 1** | - | 12 | 10 | 7 | 29 |
|  | **level 2** | - | 21 | 24 | 14 | 59 |
|  | **level 3** | - | 12 | 14 | 14 | 40 |
|  | **level 4** | - | 3 | 0 | 14 | 17 |
|  | **level 5** | - | 2 | 0 | 0 | 2 |
|  | **dose response other** | - | 1 | 6 | 0 | 7 |
|  |  |  |  |  |  |  |
| **Measure of exposure** | **cigarettes/day** | - | 22 | 43 | 49 | 114 |
|  | **years of age started** | - | 6 | 0 | 0 | 6 |
|  | **pack-years** | - | 10 | 5 | 0 | 15 |
|  | **years duration smoking** | - | 12 | 5 | 0 | 17 |
|  | **years since quit (v never/long-term)** | - | 1 | 1 | 0 | 2 |
|  |  |  |  |  |  |  |
| **Unexposed group** | **never anything** | - | 12 | 24 | 24 | 60 |
|  | **never cigarettes** | - | 22 | 2 | 4 | 28 |
|  | **low^d^ any** | - | 1 | 2 | 0 | 3 |
|  | **low^d^ cigs** | - | 15 | 20 | 21 | 56 |
|  |  |  |  |  |  |  |
| **N adjusted for** | **none** | - | 1 | 38 | 7 | 46 |
|  | **1** | - | 50 | 12 | 42 | 104 |
|  | **2** | - | 0 | 1 | 0 | 1 |
|  | **6** | - | 0 | 3 | 0 | 3 |
|  |  |  |  |  |  |  |
| **Adjusted for** |  |  |  |  |  |  |
| **sex** |  | - | 0 | 4 | 0 | 4 |
| **age** |  | - | 50 | 16 | 42 | 108 |
|  |  |  |  |  |  |  |
| **Adjusted for other confounders** | **None** | - | 51 | 51 | 49 | 151 |
|  | **4** | - | 0 | 3 | 0 | 3 |
|  |  |  |  |  |  |  |
| **Unadjusted RRs only** |  |  |  |  |  |  |
| **Number of cases available** |  | - | - | 36 | 7 | 43 |
| **Number of controls/at risk/disease free available** |  | - | - | 36 | 7 | 43 |
| **Whole 2x2 table available** |  | - | - | 36 | 7 | 43 |
|  |  |  |  |  |  |  |
| **Adjusted RRs only** |  |  |  |  |  |  |
| **Number of cases available** |  | - | 38 | 12 | 42 | 92 |
|  |  |  |  |  |  |  |
| **Relative risk value** | **missing** | - | 2 | 6 | 0 | 8 |
|  | **<0.5** | - | 0 | 2 | 1 | 3 |
|  | **0.5-<1** | - | 3 | 12 | 0 | 15 |
|  | **>1-<1.5** | - | 10 | 1 | 0 | 11 |
|  | **1.5-<2** | - | 6 | 5 | 2 | 13 |
|  | **2-<3** | - | 8 | 1 | 5 | 14 |
|  | **3-<4** | - | 8 | 2 | 11 | 21 |
|  | **4-<5** | - | 4 | 1 | 7 | 12 |
|  | **5-<10** | - | 5 | 4 | 5 | 14 |
|  | **10-<20** | - | 5 | 8 | 16 | 29 |
|  | **20+** | - | 0 | 12 | 2 | 14 |
|  | N | 0 | 49 | 48 | 49 | 146 |
|  | Median | - | 2.55 | 5.07 | 4.75 | 3.72 |
|  | Range | - | 0.51:13.30 | 0.28:4124.26 | 0.27:25.34 | 0.27:4124.26 |
|  |  |  |  |  |  |  |
| **CI available** | **present** | - | 45 | 48 | 49 | 142 |
|  |  |  |  |  |  |  |
| **Derivation of RR^e^** | **original** | - | 11 | 8 | 0 | 19 |
|  | **RR/CI from numbers** | - | 0 | 35 | 7 | 42 |
|  | **combined smoking levels/sum** | - | 0 | 2 | 0 | 2 |
|  | **other combined/sum** | - | 0 | 4 | 0 | 4 |
|  | **RR/CI calc using 0.5 for 0** | - | 2 | 4 | 0 | 6 |
|  | **combined smoking levels (Hamling *et al* [14])** | - | 2 | 1 | 0 | 3 |
|  | **other** | - | 14 | 0 | 0 | 14 |
|  | **RR orig CI est from numbers** | - | 6 | 0 | 0 | 6 |
|  | **other (CI est from numbers)** | - | 16 | 0 | 42 | 58 |

^a^ All RRs are counted in the first section of the Table. In subsequent sections, RRs for each of the disease outcomes are counted.

^b^ Principal studies are categorized as CC (case-control, including nested CC), Prosp (prospective) or CrossSec (cross-sectional). Subsidiary studies are categorized separately.

^c^ For presence/absence variables, the “Levels” column is left blank and counts for only the “presence” level are shown. “Not applicable” levels are not shown (e.g. unexposed group for “per unit dose regression”)

^d^ refers to low exposure according to the same measure of exposure as the exposed group.

^e^ See Additional file 1 (*Derivation of RRs*) for further explanation.

## Table 4 Characteristics of the sets of relative risks for the dose-response indices

| All outcomes^a^ |  |  | Study Type^b^ | | | | |
| --- | --- | --- | --- | --- | --- | --- | --- |
| Measure | Type of set/RR^c^ |  | CC | Prosp | CrossSec | Subsid | Total |
|  |  |  |  |  |  |  |  |
| Amount smoked | Full sets of categorical data vs never | 2 levels | 0 | 13 | 49 | 11 | 73 |
|  |  | 3 levels | 10 | 14 | 66 | 23 | 113 |
|  |  | 4 levels | 5 | 8 | 2 | 21 | 36 |
|  |  | 5 levels | 0 | 1 | 1 | 4 | 6 |
|  |  | 6 levels | 0 | 0 | 4 | 2 | 6 |
|  |  | 7 levels | 0 | 0 | 1 | 0 | 1 |
|  |  |  |  |  |  |  |  |
|  |  | vs never any | 12 | 20 | 78 | 34 | 144 |
|  |  | vs never cigarettes | 3 | 16 | 45 | 27 | 91 |
|  |  |  |  |  |  |  |  |
|  | Full sets of categorical data vs low | 2 levels | 0 | 15 | 50 | 9 | 74 |
|  |  | 3 levels | 10 | 15 | 67 | 20 | 112 |
|  |  | 4 levels | 4 | 7 | 2 | 19 | 32 |
|  |  | 5 levels | 0 | 2 | 2 | 3 | 7 |
|  |  | 6 levels | 0 | 0 | 2 | 2 | 4 |
|  |  | 7 levels | 0 | 0 | 2 | 0 | 2 |
|  |  |  |  |  |  |  |  |
|  |  | vs low any | 2 | 5 | 24 | 4 | 35 |
|  |  | vs low cigarettes | 12 | 34 | 101 | 49 | 196 |
|  |  |  |  |  |  |  |  |
|  | Full sets of categorical data vs non | 2 levels | 0 | 0 | 5 | 0 | 5 |
|  |  | 3 levels | 1 | 0 | 4 | 0 | 5 |
|  |  | 5 levels | 0 | 1 | 0 | 0 | 1 |
|  |  |  |  |  |  |  |  |
|  |  | vs non any | 0 | 0 | 6 | 0 | 6 |
|  |  | vs non cigarettes | 1 | 1 | 3 | 0 | 5 |
|  |  |  |  |  |  |  |  |
|  | Per unit dose regression |  | 0 | 0 | 5 | 0 | 5 |
|  | Partial categorical data |  | 0 | 1 | 2 | 0 | 3 |
|  | Other |  | 3 | 1 | 11 | 0 | 15 |
|  |  |  |  |  |  |  |  |
| Years of age of starting | Full sets of categorical data vs never | 2 levels | 0 | 3 | 6 | 0 | 9 |
|  |  | 3 levels | 0 | 2 | 0 | 1 | 3 |
|  |  | 4 levels | 2 | 1 | 0 | 0 | 3 |
|  |  | 5 levels | 4 | 0 | 0 | 0 | 4 |
|  |  |  |  |  |  |  |  |
|  |  | vs never any | 5 | 4 | 0 | 0 | 9 |
|  |  | vs never cigarettes | 1 | 2 | 6 | 1 | 10 |
|  |  |  |  |  |  |  |  |
|  | Full sets of categorical data vs low (=older start) | 2 levels | 0 | 6 | 6 | 0 | 12 |
|  |  | 3 levels | 1 | 5 | 0 | 4 | 10 |
|  |  | 4 levels | 6 | 1 | 4 | 0 | 11 |
|  |  | 5 levels | 3 | 0 | 0 | 0 | 3 |
|  |  |  |  |  |  |  |  |
|  |  | vs low any | 2 | 1 | 1 | 0 | 4 |
|  |  | vs low cigarettes | 8 | 11 | 9 | 4 | 32 |
|  |  |  |  |  |  |  |  |
|  | Full sets of categorical data vs non | 3 levels | 1 | 0 | 0 | 0 | 1 |
|  |  |  |  |  |  |  |  |
|  |  | vs non cigarettes | 1 | 0 | 0 | 0 | 1 |
|  |  |  |  |  |  |  |  |
|  | Per unit dose regression |  | 0 | 0 | 3 | 0 | 3 |
|  | Other |  | 2 | 1 | 0 | 0 | 3 |
|  |  |  |  |  |  |  |  |
| Pack-years | Full sets of categorical data vs never | 2 levels | 2 | 0 | 7 | 0 | 9 |
|  |  | 3 levels | 1 | 4 | 29 | 6 | 40 |
|  |  | 4 levels | 0 | 0 | 2 | 2 | 4 |
|  |  | 5 levels | 0 | 0 | 7 | 2 | 9 |
|  |  |  |  |  |  |  |  |
|  |  | vs never any | 0 | 0 | 7 | 5 | 12 |
|  |  | vs never cigarettes | 3 | 4 | 38 | 5 | 50 |
|  |  |  |  |  |  |  |  |
|  | Full sets of categorical data vs low | 2 levels | 2 | 0 | 8 | 0 | 10 |
|  |  | 3 levels | 3 | 5 | 30 | 4 | 42 |
|  |  | 4 levels | 0 | 0 | 5 | 1 | 6 |
|  |  | 5 levels | 0 | 0 | 5 | 2 | 7 |
|  |  | 7 levels | 0 | 0 | 1 | 0 | 1 |
|  |  |  |  |  |  |  |  |
|  |  |  |  |  |  |  |  |
|  |  | vs low any | 0 | 0 | 2 | 1 | 3 |
|  |  | vs low cigarettes | 5 | 5 | 47 | 6 | 63 |
|  |  |  |  |  |  |  |  |
|  | Full sets of categorical data vs never+low | 2 levels | 0 | 0 | 1 | 2 | 3 |
|  |  |  |  |  |  |  |  |
|  | Partial categorical data |  | 4 | 0 | 3 | 0 | 7 |
|  | Per unit dose regression |  | 0 | 0 | 10 | 0 | 10 |
|  | Other |  | 9 | 1 | 9 | 3 | 22 |
|  |  |  |  |  |  |  |  |
| Years of duration of smoking | Full sets of categorical data vs never | 2 levels | 0 | 0 | 1 | 0 | 1 |
|  |  | 3 levels | 0 | 2 | 5 | 1 | 8 |
|  |  | 5 levels | 0 | 3 | 0 | 0 | 3 |
|  |  |  |  |  |  |  |  |
|  |  |  |  |  |  |  |  |
|  |  | vs never any | 0 | 3 | 6 | 0 | 9 |
|  |  | vs never cigarettes | 0 | 2 | 0 | 1 | 3 |
|  |  |  |  |  |  |  |  |
|  |  |  |  |  |  |  |  |
|  | Full sets of categorical data vs low | 2 levels | 0 | 0 | 1 | 0 | 1 |
|  |  | 3 levels | 0 | 1 | 5 | 1 | 7 |
|  |  | 5 levels | 0 | 3 | 0 | 0 | 3 |
|  |  |  |  |  |  |  |  |
|  |  |  |  |  |  |  |  |
|  |  | vs low any | 0 | 0 | 5 | 0 | 5 |
|  |  | vs low cigarettes | 0 | 4 | 1 | 1 | 6 |
|  |  |  |  |  |  |  |  |
|  |  |  |  |  |  |  |  |
|  | Per unit dose regression |  | 0 | 0 | 3 | 0 | 3 |
|  | Other |  | 3 | 0 | 4 | 0 | 7 |
|  |  |  |  |  |  |  |  |
| Years of duration of quitting (vs never/non/long term ex) | Full sets of categorical data vs never | 2 levels | 0 | 0 | 10 | 4 | 14 |
|  |  | 3 levels | 8 | 3 | 1 | 0 | 12 |
|  |  | 4 levels | 0 | 2 | 0 | 0 | 2 |
|  |  | 5 levels | 0 | 0 | 1 | 0 | 1 |
|  |  | 6 levels | 0 | 1 | 0 | 0 | 1 |
|  |  |  |  |  |  |  |  |
|  |  | vs never any | 8 | 2 | 6 | 4 | 20 |
|  |  | vs never cigarettes | 0 | 4 | 6 | 0 | 10 |
|  |  |  |  |  |  |  |  |
|  | Full sets of categorical data vs low (=long term ex) | 2 levels | 0 | 0 | 8 | 4 | 12 |
|  |  | 3 levels | 8 | 3 | 1 | 0 | 12 |
|  |  | 4 levels | 0 | 2 | 0 | 0 | 2 |
|  |  | 5 levels | 0 | 0 | 1 | 0 | 1 |
|  |  | 6 levels | 0 | 2 | 0 | 0 | 2 |
|  |  |  |  |  |  |  |  |
|  |  | vs low any | 2 | 0 | 4 | 4 | 10 |
|  |  | vs low cigarettes | 6 | 7 | 6 | 0 | 19 |
|  |  |  |  |  |  |  |  |
|  | Partial categorical data |  | 0 | 1 | 0 | 1 | 2 |
|  | Other |  | 1 | 1 | 4 | 0 | 6 |
|  |  |  |  |  |  |  |  |
| Years of duration of quitting (vs current/recent ex) | Full sets of categorical data vs current |  |  |  |  |  |  |
|  |  | 2 levels | 0 | 0 | 8 | 4 | 12 |
|  |  | 3 levels | 8 | 3 | 1 | 0 | 12 |
|  |  | 4 levels | 0 | 2 | 0 | 0 | 2 |
|  |  | 5 levels | 0 | 0 | 1 | 0 | 1 |
|  |  |  |  |  |  |  |  |
|  |  | vs current any | 2 | 0 | 4 | 4 | 10 |
|  |  | vs current cigarettes | 6 | 5 | 6 | 0 | 17 |
|  |  |  |  |  |  |  |  |
|  | Full sets of categorical data vs low (=recent ex) | 2 levels | 0 | 0 | 8 | 4 | 12 |
|  |  | 3 levels | 8 | 3 | 1 | 0 | 12 |
|  |  | 4 levels | 0 | 2 | 0 | 0 | 2 |
|  |  | 5 levels | 0 | 0 | 1 | 0 | 1 |
|  |  | 6 levels | 0 | 2 | 0 | 0 | 2 |
|  |  |  |  |  |  |  |  |
|  |  | vs low any | 2 | 0 | 4 | 4 | 10 |
|  |  | vs low cigarettes | 6 | 7 | 6 | 0 | 19 |
|  |  |  |  |  |  |  |  |
|  | Partial categorical data |  | 0 | 0 | 0 | 1 | 1 |
|  |  |  |  |  |  |  |  |

## Table 4 (continued) COPD

| COPD^a^ |  |  | Study Type^b^ | | | | |
| --- | --- | --- | --- | --- | --- | --- | --- |
| Measure | Type of set/RR^c^ |  | CC | Prosp | CrossSec | Subsid | Total |
|  |  |  |  |  |  |  |  |
| Amount smoked | Full sets of categorical data vs never | 2 levels | 0 | 10 | 17 | 2 | 29 |
|  |  | 3 levels | 4 | 8 | 13 | 1 | 26 |
|  |  | 4 levels | 0 | 7 | 2 | 7 | 16 |
|  |  | 5 levels | 0 | 1 | 0 | 2 | 3 |
|  |  | 6 levels | 0 | 0 | 4 | 2 | 6 |
|  |  |  |  |  |  |  |  |
|  |  | vs never any | 4 | 15 | 20 | 8 | 47 |
|  |  | vs never cigarettes | 0 | 11 | 16 | 6 | 33 |
|  |  |  |  |  |  |  |  |
|  | Full sets of categorical data vs low | 2 levels | 0 | 12 | 13 | 2 | 27 |
|  |  | 3 levels | 5 | 9 | 13 | 2 | 29 |
|  |  | 4 levels | 0 | 7 | 2 | 7 | 16 |
|  |  | 5 levels | 0 | 2 | 0 | 2 | 4 |
|  |  | 6 levels | 0 | 0 | 2 | 2 | 4 |
|  |  |  |  |  |  |  |  |
|  |  | vs low any | 0 | 3 | 4 | 0 | 7 |
|  |  | vs low cigarettes | 5 | 27 | 26 | 15 | 73 |
|  |  |  |  |  |  |  |  |
|  | Full sets of categorical data vs non | 3 levels | 1 | 0 | 0 | 0 | 1 |
|  |  | 5 levels | 0 | 1 | 0 | 0 | 1 |
|  |  |  |  |  |  |  |  |
|  |  | vs non cigarettes | 1 | 1 | 0 | 0 | 2 |
|  |  |  |  |  |  |  |  |
|  | Partial categorical data |  | 0 | 1 | 2 | 0 | 3 |
|  | Per unit dose regression |  | 0 | 0 | 1 | 0 | 1 |
|  | Other |  | 2 | 1 | 2 | 0 | 5 |
|  |  |  |  |  |  |  |  |
| Years of age of starting | Full sets of categorical data vs never | 2 levels | 0 | 1 | 6 | 0 | 7 |
|  |  | 3 levels | 0 | 2 | 0 | 1 | 3 |
|  |  | 4 levels | 2 | 1 | 0 | 0 | 3 |
|  |  |  |  |  |  |  |  |
|  |  | vs never any | 2 | 4 | 0 | 0 | 6 |
|  |  | vs never cigarettes | 0 | 0 | 6 | 1 | 7 |
|  |  |  |  |  |  |  |  |
|  | Full sets of categorical data vs low (=older start) | 2 levels | 0 | 4 | 6 | 0 | 10 |
|  |  | 3 levels | 1 | 5 | 0 | 2 | 8 |
|  |  | 4 levels | 4 | 1 | 0 | 0 | 5 |
|  |  |  |  |  |  |  |  |
|  |  | vs low any | 0 | 1 | 0 | 0 | 1 |
|  |  | vs low cigarettes | 5 | 9 | 6 | 2 | 22 |
|  |  |  |  |  |  |  |  |
|  | Full sets of categorical data vs non | 3 levels | 1 | 0 | 0 | 0 | 1 |
|  |  |  |  |  |  |  |  |
|  |  | vs non cigarettes | 1 | 0 | 0 | 0 | 1 |
|  |  |  |  |  |  |  |  |
|  | Per unit dose regression |  | 0 | 0 | 3 | 0 | 3 |
|  | Other |  | 2 | 1 | 0 | 0 | 3 |
|  |  |  |  |  |  |  |  |
| Pack-years | Full sets of categorical data vs never | 2 levels | 1 | 0 | 5 | 0 | 6 |
|  |  | 3 levels | 1 | 2 | 19 | 1 | 23 |
|  |  | 5 levels | 0 | 0 | 6 | 2 | 8 |
|  |  |  |  |  |  |  |  |
|  |  | vs never any | 0 | 0 | 5 | 0 | 5 |
|  |  | vs never cigarettes | 2 | 2 | 25 | 3 | 32 |
|  |  |  |  |  |  |  |  |
|  | Full sets of categorical data vs low | 2 levels | 1 | 0 | 6 | 0 | 7 |
|  |  | 3 levels | 3 | 3 | 20 | 1 | 27 |
|  |  | 4 levels | 0 | 0 | 3 | 0 | 3 |
|  |  | 5 levels | 0 | 0 | 4 | 2 | 6 |
|  |  | 7 levels | 0 | 0 | 1 | 0 | 1 |
|  |  |  |  |  |  |  |  |
|  |  | vs low any | 0 | 0 | 2 | 0 | 2 |
|  |  | vs low cigarettes | 4 | 3 | 32 | 3 | 42 |
|  |  |  |  |  |  |  |  |
|  | Full sets of categorical data vs never+low | 2 levels | 0 | 0 | 1 | 2 | 3 |
|  |  |  |  |  |  |  |  |
|  | Partial categorical data |  | 4 | 0 | 1 | 0 | 5 |
|  | Per unit dose regression |  | 0 | 0 | 7 | 0 | 7 |
|  | Other |  | 8 | 1 | 4 | 3 | 16 |
|  |  |  |  |  |  |  |  |
| Years of duration of smoking | Full sets of categorical data vs never | 2 levels | 0 | 0 | 1 | 0 | 1 |
|  |  | 3 levels | 0 | 0 | 0 | 1 | 1 |
|  |  | 5 levels | 0 | 1 | 0 | 0 | 1 |
|  |  |  |  |  |  |  |  |
|  |  | vs never any | 0 | 1 | 1 | 0 | 2 |
|  |  | vs never cigarettes | 0 | 0 | 0 | 1 | 1 |
|  |  |  |  |  |  |  |  |
|  | Full sets of categorical data vs low | 2 levels | 0 | 0 | 1 | 0 | 1 |
|  |  | 3 levels | 0 | 0 | 0 | 1 | 1 |
|  |  | 5 levels | 0 | 1 | 0 | 0 | 1 |
|  |  |  |  |  |  |  |  |
|  |  | vs low cigarettes | 0 | 1 | 1 | 1 | 3 |
|  |  |  |  |  |  |  |  |
|  | Per unit dose regression |  | 0 | 0 | 1 | 0 | 1 |
|  | Other |  | 3 | 0 | 1 | 0 | 4 |
|  |  |  |  |  |  |  |  |
| Years of duration of quitting (vs never/non/long term ex) | Full sets of categorical data vs never | 2 levels | 0 | 0 | 3 | 2 | 5 |
|  |  | 3 levels | 6 | 3 | 0 | 0 | 9 |
|  |  | 6 levels | 0 | 1 | 0 | 0 | 1 |
|  |  |  |  |  |  |  |  |
|  |  | vs never any | 6 | 1 | 1 | 2 | 10 |
|  |  | vs never cigarettes | 0 | 3 | 2 | 0 | 5 |
|  |  |  |  |  |  |  |  |
|  | Full sets of categorical data vs low (=long term ex) | 2 levels | 0 | 0 | 3 | 2 | 5 |
|  |  | 3 levels | 6 | 3 | 0 | 0 | 9 |
|  |  | 6 levels | 0 | 2 | 0 | 0 | 2 |
|  |  |  |  |  |  |  |  |
|  |  | vs low any | 2 | 0 | 1 | 2 | 5 |
|  |  | vs low cigarettes | 4 | 5 | 2 | 0 | 11 |
|  |  |  |  |  |  |  |  |
|  | Partial categorical data |  | 0 | 1 | 0 | 1 | 2 |
|  | Other |  | 1 | 0 | 0 | 0 | 1 |
|  |  |  |  |  |  |  |  |
| Years of duration of quitting (vs current/recent ex) | Full sets of categorical data vs current | 2 levels | 0 | 0 | 3 | 2 | 5 |
|  |  | 3 levels | 6 | 3 | 0 | 0 | 9 |
|  |  |  |  |  |  |  |  |
|  |  | vs current any | 2 | 0 | 1 | 2 | 5 |
|  |  | vs current cigarettes | 4 | 3 | 2 | 0 | 9 |
|  |  |  |  |  |  |  |  |
|  | Full sets of categorical data vs low (=recent ex) | 2 levels | 0 | 0 | 3 | 2 | 5 |
|  |  | 3 levels | 6 | 3 | 0 | 0 | 9 |
|  |  | 6 levels | 0 | 2 | 0 | 0 | 2 |
|  |  |  |  |  |  |  |  |
|  |  | vs low any | 2 | 0 | 1 | 2 | 5 |
|  |  | vs low cigarettes | 4 | 5 | 2 | 0 | 11 |
|  |  |  |  |  |  |  |  |
|  | Partial categorical data |  | 0 | 0 | 0 | 1 | 1 |
|  |  |  |  |  |  |  |  |

## Table 4 (continued) CB

| CB^a^ |  |  | Study Type^b^ | | | | |
| --- | --- | --- | --- | --- | --- | --- | --- |
| Measure | Type of set/RR^c^ |  | CC | Prosp | CrossSec | Subsid | Total |
|  |  |  |  |  |  |  |  |
| Amount smoked | Full sets of categorical data vs never | 2 levels | 0 | 0 | 29 | 9 | 38 |
|  |  | 3 levels | 6 | 4 | 48 | 22 | 80 |
|  |  | 4 levels | 5 | 0 | 0 | 7 | 12 |
|  |  | 5 levels | 0 | 0 | 1 | 2 | 3 |
|  |  | 7 levels | 0 | 0 | 1 | 0 | 1 |
|  |  |  |  |  |  |  |  |
|  |  | vs never any | 8 | 2 | 50 | 20 | 80 |
|  |  | vs never cigarettes | 3 | 2 | 29 | 20 | 54 |
|  |  |  |  |  |  |  |  |
|  | Full sets of categorical data vs low | 2 levels | 0 | 1 | 32 | 7 | 40 |
|  |  | 3 levels | 5 | 4 | 47 | 18 | 74 |
|  |  | 4 levels | 4 | 0 | 0 | 5 | 9 |
|  |  | 5 levels | 0 | 0 | 2 | 1 | 3 |
|  |  | 7 levels | 0 | 0 | 2 | 0 | 2 |
|  |  |  |  |  |  |  |  |
|  |  | vs low any | 2 | 1 | 20 | 4 | 27 |
|  |  | vs low cigarettes | 7 | 4 | 63 | 27 | 101 |
|  |  |  |  |  |  |  |  |
|  | Full sets of categorical data vs non | 2 levels | 0 | 0 | 5 | 0 | 5 |
|  |  | 3 levels | 0 | 0 | 4 | 0 | 4 |
|  |  |  |  |  |  |  |  |
|  |  | vs non any | 0 | 0 | 6 | 0 | 6 |
|  |  | vs non cigarettes | 0 | 0 | 3 | 0 | 3 |
|  |  |  |  |  |  |  |  |
|  | Per unit dose regression |  | 0 | 0 | 4 | 0 | 4 |
|  | Other |  | 1 | 0 | 6 | 0 | 7 |
|  |  |  |  |  |  |  |  |
| Years of age of starting | Full sets of categorical data vs never | 5 levels | 4 | 0 | 0 | 0 | 4 |
|  |  |  |  |  |  |  |  |
|  |  | vs never any | 3 | 0 | 0 | 0 | 3 |
|  |  | vs never cigarettes | 1 | 0 | 0 | 0 | 1 |
|  |  |  |  |  |  |  |  |
|  | Full sets of categorical data vs low (=older start) | 3 levels | 0 | 0 | 0 | 2 | 2 |
|  |  | 4 levels | 2 | 0 | 4 | 0 | 6 |
|  |  | 5 levels | 3 | 0 | 0 | 0 | 3 |
|  |  |  |  |  |  |  |  |
|  |  | vs low any | 2 | 0 | 1 | 0 | 3 |
|  |  | vs low cigarettes | 3 | 0 | 3 | 2 | 8 |
|  |  |  |  |  |  |  |  |
| Pack-years | Full sets of categorical data vs never | 2 levels | 1 | 0 | 1 | 0 | 2 |
|  |  | 3 levels | 0 | 0 | 10 | 5 | 15 |
|  |  | 4 levels | 0 | 0 | 2 | 2 | 4 |
|  |  | 5 levels | 0 | 0 | 1 | 0 | 1 |
|  |  |  |  |  |  |  |  |
|  |  | vs never any | 0 | 0 | 2 | 5 | 7 |
|  |  | vs never cigarettes | 1 | 0 | 12 | 2 | 15 |
|  |  |  |  |  |  |  |  |
|  | Full sets of categorical data vs low | 2 levels | 1 | 0 | 1 | 0 | 2 |
|  |  | 3 levels | 0 | 0 | 10 | 3 | 13 |
|  |  | 4 levels | 0 | 0 | 2 | 1 | 3 |
|  |  | 5 levels | 0 | 0 | 1 | 0 | 1 |
|  |  |  |  |  |  |  |  |
|  |  | vs low any | 0 | 0 | 0 | 1 | 1 |
|  |  | vs low cigarettes | 1 | 0 | 14 | 3 | 18 |
|  |  |  |  |  |  |  |  |
|  | Partial categorical data |  | 0 | 0 | 2 | 0 | 2 |
|  | Per unit dose regression |  | 0 | 0 | 3 | 0 | 3 |
|  | Other |  | 1 | 0 | 3 | 0 | 4 |
|  |  |  |  |  |  |  |  |
| Years of duration of smoking | Full sets of categorical data vs never | 3 levels | 0 | 1 | 4 | 0 | 5 |
|  |  | 5 levels | 0 | 1 | 0 | 0 | 1 |
|  |  |  |  |  |  |  |  |
|  |  | vs never any | 0 | 1 | 4 | 0 | 5 |
|  |  | vs never cigarettes | 0 | 1 | 0 | 0 | 1 |
|  |  |  |  |  |  |  |  |
|  | Full sets of categorical data vs low | 3 levels | 0 | 1 | 4 | 0 | 5 |
|  |  | 5 levels | 0 | 1 | 0 | 0 | 1 |
|  |  |  |  |  |  |  |  |
|  |  | vs low any | 0 | 0 | 4 | 0 | 4 |
|  |  | vs low cigarettes | 0 | 2 | 0 | 0 | 2 |
|  |  |  |  |  |  |  |  |
|  | Per unit dose regression |  | 0 | 0 | 2 | 0 | 2 |
|  | Other |  | 0 | 0 | 3 | 0 | 3 |
|  |  |  |  |  |  |  |  |
| Years of duration of quitting (vs never/non/long term ex) | Full sets of categorical data vs never | 2 levels | 0 | 0 | 7 | 2 | 9 |
|  |  | 3 levels | 2 | 0 | 1 | 0 | 3 |
|  |  | 4 levels | 0 | 2 | 0 | 0 | 2 |
|  |  | 5 levels | 0 | 0 | 1 | 0 | 1 |
|  |  |  |  |  |  |  |  |
|  |  | vs never any | 2 | 1 | 5 | 2 | 10 |
|  |  | vs never cigarettes | 0 | 1 | 4 | 0 | 5 |
|  |  |  |  |  |  |  |  |
|  | Full sets of categorical data vs low (=long term ex) | 2 levels | 0 | 0 | 5 | 2 | 7 |
|  |  | 3 levels | 2 | 0 | 1 | 0 | 3 |
|  |  | 4 levels | 0 | 2 | 0 | 0 | 2 |
|  |  | 5 levels | 0 | 0 | 1 | 0 | 1 |
|  |  |  |  |  |  |  |  |
|  |  | vs low any | 0 | 0 | 3 | 2 | 5 |
|  |  | vs low cigarettes | 2 | 2 | 4 | 0 | 8 |
|  |  |  |  |  |  |  |  |
|  | Other |  | 0 | 0 | 3 | 0 | 3 |
|  |  |  |  |  |  |  |  |
| Years of duration of quitting (vs current/recent ex) | Full sets of categorical data vs current |  |  |  |  |  |  |
|  |  | 2 levels | 0 | 0 | 5 | 2 | 7 |
|  |  | 3 levels | 2 | 0 | 1 | 0 | 3 |
|  |  | 4 levels | 0 | 2 | 0 | 0 | 2 |
|  |  | 5 levels | 0 | 0 | 1 | 0 | 1 |
|  |  |  |  |  |  |  |  |
|  |  | vs current any | 0 | 0 | 3 | 2 | 5 |
|  |  | vs current cigarettes | 2 | 2 | 4 | 0 | 8 |
|  |  |  |  |  |  |  |  |
|  | Full sets of categorical data vs low (=recent ex) | 2 levels | 0 | 0 | 5 | 2 | 7 |
|  |  | 3 levels | 2 | 0 | 1 | 0 | 3 |
|  |  | 4 levels | 0 | 2 | 0 | 0 | 2 |
|  |  | 5 levels | 0 | 0 | 1 | 0 | 1 |
|  |  |  |  |  |  |  |  |
|  |  | vs low any | 0 | 0 | 3 | 2 | 5 |
|  |  | vs low cigarettes | 2 | 2 | 4 | 0 | 8 |
|  |  |  |  |  |  |  |  |

## Table 4 (continued) Emphysema

| Emphysema^a^ |  |  | Study Type^b^ | | | | |
| --- | --- | --- | --- | --- | --- | --- | --- |
| Measure | Type of set/RR^c^ |  | CC | Prosp | CrossSec | Subsid | Total |
|  |  |  |  |  |  |  |  |
| Amount smoked | Full sets of categorical data vs never | 2 levels | - | 3 | 3 | 0 | 6 |
|  |  | 3 levels | - | 2 | 5 | 0 | 7 |
|  |  | 4 levels | - | 1 | 0 | 7 | 8 |
|  |  |  |  |  |  |  |  |
|  |  | vs never any | - | 3 | 8 | 6 | 17 |
|  |  | vs never cigarettes | - | 3 | 0 | 1 | 4 |
|  |  |  |  |  |  |  |  |
|  | Full sets of categorical data vs low | 2 levels | - | 2 | 5 | 0 | 7 |
|  |  | 3 levels | - | 2 | 7 | 0 | 9 |
|  |  | 4 levels | - | 0 | 0 | 7 | 7 |
|  |  |  |  |  |  |  |  |
|  |  | vs low any | - | 1 | 0 | 0 | 1 |
|  |  | vs low cigarettes | - | 3 | 12 | 7 | 22 |
|  |  |  |  |  |  |  |  |
|  | Other |  | - | 0 | 3 | 0 | 3 |
|  |  |  |  |  |  |  |  |
| Years of age of starting | Full sets of categorical data vs never | 2 levels | - | 2 | 0 | 0 | 2 |
|  |  |  |  |  |  |  |  |
|  |  | vs never cigarettes | - | 2 | 0 | 0 | 2 |
|  |  |  |  |  |  |  |  |
|  | Full sets of categorical data vs low (=older start) | 2 levels | - | 2 | 0 | 0 | 2 |
|  |  |  |  |  |  |  |  |
|  |  | vs low cigarettes | - | 2 | 0 | 0 | 2 |
|  |  |  |  |  |  |  |  |
| Pack-years | Full sets of categorical data vs never | 2 levels | - | 0 | 1 | 0 | 1 |
|  |  | 3 levels | - | 2 | 0 | 0 | 2 |
|  |  |  |  |  |  |  |  |
|  |  | vs never cigarettes | - | 2 | 1 | 0 | 3 |
|  |  |  |  |  |  |  |  |
|  | Full sets of categorical data vs low | 2 levels | - | 0 | 1 | 0 | 1 |
|  |  | 3 levels | - | 2 | 0 | 0 | 2 |
|  |  |  |  |  |  |  |  |
|  |  | vs low cigarettes | - | 2 | 1 | 0 | 3 |
|  |  |  |  |  |  |  |  |
|  | Other |  | - | 0 | 2 | 0 | 2 |
|  |  |  |  |  |  |  |  |
| Years of duration of smoking | Full sets of categorical data vs never | 3 levels | - | 1 | 1 | 0 | 2 |
|  |  | 5 levels | - | 1 | 0 | 0 | 1 |
|  |  |  |  |  |  |  |  |
|  |  | vs never any | - | 1 | 1 | 0 | 2 |
|  |  | vs never cigarettes | - | 1 | 0 | 0 | 1 |
|  |  |  |  |  |  |  |  |
|  | Full sets of categorical data vs low | 3 levels | - | 0 | 1 | 0 | 1 |
|  |  | 5 levels | - | 1 | 0 | 0 | 1 |
|  |  |  |  |  |  |  |  |
|  |  | vs low any | - | 0 | 1 | 0 | 1 |
|  |  | vs low cigarettes | - | 1 | 0 | 0 | 1 |
|  |  |  |  |  |  |  |  |
| Years of duration of quitting (vs never/non/long term ex) | Other |  | - | 1 | 1 | 0 | 2 |

^a^ All RRs are counted in the first section of the Table. In subsequent sections, RRs for each of the disease outcomes are counted.

^b^ Principal studies are categorized as CC (case-control, including nested CC), Prosp (prospective) or CrossSec (cross-sectional). Subsidiary studies are categorized separately.

^c^ Counts are of sets of RRs, except for “Per unit dose regression”, “Partial categorical data” and “Other” which are entered as single RRs. “Other” includes comparison of mean or median exposure between cases and non-cases.

## Table 5 Studies with RRs for the major smoking indices

| Studies with RRs for COPD and: | | | | | |
| --- | --- | --- | --- | --- | --- |
| Both current and ever smoking | Current but not ever smoking | Ever but not current smoking | Ex smoking as well as current and/or ever smoking | Ex smoking but neither current nor ever smoking | No major indices (dose-response only) |
|  |  |  |  |  |  |
| ALESSA | CHEN3 | AMIGO | ALESSA | (none) | AMIGO |
| ANDER1 | KAHN | FIDAN | ANDER1 |  | FIDAN |
| ANDER3 | LIAW | GULSVI | ANDER3 |  | GULSVI |
| BEDNAR | PEAT | ITABAS | BEDNAR |  | ITABAS |
| BEST | SAWICK | KIRAZ | BEST |  | KIRAZ |
| CHEN2 | STERLI | KLAYTO | CHEN2 |  | KLAYTO |
| CLEMEN | VOLLM1 | LAI | CLEMEN |  | LAI |
| COCCI | VOLLM2 | LAM1 | DEAN1 |  | LAM1 |
| DEAN1 | WALD | PEREZP | DEJONG |  | PEREZP |
| DEJONG | WEN | SICHLE | DEMARC |  | SICHLE |
| DEMARC | WOJTYN | WILSO1 | DICKIN |  | WILSO1 |
| DICKIN |  | XU | DOLL1 |  | XU |
| DOLL1 |  | ZIELI1 | DOLL2 |  | ZIELI1 |
| DOLL2 |  |  | DONTA1 |  |  |
| DONTA1 |  |  | EKBERG |  |  |
| EKBERG |  |  | ENSTRO |  |  |
| ENSTRO |  |  | FERRI1 |  |  |
| FERRI1 |  |  | FERRI2 |  |  |
| FERRI2 |  |  | FERRI3 |  |  |
| FERRI3 |  |  | FORAST |  |  |
| FORAST |  |  | FUKUCH |  |  |
| FUKUCH |  |  | GODTFR |  |  |
| GODTFR |  |  | HAMMO2 |  |  |
| HAMMO2 |  |  | HARDIE |  |  |
| HARDIE |  |  | HARIKK |  |  |
| HARIKK |  |  | HAWTHO |  |  |
| HAWTHO |  |  | HIGGI4 |  |  |
| HIGGI4 |  |  | HO |  |  |
| HO |  |  | HOZAWA |  |  |
| HOZAWA |  |  | HUHTI1 |  |  |
| HUHTI1 |  |  | HUHTI2 |  |  |
| HUHTI2 |  |  | HUHTI3 |  |  |
| HUHTI3 |  |  | JACOBS |  |  |
| JACOBS |  |  | JOHANN |  |  |
| JOHANN |  |  | KACHEL |  |  |
| KACHEL |  |  | KAHN |  |  |
| KAHN2 |  |  | KAHN2 |  |  |
| KARAKA |  |  | KARAKA |  |  |
| KATANC |  |  | KATANC |  |  |
| KIM |  |  | KIM |  |  |
| KOJIMA |  |  | KOJIMA |  |  |
| KRZYZA |  |  | KRZYZA |  |  |
| LAM2 |  |  | LAM2 |  |  |
| LAM3 |  |  | LAM3 |  |  |
| LANGE |  |  | LANGE |  |  |
| LEBOWI |  |  | LEBOWI |  |  |
| LEE |  |  | LEE |  |  |
| LINDBE |  |  | LINDBE |  |  |
| LINDST |  |  | LINDST |  |  |
| LUNDB1 |  |  | LUNDB1 |  |  |
| MADOR |  |  | MADOR |  |  |
| MANNI1 |  |  | MANNI1 |  |  |
| MANNI2 |  |  | MANNI2 |  |  |
| MANNI3 |  |  | MANNI3 |  |  |
| MARAN1 |  |  | MARAN1 |  |  |
| MARAN2 |  |  | MARAN2 |  |  |
| MARCUS |  |  | MARCUS |  |  |
| MATHES |  |  | MATHES |  |  |
| MENEZ2 |  |  | MENEZ2 |  |  |
| MENEZ3 |  |  | MENEZ3 |  |  |
| MENEZ4 |  |  | MENEZ4 |  |  |
| MENEZ5 |  |  | MENEZ5 |  |  |
| MENEZ6 |  |  | MENEZ6 |  |  |
| MONTNE |  |  | MONTNE |  |  |
| MUELLE |  |  | MUELLE |  |  |
| NIEPSU |  |  | NIEPSU |  |  |
| NIHLEN |  |  | NIHLEN |  |  |
| NILSSO |  |  | NILSSO |  |  |
| PELKON |  |  | PELKON |  |  |
| PETO |  |  | PETO |  |  |
| RENWIC |  |  | RENWIC |  |  |
| RICCIO |  |  | RICCIO |  |  |
| SARGEA |  |  | SARGEA |  |  |
| SHAHAB |  |  | SHAHAB |  |  |
| SHIN |  |  | SHIN |  |  |
| SILVA |  |  | SILVA |  |  |
| SPEIZE |  |  | SPEIZE |  |  |
| STROM |  |  | STERLI |  |  |
| TANG |  |  | STROM |  |  |
| THUN |  |  | TANG |  |  |
| TODD |  |  | THUN |  |  |
| TRUPIN |  |  | TODD |  |  |
| TSUSHI |  |  | TRUPIN |  |  |
| TVERDA |  |  | TSUSHI |  |  |
| VESTBO |  |  | TVERDA |  |  |
| VIEGI2 |  |  | VESTBO |  |  |
| VONHER |  |  | VIEGI2 |  |  |
| WEISS |  |  | VONHER |  |  |
| XIAO |  |  | WEISS |  |  |
| YAMAGU |  |  | XIAO |  |  |
| YUAN |  |  | YAMAGU |  |  |
| ZIELI2 |  |  | YUAN |  |  |
| ZIETKO |  |  | ZIELI2 |  |  |

| Studies with RRs for CB and: | | | | | |
| --- | --- | --- | --- | --- | --- |
| Both current and ever smoking | Current but not ever smoking | Ever but not current smoking | Ex smoking as well as current and/or ever smoking | Ex smoking but neither current nor ever smoking | No major indices (dose-response only) |
| ALDERS | SAWICK | FINKLE | ALDERS | KAHN | FINKLE |
| ANDER1 | WEN | JINDA2 | ANDER1 |  | JINDA2 |
| BECK1 | WOJTYN | KIRAZ | BECK1 |  | KIRAZ |
| BECK2 |  | KUBIK | BECK2 |  | KUBIK |
| BEST |  | OSWAL2 | BEST |  | OSWAL2 |
| BJORNS |  | PEREZP | BJORNS |  | PEREZP |
| BROWN |  |  | BROWN |  |  |
| CERVER |  |  | CERVER |  |  |
| CHAPMA |  |  | CHAPMA |  |  |
| COATES |  |  | COATES |  |  |
| COLLEG |  |  | COLLEG |  |  |
| DEAN2 |  |  | DEAN2 |  |  |
| DEANE |  |  | DEANE |  |  |
| DEMARC |  |  | DEMARC |  |  |
| DOLL1 |  |  | DOLL1 |  |  |
| DONTA2 |  |  | DONTA2 |  |  |
| DOPICO |  |  | DOPICO |  |  |
| EHRLIC |  |  | EHRLIC |  |  |
| ENRIGH |  |  | ENRIGH |  |  |
| FERRI1 |  |  | FERRI1 |  |  |
| FOXMAN |  |  | FOXMAN |  |  |
| GOLDBE |  |  | GOLDBE |  |  |
| HAENSZ |  |  | HAENSZ |  |  |
| HARDIE |  |  | HARDIE |  |  |
| HARRIS |  |  | HARRIS |  |  |
| HAWTHO |  |  | HAWTHO |  |  |
| HAYES |  |  | HAYES |  |  |
| HIGGI2 |  |  | HIGGI2 |  |  |
| HIGGI3 |  |  | HIGGI3 |  |  |
| HIGGI6 |  |  | HIGGI6 |  |  |
| HO |  |  | HO |  |  |
| HOLLA2 |  |  | HOLLA2 |  |  |
| HOLLNA |  |  | HOLLNA |  |  |
| HOUSE |  |  | HOUSE |  |  |
| HUCHON |  |  | HUCHON |  |  |
| HUHTI1 |  |  | HUHTI1 |  |  |
| HUHTI2 |  |  | HUHTI2 |  |  |
| HUHTI3 |  |  | HUHTI3 |  |  |
| JOSHI |  |  | JOUSI1 |  |  |
| JOUSI1 |  |  | KAHN2 |  |  |
| KAHN2 |  |  | KATO |  |  |
| KATO |  |  | KOTAN1 |  |  |
| KOTAN1 |  |  | LAMBER |  |  |
| LAMBER |  |  | LANGE2 |  |  |
| LANGHA |  |  | LANGHA |  |  |
| LAVECC |  |  | LAVECC |  |  |
| LEBOWI |  |  | LEBOWI |  |  |
| LINDST |  |  | LINDST |  |  |
| LUNDB2 |  |  | LUNDB2 |  |  |
| MAGNUS |  |  | MAGNUS |  |  |
| MANFRE |  |  | MANFRE |  |  |
| MELLST |  |  | MELLST |  |  |
| MENEZ1 |  |  | MENEZ1 |  |  |
| MEREN |  |  | MEREN |  |  |
| MILLER |  |  | MILLER |  |  |
| MILNE |  |  | MILNE |  |  |
| MOLLER |  |  | MOLLER |  |  |
| MUELLE |  |  | MUELLE |  |  |
| NEJJAR |  |  | NEJJAR |  |  |
| OGILVI |  |  | OGILVI |  |  |
| PANDEY |  |  | PANDEY |  |  |
| PELKON |  |  | PELKON |  |  |
| REID |  |  | REID |  |  |
| SAWICK |  |  | SAWICK |  |  |
| SHIMUR |  |  | SHIMUR |  |  |
| SILVA |  |  | SILVA |  |  |
| SOBRAD |  |  | SOBRAD |  |  |
| STJERN |  |  | STJERN |  |  |
| TROISI |  |  | TROISI |  |  |
| URRUTI |  |  | URRUTI |  |  |
| VIEGI1 |  |  | VIEGI1 |  |  |
| WAGEN2 |  |  | WAGEN2 |  |  |
| WILHEL |  |  | WILHEL |  |  |
| WOODS |  |  | WOODS |  |  |
| WOOLF |  |  | WOOLF |  |  |
| YAMAGU |  |  | YAMAGU |  |  |
| ZOIA |  |  | ZOIA |  |  |
|  |  |  |  |  |  |

| Studies with RRs for emphysema and: | | | | | |
| --- | --- | --- | --- | --- | --- |
| Both current and ever smoking | Current but not ever smoking | Ever but not current smoking | Ex smoking as well as current and/or ever smoking | Ex smoking but neither current nor ever smoking | No major indices (dose-response only) |
| BEST | AUERBA | ANDER2 | BEST | KAHN | ANDER2 |
| DONTA2 | HIRAYA | GULSVI | DONTA2 |  | GULSVI |
| ENRIGH | VIKGRE | HAMMO2 | ENRIGH |  | HAMMO2 |
| HARDIE | WEN | HOZAWA | HARDIE |  | HOZAWA |
| HO |  | NAWA | HO |  | NAWA |
| HUHTI1 |  | PRATT | HUHTI1 |  | PRATT |
| KAHN2 |  | RYDER | KAHN2 |  | RYDER |
| LAVECC |  | SUTINE | LAVECC |  | SUTINE |
| LEBOWI |  | WANG2 | LEBOWI |  | WANG2 |
| MILLER |  |  | MILLER |  |  |
| OMORI |  |  | OMORI |  |  |
| SILVA |  |  | SILVA |  |  |
| WEISS |  |  | WEISS |  |  |

## Table 6 Studies with RRs for the dose-response indices

| Studies with RRs for COPD and: | Type of RRs^a^ | | | |
| --- | --- | --- | --- | --- |
|  | **FullCat** | **PartCat** | **Regr** | **Other** |
| Amount smoked |  |  |  |  |
|  | ANDER1 | KHOURY | DETORR | AMIGO |
|  | BEST | NIEPSU |  | MUELLE |
|  | CHEN1 | NIHLEN |  | SHAHAB |
|  | CHEN2 |  |  | TODD |
|  | CLEMEN |  |  |  |
|  | DEAN1 |  |  |  |
|  | DOLL1 |  |  |  |
|  | DOLL2 |  |  |  |
|  | DONTA1 |  |  |  |
|  | ENSTRO |  |  |  |
|  | FERRI1 |  |  |  |
|  | FERRI2 |  |  |  |
|  | FERRI3 |  |  |  |
|  | GODTFR |  |  |  |
|  | HAWTHO |  |  |  |
|  | HOZAWA |  |  |  |
|  | HUHTI1 |  |  |  |
|  | HUHTI3 |  |  |  |
|  | JACOBS |  |  |  |
|  | KAHN |  |  |  |
|  | KAHN2 |  |  |  |
|  | KRZYZA |  |  |  |
|  | KULLER |  |  |  |
|  | LEBOWI |  |  |  |
|  | LEE |  |  |  |
|  | LIAW |  |  |  |
|  | LINDST |  |  |  |
|  | LIU1 |  |  |  |
|  | LUNDB1 |  |  |  |
|  | MUELLE |  |  |  |
|  | NILSSO |  |  |  |
|  | PETO |  |  |  |
|  | SPEIZE |  |  |  |
|  | TODD |  |  |  |
|  | TVERDA |  |  |  |
|  | VONHER |  |  |  |
|  | WEISS |  |  |  |
|  | WEN |  |  |  |
|  | YAMAGU |  |  |  |
|  | YUAN |  |  |  |
| Age started |  |  |  |  |
|  | CHEN2 |  | DETORR | AMIGO |
|  | CHEN3 |  | JAENDI | TODD |
|  | DEAN1 |  |  |  |
|  | GEIJER |  |  |  |
|  | HAWTHO |  |  |  |
|  | KAHN |  |  |  |
|  | LEE |  |  |  |
|  | LIAW |  |  |  |
|  | LIU1 |  |  |  |
|  | NILSSO |  |  |  |
|  | TODD |  |  |  |
|  | YUAN |  |  |  |
| Pack-years |  |  |  |  |
|  | ANDER1 | AMIGO | BEDNAR | BROGGE |
|  | CHENG | NIEPSU | DETORR | COCCI |
|  | DEMARC |  | JAENDI | ITABAS |
|  | DETORR |  | TAGER | KHOURY |
|  | FERRI1 |  | VIEGI2 | MADOR |
|  | FERRI2 |  |  | MARAN1 |
|  | FUKUCH |  |  | MARAN2 |
|  | GEIJER |  |  | MATHES |
|  | HEDMAN |  |  | SARGEA |
|  | JOHANN |  |  | TSUSHI |
|  | KIM |  |  | ZIETKO |
|  | KOJIMA |  |  |  |
|  | KOTAN2 |  |  |  |
|  | LIAW |  |  |  |
|  | LIU2 |  |  |  |
|  | MANNI2 |  |  |  |
|  | MENEZ2 |  |  |  |
|  | MENEZ3 |  |  |  |
|  | MENEZ4 |  |  |  |
|  | MENEZ5 |  |  |  |
|  | MENEZ6 |  |  |  |
|  | PEREZP |  |  |  |
|  | PRICE |  |  |  |
|  | SHIN |  |  |  |
|  | SPEIZE |  |  |  |
|  | WATSON |  |  |  |
|  | XIAO |  |  |  |
|  | XU |  |  |  |
|  | ZIELI1 |  |  |  |
|  | ZIELI2 |  |  |  |
| Duration |  |  |  |  |
|  | BEST |  | DETORR | AMIGO |
|  | LIAW |  |  | VESTBO |
|  | WEISS |  |  | ZIETKO |
| Years quit (vs never/long-term) |  |  |  |  |
|  | DEAN1 | VINEIS |  | ZIETKO |
|  | FORAST | WALD |  |  |
|  | HUHTI2 |  |  |  |
|  | JACOBS |  |  |  |
|  | KAHN |  |  |  |
|  | KRZYZA |  |  |  |
|  | MANNI2 |  |  |  |
| Years quit (vs current/recent) |  |  |  |  |
|  | DEAN1 | WALD |  |  |
|  | FORAST |  |  |  |
|  | HUHTI2 |  |  |  |
|  | JACOBS |  |  |  |
|  | KAHN |  |  |  |
|  | KRZYZA |  |  |  |
|  | MANNI2 |  |  |  |

| Studies with RRs for CB and: | Type of RRs^a^ |  |  |  |
| --- | --- | --- | --- | --- |
|  | **FullCat** | **PartCat** | **Regr** | **Other** |
| Amount smoked |  |  |  |  |
|  | **FullCat** | **PartCat** | **Regr** | **Other** |
|  | ALDERS |  | SAWICK | FINKLE |
|  | ANDER1 |  |  | HIGGI3 |
|  | BEST |  |  | JOUSI1 |
|  | BROWN |  |  | MELLST |
|  | CHAPMA |  |  | OSWAL1 |
|  | COATES |  |  | SUADIC |
|  | DEAN2 |  |  |  |
|  | DOLL1 |  |  |  |
|  | DOLL2 |  |  |  |
|  | DONTA2 |  |  |  |
|  | EHRLIC |  |  |  |
|  | FERRI1 |  |  |  |
|  | FLETCH |  |  |  |
|  | HAENSZ |  |  |  |
|  | HARRIS |  |  |  |
|  | HAWTHO |  |  |  |
|  | HIGGI2 |  |  |  |
|  | HIGGI3 |  |  |  |
|  | HIGGI6 |  |  |  |
|  | HOLLA2 |  |  |  |
|  | HOLLNA |  |  |  |
|  | HRUBEC |  |  |  |
|  | HUCHON |  |  |  |
|  | HUHTI1 |  |  |  |
|  | HUHTI3 |  |  |  |
|  | KAHN2 |  |  |  |
|  | LAMBER |  |  |  |
|  | LAVECC |  |  |  |
|  | LEBOWI |  |  |  |
|  | LINDST |  |  |  |
|  | MENEZ1 |  |  |  |
|  | MEREN |  |  |  |
|  | MILNE |  |  |  |
|  | MUELLE |  |  |  |
|  | OGILVI |  |  |  |
|  | REID |  |  |  |
|  | RIMING |  |  |  |
|  | SHARP |  |  |  |
|  | TROISI |  |  |  |
|  | URRUTI |  |  |  |
|  | WAGEN2 |  |  |  |
|  | WEN |  |  |  |
|  | WIG |  |  |  |
|  | WILHEL |  |  |  |
|  | WILSO2 |  |  |  |
|  | WOOLF |  |  |  |
|  | YAMAGU |  |  |  |
| Age started |  |  |  |  |
|  | ALDERS |  |  |  |
|  | HAENSZ |  |  |  |
|  | HAWTHO |  |  |  |
|  | OGILVI |  |  |  |
| Pack-years |  |  |  |  |
|  | ANDER1 | KUBIK | LANGHA | JENSEN |
|  | CERVER |  | SCHWAR | MOLLER |
|  | COATES |  |  | NEJJAR |
|  | FERRI1 |  |  |  |
|  | HUCHON |  |  |  |
|  | PEREZP |  |  |  |
|  | SOBRAD |  |  |  |
|  | TAGER2 |  |  |  |
| Duration |  |  |  |  |
|  | BEST |  | SAWICK | HUCHON |
|  | SAWICK |  |  |  |
|  | WEN |  |  |  |
| Years quit (vs never/long-term) |  |  |  |  |
|  | ALDERS |  |  | DEAN2 |
|  | DOLL1 |  |  |  |
|  | HAENSZ |  |  |  |
|  | HUHTI2 |  |  |  |
|  | KATO |  |  |  |
|  | TROISI |  |  |  |
|  | WILHEL |  |  |  |
| Years quit (vs current/recent) |  |  |  |  |
|  | ALDERS |  |  |  |
|  | DOLL1 |  |  |  |
|  | HAENSZ |  |  |  |
|  | HUHTI2 |  |  |  |
|  | KATO |  |  |  |
|  | TROISI |  |  |  |
|  | WILHEL |  |  |  |

| Studies with RRs for emphysema and: | Type of RRs^a^ |  |  |  |
| --- | --- | --- | --- | --- |
|  | **FullCat** | **PartCat** | **Regr** | **Other** |
|  |  |  |  |  |
| Amount smoked |  |  |  |  |
|  | AUERBA |  |  | ANDER2 |
|  | BEST |  |  | AUERBA |
|  | DONTA2 |  |  |  |
|  | HAMMO2 |  |  |  |
|  | HIRAYA |  |  |  |
|  | HUHTI1 |  |  |  |
|  | KAHN2 |  |  |  |
|  | LAVECC |  |  |  |
|  | WEISS |  |  |  |
|  | WEN |  |  |  |
|  | WILSO2 |  |  |  |
| Age started |  |  |  |  |
|  | HIRAYA |  |  |  |
| Pack-years |  |  |  |  |
|  | HIRAYA |  |  | ANDER2 |
|  | WANG2 |  |  | WANG2 |
| Duration |  |  |  |  |
|  | BEST |  |  |  |
|  | SUTINE |  |  |  |
|  | WEN |  |  |  |
| Years quit (vs never/long-term) |  |  |  |  |
|  |  |  |  | AUERBA |
|  |  |  |  | VIKGRE |

^a^  RRs are categorised as FullCat (full sets of categorical data), PartCat (partial categorical data), Regr (per unit dose regression), and Other. Note that for each outcome/measure, a study may appear in more than one column

## Table 7 Relative risks with apparent errors

## Table 7A RR non-central in CI

This listing shows the statistic (CENTR) calculated as (RR*RR)/(RRL*RRU), which should have the value 1.0. This listing shows those outside the range 0.9 to 1.2, arbitrarily chosen to exclude those where the apparent non-centrality is probably due to rounding error. However when RRL is very low and given only to 1 or 2 decimal places, values of CENTR may well fall outside that range, so entry in this list is not necessarily indicative of an error of real importance.

| **REF** | **NRR^a^** | **Outcome** | **RR** | **RRL** | **RRU** | **CENTR** | **DERIVE^b^** |
| --- | --- | --- | --- | --- | --- | --- | --- |
| DOLL1 | 30 | CB | 0.10 | 0.01 | 0.75 | 1.333 | m |
| DOLL1 | 33 | CB | 0.08 | 0.01 | 0.72 | 0.889 | m |
| EHRLIC | 14 | CB | 0.80 | 0.40 | 1.80 | 0.889 | 1 |
| KRZYZA | 21 | COPD | 0.66 | 0.03 | 16.85 | 0.862 | 7 |
| KRZYZA | 29 | COPD | 0.40 | 0.02 | 6.31 | 1.268 | 7 |
| KRZYZA | 31 | COPD | 0.29 | 0.02 | 5.15 | 0.817 | 7 |

## Table 7B Number of cases less than minimum implied by CI

This listing shows RRs which fail the check shown in formula 9 of Lee (1999) [15]. The statistic (R9)is calculated as 61.47 / Q^2^ (where Q=ln (rru/rrl)), and is compared with the number of cases(CA1+CA0). The ratio (r11 = r9 / (ca1+ca0)), which should be <= 1, is also shown, with larger values likely to be indicative of a more serious error. However, as indicated by the DERIVE code, the RR and CI may have been estimated, and this may account for some failures of this check. Note also this check has not been modified for prospective studies (St type = p), and should be regarded as only approximate (see formula 16 of [15]). Footnotes used in Table 7B are common to Tables 7B to 7D and are explained after Table 7D.

Note that the same RRs may also appear in the following section of this table (7C). Some of the fields defining the RR are shown, although not all due to lack of space.

| **REF** | **NRR^a^** | **Outcome** | **Sex^c^** | **Smk sta^d^** | **Adj^e^** | **Smk meas^f^** | **CA1^g^** | **CA0^g^** | **RR** | **RRL** | **RRU** | **R9** | **DERIVE^b^** | **St type** | **R11** |
| --- | --- | --- | --- | --- | --- | --- | --- | --- | --- | --- | --- | --- | --- | --- | --- |
|  |  |  |  |  |  |  |  |  |  |  |  |  |  |  |  |
| BECK2 | 2 | CB | m | ex | 0 | yes/no | 2 | 2 | 1.58 | 0.22 | 11.03 | 4.06 | 4 | p | 1.01 |
| BEST | 41 | COPD | m | c | 1 | c | 70 | 26 | 1.65 | 1.15 | 2.35 | 120.36 | m | p | 1.25 |
| BEST | 42 | COPD | m | c | 1 | c | 19 | 26 | 1.71 | 1.01 | 2.89 | 55.62 | m | p | 1.24 |
| BEST | 48 | COPD | m | c | 1 | y | 4 | 4 | 0.70 | 0.18 | 2.73 | 8.31 | m | p | 1.04 |
| BEST | 7 | CB | m | c | 1 | c | 49 | 17 | 1.94 | 1.24 | 3.04 | 76.44 | m | p | 1.16 |
| BEST | 8 | CB | m | c | 1 | c | 12 | 17 | 2.08 | 1.07 | 4.06 | 34.57 | m | p | 1.19 |
| BEST | 14 | CB | m | c | 1 | y | 2 | 3 | 0.50 | 0.09 | 2.92 | 5.08 | m | p | 1.02 |
| BEST | 15 | CB | m | c | 1 | y | 4 | 3 | 0.61 | 0.14 | 2.64 | 7.13 | m | p | 1.02 |
| BEST | 24 | Emp | m | c | 1 | c | 21 | 9 | 1.27 | 0.69 | 2.35 | 40.93 | m | p | 1.36 |
| BEST | 25 | Emp | m | c | 1 | c | 7 | 9 | 1.44 | 0.61 | 3.42 | 20.68 | m | p | 1.29 |
| CHEN2 | 8 | COPD | m | ex | 6 | yes/no | 4 | 8 | 3.20 | 1.40 | 7.31 | 22.50 | 1 | x | 1.88 |
| DOLL1 | 10 | COPD | m | c | 2 | c | 61 | 58 | 1.30 | 0.91 | 1.86 | 120.28 | other | p | 1.01 |
| DONTA2 | 2 | CB | m | ex | 0 | yes/no | 6 | 8 | 1.64 | 0.60 | 4.52 | 15.01 | 2 | p | 1.07 |
| DONTA2 | 5 | CB | m | c | 0 | c | 15 | 8 | 3.13 | 1.39 | 7.04 | 23.44 | 2 | p | 1.02 |
| DONTA2 | 7 | CB | m | c | 0 | c | 5 | 8 | 5.67 | 2.15 | 14.98 | 16.28 | 2 | p | 1.25 |
| DONTA2 | 8 | CB | m | c | 0 | c | 15 | 18 | 1.29 | 0.69 | 2.41 | 39.77 | 2 | p | 1.21 |
| DONTA2 | 9 | CB | m | c | 0 | c | 19 | 18 | 1.86 | 1.05 | 3.29 | 47.18 | 2 | p | 1.28 |
| DONTA2 | 15 | Emp | m | c | 0 | c | 4 | 4 | 1.67 | 0.43 | 6.49 | 8.35 | 2 | p | 1.04 |
| DONTA2 | 16 | Emp | m | c | 0 | c | 4 | 4 | 1.90 | 0.49 | 7.34 | 8.38 | 2 | p | 1.05 |
| DONTA2 | 20 | Emp | m | c | 0 | c | 1 | 1 | 8.43 | 0.56 | 127.42 | 2.08 | 2 | p | 1.04 |
| HARDIE | 5 | COPD | f | ex | 1 | yes/no | 25 | 31 | 2.32 | 1.39 | 3.88 | 58.33 | other | x | 1.04 |
| HARDIE | 10 | CB | f | c | 1 | yes/no | 7 | 11 | 4.60 | 1.88 | 11.24 | 19.22 | other | x | 1.07 |
| HARDIE | 11 | CB | f | ex | 1 | yes/no | 10 | 11 | 2.65 | 1.19 | 5.86 | 24.19 | other | x | 1.15 |
| HARDIE | 12 | CB | f | ever | 1 | yes/no | 16 | 11 | 3.33 | 1.59 | 6.98 | 28.09 | other | x | 1.04 |
| HARDIE | 16 | Emp | f | c | 1 | yes/no | 5 | 11 | 3.59 | 1.38 | 9.29 | 16.91 | other | x | 1.06 |
| HARDIE | 17 | Emp | f | ex | 1 | yes/no | 6 | 11 | 1.86 | 0.80 | 4.31 | 21.67 | other | x | 1.27 |
| HARDIE | 18 | Emp | f | ever | 1 | yes/no | 11 | 11 | 2.55 | 1.18 | 5.48 | 26.07 | other | x | 1.18 |
| HIGGI3 | 2 | CB | m | ex | 0 | yes/no | 1 | 0 | 1.56 | 0.06 | 43.93 | 1.38 | 7 | x | 1.38 |
| HIGGI3 | 7 | CB | f | c | 0 | yes/no | 1 | 0 | 9.92 | 0.39 | 253.48 | 1.46 | 7 | x | 1.46 |
| HIGGI3 | 8 | CB | f | ex | 0 | yes/no | 1 | 0 | 25.80 | 0.96 | 691.65 | 1.42 | 7 | x | 1.42 |
| JACOBS | 25 | COPD | m | c | 2 | c | 12 | 9 | 2.47 | 1.09 | 5.61 | 22.90 | other | p | 1.09 |
| KAHN | 12 | COPD | m | ex | 2 | a | 295 | 243 | 4.70 | 4.00 | 5.60 | 542.96 | 1 | p | 1.01 |
| KAHN | 16 | COPD | m | ex | 2 | a | 82 | 88 | 2.58 | 1.92 | 3.46 | 177.22 | e | p | 1.04 |
| KAHN | 18 | COPD | m | ex | 2 | c | 282 | 243 | 3.90 | 3.30 | 4.60 | 557.23 | 1 | p | 1.06 |
| KAHN | 23 | COPD | m | ex | 2 | c | 86 | 64 | 3.53 | 2.57 | 4.84 | 153.41 | e | p | 1.02 |
| KAHN2 | 105 | COPD | m | c | 1 | c | 26 | 28 | 3.63 | 2.15 | 6.13 | 56.00 | m | p | 1.04 |
| KAHN2 | 113 | COPD | m | c | 1 | c | 21 | 15 | 3.95 | 2.07 | 7.56 | 36.64 | m | p | 1.02 |
| KAHN2 | 139 | COPD | m | ever | 1 | c | 42 | 37 | 3.79 | 2.45 | 5.86 | 80.83 | m | p | 1.02 |
| KAHN2 | 9 | CB | m | c | 1 | c | 5 | 6 | 3.24 | 1.01 | 10.39 | 11.31 | m | p | 1.03 |
| KAHN2 | 13 | CB | m | c | 1 | c | 12 | 13 | 4.57 | 2.09 | 10.02 | 25.02 | m | p | 1.00 |
| KAHN2 | 17 | CB | m | c | 1 | c | 4 | 5 | 2.29 | 0.63 | 8.28 | 9.26 | m | p | 1.03 |
| KAHN2 | 54 | Emp | m | c | 1 | c | 21 | 22 | 3.74 | 2.08 | 6.73 | 44.58 | m | p | 1.04 |
| KAHN2 | 71 | Emp | m | ex | 1 | c | 10 | 8 | 3.45 | 1.37 | 8.68 | 18.03 | m | p | 1.00 |
| KAHN2 | 88 | Emp | m | ever | 1 | c | 31 | 30 | 3.49 | 2.13 | 5.72 | 62.99 | m | p | 1.03 |
| KRZYZA | 6 | COPD | f | ever | 1 | yes/no | 19 | 32 | 1.41 | 0.82 | 2.41 | 52.89 | other | p | 1.04 |
| KRZYZA | 7 | COPD | m | c | 1 | c | 8 | 5 | 2.24 | 0.79 | 6.39 | 14.07 | other | p | 1.08 |
| KRZYZA | 12 | COPD | f | c | 1 | c | 6 | 6 | 1.88 | 0.68 | 5.21 | 14.83 | other | p | 1.24 |
| KRZYZA | 14 | COPD | m | ex | 1 | q | 4 | 5 | 2.57 | 0.70 | 9.41 | 9.10 | other | p | 1.01 |
| KRZYZA | 21 | COPD | f | ex | 1 | q | 0 | 1 | 0.66 | 0.03 | 16.85 | 1.53 | 7 | p | 1.53 |
| LEE | 14 | COPD | m | c | 0 | c | 5 | 4 | 0.90 | 0.25 | 3.32 | 9.07 | 2 | p | 1.01 |
| LEE | 28 | COPD | m | c | 1 | c | 5 | 4 | 1.02 | 0.28 | 3.73 | 9.17 | m | p | 1.02 |
| LEE | 38 | COPD | f | c | 0 | yes/no | 5 | 5 | 1.34 | 0.39 | 4.62 | 10.04 | 4 | p | 1.00 |
| LEE | 47 | COPD | f | c | 0 | c | 2 | 2 | 0.56 | 0.08 | 3.99 | 4.02 | 2 | p | 1.00 |
| MANNI3 | 1 | COPD | b | c | 0 | yes/no | 48 | 36 | 1.39 | 0.91 | 2.13 | 84.16 | 6 | p | 1.00 |
| MANNI3 | 2 | COPD | b | ex | 0 | yes/no | 28 | 36 | 1.62 | 1.00 | 2.64 | 64.66 | 6 | p | 1.01 |
| MARAN2 | 2 | COPD | b | ex | 0 | yes/no | 16 | 20 | 4.36 | 2.29 | 8.29 | 37.17 | 1 | p | 1.03 |
| MENEZ2 | 5 | COPD | b | ex | 5 | yes/no | 50 | 52 | 1.27 | 0.86 | 1.85 | 104.76 | other | x | 1.03 |
| MILLER | 11 | Emp | f | ex | 1 | yes/no | 0 | 1 | 2.85 | 0.08 | 99.49 | 1.21 | 7 | x | 1.21 |
| NILSSO | 8 | COPD | m | ex | 2 | yes/no | 14 | 13 | 1.80 | 0.85 | 3.83 | 27.12 | k | p | 1.00 |
| NILSSO | 13 | COPD | m | c | 2 | c | 36 | 19 | 2.56 | 1.54 | 4.26 | 59.38 | e | p | 1.08 |
| NILSSO | 18 | COPD | f | c | 2 | c | 31 | 18 | 3.11 | 1.78 | 5.42 | 49.58 | e | p | 1.01 |
| TODD | 14 | COPD | m | c | 0 | c | 6 | 6 | 0.93 | 0.30 | 2.87 | 12.23 | 2 | p | 1.02 |
| TODD | 28 | COPD | m | c | 1 | c | 6 | 6 | 1.15 | 0.37 | 3.52 | 12.11 | m | p | 1.01 |
| TODD | 33 | COPD | m | c | 0 | a | 7 | 8 | 0.51 | 0.19 | 1.40 | 15.17 | 2 | p | 1.01 |
| TODD | 35 | COPD | m | c | 2 | a | 7 | 8 | 0.60 | 0.22 | 1.64 | 15.23 | m | p | 1.02 |
| TODD | 41 | COPD | f | ever | 0 | yes/no | 4 | 4 | 0.67 | 0.17 | 2.67 | 8.01 | 4 | p | 1.00 |
| WALD | 2 | COPD | m | ex | 0 | q | 1 | 1 | 4.46 | 0.28 | 71.32 | 2.00 | 2 | p | 1.00 |
| YUAN | 10 | COPD | m | c | 2 | c | 20 | 19 | 2.00 | 1.10 | 3.80 | 40.00 | 1 | p | 1.03 |

## Table 7C Number in any cell of 2x2 table less than minimum implied by CI

This listing shows RRs which fail the check shown in formula 10 of Lee (1999) [15]. The statistic (R10) is calculated as 15.3664 / Q^2^ (where Q=ln (rru/rrl)), and is compared with each of the exposed and unexposed cases (CA1, CA0) for all RRs, and also with the exposed and unexposed controls/disease-free (CO1,CO0) for unadjusted RRs from case-control or cross-sectional studies. The ratio (r12 = r10 / min(ca1,ca0,co1,co0)), which should be <= 1, is also shown. Footnotes used in Table 7C are common to Tables 7B to 7D and are explained after Table 7D.

See other notes at start of previous section (7B). Note also that this check may not apply to prospective studies with small numbers at risk ([15] section 3.5), which explains the appearance in this list of nrr 10 from DONTA2.

| **REF** | **NRR^a^** | **Outcome** | **Sex^c^** | **Smk sta^d^** | **Adj^e^** | **Smk mea^f^** | **CA1^g^** | **CA0^g^** | **CO1^g^** | **CO0^g^** | **RR** | **RRL** | **RRU** | **R10** | **DERIVE^b^** | **St type** | **R12** |
| --- | --- | --- | --- | --- | --- | --- | --- | --- | --- | --- | --- | --- | --- | --- | --- | --- | --- |
|  |  |  |  |  |  |  |  |  |  |  |  |  |  |  |  |  |  |
| BEST | 41 | COPD | m | c | 1 | c | 70 | 26 | - | - | 1.65 | 1.15 | 2.35 | 30.09 | m | p | 1.16 |
| BEST | 51 | COPD | m | c | 1 | y | 55 | 4 | - | - | 0.79 | 0.30 | 2.10 | 4.06 | m | p | 1.01 |
| BEST | 7 | CB | m | c | 1 | c | 49 | 17 | - | - | 1.94 | 1.24 | 3.04 | 19.11 | m | p | 1.12 |
| BEST | 24 | Emp | m | c | 1 | c | 21 | 9 | - | - | 1.27 | 0.69 | 2.35 | 10.23 | m | p | 1.14 |
| BEST | 34 | Emp | m | c | 1 | y | 14 | 1 | - | - | 1.03 | 0.15 | 7.24 | 1.02 | m | p | 1.02 |
| CHEN2 | 8 | COPD | m | ex | 6 | yes/no | 4 | 8 | - | - | 3.20 | 1.40 | 7.31 | 5.63 | 1 | x | 1.41 |
| CHEN2 | 11 | COPD | f | ex | 6 | yes/no | 7 | 33 | - | - | 1.26 | 0.67 | 2.34 | 9.82 | 1 | x | 1.40 |
| DONTA2 | 10 | CB | m | c | 0 | c | 5 | 18 | 14 | 118 | 2.34 | 1.03 | 5.32 | 5.69 | 2 | p | 1.14 |
| ENSTRO | 13 | COPD | m | c | 1 | c | 326 | 35 | - | - | 2.92 | 2.10 | 4.07 | 35.09 | e | p | 1.00 |
| HARDIE | 7 | CB | m | c | 1 | yes/no | 11 | 1 | - | - | 18.13 | 3.52 | 93.37 | 1.43 | other | x | 1.43 |
| HARDIE | 8 | CB | m | ex | 1 | yes/no | 26 | 1 | - | - | 8.70 | 1.78 | 42.53 | 1.53 | other | x | 1.53 |
| HARDIE | 9 | CB | m | ever | 1 | yes/no | 37 | 1 | - | - | 10.90 | 2.06 | 57.56 | 1.39 | other | x | 1.39 |
| HARDIE | 14 | Emp | m | ex | 1 | yes/no | 38 | 2 | - | - | 11.67 | 3.01 | 45.15 | 2.10 | other | x | 1.05 |
| HIRAYA | 10 | Emp | f | c | 1 | p | 2 | 42 | - | - | 10.93 | 2.80 | 42.64 | 2.07 | other | p | 1.04 |
| HUCHON | 22 | CB | b | c | 1 | c | 3 | 177 | - | - | 1.81 | 0.61 | 5.34 | 3.26 | 7 | x | 1.09 |
| HUCHON | 25 | CB | b | c | 1 | c | 114 | 3 | - | - | 1.81 | 0.61 | 5.35 | 3.26 | 7 | x | 1.09 |
| HUCHON | 26 | CB | b | c | 1 | c | 145 | 3 | - | - | 4.82 | 1.64 | 14.19 | 3.30 | 7 | x | 1.10 |
| HUHTI3 | 4 | COPD | m | c | 1 | yes/no | 68 | 3 | - | - | 8.96 | 3.02 | 26.65 | 3.24 | 7 | x | 1.08 |
| HUHTI3 | 6 | COPD | m | ever | 1 | yes/no | 84 | 3 | - | - | 7.74 | 2.62 | 22.87 | 3.27 | 7 | x | 1.09 |
| HUHTI3 | 12 | COPD | m | c | 1 | c | 42 | 3 | - | - | 9.77 | 3.22 | 29.61 | 3.12 | 7 | x | 1.04 |
| JACOBS | 13 | COPD | m | ex | 2 | x | 24 | 216 | - | - | 0.83 | 0.56 | 1.24 | 24.32 | other | p | 1.01 |
| KIM | 13 | COPD | f | ever | 1 | p | 1 | 61 | - | - | 0.47 | 0.08 | 2.69 | 1.24 | other | x | 1.24 |
| KRZYZA | 10 | COPD | f | c | 1 | c | 6 | 32 | - | - | 1.40 | 0.65 | 3.01 | 6.54 | other | p | 1.09 |
| LANGE | 1 | COPD | m | c | 1 | yes/no | 86 | 3 | - | - | 4.98 | 1.61 | 15.44 | 3.01 | e | p | 1.00 |
| LANGE | 3 | COPD | m | ever | 1 | yes/no | 111 | 3 | - | - | 4.50 | 1.45 | 13.90 | 3.01 | e | p | 1.00 |
| OMORI | 6 | Emp | m | ever | 1 | yes/no | 135 | 3 | - | - | 9.73 | 3.18 | 29.82 | 3.07 | 7 | x | 1.02 |
| PANDEY | 4 | CB | m | c | 2 | yes/no | 211 | 4 | - | - | 5.33 | 2.49 | 11.38 | 6.65 | other | x | 1.66 |
| PANDEY | 6 | CB | m | ever | 2 | yes/no | 238 | 4 | - | - | 5.37 | 2.52 | 11.45 | 6.71 | other | x | 1.68 |

## Table 7D Other

This listing shows comments on the databases indicating other problems, including results or analyses which were omitted or amended due to apparent problems in the original paper(s)

Some of the fields defining the RR are shown, although not all due to lack of space.

| **REF** | **NRR^a^** | **Outcome** | **Sex^c^** | **Smok stat^d^** | **Adj^e^** | **Smok meas^f^** | **RR** | **Comment** |
| --- | --- | --- | --- | --- | --- | --- | --- | --- |
|  |  |  |  |  |  |  |  |  |
| BEST | 18 | Emp | m | curr | 1 | yes/no | 7.72 | Alternative result referring to cigarette only smokers who may have smoked cigar/pipe in the past is 5.85 (1.80-18.97), 37 deaths. However there may be an error in the source table as the number of deaths (37) is less than for those who only ever smoked cigarettes (40) |
| BEST | 20 | Emp | m | ever | 1 | yes/no | 8.47 | Alternative result including current cigarette only smokers who may have smoked cigar/pipe in the past is 6.73 (2.10-21.59). However there may be an error in the source table as the number of deaths (49) is less than for only ever cigarette smokers (52) |
| BEST | 35 | COPD | m | curr | 1 | yes/no | 9.57 | Alternative result referring to cigarette only smokers who may have smoked cigar/pipe in the past is 8.75 (3.85-19.86) (or 8.71 [39] Table II), 115 deaths. However there may be an error in the source Table as the number of emphysema deaths (37) is less than among only ever cigarette smokers (40) |
| BEST | 37 | COPD | m | ever | 1 | yes/no | 10.02 | Alternative result including current cigarette only smokers who may have smoked pipe/cigar in the past is 9.24 (4.08-20.91). However there may be any error in the source table as the number of emphysema deaths (49) is less than among only ever cigarette smokers (52) |
| CHEN2 | 13 | COPD | m | curr | 6 | c | 1.72 | Table 3, p758 [47] gives prevalence of COPD (for both current and ex-smokers) from which unadjusted ORs might be estimated. However the CIs of the prevalences seem implausibly similar given the large differences in group size, so this has not been attempted |
| CHEN2 | 14 | COPD | m | curr | 6 | c | 4.66 | as above |
| CHEN2 | 15 | COPD | m | curr | 6 | c | 2.71 | as above |
| CHEN2 | 16 | COPD | f | curr | 6 | c | 2.61 | as above |
| CHEN2 | 17 | COPD | f | curr | 6 | c | 5.46 | as above |
| CHEN2 | 18 | COPD | f | curr | 6 | c | 2.09 | as above |
| CHEN2 | 19 | COPD | m | curr | 6 | a | 3.29 | as above |
| CHEN2 | 20 | COPD | m | curr | 6 | a | 3.94 | as above |
| CHEN2 | 21 | COPD | m | curr | 6 | a | 1.20 | as above |
| CHEN2 | 22 | COPD | f | curr | 6 | a | 2.89 | as above |
| CHEN2 | 23 | COPD | f | curr | 6 | a | 5.42 | as above |
| CHEN2 | 24 | COPD | f | curr | 6 | a | 1.88 | as above |
| CHEN3 | 3 | COPD | m | curr | 2 | yes/no | 3.22 | Model additionally adjusted for amount smoked is also shown, but as the base for comparison is never smokers the validity of that model is unclear |
| CHEN3 | 4 | COPD | f | curr | 2 | yes/no | 3.11 | as above |
| CHEN3 | 8 | COPD | f | curr | 0 | a | 2.60 | as above |
| CHEN3 | 9 | COPD | f | curr | 0 | a | 4.30 | as above |
| CHEN3 | 10 | COPD | f | curr | 0 | a | 1.65 | as above |
| CHEN3 | 14 | COPD | f | curr | 2 | a | 2.56 | as above |
| CHEN3 | 15 | COPD | f | curr | 2 | a | 3.60 | as above |
| CHEN3 | 16 | COPD | f | curr | 2 | a | 1.41 | as above |
| DEAN1 | 19 | COPD | m | curr | 0 | c | 4.11 | There is a small discrepancy in the numbers of controls smoking 1-12 cigarettes between Table F5 (as used) and supplementary Table 9. |
| DEAN1 | 22 | COPD | m | curr | 0 | c | 0.46 | as above |
| DEAN1 | 23 | COPD | m | curr | 0 | c | 0.73 | as above |
| DEAN1 | 24 | COPD | m | curr | 3 | c | 3.22 | as above |
| DEAN1 | 27 | COPD | m | curr | 3 | c | 0.80 | as above |
| DEAN1 | 28 | COPD | m | curr | 3 | c | 2.44 | as above |
| DETORR | 1 | COPD | m | ever | 0 | p | 0.99 | Number of cases discrepant from data given in Table 1 |
| DETORR | 2 | COPD | m | ever | 0 | p | 1.55 | Number of cases discrepant from data given in Table 1. Sex-specific data (as entered) are also discrepant from sexes-combined data also shown in Figure 1 |
| DETORR | 3 | COPD | m | ever | 0 | p | 1.72 | Number of cases discrepant from data given in Table 1. Sex-specific data (as entered) are also discrepant from sexes-combined data also shown in Figure 1 |
| DETORR | 4 | COPD | f | ever | 0 | p | 3.24 | Number of cases discrepant from data given in Table 1 |
| DETORR | 5 | COPD | f | ever | 0 | p | 4.93 | Number of cases discrepant from data given in Table 1. Sex-specific data (as entered) are also discrepant from sexes-combined data also shown in Figure 1 |
| DETORR | 6 | COPD | f | ever | 0 | p | 4.79 | as above |
| DOLL2 | 1 | COPD | f | curr | 1 | yes/no | 18.42 | RRs by amount smoked given in personal communication from J.Peto (11.4, 32.9, 43.7) differ from those calculated from rate ratios from [68] (10.5, 28.5, 32.0) |
| FERRI1 | 1 | COPD | m | curr | 1 | yes/no | 4.25 | Discrepancy in numbers of subjects between Tables 5 and 6 in [75], and unclear why. Difference in results between [75] and [77] for 1961 survey probably due to use of different standardizing population |
| FERRI1 | 2 | COPD | m | ex | 1 | yes/no | 0.98 | as above |
| FERRI1 | 3 | COPD | m | ever | 1 | yes/no | 3.50 | as above |
| FERRI1 | 4 | COPD | m | curr | 1 | yes/no | 4.80 | as above |
| FERRI1 | 5 | COPD | m | curr | 1 | yes/no | 5.20 | as above |
| FERRI1 | 6 | COPD | m | ex | 1 | yes/no | 1.31 | as above |
| FERRI1 | 7 | COPD | m | ever | 1 | yes/no | 4.93 | as above |
| FERRI1 | 8 | COPD | f | curr | 1 | yes/no | 2.09 | as above |
| FERRI1 | 9 | COPD | f | ex | 1 | yes/no | 1.11 | as above |
| FERRI1 | 10 | COPD | f | ever | 1 | yes/no | 1.92 | as above |
| FERRI1 | 11 | COPD | f | curr | 1 | yes/no | 2.09 | as above |
| FERRI1 | 12 | COPD | f | curr | 1 | yes/no | 1.80 | as above |
| FERRI1 | 13 | COPD | f | ex | 1 | yes/no | 1.06 | as above |
| FERRI1 | 14 | COPD | f | ever | 1 | yes/no | 1.70 | as above |
| FERRI1 | 15 | COPD | m | curr | 1 | yes/no | 4.71 | as above |
| FERRI1 | 16 | COPD | m | curr | 1 | yes/no | 4.26 | as above |
| FERRI1 | 17 | COPD | f | curr | 1 | yes/no | 2.09 | as above |
| FERRI1 | 18 | COPD | f | curr | 1 | yes/no | 2.09 | as above |
| FERRI1 | 19 | COPD | m | curr | 1 | c | 5.06 | Discrepancy in numbers of subjects between Tables 5 and 6 in [75], and unclear why, so alternative denominator of never any not entered for dose-response data. Results for different levels of numbers of cigarettes also available from [77], but using different standardizing population |
| FERRI1 | 20 | COPD | m | curr | 1 | c | 2.88 | as above |
| FERRI1 | 21 | COPD | m | curr | 1 | c | 3.49 | as above |
| FERRI1 | 22 | COPD | m | curr | 1 | c | 5.24 | as above |
| FERRI1 | 23 | COPD | m | curr | 1 | c | 9.47 | as above |
| FERRI1 | 24 | COPD | m | curr | 1 | c | 55.36 | as above |
| FERRI1 | 25 | COPD | m | curr | 1 | c | 0.57 | as above |
| FERRI1 | 26 | COPD | m | curr | 1 | c | 0.69 | as above |
| FERRI1 | 27 | COPD | m | curr | 1 | c | 1.04 | as above |
| FERRI1 | 28 | COPD | m | curr | 1 | c | 1.87 | as above |
| FERRI1 | 29 | COPD | m | curr | 1 | c | 10.95 | as above |
| FERRI1 | 30 | COPD | f | curr | 1 | c | 0.82 | as above |
| FERRI1 | 31 | COPD | f | curr | 1 | c | 1.56 | as above |
| FERRI1 | 32 | COPD | f | curr | 1 | c | 3.07 | as above |
| FERRI1 | 33 | COPD | f | curr | 1 | c | 2.12 | as above |
| FERRI1 | 34 | COPD | f | curr | 1 | c | 4.13 | as above |
| FERRI1 | 35 | COPD | f | curr | 1 | c | 4.50 | as above |
| FERRI1 | 36 | COPD | f | curr | 1 | c | 1.90 | as above |
| FERRI1 | 37 | COPD | f | curr | 1 | c | 3.74 | as above |
| FERRI1 | 38 | COPD | f | curr | 1 | c | 2.59 | as above |
| FERRI1 | 39 | COPD | f | curr | 1 | c | 5.04 | as above |
| FERRI1 | 40 | COPD | f | curr | 1 | c | 5.49 | as above |
| FERRI1 | 41 | COPD | m | ever | 1 | p | 1.40 | Discrepancy in numbers of subjects between Tables 5 and 6 in [75], and unclear why, so alternative denominator of never any not used for dose-response data |
| FERRI1 | 42 | COPD | m | ever | 1 | p | 3.63 | as above |
| FERRI1 | 43 | COPD | m | ever | 1 | p | 3.02 | as above |
| FERRI1 | 44 | COPD | m | ever | 1 | p | 3.98 | as above |
| FERRI1 | 45 | COPD | m | ever | 1 | p | 4.52 | as above |
| FERRI1 | 46 | COPD | m | ever | 1 | p | 2.59 | as above |
| FERRI1 | 47 | COPD | m | ever | 1 | p | 2.15 | as above |
| FERRI1 | 48 | COPD | m | ever | 1 | p | 2.85 | as above |
| FERRI1 | 49 | COPD | m | ever | 1 | p | 3.23 | as above |
| FERRI1 | 50 | COPD | f | ever | 1 | p | 0.97 | as above |
| FERRI1 | 51 | COPD | f | ever | 1 | p | 3.07 | as above |
| FERRI1 | 52 | COPD | f | ever | 1 | p | 2.33 | as above |
| FERRI1 | 53 | COPD | f | ever | 1 | p | 5.07 | as above |
| FERRI1 | 54 | COPD | f | ever | 1 | p | 2.29 | as above |
| FERRI1 | 55 | COPD | f | ever | 1 | p | 3.15 | as above |
| FERRI1 | 56 | COPD | f | ever | 1 | p | 2.40 | as above |
| FERRI1 | 57 | COPD | f | ever | 1 | p | 5.21 | as above |
| FERRI1 | 58 | COPD | f | ever | 1 | p | 2.36 | as above |
| FUKUCH | 1 | COPD | b | curr | 0 | yes/no | 2.96 | Rate ratio calculated from these data is 2.66, which disagrees with unadjusted rate ratio shown of 2.52 (without CI). Equivalent adjusted result not used because the multivariate model is over-specified by the inclusion of both smoking status and pack-years |
| FUKUCH | 2 | COPD | b | ex | 0 | yes/no | 2.99 | Rate ratio calculated from these data is 2.68, which disagrees with unadjusted rate ratio shown of 2.52 (without CI). Equivalent adjusted results not used because multivariate model is over-specified by inclusion of both smoking status and pack-years |
| FUKUCH | 3 | COPD | b | ever | 0 | yes/no | 2.97 | Equivalent adjusted result not used because the multivariate model is over-specified by inclusion of both smoking status and pack-years |
| FUKUCH | 4 | COPD | b | ever | 0 | p | 1.18 | as above |
| FUKUCH | 5 | COPD | b | ever | 0 | p | 3.53 | as above |
| FUKUCH | 6 | COPD | b | ever | 0 | p | 7.41 | as above |
| FUKUCH | 7 | COPD | b | ever | 0 | p | 2.98 | as above |
| FUKUCH | 8 | COPD | b | ever | 0 | p | 6.26 | as above |
| GEIJER | 1 | COPD | m | curr | 0 | p | 2.06 | Recalculated as RR instead of OR. There would be a minor discrepancy between the OR given in Table 4 and the OR as calculated from Table 3 (not entered) |
| GODTFR | S | COPD |  |  |  | y |  | Results for duration of smoking available from [18] for CCHS and GPS separately. However results are presented in a model with terms for current/noninhalers, current/inhalers and ex smokers, and for amount smoked, with never smokers included (although all never smoking deaths were in females), so this is difficult to interpret |
| HARDIE | 1 | COPD | m | curr | 1 | yes/no | 7.77 | Table 2 estimates total number of cases as 114 in men |
| HARDIE | 2 | COPD | m | ex | 1 | yes/no | 4.63 | as above |
| HARDIE | 3 | COPD | m | ever | 1 | yes/no | 5.43 | as above |
| HARDIE | 4 | COPD | f | curr | 1 | yes/no | 3.41 | Table 2 estimates total number of cases as 74 in women |
| HARDIE | 5 | COPD | f | ex | 1 | yes/no | 2.32 | as above |
| HARDIE | 6 | COPD | f | ever | 1 | yes/no | 2.75 | as above |
| HARDIE | 7 | CB | m | curr | 1 | yes/no | 18.13 | Table 2 estimates total number of cases as 40 in men |
| HARDIE | 8 | CB | m | ex | 1 | yes/no | 8.70 | as above |
| HARDIE | 9 | CB | m | ever | 1 | yes/no | 10.90 | as above |
| HARDIE | 13 | Emp | m | curr | 1 | yes/no | 15.04 | Table 2 estimates total number of cases as 52 in men |
| HARDIE | 14 | Emp | m | ex | 1 | yes/no | 11.67 | as above |
| HARDIE | 15 | Emp | m | ever | 1 | yes/no | 13.38 | as above |
| HUCHON | 3 | CB | b | ever | 0 | yes/no | 2.23 | Sum of subjects is greater than total subjects in study, possibly due to rounding |
| HUCHON | 27 | CB | b | ex | 0 | p | 0.75 | Percentages given for no symptoms and chronic cough groups in original table sum to 123.7% and 90.9% respectively |
| HUCHON | 28 | CB | b | ex | 0 | p | 1.54 | as above |
| HUCHON | 29 | CB | b | ex | 0 | p | 2.15 | as above |
| HUCHON | 30 | CB | b | ex | 0 | p | 2.06 | as above |
| HUCHON | 31 | CB | b | ex | 0 | p | 2.87 | as above |
| HUCHON | 37 | CB | b | ex | 1 | p | 0.80 | as above |
| HUCHON | 38 | CB | b | ex | 1 | p | 1.44 | as above |
| HUCHON | 39 | CB | b | ex | 1 | p | 1.55 | as above |
| HUCHON | 40 | CB | b | ex | 1 | p | 1.81 | as above |
| HUCHON | 41 | CB | b | ex | 1 | p | 2.03 | as above |
| HUCHON | 47 | CB | b | ever | 0 | p | 1.44 | as above |
| HUCHON | 48 | CB | b | ever | 0 | p | 2.54 | as above |
| HUCHON | 49 | CB | b | ever | 0 | p | 3.56 | as above |
| HUCHON | 50 | CB | b | ever | 0 | p | 1.77 | as above |
| HUCHON | 51 | CB | b | ever | 0 | p | 2.47 | as above |
| HUCHON | 57 | CB | b | ever | 1 | p | 1.59 | as above |
| HUCHON | 58 | CB | b | ever | 1 | p | 2.63 | as above |
| HUCHON | 59 | CB | b | ever | 1 | p | 2.97 | as above |
| HUCHON | 60 | CB | b | ever | 1 | p | 1.68 | as above |
| HUCHON | 61 | CB | b | ever | 1 | p | 2.12 | as above |
| JINDA2 | 2 | CB | b | ever | 0 | yes/no | 1.83 | Results given by author from univariate analysis as 2.076 (1.712-2.518) |
| JOHANN | 4 | COPD | b | ever | 0 | p | 1.93 | An analysis by pack-years adjusted for sex, age, BMI, education, occupational exposure and residential area is presented in Fig 2 p845 of JOHANN2005A, but as it includes both smoking status (nev/cur/ex), this appears not to be a sensible analysis |
| JOHANN | 5 | COPD | b | ever | 0 | p | 3.70 | as above |
| JOHANN | 6 | COPD | b | ever | 0 | p | 8.05 | as above |
| JOHANN | 7 | COPD | b | ever | 0 | p | 1.92 | as above |
| JOHANN | 8 | COPD | b | ever | 0 | p | 4.17 | as above |
| KACHEL | 1 | COPD | b | curr | 0 | yes/no | 5.32 | A RR of 6.52 (2.82-15.13) adjusted for sex, age and occupational exposure is also given, but has not been entered as other results from the adjusted model appear erroneous |
| KACHEL | 2 | COPD | b | ex | 0 | yes/no | 1.78 | A RR of 2.55 (1.68-3.89) adjusted for sex, age and occupational exposure is also given but appears erroneous, as the CI is too narrow for the number of ex-smoking cases |
| KHOURY | 1 | COPD | b | curr | 9 | c | 4.06 | Alternative results estimated from prevalence rates stratified by family history of COPD or lung cancer are similar: 4.09 (2.68-6.23). Alternatives estimated from results stratified by PiZ allele or ABH non-secretor status are a little higher (4.36 (2.75-6.90) and 4.59 (2.85-7.40) respectively) but are based on fewer subjects. There is an unexplained discrepancy in the stratified prevalence rates among never smokers between Tables 2 and 3 of KHOURY1986 |
| KHOURY | 2 | COPD | b | curr | 9 | p | * | Alternative results estimated from prevalence rates stratified by family history of COPD or lung cancer are similar (as are results estimated from rates stratified by PiZ allele or ABH non-secretor status, which are based on fewer subjects). There is an unexplained discrepancy between the stratified prevalence rates in never smokers between Tables 2 and 3 of KHOURY1986 |
| KOJIMA | 1 | COPD | m | curr | 0 | yes/no | 3.37 | Correcting for typographical error (i.e. assuming number of current smoking cases is 118, not 188 as shown) |
| KOJIMA | 3 | COPD | m | ever | 0 | yes/no | 3.02 | as above |
| KOTAN2 | S | COPD |  | ever |  |  |  | An analysis adjusted for sex, age and 5 other factors was rejected because it also reallocated cases with concomitant physician-diagnosed asthma and <10 pkyrs as non-cases |
| KUBIK | S |  |  |  |  |  |  | More complete results available from graph on p32 but scale is too small to be accurately extracted |
| KUBIK | 2 | CB | f | curr | 0 | p | 8.05 | Table 3 states total number of female subjects as 31613, but age groups add up to total of 31629 |
| KUBIK | 4 | CB | f | ever | 0 | yes/no | 2.02 | as above |
| LAI | 1 | COPD | m | ever | 0 | yes/no | 5.62 | Numbers estimated from %s, but assuming that male never smoker %, given as 4.9%, should be 3.9% in order to be compatible with female and sexes-combined data also given |
| LAI | 1 | COPD | m | ever | 0 | yes/no | 5.62 | RR/CI calculated from % as given (i.e. using 4.9% for never smokers rather than 3.9% corrected for assumed typographic error) are 4.45 (2.83-7.01) |
| LEBOWI | S |  |  |  |  |  |  | Results from paper [172] omitted due to considerable inconsistencies in data presented |
| LUNDB1 | 7 | COPD | b | curr | 4 | yes/no | 6.53 | One of the RRs used (5-14 cigs/day vs never, RR 6.44 (3.37-11.6) fails the centrality check (value C=1.06), cannot be explained by rounding |
| LUNDB1 | 9 | COPD | b | ever | 4 | yes/no | 3.16 | as above |
| LUNDB1 | 11 | COPD | b | curr | 4 | c | 6.44 | Fails centrality check (C=1.06), cannot be explained by rounding |
| LUNDB1 | 13 | COPD | b | curr | 4 | c | 1.47 | as above |
| LUNDB1 | 14 | COPD | b | curr | 4 | c | 1.83 | as above |
| NIHLEN | 1 | COPD | b | curr | 0 | yes/no | 3.08 | % of never smoking cases and number of never smokers at risk estimated by subtraction, numbers of cases then estimated from % distribution. There is a small discrepancy in the overall incidence rate implied by this method (2.61%) compared to that given (2.9%) |
| NIHLEN | 1 | COPD | b | curr | 0 | yes/no | 3.08 | OR originally given as 2.6, p < 0.001 (Table 3 p266 [220]) |
| NIHLEN | 2 | COPD | b | ex | 0 | yes/no | 3.26 | as above |
| NIHLEN | 2 | COPD | b | ex | 0 | yes/no | 3.26 | OR originally given as 1.2, p = 0.521 (Table 3 p266 [220]) |
| NIHLEN | 3 | COPD | b | ever | 0 | yes/no | 3.19 | as above |
| PEAT | 1 | COPD | m | curr | 0 | yes/no | 5.70 | RR calculated from % distribution by sex, which is discrepant from the distribution by also given and used for NRR 3 |
| PEAT | 2 | COPD | f | curr | 0 | yes/no | 2.74 | as above |
| PEAT | 3 | COPD | b | curr | 1 | yes/no | 4.85 | Assuming Table 3 labels in total rows are reversed, and that numbers in text p33 are correct |
| PEAT | 3 | COPD | b | curr | 1 | yes/no | 4.85 | RR calculated from % distribution by age, which is discrepant from the distribution by sex also given and used for NRRs 1 and 2 |
| RENWIC | 1 | COPD | b | curr | 0 | yes/no | 4.40 | Total number of cases estimated at 68 from % distribution, but stated to be 65 on Page 166. |
| RENWIC | 2 | COPD | b | ex | 0 | yes/no | 2.51 | as above |
| RENWIC | 3 | COPD | b | ever | 0 | yes/no | 3.31 | as above |
| STROM | S | COPD |  |  |  |  |  | Total number of cases is stated to be 173, but total by smoking status adds up to 174 |
| SUADIC | S |  |  |  |  |  |  | Results for current vs non-smoking rejected as inappropriately adjusted for inhalation |
| VIEGI2 | 5 | COPD | m | ex | 1 | yes/no | 1.42 | Number of cases lower than for all ages combined when calculated from % given in table 3 |
| VIEGI2 | 6 | COPD | m | ever | 1 | yes/no | 1.39 | as above |
| VIEGI2 | 7 | COPD | f | curr | 0 | yes/no | 0.95 | Correcting for typographical error in number of subjects (shown as 156, should be 456) |
| VIEGI2 | 8 | COPD | f | ex | 0 | yes/no | 0.70 | as above |
| VIEGI2 | 9 | COPD | f | ever | 0 | yes/no | 0.83 | as above |
| VOLLM1 | 1 | COPD | b | curr | 0 | yes/no | 2.11 | Number of non-smokers given as 211 in Table 4, but totals in Table 5 add up to 212 or 210, while % given in Table 2 gives total of 209 |
| VOLLM2 | 1 | COPD | b | curr | 0 | yes/no | 6.13 | as above |
| WANG2 | 1 | Emp | b | ever | 0 | yes/no | 5.53 | Tables 4 and 5 on Page 355 give numbers of cases as 39 for never smokers and 191 for ever smokers |
| WANG2 | 2 | Emp | b | ever | 0 | p | 4.16 | as above |
| WANG2 | 3 | Emp | b | ever | 0 | p | 7.06 | Tables 4 and 5 on Page 355 give numbers of cases as 39 for never smokers and 191 for ever smokers |
| WANG2 | 4 | Emp | b | ever | 0 | p | 1.70 | as above |
| WATSON | 1 | COPD | b | ever | 0 | p | 1.54 | Originally given as 1.61 (0.90, 2.88) |
| WATSON | 2 | COPD | b | ever | 0 | p | 1.82 | Originally given as 1.90 (1.02, 3.54) |
| WEN | S | COPD |  |  |  | y |  | Results by duration of smoking for COPD have been rejected due to apparent implausibility |

^a^ Number of relative risk within the study on database; S indicates a general comment for the study.

^b^ Method of derivation of the RR/CI. 1 indicates as given by original author; 7 indicates use of “0.5 correction” for a zero cell; k and m indicate CI estimated from crude numbers for an adjusted RR; see the extra file Databases (available on request) for meanings of the other codes.

^c^ Sex : m=male, f=female, b=both

^d^ Smoking status: ever, curr=current or ex

^e^ Adj: number of adjustment factors

^f^ Measure of exposure: yes/no for major smoking indices; for dose-related indices c=cigarettes/day (amount smoked), a=age started smoking, p=pack-years, y=duration of smoking (years), q=duration of quitting (vs never), x=duration of quitting (vs current)

^g^ CA=numbers of cases, CO=numbers of controls/at risk. 1=exposed group (e.g. smokers), 0=unexposed group (e.g never smokers)
