# Supplementary material for: Systematic review with meta-analysis of the epidemiological evidence relating smoking to COPD, chronic bronchitis and emphysema
Source: BMC Pulm Med. 2011 Jun 14;11:36. doi: 10.1186/1471-2466-11-36 (PMC3128042; doi:10.1186/1471-2466-11-36)
Supplement: Additional file 4 — MetaMajorCOPD. .RTF file giving the full results of the meta-analyses for the major smoking variables-ever smoking, current smoking, ever or current smoking, and ex smoking-for COPD. [file 1471-2466-11-36-S4.RTF]

Systematic review with meta-analysis of the epidemiological evidence relating smoking to COPD, chronic bronchitis and emphysema

Barbara A Forey, Alison J Thornton and Peter N Lee

Additional file 4 : MetaMajorCOPD

See Additional file 10 (Intro sheet) for list of tables and page numbers

                                                    Table 1 - A - 1 -

            IESCOPD - Meta-analysis of ever smoking, any product (or cigarettes if all product not available)
                                                         Any COPD


This analysis is restricted to results for:
1) Eligible study on database
2) Outcome COPD
3) Non-dose-response data
4) Ever smoking
5) Results complete enough for use in meta-analysis

Within each study, results are then selected (in the following order of preference, within each sex) for:
6) UNEXP   : never any, never cigarettes, other
7) PROD    : any product, cigarettes, cigarettes only
8) For overlapping studies: principal rather than subsidiary studies
and then for single sex results (m, f) in preference to results for both sexes combined (b).

Results adjusted for the most potential confounders are then chosen in Sections -1 to -3
and results adjusted for the least confounders in Sections -4 to -6. (Those least adjusted results which
actually differ from the most adjusted are marked 'x' in column X in Section -4)

Section -7 shows excluded studies, together with the stage (as above) at which no qualifying
results were found.

Section -8 lists the potentially overlapping studies which have been included (1=principal, 2=subsidiary),
and any results which would have been included in preference except that they had data not complete enough
for use in meta-analysis. It also lists their significance (yes/no), if known.


  ________________________________________________________________________________________________________________________
                                            International Evidence on Smoking and COPD, Phase 3, Analysis run on 27-SEP-10

                                                   Table 1 - A - 1 - 1

            IESCOPD - Meta-analysis of ever smoking, any product (or cigarettes if all product not available)
                                                         Any COPD
                                                      Most-adjusted


     REF|NRR|SEX|AGEL|AGEH|     REGION|BEGYR|PUBYR|STTYP|ONSET|      DISEAS|ADJ|SMOKSTA|   PRODUCT|    UNEXP|

  ALESSA   3   b   45   81     Eu:West  1992  1994    CC  Prev COPD:LF/symp   0    Ever       Cigs  Nev cigs
   AMIGO  11   m    0   99 Am:Sth/Cent  2001  2006    CC  Prev   COPD/CB/EM   6    Ever       Cigs  Nev cigs
   AMIGO  12   f    0   99 Am:Sth/Cent  2001  2006    CC  Prev   COPD/CB/EM   6    Ever       Cigs  Nev cigs
  ANDER1   3   m   25   74   Am:Canada  1963  1965    CS  Prev     COPD:oth   1    Ever        Any   Nev any
  ANDER1   6   f   25   74   Am:Canada  1963  1965    CS  Prev     COPD:oth   1    Ever        Any   Nev any
  ANDER3   3   m    0   99     Eu:East     *  2001    CC  Prev      COPD:LF   0    Ever        Any   Nev any
  ANDER3   6   f    0   99     Eu:East     *  2001    CC  Prev      COPD:LF   0    Ever        Any   Nev any
  BEDNAR   3   b   30   99     Eu:East  2000  2005    CS  Prev      COPD:LF   0    Ever        Any   Nev any
    BEST  37   m   30   97   Am:Canada  1955  1967    Pr   Inc    COPD:mort   1    Ever  Cigs only   Nev any
   CHEN2   9   m   35   64   Am:Canada  1994  2000    CS  Prev        CB/EM   6    Ever       Cigs  Nev cigs
   CHEN2  12   f   35   64   Am:Canada  1994  2000    CS  Prev        CB/EM   6    Ever       Cigs  Nev cigs
  CLEMEN   3   m   20   60     Eu:West  1960  1982    Pr  Prev      COPD:LF   0    Ever        Any   Nev any
   COCCI   2   b   38   78     Eu:West     *  2002    CC  Prev COPD:LF/symp   0    Ever       Cigs  Nev cigs
   DEAN1  13   m   35   99       Eu:UK  1969  1977    CC  Prev    COPD:mort   3    Ever        Any   Nev any
   DEAN1  17   f   35   99       Eu:UK  1969  1977    CC  Prev    COPD:mort   3    Ever MCigs only   Nev any
  DEJONG   3   b    0   99      Am:USA     *  2004    CS  Prev      COPD:LF   0    Ever       Cigs  Nev cigs
  DEMARC   6   b   20   44       Multi  1991  2004    CS  Prev      COPD:LF   4    Ever        Any   Nev any
  DICKIN   3   b   60   74       Eu:UK     *  1999    CS  Prev      COPD:LF   0    Ever        Any   Nev any
   DOLL1   3   m   20   99       Eu:UK  1951  1994    Pr   Inc    COPD:mort   2    Ever        Any   Nev any
   DOLL2   3   f   20   99       Eu:UK  1951  1980    Pr   Inc    COPD:mort   1    Ever  Cigs only   Nev any
  EKBERG   3   m   15   99    Eu:Scand  1974  2005    CS  Prev      COPD:LF   0    Ever        Any   Nev any
  EKBERG   6   f   15   99    Eu:Scand  1974  2005    CS  Prev      COPD:LF   0    Ever        Any   Nev any
  FERRI2   3   m   25   80      Am:USA  1967  1971    CS  Prev     COPD:oth   1    Ever        Any   Nev any
  FERRI2  10   f   25   80      Am:USA  1967  1971    CS  Prev     COPD:oth   1    Ever        Any   Nev any
   FIDAN   2   m   15   99 Eu:SE/Balkn  2000  2004    CS  Prev     COPD:oth   3    Ever       Cigs  Nev cigs
  FORAST   6   f   55   99      Am:USA  1993  1998    CS  Prev        CB/EM   1    Ever       Cigs  Nev cigs
  FUKUCH   3   b   40   99   Asia:FarE  2000  2004    CS  Prev      COPD:LF   0    Ever       Cigs  Nev cigs
  GODTFR   3   b   20   99    Eu:Scand  1964  2002    Pr   Inc     COPD:ICD   3    Ever        Any   Nev any
  GULSVI   1   m   15   70    Eu:Scand  1972  1979    CS  Prev     COPD:oth   0    Ever        Any   Nev any
  GULSVI   2   f   15   70    Eu:Scand  1972  1979    CS  Prev     COPD:oth   0    Ever        Any   Nev any
  HAMMO2   5   m   35   99      Am:USA  1959  1966    Pr   Inc    COPD:mort   1    Ever  Cigs only   Nev any
  HAMMO2   6   f   35   99      Am:USA  1959  1966    Pr   Inc    COPD:mort   1    Ever  Cigs only   Nev any
  HARDIE   3   m   70   99    Eu:Scand  1998  2005    CS  Prev    CB/EM/Ast   1    Ever       Cigs  Nev cigs
  HARDIE   6   f   70   99    Eu:Scand  1998  2005    CS  Prev    CB/EM/Ast   1    Ever       Cigs  Nev cigs
  HARIKK   3   m   40   99      Am:USA  1962  2002    Pr   Inc     COPD:oth   0    Ever        Any   Nev any
  HARIKK   6   f   40   99      Am:USA  1962  2002    Pr   Inc     COPD:oth   0    Ever        Any   Nev any
  HIGGI4   3   m   35   99      Am:USA  1962  1989    Pr   Inc    COPD:mort   1    Ever       Cigs  Nev cigs
      HO   3   m   70   99   Asia:FarE  1991  1999    CS  Prev   COPD:undef   0    Ever       Cigs  Nev cigs
      HO   6   f   70   99   Asia:FarE  1991  1999    CS  Prev   COPD:undef   0    Ever       Cigs  Nev cigs
  HOZAWA   3   b   45   64      Am:USA  1987  2006    CS  Prev      COPD:LF   0    Ever       Cigs  Nev cigs
  HUHTI1   6   m   40   64    Eu:Scand  1961  1965    CS  Prev    CB/EM/Ast   1    Ever        Any   Nev any
  HUHTI1  14   f   40   64    Eu:Scand  1961  1965    CS  Prev    CB/EM/Ast   1    Ever        Any   Nev any
  HUHTI3   6   m   25   69    Eu:Scand  1968  1978    CS  Prev      COPD:LF   1    Ever        Any   Nev any
  ITABAS   1   b   60   99   Asia:FarE     *  1990    CC  Prev      COPD:LF   0    Ever       Cigs  Nev cigs
  JACOBS   6   m   40   84       Multi  1957  1999    Pr   Inc    COPD:mort   6    Ever       Cigs  Nev cigs
  JOHANN   3   b   26   82    Eu:Scand  1996  2005    CS  Prev      COPD:LF   0    Ever       Cigs  Nev cigs
  KACHEL   3   b   20   79     Eu:East     *  2003    CS  Prev      COPD:LF   0    Ever       Cigs  Nev cigs
   KAHN2 131   m   31   84      Am:USA  1954  1966    Pr   Inc    COPD:mort   1    Ever        Any   Nev any
  KARAKA   3   b   15   99 Eu:SE/Balkn     *  2003   nCC  Prev   COPD/CB/EM   0    Ever        Any   Nev any
  KATANC   3   b   70   79      Am:USA  1997  2005    CS  Prev      COPD:LF   0    Ever       Cigs  Nev cigs
     KIM   3   m   45   99   Asia:FarE  2001  2005    CS  Prev      COPD:LF   1    Ever       Cigs  Nev cigs
     KIM   6   f   45   99   Asia:FarE  2001  2005    CS  Prev      COPD:LF   1    Ever       Cigs  Nev cigs
   KIRAZ   2   f   25   99 Eu:SE/Balkn  1999  2003    CS  Prev COPD:LF/symp   1    Ever       Cigs  Nev cigs
  KLAYTO   1   b   40   99      Am:USA     *  1975    CS  Prev      COPD:LF   0    Ever       Cigs  N/L cigs
  KOJIMA   3   m   25   74   Asia:FarE  2001  2005    CS  Prev      COPD:LF   0    Ever       Cigs  Nev cigs
  KOJIMA   6   f   25   74   Asia:FarE  2001  2005    CS  Prev      COPD:LF   0    Ever       Cigs  Nev cigs
  KRZYZA   3   m   19   83     Eu:East  1968  1986    Pr   Inc      COPD:LF   1    Ever       Cigs  Nev cigs
  KRZYZA   6   f   19   83     Eu:East  1968  1986    Pr   Inc      COPD:LF   1    Ever       Cigs  Nev cigs
     LAI   1   m   18   80   Asia:FarE  2001  2006    CS  Prev      COPD:LF   0    Ever        Any   Nev any
     LAI   2   f   18   80   Asia:FarE  2001  2006    CS  Prev      COPD:LF   0    Ever        Any   Nev any
    LAM1   1   m   35   99   Asia:FarE  1976  1997    Pr   Inc    COPD:mort   7    Ever       Cigs  Nev cigs
    LAM1   2   f   35   99   Asia:FarE  1976  1997    Pr   Inc    COPD:mort   7    Ever       Cigs  Nev cigs
    LAM2   3   m   60   99   Asia:FarE  1987  2002    CS  Prev   COPD:undef   0    Ever       Cigs  Nev cigs
    LAM3   3   m   60   99   Asia:FarE  1987  2006    Pr   Inc    COPD:mort   7    Ever       Cigs  Nev cigs
  LEBOWI   6   m   15   96      Am:USA  1972  1977    CS  Prev      COPD:LF   1    Ever       Cigs  Nev cigs
  LEBOWI  18   f   15   96      Am:USA  1972  1977    CS  Prev      COPD:LF   1    Ever       Cigs  Nev cigs
     LEE  20   m   35   82       Eu:UK  1964  1979    Pr   Inc    COPD:mort   1    Ever        Any   Nev any
     LEE  52   f   35   82       Eu:UK  1964  1979    Pr   Inc    COPD:mort   1    Ever        Any   Nev any
  LINDBE   3   b   46   84    Eu:Scand  1996  2006    Pr   Inc      COPD:LF   3    Ever        Any   Nev any
  LINDST   9   b   20   69    Eu:Scand     *  2001    CS  Prev        CB/EM   5    Ever       Cigs  Nev cigs
  LUNDB1   9   b   46   77    Eu:Scand  1996  2003    CS  Prev      COPD:LF   4    Ever       Cigs  Nev cigs
   MADOR   3   m   15   99      Am:USA     *  2003    CC  Prev      COPD:LF   0    Ever       Cigs  Nev cigs
  ________________________________________________________________________________________________________________________
                                            International Evidence on Smoking and COPD, Phase 3, Analysis run on 27-SEP-10

                                                   Table 1 - A - 1 - 1

            IESCOPD - Meta-analysis of ever smoking, any product (or cigarettes if all product not available)
                                                         Any COPD
                                                      Most-adjusted


     REF|NRR|SEX|AGEL|AGEH|     REGION|BEGYR|PUBYR|STTYP|ONSET|      DISEAS|ADJ|SMOKSTA|   PRODUCT|    UNEXP|

  MANNI1   1   m   17   99      Am:USA  1988  2000    CS  Prev      COPD:LF   2    Ever        Any   Nev any
  MANNI1  10   f   17   99      Am:USA  1988  2000    CS  Prev      COPD:LF   2    Ever       Cigs   Nev any
  MANNI2   3   b   25   74      Am:USA  1971  2003    CS  Prev      COPD:LF   1    Ever        Any   Nev any
  MANNI3   3   b   25   96      Am:USA  1971  2003    Pr   Inc    COPD:mort   0    Ever        Any   Nev any
  MARAN1   3   b   60   99 Asia:SE/Pac  1998  2002    CS  Prev     COPD:oth   0    Ever       Cigs  N/L cigs
  MARAN2   3   b   60   99 Asia:SE/Pac  1998  2002    Pr   Inc     COPD:oth   0    Ever       Cigs  N/L cigs
  MARCUS   3   m   45   85      Am:USA  1965  1989    Pr   Inc    COPD:mort   1    Ever        Any   Nev any
  MATHES   3   b   45   70      Aus/NZ     *  2006    CC  Prev      COPD:LF   0    Ever       Cigs  Nev cigs
  MENEZ2   6   b   40   99 Am:Sth/Cent  2003  2005    CS  Prev      COPD:LF   5    Ever       Cigs  Nev cigs
  MENEZ3   3   b   40   99 Am:Sth/Cent  2003  2005    CS  Prev      COPD:LF   0    Ever       Cigs  Nev cigs
  MENEZ4   3   b   40   99 Am:Sth/Cent  2003  2005    CS  Prev      COPD:LF   0    Ever       Cigs  Nev cigs
  MENEZ5   3   b   40   99 Am:Sth/Cent  2003  2005    CS  Prev      COPD:LF   0    Ever       Cigs  Nev cigs
  MENEZ6   3   b   40   99 Am:Sth/Cent  2003  2005    CS  Prev      COPD:LF   0    Ever       Cigs  Nev cigs
  MONTNE   3   b   20   59    Eu:Scand  1992  1998    CS  Prev        CB/EM   0    Ever        Any   Nev any
  MUELLE   6   m   20   69      Am:USA  1967  1971    CS  Prev      COPD:LF   1    Ever        Any   Nev any
  MUELLE  13   f   20   69      Am:USA  1967  1971    CS  Prev      COPD:LF   1    Ever        Any   Nev any
  NIEPSU   3   b   19   69     Eu:East  2001  2002    CS  Prev      COPD:LF   0    Ever       Cigs  Nev cigs
  NIHLEN   3   b   20   67    Eu:Scand  1992  2004    Pr   Inc   COPD/CB/EM   0    Ever        Any   Nev any
  NILSSO   9   m   18   99    Eu:Scand  1963  2001    Pr   Inc    COPD:mort   2    Ever        Any   Nev any
  NILSSO   6   f   18   99    Eu:Scand  1963  2001    Pr   Inc    COPD:mort   2    Ever  Cigs only   Nev any
  PEREZP   1   f   40   99 Am:Sth/Cent  1992  1996    CC  Prev      COPD:LF   0    Ever       Cigs  Nev cigs
    PETO   3   m   25   92       Eu:UK  1954  1983    Pr   Inc    COPD:mort   0    Ever       Cigs  Nev cigs
  RENWIC   3   b   45   99       Eu:UK  1992  1996    CS  Prev      COPD:LF   0    Ever        Any   Nev any
  RICCIO   3   b   25   99     Eu:West  2002  2005    CS  Prev      COPD:LF   0    Ever       Cigs  Nev cigs
  SARGEA   9   b   45   74       Eu:UK  1993  2000    CS  Prev     COPD:oth   5    Ever       Cigs  Nev cigs
  SHAHAB   3   b   35   99       Eu:UK  2001  2006    CS  Prev      COPD:LF   0    Ever       Cigs  Nev cigs
    SHIN   5   b   18   99   Asia:FarE  1999  2003    CS  Prev      COPD:LF   3    Ever       Cigs  Nev cigs
  SICHLE   1   b   21   80 Eu:SE/Balkn  2000  2005    CS  Prev     COPD:oth   3    Ever       Cigs  Nev cigs
   SILVA   3   b   20   99      Am:USA  1972  2004    Pr   Inc COPD:LF/symp   6    Ever       Cigs  Nev cigs
  SPEIZE   3   m   25   86      Am:USA  1974  1989    Pr   Inc    COPD:mort   1    Ever       Cigs  Nev cigs
  SPEIZE   6   f   25   86      Am:USA  1974  1989    Pr   Inc    COPD:mort   1    Ever       Cigs  Nev cigs
   STROM   3   m   68   68    Eu:Scand  1982  1996    CS  Prev      COPD:LF   0    Ever        Any   Nev any
    TANG   4   m   35   99       Eu:UK  1967  1995    Pr   Inc    COPD:mort   2    Ever        Any   Nev any
    THUN   5   m   30   99      Am:USA  1982  1997    Pr   Inc    COPD:mort   1    Ever  Cigs only   Nev any
    THUN   6   f   30   99      Am:USA  1982  1997    Pr   Inc    COPD:mort   1    Ever  Cigs only   Nev any
    TODD  20   m   35   81       Eu:UK  1965  1978    Pr   Inc    COPD:mort   1    Ever        Any   Nev any
    TODD  46   f   35   81       Eu:UK  1965  1978    Pr   Inc    COPD:mort   1    Ever        Any   Nev any
  TRUPIN   6   b   55   75      Am:USA  2001  2005    CS  Prev   COPD/CB/EM   4    Ever       Cigs  Nev cigs
  TSUSHI   3   b    0   99   Asia:FarE  2003  2006    CS  Prev      COPD:LF   0    Ever       Cigs  Nev cigs
  TVERDA   3   m   35   65    Eu:Scand  1972  1993    Pr   Inc    COPD:mort   2    Ever       Cigs  Nev cigs
  VESTBO   3   b   20   99    Eu:Scand  1976  2002    CS  Prev      COPD:LF   0    Ever        Any   Nev any
  VIEGI2   6   m   25   73     Eu:West  1988  2000    CS  Prev      COPD:LF   1    Ever       Cigs  Nev cigs
  VIEGI2  12   f   25   73     Eu:West  1988  2000    CS  Prev      COPD:LF   1    Ever       Cigs  Nev cigs
  VONHER   3   m   30   99    Eu:Scand  1978  2000    CS  Prev        CB/EM   0    Ever        Any   Nev any
  VONHER   6   f   30   99    Eu:Scand  1978  2000    CS  Prev        CB/EM   0    Ever        Any   Nev any
   WEISS   3   m   50   69      Am:USA  1961  1963    CS  Prev      COPD:LF   0    Ever        Any   Nev any
  WILSO1   1   b   18   99      Aus/NZ  2000  2005    CS  Prev      COPD:LF   0    Ever        Any   Nev any
    XIAO   3   b    0   99   Asia:FarE     *  2004    CC  Prev   COPD:undef   0    Ever       Cigs  Nev cigs
      XU   2   m   35   99   Asia:FarE  2000  2005    CS  Prev        CB/EM  13    Ever       Cigs  Nev cigs
      XU   4   f   35   99   Asia:FarE  2000  2005    CS  Prev        CB/EM  13    Ever       Cigs  Nev cigs
  YAMAGU   3   b   40   99   Asia:FarE  1986  1988    CS  Prev      COPD:LF   6    Ever       Cigs  Nev cigs
    YUAN   5   m   45   71   Asia:FarE  1986  1996    Pr   Inc    COPD:mort   2    Ever       Cigs  Nev cigs
  ZIELI1   2   b    0   99     Eu:East  1999  2001    CS  Prev      COPD:LF   1    Ever       Cigs  Nev cigs
  ZIELI2   3   m    0   99     Eu:East  2000  2006    CS  Prev      COPD:LF   0    Ever       Cigs  Nev cigs
  ZIELI2   6   f    0   99     Eu:East  2000  2006    CS  Prev      COPD:LF   0    Ever       Cigs  Nev cigs
  ZIETKO   2   m    0   99     Eu:East     *  2005    CC  Prev      COPD:LF   0    Ever       Cigs  Nev cigs
  ZIETKO   4   f    0   99     Eu:East     *  2005    CC  Prev      COPD:LF   0    Ever       Cigs  Nev cigs


  ________________________________________________________________________________________________________________________
                                            International Evidence on Smoking and COPD, Phase 3, Analysis run on 27-SEP-10

                                                   Table 1 - A - 1 - 2

            IESCOPD - Meta-analysis of ever smoking, any product (or cigarettes if all product not available)
                                                         Any COPD
                                                      Most-adjusted


                        Number Exposed  Non-exposed
 REF    NRR SEX ADJ     Case    Cont    Case    Cont      RR        95.00%CI
 ALESSA 3   b   0         26      16      11      14      2.07 (  0.76-   5.65)
 AMIGO  11  m   6         40       -       3       -      4.65 (  0.98-  22.09)
 AMIGO  12  f   6         38       -       6       -      7.88 (  2.55-  24.36)
 Subtotal AMIGO                                           6.57 (  2.64-  16.39)
 ANDER1 3   m   1         31       -       2       -      5.13 (  1.14-  23.05)
 ANDER1 6   f   1         11       -      16       -      1.18 (  0.53-   2.64)
 Subtotal ANDER1                                          1.63 (  0.81-   3.32)
 ANDER3 3   m   0         15       8       0       8     31.00~(  1.59- 605.65)
 ANDER3 6   f   0          2       1       3       3      2.00 (  0.11-  35.81)
 Subtotal ANDER3                                          7.56 (  0.95-  59.91)
 BEDNAR 3   b   0         52     376      20     228      1.58 (  0.92-   2.71)
*BEST   37  m   1        129       -       6       -     10.02 (  4.42-  22.72)
 CHEN2  9   m   6         49       -       8       -      3.43 (  1.60-   7.38)
 CHEN2  12  f   6         97       -      33       -      2.57 (  1.62-   4.07)
 Subtotal CHEN2                                           2.78 (  1.87-   4.12)
 CLEMEN 3   m   0         57    1629      11     709      2.26 (  1.18-   4.33)
 COCCI  2   b   0         20      13       0       6     19.74~(  1.03- 379.94)
 DEAN1  13  m   3        483       -      47       -      2.54 (  1.76-   3.66)
 DEAN1  17  f   3         98       -     120       -      1.92 (  1.37-   2.69)
 Subtotal DEAN1                                           2.18 (  1.70-   2.80)
 DEJONG 3   b   0         55     154       0      34     24.79~(  1.49- 411.13)
 DEMARC 6   b   4        381       -     156       -      1.76 (  1.44-   2.15)
 DICKIN 3   b   0         34     167       1     125     25.45 (  3.44- 188.43)
*DOLL1  3   m   2        522       -      12       -      6.54 (  3.71-  11.53)
*DOLL2  3   f   1         12       -       1       -     11.02 (  1.43-  84.78)
 EKBERG 3   m   0       2249    7692     395    2841      2.10 (  1.87-   2.36)
 EKBERG 6   f   0        546    3300     166    2581      2.57 (  2.15-   3.08)
 Subtotal EKBERG                                          2.23 (  2.02-   2.46)
 FERRI2 3   m   1        214       -      11       -      3.88 (  2.01-   7.49)
 FERRI2 10  f   1         87       -      54       -      2.28 (  1.57-   3.31)
 Subtotal FERRI2                                          2.59 (  1.88-   3.59)
 FIDAN  2   m   3         53       -       2       -      6.96 (  1.45-  33.26)
 FORAST 6   f   1         61       -      28       -      2.36 (  1.48-   3.77)
 FUKUCH 3   b   0        192    1048      64    1039      2.97 (  2.21-   4.00)
*GODTFR 3   b   3       1189       -      71       -      5.46 (  4.20-   7.10)
 GULSVI 1   m   0         54       -       1       -     18.60 (  2.52- 137.03)
 GULSVI 2   f   0         63       -      22       -      3.52 (  1.88-   6.58)
 Subtotal GULSVI                                          4.09 (  2.25-   7.43)
*HAMMO2 5   m   1          -       -       -       -      9.27 (  6.76-  12.70)
*HAMMO2 6   f   1          -       -       -       -      5.87 (  4.17-   8.26)
 Subtotal HAMMO2                                          7.51 (  5.96-   9.47)
 HARDIE 3   m   1        104       -       7       -      5.43 (  2.55-  11.58)
 HARDIE 6   f   1         41       -      31       -      2.75 (  1.72-   4.39)
 Subtotal HARDIE                                          3.32 (  2.23-   4.95)
*HARIKK 3   m   0         33     319       7     139      2.05 (  0.93-   4.53)
*HARIKK 6   f   0          7      91       0     101     16.64~(  0.96- 287.29)
 Subtotal HARIKK                                          2.39 (  1.11-   5.11)
*HIGGI4 3   m   1          -       -       -       -      3.08 (  1.72-   5.52)
 HO     3   m   0         88     604      20     282      2.05 (  1.24-   3.41)
 HO     6   f   0         27     243      30     733      2.71 (  1.58-   4.66)
 Subtotal HO                                              2.34 (  1.62-   3.38)
 HOZAWA 3   b   0       2589    5379     872    5019      2.77 (  2.54-   3.02)
 HUHTI1 6   m   1        200       -       7       -      8.18 (  3.68-  18.18)
 HUHTI1 14  f   1         21       -      56       -      2.88 (  1.62-   5.13)
 Subtotal HUHTI1                                          4.12 (  2.58-   6.57)
 HUHTI3 6   m   1         84       -       3       -      7.74 (  2.62-  22.87)
 ITABAS 1   b   0         44      35      16      25      1.96 (  0.91-   4.24)
*JACOBS 6   m   6        248       -      28       -      3.08 (  2.03-   4.65)
 JOHANN 3   b   0        134    1237      22     842      4.15 (  2.62-   6.56)
 KACHEL 3   b   0         41     688       7     503      4.28 (  1.91-   9.62)
*KAHN2  131 m   1        496       -      31       -      6.69 (  4.63-   9.66)
 KARAKA 3   b   0         59      87      25      81      2.20 (  1.26-   3.84)
 KATANC 3   b   0         54     403      21     382      2.44 (  1.44-   4.11)
 KIM    3   m   1        179       -      23       -      2.31 (  1.44-   3.72)
 KIM    6   f   1         10       -      61       -      1.74 (  0.85-   3.57)
 Subtotal KIM                                             2.12 (  1.43-   3.15)
 KIRAZ  2   f   1          9       -      25       -      6.86 (  2.47-  19.06)
 KLAYTO 1   b   0         62     117       7      76      5.75 (  2.50-  13.24)
 KOJIMA 3   m   0        169    5515      19    1871      3.02 (  1.87-   4.86)
 KOJIMA 6   f   0          5     316      20    3545      2.80 (  1.05-   7.52)
 Subtotal KOJIMA                                          2.98 (  1.94-   4.57)
  ________________________________________________________________________________________________________________________
                                            International Evidence on Smoking and COPD, Phase 3, Analysis run on 27-SEP-10

                                                   Table 1 - A - 1 - 2

            IESCOPD - Meta-analysis of ever smoking, any product (or cigarettes if all product not available)
                                                         Any COPD
                                                      Most-adjusted


                        Number Exposed  Non-exposed
 REF    NRR SEX ADJ     Case    Cont    Case    Cont      RR        95.00%CI
*KRZYZA 3   m   1         57       -       5       -      2.63 (  1.09-   6.38)
*KRZYZA 6   f   1         19       -      32       -      1.41 (  0.82-   2.41)
 Subtotal KRZYZA                                          1.67 (  1.05-   2.65)
 LAI    1   m   0        107     469      20     493      5.62 (  3.43-   9.22)
 LAI    2   f   0          7      88      27     600      1.77 (  0.75-   4.18)
 Subtotal LAI                                             4.22 (  2.75-   6.48)
*LAM1   1   m   7         19       -       4       -      4.05 (  1.31-  12.50)
*LAM1   2   f   7          2       -       2       -     26.61 (  2.08- 339.00)
 Subtotal LAM1                                            5.51 (  1.97-  15.47)
 LAM2   3   m   0        246     634      49     339      2.68 (  1.92-   3.75)
*LAM3   3   m   7         29       -       9       -      1.81 (  0.83-   3.98)
 LEBOWI 6   m   1         41       -       4       -      5.02 (  1.76-  14.35)
 LEBOWI 18  f   1         50       -      36       -      1.66 (  1.07-   2.59)
 Subtotal LEBOWI                                          1.96 (  1.31-   2.95)
*LEE    20  m   1         40       -       1       -      4.73 (  0.65-  34.28)
*LEE    52  f   1         10       -       5       -      1.74 (  0.60-   5.09)
 Subtotal LEE                                             2.18 (  0.85-   5.59)
*LINDBE 3   b   3          -       -       -       -      2.28 (  1.34-   3.88)
 LINDST 9   b   5        315       -     166       -      1.84 (  1.50-   2.25)
 LUNDB1 9   b   4        138       -      39       -      3.16 (  2.01-   4.97)
 MADOR  3   m   0         19       7       0       3     18.20~(  0.84- 396.21)
 MANNI1 1   m   2        480       -     105       -      2.69 (  2.16-   3.34)
 MANNI1 10  f   2        305       -     156       -      3.18 (  2.61-   3.88)
 Subtotal MANNI1                                          2.95 (  2.55-   3.41)
 MANNI2 3   b   1        752       -     202       -      2.64 (  2.24-   3.12)
*MANNI3 3   b   0         76    2724      36    1895      1.47 (  0.99-   2.17)
 MARAN1 3   b   0        166     968      54    1905      6.05 (  4.41-   8.30)
*MARAN2 3   b   0         50     605      20    1324      5.47 (  3.29-   9.11)
*MARCUS 3   m   1         75       -      14       -      2.17 (  1.20-   3.90)
 MATHES 3   b   0         49      89      23     131      3.14 (  1.78-   5.51)
 MENEZ2 6   b   5        100       -      52       -      1.54 (  1.08-   2.17)
 MENEZ3 3   b   0        136       -      62       -      1.11 (  0.80-   1.54)
 MENEZ4 3   b   0         44       -      34       -      1.65 (  1.04-   2.63)
 MENEZ5 3   b   0        115       -      58       -      1.64 (  1.16-   2.33)
 MENEZ6 3   b   0        121       -      36       -      2.73 (  1.85-   4.03)
 MONTNE 3   b   0        301    4686      91    3390      2.39 (  1.88-   3.04)
 MUELLE 6   m   1         35       -       2       -      4.41 (  1.01-  19.20)
 MUELLE 13  f   1          3       -       2       -      1.60 (  0.26-   9.92)
 Subtotal MUELLE                                          2.95 (  0.94-   9.28)
 NIEPSU 3   b   0         34     191       8     177      3.94 (  1.78-   8.74)
*NIHLEN 3   b   0         91    2465      21    1815      3.19 (  1.99-   5.11)
*NILSSO 9   m   2         64       -      13       -      2.78 (  1.50-   5.14)
*NILSSO 6   f   2         63       -      57       -      5.93 (  4.02-   8.75)
 Subtotal NILSSO                                          4.78 (  3.44-   6.64)
 PEREZP 1   f   0         19      47      45     145      1.30 (  0.69-   2.44)
*PETO   3   m   0        104    2423       0     295     25.48~(  1.59- 409.05)
 RENWIC 3   b   0         59     119       9      60      3.31 (  1.54-   7.12)
 RICCIO 3   b   0        321     478     178     537      2.03 (  1.62-   2.53)
 SARGEA 9   b   5        968       -     622       -      1.40 (  1.25-   1.58)
 SHAHAB 3   b   0        771    3758     322    3364      2.14 (  1.87-   2.46)
 SHIN   5   b   3         71       -      49       -      1.87 (  1.14-   3.09)
 SICHLE 1   b   3          -       -       -       -      2.17 (  1.63-   2.91)
*SILVA  3   b   6          -       -       -       -      1.59 (  0.96-   2.63)
*SPEIZE 3   m   1         26       -       0       -     11.40 (  0.69- 187.05)
*SPEIZE 6   f   1         14       -       5       -      4.20 (  1.51-  11.66)
 Subtotal SPEIZE                                          4.72 (  1.81-  12.34)
 STROM  3   m   0        157     233      17      71      2.81 (  1.60-   4.96)
*TANG   4   m   2          -       -       -       -      4.12 (  2.33-   7.28)
*THUN   5   m   1          -       -       -       -      8.96 (  6.66-  12.06)
*THUN   6   f   1          -       -       -       -      8.05 (  6.17-  10.50)
 Subtotal THUN                                            8.44 (  6.93-  10.29)
*TODD   20  m   1         59       -       1       -      6.88 (  0.96-  49.58)
*TODD   46  f   1          4       -       4       -      0.89 (  0.22-   3.57)
 Subtotal TODD                                            1.76 (  0.56-   5.49)
 TRUPIN 6   b   4        304       -      73       -      3.45 (  2.59-   4.59)
 TSUSHI 3   b   0         41    1142       7    1056      5.42 (  2.42-  12.12)
*TVERDA 3   m   2         55       -       7       -      2.88 (  1.31-   6.33)
 VESTBO 3   b   0       1667    8841     234    2366      1.91 (  1.65-   2.20)
 VIEGI2 6   m   1        168       -      21       -      1.39 (  0.83-   2.32)
 VIEGI2 12  f   1         52       -      66       -      1.20 (  0.80-   1.80)
 Subtotal VIEGI2                                          1.27 (  0.92-   1.75)
  ________________________________________________________________________________________________________________________
                                            International Evidence on Smoking and COPD, Phase 3, Analysis run on 27-SEP-10

                                                   Table 1 - A - 1 - 2

            IESCOPD - Meta-analysis of ever smoking, any product (or cigarettes if all product not available)
                                                         Any COPD
                                                      Most-adjusted


                        Number Exposed  Non-exposed
 REF    NRR SEX ADJ     Case    Cont    Case    Cont      RR        95.00%CI
 VONHER 3   m   0        655    1687      63     911      5.61 (  4.28-   7.36)
 VONHER 6   f   0        132     734     137    2888      3.79 (  2.95-   4.88)
 Subtotal VONHER                                          4.55 (  3.78-   5.47)
 WEISS  3   m   0         42     209       2      34      3.42 (  0.79-  14.77)
 WILSO1 1   b   0        112    1040      24     875      3.93 (  2.50-   6.16)
 XIAO   3   b   0         79      44      21      56      4.79 (  2.57-   8.92)
 XU     2   m   13       596       -     450       -      1.26 (  1.07-   1.48)
 XU     4   f   13        51       -     647       -      2.61 (  1.85-   3.68)
 Subtotal XU                                              1.44 (  1.24-   1.67)
 YAMAGU 3   b   6        332       -     144       -      1.97 (  1.60-   2.43)
*YUAN   5   m   2         34       -      19       -      1.40 (  0.80-   2.45)
 ZIELI1 2   b   1       2364       -     316       -      2.06 (  1.81-   2.35)
 ZIELI2 3   m   0      11618   36365     341    2611      2.45 (  2.18-   2.74)
 ZIELI2 6   f   0       5159   26196     679    4832      1.40 (  1.29-   1.53)
 Subtotal ZIELI2                                          1.71 (  1.60-   1.83)
 ZIETKO 2   m   0         35      11       0      12     77.17~(  4.23-1408.01)
 ZIETKO 4   f   0         12       6       0      11     44.23~(  2.23- 875.91)
 Subtotal ZIETKO                                         58.88 (  7.34- 472.11)
Partial Totals         41804  125697    8556   53383
*prospective study                                        ~ With 0.5 adjustment for zero


 REF    NRR SEX ADJ             Ys       Ws       Qs       Ps
 ALESSA 3   b   0              0.73     3.80     0.06       0.16
 AMIGO  11  m   6              1.54     1.58     0.75       0.05
 AMIGO  12  f   6              2.06     3.02     4.46       0.00
 Subtotal AMIGO                1.88     4.60     5.21
 ANDER1 3   m   1              1.64     1.70     1.05       0.03
 ANDER1 6   f   1              0.17     5.96     2.78       0.69
 Subtotal ANDER1               0.49     7.66     3.83
 ANDER3 3   m   0              3.43     0.43     2.91       0.02
 ANDER3 6   f   0              0.69     0.46     0.01       0.64
 Subtotal ANDER3               2.02     0.90     2.92
 BEDNAR 3   b   0              0.46    13.11     2.03       0.10
*BEST   37  m   1              2.30     5.73    12.16       0.00
 CHEN2  9   m   6              1.23     6.57     0.97       0.00
 CHEN2  12  f   6              0.94    18.11     0.17       0.00
 Subtotal CHEN2                1.02    24.68     1.14
 CLEMEN 3   m   0              0.81     9.05     0.01       0.01
 COCCI  2   b   0              2.98     0.44     2.00       0.05
 DEAN1  13  m   3              0.93    28.67     0.20       0.00
 DEAN1  17  f   3              0.65    33.75     1.30       0.00
 Subtotal DEAN1                0.78    62.42     1.50
 DEJONG 3   b   0              3.21     0.49     2.72       0.03
 DEMARC 6   b   4              0.57    95.64     7.66       0.00
 DICKIN 3   b   0              3.24     0.96     5.47       0.00
*DOLL1  3   m   2              1.88    11.95    12.67       0.00
*DOLL2  3   f   1              2.40     0.92     2.22       0.02
 EKBERG 3   m   0              0.74   289.16     3.19       0.00
 EKBERG 6   f   0              0.94   117.01     1.09       0.00
 Subtotal EKBERG               0.80   406.17     4.28
 FERRI2 3   m   1              1.36     8.88     2.29       0.00
 FERRI2 10  f   1              0.82    27.62     0.02       0.00
 Subtotal FERRI2               0.95    36.50     2.30
 FIDAN  2   m   3              1.94     1.57     1.87       0.02
 FORAST 6   f   1              0.86    17.58     0.00       0.00
 FUKUCH 3   b   0              1.09    43.96     2.57       0.00
*GODTFR 3   b   3              1.70    55.75    40.19       0.00
 GULSVI 1   m   0              2.92     0.96     4.14       0.00
 GULSVI 2   f   0              1.26     9.79     1.65       0.00
 Subtotal GULSVI               1.41    10.75     5.79
*HAMMO2 5   m   1              2.23    38.64    73.43       0.00
*HAMMO2 6   f   1              1.77    32.89    27.93       0.00
 Subtotal HAMMO2               2.02    71.53   101.36
 HARDIE 3   m   1              1.69     6.71     4.78       0.00
 HARDIE 6   f   1              1.01    17.50     0.47       0.00
 Subtotal HARDIE               1.20    24.21     5.24
*HARIKK 3   m   0              0.72     6.14     0.10       0.07
*HARIKK 6   f   0              2.81     0.47     1.82       0.05
 Subtotal HARIKK               0.87     6.61     1.93
*HIGGI4 3   m   1              1.12    11.30     0.86       0.00
  ________________________________________________________________________________________________________________________
                                            International Evidence on Smoking and COPD, Phase 3, Analysis run on 27-SEP-10

                                                   Table 1 - A - 1 - 2

            IESCOPD - Meta-analysis of ever smoking, any product (or cigarettes if all product not available)
                                                         Any COPD
                                                      Most-adjusted


 REF    NRR SEX ADJ             Ys       Ws       Qs       Ps
 HO     3   m   0              0.72    15.02     0.25       0.01
 HO     6   f   0              1.00    13.18     0.30       0.00
 Subtotal HO                   0.85    28.21     0.55
 HOZAWA 3   b   0              1.02   521.32    15.18       0.00
 HUHTI1 6   m   1              2.10     6.02     9.46       0.00
 HUHTI1 14  f   1              1.06    11.56     0.51       0.00
 Subtotal HUHTI1               1.42    17.59     9.97
 HUHTI3 6   m   1              2.05     3.27     4.70       0.00
 ITABAS 1   b   0              0.68     6.50     0.20       0.09
*JACOBS 6   m   6              1.12    22.37     1.71       0.00
 JOHANN 3   b   0              1.42    18.21     6.00       0.00
 KACHEL 3   b   0              1.45     5.86     2.15       0.00
*KAHN2  131 m   1              1.90    28.41    31.46       0.00
 KARAKA 3   b   0              0.79    12.38     0.05       0.01
 KATANC 3   b   0              0.89    14.04     0.03       0.00
 KIM    3   m   1              0.84    17.06     0.00       0.00
 KIM    6   f   1              0.55     7.46     0.65       0.13
 Subtotal KIM                  0.75    24.52     0.65
 KIRAZ  2   f   1              1.93     3.68     4.27       0.00
 KLAYTO 1   b   0              1.75     5.53     4.50       0.00
 KOJIMA 3   m   0              1.10    16.87     1.11       0.00
 KOJIMA 6   f   0              1.03     3.95     0.13       0.04
 Subtotal KOJIMA               1.09    20.82     1.24
*KRZYZA 3   m   1              0.97     4.92     0.07       0.03
*KRZYZA 6   f   1              0.34    13.22     3.37       0.21
 Subtotal KRZYZA               0.51    18.14     3.44
 LAI    1   m   0              1.73    15.75    12.16       0.00
 LAI    2   f   0              0.57     5.18     0.40       0.19
 Subtotal LAI                  1.44    20.93    12.56
*LAM1   1   m   7              1.40     3.02     0.91       0.02
*LAM1   2   f   7              3.28     0.59     3.51       0.01
 Subtotal LAM1                 1.71     3.61     4.42
 LAM2   3   m   0              0.99    34.48     0.67       0.00
*LAM3   3   m   7              0.59     6.25     0.41       0.14
 LEBOWI 6   m   1              1.61     3.49     2.04       0.00
 LEBOWI 18  f   1              0.51    19.66     2.29       0.02
 Subtotal LEBOWI               0.67    23.15     4.34
*LEE    20  m   1              1.55     0.98     0.49       0.12
*LEE    52  f   1              0.55     3.36     0.29       0.31
 Subtotal LEE                  0.78     4.34     0.78
*LINDBE 3   b   3              0.82    13.59     0.01       0.00
 LINDST 9   b   5              0.61    93.46     5.32       0.00
 LUNDB1 9   b   4              1.15    18.75     1.71       0.00
 MADOR  3   m   0              2.90     0.40     1.71       0.06
 MANNI1 1   m   2              0.99    80.88     1.61       0.00
 MANNI1 10  f   2              1.16    97.75     9.31       0.00
 Subtotal MANNI1               1.08   178.63    10.92
 MANNI2 3   b   1              0.97   139.95     2.10       0.00
*MANNI3 3   b   0              0.38    24.97     5.38       0.05
 MARAN1 3   b   0              1.80    38.31    34.70       0.00
*MARAN2 3   b   0              1.70    14.79    10.72       0.00
*MARCUS 3   m   1              0.77    11.06     0.06       0.01
 MATHES 3   b   0              1.14    12.08     1.05       0.00
 MENEZ2 6   b   5              0.43    31.56     5.48       0.02
 MENEZ3 3   b   0              0.10    35.82    19.83       0.53
 MENEZ4 3   b   0              0.50    17.85     2.16       0.03
 MENEZ5 3   b   0              0.49    31.59     3.95       0.01
 MENEZ6 3   b   0              1.00    25.35     0.62       0.00
 MONTNE 3   b   0              0.87    67.48     0.04       0.00
 MUELLE 6   m   1              1.48     1.77     0.72       0.05
 MUELLE 13  f   1              0.47     1.16     0.17       0.61
 Subtotal MUELLE               1.08     2.93     0.88
 NIEPSU 3   b   0              1.37     6.05     1.65       0.00
*NIHLEN 3   b   0              1.16    17.35     1.69       0.00
*NILSSO 9   m   2              1.02    10.13     0.31       0.00
*NILSSO 6   f   2              1.78    25.40    22.05       0.00
 Subtotal NILSSO               1.56    35.53    22.36
 PEREZP 1   f   0              0.26     9.71     3.31       0.41
*PETO   3   m   0              3.24     0.50     2.85       0.02
 RENWIC 3   b   0              1.20     6.53     0.79       0.00
 RICCIO 3   b   0              0.71    78.82     1.60       0.00
  ________________________________________________________________________________________________________________________
                                            International Evidence on Smoking and COPD, Phase 3, Analysis run on 27-SEP-10

                                                   Table 1 - A - 1 - 2

            IESCOPD - Meta-analysis of ever smoking, any product (or cigarettes if all product not available)
                                                         Any COPD
                                                      Most-adjusted


 REF    NRR SEX ADJ             Ys       Ws       Qs       Ps
 SARGEA 9   b   5              0.34   279.95    73.35       0.00
 SHAHAB 3   b   0              0.76   201.37     1.49       0.00
 SHIN   5   b   3              0.63    15.45     0.76       0.01
 SICHLE 1   b   3              0.77    45.74     0.25       0.00
*SILVA  3   b   6              0.46    15.13     2.24       0.07
*SPEIZE 3   m   1              2.43     0.49     1.23       0.09
*SPEIZE 6   f   1              1.44     3.68     1.27       0.01
 Subtotal SPEIZE               1.55     4.17     2.50
 STROM  3   m   0              1.03    11.97     0.42       0.00
*TANG   4   m   2              1.42    11.84     3.81       0.00
*THUN   5   m   1              2.19    43.58    78.77       0.00
*THUN   6   f   1              2.09    54.36    83.22       0.00
 Subtotal THUN                 2.13    97.94   162.00
*TODD   20  m   1              1.93     0.99     1.15       0.06
*TODD   46  f   1             -0.12     1.98     1.84       0.87
 Subtotal TODD                 0.56     2.97     2.99
 TRUPIN 6   b   4              1.24    46.93     7.14       0.00
 TSUSHI 3   b   0              1.69     5.91     4.18       0.00
*TVERDA 3   m   2              1.06     6.19     0.27       0.01
 VESTBO 3   b   0              0.65   184.87     7.62       0.00
 VIEGI2 6   m   1              0.33    14.54     3.92       0.21
 VIEGI2 12  f   1              0.18    23.37    10.36       0.38
 Subtotal VIEGI2               0.24    37.91    14.28
 VONHER 3   m   0              1.73    52.38    40.29       0.00
 VONHER 6   f   0              1.33    60.30    14.14       0.00
 Subtotal VONHER               1.52   112.68    54.43
 WEISS  3   m   0              1.23     1.79     0.26       0.10
 WILSO1 1   b   0              1.37    18.98     5.12       0.00
 XIAO   3   b   0              1.57     9.91     5.11       0.00
 XU     2   m   13             0.23   146.03    55.63       0.01
 XU     4   f   13             0.96    32.49     0.40       0.00
 Subtotal XU                   0.36   178.52    56.03
 YAMAGU 3   b   6              0.68    87.99     2.55       0.00
*YUAN   5   m   2              0.34    12.27     3.21       0.24
 ZIELI1 2   b   1              0.72   225.41     3.56       0.00
 ZIELI2 3   m   0              0.89   291.62     0.62       0.00
 ZIELI2 6   f   0              0.34   523.09   136.49       0.00
 Subtotal ZIELI2               0.54   814.71   137.11
 ZIETKO 2   m   0              4.35     0.46     5.57       0.00
 ZIETKO 4   f   0              3.79     0.43     3.73       0.01
 Subtotal ZIETKO               4.08     0.89     9.30

                       N      129
                      NS       98


                      Wt  5116.47
                 Het Chi  1038.04
                 Het  df      128
                 Het  P       ***
               Fixed  RR     2.34
                     RRl     2.27
                     RRu     2.40
                      P       +++
              Random  RR     2.89
                     RRl     2.63
                     RRu     3.17
                      P       +++
               Asymm  P       ***


  ________________________________________________________________________________________________________________________
                                            International Evidence on Smoking and COPD, Phase 3, Analysis run on 27-SEP-10

                                                   Table 1 - A - 1 - 3

            IESCOPD - Meta-analysis of ever smoking, any product (or cigarettes if all product not available)
                                                         Any COPD
                                                      Most-adjusted


                       N      129
                      NS       98


                      Wt  5116.47
                 Het Chi  1038.04
                 Het  df      128
                 Het  P       ***
               Fixed  RR     2.34
                     RRl     2.27
                     RRu     2.40
                      P       +++
              Random  RR     2.89
                     RRl     2.63
                     RRu     3.17
                      P       +++
               Asymm  P       ***

                                   Sex
                             both      male    female     Total


                       N       45        49        35       129
                      NS       45        49        35       129


                      Wt  2609.95   1305.87   1200.64   5116.47
                 Het Chi   299.93    363.22    338.24   1038.04
                 Het  df       44        48        34       128
                 Het  P       ***       ***       ***       ***
               Fixed  RR     2.26      2.69      2.16      2.34
                     RRl     2.17      2.55      2.04      2.27
                     RRu     2.35      2.84      2.28      2.40
                      P       +++       +++       +++       +++
              Random  RR     2.51      3.60      2.73      2.89
                     RRl     2.23      2.98      2.17      2.63
                     RRu     2.82      4.34      3.43      3.17
                      P       +++       +++       +++       +++
             Between Chi                                  36.65
             Between  df                                      2
             Between  P                                     ***
             Btwn(F)  P                                    N.S.

                                        Continent
                            NAmer    Europe      Asia  oth/mult     Total


                       N       35        59        23        12       129
                      NS       25        45        17        11        98


                      Wt  1298.45   2960.03    552.45    305.55   5116.47
                 Het Chi   246.19    427.40    139.86     43.67   1038.04
                 Het  df       34        58        22        11       128
                 Het  P       ***       ***       ***       ***       ***
               Fixed  RR     3.21      2.09      2.26      1.91      2.34
                     RRl     3.04      2.01      2.08      1.71      2.27
                     RRu     3.39      2.17      2.45      2.14      2.40
                      P       +++       +++       +++       +++       +++
              Random  RR     3.48      2.79      2.73      2.13      2.89
                     RRl     2.88      2.46      2.15      1.66      2.63
                     RRu     4.20      3.16      3.46      2.73      3.17
                      P       +++       +++       +++       +++       +++
             Between Chi                                           180.92
             Between  df                                                3
             Between  P                                               ***
             Btwn(F)  P                                               ***


  ________________________________________________________________________________________________________________________
                                            International Evidence on Smoking and COPD, Phase 3, Analysis run on 27-SEP-10

                                                   Table 1 - A - 1 - 3

            IESCOPD - Meta-analysis of ever smoking, any product (or cigarettes if all product not available)
                                                         Any COPD
                                                      Most-adjusted
                        National cigarette tobacco type (excluding mixed/unkown)
                          blended  virginia     Total


                       N       96        21       117
                      NS       73        16        89


                      Wt  4013.98    652.87   4666.86
                 Het Chi   801.55    110.42    942.87
                 Het  df       95        20       116
                 Het  P       ***       ***       ***
               Fixed  RR     2.47      1.95      2.39
                     RRl     2.40      1.81      2.32
                     RRu     2.55      2.11      2.46
                      P       +++       +++       +++
              Random  RR     2.94      3.02      2.95
                     RRl     2.64      2.33      2.67
                     RRu     3.27      3.90      3.26
                      P       +++       +++       +++
             Between Chi                        30.90
             Between  df                            1
             Between  P                           ***
             Btwn(F)  P                           (*)

                                        Start year of study
                            <1970   1970-79   1980-89   1990-99     2000+   unknown     Total


                       N       35        17        12        23        27        15       129
                      NS       24        11         9        20        21        13        98


                      Wt   440.17    931.66    988.76    937.37   1668.70    149.81   5116.47
                 Het Chi   144.66     92.55    161.81    141.08    194.15     42.36   1038.04
                 Het  df       34        16        11        22        26        14       128
                 Het  P       ***       ***       ***       ***       ***       ***       ***
               Fixed  RR     3.93      2.41      2.91      2.06      1.88      2.40      2.34
                     RRl     3.58      2.26      2.73      1.93      1.79      2.05      2.27
                     RRu     4.32      2.57      3.10      2.20      1.97      2.82      2.40
                      P       +++       +++       +++       +++       +++       +++       +++
              Random  RR     3.63      2.73      2.73      2.69      2.31      4.26      2.89
                     RRl     2.91      2.22      2.04      2.22      1.97      2.74      2.63
                     RRu     4.53      3.34      3.64      3.26      2.72      6.60      3.17
                      P       +++       +++       +++       +++       +++       +++       +++
             Between Chi                                                               261.43
             Between  df                                                                    5
             Between  P                                                                   ***
             Btwn(F)  P                                                                   ***

                                Publication year
                            <1980   1980-89   1990-99     2000+     Total


                       N       25        10        19        75       129
                      NS       15         8        16        59        98


                      Wt   284.58    143.13    318.89   4369.86   5116.47
                 Het Chi   118.14     13.21    127.81    620.30   1038.04
                 Het  df       24         9        18        74       128
                 Het  P       ***      N.S.       ***       ***       ***
               Fixed  RR     3.82      2.13      3.75      2.19      2.34
                     RRl     3.40      1.80      3.36      2.13      2.27
                     RRu     4.29      2.50      4.18      2.26      2.40
                      P       +++       +++       +++       +++       +++
              Random  RR     3.79      2.34      3.31      2.62      2.89
                     RRl     2.82      1.79      2.39      2.36      2.63
                     RRu     5.10      3.05      4.58      2.90      3.17
                      P       +++       +++       +++       +++       +++
             Between Chi                                           158.58
             Between  df                                                3
             Between  P                                               ***
             Btwn(F)  P                                               ***
  ________________________________________________________________________________________________________________________
                                            International Evidence on Smoking and COPD, Phase 3, Analysis run on 27-SEP-10

                                                   Table 1 - A - 1 - 3

            IESCOPD - Meta-analysis of ever smoking, any product (or cigarettes if all product not available)
                                                         Any COPD
                                                      Most-adjusted
                               Study type
                               CC        Pr        CS     Total


                       N       16        37        76       129
                      NS       12        28        58        98


                      Wt   124.03    524.28   4468.16   5116.47
                 Het Chi    31.76    209.41    544.26   1038.04
                 Het  df       15        36        75       128
                 Het  P        **       ***       ***       ***
               Fixed  RR     2.51      4.49      2.16      2.34
                     RRl     2.10      4.12      2.10      2.27
                     RRu     2.99      4.89      2.22      2.40
                      P       +++       +++       +++       +++
              Random  RR     2.95      3.71      2.54      2.89
                     RRl     2.14      2.94      2.32      2.63
                     RRu     4.06      4.69      2.80      3.17
                      P       +++       +++       +++       +++
             Between Chi                                 252.60
             Between  df                                      2
             Between  P                                     ***
             Btwn(F)  P                                     ***

                                    Lowest age in RR
                        <25/unlim     25-39       40+   unknown     Total


                       N       46        43        40                 129
                      NS       34        29        35                  98


                      Wt  2417.10   1213.54   1485.83             5116.47
                 Het Chi   308.46    447.02    220.32             1038.04
                 Het  df       45        42        39                 128
                 Het  P       ***       ***       ***                 ***
               Fixed  RR     2.14      2.82      2.32                2.34
                     RRl     2.05      2.66      2.20                2.27
                     RRu     2.22      2.98      2.44                2.40
                      P       +++       +++       +++                 +++
              Random  RR     2.83      3.31      2.53                2.89
                     RRl     2.47      2.67      2.19                2.63
                     RRu     3.25      4.09      2.94                3.17
                      P       +++       +++       +++                 +++
             Between Chi                                            62.24
             Between  df                                                2
             Between  P                                               ***
             Btwn(F)  P                                                 *

                                         Highest age in RR
                              <65     65-74     75-84 85+/unlim   unknown     Total


                       N        8        22        21        78                 129
                      NS        6        17        16        59                  98


                      Wt   735.76    665.36    301.01   3414.33             5116.47
                 Het Chi    25.85     93.34     53.09    803.93             1038.04
                 Het  df        7        21        20        77                 128
                 Het  P       ***       ***       ***       ***                 ***
               Fixed  RR     2.59      1.87      3.05      2.33                2.34
                     RRl     2.41      1.74      2.73      2.25                2.27
                     RRu     2.79      2.02      3.42      2.41                2.40
                      P       +++       +++       +++       +++                 +++
              Random  RR     2.61      2.44      2.98      3.02                2.89
                     RRl     2.09      1.95      2.42      2.65                2.63
                     RRu     3.25      3.04      3.67      3.43                3.17
                      P       +++       +++       +++       +++                 +++
             Between Chi                                                      61.84
             Between  df                                                          3
             Between  P                                                         ***
             Btwn(F)  P                                                         (*)
  ________________________________________________________________________________________________________________________
                                            International Evidence on Smoking and COPD, Phase 3, Analysis run on 27-SEP-10

                                                   Table 1 - A - 1 - 3

            IESCOPD - Meta-analysis of ever smoking, any product (or cigarettes if all product not available)
                                                         Any COPD
                                                      Most-adjusted
                           Study weakness
                              Yes        No     Total


                       N       16       113       129
                      NS       13        85        98


                      Wt   195.86   4920.61   5116.47
                 Het Chi    25.90   1012.12   1038.04
                 Het  df       15       112       128
                 Het  P         *       ***       ***
               Fixed  RR     2.31      2.34      2.34
                     RRl     2.01      2.27      2.27
                     RRu     2.66      2.40      2.40
                      P       +++       +++       +++
              Random  RR     2.64      2.89      2.89
                     RRl     2.07      2.62      2.63
                     RRu     3.38      3.19      3.17
                      P       +++       +++       +++
             Between Chi                         0.02
             Between  df                            1
             Between  P                          N.S.
             Btwn(F)  P                          N.S.

                          COPD subtype
                             mort        LF     other     Total


                       N       29        60        40       129
                      NS       21        48        29        98


                      Wt   436.27   3469.45   1210.74   5116.47
                 Het Chi   187.79    305.43    335.94   1038.04
                 Het  df       28        59        39       128
                 Het  P       ***       ***       ***       ***
               Fixed  RR     4.50      2.16      2.31      2.34
                     RRl     4.09      2.09      2.19      2.27
                     RRu     4.94      2.23      2.45      2.40
                      P       +++       +++       +++       +++
              Random  RR     3.95      2.35      3.10      2.89
                     RRl     3.00      2.13      2.56      2.63
                     RRu     5.21      2.59      3.76      3.17
                      P       +++       +++       +++       +++
             Between Chi                                 208.88
             Between  df                                      2
             Between  P                                     ***
             Btwn(F)  P                                     ***

                               Asthma analysis type (COPD)
                        inc-irres  excl-all defn-incl     other     Total


                       N       76        21        19        13       129
                      NS       58        16        12        12        98


                      Wt  3211.22   1223.61    490.60    191.03   5116.47
                 Het Chi   634.06    108.87     72.50     33.06   1038.04
                 Het  df       75        20        18        12       128
                 Het  P       ***       ***       ***       ***       ***
               Fixed  RR     2.60      1.83      1.76      3.68      2.34
                     RRl     2.52      1.73      1.61      3.20      2.27
                     RRu     2.70      1.94      1.92      4.24      2.40
                      P       +++       +++       +++       +++       +++
              Random  RR     2.95      2.35      2.55      3.62      2.89
                     RRl     2.62      1.92      2.00      2.80      2.63
                     RRu     3.32      2.87      3.26      4.69      3.17
                      P       +++       +++       +++       +++       +++
             Between Chi                                           189.55
             Between  df                                                3
             Between  P                                               ***
             Btwn(F)  P                                               ***
  ________________________________________________________________________________________________________________________
                                            International Evidence on Smoking and COPD, Phase 3, Analysis run on 27-SEP-10

                                                   Table 1 - A - 1 - 3

            IESCOPD - Meta-analysis of ever smoking, any product (or cigarettes if all product not available)
                                                         Any COPD
                                                      Most-adjusted
                        Bronchodilator/reversibility (LF only)
                          no/unkn  yes/revs     Total


                       N       48        12        60
                      NS       37        11        48


                      Wt  3263.58    205.87   3469.45
                 Het Chi   243.05     62.38    305.43
                 Het  df       47        11        59
                 Het  P       ***       ***       ***
               Fixed  RR     2.16      2.15      2.16
                     RRl     2.09      1.87      2.09
                     RRu     2.23      2.46      2.23
                      P       +++       +++       +++
              Random  RR     2.32      2.79      2.35
                     RRl     2.09      1.93      2.13
                     RRu     2.57      4.03      2.59
                      P       +++       +++       +++
             Between Chi                         0.00
             Between  df                            1
             Between  P                          N.S.
             Btwn(F)  P                          N.S.

                                  Number of COPD cases
                             1-50    51-100   101-200      201+     Total


                       N       23        29        30        47       129
                      NS       17        25        22        34        98


                      Wt    55.18    232.20    438.43   4390.66   5116.47
                 Het Chi    29.41     57.56    100.85    832.27   1038.04
                 Het  df       22        28        29        46       128
                 Het  P      N.S.       ***       ***       ***       ***
               Fixed  RR     4.06      2.42      2.39      2.31      2.34
                     RRl     3.12      2.12      2.18      2.24      2.27
                     RRu     5.29      2.75      2.63      2.38      2.40
                      P       +++       +++       +++       +++       +++
              Random  RR     4.45      2.55      2.69      2.91      2.89
                     RRl     3.19      2.09      2.24      2.54      2.63
                     RRu     6.21      3.12      3.24      3.32      3.17
                      P       +++       +++       +++       +++       +++
             Between Chi                                            17.95
             Between  df                                                3
             Between  P                                               ***
             Btwn(F)  P                                              N.S.

                            Analysis type
                         prevlnce     onset     Total


                       N       93        36       129
                      NS       71        27        98


                      Wt  4601.24    515.22   5116.47
                 Het Chi   578.77    205.04   1038.04
                 Het  df       92        35       128
                 Het  P       ***       ***       ***
               Fixed  RR     2.17      4.55      2.34
                     RRl     2.11      4.17      2.27
                     RRu     2.23      4.96      2.40
                      P       +++       +++       +++
              Random  RR     2.57      3.77      2.89
                     RRl     2.35      2.98      2.63
                     RRu     2.81      4.78      3.17
                      P       +++       +++       +++
             Between Chi                       254.23
             Between  df                            1
             Between  P                           ***
             Btwn(F)  P                           ***
  ________________________________________________________________________________________________________________________
                                            International Evidence on Smoking and COPD, Phase 3, Analysis run on 27-SEP-10

                                                   Table 1 - A - 1 - 3

            IESCOPD - Meta-analysis of ever smoking, any product (or cigarettes if all product not available)
                                                         Any COPD
                                                      Most-adjusted
                             Smoking product
                              any      cigs  cigsonly     Total


                       N       48        73         8       129
                      NS       36        59         6       101


                      Wt  1490.61   3390.58    235.28   5116.47
                 Het Chi   219.13    454.37     63.84   1038.04
                 Het  df       47        72         7       128
                 Het  P       ***       ***       ***       ***
               Fixed  RR     2.60      2.08      6.38      2.34
                     RRl     2.47      2.01      5.61      2.27
                     RRu     2.74      2.15      7.24      2.40
                      P       +++       +++       +++       +++
              Random  RR     2.99      2.48      6.42      2.89
                     RRl     2.61      2.23      4.22      2.63
                     RRu     3.44      2.76      9.78      3.17
                      P       +++       +++       +++       +++
             Between Chi                                 300.71
             Between  df                                      2
             Between  P                                     ***
             Btwn(F)  P                                     ***

                                     Unexposed group
                          nev any   nev cig  nev+ any  nev+ cig     Total


                       N       57        69                   3       129
                      NS       40        55                   3        98


                      Wt  1823.63   3234.19               58.64   5116.47
                 Het Chi   446.98    370.08                0.11   1038.04
                 Het  df       56        68                   2       128
                 Het  P       ***       ***                N.S.       ***
               Fixed  RR     2.95      2.01                5.87      2.34
                     RRl     2.82      1.95                4.54      2.27
                     RRu     3.09      2.08                7.58      2.40
                      P       +++       +++                 +++       +++
              Random  RR     3.44      2.35                5.87      2.89
                     RRl     2.95      2.12                4.54      2.63
                     RRu     4.01      2.60                7.58      3.17
                      P       +++       +++                 +++       +++
             Between Chi                                           220.87
             Between  df                                                2
             Between  P                                               ***
             Btwn(F)  P                                               ***

                        Unexposed group (combining nev+ with main levels)
                          nev any   nev cig     Total


                       N       57        72       129
                      NS       40        58        98


                      Wt  1823.63   3292.83   5116.47
                 Het Chi   446.98    436.15   1038.04
                 Het  df       56        71       128
                 Het  P       ***       ***       ***
               Fixed  RR     2.95      2.05      2.34
                     RRl     2.82      1.98      2.27
                     RRu     3.09      2.12      2.40
                      P       +++       +++       +++
              Random  RR     3.44      2.47      2.89
                     RRl     2.95      2.22      2.63
                     RRu     4.01      2.74      3.17
                      P       +++       +++       +++
             Between Chi                       154.91
             Between  df                            1
             Between  P                           ***
             Btwn(F)  P                           ***
  ________________________________________________________________________________________________________________________
                                            International Evidence on Smoking and COPD, Phase 3, Analysis run on 27-SEP-10

                                                   Table 1 - A - 1 - 3

            IESCOPD - Meta-analysis of ever smoking, any product (or cigarettes if all product not available)
                                                         Any COPD
                                                      Most-adjusted
                        Smoking results reported in study (COPD)
                             Ever   Current      Both     Total


                       N       18                 111       129
                      NS       13                  85        98


                      Wt   535.53             4580.93   5116.47
                 Het Chi    95.65              929.63   1038.04
                 Het  df       17                 110       128
                 Het  P       ***                 ***       ***
               Fixed  RR     2.02                2.38      2.34
                     RRl     1.85                2.31      2.27
                     RRu     2.20                2.45      2.40
                      P       +++                 +++       +++
              Random  RR     3.04                2.87      2.89
                     RRl     2.30                2.60      2.63
                     RRu     4.00                3.17      3.17
                      P       +++                 +++       +++
             Between Chi                                  12.76
             Between  df                                      1
             Between  P                                     ***
             Btwn(F)  P                                    N.S.

                        Number of adjustment variables
                                0         1        2+     Total


                       N       58        38        33       129
                      NS       48        24        26        98


                      Wt  2935.22    820.87   1360.37   5116.47
                 Het Chi   387.68    308.81    254.82   1038.04
                 Het  df       57        37        32       128
                 Het  P       ***       ***       ***       ***
               Fixed  RR     2.27      3.11      2.09      2.34
                     RRl     2.19      2.90      1.98      2.27
                     RRu     2.36      3.33      2.20      2.40
                      P       +++       +++       +++       +++
              Random  RR     2.78      3.36      2.59      2.89
                     RRl     2.46      2.66      2.19      2.63
                     RRu     3.14      4.25      3.08      3.17
                      P       +++       +++       +++       +++
             Between Chi                                  86.73
             Between  df                                      2
             Between  P                                     ***
             Btwn(F)  P                                      **

                        RR adjusted for sex (combined sex RR only)
                              Yes        No     Total


                       N       12        33        45
                      NS       12        33        45


                      Wt   799.95   1810.00   2609.95
                 Het Chi   113.67    153.22    299.93
                 Het  df       11        32        44
                 Het  P       ***       ***       ***
               Fixed  RR     1.91      2.43      2.26
                     RRl     1.78      2.32      2.17
                     RRu     2.04      2.55      2.35
                      P       +++       +++       +++
              Random  RR     2.19      2.64      2.51
                     RRl     1.71      2.33      2.23
                     RRu     2.79      3.00      2.82
                      P       +++       +++       +++
             Between Chi                        33.04
             Between  df                            1
             Between  P                           ***
             Btwn(F)  P                             *
  ________________________________________________________________________________________________________________________
                                            International Evidence on Smoking and COPD, Phase 3, Analysis run on 27-SEP-10

                                                   Table 1 - A - 1 - 3

            IESCOPD - Meta-analysis of ever smoking, any product (or cigarettes if all product not available)
                                                         Any COPD
                                                      Most-adjusted
                        RR adjusted for age
                              Yes        No     Total


                       N       69        60       129
                      NS       48        50        98


                      Wt  2081.93   3034.54   5116.47
                 Het Chi   630.98    398.28   1038.04
                 Het  df       68        59       128
                 Het  P       ***       ***       ***
               Fixed  RR     2.46      2.26      2.34
                     RRl     2.35      2.18      2.27
                     RRu     2.56      2.34      2.40
                      P       +++       +++       +++
              Random  RR     2.97      2.77      2.89
                     RRl     2.56      2.46      2.63
                     RRu     3.45      3.12      3.17
                      P       +++       +++       +++
             Between Chi                         8.78
             Between  df                            1
             Between  P                            **
             Btwn(F)  P                          N.S.

                        RR adjusted for factor other than sex, age


                       N       34        95       129
                      NS       27        71        98


                      Wt  1364.05   3752.41   5116.47
                 Het Chi   260.02    755.61   1038.04
                 Het  df       33        94       128
                 Het  P       ***       ***       ***
               Fixed  RR     2.09      2.43      2.34
                     RRl     1.98      2.35      2.27
                     RRu     2.21      2.51      2.40
                      P       +++       +++       +++
              Random  RR     2.64      3.00      2.89
                     RRl     2.23      2.68      2.63
                     RRu     3.13      3.36      3.17
                      P       +++       +++       +++
             Between Chi                        22.42
             Between  df                            1
             Between  P                           ***
             Btwn(F)  P                           (*)

                        Derivation of RR/CI
                         Orig/2x2     Other     Total


                       N       18       111       129
                      NS       15        86       101


                      Wt   361.98   4754.49   5116.47
                 Het Chi    33.48   1004.54   1038.04
                 Het  df       17       110       128
                 Het  P        **       ***       ***
               Fixed  RR     2.35      2.33      2.34
                     RRl     2.12      2.27      2.27
                     RRu     2.61      2.40      2.40
                      P       +++       +++       +++
              Random  RR     2.72      2.90      2.89
                     RRl     2.21      2.61      2.63
                     RRu     3.33      3.21      3.17
                      P       +++       +++       +++
             Between Chi                         0.02
             Between  df                            1
             Between  P                          N.S.
             Btwn(F)  P                          N.S.

  ________________________________________________________________________________________________________________________
                                            International Evidence on Smoking and COPD, Phase 3, Analysis run on 27-SEP-10

                                                   Table 1 - A - 1 - 4

            IESCOPD - Meta-analysis of ever smoking, any product (or cigarettes if all product not available)
                                                         Any COPD
                                                      Least-adjusted


     REF|NRR|X|SEX|AGEL|AGEH|     REGION|BEGYR|PUBYR|STTYP|ONSET|      DISEAS|ADJ|SMOKSTA|   PRODUCT|    UNEXP|

  ALESSA   3     b   45   81     Eu:West  1992  1994    CC  Prev COPD:LF/symp   0    Ever       Cigs  Nev cigs
   AMIGO   1 x   m    0   99 Am:Sth/Cent  2001  2006    CC  Prev   COPD/CB/EM   1    Ever       Cigs  Nev cigs
   AMIGO   2 x   f    0   99 Am:Sth/Cent  2001  2006    CC  Prev   COPD/CB/EM   1    Ever       Cigs  Nev cigs
  ANDER1   3     m   25   74   Am:Canada  1963  1965    CS  Prev     COPD:oth   1    Ever        Any   Nev any
  ANDER1   6     f   25   74   Am:Canada  1963  1965    CS  Prev     COPD:oth   1    Ever        Any   Nev any
  ANDER3   3     m    0   99     Eu:East     *  2001    CC  Prev      COPD:LF   0    Ever        Any   Nev any
  ANDER3   6     f    0   99     Eu:East     *  2001    CC  Prev      COPD:LF   0    Ever        Any   Nev any
  BEDNAR   3     b   30   99     Eu:East  2000  2005    CS  Prev      COPD:LF   0    Ever        Any   Nev any
    BEST  37     m   30   97   Am:Canada  1955  1967    Pr   Inc    COPD:mort   1    Ever  Cigs only   Nev any
   CHEN2   3 x   m   35   64   Am:Canada  1994  2000    CS  Prev        CB/EM   0    Ever       Cigs  Nev cigs
   CHEN2   6 x   f   35   64   Am:Canada  1994  2000    CS  Prev        CB/EM   0    Ever       Cigs  Nev cigs
  CLEMEN   3     m   20   60     Eu:West  1960  1982    Pr  Prev      COPD:LF   0    Ever        Any   Nev any
   COCCI   2     b   38   78     Eu:West     *  2002    CC  Prev COPD:LF/symp   0    Ever       Cigs  Nev cigs
   DEAN1   3 x   m   35   99       Eu:UK  1969  1977    CC  Prev    COPD:mort   0    Ever        Any   Nev any
   DEAN1   9 x   f   35   99       Eu:UK  1969  1977    CC  Prev    COPD:mort   0    Ever MCigs only   Nev any
  DEJONG   3     b    0   99      Am:USA     *  2004    CS  Prev      COPD:LF   0    Ever       Cigs  Nev cigs
  DEMARC   3 x   b   20   44       Multi  1991  2004    CS  Prev      COPD:LF   0    Ever        Any   Nev any
  DICKIN   3     b   60   74       Eu:UK     *  1999    CS  Prev      COPD:LF   0    Ever        Any   Nev any
   DOLL1   3     m   20   99       Eu:UK  1951  1994    Pr   Inc    COPD:mort   2    Ever        Any   Nev any
   DOLL2   3     f   20   99       Eu:UK  1951  1980    Pr   Inc    COPD:mort   1    Ever  Cigs only   Nev any
  EKBERG   3     m   15   99    Eu:Scand  1974  2005    CS  Prev      COPD:LF   0    Ever        Any   Nev any
  EKBERG   6     f   15   99    Eu:Scand  1974  2005    CS  Prev      COPD:LF   0    Ever        Any   Nev any
  FERRI2   3     m   25   80      Am:USA  1967  1971    CS  Prev     COPD:oth   1    Ever        Any   Nev any
  FERRI2  10     f   25   80      Am:USA  1967  1971    CS  Prev     COPD:oth   1    Ever        Any   Nev any
   FIDAN   1 x   m   15   99 Eu:SE/Balkn  2000  2004    CS  Prev     COPD:oth   0    Ever       Cigs  Nev cigs
  FORAST   3 x   f   55   99      Am:USA  1993  1998    CS  Prev        CB/EM   0    Ever       Cigs  Nev cigs
  FUKUCH   3     b   40   99   Asia:FarE  2000  2004    CS  Prev      COPD:LF   0    Ever       Cigs  Nev cigs
  GODTFR   3     b   20   99    Eu:Scand  1964  2002    Pr   Inc     COPD:ICD   3    Ever        Any   Nev any
  GULSVI   1     m   15   70    Eu:Scand  1972  1979    CS  Prev     COPD:oth   0    Ever        Any   Nev any
  GULSVI   2     f   15   70    Eu:Scand  1972  1979    CS  Prev     COPD:oth   0    Ever        Any   Nev any
  HAMMO2   5     m   35   99      Am:USA  1959  1966    Pr   Inc    COPD:mort   1    Ever  Cigs only   Nev any
  HAMMO2   6     f   35   99      Am:USA  1959  1966    Pr   Inc    COPD:mort   1    Ever  Cigs only   Nev any
  HARDIE   3     m   70   99    Eu:Scand  1998  2005    CS  Prev    CB/EM/Ast   1    Ever       Cigs  Nev cigs
  HARDIE   6     f   70   99    Eu:Scand  1998  2005    CS  Prev    CB/EM/Ast   1    Ever       Cigs  Nev cigs
  HARIKK   3     m   40   99      Am:USA  1962  2002    Pr   Inc     COPD:oth   0    Ever        Any   Nev any
  HARIKK   6     f   40   99      Am:USA  1962  2002    Pr   Inc     COPD:oth   0    Ever        Any   Nev any
  HIGGI4   3     m   35   99      Am:USA  1962  1989    Pr   Inc    COPD:mort   1    Ever       Cigs  Nev cigs
      HO   3     m   70   99   Asia:FarE  1991  1999    CS  Prev   COPD:undef   0    Ever       Cigs  Nev cigs
      HO   6     f   70   99   Asia:FarE  1991  1999    CS  Prev   COPD:undef   0    Ever       Cigs  Nev cigs
  HOZAWA   3     b   45   64      Am:USA  1987  2006    CS  Prev      COPD:LF   0    Ever       Cigs  Nev cigs
  HUHTI1   3 x   m   40   64    Eu:Scand  1961  1965    CS  Prev    CB/EM/Ast   0    Ever        Any   Nev any
  HUHTI1  11 x   f   40   64    Eu:Scand  1961  1965    CS  Prev    CB/EM/Ast   0    Ever        Any   Nev any
  HUHTI3   3 x   m   25   69    Eu:Scand  1968  1978    CS  Prev      COPD:LF   0    Ever        Any   Nev any
  ITABAS   1     b   60   99   Asia:FarE     *  1990    CC  Prev      COPD:LF   0    Ever       Cigs  Nev cigs
  JACOBS   3 x   m   40   84       Multi  1957  1999    Pr   Inc    COPD:mort   2    Ever       Cigs  Nev cigs
  JOHANN   3     b   26   82    Eu:Scand  1996  2005    CS  Prev      COPD:LF   0    Ever       Cigs  Nev cigs
  KACHEL   3     b   20   79     Eu:East     *  2003    CS  Prev      COPD:LF   0    Ever       Cigs  Nev cigs
   KAHN2 131     m   31   84      Am:USA  1954  1966    Pr   Inc    COPD:mort   1    Ever        Any   Nev any
  KARAKA   3     b   15   99 Eu:SE/Balkn     *  2003   nCC  Prev   COPD/CB/EM   0    Ever        Any   Nev any
  KATANC   3     b   70   79      Am:USA  1997  2005    CS  Prev      COPD:LF   0    Ever       Cigs  Nev cigs
     KIM   3     m   45   99   Asia:FarE  2001  2005    CS  Prev      COPD:LF   1    Ever       Cigs  Nev cigs
     KIM   6     f   45   99   Asia:FarE  2001  2005    CS  Prev      COPD:LF   1    Ever       Cigs  Nev cigs
   KIRAZ   1 x   f   25   99 Eu:SE/Balkn  1999  2003    CS  Prev COPD:LF/symp   0    Ever       Cigs  Nev cigs
  KLAYTO   1     b   40   99      Am:USA     *  1975    CS  Prev      COPD:LF   0    Ever       Cigs  N/L cigs
  KOJIMA   3     m   25   74   Asia:FarE  2001  2005    CS  Prev      COPD:LF   0    Ever       Cigs  Nev cigs
  KOJIMA   6     f   25   74   Asia:FarE  2001  2005    CS  Prev      COPD:LF   0    Ever       Cigs  Nev cigs
  KRZYZA   3     m   19   83     Eu:East  1968  1986    Pr   Inc      COPD:LF   1    Ever       Cigs  Nev cigs
  KRZYZA   6     f   19   83     Eu:East  1968  1986    Pr   Inc      COPD:LF   1    Ever       Cigs  Nev cigs
     LAI   1     m   18   80   Asia:FarE  2001  2006    CS  Prev      COPD:LF   0    Ever        Any   Nev any
     LAI   2     f   18   80   Asia:FarE  2001  2006    CS  Prev      COPD:LF   0    Ever        Any   Nev any
    LAM1   1     m   35   99   Asia:FarE  1976  1997    Pr   Inc    COPD:mort   7    Ever       Cigs  Nev cigs
    LAM1   2     f   35   99   Asia:FarE  1976  1997    Pr   Inc    COPD:mort   7    Ever       Cigs  Nev cigs
    LAM2   3     m   60   99   Asia:FarE  1987  2002    CS  Prev   COPD:undef   0    Ever       Cigs  Nev cigs
    LAM3   3     m   60   99   Asia:FarE  1987  2006    Pr   Inc    COPD:mort   7    Ever       Cigs  Nev cigs
  LEBOWI   3 x   m   15   96      Am:USA  1972  1977    CS  Prev      COPD:LF   0    Ever       Cigs  Nev cigs
  LEBOWI  15 x   f   15   96      Am:USA  1972  1977    CS  Prev      COPD:LF   0    Ever       Cigs  Nev cigs
     LEE   6 x   m   35   82       Eu:UK  1964  1979    Pr   Inc    COPD:mort   0    Ever        Any   Nev any
     LEE  41 x   f   35   82       Eu:UK  1964  1979    Pr   Inc    COPD:mort   0    Ever        Any   Nev any
  LINDBE   3     b   46   84    Eu:Scand  1996  2006    Pr   Inc      COPD:LF   3    Ever        Any   Nev any
  LINDST   5 x   m   20   69    Eu:Scand     *  2001    CS  Prev        CB/EM   0    Ever       Cigs  Nev cigs
  LINDST   6 x   f   20   69    Eu:Scand     *  2001    CS  Prev        CB/EM   0    Ever       Cigs  Nev cigs
  LUNDB1   3 x   m   46   77    Eu:Scand  1996  2003    CS  Prev      COPD:LF   0    Ever       Cigs  Nev cigs
  ________________________________________________________________________________________________________________________
                                            International Evidence on Smoking and COPD, Phase 3, Analysis run on 27-SEP-10

                                                   Table 1 - A - 1 - 4

            IESCOPD - Meta-analysis of ever smoking, any product (or cigarettes if all product not available)
                                                         Any COPD
                                                      Least-adjusted


     REF|NRR|X|SEX|AGEL|AGEH|     REGION|BEGYR|PUBYR|STTYP|ONSET|      DISEAS|ADJ|SMOKSTA|   PRODUCT|    UNEXP|

  LUNDB1   6 x   f   46   77    Eu:Scand  1996  2003    CS  Prev      COPD:LF   0    Ever       Cigs  Nev cigs
   MADOR   3     m   15   99      Am:USA     *  2003    CC  Prev      COPD:LF   0    Ever       Cigs  Nev cigs
  MANNI1   1     m   17   99      Am:USA  1988  2000    CS  Prev      COPD:LF   2    Ever        Any   Nev any
  MANNI1  10     f   17   99      Am:USA  1988  2000    CS  Prev      COPD:LF   2    Ever       Cigs   Nev any
  MANNI2   3     b   25   74      Am:USA  1971  2003    CS  Prev      COPD:LF   1    Ever        Any   Nev any
  MANNI3   3     b   25   96      Am:USA  1971  2003    Pr   Inc    COPD:mort   0    Ever        Any   Nev any
  MARAN1   3     b   60   99 Asia:SE/Pac  1998  2002    CS  Prev     COPD:oth   0    Ever       Cigs  N/L cigs
  MARAN2   3     b   60   99 Asia:SE/Pac  1998  2002    Pr   Inc     COPD:oth   0    Ever       Cigs  N/L cigs
  MARCUS   3     m   45   85      Am:USA  1965  1989    Pr   Inc    COPD:mort   1    Ever        Any   Nev any
  MATHES   3     b   45   70      Aus/NZ     *  2006    CC  Prev      COPD:LF   0    Ever       Cigs  Nev cigs
  MENEZ2   3 x   b   40   99 Am:Sth/Cent  2003  2005    CS  Prev      COPD:LF   0    Ever       Cigs  Nev cigs
  MENEZ3   3     b   40   99 Am:Sth/Cent  2003  2005    CS  Prev      COPD:LF   0    Ever       Cigs  Nev cigs
  MENEZ4   3     b   40   99 Am:Sth/Cent  2003  2005    CS  Prev      COPD:LF   0    Ever       Cigs  Nev cigs
  MENEZ5   3     b   40   99 Am:Sth/Cent  2003  2005    CS  Prev      COPD:LF   0    Ever       Cigs  Nev cigs
  MENEZ6   3     b   40   99 Am:Sth/Cent  2003  2005    CS  Prev      COPD:LF   0    Ever       Cigs  Nev cigs
  MONTNE   3     b   20   59    Eu:Scand  1992  1998    CS  Prev        CB/EM   0    Ever        Any   Nev any
  MUELLE   3 x   m   20   69      Am:USA  1967  1971    CS  Prev      COPD:LF   0    Ever        Any   Nev any
  MUELLE  11 x   f   20   69      Am:USA  1967  1971    CS  Prev      COPD:LF   0    Ever        Any   Nev any
  NIEPSU   3     b   19   69     Eu:East  2001  2002    CS  Prev      COPD:LF   0    Ever       Cigs  Nev cigs
  NIHLEN   3     b   20   67    Eu:Scand  1992  2004    Pr   Inc   COPD/CB/EM   0    Ever        Any   Nev any
  NILSSO   9     m   18   99    Eu:Scand  1963  2001    Pr   Inc    COPD:mort   2    Ever        Any   Nev any
  NILSSO   6     f   18   99    Eu:Scand  1963  2001    Pr   Inc    COPD:mort   2    Ever  Cigs only   Nev any
  PEREZP   1     f   40   99 Am:Sth/Cent  1992  1996    CC  Prev      COPD:LF   0    Ever       Cigs  Nev cigs
    PETO   3     m   25   92       Eu:UK  1954  1983    Pr   Inc    COPD:mort   0    Ever       Cigs  Nev cigs
  RENWIC   3     b   45   99       Eu:UK  1992  1996    CS  Prev      COPD:LF   0    Ever        Any   Nev any
  RICCIO   3     b   25   99     Eu:West  2002  2005    CS  Prev      COPD:LF   0    Ever       Cigs  Nev cigs
  SARGEA   3 x   m   45   74       Eu:UK  1993  2000    CS  Prev     COPD:oth   0    Ever       Cigs  Nev cigs
  SARGEA   6 x   f   45   74       Eu:UK  1993  2000    CS  Prev     COPD:oth   0    Ever       Cigs  Nev cigs
  SHAHAB   3     b   35   99       Eu:UK  2001  2006    CS  Prev      COPD:LF   0    Ever       Cigs  Nev cigs
    SHIN   3 x   m   18   99   Asia:FarE  1999  2003    CS  Prev      COPD:LF   0    Ever       Cigs  Nev cigs
    SHIN   4 x   f   18   99   Asia:FarE  1999  2003    CS  Prev      COPD:LF   0    Ever       Cigs  Nev cigs
  SICHLE   1     b   21   80 Eu:SE/Balkn  2000  2005    CS  Prev     COPD:oth   3    Ever       Cigs  Nev cigs
   SILVA   3     b   20   99      Am:USA  1972  2004    Pr   Inc COPD:LF/symp   6    Ever       Cigs  Nev cigs
  SPEIZE   3     m   25   86      Am:USA  1974  1989    Pr   Inc    COPD:mort   1    Ever       Cigs  Nev cigs
  SPEIZE   6     f   25   86      Am:USA  1974  1989    Pr   Inc    COPD:mort   1    Ever       Cigs  Nev cigs
   STROM   3     m   68   68    Eu:Scand  1982  1996    CS  Prev      COPD:LF   0    Ever        Any   Nev any
    TANG   4     m   35   99       Eu:UK  1967  1995    Pr   Inc    COPD:mort   2    Ever        Any   Nev any
    THUN   5     m   30   99      Am:USA  1982  1997    Pr   Inc    COPD:mort   1    Ever  Cigs only   Nev any
    THUN   6     f   30   99      Am:USA  1982  1997    Pr   Inc    COPD:mort   1    Ever  Cigs only   Nev any
    TODD   6 x   m   35   81       Eu:UK  1965  1978    Pr   Inc    COPD:mort   0    Ever        Any   Nev any
    TODD  41 x   f   35   81       Eu:UK  1965  1978    Pr   Inc    COPD:mort   0    Ever        Any   Nev any
  TRUPIN   3 x   b   55   75      Am:USA  2001  2005    CS  Prev   COPD/CB/EM   0    Ever       Cigs  Nev cigs
  TSUSHI   3     b    0   99   Asia:FarE  2003  2006    CS  Prev      COPD:LF   0    Ever       Cigs  Nev cigs
  TVERDA   3     m   35   65    Eu:Scand  1972  1993    Pr   Inc    COPD:mort   2    Ever       Cigs  Nev cigs
  VESTBO   3     b   20   99    Eu:Scand  1976  2002    CS  Prev      COPD:LF   0    Ever        Any   Nev any
  VIEGI2   3 x   m   25   73     Eu:West  1988  2000    CS  Prev      COPD:LF   0    Ever       Cigs  Nev cigs
  VIEGI2   9 x   f   25   73     Eu:West  1988  2000    CS  Prev      COPD:LF   0    Ever       Cigs  Nev cigs
  VONHER   3     m   30   99    Eu:Scand  1978  2000    CS  Prev        CB/EM   0    Ever        Any   Nev any
  VONHER   6     f   30   99    Eu:Scand  1978  2000    CS  Prev        CB/EM   0    Ever        Any   Nev any
   WEISS   3     m   50   69      Am:USA  1961  1963    CS  Prev      COPD:LF   0    Ever        Any   Nev any
  WILSO1   1     b   18   99      Aus/NZ  2000  2005    CS  Prev      COPD:LF   0    Ever        Any   Nev any
    XIAO   3     b    0   99   Asia:FarE     *  2004    CC  Prev   COPD:undef   0    Ever       Cigs  Nev cigs
      XU   1 x   m   35   99   Asia:FarE  2000  2005    CS  Prev        CB/EM   0    Ever       Cigs  Nev cigs
      XU   3 x   f   35   99   Asia:FarE  2000  2005    CS  Prev        CB/EM   0    Ever       Cigs  Nev cigs
  YAMAGU   3     b   40   99   Asia:FarE  1986  1988    CS  Prev      COPD:LF   6    Ever       Cigs  Nev cigs
    YUAN   3 x   m   45   71   Asia:FarE  1986  1996    Pr   Inc    COPD:mort   1    Ever       Cigs  Nev cigs
  ZIELI1   1 x   b    0   99     Eu:East  1999  2001    CS  Prev      COPD:LF   0    Ever       Cigs  Nev cigs
  ZIELI2   3     m    0   99     Eu:East  2000  2006    CS  Prev      COPD:LF   0    Ever       Cigs  Nev cigs
  ZIELI2   6     f    0   99     Eu:East  2000  2006    CS  Prev      COPD:LF   0    Ever       Cigs  Nev cigs
  ZIETKO   2     m    0   99     Eu:East     *  2005    CC  Prev      COPD:LF   0    Ever       Cigs  Nev cigs
  ZIETKO   4     f    0   99     Eu:East     *  2005    CC  Prev      COPD:LF   0    Ever       Cigs  Nev cigs


  ________________________________________________________________________________________________________________________
                                            International Evidence on Smoking and COPD, Phase 3, Analysis run on 27-SEP-10

                                                   Table 1 - A - 1 - 5

            IESCOPD - Meta-analysis of ever smoking, any product (or cigarettes if all product not available)
                                                         Any COPD
                                                      Least-adjusted


                        Number Exposed  Non-exposed
 REF    NRR SEX ADJ     Case    Cont    Case    Cont      RR        95.00%CI
 ALESSA 3   b   0         26      16      11      14      2.07 (  0.76-   5.65)
 AMIGO  1   m   1         40       -       3       -      6.73 (  1.50-  30.05)
 AMIGO  2   f   1         38       -       6       -      7.14 (  2.44-  20.88)
 Subtotal AMIGO                                           7.00 (  2.92-  16.75)
 ANDER1 3   m   1         31       -       2       -      5.13 (  1.14-  23.05)
 ANDER1 6   f   1         11       -      16       -      1.18 (  0.53-   2.64)
 Subtotal ANDER1                                          1.63 (  0.81-   3.32)
 ANDER3 3   m   0         15       8       0       8     31.00~(  1.59- 605.65)
 ANDER3 6   f   0          2       1       3       3      2.00 (  0.11-  35.81)
 Subtotal ANDER3                                          7.56 (  0.95-  59.91)
 BEDNAR 3   b   0         52     376      20     228      1.58 (  0.92-   2.71)
*BEST   37  m   1        129       -       6       -     10.02 (  4.42-  22.72)
 CHEN2  3   m   0         49    1402       8    1021      4.46 (  2.10-   9.46)
 CHEN2  6   f   0         97    1272      33    1542      3.56 (  2.38-   5.33)
 Subtotal CHEN2                                           3.75 (  2.63-   5.34)
 CLEMEN 3   m   0         57    1629      11     709      2.26 (  1.18-   4.33)
 COCCI  2   b   0         20      13       0       6     19.74~(  1.03- 379.94)
 DEAN1  3   m   0        483    2054      47     510      2.55 (  1.86-   3.50)
 DEAN1  9   f   0         98    1420     120    1538      0.88 (  0.67-   1.17)
 Subtotal DEAN1                                           1.40 (  1.14-   1.73)
 DEJONG 3   b   0         55     154       0      34     24.79~(  1.49- 411.13)
 DEMARC 3   b   0        381    8144     156    6176      1.85 (  1.53-   2.24)
 DICKIN 3   b   0         34     167       1     125     25.45 (  3.44- 188.43)
*DOLL1  3   m   2        522       -      12       -      6.54 (  3.71-  11.53)
*DOLL2  3   f   1         12       -       1       -     11.02 (  1.43-  84.78)
 EKBERG 3   m   0       2249    7692     395    2841      2.10 (  1.87-   2.36)
 EKBERG 6   f   0        546    3300     166    2581      2.57 (  2.15-   3.08)
 Subtotal EKBERG                                          2.23 (  2.02-   2.46)
 FERRI2 3   m   1        214       -      11       -      3.88 (  2.01-   7.49)
 FERRI2 10  f   1         87       -      54       -      2.28 (  1.57-   3.31)
 Subtotal FERRI2                                          2.59 (  1.88-   3.59)
 FIDAN  1   m   0         53     124       2      28      5.98 (  1.38-  26.03)
 FORAST 3   f   0         61     520      28     531      2.22 (  1.40-   3.54)
 FUKUCH 3   b   0        192    1048      64    1039      2.97 (  2.21-   4.00)
*GODTFR 3   b   3       1189       -      71       -      5.46 (  4.20-   7.10)
 GULSVI 1   m   0         54       -       1       -     18.60 (  2.52- 137.03)
 GULSVI 2   f   0         63       -      22       -      3.52 (  1.88-   6.58)
 Subtotal GULSVI                                          4.09 (  2.25-   7.43)
*HAMMO2 5   m   1          -       -       -       -      9.27 (  6.76-  12.70)
*HAMMO2 6   f   1          -       -       -       -      5.87 (  4.17-   8.26)
 Subtotal HAMMO2                                          7.51 (  5.96-   9.47)
 HARDIE 3   m   1        104       -       7       -      5.43 (  2.55-  11.58)
 HARDIE 6   f   1         41       -      31       -      2.75 (  1.72-   4.39)
 Subtotal HARDIE                                          3.32 (  2.23-   4.95)
*HARIKK 3   m   0         33     319       7     139      2.05 (  0.93-   4.53)
*HARIKK 6   f   0          7      91       0     101     16.64~(  0.96- 287.29)
 Subtotal HARIKK                                          2.39 (  1.11-   5.11)
*HIGGI4 3   m   1          -       -       -       -      3.08 (  1.72-   5.52)
 HO     3   m   0         88     604      20     282      2.05 (  1.24-   3.41)
 HO     6   f   0         27     243      30     733      2.71 (  1.58-   4.66)
 Subtotal HO                                              2.34 (  1.62-   3.38)
 HOZAWA 3   b   0       2589    5379     872    5019      2.77 (  2.54-   3.02)
 HUHTI1 3   m   0        200     331       7     115      9.93 (  4.54-  21.72)
 HUHTI1 11  f   0         21      93      56     653      2.63 (  1.52-   4.55)
 Subtotal HUHTI1                                          4.07 (  2.60-   6.37)
 HUHTI3 3   m   0         84     726       3     236      9.10 (  2.85-  29.06)
 ITABAS 1   b   0         44      35      16      25      1.96 (  0.91-   4.24)
*JACOBS 3   m   2        257       -      28       -      3.86 (  2.45-   6.09)
 JOHANN 3   b   0        134    1237      22     842      4.15 (  2.62-   6.56)
 KACHEL 3   b   0         41     688       7     503      4.28 (  1.91-   9.62)
*KAHN2  131 m   1        496       -      31       -      6.69 (  4.63-   9.66)
 KARAKA 3   b   0         59      87      25      81      2.20 (  1.26-   3.84)
 KATANC 3   b   0         54     403      21     382      2.44 (  1.44-   4.11)
 KIM    3   m   1        179       -      23       -      2.31 (  1.44-   3.72)
 KIM    6   f   1         10       -      61       -      1.74 (  0.85-   3.57)
 Subtotal KIM                                             2.12 (  1.43-   3.15)
 KIRAZ  1   f   0          9      32      25     278      3.13 (  1.34-   7.28)
 KLAYTO 1   b   0         62     117       7      76      5.75 (  2.50-  13.24)
 KOJIMA 3   m   0        169    5515      19    1871      3.02 (  1.87-   4.86)
 KOJIMA 6   f   0          5     316      20    3545      2.80 (  1.05-   7.52)
 Subtotal KOJIMA                                          2.98 (  1.94-   4.57)
  ________________________________________________________________________________________________________________________
                                            International Evidence on Smoking and COPD, Phase 3, Analysis run on 27-SEP-10

                                                   Table 1 - A - 1 - 5

            IESCOPD - Meta-analysis of ever smoking, any product (or cigarettes if all product not available)
                                                         Any COPD
                                                      Least-adjusted


                        Number Exposed  Non-exposed
 REF    NRR SEX ADJ     Case    Cont    Case    Cont      RR        95.00%CI
*KRZYZA 3   m   1         57       -       5       -      2.63 (  1.09-   6.38)
*KRZYZA 6   f   1         19       -      32       -      1.41 (  0.82-   2.41)
 Subtotal KRZYZA                                          1.67 (  1.05-   2.65)
 LAI    1   m   0        107     469      20     493      5.62 (  3.43-   9.22)
 LAI    2   f   0          7      88      27     600      1.77 (  0.75-   4.18)
 Subtotal LAI                                             4.22 (  2.75-   6.48)
*LAM1   1   m   7         19       -       4       -      4.05 (  1.31-  12.50)
*LAM1   2   f   7          2       -       2       -     26.61 (  2.08- 339.00)
 Subtotal LAM1                                            5.51 (  1.97-  15.47)
 LAM2   3   m   0        246     634      49     339      2.68 (  1.92-   3.75)
*LAM3   3   m   7         29       -       9       -      1.81 (  0.83-   3.98)
 LEBOWI 3   m   0         41     298       4     172      5.92 (  2.08-  16.80)
 LEBOWI 15  f   0         50     666      36     766      1.60 (  1.03-   2.48)
 Subtotal LEBOWI                                          1.95 (  1.30-   2.92)
*LEE    6   m   0         40    2972       1     347      4.67 (  0.64-  33.87)
*LEE    41  f   0         10    2620       5    1694      1.29 (  0.44-   3.78)
 Subtotal LEE                                             1.73 (  0.67-   4.44)
*LINDBE 3   b   3          -       -       -       -      2.28 (  1.34-   3.88)
 LINDST 5   m   0        170    3624      69    2984      2.03 (  1.53-   2.69)
 LINDST 6   f   0        145    2969      97    3670      1.85 (  1.42-   2.40)
 Subtotal LINDST                                          1.93 (  1.59-   2.34)
 LUNDB1 3   m   0         80     347      14     163      2.68 (  1.48-   4.88)
 LUNDB1 6   f   0         58     254      25     295      2.69 (  1.64-   4.43)
 Subtotal LUNDB1                                          2.69 (  1.84-   3.94)
 MADOR  3   m   0         19       7       0       3     18.20~(  0.84- 396.21)
 MANNI1 1   m   2        480       -     105       -      2.69 (  2.16-   3.34)
 MANNI1 10  f   2        305       -     156       -      3.18 (  2.61-   3.88)
 Subtotal MANNI1                                          2.95 (  2.55-   3.41)
 MANNI2 3   b   1        752       -     202       -      2.64 (  2.24-   3.12)
*MANNI3 3   b   0         76    2724      36    1895      1.47 (  0.99-   2.17)
 MARAN1 3   b   0        166     968      54    1905      6.05 (  4.41-   8.30)
*MARAN2 3   b   0         50     605      20    1324      5.47 (  3.29-   9.11)
*MARCUS 3   m   1         75       -      14       -      2.17 (  1.20-   3.90)
 MATHES 3   b   0         49      89      23     131      3.14 (  1.78-   5.51)
 MENEZ2 3   b   0        100       -      52       -      1.56 (  1.08-   2.24)
 MENEZ3 3   b   0        136       -      62       -      1.11 (  0.80-   1.54)
 MENEZ4 3   b   0         44       -      34       -      1.65 (  1.04-   2.63)
 MENEZ5 3   b   0        115       -      58       -      1.64 (  1.16-   2.33)
 MENEZ6 3   b   0        121       -      36       -      2.73 (  1.85-   4.03)
 MONTNE 3   b   0        301    4686      91    3390      2.39 (  1.88-   3.04)
 MUELLE 3   m   0         35     187       2      57      5.33 (  1.24-  22.87)
 MUELLE 11  f   0          3     154       2     169      1.65 (  0.27-   9.98)
 Subtotal MUELLE                                          3.35 (  1.08-  10.41)
 NIEPSU 3   b   0         34     191       8     177      3.94 (  1.78-   8.74)
*NIHLEN 3   b   0         91    2465      21    1815      3.19 (  1.99-   5.11)
*NILSSO 9   m   2         64       -      13       -      2.78 (  1.50-   5.14)
*NILSSO 6   f   2         63       -      57       -      5.93 (  4.02-   8.75)
 Subtotal NILSSO                                          4.78 (  3.44-   6.64)
 PEREZP 1   f   0         19      47      45     145      1.30 (  0.69-   2.44)
*PETO   3   m   0        104    2423       0     295     25.48~(  1.59- 409.05)
 RENWIC 3   b   0         59     119       9      60      3.31 (  1.54-   7.12)
 RICCIO 3   b   0        321     478     178     537      2.03 (  1.62-   2.53)
 SARGEA 3   m   0        585    1842     218    1051      1.53 (  1.29-   1.82)
 SARGEA 6   f   0        383    1354     404    2086      1.46 (  1.25-   1.71)
 Subtotal SARGEA                                          1.49 (  1.33-   1.67)
 SHAHAB 3   b   0        771    3758     322    3364      2.14 (  1.87-   2.46)
 SHIN   3   m   0         68     285      14     116      1.98 (  1.07-   3.65)
 SHIN   4   f   0          3      25      35     614      2.11 (  0.61-   7.31)
 Subtotal SHIN                                            2.00 (  1.15-   3.47)
 SICHLE 1   b   3          -       -       -       -      2.17 (  1.63-   2.91)
*SILVA  3   b   6          -       -       -       -      1.59 (  0.96-   2.63)
*SPEIZE 3   m   1         26       -       0       -     11.40 (  0.69- 187.05)
*SPEIZE 6   f   1         14       -       5       -      4.20 (  1.51-  11.66)
 Subtotal SPEIZE                                          4.72 (  1.81-  12.34)
 STROM  3   m   0        157     233      17      71      2.81 (  1.60-   4.96)
*TANG   4   m   2          -       -       -       -      4.12 (  2.33-   7.28)
*THUN   5   m   1          -       -       -       -      8.96 (  6.66-  12.06)
*THUN   6   f   1          -       -       -       -      8.05 (  6.17-  10.50)
 Subtotal THUN                                            8.44 (  6.93-  10.29)
*TODD   6   m   0         59    4250       1     520      7.22 (  1.00-  51.99)
*TODD   41  f   0          4    3173       4    2120      0.67 (  0.17-   2.67)
  ________________________________________________________________________________________________________________________
                                            International Evidence on Smoking and COPD, Phase 3, Analysis run on 27-SEP-10

                                                   Table 1 - A - 1 - 5

            IESCOPD - Meta-analysis of ever smoking, any product (or cigarettes if all product not available)
                                                         Any COPD
                                                      Least-adjusted


                        Number Exposed  Non-exposed
 REF    NRR SEX ADJ     Case    Cont    Case    Cont      RR        95.00%CI
 Subtotal TODD                                            1.46 (  0.47-   4.55)
 TRUPIN 3   b   0        304     943      73     741      3.27 (  2.49-   4.30)
 TSUSHI 3   b   0         41    1142       7    1056      5.42 (  2.42-  12.12)
*TVERDA 3   m   2         55       -       7       -      2.88 (  1.31-   6.33)
 VESTBO 3   b   0       1667    8841     234    2366      1.91 (  1.65-   2.20)
 VIEGI2 3   m   0        177     533      21     118      1.87 (  1.14-   3.06)
 VIEGI2 9   f   0         52     370      66     390      0.83 (  0.56-   1.23)
 Subtotal VIEGI2                                          1.13 (  0.83-   1.54)
 VONHER 3   m   0        655    1687      63     911      5.61 (  4.28-   7.36)
 VONHER 6   f   0        132     734     137    2888      3.79 (  2.95-   4.88)
 Subtotal VONHER                                          4.55 (  3.78-   5.47)
 WEISS  3   m   0         42     209       2      34      3.42 (  0.79-  14.77)
 WILSO1 1   b   0        112    1040      24     875      3.93 (  2.50-   6.16)
 XIAO   3   b   0         79      44      21      56      4.79 (  2.57-   8.92)
 XU     1   m   0        596    7945     450    5584      0.93 (  0.82-   1.06)
 XU     3   f   0         51     273     647   13773      3.98 (  2.92-   5.42)
 Subtotal XU                                              1.15 (  1.02-   1.29)
 YAMAGU 3   b   6        332       -     144       -      1.97 (  1.60-   2.43)
*YUAN   3   m   1         34       -      19       -      1.33 (  0.76-   2.34)
 ZIELI1 1   b   0       2364    6446     316    1884      2.19 (  1.92-   2.49)
 ZIELI2 3   m   0      11618   36365     341    2611      2.45 (  2.18-   2.74)
 ZIELI2 6   f   0       5159   26196     679    4832      1.40 (  1.29-   1.53)
 Subtotal ZIELI2                                          1.71 (  1.60-   1.83)
 ZIETKO 2   m   0         35      11       0      12     77.17~(  4.23-1408.01)
 ZIETKO 4   f   0         12       6       0      11     44.23~(  2.23- 875.91)
 Subtotal ZIETKO                                         58.88 (  7.34- 472.11)
Partial Totals         41822  183345    8556  105325
*prospective study                                        ~ With 0.5 adjustment for zero


 REF    NRR SEX ADJ             Ys       Ws       Qs       Ps
 ALESSA 3   b   0              0.73     3.80     0.04       0.16
 AMIGO  1   m   1              1.91     1.71     2.00       0.01
 AMIGO  2   f   1              1.97     3.33     4.33       0.00
 Subtotal AMIGO                1.95     5.04     6.33
 ANDER1 3   m   1              1.64     1.70     1.11       0.03
 ANDER1 6   f   1              0.17     5.96     2.60       0.69
 Subtotal ANDER1               0.49     7.66     3.71
 ANDER3 3   m   0              3.43     0.43     2.96       0.02
 ANDER3 6   f   0              0.69     0.46     0.01       0.64
 Subtotal ANDER3               2.02     0.90     2.97
 BEDNAR 3   b   0              0.46    13.11     1.80       0.10
*BEST   37  m   1              2.30     5.73    12.53       0.00
 CHEN2  3   m   0              1.50     6.80     3.04       0.00
 CHEN2  6   f   0              1.27    23.78     4.70       0.00
 Subtotal CHEN2                1.32    30.58     7.74
 CLEMEN 3   m   0              0.81     9.05     0.00       0.01
 COCCI  2   b   0              2.98     0.44     2.04       0.05
 DEAN1  3   m   0              0.94    38.77     0.47       0.00
 DEAN1  9   f   0             -0.12    50.27    45.27       0.38
 Subtotal DEAN1                0.34    89.04    45.74
 DEJONG 3   b   0              3.21     0.49     2.77       0.03
 DEMARC 3   b   0              0.62   107.30     4.73       0.00
 DICKIN 3   b   0              3.24     0.96     5.57       0.00
*DOLL1  3   m   2              1.88    11.95    13.22       0.00
*DOLL2  3   f   1              2.40     0.92     2.28       0.02
 EKBERG 3   m   0              0.74   289.16     1.99       0.00
 EKBERG 6   f   0              0.94   117.01     1.65       0.00
 Subtotal EKBERG               0.80   406.17     3.63
 FERRI2 3   m   1              1.36     8.88     2.49       0.00
 FERRI2 10  f   1              0.82    27.62     0.00       0.00
 Subtotal FERRI2               0.95    36.50     2.49
 FIDAN  1   m   0              1.79     1.78     1.65       0.02
 FORAST 3   f   0              0.80    17.88     0.01       0.00
 FUKUCH 3   b   0              1.09    43.96     3.06       0.00
*GODTFR 3   b   3              1.70    55.75    42.31       0.00
 GULSVI 1   m   0              2.92     0.96     4.23       0.00
 GULSVI 2   f   0              1.26     9.79     1.83       0.00
 Subtotal GULSVI               1.41    10.75     6.06
*HAMMO2 5   m   1              2.23    38.64    75.80       0.00
*HAMMO2 6   f   1              1.77    32.89    29.29       0.00
  ________________________________________________________________________________________________________________________
                                            International Evidence on Smoking and COPD, Phase 3, Analysis run on 27-SEP-10

                                                   Table 1 - A - 1 - 5

            IESCOPD - Meta-analysis of ever smoking, any product (or cigarettes if all product not available)
                                                         Any COPD
                                                      Least-adjusted


 REF    NRR SEX ADJ             Ys       Ws       Qs       Ps
 Subtotal HAMMO2               2.02    71.53   105.09
 HARDIE 3   m   1              1.69     6.71     5.03       0.00
 HARDIE 6   f   1              1.01    17.50     0.60       0.00
 Subtotal HARDIE               1.20    24.21     5.63
*HARIKK 3   m   0              0.72     6.14     0.07       0.07
*HARIKK 6   f   0              2.81     0.47     1.87       0.05
 Subtotal HARIKK               0.87     6.61     1.94
*HIGGI4 3   m   1              1.12    11.30     1.01       0.00
 HO     3   m   0              0.72    15.02     0.17       0.01
 HO     6   f   0              1.00    13.18     0.39       0.00
 Subtotal HO                   0.85    28.21     0.56
 HOZAWA 3   b   0              1.02   521.32    19.37       0.00
 HUHTI1 3   m   0              2.30     6.27    13.52       0.00
 HUHTI1 11  f   0              0.97    12.86     0.26       0.00
 Subtotal HUHTI1               1.40    19.13    13.78
 HUHTI3 3   m   0              2.21     2.85     5.45       0.00
 ITABAS 1   b   0              0.68     6.50     0.15       0.09
*JACOBS 3   m   2              1.35    18.53     5.10       0.00
 JOHANN 3   b   0              1.42    18.21     6.47       0.00
 KACHEL 3   b   0              1.45     5.86     2.31       0.00
*KAHN2  131 m   1              1.90    28.41    32.79       0.00
 KARAKA 3   b   0              0.79    12.38     0.02       0.01
 KATANC 3   b   0              0.89    14.04     0.06       0.00
 KIM    3   m   1              0.84    17.06     0.00       0.00
 KIM    6   f   1              0.55     7.46     0.55       0.13
 Subtotal KIM                  0.75    24.52     0.56
 KIRAZ  1   f   0              1.14     5.38     0.53       0.01
 KLAYTO 1   b   0              1.75     5.53     4.72       0.00
 KOJIMA 3   m   0              1.10    16.87     1.31       0.00
 KOJIMA 6   f   0              1.03     3.95     0.17       0.04
 Subtotal KOJIMA               1.09    20.82     1.47
*KRZYZA 3   m   1              0.97     4.92     0.10       0.03
*KRZYZA 6   f   1              0.34    13.22     3.08       0.21
 Subtotal KRZYZA               0.51    18.14     3.18
 LAI    1   m   0              1.73    15.75    12.78       0.00
 LAI    2   f   0              0.57     5.18     0.34       0.19
 Subtotal LAI                  1.44    20.93    13.12
*LAM1   1   m   7              1.40     3.02     0.99       0.02
*LAM1   2   f   7              3.28     0.59     3.57       0.01
 Subtotal LAM1                 1.71     3.61     4.56
 LAM2   3   m   0              0.99    34.48     0.90       0.00
*LAM3   3   m   7              0.59     6.25     0.34       0.14
 LEBOWI 3   m   0              1.78     3.53     3.19       0.00
 LEBOWI 15  f   0              0.47    19.77     2.53       0.04
 Subtotal LEBOWI               0.67    23.30     5.72
*LEE    6   m   0              1.54     0.98     0.50       0.13
*LEE    41  f   0              0.26     3.34     1.08       0.64
 Subtotal LEE                  0.55     4.32     1.58
*LINDBE 3   b   3              0.82    13.59     0.00       0.00
 LINDST 5   m   0              0.71    47.65     0.67       0.00
 LINDST 6   f   0              0.61    56.13     2.53       0.00
 Subtotal LINDST               0.66   103.78     3.20
 LUNDB1 3   m   0              0.99    10.76     0.28       0.00
 LUNDB1 6   f   0              0.99    15.49     0.42       0.00
 Subtotal LUNDB1               0.99    26.25     0.70
 MADOR  3   m   0              2.90     0.40     1.74       0.06
 MANNI1 1   m   2              0.99    80.88     2.16       0.00
 MANNI1 10  f   2              1.16    97.75    10.69       0.00
 Subtotal MANNI1               1.08   178.63    12.84
 MANNI2 3   b   1              0.97   139.95     2.92       0.00
*MANNI3 3   b   0              0.38    24.97     4.88       0.05
 MARAN1 3   b   0              1.80    38.31    36.33       0.00
*MARAN2 3   b   0              1.70    14.79    11.28       0.00
*MARCUS 3   m   1              0.77    11.06     0.03       0.01
 MATHES 3   b   0              1.14    12.08     1.21       0.00
 MENEZ2 3   b   0              0.44    28.87     4.20       0.02
 MENEZ3 3   b   0              0.10    35.82    18.67       0.53
 MENEZ4 3   b   0              0.50    17.85     1.89       0.03
 MENEZ5 3   b   0              0.49    31.59     3.47       0.01
 MENEZ6 3   b   0              1.00    25.35     0.80       0.00
 MONTNE 3   b   0              0.87    67.48     0.14       0.00
  ________________________________________________________________________________________________________________________
                                            International Evidence on Smoking and COPD, Phase 3, Analysis run on 27-SEP-10

                                                   Table 1 - A - 1 - 5

            IESCOPD - Meta-analysis of ever smoking, any product (or cigarettes if all product not available)
                                                         Any COPD
                                                      Least-adjusted


 REF    NRR SEX ADJ             Ys       Ws       Qs       Ps
 MUELLE 3   m   0              1.67     1.81     1.30       0.02
 MUELLE 11  f   0              0.50     1.18     0.13       0.59
 Subtotal MUELLE               1.21     3.00     1.43
 NIEPSU 3   b   0              1.37     6.05     1.79       0.00
*NIHLEN 3   b   0              1.16    17.35     1.93       0.00
*NILSSO 9   m   2              1.02    10.13     0.39       0.00
*NILSSO 6   f   2              1.78    25.40    23.11       0.00
 Subtotal NILSSO               1.56    35.53    23.50
 PEREZP 1   f   0              0.26     9.71     3.06       0.41
*PETO   3   m   0              3.24     0.50     2.90       0.02
 RENWIC 3   b   0              1.20     6.53     0.89       0.00
 RICCIO 3   b   0              0.71    78.82     1.14       0.00
 SARGEA 3   m   0              0.43   128.35    20.56       0.00
 SARGEA 6   f   0              0.38   158.63    31.76       0.00
 Subtotal SARGEA               0.40   286.98    52.32
 SHAHAB 3   b   0              0.76   201.37     0.82       0.00
 SHIN   3   m   0              0.68    10.18     0.21       0.03
 SHIN   4   f   0              0.74     2.48     0.02       0.24
 Subtotal SHIN                 0.69    12.65     0.23
 SICHLE 1   b   3              0.77    45.74     0.12       0.00
*SILVA  3   b   6              0.46    15.13     1.99       0.07
*SPEIZE 3   m   1              2.43     0.49     1.26       0.09
*SPEIZE 6   f   1              1.44     3.68     1.36       0.01
 Subtotal SPEIZE               1.55     4.17     2.63
 STROM  3   m   0              1.03    11.97     0.52       0.00
*TANG   4   m   2              1.42    11.84     4.12       0.00
*THUN   5   m   1              2.19    43.58    81.39       0.00
*THUN   6   f   1              2.09    54.36    86.22       0.00
 Subtotal THUN                 2.13    97.94   167.61
*TODD   6   m   0              1.98     0.99     1.30       0.05
*TODD   41  f   0             -0.40     2.00     3.03       0.57
 Subtotal TODD                 0.38     2.99     4.33
 TRUPIN 3   b   0              1.19    51.55     6.65       0.00
 TSUSHI 3   b   0              1.69     5.91     4.41       0.00
*TVERDA 3   m   2              1.06     6.19     0.33       0.01
 VESTBO 3   b   0              0.65   184.87     6.05       0.00
 VIEGI2 3   m   0              0.62    15.72     0.64       0.01
 VIEGI2 9   f   0             -0.19    25.22    25.83       0.35
 Subtotal VIEGI2               0.13    40.94    26.47
 VONHER 3   m   0              1.73    52.38    42.35       0.00
 VONHER 6   f   0              1.33    60.30    15.46       0.00
 Subtotal VONHER               1.52   112.68    57.81
 WEISS  3   m   0              1.23     1.79     0.29       0.10
 WILSO1 1   b   0              1.37    18.98     5.56       0.00
 XIAO   3   b   0              1.57     9.91     5.43       0.00
 XU     1   m   0             -0.07   237.81   191.72       0.27
 XU     3   f   0              1.38    40.18    12.34       0.00
 Subtotal XU                   0.14   277.99   204.06
 YAMAGU 3   b   6              0.68    87.99     1.93       0.00
*YUAN   3   m   1              0.29    12.15     3.56       0.32
 ZIELI1 1   b   0              0.78   234.00     0.45       0.00
 ZIELI2 3   m   0              0.89   291.62     1.36       0.00
 ZIELI2 6   f   0              0.34   523.09   124.93       0.00
 Subtotal ZIELI2               0.54   814.71   126.29
 ZIETKO 2   m   0              4.35     0.46     5.64       0.00
 ZIETKO 4   f   0              3.79     0.43     3.78       0.01
 Subtotal ZIETKO               4.08     0.89     9.43


  ________________________________________________________________________________________________________________________
                                            International Evidence on Smoking and COPD, Phase 3, Analysis run on 27-SEP-10

                                                   Table 1 - A - 1 - 5

            IESCOPD - Meta-analysis of ever smoking, any product (or cigarettes if all product not available)
                                                         Any COPD
                                                      Least-adjusted


                       N      133
                      NS       98


                      Wt  5295.76
                 Het Chi  1241.46
                 Het  df      132
                 Het  P       ***
               Fixed  RR     2.28
                     RRl     2.22
                     RRu     2.35
                      P       +++
              Random  RR     2.85
                     RRl     2.59
                     RRu     3.15
                      P       +++
               Asymm  P       ***


  ________________________________________________________________________________________________________________________
                                            International Evidence on Smoking and COPD, Phase 3, Analysis run on 27-SEP-10

                                                   Table 1 - A - 1 - 6

            IESCOPD - Meta-analysis of ever smoking, any product (or cigarettes if all product not available)
                                                         Any COPD
                                                      Least-adjusted


                       N      133
                      NS       98


                      Wt  5295.76
                 Het Chi  1241.46
                 Het  df      132
                 Het  P       ***
               Fixed  RR     2.28
                     RRl     2.22
                     RRu     2.35
                      P       +++
              Random  RR     2.85
                     RRl     2.59
                     RRu     3.15
                      P       +++
               Asymm  P       ***

                                   Sex
                             both      male    female     Total


                       N       41        53        39       133
                      NS       41        53        39       133


                      Wt  2224.52   1602.38   1468.86   5295.76
                 Het Chi   211.60    568.33    428.95   1241.46
                 Het  df       40        52        38       132
                 Het  P       ***       ***       ***       ***
               Fixed  RR     2.43      2.35      2.02      2.28
                     RRl     2.33      2.23      1.92      2.22
                     RRu     2.54      2.46      2.12      2.35
                      P       +++       +++       +++       +++
              Random  RR     2.58      3.51      2.50      2.85
                     RRl     2.30      2.89      2.02      2.59
                     RRu     2.90      4.28      3.09      3.15
                      P       +++       +++       +++       +++
             Between Chi                                  32.58
             Between  df                                      2
             Between  P                                     ***
             Btwn(F)  P                                    N.S.

                                        Continent
                            NAmer    Europe      Asia  oth/mult     Total


                       N       35        62        24        12       133
                      NS       25        45        17        11        98


                      Wt  1309.49   3026.14    649.00    311.13   5295.76
                 Het Chi   248.72    464.22    271.63     47.88   1241.46
                 Het  df       34        61        23        11       132
                 Het  P       ***       ***       ***       ***       ***
               Fixed  RR     3.22      2.08      1.91      1.96      2.28
                     RRl     3.05      2.00      1.77      1.76      2.22
                     RRu     3.40      2.15      2.07      2.19      2.35
                      P       +++       +++       +++       +++       +++
              Random  RR     3.54      2.64      2.76      2.22      2.85
                     RRl     2.93      2.34      2.03      1.71      2.59
                     RRu     4.28      2.99      3.74      2.88      3.15
                      P       +++       +++       +++       +++       +++
             Between Chi                                           209.01
             Between  df                                                3
             Between  P                                               ***
             Btwn(F)  P                                               ***


  ________________________________________________________________________________________________________________________
                                            International Evidence on Smoking and COPD, Phase 3, Analysis run on 27-SEP-10

                                                   Table 1 - A - 1 - 6

            IESCOPD - Meta-analysis of ever smoking, any product (or cigarettes if all product not available)
                                                         Any COPD
                                                      Least-adjusted
                               Study type
                               CC        Pr        CS     Total


                       N       16        37        80       133
                      NS       12        28        58        98


                      Wt   151.09    520.33   4624.34   5295.76
                 Het Chi    72.90    212.25    685.14   1241.46
                 Het  df       15        36        79       132
                 Het  P       ***       ***       ***       ***
               Fixed  RR     1.90      4.52      2.13      2.28
                     RRl     1.62      4.15      2.07      2.22
                     RRu     2.22      4.93      2.19      2.35
                      P       +++       +++       +++       +++
              Random  RR     3.17      3.69      2.55      2.85
                     RRl     2.00      2.92      2.30      2.59
                     RRu     5.01      4.68      2.81      3.15
                      P       +++       +++       +++       +++
             Between Chi                                 271.17
             Between  df                                      2
             Between  P                                     ***
             Btwn(F)  P                                     ***

                          COPD subtype
                             mort        LF     other     Total


                       N       29        62        42       133
                      NS       21        48        29        98


                      Wt   458.95   3494.52   1342.29   5295.76
                 Het Chi   295.61    316.36    473.45   1241.46
                 Het  df       28        61        41       132
                 Het  P       ***       ***       ***       ***
               Fixed  RR     3.99      2.17      2.17      2.28
                     RRl     3.64      2.10      2.06      2.22
                     RRu     4.37      2.24      2.29      2.35
                      P       +++       +++       +++       +++
              Random  RR     3.82      2.35      3.05      2.85
                     RRl     2.73      2.13      2.49      2.59
                     RRu     5.33      2.60      3.75      3.15
                      P       +++       +++       +++       +++
             Between Chi                                 156.03
             Between  df                                      2
             Between  P                                     ***
             Btwn(F)  P                                     ***

                             Smoking product
                              any      cigs  cigsonly     Total


                       N       48        77         8       133
                      NS       36        59         6       101


                      Wt  1513.56   3530.40    251.80   5295.76
                 Het Chi   223.71    592.11    197.63   1241.46
                 Het  df       47        76         7       132
                 Het  P       ***       ***       ***       ***
               Fixed  RR     2.60      2.04      5.05      2.28
                     RRl     2.47      1.98      4.46      2.22
                     RRu     2.74      2.11      5.71      2.35
                      P       +++       +++       +++       +++
              Random  RR     2.99      2.50      5.92      2.85
                     RRl     2.61      2.23      2.94      2.59
                     RRu     3.44      2.79     11.92      3.15
                      P       +++       +++       +++       +++
             Between Chi                                 228.01
             Between  df                                      2
             Between  P                                     ***
             Btwn(F)  P                                     ***
  ________________________________________________________________________________________________________________________
                                            International Evidence on Smoking and COPD, Phase 3, Analysis run on 27-SEP-10

                                                   Table 1 - A - 1 - 6

            IESCOPD - Meta-analysis of ever smoking, any product (or cigarettes if all product not available)
                                                         Any COPD
                                                      Least-adjusted
                                     Unexposed group
                          nev any   nev cig  nev+ any  nev+ cig     Total


                       N       57        73                   3       133
                      NS       40        55                   3        98


                      Wt  1863.11   3374.02               58.64   5295.76
                 Het Chi   517.35    504.20                0.11   1241.46
                 Het  df       56        72                   2       132
                 Het  P       ***       ***                N.S.       ***
               Fixed  RR     2.87      1.98                5.87      2.28
                     RRl     2.75      1.91                4.54      2.22
                     RRu     3.01      2.05                7.58      2.35
                      P       +++       +++                 +++       +++
              Random  RR     3.38      2.37                5.87      2.85
                     RRl     2.87      2.12                4.54      2.59
                     RRu     3.97      2.65                7.58      3.15
                      P       +++       +++                 +++       +++
             Between Chi                                           219.80
             Between  df                                                2
             Between  P                                               ***
             Btwn(F)  P                                               ***

                        Unexposed group (combining nev+ with main levels)
                          nev any   nev cig     Total


                       N       57        76       133
                      NS       40        58        98


                      Wt  1863.11   3432.66   5295.76
                 Het Chi   517.35    572.41   1241.46
                 Het  df       56        75       132
                 Het  P       ***       ***       ***
               Fixed  RR     2.87      2.02      2.28
                     RRl     2.75      1.95      2.22
                     RRu     3.01      2.09      2.35
                      P       +++       +++       +++
              Random  RR     3.38      2.48      2.85
                     RRl     2.87      2.22      2.59
                     RRu     3.97      2.78      3.15
                      P       +++       +++       +++
             Between Chi                       151.70
             Between  df                            1
             Between  P                           ***
             Btwn(F)  P                           ***


  ________________________________________________________________________________________________________________________
                                            International Evidence on Smoking and COPD, Phase 3, Analysis run on 27-SEP-10

                                                   Table 1 - A - 1 - 7

            IESCOPD - Meta-analysis of ever smoking, any product (or cigarettes if all product not available)
                                                         Any COPD
                                 Excluded studies (and stage at which they were excluded)


1       CLARK COTTON  MEYER REMYJA RUTGER SNYDER SOBRAX     SU TAKEMU  WANG4   WEIR WHICKE ZALACA
2      ALDERS ANDER2 AUERBA   BANG  BECK1  BECK2 BJORNS  BROWN CERVER CHAPMA COATES COLLEG  DEAN2  DEANE DONTA2 DOPICO
       EHRLIC ENRIGH FINKLE FLETCH FOXMAN GOLDBE HAENSZ HARRIS  HAYES HIGGI2 HIGGI3 HIGGI6 HIRAYA HOLLA2 HOLLNA  HOUSE
       HRUBEC HUCHON JENSEN JINDA2  JOSHI JOUSI1   KATO KOTAN1  KUBIK LAMBER LANGE2 LANGHA LAVECC LUNDB2 MAGNUS MANFRE
       MELLST MENEZ1  MEREN MILLER  MILNE MOLLER   NAWA NEJJAR OGILVI  OMORI OSWAL1 OSWAL2 PANDEY  PRATT   REID RIMING
        RYDER SCHWAR  SHARP SHIMUR SOBRAD STJERN SUADIC SUTINE TAGER2 TROISI URRUTI VIEGI1 VIKGRE WAGEN2  WANG2    WIG
       WILHEL WILSO2  WOODS  WOOLF   ZOIA
3      BROGGE  CHEN1  CHENG DETORR GEIJER HEDMAN JAENDI KHOURY KOTAN2 KULLER   LIU1   LIU2  PRICE  TAGER VINEIS WATSON
4       CHEN3   KAHN   LIAW   PEAT SAWICK STERLI VOLLM1 VOLLM2   WALD    WEN WOJTYN
8      DONTA1 ENSTRO FERRI1 FERRI3 HAWTHO HUHTI2  LANGE PELKON


  ________________________________________________________________________________________________________________________
                                            International Evidence on Smoking and COPD, Phase 3, Analysis run on 27-SEP-10

                                                   Table 1 - A - 1 - 8

            IESCOPD - Meta-analysis of ever smoking, any product (or cigarettes if all product not available)
                                                         Any COPD
                                             Potentially overlapping studies


     REF| REFGP|PRINC|                     OVERLAP|

  DEMARC DEMARC     1         DEMARC/URRUTI/DEMEER
  MONTNE MONTNE     1   EKBERG/STROM/NIHLEN/MONTNE
  EKBERG EKBERG     1   EKBERG/STROM/NIHLEN/MONTNE
  NIHLEN NIHLEN     1   EKBERG/STROM/NIHLEN/MONTNE
   STROM  STROM     1   EKBERG/STROM/NIHLEN/MONTNE
  HARIKK HARIKK     1         ENRIGH/HOZAWA/HARIKK
  HOZAWA HOZAWA     1         ENRIGH/HOZAWA/HARIKK
  JACOBS JACOBS     1  JACOBS/DONTA1/DONTA2/PELKON
  LUNDB1 LUNDBA     1  LINDBE/LUNDB1/LUNDB2/HEDLUN
  LINDBE LINDBE     1  LINDBE/LUNDB1/LUNDB2/HEDLUN
    PETO   PETO     1    PETO/HIGGI1/HIGGI2/HIGGI5
    TANG   TANG     1             TANG/HAWTHO/WALD
  KARAKA VINEIS     2         VINEIS/KARAKA/SARGEA
  SARGEA VINEIS     2         VINEIS/KARAKA/SARGEA
  HUHTI1 HUHTI1     1                HUHTI1/HUHTI2
  HARDIE HARDIE     1         HARDIE/JOHANN/BROGGE
  JOHANN JOHANN     1         HARDIE/JOHANN/BROGGE
  MARAN1 MARAN1     1                MARAN1/MARAN2
  MARAN2 MARAN2     1                MARAN1/MARAN2
    LAM3   LAM3     1                    LAM2/LAM3
    LAM2   LAM2     1                    LAM2/LAM3
  HAMMO2 HAMMO2     1                HAMMO2/ENSTRO
  LEBOWI LEBOWI     1                 LEBOWI/SILVA
   SILVA  SILVA     1                 LEBOWI/SILVA
  FERRI2 FERRIS     1         FERRI1/FERRI2/FERRI3
  MANNI2 MANNI2     1                MANNI2/MANNI3
  MANNI3 MANNI3     1                MANNI2/MANNI3
  WILSO1 WILSO1     1                WILSO1/WILSO2
  MENEZ3 MENEZ3     1                 AMIGO/MENEZ3
   AMIGO  AMIGO     1                 AMIGO/MENEZ3
    TODD   TODD     1                  LAMBER/TODD
  KRZYZA KRZYZA     1         SAWICK/KRZYZA/WOJTYN
  GODTFR GODTFR     1 GODT/VEST/LANG1+2/SUAD/HOLLN
  VESTBO VESTBO     1 GODT/VEST/LANG1+2/SUAD/HOLLN
  HIGGI4 HIGGI4     1                HIGGI4/HIGGI6
   KAHN2   KAHN     2                   KAHN/KAHN2

                                    Most-adjusted - insufficient data for meta-analysis
     REF|NRR|SEX|AGEL|AGEH|     REGION|BEGYR|PUBYR|STTYP|ONSET|      DISEAS|ADJ|SMOKSTA|   PRODUCT|    UNEXP|        RR|

   STROM   6   m   68   68    Eu:Scand  1982  1996    CS  Prev      COPD:LF   8    Ever        Any   Nev any          *
  SIG|

    y


  ________________________________________________________________________________________________________________________
                                            International Evidence on Smoking and COPD, Phase 3, Analysis run on 27-SEP-10

                                                    Table 1 - A - 2 -

            IESCOPD - Meta-analysis of ever smoking, cigarettes (or all products if cigarettes not available)
                                                         Any COPD


This analysis is restricted to results for:
1) Eligible study on database
2) Outcome COPD
3) Non-dose-response data
4) Ever smoking
5) Results complete enough for use in meta-analysis

Within each study, results are then selected (in the following order of preference, within each sex) for:
6) UNEXP   : never cigarettes, never any, other
7) PROD    : cigarettes, cigarettes only, any product
8) For overlapping studies: principal rather than subsidiary studies
and then for single sex results (m, f) in preference to results for both sexes combined (b).

Results adjusted for the most potential confounders are then chosen in Sections -1 to -3
(and those which actually differ from the adjusted results in Table 1 - A - 1 - 1 are marked 'x' in Section -1)
and results adjusted for the least confounders in Sections -4 to -6. (Those least adjusted results which
actually differ from the most adjusted are marked 'x' in column X in Section -4)

Section -7 shows excluded studies, together with the stage (as above) at which no qualifying
results were found.

Section -8 lists the potentially overlapping studies which have been included (1=principal, 2=subsidiary),
and any results which would have been included in preference except that they had data not complete enough
for use in meta-analysis. It also lists their significance (yes/no), if known.


  ________________________________________________________________________________________________________________________
                                            International Evidence on Smoking and COPD, Phase 3, Analysis run on 27-SEP-10

                                                   Table 1 - A - 2 - 1

            IESCOPD - Meta-analysis of ever smoking, cigarettes (or all products if cigarettes not available)
                                                         Any COPD
                                                      Most-adjusted


     REF|NRR|Cmp1A1|SEX|AGEL|AGEH|     REGION|BEGYR|PUBYR|STTYP|ONSET|      DISEAS|ADJ|SMOKSTA|   PRODUCT|    UNEXP|

  ALESSA   3          b   45   81     Eu:West  1992  1994    CC  Prev COPD:LF/symp   0    Ever       Cigs  Nev cigs
   AMIGO  11          m    0   99 Am:Sth/Cent  2001  2006    CC  Prev   COPD/CB/EM   6    Ever       Cigs  Nev cigs
   AMIGO  12          f    0   99 Am:Sth/Cent  2001  2006    CC  Prev   COPD/CB/EM   6    Ever       Cigs  Nev cigs
  ANDER1   3          m   25   74   Am:Canada  1963  1965    CS  Prev     COPD:oth   1    Ever        Any   Nev any
  ANDER1   6          f   25   74   Am:Canada  1963  1965    CS  Prev     COPD:oth   1    Ever        Any   Nev any
  ANDER3   3          m    0   99     Eu:East     *  2001    CC  Prev      COPD:LF   0    Ever        Any   Nev any
  ANDER3   6          f    0   99     Eu:East     *  2001    CC  Prev      COPD:LF   0    Ever        Any   Nev any
  BEDNAR   3          b   30   99     Eu:East  2000  2005    CS  Prev      COPD:LF   0    Ever        Any   Nev any
    BEST  37          m   30   97   Am:Canada  1955  1967    Pr   Inc    COPD:mort   1    Ever  Cigs only   Nev any
   CHEN2   9          m   35   64   Am:Canada  1994  2000    CS  Prev        CB/EM   6    Ever       Cigs  Nev cigs
   CHEN2  12          f   35   64   Am:Canada  1994  2000    CS  Prev        CB/EM   6    Ever       Cigs  Nev cigs
  CLEMEN   3          m   20   60     Eu:West  1960  1982    Pr  Prev      COPD:LF   0    Ever        Any   Nev any
   COCCI   2          b   38   78     Eu:West     *  2002    CC  Prev COPD:LF/symp   0    Ever       Cigs  Nev cigs
   DEAN1   6      x   m   35   99       Eu:UK  1969  1977    CC  Prev    COPD:mort   0    Ever MCigs only   Nev any
   DEAN1  17          f   35   99       Eu:UK  1969  1977    CC  Prev    COPD:mort   3    Ever MCigs only   Nev any
  DEJONG   3          b    0   99      Am:USA     *  2004    CS  Prev      COPD:LF   0    Ever       Cigs  Nev cigs
  DEMARC   6          b   20   44       Multi  1991  2004    CS  Prev      COPD:LF   4    Ever        Any   Nev any
  DICKIN   3          b   60   74       Eu:UK     *  1999    CS  Prev      COPD:LF   0    Ever        Any   Nev any
   DOLL1   6      x   m   20   99       Eu:UK  1951  1994    Pr   Inc    COPD:mort   2    Ever  Cigs only   Nev any
   DOLL2   3          f   20   99       Eu:UK  1951  1980    Pr   Inc    COPD:mort   1    Ever  Cigs only   Nev any
  EKBERG   3          m   15   99    Eu:Scand  1974  2005    CS  Prev      COPD:LF   0    Ever        Any   Nev any
  EKBERG   6          f   15   99    Eu:Scand  1974  2005    CS  Prev      COPD:LF   0    Ever        Any   Nev any
  FERRI2   7      x   m   25   80      Am:USA  1967  1971    CS  Prev     COPD:oth   1    Ever       Cigs  Nev cigs
  FERRI2  14      x   f   25   80      Am:USA  1967  1971    CS  Prev     COPD:oth   1    Ever       Cigs  Nev cigs
   FIDAN   2          m   15   99 Eu:SE/Balkn  2000  2004    CS  Prev     COPD:oth   3    Ever       Cigs  Nev cigs
  FORAST   6          f   55   99      Am:USA  1993  1998    CS  Prev        CB/EM   1    Ever       Cigs  Nev cigs
  FUKUCH   3          b   40   99   Asia:FarE  2000  2004    CS  Prev      COPD:LF   0    Ever       Cigs  Nev cigs
  GODTFR   3          b   20   99    Eu:Scand  1964  2002    Pr   Inc     COPD:ICD   3    Ever        Any   Nev any
  GULSVI   1          m   15   70    Eu:Scand  1972  1979    CS  Prev     COPD:oth   0    Ever        Any   Nev any
  GULSVI   2          f   15   70    Eu:Scand  1972  1979    CS  Prev     COPD:oth   0    Ever        Any   Nev any
  HAMMO2   5          m   35   99      Am:USA  1959  1966    Pr   Inc    COPD:mort   1    Ever  Cigs only   Nev any
  HAMMO2   6          f   35   99      Am:USA  1959  1966    Pr   Inc    COPD:mort   1    Ever  Cigs only   Nev any
  HARDIE   3          m   70   99    Eu:Scand  1998  2005    CS  Prev    CB/EM/Ast   1    Ever       Cigs  Nev cigs
  HARDIE   6          f   70   99    Eu:Scand  1998  2005    CS  Prev    CB/EM/Ast   1    Ever       Cigs  Nev cigs
  HARIKK   3          m   40   99      Am:USA  1962  2002    Pr   Inc     COPD:oth   0    Ever        Any   Nev any
  HARIKK   6          f   40   99      Am:USA  1962  2002    Pr   Inc     COPD:oth   0    Ever        Any   Nev any
  HIGGI4   3          m   35   99      Am:USA  1962  1989    Pr   Inc    COPD:mort   1    Ever       Cigs  Nev cigs
      HO   3          m   70   99   Asia:FarE  1991  1999    CS  Prev   COPD:undef   0    Ever       Cigs  Nev cigs
      HO   6          f   70   99   Asia:FarE  1991  1999    CS  Prev   COPD:undef   0    Ever       Cigs  Nev cigs
  HOZAWA   3          b   45   64      Am:USA  1987  2006    CS  Prev      COPD:LF   0    Ever       Cigs  Nev cigs
  HUHTI1   6          m   40   64    Eu:Scand  1961  1965    CS  Prev    CB/EM/Ast   1    Ever        Any   Nev any
  HUHTI1 201      x   f   40   64    Eu:Scand  1961  1965    CS  Prev    CB/EM/Ast   1    Ever       Cigs  Nev cigs
  HUHTI3   6          m   25   69    Eu:Scand  1968  1978    CS  Prev      COPD:LF   1    Ever        Any   Nev any
  ITABAS   1          b   60   99   Asia:FarE     *  1990    CC  Prev      COPD:LF   0    Ever       Cigs  Nev cigs
  JACOBS   6          m   40   84       Multi  1957  1999    Pr   Inc    COPD:mort   6    Ever       Cigs  Nev cigs
  JOHANN   3          b   26   82    Eu:Scand  1996  2005    CS  Prev      COPD:LF   0    Ever       Cigs  Nev cigs
  KACHEL   3          b   20   79     Eu:East     *  2003    CS  Prev      COPD:LF   0    Ever       Cigs  Nev cigs
   KAHN2 132      x   m   31   84      Am:USA  1954  1966    Pr   Inc    COPD:mort   1    Ever       Cigs   Nev any
  KARAKA   3          b   15   99 Eu:SE/Balkn     *  2003   nCC  Prev   COPD/CB/EM   0    Ever        Any   Nev any
  KATANC   3          b   70   79      Am:USA  1997  2005    CS  Prev      COPD:LF   0    Ever       Cigs  Nev cigs
     KIM   3          m   45   99   Asia:FarE  2001  2005    CS  Prev      COPD:LF   1    Ever       Cigs  Nev cigs
     KIM   6          f   45   99   Asia:FarE  2001  2005    CS  Prev      COPD:LF   1    Ever       Cigs  Nev cigs
   KIRAZ   2          f   25   99 Eu:SE/Balkn  1999  2003    CS  Prev COPD:LF/symp   1    Ever       Cigs  Nev cigs
  KLAYTO   1          b   40   99      Am:USA     *  1975    CS  Prev      COPD:LF   0    Ever       Cigs  N/L cigs
  KOJIMA   3          m   25   74   Asia:FarE  2001  2005    CS  Prev      COPD:LF   0    Ever       Cigs  Nev cigs
  KOJIMA   6          f   25   74   Asia:FarE  2001  2005    CS  Prev      COPD:LF   0    Ever       Cigs  Nev cigs
  KRZYZA   3          m   19   83     Eu:East  1968  1986    Pr   Inc      COPD:LF   1    Ever       Cigs  Nev cigs
  KRZYZA   6          f   19   83     Eu:East  1968  1986    Pr   Inc      COPD:LF   1    Ever       Cigs  Nev cigs
     LAI   1          m   18   80   Asia:FarE  2001  2006    CS  Prev      COPD:LF   0    Ever        Any   Nev any
     LAI   2          f   18   80   Asia:FarE  2001  2006    CS  Prev      COPD:LF   0    Ever        Any   Nev any
    LAM1   1          m   35   99   Asia:FarE  1976  1997    Pr   Inc    COPD:mort   7    Ever       Cigs  Nev cigs
    LAM1   2          f   35   99   Asia:FarE  1976  1997    Pr   Inc    COPD:mort   7    Ever       Cigs  Nev cigs
    LAM2   3          m   60   99   Asia:FarE  1987  2002    CS  Prev   COPD:undef   0    Ever       Cigs  Nev cigs
    LAM3   3          m   60   99   Asia:FarE  1987  2006    Pr   Inc    COPD:mort   7    Ever       Cigs  Nev cigs
  LEBOWI   6          m   15   96      Am:USA  1972  1977    CS  Prev      COPD:LF   1    Ever       Cigs  Nev cigs
  LEBOWI  18          f   15   96      Am:USA  1972  1977    CS  Prev      COPD:LF   1    Ever       Cigs  Nev cigs
     LEE  21      x   m   35   82       Eu:UK  1964  1979    Pr   Inc    COPD:mort   1    Ever       Cigs   Nev any
     LEE  42      x   f   35   82       Eu:UK  1964  1979    Pr   Inc    COPD:mort   0    Ever       Cigs   Nev any
  LINDBE   3          b   46   84    Eu:Scand  1996  2006    Pr   Inc      COPD:LF   3    Ever        Any   Nev any
  LINDST   9          b   20   69    Eu:Scand     *  2001    CS  Prev        CB/EM   5    Ever       Cigs  Nev cigs
  LUNDB1   9          b   46   77    Eu:Scand  1996  2003    CS  Prev      COPD:LF   4    Ever       Cigs  Nev cigs
   MADOR   3          m   15   99      Am:USA     *  2003    CC  Prev      COPD:LF   0    Ever       Cigs  Nev cigs
  ________________________________________________________________________________________________________________________
                                            International Evidence on Smoking and COPD, Phase 3, Analysis run on 27-SEP-10

                                                   Table 1 - A - 2 - 1

            IESCOPD - Meta-analysis of ever smoking, cigarettes (or all products if cigarettes not available)
                                                         Any COPD
                                                      Most-adjusted


     REF|NRR|Cmp1A1|SEX|AGEL|AGEH|     REGION|BEGYR|PUBYR|STTYP|ONSET|      DISEAS|ADJ|SMOKSTA|   PRODUCT|    UNEXP|

  MANNI1   7      x   m   17   99      Am:USA  1988  2000    CS  Prev      COPD:LF   2    Ever       Cigs  Nev cigs
  MANNI1  10          f   17   99      Am:USA  1988  2000    CS  Prev      COPD:LF   2    Ever       Cigs   Nev any
  MANNI2   3          b   25   74      Am:USA  1971  2003    CS  Prev      COPD:LF   1    Ever        Any   Nev any
  MANNI3   3          b   25   96      Am:USA  1971  2003    Pr   Inc    COPD:mort   0    Ever        Any   Nev any
  MARAN1   3          b   60   99 Asia:SE/Pac  1998  2002    CS  Prev     COPD:oth   0    Ever       Cigs  N/L cigs
  MARAN2   3          b   60   99 Asia:SE/Pac  1998  2002    Pr   Inc     COPD:oth   0    Ever       Cigs  N/L cigs
  MARCUS   3          m   45   85      Am:USA  1965  1989    Pr   Inc    COPD:mort   1    Ever        Any   Nev any
  MATHES   3          b   45   70      Aus/NZ     *  2006    CC  Prev      COPD:LF   0    Ever       Cigs  Nev cigs
  MENEZ2   6          b   40   99 Am:Sth/Cent  2003  2005    CS  Prev      COPD:LF   5    Ever       Cigs  Nev cigs
  MENEZ3   3          b   40   99 Am:Sth/Cent  2003  2005    CS  Prev      COPD:LF   0    Ever       Cigs  Nev cigs
  MENEZ4   3          b   40   99 Am:Sth/Cent  2003  2005    CS  Prev      COPD:LF   0    Ever       Cigs  Nev cigs
  MENEZ5   3          b   40   99 Am:Sth/Cent  2003  2005    CS  Prev      COPD:LF   0    Ever       Cigs  Nev cigs
  MENEZ6   3          b   40   99 Am:Sth/Cent  2003  2005    CS  Prev      COPD:LF   0    Ever       Cigs  Nev cigs
  MONTNE   3          b   20   59    Eu:Scand  1992  1998    CS  Prev        CB/EM   0    Ever        Any   Nev any
  MUELLE   6          m   20   69      Am:USA  1967  1971    CS  Prev      COPD:LF   1    Ever        Any   Nev any
  MUELLE  13          f   20   69      Am:USA  1967  1971    CS  Prev      COPD:LF   1    Ever        Any   Nev any
  NIEPSU   3          b   19   69     Eu:East  2001  2002    CS  Prev      COPD:LF   0    Ever       Cigs  Nev cigs
  NIHLEN   3          b   20   67    Eu:Scand  1992  2004    Pr   Inc   COPD/CB/EM   0    Ever        Any   Nev any
  NILSSO   3      x   m   18   99    Eu:Scand  1963  2001    Pr   Inc    COPD:mort   2    Ever  Cigs only   Nev any
  NILSSO   6          f   18   99    Eu:Scand  1963  2001    Pr   Inc    COPD:mort   2    Ever  Cigs only   Nev any
  PEREZP   1          f   40   99 Am:Sth/Cent  1992  1996    CC  Prev      COPD:LF   0    Ever       Cigs  Nev cigs
    PETO   3          m   25   92       Eu:UK  1954  1983    Pr   Inc    COPD:mort   0    Ever       Cigs  Nev cigs
  RENWIC   3          b   45   99       Eu:UK  1992  1996    CS  Prev      COPD:LF   0    Ever        Any   Nev any
  RICCIO   3          b   25   99     Eu:West  2002  2005    CS  Prev      COPD:LF   0    Ever       Cigs  Nev cigs
  SARGEA   9          b   45   74       Eu:UK  1993  2000    CS  Prev     COPD:oth   5    Ever       Cigs  Nev cigs
  SHAHAB   3          b   35   99       Eu:UK  2001  2006    CS  Prev      COPD:LF   0    Ever       Cigs  Nev cigs
    SHIN   5          b   18   99   Asia:FarE  1999  2003    CS  Prev      COPD:LF   3    Ever       Cigs  Nev cigs
  SICHLE   1          b   21   80 Eu:SE/Balkn  2000  2005    CS  Prev     COPD:oth   3    Ever       Cigs  Nev cigs
   SILVA   3          b   20   99      Am:USA  1972  2004    Pr   Inc COPD:LF/symp   6    Ever       Cigs  Nev cigs
  SPEIZE   3          m   25   86      Am:USA  1974  1989    Pr   Inc    COPD:mort   1    Ever       Cigs  Nev cigs
  SPEIZE   6          f   25   86      Am:USA  1974  1989    Pr   Inc    COPD:mort   1    Ever       Cigs  Nev cigs
   STROM   3          m   68   68    Eu:Scand  1982  1996    CS  Prev      COPD:LF   0    Ever        Any   Nev any
    TANG   4          m   35   99       Eu:UK  1967  1995    Pr   Inc    COPD:mort   2    Ever        Any   Nev any
    THUN   5          m   30   99      Am:USA  1982  1997    Pr   Inc    COPD:mort   1    Ever  Cigs only   Nev any
    THUN   6          f   30   99      Am:USA  1982  1997    Pr   Inc    COPD:mort   1    Ever  Cigs only   Nev any
    TODD  21      x   m   35   81       Eu:UK  1965  1978    Pr   Inc    COPD:mort   1    Ever       Cigs   Nev any
    TODD  42      x   f   35   81       Eu:UK  1965  1978    Pr   Inc    COPD:mort   0    Ever       Cigs   Nev any
  TRUPIN   6          b   55   75      Am:USA  2001  2005    CS  Prev   COPD/CB/EM   4    Ever       Cigs  Nev cigs
  TSUSHI   3          b    0   99   Asia:FarE  2003  2006    CS  Prev      COPD:LF   0    Ever       Cigs  Nev cigs
  TVERDA   3          m   35   65    Eu:Scand  1972  1993    Pr   Inc    COPD:mort   2    Ever       Cigs  Nev cigs
  VESTBO   3          b   20   99    Eu:Scand  1976  2002    CS  Prev      COPD:LF   0    Ever        Any   Nev any
  VIEGI2   6          m   25   73     Eu:West  1988  2000    CS  Prev      COPD:LF   1    Ever       Cigs  Nev cigs
  VIEGI2  12          f   25   73     Eu:West  1988  2000    CS  Prev      COPD:LF   1    Ever       Cigs  Nev cigs
  VONHER   3          m   30   99    Eu:Scand  1978  2000    CS  Prev        CB/EM   0    Ever        Any   Nev any
  VONHER   6          f   30   99    Eu:Scand  1978  2000    CS  Prev        CB/EM   0    Ever        Any   Nev any
   WEISS   3          m   50   69      Am:USA  1961  1963    CS  Prev      COPD:LF   0    Ever        Any   Nev any
  WILSO1   1          b   18   99      Aus/NZ  2000  2005    CS  Prev      COPD:LF   0    Ever        Any   Nev any
    XIAO   3          b    0   99   Asia:FarE     *  2004    CC  Prev   COPD:undef   0    Ever       Cigs  Nev cigs
      XU   2          m   35   99   Asia:FarE  2000  2005    CS  Prev        CB/EM  13    Ever       Cigs  Nev cigs
      XU   4          f   35   99   Asia:FarE  2000  2005    CS  Prev        CB/EM  13    Ever       Cigs  Nev cigs
  YAMAGU   3          b   40   99   Asia:FarE  1986  1988    CS  Prev      COPD:LF   6    Ever       Cigs  Nev cigs
    YUAN   5          m   45   71   Asia:FarE  1986  1996    Pr   Inc    COPD:mort   2    Ever       Cigs  Nev cigs
  ZIELI1   2          b    0   99     Eu:East  1999  2001    CS  Prev      COPD:LF   1    Ever       Cigs  Nev cigs
  ZIELI2   3          m    0   99     Eu:East  2000  2006    CS  Prev      COPD:LF   0    Ever       Cigs  Nev cigs
  ZIELI2   6          f    0   99     Eu:East  2000  2006    CS  Prev      COPD:LF   0    Ever       Cigs  Nev cigs
  ZIETKO   2          m    0   99     Eu:East     *  2005    CC  Prev      COPD:LF   0    Ever       Cigs  Nev cigs
  ZIETKO   4          f    0   99     Eu:East     *  2005    CC  Prev      COPD:LF   0    Ever       Cigs  Nev cigs


  ________________________________________________________________________________________________________________________
                                            International Evidence on Smoking and COPD, Phase 3, Analysis run on 27-SEP-10

                                                   Table 1 - A - 2 - 2

            IESCOPD - Meta-analysis of ever smoking, cigarettes (or all products if cigarettes not available)
                                                         Any COPD
                                                      Most-adjusted


                        Number Exposed  Non-exposed
 REF    NRR SEX ADJ     Case    Cont    Case    Cont      RR        95.00%CI
 ALESSA 3   b   0         26      16      11      14      2.07 (  0.76-   5.65)
 AMIGO  11  m   6         40       -       3       -      4.65 (  0.98-  22.09)
 AMIGO  12  f   6         38       -       6       -      7.88 (  2.55-  24.36)
 Subtotal AMIGO                                           6.57 (  2.64-  16.39)
 ANDER1 3   m   1         31       -       2       -      5.13 (  1.14-  23.05)
 ANDER1 6   f   1         11       -      16       -      1.18 (  0.53-   2.64)
 Subtotal ANDER1                                          1.63 (  0.81-   3.32)
 ANDER3 3   m   0         15       8       0       8     31.00~(  1.59- 605.65)
 ANDER3 6   f   0          2       1       3       3      2.00 (  0.11-  35.81)
 Subtotal ANDER3                                          7.56 (  0.95-  59.91)
 BEDNAR 3   b   0         52     376      20     228      1.58 (  0.92-   2.71)
*BEST   37  m   1        129       -       6       -     10.02 (  4.42-  22.72)
 CHEN2  9   m   6         49       -       8       -      3.43 (  1.60-   7.38)
 CHEN2  12  f   6         97       -      33       -      2.57 (  1.62-   4.07)
 Subtotal CHEN2                                           2.78 (  1.87-   4.12)
 CLEMEN 3   m   0         57    1629      11     709      2.26 (  1.18-   4.33)
 COCCI  2   b   0         20      13       0       6     19.74~(  1.03- 379.94)
 DEAN1  6   m   0        319    1227      47     510      2.82 (  2.04-   3.90)
 DEAN1  17  f   3         98       -     120       -      1.92 (  1.37-   2.69)
 Subtotal DEAN1                                           2.35 (  1.86-   2.96)
 DEJONG 3   b   0         55     154       0      34     24.79~(  1.49- 411.13)
 DEMARC 6   b   4        381       -     156       -      1.76 (  1.44-   2.15)
 DICKIN 3   b   0         34     167       1     125     25.45 (  3.44- 188.43)
*DOLL1  6   m   2        334       -      12       -      8.48 (  4.80-  15.01)
*DOLL2  3   f   1         12       -       1       -     11.02 (  1.43-  84.78)
 EKBERG 3   m   0       2249    7692     395    2841      2.10 (  1.87-   2.36)
 EKBERG 6   f   0        546    3300     166    2581      2.57 (  2.15-   3.08)
 Subtotal EKBERG                                          2.23 (  2.02-   2.46)
 FERRI2 7   m   1        208       -      18       -      4.40 (  2.59-   7.47)
 FERRI2 14  f   1         88       -      54       -      2.31 (  1.59-   3.35)
 Subtotal FERRI2                                          2.86 (  2.11-   3.88)
 FIDAN  2   m   3         53       -       2       -      6.96 (  1.45-  33.26)
 FORAST 6   f   1         61       -      28       -      2.36 (  1.48-   3.77)
 FUKUCH 3   b   0        192    1048      64    1039      2.97 (  2.21-   4.00)
*GODTFR 3   b   3       1189       -      71       -      5.46 (  4.20-   7.10)
 GULSVI 1   m   0         54       -       1       -     18.60 (  2.52- 137.03)
 GULSVI 2   f   0         63       -      22       -      3.52 (  1.88-   6.58)
 Subtotal GULSVI                                          4.09 (  2.25-   7.43)
*HAMMO2 5   m   1          -       -       -       -      9.27 (  6.76-  12.70)
*HAMMO2 6   f   1          -       -       -       -      5.87 (  4.17-   8.26)
 Subtotal HAMMO2                                          7.51 (  5.96-   9.47)
 HARDIE 3   m   1        104       -       7       -      5.43 (  2.55-  11.58)
 HARDIE 6   f   1         41       -      31       -      2.75 (  1.72-   4.39)
 Subtotal HARDIE                                          3.32 (  2.23-   4.95)
*HARIKK 3   m   0         33     319       7     139      2.05 (  0.93-   4.53)
*HARIKK 6   f   0          7      91       0     101     16.64~(  0.96- 287.29)
 Subtotal HARIKK                                          2.39 (  1.11-   5.11)
*HIGGI4 3   m   1          -       -       -       -      3.08 (  1.72-   5.52)
 HO     3   m   0         88     604      20     282      2.05 (  1.24-   3.41)
 HO     6   f   0         27     243      30     733      2.71 (  1.58-   4.66)
 Subtotal HO                                              2.34 (  1.62-   3.38)
 HOZAWA 3   b   0       2589    5379     872    5019      2.77 (  2.54-   3.02)
 HUHTI1 6   m   1        200       -       7       -      8.18 (  3.68-  18.18)
 HUHTI1 201 f   1         21       -      56       -      2.88 (  1.62-   5.13)
 Subtotal HUHTI1                                          4.12 (  2.58-   6.57)
 HUHTI3 6   m   1         84       -       3       -      7.74 (  2.62-  22.87)
 ITABAS 1   b   0         44      35      16      25      1.96 (  0.91-   4.24)
*JACOBS 6   m   6        248       -      28       -      3.08 (  2.03-   4.65)
 JOHANN 3   b   0        134    1237      22     842      4.15 (  2.62-   6.56)
 KACHEL 3   b   0         41     688       7     503      4.28 (  1.91-   9.62)
*KAHN2  132 m   1        476       -      31       -      8.49 (  5.87-  12.27)
 KARAKA 3   b   0         59      87      25      81      2.20 (  1.26-   3.84)
 KATANC 3   b   0         54     403      21     382      2.44 (  1.44-   4.11)
 KIM    3   m   1        179       -      23       -      2.31 (  1.44-   3.72)
 KIM    6   f   1         10       -      61       -      1.74 (  0.85-   3.57)
 Subtotal KIM                                             2.12 (  1.43-   3.15)
 KIRAZ  2   f   1          9       -      25       -      6.86 (  2.47-  19.06)
 KLAYTO 1   b   0         62     117       7      76      5.75 (  2.50-  13.24)
 KOJIMA 3   m   0        169    5515      19    1871      3.02 (  1.87-   4.86)
 KOJIMA 6   f   0          5     316      20    3545      2.80 (  1.05-   7.52)
 Subtotal KOJIMA                                          2.98 (  1.94-   4.57)
  ________________________________________________________________________________________________________________________
                                            International Evidence on Smoking and COPD, Phase 3, Analysis run on 27-SEP-10

                                                   Table 1 - A - 2 - 2

            IESCOPD - Meta-analysis of ever smoking, cigarettes (or all products if cigarettes not available)
                                                         Any COPD
                                                      Most-adjusted


                        Number Exposed  Non-exposed
 REF    NRR SEX ADJ     Case    Cont    Case    Cont      RR        95.00%CI
*KRZYZA 3   m   1         57       -       5       -      2.63 (  1.09-   6.38)
*KRZYZA 6   f   1         19       -      32       -      1.41 (  0.82-   2.41)
 Subtotal KRZYZA                                          1.67 (  1.05-   2.65)
 LAI    1   m   0        107     469      20     493      5.62 (  3.43-   9.22)
 LAI    2   f   0          7      88      27     600      1.77 (  0.75-   4.18)
 Subtotal LAI                                             4.22 (  2.75-   6.48)
*LAM1   1   m   7         19       -       4       -      4.05 (  1.31-  12.50)
*LAM1   2   f   7          2       -       2       -     26.61 (  2.08- 339.00)
 Subtotal LAM1                                            5.51 (  1.97-  15.47)
 LAM2   3   m   0        246     634      49     339      2.68 (  1.92-   3.75)
*LAM3   3   m   7         29       -       9       -      1.81 (  0.83-   3.98)
 LEBOWI 6   m   1         41       -       4       -      5.02 (  1.76-  14.35)
 LEBOWI 18  f   1         50       -      36       -      1.66 (  1.07-   2.59)
 Subtotal LEBOWI                                          1.96 (  1.31-   2.95)
*LEE    21  m   1         38       -       1       -      5.44 (  0.75-  39.49)
*LEE    42  f   0          8    1855       5    1694      1.46 (  0.48-   4.46)
 Subtotal LEE                                             2.00 (  0.76-   5.30)
*LINDBE 3   b   3          -       -       -       -      2.28 (  1.34-   3.88)
 LINDST 9   b   5        315       -     166       -      1.84 (  1.50-   2.25)
 LUNDB1 9   b   4        138       -      39       -      3.16 (  2.01-   4.97)
 MADOR  3   m   0         19       7       0       3     18.20~(  0.84- 396.21)
 MANNI1 7   m   2        458       -     127       -      2.45 (  2.00-   3.00)
 MANNI1 10  f   2        305       -     156       -      3.18 (  2.61-   3.88)
 Subtotal MANNI1                                          2.80 (  2.43-   3.23)
 MANNI2 3   b   1        752       -     202       -      2.64 (  2.24-   3.12)
*MANNI3 3   b   0         76    2724      36    1895      1.47 (  0.99-   2.17)
 MARAN1 3   b   0        166     968      54    1905      6.05 (  4.41-   8.30)
*MARAN2 3   b   0         50     605      20    1324      5.47 (  3.29-   9.11)
*MARCUS 3   m   1         75       -      14       -      2.17 (  1.20-   3.90)
 MATHES 3   b   0         49      89      23     131      3.14 (  1.78-   5.51)
 MENEZ2 6   b   5        100       -      52       -      1.54 (  1.08-   2.17)
 MENEZ3 3   b   0        136       -      62       -      1.11 (  0.80-   1.54)
 MENEZ4 3   b   0         44       -      34       -      1.65 (  1.04-   2.63)
 MENEZ5 3   b   0        115       -      58       -      1.64 (  1.16-   2.33)
 MENEZ6 3   b   0        121       -      36       -      2.73 (  1.85-   4.03)
 MONTNE 3   b   0        301    4686      91    3390      2.39 (  1.88-   3.04)
 MUELLE 6   m   1         35       -       2       -      4.41 (  1.01-  19.20)
 MUELLE 13  f   1          3       -       2       -      1.60 (  0.26-   9.92)
 Subtotal MUELLE                                          2.95 (  0.94-   9.28)
 NIEPSU 3   b   0         34     191       8     177      3.94 (  1.78-   8.74)
*NIHLEN 3   b   0         91    2465      21    1815      3.19 (  1.99-   5.11)
*NILSSO 3   m   2        124       -      31       -      3.86 (  2.58-   5.77)
*NILSSO 6   f   2         63       -      57       -      5.93 (  4.02-   8.75)
 Subtotal NILSSO                                          4.82 (  3.64-   6.37)
 PEREZP 1   f   0         19      47      45     145      1.30 (  0.69-   2.44)
*PETO   3   m   0        104    2423       0     295     25.48~(  1.59- 409.05)
 RENWIC 3   b   0         59     119       9      60      3.31 (  1.54-   7.12)
 RICCIO 3   b   0        321     478     178     537      2.03 (  1.62-   2.53)
 SARGEA 9   b   5        968       -     622       -      1.40 (  1.25-   1.58)
 SHAHAB 3   b   0        771    3758     322    3364      2.14 (  1.87-   2.46)
 SHIN   5   b   3         71       -      49       -      1.87 (  1.14-   3.09)
 SICHLE 1   b   3          -       -       -       -      2.17 (  1.63-   2.91)
*SILVA  3   b   6          -       -       -       -      1.59 (  0.96-   2.63)
*SPEIZE 3   m   1         26       -       0       -     11.40 (  0.69- 187.05)
*SPEIZE 6   f   1         14       -       5       -      4.20 (  1.51-  11.66)
 Subtotal SPEIZE                                          4.72 (  1.81-  12.34)
 STROM  3   m   0        157     233      17      71      2.81 (  1.60-   4.96)
*TANG   4   m   2          -       -       -       -      4.12 (  2.33-   7.28)
*THUN   5   m   1          -       -       -       -      8.96 (  6.66-  12.06)
*THUN   6   f   1          -       -       -       -      8.05 (  6.17-  10.50)
 Subtotal THUN                                            8.44 (  6.93-  10.29)
*TODD   21  m   1         51       -       1       -      7.29 (  1.01-  52.67)
*TODD   42  f   0          1    2203       4    2120      0.24 (  0.03-   2.15)
 Subtotal TODD                                            1.58 (  0.36-   6.84)
 TRUPIN 6   b   4        304       -      73       -      3.45 (  2.59-   4.59)
 TSUSHI 3   b   0         41    1142       7    1056      5.42 (  2.42-  12.12)
*TVERDA 3   m   2         55       -       7       -      2.88 (  1.31-   6.33)
 VESTBO 3   b   0       1667    8841     234    2366      1.91 (  1.65-   2.20)
 VIEGI2 6   m   1        168       -      21       -      1.39 (  0.83-   2.32)
 VIEGI2 12  f   1         52       -      66       -      1.20 (  0.80-   1.80)
 Subtotal VIEGI2                                          1.27 (  0.92-   1.75)
  ________________________________________________________________________________________________________________________
                                            International Evidence on Smoking and COPD, Phase 3, Analysis run on 27-SEP-10

                                                   Table 1 - A - 2 - 2

            IESCOPD - Meta-analysis of ever smoking, cigarettes (or all products if cigarettes not available)
                                                         Any COPD
                                                      Most-adjusted


                        Number Exposed  Non-exposed
 REF    NRR SEX ADJ     Case    Cont    Case    Cont      RR        95.00%CI
 VONHER 3   m   0        655    1687      63     911      5.61 (  4.28-   7.36)
 VONHER 6   f   0        132     734     137    2888      3.79 (  2.95-   4.88)
 Subtotal VONHER                                          4.55 (  3.78-   5.47)
 WEISS  3   m   0         42     209       2      34      3.42 (  0.79-  14.77)
 WILSO1 1   b   0        112    1040      24     875      3.93 (  2.50-   6.16)
 XIAO   3   b   0         79      44      21      56      4.79 (  2.57-   8.92)
 XU     2   m   13       596       -     450       -      1.26 (  1.07-   1.48)
 XU     4   f   13        51       -     647       -      2.61 (  1.85-   3.68)
 Subtotal XU                                              1.44 (  1.24-   1.67)
 YAMAGU 3   b   6        332       -     144       -      1.97 (  1.60-   2.43)
*YUAN   5   m   2         34       -      19       -      1.40 (  0.80-   2.45)
 ZIELI1 2   b   1       2364       -     316       -      2.06 (  1.81-   2.35)
 ZIELI2 3   m   0      11618   36365     341    2611      2.45 (  2.18-   2.74)
 ZIELI2 6   f   0       5159   26196     679    4832      1.40 (  1.29-   1.53)
 Subtotal ZIELI2                                          1.71 (  1.60-   1.83)
 ZIETKO 2   m   0         35      11       0      12     77.17~(  4.23-1408.01)
 ZIETKO 4   f   0         12       6       0      11     44.23~(  2.23- 875.91)
 Subtotal ZIETKO                                         58.88 (  7.34- 472.11)
Partial Totals         41450  130982    8603   57707
*prospective study                                        ~ With 0.5 adjustment for zero


 REF    NRR SEX ADJ             Ys       Ws       Qs       Ps
 ALESSA 3   b   0              0.73     3.80     0.06       0.16
 AMIGO  11  m   6              1.54     1.58     0.74       0.05
 AMIGO  12  f   6              2.06     3.02     4.43       0.00
 Subtotal AMIGO                1.88     4.60     5.17
 ANDER1 3   m   1              1.64     1.70     1.04       0.03
 ANDER1 6   f   1              0.17     5.96     2.81       0.69
 Subtotal ANDER1               0.49     7.66     3.85
 ANDER3 3   m   0              3.43     0.43     2.90       0.02
 ANDER3 6   f   0              0.69     0.46     0.01       0.64
 Subtotal ANDER3               2.02     0.90     2.91
 BEDNAR 3   b   0              0.46    13.11     2.07       0.10
*BEST   37  m   1              2.30     5.73    12.09       0.00
 CHEN2  9   m   6              1.23     6.57     0.95       0.00
 CHEN2  12  f   6              0.94    18.11     0.15       0.00
 Subtotal CHEN2                1.02    24.68     1.10
 CLEMEN 3   m   0              0.81     9.05     0.01       0.01
 COCCI  2   b   0              2.98     0.44     1.99       0.05
 DEAN1  6   m   0              1.04    36.78     1.25       0.00
 DEAN1  17  f   3              0.65    33.75     1.35       0.00
 Subtotal DEAN1                0.85    70.53     2.61
 DEJONG 3   b   0              3.21     0.49     2.71       0.03
 DEMARC 6   b   4              0.57    95.64     7.89       0.00
 DICKIN 3   b   0              3.24     0.96     5.45       0.00
*DOLL1  6   m   2              2.14    11.82    19.53       0.00
*DOLL2  3   f   1              2.40     0.92     2.21       0.02
 EKBERG 3   m   0              0.74   289.16     3.45       0.00
 EKBERG 6   f   0              0.94   117.01     1.00       0.00
 Subtotal EKBERG               0.80   406.17     4.45
 FERRI2 7   m   1              1.48    13.70     5.42       0.00
 FERRI2 14  f   1              0.84    27.67     0.01       0.00
 Subtotal FERRI2               1.05    41.36     5.43
 FIDAN  2   m   3              1.94     1.57     1.85       0.02
 FORAST 6   f   1              0.86    17.58     0.00       0.00
 FUKUCH 3   b   0              1.09    43.96     2.48       0.00
*GODTFR 3   b   3              1.70    55.75    39.80       0.00
 GULSVI 1   m   0              2.92     0.96     4.13       0.00
 GULSVI 2   f   0              1.26     9.79     1.61       0.00
 Subtotal GULSVI               1.41    10.75     5.74
*HAMMO2 5   m   1              2.23    38.64    72.98       0.00
*HAMMO2 6   f   1              1.77    32.89    27.68       0.00
 Subtotal HAMMO2               2.02    71.53   100.66
 HARDIE 3   m   1              1.69     6.71     4.73       0.00
 HARDIE 6   f   1              1.01    17.50     0.44       0.00
 Subtotal HARDIE               1.20    24.21     5.17
*HARIKK 3   m   0              0.72     6.14     0.11       0.07
*HARIKK 6   f   0              2.81     0.47     1.82       0.05
 Subtotal HARIKK               0.87     6.61     1.93
*HIGGI4 3   m   1              1.12    11.30     0.84       0.00
  ________________________________________________________________________________________________________________________
                                            International Evidence on Smoking and COPD, Phase 3, Analysis run on 27-SEP-10

                                                   Table 1 - A - 2 - 2

            IESCOPD - Meta-analysis of ever smoking, cigarettes (or all products if cigarettes not available)
                                                         Any COPD
                                                      Most-adjusted


 REF    NRR SEX ADJ             Ys       Ws       Qs       Ps
 HO     3   m   0              0.72    15.02     0.26       0.01
 HO     6   f   0              1.00    13.18     0.28       0.00
 Subtotal HO                   0.85    28.21     0.55
 HOZAWA 3   b   0              1.02   521.32    14.44       0.00
 HUHTI1 6   m   1              2.10     6.02     9.40       0.00
 HUHTI1 201 f   1              1.06    11.56     0.49       0.00
 Subtotal HUHTI1               1.42    17.59     9.88
 HUHTI3 6   m   1              2.05     3.27     4.67       0.00
 ITABAS 1   b   0              0.68     6.50     0.20       0.09
*JACOBS 6   m   6              1.12    22.37     1.66       0.00
 JOHANN 3   b   0              1.42    18.21     5.91       0.00
 KACHEL 3   b   0              1.45     5.86     2.12       0.00
*KAHN2  132 m   1              2.14    28.27    46.77       0.00
 KARAKA 3   b   0              0.79    12.38     0.05       0.01
 KATANC 3   b   0              0.89    14.04     0.02       0.00
 KIM    3   m   1              0.84    17.06     0.00       0.00
 KIM    6   f   1              0.55     7.46     0.67       0.13
 Subtotal KIM                  0.75    24.52     0.67
 KIRAZ  2   f   1              1.93     3.68     4.24       0.00
 KLAYTO 1   b   0              1.75     5.53     4.46       0.00
 KOJIMA 3   m   0              1.10    16.87     1.07       0.00
 KOJIMA 6   f   0              1.03     3.95     0.13       0.04
 Subtotal KOJIMA               1.09    20.82     1.20
*KRZYZA 3   m   1              0.97     4.92     0.06       0.03
*KRZYZA 6   f   1              0.34    13.22     3.42       0.21
 Subtotal KRZYZA               0.51    18.14     3.49
 LAI    1   m   0              1.73    15.75    12.04       0.00
 LAI    2   f   0              0.57     5.18     0.41       0.19
 Subtotal LAI                  1.44    20.93    12.46
*LAM1   1   m   7              1.40     3.02     0.90       0.02
*LAM1   2   f   7              3.28     0.59     3.49       0.01
 Subtotal LAM1                 1.71     3.61     4.39
 LAM2   3   m   0              0.99    34.48     0.63       0.00
*LAM3   3   m   7              0.59     6.25     0.42       0.14
 LEBOWI 6   m   1              1.61     3.49     2.02       0.00
 LEBOWI 18  f   1              0.51    19.66     2.35       0.02
 Subtotal LEBOWI               0.67    23.15     4.37
*LEE    21  m   1              1.69     0.98     0.69       0.09
*LEE    42  f   0              0.38     3.09     0.69       0.51
 Subtotal LEE                  0.70     4.07     1.38
*LINDBE 3   b   3              0.82    13.59     0.01       0.00
 LINDST 9   b   5              0.61    93.46     5.51       0.00
 LUNDB1 9   b   4              1.15    18.75     1.67       0.00
 MADOR  3   m   0              2.90     0.40     1.70       0.06
 MANNI1 7   m   2              0.90    93.46     0.18       0.00
 MANNI1 10  f   2              1.16    97.75     9.05       0.00
 Subtotal MANNI1               1.03   191.21     9.23
 MANNI2 3   b   1              0.97   139.95     1.96       0.00
*MANNI3 3   b   0              0.38    24.97     5.47       0.05
 MARAN1 3   b   0              1.80    38.31    34.39       0.00
*MARAN2 3   b   0              1.70    14.79    10.61       0.00
*MARCUS 3   m   1              0.77    11.06     0.07       0.01
 MATHES 3   b   0              1.14    12.08     1.02       0.00
 MENEZ2 6   b   5              0.43    31.56     5.59       0.02
 MENEZ3 3   b   0              0.10    35.82    20.05       0.53
 MENEZ4 3   b   0              0.50    17.85     2.21       0.03
 MENEZ5 3   b   0              0.49    31.59     4.04       0.01
 MENEZ6 3   b   0              1.00    25.35     0.58       0.00
 MONTNE 3   b   0              0.87    67.48     0.03       0.00
 MUELLE 6   m   1              1.48     1.77     0.71       0.05
 MUELLE 13  f   1              0.47     1.16     0.17       0.61
 Subtotal MUELLE               1.08     2.93     0.88
 NIEPSU 3   b   0              1.37     6.05     1.62       0.00
*NIHLEN 3   b   0              1.16    17.35     1.64       0.00
*NILSSO 3   m   2              1.35    23.72     5.89       0.00
*NILSSO 6   f   2              1.78    25.40    21.85       0.00
 Subtotal NILSSO               1.57    49.12    27.74
 PEREZP 1   f   0              0.26     9.71     3.36       0.41
*PETO   3   m   0              3.24     0.50     2.84       0.02
 RENWIC 3   b   0              1.20     6.53     0.77       0.00
 RICCIO 3   b   0              0.71    78.82     1.69       0.00
  ________________________________________________________________________________________________________________________
                                            International Evidence on Smoking and COPD, Phase 3, Analysis run on 27-SEP-10

                                                   Table 1 - A - 2 - 2

            IESCOPD - Meta-analysis of ever smoking, cigarettes (or all products if cigarettes not available)
                                                         Any COPD
                                                      Most-adjusted


 REF    NRR SEX ADJ             Ys       Ws       Qs       Ps
 SARGEA 9   b   5              0.34   279.95    74.56       0.00
 SHAHAB 3   b   0              0.76   201.37     1.64       0.00
 SHIN   5   b   3              0.63    15.45     0.79       0.01
 SICHLE 1   b   3              0.77    45.74     0.28       0.00
*SILVA  3   b   6              0.46    15.13     2.29       0.07
*SPEIZE 3   m   1              2.43     0.49     1.22       0.09
*SPEIZE 6   f   1              1.44     3.68     1.25       0.01
 Subtotal SPEIZE               1.55     4.17     2.47
 STROM  3   m   0              1.03    11.97     0.40       0.00
*TANG   4   m   2              1.42    11.84     3.76       0.00
*THUN   5   m   1              2.19    43.58    78.28       0.00
*THUN   6   f   1              2.09    54.36    82.66       0.00
 Subtotal THUN                 2.13    97.94   160.94
*TODD   21  m   1              1.99     0.98     1.26       0.05
*TODD   42  f   0             -1.42     0.80     4.15       0.20
 Subtotal TODD                 0.46     1.78     5.42
 TRUPIN 6   b   4              1.24    46.93     6.99       0.00
 TSUSHI 3   b   0              1.69     5.91     4.14       0.00
*TVERDA 3   m   2              1.06     6.19     0.26       0.01
 VESTBO 3   b   0              0.65   184.87     7.94       0.00
 VIEGI2 6   m   1              0.33    14.54     3.98       0.21
 VIEGI2 12  f   1              0.18    23.37    10.50       0.38
 Subtotal VIEGI2               0.24    37.91    14.48
 VONHER 3   m   0              1.73    52.38    39.90       0.00
 VONHER 6   f   0              1.33    60.30    13.90       0.00
 Subtotal VONHER               1.52   112.68    53.80
 WEISS  3   m   0              1.23     1.79     0.25       0.10
 WILSO1 1   b   0              1.37    18.98     5.04       0.00
 XIAO   3   b   0              1.57     9.91     5.05       0.00
 XU     2   m   13             0.23   146.03    56.39       0.01
 XU     4   f   13             0.96    32.49     0.37       0.00
 Subtotal XU                   0.36   178.52    56.76
 YAMAGU 3   b   6              0.68    87.99     2.68       0.00
*YUAN   5   m   2              0.34    12.27     3.27       0.24
 ZIELI1 2   b   1              0.72   225.41     3.80       0.00
 ZIELI2 3   m   0              0.89   291.62     0.51       0.00
 ZIELI2 6   f   0              0.34   523.09   138.74       0.00
 Subtotal ZIELI2               0.54   814.71   139.25
 ZIETKO 2   m   0              4.35     0.46     5.56       0.00
 ZIETKO 4   f   0              3.79     0.43     3.72       0.01
 Subtotal ZIETKO               4.08     0.89     9.28

                       N      129
                      NS       98


                      Wt  5153.89
                 Het Chi  1072.15
                 Het  df      128
                 Het  P       ***
               Fixed  RR     2.35
                     RRl     2.28
                     RRu     2.41
                      P       +++
              Random  RR     2.92
                     RRl     2.65
                     RRu     3.20
                      P       +++
               Asymm  P       ***


  ________________________________________________________________________________________________________________________
                                            International Evidence on Smoking and COPD, Phase 3, Analysis run on 27-SEP-10

                                                   Table 1 - A - 2 - 3

            IESCOPD - Meta-analysis of ever smoking, cigarettes (or all products if cigarettes not available)
                                                         Any COPD
                                                      Most-adjusted


                       N      129
                      NS       98


                      Wt  5153.89
                 Het Chi  1072.15
                 Het  df      128
                 Het  P       ***
               Fixed  RR     2.35
                     RRl     2.28
                     RRu     2.41
                      P       +++
              Random  RR     2.92
                     RRl     2.65
                     RRu     3.20
                      P       +++
               Asymm  P       ***

                                   Sex
                             both      male    female     Total


                       N       45        49        35       129
                      NS       45        49        35       129


                      Wt  2609.95   1344.70   1199.24   5153.89
                 Het Chi   299.93    389.23    340.90   1072.15
                 Het  df       44        48        34       128
                 Het  P       ***       ***       ***       ***
               Fixed  RR     2.26      2.72      2.16      2.35
                     RRl     2.17      2.58      2.04      2.28
                     RRu     2.35      2.87      2.28      2.41
                      P       +++       +++       +++       +++
              Random  RR     2.51      3.70      2.71      2.92
                     RRl     2.23      3.06      2.15      2.65
                     RRu     2.82      4.48      3.42      3.20
                      P       +++       +++       +++       +++
             Between Chi                                  42.09
             Between  df                                      2
             Between  P                                     ***
             Btwn(F)  P                                     (*)

                                        Continent
                            NAmer    Europe      Asia  oth/mult     Total


                       N       35        59        23        12       129
                      NS       25        45        17        11        98


                      Wt  1315.75   2980.15    552.45    305.55   5153.89
                 Het Chi   262.71    448.22    139.86     43.67   1072.15
                 Het  df       34        58        22        11       128
                 Het  P       ***       ***       ***       ***       ***
               Fixed  RR     3.20      2.10      2.26      1.91      2.35
                     RRl     3.04      2.03      2.08      1.71      2.28
                     RRu     3.38      2.18      2.45      2.14      2.41
                      P       +++       +++       +++       +++       +++
              Random  RR     3.52      2.84      2.73      2.13      2.92
                     RRl     2.90      2.50      2.15      1.66      2.65
                     RRu     4.27      3.22      3.46      2.73      3.20
                      P       +++       +++       +++       +++       +++
             Between Chi                                           177.70
             Between  df                                                3
             Between  P                                               ***
             Btwn(F)  P                                               ***


  ________________________________________________________________________________________________________________________
                                            International Evidence on Smoking and COPD, Phase 3, Analysis run on 27-SEP-10

                                                   Table 1 - A - 2 - 3

            IESCOPD - Meta-analysis of ever smoking, cigarettes (or all products if cigarettes not available)
                                                         Any COPD
                                                      Most-adjusted
                               Study type
                               CC        Pr        CS     Total


                       N       16        37        76       129
                      NS       12        28        58        98


                      Wt   132.14    536.14   4485.61   5153.89
                 Het Chi    32.14    220.03    545.78   1072.15
                 Het  df       15        36        75       128
                 Het  P        **       ***       ***       ***
               Fixed  RR     2.59      4.59      2.16      2.35
                     RRl     2.18      4.22      2.10      2.28
                     RRu     3.07      5.00      2.22      2.41
                      P       +++       +++       +++       +++
              Random  RR     2.98      3.81      2.55      2.92
                     RRl     2.17      3.01      2.32      2.65
                     RRu     4.09      4.83      2.80      3.20
                      P       +++       +++       +++       +++
             Between Chi                                 274.20
             Between  df                                      2
             Between  P                                     ***
             Btwn(F)  P                                     ***

                          COPD subtype
                             mort        LF     other     Total


                       N       29        60        40       129
                      NS       21        48        29        98


                      Wt   456.25   3482.03   1215.60   5153.89
                 Het Chi   197.02    303.00    339.21   1072.15
                 Het  df       28        59        39       128
                 Het  P       ***       ***       ***       ***
               Fixed  RR     4.61      2.15      2.32      2.35
                     RRl     4.20      2.08      2.19      2.28
                     RRu     5.05      2.23      2.46      2.41
                      P       +++       +++       +++       +++
              Random  RR     4.10      2.34      3.12      2.92
                     RRl     3.11      2.12      2.57      2.65
                     RRu     5.42      2.58      3.78      3.20
                      P       +++       +++       +++       +++
             Between Chi                                 232.92
             Between  df                                      2
             Between  P                                     ***
             Btwn(F)  P                                     ***

                             Smoking product
                              any      cigs  cigsonly     Total


                       N       36        82        11       129
                      NS       28        64         7        99


                      Wt  1275.20   3571.09    307.60   5153.89
                 Het Chi   176.08    527.26     90.40   1072.15
                 Het  df       35        81        10       128
                 Het  P       ***       ***       ***       ***
               Fixed  RR     2.52      2.12      5.63      2.35
                     RRl     2.39      2.05      5.03      2.28
                     RRu     2.66      2.19      6.29      2.41
                      P       +++       +++       +++       +++
              Random  RR     2.92      2.56      5.75      2.92
                     RRl     2.49      2.30      4.02      2.65
                     RRu     3.42      2.84      8.23      3.20
                      P       +++       +++       +++       +++
             Between Chi                                 278.41
             Between  df                                      2
             Between  P                                     ***
             Btwn(F)  P                                     ***
  ________________________________________________________________________________________________________________________
                                            International Evidence on Smoking and COPD, Phase 3, Analysis run on 27-SEP-10

                                                   Table 1 - A - 2 - 3

            IESCOPD - Meta-analysis of ever smoking, cigarettes (or all products if cigarettes not available)
                                                         Any COPD
                                                      Most-adjusted
                                     Unexposed group
                          nev any   nev cig  nev+ any  nev+ cig     Total


                       N       53        73                   3       129
                      NS       39        58                   3       100


                      Wt  1714.66   3380.58               58.64   5153.89
                 Het Chi   465.44    383.66                0.11   1072.15
                 Het  df       52        72                   2       128
                 Het  P       ***       ***                N.S.       ***
               Fixed  RR     3.01      2.04                5.87      2.35
                     RRl     2.87      1.97                4.54      2.28
                     RRu     3.15      2.11                7.58      2.41
                      P       +++       +++                 +++       +++
              Random  RR     3.58      2.37                5.87      2.92
                     RRl     3.03      2.15                4.54      2.65
                     RRu     4.24      2.62                7.58      3.20
                      P       +++       +++                 +++       +++
             Between Chi                                           222.94
             Between  df                                                2
             Between  P                                               ***
             Btwn(F)  P                                               ***

                        Unexposed group (combining nev+ with main levels)
                          nev any   nev cig     Total


                       N       53        76       129
                      NS       39        61       100


                      Wt  1714.66   3439.22   5153.89
                 Het Chi   465.44    448.43   1072.15
                 Het  df       52        75       128
                 Het  P       ***       ***       ***
               Fixed  RR     3.01      2.07      2.35
                     RRl     2.87      2.00      2.28
                     RRu     3.15      2.14      2.41
                      P       +++       +++       +++
              Random  RR     3.58      2.49      2.92
                     RRl     3.03      2.24      2.65
                     RRu     4.24      2.75      3.20
                      P       +++       +++       +++
             Between Chi                       158.28
             Between  df                            1
             Between  P                           ***
             Btwn(F)  P                           ***


  ________________________________________________________________________________________________________________________
                                            International Evidence on Smoking and COPD, Phase 3, Analysis run on 27-SEP-10

                                                   Table 1 - A - 2 - 4

            IESCOPD - Meta-analysis of ever smoking, cigarettes (or all products if cigarettes not available)
                                                         Any COPD
                                                      Least-adjusted


     REF|NRR|X|SEX|AGEL|AGEH|     REGION|BEGYR|PUBYR|STTYP|ONSET|      DISEAS|ADJ|SMOKSTA|   PRODUCT|    UNEXP|

  ALESSA   3     b   45   81     Eu:West  1992  1994    CC  Prev COPD:LF/symp   0    Ever       Cigs  Nev cigs
   AMIGO   1 x   m    0   99 Am:Sth/Cent  2001  2006    CC  Prev   COPD/CB/EM   1    Ever       Cigs  Nev cigs
   AMIGO   2 x   f    0   99 Am:Sth/Cent  2001  2006    CC  Prev   COPD/CB/EM   1    Ever       Cigs  Nev cigs
  ANDER1   3     m   25   74   Am:Canada  1963  1965    CS  Prev     COPD:oth   1    Ever        Any   Nev any
  ANDER1   6     f   25   74   Am:Canada  1963  1965    CS  Prev     COPD:oth   1    Ever        Any   Nev any
  ANDER3   3     m    0   99     Eu:East     *  2001    CC  Prev      COPD:LF   0    Ever        Any   Nev any
  ANDER3   6     f    0   99     Eu:East     *  2001    CC  Prev      COPD:LF   0    Ever        Any   Nev any
  BEDNAR   3     b   30   99     Eu:East  2000  2005    CS  Prev      COPD:LF   0    Ever        Any   Nev any
    BEST  37     m   30   97   Am:Canada  1955  1967    Pr   Inc    COPD:mort   1    Ever  Cigs only   Nev any
   CHEN2   3 x   m   35   64   Am:Canada  1994  2000    CS  Prev        CB/EM   0    Ever       Cigs  Nev cigs
   CHEN2   6 x   f   35   64   Am:Canada  1994  2000    CS  Prev        CB/EM   0    Ever       Cigs  Nev cigs
  CLEMEN   3     m   20   60     Eu:West  1960  1982    Pr  Prev      COPD:LF   0    Ever        Any   Nev any
   COCCI   2     b   38   78     Eu:West     *  2002    CC  Prev COPD:LF/symp   0    Ever       Cigs  Nev cigs
   DEAN1   6     m   35   99       Eu:UK  1969  1977    CC  Prev    COPD:mort   0    Ever MCigs only   Nev any
   DEAN1   9 x   f   35   99       Eu:UK  1969  1977    CC  Prev    COPD:mort   0    Ever MCigs only   Nev any
  DEJONG   3     b    0   99      Am:USA     *  2004    CS  Prev      COPD:LF   0    Ever       Cigs  Nev cigs
  DEMARC   3 x   b   20   44       Multi  1991  2004    CS  Prev      COPD:LF   0    Ever        Any   Nev any
  DICKIN   3     b   60   74       Eu:UK     *  1999    CS  Prev      COPD:LF   0    Ever        Any   Nev any
   DOLL1   6     m   20   99       Eu:UK  1951  1994    Pr   Inc    COPD:mort   2    Ever  Cigs only   Nev any
   DOLL2   3     f   20   99       Eu:UK  1951  1980    Pr   Inc    COPD:mort   1    Ever  Cigs only   Nev any
  EKBERG   3     m   15   99    Eu:Scand  1974  2005    CS  Prev      COPD:LF   0    Ever        Any   Nev any
  EKBERG   6     f   15   99    Eu:Scand  1974  2005    CS  Prev      COPD:LF   0    Ever        Any   Nev any
  FERRI2   7     m   25   80      Am:USA  1967  1971    CS  Prev     COPD:oth   1    Ever       Cigs  Nev cigs
  FERRI2  14     f   25   80      Am:USA  1967  1971    CS  Prev     COPD:oth   1    Ever       Cigs  Nev cigs
   FIDAN   1 x   m   15   99 Eu:SE/Balkn  2000  2004    CS  Prev     COPD:oth   0    Ever       Cigs  Nev cigs
  FORAST   3 x   f   55   99      Am:USA  1993  1998    CS  Prev        CB/EM   0    Ever       Cigs  Nev cigs
  FUKUCH   3     b   40   99   Asia:FarE  2000  2004    CS  Prev      COPD:LF   0    Ever       Cigs  Nev cigs
  GODTFR   3     b   20   99    Eu:Scand  1964  2002    Pr   Inc     COPD:ICD   3    Ever        Any   Nev any
  GULSVI   1     m   15   70    Eu:Scand  1972  1979    CS  Prev     COPD:oth   0    Ever        Any   Nev any
  GULSVI   2     f   15   70    Eu:Scand  1972  1979    CS  Prev     COPD:oth   0    Ever        Any   Nev any
  HAMMO2   5     m   35   99      Am:USA  1959  1966    Pr   Inc    COPD:mort   1    Ever  Cigs only   Nev any
  HAMMO2   6     f   35   99      Am:USA  1959  1966    Pr   Inc    COPD:mort   1    Ever  Cigs only   Nev any
  HARDIE   3     m   70   99    Eu:Scand  1998  2005    CS  Prev    CB/EM/Ast   1    Ever       Cigs  Nev cigs
  HARDIE   6     f   70   99    Eu:Scand  1998  2005    CS  Prev    CB/EM/Ast   1    Ever       Cigs  Nev cigs
  HARIKK   3     m   40   99      Am:USA  1962  2002    Pr   Inc     COPD:oth   0    Ever        Any   Nev any
  HARIKK   6     f   40   99      Am:USA  1962  2002    Pr   Inc     COPD:oth   0    Ever        Any   Nev any
  HIGGI4   3     m   35   99      Am:USA  1962  1989    Pr   Inc    COPD:mort   1    Ever       Cigs  Nev cigs
      HO   3     m   70   99   Asia:FarE  1991  1999    CS  Prev   COPD:undef   0    Ever       Cigs  Nev cigs
      HO   6     f   70   99   Asia:FarE  1991  1999    CS  Prev   COPD:undef   0    Ever       Cigs  Nev cigs
  HOZAWA   3     b   45   64      Am:USA  1987  2006    CS  Prev      COPD:LF   0    Ever       Cigs  Nev cigs
  HUHTI1   3 x   m   40   64    Eu:Scand  1961  1965    CS  Prev    CB/EM/Ast   0    Ever        Any   Nev any
  HUHTI1 199 x   f   40   64    Eu:Scand  1961  1965    CS  Prev    CB/EM/Ast   0    Ever       Cigs  Nev cigs
  HUHTI3   3 x   m   25   69    Eu:Scand  1968  1978    CS  Prev      COPD:LF   0    Ever        Any   Nev any
  ITABAS   1     b   60   99   Asia:FarE     *  1990    CC  Prev      COPD:LF   0    Ever       Cigs  Nev cigs
  JACOBS   3 x   m   40   84       Multi  1957  1999    Pr   Inc    COPD:mort   2    Ever       Cigs  Nev cigs
  JOHANN   3     b   26   82    Eu:Scand  1996  2005    CS  Prev      COPD:LF   0    Ever       Cigs  Nev cigs
  KACHEL   3     b   20   79     Eu:East     *  2003    CS  Prev      COPD:LF   0    Ever       Cigs  Nev cigs
   KAHN2 132     m   31   84      Am:USA  1954  1966    Pr   Inc    COPD:mort   1    Ever       Cigs   Nev any
  KARAKA   3     b   15   99 Eu:SE/Balkn     *  2003   nCC  Prev   COPD/CB/EM   0    Ever        Any   Nev any
  KATANC   3     b   70   79      Am:USA  1997  2005    CS  Prev      COPD:LF   0    Ever       Cigs  Nev cigs
     KIM   3     m   45   99   Asia:FarE  2001  2005    CS  Prev      COPD:LF   1    Ever       Cigs  Nev cigs
     KIM   6     f   45   99   Asia:FarE  2001  2005    CS  Prev      COPD:LF   1    Ever       Cigs  Nev cigs
   KIRAZ   1 x   f   25   99 Eu:SE/Balkn  1999  2003    CS  Prev COPD:LF/symp   0    Ever       Cigs  Nev cigs
  KLAYTO   1     b   40   99      Am:USA     *  1975    CS  Prev      COPD:LF   0    Ever       Cigs  N/L cigs
  KOJIMA   3     m   25   74   Asia:FarE  2001  2005    CS  Prev      COPD:LF   0    Ever       Cigs  Nev cigs
  KOJIMA   6     f   25   74   Asia:FarE  2001  2005    CS  Prev      COPD:LF   0    Ever       Cigs  Nev cigs
  KRZYZA   3     m   19   83     Eu:East  1968  1986    Pr   Inc      COPD:LF   1    Ever       Cigs  Nev cigs
  KRZYZA   6     f   19   83     Eu:East  1968  1986    Pr   Inc      COPD:LF   1    Ever       Cigs  Nev cigs
     LAI   1     m   18   80   Asia:FarE  2001  2006    CS  Prev      COPD:LF   0    Ever        Any   Nev any
     LAI   2     f   18   80   Asia:FarE  2001  2006    CS  Prev      COPD:LF   0    Ever        Any   Nev any
    LAM1   1     m   35   99   Asia:FarE  1976  1997    Pr   Inc    COPD:mort   7    Ever       Cigs  Nev cigs
    LAM1   2     f   35   99   Asia:FarE  1976  1997    Pr   Inc    COPD:mort   7    Ever       Cigs  Nev cigs
    LAM2   3     m   60   99   Asia:FarE  1987  2002    CS  Prev   COPD:undef   0    Ever       Cigs  Nev cigs
    LAM3   3     m   60   99   Asia:FarE  1987  2006    Pr   Inc    COPD:mort   7    Ever       Cigs  Nev cigs
  LEBOWI   3 x   m   15   96      Am:USA  1972  1977    CS  Prev      COPD:LF   0    Ever       Cigs  Nev cigs
  LEBOWI  15 x   f   15   96      Am:USA  1972  1977    CS  Prev      COPD:LF   0    Ever       Cigs  Nev cigs
     LEE   7 x   m   35   82       Eu:UK  1964  1979    Pr   Inc    COPD:mort   0    Ever       Cigs   Nev any
     LEE  42     f   35   82       Eu:UK  1964  1979    Pr   Inc    COPD:mort   0    Ever       Cigs   Nev any
  LINDBE   3     b   46   84    Eu:Scand  1996  2006    Pr   Inc      COPD:LF   3    Ever        Any   Nev any
  LINDST   5 x   m   20   69    Eu:Scand     *  2001    CS  Prev        CB/EM   0    Ever       Cigs  Nev cigs
  LINDST   6 x   f   20   69    Eu:Scand     *  2001    CS  Prev        CB/EM   0    Ever       Cigs  Nev cigs
  LUNDB1   3 x   m   46   77    Eu:Scand  1996  2003    CS  Prev      COPD:LF   0    Ever       Cigs  Nev cigs
  ________________________________________________________________________________________________________________________
                                            International Evidence on Smoking and COPD, Phase 3, Analysis run on 27-SEP-10

                                                   Table 1 - A - 2 - 4

            IESCOPD - Meta-analysis of ever smoking, cigarettes (or all products if cigarettes not available)
                                                         Any COPD
                                                      Least-adjusted


     REF|NRR|X|SEX|AGEL|AGEH|     REGION|BEGYR|PUBYR|STTYP|ONSET|      DISEAS|ADJ|SMOKSTA|   PRODUCT|    UNEXP|

  LUNDB1   6 x   f   46   77    Eu:Scand  1996  2003    CS  Prev      COPD:LF   0    Ever       Cigs  Nev cigs
   MADOR   3     m   15   99      Am:USA     *  2003    CC  Prev      COPD:LF   0    Ever       Cigs  Nev cigs
  MANNI1   7     m   17   99      Am:USA  1988  2000    CS  Prev      COPD:LF   2    Ever       Cigs  Nev cigs
  MANNI1  10     f   17   99      Am:USA  1988  2000    CS  Prev      COPD:LF   2    Ever       Cigs   Nev any
  MANNI2   3     b   25   74      Am:USA  1971  2003    CS  Prev      COPD:LF   1    Ever        Any   Nev any
  MANNI3   3     b   25   96      Am:USA  1971  2003    Pr   Inc    COPD:mort   0    Ever        Any   Nev any
  MARAN1   3     b   60   99 Asia:SE/Pac  1998  2002    CS  Prev     COPD:oth   0    Ever       Cigs  N/L cigs
  MARAN2   3     b   60   99 Asia:SE/Pac  1998  2002    Pr   Inc     COPD:oth   0    Ever       Cigs  N/L cigs
  MARCUS   3     m   45   85      Am:USA  1965  1989    Pr   Inc    COPD:mort   1    Ever        Any   Nev any
  MATHES   3     b   45   70      Aus/NZ     *  2006    CC  Prev      COPD:LF   0    Ever       Cigs  Nev cigs
  MENEZ2   3 x   b   40   99 Am:Sth/Cent  2003  2005    CS  Prev      COPD:LF   0    Ever       Cigs  Nev cigs
  MENEZ3   3     b   40   99 Am:Sth/Cent  2003  2005    CS  Prev      COPD:LF   0    Ever       Cigs  Nev cigs
  MENEZ4   3     b   40   99 Am:Sth/Cent  2003  2005    CS  Prev      COPD:LF   0    Ever       Cigs  Nev cigs
  MENEZ5   3     b   40   99 Am:Sth/Cent  2003  2005    CS  Prev      COPD:LF   0    Ever       Cigs  Nev cigs
  MENEZ6   3     b   40   99 Am:Sth/Cent  2003  2005    CS  Prev      COPD:LF   0    Ever       Cigs  Nev cigs
  MONTNE   3     b   20   59    Eu:Scand  1992  1998    CS  Prev        CB/EM   0    Ever        Any   Nev any
  MUELLE   3 x   m   20   69      Am:USA  1967  1971    CS  Prev      COPD:LF   0    Ever        Any   Nev any
  MUELLE  11 x   f   20   69      Am:USA  1967  1971    CS  Prev      COPD:LF   0    Ever        Any   Nev any
  NIEPSU   3     b   19   69     Eu:East  2001  2002    CS  Prev      COPD:LF   0    Ever       Cigs  Nev cigs
  NIHLEN   3     b   20   67    Eu:Scand  1992  2004    Pr   Inc   COPD/CB/EM   0    Ever        Any   Nev any
  NILSSO   3     m   18   99    Eu:Scand  1963  2001    Pr   Inc    COPD:mort   2    Ever  Cigs only   Nev any
  NILSSO   6     f   18   99    Eu:Scand  1963  2001    Pr   Inc    COPD:mort   2    Ever  Cigs only   Nev any
  PEREZP   1     f   40   99 Am:Sth/Cent  1992  1996    CC  Prev      COPD:LF   0    Ever       Cigs  Nev cigs
    PETO   3     m   25   92       Eu:UK  1954  1983    Pr   Inc    COPD:mort   0    Ever       Cigs  Nev cigs
  RENWIC   3     b   45   99       Eu:UK  1992  1996    CS  Prev      COPD:LF   0    Ever        Any   Nev any
  RICCIO   3     b   25   99     Eu:West  2002  2005    CS  Prev      COPD:LF   0    Ever       Cigs  Nev cigs
  SARGEA   3 x   m   45   74       Eu:UK  1993  2000    CS  Prev     COPD:oth   0    Ever       Cigs  Nev cigs
  SARGEA   6 x   f   45   74       Eu:UK  1993  2000    CS  Prev     COPD:oth   0    Ever       Cigs  Nev cigs
  SHAHAB   3     b   35   99       Eu:UK  2001  2006    CS  Prev      COPD:LF   0    Ever       Cigs  Nev cigs
    SHIN   3 x   m   18   99   Asia:FarE  1999  2003    CS  Prev      COPD:LF   0    Ever       Cigs  Nev cigs
    SHIN   4 x   f   18   99   Asia:FarE  1999  2003    CS  Prev      COPD:LF   0    Ever       Cigs  Nev cigs
  SICHLE   1     b   21   80 Eu:SE/Balkn  2000  2005    CS  Prev     COPD:oth   3    Ever       Cigs  Nev cigs
   SILVA   3     b   20   99      Am:USA  1972  2004    Pr   Inc COPD:LF/symp   6    Ever       Cigs  Nev cigs
  SPEIZE   3     m   25   86      Am:USA  1974  1989    Pr   Inc    COPD:mort   1    Ever       Cigs  Nev cigs
  SPEIZE   6     f   25   86      Am:USA  1974  1989    Pr   Inc    COPD:mort   1    Ever       Cigs  Nev cigs
   STROM   3     m   68   68    Eu:Scand  1982  1996    CS  Prev      COPD:LF   0    Ever        Any   Nev any
    TANG   4     m   35   99       Eu:UK  1967  1995    Pr   Inc    COPD:mort   2    Ever        Any   Nev any
    THUN   5     m   30   99      Am:USA  1982  1997    Pr   Inc    COPD:mort   1    Ever  Cigs only   Nev any
    THUN   6     f   30   99      Am:USA  1982  1997    Pr   Inc    COPD:mort   1    Ever  Cigs only   Nev any
    TODD   7 x   m   35   81       Eu:UK  1965  1978    Pr   Inc    COPD:mort   0    Ever       Cigs   Nev any
    TODD  42     f   35   81       Eu:UK  1965  1978    Pr   Inc    COPD:mort   0    Ever       Cigs   Nev any
  TRUPIN   3 x   b   55   75      Am:USA  2001  2005    CS  Prev   COPD/CB/EM   0    Ever       Cigs  Nev cigs
  TSUSHI   3     b    0   99   Asia:FarE  2003  2006    CS  Prev      COPD:LF   0    Ever       Cigs  Nev cigs
  TVERDA   3     m   35   65    Eu:Scand  1972  1993    Pr   Inc    COPD:mort   2    Ever       Cigs  Nev cigs
  VESTBO   3     b   20   99    Eu:Scand  1976  2002    CS  Prev      COPD:LF   0    Ever        Any   Nev any
  VIEGI2   3 x   m   25   73     Eu:West  1988  2000    CS  Prev      COPD:LF   0    Ever       Cigs  Nev cigs
  VIEGI2   9 x   f   25   73     Eu:West  1988  2000    CS  Prev      COPD:LF   0    Ever       Cigs  Nev cigs
  VONHER   3     m   30   99    Eu:Scand  1978  2000    CS  Prev        CB/EM   0    Ever        Any   Nev any
  VONHER   6     f   30   99    Eu:Scand  1978  2000    CS  Prev        CB/EM   0    Ever        Any   Nev any
   WEISS   3     m   50   69      Am:USA  1961  1963    CS  Prev      COPD:LF   0    Ever        Any   Nev any
  WILSO1   1     b   18   99      Aus/NZ  2000  2005    CS  Prev      COPD:LF   0    Ever        Any   Nev any
    XIAO   3     b    0   99   Asia:FarE     *  2004    CC  Prev   COPD:undef   0    Ever       Cigs  Nev cigs
      XU   1 x   m   35   99   Asia:FarE  2000  2005    CS  Prev        CB/EM   0    Ever       Cigs  Nev cigs
      XU   3 x   f   35   99   Asia:FarE  2000  2005    CS  Prev        CB/EM   0    Ever       Cigs  Nev cigs
  YAMAGU   3     b   40   99   Asia:FarE  1986  1988    CS  Prev      COPD:LF   6    Ever       Cigs  Nev cigs
    YUAN   3 x   m   45   71   Asia:FarE  1986  1996    Pr   Inc    COPD:mort   1    Ever       Cigs  Nev cigs
  ZIELI1   1 x   b    0   99     Eu:East  1999  2001    CS  Prev      COPD:LF   0    Ever       Cigs  Nev cigs
  ZIELI2   3     m    0   99     Eu:East  2000  2006    CS  Prev      COPD:LF   0    Ever       Cigs  Nev cigs
  ZIELI2   6     f    0   99     Eu:East  2000  2006    CS  Prev      COPD:LF   0    Ever       Cigs  Nev cigs
  ZIETKO   2     m    0   99     Eu:East     *  2005    CC  Prev      COPD:LF   0    Ever       Cigs  Nev cigs
  ZIETKO   4     f    0   99     Eu:East     *  2005    CC  Prev      COPD:LF   0    Ever       Cigs  Nev cigs


  ________________________________________________________________________________________________________________________
                                            International Evidence on Smoking and COPD, Phase 3, Analysis run on 27-SEP-10

                                                   Table 1 - A - 2 - 5

            IESCOPD - Meta-analysis of ever smoking, cigarettes (or all products if cigarettes not available)
                                                         Any COPD
                                                      Least-adjusted


                        Number Exposed  Non-exposed
 REF    NRR SEX ADJ     Case    Cont    Case    Cont      RR        95.00%CI
 ALESSA 3   b   0         26      16      11      14      2.07 (  0.76-   5.65)
 AMIGO  1   m   1         40       -       3       -      6.73 (  1.50-  30.05)
 AMIGO  2   f   1         38       -       6       -      7.14 (  2.44-  20.88)
 Subtotal AMIGO                                           7.00 (  2.92-  16.75)
 ANDER1 3   m   1         31       -       2       -      5.13 (  1.14-  23.05)
 ANDER1 6   f   1         11       -      16       -      1.18 (  0.53-   2.64)
 Subtotal ANDER1                                          1.63 (  0.81-   3.32)
 ANDER3 3   m   0         15       8       0       8     31.00~(  1.59- 605.65)
 ANDER3 6   f   0          2       1       3       3      2.00 (  0.11-  35.81)
 Subtotal ANDER3                                          7.56 (  0.95-  59.91)
 BEDNAR 3   b   0         52     376      20     228      1.58 (  0.92-   2.71)
*BEST   37  m   1        129       -       6       -     10.02 (  4.42-  22.72)
 CHEN2  3   m   0         49    1402       8    1021      4.46 (  2.10-   9.46)
 CHEN2  6   f   0         97    1272      33    1542      3.56 (  2.38-   5.33)
 Subtotal CHEN2                                           3.75 (  2.63-   5.34)
 CLEMEN 3   m   0         57    1629      11     709      2.26 (  1.18-   4.33)
 COCCI  2   b   0         20      13       0       6     19.74~(  1.03- 379.94)
 DEAN1  6   m   0        319    1227      47     510      2.82 (  2.04-   3.90)
 DEAN1  9   f   0         98    1420     120    1538      0.88 (  0.67-   1.17)
 Subtotal DEAN1                                           1.44 (  1.17-   1.78)
 DEJONG 3   b   0         55     154       0      34     24.79~(  1.49- 411.13)
 DEMARC 3   b   0        381    8144     156    6176      1.85 (  1.53-   2.24)
 DICKIN 3   b   0         34     167       1     125     25.45 (  3.44- 188.43)
*DOLL1  6   m   2        334       -      12       -      8.48 (  4.80-  15.01)
*DOLL2  3   f   1         12       -       1       -     11.02 (  1.43-  84.78)
 EKBERG 3   m   0       2249    7692     395    2841      2.10 (  1.87-   2.36)
 EKBERG 6   f   0        546    3300     166    2581      2.57 (  2.15-   3.08)
 Subtotal EKBERG                                          2.23 (  2.02-   2.46)
 FERRI2 7   m   1        208       -      18       -      4.40 (  2.59-   7.47)
 FERRI2 14  f   1         88       -      54       -      2.31 (  1.59-   3.35)
 Subtotal FERRI2                                          2.86 (  2.11-   3.88)
 FIDAN  1   m   0         53     124       2      28      5.98 (  1.38-  26.03)
 FORAST 3   f   0         61     520      28     531      2.22 (  1.40-   3.54)
 FUKUCH 3   b   0        192    1048      64    1039      2.97 (  2.21-   4.00)
*GODTFR 3   b   3       1189       -      71       -      5.46 (  4.20-   7.10)
 GULSVI 1   m   0         54       -       1       -     18.60 (  2.52- 137.03)
 GULSVI 2   f   0         63       -      22       -      3.52 (  1.88-   6.58)
 Subtotal GULSVI                                          4.09 (  2.25-   7.43)
*HAMMO2 5   m   1          -       -       -       -      9.27 (  6.76-  12.70)
*HAMMO2 6   f   1          -       -       -       -      5.87 (  4.17-   8.26)
 Subtotal HAMMO2                                          7.51 (  5.96-   9.47)
 HARDIE 3   m   1        104       -       7       -      5.43 (  2.55-  11.58)
 HARDIE 6   f   1         41       -      31       -      2.75 (  1.72-   4.39)
 Subtotal HARDIE                                          3.32 (  2.23-   4.95)
*HARIKK 3   m   0         33     319       7     139      2.05 (  0.93-   4.53)
*HARIKK 6   f   0          7      91       0     101     16.64~(  0.96- 287.29)
 Subtotal HARIKK                                          2.39 (  1.11-   5.11)
*HIGGI4 3   m   1          -       -       -       -      3.08 (  1.72-   5.52)
 HO     3   m   0         88     604      20     282      2.05 (  1.24-   3.41)
 HO     6   f   0         27     243      30     733      2.71 (  1.58-   4.66)
 Subtotal HO                                              2.34 (  1.62-   3.38)
 HOZAWA 3   b   0       2589    5379     872    5019      2.77 (  2.54-   3.02)
 HUHTI1 3   m   0        200     331       7     115      9.93 (  4.54-  21.72)
 HUHTI1 199 f   0         21      93      56     653      2.63 (  1.52-   4.55)
 Subtotal HUHTI1                                          4.07 (  2.60-   6.37)
 HUHTI3 3   m   0         84     726       3     236      9.10 (  2.85-  29.06)
 ITABAS 1   b   0         44      35      16      25      1.96 (  0.91-   4.24)
*JACOBS 3   m   2        257       -      28       -      3.86 (  2.45-   6.09)
 JOHANN 3   b   0        134    1237      22     842      4.15 (  2.62-   6.56)
 KACHEL 3   b   0         41     688       7     503      4.28 (  1.91-   9.62)
*KAHN2  132 m   1        476       -      31       -      8.49 (  5.87-  12.27)
 KARAKA 3   b   0         59      87      25      81      2.20 (  1.26-   3.84)
 KATANC 3   b   0         54     403      21     382      2.44 (  1.44-   4.11)
 KIM    3   m   1        179       -      23       -      2.31 (  1.44-   3.72)
 KIM    6   f   1         10       -      61       -      1.74 (  0.85-   3.57)
 Subtotal KIM                                             2.12 (  1.43-   3.15)
 KIRAZ  1   f   0          9      32      25     278      3.13 (  1.34-   7.28)
 KLAYTO 1   b   0         62     117       7      76      5.75 (  2.50-  13.24)
 KOJIMA 3   m   0        169    5515      19    1871      3.02 (  1.87-   4.86)
 KOJIMA 6   f   0          5     316      20    3545      2.80 (  1.05-   7.52)
 Subtotal KOJIMA                                          2.98 (  1.94-   4.57)
  ________________________________________________________________________________________________________________________
                                            International Evidence on Smoking and COPD, Phase 3, Analysis run on 27-SEP-10

                                                   Table 1 - A - 2 - 5

            IESCOPD - Meta-analysis of ever smoking, cigarettes (or all products if cigarettes not available)
                                                         Any COPD
                                                      Least-adjusted


                        Number Exposed  Non-exposed
 REF    NRR SEX ADJ     Case    Cont    Case    Cont      RR        95.00%CI
*KRZYZA 3   m   1         57       -       5       -      2.63 (  1.09-   6.38)
*KRZYZA 6   f   1         19       -      32       -      1.41 (  0.82-   2.41)
 Subtotal KRZYZA                                          1.67 (  1.05-   2.65)
 LAI    1   m   0        107     469      20     493      5.62 (  3.43-   9.22)
 LAI    2   f   0          7      88      27     600      1.77 (  0.75-   4.18)
 Subtotal LAI                                             4.22 (  2.75-   6.48)
*LAM1   1   m   7         19       -       4       -      4.05 (  1.31-  12.50)
*LAM1   2   f   7          2       -       2       -     26.61 (  2.08- 339.00)
 Subtotal LAM1                                            5.51 (  1.97-  15.47)
 LAM2   3   m   0        246     634      49     339      2.68 (  1.92-   3.75)
*LAM3   3   m   7         29       -       9       -      1.81 (  0.83-   3.98)
 LEBOWI 3   m   0         41     298       4     172      5.92 (  2.08-  16.80)
 LEBOWI 15  f   0         50     666      36     766      1.60 (  1.03-   2.48)
 Subtotal LEBOWI                                          1.95 (  1.30-   2.92)
*LEE    7   m   0         38    2498       1     347      5.28 (  0.73-  38.32)
*LEE    42  f   0          8    1855       5    1694      1.46 (  0.48-   4.46)
 Subtotal LEE                                             1.99 (  0.75-   5.26)
*LINDBE 3   b   3          -       -       -       -      2.28 (  1.34-   3.88)
 LINDST 5   m   0        170    3624      69    2984      2.03 (  1.53-   2.69)
 LINDST 6   f   0        145    2969      97    3670      1.85 (  1.42-   2.40)
 Subtotal LINDST                                          1.93 (  1.59-   2.34)
 LUNDB1 3   m   0         80     347      14     163      2.68 (  1.48-   4.88)
 LUNDB1 6   f   0         58     254      25     295      2.69 (  1.64-   4.43)
 Subtotal LUNDB1                                          2.69 (  1.84-   3.94)
 MADOR  3   m   0         19       7       0       3     18.20~(  0.84- 396.21)
 MANNI1 7   m   2        458       -     127       -      2.45 (  2.00-   3.00)
 MANNI1 10  f   2        305       -     156       -      3.18 (  2.61-   3.88)
 Subtotal MANNI1                                          2.80 (  2.43-   3.23)
 MANNI2 3   b   1        752       -     202       -      2.64 (  2.24-   3.12)
*MANNI3 3   b   0         76    2724      36    1895      1.47 (  0.99-   2.17)
 MARAN1 3   b   0        166     968      54    1905      6.05 (  4.41-   8.30)
*MARAN2 3   b   0         50     605      20    1324      5.47 (  3.29-   9.11)
*MARCUS 3   m   1         75       -      14       -      2.17 (  1.20-   3.90)
 MATHES 3   b   0         49      89      23     131      3.14 (  1.78-   5.51)
 MENEZ2 3   b   0        100       -      52       -      1.56 (  1.08-   2.24)
 MENEZ3 3   b   0        136       -      62       -      1.11 (  0.80-   1.54)
 MENEZ4 3   b   0         44       -      34       -      1.65 (  1.04-   2.63)
 MENEZ5 3   b   0        115       -      58       -      1.64 (  1.16-   2.33)
 MENEZ6 3   b   0        121       -      36       -      2.73 (  1.85-   4.03)
 MONTNE 3   b   0        301    4686      91    3390      2.39 (  1.88-   3.04)
 MUELLE 3   m   0         35     187       2      57      5.33 (  1.24-  22.87)
 MUELLE 11  f   0          3     154       2     169      1.65 (  0.27-   9.98)
 Subtotal MUELLE                                          3.35 (  1.08-  10.41)
 NIEPSU 3   b   0         34     191       8     177      3.94 (  1.78-   8.74)
*NIHLEN 3   b   0         91    2465      21    1815      3.19 (  1.99-   5.11)
*NILSSO 3   m   2        124       -      31       -      3.86 (  2.58-   5.77)
*NILSSO 6   f   2         63       -      57       -      5.93 (  4.02-   8.75)
 Subtotal NILSSO                                          4.82 (  3.64-   6.37)
 PEREZP 1   f   0         19      47      45     145      1.30 (  0.69-   2.44)
*PETO   3   m   0        104    2423       0     295     25.48~(  1.59- 409.05)
 RENWIC 3   b   0         59     119       9      60      3.31 (  1.54-   7.12)
 RICCIO 3   b   0        321     478     178     537      2.03 (  1.62-   2.53)
 SARGEA 3   m   0        585    1842     218    1051      1.53 (  1.29-   1.82)
 SARGEA 6   f   0        383    1354     404    2086      1.46 (  1.25-   1.71)
 Subtotal SARGEA                                          1.49 (  1.33-   1.67)
 SHAHAB 3   b   0        771    3758     322    3364      2.14 (  1.87-   2.46)
 SHIN   3   m   0         68     285      14     116      1.98 (  1.07-   3.65)
 SHIN   4   f   0          3      25      35     614      2.11 (  0.61-   7.31)
 Subtotal SHIN                                            2.00 (  1.15-   3.47)
 SICHLE 1   b   3          -       -       -       -      2.17 (  1.63-   2.91)
*SILVA  3   b   6          -       -       -       -      1.59 (  0.96-   2.63)
*SPEIZE 3   m   1         26       -       0       -     11.40 (  0.69- 187.05)
*SPEIZE 6   f   1         14       -       5       -      4.20 (  1.51-  11.66)
 Subtotal SPEIZE                                          4.72 (  1.81-  12.34)
 STROM  3   m   0        157     233      17      71      2.81 (  1.60-   4.96)
*TANG   4   m   2          -       -       -       -      4.12 (  2.33-   7.28)
*THUN   5   m   1          -       -       -       -      8.96 (  6.66-  12.06)
*THUN   6   f   1          -       -       -       -      8.05 (  6.17-  10.50)
 Subtotal THUN                                            8.44 (  6.93-  10.29)
*TODD   7   m   0         51    3543       1     520      7.49 (  1.04-  54.05)
*TODD   42  f   0          1    2203       4    2120      0.24 (  0.03-   2.15)
  ________________________________________________________________________________________________________________________
                                            International Evidence on Smoking and COPD, Phase 3, Analysis run on 27-SEP-10

                                                   Table 1 - A - 2 - 5

            IESCOPD - Meta-analysis of ever smoking, cigarettes (or all products if cigarettes not available)
                                                         Any COPD
                                                      Least-adjusted


                        Number Exposed  Non-exposed
 REF    NRR SEX ADJ     Case    Cont    Case    Cont      RR        95.00%CI
 Subtotal TODD                                            1.60 (  0.37-   6.94)
 TRUPIN 3   b   0        304     943      73     741      3.27 (  2.49-   4.30)
 TSUSHI 3   b   0         41    1142       7    1056      5.42 (  2.42-  12.12)
*TVERDA 3   m   2         55       -       7       -      2.88 (  1.31-   6.33)
 VESTBO 3   b   0       1667    8841     234    2366      1.91 (  1.65-   2.20)
 VIEGI2 3   m   0        177     533      21     118      1.87 (  1.14-   3.06)
 VIEGI2 9   f   0         52     370      66     390      0.83 (  0.56-   1.23)
 Subtotal VIEGI2                                          1.13 (  0.83-   1.54)
 VONHER 3   m   0        655    1687      63     911      5.61 (  4.28-   7.36)
 VONHER 6   f   0        132     734     137    2888      3.79 (  2.95-   4.88)
 Subtotal VONHER                                          4.55 (  3.78-   5.47)
 WEISS  3   m   0         42     209       2      34      3.42 (  0.79-  14.77)
 WILSO1 1   b   0        112    1040      24     875      3.93 (  2.50-   6.16)
 XIAO   3   b   0         79      44      21      56      4.79 (  2.57-   8.92)
 XU     1   m   0        596    7945     450    5584      0.93 (  0.82-   1.06)
 XU     3   f   0         51     273     647   13773      3.98 (  2.92-   5.42)
 Subtotal XU                                              1.15 (  1.02-   1.29)
 YAMAGU 3   b   6        332       -     144       -      1.97 (  1.60-   2.43)
*YUAN   3   m   1         34       -      19       -      1.33 (  0.76-   2.34)
 ZIELI1 1   b   0       2364    6446     316    1884      2.19 (  1.92-   2.49)
 ZIELI2 3   m   0      11618   36365     341    2611      2.45 (  2.18-   2.74)
 ZIELI2 6   f   0       5159   26196     679    4832      1.40 (  1.29-   1.53)
 Subtotal ZIELI2                                          1.71 (  1.60-   1.83)
 ZIETKO 2   m   0         35      11       0      12     77.17~(  4.23-1408.01)
 ZIETKO 4   f   0         12       6       0      11     44.23~(  2.23- 875.91)
 Subtotal ZIETKO                                         58.88 (  7.34- 472.11)
Partial Totals         41468  179602    8603  105325
*prospective study                                        ~ With 0.5 adjustment for zero


 REF    NRR SEX ADJ             Ys       Ws       Qs       Ps
 ALESSA 3   b   0              0.73     3.80     0.04       0.16
 AMIGO  1   m   1              1.91     1.71     1.98       0.01
 AMIGO  2   f   1              1.97     3.33     4.30       0.00
 Subtotal AMIGO                1.95     5.04     6.28
 ANDER1 3   m   1              1.64     1.70     1.10       0.03
 ANDER1 6   f   1              0.17     5.96     2.64       0.69
 Subtotal ANDER1               0.49     7.66     3.74
 ANDER3 3   m   0              3.43     0.43     2.95       0.02
 ANDER3 6   f   0              0.69     0.46     0.01       0.64
 Subtotal ANDER3               2.02     0.90     2.96
 BEDNAR 3   b   0              0.46    13.11     1.85       0.10
*BEST   37  m   1              2.30     5.73    12.46       0.00
 CHEN2  3   m   0              1.50     6.80     3.00       0.00
 CHEN2  6   f   0              1.27    23.78     4.61       0.00
 Subtotal CHEN2                1.32    30.58     7.61
 CLEMEN 3   m   0              0.81     9.05     0.00       0.01
 COCCI  2   b   0              2.98     0.44     2.03       0.05
 DEAN1  6   m   0              1.04    36.78     1.57       0.00
 DEAN1  9   f   0             -0.12    50.27    45.68       0.38
 Subtotal DEAN1                0.37    87.05    47.25
 DEJONG 3   b   0              3.21     0.49     2.76       0.03
 DEMARC 3   b   0              0.62   107.30     4.92       0.00
 DICKIN 3   b   0              3.24     0.96     5.55       0.00
*DOLL1  6   m   2              2.14    11.82    20.20       0.00
*DOLL2  3   f   1              2.40     0.92     2.27       0.02
 EKBERG 3   m   0              0.74   289.16     2.20       0.00
 EKBERG 6   f   0              0.94   117.01     1.53       0.00
 Subtotal EKBERG               0.80   406.17     3.73
 FERRI2 7   m   1              1.48    13.70     5.81       0.00
 FERRI2 14  f   1              0.84    27.67     0.00       0.00
 Subtotal FERRI2               1.05    41.36     5.81
 FIDAN  1   m   0              1.79     1.78     1.63       0.02
 FORAST 3   f   0              0.80    17.88     0.02       0.00
 FUKUCH 3   b   0              1.09    43.96     2.96       0.00
*GODTFR 3   b   3              1.70    55.75    41.90       0.00
 GULSVI 1   m   0              2.92     0.96     4.21       0.00
 GULSVI 2   f   0              1.26     9.79     1.79       0.00
 Subtotal GULSVI               1.41    10.75     6.01
*HAMMO2 5   m   1              2.23    38.64    75.34       0.00
*HAMMO2 6   f   1              1.77    32.89    29.02       0.00
  ________________________________________________________________________________________________________________________
                                            International Evidence on Smoking and COPD, Phase 3, Analysis run on 27-SEP-10

                                                   Table 1 - A - 2 - 5

            IESCOPD - Meta-analysis of ever smoking, cigarettes (or all products if cigarettes not available)
                                                         Any COPD
                                                      Least-adjusted


 REF    NRR SEX ADJ             Ys       Ws       Qs       Ps
 Subtotal HAMMO2               2.02    71.53   104.36
 HARDIE 3   m   1              1.69     6.71     4.98       0.00
 HARDIE 6   f   1              1.01    17.50     0.57       0.00
 Subtotal HARDIE               1.20    24.21     5.55
*HARIKK 3   m   0              0.72     6.14     0.08       0.07
*HARIKK 6   f   0              2.81     0.47     1.86       0.05
 Subtotal HARIKK               0.87     6.61     1.93
*HIGGI4 3   m   1              1.12    11.30     0.98       0.00
 HO     3   m   0              0.72    15.02     0.18       0.01
 HO     6   f   0              1.00    13.18     0.37       0.00
 Subtotal HO                   0.85    28.21     0.56
 HOZAWA 3   b   0              1.02   521.32    18.52       0.00
 HUHTI1 3   m   0              2.30     6.27    13.44       0.00
 HUHTI1 199 f   0              0.97    12.86     0.24       0.00
 Subtotal HUHTI1               1.40    19.13    13.69
 HUHTI3 3   m   0              2.21     2.85     5.41       0.00
 ITABAS 1   b   0              0.68     6.50     0.16       0.09
*JACOBS 3   m   2              1.35    18.53     5.01       0.00
 JOHANN 3   b   0              1.42    18.21     6.37       0.00
 KACHEL 3   b   0              1.45     5.86     2.28       0.00
*KAHN2  132 m   1              2.14    28.27    48.39       0.00
 KARAKA 3   b   0              0.79    12.38     0.02       0.01
 KATANC 3   b   0              0.89    14.04     0.05       0.00
 KIM    3   m   1              0.84    17.06     0.00       0.00
 KIM    6   f   1              0.55     7.46     0.57       0.13
 Subtotal KIM                  0.75    24.52     0.57
 KIRAZ  1   f   0              1.14     5.38     0.52       0.01
 KLAYTO 1   b   0              1.75     5.53     4.68       0.00
 KOJIMA 3   m   0              1.10    16.87     1.27       0.00
 KOJIMA 6   f   0              1.03     3.95     0.16       0.04
 Subtotal KOJIMA               1.09    20.82     1.43
*KRZYZA 3   m   1              0.97     4.92     0.09       0.03
*KRZYZA 6   f   1              0.34    13.22     3.13       0.21
 Subtotal KRZYZA               0.51    18.14     3.23
 LAI    1   m   0              1.73    15.75    12.66       0.00
 LAI    2   f   0              0.57     5.18     0.35       0.19
 Subtotal LAI                  1.44    20.93    13.01
*LAM1   1   m   7              1.40     3.02     0.98       0.02
*LAM1   2   f   7              3.28     0.59     3.56       0.01
 Subtotal LAM1                 1.71     3.61     4.53
 LAM2   3   m   0              0.99    34.48     0.85       0.00
*LAM3   3   m   7              0.59     6.25     0.35       0.14
 LEBOWI 3   m   0              1.78     3.53     3.16       0.00
 LEBOWI 15  f   0              0.47    19.77     2.59       0.04
 Subtotal LEBOWI               0.67    23.30     5.76
*LEE    7   m   0              1.66     0.98     0.68       0.10
*LEE    42  f   0              0.38     3.09     0.63       0.51
 Subtotal LEE                  0.69     4.07     1.31
*LINDBE 3   b   3              0.82    13.59     0.00       0.00
 LINDST 5   m   0              0.71    47.65     0.72       0.00
 LINDST 6   f   0              0.61    56.13     2.63       0.00
 Subtotal LINDST               0.66   103.78     3.35
 LUNDB1 3   m   0              0.99    10.76     0.26       0.00
 LUNDB1 6   f   0              0.99    15.49     0.40       0.00
 Subtotal LUNDB1               0.99    26.25     0.66
 MADOR  3   m   0              2.90     0.40     1.74       0.06
 MANNI1 7   m   2              0.90    93.46     0.40       0.00
 MANNI1 10  f   2              1.16    97.75    10.41       0.00
 Subtotal MANNI1               1.03   191.21    10.81
 MANNI2 3   b   1              0.97   139.95     2.75       0.00
*MANNI3 3   b   0              0.38    24.97     4.97       0.05
 MARAN1 3   b   0              1.80    38.31    36.01       0.00
*MARAN2 3   b   0              1.70    14.79    11.17       0.00
*MARCUS 3   m   1              0.77    11.06     0.03       0.01
 MATHES 3   b   0              1.14    12.08     1.18       0.00
 MENEZ2 3   b   0              0.44    28.87     4.30       0.02
 MENEZ3 3   b   0              0.10    35.82    18.89       0.53
 MENEZ4 3   b   0              0.50    17.85     1.94       0.03
 MENEZ5 3   b   0              0.49    31.59     3.56       0.01
 MENEZ6 3   b   0              1.00    25.35     0.77       0.00
 MONTNE 3   b   0              0.87    67.48     0.12       0.00
  ________________________________________________________________________________________________________________________
                                            International Evidence on Smoking and COPD, Phase 3, Analysis run on 27-SEP-10

                                                   Table 1 - A - 2 - 5

            IESCOPD - Meta-analysis of ever smoking, cigarettes (or all products if cigarettes not available)
                                                         Any COPD
                                                      Least-adjusted


 REF    NRR SEX ADJ             Ys       Ws       Qs       Ps
 MUELLE 3   m   0              1.67     1.81     1.29       0.02
 MUELLE 11  f   0              0.50     1.18     0.13       0.59
 Subtotal MUELLE               1.21     3.00     1.42
 NIEPSU 3   b   0              1.37     6.05     1.77       0.00
*NIHLEN 3   b   0              1.16    17.35     1.89       0.00
*NILSSO 3   m   2              1.35    23.72     6.42       0.00
*NILSSO 6   f   2              1.78    25.40    22.90       0.00
 Subtotal NILSSO               1.57    49.12    29.32
 PEREZP 1   f   0              0.26     9.71     3.11       0.41
*PETO   3   m   0              3.24     0.50     2.89       0.02
 RENWIC 3   b   0              1.20     6.53     0.87       0.00
 RICCIO 3   b   0              0.71    78.82     1.22       0.00
 SARGEA 3   m   0              0.43   128.35    21.00       0.00
 SARGEA 6   f   0              0.38   158.63    32.36       0.00
 Subtotal SARGEA               0.40   286.98    53.37
 SHAHAB 3   b   0              0.76   201.37     0.93       0.00
 SHIN   3   m   0              0.68    10.18     0.23       0.03
 SHIN   4   f   0              0.74     2.48     0.02       0.24
 Subtotal SHIN                 0.69    12.65     0.24
 SICHLE 1   b   3              0.77    45.74     0.14       0.00
*SILVA  3   b   6              0.46    15.13     2.04       0.07
*SPEIZE 3   m   1              2.43     0.49     1.26       0.09
*SPEIZE 6   f   1              1.44     3.68     1.34       0.01
 Subtotal SPEIZE               1.55     4.17     2.60
 STROM  3   m   0              1.03    11.97     0.50       0.00
*TANG   4   m   2              1.42    11.84     4.06       0.00
*THUN   5   m   1              2.19    43.58    80.88       0.00
*THUN   6   f   1              2.09    54.36    85.64       0.00
 Subtotal THUN                 2.13    97.94   166.52
*TODD   7   m   0              2.01     0.98     1.37       0.05
*TODD   42  f   0             -1.42     0.80     4.07       0.20
 Subtotal TODD                 0.47     1.78     5.45
 TRUPIN 3   b   0              1.19    51.55     6.50       0.00
 TSUSHI 3   b   0              1.69     5.91     4.36       0.00
*TVERDA 3   m   2              1.06     6.19     0.32       0.01
 VESTBO 3   b   0              0.65   184.87     6.34       0.00
 VIEGI2 3   m   0              0.62    15.72     0.67       0.01
 VIEGI2 9   f   0             -0.19    25.22    26.05       0.35
 Subtotal VIEGI2               0.13    40.94    26.72
 VONHER 3   m   0              1.73    52.38    41.94       0.00
 VONHER 6   f   0              1.33    60.30    15.20       0.00
 Subtotal VONHER               1.52   112.68    57.15
 WEISS  3   m   0              1.23     1.79     0.28       0.10
 WILSO1 1   b   0              1.37    18.98     5.48       0.00
 XIAO   3   b   0              1.57     9.91     5.36       0.00
 XU     1   m   0             -0.07   237.81   193.55       0.27
 XU     3   f   0              1.38    40.18    12.15       0.00
 Subtotal XU                   0.14   277.99   205.70
 YAMAGU 3   b   6              0.68    87.99     2.05       0.00
*YUAN   3   m   1              0.29    12.15     3.61       0.32
 ZIELI1 1   b   0              0.78   234.00     0.54       0.00
 ZIELI2 3   m   0              0.89   291.62     1.20       0.00
 ZIELI2 6   f   0              0.34   523.09   127.12       0.00
 Subtotal ZIELI2               0.54   814.71   128.32
 ZIETKO 2   m   0              4.35     0.46     5.63       0.00
 ZIETKO 4   f   0              3.79     0.43     3.77       0.01
 Subtotal ZIETKO               4.08     0.89     9.40


  ________________________________________________________________________________________________________________________
                                            International Evidence on Smoking and COPD, Phase 3, Analysis run on 27-SEP-10

                                                   Table 1 - A - 2 - 5

            IESCOPD - Meta-analysis of ever smoking, cigarettes (or all products if cigarettes not available)
                                                         Any COPD
                                                      Least-adjusted


                       N      133
                      NS       98


                      Wt  5323.08
                 Het Chi  1274.20
                 Het  df      132
                 Het  P       ***
               Fixed  RR     2.29
                     RRl     2.23
                     RRu     2.36
                      P       +++
              Random  RR     2.89
                     RRl     2.62
                     RRu     3.19
                      P       +++
               Asymm  P       ***


  ________________________________________________________________________________________________________________________
                                            International Evidence on Smoking and COPD, Phase 3, Analysis run on 27-SEP-10

                                                   Table 1 - A - 2 - 6

            IESCOPD - Meta-analysis of ever smoking, cigarettes (or all products if cigarettes not available)
                                                         Any COPD
                                                      Least-adjusted


                       N      133
                      NS       98


                      Wt  5323.08
                 Het Chi  1274.20
                 Het  df      132
                 Het  P       ***
               Fixed  RR     2.29
                     RRl     2.23
                     RRu     2.36
                      P       +++
              Random  RR     2.89
                     RRl     2.62
                     RRu     3.19
                      P       +++
               Asymm  P       ***

                                   Sex
                             both      male    female     Total


                       N       41        53        39       133
                      NS       41        53        39       133


                      Wt  2224.52   1631.11   1467.45   5323.08
                 Het Chi   211.60    599.25    429.88   1274.20
                 Het  df       40        52        38       132
                 Het  P       ***       ***       ***       ***
               Fixed  RR     2.43      2.38      2.02      2.29
                     RRl     2.33      2.26      1.92      2.23
                     RRu     2.54      2.49      2.13      2.36
                      P       +++       +++       +++       +++
              Random  RR     2.58      3.61      2.51      2.89
                     RRl     2.30      2.96      2.03      2.62
                     RRu     2.90      4.40      3.11      3.19
                      P       +++       +++       +++       +++
             Between Chi                                  33.48
             Between  df                                      2
             Between  P                                     ***
             Btwn(F)  P                                    N.S.


  ________________________________________________________________________________________________________________________
                                            International Evidence on Smoking and COPD, Phase 3, Analysis run on 27-SEP-10

                                                   Table 1 - A - 2 - 7

            IESCOPD - Meta-analysis of ever smoking, cigarettes (or all products if cigarettes not available)
                                                         Any COPD
                                 Excluded studies (and stage at which they were excluded)


1       CLARK COTTON  MEYER REMYJA RUTGER SNYDER SOBRAX     SU TAKEMU  WANG4   WEIR WHICKE ZALACA
2      ALDERS ANDER2 AUERBA   BANG  BECK1  BECK2 BJORNS  BROWN CERVER CHAPMA COATES COLLEG  DEAN2  DEANE DONTA2 DOPICO
       EHRLIC ENRIGH FINKLE FLETCH FOXMAN GOLDBE HAENSZ HARRIS  HAYES HIGGI2 HIGGI3 HIGGI6 HIRAYA HOLLA2 HOLLNA  HOUSE
       HRUBEC HUCHON JENSEN JINDA2  JOSHI JOUSI1   KATO KOTAN1  KUBIK LAMBER LANGE2 LANGHA LAVECC LUNDB2 MAGNUS MANFRE
       MELLST MENEZ1  MEREN MILLER  MILNE MOLLER   NAWA NEJJAR OGILVI  OMORI OSWAL1 OSWAL2 PANDEY  PRATT   REID RIMING
        RYDER SCHWAR  SHARP SHIMUR SOBRAD STJERN SUADIC SUTINE TAGER2 TROISI URRUTI VIEGI1 VIKGRE WAGEN2  WANG2    WIG
       WILHEL WILSO2  WOODS  WOOLF   ZOIA
3      BROGGE  CHEN1  CHENG DETORR GEIJER HEDMAN JAENDI KHOURY KOTAN2 KULLER   LIU1   LIU2  PRICE  TAGER VINEIS WATSON
4       CHEN3   KAHN   LIAW   PEAT SAWICK STERLI VOLLM1 VOLLM2   WALD    WEN WOJTYN
8      DONTA1 ENSTRO FERRI1 FERRI3 HAWTHO HUHTI2  LANGE PELKON


  ________________________________________________________________________________________________________________________
                                            International Evidence on Smoking and COPD, Phase 3, Analysis run on 27-SEP-10

                                                   Table 1 - A - 2 - 8

            IESCOPD - Meta-analysis of ever smoking, cigarettes (or all products if cigarettes not available)
                                                         Any COPD
                                             Potentially overlapping studies


     REF| REFGP|PRINC|                     OVERLAP|

  DEMARC DEMARC     1         DEMARC/URRUTI/DEMEER
  MONTNE MONTNE     1   EKBERG/STROM/NIHLEN/MONTNE
  EKBERG EKBERG     1   EKBERG/STROM/NIHLEN/MONTNE
  NIHLEN NIHLEN     1   EKBERG/STROM/NIHLEN/MONTNE
   STROM  STROM     1   EKBERG/STROM/NIHLEN/MONTNE
  HARIKK HARIKK     1         ENRIGH/HOZAWA/HARIKK
  HOZAWA HOZAWA     1         ENRIGH/HOZAWA/HARIKK
  JACOBS JACOBS     1  JACOBS/DONTA1/DONTA2/PELKON
  LUNDB1 LUNDBA     1  LINDBE/LUNDB1/LUNDB2/HEDLUN
  LINDBE LINDBE     1  LINDBE/LUNDB1/LUNDB2/HEDLUN
    PETO   PETO     1    PETO/HIGGI1/HIGGI2/HIGGI5
    TANG   TANG     1             TANG/HAWTHO/WALD
  KARAKA VINEIS     2         VINEIS/KARAKA/SARGEA
  SARGEA VINEIS     2         VINEIS/KARAKA/SARGEA
  HUHTI1 HUHTI1     1                HUHTI1/HUHTI2
  HARDIE HARDIE     1         HARDIE/JOHANN/BROGGE
  JOHANN JOHANN     1         HARDIE/JOHANN/BROGGE
  MARAN1 MARAN1     1                MARAN1/MARAN2
  MARAN2 MARAN2     1                MARAN1/MARAN2
    LAM3   LAM3     1                    LAM2/LAM3
    LAM2   LAM2     1                    LAM2/LAM3
  HAMMO2 HAMMO2     1                HAMMO2/ENSTRO
  LEBOWI LEBOWI     1                 LEBOWI/SILVA
   SILVA  SILVA     1                 LEBOWI/SILVA
  FERRI2 FERRIS     1         FERRI1/FERRI2/FERRI3
  MANNI2 MANNI2     1                MANNI2/MANNI3
  MANNI3 MANNI3     1                MANNI2/MANNI3
  WILSO1 WILSO1     1                WILSO1/WILSO2
  MENEZ3 MENEZ3     1                 AMIGO/MENEZ3
   AMIGO  AMIGO     1                 AMIGO/MENEZ3
    TODD   TODD     1                  LAMBER/TODD
  KRZYZA KRZYZA     1         SAWICK/KRZYZA/WOJTYN
  GODTFR GODTFR     1 GODT/VEST/LANG1+2/SUAD/HOLLN
  VESTBO VESTBO     1 GODT/VEST/LANG1+2/SUAD/HOLLN
  HIGGI4 HIGGI4     1                HIGGI4/HIGGI6
   KAHN2   KAHN     2                   KAHN/KAHN2

                                    Most-adjusted - insufficient data for meta-analysis
     REF|NRR|SEX|AGEL|AGEH|     REGION|BEGYR|PUBYR|STTYP|ONSET|      DISEAS|ADJ|SMOKSTA|   PRODUCT|    UNEXP|        RR|

   STROM   6   m   68   68    Eu:Scand  1982  1996    CS  Prev      COPD:LF   8    Ever        Any   Nev any          *
  SIG|

    y


  ________________________________________________________________________________________________________________________
                                            International Evidence on Smoking and COPD, Phase 3, Analysis run on 27-SEP-10

                                                    Table 1 - A - 3 -

            IESCOPD - Meta-analysis of ever smoking, any product (or cigarettes if all product not available)
                                                 COPD based on mortality


This analysis is restricted to results for:
1) Eligible study on database
2) Outcome COPD
3) Non-dose-response data
4) Ever smoking
5) COPD based on mortality
6) Results complete enough for use in meta-analysis

Within each study, results are then selected (in the following order of preference, within each sex) for:
7) UNEXP   : never any, never cigarettes, other
8) PROD    : any product, cigarettes, cigarettes only
9) For overlapping studies: principal rather than subsidiary studies
and then for single sex results (m, f) in preference to results for both sexes combined (b).

Results adjusted for the most potential confounders are then chosen in Sections -1 to -3
and results adjusted for the least confounders in Sections -4 to -6. (Those least adjusted results which
actually differ from the most adjusted are marked 'x' in column X in Section -4)

Section -7 shows excluded studies, together with the stage (as above) at which no qualifying
results were found.

Section -8 lists the potentially overlapping studies which have been included (1=principal, 2=subsidiary),
and any results which would have been included in preference except that they had data not complete enough
for use in meta-analysis. It also lists their significance (yes/no), if known.


  ________________________________________________________________________________________________________________________
                                            International Evidence on Smoking and COPD, Phase 3, Analysis run on 27-SEP-10

                                                   Table 1 - A - 3 - 1

            IESCOPD - Meta-analysis of ever smoking, any product (or cigarettes if all product not available)
                                                 COPD based on mortality
                                                      Most-adjusted


     REF|NRR|SEX|AGEL|AGEH|     REGION|BEGYR|PUBYR|STTYP|ONSET|      DISEAS|ADJ|SMOKSTA|   PRODUCT|    UNEXP|

    BEST  37   m   30   97   Am:Canada  1955  1967    Pr   Inc    COPD:mort   1    Ever  Cigs only   Nev any
   DEAN1  13   m   35   99       Eu:UK  1969  1977    CC  Prev    COPD:mort   3    Ever        Any   Nev any
   DEAN1  17   f   35   99       Eu:UK  1969  1977    CC  Prev    COPD:mort   3    Ever MCigs only   Nev any
   DOLL1   3   m   20   99       Eu:UK  1951  1994    Pr   Inc    COPD:mort   2    Ever        Any   Nev any
   DOLL2   3   f   20   99       Eu:UK  1951  1980    Pr   Inc    COPD:mort   1    Ever  Cigs only   Nev any
  HAMMO2   5   m   35   99      Am:USA  1959  1966    Pr   Inc    COPD:mort   1    Ever  Cigs only   Nev any
  HAMMO2   6   f   35   99      Am:USA  1959  1966    Pr   Inc    COPD:mort   1    Ever  Cigs only   Nev any
  HIGGI4   3   m   35   99      Am:USA  1962  1989    Pr   Inc    COPD:mort   1    Ever       Cigs  Nev cigs
  JACOBS   6   m   40   84       Multi  1957  1999    Pr   Inc    COPD:mort   6    Ever       Cigs  Nev cigs
   KAHN2 131   m   31   84      Am:USA  1954  1966    Pr   Inc    COPD:mort   1    Ever        Any   Nev any
    LAM1   1   m   35   99   Asia:FarE  1976  1997    Pr   Inc    COPD:mort   7    Ever       Cigs  Nev cigs
    LAM1   2   f   35   99   Asia:FarE  1976  1997    Pr   Inc    COPD:mort   7    Ever       Cigs  Nev cigs
    LAM3   3   m   60   99   Asia:FarE  1987  2006    Pr   Inc    COPD:mort   7    Ever       Cigs  Nev cigs
   LANGE   3   m   20   99    Eu:Scand  1976  1992    Pr   Inc    COPD:mort   1    Ever        Any   Nev any
   LANGE   6   f   20   99    Eu:Scand  1976  1992    Pr   Inc    COPD:mort   1    Ever        Any   Nev any
     LEE  20   m   35   82       Eu:UK  1964  1979    Pr   Inc    COPD:mort   1    Ever        Any   Nev any
     LEE  52   f   35   82       Eu:UK  1964  1979    Pr   Inc    COPD:mort   1    Ever        Any   Nev any
  MANNI3   3   b   25   96      Am:USA  1971  2003    Pr   Inc    COPD:mort   0    Ever        Any   Nev any
  MARCUS   3   m   45   85      Am:USA  1965  1989    Pr   Inc    COPD:mort   1    Ever        Any   Nev any
  NILSSO   9   m   18   99    Eu:Scand  1963  2001    Pr   Inc    COPD:mort   2    Ever        Any   Nev any
  NILSSO   6   f   18   99    Eu:Scand  1963  2001    Pr   Inc    COPD:mort   2    Ever  Cigs only   Nev any
    PETO   3   m   25   92       Eu:UK  1954  1983    Pr   Inc    COPD:mort   0    Ever       Cigs  Nev cigs
  SPEIZE   3   m   25   86      Am:USA  1974  1989    Pr   Inc    COPD:mort   1    Ever       Cigs  Nev cigs
  SPEIZE   6   f   25   86      Am:USA  1974  1989    Pr   Inc    COPD:mort   1    Ever       Cigs  Nev cigs
    TANG   4   m   35   99       Eu:UK  1967  1995    Pr   Inc    COPD:mort   2    Ever        Any   Nev any
    THUN   5   m   30   99      Am:USA  1982  1997    Pr   Inc    COPD:mort   1    Ever  Cigs only   Nev any
    THUN   6   f   30   99      Am:USA  1982  1997    Pr   Inc    COPD:mort   1    Ever  Cigs only   Nev any
    TODD  20   m   35   81       Eu:UK  1965  1978    Pr   Inc    COPD:mort   1    Ever        Any   Nev any
    TODD  46   f   35   81       Eu:UK  1965  1978    Pr   Inc    COPD:mort   1    Ever        Any   Nev any
  TVERDA   3   m   35   65    Eu:Scand  1972  1993    Pr   Inc    COPD:mort   2    Ever       Cigs  Nev cigs
    YUAN   5   m   45   71   Asia:FarE  1986  1996    Pr   Inc    COPD:mort   2    Ever       Cigs  Nev cigs


  ________________________________________________________________________________________________________________________
                                            International Evidence on Smoking and COPD, Phase 3, Analysis run on 27-SEP-10

                                                   Table 1 - A - 3 - 2

            IESCOPD - Meta-analysis of ever smoking, any product (or cigarettes if all product not available)
                                                 COPD based on mortality
                                                      Most-adjusted


                        Number Exposed  Non-exposed
 REF    NRR SEX ADJ     Case    Cont    Case    Cont      RR        95.00%CI
*BEST   37  m   1        129       -       6       -     10.02 (  4.42-  22.72)
 DEAN1  13  m   3        483       -      47       -      2.54 (  1.76-   3.66)
 DEAN1  17  f   3         98       -     120       -      1.92 (  1.37-   2.69)
 Subtotal DEAN1                                           2.18 (  1.70-   2.80)
*DOLL1  3   m   2        522       -      12       -      6.54 (  3.71-  11.53)
*DOLL2  3   f   1         12       -       1       -     11.02 (  1.43-  84.78)
*HAMMO2 5   m   1          -       -       -       -      9.27 (  6.76-  12.70)
*HAMMO2 6   f   1          -       -       -       -      5.87 (  4.17-   8.26)
 Subtotal HAMMO2                                          7.51 (  5.96-   9.47)
*HIGGI4 3   m   1          -       -       -       -      3.08 (  1.72-   5.52)
*JACOBS 6   m   6        248       -      28       -      3.08 (  2.03-   4.65)
*KAHN2  131 m   1        496       -      31       -      6.69 (  4.63-   9.66)
*LAM1   1   m   7         19       -       4       -      4.05 (  1.31-  12.50)
*LAM1   2   f   7          2       -       2       -     26.61 (  2.08- 339.00)
 Subtotal LAM1                                            5.51 (  1.97-  15.47)
*LAM3   3   m   7         29       -       9       -      1.81 (  0.83-   3.98)
*LANGE  3   m   1        111       -       3       -      4.50 (  1.45-  13.90)
*LANGE  6   f   1         56       -       3       -     12.90 (  3.04-  54.84)
 Subtotal LANGE                                           6.71 (  2.75-  16.34)
*LEE    20  m   1         40       -       1       -      4.73 (  0.65-  34.28)
*LEE    52  f   1         10       -       5       -      1.74 (  0.60-   5.09)
 Subtotal LEE                                             2.18 (  0.85-   5.59)
*MANNI3 3   b   0         76    2724      36    1895      1.47 (  0.99-   2.17)
*MARCUS 3   m   1         75       -      14       -      2.17 (  1.20-   3.90)
*NILSSO 9   m   2         64       -      13       -      2.78 (  1.50-   5.14)
*NILSSO 6   f   2         63       -      57       -      5.93 (  4.02-   8.75)
 Subtotal NILSSO                                          4.78 (  3.44-   6.64)
*PETO   3   m   0        104    2423       0     295     25.48~(  1.59- 409.05)
*SPEIZE 3   m   1         26       -       0       -     11.40 (  0.69- 187.05)
*SPEIZE 6   f   1         14       -       5       -      4.20 (  1.51-  11.66)
 Subtotal SPEIZE                                          4.72 (  1.81-  12.34)
*TANG   4   m   2          -       -       -       -      4.12 (  2.33-   7.28)
*THUN   5   m   1          -       -       -       -      8.96 (  6.66-  12.06)
*THUN   6   f   1          -       -       -       -      8.05 (  6.17-  10.50)
 Subtotal THUN                                            8.44 (  6.93-  10.29)
*TODD   20  m   1         59       -       1       -      6.88 (  0.96-  49.58)
*TODD   46  f   1          4       -       4       -      0.89 (  0.22-   3.57)
 Subtotal TODD                                            1.76 (  0.56-   5.49)
*TVERDA 3   m   2         55       -       7       -      2.88 (  1.31-   6.33)
*YUAN   5   m   2         34       -      19       -      1.40 (  0.80-   2.45)
Partial Totals          2829    5147     428    2190
*prospective study                                        ~ With 0.5 adjustment for zero

                       N       31
                      NS       22


                      Wt   441.12
                 Het Chi   189.82
                 Het  df       30
                 Het  P       ***
               Fixed  RR     4.52
                     RRl     4.11
                     RRu     4.96
                      P       +++
              Random  RR     4.06
                     RRl     3.10
                     RRu     5.31
                      P       +++
               Asymm  P      N.S.


  ________________________________________________________________________________________________________________________
                                            International Evidence on Smoking and COPD, Phase 3, Analysis run on 27-SEP-10

                                                   Table 1 - A - 3 - 3

            IESCOPD - Meta-analysis of ever smoking, any product (or cigarettes if all product not available)
                                                 COPD based on mortality
                                                      Most-adjusted


                       N       31
                      NS       22


                      Wt   441.12
                 Het Chi   189.82
                 Het  df       30
                 Het  P       ***
               Fixed  RR     4.52
                     RRl     4.11
                     RRu     4.96
                      P       +++
              Random  RR     4.06
                     RRl     3.10
                     RRu     5.31
                      P       +++
               Asymm  P      N.S.

                                   Sex
                             both      male    female     Total


                       N        1        20        10        31
                      NS        1        20        10        31


                      Wt    24.97    257.38    158.77    441.12
                 Het Chi     0.00     97.75     58.46    189.82
                 Het  df        0        19         9        30
                 Het  P      N.S.       ***       ***       ***
               Fixed  RR     1.47      4.75      4.97      4.52
                     RRl     0.99      4.20      4.25      4.11
                     RRu     2.17      5.36      5.81      4.96
                      P       (+)       +++       +++       +++
              Random  RR     1.47      4.14      4.49      4.06
                     RRl     0.99      3.02      2.73      3.10
                     RRu     2.17      5.68      7.40      5.31
                      P       (+)       +++       +++       +++
             Between Chi                                  33.62
             Between  df                                      2
             Between  P                                     ***
             Btwn(F)  P                                     (*)

                                        Continent
                            NAmer    Europe      Asia  oth/mult     Total


                       N       11        15         4         1        31
                      NS        8        10         3         1        22


                      Wt   255.12    141.50     22.13     22.37    441.12
                 Het Chi    87.48     39.73      7.01      0.00    189.82
                 Het  df       10        14         3         0        30
                 Het  P       ***       ***       (*)      N.S.       ***
               Fixed  RR     6.02      3.28      1.88      3.08      4.52
                     RRl     5.32      2.78      1.24      2.04      4.11
                     RRu     6.80      3.87      2.86      4.66      4.96
                      P       +++       +++        ++       +++       +++
              Random  RR     5.19      3.63      2.41      3.08      4.06
                     RRl     3.48      2.59      1.14      2.04      3.10
                     RRu     7.74      5.08      5.11      4.66      5.31
                      P       +++       +++         +       +++       +++
             Between Chi                                            55.59
             Between  df                                                3
             Between  P                                               ***
             Btwn(F)  P                                                 *


  ________________________________________________________________________________________________________________________
                                            International Evidence on Smoking and COPD, Phase 3, Analysis run on 27-SEP-10

                                                   Table 1 - A - 3 - 3

            IESCOPD - Meta-analysis of ever smoking, any product (or cigarettes if all product not available)
                                                 COPD based on mortality
                                                      Most-adjusted
                        National cigarette tobacco type (excluding mixed/unkown)
                          blended  virginia     Total


                       N       16        11        27
                      NS       11         8        19


                      Wt   318.32    100.66    418.99
                 Het Chi   103.88     32.95    164.97
                 Het  df       15        10        26
                 Het  P       ***       ***       ***
               Fixed  RR     5.47      2.98      4.73
                     RRl     4.90      2.45      4.30
                     RRu     6.11      3.63      5.21
                      P       +++       +++       +++
              Random  RR     4.63      3.70      4.31
                     RRl     3.36      2.36      3.27
                     RRu     6.37      5.79      5.68
                      P       +++       +++       +++
             Between Chi                        28.14
             Between  df                            1
             Between  P                           ***
             Btwn(F)  P                             *

                                        Start year of study
                            <1970   1970-79   1980-89   1990-99     2000+   unknown     Total


                       N       19         8         4                                      31
                      NS       14         5         3                                      22


                      Wt   280.87     43.79    116.46                                  441.12
                 Het Chi    91.41     19.21     46.10                                  189.82
                 Het  df       18         7         3                                      30
                 Het  P       ***        **       ***                                     ***
               Fixed  RR     4.31      2.38      6.43                                    4.52
                     RRl     3.83      1.77      5.36                                    4.11
                     RRu     4.84      3.20      7.71                                    4.96
                      P       +++       +++       +++                                     +++
              Random  RR     4.10      4.04      3.90                                    4.06
                     RRl     3.04      2.15      1.76                                    3.10
                     RRu     5.53      7.59      8.62                                    5.31
                      P       +++       +++       +++                                     +++
             Between Chi                                                                33.11
             Between  df                                                                    2
             Between  P                                                                   ***
             Btwn(F)  P                                                                   (*)

                                Publication year
                            <1980   1980-89   1990-99     2000+     Total


                       N       10         6        11         4        31
                      NS        6         5         8         3        22


                      Wt   175.40     27.95    171.01     66.76    441.12
                 Het Chi    72.91      6.27     55.54     25.89    189.82
                 Het  df        9         5        10         3        30
                 Het  P       ***      N.S.       ***       ***       ***
               Fixed  RR     4.54      3.10      5.76      2.81      4.52
                     RRl     3.91      2.14      4.96      2.21      4.11
                     RRu     5.26      4.48      6.69      3.57      4.96
                      P       +++       +++       +++       +++       +++
              Random  RR     4.11      3.35      4.81      2.61      4.06
                     RRl     2.52      2.09      3.17      1.24      3.10
                     RRu     6.72      5.36      7.29      5.52      5.31
                      P       +++       +++       +++         +       +++
             Between Chi                                            29.20
             Between  df                                                3
             Between  P                                               ***
             Btwn(F)  P                                              N.S.
  ________________________________________________________________________________________________________________________
                                            International Evidence on Smoking and COPD, Phase 3, Analysis run on 27-SEP-10

                                                   Table 1 - A - 3 - 3

            IESCOPD - Meta-analysis of ever smoking, any product (or cigarettes if all product not available)
                                                 COPD based on mortality
                                                      Most-adjusted
                               Study type
                               CC        Pr        CS     Total


                       N        2        29                  31
                      NS        1        21                  22


                      Wt    62.42    378.70              441.12
                 Het Chi     1.21    150.19              189.82
                 Het  df        1        28                  30
                 Het  P      N.S.       ***                 ***
               Fixed  RR     2.18      5.09                4.52
                     RRl     1.70      4.60                4.11
                     RRu     2.80      5.63                4.96
                      P       +++       +++                 +++
              Random  RR     2.19      4.31                4.06
                     RRl     1.66      3.28                3.10
                     RRu     2.88      5.65                5.31
                      P       +++       +++                 +++
             Between Chi                                  38.42
             Between  df                                      1
             Between  P                                     ***
             Btwn(F)  P                                       *

                                    Lowest age in RR
                        <25/unlim     25-39       40+   unknown     Total


                       N        6        21         4                  31
                      NS        4        14         4                  22


                      Wt    53.25    335.92     51.95              441.12
                 Het Chi     7.09    147.61      5.27              189.82
                 Het  df        5        20         3                  30
                 Het  P      N.S.       ***      N.S.                 ***
               Fixed  RR     5.36      4.90      2.23                4.52
                     RRl     4.10      4.41      1.70                4.11
                     RRu     7.02      5.46      2.92                4.96
                      P       +++       +++       +++                 +++
              Random  RR     5.34      4.36      2.12                4.06
                     RRl     3.71      3.10      1.46                3.10
                     RRu     7.69      6.14      3.09                5.31
                      P       +++       +++       +++                 +++
             Between Chi                                            29.85
             Between  df                                                2
             Between  P                                               ***
             Btwn(F)  P                                               (*)

                                         Highest age in RR
                              <65     65-74     75-84 85+/unlim   unknown     Total


                       N                  2         6        23                  31
                      NS                  2         4        16                  22


                      Wt              18.46     58.08    364.58              441.12
                 Het Chi               2.14     15.92    154.47              189.82
                 Het  df                  1         5        22                  30
                 Het  P                N.S.        **       ***                 ***
               Fixed  RR               1.78      4.26      4.78                4.52
                     RRl               1.13      3.30      4.31                4.11
                     RRu               2.81      5.51      5.29                4.96
                      P                   +       +++       +++                 +++
              Random  RR               1.90      3.31      4.58                4.06
                     RRl               0.94      1.78      3.35                3.10
                     RRu               3.82      6.13      6.24                5.31
                      P                 (+)       +++       +++                 +++
             Between Chi                                                      17.29
             Between  df                                                          2
             Between  P                                                         ***
             Btwn(F)  P                                                        N.S.
  ________________________________________________________________________________________________________________________
                                            International Evidence on Smoking and COPD, Phase 3, Analysis run on 27-SEP-10

                                                   Table 1 - A - 3 - 3

            IESCOPD - Meta-analysis of ever smoking, any product (or cigarettes if all product not available)
                                                 COPD based on mortality
                                                      Most-adjusted
                           Study weakness
                              Yes        No     Total


                       N        5        26        31
                      NS        4        18        22


                      Wt    80.41    360.71    441.12
                 Het Chi     5.46    143.84    189.82
                 Het  df        4        25        30
                 Het  P      N.S.       ***       ***
               Fixed  RR     2.38      5.21      4.52
                     RRl     1.91      4.70      4.11
                     RRu     2.96      5.78      4.96
                      P       +++       +++       +++
              Random  RR     2.46      4.38      4.06
                     RRl     1.86      3.29      3.10
                     RRu     3.27      5.83      5.31
                      P       +++       +++       +++
             Between Chi                        40.53
             Between  df                            1
             Between  P                           ***
             Btwn(F)  P                            **

                          COPD subtype
                             mort        LF     other     Total


                       N       31                            31
                      NS       22                            22


                      Wt   441.12                        441.12
                 Het Chi   189.82                        189.82
                 Het  df       30                            30
                 Het  P       ***                           ***
               Fixed  RR     4.52                          4.52
                     RRl     4.11                          4.11
                     RRu     4.96                          4.96
                      P       +++                           +++
              Random  RR     4.06                          4.06
                     RRl     3.10                          3.10
                     RRu     5.31                          5.31
                      P       +++                           +++
             Between Chi
             Between  df
             Between  P                                    N.S.
             Btwn(F)  P                                    N.S.

                               Asthma analysis type (COPD)
                        inc-irres  excl-all defn-incl     other     Total


                       N       16                  10         5        31
                      NS       10                   7         5        22


                      Wt   263.63              124.69     52.79    441.12
                 Het Chi    38.36               15.40      9.15    189.82
                 Het  df       15                   9         4        30
                 Het  P       ***                 (*)       (*)       ***
               Fixed  RR     6.88                2.06      3.51      4.52
                     RRl     6.10                1.73      2.68      4.11
                     RRu     7.77                2.46      4.59      4.96
                      P       +++                 +++       +++       +++
              Random  RR     6.04                2.19      3.55      4.06
                     RRl     4.78                1.68      2.26      3.10
                     RRu     7.63                2.85      5.60      5.31
                      P       +++                 +++       +++       +++
             Between Chi                                           126.91
             Between  df                                                2
             Between  P                                               ***
             Btwn(F)  P                                               ***
  ________________________________________________________________________________________________________________________
                                            International Evidence on Smoking and COPD, Phase 3, Analysis run on 27-SEP-10

                                                   Table 1 - A - 3 - 3

            IESCOPD - Meta-analysis of ever smoking, any product (or cigarettes if all product not available)
                                                 COPD based on mortality
                                                      Most-adjusted
                        Bronchodilator/reversibility (LF only)
                          no/unkn  yes/revs     Total


                       N
                      NS


                      Wt
                 Het Chi
                 Het  df
                 Het  P                           (*)
               Fixed  RR
                     RRl
                     RRu
                      P                           +++
              Random  RR
                     RRl
                     RRu
                      P                           +++
             Between Chi
             Between  df
             Between  P                          N.S.
             Btwn(F)  P                          N.S.

                                  Number of COPD cases
                             1-50    51-100   101-200      201+     Total


                       N        6         7         6        12        31
                      NS        4         5         5         8        22


                      Wt    14.95     36.82     47.35    341.99    441.12
                 Het Chi     7.24      6.01     27.09     98.10    189.82
                 Het  df        5         6         5        11        30
                 Het  P      N.S.      N.S.       ***       ***       ***
               Fixed  RR     3.46      1.93      2.66      5.39      4.52
                     RRl     2.08      1.40      2.00      4.84      4.11
                     RRu     5.74      2.67      3.54      5.99      4.96
                      P       +++       +++       +++       +++       +++
              Random  RR     4.11      1.94      4.84      4.92      4.06
                     RRl     2.09      1.40      2.14      3.56      3.10
                     RRu     8.08      2.67     10.97      6.80      5.31
                      P       +++       +++       +++       +++       +++
             Between Chi                                            51.38
             Between  df                                                3
             Between  P                                               ***
             Btwn(F)  P                                                 *

                            Analysis type
                         prevlnce     onset     Total


                       N        2        29        31
                      NS        1        21        22


                      Wt    62.42    378.70    441.12
                 Het Chi     1.21    150.19    189.82
                 Het  df        1        28        30
                 Het  P      N.S.       ***       ***
               Fixed  RR     2.18      5.09      4.52
                     RRl     1.70      4.60      4.11
                     RRu     2.80      5.63      4.96
                      P       +++       +++       +++
              Random  RR     2.19      4.31      4.06
                     RRl     1.66      3.28      3.10
                     RRu     2.88      5.65      5.31
                      P       +++       +++       +++
             Between Chi                        38.42
             Between  df                            1
             Between  P                           ***
             Btwn(F)  P                             *
  ________________________________________________________________________________________________________________________
                                            International Evidence on Smoking and COPD, Phase 3, Analysis run on 27-SEP-10

                                                   Table 1 - A - 3 - 3

            IESCOPD - Meta-analysis of ever smoking, any product (or cigarettes if all product not available)
                                                 COPD based on mortality
                                                      Most-adjusted
                             Smoking product
                              any      cigs  cigsonly     Total


                       N       13        10         8        31
                      NS       10         8         6        24


                      Wt   139.18     66.66    235.28    441.12
                 Het Chi    49.99     14.74     63.84    189.82
                 Het  df       12         9         7        30
                 Het  P       ***       (*)       ***       ***
               Fixed  RR     3.22      2.71      6.38      4.52
                     RRl     2.73      2.13      5.61      4.11
                     RRu     3.80      3.45      7.24      4.96
                      P       +++       +++       +++       +++
              Random  RR     3.31      2.87      6.42      4.06
                     RRl     2.24      2.02      4.22      3.10
                     RRu     4.89      4.09      9.78      5.31
                      P       +++       +++       +++       +++
             Between Chi                                  61.25
             Between  df                                      2
             Between  P                                     ***
             Btwn(F)  P                                      **

                                     Unexposed group
                          nev any   nev cig  nev+ any  nev+ cig     Total


                       N       21        10                            31
                      NS       14         8                            22


                      Wt   374.46     66.66                        441.12
                 Het Chi   154.66     14.74                        189.82
                 Het  df       20         9                            30
                 Het  P       ***       (*)                           ***
               Fixed  RR     4.95      2.71                          4.52
                     RRl     4.47      2.13                          4.11
                     RRu     5.47      3.45                          4.96
                      P       +++       +++                           +++
              Random  RR     4.44      2.87                          4.06
                     RRl     3.23      2.02                          3.10
                     RRu     6.09      4.09                          5.31
                      P       +++       +++                           +++
             Between Chi                                            20.42
             Between  df                                                1
             Between  P                                               ***
             Btwn(F)  P                                               (*)

                        Unexposed group (combining nev+ with main levels)
                          nev any   nev cig     Total


                       N       21        10        31
                      NS       14         8        22


                      Wt   374.46     66.66    441.12
                 Het Chi   154.66     14.74    189.82
                 Het  df       20         9        30
                 Het  P       ***       (*)       ***
               Fixed  RR     4.95      2.71      4.52
                     RRl     4.47      2.13      4.11
                     RRu     5.47      3.45      4.96
                      P       +++       +++       +++
              Random  RR     4.44      2.87      4.06
                     RRl     3.23      2.02      3.10
                     RRu     6.09      4.09      5.31
                      P       +++       +++       +++
             Between Chi                        20.42
             Between  df                            1
             Between  P                           ***
             Btwn(F)  P                           (*)
  ________________________________________________________________________________________________________________________
                                            International Evidence on Smoking and COPD, Phase 3, Analysis run on 27-SEP-10

                                                   Table 1 - A - 3 - 3

            IESCOPD - Meta-analysis of ever smoking, any product (or cigarettes if all product not available)
                                                 COPD based on mortality
                                                      Most-adjusted
                        Smoking results reported in study (COPD)
                             Ever   Current      Both     Total


                       N        2                  29        31
                      NS        1                  21        22


                      Wt     3.61              437.51    441.12
                 Het Chi     1.75              187.92    189.82
                 Het  df        1                  28        30
                 Het  P      N.S.                 ***       ***
               Fixed  RR     5.51                4.51      4.52
                     RRl     1.97                4.11      4.11
                     RRu    15.47                4.95      4.96
                      P        ++                 +++       +++
              Random  RR     7.24                3.99      4.06
                     RRl     1.32                3.03      3.10
                     RRu    39.79                5.25      5.31
                      P         +                 +++       +++
             Between Chi                                   0.15
             Between  df                                      1
             Between  P                                    N.S.
             Btwn(F)  P                                    N.S.

                        Number of adjustment variables
                                0         1        2+     Total


                       N        2        17        12        31
                      NS        2        11         9        22


                      Wt    25.47    243.22    172.43    441.12
                 Het Chi     3.98     48.68     39.66    189.82
                 Het  df        1        16        11        30
                 Het  P         *       ***       ***       ***
               Fixed  RR     1.55      6.76      3.00      4.52
                     RRl     1.05      5.96      2.58      4.11
                     RRu     2.29      7.66      3.48      4.96
                      P         +       +++       +++       +++
              Random  RR     4.34      5.57      3.11      4.06
                     RRl     0.29      4.23      2.28      3.10
                     RRu    65.41      7.33      4.23      5.31
                      P      N.S.       +++       +++       +++
             Between Chi                                  97.50
             Between  df                                      2
             Between  P                                     ***
             Btwn(F)  P                                     ***

                        RR adjusted for sex (combined sex RR only)
                              Yes        No     Total


                       N                  1         1
                      NS                  1         1


                      Wt              24.97     24.97
                 Het Chi               0.00      0.00
                 Het  df                  0         0
                 Het  P                N.S.      N.S.
               Fixed  RR               1.47      1.47
                     RRl               0.99      0.99
                     RRu               2.17      2.17
                      P                 (+)       (+)
              Random  RR               1.47      1.47
                     RRl               0.99      0.99
                     RRu               2.17      2.17
                      P                 (+)       (+)
             Between Chi
             Between  df
             Between  P                          N.S.
             Btwn(F)  P                          N.S.
  ________________________________________________________________________________________________________________________
                                            International Evidence on Smoking and COPD, Phase 3, Analysis run on 27-SEP-10

                                                   Table 1 - A - 3 - 3

            IESCOPD - Meta-analysis of ever smoking, any product (or cigarettes if all product not available)
                                                 COPD based on mortality
                                                      Most-adjusted
                        RR adjusted for age
                              Yes        No     Total


                       N       29         2        31
                      NS       20         2        22


                      Wt   415.65     25.47    441.12
                 Het Chi   155.03      3.98    189.82
                 Het  df       28         1        30
                 Het  P       ***         *       ***
               Fixed  RR     4.82      1.55      4.52
                     RRl     4.38      1.05      4.11
                     RRu     5.31      2.29      4.96
                      P       +++         +       +++
              Random  RR     4.19      4.34      4.06
                     RRl     3.23      0.29      3.10
                     RRu     5.43     65.41      5.31
                      P       +++      N.S.       +++
             Between Chi                        30.81
             Between  df                            1
             Between  P                           ***
             Btwn(F)  P                             *

                        RR adjusted for factor other than sex, age


                       N       12        19        31
                      NS        9        13        22


                      Wt   172.43    268.69    441.12
                 Het Chi    39.66    102.51    189.82
                 Het  df       11        18        30
                 Het  P       ***       ***       ***
               Fixed  RR     3.00      5.88      4.52
                     RRl     2.58      5.21      4.11
                     RRu     3.48      6.62      4.96
                      P       +++       +++       +++
              Random  RR     3.11      4.94      4.06
                     RRl     2.28      3.49      3.10
                     RRu     4.23      7.01      5.31
                      P       +++       +++       +++
             Between Chi                        47.65
             Between  df                            1
             Between  P                           ***
             Btwn(F)  P                            **

                        Derivation of RR/CI
                         Orig/2x2     Other     Total


                       N        2        29        31
                      NS        1        21        22


                      Wt     3.61    437.51    441.12
                 Het Chi     1.75    187.92    189.82
                 Het  df        1        28        30
                 Het  P      N.S.       ***       ***
               Fixed  RR     5.51      4.51      4.52
                     RRl     1.97      4.11      4.11
                     RRu    15.47      4.95      4.96
                      P        ++       +++       +++
              Random  RR     7.24      3.99      4.06
                     RRl     1.32      3.03      3.10
                     RRu    39.79      5.25      5.31
                      P         +       +++       +++
             Between Chi                         0.15
             Between  df                            1
             Between  P                          N.S.
             Btwn(F)  P                          N.S.

  ________________________________________________________________________________________________________________________
                                            International Evidence on Smoking and COPD, Phase 3, Analysis run on 27-SEP-10

                                                   Table 1 - A - 3 - 4

            IESCOPD - Meta-analysis of ever smoking, any product (or cigarettes if all product not available)
                                                 COPD based on mortality
                                                      Least-adjusted


     REF|NRR|X|SEX|AGEL|AGEH|     REGION|BEGYR|PUBYR|STTYP|ONSET|      DISEAS|ADJ|SMOKSTA|   PRODUCT|    UNEXP|

    BEST  37     m   30   97   Am:Canada  1955  1967    Pr   Inc    COPD:mort   1    Ever  Cigs only   Nev any
   DEAN1   3 x   m   35   99       Eu:UK  1969  1977    CC  Prev    COPD:mort   0    Ever        Any   Nev any
   DEAN1   9 x   f   35   99       Eu:UK  1969  1977    CC  Prev    COPD:mort   0    Ever MCigs only   Nev any
   DOLL1   3     m   20   99       Eu:UK  1951  1994    Pr   Inc    COPD:mort   2    Ever        Any   Nev any
   DOLL2   3     f   20   99       Eu:UK  1951  1980    Pr   Inc    COPD:mort   1    Ever  Cigs only   Nev any
  HAMMO2   5     m   35   99      Am:USA  1959  1966    Pr   Inc    COPD:mort   1    Ever  Cigs only   Nev any
  HAMMO2   6     f   35   99      Am:USA  1959  1966    Pr   Inc    COPD:mort   1    Ever  Cigs only   Nev any
  HIGGI4   3     m   35   99      Am:USA  1962  1989    Pr   Inc    COPD:mort   1    Ever       Cigs  Nev cigs
  JACOBS   3 x   m   40   84       Multi  1957  1999    Pr   Inc    COPD:mort   2    Ever       Cigs  Nev cigs
   KAHN2 131     m   31   84      Am:USA  1954  1966    Pr   Inc    COPD:mort   1    Ever        Any   Nev any
    LAM1   1     m   35   99   Asia:FarE  1976  1997    Pr   Inc    COPD:mort   7    Ever       Cigs  Nev cigs
    LAM1   2     f   35   99   Asia:FarE  1976  1997    Pr   Inc    COPD:mort   7    Ever       Cigs  Nev cigs
    LAM3   3     m   60   99   Asia:FarE  1987  2006    Pr   Inc    COPD:mort   7    Ever       Cigs  Nev cigs
   LANGE   3     m   20   99    Eu:Scand  1976  1992    Pr   Inc    COPD:mort   1    Ever        Any   Nev any
   LANGE   6     f   20   99    Eu:Scand  1976  1992    Pr   Inc    COPD:mort   1    Ever        Any   Nev any
     LEE   6 x   m   35   82       Eu:UK  1964  1979    Pr   Inc    COPD:mort   0    Ever        Any   Nev any
     LEE  41 x   f   35   82       Eu:UK  1964  1979    Pr   Inc    COPD:mort   0    Ever        Any   Nev any
  MANNI3   3     b   25   96      Am:USA  1971  2003    Pr   Inc    COPD:mort   0    Ever        Any   Nev any
  MARCUS   3     m   45   85      Am:USA  1965  1989    Pr   Inc    COPD:mort   1    Ever        Any   Nev any
  NILSSO   9     m   18   99    Eu:Scand  1963  2001    Pr   Inc    COPD:mort   2    Ever        Any   Nev any
  NILSSO   6     f   18   99    Eu:Scand  1963  2001    Pr   Inc    COPD:mort   2    Ever  Cigs only   Nev any
    PETO   3     m   25   92       Eu:UK  1954  1983    Pr   Inc    COPD:mort   0    Ever       Cigs  Nev cigs
  SPEIZE   3     m   25   86      Am:USA  1974  1989    Pr   Inc    COPD:mort   1    Ever       Cigs  Nev cigs
  SPEIZE   6     f   25   86      Am:USA  1974  1989    Pr   Inc    COPD:mort   1    Ever       Cigs  Nev cigs
    TANG   4     m   35   99       Eu:UK  1967  1995    Pr   Inc    COPD:mort   2    Ever        Any   Nev any
    THUN   5     m   30   99      Am:USA  1982  1997    Pr   Inc    COPD:mort   1    Ever  Cigs only   Nev any
    THUN   6     f   30   99      Am:USA  1982  1997    Pr   Inc    COPD:mort   1    Ever  Cigs only   Nev any
    TODD   6 x   m   35   81       Eu:UK  1965  1978    Pr   Inc    COPD:mort   0    Ever        Any   Nev any
    TODD  41 x   f   35   81       Eu:UK  1965  1978    Pr   Inc    COPD:mort   0    Ever        Any   Nev any
  TVERDA   3     m   35   65    Eu:Scand  1972  1993    Pr   Inc    COPD:mort   2    Ever       Cigs  Nev cigs
    YUAN   3 x   m   45   71   Asia:FarE  1986  1996    Pr   Inc    COPD:mort   1    Ever       Cigs  Nev cigs


  ________________________________________________________________________________________________________________________
                                            International Evidence on Smoking and COPD, Phase 3, Analysis run on 27-SEP-10

                                                   Table 1 - A - 3 - 5

            IESCOPD - Meta-analysis of ever smoking, any product (or cigarettes if all product not available)
                                                 COPD based on mortality
                                                      Least-adjusted


                        Number Exposed  Non-exposed
 REF    NRR SEX ADJ     Case    Cont    Case    Cont      RR        95.00%CI
*BEST   37  m   1        129       -       6       -     10.02 (  4.42-  22.72)
 DEAN1  3   m   0        483    2054      47     510      2.55 (  1.86-   3.50)
 DEAN1  9   f   0         98    1420     120    1538      0.88 (  0.67-   1.17)
 Subtotal DEAN1                                           1.40 (  1.14-   1.73)
*DOLL1  3   m   2        522       -      12       -      6.54 (  3.71-  11.53)
*DOLL2  3   f   1         12       -       1       -     11.02 (  1.43-  84.78)
*HAMMO2 5   m   1          -       -       -       -      9.27 (  6.76-  12.70)
*HAMMO2 6   f   1          -       -       -       -      5.87 (  4.17-   8.26)
 Subtotal HAMMO2                                          7.51 (  5.96-   9.47)
*HIGGI4 3   m   1          -       -       -       -      3.08 (  1.72-   5.52)
*JACOBS 3   m   2        257       -      28       -      3.86 (  2.45-   6.09)
*KAHN2  131 m   1        496       -      31       -      6.69 (  4.63-   9.66)
*LAM1   1   m   7         19       -       4       -      4.05 (  1.31-  12.50)
*LAM1   2   f   7          2       -       2       -     26.61 (  2.08- 339.00)
 Subtotal LAM1                                            5.51 (  1.97-  15.47)
*LAM3   3   m   7         29       -       9       -      1.81 (  0.83-   3.98)
*LANGE  3   m   1        111       -       3       -      4.50 (  1.45-  13.90)
*LANGE  6   f   1         56       -       3       -     12.90 (  3.04-  54.84)
 Subtotal LANGE                                           6.71 (  2.75-  16.34)
*LEE    6   m   0         40    2972       1     347      4.67 (  0.64-  33.87)
*LEE    41  f   0         10    2620       5    1694      1.29 (  0.44-   3.78)
 Subtotal LEE                                             1.73 (  0.67-   4.44)
*MANNI3 3   b   0         76    2724      36    1895      1.47 (  0.99-   2.17)
*MARCUS 3   m   1         75       -      14       -      2.17 (  1.20-   3.90)
*NILSSO 9   m   2         64       -      13       -      2.78 (  1.50-   5.14)
*NILSSO 6   f   2         63       -      57       -      5.93 (  4.02-   8.75)
 Subtotal NILSSO                                          4.78 (  3.44-   6.64)
*PETO   3   m   0        104    2423       0     295     25.48~(  1.59- 409.05)
*SPEIZE 3   m   1         26       -       0       -     11.40 (  0.69- 187.05)
*SPEIZE 6   f   1         14       -       5       -      4.20 (  1.51-  11.66)
 Subtotal SPEIZE                                          4.72 (  1.81-  12.34)
*TANG   4   m   2          -       -       -       -      4.12 (  2.33-   7.28)
*THUN   5   m   1          -       -       -       -      8.96 (  6.66-  12.06)
*THUN   6   f   1          -       -       -       -      8.05 (  6.17-  10.50)
 Subtotal THUN                                            8.44 (  6.93-  10.29)
*TODD   6   m   0         59    4250       1     520      7.22 (  1.00-  51.99)
*TODD   41  f   0          4    3173       4    2120      0.67 (  0.17-   2.67)
 Subtotal TODD                                            1.46 (  0.47-   4.55)
*TVERDA 3   m   2         55       -       7       -      2.88 (  1.31-   6.33)
*YUAN   3   m   1         34       -      19       -      1.33 (  0.76-   2.34)
Partial Totals          2838   21636     428    8919
*prospective study                                        ~ With 0.5 adjustment for zero

                       N       31
                      NS       22


                      Wt   463.80
                 Het Chi   298.17
                 Het  df       30
                 Het  P       ***
               Fixed  RR     4.01
                     RRl     3.66
                     RRu     4.39
                      P       +++
              Random  RR     3.95
                     RRl     2.85
                     RRu     5.45
                      P       +++
               Asymm  P      N.S.


  ________________________________________________________________________________________________________________________
                                            International Evidence on Smoking and COPD, Phase 3, Analysis run on 27-SEP-10

                                                   Table 1 - A - 3 - 6

            IESCOPD - Meta-analysis of ever smoking, any product (or cigarettes if all product not available)
                                                 COPD based on mortality
                                                      Least-adjusted


                       N       31
                      NS       22


                      Wt   463.80
                 Het Chi   298.17
                 Het  df       30
                 Het  P       ***
               Fixed  RR     4.01
                     RRl     3.66
                     RRu     4.39
                      P       +++
              Random  RR     3.95
                     RRl     2.85
                     RRu     5.45
                      P       +++
               Asymm  P      N.S.

                                   Sex
                             both      male    female     Total


                       N        1        20        10        31
                      NS        1        20        10        31


                      Wt    24.97    263.53    175.30    463.80
                 Het Chi     0.00     99.50    164.23    298.17
                 Het  df        0        19         9        30
                 Het  P      N.S.       ***       ***       ***
               Fixed  RR     1.47      4.73      3.61      4.01
                     RRl     0.99      4.20      3.11      3.66
                     RRu     2.17      5.34      4.18      4.39
                      P       (+)       +++       +++       +++
              Random  RR     1.47      4.20      3.97      3.95
                     RRl     0.99      3.06      1.87      2.85
                     RRu     2.17      5.75      8.44      5.45
                      P       (+)       +++       +++       +++
             Between Chi                                  34.45
             Between  df                                      2
             Between  P                                     ***
             Btwn(F)  P                                    N.S.


  ________________________________________________________________________________________________________________________
                                            International Evidence on Smoking and COPD, Phase 3, Analysis run on 27-SEP-10

                                                   Table 1 - A - 3 - 7

            IESCOPD - Meta-analysis of ever smoking, any product (or cigarettes if all product not available)
                                                 COPD based on mortality
                                 Excluded studies (and stage at which they were excluded)


1       CLARK COTTON  MEYER REMYJA RUTGER SNYDER SOBRAX     SU TAKEMU  WANG4   WEIR WHICKE ZALACA
2      ALDERS ANDER2 AUERBA   BANG  BECK1  BECK2 BJORNS  BROWN CERVER CHAPMA COATES COLLEG  DEAN2  DEANE DONTA2 DOPICO
       EHRLIC ENRIGH FINKLE FLETCH FOXMAN GOLDBE HAENSZ HARRIS  HAYES HIGGI2 HIGGI3 HIGGI6 HIRAYA HOLLA2 HOLLNA  HOUSE
       HRUBEC HUCHON JENSEN JINDA2  JOSHI JOUSI1   KATO KOTAN1  KUBIK LAMBER LANGE2 LANGHA LAVECC LUNDB2 MAGNUS MANFRE
       MELLST MENEZ1  MEREN MILLER  MILNE MOLLER   NAWA NEJJAR OGILVI  OMORI OSWAL1 OSWAL2 PANDEY  PRATT   REID RIMING
        RYDER SCHWAR  SHARP SHIMUR SOBRAD STJERN SUADIC SUTINE TAGER2 TROISI URRUTI VIEGI1 VIKGRE WAGEN2  WANG2    WIG
       WILHEL WILSO2  WOODS  WOOLF   ZOIA
3      BROGGE  CHEN1  CHENG DETORR GEIJER HEDMAN JAENDI KHOURY KOTAN2 KULLER   LIU1   LIU2  PRICE  TAGER VINEIS WATSON
4       CHEN3   KAHN   LIAW   PEAT SAWICK STERLI VOLLM1 VOLLM2   WALD    WEN WOJTYN
5      ALESSA  AMIGO ANDER1 ANDER3 BEDNAR  CHEN2 CLEMEN  COCCI DEJONG DEMARC DICKIN DONTA1 EKBERG FERRI1 FERRI2 FERRI3
        FIDAN FORAST FUKUCH GODTFR GULSVI HARDIE HARIKK     HO HOZAWA HUHTI1 HUHTI2 HUHTI3 ITABAS JOHANN KACHEL KARAKA
       KATANC    KIM  KIRAZ KLAYTO KOJIMA KRZYZA    LAI   LAM2 LEBOWI LINDBE LINDST LUNDB1  MADOR MANNI1 MANNI2 MARAN1
       MARAN2 MATHES MENEZ2 MENEZ3 MENEZ4 MENEZ5 MENEZ6 MONTNE MUELLE NIEPSU NIHLEN PELKON PEREZP RENWIC RICCIO SARGEA
       SHAHAB   SHIN SICHLE  SILVA  STROM TRUPIN TSUSHI VESTBO VIEGI2 VONHER  WEISS WILSO1   XIAO     XU YAMAGU ZIELI1
       ZIELI2 ZIETKO
9      ENSTRO HAWTHO


  ________________________________________________________________________________________________________________________
                                            International Evidence on Smoking and COPD, Phase 3, Analysis run on 27-SEP-10

                                                   Table 1 - A - 3 - 8

            IESCOPD - Meta-analysis of ever smoking, any product (or cigarettes if all product not available)
                                                 COPD based on mortality
                                             Potentially overlapping studies


     REF| REFGP|PRINC|                     OVERLAP|

  JACOBS JACOBS     1  JACOBS/DONTA1/DONTA2/PELKON
    PETO   PETO     1    PETO/HIGGI1/HIGGI2/HIGGI5
    TANG   TANG     1             TANG/HAWTHO/WALD
    LAM3   LAM3     1                    LAM2/LAM3
  HAMMO2 HAMMO2     1                HAMMO2/ENSTRO
  MANNI3 MANNI3     1                MANNI2/MANNI3
    TODD   TODD     1                  LAMBER/TODD
   LANGE GODTFR     2 GODT/VEST/LANG1+2/SUAD/HOLLN
  HIGGI4 HIGGI4     1                HIGGI4/HIGGI6
   KAHN2   KAHN     2                   KAHN/KAHN2


  ________________________________________________________________________________________________________________________
                                            International Evidence on Smoking and COPD, Phase 3, Analysis run on 27-SEP-10

                                                    Table 1 - A - 4 -

            IESCOPD - Meta-analysis of ever smoking, any product (or cigarettes if all product not available)
                                             COPD based on lung function only


This analysis is restricted to results for:
1) Eligible study on database
2) Outcome COPD
3) Non-dose-response data
4) Ever smoking
5) COPD based on lung function only
6) Results complete enough for use in meta-analysis

Within each study, results are then selected (in the following order of preference, within each sex) for:
7) UNEXP   : never any, never cigarettes, other
8) PROD    : any product, cigarettes, cigarettes only
9) For overlapping studies: principal rather than subsidiary studies
and then for single sex results (m, f) in preference to results for both sexes combined (b).

Results adjusted for the most potential confounders are then chosen in Sections -1 to -3
and results adjusted for the least confounders in Sections -4 to -6. (Those least adjusted results which
actually differ from the most adjusted are marked 'x' in column X in Section -4)

Section -7 shows excluded studies, together with the stage (as above) at which no qualifying
results were found.

Section -8 lists the potentially overlapping studies which have been included (1=principal, 2=subsidiary),
and any results which would have been included in preference except that they had data not complete enough
for use in meta-analysis. It also lists their significance (yes/no), if known.


  ________________________________________________________________________________________________________________________
                                            International Evidence on Smoking and COPD, Phase 3, Analysis run on 27-SEP-10

                                                   Table 1 - A - 4 - 1

            IESCOPD - Meta-analysis of ever smoking, any product (or cigarettes if all product not available)
                                             COPD based on lung function only
                                                      Most-adjusted


     REF|NRR|SEX|AGEL|AGEH|     REGION|BEGYR|PUBYR|STTYP|ONSET|      DISEAS|ADJ|SMOKSTA|   PRODUCT|    UNEXP|

  ANDER3   3   m    0   99     Eu:East     *  2001    CC  Prev      COPD:LF   0    Ever        Any   Nev any
  ANDER3   6   f    0   99     Eu:East     *  2001    CC  Prev      COPD:LF   0    Ever        Any   Nev any
  BEDNAR   3   b   30   99     Eu:East  2000  2005    CS  Prev      COPD:LF   0    Ever        Any   Nev any
  CLEMEN   3   m   20   60     Eu:West  1960  1982    Pr  Prev      COPD:LF   0    Ever        Any   Nev any
  DEJONG   3   b    0   99      Am:USA     *  2004    CS  Prev      COPD:LF   0    Ever       Cigs  Nev cigs
  DEMARC   6   b   20   44       Multi  1991  2004    CS  Prev      COPD:LF   4    Ever        Any   Nev any
  DICKIN   3   b   60   74       Eu:UK     *  1999    CS  Prev      COPD:LF   0    Ever        Any   Nev any
  EKBERG   3   m   15   99    Eu:Scand  1974  2005    CS  Prev      COPD:LF   0    Ever        Any   Nev any
  EKBERG   6   f   15   99    Eu:Scand  1974  2005    CS  Prev      COPD:LF   0    Ever        Any   Nev any
  FUKUCH   3   b   40   99   Asia:FarE  2000  2004    CS  Prev      COPD:LF   0    Ever       Cigs  Nev cigs
  HOZAWA   3   b   45   64      Am:USA  1987  2006    CS  Prev      COPD:LF   0    Ever       Cigs  Nev cigs
  HUHTI2   5   m   50   74    Eu:Scand  1971  1980    CS  Prev      COPD:LF   0    Ever        Any   Nev any
  HUHTI2   6   f   50   74    Eu:Scand  1971  1980    CS  Prev      COPD:LF   0    Ever        Any   Nev any
  HUHTI3   6   m   25   69    Eu:Scand  1968  1978    CS  Prev      COPD:LF   1    Ever        Any   Nev any
  ITABAS   1   b   60   99   Asia:FarE     *  1990    CC  Prev      COPD:LF   0    Ever       Cigs  Nev cigs
  JOHANN   3   b   26   82    Eu:Scand  1996  2005    CS  Prev      COPD:LF   0    Ever       Cigs  Nev cigs
  KACHEL   3   b   20   79     Eu:East     *  2003    CS  Prev      COPD:LF   0    Ever       Cigs  Nev cigs
  KATANC   3   b   70   79      Am:USA  1997  2005    CS  Prev      COPD:LF   0    Ever       Cigs  Nev cigs
     KIM   3   m   45   99   Asia:FarE  2001  2005    CS  Prev      COPD:LF   1    Ever       Cigs  Nev cigs
     KIM   6   f   45   99   Asia:FarE  2001  2005    CS  Prev      COPD:LF   1    Ever       Cigs  Nev cigs
  KLAYTO   1   b   40   99      Am:USA     *  1975    CS  Prev      COPD:LF   0    Ever       Cigs  N/L cigs
  KOJIMA   3   m   25   74   Asia:FarE  2001  2005    CS  Prev      COPD:LF   0    Ever       Cigs  Nev cigs
  KOJIMA   6   f   25   74   Asia:FarE  2001  2005    CS  Prev      COPD:LF   0    Ever       Cigs  Nev cigs
  KRZYZA   3   m   19   83     Eu:East  1968  1986    Pr   Inc      COPD:LF   1    Ever       Cigs  Nev cigs
  KRZYZA   6   f   19   83     Eu:East  1968  1986    Pr   Inc      COPD:LF   1    Ever       Cigs  Nev cigs
     LAI   1   m   18   80   Asia:FarE  2001  2006    CS  Prev      COPD:LF   0    Ever        Any   Nev any
     LAI   2   f   18   80   Asia:FarE  2001  2006    CS  Prev      COPD:LF   0    Ever        Any   Nev any
  LEBOWI   6   m   15   96      Am:USA  1972  1977    CS  Prev      COPD:LF   1    Ever       Cigs  Nev cigs
  LEBOWI  18   f   15   96      Am:USA  1972  1977    CS  Prev      COPD:LF   1    Ever       Cigs  Nev cigs
  LINDBE   3   b   46   84    Eu:Scand  1996  2006    Pr   Inc      COPD:LF   3    Ever        Any   Nev any
  LUNDB1   9   b   46   77    Eu:Scand  1996  2003    CS  Prev      COPD:LF   4    Ever       Cigs  Nev cigs
   MADOR   3   m   15   99      Am:USA     *  2003    CC  Prev      COPD:LF   0    Ever       Cigs  Nev cigs
  MANNI1   1   m   17   99      Am:USA  1988  2000    CS  Prev      COPD:LF   2    Ever        Any   Nev any
  MANNI1  10   f   17   99      Am:USA  1988  2000    CS  Prev      COPD:LF   2    Ever       Cigs   Nev any
  MANNI2   3   b   25   74      Am:USA  1971  2003    CS  Prev      COPD:LF   1    Ever        Any   Nev any
  MATHES   3   b   45   70      Aus/NZ     *  2006    CC  Prev      COPD:LF   0    Ever       Cigs  Nev cigs
  MENEZ2   6   b   40   99 Am:Sth/Cent  2003  2005    CS  Prev      COPD:LF   5    Ever       Cigs  Nev cigs
  MENEZ3   3   b   40   99 Am:Sth/Cent  2003  2005    CS  Prev      COPD:LF   0    Ever       Cigs  Nev cigs
  MENEZ4   3   b   40   99 Am:Sth/Cent  2003  2005    CS  Prev      COPD:LF   0    Ever       Cigs  Nev cigs
  MENEZ5   3   b   40   99 Am:Sth/Cent  2003  2005    CS  Prev      COPD:LF   0    Ever       Cigs  Nev cigs
  MENEZ6   3   b   40   99 Am:Sth/Cent  2003  2005    CS  Prev      COPD:LF   0    Ever       Cigs  Nev cigs
  MUELLE   6   m   20   69      Am:USA  1967  1971    CS  Prev      COPD:LF   1    Ever        Any   Nev any
  MUELLE  13   f   20   69      Am:USA  1967  1971    CS  Prev      COPD:LF   1    Ever        Any   Nev any
  NIEPSU   3   b   19   69     Eu:East  2001  2002    CS  Prev      COPD:LF   0    Ever       Cigs  Nev cigs
  PEREZP   1   f   40   99 Am:Sth/Cent  1992  1996    CC  Prev      COPD:LF   0    Ever       Cigs  Nev cigs
  RENWIC   3   b   45   99       Eu:UK  1992  1996    CS  Prev      COPD:LF   0    Ever        Any   Nev any
  RICCIO   3   b   25   99     Eu:West  2002  2005    CS  Prev      COPD:LF   0    Ever       Cigs  Nev cigs
  SHAHAB   3   b   35   99       Eu:UK  2001  2006    CS  Prev      COPD:LF   0    Ever       Cigs  Nev cigs
    SHIN   5   b   18   99   Asia:FarE  1999  2003    CS  Prev      COPD:LF   3    Ever       Cigs  Nev cigs
   STROM   3   m   68   68    Eu:Scand  1982  1996    CS  Prev      COPD:LF   0    Ever        Any   Nev any
  TSUSHI   3   b    0   99   Asia:FarE  2003  2006    CS  Prev      COPD:LF   0    Ever       Cigs  Nev cigs
  VESTBO   3   b   20   99    Eu:Scand  1976  2002    CS  Prev      COPD:LF   0    Ever        Any   Nev any
  VIEGI2   6   m   25   73     Eu:West  1988  2000    CS  Prev      COPD:LF   1    Ever       Cigs  Nev cigs
  VIEGI2  12   f   25   73     Eu:West  1988  2000    CS  Prev      COPD:LF   1    Ever       Cigs  Nev cigs
   WEISS   3   m   50   69      Am:USA  1961  1963    CS  Prev      COPD:LF   0    Ever        Any   Nev any
  WILSO1   1   b   18   99      Aus/NZ  2000  2005    CS  Prev      COPD:LF   0    Ever        Any   Nev any
  YAMAGU   3   b   40   99   Asia:FarE  1986  1988    CS  Prev      COPD:LF   6    Ever       Cigs  Nev cigs
  ZIELI1   2   b    0   99     Eu:East  1999  2001    CS  Prev      COPD:LF   1    Ever       Cigs  Nev cigs
  ZIELI2   3   m    0   99     Eu:East  2000  2006    CS  Prev      COPD:LF   0    Ever       Cigs  Nev cigs
  ZIELI2   6   f    0   99     Eu:East  2000  2006    CS  Prev      COPD:LF   0    Ever       Cigs  Nev cigs
  ZIETKO   2   m    0   99     Eu:East     *  2005    CC  Prev      COPD:LF   0    Ever       Cigs  Nev cigs
  ZIETKO   4   f    0   99     Eu:East     *  2005    CC  Prev      COPD:LF   0    Ever       Cigs  Nev cigs


  ________________________________________________________________________________________________________________________
                                            International Evidence on Smoking and COPD, Phase 3, Analysis run on 27-SEP-10

                                                   Table 1 - A - 4 - 2

            IESCOPD - Meta-analysis of ever smoking, any product (or cigarettes if all product not available)
                                             COPD based on lung function only
                                                      Most-adjusted


                        Number Exposed  Non-exposed
 REF    NRR SEX ADJ     Case    Cont    Case    Cont      RR        95.00%CI
 ANDER3 3   m   0         15       8       0       8     31.00~(  1.59- 605.65)
 ANDER3 6   f   0          2       1       3       3      2.00 (  0.11-  35.81)
 Subtotal ANDER3                                          7.56 (  0.95-  59.91)
 BEDNAR 3   b   0         52     376      20     228      1.58 (  0.92-   2.71)
 CLEMEN 3   m   0         57    1629      11     709      2.26 (  1.18-   4.33)
 DEJONG 3   b   0         55     154       0      34     24.79~(  1.49- 411.13)
 DEMARC 6   b   4        381       -     156       -      1.76 (  1.44-   2.15)
 DICKIN 3   b   0         34     167       1     125     25.45 (  3.44- 188.43)
 EKBERG 3   m   0       2249    7692     395    2841      2.10 (  1.87-   2.36)
 EKBERG 6   f   0        546    3300     166    2581      2.57 (  2.15-   3.08)
 Subtotal EKBERG                                          2.23 (  2.02-   2.46)
 FUKUCH 3   b   0        192    1048      64    1039      2.97 (  2.21-   4.00)
 HOZAWA 3   b   0       2589    5379     872    5019      2.77 (  2.54-   3.02)
 HUHTI2 5   m   0         48     299       0      76     24.78~(  1.51- 406.36)
 HUHTI2 6   f   0          3      74      15     477      1.29 (  0.36-   4.56)
 Subtotal HUHTI2                                          2.13 (  0.67-   6.73)
 HUHTI3 6   m   1         84       -       3       -      7.74 (  2.62-  22.87)
 ITABAS 1   b   0         44      35      16      25      1.96 (  0.91-   4.24)
 JOHANN 3   b   0        134    1237      22     842      4.15 (  2.62-   6.56)
 KACHEL 3   b   0         41     688       7     503      4.28 (  1.91-   9.62)
 KATANC 3   b   0         54     403      21     382      2.44 (  1.44-   4.11)
 KIM    3   m   1        179       -      23       -      2.31 (  1.44-   3.72)
 KIM    6   f   1         10       -      61       -      1.74 (  0.85-   3.57)
 Subtotal KIM                                             2.12 (  1.43-   3.15)
 KLAYTO 1   b   0         62     117       7      76      5.75 (  2.50-  13.24)
 KOJIMA 3   m   0        169    5515      19    1871      3.02 (  1.87-   4.86)
 KOJIMA 6   f   0          5     316      20    3545      2.80 (  1.05-   7.52)
 Subtotal KOJIMA                                          2.98 (  1.94-   4.57)
*KRZYZA 3   m   1         57       -       5       -      2.63 (  1.09-   6.38)
*KRZYZA 6   f   1         19       -      32       -      1.41 (  0.82-   2.41)
 Subtotal KRZYZA                                          1.67 (  1.05-   2.65)
 LAI    1   m   0        107     469      20     493      5.62 (  3.43-   9.22)
 LAI    2   f   0          7      88      27     600      1.77 (  0.75-   4.18)
 Subtotal LAI                                             4.22 (  2.75-   6.48)
 LEBOWI 6   m   1         41       -       4       -      5.02 (  1.76-  14.35)
 LEBOWI 18  f   1         50       -      36       -      1.66 (  1.07-   2.59)
 Subtotal LEBOWI                                          1.96 (  1.31-   2.95)
*LINDBE 3   b   3          -       -       -       -      2.28 (  1.34-   3.88)
 LUNDB1 9   b   4        138       -      39       -      3.16 (  2.01-   4.97)
 MADOR  3   m   0         19       7       0       3     18.20~(  0.84- 396.21)
 MANNI1 1   m   2        480       -     105       -      2.69 (  2.16-   3.34)
 MANNI1 10  f   2        305       -     156       -      3.18 (  2.61-   3.88)
 Subtotal MANNI1                                          2.95 (  2.55-   3.41)
 MANNI2 3   b   1        752       -     202       -      2.64 (  2.24-   3.12)
 MATHES 3   b   0         49      89      23     131      3.14 (  1.78-   5.51)
 MENEZ2 6   b   5        100       -      52       -      1.54 (  1.08-   2.17)
 MENEZ3 3   b   0        136       -      62       -      1.11 (  0.80-   1.54)
 MENEZ4 3   b   0         44       -      34       -      1.65 (  1.04-   2.63)
 MENEZ5 3   b   0        115       -      58       -      1.64 (  1.16-   2.33)
 MENEZ6 3   b   0        121       -      36       -      2.73 (  1.85-   4.03)
 MUELLE 6   m   1         35       -       2       -      4.41 (  1.01-  19.20)
 MUELLE 13  f   1          3       -       2       -      1.60 (  0.26-   9.92)
 Subtotal MUELLE                                          2.95 (  0.94-   9.28)
 NIEPSU 3   b   0         34     191       8     177      3.94 (  1.78-   8.74)
 PEREZP 1   f   0         19      47      45     145      1.30 (  0.69-   2.44)
 RENWIC 3   b   0         59     119       9      60      3.31 (  1.54-   7.12)
 RICCIO 3   b   0        321     478     178     537      2.03 (  1.62-   2.53)
 SHAHAB 3   b   0        771    3758     322    3364      2.14 (  1.87-   2.46)
 SHIN   5   b   3         71       -      49       -      1.87 (  1.14-   3.09)
 STROM  3   m   0        157     233      17      71      2.81 (  1.60-   4.96)
 TSUSHI 3   b   0         41    1142       7    1056      5.42 (  2.42-  12.12)
 VESTBO 3   b   0       1667    8841     234    2366      1.91 (  1.65-   2.20)
 VIEGI2 6   m   1        168       -      21       -      1.39 (  0.83-   2.32)
 VIEGI2 12  f   1         52       -      66       -      1.20 (  0.80-   1.80)
 Subtotal VIEGI2                                          1.27 (  0.92-   1.75)
 WEISS  3   m   0         42     209       2      34      3.42 (  0.79-  14.77)
 WILSO1 1   b   0        112    1040      24     875      3.93 (  2.50-   6.16)
 YAMAGU 3   b   6        332       -     144       -      1.97 (  1.60-   2.43)
 ZIELI1 2   b   1       2364       -     316       -      2.06 (  1.81-   2.35)
 ZIELI2 3   m   0      11618   36365     341    2611      2.45 (  2.18-   2.74)
 ZIELI2 6   f   0       5159   26196     679    4832      1.40 (  1.29-   1.53)
  ________________________________________________________________________________________________________________________
                                            International Evidence on Smoking and COPD, Phase 3, Analysis run on 27-SEP-10

                                                   Table 1 - A - 4 - 2

            IESCOPD - Meta-analysis of ever smoking, any product (or cigarettes if all product not available)
                                             COPD based on lung function only
                                                      Most-adjusted


                        Number Exposed  Non-exposed
 REF    NRR SEX ADJ     Case    Cont    Case    Cont      RR        95.00%CI
 Subtotal ZIELI2                                          1.71 (  1.60-   1.83)
 ZIETKO 2   m   0         35      11       0      12     77.17~(  4.23-1408.01)
 ZIETKO 4   f   0         12       6       0      11     44.23~(  2.23- 875.91)
 Subtotal ZIETKO                                         58.88 (  7.34- 472.11)
Partial Totals         32617  107727    5259   37762
*prospective study                                        ~ With 0.5 adjustment for zero

                       N       62
                      NS       49


                      Wt  3472.35
                 Het Chi   308.99
                 Het  df       61
                 Het  P       ***
               Fixed  RR     2.16
                     RRl     2.09
                     RRu     2.23
                      P       +++
              Random  RR     2.35
                     RRl     2.13
                     RRu     2.59
                      P       +++
               Asymm  P         *


  ________________________________________________________________________________________________________________________
                                            International Evidence on Smoking and COPD, Phase 3, Analysis run on 27-SEP-10

                                                   Table 1 - A - 4 - 3

            IESCOPD - Meta-analysis of ever smoking, any product (or cigarettes if all product not available)
                                             COPD based on lung function only
                                                      Most-adjusted


                       N       62
                      NS       49


                      Wt  3472.35
                 Het Chi   308.99
                 Het  df       61
                 Het  P       ***
               Fixed  RR     2.16
                     RRl     2.09
                     RRu     2.23
                      P       +++
              Random  RR     2.35
                     RRl     2.13
                     RRu     2.59
                      P       +++
               Asymm  P         *

                                   Sex
                             both      male    female     Total


                       N       30        18        14        62
                      NS       30        18        14        62


                      Wt  1883.56    763.94    824.85   3472.35
                 Het Chi   114.21     43.19     87.69    308.99
                 Het  df       29        17        13        61
                 Het  P       ***       ***       ***       ***
               Fixed  RR     2.30      2.40      1.70      2.16
                     RRl     2.20      2.24      1.58      2.09
                     RRu     2.40      2.58      1.82      2.23
                      P       +++       +++       +++       +++
              Random  RR     2.33      2.77      1.83      2.35
                     RRl     2.08      2.32      1.38      2.13
                     RRu     2.61      3.32      2.43      2.59
                      P       +++       +++       +++       +++
             Between Chi                                  63.91
             Between  df                                      2
             Between  P                                     ***
             Btwn(F)  P                                      **

                                        Continent
                            NAmer    Europe      Asia  oth/mult     Total


                       N       13        29        11         9        62
                      NS       10        22         8         9        49


                      Wt   888.24   2079.44    226.09    278.58   3472.35
                 Het Chi    16.41    150.04     24.16     30.28    308.99
                 Het  df       12        28        10         8        61
                 Het  P      N.S.       ***        **       ***       ***
               Fixed  RR     2.77      1.96      2.45      1.80      2.16
                     RRl     2.60      1.88      2.15      1.60      2.09
                     RRu     2.96      2.05      2.79      2.02      2.23
                      P       +++       +++       +++       +++       +++
              Random  RR     2.78      2.26      2.61      1.89      2.35
                     RRl     2.49      1.97      2.06      1.48      2.13
                     RRu     3.10      2.60      3.31      2.42      2.59
                      P       +++       +++       +++       +++       +++
             Between Chi                                            88.10
             Between  df                                                3
             Between  P                                               ***
             Btwn(F)  P                                               ***


  ________________________________________________________________________________________________________________________
                                            International Evidence on Smoking and COPD, Phase 3, Analysis run on 27-SEP-10

                                                   Table 1 - A - 4 - 3

            IESCOPD - Meta-analysis of ever smoking, any product (or cigarettes if all product not available)
                                             COPD based on lung function only
                                                      Most-adjusted
                        National cigarette tobacco type (excluding mixed/unkown)
                          blended  virginia     Total


                       N       53         5        58
                      NS       41         5        46


                      Wt  3027.87    239.92   3267.79
                 Het Chi   274.11     13.91    289.57
                 Het  df       52         4        57
                 Het  P       ***        **       ***
               Fixed  RR     2.15      2.34      2.17
                     RRl     2.08      2.06      2.09
                     RRu     2.23      2.66      2.24
                      P       +++       +++       +++
              Random  RR     2.29      3.22      2.35
                     RRl     2.06      2.09      2.12
                     RRu     2.56      4.97      2.60
                      P       +++       +++       +++
             Between Chi                         1.55
             Between  df                            1
             Between  P                          N.S.
             Btwn(F)  P                          N.S.

                                        Start year of study
                            <1970   1970-79   1980-89   1990-99     2000+   unknown     Total


                       N        7         8         7         9        20        11        62
                      NS        5         5         5         9        16         9        49


                      Wt    35.19    757.04    837.82    417.34   1391.35     33.61   3472.35
                 Het Chi     9.25     19.39     32.65     18.79    138.83     19.08    308.99
                 Het  df        6         7         6         8        19        10        61
                 Het  P      N.S.        **       ***         *       ***         *       ***
               Fixed  RR     2.26      2.20      2.62      2.09      1.89      4.13      2.16
                     RRl     1.63      2.05      2.44      1.90      1.79      2.95      2.09
                     RRu     3.15      2.37      2.80      2.30      1.99      5.79      2.23
                      P       +++       +++       +++       +++       +++       +++       +++
              Random  RR     2.54      2.24      2.27      2.26      2.24      5.74      2.35
                     RRl     1.61      1.92      1.84      1.86      1.88      3.19      2.13
                     RRu     3.99      2.62      2.80      2.74      2.67     10.32      2.59
                      P       +++       +++       +++       +++       +++       +++       +++
             Between Chi                                                                71.00
             Between  df                                                                    5
             Between  P                                                                   ***
             Btwn(F)  P                                                                     *

                                Publication year
                            <1980   1980-89   1990-99     2000+     Total


                       N        7         6         5        44        62
                      NS        5         4         5        35        49


                      Wt    36.68    118.08     35.66   3281.92   3472.35
                 Het Chi    13.61      5.61     10.17    275.81    308.99
                 Het  df        6         5         4        43        61
                 Het  P         *      N.S.         *       ***       ***
               Fixed  RR     2.77      1.94      2.34      2.16      2.16
                     RRl     2.00      1.62      1.68      2.09      2.09
                     RRu     3.83      2.33      3.24      2.23      2.23
                      P       +++       +++       +++       +++       +++
              Random  RR     3.63      1.94      2.57      2.33      2.35
                     RRl     2.02      1.53      1.47      2.09      2.13
                     RRu     6.51      2.45      4.52      2.60      2.59
                      P       +++       +++        ++       +++       +++
             Between Chi                                             3.78
             Between  df                                                3
             Between  P                                              N.S.
             Btwn(F)  P                                              N.S.
  ________________________________________________________________________________________________________________________
                                            International Evidence on Smoking and COPD, Phase 3, Analysis run on 27-SEP-10

                                                   Table 1 - A - 4 - 3

            IESCOPD - Meta-analysis of ever smoking, any product (or cigarettes if all product not available)
                                             COPD based on lung function only
                                                      Most-adjusted
                               Study type
                               CC        Pr        CS     Total


                       N        8         4        50        62
                      NS        6         3        40        49


                      Wt    30.48     40.79   3401.08   3472.35
                 Het Chi    18.40      2.34    287.44    308.99
                 Het  df        7         3        49        61
                 Het  P         *      N.S.       ***       ***
               Fixed  RR     2.45      1.98      2.16      2.16
                     RRl     1.72      1.46      2.09      2.09
                     RRu     3.50      2.69      2.23      2.23
                      P       +++       +++       +++       +++
              Random  RR     3.83      1.98      2.35      2.35
                     RRl     1.76      1.46      2.12      2.13
                     RRu     8.32      2.69      2.61      2.59
                      P       +++       +++       +++       +++
             Between Chi                                   0.81
             Between  df                                      2
             Between  P                                    N.S.
             Btwn(F)  P                                    N.S.

                                    Lowest age in RR
                        <25/unlim     25-39       40+   unknown     Total


                       N       29        10        23                  62
                      NS       20         8        21                  49


                      Wt  2034.57    513.46    924.32             3472.35
                 Het Chi   179.38     32.62     72.21              308.99
                 Het  df       28         9        22                  61
                 Het  P       ***       ***       ***                 ***
               Fixed  RR     2.02      2.25      2.45                2.16
                     RRl     1.93      2.06      2.30                2.09
                     RRu     2.11      2.45      2.61                2.23
                      P       +++       +++       +++                 +++
              Random  RR     2.49      2.24      2.26                2.35
                     RRl     2.14      1.82      1.91                2.13
                     RRu     2.90      2.76      2.66                2.59
                      P       +++       +++       +++                 +++
             Between Chi                                            24.78
             Between  df                                                2
             Between  P                                               ***
             Btwn(F)  P                                               (*)

                                         Highest age in RR
                              <65     65-74     75-84 85+/unlim   unknown     Total


                       N        3        15         9        35                  62
                      NS        3        11         7        28                  49


                      Wt   626.02    240.63    109.52   2496.18             3472.35
                 Het Chi    16.80     34.65     19.61    189.29              308.99
                 Het  df        2        14         8        34                  61
                 Het  P       ***        **         *       ***                 ***
               Fixed  RR     2.58      2.49      2.97      2.01                2.16
                     RRl     2.38      2.19      2.46      1.93                2.09
                     RRu     2.79      2.82      3.58      2.09                2.23
                      P       +++       +++       +++       +++                 +++
              Random  RR     2.24      2.66      2.88      2.20                2.35
                     RRl     1.54      2.00      2.13      1.95                2.13
                     RRu     3.25      3.54      3.91      2.47                2.59
                      P       +++       +++       +++       +++                 +++
             Between Chi                                                      48.66
             Between  df                                                          3
             Between  P                                                         ***
             Btwn(F)  P                                                           *
  ________________________________________________________________________________________________________________________
                                            International Evidence on Smoking and COPD, Phase 3, Analysis run on 27-SEP-10

                                                   Table 1 - A - 4 - 3

            IESCOPD - Meta-analysis of ever smoking, any product (or cigarettes if all product not available)
                                             COPD based on lung function only
                                                      Most-adjusted
                           Study weakness
                              Yes        No     Total


                       N        8        54        62
                      NS        6        43        49


                      Wt    93.64   3378.71   3472.35
                 Het Chi    18.20    290.68    308.99
                 Het  df        7        53        61
                 Het  P         *       ***       ***
               Fixed  RR     2.24      2.16      2.16
                     RRl     1.83      2.09      2.09
                     RRu     2.74      2.23      2.23
                      P       +++       +++       +++
              Random  RR     4.22      2.32      2.35
                     RRl     2.08      2.10      2.13
                     RRu     8.58      2.56      2.59
                      P       +++       +++       +++
             Between Chi                         0.12
             Between  df                            1
             Between  P                          N.S.
             Btwn(F)  P                          N.S.

                          COPD subtype
                             mort        LF     other     Total


                       N                 62                  62
                      NS                 49                  49


                      Wt            3472.35             3472.35
                 Het Chi             308.99              308.99
                 Het  df                 61                  61
                 Het  P                 ***                 ***
               Fixed  RR               2.16                2.16
                     RRl               2.09                2.09
                     RRu               2.23                2.23
                      P                 +++                 +++
              Random  RR               2.35                2.35
                     RRl               2.13                2.13
                     RRu               2.59                2.59
                      P                 +++                 +++
             Between Chi
             Between  df
             Between  P                                    N.S.
             Btwn(F)  P                                    N.S.

                               Asthma analysis type (COPD)
                        inc-irres  excl-all defn-incl     other     Total


                       N       47        14                   1        62
                      NS       37        11                   1        49


                      Wt  2323.56   1136.70               12.08   3472.35
                 Het Chi   155.60     95.41                0.00    308.99
                 Het  df       46        13                   0        61
                 Het  P       ***       ***                N.S.       ***
               Fixed  RR     2.36      1.80                3.14      2.16
                     RRl     2.26      1.69                1.78      2.09
                     RRu     2.45      1.90                5.51      2.23
                      P       +++       +++                 +++       +++
              Random  RR     2.35      2.25                3.14      2.35
                     RRl     2.14      1.77                1.78      2.13
                     RRu     2.60      2.88                5.51      2.59
                      P       +++       +++                 +++       +++
             Between Chi                                            57.98
             Between  df                                                2
             Between  P                                               ***
             Btwn(F)  P                                                **
  ________________________________________________________________________________________________________________________
                                            International Evidence on Smoking and COPD, Phase 3, Analysis run on 27-SEP-10

                                                   Table 1 - A - 4 - 3

            IESCOPD - Meta-analysis of ever smoking, any product (or cigarettes if all product not available)
                                             COPD based on lung function only
                                                      Most-adjusted
                        Bronchodilator/reversibility (LF only)
                          no/unkn  yes/revs     Total


                       N       50        12        62
                      NS       38        11        49


                      Wt  3266.48    205.87   3472.35
                 Het Chi   246.61     62.38    308.99
                 Het  df       49        11        61
                 Het  P       ***       ***       ***
               Fixed  RR     2.16      2.15      2.16
                     RRl     2.09      1.87      2.09
                     RRu     2.23      2.46      2.23
                      P       +++       +++       +++
              Random  RR     2.32      2.79      2.35
                     RRl     2.09      1.93      2.13
                     RRu     2.57      4.03      2.59
                      P       +++       +++       +++
             Between Chi                         0.00
             Between  df                            1
             Between  P                          N.S.
             Btwn(F)  P                          N.S.

                                  Number of COPD cases
                             1-50    51-100   101-200      201+     Total


                       N       12        14        17        19        62
                      NS        9        13        13        14        49


                      Wt    25.69    114.66    290.72   3041.28   3472.35
                 Het Chi    12.81     25.01     64.68    186.43    308.99
                 Het  df       11        13        16        18        61
                 Het  P      N.S.         *       ***       ***       ***
               Fixed  RR     5.11      2.28      2.22      2.13      2.16
                     RRl     3.47      1.90      1.98      2.06      2.09
                     RRu     7.52      2.74      2.49      2.21      2.23
                      P       +++       +++       +++       +++       +++
              Random  RR     5.32      2.44      2.41      2.15      2.35
                     RRl     3.39      1.86      1.89      1.90      2.13
                     RRu     8.35      3.21      3.07      2.44      2.59
                      P       +++       +++       +++       +++       +++
             Between Chi                                            20.06
             Between  df                                                3
             Between  P                                               ***
             Btwn(F)  P                                              N.S.

                            Analysis type
                         prevlnce     onset     Total


                       N       59         3        62
                      NS       47         2        49


                      Wt  3440.61     31.74   3472.35
                 Het Chi   306.36      2.15    308.99
                 Het  df       58         2        61
                 Het  P       ***      N.S.       ***
               Fixed  RR     2.16      1.91      2.16
                     RRl     2.09      1.35      2.09
                     RRu     2.23      2.70      2.23
                      P       +++       +++       +++
              Random  RR     2.37      1.91      2.35
                     RRl     2.14      1.33      2.13
                     RRu     2.62      2.75      2.59
                      P       +++       +++       +++
             Between Chi                         0.49
             Between  df                            1
             Between  P                          N.S.
             Btwn(F)  P                          N.S.
  ________________________________________________________________________________________________________________________
                                            International Evidence on Smoking and COPD, Phase 3, Analysis run on 27-SEP-10

                                                   Table 1 - A - 4 - 3

            IESCOPD - Meta-analysis of ever smoking, any product (or cigarettes if all product not available)
                                             COPD based on lung function only
                                                      Most-adjusted
                             Smoking product
                              any      cigs  cigsonly     Total


                       N       23        39                  62
                      NS       18        32                  50


                      Wt  1014.42   2457.92             3472.35
                 Het Chi    61.35    243.55              308.99
                 Het  df       22        38                  61
                 Het  P       ***       ***                 ***
               Fixed  RR     2.28      2.11                2.16
                     RRl     2.14      2.03                2.09
                     RRu     2.42      2.20                2.23
                      P       +++       +++                 +++
              Random  RR     2.53      2.25                2.35
                     RRl     2.19      1.98                2.13
                     RRu     2.93      2.56                2.59
                      P       +++       +++                 +++
             Between Chi                                   4.09
             Between  df                                      1
             Between  P                                       *
             Btwn(F)  P                                    N.S.

                                     Unexposed group
                          nev any   nev cig  nev+ any  nev+ cig     Total


                       N       24        37                   1        62
                      NS       18        30                   1        49


                      Wt  1112.17   2354.64                5.53   3472.35
                 Het Chi    71.30    220.72                0.00    308.99
                 Het  df       23        36                   0        61
                 Het  P       ***       ***                N.S.       ***
               Fixed  RR     2.34      2.07                5.75      2.16
                     RRl     2.21      1.99                2.50      2.09
                     RRu     2.49      2.16               13.24      2.23
                      P       +++       +++                 +++       +++
              Random  RR     2.59      2.18                5.75      2.35
                     RRl     2.25      1.92                2.50      2.13
                     RRu     2.99      2.49               13.24      2.59
                      P       +++       +++                 +++       +++
             Between Chi                                            16.98
             Between  df                                                2
             Between  P                                               ***
             Btwn(F)  P                                              N.S.

                        Unexposed group (combining nev+ with main levels)
                          nev any   nev cig     Total


                       N       24        38        62
                      NS       18        31        49


                      Wt  1112.17   2360.18   3472.35
                 Het Chi    71.30    226.48    308.99
                 Het  df       23        37        61
                 Het  P       ***       ***       ***
               Fixed  RR     2.34      2.08      2.16
                     RRl     2.21      1.99      2.09
                     RRu     2.49      2.16      2.23
                      P       +++       +++       +++
              Random  RR     2.59      2.22      2.35
                     RRl     2.25      1.95      2.13
                     RRu     2.99      2.53      2.59
                      P       +++       +++       +++
             Between Chi                        11.21
             Between  df                            1
             Between  P                           ***
             Btwn(F)  P                          N.S.
  ________________________________________________________________________________________________________________________
                                            International Evidence on Smoking and COPD, Phase 3, Analysis run on 27-SEP-10

                                                   Table 1 - A - 4 - 3

            IESCOPD - Meta-analysis of ever smoking, any product (or cigarettes if all product not available)
                                             COPD based on lung function only
                                                      Most-adjusted
                        Smoking results reported in study (COPD)
                             Ever   Current      Both     Total


                       N        7                  55        62
                      NS        6                  43        49


                      Wt   287.06             3185.29   3472.35
                 Het Chi    29.02              279.15    308.99
                 Het  df        6                  54        61
                 Het  P       ***                 ***       ***
               Fixed  RR     2.27                2.15      2.16
                     RRl     2.02                2.08      2.09
                     RRu     2.55                2.22      2.23
                      P       +++                 +++       +++
              Random  RR     2.76                2.30      2.35
                     RRl     1.83                2.08      2.13
                     RRu     4.17                2.55      2.59
                      P       +++                 +++       +++
             Between Chi                                   0.83
             Between  df                                      1
             Between  P                                    N.S.
             Btwn(F)  P                                    N.S.

                        Number of adjustment variables
                                0         1        2+     Total


                       N       41        13         8        62
                      NS       34         8         7        49


                      Wt  2555.44    475.29    441.62   3472.35
                 Het Chi   248.63     30.19     28.78    308.99
                 Het  df       40        12         7        61
                 Het  P       ***        **       ***       ***
               Fixed  RR     2.14      2.14      2.27      2.16
                     RRl     2.06      1.95      2.07      2.09
                     RRu     2.23      2.34      2.50      2.23
                      P       +++       +++       +++       +++
              Random  RR     2.51      2.03      2.24      2.35
                     RRl     2.20      1.66      1.82      2.13
                     RRu     2.87      2.49      2.75      2.59
                      P       +++       +++       +++       +++
             Between Chi                                   1.39
             Between  df                                      2
             Between  P                                    N.S.
             Btwn(F)  P                                    N.S.

                        RR adjusted for sex (combined sex RR only)
                              Yes        No     Total


                       N        6        24        30
                      NS        6        24        30


                      Wt   262.99   1620.57   1883.56
                 Het Chi     7.37     96.20    114.21
                 Het  df        5        23        29
                 Het  P      N.S.       ***       ***
               Fixed  RR     1.91      2.37      2.30
                     RRl     1.69      2.26      2.20
                     RRu     2.15      2.49      2.40
                      P       +++       +++       +++
              Random  RR     1.94      2.45      2.33
                     RRl     1.65      2.15      2.08
                     RRu     2.28      2.79      2.61
                      P       +++       +++       +++
             Between Chi                        10.63
             Between  df                            1
             Between  P                            **
             Btwn(F)  P                          N.S.
  ________________________________________________________________________________________________________________________
                                            International Evidence on Smoking and COPD, Phase 3, Analysis run on 27-SEP-10

                                                   Table 1 - A - 4 - 3

            IESCOPD - Meta-analysis of ever smoking, any product (or cigarettes if all product not available)
                                             COPD based on lung function only
                                                      Most-adjusted
                        RR adjusted for age
                              Yes        No     Total


                       N       20        42        62
                      NS       14        35        49


                      Wt   821.27   2651.08   3472.35
                 Het Chi    54.49    252.21    308.99
                 Het  df       19        41        61
                 Het  P       ***       ***       ***
               Fixed  RR     2.26      2.13      2.16
                     RRl     2.11      2.05      2.09
                     RRu     2.42      2.21      2.23
                      P       +++       +++       +++
              Random  RR     2.17      2.47      2.35
                     RRl     1.88      2.17      2.13
                     RRu     2.51      2.81      2.59
                      P       +++       +++       +++
             Between Chi                         2.29
             Between  df                            1
             Between  P                          N.S.
             Btwn(F)  P                          N.S.

                        RR adjusted for factor other than sex, age


                       N        8        54        62
                      NS        7        42        49


                      Wt   441.62   3030.73   3472.35
                 Het Chi    28.78    278.83    308.99
                 Het  df        7        53        61
                 Het  P       ***       ***       ***
               Fixed  RR     2.27      2.14      2.16
                     RRl     2.07      2.07      2.09
                     RRu     2.50      2.22      2.23
                      P       +++       +++       +++
              Random  RR     2.24      2.38      2.35
                     RRl     1.82      2.13      2.13
                     RRu     2.75      2.66      2.59
                      P       +++       +++       +++
             Between Chi                         1.39
             Between  df                            1
             Between  P                          N.S.
             Btwn(F)  P                          N.S.

                        Derivation of RR/CI
                         Orig/2x2     Other     Total


                       N        9        53        62
                      NS        9        44        53


                      Wt   266.94   3205.40   3472.35
                 Het Chi    16.37    292.28    308.99
                 Het  df        8        52        61
                 Het  P         *       ***       ***
               Fixed  RR     2.23      2.15      2.16
                     RRl     1.98      2.08      2.09
                     RRu     2.52      2.23      2.23
                      P       +++       +++       +++
              Random  RR     2.39      2.35      2.35
                     RRl     1.80      2.11      2.13
                     RRu     3.16      2.61      2.59
                      P       +++       +++       +++
             Between Chi                         0.34
             Between  df                            1
             Between  P                          N.S.
             Btwn(F)  P                          N.S.

  ________________________________________________________________________________________________________________________
                                            International Evidence on Smoking and COPD, Phase 3, Analysis run on 27-SEP-10

                                                   Table 1 - A - 4 - 4

            IESCOPD - Meta-analysis of ever smoking, any product (or cigarettes if all product not available)
                                             COPD based on lung function only
                                                      Least-adjusted


     REF|NRR|X|SEX|AGEL|AGEH|     REGION|BEGYR|PUBYR|STTYP|ONSET|      DISEAS|ADJ|SMOKSTA|   PRODUCT|    UNEXP|

  ANDER3   3     m    0   99     Eu:East     *  2001    CC  Prev      COPD:LF   0    Ever        Any   Nev any
  ANDER3   6     f    0   99     Eu:East     *  2001    CC  Prev      COPD:LF   0    Ever        Any   Nev any
  BEDNAR   3     b   30   99     Eu:East  2000  2005    CS  Prev      COPD:LF   0    Ever        Any   Nev any
  CLEMEN   3     m   20   60     Eu:West  1960  1982    Pr  Prev      COPD:LF   0    Ever        Any   Nev any
  DEJONG   3     b    0   99      Am:USA     *  2004    CS  Prev      COPD:LF   0    Ever       Cigs  Nev cigs
  DEMARC   3 x   b   20   44       Multi  1991  2004    CS  Prev      COPD:LF   0    Ever        Any   Nev any
  DICKIN   3     b   60   74       Eu:UK     *  1999    CS  Prev      COPD:LF   0    Ever        Any   Nev any
  EKBERG   3     m   15   99    Eu:Scand  1974  2005    CS  Prev      COPD:LF   0    Ever        Any   Nev any
  EKBERG   6     f   15   99    Eu:Scand  1974  2005    CS  Prev      COPD:LF   0    Ever        Any   Nev any
  FUKUCH   3     b   40   99   Asia:FarE  2000  2004    CS  Prev      COPD:LF   0    Ever       Cigs  Nev cigs
  HOZAWA   3     b   45   64      Am:USA  1987  2006    CS  Prev      COPD:LF   0    Ever       Cigs  Nev cigs
  HUHTI2   5     m   50   74    Eu:Scand  1971  1980    CS  Prev      COPD:LF   0    Ever        Any   Nev any
  HUHTI2   6     f   50   74    Eu:Scand  1971  1980    CS  Prev      COPD:LF   0    Ever        Any   Nev any
  HUHTI3   3 x   m   25   69    Eu:Scand  1968  1978    CS  Prev      COPD:LF   0    Ever        Any   Nev any
  ITABAS   1     b   60   99   Asia:FarE     *  1990    CC  Prev      COPD:LF   0    Ever       Cigs  Nev cigs
  JOHANN   3     b   26   82    Eu:Scand  1996  2005    CS  Prev      COPD:LF   0    Ever       Cigs  Nev cigs
  KACHEL   3     b   20   79     Eu:East     *  2003    CS  Prev      COPD:LF   0    Ever       Cigs  Nev cigs
  KATANC   3     b   70   79      Am:USA  1997  2005    CS  Prev      COPD:LF   0    Ever       Cigs  Nev cigs
     KIM   3     m   45   99   Asia:FarE  2001  2005    CS  Prev      COPD:LF   1    Ever       Cigs  Nev cigs
     KIM   6     f   45   99   Asia:FarE  2001  2005    CS  Prev      COPD:LF   1    Ever       Cigs  Nev cigs
  KLAYTO   1     b   40   99      Am:USA     *  1975    CS  Prev      COPD:LF   0    Ever       Cigs  N/L cigs
  KOJIMA   3     m   25   74   Asia:FarE  2001  2005    CS  Prev      COPD:LF   0    Ever       Cigs  Nev cigs
  KOJIMA   6     f   25   74   Asia:FarE  2001  2005    CS  Prev      COPD:LF   0    Ever       Cigs  Nev cigs
  KRZYZA   3     m   19   83     Eu:East  1968  1986    Pr   Inc      COPD:LF   1    Ever       Cigs  Nev cigs
  KRZYZA   6     f   19   83     Eu:East  1968  1986    Pr   Inc      COPD:LF   1    Ever       Cigs  Nev cigs
     LAI   1     m   18   80   Asia:FarE  2001  2006    CS  Prev      COPD:LF   0    Ever        Any   Nev any
     LAI   2     f   18   80   Asia:FarE  2001  2006    CS  Prev      COPD:LF   0    Ever        Any   Nev any
  LEBOWI   3 x   m   15   96      Am:USA  1972  1977    CS  Prev      COPD:LF   0    Ever       Cigs  Nev cigs
  LEBOWI  15 x   f   15   96      Am:USA  1972  1977    CS  Prev      COPD:LF   0    Ever       Cigs  Nev cigs
  LINDBE   3     b   46   84    Eu:Scand  1996  2006    Pr   Inc      COPD:LF   3    Ever        Any   Nev any
  LUNDB1   3 x   m   46   77    Eu:Scand  1996  2003    CS  Prev      COPD:LF   0    Ever       Cigs  Nev cigs
  LUNDB1   6 x   f   46   77    Eu:Scand  1996  2003    CS  Prev      COPD:LF   0    Ever       Cigs  Nev cigs
   MADOR   3     m   15   99      Am:USA     *  2003    CC  Prev      COPD:LF   0    Ever       Cigs  Nev cigs
  MANNI1   1     m   17   99      Am:USA  1988  2000    CS  Prev      COPD:LF   2    Ever        Any   Nev any
  MANNI1  10     f   17   99      Am:USA  1988  2000    CS  Prev      COPD:LF   2    Ever       Cigs   Nev any
  MANNI2   3     b   25   74      Am:USA  1971  2003    CS  Prev      COPD:LF   1    Ever        Any   Nev any
  MATHES   3     b   45   70      Aus/NZ     *  2006    CC  Prev      COPD:LF   0    Ever       Cigs  Nev cigs
  MENEZ2   3 x   b   40   99 Am:Sth/Cent  2003  2005    CS  Prev      COPD:LF   0    Ever       Cigs  Nev cigs
  MENEZ3   3     b   40   99 Am:Sth/Cent  2003  2005    CS  Prev      COPD:LF   0    Ever       Cigs  Nev cigs
  MENEZ4   3     b   40   99 Am:Sth/Cent  2003  2005    CS  Prev      COPD:LF   0    Ever       Cigs  Nev cigs
  MENEZ5   3     b   40   99 Am:Sth/Cent  2003  2005    CS  Prev      COPD:LF   0    Ever       Cigs  Nev cigs
  MENEZ6   3     b   40   99 Am:Sth/Cent  2003  2005    CS  Prev      COPD:LF   0    Ever       Cigs  Nev cigs
  MUELLE   3 x   m   20   69      Am:USA  1967  1971    CS  Prev      COPD:LF   0    Ever        Any   Nev any
  MUELLE  11 x   f   20   69      Am:USA  1967  1971    CS  Prev      COPD:LF   0    Ever        Any   Nev any
  NIEPSU   3     b   19   69     Eu:East  2001  2002    CS  Prev      COPD:LF   0    Ever       Cigs  Nev cigs
  PEREZP   1     f   40   99 Am:Sth/Cent  1992  1996    CC  Prev      COPD:LF   0    Ever       Cigs  Nev cigs
  RENWIC   3     b   45   99       Eu:UK  1992  1996    CS  Prev      COPD:LF   0    Ever        Any   Nev any
  RICCIO   3     b   25   99     Eu:West  2002  2005    CS  Prev      COPD:LF   0    Ever       Cigs  Nev cigs
  SHAHAB   3     b   35   99       Eu:UK  2001  2006    CS  Prev      COPD:LF   0    Ever       Cigs  Nev cigs
    SHIN   3 x   m   18   99   Asia:FarE  1999  2003    CS  Prev      COPD:LF   0    Ever       Cigs  Nev cigs
    SHIN   4 x   f   18   99   Asia:FarE  1999  2003    CS  Prev      COPD:LF   0    Ever       Cigs  Nev cigs
   STROM   3     m   68   68    Eu:Scand  1982  1996    CS  Prev      COPD:LF   0    Ever        Any   Nev any
  TSUSHI   3     b    0   99   Asia:FarE  2003  2006    CS  Prev      COPD:LF   0    Ever       Cigs  Nev cigs
  VESTBO   3     b   20   99    Eu:Scand  1976  2002    CS  Prev      COPD:LF   0    Ever        Any   Nev any
  VIEGI2   3 x   m   25   73     Eu:West  1988  2000    CS  Prev      COPD:LF   0    Ever       Cigs  Nev cigs
  VIEGI2   9 x   f   25   73     Eu:West  1988  2000    CS  Prev      COPD:LF   0    Ever       Cigs  Nev cigs
   WEISS   3     m   50   69      Am:USA  1961  1963    CS  Prev      COPD:LF   0    Ever        Any   Nev any
  WILSO1   1     b   18   99      Aus/NZ  2000  2005    CS  Prev      COPD:LF   0    Ever        Any   Nev any
  YAMAGU   3     b   40   99   Asia:FarE  1986  1988    CS  Prev      COPD:LF   6    Ever       Cigs  Nev cigs
  ZIELI1   1 x   b    0   99     Eu:East  1999  2001    CS  Prev      COPD:LF   0    Ever       Cigs  Nev cigs
  ZIELI2   3     m    0   99     Eu:East  2000  2006    CS  Prev      COPD:LF   0    Ever       Cigs  Nev cigs
  ZIELI2   6     f    0   99     Eu:East  2000  2006    CS  Prev      COPD:LF   0    Ever       Cigs  Nev cigs
  ZIETKO   2     m    0   99     Eu:East     *  2005    CC  Prev      COPD:LF   0    Ever       Cigs  Nev cigs
  ZIETKO   4     f    0   99     Eu:East     *  2005    CC  Prev      COPD:LF   0    Ever       Cigs  Nev cigs


  ________________________________________________________________________________________________________________________
                                            International Evidence on Smoking and COPD, Phase 3, Analysis run on 27-SEP-10

                                                   Table 1 - A - 4 - 5

            IESCOPD - Meta-analysis of ever smoking, any product (or cigarettes if all product not available)
                                             COPD based on lung function only
                                                      Least-adjusted


                        Number Exposed  Non-exposed
 REF    NRR SEX ADJ     Case    Cont    Case    Cont      RR        95.00%CI
 ANDER3 3   m   0         15       8       0       8     31.00~(  1.59- 605.65)
 ANDER3 6   f   0          2       1       3       3      2.00 (  0.11-  35.81)
 Subtotal ANDER3                                          7.56 (  0.95-  59.91)
 BEDNAR 3   b   0         52     376      20     228      1.58 (  0.92-   2.71)
 CLEMEN 3   m   0         57    1629      11     709      2.26 (  1.18-   4.33)
 DEJONG 3   b   0         55     154       0      34     24.79~(  1.49- 411.13)
 DEMARC 3   b   0        381    8144     156    6176      1.85 (  1.53-   2.24)
 DICKIN 3   b   0         34     167       1     125     25.45 (  3.44- 188.43)
 EKBERG 3   m   0       2249    7692     395    2841      2.10 (  1.87-   2.36)
 EKBERG 6   f   0        546    3300     166    2581      2.57 (  2.15-   3.08)
 Subtotal EKBERG                                          2.23 (  2.02-   2.46)
 FUKUCH 3   b   0        192    1048      64    1039      2.97 (  2.21-   4.00)
 HOZAWA 3   b   0       2589    5379     872    5019      2.77 (  2.54-   3.02)
 HUHTI2 5   m   0         48     299       0      76     24.78~(  1.51- 406.36)
 HUHTI2 6   f   0          3      74      15     477      1.29 (  0.36-   4.56)
 Subtotal HUHTI2                                          2.13 (  0.67-   6.73)
 HUHTI3 3   m   0         84     726       3     236      9.10 (  2.85-  29.06)
 ITABAS 1   b   0         44      35      16      25      1.96 (  0.91-   4.24)
 JOHANN 3   b   0        134    1237      22     842      4.15 (  2.62-   6.56)
 KACHEL 3   b   0         41     688       7     503      4.28 (  1.91-   9.62)
 KATANC 3   b   0         54     403      21     382      2.44 (  1.44-   4.11)
 KIM    3   m   1        179       -      23       -      2.31 (  1.44-   3.72)
 KIM    6   f   1         10       -      61       -      1.74 (  0.85-   3.57)
 Subtotal KIM                                             2.12 (  1.43-   3.15)
 KLAYTO 1   b   0         62     117       7      76      5.75 (  2.50-  13.24)
 KOJIMA 3   m   0        169    5515      19    1871      3.02 (  1.87-   4.86)
 KOJIMA 6   f   0          5     316      20    3545      2.80 (  1.05-   7.52)
 Subtotal KOJIMA                                          2.98 (  1.94-   4.57)
*KRZYZA 3   m   1         57       -       5       -      2.63 (  1.09-   6.38)
*KRZYZA 6   f   1         19       -      32       -      1.41 (  0.82-   2.41)
 Subtotal KRZYZA                                          1.67 (  1.05-   2.65)
 LAI    1   m   0        107     469      20     493      5.62 (  3.43-   9.22)
 LAI    2   f   0          7      88      27     600      1.77 (  0.75-   4.18)
 Subtotal LAI                                             4.22 (  2.75-   6.48)
 LEBOWI 3   m   0         41     298       4     172      5.92 (  2.08-  16.80)
 LEBOWI 15  f   0         50     666      36     766      1.60 (  1.03-   2.48)
 Subtotal LEBOWI                                          1.95 (  1.30-   2.92)
*LINDBE 3   b   3          -       -       -       -      2.28 (  1.34-   3.88)
 LUNDB1 3   m   0         80     347      14     163      2.68 (  1.48-   4.88)
 LUNDB1 6   f   0         58     254      25     295      2.69 (  1.64-   4.43)
 Subtotal LUNDB1                                          2.69 (  1.84-   3.94)
 MADOR  3   m   0         19       7       0       3     18.20~(  0.84- 396.21)
 MANNI1 1   m   2        480       -     105       -      2.69 (  2.16-   3.34)
 MANNI1 10  f   2        305       -     156       -      3.18 (  2.61-   3.88)
 Subtotal MANNI1                                          2.95 (  2.55-   3.41)
 MANNI2 3   b   1        752       -     202       -      2.64 (  2.24-   3.12)
 MATHES 3   b   0         49      89      23     131      3.14 (  1.78-   5.51)
 MENEZ2 3   b   0        100       -      52       -      1.56 (  1.08-   2.24)
 MENEZ3 3   b   0        136       -      62       -      1.11 (  0.80-   1.54)
 MENEZ4 3   b   0         44       -      34       -      1.65 (  1.04-   2.63)
 MENEZ5 3   b   0        115       -      58       -      1.64 (  1.16-   2.33)
 MENEZ6 3   b   0        121       -      36       -      2.73 (  1.85-   4.03)
 MUELLE 3   m   0         35     187       2      57      5.33 (  1.24-  22.87)
 MUELLE 11  f   0          3     154       2     169      1.65 (  0.27-   9.98)
 Subtotal MUELLE                                          3.35 (  1.08-  10.41)
 NIEPSU 3   b   0         34     191       8     177      3.94 (  1.78-   8.74)
 PEREZP 1   f   0         19      47      45     145      1.30 (  0.69-   2.44)
 RENWIC 3   b   0         59     119       9      60      3.31 (  1.54-   7.12)
 RICCIO 3   b   0        321     478     178     537      2.03 (  1.62-   2.53)
 SHAHAB 3   b   0        771    3758     322    3364      2.14 (  1.87-   2.46)
 SHIN   3   m   0         68     285      14     116      1.98 (  1.07-   3.65)
 SHIN   4   f   0          3      25      35     614      2.11 (  0.61-   7.31)
 Subtotal SHIN                                            2.00 (  1.15-   3.47)
 STROM  3   m   0        157     233      17      71      2.81 (  1.60-   4.96)
 TSUSHI 3   b   0         41    1142       7    1056      5.42 (  2.42-  12.12)
 VESTBO 3   b   0       1667    8841     234    2366      1.91 (  1.65-   2.20)
 VIEGI2 3   m   0        177     533      21     118      1.87 (  1.14-   3.06)
 VIEGI2 9   f   0         52     370      66     390      0.83 (  0.56-   1.23)
 Subtotal VIEGI2                                          1.13 (  0.83-   1.54)
 WEISS  3   m   0         42     209       2      34      3.42 (  0.79-  14.77)
 WILSO1 1   b   0        112    1040      24     875      3.93 (  2.50-   6.16)
  ________________________________________________________________________________________________________________________
                                            International Evidence on Smoking and COPD, Phase 3, Analysis run on 27-SEP-10

                                                   Table 1 - A - 4 - 5

            IESCOPD - Meta-analysis of ever smoking, any product (or cigarettes if all product not available)
                                             COPD based on lung function only
                                                      Least-adjusted


                        Number Exposed  Non-exposed
 REF    NRR SEX ADJ     Case    Cont    Case    Cont      RR        95.00%CI
 YAMAGU 3   b   6        332       -     144       -      1.97 (  1.60-   2.43)
 ZIELI1 1   b   0       2364    6446     316    1884      2.19 (  1.92-   2.49)
 ZIELI2 3   m   0      11618   36365     341    2611      2.45 (  2.18-   2.74)
 ZIELI2 6   f   0       5159   26196     679    4832      1.40 (  1.29-   1.53)
 Subtotal ZIELI2                                          1.71 (  1.60-   1.83)
 ZIETKO 2   m   0         35      11       0      12     77.17~(  4.23-1408.01)
 ZIETKO 4   f   0         12       6       0      11     44.23~(  2.23- 875.91)
 Subtotal ZIETKO                                         58.88 (  7.34- 472.11)
Partial Totals         32626  126162    5259   48918
*prospective study                                        ~ With 0.5 adjustment for zero

                       N       64
                      NS       49


                      Wt  3497.42
                 Het Chi   319.93
                 Het  df       63
                 Het  P       ***
               Fixed  RR     2.17
                     RRl     2.10
                     RRu     2.24
                      P       +++
              Random  RR     2.35
                     RRl     2.13
                     RRu     2.60
                      P       +++
               Asymm  P         *


  ________________________________________________________________________________________________________________________
                                            International Evidence on Smoking and COPD, Phase 3, Analysis run on 27-SEP-10

                                                   Table 1 - A - 4 - 6

            IESCOPD - Meta-analysis of ever smoking, any product (or cigarettes if all product not available)
                                             COPD based on lung function only
                                                      Least-adjusted


                       N       64
                      NS       49


                      Wt  3497.42
                 Het Chi   319.93
                 Het  df       63
                 Het  P       ***
               Fixed  RR     2.17
                     RRl     2.10
                     RRu     2.24
                      P       +++
              Random  RR     2.35
                     RRl     2.13
                     RRu     2.60
                      P       +++
               Asymm  P         *

                                   Sex
                             both      male    female     Total


                       N       28        20        16        64
                      NS       28        20        16        64


                      Wt  1866.91    785.70    844.80   3497.42
                 Het Chi   106.89     42.39    101.24    319.93
                 Het  df       27        19        15        63
                 Het  P       ***        **       ***       ***
               Fixed  RR     2.32      2.41      1.69      2.17
                     RRl     2.21      2.25      1.58      2.10
                     RRu     2.42      2.59      1.81      2.24
                      P       +++       +++       +++       +++
              Random  RR     2.33      2.76      1.83      2.35
                     RRl     2.08      2.35      1.39      2.13
                     RRu     2.62      3.25      2.41      2.60
                      P       +++       +++       +++       +++
             Between Chi                                  69.41
             Between  df                                      2
             Between  P                                     ***
             Btwn(F)  P                                     ***


  ________________________________________________________________________________________________________________________
                                            International Evidence on Smoking and COPD, Phase 3, Analysis run on 27-SEP-10

                                                   Table 1 - A - 4 - 7

            IESCOPD - Meta-analysis of ever smoking, any product (or cigarettes if all product not available)
                                             COPD based on lung function only
                                 Excluded studies (and stage at which they were excluded)


1       CLARK COTTON  MEYER REMYJA RUTGER SNYDER SOBRAX     SU TAKEMU  WANG4   WEIR WHICKE ZALACA
2      ALDERS ANDER2 AUERBA   BANG  BECK1  BECK2 BJORNS  BROWN CERVER CHAPMA COATES COLLEG  DEAN2  DEANE DONTA2 DOPICO
       EHRLIC ENRIGH FINKLE FLETCH FOXMAN GOLDBE HAENSZ HARRIS  HAYES HIGGI2 HIGGI3 HIGGI6 HIRAYA HOLLA2 HOLLNA  HOUSE
       HRUBEC HUCHON JENSEN JINDA2  JOSHI JOUSI1   KATO KOTAN1  KUBIK LAMBER LANGE2 LANGHA LAVECC LUNDB2 MAGNUS MANFRE
       MELLST MENEZ1  MEREN MILLER  MILNE MOLLER   NAWA NEJJAR OGILVI  OMORI OSWAL1 OSWAL2 PANDEY  PRATT   REID RIMING
        RYDER SCHWAR  SHARP SHIMUR SOBRAD STJERN SUADIC SUTINE TAGER2 TROISI URRUTI VIEGI1 VIKGRE WAGEN2  WANG2    WIG
       WILHEL WILSO2  WOODS  WOOLF   ZOIA
3      BROGGE  CHEN1  CHENG DETORR GEIJER HEDMAN JAENDI KHOURY KOTAN2 KULLER   LIU1   LIU2  PRICE  TAGER VINEIS WATSON
4       CHEN3   KAHN   LIAW   PEAT SAWICK STERLI VOLLM1 VOLLM2   WALD    WEN WOJTYN
5      ALESSA  AMIGO ANDER1   BEST  CHEN2  COCCI  DEAN1  DOLL1  DOLL2 DONTA1 ENSTRO FERRI1 FERRI2 FERRI3  FIDAN FORAST
       GODTFR GULSVI HAMMO2 HARDIE HARIKK HAWTHO HIGGI4     HO HUHTI1 JACOBS  KAHN2 KARAKA  KIRAZ   LAM1   LAM2   LAM3
        LANGE    LEE LINDST MANNI3 MARAN1 MARAN2 MARCUS MONTNE NIHLEN NILSSO PELKON   PETO SARGEA SICHLE  SILVA SPEIZE
         TANG   THUN   TODD TRUPIN TVERDA VONHER   XIAO     XU   YUAN


  ________________________________________________________________________________________________________________________
                                            International Evidence on Smoking and COPD, Phase 3, Analysis run on 27-SEP-10

                                                   Table 1 - A - 4 - 8

            IESCOPD - Meta-analysis of ever smoking, any product (or cigarettes if all product not available)
                                             COPD based on lung function only
                                             Potentially overlapping studies


     REF| REFGP|PRINC|                     OVERLAP|

  DEMARC DEMARC     1         DEMARC/URRUTI/DEMEER
  EKBERG EKBERG     1   EKBERG/STROM/NIHLEN/MONTNE
   STROM  STROM     1   EKBERG/STROM/NIHLEN/MONTNE
  HOZAWA HOZAWA     1         ENRIGH/HOZAWA/HARIKK
  LUNDB1 LUNDBA     1  LINDBE/LUNDB1/LUNDB2/HEDLUN
  LINDBE LINDBE     1  LINDBE/LUNDB1/LUNDB2/HEDLUN
  HUHTI2 HUHTI1     2                HUHTI1/HUHTI2
  JOHANN JOHANN     1         HARDIE/JOHANN/BROGGE
  LEBOWI LEBOWI     1                 LEBOWI/SILVA
  MANNI2 MANNI2     1                MANNI2/MANNI3
  WILSO1 WILSO1     1                WILSO1/WILSO2
  MENEZ3 MENEZ3     1                 AMIGO/MENEZ3
  KRZYZA KRZYZA     1         SAWICK/KRZYZA/WOJTYN
  VESTBO VESTBO     1 GODT/VEST/LANG1+2/SUAD/HOLLN

                                    Most-adjusted - insufficient data for meta-analysis
     REF|NRR|SEX|AGEL|AGEH|     REGION|BEGYR|PUBYR|STTYP|ONSET|      DISEAS|ADJ|SMOKSTA|   PRODUCT|    UNEXP|        RR|

   STROM   6   m   68   68    Eu:Scand  1982  1996    CS  Prev      COPD:LF   8    Ever        Any   Nev any          *
  SIG|

    y


  ________________________________________________________________________________________________________________________
                                            International Evidence on Smoking and COPD, Phase 3, Analysis run on 27-SEP-10

                                                    Table 1 - A - 5 -

            IESCOPD - Meta-analysis of ever smoking, any product (or cigarettes if all product not available)
                                         COPD other than mortality/lung function


This analysis is restricted to results for:
1) Eligible study on database
2) Outcome COPD
3) Non-dose-response data
4) Ever smoking
5) COPD other than based on mortality or lung function only
6) Results complete enough for use in meta-analysis

Within each study, results are then selected (in the following order of preference, within each sex) for:
7) UNEXP   : never any, never cigarettes, other
8) PROD    : any product, cigarettes, cigarettes only
9) For overlapping studies: principal rather than subsidiary studies
and then for single sex results (m, f) in preference to results for both sexes combined (b).

Results adjusted for the most potential confounders are then chosen in Sections -1 to -3
and results adjusted for the least confounders in Sections -4 to -6. (Those least adjusted results which
actually differ from the most adjusted are marked 'x' in column X in Section -4)

Section -7 shows excluded studies, together with the stage (as above) at which no qualifying
results were found.

Section -8 lists the potentially overlapping studies which have been included (1=principal, 2=subsidiary),
and any results which would have been included in preference except that they had data not complete enough
for use in meta-analysis. It also lists their significance (yes/no), if known.


  ________________________________________________________________________________________________________________________
                                            International Evidence on Smoking and COPD, Phase 3, Analysis run on 27-SEP-10

                                                   Table 1 - A - 5 - 1

            IESCOPD - Meta-analysis of ever smoking, any product (or cigarettes if all product not available)
                                         COPD other than mortality/lung function
                                                      Most-adjusted


     REF|NRR|SEX|AGEL|AGEH|     REGION|BEGYR|PUBYR|STTYP|ONSET|      DISEAS|ADJ|SMOKSTA|   PRODUCT|    UNEXP|

  ALESSA   3   b   45   81     Eu:West  1992  1994    CC  Prev COPD:LF/symp   0    Ever       Cigs  Nev cigs
   AMIGO  11   m    0   99 Am:Sth/Cent  2001  2006    CC  Prev   COPD/CB/EM   6    Ever       Cigs  Nev cigs
   AMIGO  12   f    0   99 Am:Sth/Cent  2001  2006    CC  Prev   COPD/CB/EM   6    Ever       Cigs  Nev cigs
  ANDER1   3   m   25   74   Am:Canada  1963  1965    CS  Prev     COPD:oth   1    Ever        Any   Nev any
  ANDER1   6   f   25   74   Am:Canada  1963  1965    CS  Prev     COPD:oth   1    Ever        Any   Nev any
   CHEN2   9   m   35   64   Am:Canada  1994  2000    CS  Prev        CB/EM   6    Ever       Cigs  Nev cigs
   CHEN2  12   f   35   64   Am:Canada  1994  2000    CS  Prev        CB/EM   6    Ever       Cigs  Nev cigs
   COCCI   2   b   38   78     Eu:West     *  2002    CC  Prev COPD:LF/symp   0    Ever       Cigs  Nev cigs
  DONTA1   3   m   25   74 Eu:SE/Balkn  1960  1984    CS  Prev     COPD:oth   0    Ever       Cigs  Nev cigs
  FERRI2   3   m   25   80      Am:USA  1967  1971    CS  Prev     COPD:oth   1    Ever        Any   Nev any
  FERRI2  10   f   25   80      Am:USA  1967  1971    CS  Prev     COPD:oth   1    Ever        Any   Nev any
   FIDAN   2   m   15   99 Eu:SE/Balkn  2000  2004    CS  Prev     COPD:oth   3    Ever       Cigs  Nev cigs
  FORAST   6   f   55   99      Am:USA  1993  1998    CS  Prev        CB/EM   1    Ever       Cigs  Nev cigs
  GODTFR   3   b   20   99    Eu:Scand  1964  2002    Pr   Inc     COPD:ICD   3    Ever        Any   Nev any
  GULSVI   1   m   15   70    Eu:Scand  1972  1979    CS  Prev     COPD:oth   0    Ever        Any   Nev any
  GULSVI   2   f   15   70    Eu:Scand  1972  1979    CS  Prev     COPD:oth   0    Ever        Any   Nev any
  HARDIE   3   m   70   99    Eu:Scand  1998  2005    CS  Prev    CB/EM/Ast   1    Ever       Cigs  Nev cigs
  HARDIE   6   f   70   99    Eu:Scand  1998  2005    CS  Prev    CB/EM/Ast   1    Ever       Cigs  Nev cigs
  HARIKK   3   m   40   99      Am:USA  1962  2002    Pr   Inc     COPD:oth   0    Ever        Any   Nev any
  HARIKK   6   f   40   99      Am:USA  1962  2002    Pr   Inc     COPD:oth   0    Ever        Any   Nev any
      HO   3   m   70   99   Asia:FarE  1991  1999    CS  Prev   COPD:undef   0    Ever       Cigs  Nev cigs
      HO   6   f   70   99   Asia:FarE  1991  1999    CS  Prev   COPD:undef   0    Ever       Cigs  Nev cigs
  HUHTI1   6   m   40   64    Eu:Scand  1961  1965    CS  Prev    CB/EM/Ast   1    Ever        Any   Nev any
  HUHTI1  14   f   40   64    Eu:Scand  1961  1965    CS  Prev    CB/EM/Ast   1    Ever        Any   Nev any
  KARAKA   3   b   15   99 Eu:SE/Balkn     *  2003   nCC  Prev   COPD/CB/EM   0    Ever        Any   Nev any
   KIRAZ   2   f   25   99 Eu:SE/Balkn  1999  2003    CS  Prev COPD:LF/symp   1    Ever       Cigs  Nev cigs
    LAM2   3   m   60   99   Asia:FarE  1987  2002    CS  Prev   COPD:undef   0    Ever       Cigs  Nev cigs
  LINDST   9   b   20   69    Eu:Scand     *  2001    CS  Prev        CB/EM   5    Ever       Cigs  Nev cigs
  MARAN1   3   b   60   99 Asia:SE/Pac  1998  2002    CS  Prev     COPD:oth   0    Ever       Cigs  N/L cigs
  MARAN2   3   b   60   99 Asia:SE/Pac  1998  2002    Pr   Inc     COPD:oth   0    Ever       Cigs  N/L cigs
  MONTNE   3   b   20   59    Eu:Scand  1992  1998    CS  Prev        CB/EM   0    Ever        Any   Nev any
  NIHLEN   3   b   20   67    Eu:Scand  1992  2004    Pr   Inc   COPD/CB/EM   0    Ever        Any   Nev any
  PELKON   3   m   40   75    Eu:Scand  1959  2006    Pr   Inc     COPD:oth   1    Ever       Cigs  Nev cigs
  SARGEA   9   b   45   74       Eu:UK  1993  2000    CS  Prev     COPD:oth   5    Ever       Cigs  Nev cigs
  SICHLE   1   b   21   80 Eu:SE/Balkn  2000  2005    CS  Prev     COPD:oth   3    Ever       Cigs  Nev cigs
   SILVA   3   b   20   99      Am:USA  1972  2004    Pr   Inc COPD:LF/symp   6    Ever       Cigs  Nev cigs
  TRUPIN   6   b   55   75      Am:USA  2001  2005    CS  Prev   COPD/CB/EM   4    Ever       Cigs  Nev cigs
  VONHER   3   m   30   99    Eu:Scand  1978  2000    CS  Prev        CB/EM   0    Ever        Any   Nev any
  VONHER   6   f   30   99    Eu:Scand  1978  2000    CS  Prev        CB/EM   0    Ever        Any   Nev any
    XIAO   3   b    0   99   Asia:FarE     *  2004    CC  Prev   COPD:undef   0    Ever       Cigs  Nev cigs
      XU   2   m   35   99   Asia:FarE  2000  2005    CS  Prev        CB/EM  13    Ever       Cigs  Nev cigs
      XU   4   f   35   99   Asia:FarE  2000  2005    CS  Prev        CB/EM  13    Ever       Cigs  Nev cigs


  ________________________________________________________________________________________________________________________
                                            International Evidence on Smoking and COPD, Phase 3, Analysis run on 27-SEP-10

                                                   Table 1 - A - 5 - 2

            IESCOPD - Meta-analysis of ever smoking, any product (or cigarettes if all product not available)
                                         COPD other than mortality/lung function
                                                      Most-adjusted


                        Number Exposed  Non-exposed
 REF    NRR SEX ADJ     Case    Cont    Case    Cont      RR        95.00%CI
 ALESSA 3   b   0         26      16      11      14      2.07 (  0.76-   5.65)
 AMIGO  11  m   6         40       -       3       -      4.65 (  0.98-  22.09)
 AMIGO  12  f   6         38       -       6       -      7.88 (  2.55-  24.36)
 Subtotal AMIGO                                           6.57 (  2.64-  16.39)
 ANDER1 3   m   1         31       -       2       -      5.13 (  1.14-  23.05)
 ANDER1 6   f   1         11       -      16       -      1.18 (  0.53-   2.64)
 Subtotal ANDER1                                          1.63 (  0.81-   3.32)
 CHEN2  9   m   6         49       -       8       -      3.43 (  1.60-   7.38)
 CHEN2  12  f   6         97       -      33       -      2.57 (  1.62-   4.07)
 Subtotal CHEN2                                           2.78 (  1.87-   4.12)
 COCCI  2   b   0         20      13       0       6     19.74~(  1.03- 379.94)
 DONTA1 3   m   0         58     333      10     127      2.21 (  1.10-   4.46)
 FERRI2 3   m   1        214       -      11       -      3.88 (  2.01-   7.49)
 FERRI2 10  f   1         87       -      54       -      2.28 (  1.57-   3.31)
 Subtotal FERRI2                                          2.59 (  1.88-   3.59)
 FIDAN  2   m   3         53       -       2       -      6.96 (  1.45-  33.26)
 FORAST 6   f   1         61       -      28       -      2.36 (  1.48-   3.77)
*GODTFR 3   b   3       1189       -      71       -      5.46 (  4.20-   7.10)
 GULSVI 1   m   0         54       -       1       -     18.60 (  2.52- 137.03)
 GULSVI 2   f   0         63       -      22       -      3.52 (  1.88-   6.58)
 Subtotal GULSVI                                          4.09 (  2.25-   7.43)
 HARDIE 3   m   1        104       -       7       -      5.43 (  2.55-  11.58)
 HARDIE 6   f   1         41       -      31       -      2.75 (  1.72-   4.39)
 Subtotal HARDIE                                          3.32 (  2.23-   4.95)
*HARIKK 3   m   0         33     319       7     139      2.05 (  0.93-   4.53)
*HARIKK 6   f   0          7      91       0     101     16.64~(  0.96- 287.29)
 Subtotal HARIKK                                          2.39 (  1.11-   5.11)
 HO     3   m   0         88     604      20     282      2.05 (  1.24-   3.41)
 HO     6   f   0         27     243      30     733      2.71 (  1.58-   4.66)
 Subtotal HO                                              2.34 (  1.62-   3.38)
 HUHTI1 6   m   1        200       -       7       -      8.18 (  3.68-  18.18)
 HUHTI1 14  f   1         21       -      56       -      2.88 (  1.62-   5.13)
 Subtotal HUHTI1                                          4.12 (  2.58-   6.57)
 KARAKA 3   b   0         59      87      25      81      2.20 (  1.26-   3.84)
 KIRAZ  2   f   1          9       -      25       -      6.86 (  2.47-  19.06)
 LAM2   3   m   0        246     634      49     339      2.68 (  1.92-   3.75)
 LINDST 9   b   5        315       -     166       -      1.84 (  1.50-   2.25)
 MARAN1 3   b   0        166     968      54    1905      6.05 (  4.41-   8.30)
*MARAN2 3   b   0         50     605      20    1324      5.47 (  3.29-   9.11)
 MONTNE 3   b   0        301    4686      91    3390      2.39 (  1.88-   3.04)
*NIHLEN 3   b   0         91    2465      21    1815      3.19 (  1.99-   5.11)
*PELKON 3   m   1          -       -       -       -      2.17 (  1.48-   3.17)
 SARGEA 9   b   5        968       -     622       -      1.40 (  1.25-   1.58)
 SICHLE 1   b   3          -       -       -       -      2.17 (  1.63-   2.91)
*SILVA  3   b   6          -       -       -       -      1.59 (  0.96-   2.63)
 TRUPIN 6   b   4        304       -      73       -      3.45 (  2.59-   4.59)
 VONHER 3   m   0        655    1687      63     911      5.61 (  4.28-   7.36)
 VONHER 6   f   0        132     734     137    2888      3.79 (  2.95-   4.88)
 Subtotal VONHER                                          4.55 (  3.78-   5.47)
 XIAO   3   b   0         79      44      21      56      4.79 (  2.57-   8.92)
 XU     2   m   13       596       -     450       -      1.26 (  1.07-   1.48)
 XU     4   f   13        51       -     647       -      2.61 (  1.85-   3.68)
 Subtotal XU                                              1.44 (  1.24-   1.67)
Partial Totals          6634   13529    2900   14111
*prospective study                                        ~ With 0.5 adjustment for zero


  ________________________________________________________________________________________________________________________
                                            International Evidence on Smoking and COPD, Phase 3, Analysis run on 27-SEP-10

                                                   Table 1 - A - 5 - 2

            IESCOPD - Meta-analysis of ever smoking, any product (or cigarettes if all product not available)
                                         COPD other than mortality/lung function
                                                      Most-adjusted


                       N       42
                      NS       31


                      Wt  1245.03
                 Het Chi   336.06
                 Het  df       41
                 Het  P       ***
               Fixed  RR     2.31
                     RRl     2.18
                     RRu     2.44
                      P       +++
              Random  RR     3.04
                     RRl     2.53
                     RRu     3.65
                      P       +++
               Asymm  P       ***


  ________________________________________________________________________________________________________________________
                                            International Evidence on Smoking and COPD, Phase 3, Analysis run on 27-SEP-10

                                                   Table 1 - A - 5 - 3

            IESCOPD - Meta-analysis of ever smoking, any product (or cigarettes if all product not available)
                                         COPD other than mortality/lung function
                                                      Most-adjusted


                       N       42
                      NS       31


                      Wt  1245.03
                 Het Chi   336.06
                 Het  df       41
                 Het  P       ***
               Fixed  RR     2.31
                     RRl     2.18
                     RRu     2.44
                      P       +++
              Random  RR     3.04
                     RRl     2.53
                     RRu     3.65
                      P       +++
               Asymm  P       ***

                                   Sex
                             both      male    female     Total


                       N       14        15        13        42
                      NS       14        15        13        42


                      Wt   701.42    322.35    221.26   1245.03
                 Het Chi   179.80    121.39     19.76    336.06
                 Het  df       13        14        12        41
                 Het  P       ***       ***       (*)       ***
               Fixed  RR     2.19      2.21      2.93      2.31
                     RRl     2.03      1.98      2.56      2.18
                     RRu     2.35      2.46      3.34      2.44
                      P       +++       +++       +++       +++
              Random  RR     2.93      3.38      2.87      3.04
                     RRl     2.13      2.27      2.38      2.53
                     RRu     4.04      5.04      3.46      3.65
                      P       +++       +++       +++       +++
             Between Chi                                  15.11
             Between  df                                      2
             Between  P                                     ***
             Btwn(F)  P                                    N.S.

                                        Continent
                            NAmer    Europe      Asia  oth/mult     Total


                       N       11        21         8         2        42
                      NS        7        17         6         1        31


                      Wt   155.09    781.12    304.23      4.60   1245.03
                 Het Chi    16.45    204.49    105.63      0.29    336.06
                 Het  df       10        20         7         1        41
                 Het  P       (*)       ***       ***      N.S.       ***
               Fixed  RR     2.63      2.30      2.15      6.57      2.31
                     RRl     2.24      2.14      1.92      2.64      2.18
                     RRu     3.07      2.47      2.40     16.39      2.44
                      P       +++       +++       +++       +++       +++
              Random  RR     2.54      3.22      3.03      6.57      3.04
                     RRl     2.03      2.46      1.86      2.64      2.53
                     RRu     3.18      4.22      4.93     16.39      3.65
                      P       +++       +++       +++       +++       +++
             Between Chi                                             9.20
             Between  df                                                3
             Between  P                                                 *
             Btwn(F)  P                                              N.S.


  ________________________________________________________________________________________________________________________
                                            International Evidence on Smoking and COPD, Phase 3, Analysis run on 27-SEP-10

                                                   Table 1 - A - 5 - 3

            IESCOPD - Meta-analysis of ever smoking, any product (or cigarettes if all product not available)
                                         COPD other than mortality/lung function
                                                      Most-adjusted
                        National cigarette tobacco type (excluding mixed/unkown)
                          blended  virginia     Total


                       N       33         5        38
                      NS       25         3        28


                      Wt   709.83    312.29   1022.12
                 Het Chi   138.69     13.95    270.89
                 Het  df       32         4        37
                 Het  P       ***        **       ***
               Fixed  RR     3.10      1.48      2.48
                     RRl     2.88      1.33      2.33
                     RRu     3.34      1.66      2.63
                      P       +++       +++       +++
              Random  RR     3.27      2.04      3.13
                     RRl     2.74      1.29      2.58
                     RRu     3.89      3.25      3.81
                      P       +++        ++       +++
             Between Chi                       118.24
             Between  df                            1
             Between  P                           ***
             Btwn(F)  P                           ***

                                        Start year of study
                            <1970   1970-79   1980-89   1990-99     2000+   unknown     Total


                       N       11         5         1        14         7         4        42
                      NS        7         3         1        11         5         4        31


                      Wt   158.40    138.56     34.48    520.03    277.35    116.20   1245.03
                 Het Chi    38.46     21.50      0.00    122.14     55.21     10.54    336.06
                 Het  df       10         4         0        13         6         3        41
                 Het  P       ***       ***      N.S.       ***       ***         *       ***
               Fixed  RR     3.33      4.02      2.68      2.04      1.85      2.05      2.31
                     RRl     2.85      3.41      1.92      1.87      1.64      1.71      2.18
                     RRu     3.89      4.75      3.75      2.22      2.08      2.46      2.44
[truncated: 1,183,760 more chars]
