# Supplementary material for: Systematic review with meta-analysis of the epidemiological evidence relating smoking to COPD, chronic bronchitis and emphysema
Source: BMC Pulm Med. 2011 Jun 14;11:36. doi: 10.1186/1471-2466-11-36 (PMC3128042; doi:10.1186/1471-2466-11-36)
Supplement: Additional file 5 — MetaMajorCB. .RTF file giving the full results of the meta-analyses for the major smoking variables for CB. [file 1471-2466-11-36-S5.RTF]

Systematic review with meta-analysis of the epidemiological evidence relating smoking to COPD, chronic bronchitis and emphysema

Barbara A Forey, Alison J Thornton and Peter N Lee

Additional file 5 : MetaMajorCB

See Additional file 10 (Intro sheet) for list of tables and page numbers


                                                    Table 2 - A - 1 -

            IESCOPD - Meta-analysis of ever smoking, any product (or cigarettes if all product not available)
                                                          Any CB


This analysis is restricted to results for:
1) Eligible study on database
2) Outcome CB
3) Non-dose-response data
4) Ever smoking
5) Results complete enough for use in meta-analysis

Within each study, results are then selected (in the following order of preference, within each sex) for:
6) UNEXP   : never any, never cigarettes, other
7) PROD    : any product, cigarettes, cigarettes only
8) For overlapping studies: principal rather than subsidiary studies
and then for single sex results (m, f) in preference to results for both sexes combined (b).

Results adjusted for the most potential confounders are then chosen in Sections -1 to -3
and results adjusted for the least confounders in Sections -4 to -6. (Those least adjusted results which
actually differ from the most adjusted are marked 'x' in column X in Section -4)

Section -7 shows excluded studies, together with the stage (as above) at which no qualifying
results were found.

Section -8 lists the potentially overlapping studies which have been included (1=principal, 2=subsidiary),
and any results which would have been included in preference except that they had data not complete enough
for use in meta-analysis. It also lists their significance (yes/no), if known.


  ________________________________________________________________________________________________________________________
                                            International Evidence on Smoking and COPD, Phase 3, Analysis run on 27-SEP-10

                                                   Table 2 - A - 1 - 1

            IESCOPD - Meta-analysis of ever smoking, any product (or cigarettes if all product not available)
                                                          Any CB
                                                      Most-adjusted


     REF|NRR|SEX|AGEL|AGEH|     REGION|BEGYR|PUBYR|STTYP|ONSET|      DISEAS|ADJ|SMOKSTA|   PRODUCT|    UNEXP|

  ALDERS   5   m   35   74       Eu:UK  1977  1985    CC  Prev CB:diagnosed   1    Ever        Any   Nev any
  ALDERS   6   f   35   74       Eu:UK  1977  1985    CC  Prev CB:diagnosed   1    Ever MCigs only   Nev any
  ANDER1  32   m   25   74   Am:Canada  1963  1965    CS  Prev  CB:symptoms   1    Ever        Any   Nev any
  ANDER1  35   f   25   74   Am:Canada  1963  1965    CS  Prev  CB:symptoms   1    Ever        Any   Nev any
   BECK1   3   m   11   99      Am:USA  1972  1982    CS  Prev  CB:symptoms   0    Ever       Cigs   Nev any
   BECK1   6   f   11   99      Am:USA  1972  1982    CS  Prev  CB:symptoms   0    Ever       Cigs   Nev any
   BECK2   3   m   11   99      Am:USA  1972  1982    Pr   Inc  CB:symptoms   0    Ever       Cigs   Nev any
   BECK2   6   f   11   99      Am:USA  1972  1982    Pr   Inc  CB:symptoms   0    Ever       Cigs   Nev any
    BEST   3   m   30   97   Am:Canada  1955  1967    Pr   Inc CB:mortality   1    Ever  Cigs only   Nev any
  BJORNS   6   b   20   44    Eu:Scand  1990  1994    CS  Prev  CB:symptoms   4    Ever        Any   Nev any
   BROWN   1   m   60   69       Eu:UK  1956  1957    CS  Prev CB:diagnosed   0    Ever        Any   Nev any
  CERVER   6   b   20   44     Eu:West  1998  2003    CS  Prev  CB:symptoms   6    Ever       Cigs  Nev cigs
  COATES   3   b   40   64      Am:USA  1962  1965    CS  Prev  CB:symptoms   0    Ever       Cigs  Nev cigs
  COLLEG   6   m   40   64       Eu:UK     *  1961    CS  Prev CB:diagnosed   2    Ever        Any   Nev any
  COLLEG   9   f   40   64       Eu:UK     *  1961    CS  Prev CB:diagnosed   1    Ever        Any   Nev any
   DEANE   6   m   40   59      Am:USA  1963  1965    CS  Prev  CB:symptoms   1    Ever        Any   Nev any
  DEMARC  20   b   20   44       Multi  1991  2004    CS  Prev  CB:symptoms   1    Ever       Cigs   Nev any
   DOLL1  13   m   20   99       Eu:UK  1951  1994    Pr   Inc CB:mortality   1    Ever        Any   Nev any
  DONTA2   3   m   25   84 Eu:SE/Balkn  1960  1984    Pr   Inc CB:diagnosed   0    Ever       Cigs  Nev cigs
  DOPICO   3   m   15    *      Am:USA     *  1984    CS  Prev  CB:symptoms   0    Ever       Cigs  Nev cigs
  EHRLIC  15   m   15   99      Africa  1998  2004    CS  Prev  CB:symptoms   6    Ever        Any   Nev any
  EHRLIC  21   f   15   99      Africa  1998  2004    CS  Prev  CB:symptoms   6    Ever        Any   Nev any
  ENRIGH   3   m   65   99      Am:USA  1989  1994    CS  Prev CB:diagnosed   0    Ever       Cigs  Nev cigs
  ENRIGH   6   f   65   99      Am:USA  1989  1994    CS  Prev CB:diagnosed   0    Ever       Cigs  Nev cigs
  FERRI1  61   m   25   74      Am:USA  1961  1971    CS  Prev  CB:symptoms   1    Ever        Any   Nev any
  FERRI1  82   f   25   74      Am:USA  1961  1971    CS  Prev  CB:symptoms   1    Ever        Any   Nev any
  FINKLE   1   m   18   25      Am:USA  1969  1974    CS  Prev  CB:symptoms   0    Ever        Any   Nev any
  FOXMAN   3   m   20   69      Am:USA     *  1986    CS  Prev  CB:symptoms   1    Ever        Any   Nev any
  FOXMAN   6   f   20   69      Am:USA     *  1986    CS  Prev  CB:symptoms   1    Ever        Any   Nev any
  GOLDBE   6   m   15   99      Am:USA  1970  1974    CS  Prev  CB:symptoms   1    Ever       Cigs  Nev cigs
  GOLDBE  12   f   15   99      Am:USA  1970  1974    CS  Prev  CB:symptoms   1    Ever       Cigs  Nev cigs
  HAENSZ  10   m   35   74    Eu:Scand  1964  1972    CS  Prev  CB:symptoms   1    Ever        Any   Nev any
  HAENSZ  32   f   35   74    Eu:Scand  1964  1972    CS  Prev  CB:symptoms   1    Ever        Any   Nev any
  HARDIE   9   m   70   99    Eu:Scand  1998  2005    CS  Prev CB:diagnosed   1    Ever       Cigs  Nev cigs
  HARDIE  12   f   70   99    Eu:Scand  1998  2005    CS  Prev CB:diagnosed   1    Ever       Cigs  Nev cigs
  HARRIS   3   m   15   60      Africa     *  1993    CS  Prev  CB:symptoms   0    Ever        Any   Nev any
  HAWTHO  10   m   45   76       Eu:UK  1965  1978    Pr  Prev  CB:symptoms   0    Ever       Cigs  N/L cigs
  HAWTHO  17   f   45   76       Eu:UK  1965  1978    Pr  Prev  CB:symptoms   0    Ever       Cigs  N/L cigs
   HAYES   9   b   15   99      Am:USA  1970  1974    CS  Prev  CB:symptoms   3    Ever       Cigs  Nev cigs
  HIGGI2   3   m   25   74       Eu:UK  1956  1957    CS  Prev  CB:symptoms   0    Ever       Cigs  Nev cigs
  HIGGI2  15   f   25   74       Eu:UK  1956  1957    CS  Prev  CB:symptoms   1    Ever       Cigs  Nev cigs
  HIGGI3   3   m   55   64       Eu:UK  1956  1958    CS  Prev  CB:symptoms   0    Ever        Any   Nev any
  HIGGI3   9   f   55   64       Eu:UK  1956  1958    CS  Prev  CB:symptoms   0    Ever        Any   Nev any
  HIGGI6   3   m   25   64      Am:USA  1962  1977    CS  Prev  CB:symptoms   1    Ever        Any   Nev any
  HIGGI6  10   f   25   64      Am:USA  1962  1977    CS  Prev  CB:symptoms   1    Ever        Any   Nev any
      HO  11   b   70   99   Asia:FarE  1991  1999    CS  Prev  CB:self-rep   3    Ever       Cigs  Nev cigs
  HOLLA2   3   m   40   59      Am:USA  1962  1965    CS  Prev  CB:symptoms   0    Ever        Any   Nev any
  HOLLNA   3   m   40   40    Eu:Scand  1976  1983    CS  Prev  CB:symptoms   0    Ever        Any   Nev any
  HOLLNA  11   f   40   40    Eu:Scand  1976  1983    CS  Prev  CB:symptoms   0    Ever        Any   Nev any
   HOUSE   6   m   15   99      Am:USA  1970  1974    CS  Prev  CB:symptoms   1    Ever       Cigs  Nev cigs
   HOUSE  12   f   15   99      Am:USA  1970  1974    CS  Prev  CB:symptoms   1    Ever       Cigs  Nev cigs
  HUCHON   6   b   25   99     Eu:West     *  2002    CS  Prev  CB:symptoms   1    Ever       Cigs  Nev cigs
  HUHTI1  42   m   40   64    Eu:Scand  1961  1965    CS  Prev  CB:symptoms   1    Ever        Any   Nev any
  HUHTI1  91   f   40   64    Eu:Scand  1961  1965    CS  Prev  CB:symptoms   1    Ever        Any   Nev any
  HUHTI3  22   m   25   69    Eu:Scand  1968  1978    CS  Prev  CB:symptoms   1    Ever        Any   Nev any
  JINDA2   3   b   35   99 Asia:MidE/S     *  2006    CS  Prev  CB:symptoms   6    Ever        Any   Nev any
   JOSHI   6   m   17   64 Asia:MidE/S     *  1975    CS  Prev  CB:symptoms   0    Ever        Any   Nev any
  JOUSI1   3   m   25   64    Eu:Scand  1972  1996    CS  Prev  CB:symptoms   0    Ever        Any   Nev any
  JOUSI1   6   f   25   64    Eu:Scand  1972  1996    CS  Prev  CB:symptoms   0    Ever        Any   Nev any
   KAHN2  32   m   31   84      Am:USA  1954  1966    Pr   Inc CB:mortality   1    Ever        Any   Nev any
    KATO   3   m   40   99   Asia:FarE  1985  1989    CS  Prev  CB:self-rep   1    Ever       Cigs  Nev cigs
    KATO   6   f   40   99   Asia:FarE  1985  1989    CS  Prev  CB:self-rep   1    Ever       Cigs  Nev cigs
   KIRAZ   4   f   25   99 Eu:SE/Balkn  1999  2003    CS  Prev     CB:other   1    Ever       Cigs  Nev cigs
  KOTAN1   9   b   20   69    Eu:Scand  1995  2003    CS  Prev CB:diagnosed   7    Ever        Any   Nev any
   KUBIK   3   m   15   99     Eu:East  1972  1984    CS  Prev  CB:symptoms   0    Ever        Any   N/L any
   KUBIK   4   f   15   99     Eu:East  1972  1984    CS  Prev  CB:symptoms   0    Ever        Any   N/L any
  LAMBER   6   m   35   69       Eu:UK  1965  1970    CS  Prev  CB:symptoms   1    Ever       Cigs  Nev cigs
  LAMBER  38   f   35   69       Eu:UK  1965  1970    CS  Prev  CB:symptoms   1    Ever        Any   Nev any
  LANGHA   6   m   20   99    Eu:Scand  1995  2000    CS  Prev  CB:symptoms   1    Ever        Any   Nev any
  LANGHA  12   f   20   99    Eu:Scand  1995  2000    CS  Prev  CB:symptoms   1    Ever        Any   Nev any
  LAVECC   9   b   15   99     Eu:West  1983  1988    CS  Prev  CB:self-rep   6    Ever        Any   Nev any
  LEBOWI  30   b   15   96      Am:USA  1972  1977    CS  Prev CB:diagnosed   3    Ever       Cigs  Nev cigs
  ________________________________________________________________________________________________________________________
                                            International Evidence on Smoking and COPD, Phase 3, Analysis run on 27-SEP-10

                                                   Table 2 - A - 1 - 1

            IESCOPD - Meta-analysis of ever smoking, any product (or cigarettes if all product not available)
                                                          Any CB
                                                      Most-adjusted


     REF|NRR|SEX|AGEL|AGEH|     REGION|BEGYR|PUBYR|STTYP|ONSET|      DISEAS|ADJ|SMOKSTA|   PRODUCT|    UNEXP|

  LINDST  23   b   20   69    Eu:Scand     *  2001    CS  Prev  CB:symptoms   5    Ever       Cigs  Nev cigs
  LUNDB2   6   m   35   66    Eu:Scand  1986  1993    CC  Prev     CB:other   1    Ever        Any   Nev any
  LUNDB2  12   f   35   66    Eu:Scand  1986  1993    CC  Prev     CB:other   1    Ever        Any   Nev any
  MAGNUS   6   m   50   80    Eu:Scand  1993  1999    CS  Prev  CB:symptoms   1    Ever        Any   Nev any
  MANFRE   3   m   20   65   Am:Canada  1978  1989    CS  Prev  CB:symptoms   0    Ever        Any   Nev any
  MANFRE   6   f   20   65   Am:Canada  1978  1989    CS  Prev  CB:symptoms   0    Ever        Any   Nev any
  MELLST   3   m   70   70    Eu:Scand  1971  1982    CS  Prev     CB:other   0    Ever        Any   Nev any
  MENEZ1   6   b   40   99 Am:Sth/Cent  1990  1995    CS  Prev  CB:symptoms   8    Ever       Cigs  Nev cigs
   MEREN   6   b   15   64     Eu:East  1995  2001    CS  Prev  CB:symptoms   4    Ever       Cigs  Nev cigs
  MILLER   3   m   15   99      Am:USA  1978  1988    CS  Prev CB:diagnosed   1    Ever       Cigs  Nev cigs
  MILLER   6   f   15   99      Am:USA  1978  1988    CS  Prev CB:diagnosed   1    Ever       Cigs  Nev cigs
   MILNE   3   m   62   90       Eu:UK  1968  1972    CS  Prev  CB:symptoms   0    Ever       Cigs  Nev cigs
   MILNE  15   f   62   90       Eu:UK  1968  1972    CS  Prev  CB:symptoms   0    Ever        Any   Nev any
  MOLLER   3   b   40   72     Eu:West     *  2001    CC  Prev  CB:symptoms   0    Ever       Cigs  Nev cigs
  MUELLE  36   m   20   69      Am:USA  1967  1971    CS  Prev  CB:symptoms   1    Ever        Any   Nev any
  MUELLE  51   f   20   69      Am:USA  1967  1971    CS  Prev  CB:symptoms   1    Ever        Any   Nev any
  NEJJAR   5   b   65   99     Eu:West  1991  1996    CS  Prev  CB:symptoms   6    Ever       Cigs  Nev cigs
  OGILVI   3   m   30   99       Eu:UK  1955  1957    CC  Prev  CB:symptoms   0    Ever        Any   Nev any
  OGILVI   8   f   30   99       Eu:UK  1955  1957    CC  Prev  CB:symptoms   0    Ever        Any   Nev any
  OSWAL2   2   m   15   65       Eu:UK  1954  1955    CS  Prev  CB:symptoms   1    Ever       Cigs  N/L cigs
  OSWAL2   4   f   15   65       Eu:UK  1954  1955    CS  Prev  CB:symptoms   1    Ever       Cigs  N/L cigs
  PANDEY   6   m   20   99 Asia:MidE/S  1979  1984    CS  Prev  CB:symptoms   2    Ever        Any   Nev any
  PANDEY  12   f   20   99 Asia:MidE/S  1979  1984    CS  Prev  CB:symptoms   2    Ever        Any   Nev any
  PELKON   6   m   40   75    Eu:Scand  1959  2006    Pr   Inc     CB:other   1    Ever       Cigs  Nev cigs
  PEREZP   5   f   40   99 Am:Sth/Cent  1992  1996    CC  Prev  CB:symptoms   0    Ever       Cigs  Nev cigs
    REID   6   m   35   74      Am:USA  1962  1966    CS  Prev  CB:symptoms   1    Ever       Cigs  Nev cigs
    REID  22   f   35   74      Am:USA  1962  1966    CS  Prev  CB:symptoms   1    Ever       Cigs  Nev cigs
  SAWICK   5   m   19   70     Eu:East  1968  1972    CS  Prev  CB:symptoms   0    Ever        Any   Nev any
  SAWICK  18   f   19   70     Eu:East  1968  1972    CS  Prev  CB:symptoms   0    Ever        Any   Nev any
  SHIMUR   3   b   31   80   Asia:FarE     *  1996    CC  Prev     CB:other   0    Ever        Any   Nev any
   SILVA   6   b   20   99      Am:USA  1972  2004    Pr   Inc     CB:other   6    Ever       Cigs  Nev cigs
  SOBRAD   4   b   40   69     Eu:West  1996  2000    CS  Prev  CB:symptoms   3    Ever        Any   Nev any
  STJERN   3   b   16   72    Eu:Scand  1981  1985    CS  Prev  CB:symptoms   0    Ever        Any   Nev any
  TROISI   3   f   34   69      Am:USA  1980  1995    Pr   Inc CB:diagnosed   1    Ever       Cigs  Nev cigs
  VIEGI1   3   m   20   64     Eu:West  1980  1988    CS  Prev CB:diagnosed   0    Ever       Cigs   Nev any
  VIEGI1   6   f   20   64     Eu:West  1980  1988    CS  Prev CB:diagnosed   0    Ever       Cigs   Nev any
  WAGEN2  11   b   21   68     Eu:West  2001  2004    CC  Prev  CB:symptoms   1    Ever       Cigs  Nev cigs
  WILHEL   3   m   54   54    Eu:Scand  1967  1969    CS  Prev  CB:symptoms   0    Ever        Any   Nev any
   WOODS   3   b   20   44      Aus/NZ     *  2000    CS  Prev  CB:symptoms   5    Ever       Cigs  Nev cigs
   WOOLF   3   f   25   54   Am:Canada     *  1974    CS  Prev  CB:symptoms   0    Ever       Cigs  Nev cigs
  YAMAGU   9   b   40   99   Asia:FarE  1986  1988    CS  Prev  CB:symptoms   6    Ever       Cigs  Nev cigs
    ZOIA   3   b   18   69     Eu:West     *  1995    CS  Prev  CB:symptoms   4    Ever        Any   Nev any


  ________________________________________________________________________________________________________________________
                                            International Evidence on Smoking and COPD, Phase 3, Analysis run on 27-SEP-10

                                                   Table 2 - A - 1 - 2

            IESCOPD - Meta-analysis of ever smoking, any product (or cigarettes if all product not available)
                                                          Any CB
                                                      Most-adjusted


                        Number Exposed  Non-exposed
 REF    NRR SEX ADJ     Case    Cont    Case    Cont      RR        95.00%CI
 ALDERS 5   m   1        510       -      25       -      2.81 (  1.74-   4.54)
 ALDERS 6   f   1        333       -     105       -      2.79 (  2.09-   3.72)
 Subtotal ALDERS                                          2.80 (  2.18-   3.58)
 ANDER1 32  m   1         50       -       4       -      4.51 (  1.53-  13.29)
 ANDER1 35  f   1         19       -      15       -      2.20 (  1.08-   4.51)
 Subtotal ANDER1                                          2.74 (  1.51-   4.97)
 BECK1  3   m   0         17     243       3     189      4.41 (  1.27-  15.26)
 BECK1  6   f   0         10     198       6     318      2.68 (  0.96-   7.48)
 Subtotal BECK1                                           3.28 (  1.49-   7.24)
*BECK2  3   m   0          5     243       2     189      1.94 (  0.38-   9.91)
*BECK2  6   f   0          4     199       3     318      2.13 (  0.48-   9.42)
 Subtotal BECK2                                           2.04 (  0.68-   6.13)
*BEST   3   m   1         77       -       3       -     11.44 (  3.61-  36.25)
 BJORNS 6   b   4        836       -     354       -      2.18 (  1.90-   2.50)
 BROWN  1   m   0        238     725      15      76      1.66 (  0.94-   2.95)
 CERVER 6   b   6       1465       -     754       -      2.52 (  2.29-   2.76)
 COATES 3   b   0        135     913      21     515      3.63 (  2.26-   5.82)
 COLLEG 6   m   2          -       -       -       -      2.88 (  0.98-   8.47)
 COLLEG 9   f   1          -       -       -       -      2.64 (  1.54-   4.52)
 Subtotal COLLEG                                          2.69 (  1.66-   4.35)
 DEANE  6   m   1        104       -       3       -      6.15 (  1.83-  20.63)
 DEMARC 20  b   1        341       -      91       -      2.96 (  2.33-   3.75)
*DOLL1  13  m   1         95       -       3       -      7.40 (  2.22-  24.69)
*DONTA2 3   m   0         63     333       8     127      3.00 (  1.48-   6.09)
 DOPICO 3   m   0         37     139       6      57      2.53 (  1.01-   6.32)
 EHRLIC 15  m   6        100       -      33       -      1.27 (  0.77-   2.10)
 EHRLIC 21  f   6         88       -     138       -      1.64 (  1.15-   2.34)
 Subtotal EHRLIC                                          1.51 (  1.13-   2.01)
 ENRIGH 3   m   0         71    1432      17     699      2.04 (  1.19-   3.49)
 ENRIGH 6   f   0        102    1155      85    1558      1.62 (  1.20-   2.18)
 Subtotal ENRIGH                                          1.71 (  1.32-   2.22)
 FERRI1 61  m   1        156       -      10       -      3.22 (  1.62-   6.42)
 FERRI1 82  f   1         44       -      35       -      2.17 (  1.35-   3.51)
 Subtotal FERRI1                                          2.47 (  1.67-   3.65)
 FINKLE 1   m   0       3551   17761     596   10240      3.44 (  3.14-   3.76)
 FOXMAN 3   m   1        219       -      41       -      2.48 (  1.73-   3.54)
 FOXMAN 6   f   1        151       -      59       -      2.58 (  1.88-   3.53)
 Subtotal FOXMAN                                          2.54 (  2.00-   3.21)
 GOLDBE 6   m   1        348       -     148       -      1.48 (  1.20-   1.83)
 GOLDBE 12  f   1        283       -      61       -      2.87 (  2.15-   3.83)
 Subtotal GOLDBE                                          1.86 (  1.57-   2.21)
 HAENSZ 10  m   1        200       -      27       -      2.27 (  1.51-   3.42)
 HAENSZ 32  f   1         79       -      97       -      3.32 (  2.43-   4.53)
 Subtotal HAENSZ                                          2.89 (  2.25-   3.70)
 HARDIE 9   m   1         37       -       1       -     10.90 (  2.06-  57.56)
 HARDIE 12  f   1         16       -      11       -      3.33 (  1.59-   6.98)
 Subtotal HARDIE                                          4.05 (  2.06-   7.96)
 HARRIS 3   m   0         12     268       6     518      3.87 (  1.43-  10.41)
 HAWTHO 10  m   0        646    8503      49    2097      3.25 (  2.42-   4.36)
 HAWTHO 17  f   0        320    3628     103    3440      2.95 (  2.35-   3.70)
 Subtotal HAWTHO                                          3.06 (  2.55-   3.66)
 HAYES  9   b   3        357       -      37       -      5.70 (  4.08-   7.97)
 HIGGI2 3   m   0         25     248       1      27      2.72 (  0.35-  20.89)
 HIGGI2 15  f   1          8       -       6       -      2.46 (  0.77-   7.83)
 Subtotal HIGGI2                                          2.52 (  0.92-   6.91)
 HIGGI3 3   m   0          6      82       0       6      1.02~(  0.05-  20.27)
 HIGGI3 9   f   0          2      26       0      64     12.17~(  0.57- 262.12)
 Subtotal HIGGI3                                          3.41 (  0.40-  28.99)
 HIGGI6 3   m   1        294       -      17       -      4.59 (  2.78-   7.59)
 HIGGI6 10  f   1         86       -      40       -      2.99 (  2.03-   4.40)
 Subtotal HIGGI6                                          3.51 (  2.58-   4.76)
 HO     11  b   3          -       -       -       -      2.32 (  1.55-   3.46)
 HOLLA2 3   m   0        142     394       5      84      6.05 (  2.41-  15.23)
 HOLLNA 3   m   0        139     254      10     101      5.53 (  2.79-  10.93)
 HOLLNA 11  f   0         72     245      14     217      4.56 (  2.50-   8.31)
 Subtotal HOLLNA                                          4.96 (  3.16-   7.78)
 HOUSE  6   m   1        272       -      51       -      4.21 (  3.08-   5.75)
 HOUSE  12  f   1        194       -     103       -      4.47 (  3.48-   5.75)
 Subtotal HOUSE                                           4.37 (  3.59-   5.31)
 HUCHON 6   b   1        404       -     177       -      2.21 (  1.83-   2.66)
 HUHTI1 42  m   1        177       -       7       -      7.05 (  3.17-  15.67)
  ________________________________________________________________________________________________________________________
                                            International Evidence on Smoking and COPD, Phase 3, Analysis run on 27-SEP-10

                                                   Table 2 - A - 1 - 2

            IESCOPD - Meta-analysis of ever smoking, any product (or cigarettes if all product not available)
                                                          Any CB
                                                      Most-adjusted


                        Number Exposed  Non-exposed
 REF    NRR SEX ADJ     Case    Cont    Case    Cont      RR        95.00%CI
 HUHTI1 91  f   1         16       -      32       -      3.63 (  1.78-   7.40)
 Subtotal HUHTI1                                          4.87 (  2.86-   8.29)
 HUHTI3 22  m   1        272       -      28       -      2.91 (  1.87-   4.51)
 JINDA2 3   b   6        666       -     784       -      2.45 (  2.16-   2.79)
 JOSHI  6   m   0         50     194       9     220      6.30 (  3.02-  13.14)
 JOUSI1 3   m   0       1741    4824     360    2417      2.42 (  2.14-   2.74)
 JOUSI1 6   f   0        278    1344     940    7540      1.66 (  1.43-   1.92)
 Subtotal JOUSI1                                          2.07 (  1.88-   2.27)
*KAHN2  32  m   1         88       -      13       -      2.75 (  1.54-   4.93)
 KATO   3   m   1        827       -     118       -      1.33 (  1.09-   1.63)
 KATO   6   f   1        260       -     679       -      1.92 (  1.65-   2.23)
 Subtotal KATO                                            1.68 (  1.49-   1.90)
 KIRAZ  4   f   1         17       -      78       -      4.00 (  1.76-   9.08)
 KOTAN1 9   b   7        137       -      72       -      1.75 (  1.28-   2.39)
 KUBIK  3   m   0       3253   16409     421    8006      3.77 (  3.39-   4.19)
 KUBIK  4   f   0        298    4760     797   25758      2.02 (  1.76-   2.32)
 Subtotal KUBIK                                           2.99 (  2.75-   3.25)
 LAMBER 6   m   1        257       -      17       -      2.60 (  1.58-   4.30)
 LAMBER 38  f   1        118       -      54       -      2.88 (  2.04-   4.06)
 Subtotal LAMBER                                          2.79 (  2.10-   3.70)
 LANGHA 6   m   1        985       -     203       -      2.66 (  2.28-   3.12)
 LANGHA 12  f   1        635       -     351       -      1.95 (  1.70-   2.24)
 Subtotal LANGHA                                          2.23 (  2.01-   2.48)
 LAVECC 9   b   6       2333       -    1630       -      2.27 (  2.10-   2.46)
 LEBOWI 30  b   3         37       -      26       -      1.13 (  0.68-   1.87)
 LINDST 23  b   5        763       -     494       -      1.52 (  1.34-   1.72)
 LUNDB2 6   m   1        232       -      15       -      7.84 (  4.14-  14.83)
 LUNDB2 12  f   1        106       -      35       -      4.14 (  2.30-   7.44)
 Subtotal LUNDB2                                          5.55 (  3.60-   8.55)
 MAGNUS 6   m   1         93       -      15       -      4.18 (  2.36-   7.38)
 MANFRE 3   m   0         32      79       3      40      5.40 (  1.56-  18.72)
 MANFRE 6   f   0         48     108      22     149      3.01 (  1.72-   5.28)
 Subtotal MANFRE                                          3.32 (  1.99-   5.55)
 MELLST 3   m   0        125     559      15     196      2.92 (  1.67-   5.11)
 MENEZ1 6   b   8        105       -      29       -      2.93 (  1.79-   4.80)
 MEREN  6   b   4          -       -       -       -      1.75 (  1.39-   2.22)
 MILLER 3   m   1         97       -      10       -      5.24 (  2.70-  10.17)
 MILLER 6   f   1         35       -       2       -     20.27 (  4.89-  84.04)
 Subtotal MILLER                                          6.67 (  3.66-  12.17)
 MILNE  3   m   0         65     105       3      40      8.25 (  2.45-  27.77)
 MILNE  15  f   0         13      57      12     186      3.54 (  1.53-   8.18)
 Subtotal MILNE                                           4.65 (  2.33-   9.27)
 MOLLER 3   b   0         13       9       2      11      7.94 (  1.41-  44.80)
 MUELLE 36  m   1         47       -       2       -      7.08 (  1.66-  30.19)
 MUELLE 51  f   1         31       -       3       -     10.49 (  2.74-  40.22)
 Subtotal MUELLE                                          8.75 (  3.27-  23.44)
 NEJJAR 5   b   6        164       -     140       -      1.56 (  1.15-   2.11)
 OGILVI 3   m   0        261     210      11      29      3.28 (  1.60-   6.71)
 OGILVI 8   f   0        106      86      86     160      2.29 (  1.56-   3.38)
 Subtotal OGILVI                                          2.49 (  1.77-   3.49)
 OSWAL2 2   m   1        421       -      96       -      1.41 (  1.11-   1.79)
 OSWAL2 4   f   1        149       -     116       -      1.69 (  1.30-   2.20)
 Subtotal OSWAL2                                          1.53 (  1.28-   1.83)
 PANDEY 6   m   2        238       -       4       -      5.37 (  2.52-  11.45)
 PANDEY 12  f   2        208       -      66       -      1.76 (  1.30-   2.38)
 Subtotal PANDEY                                          2.05 (  1.55-   2.72)
*PELKON 6   m   1          -       -       -       -      1.76 (  1.32-   2.36)
 PEREZP 5   f   0         27      47      77     145      1.08 (  0.63-   1.87)
 REID   6   m   1        248       -      16       -      4.61 (  2.77-   7.70)
 REID   22  f   1         80       -      37       -      3.32 (  2.17-   5.07)
 Subtotal REID                                            3.80 (  2.74-   5.26)
 SAWICK 5   m   0        286    1272      14     353      5.67 (  3.27-   9.82)
 SAWICK 18  f   0         75     649      46    1660      4.17 (  2.86-   6.09)
 Subtotal SAWICK                                          4.60 (  3.37-   6.28)
 SHIMUR 3   b   0          3       2       3       2      1.00 (  0.08-  12.56)
*SILVA  6   b   6          -       -       -       -      1.76 (  0.99-   3.16)
 SOBRAD 4   b   3        167       -      20       -      6.68 (  3.83-  11.63)
 STJERN 3   b   0         15     555       6     873      3.93 (  1.52-  10.20)
*TROISI 3   f   1        545       -     253       -      1.93 (  1.66-   2.24)
 VIEGI1 3   m   0         32    1040       4     504      3.88 (  1.36-  11.02)
 VIEGI1 6   f   0          1     582       7    1119      0.27 (  0.03-   2.24)
  ________________________________________________________________________________________________________________________
                                            International Evidence on Smoking and COPD, Phase 3, Analysis run on 27-SEP-10

                                                   Table 2 - A - 1 - 2

            IESCOPD - Meta-analysis of ever smoking, any product (or cigarettes if all product not available)
                                                          Any CB
                                                      Most-adjusted


                        Number Exposed  Non-exposed
 REF    NRR SEX ADJ     Case    Cont    Case    Cont      RR        95.00%CI
 Subtotal VIEGI1                                          2.29 (  0.90-   5.84)
 WAGEN2 11  b   1        246       -     111       -      1.72 (  1.36-   2.17)
 WILHEL 3   m   0         18     207       1      87      7.57 (  0.99-  57.56)
 WOODS  3   b   5          -       -       -       -      2.46 (  2.12-   2.87)
 WOOLF  3   f   0         92     225      10     173      7.07 (  3.58-  13.99)
 YAMAGU 9   b   6        334       -     128       -      2.26 (  1.82-   2.81)
 ZOIA   3   b   4          -       -       -       -      1.45 (  0.66-   3.16)
Partial Totals         30729   70312   11932   70508
*prospective study                                        ~ With 0.5 adjustment for zero


 REF    NRR SEX ADJ             Ys       Ws       Qs       Ps
 ALDERS 5   m   1              1.03    16.71     0.38       0.00
 ALDERS 6   f   1              1.03    46.22     0.96       0.00
 Subtotal ALDERS               1.03    62.93     1.34
 ANDER1 32  m   1              1.51     3.29     1.28       0.01
 ANDER1 35  f   1              0.79     7.52     0.07       0.03
 Subtotal ANDER1               1.01    10.81     1.35
 BECK1  3   m   0              1.48     2.49     0.90       0.02
 BECK1  6   f   0              0.98     3.64     0.04       0.06
 Subtotal BECK1                1.19     6.13     0.94
*BECK2  3   m   0              0.66     1.45     0.07       0.42
*BECK2  6   f   0              0.76     1.74     0.03       0.32
 Subtotal BECK2                0.71     3.19     0.10
*BEST   3   m   1              2.44     2.89     6.99       0.00
 BJORNS 6   b   4              0.78   204.02     2.14       0.00
 BROWN  1   m   0              0.51    11.71     1.63       0.08
 CERVER 6   b   6              0.92   440.93     0.79       0.00
 COATES 3   b   0              1.29    17.22     2.84       0.00
 COLLEG 6   m   2              1.06     3.30     0.10       0.05
 COLLEG 9   f   1              0.97    13.25     0.10       0.00
 Subtotal COLLEG               0.99    16.56     0.21
 DEANE  6   m   1              1.82     2.62     2.29       0.00
 DEMARC 20  b   1              1.09    67.85     2.81       0.00
*DOLL1  13  m   1              2.00     2.65     3.32       0.00
*DONTA2 3   m   0              1.10     7.69     0.37       0.00
 DOPICO 3   m   0              0.93     4.58     0.01       0.05
 EHRLIC 15  m   6              0.24    15.26     6.31       0.35
 EHRLIC 21  f   6              0.49    30.45     4.56       0.01
 Subtotal EHRLIC               0.41    45.71    10.87
 ENRIGH 3   m   0              0.71    13.33     0.38       0.01
 ENRIGH 6   f   0              0.48    43.33     6.94       0.00
 Subtotal ENRIGH               0.54    56.66     7.32
 FERRI1 61  m   1              1.17     8.10     0.67       0.00
 FERRI1 82  f   1              0.77    16.83     0.19       0.00
 Subtotal FERRI1               0.90    24.93     0.86
 FINKLE 1   m   0              1.23   473.17    58.69       0.00
 FOXMAN 3   m   1              0.91    29.97     0.02       0.00
 FOXMAN 6   f   1              0.95    38.71     0.17       0.00
 Subtotal FOXMAN               0.93    68.68     0.19
 GOLDBE 6   m   1              0.39    86.29    20.70       0.00
 GOLDBE 12  f   1              1.05    46.09     1.37       0.00
 Subtotal GOLDBE               0.62   132.38    22.07
 HAENSZ 10  m   1              0.82    22.99     0.09       0.00
 HAENSZ 32  f   1              1.20    39.61     4.01       0.00
 Subtotal HAENSZ               1.06    62.60     4.10
 HARDIE 9   m   1              2.39     1.39     3.15       0.00
 HARDIE 12  f   1              1.20     7.02     0.72       0.00
 Subtotal HARDIE               1.40     8.41     3.87
 HARRIS 3   m   0              1.35     3.91     0.87       0.01
 HAWTHO 10  m   0              1.18    44.34     3.92       0.00
 HAWTHO 17  f   0              1.08    74.63     2.94       0.00
 Subtotal HAWTHO               1.12   118.97     6.86
 HAYES  9   b   3              1.74    34.27    25.27       0.00
 HIGGI2 3   m   0              1.00     0.93     0.01       0.34
 HIGGI2 15  f   1              0.90     2.86     0.00       0.13
 Subtotal HIGGI2               0.92     3.78     0.01
 HIGGI3 3   m   0              0.02     0.43     0.32       0.99
 HIGGI3 9   f   0              2.50     0.41     1.07       0.11
 Subtotal HIGGI3               1.23     0.84     1.38
 HIGGI6 3   m   1              1.52    15.23     6.28       0.00
  ________________________________________________________________________________________________________________________
                                            International Evidence on Smoking and COPD, Phase 3, Analysis run on 27-SEP-10

                                                   Table 2 - A - 1 - 2

            IESCOPD - Meta-analysis of ever smoking, any product (or cigarettes if all product not available)
                                                          Any CB
                                                      Most-adjusted


 REF    NRR SEX ADJ             Ys       Ws       Qs       Ps
 HIGGI6 10  f   1              1.10    25.68     1.17       0.00
 Subtotal HIGGI6               1.25    40.91     7.45
 HO     11  b   3              0.84    23.83     0.04       0.00
 HOLLA2 3   m   0              1.80     4.51     3.81       0.00
 HOLLNA 3   m   0              1.71     8.26     5.66       0.00
 HOLLNA 11  f   0              1.52    10.64     4.28       0.00
 Subtotal HOLLNA               1.60    18.90     9.94
 HOUSE  6   m   1              1.44    39.43    12.17       0.00
 HOUSE  12  f   1              1.50    60.93    23.09       0.00
 Subtotal HOUSE                1.47   100.36    35.26
 HUCHON 6   b   1              0.79   109.85     0.87       0.00
 HUHTI1 42  m   1              1.95     6.02     6.90       0.00
 HUHTI1 91  f   1              1.29     7.57     1.26       0.00
 Subtotal HUHTI1               1.58    13.59     8.16
 HUHTI3 22  m   1              1.07    19.83     0.69       0.00
 JINDA2 3   b   6              0.90   234.59     0.05       0.00
 JOSHI  6   m   0              1.84     7.10     6.53       0.00
 JOUSI1 3   m   0              0.89   251.69     0.00       0.00
 JOUSI1 6   f   0              0.51   180.58    25.47       0.00
 Subtotal JOUSI1               0.73   432.27    25.47
*KAHN2  32  m   1              1.01    11.35     0.19       0.00
 KATO   3   m   1              0.29    94.89    33.78       0.01
 KATO   6   f   1              0.65   169.34     8.92       0.00
 Subtotal KATO                 0.52   264.24    42.70
 KIRAZ  4   f   1              1.39     5.71     1.45       0.00
 KOTAN1 9   b   7              0.56    39.41     4.09       0.00
 KUBIK  3   m   0              1.33   348.61    69.10       0.00
 KUBIK  4   f   0              0.70   205.79     6.46       0.00
 Subtotal KUBIK                1.10   554.40    75.56
 LAMBER 6   m   1              0.96    15.33     0.08       0.00
 LAMBER 38  f   1              1.06    32.44     1.00       0.00
 Subtotal LAMBER               1.02    47.77     1.09
 LANGHA 6   m   1              0.98   156.19     1.45       0.00
 LANGHA 12  f   1              0.67   201.94     9.25       0.00
 Subtotal LANGHA               0.80   358.12    10.70
 LAVECC 9   b   6              0.82   613.78     2.36       0.00
 LEBOWI 30  b   3              0.12    15.02     8.66       0.64
 LINDST 23  b   5              0.42   246.53    52.88       0.00
 LUNDB2 6   m   1              2.06     9.44    13.08       0.00
 LUNDB2 12  f   1              1.42    11.15     3.24       0.00
 Subtotal LUNDB2               1.71    20.59    16.32
 MAGNUS 6   m   1              1.43    11.82     3.56       0.00
 MANFRE 3   m   0              1.69     2.49     1.61       0.01
 MANFRE 6   f   0              1.10    12.16     0.59       0.00
 Subtotal MANFRE               1.20    14.64     2.20
 MELLST 3   m   0              1.07    12.26     0.44       0.00
 MENEZ1 6   b   8              1.08    15.79     0.59       0.00
 MEREN  6   b   4              0.56    70.09     7.28       0.00
 MILLER 3   m   1              1.66     8.74     5.24       0.00
 MILLER 6   f   1              3.01     1.90     8.60       0.00
 Subtotal MILLER               1.90    10.64    13.84
 MILNE  3   m   0              2.11     2.61     3.94       0.00
 MILNE  15  f   0              1.26     5.46     0.79       0.00
 Subtotal MILNE                1.54     8.07     4.73
 MOLLER 3   b   0              2.07     1.28     1.82       0.02
 MUELLE 36  m   1              1.96     1.83     2.11       0.01
 MUELLE 51  f   1              2.35     2.13     4.59       0.00
 Subtotal MUELLE               2.17     3.96     6.70
 NEJJAR 5   b   6              0.44    41.71     7.97       0.00
 OGILVI 3   m   0              1.19     7.46     0.69       0.00
 OGILVI 8   f   0              0.83    25.68     0.07       0.00
 Subtotal OGILVI               0.91    33.14     0.76
 OSWAL2 2   m   1              0.34    67.29    19.50       0.00
 OSWAL2 4   f   1              0.52    55.52     7.08       0.00
 Subtotal OSWAL2               0.43   122.81    26.58
 PANDEY 6   m   2              1.68     6.71     4.28       0.00
 PANDEY 12  f   2              0.57    42.02     4.21       0.00
 Subtotal PANDEY               0.72    48.72     8.49
*PELKON 6   m   1              0.57    45.52     4.56       0.00
 PEREZP 5   f   0              0.08    12.79     8.25       0.78
 REID   6   m   1              1.53    14.70     6.14       0.00
  ________________________________________________________________________________________________________________________
                                            International Evidence on Smoking and COPD, Phase 3, Analysis run on 27-SEP-10

                                                   Table 2 - A - 1 - 2

            IESCOPD - Meta-analysis of ever smoking, any product (or cigarettes if all product not available)
                                                          Any CB
                                                      Most-adjusted


 REF    NRR SEX ADJ             Ys       Ws       Qs       Ps
 REID   22  f   1              1.20    21.34     2.16       0.00
 Subtotal REID                 1.33    36.04     8.30
 SAWICK 5   m   0              1.74    12.73     9.27       0.00
 SAWICK 18  f   0              1.43    26.87     8.01       0.00
 Subtotal SAWICK               1.53    39.60    17.28
 SHIMUR 3   b   0              0.00     0.60     0.47       1.00
*SILVA  6   b   6              0.57    11.41     1.14       0.06
 SOBRAD 4   b   3              1.90    12.46    12.89       0.00
 STJERN 3   b   0              1.37     4.23     1.01       0.00
*TROISI 3   f   1              0.66   171.12     8.61       0.00
 VIEGI1 3   m   0              1.36     3.52     0.79       0.01
 VIEGI1 6   f   0             -1.29     0.87     4.13       0.23
 Subtotal VIEGI1               0.83     4.39     4.91
 WAGEN2 11  b   1              0.54    70.38     8.11       0.00
 WILHEL 3   m   0              2.02     0.93     1.22       0.05
 WOODS  3   b   5              0.90   167.48     0.06       0.00
 WOOLF  3   f   0              1.96     8.26     9.54       0.00
 YAMAGU 9   b   6              0.82    81.45     0.36       0.00
 ZOIA   3   b   4              0.37     6.27     1.63       0.35

                       N      114
                      NS       79


                      Wt  6146.59
                 Het Chi   657.06
                 Het  df      113
                 Het  P       ***
               Fixed  RR     2.42
                     RRl     2.36
                     RRu     2.48
                      P       +++
              Random  RR     2.69
                     RRl     2.50
                     RRu     2.90
                      P       +++
               Asymm  P         *


  ________________________________________________________________________________________________________________________
                                            International Evidence on Smoking and COPD, Phase 3, Analysis run on 27-SEP-10

                                                   Table 2 - A - 1 - 3

            IESCOPD - Meta-analysis of ever smoking, any product (or cigarettes if all product not available)
                                                          Any CB
                                                      Most-adjusted


                       N      114
                      NS       79


                      Wt  6146.59
                 Het Chi   657.06
                 Het  df      113
                 Het  P       ***
               Fixed  RR     2.42
                     RRl     2.36
                     RRu     2.48
                      P       +++
              Random  RR     2.69
                     RRl     2.50
                     RRu     2.90
                      P       +++
               Asymm  P         *

                                   Sex
                             both      male    female     Total


                       N       24        51        39       114
                      NS       24        51        39       114


                      Wt  2530.44   1945.95   1670.19   6146.59
                 Het Chi   131.74    277.78    162.89    657.06
                 Het  df       23        50        38       113
                 Het  P       ***       ***       ***       ***
               Fixed  RR     2.24      2.87      2.22      2.42
                     RRl     2.15      2.74      2.11      2.36
                     RRu     2.33      3.00      2.32      2.48
                      P       +++       +++       +++       +++
              Random  RR     2.29      3.18      2.57      2.69
                     RRl     2.05      2.77      2.28      2.50
                     RRu     2.57      3.65      2.89      2.90
                      P       +++       +++       +++       +++
             Between Chi                                  84.65
             Between  df                                      2
             Between  P                                     ***
             Btwn(F)  P                                     ***

                                        Continent
                            NAmer    Europe      Asia  oth/mult     Total


                       N       38        60         9         7       114
                      NS       25        41         7         6        79


                      Wt  1265.74   3906.79    660.52    313.54   6146.59
                 Het Chi   190.81    336.35     43.44     23.11    657.06
                 Het  df       37        59         8         6       113
                 Het  P       ***       ***       ***       ***       ***
               Fixed  RR     2.91      2.34      2.08      2.34      2.42
                     RRl     2.75      2.27      1.92      2.09      2.36
                     RRu     3.07      2.41      2.24      2.61      2.48
                      P       +++       +++       +++       +++       +++
              Random  RR     3.17      2.62      2.21      2.09      2.69
                     RRl     2.70      2.39      1.78      1.59      2.50
                     RRu     3.71      2.89      2.75      2.74      2.90
                      P       +++       +++       +++       +++       +++
             Between Chi                                            63.35
             Between  df                                                3
             Between  P                                               ***
             Btwn(F)  P                                                 *


  ________________________________________________________________________________________________________________________
                                            International Evidence on Smoking and COPD, Phase 3, Analysis run on 27-SEP-10

                                                   Table 2 - A - 1 - 3

            IESCOPD - Meta-analysis of ever smoking, any product (or cigarettes if all product not available)
                                                          Any CB
                                                      Most-adjusted
                        National cigarette tobacco type (excluding mixed/unkown)
                          blended  virginia     Total


                       N       78        34       112
                      NS       56        21        77


                      Wt  5023.95    973.34   5997.29
                 Het Chi   559.04     94.84    653.88
                 Het  df       77        33       111
                 Het  P       ***       ***       ***
               Fixed  RR     2.41      2.42      2.41
                     RRl     2.35      2.27      2.35
                     RRu     2.48      2.57      2.47
                      P       +++       +++       +++
              Random  RR     2.71      2.64      2.70
                     RRl     2.47      2.31      2.50
                     RRu     2.97      3.02      2.91
                      P       +++       +++       +++
             Between Chi                         0.00
             Between  df                            1
             Between  P                          N.S.
             Btwn(F)  P                          N.S.

                                        Start year of study
                            <1970   1970-79   1980-89   1990-99     2000+   unknown     Total


                       N       42        26        12        18         1        15       114
                      NS       27        15         8        15         1        13        79


                      Wt  1167.90   1457.51   1216.46   1358.65     70.38    875.69   6146.59
                 Het Chi   129.95    219.96     57.55     73.33      0.00     64.38    657.06
                 Het  df       41        25        11        17         0        14       113
                 Het  P       ***       ***       ***       ***      N.S.       ***       ***
               Fixed  RR     2.98      2.63      2.09      2.29      1.72      2.16      2.42
                     RRl     2.82      2.50      1.97      2.17      1.36      2.02      2.36
                     RRu     3.16      2.77      2.21      2.41      2.17      2.31      2.48
                      P       +++       +++       +++       +++       +++       +++       +++
              Random  RR     3.11      2.94      2.17      2.27      1.72      2.55      2.69
                     RRl     2.72      2.45      1.82      1.98      1.36      2.10      2.50
                     RRu     3.55      3.54      2.60      2.60      2.17      3.10      2.90
                      P       +++       +++       +++       +++       +++       +++       +++
             Between Chi                                                               111.89
             Between  df                                                                    5
             Between  P                                                                   ***
             Btwn(F)  P                                                                    **

                                Publication year
                            <1980   1980-89   1990-99     2000+     Total


                       N       49        28        17        20       114
                      NS       31        17        14        17        79


                      Wt  1425.99   1780.85   1004.03   1935.72   6146.59
                 Het Chi   210.86    164.52     64.84    105.48    657.06
                 Het  df       48        27        16        19       113
                 Het  P       ***       ***       ***       ***       ***
               Fixed  RR     3.02      2.44      2.09      2.19      2.42
                     RRl     2.86      2.33      1.96      2.09      2.36
                     RRu     3.18      2.56      2.22      2.29      2.48
                      P       +++       +++       +++       +++       +++
              Random  RR     3.22      2.72      2.24      2.19      2.69
                     RRl     2.82      2.33      1.91      1.93      2.50
                     RRu     3.68      3.19      2.63      2.48      2.90
                      P       +++       +++       +++       +++       +++
             Between Chi                                           111.36
             Between  df                                                3
             Between  P                                               ***
             Btwn(F)  P                                               ***
  ________________________________________________________________________________________________________________________
                                            International Evidence on Smoking and COPD, Phase 3, Analysis run on 27-SEP-10

                                                   Table 2 - A - 1 - 3

            IESCOPD - Meta-analysis of ever smoking, any product (or cigarettes if all product not available)
                                                          Any CB
                                                      Most-adjusted
                               Study type
                               CC        Pr        CS     Total


                       N       10        11        93       114
                      NS        7         9        63        79


                      Wt   201.72    374.78   5570.09   6146.59
                 Het Chi    36.91     31.23    587.75    657.06
                 Het  df        9        10        92       113
                 Het  P       ***       ***       ***       ***
               Fixed  RR     2.35      2.30      2.43      2.42
                     RRl     2.04      2.08      2.36      2.36
                     RRu     2.69      2.55      2.49      2.48
                      P       +++       +++       +++       +++
              Random  RR     2.72      2.60      2.70      2.69
                     RRl     1.95      2.04      2.49      2.50
                     RRu     3.77      3.30      2.93      2.90
                      P       +++       +++       +++       +++
             Between Chi                                   1.18
             Between  df                                      2
             Between  P                                    N.S.
             Btwn(F)  P                                    N.S.

                                    Lowest age in RR
                        <25/unlim     25-39       40+   unknown     Total


                       N       49        32        33                 114
                      NS       34        21        24                  79


                      Wt  3996.80   1347.65    802.13             6146.59
                 Het Chi   421.91     96.19    132.15              657.06
                 Het  df       48        31        32                 113
                 Het  P       ***       ***       ***                 ***
               Fixed  RR     2.46      2.40      2.23                2.42
                     RRl     2.39      2.27      2.08                2.36
                     RRu     2.54      2.53      2.39                2.48
                      P       +++       +++       +++                 +++
              Random  RR     2.56      2.83      2.80                2.69
                     RRl     2.29      2.52      2.36                2.50
                     RRu     2.87      3.19      3.31                2.90
                      P       +++       +++       +++                 +++
             Between Chi                                             6.82
             Between  df                                                2
             Between  P                                                 *
             Btwn(F)  P                                              N.S.

                                         Highest age in RR
                              <65     65-74     75-84 85+/unlim   unknown     Total


                       N       27        37         7        42         1       114
                      NS       20        24         6        28         1        79


                      Wt  1995.55   1114.62    195.95   2835.89      4.58   6146.59
                 Het Chi   142.89    165.16     13.37    303.36      0.00    657.06
                 Het  df       26        36         6        41         0       113
                 Het  P       ***       ***         *       ***      N.S.       ***
               Fixed  RR     2.62      2.15      2.71      2.37      2.53      2.42
                     RRl     2.51      2.03      2.36      2.28      1.01      2.36
                     RRu     2.74      2.28      3.12      2.46      6.32      2.48
                      P       +++       +++       +++       +++         +       +++
              Random  RR     3.02      2.77      2.76      2.47      2.53      2.69
                     RRl     2.62      2.39      2.16      2.18      1.01      2.50
                     RRu     3.49      3.21      3.53      2.79      6.32      2.90
                      P       +++       +++       +++       +++         +       +++
             Between Chi                                                      32.28
             Between  df                                                          4
             Between  P                                                         ***
             Btwn(F)  P                                                        N.S.
  ________________________________________________________________________________________________________________________
                                            International Evidence on Smoking and COPD, Phase 3, Analysis run on 27-SEP-10

                                                   Table 2 - A - 1 - 3

            IESCOPD - Meta-analysis of ever smoking, any product (or cigarettes if all product not available)
                                                          Any CB
                                                      Most-adjusted
                           Study weakness
                              Yes        No     Total


                       N        5       109       114
                      NS        4        75        79


                      Wt    30.16   6116.42   6146.59
                 Het Chi     5.98    638.02    657.06
                 Het  df        4       108       113
                 Het  P      N.S.       ***       ***
               Fixed  RR     4.66      2.41      2.42
                     RRl     3.26      2.35      2.36
                     RRu     6.65      2.47      2.48
                      P       +++       +++       +++
              Random  RR     4.59      2.65      2.69
                     RRl     2.84      2.46      2.50
                     RRu     7.42      2.86      2.90
                      P       +++       +++       +++
             Between Chi                        13.06
             Between  df                            1
             Between  P                           ***
             Btwn(F)  P                          N.S.

                           CB subtype
                             mort     sympt     other     Total


                       N        3        83        28       114
                      NS        3        56        20        79


                      Wt    16.89   4727.25   1402.45   6146.59
                 Het Chi     5.77    515.30     98.70    657.06
                 Het  df        2        82        27       113
                 Het  P       (*)       ***       ***       ***
               Fixed  RR     4.10      2.51      2.11      2.42
                     RRl     2.54      2.44      2.00      2.36
                     RRu     6.60      2.58      2.22      2.48
                      P       +++       +++       +++       +++
              Random  RR     5.49      2.78      2.31      2.69
                     RRl     2.14      2.55      2.01      2.50
                     RRu    14.11      3.03      2.65      2.90
                      P       +++       +++       +++       +++
             Between Chi                                  37.28
             Between  df                                      2
             Between  P                                     ***
             Btwn(F)  P                                       *

                           Asthma analysis type (CB)
                        inc-irres  excl-all excl-cntr     Total


                       N      103         7         4       114
                      NS       69         7         3        79


                      Wt  5804.45    319.67     22.47   6146.59
                 Het Chi   597.60     22.90      4.00    657.06
                 Het  df      102         6         3       113
                 Het  P       ***       ***      N.S.       ***
               Fixed  RR     2.44      1.91      5.41      2.42
                     RRl     2.38      1.71      3.58      2.36
                     RRu     2.50      2.13      8.18      2.48
                      P       +++       +++       +++       +++
              Random  RR     2.69      2.13      5.37      2.69
                     RRl     2.50      1.61      3.13      2.50
                     RRu     2.91      2.80      9.21      2.90
                      P       +++       +++       +++       +++
             Between Chi                                  32.56
             Between  df                                      2
             Between  P                                     ***
             Btwn(F)  P                                     (*)
  ________________________________________________________________________________________________________________________
                                            International Evidence on Smoking and COPD, Phase 3, Analysis run on 27-SEP-10

                                                   Table 2 - A - 1 - 3

            IESCOPD - Meta-analysis of ever smoking, any product (or cigarettes if all product not available)
                                                          Any CB
                                                      Most-adjusted
                                   Number of CB cases
                             1-50    51-100   101-200      201+     Total


                       N       17        13        19        65       114
                      NS       12         9        16        42        79


                      Wt    40.13     66.04    192.41   5848.01   6146.59
                 Het Chi    13.85     20.99     61.64    535.84    657.06
                 Het  df       16        12        18        64       113
                 Het  P      N.S.       (*)       ***       ***       ***
               Fixed  RR     2.70      3.71      3.07      2.38      2.42
                     RRl     1.98      2.91      2.66      2.32      2.36
                     RRu     3.69      4.72      3.53      2.44      2.48
                      P       +++       +++       +++       +++       +++
              Random  RR     2.70      4.15      3.48      2.51      2.69
                     RRl     1.98      2.97      2.64      2.31      2.50
                     RRu     3.69      5.80      4.58      2.72      2.90
                      P       +++       +++       +++       +++       +++
             Between Chi                                            24.74
             Between  df                                                3
             Between  P                                               ***
             Btwn(F)  P                                              N.S.

                            Analysis type
                         prevlnce     onset     Total


                       N      105         9       114
                      NS       71         8        79


                      Wt  5890.78    255.81   6146.59
                 Het Chi   631.43     16.88    657.06
                 Het  df      104         8       113
                 Het  P       ***         *       ***
               Fixed  RR     2.43      2.02      2.42
                     RRl     2.37      1.78      2.36
                     RRu     2.50      2.28      2.48
                      P       +++       +++       +++
              Random  RR     2.71      2.37      2.69
                     RRl     2.51      1.80      2.50
                     RRu     2.93      3.13      2.90
                      P       +++       +++       +++
             Between Chi                         8.76
             Between  df                            1
             Between  P                            **
             Btwn(F)  P                          N.S.

                             Smoking product
                              any      cigs  cigsonly     Total


                       N       61        51         2       114
                      NS       42        38         2        82


                      Wt  3540.79   2556.69     49.11   6146.59
                 Het Chi   300.01    303.82      5.41    657.06
                 Het  df       60        50         1       113
                 Het  P       ***       ***         *       ***
               Fixed  RR     2.59      2.18      3.03      2.42
                     RRl     2.51      2.10      2.29      2.36
                     RRu     2.68      2.26      4.01      2.48
                      P       +++       +++       +++       +++
              Random  RR     2.89      2.44      5.04      2.69
                     RRl     2.63      2.17      1.29      2.50
                     RRu     3.19      2.73     19.70      2.90
                      P       +++       +++         +       +++
             Between Chi                                  47.81
             Between  df                                      2
             Between  P                                     ***
             Btwn(F)  P                                       *
  ________________________________________________________________________________________________________________________
                                            International Evidence on Smoking and COPD, Phase 3, Analysis run on 27-SEP-10

                                                   Table 2 - A - 1 - 3

            IESCOPD - Meta-analysis of ever smoking, any product (or cigarettes if all product not available)
                                                          Any CB
                                                      Most-adjusted
                                     Unexposed group
                          nev any   nev cig  nev+ any  nev+ cig     Total


                       N       68        40         2         4       114
                      NS       46        32         1         2        81


                      Wt  3117.06   2233.35    554.40    241.78   6146.59
                 Het Chi   250.98    260.72     50.12     30.18    657.06
                 Het  df       67        39         1         3       113
                 Het  P       ***       ***       ***       ***       ***
               Fixed  RR     2.54      2.16      2.99      2.15      2.42
                     RRl     2.45      2.07      2.75      1.90      2.36
                     RRu     2.63      2.25      3.25      2.44      2.48
                      P       +++       +++       +++       +++       +++
              Random  RR     2.89      2.46      2.77      2.18      2.69
                     RRl     2.64      2.16      1.50      1.46      2.50
                     RRu     3.17      2.79      5.09      3.26      2.90
                      P       +++       +++        ++       +++       +++
             Between Chi                                            65.06
             Between  df                                                3
             Between  P                                               ***
             Btwn(F)  P                                                **

                        Unexposed group (combining nev+ with main levels)
                          nev any   nev cig     Total


                       N       70        44       114
                      NS       47        34        81


                      Wt  3671.46   2475.13   6146.59
                 Het Chi   313.58    290.91    657.06
                 Het  df       69        43       113
                 Het  P       ***       ***       ***
               Fixed  RR     2.61      2.16      2.42
                     RRl     2.52      2.07      2.36
                     RRu     2.69      2.24      2.48
                      P       +++       +++       +++
              Random  RR     2.90      2.42      2.69
                     RRl     2.65      2.15      2.50
                     RRu     3.18      2.73      2.90
                      P       +++       +++       +++
             Between Chi                        52.58
             Between  df                            1
             Between  P                           ***
             Btwn(F)  P                            **

                        Smoking results reported in study (CB)
                             Ever   Current      Both     Total


                       N        8                 106       114
                      NS        6                  73        79


                      Wt  1403.46             4743.13   6146.59
                 Het Chi   134.40              475.78    657.06
                 Het  df        7                 105       113
                 Het  P       ***                 ***       ***
               Fixed  RR     2.84                2.30      2.42
                     RRl     2.69                2.24      2.36
                     RRu     2.99                2.37      2.48
                      P       +++                 +++       +++
              Random  RR     2.26                2.72      2.69
                     RRl     1.74                2.53      2.50
                     RRu     2.93                2.94      2.90
                      P       +++                 +++       +++
             Between Chi                                  46.87
             Between  df                                      1
             Between  P                                     ***
             Btwn(F)  P                                      **
  ________________________________________________________________________________________________________________________
                                            International Evidence on Smoking and COPD, Phase 3, Analysis run on 27-SEP-10

                                                   Table 2 - A - 1 - 3

            IESCOPD - Meta-analysis of ever smoking, any product (or cigarettes if all product not available)
                                                          Any CB
                                                      Most-adjusted
                        Number of adjustment variables
                                0         1        2+     Total


                       N       42        50        22       114
                      NS       29        32        20        81


                      Wt  1862.38   1927.44   2356.76   6146.59
                 Het Chi   206.41    250.16    128.96    657.06
                 Het  df       41        49        21       113
                 Het  P       ***       ***       ***       ***
               Fixed  RR     2.84      2.30      2.21      2.42
                     RRl     2.71      2.20      2.12      2.36
                     RRu     2.97      2.41      2.30      2.48
                      P       +++       +++       +++       +++
              Random  RR     3.01      2.84      2.18      2.69
                     RRl     2.60      2.53      1.93      2.50
                     RRu     3.48      3.19      2.46      2.90
                      P       +++       +++       +++       +++
             Between Chi                                  71.53
             Between  df                                      2
             Between  P                                     ***
             Btwn(F)  P                                      **

                        RR adjusted for sex (combined sex RR only)
                              Yes        No     Total


                       N       17         7        24
                      NS       17         7        24


                      Wt  2259.03    271.42   2530.44
                 Het Chi   113.70     17.57    131.74
                 Het  df       16         6        23
                 Het  P       ***        **       ***
               Fixed  RR     2.23      2.33      2.24
                     RRl     2.14      2.07      2.15
                     RRu     2.32      2.62      2.33
                      P       +++       +++       +++
              Random  RR     2.24      2.55      2.29
                     RRl     1.96      1.95      2.05
                     RRu     2.55      3.32      2.57
                      P       +++       +++       +++
             Between Chi                         0.47
             Between  df                            1
             Between  P                          N.S.
             Btwn(F)  P                          N.S.

                        RR adjusted for age


                       N       63        51       114
                      NS       44        36        80


                      Wt  3478.50   2668.09   6146.59
                 Het Chi   299.05    266.04    657.06
                 Het  df       62        50       113
                 Het  P       ***       ***       ***
               Fixed  RR     2.17      2.78      2.42
                     RRl     2.10      2.67      2.36
                     RRu     2.24      2.88      2.48
                      P       +++       +++       +++
              Random  RR     2.46      3.03      2.69
                     RRl     2.26      2.69      2.50
                     RRu     2.69      3.41      2.90
                      P       +++       +++       +++
             Between Chi                        91.97
             Between  df                            1
             Between  P                           ***
             Btwn(F)  P                           ***

  ________________________________________________________________________________________________________________________
                                            International Evidence on Smoking and COPD, Phase 3, Analysis run on 27-SEP-10

                                                   Table 2 - A - 1 - 3

            IESCOPD - Meta-analysis of ever smoking, any product (or cigarettes if all product not available)
                                                          Any CB
                                                      Most-adjusted
                        RR adjusted for factor other than sex, age
                              Yes        No     Total


                       N       30        84       114
                      NS       26        54        80


                      Wt  2721.55   3425.04   6146.59
                 Het Chi   200.98    439.88    657.06
                 Het  df       29        83       113
                 Het  P       ***       ***       ***
               Fixed  RR     2.28      2.53      2.42
                     RRl     2.20      2.45      2.36
                     RRu     2.37      2.61      2.48
                      P       +++       +++       +++
              Random  RR     2.41      2.86      2.69
                     RRl     2.14      2.60      2.50
                     RRu     2.71      3.15      2.90
                      P       +++       +++       +++
             Between Chi                        16.20
             Between  df                            1
             Between  P                           ***
             Btwn(F)  P                           (*)

                        Derivation of RR/CI
                         Orig/2x2     Other     Total


                       N        8       106       114
                      NS        5        74        79


                      Wt   637.93   5508.66   6146.59
                 Het Chi    55.86    568.36    657.06
                 Het  df        7       105       113
                 Het  P       ***       ***       ***
               Fixed  RR     2.99      2.36      2.42
                     RRl     2.77      2.29      2.36
                     RRu     3.24      2.42      2.48
                      P       +++       +++       +++
              Random  RR     2.97      2.66      2.69
                     RRl     2.16      2.47      2.50
                     RRu     4.08      2.88      2.90
                      P       +++       +++       +++
             Between Chi                        32.84
             Between  df                            1
             Between  P                           ***
             Btwn(F)  P                             *


  ________________________________________________________________________________________________________________________
                                            International Evidence on Smoking and COPD, Phase 3, Analysis run on 27-SEP-10

                                                   Table 2 - A - 1 - 4

            IESCOPD - Meta-analysis of ever smoking, any product (or cigarettes if all product not available)
                                                          Any CB
                                                      Least-adjusted


     REF|NRR|X|SEX|AGEL|AGEH|     REGION|BEGYR|PUBYR|STTYP|ONSET|      DISEAS|ADJ|SMOKSTA|   PRODUCT|    UNEXP|

  ALDERS   1 x   m   35   74       Eu:UK  1977  1985    CC  Prev CB:diagnosed   0    Ever        Any   Nev any
  ALDERS   2 x   f   35   74       Eu:UK  1977  1985    CC  Prev CB:diagnosed   0    Ever MCigs only   Nev any
  ANDER1  32     m   25   74   Am:Canada  1963  1965    CS  Prev  CB:symptoms   1    Ever        Any   Nev any
  ANDER1  35     f   25   74   Am:Canada  1963  1965    CS  Prev  CB:symptoms   1    Ever        Any   Nev any
   BECK1   3     m   11   99      Am:USA  1972  1982    CS  Prev  CB:symptoms   0    Ever       Cigs   Nev any
   BECK1   6     f   11   99      Am:USA  1972  1982    CS  Prev  CB:symptoms   0    Ever       Cigs   Nev any
   BECK2   3     m   11   99      Am:USA  1972  1982    Pr   Inc  CB:symptoms   0    Ever       Cigs   Nev any
   BECK2   6     f   11   99      Am:USA  1972  1982    Pr   Inc  CB:symptoms   0    Ever       Cigs   Nev any
    BEST   3     m   30   97   Am:Canada  1955  1967    Pr   Inc CB:mortality   1    Ever  Cigs only   Nev any
  BJORNS   3 x   b   20   44    Eu:Scand  1990  1994    CS  Prev  CB:symptoms   0    Ever        Any   Nev any
   BROWN   1     m   60   69       Eu:UK  1956  1957    CS  Prev CB:diagnosed   0    Ever        Any   Nev any
  CERVER   3 x   b   20   44     Eu:West  1998  2003    CS  Prev  CB:symptoms   3    Ever       Cigs  Nev cigs
  COATES   3     b   40   64      Am:USA  1962  1965    CS  Prev  CB:symptoms   0    Ever       Cigs  Nev cigs
  COLLEG   3 x   m   40   64       Eu:UK     *  1961    CS  Prev CB:diagnosed   1    Ever        Any   Nev any
  COLLEG   9     f   40   64       Eu:UK     *  1961    CS  Prev CB:diagnosed   1    Ever        Any   Nev any
   DEANE   5 x   m   40   59      Am:USA  1963  1965    CS  Prev  CB:symptoms   0    Ever        Any   Nev any
  DEMARC  17 x   b   20   44       Multi  1991  2004    CS  Prev  CB:symptoms   0    Ever       Cigs   Nev any
   DOLL1  13     m   20   99       Eu:UK  1951  1994    Pr   Inc CB:mortality   1    Ever        Any   Nev any
  DONTA2   3     m   25   84 Eu:SE/Balkn  1960  1984    Pr   Inc CB:diagnosed   0    Ever       Cigs  Nev cigs
  DOPICO   3     m   15    *      Am:USA     *  1984    CS  Prev  CB:symptoms   0    Ever       Cigs  Nev cigs
  EHRLIC   3 x   m   15   99      Africa  1998  2004    CS  Prev  CB:symptoms   0    Ever        Any   Nev any
  EHRLIC   9 x   f   15   99      Africa  1998  2004    CS  Prev  CB:symptoms   0    Ever        Any   Nev any
  ENRIGH   3     m   65   99      Am:USA  1989  1994    CS  Prev CB:diagnosed   0    Ever       Cigs  Nev cigs
  ENRIGH   6     f   65   99      Am:USA  1989  1994    CS  Prev CB:diagnosed   0    Ever       Cigs  Nev cigs
  FERRI1  61     m   25   74      Am:USA  1961  1971    CS  Prev  CB:symptoms   1    Ever        Any   Nev any
  FERRI1  82     f   25   74      Am:USA  1961  1971    CS  Prev  CB:symptoms   1    Ever        Any   Nev any
  FINKLE   1     m   18   25      Am:USA  1969  1974    CS  Prev  CB:symptoms   0    Ever        Any   Nev any
  FOXMAN   3     m   20   69      Am:USA     *  1986    CS  Prev  CB:symptoms   1    Ever        Any   Nev any
  FOXMAN   6     f   20   69      Am:USA     *  1986    CS  Prev  CB:symptoms   1    Ever        Any   Nev any
  GOLDBE   3 x   m   15   99      Am:USA  1970  1974    CS  Prev  CB:symptoms   0    Ever       Cigs  Nev cigs
  GOLDBE   9 x   f   15   99      Am:USA  1970  1974    CS  Prev  CB:symptoms   0    Ever       Cigs  Nev cigs
  HAENSZ   3 x   m   35   74    Eu:Scand  1964  1972    CS  Prev  CB:symptoms   0    Ever        Any   Nev any
  HAENSZ  17 x   f   35   74    Eu:Scand  1964  1972    CS  Prev  CB:symptoms   0    Ever        Any   Nev any
  HARDIE   9     m   70   99    Eu:Scand  1998  2005    CS  Prev CB:diagnosed   1    Ever       Cigs  Nev cigs
  HARDIE  12     f   70   99    Eu:Scand  1998  2005    CS  Prev CB:diagnosed   1    Ever       Cigs  Nev cigs
  HARRIS   3     m   15   60      Africa     *  1993    CS  Prev  CB:symptoms   0    Ever        Any   Nev any
  HAWTHO  10     m   45   76       Eu:UK  1965  1978    Pr  Prev  CB:symptoms   0    Ever       Cigs  N/L cigs
  HAWTHO  17     f   45   76       Eu:UK  1965  1978    Pr  Prev  CB:symptoms   0    Ever       Cigs  N/L cigs
   HAYES   3 x   m   15   99      Am:USA  1970  1974    CS  Prev  CB:symptoms   0    Ever       Cigs  Nev cigs
   HAYES   6 x   f   15   99      Am:USA  1970  1974    CS  Prev  CB:symptoms   0    Ever       Cigs  Nev cigs
  HIGGI2   3     m   25   74       Eu:UK  1956  1957    CS  Prev  CB:symptoms   0    Ever       Cigs  Nev cigs
  HIGGI2   9 x   f   25   74       Eu:UK  1956  1957    CS  Prev  CB:symptoms   0    Ever       Cigs  Nev cigs
  HIGGI3   3     m   55   64       Eu:UK  1956  1958    CS  Prev  CB:symptoms   0    Ever        Any   Nev any
  HIGGI3   9     f   55   64       Eu:UK  1956  1958    CS  Prev  CB:symptoms   0    Ever        Any   Nev any
  HIGGI6   3     m   25   64      Am:USA  1962  1977    CS  Prev  CB:symptoms   1    Ever        Any   Nev any
  HIGGI6  10     f   25   64      Am:USA  1962  1977    CS  Prev  CB:symptoms   1    Ever        Any   Nev any
      HO  11     b   70   99   Asia:FarE  1991  1999    CS  Prev  CB:self-rep   3    Ever       Cigs  Nev cigs
  HOLLA2   3     m   40   59      Am:USA  1962  1965    CS  Prev  CB:symptoms   0    Ever        Any   Nev any
  HOLLNA   3     m   40   40    Eu:Scand  1976  1983    CS  Prev  CB:symptoms   0    Ever        Any   Nev any
  HOLLNA  11     f   40   40    Eu:Scand  1976  1983    CS  Prev  CB:symptoms   0    Ever        Any   Nev any
   HOUSE   3 x   m   15   99      Am:USA  1970  1974    CS  Prev  CB:symptoms   0    Ever       Cigs  Nev cigs
   HOUSE   9 x   f   15   99      Am:USA  1970  1974    CS  Prev  CB:symptoms   0    Ever       Cigs  Nev cigs
  HUCHON   3 x   b   25   99     Eu:West     *  2002    CS  Prev  CB:symptoms   0    Ever       Cigs  Nev cigs
  HUHTI1  39 x   m   40   64    Eu:Scand  1961  1965    CS  Prev  CB:symptoms   0    Ever        Any   Nev any
  HUHTI1  88 x   f   40   64    Eu:Scand  1961  1965    CS  Prev  CB:symptoms   0    Ever        Any   Nev any
  HUHTI3  19 x   m   25   69    Eu:Scand  1968  1978    CS  Prev  CB:symptoms   0    Ever        Any   Nev any
  JINDA2   1 x   b   35   99 Asia:MidE/S     *  2006    CS  Prev  CB:symptoms   0    Ever        Any   Nev any
   JOSHI   6     m   17   64 Asia:MidE/S     *  1975    CS  Prev  CB:symptoms   0    Ever        Any   Nev any
  JOUSI1   3     m   25   64    Eu:Scand  1972  1996    CS  Prev  CB:symptoms   0    Ever        Any   Nev any
  JOUSI1   6     f   25   64    Eu:Scand  1972  1996    CS  Prev  CB:symptoms   0    Ever        Any   Nev any
   KAHN2  32     m   31   84      Am:USA  1954  1966    Pr   Inc CB:mortality   1    Ever        Any   Nev any
    KATO   3     m   40   99   Asia:FarE  1985  1989    CS  Prev  CB:self-rep   1    Ever       Cigs  Nev cigs
    KATO   6     f   40   99   Asia:FarE  1985  1989    CS  Prev  CB:self-rep   1    Ever       Cigs  Nev cigs
   KIRAZ   3 x   f   25   99 Eu:SE/Balkn  1999  2003    CS  Prev     CB:other   0    Ever       Cigs  Nev cigs
  KOTAN1   3 x   m   20   69    Eu:Scand  1995  2003    CS  Prev CB:diagnosed   0    Ever        Any   Nev any
  KOTAN1   6 x   f   20   69    Eu:Scand  1995  2003    CS  Prev CB:diagnosed   0    Ever        Any   Nev any
   KUBIK   3     m   15   99     Eu:East  1972  1984    CS  Prev  CB:symptoms   0    Ever        Any   N/L any
   KUBIK   4     f   15   99     Eu:East  1972  1984    CS  Prev  CB:symptoms   0    Ever        Any   N/L any
  LAMBER   3 x   m   35   69       Eu:UK  1965  1970    CS  Prev  CB:symptoms   0    Ever       Cigs  Nev cigs
  LAMBER  35 x   f   35   69       Eu:UK  1965  1970    CS  Prev  CB:symptoms   0    Ever        Any   Nev any
  LANGHA   3 x   m   20   99    Eu:Scand  1995  2000    CS  Prev  CB:symptoms   0    Ever        Any   Nev any
  LANGHA   9 x   f   20   99    Eu:Scand  1995  2000    CS  Prev  CB:symptoms   0    Ever        Any   Nev any
  ________________________________________________________________________________________________________________________
                                            International Evidence on Smoking and COPD, Phase 3, Analysis run on 27-SEP-10

                                                   Table 2 - A - 1 - 4

            IESCOPD - Meta-analysis of ever smoking, any product (or cigarettes if all product not available)
                                                          Any CB
                                                      Least-adjusted


     REF|NRR|X|SEX|AGEL|AGEH|     REGION|BEGYR|PUBYR|STTYP|ONSET|      DISEAS|ADJ|SMOKSTA|   PRODUCT|    UNEXP|

  LAVECC   3 x   m   15   99     Eu:West  1983  1988    CS  Prev  CB:self-rep   0    Ever        Any   Nev any
  LAVECC   6 x   f   15   99     Eu:West  1983  1988    CS  Prev  CB:self-rep   0    Ever        Any   Nev any
  LEBOWI  27 x   b   15   96      Am:USA  1972  1977    CS  Prev CB:diagnosed   2    Ever       Cigs  Nev cigs
  LINDST  17 x   m   20   69    Eu:Scand     *  2001    CS  Prev  CB:symptoms   0    Ever       Cigs  Nev cigs
  LINDST  20 x   f   20   69    Eu:Scand     *  2001    CS  Prev  CB:symptoms   0    Ever       Cigs  Nev cigs
  LUNDB2   3 x   m   35   66    Eu:Scand  1986  1993    CC  Prev     CB:other   0    Ever        Any   Nev any
  LUNDB2   9 x   f   35   66    Eu:Scand  1986  1993    CC  Prev     CB:other   0    Ever        Any   Nev any
  MAGNUS   3 x   m   50   80    Eu:Scand  1993  1999    CS  Prev  CB:symptoms   0    Ever        Any   Nev any
  MANFRE   3     m   20   65   Am:Canada  1978  1989    CS  Prev  CB:symptoms   0    Ever        Any   Nev any
  MANFRE   6     f   20   65   Am:Canada  1978  1989    CS  Prev  CB:symptoms   0    Ever        Any   Nev any
  MELLST   3     m   70   70    Eu:Scand  1971  1982    CS  Prev     CB:other   0    Ever        Any   Nev any
  MENEZ1   3 x   b   40   99 Am:Sth/Cent  1990  1995    CS  Prev  CB:symptoms   0    Ever       Cigs  Nev cigs
   MEREN   6     b   15   64     Eu:East  1995  2001    CS  Prev  CB:symptoms   4    Ever       Cigs  Nev cigs
  MILLER   3     m   15   99      Am:USA  1978  1988    CS  Prev CB:diagnosed   1    Ever       Cigs  Nev cigs
  MILLER   6     f   15   99      Am:USA  1978  1988    CS  Prev CB:diagnosed   1    Ever       Cigs  Nev cigs
   MILNE   3     m   62   90       Eu:UK  1968  1972    CS  Prev  CB:symptoms   0    Ever       Cigs  Nev cigs
   MILNE  15     f   62   90       Eu:UK  1968  1972    CS  Prev  CB:symptoms   0    Ever        Any   Nev any
  MOLLER   3     b   40   72     Eu:West     *  2001    CC  Prev  CB:symptoms   0    Ever       Cigs  Nev cigs
  MUELLE  33 x   m   20   69      Am:USA  1967  1971    CS  Prev  CB:symptoms   0    Ever        Any   Nev any
  MUELLE  49 x   f   20   69      Am:USA  1967  1971    CS  Prev  CB:symptoms   0    Ever        Any   Nev any
  NEJJAR   1 x   m   65   99     Eu:West  1991  1996    CS  Prev  CB:symptoms   0    Ever       Cigs  Nev cigs
  NEJJAR   2 x   f   65   99     Eu:West  1991  1996    CS  Prev  CB:symptoms   0    Ever       Cigs  Nev cigs
  OGILVI   3     m   30   99       Eu:UK  1955  1957    CC  Prev  CB:symptoms   0    Ever        Any   Nev any
  OGILVI   8     f   30   99       Eu:UK  1955  1957    CC  Prev  CB:symptoms   0    Ever        Any   Nev any
  OSWAL2   1 x   m   15   65       Eu:UK  1954  1955    CS  Prev  CB:symptoms   0    Ever       Cigs  N/L cigs
  OSWAL2   3 x   f   15   65       Eu:UK  1954  1955    CS  Prev  CB:symptoms   0    Ever       Cigs  N/L cigs
  PANDEY   3 x   m   20   99 Asia:MidE/S  1979  1984    CS  Prev  CB:symptoms   0    Ever        Any   Nev any
  PANDEY   9 x   f   20   99 Asia:MidE/S  1979  1984    CS  Prev  CB:symptoms   0    Ever        Any   Nev any
  PELKON   6     m   40   75    Eu:Scand  1959  2006    Pr   Inc     CB:other   1    Ever       Cigs  Nev cigs
  PEREZP   5     f   40   99 Am:Sth/Cent  1992  1996    CC  Prev  CB:symptoms   0    Ever       Cigs  Nev cigs
    REID   3 x   m   35   74      Am:USA  1962  1966    CS  Prev  CB:symptoms   0    Ever       Cigs  Nev cigs
    REID  19 x   f   35   74      Am:USA  1962  1966    CS  Prev  CB:symptoms   0    Ever       Cigs  Nev cigs
  SAWICK   5     m   19   70     Eu:East  1968  1972    CS  Prev  CB:symptoms   0    Ever        Any   Nev any
  SAWICK  18     f   19   70     Eu:East  1968  1972    CS  Prev  CB:symptoms   0    Ever        Any   Nev any
  SHIMUR   3     b   31   80   Asia:FarE     *  1996    CC  Prev     CB:other   0    Ever        Any   Nev any
   SILVA   6     b   20   99      Am:USA  1972  2004    Pr   Inc     CB:other   6    Ever       Cigs  Nev cigs
  SOBRAD   3 x   b   40   69     Eu:West  1996  2000    CS  Prev  CB:symptoms   0    Ever        Any   Nev any
  STJERN   3     b   16   72    Eu:Scand  1981  1985    CS  Prev  CB:symptoms   0    Ever        Any   Nev any
  TROISI   3     f   34   69      Am:USA  1980  1995    Pr   Inc CB:diagnosed   1    Ever       Cigs  Nev cigs
  VIEGI1   3     m   20   64     Eu:West  1980  1988    CS  Prev CB:diagnosed   0    Ever       Cigs   Nev any
  VIEGI1   6     f   20   64     Eu:West  1980  1988    CS  Prev CB:diagnosed   0    Ever       Cigs   Nev any
  WAGEN2   3 x   b   21   68     Eu:West  2001  2004    CC  Prev  CB:symptoms   0    Ever       Cigs  Nev cigs
  WILHEL   3     m   54   54    Eu:Scand  1967  1969    CS  Prev  CB:symptoms   0    Ever        Any   Nev any
   WOODS   3     b   20   44      Aus/NZ     *  2000    CS  Prev  CB:symptoms   5    Ever       Cigs  Nev cigs
   WOOLF   3     f   25   54   Am:Canada     *  1974    CS  Prev  CB:symptoms   0    Ever       Cigs  Nev cigs
  YAMAGU   9     b   40   99   Asia:FarE  1986  1988    CS  Prev  CB:symptoms   6    Ever       Cigs  Nev cigs
    ZOIA   3     b   18   69     Eu:West     *  1995    CS  Prev  CB:symptoms   4    Ever        Any   Nev any


  ________________________________________________________________________________________________________________________
                                            International Evidence on Smoking and COPD, Phase 3, Analysis run on 27-SEP-10

                                                   Table 2 - A - 1 - 5

            IESCOPD - Meta-analysis of ever smoking, any product (or cigarettes if all product not available)
                                                          Any CB
                                                      Least-adjusted


                        Number Exposed  Non-exposed
 REF    NRR SEX ADJ     Case    Cont    Case    Cont      RR        95.00%CI
 ALDERS 1   m   0        510     469      25      63      2.74 (  1.70-   4.43)
 ALDERS 2   f   0        333     239     105     203      2.69 (  2.02-   3.59)
 Subtotal ALDERS                                          2.71 (  2.11-   3.46)
 ANDER1 32  m   1         50       -       4       -      4.51 (  1.53-  13.29)
 ANDER1 35  f   1         19       -      15       -      2.20 (  1.08-   4.51)
 Subtotal ANDER1                                          2.74 (  1.51-   4.97)
 BECK1  3   m   0         17     243       3     189      4.41 (  1.27-  15.26)
 BECK1  6   f   0         10     198       6     318      2.68 (  0.96-   7.48)
 Subtotal BECK1                                           3.28 (  1.49-   7.24)
*BECK2  3   m   0          5     243       2     189      1.94 (  0.38-   9.91)
*BECK2  6   f   0          4     199       3     318      2.13 (  0.48-   9.42)
 Subtotal BECK2                                           2.04 (  0.68-   6.13)
*BEST   3   m   1         77       -       3       -     11.44 (  3.61-  36.25)
 BJORNS 3   b   0        836    3956     354    4179      2.49 (  2.19-   2.85)
 BROWN  1   m   0        238     725      15      76      1.66 (  0.94-   2.95)
 CERVER 3   b   3       1465       -     754       -      2.70 (  2.47-   2.97)
 COATES 3   b   0        135     913      21     515      3.63 (  2.26-   5.82)
 COLLEG 3   m   1          -       -       -       -      3.31 (  1.06-  10.33)
 COLLEG 9   f   1          -       -       -       -      2.64 (  1.54-   4.52)
 Subtotal COLLEG                                          2.75 (  1.69-   4.48)
 DEANE  5   m   0        104     307       3      66      7.45 (  2.29-  24.21)
 DEMARC 17  b   0        341    7197      91    5624      2.93 (  2.32-   3.70)
*DOLL1  13  m   1         95       -       3       -      7.40 (  2.22-  24.69)
*DONTA2 3   m   0         63     333       8     127      3.00 (  1.48-   6.09)
 DOPICO 3   m   0         37     139       6      57      2.53 (  1.01-   6.32)
 EHRLIC 3   m   0        100    2962      33    2672      2.73 (  1.84-   4.07)
 EHRLIC 9   f   0         88    1708     138    6208      2.32 (  1.76-   3.04)
 Subtotal EHRLIC                                          2.44 (  1.95-   3.06)
 ENRIGH 3   m   0         71    1432      17     699      2.04 (  1.19-   3.49)
 ENRIGH 6   f   0        102    1155      85    1558      1.62 (  1.20-   2.18)
 Subtotal ENRIGH                                          1.71 (  1.32-   2.22)
 FERRI1 61  m   1        156       -      10       -      3.22 (  1.62-   6.42)
 FERRI1 82  f   1         44       -      35       -      2.17 (  1.35-   3.51)
 Subtotal FERRI1                                          2.47 (  1.67-   3.65)
 FINKLE 1   m   0       3551   17761     596   10240      3.44 (  3.14-   3.76)
 FOXMAN 3   m   1        219       -      41       -      2.48 (  1.73-   3.54)
 FOXMAN 6   f   1        151       -      59       -      2.58 (  1.88-   3.53)
 Subtotal FOXMAN                                          2.54 (  2.00-   3.21)
 GOLDBE 3   m   0        348    1450     148     909      1.47 (  1.20-   1.82)
 GOLDBE 9   f   0        283    1729      61    1076      2.89 (  2.17-   3.85)
 Subtotal GOLDBE                                          1.86 (  1.57-   2.21)
 HAENSZ 3   m   0        200    9296      27    2742      2.18 (  1.46-   3.27)
 HAENSZ 17  f   0         79    3234      97   11536      2.91 (  2.15-   3.92)
 Subtotal HAENSZ                                          2.63 (  2.06-   3.34)
 HARDIE 9   m   1         37       -       1       -     10.90 (  2.06-  57.56)
 HARDIE 12  f   1         16       -      11       -      3.33 (  1.59-   6.98)
 Subtotal HARDIE                                          4.05 (  2.06-   7.96)
 HARRIS 3   m   0         12     268       6     518      3.87 (  1.43-  10.41)
 HAWTHO 10  m   0        646    8503      49    2097      3.25 (  2.42-   4.36)
 HAWTHO 17  f   0        320    3628     103    3440      2.95 (  2.35-   3.70)
 Subtotal HAWTHO                                          3.06 (  2.55-   3.66)
 HAYES  3   m   0        194    1440      12     591      6.64 (  3.67-  11.98)
 HAYES  6   f   0        163    1484      25    1386      6.09 (  3.97-   9.34)
 Subtotal HAYES                                           6.27 (  4.44-   8.87)
 HIGGI2 3   m   0         25     248       1      27      2.72 (  0.35-  20.89)
 HIGGI2 9   f   0          8      96       6     170      2.36 (  0.80-   7.01)
 Subtotal HIGGI2                                          2.44 (  0.93-   6.36)
 HIGGI3 3   m   0          6      82       0       6      1.02~(  0.05-  20.27)
 HIGGI3 9   f   0          2      26       0      64     12.17~(  0.57- 262.12)
 Subtotal HIGGI3                                          3.41 (  0.40-  28.99)
 HIGGI6 3   m   1        294       -      17       -      4.59 (  2.78-   7.59)
 HIGGI6 10  f   1         86       -      40       -      2.99 (  2.03-   4.40)
 Subtotal HIGGI6                                          3.51 (  2.58-   4.76)
 HO     11  b   3          -       -       -       -      2.32 (  1.55-   3.46)
 HOLLA2 3   m   0        142     394       5      84      6.05 (  2.41-  15.23)
 HOLLNA 3   m   0        139     254      10     101      5.53 (  2.79-  10.93)
 HOLLNA 11  f   0         72     245      14     217      4.56 (  2.50-   8.31)
 Subtotal HOLLNA                                          4.96 (  3.16-   7.78)
 HOUSE  3   m   0        273    1654      51    1327      4.29 (  3.16-   5.84)
 HOUSE  9   f   0        194    1187     103    2846      4.52 (  3.52-   5.79)
 Subtotal HOUSE                                           4.43 (  3.65-   5.37)
  ________________________________________________________________________________________________________________________
                                            International Evidence on Smoking and COPD, Phase 3, Analysis run on 27-SEP-10

                                                   Table 2 - A - 1 - 5

            IESCOPD - Meta-analysis of ever smoking, any product (or cigarettes if all product not available)
                                                          Any CB
                                                      Least-adjusted


                        Number Exposed  Non-exposed
 REF    NRR SEX ADJ     Case    Cont    Case    Cont      RR        95.00%CI
 HUCHON 3   b   0        404    7364     177    7183      2.23 (  1.86-   2.66)
 HUHTI1 39  m   0        177     354       7     115      8.21 (  3.75-  17.99)
 HUHTI1 88  f   0         16      98      32     677      3.45 (  1.83-   6.53)
 Subtotal HUHTI1                                          4.87 (  2.97-   7.99)
 HUHTI3 19  m   0        272     538      28     211      3.81 (  2.50-   5.80)
 JINDA2 1   b   0        666    8002     784   25843      2.74 (  2.47-   3.05)
 JOSHI  6   m   0         50     194       9     220      6.30 (  3.02-  13.14)
 JOUSI1 3   m   0       1741    4824     360    2417      2.42 (  2.14-   2.74)
 JOUSI1 6   f   0        278    1344     940    7540      1.66 (  1.43-   1.92)
 Subtotal JOUSI1                                          2.07 (  1.88-   2.27)
*KAHN2  32  m   1         88       -      13       -      2.75 (  1.54-   4.93)
 KATO   3   m   1        827       -     118       -      1.33 (  1.09-   1.63)
 KATO   6   f   1        260       -     679       -      1.92 (  1.65-   2.23)
 Subtotal KATO                                            1.68 (  1.49-   1.90)
 KIRAZ  3   f   0         17      24      78     225      2.04 (  1.04-   4.00)
 KOTAN1 3   m   0         81    1949      29    1160      1.66 (  1.08-   2.56)
 KOTAN1 6   f   0         56    1416      43    1899      1.75 (  1.17-   2.61)
 Subtotal KOTAN1                                          1.71 (  1.27-   2.29)
 KUBIK  3   m   0       3253   16409     421    8006      3.77 (  3.39-   4.19)
 KUBIK  4   f   0        298    4760     797   25758      2.02 (  1.76-   2.32)
 Subtotal KUBIK                                           2.99 (  2.75-   3.25)
 LAMBER 3   m   0        257    3523      17     642      2.75 (  1.67-   4.53)
 LAMBER 35  f   0        118    2120      54    2390      2.46 (  1.78-   3.42)
 Subtotal LAMBER                                          2.55 (  1.94-   3.35)
 LANGHA 3   m   0        985   17046     203   10999      3.13 (  2.69-   3.65)
 LANGHA 9   f   0        635   15691     351   15771      1.82 (  1.59-   2.08)
 Subtotal LANGHA                                          2.29 (  2.08-   2.54)
 LAVECC 3   m   0       2053   18422     588   13724      2.60 (  2.37-   2.86)
 LAVECC 6   f   0        280    7357    1042   28818      1.05 (  0.92-   1.20)
 Subtotal LAVECC                                          1.93 (  1.79-   2.08)
 LEBOWI 27  b   2         37       -      26       -      1.12 (  0.68-   1.86)
 LINDST 17  m   0        444    3350     214    2839      1.76 (  1.48-   2.09)
 LINDST 20  f   0        319    2795     280    3487      1.42 (  1.20-   1.68)
 Subtotal LINDST                                          1.58 (  1.40-   1.78)
 LUNDB2 3   m   0        232      91      15      41      6.97 (  3.68-  13.20)
 LUNDB2 9   f   0        105      58      35      73      3.78 (  2.26-   6.32)
 Subtotal LUNDB2                                          4.81 (  3.22-   7.18)
 MAGNUS 3   m   0         93     695      15     372      3.32 (  1.90-   5.81)
 MANFRE 3   m   0         32      79       3      40      5.40 (  1.56-  18.72)
 MANFRE 6   f   0         48     108      22     149      3.01 (  1.72-   5.28)
 Subtotal MANFRE                                          3.32 (  1.99-   5.55)
 MELLST 3   m   0        125     559      15     196      2.92 (  1.67-   5.11)
 MENEZ1 3   b   0        105     484      29     435      3.25 (  2.11-   5.01)
 MEREN  6   b   4          -       -       -       -      1.75 (  1.39-   2.22)
 MILLER 3   m   1         97       -      10       -      5.24 (  2.70-  10.17)
 MILLER 6   f   1         35       -       2       -     20.27 (  4.89-  84.04)
 Subtotal MILLER                                          6.67 (  3.66-  12.17)
 MILNE  3   m   0         65     105       3      40      8.25 (  2.45-  27.77)
 MILNE  15  f   0         13      57      12     186      3.54 (  1.53-   8.18)
 Subtotal MILNE                                           4.65 (  2.33-   9.27)
 MOLLER 3   b   0         13       9       2      11      7.94 (  1.41-  44.80)
 MUELLE 33  m   0         47     175       2      57      7.65 (  1.80-  32.51)
 MUELLE 49  f   0         31     126       3     168     13.78 (  4.12-  46.08)
 Subtotal MUELLE                                         10.82 (  4.28-  27.34)
 NEJJAR 1   m   0        147     553      42     229      1.45 (  1.00-   2.11)
 NEJJAR 2   f   0         17     122      98    1115      1.59 (  0.92-   2.74)
 Subtotal NEJJAR                                          1.49 (  1.09-   2.03)
 OGILVI 3   m   0        261     210      11      29      3.28 (  1.60-   6.71)
 OGILVI 8   f   0        106      86      86     160      2.29 (  1.56-   3.38)
 Subtotal OGILVI                                          2.49 (  1.77-   3.49)
 OSWAL2 1   m   0        421    2196      96     889      1.78 (  1.40-   2.25)
 OSWAL2 3   f   0        149     821     116    1156      1.81 (  1.40-   2.34)
 Subtotal OSWAL2                                          1.79 (  1.50-   2.13)
 PANDEY 3   m   0        238     914       4     219     14.26 (  5.25-  38.72)
 PANDEY 9   f   0        208     718      66     459      2.01 (  1.49-   2.72)
 Subtotal PANDEY                                          2.37 (  1.78-   3.16)
*PELKON 6   m   1          -       -       -       -      1.76 (  1.32-   2.36)
 PEREZP 5   f   0         27      47      77     145      1.08 (  0.63-   1.87)
 REID   3   m   0        248   15002      16    4127      4.26 (  2.57-   7.08)
 REID   19  f   0         80    8185      37   10430      2.76 (  1.86-   4.07)
 Subtotal REID                                            3.24 (  2.38-   4.42)
  ________________________________________________________________________________________________________________________
                                            International Evidence on Smoking and COPD, Phase 3, Analysis run on 27-SEP-10

                                                   Table 2 - A - 1 - 5

            IESCOPD - Meta-analysis of ever smoking, any product (or cigarettes if all product not available)
                                                          Any CB
                                                      Least-adjusted


                        Number Exposed  Non-exposed
 REF    NRR SEX ADJ     Case    Cont    Case    Cont      RR        95.00%CI
 SAWICK 5   m   0        286    1272      14     353      5.67 (  3.27-   9.82)
 SAWICK 18  f   0         75     649      46    1660      4.17 (  2.86-   6.09)
 Subtotal SAWICK                                          4.60 (  3.37-   6.28)
 SHIMUR 3   b   0          3       2       3       2      1.00 (  0.08-  12.56)
*SILVA  6   b   6          -       -       -       -      1.76 (  0.99-   3.16)
 SOBRAD 3   b   0        172    1838      20    2005      9.38 (  5.88-  14.97)
 STJERN 3   b   0         15     555       6     873      3.93 (  1.52-  10.20)
*TROISI 3   f   1        545       -     253       -      1.93 (  1.66-   2.24)
 VIEGI1 3   m   0         32    1040       4     504      3.88 (  1.36-  11.02)
 VIEGI1 6   f   0          1     582       7    1119      0.27 (  0.03-   2.24)
 Subtotal VIEGI1                                          2.29 (  0.90-   5.84)
 WAGEN2 3   b   0        246    2762     111    2136      1.71 (  1.36-   2.16)
 WILHEL 3   m   0         18     207       1      87      7.57 (  0.99-  57.56)
 WOODS  3   b   5          -       -       -       -      2.46 (  2.12-   2.87)
 WOOLF  3   f   0         92     225      10     173      7.07 (  3.58-  13.99)
 YAMAGU 9   b   6        334       -     128       -      2.26 (  1.82-   2.81)
 ZOIA   3   b   4          -       -       -       -      1.45 (  0.66-   3.16)
Partial Totals         30734  233509   11932  252000
*prospective study                                        ~ With 0.5 adjustment for zero


 REF    NRR SEX ADJ             Ys       Ws       Qs       Ps
 ALDERS 1   m   0              1.01    16.68     0.23       0.00
 ALDERS 2   f   0              0.99    46.22     0.48       0.00
 Subtotal ALDERS               1.00    62.89     0.71
 ANDER1 32  m   1              1.51     3.29     1.25       0.01
 ANDER1 35  f   1              0.79     7.52     0.08       0.03
 Subtotal ANDER1               1.01    10.81     1.33
 BECK1  3   m   0              1.48     2.49     0.88       0.02
 BECK1  6   f   0              0.98     3.64     0.03       0.06
 Subtotal BECK1                1.19     6.13     0.91
*BECK2  3   m   0              0.66     1.45     0.07       0.42
*BECK2  6   f   0              0.76     1.74     0.03       0.32
 Subtotal BECK2                0.71     3.19     0.10
*BEST   3   m   1              2.44     2.89     6.92       0.00
 BJORNS 3   b   0              0.91   221.58     0.14       0.00
 BROWN  1   m   0              0.51    11.71     1.70       0.08
 CERVER 3   b   3              0.99   452.17     4.88       0.00
 COATES 3   b   0              1.29    17.22     2.74       0.00
 COLLEG 3   m   1              1.20     2.96     0.28       0.04
 COLLEG 9   f   1              0.97    13.25     0.09       0.00
 Subtotal COLLEG               1.01    16.22     0.37
 DEANE  5   m   0              2.01     2.77     3.47       0.00
 DEMARC 17  b   0              1.07    70.23     2.40       0.00
*DOLL1  13  m   1              2.00     2.65     3.28       0.00
*DONTA2 3   m   0              1.10     7.69     0.34       0.00
 DOPICO 3   m   0              0.93     4.58     0.01       0.05
 EHRLIC 3   m   0              1.01    24.38     0.33       0.00
 EHRLIC 9   f   0              0.84    51.66     0.12       0.00
 Subtotal EHRLIC               0.89    76.04     0.45
 ENRIGH 3   m   0              0.71    13.33     0.42       0.01
 ENRIGH 6   f   0              0.48    43.33     7.20       0.00
 Subtotal ENRIGH               0.54    56.66     7.62
 FERRI1 61  m   1              1.17     8.10     0.64       0.00
 FERRI1 82  f   1              0.77    16.83     0.22       0.00
 Subtotal FERRI1               0.90    24.93     0.86
 FINKLE 1   m   0              1.23   473.17    56.21       0.00
 FOXMAN 3   m   1              0.91    29.97     0.01       0.00
 FOXMAN 6   f   1              0.95    38.71     0.13       0.00
 Subtotal FOXMAN               0.93    68.68     0.14
 GOLDBE 3   m   0              0.39    87.57    22.01       0.00
 GOLDBE 9   f   0              1.06    46.65     1.36       0.00
 Subtotal GOLDBE               0.62   134.22    23.37
 HAENSZ 3   m   0              0.78    23.52     0.27       0.00
 HAENSZ 17  f   0              1.07    42.80     1.34       0.00
 Subtotal HAENSZ               0.97    66.33     1.62
 HARDIE 9   m   1              2.39     1.39     3.12       0.00
 HARDIE 12  f   1              1.20     7.02     0.69       0.00
 Subtotal HARDIE               1.40     8.41     3.81
 HARRIS 3   m   0              1.35     3.91     0.84       0.01
 HAWTHO 10  m   0              1.18    44.34     3.72       0.00
  ________________________________________________________________________________________________________________________
                                            International Evidence on Smoking and COPD, Phase 3, Analysis run on 27-SEP-10

                                                   Table 2 - A - 1 - 5

            IESCOPD - Meta-analysis of ever smoking, any product (or cigarettes if all product not available)
                                                          Any CB
                                                      Least-adjusted


 REF    NRR SEX ADJ             Ys       Ws       Qs       Ps
 HAWTHO 17  f   0              1.08    74.63     2.72       0.00
 Subtotal HAWTHO               1.12   118.97     6.44
 HAYES  3   m   0              1.89    11.00    11.07       0.00
 HAYES  6   f   0              1.81    21.04    17.70       0.00
 Subtotal HAYES                1.84    32.04    28.77
 HIGGI2 3   m   0              1.00     0.93     0.01       0.34
 HIGGI2 9   f   0              0.86     3.25     0.00       0.12
 Subtotal HIGGI2               0.89     4.17     0.01
 HIGGI3 3   m   0              0.02     0.43     0.32       0.99
 HIGGI3 9   f   0              2.50     0.41     1.06       0.11
 Subtotal HIGGI3               1.23     0.84     1.38
 HIGGI6 3   m   1              1.52    15.23     6.13       0.00
 HIGGI6 10  f   1              1.10    25.68     1.09       0.00
 Subtotal HIGGI6               1.25    40.91     7.22
 HO     11  b   3              0.84    23.83     0.05       0.00
 HOLLA2 3   m   0              1.80     4.51     3.75       0.00
 HOLLNA 3   m   0              1.71     8.26     5.56       0.00
 HOLLNA 11  f   0              1.52    10.64     4.18       0.00
 Subtotal HOLLNA               1.60    18.90     9.74
 HOUSE  3   m   0              1.46    40.60    13.10       0.00
 HOUSE  9   f   0              1.51    62.28    23.80       0.00
 Subtotal HOUSE                1.49   102.88    36.90
 HUCHON 3   b   0              0.80   119.05     0.94       0.00
 HUHTI1 39  m   0              2.11     6.25     9.25       0.00
 HUHTI1 88  f   0              1.24     9.48     1.16       0.00
 Subtotal HUHTI1               1.58    15.73    10.41
 HUHTI3 19  m   0              1.34    21.74     4.37       0.00
 JINDA2 1   b   0              1.01   340.06     4.89       0.00
 JOSHI  6   m   0              1.84     7.10     6.43       0.00
 JOUSI1 3   m   0              0.89   251.69     0.00       0.00
 JOUSI1 6   f   0              0.51   180.58    26.50       0.00
 Subtotal JOUSI1               0.73   432.27    26.50
*KAHN2  32  m   1              1.01    11.35     0.17       0.00
 KATO   3   m   1              0.29    94.89    34.64       0.01
 KATO   6   f   1              0.65   169.34     9.52       0.00
 Subtotal KATO                 0.52   264.24    44.15
 KIRAZ  3   f   0              0.71     8.49     0.26       0.04
 KOTAN1 3   m   0              0.51    20.75     3.01       0.02
 KOTAN1 6   f   0              0.56    23.62     2.60       0.01
 Subtotal KOTAN1               0.53    44.36     5.61
 KUBIK  3   m   0              1.33   348.61    66.79       0.00
 KUBIK  4   f   0              0.70   205.79     7.01       0.00
 Subtotal KUBIK                1.10   554.40    73.80
 LAMBER 3   m   0              1.01    15.49     0.24       0.00
 LAMBER 35  f   0              0.90    35.86     0.01       0.00
 Subtotal LAMBER               0.94    51.35     0.24
 LANGHA 3   m   0              1.14   164.18    10.42       0.00
 LANGHA 9   f   0              0.60   219.73    18.66       0.00
 Subtotal LANGHA               0.83   383.91    29.09
 LAVECC 3   m   0              0.96   431.98     1.91       0.00
 LAVECC 6   f   0              0.05   212.69   149.40       0.45
 Subtotal LAVECC               0.66   644.67   151.32
 LEBOWI 27  b   2              0.11    15.18     9.14       0.66
 LINDST 17  m   0              0.56   132.00    13.94       0.00
 LINDST 20  f   0              0.35   136.04    39.34       0.00
 Subtotal LINDST               0.46   268.04    53.28
 LUNDB2 3   m   0              1.94     9.40    10.41       0.00
 LUNDB2 9   f   0              1.33    14.49     2.79       0.00
 Subtotal LUNDB2               1.57    23.89    13.20
 MAGNUS 3   m   0              1.20    12.26     1.18       0.00
 MANFRE 3   m   0              1.69     2.49     1.58       0.01
 MANFRE 6   f   0              1.10    12.16     0.55       0.00
 Subtotal MANFRE               1.20    14.64     2.13
 MELLST 3   m   0              1.07    12.26     0.41       0.00
 MENEZ1 3   b   0              1.18    20.67     1.75       0.00
 MEREN  6   b   4              0.56    70.09     7.62       0.00
 MILLER 3   m   1              1.66     8.74     5.14       0.00
 MILLER 6   f   1              3.01     1.90     8.54       0.00
 Subtotal MILLER               1.90    10.64    13.67
 MILNE  3   m   0              2.11     2.61     3.89       0.00
 MILNE  15  f   0              1.26     5.46     0.76       0.00
  ________________________________________________________________________________________________________________________
                                            International Evidence on Smoking and COPD, Phase 3, Analysis run on 27-SEP-10

                                                   Table 2 - A - 1 - 5

            IESCOPD - Meta-analysis of ever smoking, any product (or cigarettes if all product not available)
                                                          Any CB
                                                      Least-adjusted


 REF    NRR SEX ADJ             Ys       Ws       Qs       Ps
 Subtotal MILNE                1.54     8.07     4.65
 MOLLER 3   b   0              2.07     1.28     1.80       0.02
 MUELLE 33  m   0              2.04     1.84     2.41       0.01
 MUELLE 49  f   0              2.62     2.64     7.92       0.00
 Subtotal MUELLE               2.38     4.47    10.33
 NEJJAR 1   m   0              0.37    27.18     7.30       0.05
 NEJJAR 2   f   0              0.46    12.80     2.35       0.10
 Subtotal NEJJAR               0.40    39.98     9.65
 OGILVI 3   m   0              1.19     7.46     0.66       0.00
 OGILVI 8   f   0              0.83    25.68     0.09       0.00
 Subtotal OGILVI               0.91    33.14     0.75
 OSWAL2 1   m   0              0.57    69.58     6.92       0.00
 OSWAL2 3   f   0              0.59    57.42     5.06       0.00
 Subtotal OSWAL2               0.58   127.00    11.98
 PANDEY 3   m   0              2.66     3.85    12.03       0.00
 PANDEY 9   f   0              0.70    42.50     1.52       0.00
 Subtotal PANDEY               0.86    46.35    13.54
*PELKON 6   m   1              0.57    45.52     4.78       0.00
 PEREZP 5   f   0              0.08    12.79     8.41       0.78
 REID   3   m   0              1.45    14.96     4.71       0.00
 REID   19  f   0              1.01    25.16     0.39       0.00
 Subtotal REID                 1.18    40.12     5.09
 SAWICK 5   m   0              1.74    12.73     9.11       0.00
 SAWICK 18  f   0              1.43    26.87     7.80       0.00
 Subtotal SAWICK               1.53    39.60    16.90
 SHIMUR 3   b   0              0.00     0.60     0.47       1.00
*SILVA  6   b   6              0.57    11.41     1.20       0.06
 SOBRAD 3   b   0              2.24    17.59    32.02       0.00
 STJERN 3   b   0              1.37     4.23     0.97       0.00
*TROISI 3   f   1              0.66   171.12     9.20       0.00
 VIEGI1 3   m   0              1.36     3.52     0.76       0.01
 VIEGI1 6   f   0             -1.29     0.87     4.15       0.23
 Subtotal VIEGI1               0.83     4.39     4.92
 WAGEN2 3   b   0              0.54    71.92     8.84       0.00
 WILHEL 3   m   0              2.02     0.93     1.20       0.05
 WOODS  3   b   5              0.90   167.48     0.02       0.00
 WOOLF  3   f   0              1.96     8.26     9.40       0.00
 YAMAGU 9   b   6              0.82    81.45     0.45       0.00
 ZOIA   3   b   4              0.37     6.27     1.68       0.35

                       N      119
                      NS       79


                      Wt  6442.50
                 Het Chi   837.31
                 Het  df      118
                 Het  P       ***
               Fixed  RR     2.43
                     RRl     2.37
                     RRu     2.49
                      P       +++
              Random  RR     2.73
                     RRl     2.52
                     RRu     2.95
                      P       +++
               Asymm  P         *


  ________________________________________________________________________________________________________________________
                                            International Evidence on Smoking and COPD, Phase 3, Analysis run on 27-SEP-10

                                                   Table 2 - A - 1 - 6

            IESCOPD - Meta-analysis of ever smoking, any product (or cigarettes if all product not available)
                                                          Any CB
                                                      Least-adjusted


                       N      119
                      NS       79


                      Wt  6442.50
                 Het Chi   837.31
                 Het  df      118
                 Het  P       ***
               Fixed  RR     2.43
                     RRl     2.37
                     RRu     2.49
                      P       +++
              Random  RR     2.73
                     RRl     2.52
                     RRu     2.95
                      P       +++
               Asymm  P         *

                                   Sex
                             both      male    female     Total


                       N       19        56        44       119
                      NS       19        56        44       119


                      Wt  1712.31   2591.16   2139.04   6442.50
                 Het Chi    79.70    320.70    303.74    837.31
                 Het  df       18        55        43       118
                 Het  P       ***       ***       ***       ***
               Fixed  RR     2.52      2.79      2.00      2.43
                     RRl     2.41      2.69      1.92      2.37
                     RRu     2.65      2.90      2.09      2.49
                      P       +++       +++       +++       +++
              Random  RR     2.48      3.13      2.47      2.73
                     RRl     2.19      2.77      2.17      2.52
                     RRu     2.81      3.53      2.81      2.95
                      P       +++       +++       +++       +++
             Between Chi                                 133.17
             Between  df                                      2
             Between  P                                     ***
             Btwn(F)  P                                     ***

                                        Continent
                            NAmer    Europe      Asia  oth/mult     Total


                       N       39        64         9         7       119
                      NS       25        41         7         6        79


                      Wt  1272.78   4054.97    763.62    351.13   6442.50
                 Het Chi   200.20    504.75     65.44     13.42    837.31
                 Het  df       38        63         8         6       118
                 Het  P       ***       ***       ***         *       ***
               Fixed  RR     2.91      2.33      2.25      2.52      2.43
                     RRl     2.75      2.26      2.10      2.27      2.37
                     RRu     3.07      2.40      2.42      2.80      2.49
                      P       +++       +++       +++       +++       +++
              Random  RR     3.24      2.58      2.41      2.50      2.73
                     RRl     2.77      2.33      1.86      2.07      2.52
                     RRu     3.80      2.87      3.12      3.01      2.95
                      P       +++       +++       +++       +++       +++
             Between Chi                                            53.51
             Between  df                                                3
             Between  P                                               ***
             Btwn(F)  P                                               (*)


  ________________________________________________________________________________________________________________________
                                            International Evidence on Smoking and COPD, Phase 3, Analysis run on 27-SEP-10

                                                   Table 2 - A - 1 - 6

            IESCOPD - Meta-analysis of ever smoking, any product (or cigarettes if all product not available)
                                                          Any CB
                                                      Least-adjusted
                               Study type
                               CC        Pr        CS     Total


                       N       10        11        98       119
                      NS        7         9        63        79


                      Wt   206.52    374.78   5861.20   6442.50
                 Het Chi    33.60     31.23    770.52    837.31
                 Het  df        9        10        97       118
                 Het  P       ***       ***       ***       ***
               Fixed  RR     2.31      2.30      2.45      2.43
                     RRl     2.01      2.08      2.39      2.37
                     RRu     2.64      2.55      2.51      2.49
                      P       +++       +++       +++       +++
              Random  RR     2.63      2.60      2.75      2.73
                     RRl     1.93      2.04      2.52      2.52
                     RRu     3.58      3.30      3.00      2.95
                      P       +++       +++       +++       +++
             Between Chi                                   1.95
             Between  df                                      2
             Between  P                                    N.S.
             Btwn(F)  P                                    N.S.

                           CB subtype
                             mort     sympt     other     Total


                       N        3        86        30       119
                      NS        3        56        20        79


                      Wt    16.89   4981.45   1444.16   6442.50
                 Het Chi     5.77    534.98    203.47    837.31
                 Het  df        2        85        29       118
                 Het  P       (*)       ***       ***       ***
               Fixed  RR     4.10      2.59      1.95      2.43
                     RRl     2.54      2.52      1.86      2.37
                     RRu     6.60      2.66      2.06      2.49
                      P       +++       +++       +++       +++
              Random  RR     5.49      2.86      2.24      2.73
                     RRl     2.14      2.63      1.89      2.52
                     RRu    14.11      3.12      2.65      2.95
                      P       +++       +++       +++       +++
             Between Chi                                  93.09
             Between  df                                      2
             Between  P                                     ***
             Btwn(F)  P                                      **

                             Smoking product
                              any      cigs  cigsonly     Total


                       N       63        54         2       119
                      NS       42        38         2        82


                      Wt  3773.79   2619.61     49.10   6442.50
                 Het Chi   487.05    307.53      5.68    837.31
                 Het  df       62        53         1       118
                 Het  P       ***       ***         *       ***
               Fixed  RR     2.59      2.22      2.93      2.43
                     RRl     2.50      2.14      2.22      2.37
                     RRu     2.67      2.31      3.88      2.49
                      P       +++       +++       +++       +++
              Random  RR     3.00      2.43      4.96      2.73
                     RRl     2.69      2.18      1.22      2.52
                     RRu     3.36      2.71     20.12      2.95
                      P       +++       +++         +       +++
             Between Chi                                  37.05
             Between  df                                      2
             Between  P                                     ***
             Btwn(F)  P                                     (*)
  ________________________________________________________________________________________________________________________
                                            International Evidence on Smoking and COPD, Phase 3, Analysis run on 27-SEP-10

                                                   Table 2 - A - 1 - 6

            IESCOPD - Meta-analysis of ever smoking, any product (or cigarettes if all product not available)
                                                          Any CB
                                                      Least-adjusted
                                     Unexposed group
                          nev any   nev cig  nev+ any  nev+ cig     Total


                       N       70        43         2         4       119
                      NS       46        32         1         2        81


                      Wt  3352.44   2289.69    554.40    245.97   6442.50
                 Het Chi   437.20    277.22     50.12     17.85    837.31
                 Het  df       69        42         1         3       118
                 Het  P       ***       ***       ***       ***       ***
               Fixed  RR     2.53      2.19      2.99      2.32      2.43
                     RRl     2.45      2.10      2.75      2.05      2.37
                     RRu     2.62      2.28      3.25      2.63      2.49
                      P       +++       +++       +++       +++       +++
              Random  RR     3.01      2.43      2.77      2.35      2.73
                     RRl     2.70      2.15      1.50      1.73      2.52
                     RRu     3.36      2.75      5.09      3.19      2.95
                      P       +++       +++        ++       +++       +++
             Between Chi                                            54.92
             Between  df                                                3
             Between  P                                               ***
             Btwn(F)  P                                                 *

                        Unexposed group (combining nev+ with main levels)
                          nev any   nev cig     Total


                       N       72        47       119
                      NS       47        34        81


                      Wt  3906.83   2535.67   6442.50
                 Het Chi   500.41    295.78    837.31
                 Het  df       71        46       118
                 Het  P       ***       ***       ***
               Fixed  RR     2.60      2.20      2.43
                     RRl     2.52      2.12      2.37
                     RRu     2.68      2.29      2.49
                      P       +++       +++       +++
              Random  RR     3.00      2.41      2.73
                     RRl     2.70      2.15      2.52
                     RRu     3.33      2.70      2.95
                      P       +++       +++       +++
             Between Chi                        41.12
             Between  df                            1
             Between  P                           ***
             Btwn(F)  P                             *


  ________________________________________________________________________________________________________________________
                                            International Evidence on Smoking and COPD, Phase 3, Analysis run on 27-SEP-10

                                                   Table 2 - A - 1 - 7

            IESCOPD - Meta-analysis of ever smoking, any product (or cigarettes if all product not available)
                                                          Any CB
                                 Excluded studies (and stage at which they were excluded)


1       CLARK COTTON  MEYER REMYJA RUTGER SNYDER SOBRAX     SU TAKEMU  WANG4   WEIR WHICKE ZALACA
2      ALESSA  AMIGO ANDER2 ANDER3 AUERBA BEDNAR BROGGE  CHEN1  CHEN2  CHEN3  CHENG CLEMEN  COCCI  DEAN1 DEJONG DETORR
       DICKIN DONTA1 EKBERG ENSTRO FERRI2 FERRI3  FIDAN FORAST FUKUCH GEIJER GODTFR GULSVI HAMMO2 HARIKK HEDMAN HIGGI4
       HOZAWA ITABAS JACOBS JAENDI JOHANN KACHEL KARAKA KATANC KHOURY    KIM KLAYTO KOJIMA KOTAN2 KRZYZA KULLER    LAI
         LAM1   LAM2   LAM3  LANGE    LEE   LIAW LINDBE   LIU1   LIU2 LUNDB1  MADOR MANNI1 MANNI2 MANNI3 MARAN1 MARAN2
       MARCUS MATHES MENEZ2 MENEZ3 MENEZ4 MENEZ5 MENEZ6 MONTNE   NAWA NIEPSU NIHLEN NILSSO  OMORI   PEAT   PETO  PRATT
        PRICE RENWIC RICCIO  RYDER SARGEA SHAHAB   SHIN SICHLE SPEIZE STERLI  STROM SUTINE  TAGER   TANG   THUN   TODD
       TRUPIN TSUSHI TVERDA VESTBO VIEGI2 VIKGRE VINEIS VOLLM1 VOLLM2 VONHER   WALD  WANG2 WATSON  WEISS WILSO1   XIAO
           XU   YUAN ZIELI1 ZIELI2 ZIETKO
3       DOLL2 FLETCH HRUBEC JENSEN OSWAL1 RIMING SCHWAR  SHARP SUADIC    WIG WILSO2
4        BANG  DEAN2 HIRAYA   KAHN LANGE2 TAGER2    WEN WOJTYN
8      CHAPMA HUHTI2 URRUTI


  ________________________________________________________________________________________________________________________
                                            International Evidence on Smoking and COPD, Phase 3, Analysis run on 27-SEP-10

                                                   Table 2 - A - 1 - 8

            IESCOPD - Meta-analysis of ever smoking, any product (or cigarettes if all product not available)
                                                          Any CB
                                             Potentially overlapping studies


     REF| REFGP|PRINC|                     OVERLAP|

  DEMARC DEMARC     1         DEMARC/URRUTI/DEMEER
  ENRIGH ENRIGH     1         ENRIGH/HOZAWA/HARIKK
  DONTA2 JACOBS     2  JACOBS/DONTA1/DONTA2/PELKON
  PELKON JACOBS     2  JACOBS/DONTA1/DONTA2/PELKON
  LUNDB2 LUNDBA     2  LINDBE/LUNDB1/LUNDB2/HEDLUN
  OSWAL2 OSWAL2     1                OSWAL1/OSWAL2
  HIGGI2   PETO     2    PETO/HIGGI1/HIGGI2/HIGGI5
  HAWTHO   TANG     2             TANG/HAWTHO/WALD
  HUHTI1 HUHTI1     1                HUHTI1/HUHTI2
  HARDIE HARDIE     1         HARDIE/JOHANN/BROGGE
  LEBOWI LEBOWI     1                 LEBOWI/SILVA
   SILVA  SILVA     1                 LEBOWI/SILVA
  FERRI1 FERRIS     2         FERRI1/FERRI2/FERRI3
  KOTAN1 KOTAN1     1                KOTAN1/KOTAN2
  LAMBER   TODD     2                  LAMBER/TODD
   BECK1  BECK1     1                  BECK1/BECK2
   BECK2  BECK2     1                  BECK1/BECK2
   HOUSE  HOUSE     1                 HOUSE/CHAPMA
  SAWICK SAWICK     1         SAWICK/KRZYZA/WOJTYN
  HOLLNA HOLLNA     1 GODT/VEST/LANG1+2/SUAD/HOLLN
  HIGGI6 HIGGI4     2                HIGGI4/HIGGI6
   KAHN2   KAHN     2                   KAHN/KAHN2


  ________________________________________________________________________________________________________________________
                                            International Evidence on Smoking and COPD, Phase 3, Analysis run on 27-SEP-10

                                                    Table 2 - A - 2 -

            IESCOPD - Meta-analysis of ever smoking, cigarettes (or all products if cigarettes not available)
                                                          Any CB


This analysis is restricted to results for:
1) Eligible study on database
2) Outcome CB
3) Non-dose-response data
4) Ever smoking
5) Results complete enough for use in meta-analysis

Within each study, results are then selected (in the following order of preference, within each sex) for:
6) UNEXP   : never cigarettes, never any, other
7) PROD    : cigarettes, cigarettes only, any product
8) For overlapping studies: principal rather than subsidiary studies
and then for single sex results (m, f) in preference to results for both sexes combined (b).

Results adjusted for the most potential confounders are then chosen in Sections -1 to -3
(and those which actually differ from the adjusted results in Table 2 - A - 1 - 1 are marked 'x' in Section -1)
and results adjusted for the least confounders in Sections -4 to -6. (Those least adjusted results which
actually differ from the most adjusted are marked 'x' in column X in Section -4)

Section -7 shows excluded studies, together with the stage (as above) at which no qualifying
results were found.

Section -8 lists the potentially overlapping studies which have been included (1=principal, 2=subsidiary),
and any results which would have been included in preference except that they had data not complete enough
for use in meta-analysis. It also lists their significance (yes/no), if known.


  ________________________________________________________________________________________________________________________
                                            International Evidence on Smoking and COPD, Phase 3, Analysis run on 27-SEP-10

                                                   Table 2 - A - 2 - 1

            IESCOPD - Meta-analysis of ever smoking, cigarettes (or all products if cigarettes not available)
                                                          Any CB
                                                      Most-adjusted


     REF|NRR|Cmp2A1|SEX|AGEL|AGEH|     REGION|BEGYR|PUBYR|STTYP|ONSET|      DISEAS|ADJ|SMOKSTA|   PRODUCT|    UNEXP|

  ALDERS   8      x   m   35   74       Eu:UK  1977  1985    CC  Prev CB:diagnosed   1    Ever       Cigs  Nev cigs
  ALDERS   6          f   35   74       Eu:UK  1977  1985    CC  Prev CB:diagnosed   1    Ever MCigs only   Nev any
  ANDER1  32          m   25   74   Am:Canada  1963  1965    CS  Prev  CB:symptoms   1    Ever        Any   Nev any
  ANDER1  35          f   25   74   Am:Canada  1963  1965    CS  Prev  CB:symptoms   1    Ever        Any   Nev any
   BECK1   3          m   11   99      Am:USA  1972  1982    CS  Prev  CB:symptoms   0    Ever       Cigs   Nev any
   BECK1   6          f   11   99      Am:USA  1972  1982    CS  Prev  CB:symptoms   0    Ever       Cigs   Nev any
   BECK2   3          m   11   99      Am:USA  1972  1982    Pr   Inc  CB:symptoms   0    Ever       Cigs   Nev any
   BECK2   6          f   11   99      Am:USA  1972  1982    Pr   Inc  CB:symptoms   0    Ever       Cigs   Nev any
    BEST   3          m   30   97   Am:Canada  1955  1967    Pr   Inc CB:mortality   1    Ever  Cigs only   Nev any
  BJORNS   6          b   20   44    Eu:Scand  1990  1994    CS  Prev  CB:symptoms   4    Ever        Any   Nev any
   BROWN   6      x   m   60   69       Eu:UK  1956  1957    CS  Prev CB:diagnosed   0    Ever       Cigs  Nev cigs
  CERVER   6          b   20   44     Eu:West  1998  2003    CS  Prev  CB:symptoms   6    Ever       Cigs  Nev cigs
  COATES   3          b   40   64      Am:USA  1962  1965    CS  Prev  CB:symptoms   0    Ever       Cigs  Nev cigs
  COLLEG   6          m   40   64       Eu:UK     *  1961    CS  Prev CB:diagnosed   2    Ever        Any   Nev any
  COLLEG  18      x   f   40   64       Eu:UK     *  1961    CS  Prev CB:diagnosed   1    Ever       Cigs  Nev cigs
   DEANE   6          m   40   59      Am:USA  1963  1965    CS  Prev  CB:symptoms   1    Ever        Any   Nev any
  DEMARC  20          b   20   44       Multi  1991  2004    CS  Prev  CB:symptoms   1    Ever       Cigs   Nev any
   DOLL1  13          m   20   99       Eu:UK  1951  1994    Pr   Inc CB:mortality   1    Ever        Any   Nev any
  DONTA2   3          m   25   84 Eu:SE/Balkn  1960  1984    Pr   Inc CB:diagnosed   0    Ever       Cigs  Nev cigs
  DOPICO   3          m   15    *      Am:USA     *  1984    CS  Prev  CB:symptoms   0    Ever       Cigs  Nev cigs
  EHRLIC  15          m   15   99      Africa  1998  2004    CS  Prev  CB:symptoms   6    Ever        Any   Nev any
  EHRLIC  21          f   15   99      Africa  1998  2004    CS  Prev  CB:symptoms   6    Ever        Any   Nev any
  ENRIGH   3          m   65   99      Am:USA  1989  1994    CS  Prev CB:diagnosed   0    Ever       Cigs  Nev cigs
  ENRIGH   6          f   65   99      Am:USA  1989  1994    CS  Prev CB:diagnosed   0    Ever       Cigs  Nev cigs
  FERRI1  61          m   25   74      Am:USA  1961  1971    CS  Prev  CB:symptoms   1    Ever        Any   Nev any
  FERRI1  91      x   f   25   74      Am:USA  1961  1971    CS  Prev  CB:symptoms   1    Ever       Cigs  Nev cigs
  FINKLE   1          m   18   25      Am:USA  1969  1974    CS  Prev  CB:symptoms   0    Ever        Any   Nev any
  FOXMAN   3          m   20   69      Am:USA     *  1986    CS  Prev  CB:symptoms   1    Ever        Any   Nev any
  FOXMAN   6          f   20   69      Am:USA     *  1986    CS  Prev  CB:symptoms   1    Ever        Any   Nev any
  GOLDBE   6          m   15   99      Am:USA  1970  1974    CS  Prev  CB:symptoms   1    Ever       Cigs  Nev cigs
  GOLDBE  12          f   15   99      Am:USA  1970  1974    CS  Prev  CB:symptoms   1    Ever       Cigs  Nev cigs
  HAENSZ  13      x   m   35   74    Eu:Scand  1964  1972    CS  Prev  CB:symptoms   1    Ever       Cigs   Nev any
  HAENSZ  41      x   f   35   74    Eu:Scand  1964  1972    CS  Prev  CB:symptoms   1    Ever       Cigs  Nev cigs
  HARDIE   9          m   70   99    Eu:Scand  1998  2005    CS  Prev CB:diagnosed   1    Ever       Cigs  Nev cigs
  HARDIE  12          f   70   99    Eu:Scand  1998  2005    CS  Prev CB:diagnosed   1    Ever       Cigs  Nev cigs
  HARRIS   3          m   15   60      Africa     *  1993    CS  Prev  CB:symptoms   0    Ever        Any   Nev any
  HAWTHO  10          m   45   76       Eu:UK  1965  1978    Pr  Prev  CB:symptoms   0    Ever       Cigs  N/L cigs
  HAWTHO  17          f   45   76       Eu:UK  1965  1978    Pr  Prev  CB:symptoms   0    Ever       Cigs  N/L cigs
   HAYES   9          b   15   99      Am:USA  1970  1974    CS  Prev  CB:symptoms   3    Ever       Cigs  Nev cigs
  HIGGI2   3          m   25   74       Eu:UK  1956  1957    CS  Prev  CB:symptoms   0    Ever       Cigs  Nev cigs
  HIGGI2  15          f   25   74       Eu:UK  1956  1957    CS  Prev  CB:symptoms   1    Ever       Cigs  Nev cigs
  HIGGI3   3          m   55   64       Eu:UK  1956  1958    CS  Prev  CB:symptoms   0    Ever        Any   Nev any
  HIGGI3   9          f   55   64       Eu:UK  1956  1958    CS  Prev  CB:symptoms   0    Ever        Any   Nev any
  HIGGI6   3          m   25   64      Am:USA  1962  1977    CS  Prev  CB:symptoms   1    Ever        Any   Nev any
  HIGGI6  19      x   f   25   64      Am:USA  1962  1977    CS  Prev  CB:symptoms   1    Ever       Cigs  Nev cigs
      HO  11          b   70   99   Asia:FarE  1991  1999    CS  Prev  CB:self-rep   3    Ever       Cigs  Nev cigs
  HOLLA2   3          m   40   59      Am:USA  1962  1965    CS  Prev  CB:symptoms   0    Ever        Any   Nev any
  HOLLNA   3          m   40   40    Eu:Scand  1976  1983    CS  Prev  CB:symptoms   0    Ever        Any   Nev any
  HOLLNA  11          f   40   40    Eu:Scand  1976  1983    CS  Prev  CB:symptoms   0    Ever        Any   Nev any
   HOUSE   6          m   15   99      Am:USA  1970  1974    CS  Prev  CB:symptoms   1    Ever       Cigs  Nev cigs
   HOUSE  12          f   15   99      Am:USA  1970  1974    CS  Prev  CB:symptoms   1    Ever       Cigs  Nev cigs
  HUCHON   6          b   25   99     Eu:West     *  2002    CS  Prev  CB:symptoms   1    Ever       Cigs  Nev cigs
  HUHTI1  42          m   40   64    Eu:Scand  1961  1965    CS  Prev  CB:symptoms   1    Ever        Any   Nev any
  HUHTI1 127      x   f   40   64    Eu:Scand  1961  1965    CS  Prev  CB:symptoms   1    Ever       Cigs  Nev cigs
  HUHTI3  22          m   25   69    Eu:Scand  1968  1978    CS  Prev  CB:symptoms   1    Ever        Any   Nev any
  JINDA2   4      x   b   35   99 Asia:MidE/S     *  2006    CS  Prev  CB:symptoms   6    Ever       Cigs   Nev any
   JOSHI   9      x   m   17   64 Asia:MidE/S     *  1975    CS  Prev  CB:symptoms   0    Ever       Cigs  Nev cigs
  JOUSI1   3          m   25   64    Eu:Scand  1972  1996    CS  Prev  CB:symptoms   0    Ever        Any   Nev any
  JOUSI1   6          f   25   64    Eu:Scand  1972  1996    CS  Prev  CB:symptoms   0    Ever        Any   Nev any
   KAHN2  33      x   m   31   84      Am:USA  1954  1966    Pr   Inc CB:mortality   1    Ever       Cigs   Nev any
    KATO   3          m   40   99   Asia:FarE  1985  1989    CS  Prev  CB:self-rep   1    Ever       Cigs  Nev cigs
    KATO   6          f   40   99   Asia:FarE  1985  1989    CS  Prev  CB:self-rep   1    Ever       Cigs  Nev cigs
   KIRAZ   4          f   25   99 Eu:SE/Balkn  1999  2003    CS  Prev     CB:other   1    Ever       Cigs  Nev cigs
  KOTAN1   9          b   20   69    Eu:Scand  1995  2003    CS  Prev CB:diagnosed   7    Ever        Any   Nev any
   KUBIK   3          m   15   99     Eu:East  1972  1984    CS  Prev  CB:symptoms   0    Ever        Any   N/L any
   KUBIK   4          f   15   99     Eu:East  1972  1984    CS  Prev  CB:symptoms   0    Ever        Any   N/L any
  LAMBER   6          m   35   69       Eu:UK  1965  1970    CS  Prev  CB:symptoms   1    Ever       Cigs  Nev cigs
  LAMBER  22      x   f   35   69       Eu:UK  1965  1970    CS  Prev  CB:symptoms   1    Ever       Cigs  Nev cigs
  LANGHA   6          m   20   99    Eu:Scand  1995  2000    CS  Prev  CB:symptoms   1    Ever        Any   Nev any
  LANGHA  12          f   20   99    Eu:Scand  1995  2000    CS  Prev  CB:symptoms   1    Ever        Any   Nev any
  LAVECC   9          b   15   99     Eu:West  1983  1988    CS  Prev  CB:self-rep   6    Ever        Any   Nev any
  LEBOWI  30          b   15   96      Am:USA  1972  1977    CS  Prev CB:diagnosed   3    Ever       Cigs  Nev cigs
  ________________________________________________________________________________________________________________________
                                            International Evidence on Smoking and COPD, Phase 3, Analysis run on 27-SEP-10

                                                   Table 2 - A - 2 - 1

            IESCOPD - Meta-analysis of ever smoking, cigarettes (or all products if cigarettes not available)
                                                          Any CB
                                                      Most-adjusted


     REF|NRR|Cmp2A1|SEX|AGEL|AGEH|     REGION|BEGYR|PUBYR|STTYP|ONSET|      DISEAS|ADJ|SMOKSTA|   PRODUCT|    UNEXP|

  LINDST  23          b   20   69    Eu:Scand     *  2001    CS  Prev  CB:symptoms   5    Ever       Cigs  Nev cigs
  LUNDB2   6          m   35   66    Eu:Scand  1986  1993    CC  Prev     CB:other   1    Ever        Any   Nev any
  LUNDB2  12          f   35   66    Eu:Scand  1986  1993    CC  Prev     CB:other   1    Ever        Any   Nev any
  MAGNUS   6          m   50   80    Eu:Scand  1993  1999    CS  Prev  CB:symptoms   1    Ever        Any   Nev any
  MANFRE   3          m   20   65   Am:Canada  1978  1989    CS  Prev  CB:symptoms   0    Ever        Any   Nev any
  MANFRE   6          f   20   65   Am:Canada  1978  1989    CS  Prev  CB:symptoms   0    Ever        Any   Nev any
  MELLST   3          m   70   70    Eu:Scand  1971  1982    CS  Prev     CB:other   0    Ever        Any   Nev any
  MENEZ1   6          b   40   99 Am:Sth/Cent  1990  1995    CS  Prev  CB:symptoms   8    Ever       Cigs  Nev cigs
   MEREN   6          b   15   64     Eu:East  1995  2001    CS  Prev  CB:symptoms   4    Ever       Cigs  Nev cigs
  MILLER   3          m   15   99      Am:USA  1978  1988    CS  Prev CB:diagnosed   1    Ever       Cigs  Nev cigs
  MILLER   6          f   15   99      Am:USA  1978  1988    CS  Prev CB:diagnosed   1    Ever       Cigs  Nev cigs
   MILNE   3          m   62   90       Eu:UK  1968  1972    CS  Prev  CB:symptoms   0    Ever       Cigs  Nev cigs
   MILNE   9      x   f   62   90       Eu:UK  1968  1972    CS  Prev  CB:symptoms   0    Ever       Cigs  Nev cigs
  MOLLER   3          b   40   72     Eu:West     *  2001    CC  Prev  CB:symptoms   0    Ever       Cigs  Nev cigs
  MUELLE  36          m   20   69      Am:USA  1967  1971    CS  Prev  CB:symptoms   1    Ever        Any   Nev any
  MUELLE  51          f   20   69      Am:USA  1967  1971    CS  Prev  CB:symptoms   1    Ever        Any   Nev any
  NEJJAR   5          b   65   99     Eu:West  1991  1996    CS  Prev  CB:symptoms   6    Ever       Cigs  Nev cigs
  OGILVI   5      x   m   30   99       Eu:UK  1955  1957    CC  Prev  CB:symptoms   0    Ever       Cigs   Nev any
  OGILVI  14      x   f   30   99       Eu:UK  1955  1957    CC  Prev  CB:symptoms   0    Ever       Cigs  Nev cigs
  OSWAL2   2          m   15   65       Eu:UK  1954  1955    CS  Prev  CB:symptoms   1    Ever       Cigs  N/L cigs
  OSWAL2   4          f   15   65       Eu:UK  1954  1955    CS  Prev  CB:symptoms   1    Ever       Cigs  N/L cigs
  PANDEY   6          m   20   99 Asia:MidE/S  1979  1984    CS  Prev  CB:symptoms   2    Ever        Any   Nev any
  PANDEY  12          f   20   99 Asia:MidE/S  1979  1984    CS  Prev  CB:symptoms   2    Ever        Any   Nev any
  PELKON   6          m   40   75    Eu:Scand  1959  2006    Pr   Inc     CB:other   1    Ever       Cigs  Nev cigs
  PEREZP   5          f   40   99 Am:Sth/Cent  1992  1996    CC  Prev  CB:symptoms   0    Ever       Cigs  Nev cigs
    REID   6          m   35   74      Am:USA  1962  1966    CS  Prev  CB:symptoms   1    Ever       Cigs  Nev cigs
    REID  22          f   35   74      Am:USA  1962  1966    CS  Prev  CB:symptoms   1    Ever       Cigs  Nev cigs
  SAWICK   5          m   19   70     Eu:East  1968  1972    CS  Prev  CB:symptoms   0    Ever        Any   Nev any
  SAWICK  18          f   19   70     Eu:East  1968  1972    CS  Prev  CB:symptoms   0    Ever        Any   Nev any
  SHIMUR   3          b   31   80   Asia:FarE     *  1996    CC  Prev     CB:other   0    Ever        Any   Nev any
   SILVA   6          b   20   99      Am:USA  1972  2004    Pr   Inc     CB:other   6    Ever       Cigs  Nev cigs
  SOBRAD   4          b   40   69     Eu:West  1996  2000    CS  Prev  CB:symptoms   3    Ever        Any   Nev any
  STJERN   3          b   16   72    Eu:Scand  1981  1985    CS  Prev  CB:symptoms   0    Ever        Any   Nev any
  TROISI   3          f   34   69      Am:USA  1980  1995    Pr   Inc CB:diagnosed   1    Ever       Cigs  Nev cigs
  VIEGI1   3          m   20   64     Eu:West  1980  1988    CS  Prev CB:diagnosed   0    Ever       Cigs   Nev any
  VIEGI1   6          f   20   64     Eu:West  1980  1988    CS  Prev CB:diagnosed   0    Ever       Cigs   Nev any
  WAGEN2  11          b   21   68     Eu:West  2001  2004    CC  Prev  CB:symptoms   1    Ever       Cigs  Nev cigs
  WILHEL   3          m   54   54    Eu:Scand  1967  1969    CS  Prev  CB:symptoms   0    Ever        Any   Nev any
   WOODS   3          b   20   44      Aus/NZ     *  2000    CS  Prev  CB:symptoms   5    Ever       Cigs  Nev cigs
   WOOLF   3          f   25   54   Am:Canada     *  1974    CS  Prev  CB:symptoms   0    Ever       Cigs  Nev cigs
  YAMAGU   9          b   40   99   Asia:FarE  1986  1988    CS  Prev  CB:symptoms   6    Ever       Cigs  Nev cigs
    ZOIA   3          b   18   69     Eu:West     *  1995    CS  Prev  CB:symptoms   4    Ever        Any   Nev any


  ________________________________________________________________________________________________________________________
                                            International Evidence on Smoking and COPD, Phase 3, Analysis run on 27-SEP-10

                                                   Table 2 - A - 2 - 2

            IESCOPD - Meta-analysis of ever smoking, cigarettes (or all products if cigarettes not available)
                                                          Any CB
                                                      Most-adjusted


                        Number Exposed  Non-exposed
 REF    NRR SEX ADJ     Case    Cont    Case    Cont      RR        95.00%CI
 ALDERS 8   m   1        502       -      33       -      2.75 (  1.80-   4.20)
 ALDERS 6   f   1        333       -     105       -      2.79 (  2.09-   3.72)
 Subtotal ALDERS                                          2.78 (  2.19-   3.52)
 ANDER1 32  m   1         50       -       4       -      4.51 (  1.53-  13.29)
 ANDER1 35  f   1         19       -      15       -      2.20 (  1.08-   4.51)
 Subtotal ANDER1                                          2.74 (  1.51-   4.97)
 BECK1  3   m   0         17     243       3     189      4.41 (  1.27-  15.26)
 BECK1  6   f   0         10     198       6     318      2.68 (  0.96-   7.48)
 Subtotal BECK1                                           3.28 (  1.49-   7.24)
*BECK2  3   m   0          5     243       2     189      1.94 (  0.38-   9.91)
*BECK2  6   f   0          4     199       3     318      2.13 (  0.48-   9.42)
 Subtotal BECK2                                           2.04 (  0.68-   6.13)
*BEST   3   m   1         77       -       3       -     11.44 (  3.61-  36.25)
 BJORNS 6   b   4        836       -     354       -      2.18 (  1.90-   2.50)
 BROWN  6   m   0        212     573      41     228      2.06 (  1.42-   2.97)
 CERVER 6   b   6       1465       -     754       -      2.52 (  2.29-   2.76)
 COATES 3   b   0        135     913      21     515      3.63 (  2.26-   5.82)
 COLLEG 6   m   2          -       -       -       -      2.88 (  0.98-   8.47)
 COLLEG 18  f   1          -       -       -       -      2.64 (  1.54-   4.52)
 Subtotal COLLEG                                          2.69 (  1.66-   4.35)
 DEANE  6   m   1        104       -       3       -      6.15 (  1.83-  20.63)
 DEMARC 20  b   1        341       -      91       -      2.96 (  2.33-   3.75)
*DOLL1  13  m   1         95       -       3       -      7.40 (  2.22-  24.69)
*DONTA2 3   m   0         63     333       8     127      3.00 (  1.48-   6.09)
 DOPICO 3   m   0         37     139       6      57      2.53 (  1.01-   6.32)
 EHRLIC 15  m   6        100       -      33       -      1.27 (  0.77-   2.10)
 EHRLIC 21  f   6         88       -     138       -      1.64 (  1.15-   2.34)
 Subtotal EHRLIC                                          1.51 (  1.13-   2.01)
 ENRIGH 3   m   0         71    1432      17     699      2.04 (  1.19-   3.49)
 ENRIGH 6   f   0        102    1155      85    1558      1.62 (  1.20-   2.18)
 Subtotal ENRIGH                                          1.71 (  1.32-   2.22)
 FERRI1 61  m   1        156       -      10       -      3.22 (  1.62-   6.42)
 FERRI1 91  f   1         44       -      35       -      2.17 (  1.35-   3.51)
 Subtotal FERRI1                                          2.47 (  1.67-   3.65)
 FINKLE 1   m   0       3551   17761     596   10240      3.44 (  3.14-   3.76)
 FOXMAN 3   m   1        219       -      41       -      2.48 (  1.73-   3.54)
 FOXMAN 6   f   1        151       -      59       -      2.58 (  1.88-   3.53)
 Subtotal FOXMAN                                          2.54 (  2.00-   3.21)
 GOLDBE 6   m   1        348       -     148       -      1.48 (  1.20-   1.83)
 GOLDBE 12  f   1        283       -      61       -      2.87 (  2.15-   3.83)
 Subtotal GOLDBE                                          1.86 (  1.57-   2.21)
 HAENSZ 13  m   1        149       -      27       -      2.52 (  1.66-   3.85)
 HAENSZ 41  f   1         79       -      97       -      3.32 (  2.43-   4.53)
 Subtotal HAENSZ                                          3.01 (  2.34-   3.87)
 HARDIE 9   m   1         37       -       1       -     10.90 (  2.06-  57.56)
 HARDIE 12  f   1         16       -      11       -      3.33 (  1.59-   6.98)
 Subtotal HARDIE                                          4.05 (  2.06-   7.96)
 HARRIS 3   m   0         12     268       6     518      3.87 (  1.43-  10.41)
 HAWTHO 10  m   0        646    8503      49    2097      3.25 (  2.42-   4.36)
 HAWTHO 17  f   0        320    3628     103    3440      2.95 (  2.35-   3.70)
 Subtotal HAWTHO                                          3.06 (  2.55-   3.66)
 HAYES  9   b   3        357       -      37       -      5.70 (  4.08-   7.97)
 HIGGI2 3   m   0         25     248       1      27      2.72 (  0.35-  20.89)
 HIGGI2 15  f   1          8       -       6       -      2.46 (  0.77-   7.83)
 Subtotal HIGGI2                                          2.52 (  0.92-   6.91)
 HIGGI3 3   m   0          6      82       0       6      1.02~(  0.05-  20.27)
 HIGGI3 9   f   0          2      26       0      64     12.17~(  0.57- 262.12)
 Subtotal HIGGI3                                          3.41 (  0.40-  28.99)
 HIGGI6 3   m   1        294       -      17       -      4.59 (  2.78-   7.59)
 HIGGI6 19  f   1         86       -      40       -      2.99 (  2.03-   4.40)
 Subtotal HIGGI6                                          3.51 (  2.58-   4.76)
 HO     11  b   3          -       -       -       -      2.32 (  1.55-   3.46)
 HOLLA2 3   m   0        142     394       5      84      6.05 (  2.41-  15.23)
 HOLLNA 3   m   0        139     254      10     101      5.53 (  2.79-  10.93)
 HOLLNA 11  f   0         72     245      14     217      4.56 (  2.50-   8.31)
 Subtotal HOLLNA                                          4.96 (  3.16-   7.78)
 HOUSE  6   m   1        272       -      51       -      4.21 (  3.08-   5.75)
 HOUSE  12  f   1        194       -     103       -      4.47 (  3.48-   5.75)
 Subtotal HOUSE                                           4.37 (  3.59-   5.31)
 HUCHON 6   b   1        404       -     177       -      2.21 (  1.83-   2.66)
 HUHTI1 42  m   1        177       -       7       -      7.05 (  3.17-  15.67)
  ________________________________________________________________________________________________________________________
                                            International Evidence on Smoking and COPD, Phase 3, Analysis run on 27-SEP-10

                                                   Table 2 - A - 2 - 2

            IESCOPD - Meta-analysis of ever smoking, cigarettes (or all products if cigarettes not available)
                                                          Any CB
                                                      Most-adjusted


                        Number Exposed  Non-exposed
 REF    NRR SEX ADJ     Case    Cont    Case    Cont      RR        95.00%CI
 HUHTI1 127 f   1         16       -      32       -      3.63 (  1.78-   7.40)
 Subtotal HUHTI1                                          4.87 (  2.86-   8.29)
 HUHTI3 22  m   1        272       -      28       -      2.91 (  1.87-   4.51)
 JINDA2 4   b   6        127       -     784       -      1.95 (  1.58-   2.42)
 JOSHI  9   m   0         50     194       9     220      6.30 (  3.02-  13.14)
 JOUSI1 3   m   0       1741    4824     360    2417      2.42 (  2.14-   2.74)
 JOUSI1 6   f   0        278    1344     940    7540      1.66 (  1.43-   1.92)
 Subtotal JOUSI1                                          2.07 (  1.88-   2.27)
*KAHN2  33  m   1         85       -      13       -      3.56 (  1.98-   6.41)
 KATO   3   m   1        827       -     118       -      1.33 (  1.09-   1.63)
 KATO   6   f   1        260       -     679       -      1.92 (  1.65-   2.23)
 Subtotal KATO                                            1.68 (  1.49-   1.90)
 KIRAZ  4   f   1         17       -      78       -      4.00 (  1.76-   9.08)
 KOTAN1 9   b   7        137       -      72       -      1.75 (  1.28-   2.39)
 KUBIK  3   m   0       3253   16409     421    8006      3.77 (  3.39-   4.19)
 KUBIK  4   f   0        298    4760     797   25758      2.02 (  1.76-   2.32)
 Subtotal KUBIK                                           2.99 (  2.75-   3.25)
 LAMBER 6   m   1        257       -      17       -      2.60 (  1.58-   4.30)
 LAMBER 22  f   1        118       -      54       -      2.88 (  2.04-   4.06)
 Subtotal LAMBER                                          2.79 (  2.10-   3.70)
 LANGHA 6   m   1        985       -     203       -      2.66 (  2.28-   3.12)
 LANGHA 12  f   1        635       -     351       -      1.95 (  1.70-   2.24)
 Subtotal LANGHA                                          2.23 (  2.01-   2.48)
 LAVECC 9   b   6       2333       -    1630       -      2.27 (  2.10-   2.46)
 LEBOWI 30  b   3         37       -      26       -      1.13 (  0.68-   1.87)
 LINDST 23  b   5        763       -     494       -      1.52 (  1.34-   1.72)
 LUNDB2 6   m   1        232       -      15       -      7.84 (  4.14-  14.83)
 LUNDB2 12  f   1        106       -      35       -      4.14 (  2.30-   7.44)
 Subtotal LUNDB2                                          5.55 (  3.60-   8.55)
 MAGNUS 6   m   1         93       -      15       -      4.18 (  2.36-   7.38)
 MANFRE 3   m   0         32      79       3      40      5.40 (  1.56-  18.72)
 MANFRE 6   f   0         48     108      22     149      3.01 (  1.72-   5.28)
 Subtotal MANFRE                                          3.32 (  1.99-   5.55)
 MELLST 3   m   0        125     559      15     196      2.92 (  1.67-   5.11)
 MENEZ1 6   b   8        105       -      29       -      2.93 (  1.79-   4.80)
 MEREN  6   b   4          -       -       -       -      1.75 (  1.39-   2.22)
 MILLER 3   m   1         97       -      10       -      5.24 (  2.70-  10.17)
 MILLER 6   f   1         35       -       2       -     20.27 (  4.89-  84.04)
 Subtotal MILLER                                          6.67 (  3.66-  12.17)
 MILNE  3   m   0         65     105       3      40      8.25 (  2.45-  27.77)
 MILNE  9   f   0         13      57      12     186      3.54 (  1.53-   8.18)
 Subtotal MILNE                                           4.65 (  2.33-   9.27)
 MOLLER 3   b   0         13       9       2      11      7.94 (  1.41-  44.80)
 MUELLE 36  m   1         47       -       2       -      7.08 (  1.66-  30.19)
 MUELLE 51  f   1         31       -       3       -     10.49 (  2.74-  40.22)
 Subtotal MUELLE                                          8.75 (  3.27-  23.44)
 NEJJAR 5   b   6        164       -     140       -      1.56 (  1.15-   2.11)
 OGILVI 5   m   0        227     163      11      29      3.67 (  1.78-   7.56)
 OGILVI 14  f   0        106      86      86     160      2.29 (  1.56-   3.38)
 Subtotal OGILVI                                          2.55 (  1.81-   3.58)
 OSWAL2 2   m   1        421       -      96       -      1.41 (  1.11-   1.79)
 OSWAL2 4   f   1        149       -     116       -      1.69 (  1.30-   2.20)
 Subtotal OSWAL2                                          1.53 (  1.28-   1.83)
 PANDEY 6   m   2        238       -       4       -      5.37 (  2.52-  11.45)
 PANDEY 12  f   2        208       -      66       -      1.76 (  1.30-   2.38)
 Subtotal PANDEY                                          2.05 (  1.55-   2.72)
*PELKON 6   m   1          -       -       -       -      1.76 (  1.32-   2.36)
 PEREZP 5   f   0         27      47      77     145      1.08 (  0.63-   1.87)
 REID   6   m   1        248       -      16       -      4.61 (  2.77-   7.70)
 REID   22  f   1         80       -      37       -      3.32 (  2.17-   5.07)
 Subtotal REID                                            3.80 (  2.74-   5.26)
 SAWICK 5   m   0        286    1272      14     353      5.67 (  3.27-   9.82)
 SAWICK 18  f   0         75     649      46    1660      4.17 (  2.86-   6.09)
 Subtotal SAWICK                                          4.60 (  3.37-   6.28)
 SHIMUR 3   b   0          3       2       3       2      1.00 (  0.08-  12.56)
*SILVA  6   b   6          -       -       -       -      1.76 (  0.99-   3.16)
 SOBRAD 4   b   3        167       -      20       -      6.68 (  3.83-  11.63)
 STJERN 3   b   0         15     555       6     873      3.93 (  1.52-  10.20)
*TROISI 3   f   1        545       -     253       -      1.93 (  1.66-   2.24)
 VIEGI1 3   m   0         32    1040       4     504      3.88 (  1.36-  11.02)
 VIEGI1 6   f   0          1     582       7    1119      0.27 (  0.03-   2.24)
  ________________________________________________________________________________________________________________________
                                            International Evidence on Smoking and COPD, Phase 3, Analysis run on 27-SEP-10

                                                   Table 2 - A - 2 - 2

            IESCOPD - Meta-analysis of ever smoking, cigarettes (or all products if cigarettes not available)
                                                          Any CB
                                                      Most-adjusted


                        Number Exposed  Non-exposed
 REF    NRR SEX ADJ     Case    Cont    Case    Cont      RR        95.00%CI
 Subtotal VIEGI1                                          2.29 (  0.90-   5.84)
 WAGEN2 11  b   1        246       -     111       -      1.72 (  1.36-   2.17)
 WILHEL 3   m   0         18     207       1      87      7.57 (  0.99-  57.56)
 WOODS  3   b   5          -       -       -       -      2.46 (  2.12-   2.87)
 WOOLF  3   f   0         92     225      10     173      7.07 (  3.58-  13.99)
 YAMAGU 9   b   6        334       -     128       -      2.26 (  1.82-   2.81)
 ZOIA   3   b   4          -       -       -       -      1.45 (  0.66-   3.16)
Partial Totals         30068   70113   11966   70660
*prospective study                                        ~ With 0.5 adjustment for zero


 REF    NRR SEX ADJ             Ys       Ws       Qs       Ps
 ALDERS 8   m   1              1.01    21.40     0.37       0.00
 ALDERS 6   f   1              1.03    46.22     1.00       0.00
 Subtotal ALDERS               1.02    67.63     1.37
 ANDER1 32  m   1              1.51     3.29     1.29       0.01
 ANDER1 35  f   1              0.79     7.52     0.06       0.03
 Subtotal ANDER1               1.01    10.81     1.35
 BECK1  3   m   0              1.48     2.49     0.91       0.02
 BECK1  6   f   0              0.98     3.64     0.04       0.06
 Subtotal BECK1                1.19     6.13     0.95
*BECK2  3   m   0              0.66     1.45     0.07       0.42
*BECK2  6   f   0              0.76     1.74     0.03       0.32
 Subtotal BECK2                0.71     3.19     0.09
*BEST   3   m   1              2.44     2.89     7.01       0.00
 BJORNS 6   b   4              0.78   204.02     2.04       0.00
 BROWN  6   m   0              0.72    28.38     0.71       0.00
 CERVER 6   b   6              0.92   440.93     0.89       0.00
 COATES 3   b   0              1.29    17.22     2.88       0.00
 COLLEG 6   m   2              1.06     3.30     0.11       0.05
 COLLEG 18  f   1              0.97    13.25     0.11       0.00
 Subtotal COLLEG               0.99    16.56     0.22
 DEANE  6   m   1              1.82     2.62     2.30       0.00
 DEMARC 20  b   1              1.09    67.85     2.88       0.00
*DOLL1  13  m   1              2.00     2.65     3.33       0.00
*DONTA2 3   m   0              1.10     7.69     0.37       0.00
 DOPICO 3   m   0              0.93     4.58     0.01       0.05
 EHRLIC 15  m   6              0.24    15.26     6.26       0.35
 EHRLIC 21  f   6              0.49    30.45     4.50       0.01
 Subtotal EHRLIC               0.41    45.71    10.76
 ENRIGH 3   m   0              0.71    13.33     0.37       0.01
 ENRIGH 6   f   0              0.48    43.33     6.85       0.00
 Subtotal ENRIGH               0.54    56.66     7.22
 FERRI1 61  m   1              1.17     8.10     0.68       0.00
 FERRI1 91  f   1              0.77    16.83     0.18       0.00
 Subtotal FERRI1               0.90    24.93     0.87
 FINKLE 1   m   0              1.23   473.17    59.55       0.00
 FOXMAN 3   m   1              0.91    29.97     0.03       0.00
 FOXMAN 6   f   1              0.95    38.71     0.18       0.00
 Subtotal FOXMAN               0.93    68.68     0.21
 GOLDBE 6   m   1              0.39    86.29    20.49       0.00
 GOLDBE 12  f   1              1.05    46.09     1.41       0.00
 Subtotal GOLDBE               0.62   132.38    21.90
 HAENSZ 13  m   1              0.92    21.71     0.04       0.00
 HAENSZ 41  f   1              1.20    39.61     4.07       0.00
 Subtotal HAENSZ               1.10    61.32     4.12
 HARDIE 9   m   1              2.39     1.39     3.16       0.00
 HARDIE 12  f   1              1.20     7.02     0.74       0.00
 Subtotal HARDIE               1.40     8.41     3.89
 HARRIS 3   m   0              1.35     3.91     0.87       0.01
 HAWTHO 10  m   0              1.18    44.34     3.99       0.00
 HAWTHO 17  f   0              1.08    74.63     3.02       0.00
 Subtotal HAWTHO               1.12   118.97     7.00
 HAYES  9   b   3              1.74    34.27    25.42       0.00
 HIGGI2 3   m   0              1.00     0.93     0.01       0.34
 HIGGI2 15  f   1              0.90     2.86     0.00       0.13
 Subtotal HIGGI2               0.92     3.78     0.02
 HIGGI3 3   m   0              0.02     0.43     0.32       0.99
 HIGGI3 9   f   0              2.50     0.41     1.07       0.11
 Subtotal HIGGI3               1.23     0.84     1.38
 HIGGI6 3   m   1              1.52    15.23     6.33       0.00
  ________________________________________________________________________________________________________________________
                                            International Evidence on Smoking and COPD, Phase 3, Analysis run on 27-SEP-10

                                                   Table 2 - A - 2 - 2

            IESCOPD - Meta-analysis of ever smoking, cigarettes (or all products if cigarettes not available)
                                                          Any CB
                                                      Most-adjusted


 REF    NRR SEX ADJ             Ys       Ws       Qs       Ps
 HIGGI6 19  f   1              1.10    25.68     1.20       0.00
 Subtotal HIGGI6               1.25    40.91     7.53
 HO     11  b   3              0.84    23.83     0.03       0.00
 HOLLA2 3   m   0              1.80     4.51     3.83       0.00
 HOLLNA 3   m   0              1.71     8.26     5.70       0.00
 HOLLNA 11  f   0              1.52    10.64     4.32       0.00
 Subtotal HOLLNA               1.60    18.90    10.01
 HOUSE  6   m   1              1.44    39.43    12.28       0.00
 HOUSE  12  f   1              1.50    60.93    23.28       0.00
 Subtotal HOUSE                1.47   100.36    35.56
 HUCHON 6   b   1              0.79   109.85     0.82       0.00
 HUHTI1 42  m   1              1.95     6.02     6.94       0.00
 HUHTI1 127 f   1              1.29     7.57     1.27       0.00
 Subtotal HUHTI1               1.58    13.59     8.21
 HUHTI3 22  m   1              1.07    19.83     0.71       0.00
 JINDA2 4   b   6              0.67    84.54     3.78       0.00
 JOSHI  9   m   0              1.84     7.10     6.56       0.00
 JOUSI1 3   m   0              0.89   251.69     0.01       0.00
 JOUSI1 6   f   0              0.51   180.58    25.12       0.00
 Subtotal JOUSI1               0.73   432.27    25.13
*KAHN2  33  m   1              1.27    11.13     1.70       0.00
 KATO   3   m   1              0.29    94.89    33.49       0.01
 KATO   6   f   1              0.65   169.34     8.72       0.00
 Subtotal KATO                 0.52   264.24    42.22
 KIRAZ  4   f   1              1.39     5.71     1.47       0.00
 KOTAN1 9   b   7              0.56    39.41     4.03       0.00
 KUBIK  3   m   0              1.33   348.61    69.90       0.00
 KUBIK  4   f   0              0.70   205.79     6.27       0.00
 Subtotal KUBIK                1.10   554.40    76.17
 LAMBER 6   m   1              0.96    15.33     0.09       0.00
 LAMBER 22  f   1              1.06    32.44     1.03       0.00
 Subtotal LAMBER               1.02    47.77     1.12
 LANGHA 6   m   1              0.98   156.19     1.53       0.00
 LANGHA 12  f   1              0.67   201.94     9.03       0.00
 Subtotal LANGHA               0.80   358.12    10.56
 LAVECC 9   b   6              0.82   613.78     2.17       0.00
 LEBOWI 30  b   3              0.12    15.02     8.61       0.64
 LINDST 23  b   5              0.42   246.53    52.30       0.00
 LUNDB2 6   m   1              2.06     9.44    13.14       0.00
 LUNDB2 12  f   1              1.42    11.15     3.27       0.00
 Subtotal LUNDB2               1.71    20.59    16.41
 MAGNUS 6   m   1              1.43    11.82     3.59       0.00
 MANFRE 3   m   0              1.69     2.49     1.62       0.01
 MANFRE 6   f   0              1.10    12.16     0.60       0.00
 Subtotal MANFRE               1.20    14.64     2.22
 MELLST 3   m   0              1.07    12.26     0.46       0.00
 MENEZ1 6   b   8              1.08    15.79     0.60       0.00
 MEREN  6   b   4              0.56    70.09     7.16       0.00
 MILLER 3   m   1              1.66     8.74     5.27       0.00
 MILLER 6   f   1              3.01     1.90     8.62       0.00
 Subtotal MILLER               1.90    10.64    13.89
 MILNE  3   m   0              2.11     2.61     3.96       0.00
 MILNE  9   f   0              1.26     5.46     0.80       0.00
 Subtotal MILNE                1.54     8.07     4.76
 MOLLER 3   b   0              2.07     1.28     1.83       0.02
 MUELLE 36  m   1              1.96     1.83     2.12       0.01
 MUELLE 51  f   1              2.35     2.13     4.61       0.00
 Subtotal MUELLE               2.17     3.96     6.73
 NEJJAR 5   b   6              0.44    41.71     7.88       0.00
 OGILVI 5   m   0              1.30     7.36     1.31       0.00
 OGILVI 14  f   0              0.83    25.68     0.06       0.00
 Subtotal OGILVI               0.93    33.04     1.37
 OSWAL2 2   m   1              0.34    67.29    19.31       0.00
 OSWAL2 4   f   1              0.52    55.52     6.98       0.00
 Subtotal OSWAL2               0.43   122.81    26.29
 PANDEY 6   m   2              1.68     6.71     4.31       0.00
 PANDEY 12  f   2              0.57    42.02     4.14       0.00
 Subtotal PANDEY               0.72    48.72     8.45
*PELKON 6   m   1              0.57    45.52     4.49       0.00
 PEREZP 5   f   0              0.08    12.79     8.20       0.78
 REID   6   m   1              1.53    14.70     6.19       0.00
  ________________________________________________________________________________________________________________________
                                            International Evidence on Smoking and COPD, Phase 3, Analysis run on 27-SEP-10

                                                   Table 2 - A - 2 - 2

            IESCOPD - Meta-analysis of ever smoking, cigarettes (or all products if cigarettes not available)
                                                          Any CB
                                                      Most-adjusted


 REF    NRR SEX ADJ             Ys       Ws       Qs       Ps
 REID   22  f   1              1.20    21.34     2.19       0.00
 Subtotal REID                 1.33    36.04     8.38
 SAWICK 5   m   0              1.74    12.73     9.32       0.00
 SAWICK 18  f   0              1.43    26.87     8.09       0.00
 Subtotal SAWICK               1.53    39.60    17.41
 SHIMUR 3   b   0              0.00     0.60     0.46       1.00
*SILVA  6   b   6              0.57    11.41     1.12       0.06
 SOBRAD 4   b   3              1.90    12.46    12.95       0.00
 STJERN 3   b   0              1.37     4.23     1.02       0.00
*TROISI 3   f   1              0.66   171.12     8.42       0.00
 VIEGI1 3   m   0              1.36     3.52     0.80       0.01
 VIEGI1 6   f   0             -1.29     0.87     4.12       0.23
 Subtotal VIEGI1               0.83     4.39     4.91
 WAGEN2 11  b   1              0.54    70.38     7.99       0.00
 WILHEL 3   m   0              2.02     0.93     1.22       0.05
 WOODS  3   b   5              0.90   167.48     0.07       0.00
 WOOLF  3   f   0              1.96     8.26     9.58       0.00
 YAMAGU 9   b   6              0.82    81.45     0.33       0.00
 ZOIA   3   b   4              0.37     6.27     1.62       0.35

                       N      114
                      NS       79


                      Wt  6016.30
                 Het Chi   661.95
                 Het  df      113
                 Het  P       ***
               Fixed  RR     2.41
                     RRl     2.35
                     RRu     2.47
                      P       +++
              Random  RR     2.70
                     RRl     2.50
                     RRu     2.91
                      P       +++
               Asymm  P        **


  ________________________________________________________________________________________________________________________
                                            International Evidence on Smoking and COPD, Phase 3, Analysis run on 27-SEP-10

                                                   Table 2 - A - 2 - 3

            IESCOPD - Meta-analysis of ever smoking, cigarettes (or all products if cigarettes not available)
                                                          Any CB
                                                      Most-adjusted


                       N      114
                      NS       79


                      Wt  6016.30
                 Het Chi   661.95
                 Het  df      113
                 Het  P       ***
               Fixed  RR     2.41
                     RRl     2.35
                     RRu     2.47
                      P       +++
              Random  RR     2.70
                     RRl     2.50
                     RRu     2.91
                      P       +++
               Asymm  P        **

                                   Sex
                             both      male    female     Total


                       N       24        51        39       114
                      NS       24        51        39       114


                      Wt  2380.39   1965.72   1670.19   6016.30
                 Het Chi   131.03    277.38    162.89    661.95
                 Het  df       23        50        38       113
                 Het  P       ***       ***       ***       ***
               Fixed  RR     2.21      2.87      2.22      2.41
                     RRl     2.12      2.75      2.11      2.35
                     RRu     2.30      3.00      2.32      2.47
                      P       +++       +++       +++       +++
              Random  RR     2.27      3.21      2.57      2.70
                     RRl     2.02      2.81      2.28      2.50
                     RRu     2.55      3.68      2.89      2.91
                      P       +++       +++       +++       +++
             Between Chi                                  90.65
             Between  df                                      2
             Between  P                                     ***
             Btwn(F)  P                                     ***

                                        Continent
                            NAmer    Europe      Asia  oth/mult     Total


                       N       38        60         9         7       114
                      NS       25        41         7         6        79


                      Wt  1265.52   3926.77    510.47    313.54   6016.30
                 Het Chi   191.22    336.20     33.49     23.11    661.95
                 Het  df       37        59         8         6       113
                 Het  P       ***       ***       ***       ***       ***
               Fixed  RR     2.92      2.34      1.90      2.34      2.41
                     RRl     2.76      2.27      1.75      2.09      2.35
                     RRu     3.08      2.42      2.08      2.61      2.47
                      P       +++       +++       +++       +++       +++
              Random  RR     3.19      2.64      2.12      2.09      2.70
                     RRl     2.72      2.40      1.72      1.59      2.50
                     RRu     3.74      2.90      2.62      2.74      2.91
                      P       +++       +++       +++       +++       +++
             Between Chi                                            77.94
             Between  df                                                3
             Between  P                                               ***
             Btwn(F)  P                                                **


  ________________________________________________________________________________________________________________________
                                            International Evidence on Smoking and COPD, Phase 3, Analysis run on 27-SEP-10

                                                   Table 2 - A - 2 - 3

            IESCOPD - Meta-analysis of ever smoking, cigarettes (or all products if cigarettes not available)
                                                          Any CB
                                                      Most-adjusted
                               Study type
                               CC        Pr        CS     Total


                       N       10        11        93       114
                      NS        7         9        63        79


                      Wt   206.31    374.56   5435.43   6016.30
                 Het Chi    37.54     32.97    590.72    661.95
                 Het  df        9        10        92       113
                 Het  P       ***       ***       ***       ***
               Fixed  RR     2.36      2.32      2.42      2.41
                     RRl     2.06      2.09      2.35      2.35
                     RRu     2.70      2.56      2.48      2.47
                      P       +++       +++       +++       +++
              Random  RR     2.74      2.67      2.70      2.70
                     RRl     1.97      2.09      2.49      2.50
                     RRu     3.79      3.42      2.94      2.91
                      P       +++       +++       +++       +++
             Between Chi                                   0.72
             Between  df                                      2
             Between  P                                    N.S.
             Btwn(F)  P                                    N.S.

                           CB subtype
                             mort     sympt     other     Total


                       N        3        83        28       114
                      NS        3        56        20        79


                      Wt    16.67   4575.81   1423.82   6016.30
                 Het Chi     3.66    520.81     98.18    661.95
                 Het  df        2        82        27       113
                 Het  P      N.S.       ***       ***       ***
               Fixed  RR     4.90      2.50      2.11      2.41
                     RRl     3.03      2.43      2.01      2.35
                     RRu     7.91      2.58      2.23      2.47
                      P       +++       +++       +++       +++
              Random  RR     5.78      2.78      2.31      2.70
                     RRl     2.76      2.55      2.02      2.50
                     RRu    12.13      3.04      2.65      2.91
                      P       +++       +++       +++       +++
             Between Chi                                  39.29
             Between  df                                      2
             Between  P                                     ***
             Btwn(F)  P                                       *

                             Smoking product
                              any      cigs  cigsonly     Total


                       N       46        66         2       114
                      NS       33        49         2        84


                      Wt  3062.36   2904.83     49.11   6016.30
                 Het Chi   284.78    334.06      5.41    661.95
                 Het  df       45        65         1       113
                 Het  P       ***       ***         *       ***
               Fixed  RR     2.59      2.22      3.03      2.41
                     RRl     2.50      2.14      2.29      2.35
                     RRu     2.68      2.30      4.01      2.47
                      P       +++       +++       +++       +++
              Random  RR     2.98      2.51      5.04      2.70
                     RRl     2.64      2.28      1.29      2.50
                     RRu     3.36      2.77     19.70      2.91
                      P       +++       +++         +       +++
             Between Chi                                  37.71
             Between  df                                      2
             Between  P                                     ***
             Btwn(F)  P                                       *
  ________________________________________________________________________________________________________________________
                                            International Evidence on Smoking and COPD, Phase 3, Analysis run on 27-SEP-10

                                                   Table 2 - A - 2 - 3

            IESCOPD - Meta-analysis of ever smoking, cigarettes (or all products if cigarettes not available)
                                                          Any CB
                                                      Most-adjusted
                                     Unexposed group
                          nev any   nev cig  nev+ any  nev+ cig     Total


                       N       57        51         2         4       114
                      NS       42        41         1         2        86


                      Wt  2763.37   2456.76    554.40    241.78   6016.30
                 Het Chi   243.32    285.62     50.12     30.18    661.95
                 Het  df       56        50         1         3       113
                 Het  P       ***       ***       ***       ***       ***
               Fixed  RR     2.52      2.21      2.99      2.15      2.41
                     RRl     2.42      2.12      2.75      1.90      2.35
                     RRu     2.61      2.30      3.25      2.44      2.47
                      P       +++       +++       +++       +++       +++
              Random  RR     2.94      2.54      2.77      2.18      2.70
                     RRl     2.64      2.27      1.50      1.46      2.50
                     RRu     3.27      2.83      5.09      3.26      2.91
                      P       +++       +++        ++       +++       +++
             Between Chi                                            52.72
             Between  df                                                3
             Between  P                                               ***
             Btwn(F)  P                                                 *

                        Unexposed group (combining nev+ with main levels)
                          nev any   nev cig     Total


                       N       59        55       114
                      NS       43        43        86


                      Wt  3317.77   2698.54   6016.30
                 Het Chi   307.33    315.96    661.95
                 Het  df       58        54       113
                 Het  P       ***       ***       ***
               Fixed  RR     2.59      2.20      2.41
                     RRl     2.50      2.12      2.35
                     RRu     2.68      2.29      2.47
                      P       +++       +++       +++
              Random  RR     2.94      2.50      2.70
                     RRl     2.65      2.25      2.50
                     RRu     3.27      2.78      2.91
                      P       +++       +++       +++
             Between Chi                        38.67
             Between  df                            1
             Between  P                           ***
             Btwn(F)  P                            **


  ________________________________________________________________________________________________________________________
                                            International Evidence on Smoking and COPD, Phase 3, Analysis run on 27-SEP-10

                                                   Table 2 - A - 2 - 4

            IESCOPD - Meta-analysis of ever smoking, cigarettes (or all products if cigarettes not available)
                                                          Any CB
                                                      Least-adjusted


     REF|NRR|X|SEX|AGEL|AGEH|     REGION|BEGYR|PUBYR|STTYP|ONSET|      DISEAS|ADJ|SMOKSTA|   PRODUCT|    UNEXP|

  ALDERS   4 x   m   35   74       Eu:UK  1977  1985    CC  Prev CB:diagnosed   0    Ever       Cigs  Nev cigs
  ALDERS   2 x   f   35   74       Eu:UK  1977  1985    CC  Prev CB:diagnosed   0    Ever MCigs only   Nev any
  ANDER1  32     m   25   74   Am:Canada  1963  1965    CS  Prev  CB:symptoms   1    Ever        Any   Nev any
  ANDER1  35     f   25   74   Am:Canada  1963  1965    CS  Prev  CB:symptoms   1    Ever        Any   Nev any
   BECK1   3     m   11   99      Am:USA  1972  1982    CS  Prev  CB:symptoms   0    Ever       Cigs   Nev any
   BECK1   6     f   11   99      Am:USA  1972  1982    CS  Prev  CB:symptoms   0    Ever       Cigs   Nev any
   BECK2   3     m   11   99      Am:USA  1972  1982    Pr   Inc  CB:symptoms   0    Ever       Cigs   Nev any
   BECK2   6     f   11   99      Am:USA  1972  1982    Pr   Inc  CB:symptoms   0    Ever       Cigs   Nev any
    BEST   3     m   30   97   Am:Canada  1955  1967    Pr   Inc CB:mortality   1    Ever  Cigs only   Nev any
  BJORNS   3 x   b   20   44    Eu:Scand  1990  1994    CS  Prev  CB:symptoms   0    Ever        Any   Nev any
   BROWN   6     m   60   69       Eu:UK  1956  1957    CS  Prev CB:diagnosed   0    Ever       Cigs  Nev cigs
  CERVER   3 x   b   20   44     Eu:West  1998  2003    CS  Prev  CB:symptoms   3    Ever       Cigs  Nev cigs
  COATES   3     b   40   64      Am:USA  1962  1965    CS  Prev  CB:symptoms   0    Ever       Cigs  Nev cigs
  COLLEG   3 x   m   40   64       Eu:UK     *  1961    CS  Prev CB:diagnosed   1    Ever        Any   Nev any
  COLLEG  18     f   40   64       Eu:UK     *  1961    CS  Prev CB:diagnosed   1    Ever       Cigs  Nev cigs
   DEANE   5 x   m   40   59      Am:USA  1963  1965    CS  Prev  CB:symptoms   0    Ever        Any   Nev any
  DEMARC  17 x   b   20   44       Multi  1991  2004    CS  Prev  CB:symptoms   0    Ever       Cigs   Nev any
   DOLL1  13     m   20   99       Eu:UK  1951  1994    Pr   Inc CB:mortality   1    Ever        Any   Nev any
  DONTA2   3     m   25   84 Eu:SE/Balkn  1960  1984    Pr   Inc CB:diagnosed   0    Ever       Cigs  Nev cigs
  DOPICO   3     m   15    *      Am:USA     *  1984    CS  Prev  CB:symptoms   0    Ever       Cigs  Nev cigs
  EHRLIC   3 x   m   15   99      Africa  1998  2004    CS  Prev  CB:symptoms   0    Ever        Any   Nev any
  EHRLIC   9 x   f   15   99      Africa  1998  2004    CS  Prev  CB:symptoms   0    Ever        Any   Nev any
  ENRIGH   3     m   65   99      Am:USA  1989  1994    CS  Prev CB:diagnosed   0    Ever       Cigs  Nev cigs
  ENRIGH   6     f   65   99      Am:USA  1989  1994    CS  Prev CB:diagnosed   0    Ever       Cigs  Nev cigs
  FERRI1  61     m   25   74      Am:USA  1961  1971    CS  Prev  CB:symptoms   1    Ever        Any   Nev any
  FERRI1  91     f   25   74      Am:USA  1961  1971    CS  Prev  CB:symptoms   1    Ever       Cigs  Nev cigs
  FINKLE   1     m   18   25      Am:USA  1969  1974    CS  Prev  CB:symptoms   0    Ever        Any   Nev any
  FOXMAN   3     m   20   69      Am:USA     *  1986    CS  Prev  CB:symptoms   1    Ever        Any   Nev any
  FOXMAN   6     f   20   69      Am:USA     *  1986    CS  Prev  CB:symptoms   1    Ever        Any   Nev any
  GOLDBE   3 x   m   15   99      Am:USA  1970  1974    CS  Prev  CB:symptoms   0    Ever       Cigs  Nev cigs
  GOLDBE   9 x   f   15   99      Am:USA  1970  1974    CS  Prev  CB:symptoms   0    Ever       Cigs  Nev cigs
  HAENSZ   6 x   m   35   74    Eu:Scand  1964  1972    CS  Prev  CB:symptoms   0    Ever       Cigs   Nev any
  HAENSZ  26 x   f   35   74    Eu:Scand  1964  1972    CS  Prev  CB:symptoms   0    Ever       Cigs  Nev cigs
  HARDIE   9     m   70   99    Eu:Scand  1998  2005    CS  Prev CB:diagnosed   1    Ever       Cigs  Nev cigs
  HARDIE  12     f   70   99    Eu:Scand  1998  2005    CS  Prev CB:diagnosed   1    Ever       Cigs  Nev cigs
  HARRIS   3     m   15   60      Africa     *  1993    CS  Prev  CB:symptoms   0    Ever        Any   Nev any
  HAWTHO  10     m   45   76       Eu:UK  1965  1978    Pr  Prev  CB:symptoms   0    Ever       Cigs  N/L cigs
  HAWTHO  17     f   45   76       Eu:UK  1965  1978    Pr  Prev  CB:symptoms   0    Ever       Cigs  N/L cigs
   HAYES   3 x   m   15   99      Am:USA  1970  1974    CS  Prev  CB:symptoms   0    Ever       Cigs  Nev cigs
   HAYES   6 x   f   15   99      Am:USA  1970  1974    CS  Prev  CB:symptoms   0    Ever       Cigs  Nev cigs
  HIGGI2   3     m   25   74       Eu:UK  1956  1957    CS  Prev  CB:symptoms   0    Ever       Cigs  Nev cigs
  HIGGI2   9 x   f   25   74       Eu:UK  1956  1957    CS  Prev  CB:symptoms   0    Ever       Cigs  Nev cigs
  HIGGI3   3     m   55   64       Eu:UK  1956  1958    CS  Prev  CB:symptoms   0    Ever        Any   Nev any
  HIGGI3   9     f   55   64       Eu:UK  1956  1958    CS  Prev  CB:symptoms   0    Ever        Any   Nev any
  HIGGI6   3     m   25   64      Am:USA  1962  1977    CS  Prev  CB:symptoms   1    Ever        Any   Nev any
  HIGGI6  19     f   25   64      Am:USA  1962  1977    CS  Prev  CB:symptoms   1    Ever       Cigs  Nev cigs
      HO  11     b   70   99   Asia:FarE  1991  1999    CS  Prev  CB:self-rep   3    Ever       Cigs  Nev cigs
  HOLLA2   3     m   40   59      Am:USA  1962  1965    CS  Prev  CB:symptoms   0    Ever        Any   Nev any
  HOLLNA   3     m   40   40    Eu:Scand  1976  1983    CS  Prev  CB:symptoms   0    Ever        Any   Nev any
  HOLLNA  11     f   40   40    Eu:Scand  1976  1983    CS  Prev  CB:symptoms   0    Ever        Any   Nev any
   HOUSE   3 x   m   15   99      Am:USA  1970  1974    CS  Prev  CB:symptoms   0    Ever       Cigs  Nev cigs
   HOUSE   9 x   f   15   99      Am:USA  1970  1974    CS  Prev  CB:symptoms   0    Ever       Cigs  Nev cigs
  HUCHON   3 x   b   25   99     Eu:West     *  2002    CS  Prev  CB:symptoms   0    Ever       Cigs  Nev cigs
  HUHTI1  39 x   m   40   64    Eu:Scand  1961  1965    CS  Prev  CB:symptoms   0    Ever        Any   Nev any
  HUHTI1 124 x   f   40   64    Eu:Scand  1961  1965    CS  Prev  CB:symptoms   0    Ever       Cigs  Nev cigs
  HUHTI3  19 x   m   25   69    Eu:Scand  1968  1978    CS  Prev  CB:symptoms   0    Ever        Any   Nev any
  JINDA2   2 x   b   35   99 Asia:MidE/S     *  2006    CS  Prev  CB:symptoms   0    Ever       Cigs   Nev any
   JOSHI   9     m   17   64 Asia:MidE/S     *  1975    CS  Prev  CB:symptoms   0    Ever       Cigs  Nev cigs
  JOUSI1   3     m   25   64    Eu:Scand  1972  1996    CS  Prev  CB:symptoms   0    Ever        Any   Nev any
  JOUSI1   6     f   25   64    Eu:Scand  1972  1996    CS  Prev  CB:symptoms   0    Ever        Any   Nev any
   KAHN2  33     m   31   84      Am:USA  1954  1966    Pr   Inc CB:mortality   1    Ever       Cigs   Nev any
    KATO   3     m   40   99   Asia:FarE  1985  1989    CS  Prev  CB:self-rep   1    Ever       Cigs  Nev cigs
    KATO   6     f   40   99   Asia:FarE  1985  1989    CS  Prev  CB:self-rep   1    Ever       Cigs  Nev cigs
   KIRAZ   3 x   f   25   99 Eu:SE/Balkn  1999  2003    CS  Prev     CB:other   0    Ever       Cigs  Nev cigs
  KOTAN1   3 x   m   20   69    Eu:Scand  1995  2003    CS  Prev CB:diagnosed   0    Ever        Any   Nev any
  KOTAN1   6 x   f   20   69    Eu:Scand  1995  2003    CS  Prev CB:diagnosed   0    Ever        Any   Nev any
   KUBIK   3     m   15   99     Eu:East  1972  1984    CS  Prev  CB:symptoms   0    Ever        Any   N/L any
   KUBIK   4     f   15   99     Eu:East  1972  1984    CS  Prev  CB:symptoms   0    Ever        Any   N/L any
  LAMBER   3 x   m   35   69       Eu:UK  1965  1970    CS  Prev  CB:symptoms   0    Ever       Cigs  Nev cigs
  LAMBER  19 x   f   35   69       Eu:UK  1965  1970    CS  Prev  CB:symptoms   0    Ever       Cigs  Nev cigs
  LANGHA   3 x   m   20   99    Eu:Scand  1995  2000    CS  Prev  CB:symptoms   0    Ever        Any   Nev any
  LANGHA   9 x   f   20   99    Eu:Scand  1995  2000    CS  Prev  CB:symptoms   0    Ever        Any   Nev any
  ________________________________________________________________________________________________________________________
                                            International Evidence on Smoking and COPD, Phase 3, Analysis run on 27-SEP-10

                                                   Table 2 - A - 2 - 4

            IESCOPD - Meta-analysis of ever smoking, cigarettes (or all products if cigarettes not available)
                                                          Any CB
                                                      Least-adjusted


     REF|NRR|X|SEX|AGEL|AGEH|     REGION|BEGYR|PUBYR|STTYP|ONSET|      DISEAS|ADJ|SMOKSTA|   PRODUCT|    UNEXP|

  LAVECC   3 x   m   15   99     Eu:West  1983  1988    CS  Prev  CB:self-rep   0    Ever        Any   Nev any
  LAVECC   6 x   f   15   99     Eu:West  1983  1988    CS  Prev  CB:self-rep   0    Ever        Any   Nev any
  LEBOWI  27 x   b   15   96      Am:USA  1972  1977    CS  Prev CB:diagnosed   2    Ever       Cigs  Nev cigs
  LINDST  17 x   m   20   69    Eu:Scand     *  2001    CS  Prev  CB:symptoms   0    Ever       Cigs  Nev cigs
  LINDST  20 x   f   20   69    Eu:Scand     *  2001    CS  Prev  CB:symptoms   0    Ever       Cigs  Nev cigs
  LUNDB2   3 x   m   35   66    Eu:Scand  1986  1993    CC  Prev     CB:other   0    Ever        Any   Nev any
  LUNDB2   9 x   f   35   66    Eu:Scand  1986  1993    CC  Prev     CB:other   0    Ever        Any   Nev any
  MAGNUS   3 x   m   50   80    Eu:Scand  1993  1999    CS  Prev  CB:symptoms   0    Ever        Any   Nev any
  MANFRE   3     m   20   65   Am:Canada  1978  1989    CS  Prev  CB:symptoms   0    Ever        Any   Nev any
  MANFRE   6     f   20   65   Am:Canada  1978  1989    CS  Prev  CB:symptoms   0    Ever        Any   Nev any
  MELLST   3     m   70   70    Eu:Scand  1971  1982    CS  Prev     CB:other   0    Ever        Any   Nev any
  MENEZ1   3 x   b   40   99 Am:Sth/Cent  1990  1995    CS  Prev  CB:symptoms   0    Ever       Cigs  Nev cigs
   MEREN   6     b   15   64     Eu:East  1995  2001    CS  Prev  CB:symptoms   4    Ever       Cigs  Nev cigs
  MILLER   3     m   15   99      Am:USA  1978  1988    CS  Prev CB:diagnosed   1    Ever       Cigs  Nev cigs
  MILLER   6     f   15   99      Am:USA  1978  1988    CS  Prev CB:diagnosed   1    Ever       Cigs  Nev cigs
   MILNE   3     m   62   90       Eu:UK  1968  1972    CS  Prev  CB:symptoms   0    Ever       Cigs  Nev cigs
   MILNE   9     f   62   90       Eu:UK  1968  1972    CS  Prev  CB:symptoms   0    Ever       Cigs  Nev cigs
  MOLLER   3     b   40   72     Eu:West     *  2001    CC  Prev  CB:symptoms   0    Ever       Cigs  Nev cigs
  MUELLE  33 x   m   20   69      Am:USA  1967  1971    CS  Prev  CB:symptoms   0    Ever        Any   Nev any
  MUELLE  49 x   f   20   69      Am:USA  1967  1971    CS  Prev  CB:symptoms   0    Ever        Any   Nev any
  NEJJAR   1 x   m   65   99     Eu:West  1991  1996    CS  Prev  CB:symptoms   0    Ever       Cigs  Nev cigs
  NEJJAR   2 x   f   65   99     Eu:West  1991  1996    CS  Prev  CB:symptoms   0    Ever       Cigs  Nev cigs
  OGILVI   5     m   30   99       Eu:UK  1955  1957    CC  Prev  CB:symptoms   0    Ever       Cigs   Nev any
  OGILVI  14     f   30   99       Eu:UK  1955  1957    CC  Prev  CB:symptoms   0    Ever       Cigs  Nev cigs
  OSWAL2   1 x   m   15   65       Eu:UK  1954  1955    CS  Prev  CB:symptoms   0    Ever       Cigs  N/L cigs
  OSWAL2   3 x   f   15   65       Eu:UK  1954  1955    CS  Prev  CB:symptoms   0    Ever       Cigs  N/L cigs
  PANDEY   3 x   m   20   99 Asia:MidE/S  1979  1984    CS  Prev  CB:symptoms   0    Ever        Any   Nev any
  PANDEY   9 x   f   20   99 Asia:MidE/S  1979  1984    CS  Prev  CB:symptoms   0    Ever        Any   Nev any
  PELKON   6     m   40   75    Eu:Scand  1959  2006    Pr   Inc     CB:other   1    Ever       Cigs  Nev cigs
  PEREZP   5     f   40   99 Am:Sth/Cent  1992  1996    CC  Prev  CB:symptoms   0    Ever       Cigs  Nev cigs
    REID   3 x   m   35   74      Am:USA  1962  1966    CS  Prev  CB:symptoms   0    Ever       Cigs  Nev cigs
    REID  19 x   f   35   74      Am:USA  1962  1966    CS  Prev  CB:symptoms   0    Ever       Cigs  Nev cigs
  SAWICK   5     m   19   70     Eu:East  1968  1972    CS  Prev  CB:symptoms   0    Ever        Any   Nev any
  SAWICK  18     f   19   70     Eu:East  1968  1972    CS  Prev  CB:symptoms   0    Ever        Any   Nev any
  SHIMUR   3     b   31   80   Asia:FarE     *  1996    CC  Prev     CB:other   0    Ever        Any   Nev any
   SILVA   6     b   20   99      Am:USA  1972  2004    Pr   Inc     CB:other   6    Ever       Cigs  Nev cigs
  SOBRAD   3 x   b   40   69     Eu:West  1996  2000    CS  Prev  CB:symptoms   0    Ever        Any   Nev any
  STJERN   3     b   16   72    Eu:Scand  1981  1985    CS  Prev  CB:symptoms   0    Ever        Any   Nev any
  TROISI   3     f   34   69      Am:USA  1980  1995    Pr   Inc CB:diagnosed   1    Ever       Cigs  Nev cigs
  VIEGI1   3     m   20   64     Eu:West  1980  1988    CS  Prev CB:diagnosed   0    Ever       Cigs   Nev any
  VIEGI1   6     f   20   64     Eu:West  1980  1988    CS  Prev CB:diagnosed   0    Ever       Cigs   Nev any
  WAGEN2   3 x   b   21   68     Eu:West  2001  2004    CC  Prev  CB:symptoms   0    Ever       Cigs  Nev cigs
  WILHEL   3     m   54   54    Eu:Scand  1967  1969    CS  Prev  CB:symptoms   0    Ever        Any   Nev any
   WOODS   3     b   20   44      Aus/NZ     *  2000    CS  Prev  CB:symptoms   5    Ever       Cigs  Nev cigs
   WOOLF   3     f   25   54   Am:Canada     *  1974    CS  Prev  CB:symptoms   0    Ever       Cigs  Nev cigs
  YAMAGU   9     b   40   99   Asia:FarE  1986  1988    CS  Prev  CB:symptoms   6    Ever       Cigs  Nev cigs
    ZOIA   3     b   18   69     Eu:West     *  1995    CS  Prev  CB:symptoms   4    Ever        Any   Nev any


  ________________________________________________________________________________________________________________________
                                            International Evidence on Smoking and COPD, Phase 3, Analysis run on 27-SEP-10

                                                   Table 2 - A - 2 - 5

            IESCOPD - Meta-analysis of ever smoking, cigarettes (or all products if cigarettes not available)
                                                          Any CB
                                                      Least-adjusted


                        Number Exposed  Non-exposed
 REF    NRR SEX ADJ     Case    Cont    Case    Cont      RR        95.00%CI
 ALDERS 4   m   0        502     450      33      82      2.77 (  1.81-   4.23)
 ALDERS 2   f   0        333     239     105     203      2.69 (  2.02-   3.59)
 Subtotal ALDERS                                          2.72 (  2.14-   3.45)
 ANDER1 32  m   1         50       -       4       -      4.51 (  1.53-  13.29)
 ANDER1 35  f   1         19       -      15       -      2.20 (  1.08-   4.51)
 Subtotal ANDER1                                          2.74 (  1.51-   4.97)
 BECK1  3   m   0         17     243       3     189      4.41 (  1.27-  15.26)
 BECK1  6   f   0         10     198       6     318      2.68 (  0.96-   7.48)
 Subtotal BECK1                                           3.28 (  1.49-   7.24)
*BECK2  3   m   0          5     243       2     189      1.94 (  0.38-   9.91)
*BECK2  6   f   0          4     199       3     318      2.13 (  0.48-   9.42)
 Subtotal BECK2                                           2.04 (  0.68-   6.13)
*BEST   3   m   1         77       -       3       -     11.44 (  3.61-  36.25)
 BJORNS 3   b   0        836    3956     354    4179      2.49 (  2.19-   2.85)
 BROWN  6   m   0        212     573      41     228      2.06 (  1.42-   2.97)
 CERVER 3   b   3       1465       -     754       -      2.70 (  2.47-   2.97)
 COATES 3   b   0        135     913      21     515      3.63 (  2.26-   5.82)
 COLLEG 3   m   1          -       -       -       -      3.31 (  1.06-  10.33)
 COLLEG 18  f   1          -       -       -       -      2.64 (  1.54-   4.52)
 Subtotal COLLEG                                          2.75 (  1.69-   4.48)
 DEANE  5   m   0        104     307       3      66      7.45 (  2.29-  24.21)
 DEMARC 17  b   0        341    7197      91    5624      2.93 (  2.32-   3.70)
*DOLL1  13  m   1         95       -       3       -      7.40 (  2.22-  24.69)
*DONTA2 3   m   0         63     333       8     127      3.00 (  1.48-   6.09)
 DOPICO 3   m   0         37     139       6      57      2.53 (  1.01-   6.32)
 EHRLIC 3   m   0        100    2962      33    2672      2.73 (  1.84-   4.07)
 EHRLIC 9   f   0         88    1708     138    6208      2.32 (  1.76-   3.04)
 Subtotal EHRLIC                                          2.44 (  1.95-   3.06)
 ENRIGH 3   m   0         71    1432      17     699      2.04 (  1.19-   3.49)
 ENRIGH 6   f   0        102    1155      85    1558      1.62 (  1.20-   2.18)
 Subtotal ENRIGH                                          1.71 (  1.32-   2.22)
 FERRI1 61  m   1        156       -      10       -      3.22 (  1.62-   6.42)
 FERRI1 91  f   1         44       -      35       -      2.17 (  1.35-   3.51)
 Subtotal FERRI1                                          2.47 (  1.67-   3.65)
 FINKLE 1   m   0       3551   17761     596   10240      3.44 (  3.14-   3.76)
 FOXMAN 3   m   1        219       -      41       -      2.48 (  1.73-   3.54)
 FOXMAN 6   f   1        151       -      59       -      2.58 (  1.88-   3.53)
 Subtotal FOXMAN                                          2.54 (  2.00-   3.21)
 GOLDBE 3   m   0        348    1450     148     909      1.47 (  1.20-   1.82)
 GOLDBE 9   f   0        283    1729      61    1076      2.89 (  2.17-   3.85)
 Subtotal GOLDBE                                          1.86 (  1.57-   2.21)
 HAENSZ 6   m   0        149    6593      27    2742      2.30 (  1.52-   3.47)
 HAENSZ 26  f   0         79    3234      97   11536      2.91 (  2.15-   3.92)
 Subtotal HAENSZ                                          2.68 (  2.10-   3.41)
 HARDIE 9   m   1         37       -       1       -     10.90 (  2.06-  57.56)
 HARDIE 12  f   1         16       -      11       -      3.33 (  1.59-   6.98)
 Subtotal HARDIE                                          4.05 (  2.06-   7.96)
 HARRIS 3   m   0         12     268       6     518      3.87 (  1.43-  10.41)
 HAWTHO 10  m   0        646    8503      49    2097      3.25 (  2.42-   4.36)
 HAWTHO 17  f   0        320    3628     103    3440      2.95 (  2.35-   3.70)
 Subtotal HAWTHO                                          3.06 (  2.55-   3.66)
 HAYES  3   m   0        194    1440      12     591      6.64 (  3.67-  11.98)
 HAYES  6   f   0        163    1484      25    1386      6.09 (  3.97-   9.34)
 Subtotal HAYES                                           6.27 (  4.44-   8.87)
 HIGGI2 3   m   0         25     248       1      27      2.72 (  0.35-  20.89)
 HIGGI2 9   f   0          8      96       6     170      2.36 (  0.80-   7.01)
 Subtotal HIGGI2                                          2.44 (  0.93-   6.36)
 HIGGI3 3   m   0          6      82       0       6      1.02~(  0.05-  20.27)
 HIGGI3 9   f   0          2      26       0      64     12.17~(  0.57- 262.12)
 Subtotal HIGGI3                                          3.41 (  0.40-  28.99)
 HIGGI6 3   m   1        294       -      17       -      4.59 (  2.78-   7.59)
 HIGGI6 19  f   1         86       -      40       -      2.99 (  2.03-   4.40)
 Subtotal HIGGI6                                          3.51 (  2.58-   4.76)
 HO     11  b   3          -       -       -       -      2.32 (  1.55-   3.46)
 HOLLA2 3   m   0        142     394       5      84      6.05 (  2.41-  15.23)
 HOLLNA 3   m   0        139     254      10     101      5.53 (  2.79-  10.93)
 HOLLNA 11  f   0         72     245      14     217      4.56 (  2.50-   8.31)
 Subtotal HOLLNA                                          4.96 (  3.16-   7.78)
 HOUSE  3   m   0        273    1654      51    1327      4.29 (  3.16-   5.84)
 HOUSE  9   f   0        194    1187     103    2846      4.52 (  3.52-   5.79)
 Subtotal HOUSE                                           4.43 (  3.65-   5.37)
  ________________________________________________________________________________________________________________________
                                            International Evidence on Smoking and COPD, Phase 3, Analysis run on 27-SEP-10

                                                   Table 2 - A - 2 - 5

            IESCOPD - Meta-analysis of ever smoking, cigarettes (or all products if cigarettes not available)
                                                          Any CB
                                                      Least-adjusted


                        Number Exposed  Non-exposed
 REF    NRR SEX ADJ     Case    Cont    Case    Cont      RR        95.00%CI
 HUCHON 3   b   0        404    7364     177    7183      2.23 (  1.86-   2.66)
 HUHTI1 39  m   0        177     354       7     115      8.21 (  3.75-  17.99)
 HUHTI1 124 f   0         16      98      32     677      3.45 (  1.83-   6.53)
 Subtotal HUHTI1                                          4.87 (  2.97-   7.99)
 HUHTI3 19  m   0        272     538      28     211      3.81 (  2.50-   5.80)
 JINDA2 2   b   0        127    2286     784   25843      1.83 (  1.51-   2.22)
 JOSHI  9   m   0         50     194       9     220      6.30 (  3.02-  13.14)
 JOUSI1 3   m   0       1741    4824     360    2417      2.42 (  2.14-   2.74)
 JOUSI1 6   f   0        278    1344     940    7540      1.66 (  1.43-   1.92)
 Subtotal JOUSI1                                          2.07 (  1.88-   2.27)
*KAHN2  33  m   1         85       -      13       -      3.56 (  1.98-   6.41)
 KATO   3   m   1        827       -     118       -      1.33 (  1.09-   1.63)
 KATO   6   f   1        260       -     679       -      1.92 (  1.65-   2.23)
 Subtotal KATO                                            1.68 (  1.49-   1.90)
 KIRAZ  3   f   0         17      24      78     225      2.04 (  1.04-   4.00)
 KOTAN1 3   m   0         81    1949      29    1160      1.66 (  1.08-   2.56)
 KOTAN1 6   f   0         56    1416      43    1899      1.75 (  1.17-   2.61)
 Subtotal KOTAN1                                          1.71 (  1.27-   2.29)
 KUBIK  3   m   0       3253   16409     421    8006      3.77 (  3.39-   4.19)
 KUBIK  4   f   0        298    4760     797   25758      2.02 (  1.76-   2.32)
 Subtotal KUBIK                                           2.99 (  2.75-   3.25)
 LAMBER 3   m   0        257    3523      17     642      2.75 (  1.67-   4.53)
 LAMBER 19  f   0        118    2120      54    2390      2.46 (  1.78-   3.42)
 Subtotal LAMBER                                          2.55 (  1.94-   3.35)
 LANGHA 3   m   0        985   17046     203   10999      3.13 (  2.69-   3.65)
 LANGHA 9   f   0        635   15691     351   15771      1.82 (  1.59-   2.08)
 Subtotal LANGHA                                          2.29 (  2.08-   2.54)
 LAVECC 3   m   0       2053   18422     588   13724      2.60 (  2.37-   2.86)
 LAVECC 6   f   0        280    7357    1042   28818      1.05 (  0.92-   1.20)
 Subtotal LAVECC                                          1.93 (  1.79-   2.08)
 LEBOWI 27  b   2         37       -      26       -      1.12 (  0.68-   1.86)
 LINDST 17  m   0        444    3350     214    2839      1.76 (  1.48-   2.09)
 LINDST 20  f   0        319    2795     280    3487      1.42 (  1.20-   1.68)
 Subtotal LINDST                                          1.58 (  1.40-   1.78)
 LUNDB2 3   m   0        232      91      15      41      6.97 (  3.68-  13.20)
 LUNDB2 9   f   0        105      58      35      73      3.78 (  2.26-   6.32)
 Subtotal LUNDB2                                          4.81 (  3.22-   7.18)
 MAGNUS 3   m   0         93     695      15     372      3.32 (  1.90-   5.81)
 MANFRE 3   m   0         32      79       3      40      5.40 (  1.56-  18.72)
 MANFRE 6   f   0         48     108      22     149      3.01 (  1.72-   5.28)
 Subtotal MANFRE                                          3.32 (  1.99-   5.55)
 MELLST 3   m   0        125     559      15     196      2.92 (  1.67-   5.11)
 MENEZ1 3   b   0        105     484      29     435      3.25 (  2.11-   5.01)
 MEREN  6   b   4          -       -       -       -      1.75 (  1.39-   2.22)
 MILLER 3   m   1         97       -      10       -      5.24 (  2.70-  10.17)
 MILLER 6   f   1         35       -       2       -     20.27 (  4.89-  84.04)
 Subtotal MILLER                                          6.67 (  3.66-  12.17)
 MILNE  3   m   0         65     105       3      40      8.25 (  2.45-  27.77)
 MILNE  9   f   0         13      57      12     186      3.54 (  1.53-   8.18)
 Subtotal MILNE                                           4.65 (  2.33-   9.27)
 MOLLER 3   b   0         13       9       2      11      7.94 (  1.41-  44.80)
 MUELLE 33  m   0         47     175       2      57      7.65 (  1.80-  32.51)
 MUELLE 49  f   0         31     126       3     168     13.78 (  4.12-  46.08)
 Subtotal MUELLE                                         10.82 (  4.28-  27.34)
 NEJJAR 1   m   0        147     553      42     229      1.45 (  1.00-   2.11)
 NEJJAR 2   f   0         17     122      98    1115      1.59 (  0.92-   2.74)
 Subtotal NEJJAR                                          1.49 (  1.09-   2.03)
 OGILVI 5   m   0        227     163      11      29      3.67 (  1.78-   7.56)
 OGILVI 14  f   0        106      86      86     160      2.29 (  1.56-   3.38)
 Subtotal OGILVI                                          2.55 (  1.81-   3.58)
 OSWAL2 1   m   0        421    2196      96     889      1.78 (  1.40-   2.25)
 OSWAL2 3   f   0        149     821     116    1156      1.81 (  1.40-   2.34)
 Subtotal OSWAL2                                          1.79 (  1.50-   2.13)
 PANDEY 3   m   0        238     914       4     219     14.26 (  5.25-  38.72)
 PANDEY 9   f   0        208     718      66     459      2.01 (  1.49-   2.72)
 Subtotal PANDEY                                          2.37 (  1.78-   3.16)
*PELKON 6   m   1          -       -       -       -      1.76 (  1.32-   2.36)
 PEREZP 5   f   0         27      47      77     145      1.08 (  0.63-   1.87)
 REID   3   m   0        248   15002      16    4127      4.26 (  2.57-   7.08)
 REID   19  f   0         80    8185      37   10430      2.76 (  1.86-   4.07)
 Subtotal REID                                            3.24 (  2.38-   4.42)
  ________________________________________________________________________________________________________________________
                                            International Evidence on Smoking and COPD, Phase 3, Analysis run on 27-SEP-10

                                                   Table 2 - A - 2 - 5

            IESCOPD - Meta-analysis of ever smoking, cigarettes (or all products if cigarettes not available)
                                                          Any CB
                                                      Least-adjusted


                        Number Exposed  Non-exposed
 REF    NRR SEX ADJ     Case    Cont    Case    Cont      RR        95.00%CI
 SAWICK 5   m   0        286    1272      14     353      5.67 (  3.27-   9.82)
 SAWICK 18  f   0         75     649      46    1660      4.17 (  2.86-   6.09)
 Subtotal SAWICK                                          4.60 (  3.37-   6.28)
 SHIMUR 3   b   0          3       2       3       2      1.00 (  0.08-  12.56)
*SILVA  6   b   6          -       -       -       -      1.76 (  0.99-   3.16)
 SOBRAD 3   b   0        172    1838      20    2005      9.38 (  5.88-  14.97)
 STJERN 3   b   0         15     555       6     873      3.93 (  1.52-  10.20)
*TROISI 3   f   1        545       -     253       -      1.93 (  1.66-   2.24)
 VIEGI1 3   m   0         32    1040       4     504      3.88 (  1.36-  11.02)
 VIEGI1 6   f   0          1     582       7    1119      0.27 (  0.03-   2.24)
 Subtotal VIEGI1                                          2.29 (  0.90-   5.84)
 WAGEN2 3   b   0        246    2762     111    2136      1.71 (  1.36-   2.16)
 WILHEL 3   m   0         18     207       1      87      7.57 (  0.99-  57.56)
 WOODS  3   b   5          -       -       -       -      2.46 (  2.12-   2.87)
 WOOLF  3   f   0         92     225      10     173      7.07 (  3.58-  13.99)
 YAMAGU 9   b   6        334       -     128       -      2.26 (  1.82-   2.81)
 ZOIA   3   b   4          -       -       -       -      1.45 (  0.66-   3.16)
Partial Totals         30073  224872   11966  252171
*prospective study                                        ~ With 0.5 adjustment for zero


 REF    NRR SEX ADJ             Ys       Ws       Qs       Ps
 ALDERS 4   m   0              1.02    21.41     0.42       0.00
 ALDERS 2   f   0              0.99    46.22     0.58       0.00
 Subtotal ALDERS               1.00    67.62     1.00
 ANDER1 32  m   1              1.51     3.29     1.29       0.01
 ANDER1 35  f   1              0.79     7.52     0.06       0.03
 Subtotal ANDER1               1.01    10.81     1.36
 BECK1  3   m   0              1.48     2.49     0.91       0.02
 BECK1  6   f   0              0.98     3.64     0.04       0.06
 Subtotal BECK1                1.19     6.13     0.95
*BECK2  3   m   0              0.66     1.45     0.07       0.42
*BECK2  6   f   0              0.76     1.74     0.03       0.32
 Subtotal BECK2                0.71     3.19     0.09
*BEST   3   m   1              2.44     2.89     7.01       0.00
 BJORNS 3   b   0              0.91   221.58     0.28       0.00
 BROWN  6   m   0              0.72    28.38     0.70       0.00
 CERVER 3   b   3              0.99   452.17     5.91       0.00
 COATES 3   b   0              1.29    17.22     2.88       0.00
 COLLEG 3   m   1              1.20     2.96     0.30       0.04
 COLLEG 18  f   1              0.97    13.25     0.11       0.00
 Subtotal COLLEG               1.01    16.22     0.41
 DEANE  5   m   0              2.01     2.77     3.53       0.00
 DEMARC 17  b   0              1.07    70.23     2.68       0.00
*DOLL1  13  m   1              2.00     2.65     3.34       0.00
*DONTA2 3   m   0              1.10     7.69     0.38       0.00
 DOPICO 3   m   0              0.93     4.58     0.01       0.05
 EHRLIC 3   m   0              1.01    24.38     0.39       0.00
 EHRLIC 9   f   0              0.84    51.66     0.08       0.00
 Subtotal EHRLIC               0.89    76.04     0.47
 ENRIGH 3   m   0              0.71    13.33     0.37       0.01
 ENRIGH 6   f   0              0.48    43.33     6.84       0.00
 Subtotal ENRIGH               0.54    56.66     7.21
 FERRI1 61  m   1              1.17     8.10     0.68       0.00
 FERRI1 91  f   1              0.77    16.83     0.18       0.00
 Subtotal FERRI1               0.90    24.93     0.87
 FINKLE 1   m   0              1.23   473.17    59.67       0.00
 FOXMAN 3   m   1              0.91    29.97     0.03       0.00
 FOXMAN 6   f   1              0.95    38.71     0.18       0.00
 Subtotal FOXMAN               0.93    68.68     0.21
 GOLDBE 3   m   0              0.39    87.57    21.10       0.00
 GOLDBE 9   f   0              1.06    46.65     1.53       0.00
 Subtotal GOLDBE               0.62   134.22    22.64
 HAENSZ 6   m   0              0.83    22.59     0.05       0.00
 HAENSZ 26  f   0              1.07    42.80     1.51       0.00
 Subtotal HAENSZ               0.99    65.39     1.56
 HARDIE 9   m   1              2.39     1.39     3.16       0.00
 HARDIE 12  f   1              1.20     7.02     0.74       0.00
 Subtotal HARDIE               1.40     8.41     3.90
 HARRIS 3   m   0              1.35     3.91     0.88       0.01
 HAWTHO 10  m   0              1.18    44.34     3.99       0.00
  ________________________________________________________________________________________________________________________
                                            International Evidence on Smoking and COPD, Phase 3, Analysis run on 27-SEP-10

                                                   Table 2 - A - 2 - 5

            IESCOPD - Meta-analysis of ever smoking, cigarettes (or all products if cigarettes not available)
                                                          Any CB
                                                      Least-adjusted


 REF    NRR SEX ADJ             Ys       Ws       Qs       Ps
 HAWTHO 17  f   0              1.08    74.63     3.03       0.00
 Subtotal HAWTHO               1.12   118.97     7.02
 HAYES  3   m   0              1.89    11.00    11.30       0.00
 HAYES  6   f   0              1.81    21.04    18.10       0.00
 Subtotal HAYES                1.84    32.04    29.41
 HIGGI2 3   m   0              1.00     0.93     0.01       0.34
 HIGGI2 9   f   0              0.86     3.25     0.00       0.12
 Subtotal HIGGI2               0.89     4.17     0.02
 HIGGI3 3   m   0              0.02     0.43     0.32       0.99
 HIGGI3 9   f   0              2.50     0.41     1.07       0.11
 Subtotal HIGGI3               1.23     0.84     1.38
 HIGGI6 3   m   1              1.52    15.23     6.34       0.00
 HIGGI6 19  f   1              1.10    25.68     1.20       0.00
 Subtotal HIGGI6               1.25    40.91     7.54
 HO     11  b   3              0.84    23.83     0.03       0.00
 HOLLA2 3   m   0              1.80     4.51     3.84       0.00
 HOLLNA 3   m   0              1.71     8.26     5.70       0.00
 HOLLNA 11  f   0              1.52    10.64     4.32       0.00
 Subtotal HOLLNA               1.60    18.90    10.02
 HOUSE  3   m   0              1.46    40.60    13.59       0.00
 HOUSE  9   f   0              1.51    62.28    24.61       0.00
 Subtotal HOUSE                1.49   102.88    38.20
 HUCHON 3   b   0              0.80   119.05     0.73       0.00
 HUHTI1 39  m   0              2.11     6.25     9.41       0.00
 HUHTI1 124 f   0              1.24     9.48     1.23       0.00
 Subtotal HUHTI1               1.58    15.73    10.64
 HUHTI3 19  m   0              1.34    21.74     4.57       0.00
 JINDA2 2   b   0              0.61   103.89     7.80       0.00
 JOSHI  9   m   0              1.84     7.10     6.57       0.00
 JOUSI1 3   m   0              0.89   251.69     0.01       0.00
 JOUSI1 6   f   0              0.51   180.58    25.07       0.00
 Subtotal JOUSI1               0.73   432.27    25.08
*KAHN2  33  m   1              1.27    11.13     1.70       0.00
 KATO   3   m   1              0.29    94.89    33.45       0.01
 KATO   6   f   1              0.65   169.34     8.70       0.00
 Subtotal KATO                 0.52   264.24    42.15
 KIRAZ  3   f   0              0.71     8.49     0.23       0.04
 KOTAN1 3   m   0              0.51    20.75     2.85       0.02
 KOTAN1 6   f   0              0.56    23.62     2.44       0.01
 Subtotal KOTAN1               0.53    44.36     5.29
 KUBIK  3   m   0              1.33   348.61    70.01       0.00
 KUBIK  4   f   0              0.70   205.79     6.24       0.00
 Subtotal KUBIK                1.10   554.40    76.25
 LAMBER 3   m   0              1.01    15.49     0.28       0.00
 LAMBER 19  f   0              0.90    35.86     0.02       0.00
 Subtotal LAMBER               0.94    51.35     0.30
 LANGHA 3   m   0              1.14   164.18    11.30       0.00
 LANGHA 9   f   0              0.60   219.73    17.35       0.00
 Subtotal LANGHA               0.83   383.91    28.66
 LAVECC 3   m   0              0.96   431.98     2.56       0.00
 LAVECC 6   f   0              0.05   212.69   145.71       0.45
 Subtotal LAVECC               0.66   644.67   148.27
 LEBOWI 27  b   2              0.11    15.18     8.90       0.66
 LINDST 17  m   0              0.56   132.00    13.06       0.00
 LINDST 20  f   0              0.35   136.04    37.83       0.00
 Subtotal LINDST               0.46   268.04    50.89
 LUNDB2 3   m   0              1.94     9.40    10.61       0.00
 LUNDB2 9   f   0              1.33    14.49     2.93       0.00
 Subtotal LUNDB2               1.57    23.89    13.54
 MAGNUS 3   m   0              1.20    12.26     1.26       0.00
 MANFRE 3   m   0              1.69     2.49     1.62       0.01
 MANFRE 6   f   0              1.10    12.16     0.60       0.00
 Subtotal MANFRE               1.20    14.64     2.23
 MELLST 3   m   0              1.07    12.26     0.46       0.00
 MENEZ1 3   b   0              1.18    20.67     1.87       0.00
 MEREN  6   b   4              0.56    70.09     7.15       0.00
 MILLER 3   m   1              1.66     8.74     5.28       0.00
 MILLER 6   f   1              3.01     1.90     8.62       0.00
 Subtotal MILLER               1.90    10.64    13.90
 MILNE  3   m   0              2.11     2.61     3.96       0.00
 MILNE  9   f   0              1.26     5.46     0.80       0.00
  ________________________________________________________________________________________________________________________
                                            International Evidence on Smoking and COPD, Phase 3, Analysis run on 27-SEP-10

                                                   Table 2 - A - 2 - 5

            IESCOPD - Meta-analysis of ever smoking, cigarettes (or all products if cigarettes not available)
                                                          Any CB
                                                      Least-adjusted


 REF    NRR SEX ADJ             Ys       Ws       Qs       Ps
 Subtotal MILNE                1.54     8.07     4.76
 MOLLER 3   b   0              2.07     1.28     1.83       0.02
 MUELLE 33  m   0              2.04     1.84     2.46       0.01
 MUELLE 49  f   0              2.62     2.64     8.02       0.00
 Subtotal MUELLE               2.38     4.47    10.47
 NEJJAR 1   m   0              0.37    27.18     7.01       0.05
 NEJJAR 2   f   0              0.46    12.80     2.24       0.10
 Subtotal NEJJAR               0.40    39.98     9.25
 OGILVI 5   m   0              1.30     7.36     1.31       0.00
 OGILVI 14  f   0              0.83    25.68     0.06       0.00
 Subtotal OGILVI               0.93    33.04     1.37
 OSWAL2 1   m   0              0.57    69.58     6.47       0.00
 OSWAL2 3   f   0              0.59    57.42     4.71       0.00
 Subtotal OSWAL2               0.58   127.00    11.18
 PANDEY 3   m   0              2.66     3.85    12.17       0.00
 PANDEY 9   f   0              0.70    42.50     1.35       0.00
 Subtotal PANDEY               0.86    46.35    13.52
*PELKON 6   m   1              0.57    45.52     4.48       0.00
 PEREZP 5   f   0              0.08    12.79     8.19       0.78
 REID   3   m   0              1.45    14.96     4.88       0.00
 REID   19  f   0              1.01    25.16     0.46       0.00
 Subtotal REID                 1.18    40.12     5.34
 SAWICK 5   m   0              1.74    12.73     9.33       0.00
 SAWICK 18  f   0              1.43    26.87     8.10       0.00
 Subtotal SAWICK               1.53    39.60    17.43
 SHIMUR 3   b   0              0.00     0.60     0.46       1.00
*SILVA  6   b   6              0.57    11.41     1.12       0.06
 SOBRAD 3   b   0              2.24    17.59    32.52       0.00
 STJERN 3   b   0              1.37     4.23     1.02       0.00
*TROISI 3   f   1              0.66   171.12     8.39       0.00
 VIEGI1 3   m   0              1.36     3.52     0.80       0.01
 VIEGI1 6   f   0             -1.29     0.87     4.12       0.23
 Subtotal VIEGI1               0.83     4.39     4.91
 WAGEN2 3   b   0              0.54    71.92     8.32       0.00
 WILHEL 3   m   0              2.02     0.93     1.22       0.05
 WOODS  3   b   5              0.90   167.48     0.08       0.00
 WOOLF  3   f   0              1.96     8.26     9.59       0.00
 YAMAGU 9   b   6              0.82    81.45     0.33       0.00
 ZOIA   3   b   4              0.37     6.27     1.61       0.35

                       N      119
                      NS       79


                      Wt  6226.48
                 Het Chi   841.21
                 Het  df      118
                 Het  P       ***
               Fixed  RR     2.41
                     RRl     2.35
                     RRu     2.47
                      P       +++
              Random  RR     2.73
                     RRl     2.52
                     RRu     2.96
                      P       +++
               Asymm  P         *


  ________________________________________________________________________________________________________________________
                                            International Evidence on Smoking and COPD, Phase 3, Analysis run on 27-SEP-10

                                                   Table 2 - A - 2 - 6

            IESCOPD - Meta-analysis of ever smoking, cigarettes (or all products if cigarettes not available)
                                                          Any CB
                                                      Least-adjusted


                       N      119
                      NS       79


                      Wt  6226.48
                 Het Chi   841.21
                 Het  df      118
                 Het  P       ***
               Fixed  RR     2.41
                     RRl     2.35
                     RRu     2.47
                      P       +++
              Random  RR     2.73
                     RRl     2.52
                     RRu     2.96
                      P       +++
               Asymm  P         *

                                   Sex
                             both      male    female     Total


                       N       19        56        44       119
                      NS       19        56        44       119


                      Wt  1476.14   2611.30   2139.04   6226.48
                 Het Chi    85.48    320.67    303.74    841.21
                 Het  df       18        55        43       118
                 Het  P       ***       ***       ***       ***
               Fixed  RR     2.42      2.79      2.00      2.41
                     RRl     2.30      2.69      1.92      2.35
                     RRu     2.55      2.90      2.09      2.47
                      P       +++       +++       +++       +++
              Random  RR     2.41      3.16      2.47      2.73
                     RRl     2.10      2.80      2.17      2.52
                     RRu     2.76      3.56      2.81      2.96
                      P       +++       +++       +++       +++
             Between Chi                                 131.32
             Between  df                                      2
             Between  P                                     ***
             Btwn(F)  P                                     ***


  ________________________________________________________________________________________________________________________
                                            International Evidence on Smoking and COPD, Phase 3, Analysis run on 27-SEP-10

                                                   Table 2 - A - 2 - 7

            IESCOPD - Meta-analysis of ever smoking, cigarettes (or all products if cigarettes not available)
                                                          Any CB
                                 Excluded studies (and stage at which they were excluded)


1       CLARK COTTON  MEYER REMYJA RUTGER SNYDER SOBRAX     SU TAKEMU  WANG4   WEIR WHICKE ZALACA
2      ALESSA  AMIGO ANDER2 ANDER3 AUERBA BEDNAR BROGGE  CHEN1  CHEN2  CHEN3  CHENG CLEMEN  COCCI  DEAN1 DEJONG DETORR
       DICKIN DONTA1 EKBERG ENSTRO FERRI2 FERRI3  FIDAN FORAST FUKUCH GEIJER GODTFR GULSVI HAMMO2 HARIKK HEDMAN HIGGI4
       HOZAWA ITABAS JACOBS JAENDI JOHANN KACHEL KARAKA KATANC KHOURY    KIM KLAYTO KOJIMA KOTAN2 KRZYZA KULLER    LAI
         LAM1   LAM2   LAM3  LANGE    LEE   LIAW LINDBE   LIU1   LIU2 LUNDB1  MADOR MANNI1 MANNI2 MANNI3 MARAN1 MARAN2
       MARCUS MATHES MENEZ2 MENEZ3 MENEZ4 MENEZ5 MENEZ6 MONTNE   NAWA NIEPSU NIHLEN NILSSO  OMORI   PEAT   PETO  PRATT
        PRICE RENWIC RICCIO  RYDER SARGEA SHAHAB   SHIN SICHLE SPEIZE STERLI  STROM SUTINE  TAGER   TANG   THUN   TODD
       TRUPIN TSUSHI TVERDA VESTBO VIEGI2 VIKGRE VINEIS VOLLM1 VOLLM2 VONHER   WALD  WANG2 WATSON  WEISS WILSO1   XIAO
           XU   YUAN ZIELI1 ZIELI2 ZIETKO
3       DOLL2 FLETCH HRUBEC JENSEN OSWAL1 RIMING SCHWAR  SHARP SUADIC    WIG WILSO2
4        BANG  DEAN2 HIRAYA   KAHN LANGE2 TAGER2    WEN WOJTYN
8      CHAPMA HUHTI2 URRUTI


  ________________________________________________________________________________________________________________________
                                            International Evidence on Smoking and COPD, Phase 3, Analysis run on 27-SEP-10

                                                   Table 2 - A - 2 - 8

            IESCOPD - Meta-analysis of ever smoking, cigarettes (or all products if cigarettes not available)
                                                          Any CB
                                             Potentially overlapping studies


     REF| REFGP|PRINC|                     OVERLAP|

  DEMARC DEMARC     1         DEMARC/URRUTI/DEMEER
  ENRIGH ENRIGH     1         ENRIGH/HOZAWA/HARIKK
  DONTA2 JACOBS     2  JACOBS/DONTA1/DONTA2/PELKON
  PELKON JACOBS     2  JACOBS/DONTA1/DONTA2/PELKON
  LUNDB2 LUNDBA     2  LINDBE/LUNDB1/LUNDB2/HEDLUN
  OSWAL2 OSWAL2     1                OSWAL1/OSWAL2
  HIGGI2   PETO     2    PETO/HIGGI1/HIGGI2/HIGGI5
  HAWTHO   TANG     2             TANG/HAWTHO/WALD
  HUHTI1 HUHTI1     1                HUHTI1/HUHTI2
  HARDIE HARDIE     1         HARDIE/JOHANN/BROGGE
  LEBOWI LEBOWI     1                 LEBOWI/SILVA
   SILVA  SILVA     1                 LEBOWI/SILVA
  FERRI1 FERRIS     2         FERRI1/FERRI2/FERRI3
  KOTAN1 KOTAN1     1                KOTAN1/KOTAN2
  LAMBER   TODD     2                  LAMBER/TODD
   BECK1  BECK1     1                  BECK1/BECK2
   BECK2  BECK2     1                  BECK1/BECK2
   HOUSE  HOUSE     1                 HOUSE/CHAPMA
  SAWICK SAWICK     1         SAWICK/KRZYZA/WOJTYN
  HOLLNA HOLLNA     1 GODT/VEST/LANG1+2/SUAD/HOLLN
  HIGGI6 HIGGI4     2                HIGGI4/HIGGI6
   KAHN2   KAHN     2                   KAHN/KAHN2


  ________________________________________________________________________________________________________________________
                                            International Evidence on Smoking and COPD, Phase 3, Analysis run on 27-SEP-10

                                                    Table 2 - A - 3 -

            IESCOPD - Meta-analysis of ever smoking, any product (or cigarettes if all product not available)
                                                  CB based on mortality


This analysis is restricted to results for:
1) Eligible study on database
2) Outcome CB
3) Non-dose-response data
4) Ever smoking
5) CB based on mortality
6) Results complete enough for use in meta-analysis

Within each study, results are then selected (in the following order of preference, within each sex) for:
7) UNEXP   : never any, never cigarettes, other
8) PROD    : any product, cigarettes, cigarettes only
9) For overlapping studies: principal rather than subsidiary studies
and then for single sex results (m, f) in preference to results for both sexes combined (b).

Results adjusted for the most potential confounders are then chosen in Sections -1 to -3
(Sections 4-6 not presented)

Section -7 shows excluded studies, together with the stage (as above) at which no qualifying
results were found.

Section -8 lists the potentially overlapping studies which have been included (1=principal, 2=subsidiary),
and any results which would have been included in preference except that they had data not complete enough
for use in meta-analysis. It also lists their significance (yes/no), if known.


  ________________________________________________________________________________________________________________________
                                            International Evidence on Smoking and COPD, Phase 3, Analysis run on 27-SEP-10

                                                   Table 2 - A - 3 - 1

            IESCOPD - Meta-analysis of ever smoking, any product (or cigarettes if all product not available)
                                                  CB based on mortality
                                                      Most-adjusted


     REF|NRR|SEX|AGEL|AGEH|     REGION|BEGYR|PUBYR|STTYP|ONSET|      DISEAS|ADJ|SMOKSTA|   PRODUCT|    UNEXP|

    BEST   3   m   30   97   Am:Canada  1955  1967    Pr   Inc CB:mortality   1    Ever  Cigs only   Nev any
   DOLL1  13   m   20   99       Eu:UK  1951  1994    Pr   Inc CB:mortality   1    Ever        Any   Nev any
   KAHN2  32   m   31   84      Am:USA  1954  1966    Pr   Inc CB:mortality   1    Ever        Any   Nev any


  ________________________________________________________________________________________________________________________
                                            International Evidence on Smoking and COPD, Phase 3, Analysis run on 27-SEP-10

                                                   Table 2 - A - 3 - 2

            IESCOPD - Meta-analysis of ever smoking, any product (or cigarettes if all product not available)
                                                  CB based on mortality
                                                      Most-adjusted


                        Number Exposed  Non-exposed
 REF    NRR SEX ADJ     Case    Cont    Case    Cont      RR        95.00%CI
*BEST   3   m   1         77       -       3       -     11.44 (  3.61-  36.25)
*DOLL1  13  m   1         95       -       3       -      7.40 (  2.22-  24.69)
*KAHN2  32  m   1         88       -      13       -      2.75 (  1.54-   4.93)
Partial Totals           260       0      19       0
*prospective study

                       N        3
                      NS        3


                      Wt    16.89
                 Het Chi     5.77
                 Het  df        2
                 Het  P       (*)
               Fixed  RR     4.10
                     RRl     2.54
                     RRu     6.60
                      P       +++
              Random  RR     5.49
                     RRl     2.14
                     RRu    14.11
                      P       +++
               Asymm  P      N.S.


  ________________________________________________________________________________________________________________________
                                            International Evidence on Smoking and COPD, Phase 3, Analysis run on 27-SEP-10

                                                   Table 2 - A - 3 - 3

            IESCOPD - Meta-analysis of ever smoking, any product (or cigarettes if all product not available)
                                                  CB based on mortality
                                                      Most-adjusted


                       N        3
                      NS        3


                      Wt    16.89
                 Het Chi     5.77
                 Het  df        2
                 Het  P       (*)
               Fixed  RR     4.10
                     RRl     2.54
                     RRu     6.60
                      P       +++
              Random  RR     5.49
                     RRl     2.14
                     RRu    14.11
                      P       +++
               Asymm  P      N.S.

                                   Sex
                             both      male    female     Total


                       N                  3                   3
                      NS                  3                   3


                      Wt              16.89               16.89
                 Het Chi               5.77                5.77
                 Het  df                  2                   2
                 Het  P                 (*)                 (*)
               Fixed  RR               4.10                4.10
                     RRl               2.54                2.54
                     RRu               6.60                6.60
                      P                 +++                 +++
              Random  RR               5.49                5.49
                     RRl               2.14                2.14
                     RRu              14.11               14.11
                      P                 +++                 +++
             Between Chi
             Between  df
             Between  P                                    N.S.
             Btwn(F)  P                                    N.S.


  ________________________________________________________________________________________________________________________
                                            International Evidence on Smoking and COPD, Phase 3, Analysis run on 27-SEP-10

                                                   Table 2 - A - 3 - 7

            IESCOPD - Meta-analysis of ever smoking, any product (or cigarettes if all product not available)
                                                  CB based on mortality
                                 Excluded studies (and stage at which they were excluded)


1       CLARK COTTON  MEYER REMYJA RUTGER SNYDER SOBRAX     SU TAKEMU  WANG4   WEIR WHICKE ZALACA
2      ALESSA  AMIGO ANDER2 ANDER3 AUERBA BEDNAR BROGGE  CHEN1  CHEN2  CHEN3  CHENG CLEMEN  COCCI  DEAN1 DEJONG DETORR
       DICKIN DONTA1 EKBERG ENSTRO FERRI2 FERRI3  FIDAN FORAST FUKUCH GEIJER GODTFR GULSVI HAMMO2 HARIKK HEDMAN HIGGI4
       HOZAWA ITABAS JACOBS JAENDI JOHANN KACHEL KARAKA KATANC KHOURY    KIM KLAYTO KOJIMA KOTAN2 KRZYZA KULLER    LAI
         LAM1   LAM2   LAM3  LANGE    LEE   LIAW LINDBE   LIU1   LIU2 LUNDB1  MADOR MANNI1 MANNI2 MANNI3 MARAN1 MARAN2
       MARCUS MATHES MENEZ2 MENEZ3 MENEZ4 MENEZ5 MENEZ6 MONTNE   NAWA NIEPSU NIHLEN NILSSO  OMORI   PEAT   PETO  PRATT
        PRICE RENWIC RICCIO  RYDER SARGEA SHAHAB   SHIN SICHLE SPEIZE STERLI  STROM SUTINE  TAGER   TANG   THUN   TODD
       TRUPIN TSUSHI TVERDA VESTBO VIEGI2 VIKGRE VINEIS VOLLM1 VOLLM2 VONHER   WALD  WANG2 WATSON  WEISS WILSO1   XIAO
           XU   YUAN ZIELI1 ZIELI2 ZIETKO
3       DOLL2 FLETCH HRUBEC JENSEN OSWAL1 RIMING SCHWAR  SHARP SUADIC    WIG WILSO2
4        BANG  DEAN2 HIRAYA   KAHN LANGE2 TAGER2    WEN WOJTYN
5      ALDERS ANDER1  BECK1  BECK2 BJORNS  BROWN CERVER CHAPMA COATES COLLEG  DEANE DEMARC DONTA2 DOPICO EHRLIC ENRIGH
       FERRI1 FINKLE FOXMAN GOLDBE HAENSZ HARDIE HARRIS HAWTHO  HAYES HIGGI2 HIGGI3 HIGGI6     HO HOLLA2 HOLLNA  HOUSE
       HUCHON HUHTI1 HUHTI2 HUHTI3 JINDA2  JOSHI JOUSI1   KATO  KIRAZ KOTAN1  KUBIK LAMBER LANGHA LAVECC LEBOWI LINDST
       LUNDB2 MAGNUS MANFRE MELLST MENEZ1  MEREN MILLER  MILNE MOLLER MUELLE NEJJAR OGILVI OSWAL2 PANDEY PELKON PEREZP
         REID SAWICK SHIMUR  SILVA SOBRAD STJERN TROISI URRUTI VIEGI1 WAGEN2 WILHEL  WOODS  WOOLF YAMAGU   ZOIA


  ________________________________________________________________________________________________________________________
                                            International Evidence on Smoking and COPD, Phase 3, Analysis run on 27-SEP-10

                                                   Table 2 - A - 3 - 8

            IESCOPD - Meta-analysis of ever smoking, any product (or cigarettes if all product not available)
                                                  CB based on mortality
                                             Potentially overlapping studies


     REF| REFGP|PRINC|                     OVERLAP|

   KAHN2   KAHN     2                   KAHN/KAHN2


  ________________________________________________________________________________________________________________________
                                            International Evidence on Smoking and COPD, Phase 3, Analysis run on 27-SEP-10

                                                    Table 2 - A - 4 -

            IESCOPD - Meta-analysis of ever smoking, any product (or cigarettes if all product not available)
                                                CB based on symptoms only


This analysis is restricted to results for:
1) Eligible study on database
2) Outcome CB
3) Non-dose-response data
4) Ever smoking
5) CB based on symptoms only
6) Results complete enough for use in meta-analysis

Within each study, results are then selected (in the following order of preference, within each sex) for:
7) UNEXP   : never any, never cigarettes, other
8) PROD    : any product, cigarettes, cigarettes only
9) For overlapping studies: principal rather than subsidiary studies
and then for single sex results (m, f) in preference to results for both sexes combined (b).

Results adjusted for the most potential confounders are then chosen in Sections -1 to -3
and results adjusted for the least confounders in Sections -4 to -6. (Those least adjusted results which
actually differ from the most adjusted are marked 'x' in column X in Section -4)

Section -7 shows excluded studies, together with the stage (as above) at which no qualifying
results were found.

Section -8 lists the potentially overlapping studies which have been included (1=principal, 2=subsidiary),
and any results which would have been included in preference except that they had data not complete enough
for use in meta-analysis. It also lists their significance (yes/no), if known.


  ________________________________________________________________________________________________________________________
                                            International Evidence on Smoking and COPD, Phase 3, Analysis run on 27-SEP-10

                                                   Table 2 - A - 4 - 1

            IESCOPD - Meta-analysis of ever smoking, any product (or cigarettes if all product not available)
                                                CB based on symptoms only
                                                      Most-adjusted


     REF|NRR|SEX|AGEL|AGEH|     REGION|BEGYR|PUBYR|STTYP|ONSET|      DISEAS|ADJ|SMOKSTA|   PRODUCT|    UNEXP|

  ANDER1  32   m   25   74   Am:Canada  1963  1965    CS  Prev  CB:symptoms   1    Ever        Any   Nev any
  ANDER1  35   f   25   74   Am:Canada  1963  1965    CS  Prev  CB:symptoms   1    Ever        Any   Nev any
   BECK1   3   m   11   99      Am:USA  1972  1982    CS  Prev  CB:symptoms   0    Ever       Cigs   Nev any
   BECK1   6   f   11   99      Am:USA  1972  1982    CS  Prev  CB:symptoms   0    Ever       Cigs   Nev any
   BECK2   3   m   11   99      Am:USA  1972  1982    Pr   Inc  CB:symptoms   0    Ever       Cigs   Nev any
   BECK2   6   f   11   99      Am:USA  1972  1982    Pr   Inc  CB:symptoms   0    Ever       Cigs   Nev any
  BJORNS   6   b   20   44    Eu:Scand  1990  1994    CS  Prev  CB:symptoms   4    Ever        Any   Nev any
  CERVER   6   b   20   44     Eu:West  1998  2003    CS  Prev  CB:symptoms   6    Ever       Cigs  Nev cigs
  COATES   3   b   40   64      Am:USA  1962  1965    CS  Prev  CB:symptoms   0    Ever       Cigs  Nev cigs
   DEANE   6   m   40   59      Am:USA  1963  1965    CS  Prev  CB:symptoms   1    Ever        Any   Nev any
  DEMARC  20   b   20   44       Multi  1991  2004    CS  Prev  CB:symptoms   1    Ever       Cigs   Nev any
  DOPICO   3   m   15    *      Am:USA     *  1984    CS  Prev  CB:symptoms   0    Ever       Cigs  Nev cigs
  EHRLIC  15   m   15   99      Africa  1998  2004    CS  Prev  CB:symptoms   6    Ever        Any   Nev any
  EHRLIC  21   f   15   99      Africa  1998  2004    CS  Prev  CB:symptoms   6    Ever        Any   Nev any
  FERRI1  61   m   25   74      Am:USA  1961  1971    CS  Prev  CB:symptoms   1    Ever        Any   Nev any
  FERRI1  82   f   25   74      Am:USA  1961  1971    CS  Prev  CB:symptoms   1    Ever        Any   Nev any
  FINKLE   1   m   18   25      Am:USA  1969  1974    CS  Prev  CB:symptoms   0    Ever        Any   Nev any
  FOXMAN   3   m   20   69      Am:USA     *  1986    CS  Prev  CB:symptoms   1    Ever        Any   Nev any
  FOXMAN   6   f   20   69      Am:USA     *  1986    CS  Prev  CB:symptoms   1    Ever        Any   Nev any
  GOLDBE   6   m   15   99      Am:USA  1970  1974    CS  Prev  CB:symptoms   1    Ever       Cigs  Nev cigs
  GOLDBE  12   f   15   99      Am:USA  1970  1974    CS  Prev  CB:symptoms   1    Ever       Cigs  Nev cigs
  HAENSZ  10   m   35   74    Eu:Scand  1964  1972    CS  Prev  CB:symptoms   1    Ever        Any   Nev any
  HAENSZ  32   f   35   74    Eu:Scand  1964  1972    CS  Prev  CB:symptoms   1    Ever        Any   Nev any
  HARRIS   3   m   15   60      Africa     *  1993    CS  Prev  CB:symptoms   0    Ever        Any   Nev any
  HAWTHO  10   m   45   76       Eu:UK  1965  1978    Pr  Prev  CB:symptoms   0    Ever       Cigs  N/L cigs
  HAWTHO  17   f   45   76       Eu:UK  1965  1978    Pr  Prev  CB:symptoms   0    Ever       Cigs  N/L cigs
   HAYES   9   b   15   99      Am:USA  1970  1974    CS  Prev  CB:symptoms   3    Ever       Cigs  Nev cigs
  HIGGI2   3   m   25   74       Eu:UK  1956  1957    CS  Prev  CB:symptoms   0    Ever       Cigs  Nev cigs
  HIGGI2  15   f   25   74       Eu:UK  1956  1957    CS  Prev  CB:symptoms   1    Ever       Cigs  Nev cigs
  HIGGI3   3   m   55   64       Eu:UK  1956  1958    CS  Prev  CB:symptoms   0    Ever        Any   Nev any
  HIGGI3   9   f   55   64       Eu:UK  1956  1958    CS  Prev  CB:symptoms   0    Ever        Any   Nev any
  HIGGI6   3   m   25   64      Am:USA  1962  1977    CS  Prev  CB:symptoms   1    Ever        Any   Nev any
  HIGGI6  10   f   25   64      Am:USA  1962  1977    CS  Prev  CB:symptoms   1    Ever        Any   Nev any
  HOLLA2   3   m   40   59      Am:USA  1962  1965    CS  Prev  CB:symptoms   0    Ever        Any   Nev any
  HOLLNA   3   m   40   40    Eu:Scand  1976  1983    CS  Prev  CB:symptoms   0    Ever        Any   Nev any
  HOLLNA  11   f   40   40    Eu:Scand  1976  1983    CS  Prev  CB:symptoms   0    Ever        Any   Nev any
   HOUSE   6   m   15   99      Am:USA  1970  1974    CS  Prev  CB:symptoms   1    Ever       Cigs  Nev cigs
   HOUSE  12   f   15   99      Am:USA  1970  1974    CS  Prev  CB:symptoms   1    Ever       Cigs  Nev cigs
  HUCHON   6   b   25   99     Eu:West     *  2002    CS  Prev  CB:symptoms   1    Ever       Cigs  Nev cigs
  HUHTI1  42   m   40   64    Eu:Scand  1961  1965    CS  Prev  CB:symptoms   1    Ever        Any   Nev any
  HUHTI1  91   f   40   64    Eu:Scand  1961  1965    CS  Prev  CB:symptoms   1    Ever        Any   Nev any
  HUHTI3  22   m   25   69    Eu:Scand  1968  1978    CS  Prev  CB:symptoms   1    Ever        Any   Nev any
  JINDA2   3   b   35   99 Asia:MidE/S     *  2006    CS  Prev  CB:symptoms   6    Ever        Any   Nev any
   JOSHI   6   m   17   64 Asia:MidE/S     *  1975    CS  Prev  CB:symptoms   0    Ever        Any   Nev any
  JOUSI1   3   m   25   64    Eu:Scand  1972  1996    CS  Prev  CB:symptoms   0    Ever        Any   Nev any
  JOUSI1   6   f   25   64    Eu:Scand  1972  1996    CS  Prev  CB:symptoms   0    Ever        Any   Nev any
   KUBIK   3   m   15   99     Eu:East  1972  1984    CS  Prev  CB:symptoms   0    Ever        Any   N/L any
   KUBIK   4   f   15   99     Eu:East  1972  1984    CS  Prev  CB:symptoms   0    Ever        Any   N/L any
  LAMBER   6   m   35   69       Eu:UK  1965  1970    CS  Prev  CB:symptoms   1    Ever       Cigs  Nev cigs
  LAMBER  38   f   35   69       Eu:UK  1965  1970    CS  Prev  CB:symptoms   1    Ever        Any   Nev any
  LANGHA   6   m   20   99    Eu:Scand  1995  2000    CS  Prev  CB:symptoms   1    Ever        Any   Nev any
  LANGHA  12   f   20   99    Eu:Scand  1995  2000    CS  Prev  CB:symptoms   1    Ever        Any   Nev any
  LINDST  23   b   20   69    Eu:Scand     *  2001    CS  Prev  CB:symptoms   5    Ever       Cigs  Nev cigs
  MAGNUS   6   m   50   80    Eu:Scand  1993  1999    CS  Prev  CB:symptoms   1    Ever        Any   Nev any
  MANFRE   3   m   20   65   Am:Canada  1978  1989    CS  Prev  CB:symptoms   0    Ever        Any   Nev any
  MANFRE   6   f   20   65   Am:Canada  1978  1989    CS  Prev  CB:symptoms   0    Ever        Any   Nev any
  MENEZ1   6   b   40   99 Am:Sth/Cent  1990  1995    CS  Prev  CB:symptoms   8    Ever       Cigs  Nev cigs
   MEREN   6   b   15   64     Eu:East  1995  2001    CS  Prev  CB:symptoms   4    Ever       Cigs  Nev cigs
   MILNE   3   m   62   90       Eu:UK  1968  1972    CS  Prev  CB:symptoms   0    Ever       Cigs  Nev cigs
   MILNE  15   f   62   90       Eu:UK  1968  1972    CS  Prev  CB:symptoms   0    Ever        Any   Nev any
  MOLLER   3   b   40   72     Eu:West     *  2001    CC  Prev  CB:symptoms   0    Ever       Cigs  Nev cigs
  MUELLE  36   m   20   69      Am:USA  1967  1971    CS  Prev  CB:symptoms   1    Ever        Any   Nev any
  MUELLE  51   f   20   69      Am:USA  1967  1971    CS  Prev  CB:symptoms   1    Ever        Any   Nev any
  NEJJAR   5   b   65   99     Eu:West  1991  1996    CS  Prev  CB:symptoms   6    Ever       Cigs  Nev cigs
  OGILVI   3   m   30   99       Eu:UK  1955  1957    CC  Prev  CB:symptoms   0    Ever        Any   Nev any
  OGILVI   8   f   30   99       Eu:UK  1955  1957    CC  Prev  CB:symptoms   0    Ever        Any   Nev any
  OSWAL2   2   m   15   65       Eu:UK  1954  1955    CS  Prev  CB:symptoms   1    Ever       Cigs  N/L cigs
  OSWAL2   4   f   15   65       Eu:UK  1954  1955    CS  Prev  CB:symptoms   1    Ever       Cigs  N/L cigs
  PANDEY   6   m   20   99 Asia:MidE/S  1979  1984    CS  Prev  CB:symptoms   2    Ever        Any   Nev any
  PANDEY  12   f   20   99 Asia:MidE/S  1979  1984    CS  Prev  CB:symptoms   2    Ever        Any   Nev any
  PEREZP   5   f   40   99 Am:Sth/Cent  1992  1996    CC  Prev  CB:symptoms   0    Ever       Cigs  Nev cigs
    REID   6   m   35   74      Am:USA  1962  1966    CS  Prev  CB:symptoms   1    Ever       Cigs  Nev cigs
  ________________________________________________________________________________________________________________________
                                            International Evidence on Smoking and COPD, Phase 3, Analysis run on 27-SEP-10

                                                   Table 2 - A - 4 - 1

            IESCOPD - Meta-analysis of ever smoking, any product (or cigarettes if all product not available)
                                                CB based on symptoms only
                                                      Most-adjusted


     REF|NRR|SEX|AGEL|AGEH|     REGION|BEGYR|PUBYR|STTYP|ONSET|      DISEAS|ADJ|SMOKSTA|   PRODUCT|    UNEXP|

    REID  22   f   35   74      Am:USA  1962  1966    CS  Prev  CB:symptoms   1    Ever       Cigs  Nev cigs
  SAWICK   5   m   19   70     Eu:East  1968  1972    CS  Prev  CB:symptoms   0    Ever        Any   Nev any
  SAWICK  18   f   19   70     Eu:East  1968  1972    CS  Prev  CB:symptoms   0    Ever        Any   Nev any
  SOBRAD   4   b   40   69     Eu:West  1996  2000    CS  Prev  CB:symptoms   3    Ever        Any   Nev any
  STJERN   3   b   16   72    Eu:Scand  1981  1985    CS  Prev  CB:symptoms   0    Ever        Any   Nev any
  WAGEN2  11   b   21   68     Eu:West  2001  2004    CC  Prev  CB:symptoms   1    Ever       Cigs  Nev cigs
  WILHEL   3   m   54   54    Eu:Scand  1967  1969    CS  Prev  CB:symptoms   0    Ever        Any   Nev any
   WOODS   3   b   20   44      Aus/NZ     *  2000    CS  Prev  CB:symptoms   5    Ever       Cigs  Nev cigs
   WOOLF   3   f   25   54   Am:Canada     *  1974    CS  Prev  CB:symptoms   0    Ever       Cigs  Nev cigs
  YAMAGU   9   b   40   99   Asia:FarE  1986  1988    CS  Prev  CB:symptoms   6    Ever       Cigs  Nev cigs
    ZOIA   3   b   18   69     Eu:West     *  1995    CS  Prev  CB:symptoms   4    Ever        Any   Nev any


  ________________________________________________________________________________________________________________________
                                            International Evidence on Smoking and COPD, Phase 3, Analysis run on 27-SEP-10

                                                   Table 2 - A - 4 - 2

            IESCOPD - Meta-analysis of ever smoking, any product (or cigarettes if all product not available)
                                                CB based on symptoms only
                                                      Most-adjusted


                        Number Exposed  Non-exposed
 REF    NRR SEX ADJ     Case    Cont    Case    Cont      RR        95.00%CI
 ANDER1 32  m   1         50       -       4       -      4.51 (  1.53-  13.29)
 ANDER1 35  f   1         19       -      15       -      2.20 (  1.08-   4.51)
 Subtotal ANDER1                                          2.74 (  1.51-   4.97)
 BECK1  3   m   0         17     243       3     189      4.41 (  1.27-  15.26)
 BECK1  6   f   0         10     198       6     318      2.68 (  0.96-   7.48)
 Subtotal BECK1                                           3.28 (  1.49-   7.24)
*BECK2  3   m   0          5     243       2     189      1.94 (  0.38-   9.91)
*BECK2  6   f   0          4     199       3     318      2.13 (  0.48-   9.42)
 Subtotal BECK2                                           2.04 (  0.68-   6.13)
 BJORNS 6   b   4        836       -     354       -      2.18 (  1.90-   2.50)
 CERVER 6   b   6       1465       -     754       -      2.52 (  2.29-   2.76)
 COATES 3   b   0        135     913      21     515      3.63 (  2.26-   5.82)
 DEANE  6   m   1        104       -       3       -      6.15 (  1.83-  20.63)
 DEMARC 20  b   1        341       -      91       -      2.96 (  2.33-   3.75)
 DOPICO 3   m   0         37     139       6      57      2.53 (  1.01-   6.32)
 EHRLIC 15  m   6        100       -      33       -      1.27 (  0.77-   2.10)
 EHRLIC 21  f   6         88       -     138       -      1.64 (  1.15-   2.34)
 Subtotal EHRLIC                                          1.51 (  1.13-   2.01)
 FERRI1 61  m   1        156       -      10       -      3.22 (  1.62-   6.42)
 FERRI1 82  f   1         44       -      35       -      2.17 (  1.35-   3.51)
 Subtotal FERRI1                                          2.47 (  1.67-   3.65)
 FINKLE 1   m   0       3551   17761     596   10240      3.44 (  3.14-   3.76)
 FOXMAN 3   m   1        219       -      41       -      2.48 (  1.73-   3.54)
 FOXMAN 6   f   1        151       -      59       -      2.58 (  1.88-   3.53)
 Subtotal FOXMAN                                          2.54 (  2.00-   3.21)
 GOLDBE 6   m   1        348       -     148       -      1.48 (  1.20-   1.83)
 GOLDBE 12  f   1        283       -      61       -      2.87 (  2.15-   3.83)
 Subtotal GOLDBE                                          1.86 (  1.57-   2.21)
 HAENSZ 10  m   1        200       -      27       -      2.27 (  1.51-   3.42)
 HAENSZ 32  f   1         79       -      97       -      3.32 (  2.43-   4.53)
 Subtotal HAENSZ                                          2.89 (  2.25-   3.70)
 HARRIS 3   m   0         12     268       6     518      3.87 (  1.43-  10.41)
 HAWTHO 10  m   0        646    8503      49    2097      3.25 (  2.42-   4.36)
 HAWTHO 17  f   0        320    3628     103    3440      2.95 (  2.35-   3.70)
 Subtotal HAWTHO                                          3.06 (  2.55-   3.66)
 HAYES  9   b   3        357       -      37       -      5.70 (  4.08-   7.97)
 HIGGI2 3   m   0         25     248       1      27      2.72 (  0.35-  20.89)
 HIGGI2 15  f   1          8       -       6       -      2.46 (  0.77-   7.83)
 Subtotal HIGGI2                                          2.52 (  0.92-   6.91)
 HIGGI3 3   m   0          6      82       0       6      1.02~(  0.05-  20.27)
 HIGGI3 9   f   0          2      26       0      64     12.17~(  0.57- 262.12)
 Subtotal HIGGI3                                          3.41 (  0.40-  28.99)
 HIGGI6 3   m   1        294       -      17       -      4.59 (  2.78-   7.59)
 HIGGI6 10  f   1         86       -      40       -      2.99 (  2.03-   4.40)
 Subtotal HIGGI6                                          3.51 (  2.58-   4.76)
 HOLLA2 3   m   0        142     394       5      84      6.05 (  2.41-  15.23)
 HOLLNA 3   m   0        139     254      10     101      5.53 (  2.79-  10.93)
 HOLLNA 11  f   0         72     245      14     217      4.56 (  2.50-   8.31)
 Subtotal HOLLNA                                          4.96 (  3.16-   7.78)
 HOUSE  6   m   1        272       -      51       -      4.21 (  3.08-   5.75)
 HOUSE  12  f   1        194       -     103       -      4.47 (  3.48-   5.75)
 Subtotal HOUSE                                           4.37 (  3.59-   5.31)
 HUCHON 6   b   1        404       -     177       -      2.21 (  1.83-   2.66)
 HUHTI1 42  m   1        177       -       7       -      7.05 (  3.17-  15.67)
 HUHTI1 91  f   1         16       -      32       -      3.63 (  1.78-   7.40)
 Subtotal HUHTI1                                          4.87 (  2.86-   8.29)
 HUHTI3 22  m   1        272       -      28       -      2.91 (  1.87-   4.51)
 JINDA2 3   b   6        666       -     784       -      2.45 (  2.16-   2.79)
 JOSHI  6   m   0         50     194       9     220      6.30 (  3.02-  13.14)
 JOUSI1 3   m   0       1741    4824     360    2417      2.42 (  2.14-   2.74)
 JOUSI1 6   f   0        278    1344     940    7540      1.66 (  1.43-   1.92)
 Subtotal JOUSI1                                          2.07 (  1.88-   2.27)
 KUBIK  3   m   0       3253   16409     421    8006      3.77 (  3.39-   4.19)
 KUBIK  4   f   0        298    4760     797   25758      2.02 (  1.76-   2.32)
 Subtotal KUBIK                                           2.99 (  2.75-   3.25)
 LAMBER 6   m   1        257       -      17       -      2.60 (  1.58-   4.30)
 LAMBER 38  f   1        118       -      54       -      2.88 (  2.04-   4.06)
 Subtotal LAMBER                                          2.79 (  2.10-   3.70)
 LANGHA 6   m   1        985       -     203       -      2.66 (  2.28-   3.12)
 LANGHA 12  f   1        635       -     351       -      1.95 (  1.70-   2.24)
 Subtotal LANGHA                                          2.23 (  2.01-   2.48)
  ________________________________________________________________________________________________________________________
                                            International Evidence on Smoking and COPD, Phase 3, Analysis run on 27-SEP-10

                                                   Table 2 - A - 4 - 2

            IESCOPD - Meta-analysis of ever smoking, any product (or cigarettes if all product not available)
                                                CB based on symptoms only
                                                      Most-adjusted


                        Number Exposed  Non-exposed
 REF    NRR SEX ADJ     Case    Cont    Case    Cont      RR        95.00%CI
 LINDST 23  b   5        763       -     494       -      1.52 (  1.34-   1.72)
 MAGNUS 6   m   1         93       -      15       -      4.18 (  2.36-   7.38)
 MANFRE 3   m   0         32      79       3      40      5.40 (  1.56-  18.72)
 MANFRE 6   f   0         48     108      22     149      3.01 (  1.72-   5.28)
 Subtotal MANFRE                                          3.32 (  1.99-   5.55)
 MENEZ1 6   b   8        105       -      29       -      2.93 (  1.79-   4.80)
 MEREN  6   b   4          -       -       -       -      1.75 (  1.39-   2.22)
 MILNE  3   m   0         65     105       3      40      8.25 (  2.45-  27.77)
 MILNE  15  f   0         13      57      12     186      3.54 (  1.53-   8.18)
 Subtotal MILNE                                           4.65 (  2.33-   9.27)
 MOLLER 3   b   0         13       9       2      11      7.94 (  1.41-  44.80)
 MUELLE 36  m   1         47       -       2       -      7.08 (  1.66-  30.19)
 MUELLE 51  f   1         31       -       3       -     10.49 (  2.74-  40.22)
 Subtotal MUELLE                                          8.75 (  3.27-  23.44)
 NEJJAR 5   b   6        164       -     140       -      1.56 (  1.15-   2.11)
 OGILVI 3   m   0        261     210      11      29      3.28 (  1.60-   6.71)
 OGILVI 8   f   0        106      86      86     160      2.29 (  1.56-   3.38)
 Subtotal OGILVI                                          2.49 (  1.77-   3.49)
 OSWAL2 2   m   1        421       -      96       -      1.41 (  1.11-   1.79)
 OSWAL2 4   f   1        149       -     116       -      1.69 (  1.30-   2.20)
 Subtotal OSWAL2                                          1.53 (  1.28-   1.83)
 PANDEY 6   m   2        238       -       4       -      5.37 (  2.52-  11.45)
 PANDEY 12  f   2        208       -      66       -      1.76 (  1.30-   2.38)
 Subtotal PANDEY                                          2.05 (  1.55-   2.72)
 PEREZP 5   f   0         27      47      77     145      1.08 (  0.63-   1.87)
 REID   6   m   1        248       -      16       -      4.61 (  2.77-   7.70)
 REID   22  f   1         80       -      37       -      3.32 (  2.17-   5.07)
 Subtotal REID                                            3.80 (  2.74-   5.26)
 SAWICK 5   m   0        286    1272      14     353      5.67 (  3.27-   9.82)
 SAWICK 18  f   0         75     649      46    1660      4.17 (  2.86-   6.09)
 Subtotal SAWICK                                          4.60 (  3.37-   6.28)
 SOBRAD 4   b   3        167       -      20       -      6.68 (  3.83-  11.63)
 STJERN 3   b   0         15     555       6     873      3.93 (  1.52-  10.20)
 WAGEN2 11  b   1        246       -     111       -      1.72 (  1.36-   2.17)
 WILHEL 3   m   0         18     207       1      87      7.57 (  0.99-  57.56)
 WOODS  3   b   5          -       -       -       -      2.46 (  2.12-   2.87)
 WOOLF  3   f   0         92     225      10     173      7.07 (  3.58-  13.99)
 YAMAGU 9   b   6        334       -     128       -      2.26 (  1.82-   2.81)
 ZOIA   3   b   4          -       -       -       -      1.45 (  0.66-   3.16)
Partial Totals         24312   64484    8699   66227
*prospective study                                        ~ With 0.5 adjustment for zero

                       N       83
                      NS       56


                      Wt  4727.25
                 Het Chi   515.30
                 Het  df       82
                 Het  P       ***
               Fixed  RR     2.51
                     RRl     2.44
                     RRu     2.58
                      P       +++
              Random  RR     2.78
                     RRl     2.55
                     RRu     3.03
                      P       +++
               Asymm  P         *


  ________________________________________________________________________________________________________________________
                                            International Evidence on Smoking and COPD, Phase 3, Analysis run on 27-SEP-10

                                                   Table 2 - A - 4 - 3

            IESCOPD - Meta-analysis of ever smoking, any product (or cigarettes if all product not available)
                                                CB based on symptoms only
                                                      Most-adjusted


                       N       83
                      NS       56


                      Wt  4727.25
                 Het Chi   515.30
                 Het  df       82
                 Het  P       ***
               Fixed  RR     2.51
                     RRl     2.44
                     RRu     2.58
                      P       +++
              Random  RR     2.78
                     RRl     2.55
                     RRu     3.03
                      P       +++
               Asymm  P         *

                                   Sex
                             both      male    female     Total


                       N       18        36        29        83
                      NS       18        36        29        83


                      Wt  1826.41   1700.58   1200.26   4727.25
                 Het Chi   121.01    178.34    127.51    515.30
                 Het  df       17        35        28        82
                 Het  P       ***       ***       ***       ***
               Fixed  RR     2.26      3.01      2.28      2.51
                     RRl     2.16      2.87      2.15      2.44
                     RRu     2.36      3.16      2.41      2.58
                      P       +++       +++       +++       +++
              Random  RR     2.44      3.24      2.62      2.78
                     RRl     2.12      2.80      2.27      2.55
                     RRu     2.81      3.75      3.02      3.03
                      P       +++       +++       +++       +++
             Between Chi                                  88.45
             Between  df                                      2
             Between  P                                     ***
             Btwn(F)  P                                     ***

                                        Continent
                            NAmer    Europe      Asia  oth/mult     Total


                       N       29        42         5         7        83
                      NS       18        28         4         6        56


                      Wt   986.66   3055.20    371.86    313.54   4727.25
                 Het Chi   101.61    292.36     15.40     23.11    515.30
                 Het  df       28        41         4         6        82
                 Het  P       ***       ***        **       ***       ***
               Fixed  RR     3.25      2.34      2.39      2.34      2.51
                     RRl     3.05      2.26      2.16      2.09      2.44
                     RRu     3.46      2.42      2.65      2.61      2.58
                      P       +++       +++       +++       +++       +++
              Random  RR     3.42      2.61      2.61      2.09      2.78
                     RRl     2.91      2.32      1.99      1.59      2.55
                     RRu     4.01      2.93      3.41      2.74      3.03
                      P       +++       +++       +++       +++       +++
             Between Chi                                            82.83
             Between  df                                                3
             Between  P                                               ***
             Btwn(F)  P                                                **


  ________________________________________________________________________________________________________________________
                                            International Evidence on Smoking and COPD, Phase 3, Analysis run on 27-SEP-10

                                                   Table 2 - A - 4 - 3

            IESCOPD - Meta-analysis of ever smoking, any product (or cigarettes if all product not available)
                                                CB based on symptoms only
                                                      Most-adjusted
                        National cigarette tobacco type (excluding mixed/unkown)
                          blended  virginia     Total


                       N       54        27        81
                      NS       38        16        54


                      Wt  3701.34    876.61   4577.95
                 Het Chi   428.30     81.13    512.56
                 Het  df       53        26        80
                 Het  P       ***       ***       ***
               Fixed  RR     2.54      2.38      2.51
                     RRl     2.46      2.22      2.44
                     RRu     2.62      2.54      2.58
                      P       +++       +++       +++
              Random  RR     2.88      2.58      2.79
                     RRl     2.58      2.22      2.55
                     RRu     3.21      3.00      3.06
                      P       +++       +++       +++
             Between Chi                         3.13
             Between  df                            1
             Between  P                           (*)
             Btwn(F)  P                          N.S.

                                        Start year of study
                            <1970   1970-79   1980-89   1990-99     2000+   unknown     Total


                       N       36        19         2        13         1        12        83
                      NS       21        10         2        11         1        11        56


                      Wt  1086.10   1345.26     85.68   1281.30     70.38    858.53   4727.25
                 Het Chi   105.18    194.95      1.23     64.34      0.00     63.21    515.30
                 Het  df       35        18         1        12         0        11        82
                 Het  P       ***       ***      N.S.       ***      N.S.       ***       ***
               Fixed  RR     3.06      2.64      2.32      2.29      1.72      2.15      2.51
                     RRl     2.88      2.50      1.88      2.17      1.36      2.02      2.44
                     RRu     3.24      2.78      2.87      2.42      2.17      2.30      2.58
                      P       +++       +++       +++       +++       +++       +++       +++
              Random  RR     3.17      3.02      2.44      2.23      1.72      2.56      2.78
                     RRl     2.76      2.43      1.68      1.91      1.36      2.08      2.55
                     RRu     3.63      3.74      3.53      2.59      2.17      3.16      3.03
                      P       +++       +++       +++       +++       +++       +++       +++
             Between Chi                                                                86.37
             Between  df                                                                    5
             Between  P                                                                   ***
             Btwn(F)  P                                                                     *

                                Publication year
                            <1980   1980-89   1990-99     2000+     Total


                       N       43        17         9        14        83
                      NS       26        10         8        12        56


                      Wt  1368.47    804.92    728.58   1825.28   4727.25
                 Het Chi   186.43     77.59     34.39     93.83    515.30
                 Het  df       42        16         8        13        82
                 Het  P       ***       ***       ***       ***       ***
               Fixed  RR     3.06      2.84      2.08      2.20      2.51
                     RRl     2.91      2.65      1.94      2.11      2.44
                     RRu     3.23      3.04      2.24      2.31      2.58
                      P       +++       +++       +++       +++       +++
              Random  RR     3.34      2.91      2.06      2.20      2.78
                     RRl     2.91      2.35      1.70      1.92      2.55
                     RRu     3.84      3.60      2.50      2.53      3.03
                      P       +++       +++       +++       +++       +++
             Between Chi                                           123.06
             Between  df                                                3
             Between  P                                               ***
             Btwn(F)  P                                               ***
  ________________________________________________________________________________________________________________________
                                            International Evidence on Smoking and COPD, Phase 3, Analysis run on 27-SEP-10

                                                   Table 2 - A - 4 - 3

            IESCOPD - Meta-analysis of ever smoking, any product (or cigarettes if all product not available)
                                                CB based on symptoms only
                                                      Most-adjusted
                               Study type
                               CC        Pr        CS     Total


                       N        5         4        74        83
                      NS        4         2        50        56


                      Wt   117.60    122.16   4487.49   4727.25
                 Het Chi    10.40      0.78    488.69    515.30
                 Het  df        4         3        73        82
                 Het  P         *      N.S.       ***       ***
               Fixed  RR     1.84      3.02      2.52      2.51
                     RRl     1.54      2.53      2.44      2.44
                     RRu     2.21      3.61      2.59      2.58
                      P       +++       +++       +++       +++
              Random  RR     1.99      3.02      2.83      2.78
                     RRl     1.37      2.53      2.59      2.55
                     RRu     2.88      3.61      3.11      3.03
                      P       +++       +++       +++       +++
             Between Chi                                  15.43
             Between  df                                      2
             Between  P                                     ***
             Btwn(F)  P                                    N.S.

                                    Lowest age in RR
                        <25/unlim     25-39       40+   unknown     Total


                       N       40        22        21                  83
                      NS       27        13        16                  56


                      Wt  3299.52   1064.77    362.96             4727.25
                 Het Chi   380.05     60.47     66.37              515.30
                 Het  df       39        21        20                  82
                 Het  P       ***       ***       ***                 ***
               Fixed  RR     2.51      2.40      2.86                2.51
                     RRl     2.43      2.26      2.58                2.44
                     RRu     2.60      2.54      3.17                2.58
                      P       +++       +++       +++                 +++
              Random  RR     2.60      2.72      3.48                2.78
                     RRl     2.29      2.39      2.77                2.55
                     RRu     2.95      3.09      4.37                3.03
                      P       +++       +++       +++                 +++
             Between Chi                                             8.42
             Between  df                                                2
             Between  P                                                 *
             Btwn(F)  P                                              N.S.

                                         Highest age in RR
                              <65     65-74     75-84 85+/unlim   unknown     Total


                       N       23        29         3        27         1        83
                      NS       18        18         2        17         1        56


                      Wt  1974.60    796.61    130.79   1820.67      4.58   4727.25
                 Het Chi   137.88    134.53      1.33    208.78      0.00    515.30
                 Het  df       22        28         2        26         0        82
                 Het  P       ***       ***      N.S.       ***      N.S.       ***
               Fixed  RR     2.62      2.12      3.14      2.53      2.53      2.51
                     RRl     2.51      1.98      2.65      2.42      1.01      2.44
                     RRu     2.74      2.28      3.73      2.65      6.32      2.58
                      P       +++       +++       +++       +++         +       +++
              Random  RR     3.07      2.82      3.14      2.51      2.53      2.78
                     RRl     2.64      2.35      2.65      2.15      1.01      2.55
                     RRu     3.56      3.39      3.73      2.93      6.32      3.03
                      P       +++       +++       +++       +++         +       +++
             Between Chi                                                      32.78
             Between  df                                                          4
             Between  P                                                         ***
             Btwn(F)  P                                                        N.S.
  ________________________________________________________________________________________________________________________
                                            International Evidence on Smoking and COPD, Phase 3, Analysis run on 27-SEP-10

                                                   Table 2 - A - 4 - 3

            IESCOPD - Meta-analysis of ever smoking, any product (or cigarettes if all product not available)
                                                CB based on symptoms only
                                                      Most-adjusted
                           Study weakness
                              Yes        No     Total


                       N        1        82        83
                      NS        1        55        56


                      Wt     1.28   4725.97   4727.25
                 Het Chi     0.00    513.60    515.30
                 Het  df        0        81        82
                 Het  P      N.S.       ***       ***
               Fixed  RR     7.94      2.51      2.51
                     RRl     1.41      2.44      2.44
                     RRu    44.80      2.58      2.58
                      P         +       +++       +++
              Random  RR     7.94      2.77      2.78
                     RRl     1.41      2.54      2.55
                     RRu    44.80      3.03      3.03
                      P         +       +++       +++
             Between Chi                         1.71
             Between  df                            1
             Between  P                          N.S.
             Btwn(F)  P                          N.S.

                           CB subtype
                             mort     sympt     other     Total


                       N                 83                  83
                      NS                 56                  56


                      Wt            4727.25             4727.25
                 Het Chi             515.30              515.30
                 Het  df                 82                  82
                 Het  P                 ***                 ***
               Fixed  RR               2.51                2.51
                     RRl               2.44                2.44
                     RRu               2.58                2.58
                      P                 +++                 +++
              Random  RR               2.78                2.78
                     RRl               2.55                2.55
                     RRu               3.03                3.03
                      P                 +++                 +++
             Between Chi
             Between  df
             Between  P                                    N.S.
             Btwn(F)  P                                    N.S.

                           Asthma analysis type (CB)
                        inc-irres  excl-all excl-cntr     Total


                       N       78         4         1        83
                      NS       51         4         1        56


                      Wt  4630.63     95.34      1.28   4727.25
                 Het Chi   484.76     20.99      0.00    515.30
                 Het  df       77         3         0        82
                 Het  P       ***       ***      N.S.       ***
               Fixed  RR     2.52      1.89      7.94      2.51
                     RRl     2.45      1.55      1.41      2.44
                     RRu     2.60      2.31     44.80      2.58
                      P       +++       +++         +       +++
              Random  RR     2.80      2.51      7.94      2.78
                     RRl     2.56      1.22      1.41      2.55
                     RRu     3.05      5.18     44.80      3.03
                      P       +++         +         +       +++
             Between Chi                                   9.55
             Between  df                                      2
             Between  P                                      **
             Btwn(F)  P                                    N.S.
  ________________________________________________________________________________________________________________________
                                            International Evidence on Smoking and COPD, Phase 3, Analysis run on 27-SEP-10

                                                   Table 2 - A - 4 - 3

            IESCOPD - Meta-analysis of ever smoking, any product (or cigarettes if all product not available)
                                                CB based on symptoms only
                                                      Most-adjusted
                                   Number of CB cases
                             1-50    51-100   101-200      201+     Total


                       N       14         7        10        52        83
                      NS       10         4         9        33        56


                      Wt    35.14     29.93    100.11   4562.06   4727.25
                 Het Chi     8.18      7.83     30.71    444.91    515.30
                 Het  df       13         6         9        51        82
                 Het  P      N.S.      N.S.       ***       ***       ***
               Fixed  RR     2.81      4.49      3.57      2.48      2.51
                     RRl     2.02      3.14      2.93      2.41      2.44
                     RRu     3.91      6.42      4.34      2.55      2.58
                      P       +++       +++       +++       +++       +++
              Random  RR     2.81      4.67      3.86      2.61      2.78
                     RRl     2.02      3.06      2.64      2.37      2.55
                     RRu     3.91      7.12      5.65      2.87      3.03
                      P       +++       +++       +++       +++       +++
             Between Chi                                            23.66
             Between  df                                                3
             Between  P                                               ***
             Btwn(F)  P                                              N.S.

                            Analysis type
                         prevlnce     onset     Total


                       N       81         2        83
                      NS       55         1        56


                      Wt  4724.06      3.19   4727.25
                 Het Chi   515.16      0.01    515.30
                 Het  df       80         1        82
                 Het  P       ***      N.S.       ***
               Fixed  RR     2.51      2.04      2.51
                     RRl     2.44      0.68      2.44
                     RRu     2.58      6.13      2.58
                      P       +++      N.S.       +++
              Random  RR     2.79      2.04      2.78
                     RRl     2.55      0.68      2.55
                     RRu     3.04      6.13      3.03
                      P       +++      N.S.       +++
             Between Chi                         0.13
             Between  df                            1
             Between  P                          N.S.
             Btwn(F)  P                          N.S.

                             Smoking product
                              any      cigs  cigsonly     Total


                       N       49        34                  83
                      NS       32        26                  58


                      Wt  2795.18   1932.07             4727.25
                 Het Chi   260.53    228.45              515.30
                 Het  df       48        33                  82
                 Het  P       ***       ***                 ***
               Fixed  RR     2.67      2.29                2.51
                     RRl     2.57      2.19                2.44
                     RRu     2.77      2.40                2.58
                      P       +++       +++                 +++
              Random  RR     2.94      2.57                2.78
                     RRl     2.64      2.24                2.55
                     RRu     3.29      2.96                3.03
                      P       +++       +++                 +++
             Between Chi                                  26.31
             Between  df                                      1
             Between  P                                     ***
             Btwn(F)  P                                       *
  ________________________________________________________________________________________________________________________
                                            International Evidence on Smoking and COPD, Phase 3, Analysis run on 27-SEP-10

                                                   Table 2 - A - 4 - 3

            IESCOPD - Meta-analysis of ever smoking, any product (or cigarettes if all product not available)
                                                CB based on symptoms only
                                                      Most-adjusted
                                     Unexposed group
                          nev any   nev cig  nev+ any  nev+ cig     Total


                       N       52        25         2         4        83
                      NS       34        21         1         2        58


                      Wt  2317.95   1613.12    554.40    241.78   4727.25
                 Het Chi   203.46    191.66     50.12     30.18    515.30
                 Het  df       51        24         1         3        82
                 Het  P       ***       ***       ***       ***       ***
               Fixed  RR     2.61      2.29      2.99      2.15      2.51
                     RRl     2.50      2.18      2.75      1.90      2.44
                     RRu     2.71      2.40      3.25      2.44      2.58
                      P       +++       +++       +++       +++       +++
              Random  RR     2.93      2.64      2.77      2.18      2.78
                     RRl     2.63      2.24      1.50      1.46      2.55
                     RRu     3.26      3.11      5.09      3.26      3.03
                      P       +++       +++        ++       +++       +++
             Between Chi                                            39.89
             Between  df                                                3
             Between  P                                               ***
             Btwn(F)  P                                               (*)

                        Unexposed group (combining nev+ with main levels)
                          nev any   nev cig     Total


                       N       54        29        83
                      NS       35        23        58


                      Wt  2872.35   1854.90   4727.25
                 Het Chi   262.10    222.65    515.30
                 Het  df       53        28        82
                 Het  P       ***       ***       ***
               Fixed  RR     2.68      2.27      2.51
                     RRl     2.58      2.17      2.44
                     RRu     2.78      2.38      2.58
                      P       +++       +++       +++
              Random  RR     2.93      2.55      2.78
                     RRl     2.64      2.20      2.55
                     RRu     3.26      2.96      3.03
                      P       +++       +++       +++
             Between Chi                        30.55
             Between  df                            1
             Between  P                           ***
             Btwn(F)  P                             *

                        Smoking results reported in study (CB)
                             Ever   Current      Both     Total


                       N        7                  76        83
                      NS        5                  51        56


                      Wt  1397.75             3329.50   4727.25
                 Het Chi   133.73              352.57    515.30
                 Het  df        6                  75        82
                 Het  P       ***                 ***       ***
               Fixed  RR     2.83                2.39      2.51
                     RRl     2.69                2.31      2.44
                     RRu     2.98                2.47      2.58
                      P       +++                 +++       +++
              Random  RR     2.18                2.86      2.78
                     RRl     1.66                2.62      2.55
                     RRu     2.85                3.14      3.03
                      P       +++                 +++       +++
             Between Chi                                  29.01
             Between  df                                      1
             Between  P                                     ***
             Btwn(F)  P                                       *
  ________________________________________________________________________________________________________________________
                                            International Evidence on Smoking and COPD, Phase 3, Analysis run on 27-SEP-10

                                                   Table 2 - A - 4 - 3

            IESCOPD - Meta-analysis of ever smoking, any product (or cigarettes if all product not available)
                                                CB based on symptoms only
                                                      Most-adjusted
                        Number of adjustment variables
                                0         1        2+     Total


                       N       34        33        16        83
                      NS       23        20        14        57


                      Wt  1769.06   1308.16   1650.02   4727.25
                 Het Chi   181.41    151.12    118.73    515.30
                 Het  df       33        32        15        82
                 Het  P       ***       ***       ***       ***
               Fixed  RR     2.90      2.42      2.21      2.51
                     RRl     2.76      2.29      2.11      2.44
                     RRu     3.03      2.56      2.32      2.58
                      P       +++       +++       +++       +++
              Random  RR     3.25      2.78      2.28      2.78
                     RRl     2.78      2.43      1.95      2.55
                     RRu     3.81      3.18      2.67      3.03
                      P       +++       +++       +++       +++
             Between Chi                                  64.03
             Between  df                                      2
             Between  P                                     ***
             Btwn(F)  P                                      **

                        RR adjusted for sex (combined sex RR only)
                              Yes        No     Total


                       N       12         6        18
                      NS       12         6        18


                      Wt  1555.59    270.82   1826.41
                 Het Chi   103.51     17.14    121.01
                 Het  df       11         5        17
                 Het  P       ***        **       ***
               Fixed  RR     2.24      2.33      2.26
                     RRl     2.14      2.07      2.16
                     RRu     2.36      2.63      2.36
                      P       +++       +++       +++
              Random  RR     2.39      2.58      2.44
                     RRl     2.01      1.96      2.12
                     RRu     2.84      3.38      2.81
                      P       +++       +++       +++
             Between Chi                         0.35
             Between  df                            1
             Between  P                          N.S.
             Btwn(F)  P                          N.S.

                        RR adjusted for age


                       N       43        40        83
                      NS       29        28        57


                      Wt  2168.82   2558.43   4727.25
                 Het Chi   216.31    231.31    515.30
                 Het  df       42        39        82
                 Het  P       ***       ***       ***
               Fixed  RR     2.20      2.80      2.51
                     RRl     2.11      2.70      2.44
                     RRu     2.30      2.91      2.58
                      P       +++       +++       +++
              Random  RR     2.53      3.11      2.78
                     RRl     2.27      2.74      2.55
                     RRu     2.83      3.54      3.03
                      P       +++       +++       +++
             Between Chi                        67.68
             Between  df                            1
             Between  P                           ***
             Btwn(F)  P                           ***

  ________________________________________________________________________________________________________________________
                                            International Evidence on Smoking and COPD, Phase 3, Analysis run on 27-SEP-10

                                                   Table 2 - A - 4 - 3

            IESCOPD - Meta-analysis of ever smoking, any product (or cigarettes if all product not available)
                                                CB based on symptoms only
                                                      Most-adjusted
                        RR adjusted for factor other than sex, age
                              Yes        No     Total


                       N       21        62        83
                      NS       18        38        56


                      Wt  1998.47   2728.78   4727.25
                 Het Chi   172.88    314.13    515.30
                 Het  df       20        61        82
                 Het  P       ***       ***       ***
               Fixed  RR     2.29      2.68      2.51
                     RRl     2.19      2.58      2.44
                     RRu     2.40      2.78      2.58
                      P       +++       +++       +++
              Random  RR     2.43      2.99      2.78
                     RRl     2.11      2.68      2.55
                     RRu     2.80      3.33      3.03
                      P       +++       +++       +++
             Between Chi                        28.29
             Between  df                            1
             Between  P                           ***
             Btwn(F)  P                             *

                        Derivation of RR/CI
                         Orig/2x2     Other     Total


                       N        6        77        83
                      NS        3        53        56


                      Wt   625.07   4102.18   4727.25
                 Het Chi    55.13    437.32    515.30
                 Het  df        5        76        82
                 Het  P       ***       ***       ***
               Fixed  RR     3.00      2.44      2.51
                     RRl     2.77      2.37      2.44
                     RRu     3.24      2.52      2.58
                      P       +++       +++       +++
              Random  RR     3.04      2.76      2.78
                     RRl     2.13      2.52      2.55
                     RRu     4.34      3.02      3.03
                      P       +++       +++       +++
             Between Chi                        22.85
             Between  df                            1
             Between  P                           ***
             Btwn(F)  P                           (*)


  ________________________________________________________________________________________________________________________
                                            International Evidence on Smoking and COPD, Phase 3, Analysis run on 27-SEP-10

                                                   Table 2 - A - 4 - 4

            IESCOPD - Meta-analysis of ever smoking, any product (or cigarettes if all product not available)
                                                CB based on symptoms only
                                                      Least-adjusted


     REF|NRR|X|SEX|AGEL|AGEH|     REGION|BEGYR|PUBYR|STTYP|ONSET|      DISEAS|ADJ|SMOKSTA|   PRODUCT|    UNEXP|

  ANDER1  32     m   25   74   Am:Canada  1963  1965    CS  Prev  CB:symptoms   1    Ever        Any   Nev any
  ANDER1  35     f   25   74   Am:Canada  1963  1965    CS  Prev  CB:symptoms   1    Ever        Any   Nev any
   BECK1   3     m   11   99      Am:USA  1972  1982    CS  Prev  CB:symptoms   0    Ever       Cigs   Nev any
   BECK1   6     f   11   99      Am:USA  1972  1982    CS  Prev  CB:symptoms   0    Ever       Cigs   Nev any
   BECK2   3     m   11   99      Am:USA  1972  1982    Pr   Inc  CB:symptoms   0    Ever       Cigs   Nev any
   BECK2   6     f   11   99      Am:USA  1972  1982    Pr   Inc  CB:symptoms   0    Ever       Cigs   Nev any
  BJORNS   3 x   b   20   44    Eu:Scand  1990  1994    CS  Prev  CB:symptoms   0    Ever        Any   Nev any
  CERVER   3 x   b   20   44     Eu:West  1998  2003    CS  Prev  CB:symptoms   3    Ever       Cigs  Nev cigs
  COATES   3     b   40   64      Am:USA  1962  1965    CS  Prev  CB:symptoms   0    Ever       Cigs  Nev cigs
   DEANE   5 x   m   40   59      Am:USA  1963  1965    CS  Prev  CB:symptoms   0    Ever        Any   Nev any
  DEMARC  17 x   b   20   44       Multi  1991  2004    CS  Prev  CB:symptoms   0    Ever       Cigs   Nev any
  DOPICO   3     m   15    *      Am:USA     *  1984    CS  Prev  CB:symptoms   0    Ever       Cigs  Nev cigs
  EHRLIC   3 x   m   15   99      Africa  1998  2004    CS  Prev  CB:symptoms   0    Ever        Any   Nev any
  EHRLIC   9 x   f   15   99      Africa  1998  2004    CS  Prev  CB:symptoms   0    Ever        Any   Nev any
  FERRI1  61     m   25   74      Am:USA  1961  1971    CS  Prev  CB:symptoms   1    Ever        Any   Nev any
  FERRI1  82     f   25   74      Am:USA  1961  1971    CS  Prev  CB:symptoms   1    Ever        Any   Nev any
  FINKLE   1     m   18   25      Am:USA  1969  1974    CS  Prev  CB:symptoms   0    Ever        Any   Nev any
  FOXMAN   3     m   20   69      Am:USA     *  1986    CS  Prev  CB:symptoms   1    Ever        Any   Nev any
  FOXMAN   6     f   20   69      Am:USA     *  1986    CS  Prev  CB:symptoms   1    Ever        Any   Nev any
  GOLDBE   3 x   m   15   99      Am:USA  1970  1974    CS  Prev  CB:symptoms   0    Ever       Cigs  Nev cigs
  GOLDBE   9 x   f   15   99      Am:USA  1970  1974    CS  Prev  CB:symptoms   0    Ever       Cigs  Nev cigs
  HAENSZ   3 x   m   35   74    Eu:Scand  1964  1972    CS  Prev  CB:symptoms   0    Ever        Any   Nev any
  HAENSZ  17 x   f   35   74    Eu:Scand  1964  1972    CS  Prev  CB:symptoms   0    Ever        Any   Nev any
  HARRIS   3     m   15   60      Africa     *  1993    CS  Prev  CB:symptoms   0    Ever        Any   Nev any
  HAWTHO  10     m   45   76       Eu:UK  1965  1978    Pr  Prev  CB:symptoms   0    Ever       Cigs  N/L cigs
  HAWTHO  17     f   45   76       Eu:UK  1965  1978    Pr  Prev  CB:symptoms   0    Ever       Cigs  N/L cigs
   HAYES   3 x   m   15   99      Am:USA  1970  1974    CS  Prev  CB:symptoms   0    Ever       Cigs  Nev cigs
   HAYES   6 x   f   15   99      Am:USA  1970  1974    CS  Prev  CB:symptoms   0    Ever       Cigs  Nev cigs
  HIGGI2   3     m   25   74       Eu:UK  1956  1957    CS  Prev  CB:symptoms   0    Ever       Cigs  Nev cigs
  HIGGI2   9 x   f   25   74       Eu:UK  1956  1957    CS  Prev  CB:symptoms   0    Ever       Cigs  Nev cigs
  HIGGI3   3     m   55   64       Eu:UK  1956  1958    CS  Prev  CB:symptoms   0    Ever        Any   Nev any
  HIGGI3   9     f   55   64       Eu:UK  1956  1958    CS  Prev  CB:symptoms   0    Ever        Any   Nev any
  HIGGI6   3     m   25   64      Am:USA  1962  1977    CS  Prev  CB:symptoms   1    Ever        Any   Nev any
  HIGGI6  10     f   25   64      Am:USA  1962  1977    CS  Prev  CB:symptoms   1    Ever        Any   Nev any
  HOLLA2   3     m   40   59      Am:USA  1962  1965    CS  Prev  CB:symptoms   0    Ever        Any   Nev any
  HOLLNA   3     m   40   40    Eu:Scand  1976  1983    CS  Prev  CB:symptoms   0    Ever        Any   Nev any
  HOLLNA  11     f   40   40    Eu:Scand  1976  1983    CS  Prev  CB:symptoms   0    Ever        Any   Nev any
   HOUSE   3 x   m   15   99      Am:USA  1970  1974    CS  Prev  CB:symptoms   0    Ever       Cigs  Nev cigs
   HOUSE   9 x   f   15   99      Am:USA  1970  1974    CS  Prev  CB:symptoms   0    Ever       Cigs  Nev cigs
  HUCHON   3 x   b   25   99     Eu:West     *  2002    CS  Prev  CB:symptoms   0    Ever       Cigs  Nev cigs
  HUHTI1  39 x   m   40   64    Eu:Scand  1961  1965    CS  Prev  CB:symptoms   0    Ever        Any   Nev any
  HUHTI1  88 x   f   40   64    Eu:Scand  1961  1965    CS  Prev  CB:symptoms   0    Ever        Any   Nev any
  HUHTI3  19 x   m   25   69    Eu:Scand  1968  1978    CS  Prev  CB:symptoms   0    Ever        Any   Nev any
  JINDA2   1 x   b   35   99 Asia:MidE/S     *  2006    CS  Prev  CB:symptoms   0    Ever        Any   Nev any
   JOSHI   6     m   17   64 Asia:MidE/S     *  1975    CS  Prev  CB:symptoms   0    Ever        Any   Nev any
  JOUSI1   3     m   25   64    Eu:Scand  1972  1996    CS  Prev  CB:symptoms   0    Ever        Any   Nev any
  JOUSI1   6     f   25   64    Eu:Scand  1972  1996    CS  Prev  CB:symptoms   0    Ever        Any   Nev any
   KUBIK   3     m   15   99     Eu:East  1972  1984    CS  Prev  CB:symptoms   0    Ever        Any   N/L any
   KUBIK   4     f   15   99     Eu:East  1972  1984    CS  Prev  CB:symptoms   0    Ever        Any   N/L any
  LAMBER   3 x   m   35   69       Eu:UK  1965  1970    CS  Prev  CB:symptoms   0    Ever       Cigs  Nev cigs
  LAMBER  35 x   f   35   69       Eu:UK  1965  1970    CS  Prev  CB:symptoms   0    Ever        Any   Nev any
  LANGHA   3 x   m   20   99    Eu:Scand  1995  2000    CS  Prev  CB:symptoms   0    Ever        Any   Nev any
  LANGHA   9 x   f   20   99    Eu:Scand  1995  2000    CS  Prev  CB:symptoms   0    Ever        Any   Nev any
  LINDST  17 x   m   20   69    Eu:Scand     *  2001    CS  Prev  CB:symptoms   0    Ever       Cigs  Nev cigs
  LINDST  20 x   f   20   69    Eu:Scand     *  2001    CS  Prev  CB:symptoms   0    Ever       Cigs  Nev cigs
  MAGNUS   3 x   m   50   80    Eu:Scand  1993  1999    CS  Prev  CB:symptoms   0    Ever        Any   Nev any
  MANFRE   3     m   20   65   Am:Canada  1978  1989    CS  Prev  CB:symptoms   0    Ever        Any   Nev any
  MANFRE   6     f   20   65   Am:Canada  1978  1989    CS  Prev  CB:symptoms   0    Ever        Any   Nev any
  MENEZ1   3 x   b   40   99 Am:Sth/Cent  1990  1995    CS  Prev  CB:symptoms   0    Ever       Cigs  Nev cigs
   MEREN   6     b   15   64     Eu:East  1995  2001    CS  Prev  CB:symptoms   4    Ever       Cigs  Nev cigs
   MILNE   3     m   62   90       Eu:UK  1968  1972    CS  Prev  CB:symptoms   0    Ever       Cigs  Nev cigs
   MILNE  15     f   62   90       Eu:UK  1968  1972    CS  Prev  CB:symptoms   0    Ever        Any   Nev any
  MOLLER   3     b   40   72     Eu:West     *  2001    CC  Prev  CB:symptoms   0    Ever       Cigs  Nev cigs
  MUELLE  33 x   m   20   69      Am:USA  1967  1971    CS  Prev  CB:symptoms   0    Ever        Any   Nev any
  MUELLE  49 x   f   20   69      Am:USA  1967  1971    CS  Prev  CB:symptoms   0    Ever        Any   Nev any
  NEJJAR   1 x   m   65   99     Eu:West  1991  1996    CS  Prev  CB:symptoms   0    Ever       Cigs  Nev cigs
  NEJJAR   2 x   f   65   99     Eu:West  1991  1996    CS  Prev  CB:symptoms   0    Ever       Cigs  Nev cigs
  OGILVI   3     m   30   99       Eu:UK  1955  1957    CC  Prev  CB:symptoms   0    Ever        Any   Nev any
  OGILVI   8     f   30   99       Eu:UK  1955  1957    CC  Prev  CB:symptoms   0    Ever        Any   Nev any
  OSWAL2   1 x   m   15   65       Eu:UK  1954  1955    CS  Prev  CB:symptoms   0    Ever       Cigs  N/L cigs
  OSWAL2   3 x   f   15   65       Eu:UK  1954  1955    CS  Prev  CB:symptoms   0    Ever       Cigs  N/L cigs
  PANDEY   3 x   m   20   99 Asia:MidE/S  1979  1984    CS  Prev  CB:symptoms   0    Ever        Any   Nev any
  ________________________________________________________________________________________________________________________
                                            International Evidence on Smoking and COPD, Phase 3, Analysis run on 27-SEP-10

                                                   Table 2 - A - 4 - 4

            IESCOPD - Meta-analysis of ever smoking, any product (or cigarettes if all product not available)
                                                CB based on symptoms only
                                                      Least-adjusted


     REF|NRR|X|SEX|AGEL|AGEH|     REGION|BEGYR|PUBYR|STTYP|ONSET|      DISEAS|ADJ|SMOKSTA|   PRODUCT|    UNEXP|

  PANDEY   9 x   f   20   99 Asia:MidE/S  1979  1984    CS  Prev  CB:symptoms   0    Ever        Any   Nev any
  PEREZP   5     f   40   99 Am:Sth/Cent  1992  1996    CC  Prev  CB:symptoms   0    Ever       Cigs  Nev cigs
    REID   3 x   m   35   74      Am:USA  1962  1966    CS  Prev  CB:symptoms   0    Ever       Cigs  Nev cigs
    REID  19 x   f   35   74      Am:USA  1962  1966    CS  Prev  CB:symptoms   0    Ever       Cigs  Nev cigs
  SAWICK   5     m   19   70     Eu:East  1968  1972    CS  Prev  CB:symptoms   0    Ever        Any   Nev any
  SAWICK  18     f   19   70     Eu:East  1968  1972    CS  Prev  CB:symptoms   0    Ever        Any   Nev any
  SOBRAD   3 x   b   40   69     Eu:West  1996  2000    CS  Prev  CB:symptoms   0    Ever        Any   Nev any
  STJERN   3     b   16   72    Eu:Scand  1981  1985    CS  Prev  CB:symptoms   0    Ever        Any   Nev any
  WAGEN2   3 x   b   21   68     Eu:West  2001  2004    CC  Prev  CB:symptoms   0    Ever       Cigs  Nev cigs
  WILHEL   3     m   54   54    Eu:Scand  1967  1969    CS  Prev  CB:symptoms   0    Ever        Any   Nev any
   WOODS   3     b   20   44      Aus/NZ     *  2000    CS  Prev  CB:symptoms   5    Ever       Cigs  Nev cigs
   WOOLF   3     f   25   54   Am:Canada     *  1974    CS  Prev  CB:symptoms   0    Ever       Cigs  Nev cigs
  YAMAGU   9     b   40   99   Asia:FarE  1986  1988    CS  Prev  CB:symptoms   6    Ever       Cigs  Nev cigs
    ZOIA   3     b   18   69     Eu:West     *  1995    CS  Prev  CB:symptoms   4    Ever        Any   Nev any


  ________________________________________________________________________________________________________________________
                                            International Evidence on Smoking and COPD, Phase 3, Analysis run on 27-SEP-10

                                                   Table 2 - A - 4 - 5

            IESCOPD - Meta-analysis of ever smoking, any product (or cigarettes if all product not available)
                                                CB based on symptoms only
                                                      Least-adjusted


                        Number Exposed  Non-exposed
 REF    NRR SEX ADJ     Case    Cont    Case    Cont      RR        95.00%CI
 ANDER1 32  m   1         50       -       4       -      4.51 (  1.53-  13.29)
 ANDER1 35  f   1         19       -      15       -      2.20 (  1.08-   4.51)
 Subtotal ANDER1                                          2.74 (  1.51-   4.97)
 BECK1  3   m   0         17     243       3     189      4.41 (  1.27-  15.26)
 BECK1  6   f   0         10     198       6     318      2.68 (  0.96-   7.48)
 Subtotal BECK1                                           3.28 (  1.49-   7.24)
*BECK2  3   m   0          5     243       2     189      1.94 (  0.38-   9.91)
*BECK2  6   f   0          4     199       3     318      2.13 (  0.48-   9.42)
 Subtotal BECK2                                           2.04 (  0.68-   6.13)
 BJORNS 3   b   0        836    3956     354    4179      2.49 (  2.19-   2.85)
 CERVER 3   b   3       1465       -     754       -      2.70 (  2.47-   2.97)
 COATES 3   b   0        135     913      21     515      3.63 (  2.26-   5.82)
 DEANE  5   m   0        104     307       3      66      7.45 (  2.29-  24.21)
 DEMARC 17  b   0        341    7197      91    5624      2.93 (  2.32-   3.70)
 DOPICO 3   m   0         37     139       6      57      2.53 (  1.01-   6.32)
 EHRLIC 3   m   0        100    2962      33    2672      2.73 (  1.84-   4.07)
 EHRLIC 9   f   0         88    1708     138    6208      2.32 (  1.76-   3.04)
 Subtotal EHRLIC                                          2.44 (  1.95-   3.06)
 FERRI1 61  m   1        156       -      10       -      3.22 (  1.62-   6.42)
 FERRI1 82  f   1         44       -      35       -      2.17 (  1.35-   3.51)
 Subtotal FERRI1                                          2.47 (  1.67-   3.65)
 FINKLE 1   m   0       3551   17761     596   10240      3.44 (  3.14-   3.76)
 FOXMAN 3   m   1        219       -      41       -      2.48 (  1.73-   3.54)
 FOXMAN 6   f   1        151       -      59       -      2.58 (  1.88-   3.53)
 Subtotal FOXMAN                                          2.54 (  2.00-   3.21)
 GOLDBE 3   m   0        348    1450     148     909      1.47 (  1.20-   1.82)
 GOLDBE 9   f   0        283    1729      61    1076      2.89 (  2.17-   3.85)
 Subtotal GOLDBE                                          1.86 (  1.57-   2.21)
 HAENSZ 3   m   0        200    9296      27    2742      2.18 (  1.46-   3.27)
 HAENSZ 17  f   0         79    3234      97   11536      2.91 (  2.15-   3.92)
 Subtotal HAENSZ                                          2.63 (  2.06-   3.34)
 HARRIS 3   m   0         12     268       6     518      3.87 (  1.43-  10.41)
 HAWTHO 10  m   0        646    8503      49    2097      3.25 (  2.42-   4.36)
 HAWTHO 17  f   0        320    3628     103    3440      2.95 (  2.35-   3.70)
 Subtotal HAWTHO                                          3.06 (  2.55-   3.66)
 HAYES  3   m   0        194    1440      12     591      6.64 (  3.67-  11.98)
 HAYES  6   f   0        163    1484      25    1386      6.09 (  3.97-   9.34)
 Subtotal HAYES                                           6.27 (  4.44-   8.87)
 HIGGI2 3   m   0         25     248       1      27      2.72 (  0.35-  20.89)
 HIGGI2 9   f   0          8      96       6     170      2.36 (  0.80-   7.01)
 Subtotal HIGGI2                                          2.44 (  0.93-   6.36)
 HIGGI3 3   m   0          6      82       0       6      1.02~(  0.05-  20.27)
 HIGGI3 9   f   0          2      26       0      64     12.17~(  0.57- 262.12)
 Subtotal HIGGI3                                          3.41 (  0.40-  28.99)
 HIGGI6 3   m   1        294       -      17       -      4.59 (  2.78-   7.59)
 HIGGI6 10  f   1         86       -      40       -      2.99 (  2.03-   4.40)
 Subtotal HIGGI6                                          3.51 (  2.58-   4.76)
 HOLLA2 3   m   0        142     394       5      84      6.05 (  2.41-  15.23)
 HOLLNA 3   m   0        139     254      10     101      5.53 (  2.79-  10.93)
 HOLLNA 11  f   0         72     245      14     217      4.56 (  2.50-   8.31)
 Subtotal HOLLNA                                          4.96 (  3.16-   7.78)
 HOUSE  3   m   0        273    1654      51    1327      4.29 (  3.16-   5.84)
 HOUSE  9   f   0        194    1187     103    2846      4.52 (  3.52-   5.79)
 Subtotal HOUSE                                           4.43 (  3.65-   5.37)
 HUCHON 3   b   0        404    7364     177    7183      2.23 (  1.86-   2.66)
 HUHTI1 39  m   0        177     354       7     115      8.21 (  3.75-  17.99)
 HUHTI1 88  f   0         16      98      32     677      3.45 (  1.83-   6.53)
 Subtotal HUHTI1                                          4.87 (  2.97-   7.99)
 HUHTI3 19  m   0        272     538      28     211      3.81 (  2.50-   5.80)
 JINDA2 1   b   0        666    8002     784   25843      2.74 (  2.47-   3.05)
 JOSHI  6   m   0         50     194       9     220      6.30 (  3.02-  13.14)
 JOUSI1 3   m   0       1741    4824     360    2417      2.42 (  2.14-   2.74)
 JOUSI1 6   f   0        278    1344     940    7540      1.66 (  1.43-   1.92)
 Subtotal JOUSI1                                          2.07 (  1.88-   2.27)
 KUBIK  3   m   0       3253   16409     421    8006      3.77 (  3.39-   4.19)
 KUBIK  4   f   0        298    4760     797   25758      2.02 (  1.76-   2.32)
 Subtotal KUBIK                                           2.99 (  2.75-   3.25)
 LAMBER 3   m   0        257    3523      17     642      2.75 (  1.67-   4.53)
 LAMBER 35  f   0        118    2120      54    2390      2.46 (  1.78-   3.42)
 Subtotal LAMBER                                          2.55 (  1.94-   3.35)
 LANGHA 3   m   0        985   17046     203   10999      3.13 (  2.69-   3.65)
  ________________________________________________________________________________________________________________________
                                            International Evidence on Smoking and COPD, Phase 3, Analysis run on 27-SEP-10

                                                   Table 2 - A - 4 - 5

            IESCOPD - Meta-analysis of ever smoking, any product (or cigarettes if all product not available)
                                                CB based on symptoms only
                                                      Least-adjusted


                        Number Exposed  Non-exposed
 REF    NRR SEX ADJ     Case    Cont    Case    Cont      RR        95.00%CI
 LANGHA 9   f   0        635   15691     351   15771      1.82 (  1.59-   2.08)
 Subtotal LANGHA                                          2.29 (  2.08-   2.54)
 LINDST 17  m   0        444    3350     214    2839      1.76 (  1.48-   2.09)
 LINDST 20  f   0        319    2795     280    3487      1.42 (  1.20-   1.68)
 Subtotal LINDST                                          1.58 (  1.40-   1.78)
 MAGNUS 3   m   0         93     695      15     372      3.32 (  1.90-   5.81)
 MANFRE 3   m   0         32      79       3      40      5.40 (  1.56-  18.72)
 MANFRE 6   f   0         48     108      22     149      3.01 (  1.72-   5.28)
 Subtotal MANFRE                                          3.32 (  1.99-   5.55)
 MENEZ1 3   b   0        105     484      29     435      3.25 (  2.11-   5.01)
 MEREN  6   b   4          -       -       -       -      1.75 (  1.39-   2.22)
 MILNE  3   m   0         65     105       3      40      8.25 (  2.45-  27.77)
 MILNE  15  f   0         13      57      12     186      3.54 (  1.53-   8.18)
 Subtotal MILNE                                           4.65 (  2.33-   9.27)
 MOLLER 3   b   0         13       9       2      11      7.94 (  1.41-  44.80)
 MUELLE 33  m   0         47     175       2      57      7.65 (  1.80-  32.51)
 MUELLE 49  f   0         31     126       3     168     13.78 (  4.12-  46.08)
 Subtotal MUELLE                                         10.82 (  4.28-  27.34)
 NEJJAR 1   m   0        147     553      42     229      1.45 (  1.00-   2.11)
 NEJJAR 2   f   0         17     122      98    1115      1.59 (  0.92-   2.74)
 Subtotal NEJJAR                                          1.49 (  1.09-   2.03)
 OGILVI 3   m   0        261     210      11      29      3.28 (  1.60-   6.71)
 OGILVI 8   f   0        106      86      86     160      2.29 (  1.56-   3.38)
 Subtotal OGILVI                                          2.49 (  1.77-   3.49)
 OSWAL2 1   m   0        421    2196      96     889      1.78 (  1.40-   2.25)
 OSWAL2 3   f   0        149     821     116    1156      1.81 (  1.40-   2.34)
 Subtotal OSWAL2                                          1.79 (  1.50-   2.13)
 PANDEY 3   m   0        238     914       4     219     14.26 (  5.25-  38.72)
 PANDEY 9   f   0        208     718      66     459      2.01 (  1.49-   2.72)
 Subtotal PANDEY                                          2.37 (  1.78-   3.16)
 PEREZP 5   f   0         27      47      77     145      1.08 (  0.63-   1.87)
 REID   3   m   0        248   15002      16    4127      4.26 (  2.57-   7.08)
 REID   19  f   0         80    8185      37   10430      2.76 (  1.86-   4.07)
 Subtotal REID                                            3.24 (  2.38-   4.42)
 SAWICK 5   m   0        286    1272      14     353      5.67 (  3.27-   9.82)
 SAWICK 18  f   0         75     649      46    1660      4.17 (  2.86-   6.09)
 Subtotal SAWICK                                          4.60 (  3.37-   6.28)
 SOBRAD 3   b   0        172    1838      20    2005      9.38 (  5.88-  14.97)
 STJERN 3   b   0         15     555       6     873      3.93 (  1.52-  10.20)
 WAGEN2 3   b   0        246    2762     111    2136      1.71 (  1.36-   2.16)
 WILHEL 3   m   0         18     207       1      87      7.57 (  0.99-  57.56)
 WOODS  3   b   5          -       -       -       -      2.46 (  2.12-   2.87)
 WOOLF  3   f   0         92     225      10     173      7.07 (  3.58-  13.99)
 YAMAGU 9   b   6        334       -     128       -      2.26 (  1.82-   2.81)
 ZOIA   3   b   4          -       -       -       -      1.45 (  0.66-   3.16)
Partial Totals         24318  197656    8699  201513
*prospective study                                        ~ With 0.5 adjustment for zero

                       N       86
                      NS       56


                      Wt  4981.45
                 Het Chi   534.98
                 Het  df       85
                 Het  P       ***
               Fixed  RR     2.59
                     RRl     2.52
                     RRu     2.66
                      P       +++
              Random  RR     2.86
                     RRl     2.63
                     RRu     3.12
                      P       +++
               Asymm  P         *


  ________________________________________________________________________________________________________________________
                                            International Evidence on Smoking and COPD, Phase 3, Analysis run on 27-SEP-10

                                                   Table 2 - A - 4 - 6

            IESCOPD - Meta-analysis of ever smoking, any product (or cigarettes if all product not available)
                                                CB based on symptoms only
                                                      Least-adjusted


                       N       86
                      NS       56


                      Wt  4981.45
                 Het Chi   534.98
                 Het  df       85
                 Het  P       ***
               Fixed  RR     2.59
                     RRl     2.52
                     RRu     2.66
                      P       +++
              Random  RR     2.86
                     RRl     2.63
                     RRu     3.12
                      P       +++
               Asymm  P         *

                                   Sex
                             both      male    female     Total


                       N       15        39        32        86
                      NS       15        39        32        86


                      Wt  1661.29   1893.46   1426.69   4981.45
                 Het Chi    67.28    216.58    174.99    534.98
                 Het  df       14        38        31        85
                 Het  P       ***       ***       ***       ***
               Fixed  RR     2.55      2.97      2.19      2.59
                     RRl     2.43      2.84      2.08      2.52
                     RRu     2.68      3.11      2.31      2.66
                      P       +++       +++       +++       +++
              Random  RR     2.60      3.32      2.61      2.86
                     RRl     2.29      2.88      2.26      2.63
                     RRu     2.95      3.83      3.01      3.12
                      P       +++       +++       +++       +++
             Between Chi                                  76.12
             Between  df                                      2
             Between  P                                     ***
             Btwn(F)  P                                      **


  ________________________________________________________________________________________________________________________
                                            International Evidence on Smoking and COPD, Phase 3, Analysis run on 27-SEP-10

                                                   Table 2 - A - 4 - 7

            IESCOPD - Meta-analysis of ever smoking, any product (or cigarettes if all product not available)
                                                CB based on symptoms only
                                 Excluded studies (and stage at which they were excluded)


1       CLARK COTTON  MEYER REMYJA RUTGER SNYDER SOBRAX     SU TAKEMU  WANG4   WEIR WHICKE ZALACA
2      ALESSA  AMIGO ANDER2 ANDER3 AUERBA BEDNAR BROGGE  CHEN1  CHEN2  CHEN3  CHENG CLEMEN  COCCI  DEAN1 DEJONG DETORR
       DICKIN DONTA1 EKBERG ENSTRO FERRI2 FERRI3  FIDAN FORAST FUKUCH GEIJER GODTFR GULSVI HAMMO2 HARIKK HEDMAN HIGGI4
       HOZAWA ITABAS JACOBS JAENDI JOHANN KACHEL KARAKA KATANC KHOURY    KIM KLAYTO KOJIMA KOTAN2 KRZYZA KULLER    LAI
         LAM1   LAM2   LAM3  LANGE    LEE   LIAW LINDBE   LIU1   LIU2 LUNDB1  MADOR MANNI1 MANNI2 MANNI3 MARAN1 MARAN2
       MARCUS MATHES MENEZ2 MENEZ3 MENEZ4 MENEZ5 MENEZ6 MONTNE   NAWA NIEPSU NIHLEN NILSSO  OMORI   PEAT   PETO  PRATT
        PRICE RENWIC RICCIO  RYDER SARGEA SHAHAB   SHIN SICHLE SPEIZE STERLI  STROM SUTINE  TAGER   TANG   THUN   TODD
       TRUPIN TSUSHI TVERDA VESTBO VIEGI2 VIKGRE VINEIS VOLLM1 VOLLM2 VONHER   WALD  WANG2 WATSON  WEISS WILSO1   XIAO
           XU   YUAN ZIELI1 ZIELI2 ZIETKO
3       DOLL2 FLETCH HRUBEC JENSEN OSWAL1 RIMING SCHWAR  SHARP SUADIC    WIG WILSO2
4        BANG  DEAN2 HIRAYA   KAHN LANGE2 TAGER2    WEN WOJTYN
5      ALDERS   BEST  BROWN COLLEG  DOLL1 DONTA2 ENRIGH HARDIE     HO  KAHN2   KATO  KIRAZ KOTAN1 LAVECC LEBOWI LUNDB2
       MELLST MILLER PELKON SHIMUR  SILVA TROISI VIEGI1
9      CHAPMA HUHTI2 URRUTI


  ________________________________________________________________________________________________________________________
                                            International Evidence on Smoking and COPD, Phase 3, Analysis run on 27-SEP-10

                                                   Table 2 - A - 4 - 8

            IESCOPD - Meta-analysis of ever smoking, any product (or cigarettes if all product not available)
                                                CB based on symptoms only
                                             Potentially overlapping studies


     REF| REFGP|PRINC|                     OVERLAP|

  DEMARC DEMARC     1         DEMARC/URRUTI/DEMEER
  OSWAL2 OSWAL2     1                OSWAL1/OSWAL2
  HIGGI2   PETO     2    PETO/HIGGI1/HIGGI2/HIGGI5
  HAWTHO   TANG     2             TANG/HAWTHO/WALD
  HUHTI1 HUHTI1     1                HUHTI1/HUHTI2
  FERRI1 FERRIS     2         FERRI1/FERRI2/FERRI3
  LAMBER   TODD     2                  LAMBER/TODD
   BECK1  BECK1     1                  BECK1/BECK2
   BECK2  BECK2     1                  BECK1/BECK2
   HOUSE  HOUSE     1                 HOUSE/CHAPMA
  SAWICK SAWICK     1         SAWICK/KRZYZA/WOJTYN
  HOLLNA HOLLNA     1 GODT/VEST/LANG1+2/SUAD/HOLLN
  HIGGI6 HIGGI4     2                HIGGI4/HIGGI6


  ________________________________________________________________________________________________________________________
                                            International Evidence on Smoking and COPD, Phase 3, Analysis run on 27-SEP-10

                                                    Table 2 - A - 5 -

            IESCOPD - Meta-analysis of ever smoking, any product (or cigarettes if all product not available)
                                             CB other than mortality/symptoms


This analysis is restricted to results for:
1) Eligible study on database
2) Outcome CB
3) Non-dose-response data
4) Ever smoking
5) CB other than based on mortality or symptoms only
6) Results complete enough for use in meta-analysis

Within each study, results are then selected (in the following order of preference, within each sex) for:
7) UNEXP   : never any, never cigarettes, other
8) PROD    : any product, cigarettes, cigarettes only
9) For overlapping studies: principal rather than subsidiary studies
and then for single sex results (m, f) in preference to results for both sexes combined (b).

Results adjusted for the most potential confounders are then chosen in Sections -1 to -3
and results adjusted for the least confounders in Sections -4 to -6. (Those least adjusted results which
actually differ from the most adjusted are marked 'x' in column X in Section -4)

Section -7 shows excluded studies, together with the stage (as above) at which no qualifying
results were found.

Section -8 lists the potentially overlapping studies which have been included (1=principal, 2=subsidiary),
and any results which would have been included in preference except that they had data not complete enough
for use in meta-analysis. It also lists their significance (yes/no), if known.


  ________________________________________________________________________________________________________________________
                                            International Evidence on Smoking and COPD, Phase 3, Analysis run on 27-SEP-10

                                                   Table 2 - A - 5 - 1

            IESCOPD - Meta-analysis of ever smoking, any product (or cigarettes if all product not available)
                                             CB other than mortality/symptoms
                                                      Most-adjusted


     REF|NRR|SEX|AGEL|AGEH|     REGION|BEGYR|PUBYR|STTYP|ONSET|      DISEAS|ADJ|SMOKSTA|   PRODUCT|    UNEXP|

  ALDERS   5   m   35   74       Eu:UK  1977  1985    CC  Prev CB:diagnosed   1    Ever        Any   Nev any
  ALDERS   6   f   35   74       Eu:UK  1977  1985    CC  Prev CB:diagnosed   1    Ever MCigs only   Nev any
   BROWN   1   m   60   69       Eu:UK  1956  1957    CS  Prev CB:diagnosed   0    Ever        Any   Nev any
  COLLEG   6   m   40   64       Eu:UK     *  1961    CS  Prev CB:diagnosed   2    Ever        Any   Nev any
  COLLEG   9   f   40   64       Eu:UK     *  1961    CS  Prev CB:diagnosed   1    Ever        Any   Nev any
  DONTA2   3   m   25   84 Eu:SE/Balkn  1960  1984    Pr   Inc CB:diagnosed   0    Ever       Cigs  Nev cigs
  ENRIGH   3   m   65   99      Am:USA  1989  1994    CS  Prev CB:diagnosed   0    Ever       Cigs  Nev cigs
  ENRIGH   6   f   65   99      Am:USA  1989  1994    CS  Prev CB:diagnosed   0    Ever       Cigs  Nev cigs
  HARDIE   9   m   70   99    Eu:Scand  1998  2005    CS  Prev CB:diagnosed   1    Ever       Cigs  Nev cigs
  HARDIE  12   f   70   99    Eu:Scand  1998  2005    CS  Prev CB:diagnosed   1    Ever       Cigs  Nev cigs
      HO  11   b   70   99   Asia:FarE  1991  1999    CS  Prev  CB:self-rep   3    Ever       Cigs  Nev cigs
    KATO   3   m   40   99   Asia:FarE  1985  1989    CS  Prev  CB:self-rep   1    Ever       Cigs  Nev cigs
    KATO   6   f   40   99   Asia:FarE  1985  1989    CS  Prev  CB:self-rep   1    Ever       Cigs  Nev cigs
   KIRAZ   4   f   25   99 Eu:SE/Balkn  1999  2003    CS  Prev     CB:other   1    Ever       Cigs  Nev cigs
  KOTAN1   9   b   20   69    Eu:Scand  1995  2003    CS  Prev CB:diagnosed   7    Ever        Any   Nev any
  LAVECC   9   b   15   99     Eu:West  1983  1988    CS  Prev  CB:self-rep   6    Ever        Any   Nev any
  LEBOWI  30   b   15   96      Am:USA  1972  1977    CS  Prev CB:diagnosed   3    Ever       Cigs  Nev cigs
  LUNDB2   6   m   35   66    Eu:Scand  1986  1993    CC  Prev     CB:other   1    Ever        Any   Nev any
  LUNDB2  12   f   35   66    Eu:Scand  1986  1993    CC  Prev     CB:other   1    Ever        Any   Nev any
  MELLST   3   m   70   70    Eu:Scand  1971  1982    CS  Prev     CB:other   0    Ever        Any   Nev any
  MILLER   3   m   15   99      Am:USA  1978  1988    CS  Prev CB:diagnosed   1    Ever       Cigs  Nev cigs
  MILLER   6   f   15   99      Am:USA  1978  1988    CS  Prev CB:diagnosed   1    Ever       Cigs  Nev cigs
  PELKON   6   m   40   75    Eu:Scand  1959  2006    Pr   Inc     CB:other   1    Ever       Cigs  Nev cigs
  SHIMUR   3   b   31   80   Asia:FarE     *  1996    CC  Prev     CB:other   0    Ever        Any   Nev any
   SILVA   6   b   20   99      Am:USA  1972  2004    Pr   Inc     CB:other   6    Ever       Cigs  Nev cigs
  TROISI   3   f   34   69      Am:USA  1980  1995    Pr   Inc CB:diagnosed   1    Ever       Cigs  Nev cigs
  VIEGI1   3   m   20   64     Eu:West  1980  1988    CS  Prev CB:diagnosed   0    Ever       Cigs   Nev any
  VIEGI1   6   f   20   64     Eu:West  1980  1988    CS  Prev CB:diagnosed   0    Ever       Cigs   Nev any


  ________________________________________________________________________________________________________________________
                                            International Evidence on Smoking and COPD, Phase 3, Analysis run on 27-SEP-10

                                                   Table 2 - A - 5 - 2

            IESCOPD - Meta-analysis of ever smoking, any product (or cigarettes if all product not available)
                                             CB other than mortality/symptoms
                                                      Most-adjusted


                        Number Exposed  Non-exposed
 REF    NRR SEX ADJ     Case    Cont    Case    Cont      RR        95.00%CI
 ALDERS 5   m   1        510       -      25       -      2.81 (  1.74-   4.54)
 ALDERS 6   f   1        333       -     105       -      2.79 (  2.09-   3.72)
 Subtotal ALDERS                                          2.80 (  2.18-   3.58)
 BROWN  1   m   0        238     725      15      76      1.66 (  0.94-   2.95)
 COLLEG 6   m   2          -       -       -       -      2.88 (  0.98-   8.47)
 COLLEG 9   f   1          -       -       -       -      2.64 (  1.54-   4.52)
 Subtotal COLLEG                                          2.69 (  1.66-   4.35)
*DONTA2 3   m   0         63     333       8     127      3.00 (  1.48-   6.09)
 ENRIGH 3   m   0         71    1432      17     699      2.04 (  1.19-   3.49)
 ENRIGH 6   f   0        102    1155      85    1558      1.62 (  1.20-   2.18)
 Subtotal ENRIGH                                          1.71 (  1.32-   2.22)
 HARDIE 9   m   1         37       -       1       -     10.90 (  2.06-  57.56)
 HARDIE 12  f   1         16       -      11       -      3.33 (  1.59-   6.98)
 Subtotal HARDIE                                          4.05 (  2.06-   7.96)
 HO     11  b   3          -       -       -       -      2.32 (  1.55-   3.46)
 KATO   3   m   1        827       -     118       -      1.33 (  1.09-   1.63)
 KATO   6   f   1        260       -     679       -      1.92 (  1.65-   2.23)
 Subtotal KATO                                            1.68 (  1.49-   1.90)
 KIRAZ  4   f   1         17       -      78       -      4.00 (  1.76-   9.08)
 KOTAN1 9   b   7        137       -      72       -      1.75 (  1.28-   2.39)
 LAVECC 9   b   6       2333       -    1630       -      2.27 (  2.10-   2.46)
 LEBOWI 30  b   3         37       -      26       -      1.13 (  0.68-   1.87)
 LUNDB2 6   m   1        232       -      15       -      7.84 (  4.14-  14.83)
 LUNDB2 12  f   1        106       -      35       -      4.14 (  2.30-   7.44)
 Subtotal LUNDB2                                          5.55 (  3.60-   8.55)
 MELLST 3   m   0        125     559      15     196      2.92 (  1.67-   5.11)
 MILLER 3   m   1         97       -      10       -      5.24 (  2.70-  10.17)
 MILLER 6   f   1         35       -       2       -     20.27 (  4.89-  84.04)
 Subtotal MILLER                                          6.67 (  3.66-  12.17)
*PELKON 6   m   1          -       -       -       -      1.76 (  1.32-   2.36)
 SHIMUR 3   b   0          3       2       3       2      1.00 (  0.08-  12.56)
*SILVA  6   b   6          -       -       -       -      1.76 (  0.99-   3.16)
*TROISI 3   f   1        545       -     253       -      1.93 (  1.66-   2.24)
 VIEGI1 3   m   0         32    1040       4     504      3.88 (  1.36-  11.02)
 VIEGI1 6   f   0          1     582       7    1119      0.27 (  0.03-   2.24)
 Subtotal VIEGI1                                          2.29 (  0.90-   5.84)
Partial Totals          6157    5828    3214    4281
*prospective study

                       N       28
                      NS       20


                      Wt  1402.45
                 Het Chi    98.70
                 Het  df       27
                 Het  P       ***
               Fixed  RR     2.11
                     RRl     2.00
                     RRu     2.22
                      P       +++
              Random  RR     2.31
                     RRl     2.01
                     RRu     2.65
                      P       +++
               Asymm  P      N.S.


  ________________________________________________________________________________________________________________________
                                            International Evidence on Smoking and COPD, Phase 3, Analysis run on 27-SEP-10

                                                   Table 2 - A - 5 - 3

            IESCOPD - Meta-analysis of ever smoking, any product (or cigarettes if all product not available)
                                             CB other than mortality/symptoms
                                                      Most-adjusted


                       N       28
                      NS       20


                      Wt  1402.45
                 Het Chi    98.70
                 Het  df       27
                 Het  P       ***
               Fixed  RR     2.11
                     RRl     2.00
                     RRu     2.22
                      P       +++
              Random  RR     2.31
                     RRl     2.01
                     RRu     2.65
                      P       +++
               Asymm  P      N.S.

                                   Sex
                             both      male    female     Total


                       N        6        12        10        28
                      NS        6        12        10        28


                      Wt   704.04    228.49    469.93   1402.45
                 Het Chi    10.34     53.44     32.19     98.70
                 Het  df        5        11         9        27
                 Het  P       (*)       ***       ***       ***
               Fixed  RR     2.20      1.95      2.07      2.11
                     RRl     2.04      1.71      1.89      2.00
                     RRu     2.36      2.22      2.26      2.22
                      P       +++       +++       +++       +++
              Random  RR     1.91      2.79      2.43      2.31
                     RRl     1.53      1.99      1.94      2.01
                     RRu     2.37      3.91      3.06      2.65
                      P       +++       +++       +++       +++
             Between Chi                                   2.73
             Between  df                                      2
             Between  P                                    N.S.
             Btwn(F)  P                                    N.S.

                                        Continent
                            NAmer    Europe      Asia  oth/mult     Total


                       N        7        17         4                  28
                      NS        5        12         3                  20


                      Wt   264.84    848.95    288.67             1402.45
                 Het Chi    24.97     40.47     10.63               98.70
                 Het  df        6        16         3                  27
                 Het  P       ***       ***         *                 ***
               Fixed  RR     1.91      2.33      1.73                2.11
                     RRl     1.69      2.18      1.54                2.00
                     RRu     2.15      2.49      1.94                2.22
                      P       +++       +++       +++                 +++
              Random  RR     2.15      2.67      1.74                2.31
                     RRl     1.53      2.22      1.31                2.01
                     RRu     3.01      3.20      2.32                2.65
                      P       +++       +++       +++                 +++
             Between Chi                                            22.63
             Between  df                                                2
             Between  P                                               ***
             Btwn(F)  P                                                 *


  ________________________________________________________________________________________________________________________
                                            International Evidence on Smoking and COPD, Phase 3, Analysis run on 27-SEP-10

                                                   Table 2 - A - 5 - 3

            IESCOPD - Meta-analysis of ever smoking, any product (or cigarettes if all product not available)
                                             CB other than mortality/symptoms
                                                      Most-adjusted
                        National cigarette tobacco type (excluding mixed/unkown)
                          blended  virginia     Total


                       N       23         5        28
                      NS       17         3        20


                      Wt  1311.26     91.20   1402.45
                 Het Chi    91.80      2.70     98.70
                 Het  df       22         4        27
                 Het  P       ***      N.S.       ***
               Fixed  RR     2.08      2.60      2.11
                     RRl     1.97      2.11      2.00
                     RRu     2.20      3.19      2.22
                      P       +++       +++       +++
              Random  RR     2.28      2.60      2.31
                     RRl     1.95      2.11      2.01
                     RRu     2.66      3.19      2.65
                      P       +++       +++       +++
             Between Chi                         4.20
             Between  df                            1
             Between  P                             *
             Btwn(F)  P                          N.S.

                                        Start year of study
                            <1970   1970-79   1980-89   1990-99     2000+   unknown     Total


                       N        3         7        10         5                   3        28
                      NS        3         5         6         4                   2        20


                      Wt    64.92    112.25   1130.78     77.35               17.16   1402.45
                 Het Chi     2.05     24.96     55.25      8.92                0.59     98.70
                 Het  df        2         6         9         4                   2        27
                 Het  P      N.S.       ***       ***       (*)                N.S.       ***
               Fixed  RR     1.86      2.58      2.07      2.22                2.59      2.11
                     RRl     1.46      2.14      1.95      1.78                1.62      2.00
                     RRu     2.37      3.10      2.19      2.78                4.17      2.22
                      P       +++       +++       +++       +++                 +++       +++
              Random  RR     1.86      2.80      2.13      2.64                2.59      2.31
                     RRl     1.45      1.84      1.74      1.77                1.62      2.01
                     RRu     2.39      4.25      2.62      3.94                4.17      2.65
                      P       +++       +++       +++       +++                 +++       +++
             Between Chi                                                                 6.94
             Between  df                                                                    4
             Between  P                                                                  N.S.
             Btwn(F)  P                                                                  N.S.

                                Publication year
                            <1980   1980-89   1990-99     2000+     Total


                       N        4        11         7         6        28
                      NS        3         7         5         5        20


                      Wt    43.28    975.93    272.80    110.45   1402.45
                 Het Chi     5.96     53.62     26.18     10.12     98.70
                 Het  df        3        10         6         5        27
                 Het  P      N.S.       ***       ***       (*)       ***
               Fixed  RR     1.75      2.16      2.07      1.95      2.11
                     RRl     1.30      2.03      1.84      1.62      2.00
                     RRu     2.35      2.30      2.33      2.35      2.22
                      P       +++       +++       +++       +++       +++
              Random  RR     1.81      2.47      2.53      2.20      2.31
                     RRl     1.17      1.97      1.81      1.61      2.01
                     RRu     2.81      3.11      3.55      3.02      2.65
                      P        ++       +++       +++       +++       +++
             Between Chi                                             2.82
             Between  df                                                3
             Between  P                                              N.S.
             Btwn(F)  P                                              N.S.
  ________________________________________________________________________________________________________________________
                                            International Evidence on Smoking and COPD, Phase 3, Analysis run on 27-SEP-10

                                                   Table 2 - A - 5 - 3

            IESCOPD - Meta-analysis of ever smoking, any product (or cigarettes if all product not available)
                                             CB other than mortality/symptoms
                                                      Most-adjusted
                               Study type
                               CC        Pr        CS     Total


                       N        5         4        19        28
                      NS        3         4        13        20


                      Wt    84.12    235.74   1082.60   1402.45
                 Het Chi    10.23      1.97     67.68     98.70
                 Het  df        4         3        18        27
                 Het  P         *      N.S.       ***       ***
               Fixed  RR     3.28      1.91      2.08      2.11
                     RRl     2.65      1.69      1.96      2.00
                     RRu     4.06      2.18      2.21      2.22
                      P       +++       +++       +++       +++
              Random  RR     3.63      1.91      2.18      2.31
                     RRl     2.41      1.69      1.83      2.01
                     RRu     5.49      2.18      2.59      2.65
                      P       +++       +++       +++       +++
             Between Chi                                  18.82
             Between  df                                      2
             Between  P                                     ***
             Btwn(F)  P                                     (*)

                                    Lowest age in RR
                        <25/unlim     25-39       40+   unknown     Total


                       N        8         8        12                  28
                      NS        6         6         8                  20


                      Wt   694.64    268.64    439.18             1402.45
                 Het Chi    30.62     28.32     24.41               98.70
                 Het  df        7         7        11                  27
                 Het  P       ***       ***         *                 ***
               Fixed  RR     2.23      2.34      1.81                2.11
                     RRl     2.07      2.08      1.65                2.00
                     RRu     2.40      2.64      1.99                2.22
                      P       +++       +++       +++                 +++
              Random  RR     2.29      3.17      1.96                2.31
                     RRl     1.59      2.26      1.65                2.01
                     RRu     3.31      4.44      2.33                2.65
                      P       +++       +++       +++                 +++
             Between Chi                                            15.35
             Between  df                                                2
             Between  P                                               ***
             Btwn(F)  P                                              N.S.

                                         Highest age in RR
                              <65     65-74     75-84 85+/unlim   unknown     Total


                       N        4         8         3        13                  28
                      NS        2         6         3         9                  20


                      Wt    20.95    318.02     53.81   1009.68             1402.45
                 Het Chi     5.01     30.18      2.12     58.78               98.70
                 Het  df        3         7         2        12                  27
                 Het  P      N.S.       ***      N.S.       ***                 ***
               Fixed  RR     2.60      2.22      1.89      2.08                2.11
                     RRl     1.69      1.99      1.45      1.96                2.00
                     RRu     3.99      2.48      2.47      2.21                2.22
                      P       +++       +++       +++       +++                 +++
              Random  RR     2.44      2.67      1.92      2.16                2.31
                     RRl     1.27      2.02      1.41      1.76                2.01
                     RRu     4.70      3.52      2.61      2.66                2.65
                      P        ++       +++       +++       +++                 +++
             Between Chi                                                       2.61
             Between  df                                                          3
             Between  P                                                        N.S.
             Btwn(F)  P                                                        N.S.
  ________________________________________________________________________________________________________________________
                                            International Evidence on Smoking and COPD, Phase 3, Analysis run on 27-SEP-10

                                                   Table 2 - A - 5 - 3

            IESCOPD - Meta-analysis of ever smoking, any product (or cigarettes if all product not available)
                                             CB other than mortality/symptoms
                                                      Most-adjusted
                           Study weakness
                              Yes        No     Total


                       N        4        24        28
                      NS        3        17        20


                      Wt    28.88   1373.57   1402.45
                 Het Chi     5.60     75.72     98.70
                 Het  df        3        23        27
                 Het  P      N.S.       ***       ***
               Fixed  RR     4.55      2.08      2.11
                     RRl     3.16      1.97      2.00
                     RRu     6.55      2.19      2.22
                      P       +++       +++       +++
              Random  RR     4.36      2.14      2.31
                     RRl     2.53      1.87      2.01
                     RRu     7.50      2.44      2.65
                      P       +++       +++       +++
             Between Chi                        17.38
             Between  df                            1
             Between  P                           ***
             Btwn(F)  P                             *

                           CB subtype
                             mort     sympt     other     Total


                       N                           28        28
                      NS                           20        20


                      Wt                      1402.45   1402.45
                 Het Chi                        98.70     98.70
                 Het  df                           27        27
                 Het  P                           ***       ***
               Fixed  RR                         2.11      2.11
                     RRl                         2.00      2.00
                     RRu                         2.22      2.22
                      P                           +++       +++
              Random  RR                         2.31      2.31
                     RRl                         2.01      2.01
                     RRu                         2.65      2.65
                      P                           +++       +++
             Between Chi
             Between  df
             Between  P                                    N.S.
             Btwn(F)  P                                    N.S.

                           Asthma analysis type (CB)
                        inc-irres  excl-all excl-cntr     Total


                       N       22         3         3        28
                      NS       15         3         2        20


                      Wt  1156.94    224.33     21.19   1402.45
                 Het Chi    73.22      1.89      3.80     98.70
                 Het  df       21         2         2        27
                 Het  P       ***      N.S.      N.S.       ***
               Fixed  RR     2.11      1.92      5.29      2.11
                     RRl     1.99      1.69      3.45      2.00
                     RRu     2.24      2.19      8.09      2.22
                      P       +++       +++       +++       +++
              Random  RR     2.22      1.92      5.03      2.31
                     RRl     1.90      1.69      2.54      2.01
                     RRu     2.59      2.19      9.95      2.65
                      P       +++       +++       +++       +++
             Between Chi                                  19.79
             Between  df                                      2
             Between  P                                     ***
             Btwn(F)  P                                     (*)
  ________________________________________________________________________________________________________________________
                                            International Evidence on Smoking and COPD, Phase 3, Analysis run on 27-SEP-10

                                                   Table 2 - A - 5 - 3

            IESCOPD - Meta-analysis of ever smoking, any product (or cigarettes if all product not available)
                                             CB other than mortality/symptoms
                                                      Most-adjusted
                                   Number of CB cases
                             1-50    51-100   101-200      201+     Total


                       N        3         5         7        13        28
                      NS        2         4         5         9        20


                      Wt     4.99     33.21     78.30   1285.95   1402.45
                 Het Chi     5.26      5.99     23.11     58.85     98.70
                 Het  df        2         4         6        12        27
                 Het  P       (*)      N.S.       ***       ***       ***
               Fixed  RR     2.07      2.83      2.49      2.07      2.11
                     RRl     0.86      2.02      2.00      1.96      2.00
                     RRu     4.99      3.98      3.11      2.19      2.22
                      P      N.S.       +++       +++       +++       +++
              Random  RR     1.25      3.00      2.94      2.11      2.31
                     RRl     0.22      1.95      1.84      1.81      2.01
                     RRu     6.99      4.63      4.70      2.45      2.65
                      P      N.S.       +++       +++       +++       +++
             Between Chi                                             5.49
             Between  df                                                3
             Between  P                                              N.S.
             Btwn(F)  P                                              N.S.

                            Analysis type
                         prevlnce     onset     Total


                       N       24         4        28
                      NS       16         4        20


                      Wt  1166.72    235.74   1402.45
                 Het Chi    94.07      1.97     98.70
                 Het  df       23         3        27
                 Het  P       ***      N.S.       ***
               Fixed  RR     2.15      1.91      2.11
                     RRl     2.03      1.69      2.00
                     RRu     2.28      2.18      2.22
                      P       +++       +++       +++
              Random  RR     2.43      1.91      2.31
                     RRl     2.06      1.69      2.01
                     RRu     2.88      2.18      2.65
                      P       +++       +++       +++
             Between Chi                         2.66
             Between  df                            1
             Between  P                          N.S.
             Btwn(F)  P                          N.S.

                             Smoking product
                              any      cigs  cigsonly     Total


                       N       10        17         1        28
                      NS        8        12         1        21


                      Wt   731.61    624.62     46.22   1402.45
                 Het Chi    24.51     54.22      0.00     98.70
                 Het  df        9        16         0        27
                 Het  P        **       ***      N.S.       ***
               Fixed  RR     2.31      1.86      2.79      2.11
                     RRl     2.15      1.72      2.09      2.00
                     RRu     2.49      2.01      3.72      2.22
                      P       +++       +++       +++       +++
              Random  RR     2.66      2.11      2.79      2.31
                     RRl     2.09      1.75      2.09      2.01
                     RRu     3.39      2.54      3.72      2.65
                      P       +++       +++       +++       +++
             Between Chi                                  19.97
             Between  df                                      2
             Between  P                                     ***
             Btwn(F)  P                                     (*)
  ________________________________________________________________________________________________________________________
                                            International Evidence on Smoking and COPD, Phase 3, Analysis run on 27-SEP-10

                                                   Table 2 - A - 5 - 3

            IESCOPD - Meta-analysis of ever smoking, any product (or cigarettes if all product not available)
                                             CB other than mortality/symptoms
                                                      Most-adjusted
                                     Unexposed group
                          nev any   nev cig  nev+ any  nev+ cig     Total


                       N       13        15                            28
                      NS        9        11                            20


                      Wt   782.22    620.23                       1402.45
                 Het Chi    30.95     49.12                         98.70
                 Het  df       12        14                            27
                 Het  P        **       ***                           ***
               Fixed  RR     2.34      1.85                          2.11
                     RRl     2.18      1.71                          2.00
                     RRu     2.51      2.01                          2.22
                      P       +++       +++                           +++
              Random  RR     2.64      2.10                          2.31
                     RRl     2.14      1.74                          2.01
                     RRu     3.27      2.52                          2.65
                      P       +++       +++                           +++
             Between Chi                                            18.63
             Between  df                                                1
             Between  P                                               ***
             Btwn(F)  P                                                 *

                        Unexposed group (combining nev+ with main levels)
                          nev any   nev cig     Total


                       N       13        15        28
                      NS        9        11        20


                      Wt   782.22    620.23   1402.45
                 Het Chi    30.95     49.12     98.70
                 Het  df       12        14        27
                 Het  P        **       ***       ***
               Fixed  RR     2.34      1.85      2.11
                     RRl     2.18      1.71      2.00
                     RRu     2.51      2.01      2.22
                      P       +++       +++       +++
              Random  RR     2.64      2.10      2.31
                     RRl     2.14      1.74      2.01
                     RRu     3.27      2.52      2.65
                      P       +++       +++       +++
             Between Chi                        18.63
             Between  df                            1
             Between  P                           ***
             Btwn(F)  P                             *

                        Smoking results reported in study (CB)
                             Ever   Current      Both     Total


                       N        1                  27        28
                      NS        1                  19        20


                      Wt     5.71             1396.74   1402.45
                 Het Chi     0.00               96.36     98.70
                 Het  df        0                  26        27
                 Het  P      N.S.                 ***       ***
               Fixed  RR     4.00                2.10      2.11
                     RRl     1.76                2.00      2.00
                     RRu     9.09                2.22      2.22
                      P       +++                 +++       +++
              Random  RR     4.00                2.28      2.31
                     RRl     1.76                1.98      2.01
                     RRu     9.09                2.62      2.65
                      P       +++                 +++       +++
             Between Chi                                   2.35
             Between  df                                      1
             Between  P                                    N.S.
             Btwn(F)  P                                    N.S.
  ________________________________________________________________________________________________________________________
                                            International Evidence on Smoking and COPD, Phase 3, Analysis run on 27-SEP-10

                                                   Table 2 - A - 5 - 3

            IESCOPD - Meta-analysis of ever smoking, any product (or cigarettes if all product not available)
                                             CB other than mortality/symptoms
                                                      Most-adjusted
                        Number of adjustment variables
                                0         1        2+     Total


                       N        8        14         6        28
                      NS        6         9         6        21


                      Wt    93.32    602.40    706.74   1402.45
                 Het Chi    10.54     75.28     10.21     98.70
                 Het  df        7        13         5        27
                 Het  P      N.S.       ***       (*)       ***
               Fixed  RR     1.93      2.04      2.20      2.11
                     RRl     1.58      1.88      2.04      2.00
                     RRu     2.37      2.21      2.37      2.22
                      P       +++       +++       +++       +++
              Random  RR     2.04      2.84      1.95      2.31
                     RRl     1.53      2.24      1.57      2.01
                     RRu     2.73      3.60      2.41      2.65
                      P       +++       +++       +++       +++
             Between Chi                                   2.68
             Between  df                                      2
             Between  P                                    N.S.
             Btwn(F)  P                                    N.S.

                        RR adjusted for sex (combined sex RR only)
                              Yes        No     Total


                       N        5         1         6
                      NS        5         1         6


                      Wt   703.44      0.60    704.04
                 Het Chi     9.96      0.00     10.34
                 Het  df        4         0         5
                 Het  P         *      N.S.       (*)
               Fixed  RR     2.20      1.00      2.20
                     RRl     2.04      0.08      2.04
                     RRu     2.37     12.56      2.36
                      P       +++      N.S.       +++
              Random  RR     1.91      1.00      1.91
                     RRl     1.52      0.08      1.53
                     RRu     2.40     12.56      2.37
                      P       +++      N.S.       +++
             Between Chi                         0.37
             Between  df                            1
             Between  P                          N.S.
             Btwn(F)  P                          N.S.

                        RR adjusted for age


                       N       17        11        28
                      NS       12         8        20


                      Wt  1292.79    109.66   1402.45
                 Het Chi    68.12     29.98     98.70
                 Het  df       16        10        27
                 Het  P       ***       ***       ***
               Fixed  RR     2.10      2.27      2.11
                     RRl     1.99      1.88      2.00
                     RRu     2.21      2.73      2.22
                      P       +++       +++       +++
              Random  RR     2.20      2.72      2.31
                     RRl     1.89      1.85      2.01
                     RRu     2.56      3.98      2.65
                      P       +++       +++       +++
             Between Chi                         0.60
             Between  df                            1
             Between  P                          N.S.
             Btwn(F)  P                          N.S.

  ________________________________________________________________________________________________________________________
                                            International Evidence on Smoking and COPD, Phase 3, Analysis run on 27-SEP-10

                                                   Table 2 - A - 5 - 3

            IESCOPD - Meta-analysis of ever smoking, any product (or cigarettes if all product not available)
                                             CB other than mortality/symptoms
                                                      Most-adjusted
                        RR adjusted for factor other than sex, age
                              Yes        No     Total


                       N        9        19        28
                      NS        8        13        21


                      Wt   723.08    679.37   1402.45
                 Het Chi    27.88     64.96     98.70
                 Het  df        8        18        27
                 Het  P       ***       ***       ***
               Fixed  RR     2.25      1.97      2.11
                     RRl     2.09      1.83      2.00
                     RRu     2.42      2.13      2.22
                      P       +++       +++       +++
              Random  RR     2.39      2.32      2.31
                     RRl     1.81      1.94      2.01
                     RRu     3.17      2.78      2.65
                      P       +++       +++       +++
             Between Chi                         5.86
             Between  df                            1
             Between  P                             *
             Btwn(F)  P                          N.S.

                        Derivation of RR/CI
                         Orig/2x2     Other     Total


                       N        2        26        28
                      NS        2        18        20


                      Wt    12.86   1389.59   1402.45
                 Het Chi     0.66     97.06     98.70
                 Het  df        1        25        27
                 Het  P      N.S.       ***       ***
               Fixed  RR     2.78      2.10      2.11
                     RRl     1.61      2.00      2.00
                     RRu     4.80      2.22      2.22
                      P       +++       +++       +++
              Random  RR     2.78      2.30      2.31
                     RRl     1.61      1.99      2.01
                     RRu     4.80      2.65      2.65
                      P       +++       +++       +++
             Between Chi                         0.99
             Between  df                            1
             Between  P                          N.S.
             Btwn(F)  P                          N.S.


  ________________________________________________________________________________________________________________________
                                            International Evidence on Smoking and COPD, Phase 3, Analysis run on 27-SEP-10

                                                   Table 2 - A - 5 - 4

            IESCOPD - Meta-analysis of ever smoking, any product (or cigarettes if all product not available)
                                             CB other than mortality/symptoms
                                                      Least-adjusted


     REF|NRR|X|SEX|AGEL|AGEH|     REGION|BEGYR|PUBYR|STTYP|ONSET|      DISEAS|ADJ|SMOKSTA|   PRODUCT|    UNEXP|

  ALDERS   1 x   m   35   74       Eu:UK  1977  1985    CC  Prev CB:diagnosed   0    Ever        Any   Nev any
  ALDERS   2 x   f   35   74       Eu:UK  1977  1985    CC  Prev CB:diagnosed   0    Ever MCigs only   Nev any
   BROWN   1     m   60   69       Eu:UK  1956  1957    CS  Prev CB:diagnosed   0    Ever        Any   Nev any
  COLLEG   3 x   m   40   64       Eu:UK     *  1961    CS  Prev CB:diagnosed   1    Ever        Any   Nev any
  COLLEG   9     f   40   64       Eu:UK     *  1961    CS  Prev CB:diagnosed   1    Ever        Any   Nev any
  DONTA2   3     m   25   84 Eu:SE/Balkn  1960  1984    Pr   Inc CB:diagnosed   0    Ever       Cigs  Nev cigs
  ENRIGH   3     m   65   99      Am:USA  1989  1994    CS  Prev CB:diagnosed   0    Ever       Cigs  Nev cigs
  ENRIGH   6     f   65   99      Am:USA  1989  1994    CS  Prev CB:diagnosed   0    Ever       Cigs  Nev cigs
  HARDIE   9     m   70   99    Eu:Scand  1998  2005    CS  Prev CB:diagnosed   1    Ever       Cigs  Nev cigs
  HARDIE  12     f   70   99    Eu:Scand  1998  2005    CS  Prev CB:diagnosed   1    Ever       Cigs  Nev cigs
      HO  11     b   70   99   Asia:FarE  1991  1999    CS  Prev  CB:self-rep   3    Ever       Cigs  Nev cigs
    KATO   3     m   40   99   Asia:FarE  1985  1989    CS  Prev  CB:self-rep   1    Ever       Cigs  Nev cigs
    KATO   6     f   40   99   Asia:FarE  1985  1989    CS  Prev  CB:self-rep   1    Ever       Cigs  Nev cigs
   KIRAZ   3 x   f   25   99 Eu:SE/Balkn  1999  2003    CS  Prev     CB:other   0    Ever       Cigs  Nev cigs
  KOTAN1   3 x   m   20   69    Eu:Scand  1995  2003    CS  Prev CB:diagnosed   0    Ever        Any   Nev any
  KOTAN1   6 x   f   20   69    Eu:Scand  1995  2003    CS  Prev CB:diagnosed   0    Ever        Any   Nev any
  LAVECC   3 x   m   15   99     Eu:West  1983  1988    CS  Prev  CB:self-rep   0    Ever        Any   Nev any
  LAVECC   6 x   f   15   99     Eu:West  1983  1988    CS  Prev  CB:self-rep   0    Ever        Any   Nev any
  LEBOWI  27 x   b   15   96      Am:USA  1972  1977    CS  Prev CB:diagnosed   2    Ever       Cigs  Nev cigs
  LUNDB2   3 x   m   35   66    Eu:Scand  1986  1993    CC  Prev     CB:other   0    Ever        Any   Nev any
  LUNDB2   9 x   f   35   66    Eu:Scand  1986  1993    CC  Prev     CB:other   0    Ever        Any   Nev any
  MELLST   3     m   70   70    Eu:Scand  1971  1982    CS  Prev     CB:other   0    Ever        Any   Nev any
  MILLER   3     m   15   99      Am:USA  1978  1988    CS  Prev CB:diagnosed   1    Ever       Cigs  Nev cigs
  MILLER   6     f   15   99      Am:USA  1978  1988    CS  Prev CB:diagnosed   1    Ever       Cigs  Nev cigs
  PELKON   6     m   40   75    Eu:Scand  1959  2006    Pr   Inc     CB:other   1    Ever       Cigs  Nev cigs
  SHIMUR   3     b   31   80   Asia:FarE     *  1996    CC  Prev     CB:other   0    Ever        Any   Nev any
   SILVA   6     b   20   99      Am:USA  1972  2004    Pr   Inc     CB:other   6    Ever       Cigs  Nev cigs
  TROISI   3     f   34   69      Am:USA  1980  1995    Pr   Inc CB:diagnosed   1    Ever       Cigs  Nev cigs
  VIEGI1   3     m   20   64     Eu:West  1980  1988    CS  Prev CB:diagnosed   0    Ever       Cigs   Nev any
  VIEGI1   6     f   20   64     Eu:West  1980  1988    CS  Prev CB:diagnosed   0    Ever       Cigs   Nev any


  ________________________________________________________________________________________________________________________
                                            International Evidence on Smoking and COPD, Phase 3, Analysis run on 27-SEP-10

                                                   Table 2 - A - 5 - 5

            IESCOPD - Meta-analysis of ever smoking, any product (or cigarettes if all product not available)
                                             CB other than mortality/symptoms
                                                      Least-adjusted


                        Number Exposed  Non-exposed
 REF    NRR SEX ADJ     Case    Cont    Case    Cont      RR        95.00%CI
 ALDERS 1   m   0        510     469      25      63      2.74 (  1.70-   4.43)
 ALDERS 2   f   0        333     239     105     203      2.69 (  2.02-   3.59)
 Subtotal ALDERS                                          2.71 (  2.11-   3.46)
 BROWN  1   m   0        238     725      15      76      1.66 (  0.94-   2.95)
 COLLEG 3   m   1          -       -       -       -      3.31 (  1.06-  10.33)
 COLLEG 9   f   1          -       -       -       -      2.64 (  1.54-   4.52)
 Subtotal COLLEG                                          2.75 (  1.69-   4.48)
*DONTA2 3   m   0         63     333       8     127      3.00 (  1.48-   6.09)
 ENRIGH 3   m   0         71    1432      17     699      2.04 (  1.19-   3.49)
 ENRIGH 6   f   0        102    1155      85    1558      1.62 (  1.20-   2.18)
 Subtotal ENRIGH                                          1.71 (  1.32-   2.22)
 HARDIE 9   m   1         37       -       1       -     10.90 (  2.06-  57.56)
 HARDIE 12  f   1         16       -      11       -      3.33 (  1.59-   6.98)
 Subtotal HARDIE                                          4.05 (  2.06-   7.96)
 HO     11  b   3          -       -       -       -      2.32 (  1.55-   3.46)
 KATO   3   m   1        827       -     118       -      1.33 (  1.09-   1.63)
 KATO   6   f   1        260       -     679       -      1.92 (  1.65-   2.23)
 Subtotal KATO                                            1.68 (  1.49-   1.90)
 KIRAZ  3   f   0         17      24      78     225      2.04 (  1.04-   4.00)
 KOTAN1 3   m   0         81    1949      29    1160      1.66 (  1.08-   2.56)
 KOTAN1 6   f   0         56    1416      43    1899      1.75 (  1.17-   2.61)
 Subtotal KOTAN1                                          1.71 (  1.27-   2.29)
 LAVECC 3   m   0       2053   18422     588   13724      2.60 (  2.37-   2.86)
 LAVECC 6   f   0        280    7357    1042   28818      1.05 (  0.92-   1.20)
 Subtotal LAVECC                                          1.93 (  1.79-   2.08)
 LEBOWI 27  b   2         37       -      26       -      1.12 (  0.68-   1.86)
 LUNDB2 3   m   0        232      91      15      41      6.97 (  3.68-  13.20)
 LUNDB2 9   f   0        105      58      35      73      3.78 (  2.26-   6.32)
 Subtotal LUNDB2                                          4.81 (  3.22-   7.18)
 MELLST 3   m   0        125     559      15     196      2.92 (  1.67-   5.11)
 MILLER 3   m   1         97       -      10       -      5.24 (  2.70-  10.17)
 MILLER 6   f   1         35       -       2       -     20.27 (  4.89-  84.04)
 Subtotal MILLER                                          6.67 (  3.66-  12.17)
*PELKON 6   m   1          -       -       -       -      1.76 (  1.32-   2.36)
 SHIMUR 3   b   0          3       2       3       2      1.00 (  0.08-  12.56)
*SILVA  6   b   6          -       -       -       -      1.76 (  0.99-   3.16)
*TROISI 3   f   1        545       -     253       -      1.93 (  1.66-   2.24)
 VIEGI1 3   m   0         32    1040       4     504      3.88 (  1.36-  11.02)
 VIEGI1 6   f   0          1     582       7    1119      0.27 (  0.03-   2.24)
 Subtotal VIEGI1                                          2.29 (  0.90-   5.84)
Partial Totals          6156   35853    3214   50487
*prospective study

                       N       30
                      NS       20


                      Wt  1444.16
                 Het Chi   203.47
                 Het  df       29
                 Het  P       ***
               Fixed  RR     1.95
                     RRl     1.86
                     RRu     2.06
                      P       +++
              Random  RR     2.24
                     RRl     1.89
                     RRu     2.65
                      P       +++
               Asymm  P      N.S.


  ________________________________________________________________________________________________________________________
                                            International Evidence on Smoking and COPD, Phase 3, Analysis run on 27-SEP-10

                                                   Table 2 - A - 5 - 6

            IESCOPD - Meta-analysis of ever smoking, any product (or cigarettes if all product not available)
                                             CB other than mortality/symptoms
                                                      Least-adjusted


                       N       30
                      NS       20


                      Wt  1444.16
                 Het Chi   203.47
                 Het  df       29
                 Het  P       ***
               Fixed  RR     1.95
                     RRl     1.86
                     RRu     2.06
                      P       +++
              Random  RR     2.24
                     RRl     1.89
                     RRu     2.65
                      P       +++
               Asymm  P      N.S.

                                   Sex
                             both      male    female     Total


                       N        4        14        12        30
                      NS        4        14        12        30


                      Wt    51.01    680.81    712.34   1444.16
                 Het Chi     5.10     65.74     93.64    203.47
                 Het  df        3        13        11        29
                 Het  P      N.S.       ***       ***       ***
               Fixed  RR     1.74      2.33      1.67      1.95
                     RRl     1.32      2.16      1.55      1.86
                     RRu     2.29      2.51      1.80      2.06
                      P       +++       +++       +++       +++
              Random  RR     1.68      2.55      2.11      2.24
                     RRl     1.13      1.99      1.62      1.89
                     RRu     2.49      3.26      2.75      2.65
                      P         +       +++       +++       +++
             Between Chi                                  38.99
             Between  df                                      2
             Between  P                                     ***
             Btwn(F)  P                                     (*)


  ________________________________________________________________________________________________________________________
                                            International Evidence on Smoking and COPD, Phase 3, Analysis run on 27-SEP-10

                                                   Table 2 - A - 5 - 7

            IESCOPD - Meta-analysis of ever smoking, any product (or cigarettes if all product not available)
                                             CB other than mortality/symptoms
                                 Excluded studies (and stage at which they were excluded)


1       CLARK COTTON  MEYER REMYJA RUTGER SNYDER SOBRAX     SU TAKEMU  WANG4   WEIR WHICKE ZALACA
2      ALESSA  AMIGO ANDER2 ANDER3 AUERBA BEDNAR BROGGE  CHEN1  CHEN2  CHEN3  CHENG CLEMEN  COCCI  DEAN1 DEJONG DETORR
       DICKIN DONTA1 EKBERG ENSTRO FERRI2 FERRI3  FIDAN FORAST FUKUCH GEIJER GODTFR GULSVI HAMMO2 HARIKK HEDMAN HIGGI4
       HOZAWA ITABAS JACOBS JAENDI JOHANN KACHEL KARAKA KATANC KHOURY    KIM KLAYTO KOJIMA KOTAN2 KRZYZA KULLER    LAI
         LAM1   LAM2   LAM3  LANGE    LEE   LIAW LINDBE   LIU1   LIU2 LUNDB1  MADOR MANNI1 MANNI2 MANNI3 MARAN1 MARAN2
       MARCUS MATHES MENEZ2 MENEZ3 MENEZ4 MENEZ5 MENEZ6 MONTNE   NAWA NIEPSU NIHLEN NILSSO  OMORI   PEAT   PETO  PRATT
        PRICE RENWIC RICCIO  RYDER SARGEA SHAHAB   SHIN SICHLE SPEIZE STERLI  STROM SUTINE  TAGER   TANG   THUN   TODD
       TRUPIN TSUSHI TVERDA VESTBO VIEGI2 VIKGRE VINEIS VOLLM1 VOLLM2 VONHER   WALD  WANG2 WATSON  WEISS WILSO1   XIAO
           XU   YUAN ZIELI1 ZIELI2 ZIETKO
3       DOLL2 FLETCH HRUBEC JENSEN OSWAL1 RIMING SCHWAR  SHARP SUADIC    WIG WILSO2
4        BANG  DEAN2 HIRAYA   KAHN LANGE2 TAGER2    WEN WOJTYN
5      ANDER1  BECK1  BECK2   BEST BJORNS CERVER CHAPMA COATES  DEANE DEMARC  DOLL1 DOPICO EHRLIC FERRI1 FINKLE FOXMAN
       GOLDBE HAENSZ HARRIS HAWTHO  HAYES HIGGI2 HIGGI3 HIGGI6 HOLLA2 HOLLNA  HOUSE HUCHON HUHTI1 HUHTI2 HUHTI3 JINDA2
        JOSHI JOUSI1  KAHN2  KUBIK LAMBER LANGHA LINDST MAGNUS MANFRE MENEZ1  MEREN  MILNE MOLLER MUELLE NEJJAR OGILVI
       OSWAL2 PANDEY PEREZP   REID SAWICK SOBRAD STJERN URRUTI WAGEN2 WILHEL  WOODS  WOOLF YAMAGU   ZOIA


  ________________________________________________________________________________________________________________________
                                            International Evidence on Smoking and COPD, Phase 3, Analysis run on 27-SEP-10

                                                   Table 2 - A - 5 - 8

            IESCOPD - Meta-analysis of ever smoking, any product (or cigarettes if all product not available)
                                             CB other than mortality/symptoms
                                             Potentially overlapping studies


     REF| REFGP|PRINC|                     OVERLAP|

  ENRIGH ENRIGH     1         ENRIGH/HOZAWA/HARIKK
  DONTA2 JACOBS     2  JACOBS/DONTA1/DONTA2/PELKON
  PELKON JACOBS     2  JACOBS/DONTA1/DONTA2/PELKON
  LUNDB2 LUNDBA     2  LINDBE/LUNDB1/LUNDB2/HEDLUN
  HARDIE HARDIE     1         HARDIE/JOHANN/BROGGE
  LEBOWI LEBOWI     1                 LEBOWI/SILVA
   SILVA  SILVA     1                 LEBOWI/SILVA
  KOTAN1 KOTAN1     1                KOTAN1/KOTAN2


  ________________________________________________________________________________________________________________________
                                            International Evidence on Smoking and COPD, Phase 3, Analysis run on 27-SEP-10

                                                    Table 2 - B - 1 -

           IESCOPD - Meta-analysis of current smoking, any product (or cigarettes if all product not available)
                                                          Any CB


This analysis is restricted to results for:
1) Eligible study on database
2) Outcome CB
3) Non-dose-response data
4) Current smoking
5) Results complete enough for use in meta-analysis

Within each study, results are then selected (in the following order of preference, within each sex) for:
6) UNEXP   : never any, never cigarettes, other
7) PROD    : any product, cigarettes, cigarettes only
8) For overlapping studies: principal rather than subsidiary studies
and then for single sex results (m, f) in preference to results for both sexes combined (b).

Results adjusted for the most potential confounders are then chosen in Sections -1 to -3
and results adjusted for the least confounders in Sections -4 to -6. (Those least adjusted results which
actually differ from the most adjusted are marked 'x' in column X in Section -4)

Section -7 shows excluded studies, together with the stage (as above) at which no qualifying
results were found.

Section -8 lists the potentially overlapping studies which have been included (1=principal, 2=subsidiary),
and any results which would have been included in preference except that they had data not complete enough
for use in meta-analysis. It also lists their significance (yes/no), if known.


  ________________________________________________________________________________________________________________________
                                            International Evidence on Smoking and COPD, Phase 3, Analysis run on 27-SEP-10

                                                   Table 2 - B - 1 - 1

           IESCOPD - Meta-analysis of current smoking, any product (or cigarettes if all product not available)
                                                          Any CB
                                                      Most-adjusted


     REF|NRR|SEX|AGEL|AGEH|     REGION|BEGYR|PUBYR|STTYP|ONSET|      DISEAS|ADJ|SMOKSTA|   PRODUCT|    UNEXP|

  ALDERS   9   m   35   74       Eu:UK  1977  1985    CC  Prev CB:diagnosed   1 Current MCigs only   Nev any
  ALDERS  10   f   35   74       Eu:UK  1977  1985    CC  Prev CB:diagnosed   1 Current MCigs only   Nev any
  ANDER1  30   m   25   74   Am:Canada  1963  1965    CS  Prev  CB:symptoms   1 Current        Any   Nev any
  ANDER1  33   f   25   74   Am:Canada  1963  1965    CS  Prev  CB:symptoms   1 Current        Any   Nev any
    BANG   1   b   12   74      Am:USA  1982  1990    CS  Prev CB:diagnosed   4 Current       Cigs  Nev cigs
   BECK1   1   m   11   99      Am:USA  1972  1982    CS  Prev  CB:symptoms   0 Current       Cigs   Nev any
   BECK1   4   f   11   99      Am:USA  1972  1982    CS  Prev  CB:symptoms   0 Current       Cigs   Nev any
   BECK2   1   m   11   99      Am:USA  1972  1982    Pr   Inc  CB:symptoms   0 Current       Cigs   Nev any
   BECK2   4   f   11   99      Am:USA  1972  1982    Pr   Inc  CB:symptoms   0 Current       Cigs   Nev any
    BEST   1   m   30   97   Am:Canada  1955  1967    Pr   Inc CB:mortality   1 Current  Cigs only   Nev any
  BJORNS   4   b   20   44    Eu:Scand  1990  1994    CS  Prev  CB:symptoms   4 Current        Any   Nev any
   BROWN   4   m   60   69       Eu:UK  1956  1957    CS  Prev CB:diagnosed   0 Current  Cigs only   Nev any
  CERVER   4   b   20   44     Eu:West  1998  2003    CS  Prev  CB:symptoms   6 Current       Cigs  Nev cigs
  COATES   1   b   40   64      Am:USA  1962  1965    CS  Prev  CB:symptoms   0 Current       Cigs  Nev cigs
  COLLEG   4   m   40   64       Eu:UK     *  1961    CS  Prev CB:diagnosed   2 Current        Any   Nev any
  COLLEG   7   f   40   64       Eu:UK     *  1961    CS  Prev CB:diagnosed   1 Current        Any   Nev any
   DEAN2   1   m   37   67       Eu:UK  1972  1978    CS  Prev  CB:symptoms   1 Current        Any   Nev any
   DEAN2   3   f   37   67       Eu:UK  1972  1978    CS  Prev  CB:symptoms   1 Current        Any   Nev any
   DEANE   2   m   40   59      Am:USA  1963  1965    CS  Prev  CB:symptoms   1 Current        Any   Nev any
  DEMARC  18   b   20   44       Multi  1991  2004    CS  Prev  CB:symptoms   1 Current       Cigs   Nev any
   DOLL1  14   m   20   99       Eu:UK  1951  1994    Pr   Inc CB:mortality   1 Current       Cigs   Nev any
  DONTA2   1   m   25   84 Eu:SE/Balkn  1960  1984    Pr   Inc CB:diagnosed   0 Current       Cigs  Nev cigs
  DOPICO   1   m   15    *      Am:USA     *  1984    CS  Prev  CB:symptoms   0 Current       Cigs  Nev cigs
  EHRLIC  27   m   15   99      Africa  1998  2004    CS  Prev  CB:symptoms   6 Current        Any   Nev any
  EHRLIC  28   f   15   99      Africa  1998  2004    CS  Prev  CB:symptoms   6 Current        Any   Nev any
  ENRIGH   1   m   65   99      Am:USA  1989  1994    CS  Prev CB:diagnosed   0 Current       Cigs  Nev cigs
  ENRIGH   4   f   65   99      Am:USA  1989  1994    CS  Prev CB:diagnosed   0 Current       Cigs  Nev cigs
  FERRI1  59   m   25   74      Am:USA  1961  1971    CS  Prev  CB:symptoms   1 Current        Any   Nev any
  FERRI1  80   f   25   74      Am:USA  1961  1971    CS  Prev  CB:symptoms   1 Current        Any   Nev any
  FOXMAN   1   m   20   69      Am:USA     *  1986    CS  Prev  CB:symptoms   1 Current        Any   Nev any
  FOXMAN   4   f   20   69      Am:USA     *  1986    CS  Prev  CB:symptoms   1 Current        Any   Nev any
  GOLDBE   4   m   15   99      Am:USA  1970  1974    CS  Prev  CB:symptoms   1 Current       Cigs  Nev cigs
  GOLDBE  10   f   15   99      Am:USA  1970  1974    CS  Prev  CB:symptoms   1 Current       Cigs  Nev cigs
  HAENSZ   8   m   35   74    Eu:Scand  1964  1972    CS  Prev  CB:symptoms   1 Current        Any   Nev any
  HAENSZ  30   f   35   74    Eu:Scand  1964  1972    CS  Prev  CB:symptoms   1 Current        Any   Nev any
  HARDIE   7   m   70   99    Eu:Scand  1998  2005    CS  Prev CB:diagnosed   1 Current       Cigs  Nev cigs
  HARDIE  10   f   70   99    Eu:Scand  1998  2005    CS  Prev CB:diagnosed   1 Current       Cigs  Nev cigs
  HARRIS   1   m   15   60      Africa     *  1993    CS  Prev  CB:symptoms   0 Current        Any   Nev any
  HAWTHO   8   m   45   76       Eu:UK  1965  1978    Pr  Prev  CB:symptoms   0 Current       Cigs  N/L cigs
  HAWTHO  15   f   45   76       Eu:UK  1965  1978    Pr  Prev  CB:symptoms   0 Current       Cigs  N/L cigs
   HAYES   7   b   15   99      Am:USA  1970  1974    CS  Prev  CB:symptoms   3 Current       Cigs  Nev cigs
  HIGGI2   1   m   25   74       Eu:UK  1956  1957    CS  Prev  CB:symptoms   0 Current       Cigs  Nev cigs
  HIGGI2  13   f   25   74       Eu:UK  1956  1957    CS  Prev  CB:symptoms   1 Current       Cigs  Nev cigs
  HIGGI3   1   m   55   64       Eu:UK  1956  1958    CS  Prev  CB:symptoms   0 Current        Any   Nev any
  HIGGI3   7   f   55   64       Eu:UK  1956  1958    CS  Prev  CB:symptoms   0 Current        Any   Nev any
  HIGGI6   1   m   25   64      Am:USA  1962  1977    CS  Prev  CB:symptoms   1 Current        Any   Nev any
  HIGGI6   8   f   25   64      Am:USA  1962  1977    CS  Prev  CB:symptoms   1 Current        Any   Nev any
      HO   7   b   70   99   Asia:FarE  1991  1999    CS  Prev  CB:self-rep   3 Current       Cigs  Nev cigs
  HOLLA2   1   m   40   59      Am:USA  1962  1965    CS  Prev  CB:symptoms   0 Current        Any   Nev any
  HOLLNA   1   m   40   40    Eu:Scand  1976  1983    CS  Prev  CB:symptoms   0 Current        Any   Nev any
  HOLLNA   9   f   40   40    Eu:Scand  1976  1983    CS  Prev  CB:symptoms   0 Current        Any   Nev any
   HOUSE   4   m   15   99      Am:USA  1970  1974    CS  Prev  CB:symptoms   1 Current       Cigs  Nev cigs
   HOUSE  10   f   15   99      Am:USA  1970  1974    CS  Prev  CB:symptoms   1 Current       Cigs  Nev cigs
  HUCHON   4   b   25   99     Eu:West     *  2002    CS  Prev  CB:symptoms   1 Current       Cigs  Nev cigs
  HUHTI1  40   m   40   64    Eu:Scand  1961  1965    CS  Prev  CB:symptoms   1 Current        Any   Nev any
  HUHTI1  89   f   40   64    Eu:Scand  1961  1965    CS  Prev  CB:symptoms   1 Current        Any   Nev any
  HUHTI3  20   m   25   69    Eu:Scand  1968  1978    CS  Prev  CB:symptoms   1 Current        Any   Nev any
   JOSHI   1   m   17   64 Asia:MidE/S     *  1975    CS  Prev  CB:symptoms   0 Current        Any   Nev any
  JOUSI1   1   m   25   64    Eu:Scand  1972  1996    CS  Prev  CB:symptoms   0 Current        Any   Nev any
  JOUSI1   4   f   25   64    Eu:Scand  1972  1996    CS  Prev  CB:symptoms   0 Current        Any   Nev any
   KAHN2   1   m   31   84      Am:USA  1954  1966    Pr   Inc CB:mortality   1 Current        Any   Nev any
    KATO   1   m   40   99   Asia:FarE  1985  1989    CS  Prev  CB:self-rep   1 Current       Cigs  Nev cigs
    KATO   4   f   40   99   Asia:FarE  1985  1989    CS  Prev  CB:self-rep   1 Current       Cigs  Nev cigs
  KOTAN1   7   b   20   69    Eu:Scand  1995  2003    CS  Prev CB:diagnosed   7 Current        Any   Nev any
  LAMBER   4   m   35   69       Eu:UK  1965  1970    CS  Prev  CB:symptoms   1 Current       Cigs  Nev cigs
  LAMBER  36   f   35   69       Eu:UK  1965  1970    CS  Prev  CB:symptoms   1 Current        Any   Nev any
  LANGE2   1   b   65   99    Eu:Scand  1991  2003    CS  Prev  CB:symptoms   5 Current        Any   Nev any
  LANGHA   4   m   20   99    Eu:Scand  1995  2000    CS  Prev  CB:symptoms   1 Current        Any   Nev any
  LANGHA  10   f   20   99    Eu:Scand  1995  2000    CS  Prev  CB:symptoms   1 Current        Any   Nev any
  LAVECC   7   b   15   99     Eu:West  1983  1988    CS  Prev  CB:self-rep   6 Current        Any   Nev any
  LEBOWI  28   b   15   96      Am:USA  1972  1977    CS  Prev CB:diagnosed   3 Current       Cigs  Nev cigs
  LINDST  21   b   20   69    Eu:Scand     *  2001    CS  Prev  CB:symptoms   5 Current       Cigs  Nev cigs
  ________________________________________________________________________________________________________________________
                                            International Evidence on Smoking and COPD, Phase 3, Analysis run on 27-SEP-10

                                                   Table 2 - B - 1 - 1

           IESCOPD - Meta-analysis of current smoking, any product (or cigarettes if all product not available)
                                                          Any CB
                                                      Most-adjusted


     REF|NRR|SEX|AGEL|AGEH|     REGION|BEGYR|PUBYR|STTYP|ONSET|      DISEAS|ADJ|SMOKSTA|   PRODUCT|    UNEXP|

  LUNDB2   4   m   35   66    Eu:Scand  1986  1993    CC  Prev     CB:other   1 Current        Any   Nev any
  LUNDB2  10   f   35   66    Eu:Scand  1986  1993    CC  Prev     CB:other   1 Current        Any   Nev any
  MAGNUS   4   m   50   80    Eu:Scand  1993  1999    CS  Prev  CB:symptoms   1 Current        Any   Nev any
  MANFRE   1   m   20   65   Am:Canada  1978  1989    CS  Prev  CB:symptoms   0 Current        Any   Nev any
  MANFRE   4   f   20   65   Am:Canada  1978  1989    CS  Prev  CB:symptoms   0 Current        Any   Nev any
  MELLST   1   m   70   70    Eu:Scand  1971  1982    CS  Prev     CB:other   0 Current        Any   Nev any
  MENEZ1   4   b   40   99 Am:Sth/Cent  1990  1995    CS  Prev  CB:symptoms   8 Current       Cigs  Nev cigs
   MEREN   5   b   15   64     Eu:East  1995  2001    CS  Prev  CB:symptoms   4 Current       Cigs  Nev cigs
  MILLER   1   m   15   99      Am:USA  1978  1988    CS  Prev CB:diagnosed   1 Current       Cigs  Nev cigs
  MILLER   4   f   15   99      Am:USA  1978  1988    CS  Prev CB:diagnosed   1 Current       Cigs  Nev cigs
   MILNE   1   m   62   90       Eu:UK  1968  1972    CS  Prev  CB:symptoms   0 Current       Cigs  Nev cigs
   MILNE  13   f   62   90       Eu:UK  1968  1972    CS  Prev  CB:symptoms   0 Current        Any   Nev any
  MOLLER   1   b   40   72     Eu:West     *  2001    CC  Prev  CB:symptoms   0 Current       Cigs  Nev cigs
  MUELLE  34   m   20   69      Am:USA  1967  1971    CS  Prev  CB:symptoms   1 Current        Any   Nev any
  MUELLE  50   f   20   69      Am:USA  1967  1971    CS  Prev  CB:symptoms   1 Current        Any   Nev any
  NEJJAR   3   b   65   99     Eu:West  1991  1996    CS  Prev  CB:symptoms   6 Current       Cigs  Nev cigs
  OGILVI   1   m   30   99       Eu:UK  1955  1957    CC  Prev  CB:symptoms   0 Current        Any   Nev any
  OGILVI   6   f   30   99       Eu:UK  1955  1957    CC  Prev  CB:symptoms   0 Current        Any   Nev any
  PANDEY   4   m   20   99 Asia:MidE/S  1979  1984    CS  Prev  CB:symptoms   2 Current        Any   Nev any
  PANDEY  10   f   20   99 Asia:MidE/S  1979  1984    CS  Prev  CB:symptoms   2 Current        Any   Nev any
  PELKON   4   m   40   75    Eu:Scand  1959  2006    Pr   Inc     CB:other   1 Current       Cigs  Nev cigs
    REID   4   m   35   74      Am:USA  1962  1966    CS  Prev  CB:symptoms   1 Current       Cigs  Nev cigs
    REID  20   f   35   74      Am:USA  1962  1966    CS  Prev  CB:symptoms   1 Current       Cigs  Nev cigs
  SAWICK   3   m   19   70     Eu:East  1968  1972    CS  Prev  CB:symptoms   0 Current        Any   Nev any
  SAWICK  16   f   19   70     Eu:East  1968  1972    CS  Prev  CB:symptoms   0 Current        Any   Nev any
  SHIMUR   1   b   31   80   Asia:FarE     *  1996    CC  Prev     CB:other   0 Current        Any   Nev any
   SILVA   4   b   20   99      Am:USA  1972  2004    Pr   Inc     CB:other   6 Current       Cigs  Nev cigs
  SOBRAD   1   b   40   69     Eu:West  1996  2000    CS  Prev  CB:symptoms   0 Current        Any   Nev any
  STJERN   1   b   16   72    Eu:Scand  1981  1985    CS  Prev  CB:symptoms   0 Current        Any   Nev any
  TAGER2   2   m   15   99      Am:USA  1973  1976    CS  Prev  CB:symptoms   1 Current       Cigs  Nev cigs
  TAGER2  11   f   15   99      Am:USA  1973  1976    CS  Prev  CB:symptoms   1 Current       Cigs  Nev cigs
  TROISI   1   f   34   69      Am:USA  1980  1995    Pr   Inc CB:diagnosed   1 Current       Cigs  Nev cigs
  VIEGI1   1   m   20   64     Eu:West  1980  1988    CS  Prev CB:diagnosed   0 Current       Cigs   Nev any
  VIEGI1   4   f   20   64     Eu:West  1980  1988    CS  Prev CB:diagnosed   0 Current       Cigs   Nev any
  WAGEN2   9   b   21   68     Eu:West  2001  2004    CC  Prev  CB:symptoms   1 Current       Cigs  Nev cigs
     WEN   7   m   35   99   Asia:FarE  1982  2004    Pr   Inc CB:mortality   1 Current       Cigs  Nev cigs
  WILHEL   1   m   54   54    Eu:Scand  1967  1969    CS  Prev  CB:symptoms   0 Current        Any   Nev any
   WOODS   1   b   20   44      Aus/NZ     *  2000    CS  Prev  CB:symptoms   5 Current       Cigs  Nev cigs
   WOOLF   1   f   25   54   Am:Canada     *  1974    CS  Prev  CB:symptoms   0 Current       Cigs  Nev cigs
  YAMAGU   7   b   40   99   Asia:FarE  1986  1988    CS  Prev  CB:symptoms   6 Current       Cigs  Nev cigs
    ZOIA   1   b   18   69     Eu:West     *  1995    CS  Prev  CB:symptoms   4 Current        Any   Nev any


  ________________________________________________________________________________________________________________________
                                            International Evidence on Smoking and COPD, Phase 3, Analysis run on 27-SEP-10

                                                   Table 2 - B - 1 - 2

           IESCOPD - Meta-analysis of current smoking, any product (or cigarettes if all product not available)
                                                          Any CB
                                                      Most-adjusted


                        Number Exposed  Non-exposed
 REF    NRR SEX ADJ     Case    Cont    Case    Cont      RR        95.00%CI
 ALDERS 9   m   1        127       -      25       -      3.03 (  1.78-   5.17)
 ALDERS 10  f   1        172       -     105       -      3.45 (  2.46-   4.84)
 Subtotal ALDERS                                          3.32 (  2.50-   4.42)
 ANDER1 30  m   1         38       -       4       -      5.86 (  1.95-  17.63)
 ANDER1 33  f   1         16       -      15       -      2.56 (  1.21-   5.41)
 Subtotal ANDER1                                          3.33 (  1.79-   6.18)
 BANG   1   b   4          -       -       -       -      1.80 (  1.26-   2.55)
 BECK1  1   m   0         16     123       3     189      8.20 (  2.34-  28.71)
 BECK1  4   f   0          8     127       6     318      3.34 (  1.14-   9.81)
 Subtotal BECK1                                           4.89 (  2.16-  11.08)
*BECK2  1   m   0          3     123       2     189      2.30 (  0.39-  13.60)
*BECK2  4   f   0          4     128       3     318      3.31 (  0.75-  14.59)
 Subtotal BECK2                                           2.85 (  0.91-   8.90)
*BEST   1   m   1         64       -       3       -     11.25 (  3.54-  35.80)
 BJORNS 4   b   4        717       -     354       -      3.10 (  2.70-   3.60)
 BROWN  4   m   0        166     376      15      76      2.24 (  1.25-   4.01)
 CERVER 4   b   6       1233       -     754       -      3.07 (  2.78-   3.38)
 COATES 1   b   0        126     712      21     515      4.34 (  2.70-   6.98)
 COLLEG 4   m   2          -       -       -       -      3.05 (  1.03-   8.99)
 COLLEG 7   f   1          -       -       -       -      2.59 (  1.47-   4.55)
 Subtotal COLLEG                                          2.68 (  1.63-   4.43)
 DEAN2  1   m   1        302       -      39       -      2.28 (  1.62-   3.21)
 DEAN2  3   f   1        184       -      80       -      3.04 (  2.32-   3.97)
 Subtotal DEAN2                                           2.72 (  2.21-   3.36)
 DEANE  2   m   1        100       -       3       -      7.92 (  2.34-  26.74)
 DEMARC 18  b   1        298       -      91       -      4.07 (  3.20-   5.18)
*DOLL1  14  m   1         82       -       3       -      9.41 (  2.81-  31.49)
*DONTA2 1   m   0         57     275       8     127      3.29 (  1.62-   6.69)
 DOPICO 1   m   0         32      74       6      57      4.11 (  1.61-  10.49)
 EHRLIC 27  m   6         88       -      33       -      1.58 (  0.92-   2.72)
 EHRLIC 28  f   6         48       -     138       -      2.00 (  1.29-   3.09)
 Subtotal EHRLIC                                          1.82 (  1.30-   2.56)
 ENRIGH 1   m   0         15     216      17     699      2.86 (  1.40-   5.81)
 ENRIGH 4   f   0         30     352      85    1558      1.56 (  1.01-   2.41)
[truncated: 1,027,064 more chars]
