# Supplementary material for: Systematic review with meta-analysis of the epidemiological evidence relating smoking to COPD, chronic bronchitis and emphysema
Source: BMC Pulm Med. 2011 Jun 14;11:36. doi: 10.1186/1471-2466-11-36 (PMC3128042; doi:10.1186/1471-2466-11-36)
Supplement: Additional file 6 — MetaMajorEMP. .RTF file giving the full results of the meta-analyses for the major smoking variables for EMP. [file 1471-2466-11-36-S6.RTF]

Systematic review with meta-analysis of the epidemiological evidence relating smoking to COPD, chronic bronchitis and emphysema

Barbara A Forey, Alison J Thornton and Peter N Lee

Additional file 6 : MetaMajorEMP

See Additional file 10 (Intro sheet) for list of tables and page numbers


                                                    Table 3 - A - 1 -

            IESCOPD - Meta-analysis of ever smoking, any product (or cigarettes if all product not available)
                                                      Any Emphysema


This analysis is restricted to results for:
1) Eligible study on database
2) Outcome Emphysema
3) Non-dose-response data
4) Ever smoking
5) Results complete enough for use in meta-analysis

Within each study, results are then selected (in the following order of preference, within each sex) for:
6) UNEXP   : never any, never cigarettes, other
7) PROD    : any product, cigarettes, cigarettes only
8) For overlapping studies: principal rather than subsidiary studies
and then for single sex results (m, f) in preference to results for both sexes combined (b).

Results adjusted for the most potential confounders are then chosen in Sections -1 to -3
and results adjusted for the least confounders in Sections -4 to -6. (Those least adjusted results which
actually differ from the most adjusted are marked 'x' in column X in Section -4)

Section -7 shows excluded studies, together with the stage (as above) at which no qualifying
results were found.

Section -8 lists the potentially overlapping studies which have been included (1=principal, 2=subsidiary),
and any results which would have been included in preference except that they had data not complete enough
for use in meta-analysis. It also lists their significance (yes/no), if known.


  ________________________________________________________________________________________________________________________
                                            International Evidence on Smoking and COPD, Phase 3, Analysis run on 27-SEP-10

                                                   Table 3 - A - 1 - 1

            IESCOPD - Meta-analysis of ever smoking, any product (or cigarettes if all product not available)
                                                      Any Emphysema
                                                      Most-adjusted


     REF|NRR|SEX|AGEL|AGEH|     REGION|BEGYR|PUBYR|STTYP|ONSET|      DISEAS|ADJ|SMOKSTA|   PRODUCT|    UNEXP|

  ANDER2   1   b   15   99      Am:USA     *  1966    CS  Prev  Emp:viscomp   0    Ever  Cigs only   Nev any
    BEST  20   m   30   97   Am:Canada  1955  1967    Pr   Inc     Emp:mort   1    Ever  Cigs only   Nev any
  DONTA2  13   m   25   84 Eu:SE/Balkn  1960  1984    Pr   Inc Emp:diagnosd   0    Ever       Cigs  Nev cigs
  ENRIGH   9   m   65   99      Am:USA  1989  1994    CS  Prev Emp:diagnosd   0    Ever       Cigs  Nev cigs
  ENRIGH  12   f   65   99      Am:USA  1989  1994    CS  Prev Emp:diagnosd   0    Ever       Cigs  Nev cigs
  GULSVI   4   m   15   70    Eu:Scand  1972  1979    CS  Prev Emp:diagnosd   1    Ever        Any   Nev any
  GULSVI   6   f   15   70    Eu:Scand  1972  1979    CS  Prev Emp:diagnosd   1    Ever        Any   Nev any
  HAMMO2   7   m   35   99      Am:USA  1959  1966    Pr   Inc     Emp:mort   1    Ever       Cigs   Nev any
  HAMMO2   8   f   35   99      Am:USA  1959  1966    Pr   Inc     Emp:mort   1    Ever        Any   Nev any
  HARDIE  15   m   70   99    Eu:Scand  1998  2005    CS  Prev Emp:diagnosd   1    Ever       Cigs  Nev cigs
  HARDIE  18   f   70   99    Eu:Scand  1998  2005    CS  Prev Emp:diagnosd   1    Ever       Cigs  Nev cigs
      HO  12   b   70   99   Asia:FarE  1991  1999    CS  Prev Emp:self-rep   3    Ever       Cigs  Nev cigs
  HOZAWA   9   b   45   64      Am:USA  1987  2006    CS  Prev Emp:diagnosd   0    Ever       Cigs  Nev cigs
  HUHTI1  69   m   40   64    Eu:Scand  1961  1965    CS  Prev    Emp:other   0    Ever        Any   Nev any
  HUHTI1 145   f   40   64    Eu:Scand  1961  1965    CS  Prev    Emp:other   1    Ever        Any   Nev any
   KAHN2  80   m   31   84      Am:USA  1954  1966    Pr   Inc     Emp:mort   1    Ever        Any   Nev any
  LAVECC  30   b   15   99     Eu:West  1983  1988    CS  Prev Emp:self-rep   6    Ever        Any   Nev any
  LEBOWI  36   b   15   96      Am:USA  1972  1977    CS  Prev Emp:diagnosd   3    Ever       Cigs  Nev cigs
  MILLER   9   m   15   99      Am:USA  1978  1988    CS  Prev Emp:diagnosd   1    Ever       Cigs  Nev cigs
  MILLER  12   f   15   99      Am:USA  1978  1988    CS  Prev Emp:diagnosd   1    Ever       Cigs  Nev cigs
    NAWA   1   m   50   69   Asia:FarE  1998  2002    CS  Prev  Emp:viscomp   0    Ever       Cigs  Nev cigs
   OMORI   6   m   40   69   Asia:FarE     *  2006    CS  Prev  Emp:viscomp   1    Ever       Cigs  Nev cigs
   PRATT   1   m   15   99      Am:USA     *  1980    CS  Prev  Emp:viscomp   0    Ever        Any   Nev any
   RYDER   3   b   22   95       Eu:UK     *  1971    CS  Prev  Emp:viscomp   1    Ever        Any   Nev any
   SILVA   9   b   20   99      Am:USA  1972  2004    Pr   Inc    Emp:other   6    Ever       Cigs  Nev cigs
  SUTINE   1   b   10   99    Eu:Scand  1971  1978    CS  Prev  Emp:viscomp   0    Ever        Any   Nev any
   WANG2   1   b   19   92   Asia:FarE  1996  2001    CS  Prev  Emp:viscomp   0    Ever       Cigs  Nev cigs
   WEISS  16   m   50   69      Am:USA  1961  1963    CS  Prev  Emp:viscomp   0    Ever        Any   Nev any


  ________________________________________________________________________________________________________________________
                                            International Evidence on Smoking and COPD, Phase 3, Analysis run on 27-SEP-10

                                                   Table 3 - A - 1 - 2

            IESCOPD - Meta-analysis of ever smoking, any product (or cigarettes if all product not available)
                                                      Any Emphysema
                                                      Most-adjusted


                        Number Exposed  Non-exposed
 REF    NRR SEX ADJ     Case    Cont    Case    Cont      RR        95.00%CI
 ANDER2 1   b   0        102      12      30      21      5.95 (  2.63-  13.48)
*BEST   20  m   1         52       -       3       -      8.47 (  2.65-  27.11)
*DONTA2 13  m   0         11     333       4     127      1.05 (  0.34-   3.23)
 ENRIGH 9   m   0        113    1390      22     694      2.56 (  1.61-   4.09)
 ENRIGH 12  f   0         75    1182      26    1617      3.95 (  2.51-   6.20)
 Subtotal ENRIGH                                          3.20 (  2.31-   4.43)
 GULSVI 4   m   1         40       -       4       -      2.37 (  0.84-   6.66)
 GULSVI 6   f   1         28       -      16       -      1.82 (  0.98-   3.38)
 Subtotal GULSVI                                          1.95 (  1.15-   3.32)
*HAMMO2 7   m   1        369       -      20       -      8.64 (  5.51-  13.55)
*HAMMO2 8   f   1         24       -      21       -      6.44 (  3.57-  11.63)
 Subtotal HAMMO2                                          7.76 (  5.42-  11.09)
 HARDIE 15  m   1         48       -       2       -     13.38 (  3.14-  56.98)
 HARDIE 18  f   1         11       -      11       -      2.55 (  1.18-   5.48)
 Subtotal HARDIE                                          3.67 (  1.86-   7.23)
 HO     12  b   3          -       -       -       -      2.87 (  1.42-   5.81)
 HOZAWA 9   b   0        185    8775      13    6533     10.59 (  6.03-  18.61)
 HUHTI1 69  m   0         64     467       1     121     16.58 (  2.28- 120.74)
 HUHTI1 145 f   1          3       -      16       -      1.97 (  0.54-   7.17)
 Subtotal HUHTI1                                          3.72 (  1.26-  10.98)
*KAHN2  80  m   1        408       -      18       -      9.11 (  5.68-  14.61)
 LAVECC 30  b   6        789       -     595       -      2.05 (  1.81-   2.33)
 LEBOWI 36  b   3         31       -       5       -      4.86 (  1.86-  12.74)
 MILLER 9   m   1          6       -       0       -      6.56 (  0.37- 116.09)
 MILLER 12  f   1          2       -       1       -      6.03 (  0.12- 308.75)
 Subtotal MILLER                                          6.37 (  0.63-  64.77)
 NAWA   1   m   0        654    4086      32    1372      6.86 (  4.79-   9.84)
 OMORI  6   m   1        135       -       3       -      9.73 (  3.18-  29.82)
 PRATT  1   m   0        178     246      15      82      3.96 (  2.21-   7.09)
 RYDER  3   b   1         80       -      21       -     11.69 (  5.16-  26.51)
*SILVA  9   b   6          -       -       -       -      2.10 (  1.09-   4.06)
 SUTINE 1   b   0         63      28      28      45      3.62 (  1.89-   6.92)
 WANG2  1   b   0        188    3408      42    4209      5.53 (  3.94-   7.75)
 WEISS  16  m   0         28     223       1      35      4.39 (  0.58-  33.34)
Partial Totals          3687   20150     950   14856
*prospective study


 REF    NRR SEX ADJ             Ys       Ws       Qs       Ps
 ANDER2 1   b   0              1.78     5.74     2.17       0.00
*BEST   20  m   1              2.14     2.84     2.66       0.00
*DONTA2 13  m   0              0.05     3.03     3.81       0.93
 ENRIGH 9   m   0              0.94    17.71     0.92       0.00
 ENRIGH 12  f   0              1.37    18.78     0.78       0.00
 Subtotal ENRIGH               1.16    36.49     1.69
 GULSVI 4   m   1              0.86     3.58     0.34       0.10
 GULSVI 6   f   1              0.60    10.02     3.26       0.06
 Subtotal GULSVI               0.67    13.61     3.60
*HAMMO2 7   m   1              2.16    18.98    18.50       0.00
*HAMMO2 8   f   1              1.86    11.02     5.30       0.00
 Subtotal HAMMO2               2.05    29.99    23.79
 HARDIE 15  m   1              2.59     1.83     3.71       0.00
 HARDIE 18  f   1              0.94     6.52     0.35       0.02
 Subtotal HARDIE               1.30     8.35     4.07
 HO     12  b   3              1.05     7.74     0.10       0.00
 HOZAWA 9   b   0              2.36    12.11    17.18       0.00
 HUHTI1 69  m   0              2.81     0.97     2.62       0.01
 HUHTI1 145 f   1              0.68     2.30     0.55       0.30
 Subtotal HUHTI1               1.31     3.27     3.17
*KAHN2  80  m   1              2.21    17.22    18.63       0.00
 LAVECC 30  b   6              0.72   240.93    49.08       0.00
 LEBOWI 36  b   3              1.58     4.15     0.70       0.00
 MILLER 9   m   1              1.88     0.46     0.24       0.20
 MILLER 12  f   1              1.80     0.25     0.10       0.37
 Subtotal MILLER               1.85     0.71     0.33
 NAWA   1   m   0              1.93    29.63    16.97       0.00
 OMORI  6   m   1              2.28     3.07     3.75       0.00
 PRATT  1   m   0              1.38    11.29     0.48       0.00
 RYDER  3   b   1              2.46     5.74     9.54       0.00
*SILVA  9   b   6              0.74     8.89     1.62       0.03
 SUTINE 1   b   0              1.29     9.13     0.12       0.00
  ________________________________________________________________________________________________________________________
                                            International Evidence on Smoking and COPD, Phase 3, Analysis run on 27-SEP-10

                                                   Table 3 - A - 1 - 2

            IESCOPD - Meta-analysis of ever smoking, any product (or cigarettes if all product not available)
                                                      Any Emphysema
                                                      Most-adjusted


 REF    NRR SEX ADJ             Ys       Ws       Qs       Ps
 WANG2  1   b   0              1.71    33.72     9.86       0.00
 WEISS  16  m   0              1.48     0.94     0.09       0.15

                       N       28
                      NS       22


                      Wt   488.57
                 Het Chi   173.42
                 Het  df       27
                 Het  P       ***
               Fixed  RR     3.22
                     RRl     2.95
                     RRu     3.52
                      P       +++
              Random  RR     4.51
                     RRl     3.38
                     RRu     6.02
                      P       +++
               Asymm  P        **


  ________________________________________________________________________________________________________________________
                                            International Evidence on Smoking and COPD, Phase 3, Analysis run on 27-SEP-10

                                                   Table 3 - A - 1 - 3

            IESCOPD - Meta-analysis of ever smoking, any product (or cigarettes if all product not available)
                                                      Any Emphysema
                                                      Most-adjusted


                       N       28
                      NS       22


                      Wt   488.57
                 Het Chi   173.42
                 Het  df       27
                 Het  P       ***
               Fixed  RR     3.22
                     RRl     2.95
                     RRu     3.52
                      P       +++
              Random  RR     4.51
                     RRl     3.38
                     RRu     6.02
                      P       +++
               Asymm  P        **

                                   Sex
                             both      male    female     Total


                       N        9        13         6        28
                      NS        9        13         6        28


                      Wt   328.14    111.55     48.88    488.57
                 Het Chi    76.66     36.18     10.12    173.42
                 Het  df        8        12         5        27
                 Het  P       ***       ***       (*)       ***
               Fixed  RR     2.62      5.71      3.44      3.22
                     RRl     2.36      4.74      2.60      2.95
                     RRu     2.92      6.87      4.55      3.52
                      P       +++       +++       +++       +++
              Random  RR     4.47      5.42      3.25      4.51
                     RRl     2.72      3.69      2.07      3.38
                     RRu     7.34      7.96      5.10      6.02
                      P       +++       +++       +++       +++
             Between Chi                                  50.46
             Between  df                                      2
             Between  P                                     ***
             Btwn(F)  P                                       *

                                        Continent
                            NAmer    Europe      Asia  oth/mult     Total


                       N       14        10         4                  28
                      NS       11         7         4                  22


                      Wt   130.37    284.05     74.15              488.57
                 Het Chi    36.15     31.61      5.57              173.42
                 Het  df       13         9         3                  27
                 Het  P       ***       ***      N.S.                 ***
               Fixed  RR     5.33      2.19      5.76                3.22
                     RRl     4.49      1.95      4.59                2.95
                     RRu     6.33      2.46      7.23                3.52
                      P       +++       +++       +++                 +++
              Random  RR     5.32      3.13      5.59                4.51
                     RRl     3.86      2.01      3.93                3.38
                     RRu     7.32      4.87      7.95                6.02
                      P       +++       +++       +++                 +++
             Between Chi                                           100.10
             Between  df                                                2
             Between  P                                               ***
             Btwn(F)  P                                               ***


  ________________________________________________________________________________________________________________________
                                            International Evidence on Smoking and COPD, Phase 3, Analysis run on 27-SEP-10

                                                   Table 3 - A - 1 - 3

            IESCOPD - Meta-analysis of ever smoking, any product (or cigarettes if all product not available)
                                                      Any Emphysema
                                                      Most-adjusted
                        National cigarette tobacco type (excluding mixed/unkown)
                          blended  virginia     Total


                       N       26         2        28
                      NS       20         2        22


                      Wt   479.99      8.58    488.57
                 Het Chi   161.01      0.20    173.42
                 Het  df       25         1        27
                 Het  P       ***      N.S.       ***
               Fixed  RR     3.15     10.51      3.22
                     RRl     2.88      5.38      2.95
                     RRu     3.45     20.52      3.52
                      P       +++       +++       +++
              Random  RR     4.26     10.51      4.51
                     RRl     3.17      5.38      3.38
                     RRu     5.71     20.52      6.02
                      P       +++       +++       +++
             Between Chi                        12.22
             Between  df                            1
             Between  P                           ***
             Btwn(F)  P                          N.S.

                                        Start year of study
                            <1970   1970-79   1980-89   1990-99     2000+   unknown     Total


                       N        8         7         4         5                   4        28
                      NS        6         5         3         4                   4        22


                      Wt    57.29     36.49    289.52     79.43               25.84    488.57
                 Het Chi    17.75      4.90     37.09      9.99                5.22    173.42
                 Het  df        7         6         3         4                   3        27
                 Het  P         *      N.S.       ***         *                N.S.       ***
               Fixed  RR     6.99      2.63      2.32      5.38                6.13      3.22
                     RRl     5.39      1.90      2.07      4.32                4.17      2.95
                     RRu     9.05      3.64      2.61      6.71                9.01      3.52
                      P       +++       +++       +++       +++                 +++       +++
              Random  RR     5.73      2.63      3.72      4.90                6.61      4.51
                     RRl     3.51      1.90      1.94      3.26                3.89      3.38
                     RRu     9.37      3.64      7.15      7.36               11.23      6.02
                      P       +++       +++       +++       +++                 +++       +++
             Between Chi                                                                98.47
             Between  df                                                                    4
             Between  P                                                                   ***
             Btwn(F)  P                                                                   ***

                                Publication year
                            <1980   1980-89   1990-99     2000+     Total


                       N       13         5         3         7        28
                      NS       10         4         2         6        22


                      Wt    92.63    255.97     44.23     95.75    488.57
                 Het Chi    32.21      7.01      1.77     20.98    173.42
                 Het  df       12         4         2         6        27
                 Het  P        **      N.S.      N.S.        **       ***
               Fixed  RR     5.81      2.10      3.14      5.76      3.22
                     RRl     4.74      1.86      2.34      4.72      2.95
                     RRu     7.12      2.37      4.22      7.04      3.52
                      P       +++       +++       +++       +++       +++
              Random  RR     5.37      2.39      3.14      5.58      4.51
                     RRl     3.72      1.49      2.34      3.63      3.38
                     RRu     7.75      3.82      4.22      8.58      6.02
                      P       +++       +++       +++       +++       +++
             Between Chi                                           111.46
             Between  df                                                3
             Between  P                                               ***
             Btwn(F)  P                                               ***
  ________________________________________________________________________________________________________________________
                                            International Evidence on Smoking and COPD, Phase 3, Analysis run on 27-SEP-10

                                                   Table 3 - A - 1 - 3

            IESCOPD - Meta-analysis of ever smoking, any product (or cigarettes if all product not available)
                                                      Any Emphysema
                                                      Most-adjusted
                               Study type
                               CC        Pr        CS     Total


                       N                  6        22        28
                      NS                  5        17        22


                      Wt              61.97    426.61    488.57
                 Het Chi              24.90    119.19    173.42
                 Het  df                  5        21        27
                 Het  P                 ***       ***       ***
               Fixed  RR               6.12      2.93      3.22
                     RRl               4.77      2.67      2.95
                     RRu               7.85      3.22      3.52
                      P                 +++       +++       +++
              Random  RR               4.95      4.36      4.51
                     RRl               2.72      3.19      3.38
                     RRu               8.99      5.96      6.02
                      P                 +++       +++       +++
             Between Chi                                  29.33
             Between  df                                      1
             Between  P                                     ***
             Btwn(F)  P                                       *

                                    Lowest age in RR
                        <25/unlim     25-39       40+   unknown     Total


                       N       12         5        11                  28
                      NS       10         4         8                  22


                      Wt   333.91     53.08    101.58              488.57
                 Het Chi    56.06     13.03     30.49              173.42
                 Het  df       11         4        10                  27
                 Het  P       ***         *       ***                 ***
               Fixed  RR     2.50      7.32      4.82                3.22
                     RRl     2.24      5.60      3.97                2.95
                     RRu     2.78      9.58      5.85                3.52
                      P       +++       +++       +++                 +++
              Random  RR     3.66      6.18      4.83                4.51
                     RRl     2.48      3.62      3.25                3.38
                     RRu     5.40     10.57      7.17                6.02
                      P       +++       +++       +++                 +++
             Between Chi                                            73.85
             Between  df                                                2
             Between  P                                               ***
             Btwn(F)  P                                               ***

                                         Highest age in RR
                              <65     65-74     75-84 85+/unlim   unknown     Total


                       N        3         5         2        18                  28
                      NS        2         4         2        14                  22


                      Wt    15.38     47.24     20.25    405.71              488.57
                 Het Chi     5.93     16.53     12.04    100.38              173.42
                 Het  df        2         4         1        17                  27
                 Het  P       (*)        **       ***       ***                 ***
               Fixed  RR     8.48      4.84      6.59      2.86                3.22
                     RRl     5.14      3.64      4.26      2.59                2.95
                     RRu    13.97      6.44     10.19      3.15                3.52
                      P       +++       +++       +++       +++                 +++
              Random  RR     6.78      4.10      3.29      4.41                4.51
                     RRl     2.05      1.94      0.40      3.19                3.38
                     RRu    22.45      8.66     27.28      6.10                6.02
                      P        ++       +++      N.S.       +++                 +++
             Between Chi                                                      38.54
             Between  df                                                          3
             Between  P                                                         ***
             Btwn(F)  P                                                        N.S.
  ________________________________________________________________________________________________________________________
                                            International Evidence on Smoking and COPD, Phase 3, Analysis run on 27-SEP-10

                                                   Table 3 - A - 1 - 3

            IESCOPD - Meta-analysis of ever smoking, any product (or cigarettes if all product not available)
                                                      Any Emphysema
                                                      Most-adjusted
                           Study weakness
                              Yes        No     Total


                       N        3        25        28
                      NS        3        19        22


                      Wt    17.39    471.18    488.57
                 Het Chi     7.73    165.37    173.42
                 Het  df        2        24        27
                 Het  P         *       ***       ***
               Fixed  RR     3.68      3.20      3.22
                     RRl     2.30      2.93      2.95
                     RRu     5.89      3.51      3.52
                      P       +++       +++       +++
              Random  RR     3.48      4.65      4.51
                     RRl     1.21      3.41      3.38
                     RRu    10.01      6.32      6.02
                      P         +       +++       +++
             Between Chi                         0.32
             Between  df                            1
             Between  P                          N.S.
             Btwn(F)  P                          N.S.

                        Emp subtype
                             mort     other     Total


                       N        4        24        28
                      NS        3        19        22


                      Wt    50.05    438.52    488.57
                 Het Chi     0.89    123.30    173.42
                 Het  df        3        23        27
                 Het  P      N.S.       ***       ***
               Fixed  RR     8.24      2.89      3.22
                     RRl     6.24      2.63      2.95
                     RRu    10.87      3.18      3.52
                      P       +++       +++       +++
              Random  RR     8.24      4.00      4.51
                     RRl     6.24      2.97      3.38
                     RRu    10.87      5.37      6.02
                      P       +++       +++       +++
             Between Chi                        49.23
             Between  df                            1
             Between  P                           ***
             Btwn(F)  P                            **

                        Asthma analysis type (Emphysema)
                        inc-irres  excl-all     Total


                       N       26         2        28
                      NS       20         2        22


                      Wt   482.48      6.10    488.57
                 Het Chi   165.86      7.56    173.42
                 Het  df       25         1        27
                 Het  P       ***        **       ***
               Fixed  RR     3.22      3.22      3.22
                     RRl     2.94      1.45      2.95
                     RRu     3.52      7.11      3.52
                      P       +++        ++       +++
              Random  RR     4.61      3.20      4.51
                     RRl     3.43      0.36      3.38
                     RRu     6.19     28.37      6.02
                      P       +++      N.S.       +++
             Between Chi                         0.00
             Between  df                            1
             Between  P                          N.S.
             Btwn(F)  P                          N.S.
  ________________________________________________________________________________________________________________________
                                            International Evidence on Smoking and COPD, Phase 3, Analysis run on 27-SEP-10

                                                   Table 3 - A - 1 - 3

            IESCOPD - Meta-analysis of ever smoking, any product (or cigarettes if all product not available)
                                                      Any Emphysema
                                                      Most-adjusted
                                Number of emphysema cases
                             1-50    51-100   101-200      201+     Total


                       N        6         9         5         8        28
                      NS        5         6         5         6        22


                      Wt    16.57     46.08     37.95    387.97    488.57
                 Het Chi     4.85     14.64      7.69    118.54    173.42
                 Het  df        5         8         4         7        27
                 Het  P      N.S.       (*)      N.S.       ***       ***
               Fixed  RR     2.89      2.87      7.30      3.03      3.22
                     RRl     1.78      2.15      5.31      2.74      2.95
                     RRu     4.67      3.83     10.03      3.34      3.52
                      P       +++       +++       +++       +++       +++
              Random  RR     2.89      3.19      7.48      4.96      4.51
                     RRl     1.78      2.10      4.73      2.99      3.38
                     RRu     4.67      4.85     11.85      8.22      6.02
                      P       +++       +++       +++       +++       +++
             Between Chi                                            27.71
             Between  df                                                3
             Between  P                                               ***
             Btwn(F)  P                                              N.S.

                            Analysis type
                         prevlnce     onset     Total


                       N       22         6        28
                      NS       17         5        22


                      Wt   426.61     61.97    488.57
                 Het Chi   119.19     24.90    173.42
                 Het  df       21         5        27
                 Het  P       ***       ***       ***
               Fixed  RR     2.93      6.12      3.22
                     RRl     2.67      4.77      2.95
                     RRu     3.22      7.85      3.52
                      P       +++       +++       +++
              Random  RR     4.36      4.95      4.51
                     RRl     3.19      2.72      3.38
                     RRu     5.96      8.99      6.02
                      P       +++       +++       +++
             Between Chi                        29.33
             Between  df                            1
             Between  P                           ***
             Btwn(F)  P                             *

                             Smoking product
                              any      cigs  cigsonly     Total


                       N       11        15         2        28
                      NS        9        12         2        23


                      Wt   313.14    166.85      8.59    488.57
                 Het Chi    70.34     47.35      0.24    173.42
                 Het  df       10        14         1        27
                 Het  P       ***       ***      N.S.       ***
               Fixed  RR     2.51      4.96      6.69      3.22
                     RRl     2.24      4.26      3.43      2.95
                     RRu     2.80      5.78     13.05      3.52
                      P       +++       +++       +++       +++
              Random  RR     4.16      4.58      6.69      4.51
                     RRl     2.55      3.33      3.43      3.38
                     RRu     6.78      6.30     13.05      6.02
                      P       +++       +++       +++       +++
             Between Chi                                  55.49
             Between  df                                      2
             Between  P                                     ***
             Btwn(F)  P                                      **
  ________________________________________________________________________________________________________________________
                                            International Evidence on Smoking and COPD, Phase 3, Analysis run on 27-SEP-10

                                                   Table 3 - A - 1 - 3

            IESCOPD - Meta-analysis of ever smoking, any product (or cigarettes if all product not available)
                                                      Any Emphysema
                                                      Most-adjusted
                                     Unexposed group
                          nev any   nev cig  nev+ any  nev+ cig     Total


                       N       14        14                            28
                      NS       11        11                            22


                      Wt   340.70    147.87                        488.57
                 Het Chi   104.94     40.76                        173.42
                 Het  df       13        13                            27
                 Het  P       ***       ***                           ***
               Fixed  RR     2.75      4.62                          3.22
                     RRl     2.47      3.93                          2.95
                     RRu     3.06      5.43                          3.52
                      P       +++       +++                           +++
              Random  RR     4.76      4.28                          4.51
                     RRl     3.02      3.08                          3.38
                     RRu     7.50      5.96                          6.02
                      P       +++       +++                           +++
             Between Chi                                            27.72
             Between  df                                                1
             Between  P                                               ***
             Btwn(F)  P                                                 *

                        Unexposed group (combining nev+ with main levels)
                          nev any   nev cig     Total


                       N       14        14        28
                      NS       11        11        22


                      Wt   340.70    147.87    488.57
                 Het Chi   104.94     40.76    173.42
                 Het  df       13        13        27
                 Het  P       ***       ***       ***
               Fixed  RR     2.75      4.62      3.22
                     RRl     2.47      3.93      2.95
                     RRu     3.06      5.43      3.52
                      P       +++       +++       +++
              Random  RR     4.76      4.28      4.51
                     RRl     3.02      3.08      3.38
                     RRu     7.50      5.96      6.02
                      P       +++       +++       +++
             Between Chi                        27.72
             Between  df                            1
             Between  P                           ***
             Btwn(F)  P                             *

                        Smoking results reported in study (Emphysema)
                             Ever   Current      Both     Total


                       N       11                  17        28
                      NS        9                  13        22


                      Wt   150.96              337.61    488.57
                 Het Chi    31.30               66.29    173.42
                 Het  df       10                  16        27
                 Het  P       ***                 ***       ***
               Fixed  RR     5.80                2.47      3.22
                     RRl     4.95                2.22      2.95
                     RRu     6.81                2.75      3.52
                      P       +++                 +++       +++
              Random  RR     5.51                3.77      4.51
                     RRl     4.08                2.63      3.38
                     RRu     7.43                5.42      6.02
                      P       +++                 +++       +++
             Between Chi                                  75.84
             Between  df                                      1
             Between  P                                     ***
             Btwn(F)  P                                     ***
  ________________________________________________________________________________________________________________________
                                            International Evidence on Smoking and COPD, Phase 3, Analysis run on 27-SEP-10

                                                   Table 3 - A - 1 - 3

            IESCOPD - Meta-analysis of ever smoking, any product (or cigarettes if all product not available)
                                                      Any Emphysema
                                                      Most-adjusted
                        Number of adjustment variables
                                0         1        2+     Total


                       N       11        13         4        28
                      NS       10         9         4        23


                      Wt   143.05     83.82    261.71    488.57
                 Het Chi    29.59     35.34      3.82    173.42
                 Het  df       10        12         3        27
                 Het  P       ***       ***      N.S.       ***
               Fixed  RR     4.91      5.95      2.10      3.22
                     RRl     4.16      4.80      1.86      2.95
                     RRu     5.78      7.37      2.37      3.52
                      P       +++       +++       +++       +++
              Random  RR     4.64      5.52      2.26      4.51
                     RRl     3.38      3.63      1.75      3.38
                     RRu     6.38      8.39      2.93      6.02
                      P       +++       +++       +++       +++
             Between Chi                                 104.67
             Between  df                                      2
             Between  P                                     ***
             Btwn(F)  P                                     ***

                        RR adjusted for sex (combined sex RR only)
                              Yes        No     Total


                       N        4         5         9
                      NS        4         5         9


                      Wt   261.71     66.43    328.14
                 Het Chi     3.82      8.87     76.66
                 Het  df        3         4         8
                 Het  P      N.S.       (*)       ***
               Fixed  RR     2.10      6.30      2.62
                     RRl     1.86      4.96      2.36
                     RRu     2.37      8.02      2.92
                      P       +++       +++       +++
              Random  RR     2.26      6.62      4.47
                     RRl     1.75      4.44      2.72
                     RRu     2.93      9.87      7.34
                      P       +++       +++       +++
             Between Chi                        63.97
             Between  df                            1
             Between  P                           ***
             Btwn(F)  P                           ***

                        RR adjusted for age


                       N       15        13        28
                      NS       12        11        23


                      Wt   344.81    143.76    488.57
                 Het Chi   107.39     29.64    173.42
                 Het  df       14        12        27
                 Het  P       ***        **       ***
               Fixed  RR     2.70      4.91      3.22
                     RRl     2.43      4.17      2.95
                     RRu     3.00      5.79      3.52
                      P       +++       +++       +++
              Random  RR     4.44      4.67      4.51
                     RRl     2.90      3.45      3.38
                     RRu     6.82      6.33      6.02
                      P       +++       +++       +++
             Between Chi                        36.39
             Between  df                            1
             Between  P                           ***
             Btwn(F)  P                             *

  ________________________________________________________________________________________________________________________
                                            International Evidence on Smoking and COPD, Phase 3, Analysis run on 27-SEP-10

                                                   Table 3 - A - 1 - 3

            IESCOPD - Meta-analysis of ever smoking, any product (or cigarettes if all product not available)
                                                      Any Emphysema
                                                      Most-adjusted
                        RR adjusted for factor other than sex, age
                              Yes        No     Total


                       N        6        22        28
                      NS        5        17        22


                      Wt   262.42    226.15    488.57
                 Het Chi     4.69     66.86    173.42
                 Het  df        5        21        27
                 Het  P      N.S.       ***       ***
               Fixed  RR     2.11      5.26      3.22
                     RRl     1.87      4.62      2.95
                     RRu     2.38      6.00      3.52
                      P       +++       +++       +++
              Random  RR     2.11      5.02      4.51
                     RRl     1.87      3.88      3.38
                     RRu     2.38      6.50      6.02
                      P       +++       +++       +++
             Between Chi                       101.86
             Between  df                            1
             Between  P                           ***
             Btwn(F)  P                           ***

                        Derivation of RR/CI
                         Orig/2x2     Other     Total


                       N        4        24        28
                      NS        4        18        22


                      Wt    55.79    432.78    488.57
                 Het Chi     7.35    143.76    173.42
                 Het  df        3        23        27
                 Het  P       (*)       ***       ***
               Fixed  RR     5.84      2.98      3.22
                     RRl     4.49      2.71      2.95
                     RRu     7.59      3.28      3.52
                      P       +++       +++       +++
              Random  RR     5.67      4.29      4.51
                     RRl     3.61      3.12      3.38
                     RRu     8.91      5.91      6.02
                      P       +++       +++       +++
             Between Chi                        22.32
             Between  df                            1
             Between  P                           ***
             Btwn(F)  P                           (*)


  ________________________________________________________________________________________________________________________
                                            International Evidence on Smoking and COPD, Phase 3, Analysis run on 27-SEP-10

                                                   Table 3 - A - 1 - 4

            IESCOPD - Meta-analysis of ever smoking, any product (or cigarettes if all product not available)
                                                      Any Emphysema
                                                      Least-adjusted


     REF|NRR|X|SEX|AGEL|AGEH|     REGION|BEGYR|PUBYR|STTYP|ONSET|      DISEAS|ADJ|SMOKSTA|   PRODUCT|    UNEXP|

  ANDER2   1     b   15   99      Am:USA     *  1966    CS  Prev  Emp:viscomp   0    Ever  Cigs only   Nev any
    BEST  20     m   30   97   Am:Canada  1955  1967    Pr   Inc     Emp:mort   1    Ever  Cigs only   Nev any
  DONTA2  13     m   25   84 Eu:SE/Balkn  1960  1984    Pr   Inc Emp:diagnosd   0    Ever       Cigs  Nev cigs
  ENRIGH   9     m   65   99      Am:USA  1989  1994    CS  Prev Emp:diagnosd   0    Ever       Cigs  Nev cigs
  ENRIGH  12     f   65   99      Am:USA  1989  1994    CS  Prev Emp:diagnosd   0    Ever       Cigs  Nev cigs
  GULSVI   3 x   m   15   70    Eu:Scand  1972  1979    CS  Prev Emp:diagnosd   0    Ever        Any   Nev any
  GULSVI   5 x   f   15   70    Eu:Scand  1972  1979    CS  Prev Emp:diagnosd   0    Ever        Any   Nev any
  HAMMO2   7     m   35   99      Am:USA  1959  1966    Pr   Inc     Emp:mort   1    Ever       Cigs   Nev any
  HAMMO2   8     f   35   99      Am:USA  1959  1966    Pr   Inc     Emp:mort   1    Ever        Any   Nev any
  HARDIE  15     m   70   99    Eu:Scand  1998  2005    CS  Prev Emp:diagnosd   1    Ever       Cigs  Nev cigs
  HARDIE  18     f   70   99    Eu:Scand  1998  2005    CS  Prev Emp:diagnosd   1    Ever       Cigs  Nev cigs
      HO  12     b   70   99   Asia:FarE  1991  1999    CS  Prev Emp:self-rep   3    Ever       Cigs  Nev cigs
  HOZAWA   9     b   45   64      Am:USA  1987  2006    CS  Prev Emp:diagnosd   0    Ever       Cigs  Nev cigs
  HUHTI1  69     m   40   64    Eu:Scand  1961  1965    CS  Prev    Emp:other   0    Ever        Any   Nev any
  HUHTI1 144 x   f   40   64    Eu:Scand  1961  1965    CS  Prev    Emp:other   0    Ever        Any   Nev any
   KAHN2  80     m   31   84      Am:USA  1954  1966    Pr   Inc     Emp:mort   1    Ever        Any   Nev any
  LAVECC  24 x   m   15   99     Eu:West  1983  1988    CS  Prev Emp:self-rep   0    Ever        Any   Nev any
  LAVECC  27 x   f   15   99     Eu:West  1983  1988    CS  Prev Emp:self-rep   0    Ever        Any   Nev any
  LEBOWI  33 x   b   15   96      Am:USA  1972  1977    CS  Prev Emp:diagnosd   2    Ever       Cigs  Nev cigs
  MILLER   9     m   15   99      Am:USA  1978  1988    CS  Prev Emp:diagnosd   1    Ever       Cigs  Nev cigs
  MILLER  12     f   15   99      Am:USA  1978  1988    CS  Prev Emp:diagnosd   1    Ever       Cigs  Nev cigs
    NAWA   1     m   50   69   Asia:FarE  1998  2002    CS  Prev  Emp:viscomp   0    Ever       Cigs  Nev cigs
   OMORI   3 x   m   40   69   Asia:FarE     *  2006    CS  Prev  Emp:viscomp   0    Ever       Cigs  Nev cigs
   PRATT   1     m   15   99      Am:USA     *  1980    CS  Prev  Emp:viscomp   0    Ever        Any   Nev any
   RYDER   1 x   m   22   95       Eu:UK     *  1971    CS  Prev  Emp:viscomp   0    Ever        Any   Nev any
   RYDER   2 x   f   22   95       Eu:UK     *  1971    CS  Prev  Emp:viscomp   0    Ever        Any   Nev any
   SILVA   9     b   20   99      Am:USA  1972  2004    Pr   Inc    Emp:other   6    Ever       Cigs  Nev cigs
  SUTINE   1     b   10   99    Eu:Scand  1971  1978    CS  Prev  Emp:viscomp   0    Ever        Any   Nev any
   WANG2   1     b   19   92   Asia:FarE  1996  2001    CS  Prev  Emp:viscomp   0    Ever       Cigs  Nev cigs
   WEISS  16     m   50   69      Am:USA  1961  1963    CS  Prev  Emp:viscomp   0    Ever        Any   Nev any


  ________________________________________________________________________________________________________________________
                                            International Evidence on Smoking and COPD, Phase 3, Analysis run on 27-SEP-10

                                                   Table 3 - A - 1 - 5

            IESCOPD - Meta-analysis of ever smoking, any product (or cigarettes if all product not available)
                                                      Any Emphysema
                                                      Least-adjusted


                        Number Exposed  Non-exposed
 REF    NRR SEX ADJ     Case    Cont    Case    Cont      RR        95.00%CI
 ANDER2 1   b   0        102      12      30      21      5.95 (  2.63-  13.48)
*BEST   20  m   1         52       -       3       -      8.47 (  2.65-  27.11)
*DONTA2 13  m   0         11     333       4     127      1.05 (  0.34-   3.23)
 ENRIGH 9   m   0        113    1390      22     694      2.56 (  1.61-   4.09)
 ENRIGH 12  f   0         75    1182      26    1617      3.95 (  2.51-   6.20)
 Subtotal ENRIGH                                          3.20 (  2.31-   4.43)
 GULSVI 3   m   0         40    5812       4    2090      3.60 (  1.29-  10.06)
 GULSVI 5   f   0         28    4913      16    4231      1.51 (  0.81-   2.79)
 Subtotal GULSVI                                          1.90 (  1.12-   3.21)
*HAMMO2 7   m   1        369       -      20       -      8.64 (  5.51-  13.55)
*HAMMO2 8   f   1         24       -      21       -      6.44 (  3.57-  11.63)
 Subtotal HAMMO2                                          7.76 (  5.42-  11.09)
 HARDIE 15  m   1         48       -       2       -     13.38 (  3.14-  56.98)
 HARDIE 18  f   1         11       -      11       -      2.55 (  1.18-   5.48)
 Subtotal HARDIE                                          3.67 (  1.86-   7.23)
 HO     12  b   3          -       -       -       -      2.87 (  1.42-   5.81)
 HOZAWA 9   b   0        185    8775      13    6533     10.59 (  6.03-  18.61)
 HUHTI1 69  m   0         64     467       1     121     16.58 (  2.28- 120.74)
 HUHTI1 144 f   0          3     111      16     693      1.17 (  0.34-   4.08)
 Subtotal HUHTI1                                          2.48 (  0.86-   7.15)
*KAHN2  80  m   1        408       -      18       -      9.11 (  5.68-  14.61)
 LAVECC 24  m   0        700   19775     183   14129      2.73 (  2.32-   3.22)
 LAVECC 27  f   0         89    7548     412   29448      0.84 (  0.67-   1.06)
 Subtotal LAVECC                                          1.84 (  1.61-   2.10)
 LEBOWI 33  b   2         31       -       5       -      5.08 (  1.95-  13.23)
 MILLER 9   m   1          6       -       0       -      6.56 (  0.37- 116.09)
 MILLER 12  f   1          2       -       1       -      6.03 (  0.12- 308.75)
 Subtotal MILLER                                          6.37 (  0.63-  64.77)
 NAWA   1   m   0        654    4086      32    1372      6.86 (  4.79-   9.84)
 OMORI  3   m   0        135     380       3      97     11.49 (  3.58-  36.85)
 PRATT  1   m   0        178     246      15      82      3.96 (  2.21-   7.09)
 RYDER  1   m   0         56      14       7       9      5.14 (  1.63-  16.21)
 RYDER  2   f   0         24      12      14      43      6.14 (  2.45-  15.39)
 Subtotal RYDER                                           5.73 (  2.80-  11.74)
*SILVA  9   b   6          -       -       -       -      2.10 (  1.09-   4.06)
 SUTINE 1   b   0         63      28      28      45      3.62 (  1.89-   6.92)
 WANG2  1   b   0        188    3408      42    4209      5.53 (  3.94-   7.75)
 WEISS  16  m   0         28     223       1      35      4.39 (  0.58-  33.34)
Partial Totals          3687   58715     950   65596
*prospective study


 REF    NRR SEX ADJ             Ys       Ws       Qs       Ps
 ANDER2 1   b   0              1.78     5.74     2.42       0.00
*BEST   20  m   1              2.14     2.84     2.85       0.00
*DONTA2 13  m   0              0.05     3.03     3.58       0.93
 ENRIGH 9   m   0              0.94    17.71     0.66       0.00
 ENRIGH 12  f   0              1.37    18.78     1.06       0.00
 Subtotal ENRIGH               1.16    36.49     1.72
 GULSVI 3   m   0              1.28     3.63     0.08       0.01
 GULSVI 5   f   0              0.41    10.14     5.32       0.19
 Subtotal GULSVI               0.64    13.76     5.40
*HAMMO2 7   m   1              2.16    18.98    19.81       0.00
*HAMMO2 8   f   1              1.86    11.02     5.84       0.00
 Subtotal HAMMO2               2.05    29.99    25.65
 HARDIE 15  m   1              2.59     1.83     3.89       0.00
 HARDIE 18  f   1              0.94     6.52     0.26       0.02
 Subtotal HARDIE               1.30     8.35     4.15
 HO     12  b   3              1.05     7.74     0.05       0.00
 HOZAWA 9   b   0              2.36    12.11    18.19       0.00
 HUHTI1 69  m   0              2.81     0.97     2.73       0.01
 HUHTI1 144 f   0              0.16     2.46     2.35       0.80
 Subtotal HUHTI1               0.91     3.44     5.08
*KAHN2  80  m   1              2.21    17.22    19.88       0.00
 LAVECC 24  m   0              1.01   142.56     2.38       0.00
 LAVECC 27  f   0             -0.17    72.31   123.28       0.15
 Subtotal LAVECC               0.61   214.87   125.66
 LEBOWI 33  b   2              1.63     4.19     1.01       0.00
 MILLER 9   m   1              1.88     0.46     0.26       0.20
 MILLER 12  f   1              1.80     0.25     0.11       0.37
 Subtotal MILLER               1.85     0.71     0.37
  ________________________________________________________________________________________________________________________
                                            International Evidence on Smoking and COPD, Phase 3, Analysis run on 27-SEP-10

                                                   Table 3 - A - 1 - 5

            IESCOPD - Meta-analysis of ever smoking, any product (or cigarettes if all product not available)
                                                      Any Emphysema
                                                      Least-adjusted


 REF    NRR SEX ADJ             Ys       Ws       Qs       Ps
 NAWA   1   m   0              1.93    29.63    18.56       0.00
 OMORI  3   m   0              2.44     2.83     4.83       0.00
 PRATT  1   m   0              1.38    11.29     0.65       0.00
 RYDER  1   m   0              1.64     2.91     0.74       0.01
 RYDER  2   f   0              1.82     4.55     2.11       0.00
 Subtotal RYDER                1.75     7.47     2.85
*SILVA  9   b   6              0.74     8.89     1.37       0.03
 SUTINE 1   b   0              1.29     9.13     0.21       0.00
 WANG2  1   b   0              1.71    33.72    11.16       0.00
 WEISS  16  m   0              1.48     0.94     0.11       0.15

                       N       30
                      NS       22


                      Wt   464.36
                 Het Chi   255.74
                 Het  df       29
                 Het  P       ***
               Fixed  RR     3.11
                     RRl     2.84
                     RRu     3.41
                      P       +++
              Random  RR     4.16
                     RRl     3.03
                     RRu     5.71
                      P       +++
               Asymm  P       (*)


  ________________________________________________________________________________________________________________________
                                            International Evidence on Smoking and COPD, Phase 3, Analysis run on 27-SEP-10

                                                   Table 3 - A - 1 - 6

            IESCOPD - Meta-analysis of ever smoking, any product (or cigarettes if all product not available)
                                                      Any Emphysema
                                                      Least-adjusted


                       N       30
                      NS       22


                      Wt   464.36
                 Het Chi   255.74
                 Het  df       29
                 Het  P       ***
               Fixed  RR     3.11
                     RRl     2.84
                     RRu     3.41
                      P       +++
              Random  RR     4.16
                     RRl     3.03
                     RRu     5.71
                      P       +++
               Asymm  P       (*)

                                   Sex
                             both      male    female     Total


                       N        7        15         8        30
                      NS        7        15         8        30


                      Wt    81.52    256.83    126.02    464.36
                 Het Chi    17.35     70.31     76.47    255.74
                 Het  df        6        14         7        29
                 Het  P        **       ***       ***       ***
               Fixed  RR     4.91      3.81      1.53      3.11
                     RRl     3.95      3.38      1.28      2.84
                     RRu     6.10      4.31      1.82      3.41
                      P       +++       +++       +++       +++
              Random  RR     4.60      5.18      2.56      4.16
                     RRl     3.07      3.53      1.23      3.03
                     RRu     6.87      7.58      5.32      5.71
                      P       +++       +++         +       +++
             Between Chi                                  91.61
             Between  df                                      2
             Between  P                                     ***
             Btwn(F)  P                                      **

                                        Continent
                            NAmer    Europe      Asia  oth/mult     Total


                       N       14        12         4                  30
                      NS       11         7         4                  22


                      Wt   130.41    260.04     73.91              464.36
                 Het Chi    36.12     94.81      6.07              255.74
                 Het  df       13        11         3                  29
                 Het  P       ***       ***      N.S.                 ***
               Fixed  RR     5.34      1.99      5.79                3.11
                     RRl     4.50      1.76      4.61                2.84
                     RRu     6.34      2.24      7.27                3.41
                      P       +++       +++       +++                 +++
              Random  RR     5.33      2.73      5.64                4.16
                     RRl     3.87      1.67      3.89                3.03
                     RRu     7.34      4.45      8.18                5.71
                      P       +++       +++       +++                 +++
             Between Chi                                           118.74
             Between  df                                                2
             Between  P                                               ***
             Btwn(F)  P                                               ***


  ________________________________________________________________________________________________________________________
                                            International Evidence on Smoking and COPD, Phase 3, Analysis run on 27-SEP-10

                                                   Table 3 - A - 1 - 6

            IESCOPD - Meta-analysis of ever smoking, any product (or cigarettes if all product not available)
                                                      Any Emphysema
                                                      Least-adjusted
                               Study type
                               CC        Pr        CS     Total


                       N                  6        24        30
                      NS                  5        17        22


                      Wt              61.97    402.40    464.36
                 Het Chi              24.90    198.02    255.74
                 Het  df                  5        23        29
                 Het  P                 ***       ***       ***
               Fixed  RR               6.12      2.80      3.11
                     RRl               4.77      2.54      2.84
                     RRu               7.85      3.09      3.41
                      P                 +++       +++       +++
              Random  RR               4.95      3.97      4.16
                     RRl               2.72      2.80      3.03
                     RRu               8.99      5.62      5.71
                      P                 +++       +++       +++
             Between Chi                                  32.81
             Between  df                                      1
             Between  P                                     ***
             Btwn(F)  P                                     (*)

                        Emp subtype
                             mort     other     Total


                       N        4        26        30
                      NS        3        19        22


                      Wt    50.05    414.31    464.36
                 Het Chi     0.89    201.61    255.74
                 Het  df        3        25        29
                 Het  P      N.S.       ***       ***
               Fixed  RR     8.24      2.76      3.11
                     RRl     6.24      2.51      2.84
                     RRu    10.87      3.04      3.41
                      P       +++       +++       +++
              Random  RR     8.24      3.68      4.16
                     RRl     6.24      2.64      3.03
                     RRu    10.87      5.12      5.71
                      P       +++       +++       +++
             Between Chi                        53.24
             Between  df                            1
             Between  P                           ***
             Btwn(F)  P                             *

                             Smoking product
                              any      cigs  cigsonly     Total


                       N       13        15         2        30
                      NS        9        12         2        23


                      Wt   289.13    166.65      8.59    464.36
                 Het Chi   140.72     47.95      0.24    255.74
                 Het  df       12        14         1        29
                 Het  P       ***       ***      N.S.       ***
               Fixed  RR     2.32      4.98      6.69      3.11
                     RRl     2.07      4.28      3.43      2.84
                     RRu     2.60      5.79     13.05      3.41
                      P       +++       +++       +++       +++
              Random  RR     3.46      4.62      6.69      4.16
                     RRl     2.09      3.34      3.43      3.03
                     RRu     5.72      6.37     13.05      5.71
                      P       +++       +++       +++       +++
             Between Chi                                  66.83
             Between  df                                      2
             Between  P                                     ***
             Btwn(F)  P                                       *
  ________________________________________________________________________________________________________________________
                                            International Evidence on Smoking and COPD, Phase 3, Analysis run on 27-SEP-10

                                                   Table 3 - A - 1 - 6

            IESCOPD - Meta-analysis of ever smoking, any product (or cigarettes if all product not available)
                                                      Any Emphysema
                                                      Least-adjusted
                                     Unexposed group
                          nev any   nev cig  nev+ any  nev+ cig     Total


                       N       16        14                            30
                      NS       11        11                            22


                      Wt   316.69    147.67                        464.36
                 Het Chi   179.78     41.43                        255.74
                 Het  df       15        13                            29
                 Het  P       ***       ***                           ***
               Fixed  RR     2.58      4.64                          3.11
                     RRl     2.31      3.95                          2.84
                     RRu     2.88      5.45                          3.41
                      P       +++       +++                           +++
              Random  RR     4.04      4.32                          4.16
                     RRl     2.53      3.09                          3.03
                     RRu     6.45      6.04                          5.71
                      P       +++       +++                           +++
             Between Chi                                            34.53
             Between  df                                                1
             Between  P                                               ***
             Btwn(F)  P                                                 *

                        Unexposed group (combining nev+ with main levels)
                          nev any   nev cig     Total


                       N       16        14        30
                      NS       11        11        22


                      Wt   316.69    147.67    464.36
                 Het Chi   179.78     41.43    255.74
                 Het  df       15        13        29
                 Het  P       ***       ***       ***
               Fixed  RR     2.58      4.64      3.11
                     RRl     2.31      3.95      2.84
                     RRu     2.88      5.45      3.41
                      P       +++       +++       +++
              Random  RR     4.04      4.32      4.16
                     RRl     2.53      3.09      3.03
                     RRu     6.45      6.04      5.71
                      P       +++       +++       +++
             Between Chi                        34.53
             Between  df                            1
             Between  P                           ***
             Btwn(F)  P                             *


  ________________________________________________________________________________________________________________________
                                            International Evidence on Smoking and COPD, Phase 3, Analysis run on 27-SEP-10

                                                   Table 3 - A - 1 - 7

            IESCOPD - Meta-analysis of ever smoking, any product (or cigarettes if all product not available)
                                                      Any Emphysema
                                 Excluded studies (and stage at which they were excluded)


1       CLARK COTTON  MEYER REMYJA RUTGER SNYDER SOBRAX     SU TAKEMU  WANG4   WEIR WHICKE ZALACA
2      ALDERS ALESSA  AMIGO ANDER1 ANDER3   BANG  BECK1  BECK2 BEDNAR BJORNS BROGGE  BROWN CERVER CHAPMA  CHEN1  CHEN2
        CHEN3  CHENG CLEMEN COATES  COCCI COLLEG  DEAN1  DEAN2  DEANE DEJONG DEMARC DETORR DICKIN  DOLL1  DOLL2 DONTA1
       DOPICO EHRLIC EKBERG ENSTRO FERRI1 FERRI2 FERRI3  FIDAN FINKLE FLETCH FORAST FOXMAN FUKUCH GEIJER GODTFR GOLDBE
       HAENSZ HARIKK HARRIS HAWTHO  HAYES HEDMAN HIGGI2 HIGGI3 HIGGI4 HIGGI6 HOLLA2 HOLLNA  HOUSE HRUBEC HUCHON HUHTI2
       HUHTI3 ITABAS JACOBS JAENDI JENSEN JINDA2 JOHANN  JOSHI JOUSI1 KACHEL KARAKA KATANC   KATO KHOURY    KIM  KIRAZ
       KLAYTO KOJIMA KOTAN1 KOTAN2 KRZYZA  KUBIK KULLER    LAI   LAM1   LAM2   LAM3 LAMBER  LANGE LANGE2 LANGHA    LEE
         LIAW LINDBE LINDST   LIU1   LIU2 LUNDB1 LUNDB2  MADOR MAGNUS MANFRE MANNI1 MANNI2 MANNI3 MARAN1 MARAN2 MARCUS
       MATHES MELLST MENEZ1 MENEZ2 MENEZ3 MENEZ4 MENEZ5 MENEZ6  MEREN  MILNE MOLLER MONTNE MUELLE NEJJAR NIEPSU NIHLEN
       NILSSO OGILVI OSWAL1 OSWAL2 PANDEY   PEAT PELKON PEREZP   PETO  PRICE   REID RENWIC RICCIO RIMING SARGEA SAWICK
       SCHWAR SHAHAB  SHARP SHIMUR   SHIN SICHLE SOBRAD SPEIZE STERLI STJERN  STROM SUADIC  TAGER TAGER2   TANG   THUN
         TODD TROISI TRUPIN TSUSHI TVERDA URRUTI VESTBO VIEGI1 VIEGI2 VINEIS VOLLM1 VOLLM2 VONHER WAGEN2   WALD WATSON
          WIG WILHEL WILSO1 WOJTYN  WOODS  WOOLF   XIAO     XU YAMAGU   YUAN ZIELI1 ZIELI2 ZIETKO   ZOIA
3      WILSO2
4      AUERBA HIRAYA   KAHN VIKGRE    WEN


  ________________________________________________________________________________________________________________________
                                            International Evidence on Smoking and COPD, Phase 3, Analysis run on 27-SEP-10

                                                   Table 3 - A - 1 - 8

            IESCOPD - Meta-analysis of ever smoking, any product (or cigarettes if all product not available)
                                                      Any Emphysema
                                             Potentially overlapping studies


     REF| REFGP|PRINC|                     OVERLAP|

  HOZAWA HOZAWA     1         ENRIGH/HOZAWA/HARIKK
  ENRIGH ENRIGH     1         ENRIGH/HOZAWA/HARIKK
  DONTA2 JACOBS     2  JACOBS/DONTA1/DONTA2/PELKON
  HUHTI1 HUHTI1     1                HUHTI1/HUHTI2
  HARDIE HARDIE     1         HARDIE/JOHANN/BROGGE
  HAMMO2 HAMMO2     1                HAMMO2/ENSTRO
  LEBOWI LEBOWI     1                 LEBOWI/SILVA
   SILVA  SILVA     1                 LEBOWI/SILVA
   KAHN2   KAHN     2                   KAHN/KAHN2


  ________________________________________________________________________________________________________________________
                                            International Evidence on Smoking and COPD, Phase 3, Analysis run on 27-SEP-10

                                                    Table 3 - A - 2 -

            IESCOPD - Meta-analysis of ever smoking, cigarettes (or all products if cigarettes not available)
                                                      Any Emphysema


This analysis is restricted to results for:
1) Eligible study on database
2) Outcome Emphysema
3) Non-dose-response data
4) Ever smoking
5) Results complete enough for use in meta-analysis

Within each study, results are then selected (in the following order of preference, within each sex) for:
6) UNEXP   : never cigarettes, never any, other
7) PROD    : cigarettes, cigarettes only, any product
8) For overlapping studies: principal rather than subsidiary studies
and then for single sex results (m, f) in preference to results for both sexes combined (b).

Results adjusted for the most potential confounders are then chosen in Sections -1 to -3
(and those which actually differ from the adjusted results in Table 3 - A - 1 - 1 are marked 'x' in Section -1)
and results adjusted for the least confounders in Sections -4 to -6. (Those least adjusted results which
actually differ from the most adjusted are marked 'x' in column X in Section -4)

Section -7 shows excluded studies, together with the stage (as above) at which no qualifying
results were found.

Section -8 lists the potentially overlapping studies which have been included (1=principal, 2=subsidiary),
and any results which would have been included in preference except that they had data not complete enough
for use in meta-analysis. It also lists their significance (yes/no), if known.


  ________________________________________________________________________________________________________________________
                                            International Evidence on Smoking and COPD, Phase 3, Analysis run on 27-SEP-10

                                                   Table 3 - A - 2 - 1

            IESCOPD - Meta-analysis of ever smoking, cigarettes (or all products if cigarettes not available)
                                                      Any Emphysema
                                                      Most-adjusted


     REF|NRR|Cmp3A1|SEX|AGEL|AGEH|     REGION|BEGYR|PUBYR|STTYP|ONSET|      DISEAS|ADJ|SMOKSTA|   PRODUCT|    UNEXP|

  ANDER2   1          b   15   99      Am:USA     *  1966    CS  Prev  Emp:viscomp   0    Ever  Cigs only   Nev any
    BEST  20          m   30   97   Am:Canada  1955  1967    Pr   Inc     Emp:mort   1    Ever  Cigs only   Nev any
  DONTA2  13          m   25   84 Eu:SE/Balkn  1960  1984    Pr   Inc Emp:diagnosd   0    Ever       Cigs  Nev cigs
  ENRIGH   9          m   65   99      Am:USA  1989  1994    CS  Prev Emp:diagnosd   0    Ever       Cigs  Nev cigs
  ENRIGH  12          f   65   99      Am:USA  1989  1994    CS  Prev Emp:diagnosd   0    Ever       Cigs  Nev cigs
  GULSVI   4          m   15   70    Eu:Scand  1972  1979    CS  Prev Emp:diagnosd   1    Ever        Any   Nev any
  GULSVI   6          f   15   70    Eu:Scand  1972  1979    CS  Prev Emp:diagnosd   1    Ever        Any   Nev any
  HAMMO2   7          m   35   99      Am:USA  1959  1966    Pr   Inc     Emp:mort   1    Ever       Cigs   Nev any
  HAMMO2  16      x   f   35   99      Am:USA  1959  1966    Pr   Inc     Emp:mort   1    Ever       Cigs  Nev cigs
  HARDIE  15          m   70   99    Eu:Scand  1998  2005    CS  Prev Emp:diagnosd   1    Ever       Cigs  Nev cigs
  HARDIE  18          f   70   99    Eu:Scand  1998  2005    CS  Prev Emp:diagnosd   1    Ever       Cigs  Nev cigs
      HO  12          b   70   99   Asia:FarE  1991  1999    CS  Prev Emp:self-rep   3    Ever       Cigs  Nev cigs
  HOZAWA   9          b   45   64      Am:USA  1987  2006    CS  Prev Emp:diagnosd   0    Ever       Cigs  Nev cigs
  HUHTI1  69          m   40   64    Eu:Scand  1961  1965    CS  Prev    Emp:other   0    Ever        Any   Nev any
  HUHTI1 157      x   f   40   64    Eu:Scand  1961  1965    CS  Prev    Emp:other   1    Ever       Cigs  Nev cigs
   KAHN2  81      x   m   31   84      Am:USA  1954  1966    Pr   Inc     Emp:mort   1    Ever       Cigs   Nev any
  LAVECC  30          b   15   99     Eu:West  1983  1988    CS  Prev Emp:self-rep   6    Ever        Any   Nev any
  LEBOWI  36          b   15   96      Am:USA  1972  1977    CS  Prev Emp:diagnosd   3    Ever       Cigs  Nev cigs
  MILLER   9          m   15   99      Am:USA  1978  1988    CS  Prev Emp:diagnosd   1    Ever       Cigs  Nev cigs
  MILLER  12          f   15   99      Am:USA  1978  1988    CS  Prev Emp:diagnosd   1    Ever       Cigs  Nev cigs
    NAWA   1          m   50   69   Asia:FarE  1998  2002    CS  Prev  Emp:viscomp   0    Ever       Cigs  Nev cigs
   OMORI   6          m   40   69   Asia:FarE     *  2006    CS  Prev  Emp:viscomp   1    Ever       Cigs  Nev cigs
   PRATT   1          m   15   99      Am:USA     *  1980    CS  Prev  Emp:viscomp   0    Ever        Any   Nev any
   RYDER   3          b   22   95       Eu:UK     *  1971    CS  Prev  Emp:viscomp   1    Ever        Any   Nev any
   SILVA   9          b   20   99      Am:USA  1972  2004    Pr   Inc    Emp:other   6    Ever       Cigs  Nev cigs
  SUTINE   1          b   10   99    Eu:Scand  1971  1978    CS  Prev  Emp:viscomp   0    Ever        Any   Nev any
   WANG2   1          b   19   92   Asia:FarE  1996  2001    CS  Prev  Emp:viscomp   0    Ever       Cigs  Nev cigs
   WEISS  16          m   50   69      Am:USA  1961  1963    CS  Prev  Emp:viscomp   0    Ever        Any   Nev any


  ________________________________________________________________________________________________________________________
                                            International Evidence on Smoking and COPD, Phase 3, Analysis run on 27-SEP-10

                                                   Table 3 - A - 2 - 2

            IESCOPD - Meta-analysis of ever smoking, cigarettes (or all products if cigarettes not available)
                                                      Any Emphysema
                                                      Most-adjusted


                        Number Exposed  Non-exposed
 REF    NRR SEX ADJ     Case    Cont    Case    Cont      RR        95.00%CI
 ANDER2 1   b   0        102      12      30      21      5.95 (  2.63-  13.48)
*BEST   20  m   1         52       -       3       -      8.47 (  2.65-  27.11)
*DONTA2 13  m   0         11     333       4     127      1.05 (  0.34-   3.23)
 ENRIGH 9   m   0        113    1390      22     694      2.56 (  1.61-   4.09)
 ENRIGH 12  f   0         75    1182      26    1617      3.95 (  2.51-   6.20)
 Subtotal ENRIGH                                          3.20 (  2.31-   4.43)
 GULSVI 4   m   1         40       -       4       -      2.37 (  0.84-   6.66)
 GULSVI 6   f   1         28       -      16       -      1.82 (  0.98-   3.38)
 Subtotal GULSVI                                          1.95 (  1.15-   3.32)
*HAMMO2 7   m   1        369       -      20       -      8.64 (  5.51-  13.55)
*HAMMO2 16  f   1         24       -      21       -      6.44 (  3.57-  11.63)
 Subtotal HAMMO2                                          7.76 (  5.42-  11.09)
 HARDIE 15  m   1         48       -       2       -     13.38 (  3.14-  56.98)
 HARDIE 18  f   1         11       -      11       -      2.55 (  1.18-   5.48)
 Subtotal HARDIE                                          3.67 (  1.86-   7.23)
 HO     12  b   3          -       -       -       -      2.87 (  1.42-   5.81)
 HOZAWA 9   b   0        185    8775      13    6533     10.59 (  6.03-  18.61)
 HUHTI1 69  m   0         64     467       1     121     16.58 (  2.28- 120.74)
 HUHTI1 157 f   1          3       -      16       -      1.97 (  0.54-   7.17)
 Subtotal HUHTI1                                          3.72 (  1.26-  10.98)
*KAHN2  81  m   1        391       -      18       -     11.81 (  7.36-  18.97)
 LAVECC 30  b   6        789       -     595       -      2.05 (  1.81-   2.33)
 LEBOWI 36  b   3         31       -       5       -      4.86 (  1.86-  12.74)
 MILLER 9   m   1          6       -       0       -      6.56 (  0.37- 116.09)
 MILLER 12  f   1          2       -       1       -      6.03 (  0.12- 308.75)
 Subtotal MILLER                                          6.37 (  0.63-  64.77)
 NAWA   1   m   0        654    4086      32    1372      6.86 (  4.79-   9.84)
 OMORI  6   m   1        135       -       3       -      9.73 (  3.18-  29.82)
 PRATT  1   m   0        178     246      15      82      3.96 (  2.21-   7.09)
 RYDER  3   b   1         80       -      21       -     11.69 (  5.16-  26.51)
*SILVA  9   b   6          -       -       -       -      2.10 (  1.09-   4.06)
 SUTINE 1   b   0         63      28      28      45      3.62 (  1.89-   6.92)
 WANG2  1   b   0        188    3408      42    4209      5.53 (  3.94-   7.75)
 WEISS  16  m   0         28     223       1      35      4.39 (  0.58-  33.34)
Partial Totals          3670   20150     950   14856
*prospective study


 REF    NRR SEX ADJ             Ys       Ws       Qs       Ps
 ANDER2 1   b   0              1.78     5.74     2.10       0.00
*BEST   20  m   1              2.14     2.84     2.61       0.00
*DONTA2 13  m   0              0.05     3.03     3.87       0.93
 ENRIGH 9   m   0              0.94    17.71     0.99       0.00
 ENRIGH 12  f   0              1.37    18.78     0.71       0.00
 Subtotal ENRIGH               1.16    36.49     1.70
 GULSVI 4   m   1              0.86     3.58     0.36       0.10
 GULSVI 6   f   1              0.60    10.02     3.36       0.06
 Subtotal GULSVI               0.67    13.61     3.72
*HAMMO2 7   m   1              2.16    18.98    18.16       0.00
*HAMMO2 16  f   1              1.86    11.02     5.16       0.00
 Subtotal HAMMO2               2.05    29.99    23.32
 HARDIE 15  m   1              2.59     1.83     3.67       0.00
 HARDIE 18  f   1              0.94     6.52     0.38       0.02
 Subtotal HARDIE               1.30     8.35     4.05
 HO     12  b   3              1.05     7.74     0.12       0.00
 HOZAWA 9   b   0              2.36    12.11    16.92       0.00
 HUHTI1 69  m   0              2.81     0.97     2.59       0.01
 HUHTI1 157 f   1              0.68     2.30     0.57       0.30
 Subtotal HUHTI1               1.31     3.27     3.16
*KAHN2  81  m   1              2.47    17.14    28.56       0.00
 LAVECC 30  b   6              0.72   240.93    51.05       0.00
 LEBOWI 36  b   3              1.58     4.15     0.67       0.00
 MILLER 9   m   1              1.88     0.46     0.23       0.20
 MILLER 12  f   1              1.80     0.25     0.10       0.37
 Subtotal MILLER               1.85     0.71     0.33
 NAWA   1   m   0              1.93    29.63    16.57       0.00
 OMORI  6   m   1              2.28     3.07     3.69       0.00
 PRATT  1   m   0              1.38    11.29     0.44       0.00
 RYDER  3   b   1              2.46     5.74     9.41       0.00
*SILVA  9   b   6              0.74     8.89     1.69       0.03
 SUTINE 1   b   0              1.29     9.13     0.11       0.00
  ________________________________________________________________________________________________________________________
                                            International Evidence on Smoking and COPD, Phase 3, Analysis run on 27-SEP-10

                                                   Table 3 - A - 2 - 2

            IESCOPD - Meta-analysis of ever smoking, cigarettes (or all products if cigarettes not available)
                                                      Any Emphysema
                                                      Most-adjusted


 REF    NRR SEX ADJ             Ys       Ws       Qs       Ps
 WANG2  1   b   0              1.71    33.72     9.53       0.00
 WEISS  16  m   0              1.48     0.94     0.09       0.15

                       N       28
                      NS       22


                      Wt   488.50
                 Het Chi   183.71
                 Het  df       27
                 Het  P       ***
               Fixed  RR     3.25
                     RRl     2.97
                     RRu     3.55
                      P       +++
              Random  RR     4.57
                     RRl     3.40
                     RRu     6.15
                      P       +++
               Asymm  P        **


  ________________________________________________________________________________________________________________________
                                            International Evidence on Smoking and COPD, Phase 3, Analysis run on 27-SEP-10

                                                   Table 3 - A - 2 - 3

            IESCOPD - Meta-analysis of ever smoking, cigarettes (or all products if cigarettes not available)
                                                      Any Emphysema
                                                      Most-adjusted


                       N       28
                      NS       22


                      Wt   488.50
                 Het Chi   183.71
                 Het  df       27
                 Het  P       ***
               Fixed  RR     3.25
                     RRl     2.97
                     RRu     3.55
                      P       +++
              Random  RR     4.57
                     RRl     3.40
                     RRu     6.15
                      P       +++
               Asymm  P        **

                                   Sex
                             both      male    female     Total


                       N        9        13         6        28
                      NS        9        13         6        28


                      Wt   328.14    111.48     48.88    488.50
                 Het Chi    76.66     41.30     10.12    183.71
                 Het  df        8        12         5        27
                 Het  P       ***       ***       (*)       ***
               Fixed  RR     2.62      5.94      3.44      3.25
                     RRl     2.36      4.93      2.60      2.97
                     RRu     2.92      7.15      4.55      3.55
                      P       +++       +++       +++       +++
              Random  RR     4.47      5.59      3.25      4.57
                     RRl     2.72      3.71      2.07      3.40
                     RRu     7.34      8.42      5.10      6.15
                      P       +++       +++       +++       +++
             Between Chi                                  55.62
             Between  df                                      2
             Between  P                                     ***
             Btwn(F)  P                                       *

                                        Continent
                            NAmer    Europe      Asia  oth/mult     Total


                       N       14        10         4                  28
                      NS       11         7         4                  22


                      Wt   130.29    284.05     74.15              488.50
                 Het Chi    41.90     31.61      5.57              183.71
                 Het  df       13         9         3                  27
                 Het  P       ***       ***      N.S.                 ***
               Fixed  RR     5.52      2.19      5.76                3.25
                     RRl     4.65      1.95      4.59                2.97
                     RRu     6.55      2.46      7.23                3.55
                      P       +++       +++       +++                 +++
              Random  RR     5.46      3.13      5.59                4.57
                     RRl     3.87      2.01      3.93                3.40
                     RRu     7.71      4.87      7.95                6.15
                      P       +++       +++       +++                 +++
             Between Chi                                           104.64
             Between  df                                                2
             Between  P                                               ***
             Btwn(F)  P                                               ***


  ________________________________________________________________________________________________________________________
                                            International Evidence on Smoking and COPD, Phase 3, Analysis run on 27-SEP-10

                                                   Table 3 - A - 2 - 3

            IESCOPD - Meta-analysis of ever smoking, cigarettes (or all products if cigarettes not available)
                                                      Any Emphysema
                                                      Most-adjusted
                               Study type
                               CC        Pr        CS     Total


                       N                  6        22        28
                      NS                  5        17        22


                      Wt              61.89    426.61    488.50
                 Het Chi              29.27    119.19    183.71
                 Het  df                  5        21        27
                 Het  P                 ***       ***       ***
               Fixed  RR               6.58      2.93      3.25
                     RRl               5.13      2.67      2.97
                     RRu               8.44      3.22      3.55
                      P                 +++       +++       +++
              Random  RR               5.15      4.36      4.57
                     RRl               2.70      3.19      3.40
                     RRu               9.83      5.96      6.15
                      P                 +++       +++       +++
             Between Chi                                  35.25
             Between  df                                      1
             Between  P                                     ***
             Btwn(F)  P                                       *

                        Emp subtype
                             mort     other     Total


                       N        4        24        28
                      NS        3        19        22


                      Wt    49.98    438.52    488.50
                 Het Chi     2.54    123.30    183.71
                 Het  df        3        23        27
                 Het  P      N.S.       ***       ***
               Fixed  RR     9.00      2.89      3.25
                     RRl     6.82      2.63      2.97
                     RRu    11.88      3.18      3.55
                      P       +++       +++       +++
              Random  RR     9.00      4.00      4.57
                     RRl     6.82      2.97      3.40
                     RRu    11.88      5.37      6.15
                      P       +++       +++       +++
             Between Chi                        57.87
             Between  df                            1
             Between  P                           ***
             Btwn(F)  P                            **

                             Smoking product
                              any      cigs  cigsonly     Total


                       N        8        18         2        28
                      NS        7        14         2        23


                      Wt   282.61    197.30      8.59    488.50
                 Het Chi    28.07     61.71      0.24    183.71
                 Het  df        7        17         1        27
                 Het  P       ***       ***      N.S.       ***
               Fixed  RR     2.24      5.37      6.69      3.25
                     RRl     1.99      4.67      3.43      2.97
                     RRu     2.51      6.18     13.05      3.55
                      P       +++       +++       +++       +++
              Random  RR     3.52      4.89      6.69      4.57
                     RRl     2.19      3.62      3.43      3.40
                     RRu     5.67      6.60     13.05      6.15
                      P       +++       +++       +++       +++
             Between Chi                                  93.69
             Between  df                                      2
             Between  P                                     ***
             Btwn(F)  P                                     ***
  ________________________________________________________________________________________________________________________
                                            International Evidence on Smoking and COPD, Phase 3, Analysis run on 27-SEP-10

                                                   Table 3 - A - 2 - 3

            IESCOPD - Meta-analysis of ever smoking, cigarettes (or all products if cigarettes not available)
                                                      Any Emphysema
                                                      Most-adjusted
                                     Unexposed group
                          nev any   nev cig  nev+ any  nev+ cig     Total


                       N       12        16                            28
                      NS       11        13                            24


                      Wt   327.31    161.18                        488.50
                 Het Chi   108.37     43.63                        183.71
                 Het  df       11        15                            27
                 Het  P       ***       ***                           ***
               Fixed  RR     2.72      4.67                          3.25
                     RRl     2.44      4.00                          2.97
                     RRu     3.03      5.45                          3.55
                      P       +++       +++                           +++
              Random  RR     5.05      4.30                          4.57
                     RRl     2.98      3.18                          3.40
                     RRu     8.56      5.83                          6.15
                      P       +++       +++                           +++
             Between Chi                                            31.72
             Between  df                                                1
             Between  P                                               ***
             Btwn(F)  P                                                 *

                        Unexposed group (combining nev+ with main levels)
                          nev any   nev cig     Total


                       N       12        16        28
                      NS       11        13        24


                      Wt   327.31    161.18    488.50
                 Het Chi   108.37     43.63    183.71
                 Het  df       11        15        27
                 Het  P       ***       ***       ***
               Fixed  RR     2.72      4.67      3.25
                     RRl     2.44      4.00      2.97
                     RRu     3.03      5.45      3.55
                      P       +++       +++       +++
              Random  RR     5.05      4.30      4.57
                     RRl     2.98      3.18      3.40
                     RRu     8.56      5.83      6.15
                      P       +++       +++       +++
             Between Chi                        31.72
             Between  df                            1
             Between  P                           ***
             Btwn(F)  P                             *


  ________________________________________________________________________________________________________________________
                                            International Evidence on Smoking and COPD, Phase 3, Analysis run on 27-SEP-10

                                                   Table 3 - A - 2 - 4

            IESCOPD - Meta-analysis of ever smoking, cigarettes (or all products if cigarettes not available)
                                                      Any Emphysema
                                                      Least-adjusted


     REF|NRR|X|SEX|AGEL|AGEH|     REGION|BEGYR|PUBYR|STTYP|ONSET|      DISEAS|ADJ|SMOKSTA|   PRODUCT|    UNEXP|

  ANDER2   1     b   15   99      Am:USA     *  1966    CS  Prev  Emp:viscomp   0    Ever  Cigs only   Nev any
    BEST  20     m   30   97   Am:Canada  1955  1967    Pr   Inc     Emp:mort   1    Ever  Cigs only   Nev any
  DONTA2  13     m   25   84 Eu:SE/Balkn  1960  1984    Pr   Inc Emp:diagnosd   0    Ever       Cigs  Nev cigs
  ENRIGH   9     m   65   99      Am:USA  1989  1994    CS  Prev Emp:diagnosd   0    Ever       Cigs  Nev cigs
  ENRIGH  12     f   65   99      Am:USA  1989  1994    CS  Prev Emp:diagnosd   0    Ever       Cigs  Nev cigs
  GULSVI   3 x   m   15   70    Eu:Scand  1972  1979    CS  Prev Emp:diagnosd   0    Ever        Any   Nev any
  GULSVI   5 x   f   15   70    Eu:Scand  1972  1979    CS  Prev Emp:diagnosd   0    Ever        Any   Nev any
  HAMMO2   7     m   35   99      Am:USA  1959  1966    Pr   Inc     Emp:mort   1    Ever       Cigs   Nev any
  HAMMO2  16     f   35   99      Am:USA  1959  1966    Pr   Inc     Emp:mort   1    Ever       Cigs  Nev cigs
  HARDIE  15     m   70   99    Eu:Scand  1998  2005    CS  Prev Emp:diagnosd   1    Ever       Cigs  Nev cigs
  HARDIE  18     f   70   99    Eu:Scand  1998  2005    CS  Prev Emp:diagnosd   1    Ever       Cigs  Nev cigs
      HO  12     b   70   99   Asia:FarE  1991  1999    CS  Prev Emp:self-rep   3    Ever       Cigs  Nev cigs
  HOZAWA   9     b   45   64      Am:USA  1987  2006    CS  Prev Emp:diagnosd   0    Ever       Cigs  Nev cigs
  HUHTI1  69     m   40   64    Eu:Scand  1961  1965    CS  Prev    Emp:other   0    Ever        Any   Nev any
  HUHTI1 156 x   f   40   64    Eu:Scand  1961  1965    CS  Prev    Emp:other   0    Ever       Cigs  Nev cigs
   KAHN2  81     m   31   84      Am:USA  1954  1966    Pr   Inc     Emp:mort   1    Ever       Cigs   Nev any
  LAVECC  24 x   m   15   99     Eu:West  1983  1988    CS  Prev Emp:self-rep   0    Ever        Any   Nev any
  LAVECC  27 x   f   15   99     Eu:West  1983  1988    CS  Prev Emp:self-rep   0    Ever        Any   Nev any
  LEBOWI  33 x   b   15   96      Am:USA  1972  1977    CS  Prev Emp:diagnosd   2    Ever       Cigs  Nev cigs
  MILLER   9     m   15   99      Am:USA  1978  1988    CS  Prev Emp:diagnosd   1    Ever       Cigs  Nev cigs
  MILLER  12     f   15   99      Am:USA  1978  1988    CS  Prev Emp:diagnosd   1    Ever       Cigs  Nev cigs
    NAWA   1     m   50   69   Asia:FarE  1998  2002    CS  Prev  Emp:viscomp   0    Ever       Cigs  Nev cigs
   OMORI   3 x   m   40   69   Asia:FarE     *  2006    CS  Prev  Emp:viscomp   0    Ever       Cigs  Nev cigs
   PRATT   1     m   15   99      Am:USA     *  1980    CS  Prev  Emp:viscomp   0    Ever        Any   Nev any
   RYDER   1 x   m   22   95       Eu:UK     *  1971    CS  Prev  Emp:viscomp   0    Ever        Any   Nev any
   RYDER   2 x   f   22   95       Eu:UK     *  1971    CS  Prev  Emp:viscomp   0    Ever        Any   Nev any
   SILVA   9     b   20   99      Am:USA  1972  2004    Pr   Inc    Emp:other   6    Ever       Cigs  Nev cigs
  SUTINE   1     b   10   99    Eu:Scand  1971  1978    CS  Prev  Emp:viscomp   0    Ever        Any   Nev any
   WANG2   1     b   19   92   Asia:FarE  1996  2001    CS  Prev  Emp:viscomp   0    Ever       Cigs  Nev cigs
   WEISS  16     m   50   69      Am:USA  1961  1963    CS  Prev  Emp:viscomp   0    Ever        Any   Nev any


  ________________________________________________________________________________________________________________________
                                            International Evidence on Smoking and COPD, Phase 3, Analysis run on 27-SEP-10

                                                   Table 3 - A - 2 - 5

            IESCOPD - Meta-analysis of ever smoking, cigarettes (or all products if cigarettes not available)
                                                      Any Emphysema
                                                      Least-adjusted


                        Number Exposed  Non-exposed
 REF    NRR SEX ADJ     Case    Cont    Case    Cont      RR        95.00%CI
 ANDER2 1   b   0        102      12      30      21      5.95 (  2.63-  13.48)
*BEST   20  m   1         52       -       3       -      8.47 (  2.65-  27.11)
*DONTA2 13  m   0         11     333       4     127      1.05 (  0.34-   3.23)
 ENRIGH 9   m   0        113    1390      22     694      2.56 (  1.61-   4.09)
 ENRIGH 12  f   0         75    1182      26    1617      3.95 (  2.51-   6.20)
 Subtotal ENRIGH                                          3.20 (  2.31-   4.43)
 GULSVI 3   m   0         40    5812       4    2090      3.60 (  1.29-  10.06)
 GULSVI 5   f   0         28    4913      16    4231      1.51 (  0.81-   2.79)
 Subtotal GULSVI                                          1.90 (  1.12-   3.21)
*HAMMO2 7   m   1        369       -      20       -      8.64 (  5.51-  13.55)
*HAMMO2 16  f   1         24       -      21       -      6.44 (  3.57-  11.63)
 Subtotal HAMMO2                                          7.76 (  5.42-  11.09)
 HARDIE 15  m   1         48       -       2       -     13.38 (  3.14-  56.98)
 HARDIE 18  f   1         11       -      11       -      2.55 (  1.18-   5.48)
 Subtotal HARDIE                                          3.67 (  1.86-   7.23)
 HO     12  b   3          -       -       -       -      2.87 (  1.42-   5.81)
 HOZAWA 9   b   0        185    8775      13    6533     10.59 (  6.03-  18.61)
 HUHTI1 69  m   0         64     467       1     121     16.58 (  2.28- 120.74)
 HUHTI1 156 f   0          3     111      16     693      1.17 (  0.34-   4.08)
 Subtotal HUHTI1                                          2.48 (  0.86-   7.15)
*KAHN2  81  m   1        391       -      18       -     11.81 (  7.36-  18.97)
 LAVECC 24  m   0        700   19775     183   14129      2.73 (  2.32-   3.22)
 LAVECC 27  f   0         89    7548     412   29448      0.84 (  0.67-   1.06)
 Subtotal LAVECC                                          1.84 (  1.61-   2.10)
 LEBOWI 33  b   2         31       -       5       -      5.08 (  1.95-  13.23)
 MILLER 9   m   1          6       -       0       -      6.56 (  0.37- 116.09)
 MILLER 12  f   1          2       -       1       -      6.03 (  0.12- 308.75)
 Subtotal MILLER                                          6.37 (  0.63-  64.77)
 NAWA   1   m   0        654    4086      32    1372      6.86 (  4.79-   9.84)
 OMORI  3   m   0        135     380       3      97     11.49 (  3.58-  36.85)
 PRATT  1   m   0        178     246      15      82      3.96 (  2.21-   7.09)
 RYDER  1   m   0         56      14       7       9      5.14 (  1.63-  16.21)
 RYDER  2   f   0         24      12      14      43      6.14 (  2.45-  15.39)
 Subtotal RYDER                                           5.73 (  2.80-  11.74)
*SILVA  9   b   6          -       -       -       -      2.10 (  1.09-   4.06)
 SUTINE 1   b   0         63      28      28      45      3.62 (  1.89-   6.92)
 WANG2  1   b   0        188    3408      42    4209      5.53 (  3.94-   7.75)
 WEISS  16  m   0         28     223       1      35      4.39 (  0.58-  33.34)
Partial Totals          3670   58715     950   65596
*prospective study


 REF    NRR SEX ADJ             Ys       Ws       Qs       Ps
 ANDER2 1   b   0              1.78     5.74     2.35       0.00
*BEST   20  m   1              2.14     2.84     2.80       0.00
*DONTA2 13  m   0              0.05     3.03     3.64       0.93
 ENRIGH 9   m   0              0.94    17.71     0.72       0.00
 ENRIGH 12  f   0              1.37    18.78     0.98       0.00
 Subtotal ENRIGH               1.16    36.49     1.71
 GULSVI 3   m   0              1.28     3.63     0.07       0.01
 GULSVI 5   f   0              0.41    10.14     5.46       0.19
 Subtotal GULSVI               0.64    13.76     5.53
*HAMMO2 7   m   1              2.16    18.98    19.45       0.00
*HAMMO2 16  f   1              1.86    11.02     5.69       0.00
 Subtotal HAMMO2               2.05    29.99    25.14
 HARDIE 15  m   1              2.59     1.83     3.84       0.00
 HARDIE 18  f   1              0.94     6.52     0.28       0.02
 Subtotal HARDIE               1.30     8.35     4.13
 HO     12  b   3              1.05     7.74     0.06       0.00
 HOZAWA 9   b   0              2.36    12.11    17.91       0.00
 HUHTI1 69  m   0              2.81     0.97     2.70       0.01
 HUHTI1 156 f   0              0.16     2.46     2.40       0.80
 Subtotal HUHTI1               0.91     3.44     5.10
*KAHN2  81  m   1              2.47    17.14    30.09       0.00
 LAVECC 24  m   0              1.01   142.56     2.74       0.00
 LAVECC 27  f   0             -0.17    72.31   125.06       0.15
 Subtotal LAVECC               0.61   214.87   127.80
 LEBOWI 33  b   2              1.63     4.19     0.97       0.00
 MILLER 9   m   1              1.88     0.46     0.25       0.20
 MILLER 12  f   1              1.80     0.25     0.11       0.37
 Subtotal MILLER               1.85     0.71     0.36
  ________________________________________________________________________________________________________________________
                                            International Evidence on Smoking and COPD, Phase 3, Analysis run on 27-SEP-10

                                                   Table 3 - A - 2 - 5

            IESCOPD - Meta-analysis of ever smoking, cigarettes (or all products if cigarettes not available)
                                                      Any Emphysema
                                                      Least-adjusted


 REF    NRR SEX ADJ             Ys       Ws       Qs       Ps
 NAWA   1   m   0              1.93    29.63    18.12       0.00
 OMORI  3   m   0              2.44     2.83     4.76       0.00
 PRATT  1   m   0              1.38    11.29     0.60       0.00
 RYDER  1   m   0              1.64     2.91     0.71       0.01
 RYDER  2   f   0              1.82     4.55     2.05       0.00
 Subtotal RYDER                1.75     7.47     2.76
*SILVA  9   b   6              0.74     8.89     1.44       0.03
 SUTINE 1   b   0              1.29     9.13     0.18       0.00
 WANG2  1   b   0              1.71    33.72    10.79       0.00
 WEISS  16  m   0              1.48     0.94     0.11       0.15

                       N       30
                      NS       22


                      Wt   464.29
                 Het Chi   266.33
                 Het  df       29
                 Het  P       ***
               Fixed  RR     3.14
                     RRl     2.87
                     RRu     3.44
                      P       +++
              Random  RR     4.21
                     RRl     3.05
                     RRu     5.81
                      P       +++
               Asymm  P       (*)


  ________________________________________________________________________________________________________________________
                                            International Evidence on Smoking and COPD, Phase 3, Analysis run on 27-SEP-10

                                                   Table 3 - A - 2 - 6

            IESCOPD - Meta-analysis of ever smoking, cigarettes (or all products if cigarettes not available)
                                                      Any Emphysema
                                                      Least-adjusted


                       N       30
                      NS       22


                      Wt   464.29
                 Het Chi   266.33
                 Het  df       29
                 Het  P       ***
               Fixed  RR     3.14
                     RRl     2.87
                     RRu     3.44
                      P       +++
              Random  RR     4.21
                     RRl     3.05
                     RRu     5.81
                      P       +++
               Asymm  P       (*)

                                   Sex
                             both      male    female     Total


                       N        7        15         8        30
                      NS        7        15         8        30


                      Wt    81.52    256.76    126.02    464.29
                 Het Chi    17.35     79.09     76.47    266.33
                 Het  df        6        14         7        29
                 Het  P        **       ***       ***       ***
               Fixed  RR     4.91      3.88      1.53      3.14
                     RRl     3.95      3.43      1.28      2.87
                     RRu     6.10      4.38      1.82      3.44
                      P       +++       +++       +++       +++
              Random  RR     4.60      5.33      2.56      4.21
                     RRl     3.07      3.57      1.23      3.05
                     RRu     6.87      7.97      5.32      5.81
                      P       +++       +++         +       +++
             Between Chi                                  93.43
             Between  df                                      2
             Between  P                                     ***
             Btwn(F)  P                                      **


  ________________________________________________________________________________________________________________________
                                            International Evidence on Smoking and COPD, Phase 3, Analysis run on 27-SEP-10

                                                   Table 3 - A - 2 - 7

            IESCOPD - Meta-analysis of ever smoking, cigarettes (or all products if cigarettes not available)
                                                      Any Emphysema
                                 Excluded studies (and stage at which they were excluded)


1       CLARK COTTON  MEYER REMYJA RUTGER SNYDER SOBRAX     SU TAKEMU  WANG4   WEIR WHICKE ZALACA
2      ALDERS ALESSA  AMIGO ANDER1 ANDER3   BANG  BECK1  BECK2 BEDNAR BJORNS BROGGE  BROWN CERVER CHAPMA  CHEN1  CHEN2
        CHEN3  CHENG CLEMEN COATES  COCCI COLLEG  DEAN1  DEAN2  DEANE DEJONG DEMARC DETORR DICKIN  DOLL1  DOLL2 DONTA1
       DOPICO EHRLIC EKBERG ENSTRO FERRI1 FERRI2 FERRI3  FIDAN FINKLE FLETCH FORAST FOXMAN FUKUCH GEIJER GODTFR GOLDBE
       HAENSZ HARIKK HARRIS HAWTHO  HAYES HEDMAN HIGGI2 HIGGI3 HIGGI4 HIGGI6 HOLLA2 HOLLNA  HOUSE HRUBEC HUCHON HUHTI2
       HUHTI3 ITABAS JACOBS JAENDI JENSEN JINDA2 JOHANN  JOSHI JOUSI1 KACHEL KARAKA KATANC   KATO KHOURY    KIM  KIRAZ
       KLAYTO KOJIMA KOTAN1 KOTAN2 KRZYZA  KUBIK KULLER    LAI   LAM1   LAM2   LAM3 LAMBER  LANGE LANGE2 LANGHA    LEE
         LIAW LINDBE LINDST   LIU1   LIU2 LUNDB1 LUNDB2  MADOR MAGNUS MANFRE MANNI1 MANNI2 MANNI3 MARAN1 MARAN2 MARCUS
       MATHES MELLST MENEZ1 MENEZ2 MENEZ3 MENEZ4 MENEZ5 MENEZ6  MEREN  MILNE MOLLER MONTNE MUELLE NEJJAR NIEPSU NIHLEN
       NILSSO OGILVI OSWAL1 OSWAL2 PANDEY   PEAT PELKON PEREZP   PETO  PRICE   REID RENWIC RICCIO RIMING SARGEA SAWICK
       SCHWAR SHAHAB  SHARP SHIMUR   SHIN SICHLE SOBRAD SPEIZE STERLI STJERN  STROM SUADIC  TAGER TAGER2   TANG   THUN
         TODD TROISI TRUPIN TSUSHI TVERDA URRUTI VESTBO VIEGI1 VIEGI2 VINEIS VOLLM1 VOLLM2 VONHER WAGEN2   WALD WATSON
          WIG WILHEL WILSO1 WOJTYN  WOODS  WOOLF   XIAO     XU YAMAGU   YUAN ZIELI1 ZIELI2 ZIETKO   ZOIA
3      WILSO2
4      AUERBA HIRAYA   KAHN VIKGRE    WEN


  ________________________________________________________________________________________________________________________
                                            International Evidence on Smoking and COPD, Phase 3, Analysis run on 27-SEP-10

                                                   Table 3 - A - 2 - 8

            IESCOPD - Meta-analysis of ever smoking, cigarettes (or all products if cigarettes not available)
                                                      Any Emphysema
                                             Potentially overlapping studies


     REF| REFGP|PRINC|                     OVERLAP|

  HOZAWA HOZAWA     1         ENRIGH/HOZAWA/HARIKK
  ENRIGH ENRIGH     1         ENRIGH/HOZAWA/HARIKK
  DONTA2 JACOBS     2  JACOBS/DONTA1/DONTA2/PELKON
  HUHTI1 HUHTI1     1                HUHTI1/HUHTI2
  HARDIE HARDIE     1         HARDIE/JOHANN/BROGGE
  HAMMO2 HAMMO2     1                HAMMO2/ENSTRO
  LEBOWI LEBOWI     1                 LEBOWI/SILVA
   SILVA  SILVA     1                 LEBOWI/SILVA
   KAHN2   KAHN     2                   KAHN/KAHN2


  ________________________________________________________________________________________________________________________
                                            International Evidence on Smoking and COPD, Phase 3, Analysis run on 27-SEP-10

                                                    Table 3 - B - 1 -

           IESCOPD - Meta-analysis of current smoking, any product (or cigarettes if all product not available)
                                                      Any Emphysema


This analysis is restricted to results for:
1) Eligible study on database
2) Outcome Emphysema
3) Non-dose-response data
4) Current smoking
5) Results complete enough for use in meta-analysis

Within each study, results are then selected (in the following order of preference, within each sex) for:
6) UNEXP   : never any, never cigarettes, other
7) PROD    : any product, cigarettes, cigarettes only
8) For overlapping studies: principal rather than subsidiary studies
and then for single sex results (m, f) in preference to results for both sexes combined (b).

Results adjusted for the most potential confounders are then chosen in Sections -1 to -3
and results adjusted for the least confounders in Sections -4 to -6. (Those least adjusted results which
actually differ from the most adjusted are marked 'x' in column X in Section -4)

Section -7 shows excluded studies, together with the stage (as above) at which no qualifying
results were found.

Section -8 lists the potentially overlapping studies which have been included (1=principal, 2=subsidiary),
and any results which would have been included in preference except that they had data not complete enough
for use in meta-analysis. It also lists their significance (yes/no), if known.


  ________________________________________________________________________________________________________________________
                                            International Evidence on Smoking and COPD, Phase 3, Analysis run on 27-SEP-10

                                                   Table 3 - B - 1 - 1

           IESCOPD - Meta-analysis of current smoking, any product (or cigarettes if all product not available)
                                                      Any Emphysema
                                                      Most-adjusted


     REF|NRR|SEX|AGEL|AGEH|     REGION|BEGYR|PUBYR|STTYP|ONSET|      DISEAS|ADJ|SMOKSTA|   PRODUCT|    UNEXP|

  AUERBA   2   m   15   99      Am:USA  1963  1972    CS  Prev  Emp:viscomp   1 Current       Cigs   Nev any
    BEST  18   m   30   97   Am:Canada  1955  1967    Pr   Inc     Emp:mort   1 Current  Cigs only   Nev any
  DONTA2  11   m   25   84 Eu:SE/Balkn  1960  1984    Pr   Inc Emp:diagnosd   0 Current       Cigs  Nev cigs
  ENRIGH   7   m   65   99      Am:USA  1989  1994    CS  Prev Emp:diagnosd   0 Current       Cigs  Nev cigs
  ENRIGH  10   f   65   99      Am:USA  1989  1994    CS  Prev Emp:diagnosd   0 Current       Cigs  Nev cigs
  HARDIE  13   m   70   99    Eu:Scand  1998  2005    CS  Prev Emp:diagnosd   1 Current       Cigs  Nev cigs
  HARDIE  16   f   70   99    Eu:Scand  1998  2005    CS  Prev Emp:diagnosd   1 Current       Cigs  Nev cigs
  HIRAYA   1   m   40   99   Asia:FarE  1965  1982    Pr   Inc     Emp:mort   1 Current       Cigs  Nev cigs
  HIRAYA   2   f   40   99   Asia:FarE  1965  1982    Pr   Inc     Emp:mort   1 Current       Cigs  Nev cigs
      HO   9   b   70   99   Asia:FarE  1991  1999    CS  Prev Emp:self-rep   3 Current       Cigs  Nev cigs
  HUHTI1  67   m   40   64    Eu:Scand  1961  1965    CS  Prev    Emp:other   0 Current        Any   Nev any
  HUHTI1 142   f   40   64    Eu:Scand  1961  1965    CS  Prev    Emp:other   0 Current        Any   Nev any
   KAHN2  46   m   31   84      Am:USA  1954  1966    Pr   Inc     Emp:mort   1 Current        Any   Nev any
  LAVECC  28   b   15   99     Eu:West  1983  1988    CS  Prev Emp:self-rep   6 Current        Any   Nev any
  LEBOWI  34   b   15   96      Am:USA  1972  1977    CS  Prev Emp:diagnosd   3 Current       Cigs  Nev cigs
  MILLER   7   m   15   99      Am:USA  1978  1988    CS  Prev Emp:diagnosd   1 Current       Cigs  Nev cigs
  MILLER  10   f   15   99      Am:USA  1978  1988    CS  Prev Emp:diagnosd   1 Current       Cigs  Nev cigs
   OMORI   4   m   40   69   Asia:FarE     *  2006    CS  Prev  Emp:viscomp   1 Current       Cigs  Nev cigs
   SILVA   7   b   20   99      Am:USA  1972  2004    Pr   Inc    Emp:other   6 Current       Cigs  Nev cigs
  VIKGRE   1   m   61   68    Eu:Scand  1994  2004    Pr   Inc  Emp:viscomp   0 Current       Cigs  Nev cigs
   WEISS  14   m   50   69      Am:USA  1961  1963    CS  Prev  Emp:viscomp   0 Current        Any   Nev any
     WEN  18   m   35   99   Asia:FarE  1982  2004    Pr   Inc     Emp:mort   1 Current       Cigs  Nev cigs


  ________________________________________________________________________________________________________________________
                                            International Evidence on Smoking and COPD, Phase 3, Analysis run on 27-SEP-10

                                                   Table 3 - B - 1 - 2

           IESCOPD - Meta-analysis of current smoking, any product (or cigarettes if all product not available)
                                                      Any Emphysema
                                                      Most-adjusted


                        Number Exposed  Non-exposed
 REF    NRR SEX ADJ     Case    Cont    Case    Cont      RR        95.00%CI
 AUERBA 2   m   1        816       -      20       -    489.54 (211.74-1131.81)
*BEST   18  m   1         40       -       3       -      7.72 (  2.39-  24.95)
*DONTA2 11  m   0         10     275       4     127      1.15 (  0.37-   3.61)
 ENRIGH 7   m   0         32     199      22     694      5.07 (  2.88-   8.93)
 ENRIGH 10  f   0         18     364      26    1617      3.08 (  1.67-   5.67)
 Subtotal ENRIGH                                          4.03 (  2.66-   6.10)
 HARDIE 13  m   1         11       -       2       -     15.04 (  3.40-  66.58)
 HARDIE 16  f   1          5       -      11       -      3.59 (  1.38-   9.29)
 Subtotal HARDIE                                          5.45 (  2.44-  12.16)
*HIRAYA 1   m   1        156       -      17       -      2.22 (  1.34-   3.67)
*HIRAYA 2   f   1         14       -      42       -      2.72 (  1.48-   5.00)
 Subtotal HIRAYA                                          2.41 (  1.64-   3.55)
 HO     9   b   3          -       -       -       -      2.30 (  0.94-   5.62)
 HUHTI1 67  m   0         53     337       1     121     19.03 (  2.60- 139.11)
 HUHTI1 142 f   0          1      83      16     693      0.52 (  0.07-   3.99)
 Subtotal HUHTI1                                          3.28 (  0.79-  13.58)
*KAHN2  46  m   1        284       -      18       -      9.09 (  5.65-  14.64)
 LAVECC 28  b   6        437       -     595       -      1.76 (  1.52-   2.04)
 LEBOWI 34  b   3         14       -       5       -      3.82 (  1.34-  10.88)
 MILLER 7   m   1          4       -       0       -      7.21 (  0.39- 133.19)
 MILLER 10  f   1          2       -       1       -      9.07 (  0.18- 446.54)
 Subtotal MILLER                                          7.83 (  0.76-  81.05)
 OMORI  4   m   1        116       -       3       -     14.50 (  4.63-  45.40)
*SILVA  7   b   6          -       -       -       -      4.10 (  2.03-   8.18)
*VIKGRE 1   m   0          2      40       0      26      3.27~(  0.16-  65.49)
 WEISS  14  m   0         23     160       1      35      5.03 (  0.66-  38.51)
*WEN    18  m   1         12       -       -       -      1.12 (  0.44-   2.88)
Partial Totals          2050    1458     787    3313
*prospective study                                        ~ With 0.5 adjustment for zero


 REF    NRR SEX ADJ             Ys       Ws       Qs       Ps
 AUERBA 2   m   1              6.19     5.47   149.81       0.00
*BEST   18  m   1              2.04     2.79     3.28       0.00
*DONTA2 11  m   0              0.14     2.95     1.97       0.80
 ENRIGH 7   m   0              1.62    12.02     5.30       0.00
 ENRIGH 10  f   0              1.12    10.27     0.28       0.00
 Subtotal ENRIGH               1.39    22.29     5.58
 HARDIE 13  m   1              2.71     1.74     5.32       0.00
 HARDIE 16  f   1              1.28     4.23     0.43       0.01
 Subtotal HARDIE               1.70     5.96     5.75
*HIRAYA 1   m   1              0.80    15.14     0.40       0.00
*HIRAYA 2   f   1              1.00    10.37     0.02       0.00
 Subtotal HIRAYA               0.88    25.51     0.42
 HO     9   b   3              0.83     4.81     0.08       0.07
 HUHTI1 67  m   0              2.95     0.97     3.83       0.00
 HUHTI1 142 f   0             -0.65     0.93     2.41       0.53
 Subtotal HUHTI1               1.19     1.90     6.24
*KAHN2  46  m   1              2.21    16.95    26.38       0.00
 LAVECC 28  b   6              0.57   177.48    27.60       0.00
 LEBOWI 34  b   3              1.34     3.50     0.51       0.01
 MILLER 7   m   1              1.98     0.45     0.47       0.18
 MILLER 10  f   1              2.20     0.25     0.39       0.27
 Subtotal MILLER               2.06     0.70     0.86
 OMORI  4   m   1              2.67     2.95     8.67       0.00
*SILVA  7   b   6              1.41     7.91     1.61       0.00
*VIKGRE 1   m   0              1.19     0.43     0.02       0.44
 WEISS  14  m   0              1.62     0.93     0.40       0.12
*WEN    18  m   1              0.11     4.35     3.12       0.81


  ________________________________________________________________________________________________________________________
                                            International Evidence on Smoking and COPD, Phase 3, Analysis run on 27-SEP-10

                                                   Table 3 - B - 1 - 2

           IESCOPD - Meta-analysis of current smoking, any product (or cigarettes if all product not available)
                                                      Any Emphysema
                                                      Most-adjusted


                       N       22
                      NS       17


                      Wt   286.89
                 Het Chi   242.28
                 Het  df       21
                 Het  P       ***
               Fixed  RR     2.61
                     RRl     2.33
                     RRu     2.93
                      P       +++
              Random  RR     4.87
                     RRl     2.83
                     RRu     8.41
                      P       +++
               Asymm  P         *


  ________________________________________________________________________________________________________________________
                                            International Evidence on Smoking and COPD, Phase 3, Analysis run on 27-SEP-10

                                                   Table 3 - B - 1 - 3

           IESCOPD - Meta-analysis of current smoking, any product (or cigarettes if all product not available)
                                                      Any Emphysema
                                                      Most-adjusted


                       N       22
                      NS       17


                      Wt   286.89
                 Het Chi   242.28
                 Het  df       21
                 Het  P       ***
               Fixed  RR     2.61
                     RRl     2.33
                     RRu     2.93
                      P       +++
              Random  RR     4.87
                     RRl     2.83
                     RRu     8.41
                      P       +++
               Asymm  P         *

                                   Sex
                             both      male    female     Total


                       N        4        13         5        22
                      NS        4        13         5        22


                      Wt   193.70     67.14     26.04    286.89
                 Het Chi     7.52    149.06      3.32    242.28
                 Het  df        3        12         4        21
                 Het  P       (*)       ***      N.S.       ***
               Fixed  RR     1.86      6.71      2.85      2.61
                     RRl     1.62      5.29      1.94      2.33
                     RRu     2.14      8.53      4.18      2.93
                      P       +++       +++       +++       +++
              Random  RR     2.54      7.66      2.85      4.87
                     RRl     1.54      3.00      1.94      2.83
                     RRu     4.20     19.61      4.18      8.41
                      P       +++       +++       +++       +++
             Between Chi                                  82.38
             Between  df                                      2
             Between  P                                     ***
             Btwn(F)  P                                       *

                                        Continent
                            NAmer    Europe      Asia  oth/mult     Total


                       N       10         7         5                  22
                      NS        8         5         4                  17


                      Wt    60.55    188.73     37.61              286.89
                 Het Chi   110.36     17.45     12.23              242.28
                 Het  df        9         6         4                  21
                 Het  P       ***        **         *                 ***
               Fixed  RR     8.13      1.83      2.52                2.61
                     RRl     6.32      1.58      1.83                2.33
                     RRu    10.45      2.11      3.47                2.93
                      P       +++       +++       +++                 +++
              Random  RR     8.99      2.88      2.74                4.87
                     RRl     3.34      1.36      1.50                2.83
                     RRu    24.26      6.09      4.99                8.41
                      P       +++        ++        ++                 +++
             Between Chi                                           102.23
             Between  df                                                2
             Between  P                                               ***
             Btwn(F)  P                                                **


  ________________________________________________________________________________________________________________________
                                            International Evidence on Smoking and COPD, Phase 3, Analysis run on 27-SEP-10

                                                   Table 3 - B - 1 - 3

           IESCOPD - Meta-analysis of current smoking, any product (or cigarettes if all product not available)
                                                      Any Emphysema
                                                      Most-adjusted
                        National cigarette tobacco type (excluding mixed/unkown)
                          blended  virginia     Total


                       N       20         1        21
                      NS       15         1        16


                      Wt   279.74      2.79    282.54
                 Het Chi   235.88      0.00    239.12
                 Het  df       19         0        20
                 Het  P       ***      N.S.       ***
               Fixed  RR     2.62      7.72      2.65
                     RRl     2.33      2.39      2.35
                     RRu     2.94     24.94      2.97
                      P       +++       +++       +++
              Random  RR     5.17      7.72      5.28
                     RRl     2.89      2.39      3.00
                     RRu     9.27     24.94      9.29
                      P       +++       +++       +++
             Between Chi                         3.24
             Between  df                            1
             Between  P                           (*)
             Btwn(F)  P                          N.S.

                                        Start year of study
                            <1970   1970-79   1980-89   1990-99     2000+   unknown     Total


                       N        9         4         4         4                   1        22
                      NS        7         3         3         3                   1        17


                      Wt    56.50     12.12    204.13     11.20                2.95    286.89
                 Het Chi   145.30      0.32     16.23      4.51                0.00    242.28
                 Het  df        8         3         3         3                   0        21
                 Het  P       ***      N.S.        **      N.S.                N.S.       ***
               Fixed  RR     6.26      4.17      1.91      3.69               14.50      2.61
                     RRl     4.82      2.38      1.66      2.05                4.63      2.33
                     RRu     8.12      7.32      2.19      6.63               45.41      2.93
                      P       +++       +++       +++       +++                 +++       +++
              Random  RR     6.38      4.17      2.43      4.02               14.50      4.87
                     RRl     1.88      2.38      1.35      1.84                4.63      2.83
                     RRu    21.69      7.32      4.38      8.78               45.41      8.41
                      P        ++       +++        ++       +++                 +++       +++
             Between Chi                                                                75.92
             Between  df                                                                    4
             Between  P                                                                   ***
             Btwn(F)  P                                                                  N.S.

                                Publication year
                            <1980   1980-89   1990-99     2000+     Total


                       N        7         6         3         6        22
                      NS        6         4         2         5        17


                      Wt    31.54    206.64     27.10     21.60    286.89
                 Het Chi    90.12      4.58      2.63     15.06    242.28
                 Het  df        6         5         2         5        21
                 Het  P       ***      N.S.      N.S.         *       ***
               Fixed  RR    15.01      1.83      3.65      4.04      2.61
                     RRl    10.59      1.59      2.50      2.65      2.33
                     RRu    21.28      2.10      5.31      6.16      2.93
                      P       +++       +++       +++       +++       +++
              Random  RR    10.21      1.83      3.57      4.57      4.87
                     RRl     2.09      1.59      2.30      2.05      2.83
                     RRu    49.96      2.10      5.55     10.20      8.41
                      P        ++       +++       +++       +++       +++
             Between Chi                                           129.89
             Between  df                                                3
             Between  P                                               ***
             Btwn(F)  P                                                **
  ________________________________________________________________________________________________________________________
                                            International Evidence on Smoking and COPD, Phase 3, Analysis run on 27-SEP-10

                                                   Table 3 - B - 1 - 3

           IESCOPD - Meta-analysis of current smoking, any product (or cigarettes if all product not available)
                                                      Any Emphysema
                                                      Most-adjusted
                               Study type
                               CC        Pr        CS     Total


                       N                  8        14        22
                      NS                  7        10        17


                      Wt              60.90    225.99    286.89
                 Het Chi              30.41    203.76    242.28
                 Het  df                  7        13        21
                 Het  P                 ***       ***       ***
               Fixed  RR               3.61      2.39      2.61
                     RRl               2.81      2.10      2.33
                     RRu               4.64      2.73      2.93
                      P                 +++       +++       +++
              Random  RR               3.12      6.60      4.87
                     RRl               1.74      2.74      2.83
                     RRu               5.60     15.92      8.41
                      P                 +++       +++       +++
             Between Chi                                   8.10
             Between  df                                      1
             Between  P                                      **
             Btwn(F)  P                                    N.S.

                                    Lowest age in RR
                        <25/unlim     25-39       40+   unknown     Total


                       N        6         4        12                  22
                      NS        5         4         8                  17


                      Wt   195.07     27.05     64.77              286.89
                 Het Chi   173.85     22.66     22.37              242.28
                 Het  df        5         3        11                  21
                 Het  P       ***       ***         *                 ***
               Fixed  RR     2.17      5.09      3.43                2.61
                     RRl     1.89      3.49      2.69                2.33
                     RRu     2.50      7.42      4.37                2.93
                      P       +++       +++       +++                 +++
              Random  RR     9.92      3.19      3.80                4.87
                     RRl     1.41      0.95      2.55                2.83
                     RRu    69.88     10.75      5.66                8.41
                      P         +       (+)       +++                 +++
             Between Chi                                            23.40
             Between  df                                                2
             Between  P                                               ***
             Btwn(F)  P                                              N.S.

                                         Highest age in RR
                              <65     65-74     75-84 85+/unlim   unknown     Total


                       N        2         3         2        15                  22
                      NS        1         3         2        11                  17


                      Wt     1.90      4.30     19.91    260.78              286.89
                 Het Chi     6.14      1.38     10.71    196.22              242.28
                 Het  df        1         2         1        14                  21
                 Het  P         *      N.S.        **       ***                 ***
               Fixed  RR     3.28      9.95      6.69      2.37                2.61
                     RRl     0.79      3.87      4.31      2.10                2.33
                     RRu    13.58     25.61     10.38      2.68                2.93
                      P      N.S.       +++       +++       +++                 +++
              Random  RR     3.17      9.95      3.47      4.99                4.87
                     RRl     0.09      3.87      0.46      2.60                2.83
                     RRu   107.62     25.61     26.07      9.57                8.41
                      P      N.S.       +++      N.S.       +++                 +++
             Between Chi                                                      27.83
             Between  df                                                          3
             Between  P                                                         ***
             Btwn(F)  P                                                        N.S.
  ________________________________________________________________________________________________________________________
                                            International Evidence on Smoking and COPD, Phase 3, Analysis run on 27-SEP-10

                                                   Table 3 - B - 1 - 3

           IESCOPD - Meta-analysis of current smoking, any product (or cigarettes if all product not available)
                                                      Any Emphysema
                                                      Most-adjusted
                           Study weakness
                              Yes        No     Total


                       N        2        20        22
                      NS        2        15        17


                      Wt     5.90    280.99    286.89
                 Het Chi     9.45    231.62    242.28
                 Het  df        1        19        21
                 Het  P        **       ***       ***
               Fixed  RR     4.09      2.59      2.61
                     RRl     1.82      2.30      2.33
                     RRu     9.16      2.91      2.93
                      P       +++       +++       +++
              Random  RR     4.09      4.97      4.87
                     RRl     0.34      2.79      2.83
                     RRu    48.84      8.83      8.41
                      P      N.S.       +++       +++
             Between Chi                         1.21
             Between  df                            1
             Between  P                          N.S.
             Btwn(F)  P                          N.S.

                        Emp subtype
                             mort     other     Total


                       N        5        17        22
                      NS        4        13        17


                      Wt    49.60    237.29    286.89
                 Het Chi    26.33    207.65    242.28
                 Het  df        4        16        21
                 Het  P       ***       ***       ***
               Fixed  RR     3.79      2.42      2.61
                     RRl     2.87      2.13      2.33
                     RRu     5.00      2.74      2.93
                      P       +++       +++       +++
              Random  RR     3.42      5.56      4.87
                     RRl     1.60      2.65      2.83
                     RRu     7.35     11.68      8.41
                      P        ++       +++       +++
             Between Chi                         8.31
             Between  df                            1
             Between  P                            **
             Btwn(F)  P                          N.S.

                        Asthma analysis type (Emphysema)
                        inc-irres  excl-all     Total


                       N       19         3        22
                      NS       14         3        17


                      Wt   280.56      6.33    286.89
                 Het Chi   231.60      9.47    242.28
                 Het  df       18         2        21
                 Het  P       ***        **       ***
               Fixed  RR     2.59      4.03      2.61
                     RRl     2.30      1.85      2.33
                     RRu     2.91      8.77      2.93
                      P       +++       +++       +++
              Random  RR     5.02      3.90      4.87
                     RRl     2.80      0.57      2.83
                     RRu     9.00     26.55      8.41
                      P       +++      N.S.       +++
             Between Chi                         1.21
             Between  df                            1
             Between  P                          N.S.
             Btwn(F)  P                          N.S.
  ________________________________________________________________________________________________________________________
                                            International Evidence on Smoking and COPD, Phase 3, Analysis run on 27-SEP-10

                                                   Table 3 - B - 1 - 3

           IESCOPD - Meta-analysis of current smoking, any product (or cigarettes if all product not available)
                                                      Any Emphysema
                                                      Most-adjusted
                                Number of emphysema cases
                             1-50    51-100   101-200      201+     Total


                       N        8         6         1         7        22
                      NS        7         4         1         5        17


                      Wt    17.67     18.57      2.95    247.70    286.89
                 Het Chi     6.09      9.87      0.00    209.14    242.28
                 Het  df        7         5         0         6        21
                 Het  P      N.S.       (*)      N.S.       ***       ***
               Fixed  RR     2.09      4.83     14.50      2.48      2.61
                     RRl     1.31      3.06      4.63      2.19      2.33
                     RRu     3.34      7.61     45.41      2.81      2.93
                      P        ++       +++       +++       +++       +++
              Random  RR     2.09      5.14     14.50      6.62      4.87
                     RRl     1.31      2.53      4.63      2.44      2.83
                     RRu     3.34     10.45     45.41     18.00      8.41
                      P        ++       +++       +++       +++       +++
             Between Chi                                            17.18
             Between  df                                                3
             Between  P                                               ***
             Btwn(F)  P                                              N.S.

                            Analysis type
                         prevlnce     onset     Total


                       N       14         8        22
                      NS       10         7        17


                      Wt   225.99     60.90    286.89
                 Het Chi   203.76     30.41    242.28
                 Het  df       13         7        21
                 Het  P       ***       ***       ***
               Fixed  RR     2.39      3.61      2.61
                     RRl     2.10      2.81      2.33
                     RRu     2.73      4.64      2.93
                      P       +++       +++       +++
              Random  RR     6.60      3.12      4.87
                     RRl     2.74      1.74      2.83
                     RRu    15.92      5.60      8.41
                      P       +++       +++       +++
             Between Chi                         8.10
             Between  df                            1
             Between  P                            **
             Btwn(F)  P                          N.S.

                             Smoking product
                              any      cigs  cigsonly     Total


                       N        5        16         1        22
                      NS        4        12         1        17


                      Wt   197.26     86.84      2.79    286.89
                 Het Chi    49.03    155.30      0.00    242.28
                 Het  df        4        15         0        21
                 Het  P       ***       ***      N.S.       ***
               Fixed  RR     2.05      4.37      7.72      2.61
                     RRl     1.78      3.54      2.39      2.33
                     RRu     2.36      5.40     24.94      2.93
                      P       +++       +++       +++       +++
              Random  RR     3.84      5.11      7.72      4.87
                     RRl     1.20      2.45      2.39      2.83
                     RRu    12.22     10.65     24.94      8.41
                      P         +       +++       +++       +++
             Between Chi                                  37.95
             Between  df                                      2
             Between  P                                     ***
             Btwn(F)  P                                    N.S.
  ________________________________________________________________________________________________________________________
                                            International Evidence on Smoking and COPD, Phase 3, Analysis run on 27-SEP-10

                                                   Table 3 - B - 1 - 3

           IESCOPD - Meta-analysis of current smoking, any product (or cigarettes if all product not available)
                                                      Any Emphysema
                                                      Most-adjusted
                                     Unexposed group
                          nev any   nev cig  nev+ any  nev+ cig     Total


                       N        7        15                            22
                      NS        6        11                            17


                      Wt   205.52     81.37                        286.89
                 Het Chi   212.44     25.37                        242.28
                 Het  df        6        14                            21
                 Het  P       ***         *                           ***
               Fixed  RR     2.41      3.18                          2.61
                     RRl     2.11      2.56                          2.33
                     RRu     2.77      3.96                          2.93
                      P       +++       +++                           +++
              Random  RR     8.93      3.30                          4.87
                     RRl     1.83      2.39                          2.83
                     RRu    43.50      4.56                          8.41
                      P        ++       +++                           +++
             Between Chi                                             4.47
             Between  df                                                1
             Between  P                                                 *
             Btwn(F)  P                                              N.S.

                        Unexposed group (combining nev+ with main levels)
                          nev any   nev cig     Total


                       N        7        15        22
                      NS        6        11        17


                      Wt   205.52     81.37    286.89
                 Het Chi   212.44     25.37    242.28
                 Het  df        6        14        21
                 Het  P       ***         *       ***
               Fixed  RR     2.41      3.18      2.61
                     RRl     2.11      2.56      2.33
                     RRu     2.77      3.96      2.93
                      P       +++       +++       +++
              Random  RR     8.93      3.30      4.87
                     RRl     1.83      2.39      2.83
                     RRu    43.50      4.56      8.41
                      P        ++       +++       +++
             Between Chi                         4.47
             Between  df                            1
             Between  P                             *
             Btwn(F)  P                          N.S.

                        Smoking results reported in study (Emphysema)
                             Ever   Current      Both     Total


                       N                  5        17        22
                      NS                  4        13        17


                      Wt              35.76    251.13    286.89
                 Het Chi             138.56     86.81    242.28
                 Het  df                  4        16        21
                 Het  P                 ***       ***       ***
               Fixed  RR               4.97      2.38      2.61
                     RRl               3.58      2.11      2.33
                     RRu               6.90      2.70      2.93
                      P                 +++       +++       +++
              Random  RR               6.64      4.27      4.87
                     RRl               0.79      2.69      2.83
                     RRu              55.47      6.78      8.41
                      P                 (+)       +++       +++
             Between Chi                                  16.91
             Between  df                                      1
             Between  P                                     ***
             Btwn(F)  P                                    N.S.
  ________________________________________________________________________________________________________________________
                                            International Evidence on Smoking and COPD, Phase 3, Analysis run on 27-SEP-10

                                                   Table 3 - B - 1 - 3

           IESCOPD - Meta-analysis of current smoking, any product (or cigarettes if all product not available)
                                                      Any Emphysema
                                                      Most-adjusted
                        Number of adjustment variables
                                0         1        2+     Total


                       N        7        11         4        22
                      NS        5         8         4        17


                      Wt    28.50     64.68    193.70    286.89
                 Het Chi    11.73    147.53      7.52    242.28
                 Het  df        6        10         3        21
                 Het  P       (*)       ***       (*)       ***
               Fixed  RR     3.50      6.33      1.86      2.61
                     RRl     2.43      4.96      1.62      2.33
                     RRu     5.06      8.08      2.14      2.93
                      P       +++       +++       +++       +++
              Random  RR     3.15      8.12      2.54      4.87
                     RRl     1.66      2.89      1.54      2.83
                     RRu     5.99     22.86      4.20      8.41
                      P       +++       +++       +++       +++
             Between Chi                                  75.50
             Between  df                                      2
             Between  P                                     ***
             Btwn(F)  P                                       *

                        RR adjusted for sex (combined sex RR only)
                              Yes        No     Total


                       N        4                   4
                      NS        4                   4


                      Wt   193.70              193.70
                 Het Chi     7.52                7.52
                 Het  df        3                   3
                 Het  P       (*)                 (*)
               Fixed  RR     1.86                1.86
                     RRl     1.62                1.62
                     RRu     2.14                2.14
                      P       +++                 +++
              Random  RR     2.54                2.54
                     RRl     1.54                1.54
                     RRu     4.20                4.20
                      P       +++                 +++
             Between Chi
             Between  df
             Between  P                          N.S.
             Btwn(F)  P                          N.S.

                        RR adjusted for age


                       N       13         9        22
                      NS       11         6        17


                      Wt   257.68     29.20    286.89
                 Het Chi   226.89     12.19    242.28
                 Het  df       12         8        21
                 Het  P       ***      N.S.       ***
               Fixed  RR     2.52      3.57      2.61
                     RRl     2.23      2.49      2.33
                     RRu     2.85      5.14      2.93
                      P       +++       +++       +++
              Random  RR     5.77      3.35      4.87
                     RRl     2.77      1.90      2.83
                     RRu    12.02      5.91      8.41
                      P       +++       +++       +++
             Between Chi                         3.20
             Between  df                            1
             Between  P                           (*)
             Btwn(F)  P                          N.S.

  ________________________________________________________________________________________________________________________
                                            International Evidence on Smoking and COPD, Phase 3, Analysis run on 27-SEP-10

                                                   Table 3 - B - 1 - 3

           IESCOPD - Meta-analysis of current smoking, any product (or cigarettes if all product not available)
                                                      Any Emphysema
                                                      Most-adjusted
                        RR adjusted for factor other than sex, age
                              Yes        No     Total


                       N        6        16        22
                      NS        5        12        17


                      Wt   194.40     92.48    286.89
                 Het Chi     8.97    166.06    242.28
                 Het  df        5        15        21
                 Het  P      N.S.       ***       ***
               Fixed  RR     1.87      5.27      2.61
                     RRl     1.62      4.30      2.33
                     RRu     2.15      6.46      2.93
                      P       +++       +++       +++
              Random  RR     2.62      5.57      4.87
                     RRl     1.65      2.67      2.83
                     RRu     4.17     11.62      8.41
                      P       +++       +++       +++
             Between Chi                        67.25
             Between  df                            1
             Between  P                           ***
             Btwn(F)  P                             *

                        Derivation of RR/CI
                         Orig/2x2     Other     Total


                       N        6        16        22
                      NS        5        12        17


                      Wt    40.29    246.60    286.89
                 Het Chi     8.52    231.13    242.28
                 Het  df        5        15        21
                 Het  P      N.S.       ***       ***
               Fixed  RR     3.31      2.51      2.61
                     RRl     2.43      2.22      2.33
                     RRu     4.51      2.85      2.93
                      P       +++       +++       +++
              Random  RR     3.12      6.02      4.87
                     RRl     2.04      2.81      2.83
                     RRu     4.79     12.89      8.41
                      P       +++       +++       +++
             Between Chi                         2.64
             Between  df                            1
             Between  P                          N.S.
             Btwn(F)  P                          N.S.


  ________________________________________________________________________________________________________________________
                                            International Evidence on Smoking and COPD, Phase 3, Analysis run on 27-SEP-10

                                                   Table 3 - B - 1 - 4

           IESCOPD - Meta-analysis of current smoking, any product (or cigarettes if all product not available)
                                                      Any Emphysema
                                                      Least-adjusted


     REF|NRR|X|SEX|AGEL|AGEH|     REGION|BEGYR|PUBYR|STTYP|ONSET|      DISEAS|ADJ|SMOKSTA|   PRODUCT|    UNEXP|

  AUERBA   1 x   m   15   99      Am:USA  1963  1972    CS  Prev  Emp:viscomp   0 Current       Cigs   Nev any
    BEST  18     m   30   97   Am:Canada  1955  1967    Pr   Inc     Emp:mort   1 Current  Cigs only   Nev any
  DONTA2  11     m   25   84 Eu:SE/Balkn  1960  1984    Pr   Inc Emp:diagnosd   0 Current       Cigs  Nev cigs
  ENRIGH   7     m   65   99      Am:USA  1989  1994    CS  Prev Emp:diagnosd   0 Current       Cigs  Nev cigs
  ENRIGH  10     f   65   99      Am:USA  1989  1994    CS  Prev Emp:diagnosd   0 Current       Cigs  Nev cigs
  HARDIE  13     m   70   99    Eu:Scand  1998  2005    CS  Prev Emp:diagnosd   1 Current       Cigs  Nev cigs
  HARDIE  16     f   70   99    Eu:Scand  1998  2005    CS  Prev Emp:diagnosd   1 Current       Cigs  Nev cigs
  HIRAYA   1     m   40   99   Asia:FarE  1965  1982    Pr   Inc     Emp:mort   1 Current       Cigs  Nev cigs
  HIRAYA   2     f   40   99   Asia:FarE  1965  1982    Pr   Inc     Emp:mort   1 Current       Cigs  Nev cigs
      HO   9     b   70   99   Asia:FarE  1991  1999    CS  Prev Emp:self-rep   3 Current       Cigs  Nev cigs
  HUHTI1  67     m   40   64    Eu:Scand  1961  1965    CS  Prev    Emp:other   0 Current        Any   Nev any
  HUHTI1 142     f   40   64    Eu:Scand  1961  1965    CS  Prev    Emp:other   0 Current        Any   Nev any
   KAHN2  46     m   31   84      Am:USA  1954  1966    Pr   Inc     Emp:mort   1 Current        Any   Nev any
  LAVECC  22 x   m   15   99     Eu:West  1983  1988    CS  Prev Emp:self-rep   0 Current        Any   Nev any
  LAVECC  25 x   f   15   99     Eu:West  1983  1988    CS  Prev Emp:self-rep   0 Current        Any   Nev any
  LEBOWI  31 x   b   15   96      Am:USA  1972  1977    CS  Prev Emp:diagnosd   2 Current       Cigs  Nev cigs
  MILLER   7     m   15   99      Am:USA  1978  1988    CS  Prev Emp:diagnosd   1 Current       Cigs  Nev cigs
  MILLER  10     f   15   99      Am:USA  1978  1988    CS  Prev Emp:diagnosd   1 Current       Cigs  Nev cigs
   OMORI   1 x   m   40   69   Asia:FarE     *  2006    CS  Prev  Emp:viscomp   0 Current       Cigs  Nev cigs
   SILVA   7     b   20   99      Am:USA  1972  2004    Pr   Inc    Emp:other   6 Current       Cigs  Nev cigs
  VIKGRE   1     m   61   68    Eu:Scand  1994  2004    Pr   Inc  Emp:viscomp   0 Current       Cigs  Nev cigs
   WEISS  14     m   50   69      Am:USA  1961  1963    CS  Prev  Emp:viscomp   0 Current        Any   Nev any
     WEN  18     m   35   99   Asia:FarE  1982  2004    Pr   Inc     Emp:mort   1 Current       Cigs  Nev cigs


  ________________________________________________________________________________________________________________________
                                            International Evidence on Smoking and COPD, Phase 3, Analysis run on 27-SEP-10

                                                   Table 3 - B - 1 - 5

           IESCOPD - Meta-analysis of current smoking, any product (or cigarettes if all product not available)
                                                      Any Emphysema
                                                      Least-adjusted


                        Number Exposed  Non-exposed
 REF    NRR SEX ADJ     Case    Cont    Case    Cont      RR        95.00%CI
 AUERBA 1   m   0        816      23      20     156    276.73 (148.38- 516.09)
*BEST   18  m   1         40       -       3       -      7.72 (  2.39-  24.95)
*DONTA2 11  m   0         10     275       4     127      1.15 (  0.37-   3.61)
 ENRIGH 7   m   0         32     199      22     694      5.07 (  2.88-   8.93)
 ENRIGH 10  f   0         18     364      26    1617      3.08 (  1.67-   5.67)
 Subtotal ENRIGH                                          4.03 (  2.66-   6.10)
 HARDIE 13  m   1         11       -       2       -     15.04 (  3.40-  66.58)
 HARDIE 16  f   1          5       -      11       -      3.59 (  1.38-   9.29)
 Subtotal HARDIE                                          5.45 (  2.44-  12.16)
*HIRAYA 1   m   1        156       -      17       -      2.22 (  1.34-   3.67)
*HIRAYA 2   f   1         14       -      42       -      2.72 (  1.48-   5.00)
 Subtotal HIRAYA                                          2.41 (  1.64-   3.55)
 HO     9   b   3          -       -       -       -      2.30 (  0.94-   5.62)
 HUHTI1 67  m   0         53     337       1     121     19.03 (  2.60- 139.11)
 HUHTI1 142 f   0          1      83      16     693      0.52 (  0.07-   3.99)
 Subtotal HUHTI1                                          3.28 (  0.79-  13.58)
*KAHN2  46  m   1        284       -      18       -      9.09 (  5.65-  14.64)
 LAVECC 22  m   0        375   15285     183   14129      1.89 (  1.59-   2.26)
 LAVECC 25  f   0         62    6654     412   29448      0.67 (  0.51-   0.87)
 Subtotal LAVECC                                          1.38 (  1.19-   1.60)
 LEBOWI 31  b   2         14       -       5       -      3.84 (  1.36-  10.84)
 MILLER 7   m   1          4       -       0       -      7.21 (  0.39- 133.19)
 MILLER 10  f   1          2       -       1       -      9.07 (  0.18- 446.54)
 Subtotal MILLER                                          7.83 (  0.76-  81.05)
 OMORI  1   m   0        116     264       3      97     14.21 (  4.41-  45.75)
*SILVA  7   b   6          -       -       -       -      4.10 (  2.03-   8.18)
*VIKGRE 1   m   0          2      40       0      26      3.27~(  0.16-  65.49)
 WEISS  14  m   0         23     160       1      35      5.03 (  0.66-  38.51)
*WEN    18  m   1         12       -       -       -      1.12 (  0.44-   2.88)
Partial Totals          2050   23684     787   47143
*prospective study                                        ~ With 0.5 adjustment for zero


 REF    NRR SEX ADJ             Ys       Ws       Qs       Ps
 AUERBA 1   m   0              5.62     9.89   222.99       0.00
*BEST   18  m   1              2.04     2.79     3.82       0.00
*DONTA2 11  m   0              0.14     2.95     1.58       0.80
 ENRIGH 7   m   0              1.62    12.02     6.75       0.00
 ENRIGH 10  f   0              1.12    10.27     0.64       0.00
 Subtotal ENRIGH               1.39    22.29     7.38
 HARDIE 13  m   1              2.71     1.74     5.85       0.00
 HARDIE 16  f   1              1.28     4.23     0.69       0.01
 Subtotal HARDIE               1.70     5.96     6.54
*HIRAYA 1   m   1              0.80    15.14     0.09       0.00
*HIRAYA 2   f   1              1.00    10.37     0.16       0.00
 Subtotal HIRAYA               0.88    25.51     0.25
 HO     9   b   3              0.83     4.81     0.01       0.07
 HUHTI1 67  m   0              2.95     0.97     4.17       0.00
 HUHTI1 142 f   0             -0.65     0.93     2.16       0.53
 Subtotal HUHTI1               1.19     1.90     6.33
*KAHN2  46  m   1              2.21    16.95    30.10       0.00
 LAVECC 22  m   0              0.64   120.96     6.73       0.00
 LAVECC 25  f   0             -0.41    53.36    87.58       0.00
 Subtotal LAVECC               0.32   174.32    94.31
 LEBOWI 31  b   2              1.35     3.57     0.79       0.01
 MILLER 7   m   1              1.98     0.45     0.55       0.18
 MILLER 10  f   1              2.20     0.25     0.45       0.27
 Subtotal MILLER               2.06     0.70     0.99
 OMORI  1   m   0              2.65     2.81     8.89       0.00
*SILVA  7   b   6              1.41     7.91     2.28       0.00
*VIKGRE 1   m   0              1.19     0.43     0.04       0.44
 WEISS  14  m   0              1.62     0.93     0.51       0.12
*WEN    18  m   1              0.11     4.35     2.52       0.81


  ________________________________________________________________________________________________________________________
                                            International Evidence on Smoking and COPD, Phase 3, Analysis run on 27-SEP-10

                                                   Table 3 - B - 1 - 5

           IESCOPD - Meta-analysis of current smoking, any product (or cigarettes if all product not available)
                                                      Any Emphysema
                                                      Least-adjusted


                       N       23
                      NS       17


                      Wt   288.07
                 Het Chi   389.33
                 Het  df       22
                 Het  P       ***
               Fixed  RR     2.40
                     RRl     2.14
                     RRu     2.69
                      P       +++
              Random  RR     4.32
                     RRl     2.40
                     RRu     7.78
                      P       +++
               Asymm  P       (*)


  ________________________________________________________________________________________________________________________
                                            International Evidence on Smoking and COPD, Phase 3, Analysis run on 27-SEP-10

                                                   Table 3 - B - 1 - 6

           IESCOPD - Meta-analysis of current smoking, any product (or cigarettes if all product not available)
                                                      Any Emphysema
                                                      Least-adjusted


                       N       23
                      NS       17


                      Wt   288.07
                 Het Chi   389.33
                 Het  df       22
                 Het  P       ***
               Fixed  RR     2.40
                     RRl     2.14
                     RRu     2.69
                      P       +++
              Random  RR     4.32
                     RRl     2.40
                     RRu     7.78
                      P       +++
               Asymm  P       (*)

                                   Sex
                             both      male    female     Total


                       N        3        14         6        23
                      NS        3        14         6        23


                      Wt    16.28    192.38     79.40    288.07
                 Het Chi     1.06    276.99     40.29    389.33
                 Het  df        2        13         5        22
                 Het  P      N.S.       ***       ***       ***
               Fixed  RR     3.41      3.24      1.07      2.40
                     RRl     2.10      2.82      0.86      2.14
                     RRu     5.54      3.74      1.34      2.69
                      P       +++       +++      N.S.       +++
              Random  RR     3.41      6.55      1.87      4.32
                     RRl     2.10      2.71      0.77      2.40
                     RRu     5.54     15.83      4.56      7.78
                      P       +++       +++      N.S.       +++
             Between Chi                                  70.98
             Between  df                                      2
             Between  P                                     ***
             Btwn(F)  P                                    N.S.

                                        Continent
                            NAmer    Europe      Asia  oth/mult     Total


                       N       10         8         5                  23
                      NS        8         5         4                  17


                      Wt    65.03    185.56     37.47              288.07
                 Het Chi   139.22     61.76     11.61              389.33
                 Het  df        9         7         4                  22
                 Het  P       ***       ***         *                 ***
               Fixed  RR     9.84      1.45      2.50                2.40
                     RRl     7.72      1.26      1.82                2.14
                     RRu    12.55      1.67      3.45                2.69
                      P       +++       +++       +++                 +++
              Random  RR     8.52      2.19      2.70                4.32
                     RRl     2.94      1.09      1.50                2.40
                     RRu    24.72      4.39      4.86                7.78
                      P       +++         +       +++                 +++
             Between Chi                                           176.74
             Between  df                                                2
             Between  P                                               ***
             Btwn(F)  P                                                **


  ________________________________________________________________________________________________________________________
                                            International Evidence on Smoking and COPD, Phase 3, Analysis run on 27-SEP-10

                                                   Table 3 - B - 1 - 6

           IESCOPD - Meta-analysis of current smoking, any product (or cigarettes if all product not available)
                                                      Any Emphysema
                                                      Least-adjusted
                               Study type
                               CC        Pr        CS     Total


                       N                  8        15        23
                      NS                  7        10        17


                      Wt              60.90    227.17    288.07
                 Het Chi              30.41    346.01    389.33
                 Het  df                  7        14        22
                 Het  P                 ***       ***       ***
               Fixed  RR               3.61      2.15      2.40
                     RRl               2.81      1.89      2.14
                     RRu               4.64      2.45      2.69
                      P                 +++       +++       +++
              Random  RR               3.12      5.32      4.32
                     RRl               1.74      2.29      2.40
                     RRu               5.60     12.34      7.78
                      P                 +++       +++       +++
             Between Chi                                  12.90
             Between  df                                      1
             Between  P                                     ***
             Btwn(F)  P                                    N.S.

                        Emp subtype
                             mort     other     Total


                       N        5        18        23
                      NS        4        13        17


                      Wt    49.60    238.47    288.07
                 Het Chi    26.33    350.48    389.33
                 Het  df        4        17        22
                 Het  P       ***       ***       ***
               Fixed  RR     3.79      2.18      2.40
                     RRl     2.87      1.92      2.14
                     RRu     5.00      2.48      2.69
                      P       +++       +++       +++
              Random  RR     3.42      4.68      4.32
                     RRl     1.60      2.23      2.40
                     RRu     7.35      9.85      7.78
                      P        ++       +++       +++
             Between Chi                        12.52
             Between  df                            1
             Between  P                           ***
             Btwn(F)  P                          N.S.

                             Smoking product
                              any      cigs  cigsonly     Total


                       N        6        16         1        23
                      NS        4        12         1        17


                      Wt   194.10     91.18      2.79    288.07
                 Het Chi   103.77    200.89      0.00    389.33
                 Het  df        5        15         0        22
                 Het  P       ***       ***      N.S.       ***
               Fixed  RR     1.65      5.15      7.72      2.40
                     RRl     1.43      4.20      2.39      2.14
                     RRu     1.89      6.33     24.94      2.69
                      P       +++       +++       +++       +++
              Random  RR     2.63      4.97      7.72      4.32
                     RRl     1.04      2.21      2.39      2.40
                     RRu     6.61     11.18     24.94      7.78
                      P         +       +++       +++       +++
             Between Chi                                  84.67
             Between  df                                      2
             Between  P                                     ***
             Btwn(F)  P                                     (*)
  ________________________________________________________________________________________________________________________
                                            International Evidence on Smoking and COPD, Phase 3, Analysis run on 27-SEP-10

                                                   Table 3 - B - 1 - 6

           IESCOPD - Meta-analysis of current smoking, any product (or cigarettes if all product not available)
                                                      Any Emphysema
                                                      Least-adjusted
                                     Unexposed group
                          nev any   nev cig  nev+ any  nev+ cig     Total


                       N        8        15                            23
                      NS        6        11                            17


                      Wt   206.78     81.29                        288.07
                 Het Chi   355.54     24.88                        389.33
                 Het  df        7        14                            22
                 Het  P       ***         *                           ***
               Fixed  RR     2.15      3.17                          2.40
                     RRl     1.87      2.55                          2.14
                     RRu     2.46      3.95                          2.69
                      P       +++       +++                           +++
              Random  RR     5.95      3.29                          4.32
                     RRl     1.69      2.38                          2.40
                     RRu    20.95      4.53                          7.78
                      P        ++       +++                           +++
             Between Chi                                             8.91
             Between  df                                                1
             Between  P                                                **
             Btwn(F)  P                                              N.S.

                        Unexposed group (combining nev+ with main levels)
                          nev any   nev cig     Total


                       N        8        15        23
                      NS        6        11        17


                      Wt   206.78     81.29    288.07
                 Het Chi   355.54     24.88    389.33
                 Het  df        7        14        22
                 Het  P       ***         *       ***
               Fixed  RR     2.15      3.17      2.40
                     RRl     1.87      2.55      2.14
                     RRu     2.46      3.95      2.69
                      P       +++       +++       +++
              Random  RR     5.95      3.29      4.32
                     RRl     1.69      2.38      2.40
                     RRu    20.95      4.53      7.78
                      P        ++       +++       +++
             Between Chi                         8.91
             Between  df                            1
             Between  P                            **
             Btwn(F)  P                          N.S.


  ________________________________________________________________________________________________________________________
                                            International Evidence on Smoking and COPD, Phase 3, Analysis run on 27-SEP-10

                                                   Table 3 - B - 1 - 7

           IESCOPD - Meta-analysis of current smoking, any product (or cigarettes if all product not available)
                                                      Any Emphysema
                                 Excluded studies (and stage at which they were excluded)


1       CLARK COTTON  MEYER REMYJA RUTGER SNYDER SOBRAX     SU TAKEMU  WANG4   WEIR WHICKE ZALACA
2      ALDERS ALESSA  AMIGO ANDER1 ANDER3   BANG  BECK1  BECK2 BEDNAR BJORNS BROGGE  BROWN CERVER CHAPMA  CHEN1  CHEN2
        CHEN3  CHENG CLEMEN COATES  COCCI COLLEG  DEAN1  DEAN2  DEANE DEJONG DEMARC DETORR DICKIN  DOLL1  DOLL2 DONTA1
       DOPICO EHRLIC EKBERG ENSTRO FERRI1 FERRI2 FERRI3  FIDAN FINKLE FLETCH FORAST FOXMAN FUKUCH GEIJER GODTFR GOLDBE
       HAENSZ HARIKK HARRIS HAWTHO  HAYES HEDMAN HIGGI2 HIGGI3 HIGGI4 HIGGI6 HOLLA2 HOLLNA  HOUSE HRUBEC HUCHON HUHTI2
       HUHTI3 ITABAS JACOBS JAENDI JENSEN JINDA2 JOHANN  JOSHI JOUSI1 KACHEL KARAKA KATANC   KATO KHOURY    KIM  KIRAZ
       KLAYTO KOJIMA KOTAN1 KOTAN2 KRZYZA  KUBIK KULLER    LAI   LAM1   LAM2   LAM3 LAMBER  LANGE LANGE2 LANGHA    LEE
         LIAW LINDBE LINDST   LIU1   LIU2 LUNDB1 LUNDB2  MADOR MAGNUS MANFRE MANNI1 MANNI2 MANNI3 MARAN1 MARAN2 MARCUS
       MATHES MELLST MENEZ1 MENEZ2 MENEZ3 MENEZ4 MENEZ5 MENEZ6  MEREN  MILNE MOLLER MONTNE MUELLE NEJJAR NIEPSU NIHLEN
       NILSSO OGILVI OSWAL1 OSWAL2 PANDEY   PEAT PELKON PEREZP   PETO  PRICE   REID RENWIC RICCIO RIMING SARGEA SAWICK
       SCHWAR SHAHAB  SHARP SHIMUR   SHIN SICHLE SOBRAD SPEIZE STERLI STJERN  STROM SUADIC  TAGER TAGER2   TANG   THUN
         TODD TROISI TRUPIN TSUSHI TVERDA URRUTI VESTBO VIEGI1 VIEGI2 VINEIS VOLLM1 VOLLM2 VONHER WAGEN2   WALD WATSON
          WIG WILHEL WILSO1 WOJTYN  WOODS  WOOLF   XIAO     XU YAMAGU   YUAN ZIELI1 ZIELI2 ZIETKO   ZOIA
3      WILSO2
4      ANDER2 GULSVI HAMMO2 HOZAWA   KAHN   NAWA  PRATT  RYDER SUTINE  WANG2


  ________________________________________________________________________________________________________________________
                                            International Evidence on Smoking and COPD, Phase 3, Analysis run on 27-SEP-10

                                                   Table 3 - B - 1 - 8

           IESCOPD - Meta-analysis of current smoking, any product (or cigarettes if all product not available)
                                                      Any Emphysema
                                             Potentially overlapping studies


     REF| REFGP|PRINC|                     OVERLAP|

  ENRIGH ENRIGH     1         ENRIGH/HOZAWA/HARIKK
  DONTA2 JACOBS     2  JACOBS/DONTA1/DONTA2/PELKON
  HUHTI1 HUHTI1     1                HUHTI1/HUHTI2
  HARDIE HARDIE     1         HARDIE/JOHANN/BROGGE
  LEBOWI LEBOWI     1                 LEBOWI/SILVA
   SILVA  SILVA     1                 LEBOWI/SILVA
     WEN    WEN     1                     WEN/LIAW
   KAHN2   KAHN     2                   KAHN/KAHN2


  ________________________________________________________________________________________________________________________
                                            International Evidence on Smoking and COPD, Phase 3, Analysis run on 27-SEP-10

                                                    Table 3 - B - 2 -

           IESCOPD - Meta-analysis of current smoking, cigarettes (or all products if cigarettes not available)
                                                      Any Emphysema


This analysis is restricted to results for:
1) Eligible study on database
2) Outcome Emphysema
3) Non-dose-response data
4) Current smoking
5) Results complete enough for use in meta-analysis

Within each study, results are then selected (in the following order of preference, within each sex) for:
6) UNEXP   : never cigarettes, never any, other
7) PROD    : cigarettes, cigarettes only, any product
8) For overlapping studies: principal rather than subsidiary studies
and then for single sex results (m, f) in preference to results for both sexes combined (b).

Results adjusted for the most potential confounders are then chosen in Sections -1 to -3
(and those which actually differ from the adjusted results in Table 3 - B - 1 - 1 are marked 'x' in Section -1)
and results adjusted for the least confounders in Sections -4 to -6. (Those least adjusted results which
actually differ from the most adjusted are marked 'x' in column X in Section -4)

Section -7 shows excluded studies, together with the stage (as above) at which no qualifying
results were found.

Section -8 lists the potentially overlapping studies which have been included (1=principal, 2=subsidiary),
and any results which would have been included in preference except that they had data not complete enough
for use in meta-analysis. It also lists their significance (yes/no), if known.


  ________________________________________________________________________________________________________________________
                                            International Evidence on Smoking and COPD, Phase 3, Analysis run on 27-SEP-10

                                                   Table 3 - B - 2 - 1

           IESCOPD - Meta-analysis of current smoking, cigarettes (or all products if cigarettes not available)
                                                      Any Emphysema
                                                      Most-adjusted


     REF|NRR|Cmp3B1|SEX|AGEL|AGEH|     REGION|BEGYR|PUBYR|STTYP|ONSET|      DISEAS|ADJ|SMOKSTA|   PRODUCT|    UNEXP|

  AUERBA   2          m   15   99      Am:USA  1963  1972    CS  Prev  Emp:viscomp   1 Current       Cigs   Nev any
    BEST  18          m   30   97   Am:Canada  1955  1967    Pr   Inc     Emp:mort   1 Current  Cigs only   Nev any
  DONTA2  11          m   25   84 Eu:SE/Balkn  1960  1984    Pr   Inc Emp:diagnosd   0 Current       Cigs  Nev cigs
  ENRIGH   7          m   65   99      Am:USA  1989  1994    CS  Prev Emp:diagnosd   0 Current       Cigs  Nev cigs
  ENRIGH  10          f   65   99      Am:USA  1989  1994    CS  Prev Emp:diagnosd   0 Current       Cigs  Nev cigs
  HARDIE  13          m   70   99    Eu:Scand  1998  2005    CS  Prev Emp:diagnosd   1 Current       Cigs  Nev cigs
  HARDIE  16          f   70   99    Eu:Scand  1998  2005    CS  Prev Emp:diagnosd   1 Current       Cigs  Nev cigs
  HIRAYA   1          m   40   99   Asia:FarE  1965  1982    Pr   Inc     Emp:mort   1 Current       Cigs  Nev cigs
  HIRAYA   2          f   40   99   Asia:FarE  1965  1982    Pr   Inc     Emp:mort   1 Current       Cigs  Nev cigs
      HO   9          b   70   99   Asia:FarE  1991  1999    CS  Prev Emp:self-rep   3 Current       Cigs  Nev cigs
  HUHTI1  70      x   m   40   64    Eu:Scand  1961  1965    CS  Prev    Emp:other   0 Current       Cigs   Nev any
  HUHTI1 154      x   f   40   64    Eu:Scand  1961  1965    CS  Prev    Emp:other   0 Current       Cigs  Nev cigs
   KAHN2  47      x   m   31   84      Am:USA  1954  1966    Pr   Inc     Emp:mort   1 Current       Cigs   Nev any
  LAVECC  33      x   b   15   99     Eu:West  1983  1988    CS  Prev Emp:self-rep   6 Current       Cigs   Nev any
  LEBOWI  34          b   15   96      Am:USA  1972  1977    CS  Prev Emp:diagnosd   3 Current       Cigs  Nev cigs
  MILLER   7          m   15   99      Am:USA  1978  1988    CS  Prev Emp:diagnosd   1 Current       Cigs  Nev cigs
  MILLER  10          f   15   99      Am:USA  1978  1988    CS  Prev Emp:diagnosd   1 Current       Cigs  Nev cigs
   OMORI   4          m   40   69   Asia:FarE     *  2006    CS  Prev  Emp:viscomp   1 Current       Cigs  Nev cigs
   SILVA   7          b   20   99      Am:USA  1972  2004    Pr   Inc    Emp:other   6 Current       Cigs  Nev cigs
  VIKGRE   1          m   61   68    Eu:Scand  1994  2004    Pr   Inc  Emp:viscomp   0 Current       Cigs  Nev cigs
   WEISS  17      x   m   50   69      Am:USA  1961  1963    CS  Prev  Emp:viscomp   0 Current       Cigs   Nev any
     WEN  18          m   35   99   Asia:FarE  1982  2004    Pr   Inc     Emp:mort   1 Current       Cigs  Nev cigs


  ________________________________________________________________________________________________________________________
                                            International Evidence on Smoking and COPD, Phase 3, Analysis run on 27-SEP-10

                                                   Table 3 - B - 2 - 2

           IESCOPD - Meta-analysis of current smoking, cigarettes (or all products if cigarettes not available)
                                                      Any Emphysema
                                                      Most-adjusted


                        Number Exposed  Non-exposed
 REF    NRR SEX ADJ     Case    Cont    Case    Cont      RR        95.00%CI
 AUERBA 2   m   1        816       -      20       -    489.54 (211.74-1131.81)
*BEST   18  m   1         40       -       3       -      7.72 (  2.39-  24.95)
*DONTA2 11  m   0         10     275       4     127      1.15 (  0.37-   3.61)
 ENRIGH 7   m   0         32     199      22     694      5.07 (  2.88-   8.93)
 ENRIGH 10  f   0         18     364      26    1617      3.08 (  1.67-   5.67)
 Subtotal ENRIGH                                          4.03 (  2.66-   6.10)
 HARDIE 13  m   1         11       -       2       -     15.04 (  3.40-  66.58)
 HARDIE 16  f   1          5       -      11       -      3.59 (  1.38-   9.29)
 Subtotal HARDIE                                          5.45 (  2.44-  12.16)
*HIRAYA 1   m   1        156       -      17       -      2.22 (  1.34-   3.67)
*HIRAYA 2   f   1         14       -      42       -      2.72 (  1.48-   5.00)
 Subtotal HIRAYA                                          2.41 (  1.64-   3.55)
 HO     9   b   3          -       -       -       -      2.30 (  0.94-   5.62)
 HUHTI1 70  m   0         53     331       1     121     19.37 (  2.65- 141.64)
 HUHTI1 154 f   0          1      83      16     693      0.52 (  0.07-   3.99)
 Subtotal HUHTI1                                          3.31 (  0.80-  13.71)
*KAHN2  47  m   1        272       -      18       -     12.18 (  7.56-  19.62)
 LAVECC 33  b   6        432       -     595       -      1.81 (  1.57-   2.10)
 LEBOWI 34  b   3         14       -       5       -      3.82 (  1.34-  10.88)
 MILLER 7   m   1          4       -       0       -      7.21 (  0.39- 133.19)
 MILLER 10  f   1          2       -       1       -      9.07 (  0.18- 446.54)
 Subtotal MILLER                                          7.83 (  0.76-  81.05)
 OMORI  4   m   1        116       -       3       -     14.50 (  4.63-  45.40)
*SILVA  7   b   6          -       -       -       -      4.10 (  2.03-   8.18)
*VIKGRE 1   m   0          2      40       0      26      3.27~(  0.16-  65.49)
 WEISS  17  m   0         22     124       1      35      6.21 (  0.81-  47.70)
*WEN    18  m   1         12       -       -       -      1.12 (  0.44-   2.88)
Partial Totals          2032    1416     787    3313
*prospective study                                        ~ With 0.5 adjustment for zero


 REF    NRR SEX ADJ             Ys       Ws       Qs       Ps
 AUERBA 2   m   1              6.19     5.47   148.13       0.00
*BEST   18  m   1              2.04     2.79     3.11       0.00
*DONTA2 11  m   0              0.14     2.95     2.11       0.80
 ENRIGH 7   m   0              1.62    12.02     4.85       0.00
 ENRIGH 10  f   0              1.12    10.27     0.19       0.00
 Subtotal ENRIGH               1.39    22.29     5.03
 HARDIE 13  m   1              2.71     1.74     5.15       0.00
 HARDIE 16  f   1              1.28     4.23     0.35       0.01
 Subtotal HARDIE               1.70     5.96     5.50
*HIRAYA 1   m   1              0.80    15.14     0.55       0.00
*HIRAYA 2   f   1              1.00    10.37     0.00       0.00
 Subtotal HIRAYA               0.88    25.51     0.56
 HO     9   b   3              0.83     4.81     0.12       0.07
 HUHTI1 70  m   0              2.96     0.97     3.79       0.00
 HUHTI1 154 f   0             -0.65     0.93     2.50       0.53
 Subtotal HUHTI1               1.20     1.90     6.28
*KAHN2  47  m   1              2.50    16.89    38.56       0.00
 LAVECC 33  b   6              0.59   181.63    28.43       0.00
 LEBOWI 34  b   3              1.34     3.50     0.43       0.01
 MILLER 7   m   1              1.98     0.45     0.44       0.18
 MILLER 10  f   1              2.20     0.25     0.37       0.27
 Subtotal MILLER               2.06     0.70     0.81
 OMORI  4   m   1              2.67     2.95     8.37       0.00
*SILVA  7   b   6              1.41     7.91     1.41       0.00
*VIKGRE 1   m   0              1.19     0.43     0.02       0.44
 WEISS  17  m   0              1.83     0.92     0.65       0.08
*WEN    18  m   1              0.11     4.35     3.34       0.81


  ________________________________________________________________________________________________________________________
                                            International Evidence on Smoking and COPD, Phase 3, Analysis run on 27-SEP-10

                                                   Table 3 - B - 2 - 2

           IESCOPD - Meta-analysis of current smoking, cigarettes (or all products if cigarettes not available)
                                                      Any Emphysema
                                                      Most-adjusted


                       N       22
                      NS       17


                      Wt   290.98
                 Het Chi   252.86
                 Het  df       21
                 Het  P       ***
               Fixed  RR     2.69
                     RRl     2.40
                     RRu     3.02
                      P       +++
              Random  RR     5.00
                     RRl     2.87
                     RRu     8.72
                      P       +++
               Asymm  P         *


  ________________________________________________________________________________________________________________________
                                            International Evidence on Smoking and COPD, Phase 3, Analysis run on 27-SEP-10

                                                   Table 3 - B - 2 - 3

           IESCOPD - Meta-analysis of current smoking, cigarettes (or all products if cigarettes not available)
                                                      Any Emphysema
                                                      Most-adjusted


                       N       22
                      NS       17


                      Wt   290.98
                 Het Chi   252.86
                 Het  df       21
                 Het  P       ***
               Fixed  RR     2.69
                     RRl     2.40
                     RRu     3.02
                      P       +++
              Random  RR     5.00
                     RRl     2.87
                     RRu     8.72
                      P       +++
               Asymm  P         *

                                   Sex
                             both      male    female     Total


                       N        4        13         5        22
                      NS        4        13         5        22


                      Wt   197.85     67.08     26.04    290.98
                 Het Chi     6.99    153.07      3.32    252.86
                 Het  df        3        12         4        21
                 Het  P       (*)       ***      N.S.       ***
               Fixed  RR     1.91      7.25      2.85      2.69
                     RRl     1.66      5.71      1.94      2.40
                     RRu     2.19      9.21      4.18      3.02
                      P       +++       +++       +++       +++
              Random  RR     2.55      7.99      2.85      5.00
                     RRl     1.58      3.09      1.94      2.87
                     RRu     4.12     20.69      4.18      8.72
                      P       +++       +++       +++       +++
             Between Chi                                  89.48
             Between  df                                      2
             Between  P                                     ***
             Btwn(F)  P                                       *

                                        Continent
                            NAmer    Europe      Asia  oth/mult     Total


                       N       10         7         5                  22
                      NS        8         5         4                  17


                      Wt    60.49    192.87     37.61              290.98
                 Het Chi   112.34     17.18     12.23              252.86
                 Het  df        9         6         4                  21
                 Het  P       ***        **         *                 ***
               Fixed  RR     8.85      1.87      2.52                2.69
                     RRl     6.88      1.63      1.83                2.40
                     RRu    11.38      2.16      3.47                3.02
                      P       +++       +++       +++                 +++
              Random  RR     9.48      2.90      2.74                5.00
                     RRl     3.49      1.38      1.50                2.87
                     RRu    25.79      6.10      4.99                8.72
                      P       +++        ++        ++                 +++
             Between Chi                                           111.11
             Between  df                                                2
             Between  P                                               ***
             Btwn(F)  P                                                **


  ________________________________________________________________________________________________________________________
                                            International Evidence on Smoking and COPD, Phase 3, Analysis run on 27-SEP-10

                                                   Table 3 - B - 2 - 3

           IESCOPD - Meta-analysis of current smoking, cigarettes (or all products if cigarettes not available)
                                                      Any Emphysema
                                                      Most-adjusted
                               Study type
                               CC        Pr        CS     Total


                       N                  8        14        22
                      NS                  7        10        17


                      Wt              60.84    230.14    290.98
                 Het Chi              40.55    201.50    252.86
                 Het  df                  7        13        21
                 Het  P                 ***       ***       ***
               Fixed  RR               3.91      2.43      2.69
                     RRl               3.04      2.14      2.40
                     RRu               5.03      2.77      3.02
                      P                 +++       +++       +++
              Random  RR               3.24      6.71      5.00
                     RRl               1.66      2.80      2.87
                     RRu               6.34     16.10      8.72
                      P                 +++       +++       +++
             Between Chi                                  10.81
             Between  df                                      1
             Between  P                                      **
             Btwn(F)  P                                    N.S.

                        Emp subtype
                             mort     other     Total


                       N        5        17        22
                      NS        4        13        17


                      Wt    49.55    241.43    290.98
                 Het Chi    35.90    205.32    252.86
                 Het  df        4        16        21
                 Het  P       ***       ***       ***
               Fixed  RR     4.18      2.46      2.69
                     RRl     3.17      2.16      2.40
                     RRu     5.52      2.79      3.02
                      P       +++       +++       +++
              Random  RR     3.65      5.63      5.00
                     RRl     1.50      2.69      2.87
                     RRu     8.86     11.78      8.72
                      P        ++       +++       +++
             Between Chi                        11.65
             Between  df                            1
             Between  P                           ***
             Btwn(F)  P                          N.S.

                             Smoking product
                              any      cigs  cigsonly     Total


                       N                 21         1        22
                      NS                 16         1        17


                      Wt             288.18      2.79    290.98
                 Het Chi             249.73      0.00    252.86
                 Het  df                 20         0        21
                 Het  P                 ***      N.S.       ***
               Fixed  RR               2.66      7.72      2.69
                     RRl               2.37      2.39      2.40
                     RRu               2.99     24.94      3.02
                      P                 +++       +++       +++
              Random  RR               4.90      7.72      5.00
                     RRl               2.77      2.39      2.87
                     RRu               8.68     24.94      8.72
                      P                 +++       +++       +++
             Between Chi                                   3.14
             Between  df                                      1
             Between  P                                     (*)
             Btwn(F)  P                                    N.S.
  ________________________________________________________________________________________________________________________
                                            International Evidence on Smoking and COPD, Phase 3, Analysis run on 27-SEP-10

                                                   Table 3 - B - 2 - 3

           IESCOPD - Meta-analysis of current smoking, cigarettes (or all products if cigarettes not available)
                                                      Any Emphysema
                                                      Most-adjusted
                                     Unexposed group
                          nev any   nev cig  nev+ any  nev+ cig     Total


                       N        6        16                            22
                      NS        6        12                            18


                      Wt   208.68     82.30                        290.98
                 Het Chi   221.95     28.37                        252.86
                 Het  df        5        15                            21
                 Het  P       ***         *                           ***
               Fixed  RR     2.54      3.12                          2.69
                     RRl     2.21      2.51                          2.40
                     RRu     2.90      3.87                          3.02
                      P       +++       +++                           +++
              Random  RR    14.77      3.18                          5.00
                     RRl     2.54      2.28                          2.87
                     RRu    85.84      4.43                          8.72
                      P        ++       +++                           +++
             Between Chi                                             2.54
             Between  df                                                1
             Between  P                                              N.S.
             Btwn(F)  P                                              N.S.

                        Unexposed group (combining nev+ with main levels)
                          nev any   nev cig     Total


                       N        6        16        22
                      NS        6        12        18


                      Wt   208.68     82.30    290.98
                 Het Chi   221.95     28.37    252.86
                 Het  df        5        15        21
                 Het  P       ***         *       ***
               Fixed  RR     2.54      3.12      2.69
                     RRl     2.21      2.51      2.40
                     RRu     2.90      3.87      3.02
                      P       +++       +++       +++
              Random  RR    14.77      3.18      5.00
                     RRl     2.54      2.28      2.87
                     RRu    85.84      4.43      8.72
                      P        ++       +++       +++
             Between Chi                         2.54
             Between  df                            1
             Between  P                          N.S.
             Btwn(F)  P                          N.S.


  ________________________________________________________________________________________________________________________
                                            International Evidence on Smoking and COPD, Phase 3, Analysis run on 27-SEP-10

                                                   Table 3 - B - 2 - 4

           IESCOPD - Meta-analysis of current smoking, cigarettes (or all products if cigarettes not available)
                                                      Any Emphysema
                                                      Least-adjusted


     REF|NRR|X|SEX|AGEL|AGEH|     REGION|BEGYR|PUBYR|STTYP|ONSET|      DISEAS|ADJ|SMOKSTA|   PRODUCT|    UNEXP|

  AUERBA   1 x   m   15   99      Am:USA  1963  1972    CS  Prev  Emp:viscomp   0 Current       Cigs   Nev any
    BEST  18     m   30   97   Am:Canada  1955  1967    Pr   Inc     Emp:mort   1 Current  Cigs only   Nev any
  DONTA2  11     m   25   84 Eu:SE/Balkn  1960  1984    Pr   Inc Emp:diagnosd   0 Current       Cigs  Nev cigs
  ENRIGH   7     m   65   99      Am:USA  1989  1994    CS  Prev Emp:diagnosd   0 Current       Cigs  Nev cigs
  ENRIGH  10     f   65   99      Am:USA  1989  1994    CS  Prev Emp:diagnosd   0 Current       Cigs  Nev cigs
  HARDIE  13     m   70   99    Eu:Scand  1998  2005    CS  Prev Emp:diagnosd   1 Current       Cigs  Nev cigs
  HARDIE  16     f   70   99    Eu:Scand  1998  2005    CS  Prev Emp:diagnosd   1 Current       Cigs  Nev cigs
  HIRAYA   1     m   40   99   Asia:FarE  1965  1982    Pr   Inc     Emp:mort   1 Current       Cigs  Nev cigs
  HIRAYA   2     f   40   99   Asia:FarE  1965  1982    Pr   Inc     Emp:mort   1 Current       Cigs  Nev cigs
      HO   9     b   70   99   Asia:FarE  1991  1999    CS  Prev Emp:self-rep   3 Current       Cigs  Nev cigs
  HUHTI1  70     m   40   64    Eu:Scand  1961  1965    CS  Prev    Emp:other   0 Current       Cigs   Nev any
  HUHTI1 154     f   40   64    Eu:Scand  1961  1965    CS  Prev    Emp:other   0 Current       Cigs  Nev cigs
   KAHN2  47     m   31   84      Am:USA  1954  1966    Pr   Inc     Emp:mort   1 Current       Cigs   Nev any
  LAVECC  31 x   m   15   99     Eu:West  1983  1988    CS  Prev Emp:self-rep   0 Current       Cigs   Nev any
  LAVECC  32 x   f   15   99     Eu:West  1983  1988    CS  Prev Emp:self-rep   0 Current       Cigs   Nev any
  LEBOWI  31 x   b   15   96      Am:USA  1972  1977    CS  Prev Emp:diagnosd   2 Current       Cigs  Nev cigs
  MILLER   7     m   15   99      Am:USA  1978  1988    CS  Prev Emp:diagnosd   1 Current       Cigs  Nev cigs
  MILLER  10     f   15   99      Am:USA  1978  1988    CS  Prev Emp:diagnosd   1 Current       Cigs  Nev cigs
   OMORI   1 x   m   40   69   Asia:FarE     *  2006    CS  Prev  Emp:viscomp   0 Current       Cigs  Nev cigs
   SILVA   7     b   20   99      Am:USA  1972  2004    Pr   Inc    Emp:other   6 Current       Cigs  Nev cigs
  VIKGRE   1     m   61   68    Eu:Scand  1994  2004    Pr   Inc  Emp:viscomp   0 Current       Cigs  Nev cigs
   WEISS  17     m   50   69      Am:USA  1961  1963    CS  Prev  Emp:viscomp   0 Current       Cigs   Nev any
     WEN  18     m   35   99   Asia:FarE  1982  2004    Pr   Inc     Emp:mort   1 Current       Cigs  Nev cigs


  ________________________________________________________________________________________________________________________
                                            International Evidence on Smoking and COPD, Phase 3, Analysis run on 27-SEP-10

                                                   Table 3 - B - 2 - 5

           IESCOPD - Meta-analysis of current smoking, cigarettes (or all products if cigarettes not available)
                                                      Any Emphysema
                                                      Least-adjusted


                        Number Exposed  Non-exposed
 REF    NRR SEX ADJ     Case    Cont    Case    Cont      RR        95.00%CI
 AUERBA 1   m   0        816      23      20     156    276.73 (148.38- 516.09)
*BEST   18  m   1         40       -       3       -      7.72 (  2.39-  24.95)
*DONTA2 11  m   0         10     275       4     127      1.15 (  0.37-   3.61)
 ENRIGH 7   m   0         32     199      22     694      5.07 (  2.88-   8.93)
 ENRIGH 10  f   0         18     364      26    1617      3.08 (  1.67-   5.67)
 Subtotal ENRIGH                                          4.03 (  2.66-   6.10)
 HARDIE 13  m   1         11       -       2       -     15.04 (  3.40-  66.58)
 HARDIE 16  f   1          5       -      11       -      3.59 (  1.38-   9.29)
 Subtotal HARDIE                                          5.45 (  2.44-  12.16)
*HIRAYA 1   m   1        156       -      17       -      2.22 (  1.34-   3.67)
*HIRAYA 2   f   1         14       -      42       -      2.72 (  1.48-   5.00)
 Subtotal HIRAYA                                          2.41 (  1.64-   3.55)
 HO     9   b   3          -       -       -       -      2.30 (  0.94-   5.62)
 HUHTI1 70  m   0         53     331       1     121     19.37 (  2.65- 141.64)
 HUHTI1 154 f   0          1      83      16     693      0.52 (  0.07-   3.99)
 Subtotal HUHTI1                                          3.31 (  0.80-  13.71)
*KAHN2  47  m   1        272       -      18       -     12.18 (  7.56-  19.62)
 LAVECC 31  m   0        371   14934     183   14129      1.92 (  1.60-   2.29)
 LAVECC 32  f   0         61    6565     412   29448      0.66 (  0.51-   0.87)
 Subtotal LAVECC                                          1.39 (  1.20-   1.61)
 LEBOWI 31  b   2         14       -       5       -      3.84 (  1.36-  10.84)
 MILLER 7   m   1          4       -       0       -      7.21 (  0.39- 133.19)
 MILLER 10  f   1          2       -       1       -      9.07 (  0.18- 446.54)
 Subtotal MILLER                                          7.83 (  0.76-  81.05)
 OMORI  1   m   0        116     264       3      97     14.21 (  4.41-  45.75)
*SILVA  7   b   6          -       -       -       -      4.10 (  2.03-   8.18)
*VIKGRE 1   m   0          2      40       0      26      3.27~(  0.16-  65.49)
 WEISS  17  m   0         22     124       1      35      6.21 (  0.81-  47.70)
*WEN    18  m   1         12       -       -       -      1.12 (  0.44-   2.88)
Partial Totals          2032   23202     787   47143
*prospective study                                        ~ With 0.5 adjustment for zero


 REF    NRR SEX ADJ             Ys       Ws       Qs       Ps
 AUERBA 1   m   0              5.62     9.89   220.54       0.00
*BEST   18  m   1              2.04     2.79     3.65       0.00
*DONTA2 11  m   0              0.14     2.95     1.69       0.80
 ENRIGH 7   m   0              1.62    12.02     6.29       0.00
 ENRIGH 10  f   0              1.12    10.27     0.51       0.00
 Subtotal ENRIGH               1.39    22.29     6.79
 HARDIE 13  m   1              2.71     1.74     5.69       0.00
 HARDIE 16  f   1              1.28     4.23     0.60       0.01
 Subtotal HARDIE               1.70     5.96     6.29
*HIRAYA 1   m   1              0.80    15.14     0.16       0.00
*HIRAYA 2   f   1              1.00    10.37     0.10       0.00
 Subtotal HIRAYA               0.88    25.51     0.26
 HO     9   b   3              0.83     4.81     0.02       0.07
 HUHTI1 70  m   0              2.96     0.97     4.13       0.00
 HUHTI1 154 f   0             -0.65     0.93     2.24       0.53
 Subtotal HUHTI1               1.20     1.90     6.37
*KAHN2  47  m   1              2.50    16.89    43.19       0.00
 LAVECC 31  m   0              0.65   120.52     7.50       0.00
 LAVECC 32  f   0             -0.41    52.61    90.30       0.00
 Subtotal LAVECC               0.33   173.13    97.81
 LEBOWI 31  b   2              1.35     3.57     0.71       0.01
 MILLER 7   m   1              1.98     0.45     0.52       0.18
 MILLER 10  f   1              2.20     0.25     0.43       0.27
 Subtotal MILLER               2.06     0.70     0.95
 OMORI  1   m   0              2.65     2.81     8.63       0.00
*SILVA  7   b   6              1.41     7.91     2.06       0.00
*VIKGRE 1   m   0              1.19     0.43     0.03       0.44
 WEISS  17  m   0              1.83     0.92     0.79       0.08
*WEN    18  m   1              0.11     4.35     2.70       0.81


  ________________________________________________________________________________________________________________________
                                            International Evidence on Smoking and COPD, Phase 3, Analysis run on 27-SEP-10

                                                   Table 3 - B - 2 - 5

           IESCOPD - Meta-analysis of current smoking, cigarettes (or all products if cigarettes not available)
                                                      Any Emphysema
                                                      Least-adjusted


                       N       23
                      NS       17


                      Wt   286.82
                 Het Chi   402.49
                 Het  df       22
                 Het  P       ***
               Fixed  RR     2.46
                     RRl     2.19
                     RRu     2.76
                      P       +++
              Random  RR     4.43
                     RRl     2.43
                     RRu     8.04
                      P       +++
               Asymm  P       (*)


  ________________________________________________________________________________________________________________________
                                            International Evidence on Smoking and COPD, Phase 3, Analysis run on 27-SEP-10

                                                   Table 3 - B - 2 - 6

           IESCOPD - Meta-analysis of current smoking, cigarettes (or all products if cigarettes not available)
                                                      Any Emphysema
                                                      Least-adjusted


                       N       23
                      NS       17


                      Wt   286.82
                 Het Chi   402.49
                 Het  df       22
                 Het  P       ***
               Fixed  RR     2.46
                     RRl     2.19
                     RRu     2.76
                      P       +++
              Random  RR     4.43
                     RRl     2.43
                     RRu     8.04
                      P       +++
               Asymm  P       (*)

                                   Sex
                             both      male    female     Total


                       N        3        14         6        23
                      NS        3        14         6        23


                      Wt    16.28    191.88     78.66    286.82
                 Het Chi     1.06    286.86     40.26    402.49
                 Het  df        2        13         5        22
                 Het  P      N.S.       ***       ***       ***
               Fixed  RR     3.41      3.36      1.08      2.46
                     RRl     2.10      2.92      0.86      2.19
                     RRu     5.54      3.87      1.34      2.76
                      P       +++       +++      N.S.       +++
              Random  RR     3.41      6.81      1.87      4.43
                     RRl     2.10      2.77      0.77      2.43
                     RRu     5.54     16.71      4.56      8.04
                      P       +++       +++      N.S.       +++
             Between Chi                                  74.30
             Between  df                                      2
             Between  P                                     ***
             Btwn(F)  P                                    N.S.


  ________________________________________________________________________________________________________________________
                                            International Evidence on Smoking and COPD, Phase 3, Analysis run on 27-SEP-10

                                                   Table 3 - B - 2 - 7

           IESCOPD - Meta-analysis of current smoking, cigarettes (or all products if cigarettes not available)
                                                      Any Emphysema
                                 Excluded studies (and stage at which they were excluded)


1       CLARK COTTON  MEYER REMYJA RUTGER SNYDER SOBRAX     SU TAKEMU  WANG4   WEIR WHICKE ZALACA
2      ALDERS ALESSA  AMIGO ANDER1 ANDER3   BANG  BECK1  BECK2 BEDNAR BJORNS BROGGE  BROWN CERVER CHAPMA  CHEN1  CHEN2
        CHEN3  CHENG CLEMEN COATES  COCCI COLLEG  DEAN1  DEAN2  DEANE DEJONG DEMARC DETORR DICKIN  DOLL1  DOLL2 DONTA1
       DOPICO EHRLIC EKBERG ENSTRO FERRI1 FERRI2 FERRI3  FIDAN FINKLE FLETCH FORAST FOXMAN FUKUCH GEIJER GODTFR GOLDBE
       HAENSZ HARIKK HARRIS HAWTHO  HAYES HEDMAN HIGGI2 HIGGI3 HIGGI4 HIGGI6 HOLLA2 HOLLNA  HOUSE HRUBEC HUCHON HUHTI2
       HUHTI3 ITABAS JACOBS JAENDI JENSEN JINDA2 JOHANN  JOSHI JOUSI1 KACHEL KARAKA KATANC   KATO KHOURY    KIM  KIRAZ
       KLAYTO KOJIMA KOTAN1 KOTAN2 KRZYZA  KUBIK KULLER    LAI   LAM1   LAM2   LAM3 LAMBER  LANGE LANGE2 LANGHA    LEE
         LIAW LINDBE LINDST   LIU1   LIU2 LUNDB1 LUNDB2  MADOR MAGNUS MANFRE MANNI1 MANNI2 MANNI3 MARAN1 MARAN2 MARCUS
       MATHES MELLST MENEZ1 MENEZ2 MENEZ3 MENEZ4 MENEZ5 MENEZ6  MEREN  MILNE MOLLER MONTNE MUELLE NEJJAR NIEPSU NIHLEN
       NILSSO OGILVI OSWAL1 OSWAL2 PANDEY   PEAT PELKON PEREZP   PETO  PRICE   REID RENWIC RICCIO RIMING SARGEA SAWICK
       SCHWAR SHAHAB  SHARP SHIMUR   SHIN SICHLE SOBRAD SPEIZE STERLI STJERN  STROM SUADIC  TAGER TAGER2   TANG   THUN
         TODD TROISI TRUPIN TSUSHI TVERDA URRUTI VESTBO VIEGI1 VIEGI2 VINEIS VOLLM1 VOLLM2 VONHER WAGEN2   WALD WATSON
          WIG WILHEL WILSO1 WOJTYN  WOODS  WOOLF   XIAO     XU YAMAGU   YUAN ZIELI1 ZIELI2 ZIETKO   ZOIA
3      WILSO2
4      ANDER2 GULSVI HAMMO2 HOZAWA   KAHN   NAWA  PRATT  RYDER SUTINE  WANG2


  ________________________________________________________________________________________________________________________
                                            International Evidence on Smoking and COPD, Phase 3, Analysis run on 27-SEP-10

                                                   Table 3 - B - 2 - 8

           IESCOPD - Meta-analysis of current smoking, cigarettes (or all products if cigarettes not available)
                                                      Any Emphysema
                                             Potentially overlapping studies


     REF| REFGP|PRINC|                     OVERLAP|

  ENRIGH ENRIGH     1         ENRIGH/HOZAWA/HARIKK
  DONTA2 JACOBS     2  JACOBS/DONTA1/DONTA2/PELKON
  HUHTI1 HUHTI1     1                HUHTI1/HUHTI2
  HARDIE HARDIE     1         HARDIE/JOHANN/BROGGE
  LEBOWI LEBOWI     1                 LEBOWI/SILVA
   SILVA  SILVA     1                 LEBOWI/SILVA
     WEN    WEN     1                     WEN/LIAW
   KAHN2   KAHN     2                   KAHN/KAHN2


  ________________________________________________________________________________________________________________________
                                            International Evidence on Smoking and COPD, Phase 3, Analysis run on 27-SEP-10

                                                    Table 3 - C - 1 -

IESCOPD - Meta-analysis of ever smoking (or current if ever not available), any product (or cigarettes if all product not
                                                      Any Emphysema


This analysis is restricted to results for:
1) Eligible study on database
2) Outcome Emphysema
3) Non-dose-response data
4) Ever or current smoking
5) Results complete enough for use in meta-analysis

Within each study, results are then selected (in the following order of preference, within each sex) for:
6) SMKSTA  : ever, current
7) UNEXP   : never any, never cigarettes, other
8) PROD    : any product, cigarettes, cigarettes only
9) For overlapping studies: principal rather than subsidiary studies
and then for single sex results (m, f) in preference to results for both sexes combined (b).

Results adjusted for the most potential confounders are then chosen in Sections -1 to -3
(and those which actually differ from the adjusted results in Table 3 - A - 1 - 1 are marked 'x' in Section -1)
and results adjusted for the least confounders in Sections -4 to -6. (Those least adjusted results which
actually differ from the most adjusted are marked 'x' in column X in Section -4)

Section -7 shows excluded studies, together with the stage (as above) at which no qualifying
results were found.

Section -8 lists the potentially overlapping studies which have been included (1=principal, 2=subsidiary),
and any results which would have been included in preference except that they had data not complete enough
for use in meta-analysis. It also lists their significance (yes/no), if known.


  ________________________________________________________________________________________________________________________
                                            International Evidence on Smoking and COPD, Phase 3, Analysis run on 27-SEP-10

                                                   Table 3 - C - 1 - 1

IESCOPD - Meta-analysis of ever smoking (or current if ever not available), any product (or cigarettes if all product not
                                                      Any Emphysema
                                                      Most-adjusted


     REF|NRR|Cmp3A1|SEX|AGEL|AGEH|     REGION|BEGYR|PUBYR|STTYP|ONSET|      DISEAS|ADJ|SMOKSTA|   PRODUCT|    UNEXP|

  ANDER2   1          b   15   99      Am:USA     *  1966    CS  Prev  Emp:viscomp   0    Ever  Cigs only   Nev any
  AUERBA   2      x   m   15   99      Am:USA  1963  1972    CS  Prev  Emp:viscomp   1 Current       Cigs   Nev any
    BEST  20          m   30   97   Am:Canada  1955  1967    Pr   Inc     Emp:mort   1    Ever  Cigs only   Nev any
  DONTA2  13          m   25   84 Eu:SE/Balkn  1960  1984    Pr   Inc Emp:diagnosd   0    Ever       Cigs  Nev cigs
  ENRIGH   9          m   65   99      Am:USA  1989  1994    CS  Prev Emp:diagnosd   0    Ever       Cigs  Nev cigs
  ENRIGH  12          f   65   99      Am:USA  1989  1994    CS  Prev Emp:diagnosd   0    Ever       Cigs  Nev cigs
  GULSVI   4          m   15   70    Eu:Scand  1972  1979    CS  Prev Emp:diagnosd   1    Ever        Any   Nev any
  GULSVI   6          f   15   70    Eu:Scand  1972  1979    CS  Prev Emp:diagnosd   1    Ever        Any   Nev any
  HAMMO2   7          m   35   99      Am:USA  1959  1966    Pr   Inc     Emp:mort   1    Ever       Cigs   Nev any
  HAMMO2   8          f   35   99      Am:USA  1959  1966    Pr   Inc     Emp:mort   1    Ever        Any   Nev any
  HARDIE  15          m   70   99    Eu:Scand  1998  2005    CS  Prev Emp:diagnosd   1    Ever       Cigs  Nev cigs
  HARDIE  18          f   70   99    Eu:Scand  1998  2005    CS  Prev Emp:diagnosd   1    Ever       Cigs  Nev cigs
  HIRAYA   1      x   m   40   99   Asia:FarE  1965  1982    Pr   Inc     Emp:mort   1 Current       Cigs  Nev cigs
  HIRAYA   2      x   f   40   99   Asia:FarE  1965  1982    Pr   Inc     Emp:mort   1 Current       Cigs  Nev cigs
      HO  12          b   70   99   Asia:FarE  1991  1999    CS  Prev Emp:self-rep   3    Ever       Cigs  Nev cigs
  HOZAWA   9          b   45   64      Am:USA  1987  2006    CS  Prev Emp:diagnosd   0    Ever       Cigs  Nev cigs
  HUHTI1  69          m   40   64    Eu:Scand  1961  1965    CS  Prev    Emp:other   0    Ever        Any   Nev any
  HUHTI1 145          f   40   64    Eu:Scand  1961  1965    CS  Prev    Emp:other   1    Ever        Any   Nev any
   KAHN2  80          m   31   84      Am:USA  1954  1966    Pr   Inc     Emp:mort   1    Ever        Any   Nev any
  LAVECC  30          b   15   99     Eu:West  1983  1988    CS  Prev Emp:self-rep   6    Ever        Any   Nev any
  LEBOWI  36          b   15   96      Am:USA  1972  1977    CS  Prev Emp:diagnosd   3    Ever       Cigs  Nev cigs
  MILLER   9          m   15   99      Am:USA  1978  1988    CS  Prev Emp:diagnosd   1    Ever       Cigs  Nev cigs
  MILLER  12          f   15   99      Am:USA  1978  1988    CS  Prev Emp:diagnosd   1    Ever       Cigs  Nev cigs
    NAWA   1          m   50   69   Asia:FarE  1998  2002    CS  Prev  Emp:viscomp   0    Ever       Cigs  Nev cigs
   OMORI   6          m   40   69   Asia:FarE     *  2006    CS  Prev  Emp:viscomp   1    Ever       Cigs  Nev cigs
   PRATT   1          m   15   99      Am:USA     *  1980    CS  Prev  Emp:viscomp   0    Ever        Any   Nev any
   RYDER   3          b   22   95       Eu:UK     *  1971    CS  Prev  Emp:viscomp   1    Ever        Any   Nev any
   SILVA   9          b   20   99      Am:USA  1972  2004    Pr   Inc    Emp:other   6    Ever       Cigs  Nev cigs
  SUTINE   1          b   10   99    Eu:Scand  1971  1978    CS  Prev  Emp:viscomp   0    Ever        Any   Nev any
  VIKGRE   1      x   m   61   68    Eu:Scand  1994  2004    Pr   Inc  Emp:viscomp   0 Current       Cigs  Nev cigs
   WANG2   1          b   19   92   Asia:FarE  1996  2001    CS  Prev  Emp:viscomp   0    Ever       Cigs  Nev cigs
   WEISS  16          m   50   69      Am:USA  1961  1963    CS  Prev  Emp:viscomp   0    Ever        Any   Nev any
     WEN  18      x   m   35   99   Asia:FarE  1982  2004    Pr   Inc     Emp:mort   1 Current       Cigs  Nev cigs


  ________________________________________________________________________________________________________________________
                                            International Evidence on Smoking and COPD, Phase 3, Analysis run on 27-SEP-10

                                                   Table 3 - C - 1 - 2

IESCOPD - Meta-analysis of ever smoking (or current if ever not available), any product (or cigarettes if all product not
                                                      Any Emphysema
                                                      Most-adjusted


                        Number Exposed  Non-exposed
 REF    NRR SEX ADJ     Case    Cont    Case    Cont      RR        95.00%CI
 ANDER2 1   b   0        102      12      30      21      5.95 (  2.63-  13.48)
 AUERBA 2   m   1        816       -      20       -    489.54 (211.74-1131.81)
*BEST   20  m   1         52       -       3       -      8.47 (  2.65-  27.11)
*DONTA2 13  m   0         11     333       4     127      1.05 (  0.34-   3.23)
 ENRIGH 9   m   0        113    1390      22     694      2.56 (  1.61-   4.09)
 ENRIGH 12  f   0         75    1182      26    1617      3.95 (  2.51-   6.20)
 Subtotal ENRIGH                                          3.20 (  2.31-   4.43)
 GULSVI 4   m   1         40       -       4       -      2.37 (  0.84-   6.66)
 GULSVI 6   f   1         28       -      16       -      1.82 (  0.98-   3.38)
 Subtotal GULSVI                                          1.95 (  1.15-   3.32)
*HAMMO2 7   m   1        369       -      20       -      8.64 (  5.51-  13.55)
*HAMMO2 8   f   1         24       -      21       -      6.44 (  3.57-  11.63)
 Subtotal HAMMO2                                          7.76 (  5.42-  11.09)
 HARDIE 15  m   1         48       -       2       -     13.38 (  3.14-  56.98)
 HARDIE 18  f   1         11       -      11       -      2.55 (  1.18-   5.48)
 Subtotal HARDIE                                          3.67 (  1.86-   7.23)
*HIRAYA 1   m   1        156       -      17       -      2.22 (  1.34-   3.67)
*HIRAYA 2   f   1         14       -      42       -      2.72 (  1.48-   5.00)
 Subtotal HIRAYA                                          2.41 (  1.64-   3.55)
 HO     12  b   3          -       -       -       -      2.87 (  1.42-   5.81)
 HOZAWA 9   b   0        185    8775      13    6533     10.59 (  6.03-  18.61)
 HUHTI1 69  m   0         64     467       1     121     16.58 (  2.28- 120.74)
 HUHTI1 145 f   1          3       -      16       -      1.97 (  0.54-   7.17)
 Subtotal HUHTI1                                          3.72 (  1.26-  10.98)
*KAHN2  80  m   1        408       -      18       -      9.11 (  5.68-  14.61)
 LAVECC 30  b   6        789       -     595       -      2.05 (  1.81-   2.33)
 LEBOWI 36  b   3         31       -       5       -      4.86 (  1.86-  12.74)
 MILLER 9   m   1          6       -       0       -      6.56 (  0.37- 116.09)
 MILLER 12  f   1          2       -       1       -      6.03 (  0.12- 308.75)
 Subtotal MILLER                                          6.37 (  0.63-  64.77)
 NAWA   1   m   0        654    4086      32    1372      6.86 (  4.79-   9.84)
 OMORI  6   m   1        135       -       3       -      9.73 (  3.18-  29.82)
 PRATT  1   m   0        178     246      15      82      3.96 (  2.21-   7.09)
 RYDER  3   b   1         80       -      21       -     11.69 (  5.16-  26.51)
*SILVA  9   b   6          -       -       -       -      2.10 (  1.09-   4.06)
 SUTINE 1   b   0         63      28      28      45      3.62 (  1.89-   6.92)
*VIKGRE 1   m   0          2      40       0      26      3.27~(  0.16-  65.49)
 WANG2  1   b   0        188    3408      42    4209      5.53 (  3.94-   7.75)
 WEISS  16  m   0         28     223       1      35      4.39 (  0.58-  33.34)
*WEN    18  m   1         12       -       -       -      1.12 (  0.44-   2.88)
Partial Totals          4687   20190    1029   14882
*prospective study                                        ~ With 0.5 adjustment for zero


 REF    NRR SEX ADJ             Ys       Ws       Qs       Ps
 ANDER2 1   b   0              1.78     5.74     1.96       0.00
 AUERBA 2   m   1              6.19     5.47   136.43       0.00
*BEST   20  m   1              2.14     2.84     2.50       0.00
*DONTA2 13  m   0              0.05     3.03     4.02       0.93
 ENRIGH 9   m   0              0.94    17.71     1.17       0.00
 ENRIGH 12  f   0              1.37    18.78     0.57       0.00
 Subtotal ENRIGH               1.16    36.49     1.74
 GULSVI 4   m   1              0.86     3.58     0.40       0.10
 GULSVI 6   f   1              0.60    10.02     3.61       0.06
 Subtotal GULSVI               0.67    13.61     4.01
*HAMMO2 7   m   1              2.16    18.98    17.40       0.00
*HAMMO2 8   f   1              1.86    11.02     4.85       0.00
 Subtotal HAMMO2               2.05    29.99    22.26
 HARDIE 15  m   1              2.59     1.83     3.56       0.00
 HARDIE 18  f   1              0.94     6.52     0.45       0.02
 Subtotal HARDIE               1.30     8.35     4.01
*HIRAYA 1   m   1              0.80    15.14     2.44       0.00
*HIRAYA 2   f   1              1.00    10.37     0.41       0.00
 Subtotal HIRAYA               0.88    25.51     2.84
 HO     12  b   3              1.05     7.74     0.16       0.00
 HOZAWA 9   b   0              2.36    12.11    16.34       0.00
 HUHTI1 69  m   0              2.81     0.97     2.52       0.01
 HUHTI1 145 f   1              0.68     2.30     0.62       0.30
 Subtotal HUHTI1               1.31     3.27     3.15
*KAHN2  80  m   1              2.21    17.22    17.58       0.00
 LAVECC 30  b   6              0.72   240.93    55.73       0.00
  ________________________________________________________________________________________________________________________
                                            International Evidence on Smoking and COPD, Phase 3, Analysis run on 27-SEP-10

                                                   Table 3 - C - 1 - 2

IESCOPD - Meta-analysis of ever smoking (or current if ever not available), any product (or cigarettes if all product not
                                                      Any Emphysema
                                                      Most-adjusted


 REF    NRR SEX ADJ             Ys       Ws       Qs       Ps
 LEBOWI 36  b   3              1.58     4.15     0.61       0.00
 MILLER 9   m   1              1.88     0.46     0.22       0.20
 MILLER 12  f   1              1.80     0.25     0.09       0.37
 Subtotal MILLER               1.85     0.71     0.31
 NAWA   1   m   0              1.93    29.63    15.67       0.00
 OMORI  6   m   1              2.28     3.07     3.55       0.00
 PRATT  1   m   0              1.38    11.29     0.35       0.00
 RYDER  3   b   1              2.46     5.74     9.11       0.00
*SILVA  9   b   6              0.74     8.89     1.85       0.03
 SUTINE 1   b   0              1.29     9.13     0.07       0.00
*VIKGRE 1   m   0              1.19     0.43     0.00       0.44
 WANG2  1   b   0              1.71    33.72     8.81       0.00
 WEISS  16  m   0              1.48     0.94     0.07       0.15
*WEN    18  m   1              0.11     4.35     5.13       0.81

                       N       33
                      NS       26


                      Wt   524.33
                 Het Chi   318.25
                 Het  df       32
                 Het  P       ***
               Fixed  RR     3.32
                     RRl     3.04
                     RRu     3.61
                      P       +++
              Random  RR     4.83
                     RRl     3.46
                     RRu     6.73
                      P       +++
               Asymm  P         *


  ________________________________________________________________________________________________________________________
                                            International Evidence on Smoking and COPD, Phase 3, Analysis run on 27-SEP-10

                                                   Table 3 - C - 1 - 3

IESCOPD - Meta-analysis of ever smoking (or current if ever not available), any product (or cigarettes if all product not
                                                      Any Emphysema
                                                      Most-adjusted


                       N       33
                      NS       26


                      Wt   524.33
                 Het Chi   318.25
                 Het  df       32
                 Het  P       ***
               Fixed  RR     3.32
                     RRl     3.04
                     RRu     3.61
                      P       +++
              Random  RR     4.83
                     RRl     3.46
                     RRu     6.73
                      P       +++
               Asymm  P         *

                                   Sex
                             both      male    female     Total


                       N        9        17         7        33
                      NS        9        17         7        33


                      Wt   328.14    136.94     59.25    524.33
                 Het Chi    76.66    169.68     10.60    318.25
                 Het  df        8        16         6        32
                 Het  P       ***       ***      N.S.       ***
               Fixed  RR     2.62      5.82      3.30      3.32
                     RRl     2.36      4.92      2.56      3.04
                     RRu     2.92      6.88      4.26      3.61
                      P       +++       +++       +++       +++
              Random  RR     4.47      6.20      3.17      4.83
                     RRl     2.72      3.37      2.19      3.46
                     RRu     7.34     11.42      4.59      6.73
                      P       +++       +++       +++       +++
             Between Chi                                  61.32
             Between  df                                      2
             Between  P                                     ***
             Btwn(F)  P                                       *

                                        Continent
                            NAmer    Europe      Asia  oth/mult     Total


                       N       15        11         7                  33
                      NS       12         8         6                  26


                      Wt   135.84    284.48    104.01              524.33
                 Het Chi   143.36     31.68     28.57              318.25
                 Het  df       14        10         6                  32
                 Het  P       ***       ***       ***                 ***
               Fixed  RR     6.40      2.20      4.35                3.32
                     RRl     5.41      1.95      3.59                3.04
                     RRu     7.57      2.47      5.27                3.61
                      P       +++       +++       +++                 +++
              Random  RR     7.42      3.12      3.62                4.83
                     RRl     4.12      2.03      2.28                3.46
                     RRu    13.36      4.80      5.74                6.73
                      P       +++       +++       +++                 +++
             Between Chi                                           114.64
             Between  df                                                2
             Between  P                                               ***
             Btwn(F)  P                                                **


  ________________________________________________________________________________________________________________________
                                            International Evidence on Smoking and COPD, Phase 3, Analysis run on 27-SEP-10

                                                   Table 3 - C - 1 - 3

IESCOPD - Meta-analysis of ever smoking (or current if ever not available), any product (or cigarettes if all product not
                                                      Any Emphysema
                                                      Most-adjusted
                        National cigarette tobacco type (excluding mixed/unkown)
                          blended  virginia     Total


                       N       30         2        32
                      NS       23         2        25


                      Wt   511.40      8.58    519.97
                 Het Chi   301.47      0.20    313.08
                 Het  df       29         1        31
                 Het  P       ***      N.S.       ***
               Fixed  RR     3.28     10.51      3.35
                     RRl     3.01      5.38      3.07
                     RRu     3.58     20.52      3.65
                      P       +++       +++       +++
              Random  RR     4.83     10.51      5.06
                     RRl     3.42      5.38      3.61
                     RRu     6.84     20.52      7.08
                      P       +++       +++       +++
             Between Chi                        11.42
             Between  df                            1
             Between  P                           ***
             Btwn(F)  P                          N.S.

                                        Start year of study
                            <1970   1970-79   1980-89   1990-99     2000+   unknown     Total


                       N       11         7         5         6                   4        33
                      NS        8         5         4         5                   4        26


                      Wt    88.26     36.49    293.88     79.86               25.84    524.33
                 Het Chi   145.46      4.90     39.37     10.09                5.22    318.25
                 Het  df       10         6         4         5                   3        32
                 Het  P       ***      N.S.       ***       (*)                N.S.       ***
               Fixed  RR     6.69      2.63      2.30      5.37                6.13      3.32
                     RRl     5.43      1.90      2.05      4.31                4.17      3.04
                     RRu     8.24      3.64      2.58      6.69                9.01      3.61
                      P       +++       +++       +++       +++                 +++       +++
              Random  RR     7.02      2.63      3.11      4.89                6.61      4.83
                     RRl     2.98      1.90      1.72      3.34                3.89      3.46
                     RRu    16.51      3.64      5.61      7.18               11.23      6.73
                      P       +++       +++       +++       +++                 +++       +++
             Between Chi                                                               113.21
             Between  df                                                                    4
             Between  P                                                                   ***
             Btwn(F)  P                                                                     *

                                Publication year
                            <1980   1980-89   1990-99     2000+     Total


                       N       14         7         3         9        33
                      NS       11         5         2         8        26


                      Wt    98.10    281.47     44.23    100.53    524.33
                 Het Chi   133.73      7.70      1.77     32.26    318.25
                 Het  df       13         6         2         8        32
                 Het  P       ***      N.S.      N.S.       ***       ***
               Fixed  RR     7.44      2.13      3.14      5.36      3.32
                     RRl     6.10      1.89      2.34      4.40      3.04
                     RRu     9.07      2.39      4.22      6.51      3.61
                      P       +++       +++       +++       +++       +++
              Random  RR     7.48      2.30      3.14      4.70      4.83
                     RRl     3.81      1.82      2.34      2.95      3.46
                     RRu    14.67      2.92      4.22      7.48      6.73
                      P       +++       +++       +++       +++       +++
             Between Chi                                           142.79
             Between  df                                                3
             Between  P                                               ***
             Btwn(F)  P                                               ***
  ________________________________________________________________________________________________________________________
                                            International Evidence on Smoking and COPD, Phase 3, Analysis run on 27-SEP-10

                                                   Table 3 - C - 1 - 3

IESCOPD - Meta-analysis of ever smoking (or current if ever not available), any product (or cigarettes if all product not
                                                      Any Emphysema
                                                      Most-adjusted
                               Study type
                               CC        Pr        CS     Total


                       N                 10        23        33
                      NS                  8        18        26


                      Wt              92.25    432.07    524.33
                 Het Chi              49.33    260.61    318.25
                 Het  df                  9        22        32
                 Het  P                 ***       ***       ***
               Fixed  RR               4.35      3.13      3.32
                     RRl               3.55      2.85      3.04
                     RRu               5.34      3.44      3.61
                      P                 +++       +++       +++
              Random  RR               3.58      5.60      4.83
                     RRl               2.13      3.65      3.46
                     RRu               6.01      8.58      6.73
                      P                 +++       +++       +++
             Between Chi                                   8.31
             Between  df                                      1
             Between  P                                      **
             Btwn(F)  P                                    N.S.

                                    Lowest age in RR
                        <25/unlim     25-39       40+   unknown     Total


                       N       13         6        14                  33
                      NS       11         5        10                  26


                      Wt   339.38     57.43    127.51              524.33
                 Het Chi   205.94     27.21     40.55              318.25
                 Het  df       12         5        13                  32
                 Het  P       ***       ***       ***                 ***
               Fixed  RR     2.72      6.35      4.19                3.32
                     RRl     2.45      4.90      3.52                3.04
                     RRu     3.03      8.23      4.99                3.61
                      P       +++       +++       +++                 +++
              Random  RR     5.68      4.56      4.20                4.83
                     RRl     2.94      2.36      2.95                3.46
                     RRu    10.99      8.80      5.98                6.73
                      P       +++       +++       +++                 +++
             Between Chi                                            44.56
             Between  df                                                2
             Between  P                                               ***
             Btwn(F)  P                                              N.S.

                                         Highest age in RR
                              <65     65-74     75-84 85+/unlim   unknown     Total


                       N        3         6         2        22                  33
                      NS        2         5         2        17                  26


                      Wt    15.38     47.67     20.25    441.04              524.33
                 Het Chi     5.93     16.60     12.04    249.01              318.25
                 Het  df        2         5         1        21                  32
                 Het  P       (*)        **       ***       ***                 ***
               Fixed  RR     8.48      4.83      6.59      2.99                3.32
                     RRl     5.14      3.63      4.26      2.72                3.04
                     RRu    13.97      6.41     10.19      3.28                3.61
                      P       +++       +++       +++       +++                 +++
              Random  RR     6.78      4.05      3.29      4.98                4.83
                     RRl     2.05      2.00      0.40      3.31                3.46
                     RRu    22.45      8.20     27.28      7.48                6.73
                      P        ++       +++      N.S.       +++                 +++
             Between Chi                                                      34.67
             Between  df                                                          3
             Between  P                                                         ***
             Btwn(F)  P                                                        N.S.
  ________________________________________________________________________________________________________________________
                                            International Evidence on Smoking and COPD, Phase 3, Analysis run on 27-SEP-10

                                                   Table 3 - C - 1 - 3

IESCOPD - Meta-analysis of ever smoking (or current if ever not available), any product (or cigarettes if all product not
                                                      Any Emphysema
                                                      Most-adjusted
                           Study weakness
                              Yes        No     Total


                       N        3        30        33
                      NS        3        23        26


                      Wt    17.39    506.94    524.33
                 Het Chi     7.73    310.33    318.25
                 Het  df        2        29        32
                 Het  P         *       ***       ***
               Fixed  RR     3.68      3.30      3.32
                     RRl     2.30      3.03      3.04
                     RRu     5.89      3.60      3.61
                      P       +++       +++       +++
              Random  RR     3.48      4.99      4.83
                     RRl     1.21      3.51      3.46
                     RRu    10.01      7.11      6.73
                      P         +       +++       +++
             Between Chi                         0.19
             Between  df                            1
             Between  P                          N.S.
             Btwn(F)  P                          N.S.

                        Emp subtype
                             mort     other     Total


                       N        7        26        33
                      NS        5        21        26


                      Wt    79.91    444.42    524.33
                 Het Chi    36.93    265.54    318.25
                 Het  df        6        25        32
                 Het  P       ***       ***       ***
               Fixed  RR     4.99      3.08      3.32
                     RRl     4.01      2.81      3.04
                     RRu     6.22      3.38      3.61
                      P       +++       +++       +++
              Random  RR     4.42      4.99      4.83
                     RRl     2.50      3.35      3.46
                     RRu     7.82      7.43      6.73
                      P       +++       +++       +++
             Between Chi                        15.78
             Between  df                            1
             Between  P                           ***
             Btwn(F)  P                          N.S.

                        Asthma analysis type (Emphysema)
                        inc-irres  excl-all     Total


                       N       30         3        33
                      NS       23         3        26


                      Wt   517.80      6.52    524.33
                 Het Chi   310.68      7.56    318.25
                 Het  df       29         2        32
                 Het  P       ***         *       ***
               Fixed  RR     3.32      3.22      3.32
                     RRl     3.04      1.49      3.04
                     RRu     3.62      6.94      3.61
                      P       +++        ++       +++
              Random  RR     4.97      3.21      4.83
                     RRl     3.52      0.59      3.46
                     RRu     7.01     17.48      6.73
                      P       +++      N.S.       +++
             Between Chi                         0.01
             Between  df                            1
             Between  P                          N.S.
             Btwn(F)  P                          N.S.
  ________________________________________________________________________________________________________________________
                                            International Evidence on Smoking and COPD, Phase 3, Analysis run on 27-SEP-10

                                                   Table 3 - C - 1 - 3

IESCOPD - Meta-analysis of ever smoking (or current if ever not available), any product (or cigarettes if all product not
                                                      Any Emphysema
                                                      Most-adjusted
                                Number of emphysema cases
                             1-50    51-100   101-200      201+     Total


                       N        8         9         5        11        33
                      NS        7         6         5         8        26


                      Wt    21.35     46.08     37.95    418.94    524.33
                 Het Chi     7.98     14.64      7.69    260.42    318.25
                 Het  df        7         8         4        10        32
                 Het  P      N.S.       (*)      N.S.       ***       ***
               Fixed  RR     2.39      2.87      7.30      3.19      3.32
                     RRl     1.56      2.15      5.31      2.90      3.04
                     RRu     3.65      3.83     10.03      3.51      3.61
                      P       +++       +++       +++       +++       +++
              Random  RR     2.38      3.19      7.48      6.37      4.83
                     RRl     1.47      2.10      4.73      3.54      3.46
                     RRu     3.85      4.85     11.85     11.46      6.73
                      P       +++       +++       +++       +++       +++
             Between Chi                                            27.52
             Between  df                                                3
             Between  P                                               ***
             Btwn(F)  P                                              N.S.

                            Analysis type
                         prevlnce     onset     Total


                       N       23        10        33
                      NS       18         8        26


                      Wt   432.07     92.25    524.33
                 Het Chi   260.61     49.33    318.25
                 Het  df       22         9        32
                 Het  P       ***       ***       ***
               Fixed  RR     3.13      4.35      3.32
                     RRl     2.85      3.55      3.04
                     RRu     3.44      5.34      3.61
                      P       +++       +++       +++
              Random  RR     5.60      3.58      4.83
                     RRl     3.65      2.13      3.46
                     RRu     8.58      6.01      6.73
                      P       +++       +++       +++
             Between Chi                         8.31
             Between  df                            1
             Between  P                            **
             Btwn(F)  P                          N.S.

                             Smoking product
                              any      cigs  cigsonly     Total


                       N       11        20         2        33
                      NS        9        16         2        27


                      Wt   313.14    202.60      8.59    524.33
                 Het Chi    70.34    185.91      0.24    318.25
                 Het  df       10        19         1        32
                 Het  P       ***       ***      N.S.       ***
               Fixed  RR     2.51      4.96      6.69      3.32
                     RRl     2.24      4.32      3.43      3.04
                     RRu     2.80      5.70     13.05      3.61
                      P       +++       +++       +++       +++
              Random  RR     4.16      5.06      6.69      4.83
                     RRl     2.55      3.14      3.43      3.46
                     RRu     6.78      8.13     13.05      6.73
                      P       +++       +++       +++       +++
             Between Chi                                  61.76
             Between  df                                      2
             Between  P                                     ***
             Btwn(F)  P                                       *
  ________________________________________________________________________________________________________________________
                                            International Evidence on Smoking and COPD, Phase 3, Analysis run on 27-SEP-10

                                                   Table 3 - C - 1 - 3

IESCOPD - Meta-analysis of ever smoking (or current if ever not available), any product (or cigarettes if all product not
                                                      Any Emphysema
                                                      Most-adjusted
                           Smoking status
                             ever   current     Total


                       N       28         5        33
                      NS       22         4        26


                      Wt   488.57     35.76    524.33
                 Het Chi   173.42    138.56    318.25
                 Het  df       27         4        32
                 Het  P       ***       ***       ***
               Fixed  RR     3.22      4.97      3.32
                     RRl     2.95      3.58      3.04
                     RRu     3.52      6.90      3.61
                      P       +++       +++       +++
              Random  RR     4.51      6.64      4.83
                     RRl     3.38      0.79      3.46
                     RRu     6.02     55.47      6.73
                      P       +++       (+)       +++
             Between Chi                         6.27
             Between  df                            1
             Between  P                             *
             Btwn(F)  P                          N.S.

                                     Unexposed group
                          nev any   nev cig  nev+ any  nev+ cig     Total


                       N       15        18                            33
                      NS       12        14                            26


                      Wt   346.17    178.16                        524.33
                 Het Chi   249.43     57.67                        318.25
                 Het  df       14        17                            32
                 Het  P       ***       ***                           ***
               Fixed  RR     2.99      4.06                          3.32
                     RRl     2.69      3.51                          3.04
                     RRu     3.32      4.71                          3.61
                      P       +++       +++                           +++
              Random  RR     6.64      3.66                          4.83
                     RRl     3.52      2.69                          3.46
                     RRu    12.54      5.00                          6.73
                      P       +++       +++                           +++
             Between Chi                                            11.16
             Between  df                                                1
             Between  P                                               ***
             Btwn(F)  P                                              N.S.

                        Unexposed group (combining nev+ with main levels)
                          nev any   nev cig     Total


                       N       15        18        33
                      NS       12        14        26


                      Wt   346.17    178.16    524.33
                 Het Chi   249.43     57.67    318.25
                 Het  df       14        17        32
                 Het  P       ***       ***       ***
               Fixed  RR     2.99      4.06      3.32
                     RRl     2.69      3.51      3.04
                     RRu     3.32      4.71      3.61
                      P       +++       +++       +++
              Random  RR     6.64      3.66      4.83
                     RRl     3.52      2.69      3.46
                     RRu    12.54      5.00      6.73
                      P       +++       +++       +++
             Between Chi                        11.16
             Between  df                            1
             Between  P                           ***
             Btwn(F)  P                          N.S.
  ________________________________________________________________________________________________________________________
                                            International Evidence on Smoking and COPD, Phase 3, Analysis run on 27-SEP-10

                                                   Table 3 - C - 1 - 3

IESCOPD - Meta-analysis of ever smoking (or current if ever not available), any product (or cigarettes if all product not
                                                      Any Emphysema
                                                      Most-adjusted
                        Smoking results reported in study (Emphysema)
                             Ever   Current      Both     Total


                       N       11         5        17        33
                      NS        9         4        13        26


                      Wt   150.96     35.76    337.61    524.33
                 Het Chi    31.30    138.56     66.29    318.25
                 Het  df       10         4        16        32
                 Het  P       ***       ***       ***       ***
               Fixed  RR     5.80      4.97      2.47      3.32
                     RRl     4.95      3.58      2.22      3.04
                     RRu     6.81      6.90      2.75      3.61
                      P       +++       +++       +++       +++
              Random  RR     5.51      6.64      3.77      4.83
                     RRl     4.08      0.79      2.63      3.46
                     RRu     7.43     55.47      5.42      6.73
                      P       +++       (+)       +++       +++
             Between Chi                                  82.11
             Between  df                                      2
             Between  P                                     ***
             Btwn(F)  P                                       *

                        Number of adjustment variables
                                0         1        2+     Total


                       N       12        17         4        33
                      NS       11        12         4        27


                      Wt   143.47    119.15    261.71    524.33
                 Het Chi    29.66    174.58      3.82    318.25
                 Het  df       11        16         3        32
                 Het  P        **       ***      N.S.       ***
               Fixed  RR     4.90      5.65      2.10      3.32
                     RRl     4.16      4.72      1.86      3.04
                     RRu     5.77      6.76      2.37      3.61
                      P       +++       +++       +++       +++
              Random  RR     4.63      5.98      2.26      4.83
                     RRl     3.39      3.16      1.75      3.46
                     RRu     6.32     11.32      2.93      6.73
                      P       +++       +++       +++       +++
             Between Chi                                 110.19
             Between  df                                      2
             Between  P                                     ***
             Btwn(F)  P                                      **

                        RR adjusted for sex (combined sex RR only)
                              Yes        No     Total


                       N        4         5         9
                      NS        4         5         9


                      Wt   261.71     66.43    328.14
                 Het Chi     3.82      8.87     76.66
                 Het  df        3         4         8
                 Het  P      N.S.       (*)       ***
               Fixed  RR     2.10      6.30      2.62
                     RRl     1.86      4.96      2.36
                     RRu     2.37      8.02      2.92
                      P       +++       +++       +++
              Random  RR     2.26      6.62      4.47
                     RRl     1.75      4.44      2.72
                     RRu     2.93      9.87      7.34
                      P       +++       +++       +++
             Between Chi                        63.97
             Between  df                            1
             Between  P                           ***
             Btwn(F)  P                           ***
  ________________________________________________________________________________________________________________________
                                            International Evidence on Smoking and COPD, Phase 3, Analysis run on 27-SEP-10

                                                   Table 3 - C - 1 - 3

IESCOPD - Meta-analysis of ever smoking (or current if ever not available), any product (or cigarettes if all product not
                                                      Any Emphysema
                                                      Most-adjusted
                        RR adjusted for age
                              Yes        No     Total


                       N       19        14        33
                      NS       15        12        27


                      Wt   380.14    144.19    524.33
                 Het Chi   258.00     29.71    318.25
                 Het  df       18        13        32
                 Het  P       ***        **       ***
               Fixed  RR     2.86      4.91      3.32
                     RRl     2.58      4.17      3.04
                     RRu     3.16      5.78      3.61
                      P       +++       +++       +++
              Random  RR     5.00      4.66      4.83
                     RRl     3.03      3.47      3.46
                     RRu     8.28      6.27      6.73
                      P       +++       +++       +++
             Between Chi                        30.54
             Between  df                            1
             Between  P                           ***
             Btwn(F)  P                           (*)

                        RR adjusted for factor other than sex, age


                       N        6        27        33
                      NS        5        21        26


                      Wt   262.42    261.91    524.33
                 Het Chi     4.69    205.53    318.25
                 Het  df        5        26        32
                 Het  P      N.S.       ***       ***
               Fixed  RR     2.11      5.22      3.32
                     RRl     1.87      4.63      3.04
                     RRu     2.38      5.90      3.61
                      P       +++       +++       +++
              Random  RR     2.11      5.29      4.83
                     RRl     1.87      3.67      3.46
                     RRu     2.38      7.64      6.73
                      P       +++       +++       +++
             Between Chi                       108.03
             Between  df                            1
             Between  P                           ***
             Btwn(F)  P                           ***

                        Derivation of RR/CI
                         Orig/2x2     Other     Total


                       N        5        28        33
                      NS        5        21        26


                      Wt    60.14    464.19    524.33
                 Het Chi    18.36    286.38    318.25
                 Het  df        4        27        32
                 Het  P        **       ***       ***
               Fixed  RR     5.18      3.13      3.32
                     RRl     4.02      2.86      3.04
                     RRu     6.67      3.43      3.61
                      P       +++       +++       +++
              Random  RR     4.40      4.96      4.83
                     RRl     2.42      3.39      3.46
                     RRu     8.00      7.25      6.73
                      P       +++       +++       +++
             Between Chi                        13.52
             Between  df                            1
             Between  P                           ***
             Btwn(F)  P                          N.S.

  ________________________________________________________________________________________________________________________
                                            International Evidence on Smoking and COPD, Phase 3, Analysis run on 27-SEP-10

                                                   Table 3 - C - 1 - 4

IESCOPD - Meta-analysis of ever smoking (or current if ever not available), any product (or cigarettes if all product not
                                                      Any Emphysema
                                                      Least-adjusted


     REF|NRR|X|SEX|AGEL|AGEH|     REGION|BEGYR|PUBYR|STTYP|ONSET|      DISEAS|ADJ|SMOKSTA|   PRODUCT|    UNEXP|

  ANDER2   1     b   15   99      Am:USA     *  1966    CS  Prev  Emp:viscomp   0    Ever  Cigs only   Nev any
  AUERBA   1 x   m   15   99      Am:USA  1963  1972    CS  Prev  Emp:viscomp   0 Current       Cigs   Nev any
    BEST  20     m   30   97   Am:Canada  1955  1967    Pr   Inc     Emp:mort   1    Ever  Cigs only   Nev any
  DONTA2  13     m   25   84 Eu:SE/Balkn  1960  1984    Pr   Inc Emp:diagnosd   0    Ever       Cigs  Nev cigs
  ENRIGH   9     m   65   99      Am:USA  1989  1994    CS  Prev Emp:diagnosd   0    Ever       Cigs  Nev cigs
  ENRIGH  12     f   65   99      Am:USA  1989  1994    CS  Prev Emp:diagnosd   0    Ever       Cigs  Nev cigs
  GULSVI   3 x   m   15   70    Eu:Scand  1972  1979    CS  Prev Emp:diagnosd   0    Ever        Any   Nev any
  GULSVI   5 x   f   15   70    Eu:Scand  1972  1979    CS  Prev Emp:diagnosd   0    Ever        Any   Nev any
  HAMMO2   7     m   35   99      Am:USA  1959  1966    Pr   Inc     Emp:mort   1    Ever       Cigs   Nev any
  HAMMO2   8     f   35   99      Am:USA  1959  1966    Pr   Inc     Emp:mort   1    Ever        Any   Nev any
  HARDIE  15     m   70   99    Eu:Scand  1998  2005    CS  Prev Emp:diagnosd   1    Ever       Cigs  Nev cigs
  HARDIE  18     f   70   99    Eu:Scand  1998  2005    CS  Prev Emp:diagnosd   1    Ever       Cigs  Nev cigs
  HIRAYA   1     m   40   99   Asia:FarE  1965  1982    Pr   Inc     Emp:mort   1 Current       Cigs  Nev cigs
  HIRAYA   2     f   40   99   Asia:FarE  1965  1982    Pr   Inc     Emp:mort   1 Current       Cigs  Nev cigs
      HO  12     b   70   99   Asia:FarE  1991  1999    CS  Prev Emp:self-rep   3    Ever       Cigs  Nev cigs
  HOZAWA   9     b   45   64      Am:USA  1987  2006    CS  Prev Emp:diagnosd   0    Ever       Cigs  Nev cigs
  HUHTI1  69     m   40   64    Eu:Scand  1961  1965    CS  Prev    Emp:other   0    Ever        Any   Nev any
  HUHTI1 144 x   f   40   64    Eu:Scand  1961  1965    CS  Prev    Emp:other   0    Ever        Any   Nev any
   KAHN2  80     m   31   84      Am:USA  1954  1966    Pr   Inc     Emp:mort   1    Ever        Any   Nev any
  LAVECC  24 x   m   15   99     Eu:West  1983  1988    CS  Prev Emp:self-rep   0    Ever        Any   Nev any
  LAVECC  27 x   f   15   99     Eu:West  1983  1988    CS  Prev Emp:self-rep   0    Ever        Any   Nev any
  LEBOWI  33 x   b   15   96      Am:USA  1972  1977    CS  Prev Emp:diagnosd   2    Ever       Cigs  Nev cigs
  MILLER   9     m   15   99      Am:USA  1978  1988    CS  Prev Emp:diagnosd   1    Ever       Cigs  Nev cigs
  MILLER  12     f   15   99      Am:USA  1978  1988    CS  Prev Emp:diagnosd   1    Ever       Cigs  Nev cigs
    NAWA   1     m   50   69   Asia:FarE  1998  2002    CS  Prev  Emp:viscomp   0    Ever       Cigs  Nev cigs
   OMORI   3 x   m   40   69   Asia:FarE     *  2006    CS  Prev  Emp:viscomp   0    Ever       Cigs  Nev cigs
   PRATT   1     m   15   99      Am:USA     *  1980    CS  Prev  Emp:viscomp   0    Ever        Any   Nev any
   RYDER   1 x   m   22   95       Eu:UK     *  1971    CS  Prev  Emp:viscomp   0    Ever        Any   Nev any
   RYDER   2 x   f   22   95       Eu:UK     *  1971    CS  Prev  Emp:viscomp   0    Ever        Any   Nev any
   SILVA   9     b   20   99      Am:USA  1972  2004    Pr   Inc    Emp:other   6    Ever       Cigs  Nev cigs
  SUTINE   1     b   10   99    Eu:Scand  1971  1978    CS  Prev  Emp:viscomp   0    Ever        Any   Nev any
  VIKGRE   1     m   61   68    Eu:Scand  1994  2004    Pr   Inc  Emp:viscomp   0 Current       Cigs  Nev cigs
   WANG2   1     b   19   92   Asia:FarE  1996  2001    CS  Prev  Emp:viscomp   0    Ever       Cigs  Nev cigs
   WEISS  16     m   50   69      Am:USA  1961  1963    CS  Prev  Emp:viscomp   0    Ever        Any   Nev any
     WEN  18     m   35   99   Asia:FarE  1982  2004    Pr   Inc     Emp:mort   1 Current       Cigs  Nev cigs


  ________________________________________________________________________________________________________________________
                                            International Evidence on Smoking and COPD, Phase 3, Analysis run on 27-SEP-10

                                                   Table 3 - C - 1 - 5

IESCOPD - Meta-analysis of ever smoking (or current if ever not available), any product (or cigarettes if all product not
                                                      Any Emphysema
                                                      Least-adjusted


                        Number Exposed  Non-exposed
 REF    NRR SEX ADJ     Case    Cont    Case    Cont      RR        95.00%CI
 ANDER2 1   b   0        102      12      30      21      5.95 (  2.63-  13.48)
 AUERBA 1   m   0        816      23      20     156    276.73 (148.38- 516.09)
*BEST   20  m   1         52       -       3       -      8.47 (  2.65-  27.11)
*DONTA2 13  m   0         11     333       4     127      1.05 (  0.34-   3.23)
 ENRIGH 9   m   0        113    1390      22     694      2.56 (  1.61-   4.09)
 ENRIGH 12  f   0         75    1182      26    1617      3.95 (  2.51-   6.20)
 Subtotal ENRIGH                                          3.20 (  2.31-   4.43)
 GULSVI 3   m   0         40    5812       4    2090      3.60 (  1.29-  10.06)
 GULSVI 5   f   0         28    4913      16    4231      1.51 (  0.81-   2.79)
 Subtotal GULSVI                                          1.90 (  1.12-   3.21)
*HAMMO2 7   m   1        369       -      20       -      8.64 (  5.51-  13.55)
*HAMMO2 8   f   1         24       -      21       -      6.44 (  3.57-  11.63)
 Subtotal HAMMO2                                          7.76 (  5.42-  11.09)
 HARDIE 15  m   1         48       -       2       -     13.38 (  3.14-  56.98)
 HARDIE 18  f   1         11       -      11       -      2.55 (  1.18-   5.48)
 Subtotal HARDIE                                          3.67 (  1.86-   7.23)
*HIRAYA 1   m   1        156       -      17       -      2.22 (  1.34-   3.67)
*HIRAYA 2   f   1         14       -      42       -      2.72 (  1.48-   5.00)
 Subtotal HIRAYA                                          2.41 (  1.64-   3.55)
 HO     12  b   3          -       -       -       -      2.87 (  1.42-   5.81)
 HOZAWA 9   b   0        185    8775      13    6533     10.59 (  6.03-  18.61)
 HUHTI1 69  m   0         64     467       1     121     16.58 (  2.28- 120.74)
 HUHTI1 144 f   0          3     111      16     693      1.17 (  0.34-   4.08)
 Subtotal HUHTI1                                          2.48 (  0.86-   7.15)
*KAHN2  80  m   1        408       -      18       -      9.11 (  5.68-  14.61)
 LAVECC 24  m   0        700   19775     183   14129      2.73 (  2.32-   3.22)
 LAVECC 27  f   0         89    7548     412   29448      0.84 (  0.67-   1.06)
 Subtotal LAVECC                                          1.84 (  1.61-   2.10)
 LEBOWI 33  b   2         31       -       5       -      5.08 (  1.95-  13.23)
 MILLER 9   m   1          6       -       0       -      6.56 (  0.37- 116.09)
 MILLER 12  f   1          2       -       1       -      6.03 (  0.12- 308.75)
 Subtotal MILLER                                          6.37 (  0.63-  64.77)
 NAWA   1   m   0        654    4086      32    1372      6.86 (  4.79-   9.84)
 OMORI  3   m   0        135     380       3      97     11.49 (  3.58-  36.85)
 PRATT  1   m   0        178     246      15      82      3.96 (  2.21-   7.09)
 RYDER  1   m   0         56      14       7       9      5.14 (  1.63-  16.21)
 RYDER  2   f   0         24      12      14      43      6.14 (  2.45-  15.39)
 Subtotal RYDER                                           5.73 (  2.80-  11.74)
*SILVA  9   b   6          -       -       -       -      2.10 (  1.09-   4.06)
 SUTINE 1   b   0         63      28      28      45      3.62 (  1.89-   6.92)
*VIKGRE 1   m   0          2      40       0      26      3.27~(  0.16-  65.49)
 WANG2  1   b   0        188    3408      42    4209      5.53 (  3.94-   7.75)
 WEISS  16  m   0         28     223       1      35      4.39 (  0.58-  33.34)
*WEN    18  m   1         12       -       -       -      1.12 (  0.44-   2.88)
Partial Totals          4687   58778    1029   65778
*prospective study                                        ~ With 0.5 adjustment for zero


 REF    NRR SEX ADJ             Ys       Ws       Qs       Ps
 ANDER2 1   b   0              1.78     5.74     1.95       0.00
 AUERBA 1   m   0              5.62     9.89   193.39       0.00
*BEST   20  m   1              2.14     2.84     2.49       0.00
*DONTA2 13  m   0              0.05     3.03     4.03       0.93
 ENRIGH 9   m   0              0.94    17.71     1.19       0.00
 ENRIGH 12  f   0              1.37    18.78     0.55       0.00
 Subtotal ENRIGH               1.16    36.49     1.74
 GULSVI 3   m   0              1.28     3.63     0.02       0.01
 GULSVI 5   f   0              0.41    10.14     6.34       0.19
 Subtotal GULSVI               0.64    13.76     6.36
*HAMMO2 7   m   1              2.16    18.98    17.32       0.00
*HAMMO2 8   f   1              1.86    11.02     4.82       0.00
 Subtotal HAMMO2               2.05    29.99    22.14
 HARDIE 15  m   1              2.59     1.83     3.55       0.00
 HARDIE 18  f   1              0.94     6.52     0.46       0.02
 Subtotal HARDIE               1.30     8.35     4.01
*HIRAYA 1   m   1              0.80    15.14     2.46       0.00
*HIRAYA 2   f   1              1.00    10.37     0.42       0.00
 Subtotal HIRAYA               0.88    25.51     2.88
 HO     12  b   3              1.05     7.74     0.17       0.00
 HOZAWA 9   b   0              2.36    12.11    16.27       0.00
 HUHTI1 69  m   0              2.81     0.97     2.52       0.01
  ________________________________________________________________________________________________________________________
                                            International Evidence on Smoking and COPD, Phase 3, Analysis run on 27-SEP-10

                                                   Table 3 - C - 1 - 5

IESCOPD - Meta-analysis of ever smoking (or current if ever not available), any product (or cigarettes if all product not
                                                      Any Emphysema
                                                      Least-adjusted


 REF    NRR SEX ADJ             Ys       Ws       Qs       Ps
 HUHTI1 144 f   0              0.16     2.46     2.68       0.80
 Subtotal HUHTI1               0.91     3.44     5.20
*KAHN2  80  m   1              2.21    17.22    17.50       0.00
 LAVECC 24  m   0              1.01   142.56     5.45       0.00
 LAVECC 27  f   0             -0.17    72.31   136.12       0.15
 Subtotal LAVECC               0.61   214.87   141.58
 LEBOWI 33  b   2              1.63     4.19     0.75       0.00
 MILLER 9   m   1              1.88     0.46     0.21       0.20
 MILLER 12  f   1              1.80     0.25     0.09       0.37
 Subtotal MILLER               1.85     0.71     0.30
 NAWA   1   m   0              1.93    29.63    15.58       0.00
 OMORI  3   m   0              2.44     2.83     4.35       0.00
 PRATT  1   m   0              1.38    11.29     0.34       0.00
 RYDER  1   m   0              1.64     2.91     0.56       0.01
 RYDER  2   f   0              1.82     4.55     1.72       0.00
 Subtotal RYDER                1.75     7.47     2.27
*SILVA  9   b   6              0.74     8.89     1.87       0.03
 SUTINE 1   b   0              1.29     9.13     0.07       0.00
*VIKGRE 1   m   0              1.19     0.43     0.00       0.44
 WANG2  1   b   0              1.71    33.72     8.73       0.00
 WEISS  16  m   0              1.48     0.94     0.07       0.15
*WEN    18  m   1              0.11     4.35     5.15       0.81

                       N       35
                      NS       26


                      Wt   504.54
                 Het Chi   459.20
                 Het  df       34
                 Het  P       ***
               Fixed  RR     3.32
                     RRl     3.05
                     RRu     3.63
                      P       +++
              Random  RR     4.47
                     RRl     3.11
                     RRu     6.44
                      P       +++
               Asymm  P       (*)


  ________________________________________________________________________________________________________________________
                                            International Evidence on Smoking and COPD, Phase 3, Analysis run on 27-SEP-10

                                                   Table 3 - C - 1 - 6

IESCOPD - Meta-analysis of ever smoking (or current if ever not available), any product (or cigarettes if all product not
                                                      Any Emphysema
                                                      Least-adjusted


                       N       35
                      NS       26


                      Wt   504.54
                 Het Chi   459.20
                 Het  df       34
                 Het  P       ***
               Fixed  RR     3.32
                     RRl     3.05
                     RRu     3.63
                      P       +++
              Random  RR     4.47
                     RRl     3.11
                     RRu     6.44
                      P       +++
               Asymm  P       (*)

                                   Sex
                             both      male    female     Total


                       N        7        19         9        35
                      NS        7        19         9        35


                      Wt    81.52    286.64    136.38    504.54
                 Het Chi    17.35    259.93     79.67    459.20
                 Het  df        6        18         8        34
                 Het  P        **       ***       ***       ***
               Fixed  RR     4.91      4.22      1.59      3.32
                     RRl     3.95      3.76      1.35      3.05
                     RRu     6.10      4.73      1.89      3.63
                      P       +++       +++       +++       +++
              Random  RR     4.60      5.88      2.57      4.47
                     RRl     3.07      3.39      1.34      3.11
                     RRu     6.87     10.20      4.93      6.44
                      P       +++       +++        ++       +++
             Between Chi                                 102.25
             Between  df                                      2
             Between  P                                     ***
             Btwn(F)  P                                       *

                                        Continent
                            NAmer    Europe      Asia  oth/mult     Total


                       N       15        13         7                  35
                      NS       12         8         6                  26


                      Wt   140.30    260.47    103.77              504.54
                 Het Chi   179.39     94.91     29.25              459.20
                 Het  df       14        12         6                  34
                 Het  P       ***       ***       ***                 ***
               Fixed  RR     7.05      1.99      4.36                3.32
                     RRl     5.98      1.76      3.59                3.05
                     RRu     8.32      2.25      5.28                3.63
                      P       +++       +++       +++                 +++
              Random  RR     7.26      2.73      3.66                4.47
                     RRl     3.82      1.69      2.29                3.11
                     RRu    13.80      4.43      5.84                6.44
                      P       +++       +++       +++                 +++
             Between Chi                                           155.65
             Between  df                                                2
             Between  P                                               ***
             Btwn(F)  P                                                **


  ________________________________________________________________________________________________________________________
                                            International Evidence on Smoking and COPD, Phase 3, Analysis run on 27-SEP-10

                                                   Table 3 - C - 1 - 6

IESCOPD - Meta-analysis of ever smoking (or current if ever not available), any product (or cigarettes if all product not
                                                      Any Emphysema
                                                      Least-adjusted
                               Study type
                               CC        Pr        CS     Total


                       N                 10        25        35
                      NS                  8        18        26


                      Wt              92.25    412.29    504.54
                 Het Chi              49.33    401.62    459.20
                 Het  df                  9        24        34
                 Het  P                 ***       ***       ***
               Fixed  RR               4.35      3.13      3.32
                     RRl               3.55      2.84      3.05
                     RRu               5.34      3.45      3.63
                      P                 +++       +++       +++
              Random  RR               3.58      4.97      4.47
                     RRl               2.13      3.13      3.11
                     RRu               6.01      7.87      6.44
                      P                 +++       +++       +++
             Between Chi                                   8.25
             Between  df                                      1
             Between  P                                      **
             Btwn(F)  P                                    N.S.

                        Emp subtype
                             mort     other     Total


                       N        7        28        35
                      NS        5        21        26


                      Wt    79.91    424.63    504.54
                 Het Chi    36.93    406.54    459.20
                 Het  df        6        27        34
                 Het  P       ***       ***       ***
               Fixed  RR     4.99      3.08      3.32
                     RRl     4.01      2.80      3.05
                     RRu     6.22      3.39      3.63
                      P       +++       +++       +++
              Random  RR     4.42      4.51      4.47
                     RRl     2.50      2.93      3.11
                     RRu     7.82      6.95      6.44
                      P       +++       +++       +++
             Between Chi                        15.72
             Between  df                            1
             Between  P                           ***
             Btwn(F)  P                          N.S.

                             Smoking product
                              any      cigs  cigsonly     Total


                       N       13        20         2        35
                      NS        9        16         2        27


                      Wt   289.13    206.83      8.59    504.54
                 Het Chi   140.72    230.01      0.24    459.20
                 Het  df       12        19         1        34
                 Het  P       ***       ***      N.S.       ***
               Fixed  RR     2.32      5.34      6.69      3.32
                     RRl     2.07      4.66      3.43      3.05
                     RRu     2.60      6.12     13.05      3.63
                      P       +++       +++       +++       +++
              Random  RR     3.46      5.03      6.69      4.47
                     RRl     2.09      2.99      3.43      3.11
                     RRu     5.72      8.46     13.05      6.44
                      P       +++       +++       +++       +++
             Between Chi                                  88.23
             Between  df                                      2
             Between  P                                     ***
             Btwn(F)  P                                       *
  ________________________________________________________________________________________________________________________
                                            International Evidence on Smoking and COPD, Phase 3, Analysis run on 27-SEP-10

                                                   Table 3 - C - 1 - 6

IESCOPD - Meta-analysis of ever smoking (or current if ever not available), any product (or cigarettes if all product not
                                                      Any Emphysema
                                                      Least-adjusted
                                     Unexposed group
                          nev any   nev cig  nev+ any  nev+ cig     Total


                       N       17        18                            35
                      NS       12        14                            26


                      Wt   326.58    177.96                        504.54
                 Het Chi   389.34     58.46                        459.20
                 Het  df       16        17                            34
                 Het  P       ***       ***                           ***
               Fixed  RR     2.97      4.07                          3.32
                     RRl     2.67      3.52                          3.05
                     RRu     3.32      4.72                          3.63
                      P       +++       +++                           +++
              Random  RR     5.38      3.69                          4.47
                     RRl     2.87      2.70                          3.11
                     RRu    10.10      5.05                          6.44
                      P       +++       +++                           +++
             Between Chi                                            11.40
             Between  df                                                1
             Between  P                                               ***
             Btwn(F)  P                                              N.S.

                        Unexposed group (combining nev+ with main levels)
                          nev any   nev cig     Total


                       N       17        18        35
                      NS       12        14        26


                      Wt   326.58    177.96    504.54
                 Het Chi   389.34     58.46    459.20
                 Het  df       16        17        34
                 Het  P       ***       ***       ***
               Fixed  RR     2.97      4.07      3.32
                     RRl     2.67      3.52      3.05
                     RRu     3.32      4.72      3.63
                      P       +++       +++       +++
              Random  RR     5.38      3.69      4.47
                     RRl     2.87      2.70      3.11
                     RRu    10.10      5.05      6.44
                      P       +++       +++       +++
             Between Chi                        11.40
             Between  df                            1
             Between  P                           ***
             Btwn(F)  P                          N.S.


  ________________________________________________________________________________________________________________________
                                            International Evidence on Smoking and COPD, Phase 3, Analysis run on 27-SEP-10

                                                   Table 3 - C - 1 - 7

IESCOPD - Meta-analysis of ever smoking (or current if ever not available), any product (or cigarettes if all product not
                                                      Any Emphysema
                                 Excluded studies (and stage at which they were excluded)


1       CLARK COTTON  MEYER REMYJA RUTGER SNYDER SOBRAX     SU TAKEMU  WANG4   WEIR WHICKE ZALACA
2      ALDERS ALESSA  AMIGO ANDER1 ANDER3   BANG  BECK1  BECK2 BEDNAR BJORNS BROGGE  BROWN CERVER CHAPMA  CHEN1  CHEN2
        CHEN3  CHENG CLEMEN COATES  COCCI COLLEG  DEAN1  DEAN2  DEANE DEJONG DEMARC DETORR DICKIN  DOLL1  DOLL2 DONTA1
       DOPICO EHRLIC EKBERG ENSTRO FERRI1 FERRI2 FERRI3  FIDAN FINKLE FLETCH FORAST FOXMAN FUKUCH GEIJER GODTFR GOLDBE
       HAENSZ HARIKK HARRIS HAWTHO  HAYES HEDMAN HIGGI2 HIGGI3 HIGGI4 HIGGI6 HOLLA2 HOLLNA  HOUSE HRUBEC HUCHON HUHTI2
       HUHTI3 ITABAS JACOBS JAENDI JENSEN JINDA2 JOHANN  JOSHI JOUSI1 KACHEL KARAKA KATANC   KATO KHOURY    KIM  KIRAZ
       KLAYTO KOJIMA KOTAN1 KOTAN2 KRZYZA  KUBIK KULLER    LAI   LAM1   LAM2   LAM3 LAMBER  LANGE LANGE2 LANGHA    LEE
         LIAW LINDBE LINDST   LIU1   LIU2 LUNDB1 LUNDB2  MADOR MAGNUS MANFRE MANNI1 MANNI2 MANNI3 MARAN1 MARAN2 MARCUS
       MATHES MELLST MENEZ1 MENEZ2 MENEZ3 MENEZ4 MENEZ5 MENEZ6  MEREN  MILNE MOLLER MONTNE MUELLE NEJJAR NIEPSU NIHLEN
       NILSSO OGILVI OSWAL1 OSWAL2 PANDEY   PEAT PELKON PEREZP   PETO  PRICE   REID RENWIC RICCIO RIMING SARGEA SAWICK
       SCHWAR SHAHAB  SHARP SHIMUR   SHIN SICHLE SOBRAD SPEIZE STERLI STJERN  STROM SUADIC  TAGER TAGER2   TANG   THUN
         TODD TROISI TRUPIN TSUSHI TVERDA URRUTI VESTBO VIEGI1 VIEGI2 VINEIS VOLLM1 VOLLM2 VONHER WAGEN2   WALD WATSON
          WIG WILHEL WILSO1 WOJTYN  WOODS  WOOLF   XIAO     XU YAMAGU   YUAN ZIELI1 ZIELI2 ZIETKO   ZOIA
3      WILSO2
4        KAHN


  ________________________________________________________________________________________________________________________
                                            International Evidence on Smoking and COPD, Phase 3, Analysis run on 27-SEP-10

                                                   Table 3 - C - 1 - 8

IESCOPD - Meta-analysis of ever smoking (or current if ever not available), any product (or cigarettes if all product not
                                                      Any Emphysema
                                             Potentially overlapping studies


     REF| REFGP|PRINC|                     OVERLAP|

  HOZAWA HOZAWA     1         ENRIGH/HOZAWA/HARIKK
  ENRIGH ENRIGH     1         ENRIGH/HOZAWA/HARIKK
  DONTA2 JACOBS     2  JACOBS/DONTA1/DONTA2/PELKON
  HUHTI1 HUHTI1     1                HUHTI1/HUHTI2
  HARDIE HARDIE     1         HARDIE/JOHANN/BROGGE
  HAMMO2 HAMMO2     1                HAMMO2/ENSTRO
  LEBOWI LEBOWI     1                 LEBOWI/SILVA
   SILVA  SILVA     1                 LEBOWI/SILVA
     WEN    WEN     1                     WEN/LIAW
   KAHN2   KAHN     2                   KAHN/KAHN2


  ________________________________________________________________________________________________________________________
                                            International Evidence on Smoking and COPD, Phase 3, Analysis run on 27-SEP-10

                                                    Table 3 - C - 2 -

IESCOPD - Meta-analysis of ever smoking (or current if ever not available), cigarettes (or all products if cigarettes not
                                                      Any Emphysema


This analysis is restricted to results for:
1) Eligible study on database
2) Outcome Emphysema
3) Non-dose-response data
4) Ever or current smoking
5) Results complete enough for use in meta-analysis

Within each study, results are then selected (in the following order of preference, within each sex) for:
6) SMKSTA  : ever, current
7) UNEXP   : never cigarettes, never any, other
8) PROD    : cigarettes, cigarettes only, any product
9) For overlapping studies: principal rather than subsidiary studies
and then for single sex results (m, f) in preference to results for both sexes combined (b).

Results adjusted for the most potential confounders are then chosen in Sections -1 to -3
(and those which actually differ from the adjusted results in Table 3 - C - 1 - 1 are marked 'x' in Section -1)
and results adjusted for the least confounders in Sections -4 to -6. (Those least adjusted results which
actually differ from the most adjusted are marked 'x' in column X in Section -4)

Section -7 shows excluded studies, together with the stage (as above) at which no qualifying
results were found.

Section -8 lists the potentially overlapping studies which have been included (1=principal, 2=subsidiary),
and any results which would have been included in preference except that they had data not complete enough
for use in meta-analysis. It also lists their significance (yes/no), if known.


  ________________________________________________________________________________________________________________________
                                            International Evidence on Smoking and COPD, Phase 3, Analysis run on 27-SEP-10

                                                   Table 3 - C - 2 - 1

IESCOPD - Meta-analysis of ever smoking (or current if ever not available), cigarettes (or all products if cigarettes not
                                                      Any Emphysema
                                                      Most-adjusted


     REF|NRR|Cmp3C1|SEX|AGEL|AGEH|     REGION|BEGYR|PUBYR|STTYP|ONSET|      DISEAS|ADJ|SMOKSTA|   PRODUCT|    UNEXP|

  ANDER2   1          b   15   99      Am:USA     *  1966    CS  Prev  Emp:viscomp   0    Ever  Cigs only   Nev any
  AUERBA   2          m   15   99      Am:USA  1963  1972    CS  Prev  Emp:viscomp   1 Current       Cigs   Nev any
    BEST  20          m   30   97   Am:Canada  1955  1967    Pr   Inc     Emp:mort   1    Ever  Cigs only   Nev any
  DONTA2  13          m   25   84 Eu:SE/Balkn  1960  1984    Pr   Inc Emp:diagnosd   0    Ever       Cigs  Nev cigs
  ENRIGH   9          m   65   99      Am:USA  1989  1994    CS  Prev Emp:diagnosd   0    Ever       Cigs  Nev cigs
  ENRIGH  12          f   65   99      Am:USA  1989  1994    CS  Prev Emp:diagnosd   0    Ever       Cigs  Nev cigs
  GULSVI   4          m   15   70    Eu:Scand  1972  1979    CS  Prev Emp:diagnosd   1    Ever        Any   Nev any
  GULSVI   6          f   15   70    Eu:Scand  1972  1979    CS  Prev Emp:diagnosd   1    Ever        Any   Nev any
  HAMMO2   7          m   35   99      Am:USA  1959  1966    Pr   Inc     Emp:mort   1    Ever       Cigs   Nev any
  HAMMO2  16      x   f   35   99      Am:USA  1959  1966    Pr   Inc     Emp:mort   1    Ever       Cigs  Nev cigs
  HARDIE  15          m   70   99    Eu:Scand  1998  2005    CS  Prev Emp:diagnosd   1    Ever       Cigs  Nev cigs
  HARDIE  18          f   70   99    Eu:Scand  1998  2005    CS  Prev Emp:diagnosd   1    Ever       Cigs  Nev cigs
  HIRAYA   1          m   40   99   Asia:FarE  1965  1982    Pr   Inc     Emp:mort   1 Current       Cigs  Nev cigs
  HIRAYA   2          f   40   99   Asia:FarE  1965  1982    Pr   Inc     Emp:mort   1 Current       Cigs  Nev cigs
      HO  12          b   70   99   Asia:FarE  1991  1999    CS  Prev Emp:self-rep   3    Ever       Cigs  Nev cigs
  HOZAWA   9          b   45   64      Am:USA  1987  2006    CS  Prev Emp:diagnosd   0    Ever       Cigs  Nev cigs
  HUHTI1  69          m   40   64    Eu:Scand  1961  1965    CS  Prev    Emp:other   0    Ever        Any   Nev any
  HUHTI1 157      x   f   40   64    Eu:Scand  1961  1965    CS  Prev    Emp:other   1    Ever       Cigs  Nev cigs
   KAHN2  81      x   m   31   84      Am:USA  1954  1966    Pr   Inc     Emp:mort   1    Ever       Cigs   Nev any
  LAVECC  30          b   15   99     Eu:West  1983  1988    CS  Prev Emp:self-rep   6    Ever        Any   Nev any
  LEBOWI  36          b   15   96      Am:USA  1972  1977    CS  Prev Emp:diagnosd   3    Ever       Cigs  Nev cigs
  MILLER   9          m   15   99      Am:USA  1978  1988    CS  Prev Emp:diagnosd   1    Ever       Cigs  Nev cigs
  MILLER  12          f   15   99      Am:USA  1978  1988    CS  Prev Emp:diagnosd   1    Ever       Cigs  Nev cigs
    NAWA   1          m   50   69   Asia:FarE  1998  2002    CS  Prev  Emp:viscomp   0    Ever       Cigs  Nev cigs
   OMORI   6          m   40   69   Asia:FarE     *  2006    CS  Prev  Emp:viscomp   1    Ever       Cigs  Nev cigs
   PRATT   1          m   15   99      Am:USA     *  1980    CS  Prev  Emp:viscomp   0    Ever        Any   Nev any
   RYDER   3          b   22   95       Eu:UK     *  1971    CS  Prev  Emp:viscomp   1    Ever        Any   Nev any
   SILVA   9          b   20   99      Am:USA  1972  2004    Pr   Inc    Emp:other   6    Ever       Cigs  Nev cigs
  SUTINE   1          b   10   99    Eu:Scand  1971  1978    CS  Prev  Emp:viscomp   0    Ever        Any   Nev any
  VIKGRE   1          m   61   68    Eu:Scand  1994  2004    Pr   Inc  Emp:viscomp   0 Current       Cigs  Nev cigs
   WANG2   1          b   19   92   Asia:FarE  1996  2001    CS  Prev  Emp:viscomp   0    Ever       Cigs  Nev cigs
   WEISS  16          m   50   69      Am:USA  1961  1963    CS  Prev  Emp:viscomp   0    Ever        Any   Nev any
     WEN  18          m   35   99   Asia:FarE  1982  2004    Pr   Inc     Emp:mort   1 Current       Cigs  Nev cigs


  ________________________________________________________________________________________________________________________
                                            International Evidence on Smoking and COPD, Phase 3, Analysis run on 27-SEP-10

                                                   Table 3 - C - 2 - 2

IESCOPD - Meta-analysis of ever smoking (or current if ever not available), cigarettes (or all products if cigarettes not
                                                      Any Emphysema
                                                      Most-adjusted


                        Number Exposed  Non-exposed
 REF    NRR SEX ADJ     Case    Cont    Case    Cont      RR        95.00%CI
 ANDER2 1   b   0        102      12      30      21      5.95 (  2.63-  13.48)
 AUERBA 2   m   1        816       -      20       -    489.54 (211.74-1131.81)
*BEST   20  m   1         52       -       3       -      8.47 (  2.65-  27.11)
*DONTA2 13  m   0         11     333       4     127      1.05 (  0.34-   3.23)
 ENRIGH 9   m   0        113    1390      22     694      2.56 (  1.61-   4.09)
 ENRIGH 12  f   0         75    1182      26    1617      3.95 (  2.51-   6.20)
 Subtotal ENRIGH                                          3.20 (  2.31-   4.43)
 GULSVI 4   m   1         40       -       4       -      2.37 (  0.84-   6.66)
 GULSVI 6   f   1         28       -      16       -      1.82 (  0.98-   3.38)
 Subtotal GULSVI                                          1.95 (  1.15-   3.32)
*HAMMO2 7   m   1        369       -      20       -      8.64 (  5.51-  13.55)
*HAMMO2 16  f   1         24       -      21       -      6.44 (  3.57-  11.63)
 Subtotal HAMMO2                                          7.76 (  5.42-  11.09)
 HARDIE 15  m   1         48       -       2       -     13.38 (  3.14-  56.98)
 HARDIE 18  f   1         11       -      11       -      2.55 (  1.18-   5.48)
 Subtotal HARDIE                                          3.67 (  1.86-   7.23)
*HIRAYA 1   m   1        156       -      17       -      2.22 (  1.34-   3.67)
*HIRAYA 2   f   1         14       -      42       -      2.72 (  1.48-   5.00)
 Subtotal HIRAYA                                          2.41 (  1.64-   3.55)
 HO     12  b   3          -       -       -       -      2.87 (  1.42-   5.81)
 HOZAWA 9   b   0        185    8775      13    6533     10.59 (  6.03-  18.61)
 HUHTI1 69  m   0         64     467       1     121     16.58 (  2.28- 120.74)
 HUHTI1 157 f   1          3       -      16       -      1.97 (  0.54-   7.17)
 Subtotal HUHTI1                                          3.72 (  1.26-  10.98)
*KAHN2  81  m   1        391       -      18       -     11.81 (  7.36-  18.97)
 LAVECC 30  b   6        789       -     595       -      2.05 (  1.81-   2.33)
 LEBOWI 36  b   3         31       -       5       -      4.86 (  1.86-  12.74)
 MILLER 9   m   1          6       -       0       -      6.56 (  0.37- 116.09)
 MILLER 12  f   1          2       -       1       -      6.03 (  0.12- 308.75)
 Subtotal MILLER                                          6.37 (  0.63-  64.77)
 NAWA   1   m   0        654    4086      32    1372      6.86 (  4.79-   9.84)
 OMORI  6   m   1        135       -       3       -      9.73 (  3.18-  29.82)
 PRATT  1   m   0        178     246      15      82      3.96 (  2.21-   7.09)
 RYDER  3   b   1         80       -      21       -     11.69 (  5.16-  26.51)
*SILVA  9   b   6          -       -       -       -      2.10 (  1.09-   4.06)
 SUTINE 1   b   0         63      28      28      45      3.62 (  1.89-   6.92)
*VIKGRE 1   m   0          2      40       0      26      3.27~(  0.16-  65.49)
 WANG2  1   b   0        188    3408      42    4209      5.53 (  3.94-   7.75)
 WEISS  16  m   0         28     223       1      35      4.39 (  0.58-  33.34)
*WEN    18  m   1         12       -       -       -      1.12 (  0.44-   2.88)
Partial Totals          4670   20190    1029   14882
*prospective study                                        ~ With 0.5 adjustment for zero


 REF    NRR SEX ADJ             Ys       Ws       Qs       Ps
 ANDER2 1   b   0              1.78     5.74     1.91       0.00
 AUERBA 2   m   1              6.19     5.47   135.98       0.00
*BEST   20  m   1              2.14     2.84     2.45       0.00
*DONTA2 13  m   0              0.05     3.03     4.07       0.93
 ENRIGH 9   m   0              0.94    17.71     1.25       0.00
 ENRIGH 12  f   0              1.37    18.78     0.52       0.00
 Subtotal ENRIGH               1.16    36.49     1.76
 GULSVI 4   m   1              0.86     3.58     0.42       0.10
 GULSVI 6   f   1              0.60    10.02     3.71       0.06
 Subtotal GULSVI               0.67    13.61     4.13
*HAMMO2 7   m   1              2.16    18.98    17.10       0.00
*HAMMO2 16  f   1              1.86    11.02     4.73       0.00
 Subtotal HAMMO2               2.05    29.99    21.83
 HARDIE 15  m   1              2.59     1.83     3.52       0.00
 HARDIE 18  f   1              0.94     6.52     0.48       0.02
 Subtotal HARDIE               1.30     8.35     4.00
*HIRAYA 1   m   1              0.80    15.14     2.54       0.00
*HIRAYA 2   f   1              1.00    10.37     0.44       0.00
 Subtotal HIRAYA               0.88    25.51     2.98
 HO     12  b   3              1.05     7.74     0.18       0.00
 HOZAWA 9   b   0              2.36    12.11    16.10       0.00
 HUHTI1 69  m   0              2.81     0.97     2.50       0.01
 HUHTI1 157 f   1              0.68     2.30     0.64       0.30
 Subtotal HUHTI1               1.31     3.27     3.14
*KAHN2  81  m   1              2.47    17.14    27.29       0.00
 LAVECC 30  b   6              0.72   240.93    57.68       0.00
  ________________________________________________________________________________________________________________________
                                            International Evidence on Smoking and COPD, Phase 3, Analysis run on 27-SEP-10

                                                   Table 3 - C - 2 - 2

IESCOPD - Meta-analysis of ever smoking (or current if ever not available), cigarettes (or all products if cigarettes not
                                                      Any Emphysema
                                                      Most-adjusted


 REF    NRR SEX ADJ             Ys       Ws       Qs       Ps
 LEBOWI 36  b   3              1.58     4.15     0.58       0.00
 MILLER 9   m   1              1.88     0.46     0.21       0.20
 MILLER 12  f   1              1.80     0.25     0.09       0.37
 Subtotal MILLER               1.85     0.71     0.30
 NAWA   1   m   0              1.93    29.63    15.31       0.00
 OMORI  6   m   1              2.28     3.07     3.50       0.00
 PRATT  1   m   0              1.38    11.29     0.32       0.00
 RYDER  3   b   1              2.46     5.74     8.99       0.00
*SILVA  9   b   6              0.74     8.89     1.92       0.03
 SUTINE 1   b   0              1.29     9.13     0.06       0.00
*VIKGRE 1   m   0              1.19     0.43     0.00       0.44
 WANG2  1   b   0              1.71    33.72     8.52       0.00
 WEISS  16  m   0              1.48     0.94     0.07       0.15
*WEN    18  m   1              0.11     4.35     5.21       0.81

                       N       33
                      NS       26


                      Wt   524.25
                 Het Chi   328.29
                 Het  df       32
                 Het  P       ***
               Fixed  RR     3.34
                     RRl     3.07
                     RRu     3.64
                      P       +++
              Random  RR     4.88
                     RRl     3.48
                     RRu     6.83
                      P       +++
               Asymm  P         *


  ________________________________________________________________________________________________________________________
                                            International Evidence on Smoking and COPD, Phase 3, Analysis run on 27-SEP-10

                                                   Table 3 - C - 2 - 3

IESCOPD - Meta-analysis of ever smoking (or current if ever not available), cigarettes (or all products if cigarettes not
                                                      Any Emphysema
                                                      Most-adjusted


                       N       33
                      NS       26


                      Wt   524.25
                 Het Chi   328.29
                 Het  df       32
                 Het  P       ***
               Fixed  RR     3.34
                     RRl     3.07
                     RRu     3.64
                      P       +++
              Random  RR     4.88
                     RRl     3.48
                     RRu     6.83
                      P       +++
               Asymm  P         *

                                   Sex
                             both      male    female     Total


                       N        9        17         7        33
                      NS        9        17         7        33


                      Wt   328.14    136.86     59.25    524.25
                 Het Chi    76.66    174.66     10.60    328.29
                 Het  df        8        16         6        32
                 Het  P       ***       ***      N.S.       ***
               Fixed  RR     2.62      6.01      3.30      3.34
                     RRl     2.36      5.08      2.56      3.07
                     RRu     2.92      7.11      4.26      3.64
                      P       +++       +++       +++       +++
              Random  RR     4.47      6.32      3.17      4.88
                     RRl     2.72      3.41      2.19      3.48
                     RRu     7.34     11.74      4.59      6.83
                      P       +++       +++       +++       +++
             Between Chi                                  66.37
             Between  df                                      2
             Between  P                                     ***
             Btwn(F)  P                                       *

                                        Continent
                            NAmer    Europe      Asia  oth/mult     Total


                       N       15        11         7                  33
                      NS       12         8         6                  26


                      Wt   135.76    284.48    104.01              524.25
                 Het Chi   147.51     31.68     28.57              328.29
                 Het  df       14        10         6                  32
                 Het  P       ***       ***       ***                 ***
               Fixed  RR     6.61      2.20      4.35                3.34
                     RRl     5.59      1.95      3.59                3.07
                     RRu     7.82      2.47      5.27                3.64
                      P       +++       +++       +++                 +++
              Random  RR     7.58      3.12      3.62                4.88
                     RRl     4.18      2.03      2.28                3.48
                     RRu    13.75      4.80      5.74                6.83
                      P       +++       +++       +++                 +++
             Between Chi                                           120.53
             Between  df                                                2
             Between  P                                               ***
             Btwn(F)  P                                                **


  ________________________________________________________________________________________________________________________
                                            International Evidence on Smoking and COPD, Phase 3, Analysis run on 27-SEP-10

                                                   Table 3 - C - 2 - 3

IESCOPD - Meta-analysis of ever smoking (or current if ever not available), cigarettes (or all products if cigarettes not
                                                      Any Emphysema
                                                      Most-adjusted
                               Study type
                               CC        Pr        CS     Total


                       N                 10        23        33
                      NS                  8        18        26


                      Wt              92.18    432.07    524.25
                 Het Chi              56.80    260.61    328.29
                 Het  df                  9        22        32
                 Het  P                 ***       ***       ***
               Fixed  RR               4.57      3.13      3.34
                     RRl               3.72      2.85      3.07
                     RRu               5.60      3.44      3.64
                      P                 +++       +++       +++
              Random  RR               3.67      5.60      4.88
                     RRl               2.10      3.65      3.48
                     RRu               6.40      8.58      6.83
                      P                 +++       +++       +++
             Between Chi                                  10.88
             Between  df                                      1
             Between  P                                     ***
             Btwn(F)  P                                    N.S.

                        Emp subtype
                             mort     other     Total


                       N        7        26        33
                      NS        5        21        26


                      Wt    79.84    444.42    524.25
                 Het Chi    43.17    265.54    328.29
                 Het  df        6        25        32
                 Het  P       ***       ***       ***
               Fixed  RR     5.28      3.08      3.34
                     RRl     4.24      2.81      3.07
                     RRu     6.57      3.38      3.64
                      P       +++       +++       +++
              Random  RR     4.59      4.99      4.88
                     RRl     2.48      3.35      3.48
                     RRu     8.49      7.43      6.83
                      P       +++       +++       +++
             Between Chi                        19.58
             Between  df                            1
             Between  P                           ***
             Btwn(F)  P                          N.S.

                             Smoking product
                              any      cigs  cigsonly     Total


                       N        8        23         2        33
                      NS        7        18         2        27


                      Wt   282.61    233.06      8.59    524.25
                 Het Chi    28.07    200.45      0.24    328.29
                 Het  df        7        22         1        32
                 Het  P       ***       ***      N.S.       ***
               Fixed  RR     2.24      5.31      6.69      3.34
                     RRl     1.99      4.67      3.43      3.07
                     RRu     2.51      6.03     13.05      3.64
                      P       +++       +++       +++       +++
              Random  RR     3.52      5.17      6.69      4.88
                     RRl     2.19      3.38      3.43      3.48
                     RRu     5.67      7.90     13.05      6.83
                      P       +++       +++       +++       +++
             Between Chi                                  99.53
             Between  df                                      2
             Between  P                                     ***
             Btwn(F)  P                                      **
  ________________________________________________________________________________________________________________________
                                            International Evidence on Smoking and COPD, Phase 3, Analysis run on 27-SEP-10

                                                   Table 3 - C - 2 - 3

IESCOPD - Meta-analysis of ever smoking (or current if ever not available), cigarettes (or all products if cigarettes not
                                                      Any Emphysema
                                                      Most-adjusted
                                     Unexposed group
                          nev any   nev cig  nev+ any  nev+ cig     Total


                       N       13        20                            33
                      NS       12        16                            28


                      Wt   332.78    191.47                        524.25
                 Het Chi   253.49     61.15                        328.29
                 Het  df       12        19                            32
                 Het  P       ***       ***                           ***
               Fixed  RR     2.96      4.14                          3.34
                     RRl     2.66      3.59                          3.07
                     RRu     3.29      4.77                          3.64
                      P       +++       +++                           +++
              Random  RR     7.42      3.73                          4.88
                     RRl     3.58      2.79                          3.48
                     RRu    15.36      4.98                          6.83
                      P       +++       +++                           +++
             Between Chi                                            13.66
             Between  df                                                1
             Between  P                                               ***
             Btwn(F)  P                                              N.S.

                        Unexposed group (combining nev+ with main levels)
                          nev any   nev cig     Total


                       N       13        20        33
                      NS       12        16        28


                      Wt   332.78    191.47    524.25
                 Het Chi   253.49     61.15    328.29
                 Het  df       12        19        32
                 Het  P       ***       ***       ***
               Fixed  RR     2.96      4.14      3.34
                     RRl     2.66      3.59      3.07
                     RRu     3.29      4.77      3.64
                      P       +++       +++       +++
              Random  RR     7.42      3.73      4.88
                     RRl     3.58      2.79      3.48
                     RRu    15.36      4.98      6.83
                      P       +++       +++       +++
             Between Chi                        13.66
             Between  df                            1
             Between  P                           ***
             Btwn(F)  P                          N.S.


  ________________________________________________________________________________________________________________________
                                            International Evidence on Smoking and COPD, Phase 3, Analysis run on 27-SEP-10

                                                   Table 3 - C - 2 - 4

IESCOPD - Meta-analysis of ever smoking (or current if ever not available), cigarettes (or all products if cigarettes not
                                                      Any Emphysema
                                                      Least-adjusted


     REF|NRR|X|SEX|AGEL|AGEH|     REGION|BEGYR|PUBYR|STTYP|ONSET|      DISEAS|ADJ|SMOKSTA|   PRODUCT|    UNEXP|

  ANDER2   1     b   15   99      Am:USA     *  1966    CS  Prev  Emp:viscomp   0    Ever  Cigs only   Nev any
  AUERBA   1 x   m   15   99      Am:USA  1963  1972    CS  Prev  Emp:viscomp   0 Current       Cigs   Nev any
    BEST  20     m   30   97   Am:Canada  1955  1967    Pr   Inc     Emp:mort   1    Ever  Cigs only   Nev any
  DONTA2  13     m   25   84 Eu:SE/Balkn  1960  1984    Pr   Inc Emp:diagnosd   0    Ever       Cigs  Nev cigs
  ENRIGH   9     m   65   99      Am:USA  1989  1994    CS  Prev Emp:diagnosd   0    Ever       Cigs  Nev cigs
  ENRIGH  12     f   65   99      Am:USA  1989  1994    CS  Prev Emp:diagnosd   0    Ever       Cigs  Nev cigs
  GULSVI   3 x   m   15   70    Eu:Scand  1972  1979    CS  Prev Emp:diagnosd   0    Ever        Any   Nev any
  GULSVI   5 x   f   15   70    Eu:Scand  1972  1979    CS  Prev Emp:diagnosd   0    Ever        Any   Nev any
  HAMMO2   7     m   35   99      Am:USA  1959  1966    Pr   Inc     Emp:mort   1    Ever       Cigs   Nev any
  HAMMO2  16     f   35   99      Am:USA  1959  1966    Pr   Inc     Emp:mort   1    Ever       Cigs  Nev cigs
  HARDIE  15     m   70   99    Eu:Scand  1998  2005    CS  Prev Emp:diagnosd   1    Ever       Cigs  Nev cigs
  HARDIE  18     f   70   99    Eu:Scand  1998  2005    CS  Prev Emp:diagnosd   1    Ever       Cigs  Nev cigs
  HIRAYA   1     m   40   99   Asia:FarE  1965  1982    Pr   Inc     Emp:mort   1 Current       Cigs  Nev cigs
  HIRAYA   2     f   40   99   Asia:FarE  1965  1982    Pr   Inc     Emp:mort   1 Current       Cigs  Nev cigs
      HO  12     b   70   99   Asia:FarE  1991  1999    CS  Prev Emp:self-rep   3    Ever       Cigs  Nev cigs
  HOZAWA   9     b   45   64      Am:USA  1987  2006    CS  Prev Emp:diagnosd   0    Ever       Cigs  Nev cigs
  HUHTI1  69     m   40   64    Eu:Scand  1961  1965    CS  Prev    Emp:other   0    Ever        Any   Nev any
  HUHTI1 156 x   f   40   64    Eu:Scand  1961  1965    CS  Prev    Emp:other   0    Ever       Cigs  Nev cigs
   KAHN2  81     m   31   84      Am:USA  1954  1966    Pr   Inc     Emp:mort   1    Ever       Cigs   Nev any
  LAVECC  24 x   m   15   99     Eu:West  1983  1988    CS  Prev Emp:self-rep   0    Ever        Any   Nev any
  LAVECC  27 x   f   15   99     Eu:West  1983  1988    CS  Prev Emp:self-rep   0    Ever        Any   Nev any
  LEBOWI  33 x   b   15   96      Am:USA  1972  1977    CS  Prev Emp:diagnosd   2    Ever       Cigs  Nev cigs
  MILLER   9     m   15   99      Am:USA  1978  1988    CS  Prev Emp:diagnosd   1    Ever       Cigs  Nev cigs
  MILLER  12     f   15   99      Am:USA  1978  1988    CS  Prev Emp:diagnosd   1    Ever       Cigs  Nev cigs
    NAWA   1     m   50   69   Asia:FarE  1998  2002    CS  Prev  Emp:viscomp   0    Ever       Cigs  Nev cigs
   OMORI   3 x   m   40   69   Asia:FarE     *  2006    CS  Prev  Emp:viscomp   0    Ever       Cigs  Nev cigs
   PRATT   1     m   15   99      Am:USA     *  1980    CS  Prev  Emp:viscomp   0    Ever        Any   Nev any
   RYDER   1 x   m   22   95       Eu:UK     *  1971    CS  Prev  Emp:viscomp   0    Ever        Any   Nev any
   RYDER   2 x   f   22   95       Eu:UK     *  1971    CS  Prev  Emp:viscomp   0    Ever        Any   Nev any
   SILVA   9     b   20   99      Am:USA  1972  2004    Pr   Inc    Emp:other   6    Ever       Cigs  Nev cigs
  SUTINE   1     b   10   99    Eu:Scand  1971  1978    CS  Prev  Emp:viscomp   0    Ever        Any   Nev any
  VIKGRE   1     m   61   68    Eu:Scand  1994  2004    Pr   Inc  Emp:viscomp   0 Current       Cigs  Nev cigs
   WANG2   1     b   19   92   Asia:FarE  1996  2001    CS  Prev  Emp:viscomp   0    Ever       Cigs  Nev cigs
   WEISS  16     m   50   69      Am:USA  1961  1963    CS  Prev  Emp:viscomp   0    Ever        Any   Nev any
     WEN  18     m   35   99   Asia:FarE  1982  2004    Pr   Inc     Emp:mort   1 Current       Cigs  Nev cigs


  ________________________________________________________________________________________________________________________
                                            International Evidence on Smoking and COPD, Phase 3, Analysis run on 27-SEP-10

                                                   Table 3 - C - 2 - 5

IESCOPD - Meta-analysis of ever smoking (or current if ever not available), cigarettes (or all products if cigarettes not
                                                      Any Emphysema
                                                      Least-adjusted


                        Number Exposed  Non-exposed
 REF    NRR SEX ADJ     Case    Cont    Case    Cont      RR        95.00%CI
 ANDER2 1   b   0        102      12      30      21      5.95 (  2.63-  13.48)
 AUERBA 1   m   0        816      23      20     156    276.73 (148.38- 516.09)
*BEST   20  m   1         52       -       3       -      8.47 (  2.65-  27.11)
*DONTA2 13  m   0         11     333       4     127      1.05 (  0.34-   3.23)
 ENRIGH 9   m   0        113    1390      22     694      2.56 (  1.61-   4.09)
 ENRIGH 12  f   0         75    1182      26    1617      3.95 (  2.51-   6.20)
 Subtotal ENRIGH                                          3.20 (  2.31-   4.43)
 GULSVI 3   m   0         40    5812       4    2090      3.60 (  1.29-  10.06)
 GULSVI 5   f   0         28    4913      16    4231      1.51 (  0.81-   2.79)
 Subtotal GULSVI                                          1.90 (  1.12-   3.21)
*HAMMO2 7   m   1        369       -      20       -      8.64 (  5.51-  13.55)
*HAMMO2 16  f   1         24       -      21       -      6.44 (  3.57-  11.63)
 Subtotal HAMMO2                                          7.76 (  5.42-  11.09)
 HARDIE 15  m   1         48       -       2       -     13.38 (  3.14-  56.98)
 HARDIE 18  f   1         11       -      11       -      2.55 (  1.18-   5.48)
 Subtotal HARDIE                                          3.67 (  1.86-   7.23)
*HIRAYA 1   m   1        156       -      17       -      2.22 (  1.34-   3.67)
*HIRAYA 2   f   1         14       -      42       -      2.72 (  1.48-   5.00)
 Subtotal HIRAYA                                          2.41 (  1.64-   3.55)
 HO     12  b   3          -       -       -       -      2.87 (  1.42-   5.81)
 HOZAWA 9   b   0        185    8775      13    6533     10.59 (  6.03-  18.61)
 HUHTI1 69  m   0         64     467       1     121     16.58 (  2.28- 120.74)
 HUHTI1 156 f   0          3     111      16     693      1.17 (  0.34-   4.08)
 Subtotal HUHTI1                                          2.48 (  0.86-   7.15)
*KAHN2  81  m   1        391       -      18       -     11.81 (  7.36-  18.97)
 LAVECC 24  m   0        700   19775     183   14129      2.73 (  2.32-   3.22)
 LAVECC 27  f   0         89    7548     412   29448      0.84 (  0.67-   1.06)
 Subtotal LAVECC                                          1.84 (  1.61-   2.10)
 LEBOWI 33  b   2         31       -       5       -      5.08 (  1.95-  13.23)
 MILLER 9   m   1          6       -       0       -      6.56 (  0.37- 116.09)
 MILLER 12  f   1          2       -       1       -      6.03 (  0.12- 308.75)
 Subtotal MILLER                                          6.37 (  0.63-  64.77)
 NAWA   1   m   0        654    4086      32    1372      6.86 (  4.79-   9.84)
 OMORI  3   m   0        135     380       3      97     11.49 (  3.58-  36.85)
 PRATT  1   m   0        178     246      15      82      3.96 (  2.21-   7.09)
 RYDER  1   m   0         56      14       7       9      5.14 (  1.63-  16.21)
 RYDER  2   f   0         24      12      14      43      6.14 (  2.45-  15.39)
 Subtotal RYDER                                           5.73 (  2.80-  11.74)
*SILVA  9   b   6          -       -       -       -      2.10 (  1.09-   4.06)
 SUTINE 1   b   0         63      28      28      45      3.62 (  1.89-   6.92)
*VIKGRE 1   m   0          2      40       0      26      3.27~(  0.16-  65.49)
 WANG2  1   b   0        188    3408      42    4209      5.53 (  3.94-   7.75)
 WEISS  16  m   0         28     223       1      35      4.39 (  0.58-  33.34)
*WEN    18  m   1         12       -       -       -      1.12 (  0.44-   2.88)
Partial Totals          4670   58778    1029   65778
*prospective study                                        ~ With 0.5 adjustment for zero


 REF    NRR SEX ADJ             Ys       Ws       Qs       Ps
 ANDER2 1   b   0              1.78     5.74     1.89       0.00
 AUERBA 1   m   0              5.62     9.89   192.63       0.00
*BEST   20  m   1              2.14     2.84     2.44       0.00
*DONTA2 13  m   0              0.05     3.03     4.09       0.93
 ENRIGH 9   m   0              0.94    17.71     1.27       0.00
 ENRIGH 12  f   0              1.37    18.78     0.50       0.00
 Subtotal ENRIGH               1.16    36.49     1.77
 GULSVI 3   m   0              1.28     3.63     0.02       0.01
 GULSVI 5   f   0              0.41    10.14     6.48       0.19
 Subtotal GULSVI               0.64    13.76     6.50
*HAMMO2 7   m   1              2.16    18.98    17.01       0.00
*HAMMO2 16  f   1              1.86    11.02     4.70       0.00
 Subtotal HAMMO2               2.05    29.99    21.71
 HARDIE 15  m   1              2.59     1.83     3.50       0.00
 HARDIE 18  f   1              0.94     6.52     0.49       0.02
 Subtotal HARDIE               1.30     8.35     3.99
*HIRAYA 1   m   1              0.80    15.14     2.57       0.00
*HIRAYA 2   f   1              1.00    10.37     0.45       0.00
 Subtotal HIRAYA               0.88    25.51     3.02
 HO     12  b   3              1.05     7.74     0.19       0.00
 HOZAWA 9   b   0              2.36    12.11    16.03       0.00
 HUHTI1 69  m   0              2.81     0.97     2.49       0.01
  ________________________________________________________________________________________________________________________
                                            International Evidence on Smoking and COPD, Phase 3, Analysis run on 27-SEP-10

                                                   Table 3 - C - 2 - 5

IESCOPD - Meta-analysis of ever smoking (or current if ever not available), cigarettes (or all products if cigarettes not
                                                      Any Emphysema
                                                      Least-adjusted


 REF    NRR SEX ADJ             Ys       Ws       Qs       Ps
 HUHTI1 156 f   0              0.16     2.46     2.72       0.80
 Subtotal HUHTI1               0.91     3.44     5.22
*KAHN2  81  m   1              2.47    17.14    27.18       0.00
 LAVECC 24  m   0              1.01   142.56     5.95       0.00
 LAVECC 27  f   0             -0.17    72.31   137.85       0.15
 Subtotal LAVECC               0.61   214.87   143.80
 LEBOWI 33  b   2              1.63     4.19     0.72       0.00
 MILLER 9   m   1              1.88     0.46     0.21       0.20
 MILLER 12  f   1              1.80     0.25     0.09       0.37
 Subtotal MILLER               1.85     0.71     0.30
 NAWA   1   m   0              1.93    29.63    15.21       0.00
 OMORI  3   m   0              2.44     2.83     4.29       0.00
 PRATT  1   m   0              1.38    11.29     0.31       0.00
 RYDER  1   m   0              1.64     2.91     0.53       0.01
 RYDER  2   f   0              1.82     4.55     1.67       0.00
 Subtotal RYDER                1.75     7.47     2.20
*SILVA  9   b   6              0.74     8.89     1.94       0.03
 SUTINE 1   b   0              1.29     9.13     0.05       0.00
*VIKGRE 1   m   0              1.19     0.43     0.00       0.44
 WANG2  1   b   0              1.71    33.72     8.44       0.00
 WEISS  16  m   0              1.48     0.94     0.07       0.15
*WEN    18  m   1              0.11     4.35     5.23       0.81

                       N       35
                      NS       26


                      Wt   504.47
                 Het Chi   469.22
                 Het  df       34
                 Het  P       ***
               Fixed  RR     3.35
                     RRl     3.07
                     RRu     3.66
                      P       +++
              Random  RR     4.51
                     RRl     3.13
                     RRu     6.52
                      P       +++
               Asymm  P       (*)


  ________________________________________________________________________________________________________________________
                                            International Evidence on Smoking and COPD, Phase 3, Analysis run on 27-SEP-10

                                                   Table 3 - C - 2 - 6

IESCOPD - Meta-analysis of ever smoking (or current if ever not available), cigarettes (or all products if cigarettes not
                                                      Any Emphysema
                                                      Least-adjusted


                       N       35
                      NS       26


                      Wt   504.47
                 Het Chi   469.22
                 Het  df       34
                 Het  P       ***
               Fixed  RR     3.35
                     RRl     3.07
                     RRu     3.66
                      P       +++
              Random  RR     4.51
                     RRl     3.13
                     RRu     6.52
                      P       +++
               Asymm  P       (*)

                                   Sex
                             both      male    female     Total


                       N        7        19         9        35
                      NS        7        19         9        35


                      Wt    81.52    286.57    136.38    504.47
                 Het Chi    17.35    267.83     79.67    469.22
                 Het  df        6        18         8        34
                 Het  P        **       ***       ***       ***
               Fixed  RR     4.91      4.28      1.59      3.35
                     RRl     3.95      3.81      1.35      3.07
                     RRu     6.10      4.81      1.89      3.66
                      P       +++       +++       +++       +++
              Random  RR     4.60      5.98      2.57      4.51
                     RRl     3.07      3.42      1.34      3.13
                     RRu     6.87     10.45      4.93      6.52
                      P       +++       +++        ++       +++
             Between Chi                                 104.37
             Between  df                                      2
             Between  P                                     ***
             Btwn(F)  P                                       *


  ________________________________________________________________________________________________________________________
                                            International Evidence on Smoking and COPD, Phase 3, Analysis run on 27-SEP-10

                                                   Table 3 - C - 2 - 7

IESCOPD - Meta-analysis of ever smoking (or current if ever not available), cigarettes (or all products if cigarettes not
                                                      Any Emphysema
                                 Excluded studies (and stage at which they were excluded)


1       CLARK COTTON  MEYER REMYJA RUTGER SNYDER SOBRAX     SU TAKEMU  WANG4   WEIR WHICKE ZALACA
2      ALDERS ALESSA  AMIGO ANDER1 ANDER3   BANG  BECK1  BECK2 BEDNAR BJORNS BROGGE  BROWN CERVER CHAPMA  CHEN1  CHEN2
        CHEN3  CHENG CLEMEN COATES  COCCI COLLEG  DEAN1  DEAN2  DEANE DEJONG DEMARC DETORR DICKIN  DOLL1  DOLL2 DONTA1
       DOPICO EHRLIC EKBERG ENSTRO FERRI1 FERRI2 FERRI3  FIDAN FINKLE FLETCH FORAST FOXMAN FUKUCH GEIJER GODTFR GOLDBE
       HAENSZ HARIKK HARRIS HAWTHO  HAYES HEDMAN HIGGI2 HIGGI3 HIGGI4 HIGGI6 HOLLA2 HOLLNA  HOUSE HRUBEC HUCHON HUHTI2
       HUHTI3 ITABAS JACOBS JAENDI JENSEN JINDA2 JOHANN  JOSHI JOUSI1 KACHEL KARAKA KATANC   KATO KHOURY    KIM  KIRAZ
       KLAYTO KOJIMA KOTAN1 KOTAN2 KRZYZA  KUBIK KULLER    LAI   LAM1   LAM2   LAM3 LAMBER  LANGE LANGE2 LANGHA    LEE
         LIAW LINDBE LINDST   LIU1   LIU2 LUNDB1 LUNDB2  MADOR MAGNUS MANFRE MANNI1 MANNI2 MANNI3 MARAN1 MARAN2 MARCUS
       MATHES MELLST MENEZ1 MENEZ2 MENEZ3 MENEZ4 MENEZ5 MENEZ6  MEREN  MILNE MOLLER MONTNE MUELLE NEJJAR NIEPSU NIHLEN
       NILSSO OGILVI OSWAL1 OSWAL2 PANDEY   PEAT PELKON PEREZP   PETO  PRICE   REID RENWIC RICCIO RIMING SARGEA SAWICK
       SCHWAR SHAHAB  SHARP SHIMUR   SHIN SICHLE SOBRAD SPEIZE STERLI STJERN  STROM SUADIC  TAGER TAGER2   TANG   THUN
         TODD TROISI TRUPIN TSUSHI TVERDA URRUTI VESTBO VIEGI1 VIEGI2 VINEIS VOLLM1 VOLLM2 VONHER WAGEN2   WALD WATSON
          WIG WILHEL WILSO1 WOJTYN  WOODS  WOOLF   XIAO     XU YAMAGU   YUAN ZIELI1 ZIELI2 ZIETKO   ZOIA
3      WILSO2
4        KAHN


  ________________________________________________________________________________________________________________________
                                            International Evidence on Smoking and COPD, Phase 3, Analysis run on 27-SEP-10

                                                   Table 3 - C - 2 - 8

IESCOPD - Meta-analysis of ever smoking (or current if ever not available), cigarettes (or all products if cigarettes not
                                                      Any Emphysema
                                             Potentially overlapping studies


     REF| REFGP|PRINC|                     OVERLAP|

  HOZAWA HOZAWA     1         ENRIGH/HOZAWA/HARIKK
  ENRIGH ENRIGH     1         ENRIGH/HOZAWA/HARIKK
  DONTA2 JACOBS     2  JACOBS/DONTA1/DONTA2/PELKON
  HUHTI1 HUHTI1     1                HUHTI1/HUHTI2
  HARDIE HARDIE     1         HARDIE/JOHANN/BROGGE
  HAMMO2 HAMMO2     1                HAMMO2/ENSTRO
  LEBOWI LEBOWI     1                 LEBOWI/SILVA
   SILVA  SILVA     1                 LEBOWI/SILVA
     WEN    WEN     1                     WEN/LIAW
   KAHN2   KAHN     2                   KAHN/KAHN2


  ________________________________________________________________________________________________________________________
                                            International Evidence on Smoking and COPD, Phase 3, Analysis run on 27-SEP-10

                                                    Table 3 - C - 6 -

IESCOPD - Meta-analysis of current smoking (or ever if current not available), any product (or cigarettes if all product n
                                                      Any Emphysema


This analysis is restricted to results for:
1) Eligible study on database
2) Outcome Emphysema
3) Non-dose-response data
4) Ever or current smoking
5) Results complete enough for use in meta-analysis

Within each study, results are then selected (in the following order of preference, within each sex) for:
6) SMKSTA  : current, ever
7) UNEXP   : never any, never cigarettes, other
8) PROD    : any product, cigarettes, cigarettes only
9) For overlapping studies: principal rather than subsidiary studies
and then for single sex results (m, f) in preference to results for both sexes combined (b).

Results adjusted for the most potential confounders are then chosen in Sections -1 to -3
(and those which actually differ from the adjusted results in Table 3 - C - 1 - 1 are marked 'x' in Section -1)
and results adjusted for the least confounders in Sections -4 to -6. (Those least adjusted results which
actually differ from the most adjusted are marked 'x' in column X in Section -4)

Section -7 shows excluded studies, together with the stage (as above) at which no qualifying
results were found.

Section -8 lists the potentially overlapping studies which have been included (1=principal, 2=subsidiary),
and any results which would have been included in preference except that they had data not complete enough
for use in meta-analysis. It also lists their significance (yes/no), if known.


  ________________________________________________________________________________________________________________________
                                            International Evidence on Smoking and COPD, Phase 3, Analysis run on 27-SEP-10

                                                   Table 3 - C - 6 - 1

IESCOPD - Meta-analysis of current smoking (or ever if current not available), any product (or cigarettes if all product n
                                                      Any Emphysema
                                                      Most-adjusted


     REF|NRR|Cmp3C1|SEX|AGEL|AGEH|     REGION|BEGYR|PUBYR|STTYP|ONSET|      DISEAS|ADJ|SMOKSTA|   PRODUCT|    UNEXP|

  ANDER2   1      x   b   15   99      Am:USA     *  1966    CS  Prev  Emp:viscomp   0    Ever  Cigs only   Nev any
  AUERBA   2          m   15   99      Am:USA  1963  1972    CS  Prev  Emp:viscomp   1 Current       Cigs   Nev any
    BEST  18          m   30   97   Am:Canada  1955  1967    Pr   Inc     Emp:mort   1 Current  Cigs only   Nev any
  DONTA2  11          m   25   84 Eu:SE/Balkn  1960  1984    Pr   Inc Emp:diagnosd   0 Current       Cigs  Nev cigs
  ENRIGH   7          m   65   99      Am:USA  1989  1994    CS  Prev Emp:diagnosd   0 Current       Cigs  Nev cigs
  ENRIGH  10          f   65   99      Am:USA  1989  1994    CS  Prev Emp:diagnosd   0 Current       Cigs  Nev cigs
  GULSVI   4      x   m   15   70    Eu:Scand  1972  1979    CS  Prev Emp:diagnosd   1    Ever        Any   Nev any
  GULSVI   6      x   f   15   70    Eu:Scand  1972  1979    CS  Prev Emp:diagnosd   1    Ever        Any   Nev any
  HAMMO2   7      x   m   35   99      Am:USA  1959  1966    Pr   Inc     Emp:mort   1    Ever       Cigs   Nev any
  HAMMO2   8      x   f   35   99      Am:USA  1959  1966    Pr   Inc     Emp:mort   1    Ever        Any   Nev any
  HARDIE  13          m   70   99    Eu:Scand  1998  2005    CS  Prev Emp:diagnosd   1 Current       Cigs  Nev cigs
  HARDIE  16          f   70   99    Eu:Scand  1998  2005    CS  Prev Emp:diagnosd   1 Current       Cigs  Nev cigs
  HIRAYA   1          m   40   99   Asia:FarE  1965  1982    Pr   Inc     Emp:mort   1 Current       Cigs  Nev cigs
  HIRAYA   2          f   40   99   Asia:FarE  1965  1982    Pr   Inc     Emp:mort   1 Current       Cigs  Nev cigs
      HO   9          b   70   99   Asia:FarE  1991  1999    CS  Prev Emp:self-rep   3 Current       Cigs  Nev cigs
  HOZAWA   9      x   b   45   64      Am:USA  1987  2006    CS  Prev Emp:diagnosd   0    Ever       Cigs  Nev cigs
  HUHTI1  67          m   40   64    Eu:Scand  1961  1965    CS  Prev    Emp:other   0 Current        Any   Nev any
  HUHTI1 142          f   40   64    Eu:Scand  1961  1965    CS  Prev    Emp:other   0 Current        Any   Nev any
   KAHN2  46          m   31   84      Am:USA  1954  1966    Pr   Inc     Emp:mort   1 Current        Any   Nev any
  LAVECC  28          b   15   99     Eu:West  1983  1988    CS  Prev Emp:self-rep   6 Current        Any   Nev any
  LEBOWI  34          b   15   96      Am:USA  1972  1977    CS  Prev Emp:diagnosd   3 Current       Cigs  Nev cigs
  MILLER   7          m   15   99      Am:USA  1978  1988    CS  Prev Emp:diagnosd   1 Current       Cigs  Nev cigs
  MILLER  10          f   15   99      Am:USA  1978  1988    CS  Prev Emp:diagnosd   1 Current       Cigs  Nev cigs
    NAWA   1      x   m   50   69   Asia:FarE  1998  2002    CS  Prev  Emp:viscomp   0    Ever       Cigs  Nev cigs
   OMORI   4          m   40   69   Asia:FarE     *  2006    CS  Prev  Emp:viscomp   1 Current       Cigs  Nev cigs
   PRATT   1      x   m   15   99      Am:USA     *  1980    CS  Prev  Emp:viscomp   0    Ever        Any   Nev any
   RYDER   3      x   b   22   95       Eu:UK     *  1971    CS  Prev  Emp:viscomp   1    Ever        Any   Nev any
   SILVA   7          b   20   99      Am:USA  1972  2004    Pr   Inc    Emp:other   6 Current       Cigs  Nev cigs
  SUTINE   1      x   b   10   99    Eu:Scand  1971  1978    CS  Prev  Emp:viscomp   0    Ever        Any   Nev any
  VIKGRE   1          m   61   68    Eu:Scand  1994  2004    Pr   Inc  Emp:viscomp   0 Current       Cigs  Nev cigs
   WANG2   1      x   b   19   92   Asia:FarE  1996  2001    CS  Prev  Emp:viscomp   0    Ever       Cigs  Nev cigs
   WEISS  14          m   50   69      Am:USA  1961  1963    CS  Prev  Emp:viscomp   0 Current        Any   Nev any
     WEN  18          m   35   99   Asia:FarE  1982  2004    Pr   Inc     Emp:mort   1 Current       Cigs  Nev cigs


  ________________________________________________________________________________________________________________________
                                            International Evidence on Smoking and COPD, Phase 3, Analysis run on 27-SEP-10

                                                   Table 3 - C - 6 - 2

IESCOPD - Meta-analysis of current smoking (or ever if current not available), any product (or cigarettes if all product n
                                                      Any Emphysema
                                                      Most-adjusted


                        Number Exposed  Non-exposed
 REF    NRR SEX ADJ     Case    Cont    Case    Cont      RR        95.00%CI
 ANDER2 1   b   0        102      12      30      21      5.95 (  2.63-  13.48)
 AUERBA 2   m   1        816       -      20       -    489.54 (211.74-1131.81)
*BEST   18  m   1         40       -       3       -      7.72 (  2.39-  24.95)
*DONTA2 11  m   0         10     275       4     127      1.15 (  0.37-   3.61)
 ENRIGH 7   m   0         32     199      22     694      5.07 (  2.88-   8.93)
 ENRIGH 10  f   0         18     364      26    1617      3.08 (  1.67-   5.67)
 Subtotal ENRIGH                                          4.03 (  2.66-   6.10)
 GULSVI 4   m   1         40       -       4       -      2.37 (  0.84-   6.66)
 GULSVI 6   f   1         28       -      16       -      1.82 (  0.98-   3.38)
 Subtotal GULSVI                                          1.95 (  1.15-   3.32)
*HAMMO2 7   m   1        369       -      20       -      8.64 (  5.51-  13.55)
*HAMMO2 8   f   1         24       -      21       -      6.44 (  3.57-  11.63)
 Subtotal HAMMO2                                          7.76 (  5.42-  11.09)
 HARDIE 13  m   1         11       -       2       -     15.04 (  3.40-  66.58)
 HARDIE 16  f   1          5       -      11       -      3.59 (  1.38-   9.29)
 Subtotal HARDIE                                          5.45 (  2.44-  12.16)
*HIRAYA 1   m   1        156       -      17       -      2.22 (  1.34-   3.67)
*HIRAYA 2   f   1         14       -      42       -      2.72 (  1.48-   5.00)
 Subtotal HIRAYA                                          2.41 (  1.64-   3.55)
 HO     9   b   3          -       -       -       -      2.30 (  0.94-   5.62)
 HOZAWA 9   b   0        185    8775      13    6533     10.59 (  6.03-  18.61)
 HUHTI1 67  m   0         53     337       1     121     19.03 (  2.60- 139.11)
 HUHTI1 142 f   0          1      83      16     693      0.52 (  0.07-   3.99)
 Subtotal HUHTI1                                          3.28 (  0.79-  13.58)
*KAHN2  46  m   1        284       -      18       -      9.09 (  5.65-  14.64)
 LAVECC 28  b   6        437       -     595       -      1.76 (  1.52-   2.04)
 LEBOWI 34  b   3         14       -       5       -      3.82 (  1.34-  10.88)
 MILLER 7   m   1          4       -       0       -      7.21 (  0.39- 133.19)
 MILLER 10  f   1          2       -       1       -      9.07 (  0.18- 446.54)
 Subtotal MILLER                                          7.83 (  0.76-  81.05)
 NAWA   1   m   0        654    4086      32    1372      6.86 (  4.79-   9.84)
 OMORI  4   m   1        116       -       3       -     14.50 (  4.63-  45.40)
 PRATT  1   m   0        178     246      15      82      3.96 (  2.21-   7.09)
 RYDER  3   b   1         80       -      21       -     11.69 (  5.16-  26.51)
*SILVA  7   b   6          -       -       -       -      4.10 (  2.03-   8.18)
 SUTINE 1   b   0         63      28      28      45      3.62 (  1.89-   6.92)
*VIKGRE 1   m   0          2      40       0      26      3.27~(  0.16-  65.49)
 WANG2  1   b   0        188    3408      42    4209      5.53 (  3.94-   7.75)
 WEISS  14  m   0         23     160       1      35      5.03 (  0.66-  38.51)
*WEN    18  m   1         12       -       -       -      1.12 (  0.44-   2.88)
Partial Totals          3961   18013    1029   15575
*prospective study                                        ~ With 0.5 adjustment for zero


 REF    NRR SEX ADJ             Ys       Ws       Qs       Ps
 ANDER2 1   b   0              1.78     5.74     1.73       0.00
 AUERBA 2   m   1              6.19     5.47   134.46       0.00
*BEST   18  m   1              2.04     2.79     1.83       0.00
*DONTA2 11  m   0              0.14     2.95     3.52       0.80
 ENRIGH 7   m   0              1.62    12.02     1.82       0.00
 ENRIGH 10  f   0              1.12    10.27     0.13       0.00
 Subtotal ENRIGH               1.39    22.29     1.95
 GULSVI 4   m   1              0.86     3.58     0.50       0.10
 GULSVI 6   f   1              0.60    10.02     4.06       0.06
 Subtotal GULSVI               0.67    13.61     4.55
*HAMMO2 7   m   1              2.16    18.98    16.11       0.00
*HAMMO2 8   f   1              1.86    11.02     4.34       0.00
 Subtotal HAMMO2               2.05    29.99    20.45
 HARDIE 13  m   1              2.71     1.74     3.78       0.00
 HARDIE 16  f   1              1.28     4.23     0.01       0.01
 Subtotal HARDIE               1.70     5.96     3.79
*HIRAYA 1   m   1              0.80    15.14     2.90       0.00
*HIRAYA 2   f   1              1.00    10.37     0.57       0.00
 Subtotal HIRAYA               0.88    25.51     3.47
 HO     9   b   3              0.83     4.81     0.78       0.07
 HOZAWA 9   b   0              2.36    12.11    15.33       0.00
 HUHTI1 67  m   0              2.95     0.97     2.84       0.00
 HUHTI1 142 f   0             -0.65     0.93     3.30       0.53
 Subtotal HUHTI1               1.19     1.90     6.15
*KAHN2  46  m   1              2.21    16.95    16.02       0.00
 LAVECC 28  b   6              0.57   177.48    79.61       0.00
  ________________________________________________________________________________________________________________________
                                            International Evidence on Smoking and COPD, Phase 3, Analysis run on 27-SEP-10

                                                   Table 3 - C - 6 - 2

IESCOPD - Meta-analysis of current smoking (or ever if current not available), any product (or cigarettes if all product n
                                                      Any Emphysema
                                                      Most-adjusted


 REF    NRR SEX ADJ             Ys       Ws       Qs       Ps
 LEBOWI 34  b   3              1.34     3.50     0.04       0.01
 MILLER 7   m   1              1.98     0.45     0.25       0.18
 MILLER 10  f   1              2.20     0.25     0.24       0.27
 Subtotal MILLER               2.06     0.70     0.48
 NAWA   1   m   0              1.93    29.63    14.15       0.00
 OMORI  4   m   1              2.67     2.95     6.11       0.00
 PRATT  1   m   0              1.38    11.29     0.22       0.00
 RYDER  3   b   1              2.46     5.74     8.59       0.00
*SILVA  7   b   6              1.41     7.91     0.24       0.00
 SUTINE 1   b   0              1.29     9.13     0.02       0.00
*VIKGRE 1   m   0              1.19     0.43     0.00       0.44
 WANG2  1   b   0              1.71    33.72     7.60       0.00
 WEISS  14  m   0              1.62     0.93     0.13       0.12
*WEN    18  m   1              0.11     4.35     5.48       0.81

                       N       33
                      NS       26


                      Wt   437.85
                 Het Chi   336.69
                 Het  df       32
                 Het  P       ***
               Fixed  RR     3.44
                     RRl     3.13
                     RRu     3.78
                      P       +++
              Random  RR     5.05
                     RRl     3.51
                     RRu     7.25
                      P       +++
               Asymm  P         *


  ________________________________________________________________________________________________________________________
                                            International Evidence on Smoking and COPD, Phase 3, Analysis run on 27-SEP-10

                                                   Table 3 - C - 6 - 3

IESCOPD - Meta-analysis of current smoking (or ever if current not available), any product (or cigarettes if all product n
                                                      Any Emphysema
                                                      Most-adjusted


                       N       33
                      NS       26


                      Wt   437.85
                 Het Chi   336.69
                 Het  df       32
                 Het  P       ***
               Fixed  RR     3.44
                     RRl     3.13
                     RRu     3.78
                      P       +++
              Random  RR     5.05
                     RRl     3.51
                     RRu     7.25
                      P       +++
               Asymm  P         *

                                   Sex
                             both      male    female     Total


                       N        9        17         7        33
                      NS        9        17         7        33


                      Wt   260.14    130.63     47.08    437.85
                 Het Chi    90.09    157.19     12.24    336.69
                 Het  df        8        16         6        32
                 Het  P       ***       ***       (*)       ***
               Fixed  RR     2.54      6.50      3.13      3.44
                     RRl     2.25      5.47      2.36      3.13
                     RRu     2.87      7.71      4.17      3.78
                      P       +++       +++       +++       +++
              Random  RR     4.56      6.81      3.02      5.05
                     RRl     2.60      3.73      1.92      3.51
                     RRu     8.01     12.45      4.75      7.25
                      P       +++       +++       +++       +++
             Between Chi                                  77.17
             Between  df                                      2
             Between  P                                     ***
             Btwn(F)  P                                       *

                             Smoking product
                              any      cigs  cigsonly     Total


                       N       11        20         2        33
                      NS        9        16         2        27


                      Wt   248.05    181.26      8.54    437.85
                 Het Chi    84.12    170.34      0.13    336.69
                 Het  df       10        19         1        32
                 Het  P       ***       ***      N.S.       ***
               Fixed  RR     2.36      5.60      6.48      3.44
                     RRl     2.08      4.84      3.31      3.13
                     RRu     2.67      6.48     12.67      3.78
                      P       +++       +++       +++       +++
              Random  RR     3.99      5.60      6.48      5.05
                     RRl     2.30      3.45      3.31      3.51
                     RRu     6.92      9.08     12.67      7.25
                      P       +++       +++       +++       +++
             Between Chi                                  82.11
             Between  df                                      2
             Between  P                                     ***
             Btwn(F)  P                                       *


  ________________________________________________________________________________________________________________________
                                            International Evidence on Smoking and COPD, Phase 3, Analysis run on 27-SEP-10

                                                   Table 3 - C - 6 - 3

IESCOPD - Meta-analysis of current smoking (or ever if current not available), any product (or cigarettes if all product n
                                                      Any Emphysema
                                                      Most-adjusted
                           Smoking status
                             ever   current     Total


                       N       11        22        33
                      NS        9        17        26


                      Wt   150.96    286.89    437.85
                 Het Chi    31.30    242.28    336.69
                 Het  df       10        21        32
                 Het  P       ***       ***       ***
               Fixed  RR     5.80      2.61      3.44
                     RRl     4.95      2.33      3.13
                     RRu     6.81      2.93      3.78
                      P       +++       +++       +++
              Random  RR     5.51      4.87      5.05
                     RRl     4.08      2.83      3.51
                     RRu     7.43      8.41      7.25
                      P       +++       +++       +++
             Between Chi                        63.11
             Between  df                            1
             Between  P                           ***
             Btwn(F)  P                             *

                        Smoking results reported in study (Emphysema)
                             Ever   Current      Both     Total


                       N       11         5        17        33
                      NS        9         4        13        26


                      Wt   150.96     35.76    251.13    437.85
                 Het Chi    31.30    138.56     86.81    336.69
                 Het  df       10         4        16        32
                 Het  P       ***       ***       ***       ***
               Fixed  RR     5.80      4.97      2.38      3.44
                     RRl     4.95      3.58      2.11      3.13
                     RRu     6.81      6.90      2.70      3.78
                      P       +++       +++       +++       +++
              Random  RR     5.51      6.64      4.27      5.05
                     RRl     4.08      0.79      2.69      3.51
                     RRu     7.43     55.47      6.78      7.25
                      P       +++       (+)       +++       +++
             Between Chi                                  80.02
             Between  df                                      2
             Between  P                                     ***
             Btwn(F)  P                                       *


  ________________________________________________________________________________________________________________________
                                            International Evidence on Smoking and COPD, Phase 3, Analysis run on 27-SEP-10

                                                   Table 3 - C - 6 - 4

IESCOPD - Meta-analysis of current smoking (or ever if current not available), any product (or cigarettes if all product n
                                                      Any Emphysema
                                                      Least-adjusted


     REF|NRR|X|SEX|AGEL|AGEH|     REGION|BEGYR|PUBYR|STTYP|ONSET|      DISEAS|ADJ|SMOKSTA|   PRODUCT|    UNEXP|

  ANDER2   1     b   15   99      Am:USA     *  1966    CS  Prev  Emp:viscomp   0    Ever  Cigs only   Nev any
  AUERBA   1 x   m   15   99      Am:USA  1963  1972    CS  Prev  Emp:viscomp   0 Current       Cigs   Nev any
    BEST  18     m   30   97   Am:Canada  1955  1967    Pr   Inc     Emp:mort   1 Current  Cigs only   Nev any
  DONTA2  11     m   25   84 Eu:SE/Balkn  1960  1984    Pr   Inc Emp:diagnosd   0 Current       Cigs  Nev cigs
  ENRIGH   7     m   65   99      Am:USA  1989  1994    CS  Prev Emp:diagnosd   0 Current       Cigs  Nev cigs
  ENRIGH  10     f   65   99      Am:USA  1989  1994    CS  Prev Emp:diagnosd   0 Current       Cigs  Nev cigs
  GULSVI   3 x   m   15   70    Eu:Scand  1972  1979    CS  Prev Emp:diagnosd   0    Ever        Any   Nev any
  GULSVI   5 x   f   15   70    Eu:Scand  1972  1979    CS  Prev Emp:diagnosd   0    Ever        Any   Nev any
  HAMMO2   7     m   35   99      Am:USA  1959  1966    Pr   Inc     Emp:mort   1    Ever       Cigs   Nev any
  HAMMO2   8     f   35   99      Am:USA  1959  1966    Pr   Inc     Emp:mort   1    Ever        Any   Nev any
  HARDIE  13     m   70   99    Eu:Scand  1998  2005    CS  Prev Emp:diagnosd   1 Current       Cigs  Nev cigs
  HARDIE  16     f   70   99    Eu:Scand  1998  2005    CS  Prev Emp:diagnosd   1 Current       Cigs  Nev cigs
  HIRAYA   1     m   40   99   Asia:FarE  1965  1982    Pr   Inc     Emp:mort   1 Current       Cigs  Nev cigs
  HIRAYA   2     f   40   99   Asia:FarE  1965  1982    Pr   Inc     Emp:mort   1 Current       Cigs  Nev cigs
      HO   9     b   70   99   Asia:FarE  1991  1999    CS  Prev Emp:self-rep   3 Current       Cigs  Nev cigs
  HOZAWA   9     b   45   64      Am:USA  1987  2006    CS  Prev Emp:diagnosd   0    Ever       Cigs  Nev cigs
  HUHTI1  67     m   40   64    Eu:Scand  1961  1965    CS  Prev    Emp:other   0 Current        Any   Nev any
  HUHTI1 142     f   40   64    Eu:Scand  1961  1965    CS  Prev    Emp:other   0 Current        Any   Nev any
   KAHN2  46     m   31   84      Am:USA  1954  1966    Pr   Inc     Emp:mort   1 Current        Any   Nev any
  LAVECC  22 x   m   15   99     Eu:West  1983  1988    CS  Prev Emp:self-rep   0 Current        Any   Nev any
  LAVECC  25 x   f   15   99     Eu:West  1983  1988    CS  Prev Emp:self-rep   0 Current        Any   Nev any
  LEBOWI  31 x   b   15   96      Am:USA  1972  1977    CS  Prev Emp:diagnosd   2 Current       Cigs  Nev cigs
  MILLER   7     m   15   99      Am:USA  1978  1988    CS  Prev Emp:diagnosd   1 Current       Cigs  Nev cigs
  MILLER  10     f   15   99      Am:USA  1978  1988    CS  Prev Emp:diagnosd   1 Current       Cigs  Nev cigs
    NAWA   1     m   50   69   Asia:FarE  1998  2002    CS  Prev  Emp:viscomp   0    Ever       Cigs  Nev cigs
   OMORI   1 x   m   40   69   Asia:FarE     *  2006    CS  Prev  Emp:viscomp   0 Current       Cigs  Nev cigs
   PRATT   1     m   15   99      Am:USA     *  1980    CS  Prev  Emp:viscomp   0    Ever        Any   Nev any
   RYDER   1 x   m   22   95       Eu:UK     *  1971    CS  Prev  Emp:viscomp   0    Ever        Any   Nev any
   RYDER   2 x   f   22   95       Eu:UK     *  1971    CS  Prev  Emp:viscomp   0    Ever        Any   Nev any
   SILVA   7     b   20   99      Am:USA  1972  2004    Pr   Inc    Emp:other   6 Current       Cigs  Nev cigs
  SUTINE   1     b   10   99    Eu:Scand  1971  1978    CS  Prev  Emp:viscomp   0    Ever        Any   Nev any
  VIKGRE   1     m   61   68    Eu:Scand  1994  2004    Pr   Inc  Emp:viscomp   0 Current       Cigs  Nev cigs
   WANG2   1     b   19   92   Asia:FarE  1996  2001    CS  Prev  Emp:viscomp   0    Ever       Cigs  Nev cigs
   WEISS  14     m   50   69      Am:USA  1961  1963    CS  Prev  Emp:viscomp   0 Current        Any   Nev any
     WEN  18     m   35   99   Asia:FarE  1982  2004    Pr   Inc     Emp:mort   1 Current       Cigs  Nev cigs


  ________________________________________________________________________________________________________________________
                                            International Evidence on Smoking and COPD, Phase 3, Analysis run on 27-SEP-10

                                                   Table 3 - C - 6 - 5

IESCOPD - Meta-analysis of current smoking (or ever if current not available), any product (or cigarettes if all product n
                                                      Any Emphysema
                                                      Least-adjusted


                        Number Exposed  Non-exposed
 REF    NRR SEX ADJ     Case    Cont    Case    Cont      RR        95.00%CI
 ANDER2 1   b   0        102      12      30      21      5.95 (  2.63-  13.48)
 AUERBA 1   m   0        816      23      20     156    276.73 (148.38- 516.09)
*BEST   18  m   1         40       -       3       -      7.72 (  2.39-  24.95)
*DONTA2 11  m   0         10     275       4     127      1.15 (  0.37-   3.61)
 ENRIGH 7   m   0         32     199      22     694      5.07 (  2.88-   8.93)
 ENRIGH 10  f   0         18     364      26    1617      3.08 (  1.67-   5.67)
 Subtotal ENRIGH                                          4.03 (  2.66-   6.10)
 GULSVI 3   m   0         40    5812       4    2090      3.60 (  1.29-  10.06)
 GULSVI 5   f   0         28    4913      16    4231      1.51 (  0.81-   2.79)
 Subtotal GULSVI                                          1.90 (  1.12-   3.21)
*HAMMO2 7   m   1        369       -      20       -      8.64 (  5.51-  13.55)
*HAMMO2 8   f   1         24       -      21       -      6.44 (  3.57-  11.63)
 Subtotal HAMMO2                                          7.76 (  5.42-  11.09)
 HARDIE 13  m   1         11       -       2       -     15.04 (  3.40-  66.58)
 HARDIE 16  f   1          5       -      11       -      3.59 (  1.38-   9.29)
 Subtotal HARDIE                                          5.45 (  2.44-  12.16)
*HIRAYA 1   m   1        156       -      17       -      2.22 (  1.34-   3.67)
*HIRAYA 2   f   1         14       -      42       -      2.72 (  1.48-   5.00)
 Subtotal HIRAYA                                          2.41 (  1.64-   3.55)
 HO     9   b   3          -       -       -       -      2.30 (  0.94-   5.62)
 HOZAWA 9   b   0        185    8775      13    6533     10.59 (  6.03-  18.61)
 HUHTI1 67  m   0         53     337       1     121     19.03 (  2.60- 139.11)
 HUHTI1 142 f   0          1      83      16     693      0.52 (  0.07-   3.99)
 Subtotal HUHTI1                                          3.28 (  0.79-  13.58)
*KAHN2  46  m   1        284       -      18       -      9.09 (  5.65-  14.64)
 LAVECC 22  m   0        375   15285     183   14129      1.89 (  1.59-   2.26)
 LAVECC 25  f   0         62    6654     412   29448      0.67 (  0.51-   0.87)
 Subtotal LAVECC                                          1.38 (  1.19-   1.60)
 LEBOWI 31  b   2         14       -       5       -      3.84 (  1.36-  10.84)
 MILLER 7   m   1          4       -       0       -      7.21 (  0.39- 133.19)
 MILLER 10  f   1          2       -       1       -      9.07 (  0.18- 446.54)
 Subtotal MILLER                                          7.83 (  0.76-  81.05)
 NAWA   1   m   0        654    4086      32    1372      6.86 (  4.79-   9.84)
 OMORI  1   m   0        116     264       3      97     14.21 (  4.41-  45.75)
 PRATT  1   m   0        178     246      15      82      3.96 (  2.21-   7.09)
 RYDER  1   m   0         56      14       7       9      5.14 (  1.63-  16.21)
 RYDER  2   f   0         24      12      14      43      6.14 (  2.45-  15.39)
 Subtotal RYDER                                           5.73 (  2.80-  11.74)
*SILVA  7   b   6          -       -       -       -      4.10 (  2.03-   8.18)
 SUTINE 1   b   0         63      28      28      45      3.62 (  1.89-   6.92)
*VIKGRE 1   m   0          2      40       0      26      3.27~(  0.16-  65.49)
 WANG2  1   b   0        188    3408      42    4209      5.53 (  3.94-   7.75)
 WEISS  14  m   0         23     160       1      35      5.03 (  0.66-  38.51)
*WEN    18  m   1         12       -       -       -      1.12 (  0.44-   2.88)
Partial Totals          3961   50990    1029   65778
*prospective study                                        ~ With 0.5 adjustment for zero


 REF    NRR SEX ADJ             Ys       Ws       Qs       Ps
 ANDER2 1   b   0              1.78     5.74     2.16       0.00
 AUERBA 1   m   0              5.62     9.89   196.08       0.00
*BEST   18  m   1              2.04     2.79     2.13       0.00
*DONTA2 11  m   0              0.14     2.95     3.11       0.80
 ENRIGH 7   m   0              1.62    12.02     2.47       0.00
 ENRIGH 10  f   0              1.12    10.27     0.02       0.00
 Subtotal ENRIGH               1.39    22.29     2.49
 GULSVI 3   m   0              1.28     3.63     0.04       0.01
 GULSVI 5   f   0              0.41    10.14     5.86       0.19
 Subtotal GULSVI               0.64    13.76     5.90
*HAMMO2 7   m   1              2.16    18.98    18.45       0.00
*HAMMO2 8   f   1              1.86    11.02     5.28       0.00
 Subtotal HAMMO2               2.05    29.99    23.73
 HARDIE 13  m   1              2.71     1.74     4.12       0.00
 HARDIE 16  f   1              1.28     4.23     0.05       0.01
 Subtotal HARDIE               1.70     5.96     4.17
*HIRAYA 1   m   1              0.80    15.14     2.10       0.00
*HIRAYA 2   f   1              1.00    10.37     0.30       0.00
 Subtotal HIRAYA               0.88    25.51     2.40
 HO     9   b   3              0.83     4.81     0.55       0.07
 HOZAWA 9   b   0              2.36    12.11    17.14       0.00
 HUHTI1 67  m   0              2.95     0.97     3.06       0.00
  ________________________________________________________________________________________________________________________
                                            International Evidence on Smoking and COPD, Phase 3, Analysis run on 27-SEP-10

                                                   Table 3 - C - 6 - 5

IESCOPD - Meta-analysis of current smoking (or ever if current not available), any product (or cigarettes if all product n
                                                      Any Emphysema
                                                      Least-adjusted


 REF    NRR SEX ADJ             Ys       Ws       Qs       Ps
 HUHTI1 142 f   0             -0.65     0.93     3.08       0.53
 Subtotal HUHTI1               1.19     1.90     6.14
*KAHN2  46  m   1              2.21    16.95    18.22       0.00
 LAVECC 22  m   0              0.64   120.96    34.18       0.00
 LAVECC 25  f   0             -0.41    53.36   132.69       0.00
 Subtotal LAVECC               0.32   174.32   166.87
 LEBOWI 31  b   2              1.35     3.57     0.11       0.01
 MILLER 7   m   1              1.98     0.45     0.29       0.18
 MILLER 10  f   1              2.20     0.25     0.27       0.27
 Subtotal MILLER               2.06     0.70     0.56
 NAWA   1   m   0              1.93    29.63    16.92       0.00
 OMORI  1   m   0              2.65     2.81     6.18       0.00
 PRATT  1   m   0              1.38    11.29     0.47       0.00
 RYDER  1   m   0              1.64     2.91     0.64       0.01
 RYDER  2   f   0              1.82     4.55     1.89       0.00
 Subtotal RYDER                1.75     7.47     2.53
*SILVA  7   b   6              1.41     7.91     0.46       0.00
 SUTINE 1   b   0              1.29     9.13     0.12       0.00
*VIKGRE 1   m   0              1.19     0.43     0.00       0.44
 WANG2  1   b   0              1.71    33.72     9.81       0.00
 WEISS  14  m   0              1.62     0.93     0.18       0.12
*WEN    18  m   1              0.11     4.35     4.86       0.81

                       N       35
                      NS       26


                      Wt   440.91
                 Het Chi   493.31
                 Het  df       34
                 Het  P       ***
               Fixed  RR     3.22
                     RRl     2.94
                     RRu     3.54
                      P       +++
              Random  RR     4.61
                     RRl     3.09
                     RRu     6.87
                      P       +++
               Asymm  P       (*)


  ________________________________________________________________________________________________________________________
                                            International Evidence on Smoking and COPD, Phase 3, Analysis run on 27-SEP-10

                                                   Table 3 - C - 6 - 6

IESCOPD - Meta-analysis of current smoking (or ever if current not available), any product (or cigarettes if all product n
                                                      Any Emphysema
                                                      Least-adjusted


                       N       35
                      NS       26


                      Wt   440.91
                 Het Chi   493.31
                 Het  df       34
                 Het  P       ***
               Fixed  RR     3.22
                     RRl     2.94
                     RRu     3.54
                      P       +++
              Random  RR     4.61
                     RRl     3.09
                     RRu     6.87
                      P       +++
               Asymm  P       (*)

                                   Sex
                             both      male    female     Total


                       N        7        19         9        35
                      NS        7        19         9        35


                      Wt    76.98    258.82    105.11    440.91
                 Het Chi    11.52    305.19     81.49    493.31
                 Het  df        6        18         8        34
                 Het  P       (*)       ***       ***       ***
               Fixed  RR     5.29      3.86      1.44      3.22
                     RRl     4.23      3.41      1.19      2.94
                     RRu     6.61      4.36      1.75      3.54
                      P       +++       +++       +++       +++
              Random  RR     4.99      6.18      2.47      4.61
                     RRl     3.54      3.36      1.17      3.09
                     RRu     7.03     11.37      5.19      6.87
                      P       +++       +++         +       +++
             Between Chi                                  95.11
             Between  df                                      2
             Between  P                                     ***
             Btwn(F)  P                                       *


  ________________________________________________________________________________________________________________________
                                            International Evidence on Smoking and COPD, Phase 3, Analysis run on 27-SEP-10

                                                   Table 3 - C - 6 - 7

IESCOPD - Meta-analysis of current smoking (or ever if current not available), any product (or cigarettes if all product n
                                                      Any Emphysema
                                 Excluded studies (and stage at which they were excluded)


1       CLARK COTTON  MEYER REMYJA RUTGER SNYDER SOBRAX     SU TAKEMU  WANG4   WEIR WHICKE ZALACA
2      ALDERS ALESSA  AMIGO ANDER1 ANDER3   BANG  BECK1  BECK2 BEDNAR BJORNS BROGGE  BROWN CERVER CHAPMA  CHEN1  CHEN2
        CHEN3  CHENG CLEMEN COATES  COCCI COLLEG  DEAN1  DEAN2  DEANE DEJONG DEMARC DETORR DICKIN  DOLL1  DOLL2 DONTA1
       DOPICO EHRLIC EKBERG ENSTRO FERRI1 FERRI2 FERRI3  FIDAN FINKLE FLETCH FORAST FOXMAN FUKUCH GEIJER GODTFR GOLDBE
       HAENSZ HARIKK HARRIS HAWTHO  HAYES HEDMAN HIGGI2 HIGGI3 HIGGI4 HIGGI6 HOLLA2 HOLLNA  HOUSE HRUBEC HUCHON HUHTI2
       HUHTI3 ITABAS JACOBS JAENDI JENSEN JINDA2 JOHANN  JOSHI JOUSI1 KACHEL KARAKA KATANC   KATO KHOURY    KIM  KIRAZ
       KLAYTO KOJIMA KOTAN1 KOTAN2 KRZYZA  KUBIK KULLER    LAI   LAM1   LAM2   LAM3 LAMBER  LANGE LANGE2 LANGHA    LEE
         LIAW LINDBE LINDST   LIU1   LIU2 LUNDB1 LUNDB2  MADOR MAGNUS MANFRE MANNI1 MANNI2 MANNI3 MARAN1 MARAN2 MARCUS
       MATHES MELLST MENEZ1 MENEZ2 MENEZ3 MENEZ4 MENEZ5 MENEZ6  MEREN  MILNE MOLLER MONTNE MUELLE NEJJAR NIEPSU NIHLEN
       NILSSO OGILVI OSWAL1 OSWAL2 PANDEY   PEAT PELKON PEREZP   PETO  PRICE   REID RENWIC RICCIO RIMING SARGEA SAWICK
       SCHWAR SHAHAB  SHARP SHIMUR   SHIN SICHLE SOBRAD SPEIZE STERLI STJERN  STROM SUADIC  TAGER TAGER2   TANG   THUN
         TODD TROISI TRUPIN TSUSHI TVERDA URRUTI VESTBO VIEGI1 VIEGI2 VINEIS VOLLM1 VOLLM2 VONHER WAGEN2   WALD WATSON
          WIG WILHEL WILSO1 WOJTYN  WOODS  WOOLF   XIAO     XU YAMAGU   YUAN ZIELI1 ZIELI2 ZIETKO   ZOIA
3      WILSO2
4        KAHN


  ________________________________________________________________________________________________________________________
                                            International Evidence on Smoking and COPD, Phase 3, Analysis run on 27-SEP-10

                                                   Table 3 - C - 6 - 8

IESCOPD - Meta-analysis of current smoking (or ever if current not available), any product (or cigarettes if all product n
                                                      Any Emphysema
                                             Potentially overlapping studies


     REF| REFGP|PRINC|                     OVERLAP|

  HOZAWA HOZAWA     1         ENRIGH/HOZAWA/HARIKK
  ENRIGH ENRIGH     1         ENRIGH/HOZAWA/HARIKK
  DONTA2 JACOBS     2  JACOBS/DONTA1/DONTA2/PELKON
  HUHTI1 HUHTI1     1                HUHTI1/HUHTI2
  HARDIE HARDIE     1         HARDIE/JOHANN/BROGGE
  HAMMO2 HAMMO2     1                HAMMO2/ENSTRO
  LEBOWI LEBOWI     1                 LEBOWI/SILVA
   SILVA  SILVA     1                 LEBOWI/SILVA
     WEN    WEN     1                     WEN/LIAW
   KAHN2   KAHN     2                   KAHN/KAHN2


  ________________________________________________________________________________________________________________________
                                            International Evidence on Smoking and COPD, Phase 3, Analysis run on 27-SEP-10

                                                    Table 3 - C - 7 -

IESCOPD - Meta-analysis of current smoking (or ever if current not available), cigarettes (or all products if cigarettes n
                                                      Any Emphysema


This analysis is restricted to results for:
1) Eligible study on database
2) Outcome Emphysema
3) Non-dose-response data
4) Ever or current smoking
5) Results complete enough for use in meta-analysis

Within each study, results are then selected (in the following order of preference, within each sex) for:
6) SMKSTA  : current, ever
7) UNEXP   : never cigarettes, never any, other
8) PROD    : cigarettes, cigarettes only, any product
9) For overlapping studies: principal rather than subsidiary studies
and then for single sex results (m, f) in preference to results for both sexes combined (b).

Results adjusted for the most potential confounders are then chosen in Sections -1 to -3
(and those which actually differ from the adjusted results in Table 3 - C - 2 - 1 are marked 'x' in Section -1)
and results adjusted for the least confounders in Sections -4 to -6. (Those least adjusted results which
actually differ from the most adjusted are marked 'x' in column X in Section -4)

Section -7 shows excluded studies, together with the stage (as above) at which no qualifying
results were found.

Section -8 lists the potentially overlapping studies which have been included (1=principal, 2=subsidiary),
and any results which would have been included in preference except that they had data not complete enough
for use in meta-analysis. It also lists their significance (yes/no), if known.


  ________________________________________________________________________________________________________________________
                                            International Evidence on Smoking and COPD, Phase 3, Analysis run on 27-SEP-10

                                                   Table 3 - C - 7 - 1

IESCOPD - Meta-analysis of current smoking (or ever if current not available), cigarettes (or all products if cigarettes n
                                                      Any Emphysema
                                                      Most-adjusted


     REF|NRR|Cmp3C2|SEX|AGEL|AGEH|     REGION|BEGYR|PUBYR|STTYP|ONSET|      DISEAS|ADJ|SMOKSTA|   PRODUCT|    UNEXP|

  ANDER2   1      x   b   15   99      Am:USA     *  1966    CS  Prev  Emp:viscomp   0    Ever  Cigs only   Nev any
  AUERBA   2          m   15   99      Am:USA  1963  1972    CS  Prev  Emp:viscomp   1 Current       Cigs   Nev any
    BEST  18          m   30   97   Am:Canada  1955  1967    Pr   Inc     Emp:mort   1 Current  Cigs only   Nev any
  DONTA2  11          m   25   84 Eu:SE/Balkn  1960  1984    Pr   Inc Emp:diagnosd   0 Current       Cigs  Nev cigs
  ENRIGH   7          m   65   99      Am:USA  1989  1994    CS  Prev Emp:diagnosd   0 Current       Cigs  Nev cigs
  ENRIGH  10          f   65   99      Am:USA  1989  1994    CS  Prev Emp:diagnosd   0 Current       Cigs  Nev cigs
  GULSVI   4      x   m   15   70    Eu:Scand  1972  1979    CS  Prev Emp:diagnosd   1    Ever        Any   Nev any
  GULSVI   6      x   f   15   70    Eu:Scand  1972  1979    CS  Prev Emp:diagnosd   1    Ever        Any   Nev any
  HAMMO2   7      x   m   35   99      Am:USA  1959  1966    Pr   Inc     Emp:mort   1    Ever       Cigs   Nev any
  HAMMO2  16      x   f   35   99      Am:USA  1959  1966    Pr   Inc     Emp:mort   1    Ever       Cigs  Nev cigs
  HARDIE  13          m   70   99    Eu:Scand  1998  2005    CS  Prev Emp:diagnosd   1 Current       Cigs  Nev cigs
  HARDIE  16          f   70   99    Eu:Scand  1998  2005    CS  Prev Emp:diagnosd   1 Current       Cigs  Nev cigs
  HIRAYA   1          m   40   99   Asia:FarE  1965  1982    Pr   Inc     Emp:mort   1 Current       Cigs  Nev cigs
  HIRAYA   2          f   40   99   Asia:FarE  1965  1982    Pr   Inc     Emp:mort   1 Current       Cigs  Nev cigs
      HO   9          b   70   99   Asia:FarE  1991  1999    CS  Prev Emp:self-rep   3 Current       Cigs  Nev cigs
  HOZAWA   9      x   b   45   64      Am:USA  1987  2006    CS  Prev Emp:diagnosd   0    Ever       Cigs  Nev cigs
  HUHTI1  70          m   40   64    Eu:Scand  1961  1965    CS  Prev    Emp:other   0 Current       Cigs   Nev any
  HUHTI1 154          f   40   64    Eu:Scand  1961  1965    CS  Prev    Emp:other   0 Current       Cigs  Nev cigs
   KAHN2  47          m   31   84      Am:USA  1954  1966    Pr   Inc     Emp:mort   1 Current       Cigs   Nev any
  LAVECC  33          b   15   99     Eu:West  1983  1988    CS  Prev Emp:self-rep   6 Current       Cigs   Nev any
  LEBOWI  34          b   15   96      Am:USA  1972  1977    CS  Prev Emp:diagnosd   3 Current       Cigs  Nev cigs
  MILLER   7          m   15   99      Am:USA  1978  1988    CS  Prev Emp:diagnosd   1 Current       Cigs  Nev cigs
  MILLER  10          f   15   99      Am:USA  1978  1988    CS  Prev Emp:diagnosd   1 Current       Cigs  Nev cigs
    NAWA   1      x   m   50   69   Asia:FarE  1998  2002    CS  Prev  Emp:viscomp   0    Ever       Cigs  Nev cigs
   OMORI   4          m   40   69   Asia:FarE     *  2006    CS  Prev  Emp:viscomp   1 Current       Cigs  Nev cigs
   PRATT   1      x   m   15   99      Am:USA     *  1980    CS  Prev  Emp:viscomp   0    Ever        Any   Nev any
   RYDER   3      x   b   22   95       Eu:UK     *  1971    CS  Prev  Emp:viscomp   1    Ever        Any   Nev any
   SILVA   7          b   20   99      Am:USA  1972  2004    Pr   Inc    Emp:other   6 Current       Cigs  Nev cigs
  SUTINE   1      x   b   10   99    Eu:Scand  1971  1978    CS  Prev  Emp:viscomp   0    Ever        Any   Nev any
  VIKGRE   1          m   61   68    Eu:Scand  1994  2004    Pr   Inc  Emp:viscomp   0 Current       Cigs  Nev cigs
   WANG2   1      x   b   19   92   Asia:FarE  1996  2001    CS  Prev  Emp:viscomp   0    Ever       Cigs  Nev cigs
   WEISS  17          m   50   69      Am:USA  1961  1963    CS  Prev  Emp:viscomp   0 Current       Cigs   Nev any
     WEN  18          m   35   99   Asia:FarE  1982  2004    Pr   Inc     Emp:mort   1 Current       Cigs  Nev cigs


  ________________________________________________________________________________________________________________________
                                            International Evidence on Smoking and COPD, Phase 3, Analysis run on 27-SEP-10

                                                   Table 3 - C - 7 - 2

IESCOPD - Meta-analysis of current smoking (or ever if current not available), cigarettes (or all products if cigarettes n
                                                      Any Emphysema
                                                      Most-adjusted


                        Number Exposed  Non-exposed
 REF    NRR SEX ADJ     Case    Cont    Case    Cont      RR        95.00%CI
 ANDER2 1   b   0        102      12      30      21      5.95 (  2.63-  13.48)
 AUERBA 2   m   1        816       -      20       -    489.54 (211.74-1131.81)
*BEST   18  m   1         40       -       3       -      7.72 (  2.39-  24.95)
*DONTA2 11  m   0         10     275       4     127      1.15 (  0.37-   3.61)
 ENRIGH 7   m   0         32     199      22     694      5.07 (  2.88-   8.93)
 ENRIGH 10  f   0         18     364      26    1617      3.08 (  1.67-   5.67)
 Subtotal ENRIGH                                          4.03 (  2.66-   6.10)
 GULSVI 4   m   1         40       -       4       -      2.37 (  0.84-   6.66)
 GULSVI 6   f   1         28       -      16       -      1.82 (  0.98-   3.38)
 Subtotal GULSVI                                          1.95 (  1.15-   3.32)
*HAMMO2 7   m   1        369       -      20       -      8.64 (  5.51-  13.55)
*HAMMO2 16  f   1         24       -      21       -      6.44 (  3.57-  11.63)
 Subtotal HAMMO2                                          7.76 (  5.42-  11.09)
 HARDIE 13  m   1         11       -       2       -     15.04 (  3.40-  66.58)
 HARDIE 16  f   1          5       -      11       -      3.59 (  1.38-   9.29)
 Subtotal HARDIE                                          5.45 (  2.44-  12.16)
*HIRAYA 1   m   1        156       -      17       -      2.22 (  1.34-   3.67)
*HIRAYA 2   f   1         14       -      42       -      2.72 (  1.48-   5.00)
 Subtotal HIRAYA                                          2.41 (  1.64-   3.55)
 HO     9   b   3          -       -       -       -      2.30 (  0.94-   5.62)
 HOZAWA 9   b   0        185    8775      13    6533     10.59 (  6.03-  18.61)
 HUHTI1 70  m   0         53     331       1     121     19.37 (  2.65- 141.64)
 HUHTI1 154 f   0          1      83      16     693      0.52 (  0.07-   3.99)
 Subtotal HUHTI1                                          3.31 (  0.80-  13.71)
*KAHN2  47  m   1        272       -      18       -     12.18 (  7.56-  19.62)
 LAVECC 33  b   6        432       -     595       -      1.81 (  1.57-   2.10)
 LEBOWI 34  b   3         14       -       5       -      3.82 (  1.34-  10.88)
 MILLER 7   m   1          4       -       0       -      7.21 (  0.39- 133.19)
 MILLER 10  f   1          2       -       1       -      9.07 (  0.18- 446.54)
 Subtotal MILLER                                          7.83 (  0.76-  81.05)
 NAWA   1   m   0        654    4086      32    1372      6.86 (  4.79-   9.84)
 OMORI  4   m   1        116       -       3       -     14.50 (  4.63-  45.40)
 PRATT  1   m   0        178     246      15      82      3.96 (  2.21-   7.09)
 RYDER  3   b   1         80       -      21       -     11.69 (  5.16-  26.51)
*SILVA  7   b   6          -       -       -       -      4.10 (  2.03-   8.18)
 SUTINE 1   b   0         63      28      28      45      3.62 (  1.89-   6.92)
*VIKGRE 1   m   0          2      40       0      26      3.27~(  0.16-  65.49)
 WANG2  1   b   0        188    3408      42    4209      5.53 (  3.94-   7.75)
 WEISS  17  m   0         22     124       1      35      6.21 (  0.81-  47.70)
*WEN    18  m   1         12       -       -       -      1.12 (  0.44-   2.88)
Partial Totals          3943   17971    1029   15575
*prospective study                                        ~ With 0.5 adjustment for zero


 REF    NRR SEX ADJ             Ys       Ws       Qs       Ps
 ANDER2 1   b   0              1.78     5.74     1.62       0.00
 AUERBA 2   m   1              6.19     5.47   133.55       0.00
*BEST   18  m   1              2.04     2.79     1.75       0.00
*DONTA2 11  m   0              0.14     2.95     3.63       0.80
 ENRIGH 7   m   0              1.62    12.02     1.66       0.00
 ENRIGH 10  f   0              1.12    10.27     0.17       0.00
 Subtotal ENRIGH               1.39    22.29     1.83
 GULSVI 4   m   1              0.86     3.58     0.54       0.10
 GULSVI 6   f   1              0.60    10.02     4.27       0.06
 Subtotal GULSVI               0.67    13.61     4.82
*HAMMO2 7   m   1              2.16    18.98    15.53       0.00
*HAMMO2 16  f   1              1.86    11.02     4.11       0.00
 Subtotal HAMMO2               2.05    29.99    19.64
 HARDIE 13  m   1              2.71     1.74     3.70       0.00
 HARDIE 16  f   1              1.28     4.23     0.00       0.01
 Subtotal HARDIE               1.70     5.96     3.70
*HIRAYA 1   m   1              0.80    15.14     3.12       0.00
*HIRAYA 2   f   1              1.00    10.37     0.65       0.00
 Subtotal HIRAYA               0.88    25.51     3.78
 HO     9   b   3              0.83     4.81     0.84       0.07
 HOZAWA 9   b   0              2.36    12.11    14.88       0.00
 HUHTI1 70  m   0              2.96     0.97     2.85       0.00
 HUHTI1 154 f   0             -0.65     0.93     3.36       0.53
 Subtotal HUHTI1               1.20     1.90     6.21
*KAHN2  47  m   1              2.50    16.89    26.31       0.00
 LAVECC 33  b   6              0.59   181.63    78.75       0.00
  ________________________________________________________________________________________________________________________
                                            International Evidence on Smoking and COPD, Phase 3, Analysis run on 27-SEP-10

                                                   Table 3 - C - 7 - 2

IESCOPD - Meta-analysis of current smoking (or ever if current not available), cigarettes (or all products if cigarettes n
                                                      Any Emphysema
                                                      Most-adjusted


 REF    NRR SEX ADJ             Ys       Ws       Qs       Ps
 LEBOWI 34  b   3              1.34     3.50     0.03       0.01
 MILLER 7   m   1              1.98     0.45     0.24       0.18
 MILLER 10  f   1              2.20     0.25     0.23       0.27
 Subtotal MILLER               2.06     0.70     0.46
 NAWA   1   m   0              1.93    29.63    13.47       0.00
 OMORI  4   m   1              2.67     2.95     5.96       0.00
 PRATT  1   m   0              1.38    11.29     0.17       0.00
 RYDER  3   b   1              2.46     5.74     8.36       0.00
*SILVA  7   b   6              1.41     7.91     0.20       0.00
 SUTINE 1   b   0              1.29     9.13     0.01       0.00
*VIKGRE 1   m   0              1.19     0.43     0.00       0.44
 WANG2  1   b   0              1.71    33.72     7.07       0.00
 WEISS  17  m   0              1.83     0.92     0.30       0.08
*WEN    18  m   1              0.11     4.35     5.64       0.81

                       N       33
                      NS       26


                      Wt   441.93
                 Het Chi   343.00
                 Het  df       32
                 Het  P       ***
               Fixed  RR     3.50
                     RRl     3.19
                     RRu     3.84
                      P       +++
              Random  RR     5.13
                     RRl     3.56
                     RRu     7.39
                      P       +++
               Asymm  P         *


  ________________________________________________________________________________________________________________________
                                            International Evidence on Smoking and COPD, Phase 3, Analysis run on 27-SEP-10

                                                   Table 3 - C - 7 - 3

IESCOPD - Meta-analysis of current smoking (or ever if current not available), cigarettes (or all products if cigarettes n
                                                      Any Emphysema
                                                      Most-adjusted


                       N       33
                      NS       26


                      Wt   441.93
                 Het Chi   343.00
                 Het  df       32
                 Het  P       ***
               Fixed  RR     3.50
                     RRl     3.19
                     RRu     3.84
                      P       +++
              Random  RR     5.13
                     RRl     3.56
                     RRu     7.39
                      P       +++
               Asymm  P         *

                                   Sex
                             both      male    female     Total


                       N        9        17         7        33
                      NS        9        17         7        33


                      Wt   264.28    130.57     47.08    441.93
                 Het Chi    87.01    161.73     12.24    343.00
                 Het  df        8        16         6        32
                 Het  P       ***       ***       (*)       ***
               Fixed  RR     2.57      6.76      3.13      3.50
                     RRl     2.28      5.69      2.36      3.19
                     RRu     2.90      8.02      4.17      3.84
                      P       +++       +++       +++       +++
              Random  RR     4.58      7.03      3.02      5.13
                     RRl     2.64      3.82      1.92      3.56
                     RRu     7.95     12.95      4.75      7.39
                      P       +++       +++       +++       +++
             Between Chi                                  82.03
             Between  df                                      2
             Between  P                                     ***
             Btwn(F)  P                                       *

                             Smoking product
                              any      cigs  cigsonly     Total


                       N        5        26         2        33
                      NS        4        20         2        26


                      Wt    39.77    393.63      8.54    441.93
                 Het Chi    13.34    326.18      0.13    343.00
                 Het  df        4        25         1        32
                 Het  P        **       ***      N.S.       ***
               Fixed  RR     3.56      3.44      6.48      3.50
                     RRl     2.61      3.12      3.31      3.19
                     RRu     4.85      3.80     12.67      3.84
                      P       +++       +++       +++       +++
              Random  RR     3.70      5.39      6.48      5.13
                     RRl     2.06      3.47      3.31      3.56
                     RRu     6.63      8.39     12.67      7.39
                      P       +++       +++       +++       +++
             Between Chi                                   3.35
             Between  df                                      2
             Between  P                                    N.S.
             Btwn(F)  P                                    N.S.


  ________________________________________________________________________________________________________________________
                                            International Evidence on Smoking and COPD, Phase 3, Analysis run on 27-SEP-10

                                                   Table 3 - C - 7 - 3

IESCOPD - Meta-analysis of current smoking (or ever if current not available), cigarettes (or all products if cigarettes n
                                                      Any Emphysema
                                                      Most-adjusted
                           Smoking status
                             ever   current     Total


                       N       11        22        33
                      NS        9        17        26


                      Wt   150.96    290.98    441.93
                 Het Chi    31.30    252.86    343.00
                 Het  df       10        21        32
                 Het  P       ***       ***       ***
               Fixed  RR     5.80      2.69      3.50
                     RRl     4.95      2.40      3.19
                     RRu     6.81      3.02      3.84
                      P       +++       +++       +++
              Random  RR     5.51      5.00      5.13
                     RRl     4.08      2.87      3.56
                     RRu     7.43      8.72      7.39
                      P       +++       +++       +++
             Between Chi                        58.84
             Between  df                            1
             Between  P                           ***
             Btwn(F)  P                             *

                        Smoking results reported in study (Emphysema)
                             Ever   Current      Both     Total


                       N       11         5        17        33
                      NS        9         4        13        26


                      Wt   150.96     35.76    255.22    441.93
                 Het Chi    31.30    138.56     98.93    343.00
                 Het  df       10         4        16        32
                 Het  P       ***       ***       ***       ***
               Fixed  RR     5.80      4.97      2.47      3.50
                     RRl     4.95      3.58      2.18      3.19
                     RRu     6.81      6.90      2.79      3.84
                      P       +++       +++       +++       +++
              Random  RR     5.51      6.64      4.45      5.13
                     RRl     4.08      0.79      2.73      3.56
                     RRu     7.43     55.47      7.25      7.39
                      P       +++       (+)       +++       +++
             Between Chi                                  74.21
             Between  df                                      2
             Between  P                                     ***
             Btwn(F)  P                                       *


  ________________________________________________________________________________________________________________________
                                            International Evidence on Smoking and COPD, Phase 3, Analysis run on 27-SEP-10

                                                   Table 3 - C - 7 - 4

IESCOPD - Meta-analysis of current smoking (or ever if current not available), cigarettes (or all products if cigarettes n
                                                      Any Emphysema
                                                      Least-adjusted


     REF|NRR|X|SEX|AGEL|AGEH|     REGION|BEGYR|PUBYR|STTYP|ONSET|      DISEAS|ADJ|SMOKSTA|   PRODUCT|    UNEXP|

  ANDER2   1     b   15   99      Am:USA     *  1966    CS  Prev  Emp:viscomp   0    Ever  Cigs only   Nev any
  AUERBA   1 x   m   15   99      Am:USA  1963  1972    CS  Prev  Emp:viscomp   0 Current       Cigs   Nev any
    BEST  18     m   30   97   Am:Canada  1955  1967    Pr   Inc     Emp:mort   1 Current  Cigs only   Nev any
  DONTA2  11     m   25   84 Eu:SE/Balkn  1960  1984    Pr   Inc Emp:diagnosd   0 Current       Cigs  Nev cigs
  ENRIGH   7     m   65   99      Am:USA  1989  1994    CS  Prev Emp:diagnosd   0 Current       Cigs  Nev cigs
  ENRIGH  10     f   65   99      Am:USA  1989  1994    CS  Prev Emp:diagnosd   0 Current       Cigs  Nev cigs
  GULSVI   3 x   m   15   70    Eu:Scand  1972  1979    CS  Prev Emp:diagnosd   0    Ever        Any   Nev any
  GULSVI   5 x   f   15   70    Eu:Scand  1972  1979    CS  Prev Emp:diagnosd   0    Ever        Any   Nev any
  HAMMO2   7     m   35   99      Am:USA  1959  1966    Pr   Inc     Emp:mort   1    Ever       Cigs   Nev any
  HAMMO2  16     f   35   99      Am:USA  1959  1966    Pr   Inc     Emp:mort   1    Ever       Cigs  Nev cigs
  HARDIE  13     m   70   99    Eu:Scand  1998  2005    CS  Prev Emp:diagnosd   1 Current       Cigs  Nev cigs
  HARDIE  16     f   70   99    Eu:Scand  1998  2005    CS  Prev Emp:diagnosd   1 Current       Cigs  Nev cigs
  HIRAYA   1     m   40   99   Asia:FarE  1965  1982    Pr   Inc     Emp:mort   1 Current       Cigs  Nev cigs
  HIRAYA   2     f   40   99   Asia:FarE  1965  1982    Pr   Inc     Emp:mort   1 Current       Cigs  Nev cigs
      HO   9     b   70   99   Asia:FarE  1991  1999    CS  Prev Emp:self-rep   3 Current       Cigs  Nev cigs
  HOZAWA   9     b   45   64      Am:USA  1987  2006    CS  Prev Emp:diagnosd   0    Ever       Cigs  Nev cigs
  HUHTI1  70     m   40   64    Eu:Scand  1961  1965    CS  Prev    Emp:other   0 Current       Cigs   Nev any
  HUHTI1 154     f   40   64    Eu:Scand  1961  1965    CS  Prev    Emp:other   0 Current       Cigs  Nev cigs
   KAHN2  47     m   31   84      Am:USA  1954  1966    Pr   Inc     Emp:mort   1 Current       Cigs   Nev any
  LAVECC  31 x   m   15   99     Eu:West  1983  1988    CS  Prev Emp:self-rep   0 Current       Cigs   Nev any
  LAVECC  32 x   f   15   99     Eu:West  1983  1988    CS  Prev Emp:self-rep   0 Current       Cigs   Nev any
  LEBOWI  31 x   b   15   96      Am:USA  1972  1977    CS  Prev Emp:diagnosd   2 Current       Cigs  Nev cigs
  MILLER   7     m   15   99      Am:USA  1978  1988    CS  Prev Emp:diagnosd   1 Current       Cigs  Nev cigs
  MILLER  10     f   15   99      Am:USA  1978  1988    CS  Prev Emp:diagnosd   1 Current       Cigs  Nev cigs
    NAWA   1     m   50   69   Asia:FarE  1998  2002    CS  Prev  Emp:viscomp   0    Ever       Cigs  Nev cigs
   OMORI   1 x   m   40   69   Asia:FarE     *  2006    CS  Prev  Emp:viscomp   0 Current       Cigs  Nev cigs
   PRATT   1     m   15   99      Am:USA     *  1980    CS  Prev  Emp:viscomp   0    Ever        Any   Nev any
   RYDER   1 x   m   22   95       Eu:UK     *  1971    CS  Prev  Emp:viscomp   0    Ever        Any   Nev any
[truncated: 112,110 more chars]
