# Supplementary material for: Systematic review with meta-analysis of the epidemiological evidence relating smoking to COPD, chronic bronchitis and emphysema
Source: BMC Pulm Med. 2011 Jun 14;11:36. doi: 10.1186/1471-2466-11-36 (PMC3128042; doi:10.1186/1471-2466-11-36)
Supplement: Additional file 7 — MetaDoseCOPD. .RTF file giving the full results of the meta-analyses for the dose-related smoking variables-amount smoked, age of starting to smoke, pack-years, duration of smoking, duration of quitting vs. never smoking, and duration of quitting vs. current smoking-for COPD. [file 1471-2466-11-36-S7.RTF]

Systematic review with meta-analysis of the epidemiological evidence relating smoking to COPD, chronic bronchitis and emphysema

Barbara A Forey, Alison J Thornton and Peter N Lee

Additional file 7 : MetaDoseCOPD

See Additional file 10 (Intro sheet) for list of tables and page numbers


                                                    Table 1 - E - 1 -

                                IESCOPD - Meta-analysis of amount smoked : key value (1) 5
                            Any COPD, cigarettes (or any product if cigarettes not available)


This analysis is restricted to results for:
1) Eligible study on database
2) Outcome COPD
3) Current or ever smoking
4) Categorical dose-response data for amount smoked
5) vs never smoking base
6) Key value (scheme 1) = 5
7) Results complete enough for use in meta-analysis

Within each study, results are then selected (in the following order of preference, within each sex) for:
8) SMKSTA  : current, ever
9) UNEXP   : never any, never cigarettes
10) PROD    : cigarettes, cigarettes only, any product
11) For overlapping studies: principal rather than subsidiary studies
and then for single sex results (m, f) in preference to results for both sexes combined (b).

Results adjusted for the most potential confounders are then chosen in Sections -1 to -3
and results adjusted for the least confounders in Sections -4 to -6. (Those least-adjusted results which
actually differ from the most-adjusted are marked 'x' in column X in Section -4)

Section -7 shows excluded studies, together with the stage (as above) at which no qualifying
results were found.

Section -8 lists the potentially overlapping studies which have been included (1=principal, 2=subsidiary),
and any results which would have been included in preference except that they had data not complete enough
for use in meta-analysis. It also lists their significance (yes/no), if known.


  ________________________________________________________________________________________________________________________
                                            International Evidence on Smoking and COPD, Phase 3, Analysis run on 28-SEP-10

                                                   Table 1 - E - 1 - 1

                                IESCOPD - Meta-analysis of amount smoked : key value (1) 5
                            Any COPD, cigarettes (or any product if cigarettes not available)
                                                      Most-adjusted


     REF|NRR|SEX|AGEL|AGEH|     REGION|BEGYR|PUBYR|STTYP|ONSET|      DISEAS|ADJ|SMOKSTA|   PRODUCT|    UNEXP|LOW| HI|

  ANDER1  11   m   25   74   Am:Canada  1963  1965    CS  Prev     COPD:oth   1 Current       Cigs   Nev any   1  14
    BEST  38   m   30   97   Am:Canada  1955  1967    Pr   Inc    COPD:mort   1 Current  Cigs only   Nev any   1   9
   CHEN2  13   m   35   64   Am:Canada  1994  2000    CS  Prev        CB/EM   6 Current       Cigs  Nev cigs   1  19
   CHEN2  16   f   35   64   Am:Canada  1994  2000    CS  Prev        CB/EM   6 Current       Cigs  Nev cigs   1  19
  CLEMEN   4   m   20   60     Eu:West  1960  1982    Pr  Prev      COPD:LF   0 Current       Cigs   Nev any   1  19
   DEAN1  24   m   35   99       Eu:UK  1969  1977    CC  Prev    COPD:mort   3 Current MCigs only   Nev any   1  12
   DEAN1  64   f   35   99       Eu:UK  1969  1977    CC  Prev    COPD:mort   3 Current MCigs only   Nev any   1  12
   DOLL1   7   m   20   99       Eu:UK  1951  1994    Pr   Inc    COPD:mort   2 Current  Cigs only   Nev any   1  14
   DOLL2   4   f   20   99       Eu:UK  1951  1980    Pr   Inc    COPD:mort   1 Current  Cigs only   Nev any   1  14
  ENSTRO   7   m   30   99      Am:USA  1960  2003    Pr   Inc    COPD:mort   1 Current  Cigs only   Nev any   1   9
  ENSTRO  16   f   30   99      Am:USA  1960  2003    Pr   Inc    COPD:mort   1 Current  Cigs only   Nev any   1   9
  FERRI2  26   m   25   80      Am:USA  1967  1971    CS  Prev     COPD:oth   1 Current       Cigs   Nev any   5  14
  FERRI2  43   f   25   80      Am:USA  1967  1971    CS  Prev     COPD:oth   1 Current       Cigs   Nev any   5  14
  GODTFR   6   b   20   99    Eu:Scand  1964  2002    Pr   Inc     COPD:ICD   3 Current        Any   Nev any   1  14
  HOZAWA   4   b   45   64      Am:USA  1987  2006    CS  Prev      COPD:LF   0 Current       Cigs  Nev cigs   1  14
  HUHTI1  26   m   40   64    Eu:Scand  1961  1965    CS  Prev    CB/EM/Ast   1 Current       Cigs   Nev any   1  14
  HUHTI1  34   f   40   64    Eu:Scand  1961  1965    CS  Prev    CB/EM/Ast   1 Current       Cigs   Nev any   1  14
  HUHTI3  12   m   25   69    Eu:Scand  1968  1978    CS  Prev      COPD:LF   1 Current        Any   Nev any   1  14
  JACOBS  26   m   40   84       Multi  1957  1999    Pr   Inc    COPD:mort   6 Current       Cigs  Nev cigs   1   9
   KAHN2  99   m   31   84      Am:USA  1954  1966    Pr   Inc    COPD:mort   1 Current       Cigs   Nev any   1   9
  KRZYZA   7   m   19   83     Eu:East  1968  1986    Pr   Inc      COPD:LF   1 Current       Cigs  Nev cigs   1  14
  KRZYZA  10   f   19   83     Eu:East  1968  1986    Pr   Inc      COPD:LF   1 Current       Cigs  Nev cigs   1  14
     LEE  22   m   35   82       Eu:UK  1964  1979    Pr   Inc    COPD:mort   1 Current  Cigs only   Nev any   1   9
     LEE  43   f   35   82       Eu:UK  1964  1979    Pr   Inc    COPD:mort   0 Current  Cigs only   Nev any   1   9
  LINDST  11   b   20   69    Eu:Scand     *  2001    CS  Prev        CB/EM   5 Current       Cigs  Nev cigs   5  14
  LUNDB1  11   b   46   77    Eu:Scand  1996  2003    CS  Prev      COPD:LF   4 Current       Cigs  Nev cigs   5  14
  MUELLE  25   m   20   69      Am:USA  1967  1971    CS  Prev      COPD:LF   1 Current       Cigs   Nev any   1  14
  NILSSO  10   m   18   99    Eu:Scand  1963  2001    Pr   Inc    COPD:mort   2 Current  Cigs only   Nev any   1   7
  NILSSO  15   f   18   99    Eu:Scand  1963  2001    Pr   Inc    COPD:mort   2 Current  Cigs only   Nev any   1   7
    PETO   4   m   25   92       Eu:UK  1954  1983    Pr   Inc    COPD:mort   0 Current       Cigs  Nev cigs   1  14
  SPEIZE   7   m   25   86      Am:USA  1974  1989    Pr   Inc    COPD:mort   1 Current       Cigs  Nev cigs   1  14
  SPEIZE  10   f   25   86      Am:USA  1974  1989    Pr   Inc    COPD:mort   1 Current       Cigs  Nev cigs   1  14
    TODD  22   m   35   81       Eu:UK  1965  1978    Pr   Inc    COPD:mort   1 Current  Cigs only   Nev any   1   9
  TVERDA   5   m   35   65    Eu:Scand  1972  1993    Pr   Inc    COPD:mort   2 Current  Cigs only  Nev cigs   1   9
  VONHER   9   m   30   99    Eu:Scand  1978  2000    CS  Prev        CB/EM   0 Current       Cigs   Nev any   1   9
  VONHER  16   f   30   99    Eu:Scand  1978  2000    CS  Prev        CB/EM   0 Current       Cigs   Nev any   1   9
   WEISS   6   m   50   69      Am:USA  1961  1963    CS  Prev      COPD:LF   0 Current  Cigs only   Nev any   1   9
     WEN   2   m   35   99   Asia:FarE  1982  2004    Pr   Inc    COPD:mort   1 Current       Cigs  Nev cigs   1  10
  YAMAGU   4   b   40   99   Asia:FarE  1986  1988    CS  Prev      COPD:LF   6 Current       Cigs  Nev cigs   1   9
    YUAN   9   m   45   71   Asia:FarE  1986  1996    Pr   Inc    COPD:mort   2 Current       Cigs  Nev cigs   1  19


  ________________________________________________________________________________________________________________________
                                            International Evidence on Smoking and COPD, Phase 3, Analysis run on 28-SEP-10

                                                   Table 1 - E - 1 - 2

                                IESCOPD - Meta-analysis of amount smoked : key value (1) 5
                            Any COPD, cigarettes (or any product if cigarettes not available)
                                                      Most-adjusted


                        Number Exposed  Non-exposed
 REF    NRR SEX ADJ     Case    Cont    Case    Cont      RR        95.00%CI
 ANDER1 11  m   1          3       -       2       -      2.79 (  0.41-  18.82)
*BEST   38  m   1         26       -       6       -      6.06 (  2.50-  14.71)
 CHEN2  13  m   6          -       -       8       -      1.72 (  0.50-   5.92)
 CHEN2  16  f   6          -       -      33       -      2.61 (  1.33-   5.13)
 Subtotal CHEN2                                           2.37 (  1.31-   4.29)
 CLEMEN 4   m   0         16     812      11     709      1.27 (  0.59-   2.75)
 DEAN1  24  m   3         97       -      47       -      3.22 (  2.09-   4.97)
 DEAN1  64  f   3         25       -     120       -      1.01 (  0.63-   1.62)
 Subtotal DEAN1                                           1.90 (  1.38-   2.61)
*DOLL1  7   m   2         58       -      12       -      8.60 (  4.64-  15.92)
*DOLL2  4   f   1          3       -       1       -     10.50 (  1.09- 100.95)
*ENSTRO 7   m   1         35       -     103       -      2.84 (  1.94-   4.17)
*ENSTRO 16  f   1         50       -     296       -      1.64 (  1.21-   2.22)
 Subtotal ENSTRO                                          2.03 (  1.60-   2.57)
 FERRI2 26  m   1         16       -      11       -      2.55 (  1.08-   6.04)
 FERRI2 43  f   1         13       -      54       -      1.59 (  0.82-   3.09)
 Subtotal FERRI2                                          1.90 (  1.12-   3.21)
*GODTFR 6   b   3        270       -      71       -      4.03 (  3.02-   5.37)
 HOZAWA 4   b   0        305     759     872    5019      2.31 (  1.99-   2.69)
 HUHTI1 26  m   1         47       -       7       -     10.72 (  4.39-  26.22)
 HUHTI1 34  f   1         10       -      56       -      1.84 (  0.87-   3.88)
 Subtotal HUHTI1                                          3.80 (  2.14-   6.74)
 HUHTI3 12  m   1         42       -       3       -      9.77 (  3.22-  29.61)
*JACOBS 26  m   6         39       -      28       -      2.90 (  1.75-   4.85)
*KAHN2  99  m   1         28       -      31       -      4.14 (  2.48-   6.90)
*KRZYZA 7   m   1          8       -       5       -      2.24 (  0.79-   6.39)
*KRZYZA 10  f   1          6       -      32       -      1.40 (  0.65-   3.01)
 Subtotal KRZYZA                                          1.65 (  0.89-   3.06)
*LEE    22  m   1          4       -       1       -      7.75 (  0.87-  68.85)
*LEE    43  f   0          2     342       5    1694      1.98 (  0.39-  10.17)
 Subtotal LEE                                             3.23 (  0.87-  11.98)
 LINDST 11  b   5          -       -     166       -      1.94 (  1.47-   2.57)
 LUNDB1 11  b   4          -       -      39       -      6.44 (  3.37-  11.60)
 MUELLE 25  m   1          4       -       2       -      4.51 (  0.71-  28.48)
*NILSSO 10  m   2         19       -      31       -      3.30 (  1.86-   5.85)
*NILSSO 15  f   2         18       -      57       -      3.80 (  2.20-   6.57)
 Subtotal NILSSO                                          3.55 (  2.39-   5.28)
*PETO   4   m   0         57    1181       0     295     28.76~(  1.78- 464.04)
*SPEIZE 7   m   1          6       -       0       -     13.00 (  0.73- 230.76)
*SPEIZE 10  f   1          5       -       5       -      6.33 (  1.83-  21.88)
 Subtotal SPEIZE                                          7.09 (  2.27-  22.14)
*TODD   22  m   1          6       -       1       -      8.94 (  1.08-  73.89)
*TVERDA 5   m   2          5       -       7       -      1.86 (  0.59-   5.88)
 VONHER 9   m   0         50     121      63     911      5.98 (  3.94-   9.07)
 VONHER 16  f   0         28     172     137    2888      3.43 (  2.22-   5.30)
 Subtotal VONHER                                          4.58 (  3.39-   6.19)
 WEISS  6   m   0          2      13       2      34      2.62 (  0.33-  20.55)
*WEN    2   m   1         27       -      52       -      1.83 (  1.07-   3.12)
 YAMAGU 4   b   6          -       -     144       -      1.96 (  1.53-   2.53)
*YUAN   9   m   2          9       -      19       -      0.80 (  0.36-   1.77)
Partial Totals          1339    3400    2540   11550
*prospective study                                        ~ With 0.5 adjustment for zero


  ________________________________________________________________________________________________________________________
                                            International Evidence on Smoking and COPD, Phase 3, Analysis run on 28-SEP-10

                                                   Table 1 - E - 1 - 2

                                IESCOPD - Meta-analysis of amount smoked : key value (1) 5
                            Any COPD, cigarettes (or any product if cigarettes not available)
                                                      Most-adjusted


 REF    NRR SEX ADJ             Ys       Ws       Qs       Ps
 ANDER1 11  m   1              1.03     1.05     0.01       0.29
*BEST   38  m   1              1.80     4.89     3.58       0.00
 CHEN2  13  m   6              0.54     2.52     0.41       0.39
 CHEN2  16  f   6              0.96     8.43     0.00       0.01
 Subtotal CHEN2                0.86    10.95     0.41
 CLEMEN 4   m   0              0.24     6.41     3.21       0.55
 DEAN1  24  m   3              1.17    20.48     1.02       0.00
 DEAN1  64  f   3              0.01    17.23    15.12       0.97
 Subtotal DEAN1                0.64    37.70    16.13
*DOLL1  7   m   2              2.15    10.11    14.68       0.00
*DOLL2  4   f   1              2.35     0.75     1.48       0.04
*ENSTRO 7   m   1              1.04    26.24     0.25       0.00
*ENSTRO 16  f   1              0.49    41.72     8.52       0.00
 Subtotal ENSTRO               0.71    67.96     8.77
 FERRI2 26  m   1              0.94     5.19     0.00       0.03
 FERRI2 43  f   1              0.46     8.73     2.04       0.17
 Subtotal FERRI2               0.64    13.92     2.04
*GODTFR 6   b   3              1.39    46.38     9.27       0.00
 HOZAWA 4   b   0              0.84   168.29     1.97       0.00
 HUHTI1 26  m   1              2.37     4.81     9.77       0.00
 HUHTI1 34  f   1              0.61     6.87     0.78       0.11
 Subtotal HUHTI1               1.34    11.68    10.55
 HUHTI3 12  m   1              2.28     3.12     5.54       0.00
*JACOBS 26  m   6              1.06    14.79     0.21       0.00
*KAHN2  99  m   1              1.42    14.68     3.30       0.00
*KRZYZA 7   m   1              0.81     3.52     0.07       0.13
*KRZYZA 10  f   1              0.34     6.54     2.44       0.39
 Subtotal KRZYZA               0.50    10.06     2.51
*LEE    22  m   1              2.05     0.80     0.97       0.07
*LEE    43  f   0              0.68     1.44     0.10       0.41
 Subtotal LEE                  1.17     2.24     1.07
 LINDST 11  b   5              0.66    49.24     3.97       0.00
 LUNDB1 11  b   4              1.86    10.06     8.43       0.00
 MUELLE 25  m   1              1.51     1.13     0.35       0.11
*NILSSO 10  m   2              1.19    11.70     0.72       0.00
*NILSSO 15  f   2              1.34    12.84     1.94       0.00
 Subtotal NILSSO               1.27    24.54     2.65
*PETO   4   m   0              3.36     0.50     2.89       0.02
*SPEIZE 7   m   1              2.56     0.46     1.21       0.08
*SPEIZE 10  f   1              1.85     2.50     2.02       0.00
 Subtotal SPEIZE               1.96     2.96     3.23
*TODD   22  m   1              2.19     0.86     1.33       0.04
*TVERDA 5   m   2              0.62     2.91     0.31       0.29
 VONHER 9   m   0              1.79    22.11    15.63       0.00
 VONHER 16  f   0              1.23    20.34     1.67       0.00
 Subtotal VONHER               1.52    42.44    17.30
 WEISS  6   m   0              0.96     0.90     0.00       0.36
*WEN    2   m   1              0.60    13.42     1.57       0.03
 YAMAGU 4   b   6              0.67    60.74     4.55       0.00
*YUAN   9   m   2             -0.22     6.06     8.29       0.58

                       N       40
                      NS       30


                      Wt   640.72
                 Het Chi   139.61
                 Het  df       39
                 Het  P       ***
               Fixed  RR     2.58
                     RRl     2.39
                     RRu     2.78
                      P       +++
              Random  RR     2.89
                     RRl     2.41
                     RRu     3.45
                      P       +++
               Asymm  P       (*)

  ________________________________________________________________________________________________________________________
                                            International Evidence on Smoking and COPD, Phase 3, Analysis run on 28-SEP-10

                                                   Table 1 - E - 1 - 3

                                IESCOPD - Meta-analysis of amount smoked : key value (1) 5
                            Any COPD, cigarettes (or any product if cigarettes not available)
                                                      Most-adjusted


                       N       40
                      NS       30


                      Wt   640.72
                 Het Chi   139.61
                 Het  df       39
                 Het  P       ***
               Fixed  RR     2.58
                     RRl     2.39
                     RRu     2.78
                      P       +++
              Random  RR     2.89
                     RRl     2.41
                     RRu     3.45
                      P       +++
               Asymm  P       (*)

                                   Sex
                             both      male    female     Total


                       N        5        24        11        40
                      NS        5        24        11        40


                      Wt   334.71    178.64    127.38    640.72
                 Het Chi    27.13     61.00     28.21    139.61
                 Het  df        4        23        10        39
                 Het  P       ***       ***        **       ***
               Fixed  RR     2.44      3.42      2.01      2.58
                     RRl     2.19      2.95      1.69      2.39
                     RRu     2.71      3.96      2.39      2.78
                      P       +++       +++       +++       +++
              Random  RR     2.75      3.45      2.17      2.89
                     RRl     2.01      2.62      1.56      2.41
                     RRu     3.77      4.54      3.04      3.45
                      P       +++       +++       +++       +++
             Between Chi                                  23.27
             Between  df                                      2
             Between  P                                     ***
             Btwn(F)  P                                       *

                                        Continent
                            NAmer    Europe      Asia  oth/mult     Total


                       N       14        22         3         1        40
                      NS       10        16         3         1        30


                      Wt   286.72    259.00     80.22     14.79    640.72
                 Het Chi    21.50     91.13      4.43      0.00    139.61
                 Het  df       13        21         2         0        39
                 Het  P       (*)       ***      N.S.      N.S.       ***
               Fixed  RR     2.36      3.14      1.81      2.90      2.58
                     RRl     2.11      2.78      1.45      1.74      2.39
                     RRu     2.65      3.55      2.25      4.83      2.78
                      P       +++       +++       +++       +++       +++
              Random  RR     2.57      3.41      1.61      2.90      2.89
                     RRl     2.07      2.54      1.06      1.74      2.41
                     RRu     3.20      4.57      2.47      4.83      3.45
                      P       +++       +++         +       +++       +++
             Between Chi                                            22.56
             Between  df                                                3
             Between  P                                               ***
             Btwn(F)  P                                               (*)


  ________________________________________________________________________________________________________________________
                                            International Evidence on Smoking and COPD, Phase 3, Analysis run on 28-SEP-10

                                                   Table 1 - E - 1 - 3

                                IESCOPD - Meta-analysis of amount smoked : key value (1) 5
                            Any COPD, cigarettes (or any product if cigarettes not available)
                                                      Most-adjusted
                        National cigarette tobacco type (excluding mixed/unkown)
                          blended  virginia     Total


                       N       25        12        37
                      NS       18         9        27


                      Wt   491.45     69.05    560.50
                 Het Chi    82.76     40.70    123.76
                 Het  df       24        11        36
                 Het  P       ***       ***       ***
               Fixed  RR     2.69      2.89      2.71
                     RRl     2.46      2.28      2.50
                     RRu     2.94      3.65      2.94
                      P       +++       +++       +++
              Random  RR     3.02      3.65      3.10
                     RRl     2.47      2.09      2.56
                     RRu     3.69      6.34      3.75
                      P       +++       +++       +++
             Between Chi                         0.31
             Between  df                            1
             Between  P                          N.S.
             Btwn(F)  P                          N.S.

                                        Start year of study
                            <1970   1970-79   1980-89   1990-99     2000+   unknown     Total


                       N       27         5         4         3                   1        40
                      NS       20         3         4         2                   1        30


                      Wt   273.67     48.31    248.51     21.00               49.24    640.72
                 Het Chi    87.14      6.35      7.68      5.57                0.00    139.61
                 Het  df       26         4         3         2                   0        39
                 Het  P       ***      N.S.       (*)       (*)                N.S.       ***
               Fixed  RR     2.83      4.46      2.14      3.83                1.94      2.58
                     RRl     2.52      3.36      1.89      2.49                1.47      2.39
                     RRu     3.19      5.91      2.42      5.87                2.57      2.78
                      P       +++       +++       +++       +++                 +++       +++
              Random  RR     3.09      4.34      1.91      3.40                1.94      2.89
                     RRl     2.40      2.82      1.46      1.58                1.47      2.41
                     RRu     3.98      6.69      2.50      7.33                2.57      3.45
                      P       +++       +++       +++        ++                 +++       +++
             Between Chi                                                                32.87
             Between  df                                                                    4
             Between  P                                                                   ***
             Btwn(F)  P                                                                     *

                                Publication year
                            <1980   1980-89   1990-99     2000+     Total


                       N       15         8         4        13        40
                      NS       11         6         4         9        30


                      Wt    92.17     81.41     33.86    433.27    640.72
                 Het Chi    43.48     12.75     22.46     54.20    139.61
                 Het  df       14         7         3        12        39
                 Het  P       ***       (*)       ***       ***       ***
               Fixed  RR     2.76      2.01      3.07      2.63      2.58
                     RRl     2.25      1.61      2.19      2.39      2.39
                     RRu     3.38      2.49      4.30      2.89      2.78
                      P       +++       +++       +++       +++       +++
              Random  RR     3.29      2.35      2.56      2.93      2.89
                     RRl     2.17      1.47      0.96      2.33      2.41
                     RRu     4.99      3.76      6.83      3.69      3.45
                      P       +++       +++       (+)       +++       +++
             Between Chi                                             6.73
             Between  df                                                3
             Between  P                                               (*)
             Btwn(F)  P                                              N.S.
  ________________________________________________________________________________________________________________________
                                            International Evidence on Smoking and COPD, Phase 3, Analysis run on 28-SEP-10

                                                   Table 1 - E - 1 - 3

                                IESCOPD - Meta-analysis of amount smoked : key value (1) 5
                            Any COPD, cigarettes (or any product if cigarettes not available)
                                                      Most-adjusted
                               Study type
                               CC        Pr        CS     Total


                       N        2        22        16        40
                      NS        1        17        12        30


                      Wt    37.70    229.50    373.52    640.72
                 Het Chi    12.58     66.13     54.82    139.61
                 Het  df        1        21        15        39
                 Het  P       ***       ***       ***       ***
               Fixed  RR     1.90      2.84      2.50      2.58
                     RRl     1.38      2.50      2.26      2.39
                     RRu     2.61      3.24      2.77      2.78
                      P       +++       +++       +++       +++
              Random  RR     1.81      2.96      3.03      2.89
                     RRl     0.58      2.26      2.34      2.41
                     RRu     5.64      3.89      3.93      3.45
                      P      N.S.       +++       +++       +++
             Between Chi                                   6.09
             Between  df                                      2
             Between  P                                       *
             Btwn(F)  P                                    N.S.

                                    Lowest age in RR
                        <25/unlim     25-39       40+   unknown     Total


                       N       10        22         8                  40
                      NS        8        15         7                  30


                      Wt   148.61    219.59    272.52              640.72
                 Het Chi    35.57     67.26     31.45              139.61
                 Het  df        9        21         7                  39
                 Het  P       ***       ***       ***                 ***
               Fixed  RR     2.94      2.66      2.34                2.58
                     RRl     2.50      2.33      2.08                2.39
                     RRu     3.45      3.04      2.63                2.78
                      P       +++       +++       +++                 +++
              Random  RR     3.01      2.97      2.67                2.89
                     RRl     2.05      2.25      1.86                2.41
                     RRu     4.42      3.93      3.82                3.45
                      P       +++       +++       +++                 +++
             Between Chi                                             5.33
             Between  df                                                2
             Between  P                                               (*)
             Btwn(F)  P                                              N.S.

                                         Highest age in RR
                              <65     65-74     75-84 85+/unlim   unknown     Total


                       N        6         7        10        17                  40
                      NS        4         7         7        12                  30


                      Wt   197.33     64.40     66.59    312.40              640.72
                 Het Chi    14.30     13.92     17.16     84.02              139.61
                 Het  df        5         6         9        16                  39
                 Het  P         *         *         *       ***                 ***
               Fixed  RR     2.34      1.98      3.03      2.80                2.58
                     RRl     2.03      1.55      2.38      2.50                2.39
                     RRu     2.69      2.52      3.85      3.12                2.78
                      P       +++       +++       +++       +++                 +++
              Random  RR     2.48      2.31      2.93      3.29                2.89
                     RRl     1.59      1.27      2.03      2.46                2.41
                     RRu     3.88      4.20      4.23      4.39                3.45
                      P       +++        ++       +++       +++                 +++
             Between Chi                                                      10.22
             Between  df                                                          3
             Between  P                                                           *
             Btwn(F)  P                                                        N.S.
  ________________________________________________________________________________________________________________________
                                            International Evidence on Smoking and COPD, Phase 3, Analysis run on 28-SEP-10

                                                   Table 1 - E - 1 - 3

                                IESCOPD - Meta-analysis of amount smoked : key value (1) 5
                            Any COPD, cigarettes (or any product if cigarettes not available)
                                                      Most-adjusted
                           Study weakness
                              Yes        No     Total


                       N        4        36        40
                      NS        3        27        30


                      Wt    41.11    599.61    640.72
                 Het Chi    16.21    120.07    139.61
                 Het  df        3        35        39
                 Het  P        **       ***       ***
               Fixed  RR     1.96      2.63      2.58
                     RRl     1.44      2.42      2.39
                     RRu     2.66      2.85      2.78
                      P       +++       +++       +++
              Random  RR     2.29      2.98      2.89
                     RRl     0.93      2.48      2.41
                     RRu     5.63      3.58      3.45
                      P       (+)       +++       +++
             Between Chi                         3.34
             Between  df                            1
             Between  P                           (*)
             Btwn(F)  P                          N.S.

                          COPD subtype
                             mort        LF     other     Total


                       N       20         9        11        40
                      NS       15         8         7        30


                      Wt   204.36    260.71    175.66    640.72
                 Het Chi    69.49     23.15     38.22    139.61
                 Het  df       19         8        10        39
                 Het  P       ***        **       ***       ***
               Fixed  RR     2.57      2.30      3.07      2.58
                     RRl     2.24      2.04      2.65      2.39
                     RRu     2.95      2.60      3.56      2.78
                      P       +++       +++       +++       +++
              Random  RR     3.00      2.55      3.05      2.89
                     RRl     2.21      1.83      2.18      2.41
                     RRu     4.08      3.54      4.26      3.45
                      P       +++       +++       +++       +++
             Between Chi                                   8.75
             Between  df                                      2
             Between  P                                       *
             Btwn(F)  P                                    N.S.

                               Asthma analysis type (COPD)
                        inc-irres  excl-all defn-incl     other     Total


                       N       24                  13         3        40
                      NS       19                   8         3        30


                      Wt   470.84              144.24     25.65    640.72
                 Het Chi    73.26               41.97      7.62    139.61
                 Het  df       23                  12         2        39
                 Het  P       ***                 ***         *       ***
               Fixed  RR     2.68                2.06      4.62      2.58
                     RRl     2.44                1.75      3.14      2.39
                     RRu     2.93                2.42      6.81      2.78
                      P       +++                 +++       +++       +++
              Random  RR     3.07                2.26      5.41      2.89
                     RRl     2.49                1.59      2.14      2.41
                     RRu     3.78                3.23     13.65      3.45
                      P       +++                 +++       +++       +++
             Between Chi                                            16.77
             Between  df                                                2
             Between  P                                               ***
             Btwn(F)  P                                               (*)
  ________________________________________________________________________________________________________________________
                                            International Evidence on Smoking and COPD, Phase 3, Analysis run on 28-SEP-10

                                                   Table 1 - E - 1 - 3

                                IESCOPD - Meta-analysis of amount smoked : key value (1) 5
                            Any COPD, cigarettes (or any product if cigarettes not available)
                                                      Most-adjusted
                        Bronchodilator/reversibility (LF only)
                          no/unkn  yes/revs     Total


                       N        8         1         9
                      NS        7         1         8


                      Wt   250.65     10.06    260.71
                 Het Chi    12.05      0.00     23.15
                 Het  df        7         0         8
                 Het  P       (*)      N.S.        **
               Fixed  RR     2.21      6.44      2.30
                     RRl     1.95      3.47      2.04
                     RRu     2.50     11.95      2.60
                      P       +++       +++       +++
              Random  RR     2.16      6.44      2.55
                     RRl     1.67      3.47      1.83
                     RRu     2.81     11.95      3.54
                      P       +++       +++       +++
             Between Chi                        11.10
             Between  df                            1
             Between  P                           ***
             Btwn(F)  P                             *

                                  Number of COPD cases
                             1-50    51-100   101-200      201+     Total


                       N        5         8         8        19        40
                      NS        4         7         6        13        30


                      Wt     5.74     22.64     49.87    562.47    640.72
                 Het Chi     1.23     17.75     18.97     95.38    139.61
                 Het  df        4         7         7        18        39
                 Het  P      N.S.         *        **       ***       ***
               Fixed  RR     5.83      1.91      2.83      2.57      2.58
                     RRl     2.57      1.27      2.14      2.36      2.39
                     RRu    13.22      2.89      3.73      2.79      2.78
                      P       +++        ++       +++       +++       +++
              Random  RR     5.83      2.50      2.93      2.89      2.89
                     RRl     2.57      1.21      1.78      2.34      2.41
                     RRu    13.22      5.15      4.81      3.57      3.45
                      P       +++         +       +++       +++       +++
             Between Chi                                             6.28
             Between  df                                                3
             Between  P                                               (*)
             Btwn(F)  P                                              N.S.

                        Number of COPD cases (excluding unknown)


                       N        5         8         8        19        40
                      NS        4         7         6        13        30


                      Wt     5.74     22.64     49.87    562.47    640.72
                 Het Chi     1.23     17.75     18.97     95.38    139.61
                 Het  df        4         7         7        18        39
                 Het  P      N.S.         *        **       ***       ***
               Fixed  RR     5.83      1.91      2.83      2.57      2.58
                     RRl     2.57      1.27      2.14      2.36      2.39
                     RRu    13.22      2.89      3.73      2.79      2.78
                      P       +++        ++       +++       +++       +++
              Random  RR     5.83      2.50      2.93      2.89      2.89
                     RRl     2.57      1.21      1.78      2.34      2.41
                     RRu    13.22      5.15      4.81      3.57      3.45
                      P       +++         +       +++       +++       +++
             Between Chi                                             6.28
             Between  df                                                3
             Between  P                                               (*)
             Btwn(F)  P                                              N.S.

  ________________________________________________________________________________________________________________________
                                            International Evidence on Smoking and COPD, Phase 3, Analysis run on 28-SEP-10

                                                   Table 1 - E - 1 - 3

                                IESCOPD - Meta-analysis of amount smoked : key value (1) 5
                            Any COPD, cigarettes (or any product if cigarettes not available)
                                                      Most-adjusted
                            Analysis type
                         prevlnce     onset     Total


                       N       19        21        40
                      NS       14        16        30


                      Wt   417.63    223.09    640.72
                 Het Chi    72.72     61.85    139.61
                 Het  df       18        20        39
                 Het  P       ***       ***       ***
               Fixed  RR     2.42      2.91      2.58
                     RRl     2.19      2.55      2.39
                     RRu     2.66      3.32      2.78
                      P       +++       +++       +++
              Random  RR     2.72      3.10      2.89
                     RRl     2.13      2.35      2.41
                     RRu     3.47      4.08      3.45
                      P       +++       +++       +++
             Between Chi                         5.04
             Between  df                            1
             Between  P                             *
             Btwn(F)  P                          N.S.

                             Smoking product
                              any      cigs  cigsonly     Total


                       N        2        24        14        40
                      NS        2        18        10        30


                      Wt    49.50    438.35    152.87    640.72
                 Het Chi     2.29     73.60     49.98    139.61
                 Het  df        1        23        13        39
                 Het  P      N.S.       ***       ***       ***
               Fixed  RR     4.26      2.45      2.54      2.58
                     RRl     3.23      2.23      2.17      2.39
                     RRu     5.63      2.69      2.98      2.78
                      P       +++       +++       +++       +++
              Random  RR     5.30      2.66      3.10      2.89
                     RRl     2.38      2.15      2.13      2.41
                     RRu    11.82      3.29      4.50      3.45
                      P       +++       +++       +++       +++
             Between Chi                                  13.74
             Between  df                                      2
             Between  P                                      **
             Btwn(F)  P                                    N.S.

                                     Unexposed group
                          nev any   nev cig  nev+ any  nev+ cig     Total


                       N       25        15                            40
                      NS       18        12                            30


                      Wt   290.77    349.95                        640.72
                 Het Chi    92.07     30.71                        139.61
                 Het  df       24        14                            39
                 Het  P       ***        **                           ***
               Fixed  RR     3.08      2.22                          2.58
                     RRl     2.74      2.00                          2.39
                     RRu     3.45      2.47                          2.78
                      P       +++       +++                           +++
              Random  RR     3.34      2.28                          2.89
                     RRl     2.57      1.84                          2.41
                     RRu     4.33      2.83                          3.45
                      P       +++       +++                           +++
             Between Chi                                            16.84
             Between  df                                                1
             Between  P                                               ***
             Btwn(F)  P                                                 *
  ________________________________________________________________________________________________________________________
                                            International Evidence on Smoking and COPD, Phase 3, Analysis run on 28-SEP-10

                                                   Table 1 - E - 1 - 3

                                IESCOPD - Meta-analysis of amount smoked : key value (1) 5
                            Any COPD, cigarettes (or any product if cigarettes not available)
                                                      Most-adjusted
                        Unexposed group (combining nev+ with main levels)
                          nev any   nev cig     Total


                       N       25        15        40
                      NS       18        12        30


                      Wt   290.77    349.95    640.72
                 Het Chi    92.07     30.71    139.61
                 Het  df       24        14        39
                 Het  P       ***        **       ***
               Fixed  RR     3.08      2.22      2.58
                     RRl     2.74      2.00      2.39
                     RRu     3.45      2.47      2.78
                      P       +++       +++       +++
              Random  RR     3.34      2.28      2.89
                     RRl     2.57      1.84      2.41
                     RRu     4.33      2.83      3.45
                      P       +++       +++       +++
             Between Chi                        16.84
             Between  df                            1
             Between  P                           ***
             Btwn(F)  P                             *

                        Number of adjustment variables
                                0         1        2+     Total


                       N        7        19        14        40
                      NS        6        14        11        31


                      Wt   219.97    147.27    273.47    640.72
                 Het Chi    25.44     45.17     68.91    139.61
                 Het  df        6        18        13        39
                 Het  P       ***       ***       ***       ***
               Fixed  RR     2.61      2.52      2.58      2.58
                     RRl     2.28      2.15      2.29      2.39
                     RRu     2.97      2.97      2.91      2.78
                      P       +++       +++       +++       +++
              Random  RR     3.02      3.12      2.69      2.89
                     RRl     1.87      2.30      1.99      2.41
                     RRu     4.88      4.25      3.62      3.45
                      P       +++       +++       +++       +++
             Between Chi                                   0.10
             Between  df                                      2
             Between  P                                    N.S.
             Btwn(F)  P                                    N.S.


  ________________________________________________________________________________________________________________________
                                            International Evidence on Smoking and COPD, Phase 3, Analysis run on 28-SEP-10

                                                   Table 1 - E - 1 - 4

                                IESCOPD - Meta-analysis of amount smoked : key value (1) 5
                            Any COPD, cigarettes (or any product if cigarettes not available)
                                                      Least-adjusted


     REF|NRR|X|SEX|AGEL|AGEH|     REGION|BEGYR|PUBYR|STTYP|ONSET|      DISEAS|ADJ|SMOKSTA|   PRODUCT|    UNEXP|LOW| HI|

  ANDER1  11     m   25   74   Am:Canada  1963  1965    CS  Prev     COPD:oth   1 Current       Cigs   Nev any   1  14
    BEST  38     m   30   97   Am:Canada  1955  1967    Pr   Inc    COPD:mort   1 Current  Cigs only   Nev any   1   9
   CHEN2  13     m   35   64   Am:Canada  1994  2000    CS  Prev        CB/EM   6 Current       Cigs  Nev cigs   1  19
   CHEN2  16     f   35   64   Am:Canada  1994  2000    CS  Prev        CB/EM   6 Current       Cigs  Nev cigs   1  19
  CLEMEN   4     m   20   60     Eu:West  1960  1982    Pr  Prev      COPD:LF   0 Current       Cigs   Nev any   1  19
   DEAN1  19 x   m   35   99       Eu:UK  1969  1977    CC  Prev    COPD:mort   0 Current MCigs only   Nev any   1  12
   DEAN1  59 x   f   35   99       Eu:UK  1969  1977    CC  Prev    COPD:mort   0 Current MCigs only   Nev any   1  12
   DOLL1   7     m   20   99       Eu:UK  1951  1994    Pr   Inc    COPD:mort   2 Current  Cigs only   Nev any   1  14
   DOLL2   4     f   20   99       Eu:UK  1951  1980    Pr   Inc    COPD:mort   1 Current  Cigs only   Nev any   1  14
  ENSTRO   7     m   30   99      Am:USA  1960  2003    Pr   Inc    COPD:mort   1 Current  Cigs only   Nev any   1   9
  ENSTRO  16     f   30   99      Am:USA  1960  2003    Pr   Inc    COPD:mort   1 Current  Cigs only   Nev any   1   9
  FERRI2  26     m   25   80      Am:USA  1967  1971    CS  Prev     COPD:oth   1 Current       Cigs   Nev any   5  14
  FERRI2  43     f   25   80      Am:USA  1967  1971    CS  Prev     COPD:oth   1 Current       Cigs   Nev any   5  14
  GODTFR   6     b   20   99    Eu:Scand  1964  2002    Pr   Inc     COPD:ICD   3 Current        Any   Nev any   1  14
  HOZAWA   4     b   45   64      Am:USA  1987  2006    CS  Prev      COPD:LF   0 Current       Cigs  Nev cigs   1  14
  HUHTI1  21 x   m   40   64    Eu:Scand  1961  1965    CS  Prev    CB/EM/Ast   0 Current       Cigs   Nev any   1  14
  HUHTI1  31 x   f   40   64    Eu:Scand  1961  1965    CS  Prev    CB/EM/Ast   0 Current       Cigs   Nev any   1  14
  HUHTI3   7 x   m   25   69    Eu:Scand  1968  1978    CS  Prev      COPD:LF   0 Current        Any   Nev any   1  14
  JACOBS  18 x   m   40   84       Multi  1957  1999    Pr   Inc    COPD:mort   2 Current       Cigs  Nev cigs   5   9
   KAHN2  99     m   31   84      Am:USA  1954  1966    Pr   Inc    COPD:mort   1 Current       Cigs   Nev any   1   9
  KRZYZA   7     m   19   83     Eu:East  1968  1986    Pr   Inc      COPD:LF   1 Current       Cigs  Nev cigs   1  14
  KRZYZA  10     f   19   83     Eu:East  1968  1986    Pr   Inc      COPD:LF   1 Current       Cigs  Nev cigs   1  14
     LEE   8 x   m   35   82       Eu:UK  1964  1979    Pr   Inc    COPD:mort   0 Current  Cigs only   Nev any   1   9
     LEE  43     f   35   82       Eu:UK  1964  1979    Pr   Inc    COPD:mort   0 Current  Cigs only   Nev any   1   9
  LINDST  11     b   20   69    Eu:Scand     *  2001    CS  Prev        CB/EM   5 Current       Cigs  Nev cigs   5  14
  LUNDB1  11     b   46   77    Eu:Scand  1996  2003    CS  Prev      COPD:LF   4 Current       Cigs  Nev cigs   5  14
  MUELLE  20 x   m   20   69      Am:USA  1967  1971    CS  Prev      COPD:LF   0 Current       Cigs   Nev any   1  14
  NILSSO  10     m   18   99    Eu:Scand  1963  2001    Pr   Inc    COPD:mort   2 Current  Cigs only   Nev any   1   7
  NILSSO  15     f   18   99    Eu:Scand  1963  2001    Pr   Inc    COPD:mort   2 Current  Cigs only   Nev any   1   7
    PETO   4     m   25   92       Eu:UK  1954  1983    Pr   Inc    COPD:mort   0 Current       Cigs  Nev cigs   1  14
  SPEIZE   7     m   25   86      Am:USA  1974  1989    Pr   Inc    COPD:mort   1 Current       Cigs  Nev cigs   1  14
  SPEIZE  10     f   25   86      Am:USA  1974  1989    Pr   Inc    COPD:mort   1 Current       Cigs  Nev cigs   1  14
    TODD   8 x   m   35   81       Eu:UK  1965  1978    Pr   Inc    COPD:mort   0 Current  Cigs only   Nev any   1   9
  TVERDA   5     m   35   65    Eu:Scand  1972  1993    Pr   Inc    COPD:mort   2 Current  Cigs only  Nev cigs   1   9
  VONHER   9     m   30   99    Eu:Scand  1978  2000    CS  Prev        CB/EM   0 Current       Cigs   Nev any   1   9
  VONHER  16     f   30   99    Eu:Scand  1978  2000    CS  Prev        CB/EM   0 Current       Cigs   Nev any   1   9
   WEISS   6     m   50   69      Am:USA  1961  1963    CS  Prev      COPD:LF   0 Current  Cigs only   Nev any   1   9
     WEN   2     m   35   99   Asia:FarE  1982  2004    Pr   Inc    COPD:mort   1 Current       Cigs  Nev cigs   1  10
  YAMAGU   4     b   40   99   Asia:FarE  1986  1988    CS  Prev      COPD:LF   6 Current       Cigs  Nev cigs   1   9
    YUAN   6 x   m   45   71   Asia:FarE  1986  1996    Pr   Inc    COPD:mort   1 Current       Cigs  Nev cigs   1  19


  ________________________________________________________________________________________________________________________
                                            International Evidence on Smoking and COPD, Phase 3, Analysis run on 28-SEP-10

                                                   Table 1 - E - 1 - 5

                                IESCOPD - Meta-analysis of amount smoked : key value (1) 5
                            Any COPD, cigarettes (or any product if cigarettes not available)
                                                      Least-adjusted


                        Number Exposed  Non-exposed
 REF    NRR SEX ADJ     Case    Cont    Case    Cont      RR        95.00%CI
 ANDER1 11  m   1          3       -       2       -      2.79 (  0.41-  18.82)
*BEST   38  m   1         26       -       6       -      6.06 (  2.50-  14.71)
 CHEN2  13  m   6          -       -       8       -      1.72 (  0.50-   5.92)
 CHEN2  16  f   6          -       -      33       -      2.61 (  1.33-   5.13)
 Subtotal CHEN2                                           2.37 (  1.31-   4.29)
 CLEMEN 4   m   0         16     812      11     709      1.27 (  0.59-   2.75)
 DEAN1  19  m   0         97     256      47     510      4.11 (  2.81-   6.01)
 DEAN1  59  f   0         25     486     120    1538      0.66 (  0.42-   1.03)
 Subtotal DEAN1                                           1.89 (  1.42-   2.53)
*DOLL1  7   m   2         58       -      12       -      8.60 (  4.64-  15.92)
*DOLL2  4   f   1          3       -       1       -     10.50 (  1.09- 100.95)
*ENSTRO 7   m   1         35       -     103       -      2.84 (  1.94-   4.17)
*ENSTRO 16  f   1         50       -     296       -      1.64 (  1.21-   2.22)
 Subtotal ENSTRO                                          2.03 (  1.60-   2.57)
 FERRI2 26  m   1         16       -      11       -      2.55 (  1.08-   6.04)
 FERRI2 43  f   1         13       -      54       -      1.59 (  0.82-   3.09)
 Subtotal FERRI2                                          1.90 (  1.12-   3.21)
*GODTFR 6   b   3        270       -      71       -      4.03 (  3.02-   5.37)
 HOZAWA 4   b   0        305     759     872    5019      2.31 (  1.99-   2.69)
 HUHTI1 21  m   0         47      61       7     115     12.66 (  5.40-  29.69)
 HUHTI1 31  f   0         10      67      56     653      1.74 (  0.85-   3.57)
 Subtotal HUHTI1                                          3.97 (  2.29-   6.87)
 HUHTI3 7   m   0         42     273       3     236     12.10 (  3.70-  39.55)
*JACOBS 18  m   2         30       -      28       -      4.57 (  2.59-   8.06)
*KAHN2  99  m   1         28       -      31       -      4.14 (  2.48-   6.90)
*KRZYZA 7   m   1          8       -       5       -      2.24 (  0.79-   6.39)
*KRZYZA 10  f   1          6       -      32       -      1.40 (  0.65-   3.01)
 Subtotal KRZYZA                                          1.65 (  0.89-   3.06)
*LEE    8   m   0          4     192       1     347      7.23 (  0.81-  64.22)
*LEE    43  f   0          2     342       5    1694      1.98 (  0.39-  10.17)
 Subtotal LEE                                             3.15 (  0.85-  11.68)
 LINDST 11  b   5          -       -     166       -      1.94 (  1.47-   2.57)
 LUNDB1 11  b   4          -       -      39       -      6.44 (  3.37-  11.60)
 MUELLE 20  m   0          4      24       2      57      4.75 (  0.81-  27.69)
*NILSSO 10  m   2         19       -      31       -      3.30 (  1.86-   5.85)
*NILSSO 15  f   2         18       -      57       -      3.80 (  2.20-   6.57)
 Subtotal NILSSO                                          3.55 (  2.39-   5.28)
*PETO   4   m   0         57    1181       0     295     28.76~(  1.78- 464.04)
*SPEIZE 7   m   1          6       -       0       -     13.00 (  0.73- 230.76)
*SPEIZE 10  f   1          5       -       5       -      6.33 (  1.83-  21.88)
 Subtotal SPEIZE                                          7.09 (  2.27-  22.14)
*TODD   8   m   0          6     316       1     520      9.87 (  1.19-  81.63)
*TVERDA 5   m   2          5       -       7       -      1.86 (  0.59-   5.88)
 VONHER 9   m   0         50     121      63     911      5.98 (  3.94-   9.07)
 VONHER 16  f   0         28     172     137    2888      3.43 (  2.22-   5.30)
 Subtotal VONHER                                          4.58 (  3.39-   6.19)
 WEISS  6   m   0          2      13       2      34      2.62 (  0.33-  20.55)
*WEN    2   m   1         27       -      52       -      1.83 (  1.07-   3.12)
 YAMAGU 4   b   6          -       -     144       -      1.96 (  1.53-   2.53)
*YUAN   6   m   1          9       -      19       -      0.80 (  0.36-   1.77)
Partial Totals          1330    5075    2540   15526
*prospective study                                        ~ With 0.5 adjustment for zero


  ________________________________________________________________________________________________________________________
                                            International Evidence on Smoking and COPD, Phase 3, Analysis run on 28-SEP-10

                                                   Table 1 - E - 1 - 5

                                IESCOPD - Meta-analysis of amount smoked : key value (1) 5
                            Any COPD, cigarettes (or any product if cigarettes not available)
                                                      Least-adjusted


 REF    NRR SEX ADJ             Ys       Ws       Qs       Ps
 ANDER1 11  m   1              1.03     1.05     0.01       0.29
*BEST   38  m   1              1.80     4.89     3.53       0.00
 CHEN2  13  m   6              0.54     2.52     0.42       0.39
 CHEN2  16  f   6              0.96     8.43     0.00       0.01
 Subtotal CHEN2                0.86    10.95     0.42
 CLEMEN 4   m   0              0.24     6.41     3.26       0.55
 DEAN1  19  m   0              1.41    26.70     5.69       0.00
 DEAN1  59  f   0             -0.42    19.59    36.71       0.07
 Subtotal DEAN1                0.64    46.29    42.40
*DOLL1  7   m   2              2.15    10.11    14.54       0.00
*DOLL2  4   f   1              2.35     0.75     1.47       0.04
*ENSTRO 7   m   1              1.04    26.24     0.22       0.00
*ENSTRO 16  f   1              0.49    41.72     8.74       0.00
 Subtotal ENSTRO               0.71    67.96     8.96
 FERRI2 26  m   1              0.94     5.19     0.00       0.03
 FERRI2 43  f   1              0.46     8.73     2.08       0.17
 Subtotal FERRI2               0.64    13.92     2.09
*GODTFR 6   b   3              1.39    46.38     9.04       0.00
 HOZAWA 4   b   0              0.84   168.29     2.18       0.00
 HUHTI1 21  m   0              2.54     5.28    13.29       0.00
 HUHTI1 31  f   0              0.55     7.45     1.18       0.13
 Subtotal HUHTI1               1.38    12.73    14.47
 HUHTI3 7   m   0              2.49     2.74     6.51       0.00
*JACOBS 18  m   2              1.52    11.92     3.84       0.00
*KAHN2  99  m   1              1.42    14.68     3.22       0.00
*KRZYZA 7   m   1              0.81     3.52     0.07       0.13
*KRZYZA 10  f   1              0.34     6.54     2.48       0.39
 Subtotal KRZYZA               0.50    10.06     2.56
*LEE    8   m   0              1.98     0.81     0.85       0.08
*LEE    43  f   0              0.68     1.44     0.10       0.41
 Subtotal LEE                  1.15     2.24     0.95
 LINDST 11  b   5              0.66    49.24     4.13       0.00
 LUNDB1 11  b   4              1.86    10.06     8.33       0.00
 MUELLE 20  m   0              1.56     1.24     0.45       0.08
*NILSSO 10  m   2              1.19    11.70     0.68       0.00
*NILSSO 15  f   2              1.34    12.84     1.88       0.00
 Subtotal NILSSO               1.27    24.54     2.56
*PETO   4   m   0              3.36     0.50     2.88       0.02
*SPEIZE 7   m   1              2.56     0.46     1.21       0.08
*SPEIZE 10  f   1              1.85     2.50     1.99       0.00
 Subtotal SPEIZE               1.96     2.96     3.20
*TODD   8   m   0              2.29     0.86     1.54       0.03
*TVERDA 5   m   2              0.62     2.91     0.32       0.29
 VONHER 9   m   0              1.79    22.11    15.43       0.00
 VONHER 16  f   0              1.23    20.34     1.60       0.00
 Subtotal VONHER               1.52    42.44    17.03
 WEISS  6   m   0              0.96     0.90     0.00       0.36
*WEN    2   m   1              0.60    13.42     1.62       0.03
 YAMAGU 4   b   6              0.67    60.74     4.74       0.00
*YUAN   6   m   1             -0.22     6.06     8.37       0.58

                       N       40
                      NS       30


                      Wt   647.22
                 Het Chi   174.61
                 Het  df       39
                 Het  P       ***
               Fixed  RR     2.59
                     RRl     2.40
                     RRu     2.80
                      P       +++
              Random  RR     2.96
                     RRl     2.43
                     RRu     3.60
                      P       +++
               Asymm  P       (*)

  ________________________________________________________________________________________________________________________
                                            International Evidence on Smoking and COPD, Phase 3, Analysis run on 28-SEP-10

                                                   Table 1 - E - 1 - 6

                                IESCOPD - Meta-analysis of amount smoked : key value (1) 5
                            Any COPD, cigarettes (or any product if cigarettes not available)
                                                      Least-adjusted


                       N       40
                      NS       30


                      Wt   647.22
                 Het Chi   174.61
                 Het  df       39
                 Het  P       ***
               Fixed  RR     2.59
                     RRl     2.40
                     RRu     2.80
                      P       +++
              Random  RR     2.96
                     RRl     2.43
                     RRu     3.60
                      P       +++
               Asymm  P       (*)

                                   Sex
                             both      male    female     Total


                       N        5        24        11        40
                      NS        5        24        11        40


                      Wt   334.71    182.20    130.32    647.22
                 Het Chi    27.13     65.23     43.65    174.61
                 Het  df        4        23        10        39
                 Het  P       ***       ***       ***       ***
               Fixed  RR     2.44      3.69      1.85      2.59
                     RRl     2.19      3.19      1.56      2.40
                     RRu     2.71      4.27      2.20      2.80
                      P       +++       +++       +++       +++
              Random  RR     2.75      3.67      2.10      2.96
                     RRl     2.01      2.77      1.40      2.43
                     RRu     3.77      4.88      3.16      3.60
                      P       +++       +++       +++       +++
             Between Chi                                  38.59
             Between  df                                      2
             Between  P                                     ***
             Btwn(F)  P                                      **

                                        Continent
                            NAmer    Europe      Asia  oth/mult     Total


                       N       14        22         3         1        40
                      NS       10        16         3         1        30


                      Wt   286.83    268.25     80.22     11.92    647.22
                 Het Chi    21.63    123.29      4.43      0.00    174.61
                 Het  df       13        21         2         0        39
                 Het  P       (*)       ***      N.S.      N.S.       ***
               Fixed  RR     2.36      3.10      1.81      4.57      2.59
                     RRl     2.11      2.75      1.45      2.59      2.40
                     RRu     2.65      3.50      2.25      8.06      2.80
                      P       +++       +++       +++       +++       +++
              Random  RR     2.58      3.46      1.61      4.57      2.96
                     RRl     2.07      2.48      1.06      2.59      2.43
                     RRu     3.21      4.82      2.47      8.06      3.60
                      P       +++       +++         +       +++       +++
             Between Chi                                            25.26
             Between  df                                                3
             Between  P                                               ***
             Btwn(F)  P                                              N.S.


  ________________________________________________________________________________________________________________________
                                            International Evidence on Smoking and COPD, Phase 3, Analysis run on 28-SEP-10

                                                   Table 1 - E - 1 - 6

                                IESCOPD - Meta-analysis of amount smoked : key value (1) 5
                            Any COPD, cigarettes (or any product if cigarettes not available)
                                                      Least-adjusted
                               Study type
                               CC        Pr        CS     Total


                       N        2        22        16        40
                      NS        1        17        12        30


                      Wt    46.29    226.64    374.29    647.22
                 Het Chi    37.86     68.78     59.98    174.61
                 Het  df        1        21        15        39
                 Het  P       ***       ***       ***       ***
               Fixed  RR     1.89      2.91      2.51      2.59
                     RRl     1.42      2.56      2.27      2.40
                     RRu     2.53      3.32      2.78      2.80
                      P       +++       +++       +++       +++
              Random  RR     1.65      3.06      3.09      2.96
                     RRl     0.27      2.31      2.36      2.43
                     RRu     9.93      4.05      4.04      3.60
                      P      N.S.       +++       +++       +++
             Between Chi                                   7.99
             Between  df                                      2
             Between  P                                       *
             Btwn(F)  P                                    N.S.

                          COPD subtype
                             mort        LF     other     Total


                       N       20         9        11        40
                      NS       15         8         7        30


                      Wt   210.08    260.43    176.71    647.22
                 Het Chi    99.39     24.31     41.90    174.61
                 Het  df       19         8        10        39
                 Het  P       ***        **       ***       ***
               Fixed  RR     2.60      2.30      3.08      2.59
                     RRl     2.27      2.04      2.66      2.40
                     RRu     2.97      2.60      3.57      2.80
                      P       +++       +++       +++       +++
              Random  RR     3.15      2.58      3.08      2.96
                     RRl     2.20      1.84      2.17      2.43
                     RRu     4.52      3.61      4.36      3.60
                      P       +++       +++       +++       +++
             Between Chi                                   9.00
             Between  df                                      2
             Between  P                                       *
             Btwn(F)  P                                    N.S.

                             Smoking product
                              any      cigs  cigsonly     Total


                       N        2        24        14        40
                      NS        2        18        10        30


                      Wt    49.12    436.64    161.46    647.22
                 Het Chi     3.13     81.95     76.08    174.61
                 Het  df        1        23        13        39
                 Het  P       (*)       ***       ***       ***
               Fixed  RR     4.28      2.48      2.51      2.59
                     RRl     3.24      2.26      2.15      2.40
                     RRu     5.67      2.72      2.92      2.80
                      P       +++       +++       +++       +++
              Random  RR     5.97      2.75      3.12      2.96
                     RRl     2.13      2.19      2.00      2.43
                     RRu    16.79      3.44      4.87      3.60
                      P       +++       +++       +++       +++
             Between Chi                                  13.45
             Between  df                                      2
             Between  P                                      **
             Btwn(F)  P                                    N.S.
  ________________________________________________________________________________________________________________________
                                            International Evidence on Smoking and COPD, Phase 3, Analysis run on 28-SEP-10

                                                   Table 1 - E - 1 - 6

                                IESCOPD - Meta-analysis of amount smoked : key value (1) 5
                            Any COPD, cigarettes (or any product if cigarettes not available)
                                                      Least-adjusted
                                     Unexposed group
                          nev any   nev cig  nev+ any  nev+ cig     Total


                       N       25        15                            40
                      NS       18        12                            30


                      Wt   300.13    347.09                        647.22
                 Het Chi   124.17     35.79                        174.61
                 Het  df       24        14                            39
                 Het  P       ***        **                           ***
               Fixed  RR     3.05      2.25                          2.59
                     RRl     2.72      2.03                          2.40
                     RRu     3.41      2.50                          2.80
                      P       +++       +++                           +++
              Random  RR     3.38      2.38                          2.96
                     RRl     2.52      1.88                          2.43
                     RRu     4.54      3.01                          3.60
                      P       +++       +++                           +++
             Between Chi                                            14.65
             Between  df                                                1
             Between  P                                               ***
             Btwn(F)  P                                               (*)

                        Unexposed group (combining nev+ with main levels)
                          nev any   nev cig     Total


                       N       25        15        40
                      NS       18        12        30


                      Wt   300.13    347.09    647.22
                 Het Chi   124.17     35.79    174.61
                 Het  df       24        14        39
                 Het  P       ***        **       ***
               Fixed  RR     3.05      2.25      2.59
                     RRl     2.72      2.03      2.40
                     RRu     3.41      2.50      2.80
                      P       +++       +++       +++
              Random  RR     3.38      2.38      2.96
                     RRl     2.52      1.88      2.43
                     RRu     4.54      3.01      3.60
                      P       +++       +++       +++
             Between Chi                        14.65
             Between  df                            1
             Between  P                           ***
             Btwn(F)  P                           (*)


  ________________________________________________________________________________________________________________________
                                            International Evidence on Smoking and COPD, Phase 3, Analysis run on 28-SEP-10

                                                   Table 1 - E - 1 - 7

                                IESCOPD - Meta-analysis of amount smoked : key value (1) 5
                            Any COPD, cigarettes (or any product if cigarettes not available)
                                 Excluded studies (and stage at which they were excluded)


1       CLARK COTTON  MEYER REMYJA RUTGER SNYDER SOBRAX     SU TAKEMU  WANG4   WEIR WHICKE ZALACA
2      ALDERS ANDER2 AUERBA   BANG  BECK1  BECK2 BJORNS  BROWN CERVER CHAPMA COATES COLLEG  DEAN2  DEANE DONTA2 DOPICO
       EHRLIC ENRIGH FINKLE FLETCH FOXMAN GOLDBE HAENSZ HARRIS  HAYES HIGGI2 HIGGI3 HIGGI6 HIRAYA HOLLA2 HOLLNA  HOUSE
       HRUBEC HUCHON JENSEN JINDA2  JOSHI JOUSI1   KATO KOTAN1  KUBIK LAMBER LANGE2 LANGHA LAVECC LUNDB2 MAGNUS MANFRE
       MELLST MENEZ1  MEREN MILLER  MILNE MOLLER   NAWA NEJJAR OGILVI  OMORI OSWAL1 OSWAL2 PANDEY  PRATT   REID RIMING
        RYDER SCHWAR  SHARP SHIMUR SOBRAD STJERN SUADIC SUTINE TAGER2 TROISI URRUTI VIEGI1 VIKGRE WAGEN2  WANG2    WIG
       WILHEL WILSO2  WOODS  WOOLF   ZOIA
3      VINEIS
4      ALESSA  AMIGO ANDER3 BEDNAR BROGGE  CHEN3  CHENG  COCCI DEJONG DEMARC DETORR DICKIN EKBERG  FIDAN FORAST FUKUCH
       GEIJER GULSVI HAMMO2 HARDIE HARIKK HEDMAN HIGGI4     HO HUHTI2 ITABAS JAENDI JOHANN KACHEL KARAKA KATANC KHOURY
          KIM  KIRAZ KLAYTO KOJIMA KOTAN2    LAI   LAM1   LAM2   LAM3  LANGE LINDBE   LIU2  MADOR MANNI1 MANNI2 MANNI3
       MARAN1 MARAN2 MARCUS MATHES MENEZ2 MENEZ3 MENEZ4 MENEZ5 MENEZ6 MONTNE NIEPSU NIHLEN   PEAT PELKON PEREZP  PRICE
       RENWIC RICCIO SARGEA SAWICK SHAHAB   SHIN SICHLE  SILVA STERLI  STROM  TAGER   TANG   THUN TRUPIN TSUSHI VESTBO
       VIEGI2 VOLLM1 VOLLM2   WALD WATSON WILSO1 WOJTYN   XIAO     XU ZIELI1 ZIELI2 ZIETKO
5       CHEN1 HAWTHO KULLER   LIU1
6      FERRI3 LEBOWI
7        KAHN
11     DONTA1 FERRI1   LIAW


  ________________________________________________________________________________________________________________________
                                            International Evidence on Smoking and COPD, Phase 3, Analysis run on 28-SEP-10

                                                   Table 1 - E - 1 - 8

                                IESCOPD - Meta-analysis of amount smoked : key value (1) 5
                            Any COPD, cigarettes (or any product if cigarettes not available)
                                             Potentially overlapping studies


     REF| REFGP|PRINC|                     OVERLAP|

  HOZAWA HOZAWA     1         ENRIGH/HOZAWA/HARIKK
  JACOBS JACOBS     1  JACOBS/DONTA1/DONTA2/PELKON
  LUNDB1 LUNDBA     1  LINDBE/LUNDB1/LUNDB2/HEDLUN
    PETO   PETO     1    PETO/HIGGI1/HIGGI2/HIGGI5
  HUHTI1 HUHTI1     1                HUHTI1/HUHTI2
  ENSTRO HAMMO2     2                HAMMO2/ENSTRO
  FERRI2 FERRIS     1         FERRI1/FERRI2/FERRI3
     WEN    WEN     1                     WEN/LIAW
    TODD   TODD     1                  LAMBER/TODD
  KRZYZA KRZYZA     1         SAWICK/KRZYZA/WOJTYN
  GODTFR GODTFR     1 GODT/VEST/LANG1+2/SUAD/HOLLN
   KAHN2   KAHN     2                   KAHN/KAHN2

                                    Most-adjusted - insufficient data for meta-analysis
     REF|NRR|SEX|AGEL|AGEH|     REGION|BEGYR|PUBYR|STTYP|ONSET|      DISEAS|ADJ|SMOKSTA|   PRODUCT|    UNEXP|LOW| HI|

    KAHN   3   m   31   99      Am:USA  1954  1966    Pr   Inc    COPD:mort   2 Current       Cigs   Nev any   1   9
          RR|SIG|

        4.84   ?


  ________________________________________________________________________________________________________________________
                                            International Evidence on Smoking and COPD, Phase 3, Analysis run on 28-SEP-10

                                                    Table 1 - E - 2 -

                               IESCOPD - Meta-analysis of amount smoked : key value (1) 20
                            Any COPD, cigarettes (or any product if cigarettes not available)


This analysis is restricted to results for:
1) Eligible study on database
2) Outcome COPD
3) Current or ever smoking
4) Categorical dose-response data for amount smoked
5) vs never smoking base
6) Key value (scheme 1) = 20
7) Results complete enough for use in meta-analysis

Within each study, results are then selected (in the following order of preference, within each sex) for:
8) SMKSTA  : current, ever
9) UNEXP   : never any, never cigarettes
10) PROD    : cigarettes, cigarettes only, any product
11) For overlapping studies: principal rather than subsidiary studies
and then for single sex results (m, f) in preference to results for both sexes combined (b).

Results adjusted for the most potential confounders are then chosen in Sections -1 to -3
and results adjusted for the least confounders in Sections -4 to -6. (Those least-adjusted results which
actually differ from the most-adjusted are marked 'x' in column X in Section -4)

Section -7 shows excluded studies, together with the stage (as above) at which no qualifying
results were found.

Section -8 lists the potentially overlapping studies which have been included (1=principal, 2=subsidiary),
and any results which would have been included in preference except that they had data not complete enough
for use in meta-analysis. It also lists their significance (yes/no), if known.


  ________________________________________________________________________________________________________________________
                                            International Evidence on Smoking and COPD, Phase 3, Analysis run on 28-SEP-10

                                                   Table 1 - E - 2 - 1

                               IESCOPD - Meta-analysis of amount smoked : key value (1) 20
                            Any COPD, cigarettes (or any product if cigarettes not available)
                                                      Most-adjusted


     REF|NRR|SEX|AGEL|AGEH|     REGION|BEGYR|PUBYR|STTYP|ONSET|      DISEAS|ADJ|SMOKSTA|   PRODUCT|    UNEXP|LOW| HI|

  ANDER1  12   m   25   74   Am:Canada  1963  1965    CS  Prev     COPD:oth   1 Current       Cigs   Nev any  15  24
    BEST  39   m   30   97   Am:Canada  1955  1967    Pr   Inc    COPD:mort   1 Current  Cigs only   Nev any  10  20
   DEAN1  25   m   35   99       Eu:UK  1969  1977    CC  Prev    COPD:mort   3 Current MCigs only   Nev any  13  22
   DEAN1  65   f   35   99       Eu:UK  1969  1977    CC  Prev    COPD:mort   3 Current MCigs only   Nev any  13  22
   DOLL1   8   m   20   99       Eu:UK  1951  1994    Pr   Inc    COPD:mort   2 Current  Cigs only   Nev any  15  24
   DOLL2   5   f   20   99       Eu:UK  1951  1980    Pr   Inc    COPD:mort   1 Current  Cigs only   Nev any  15  24
  ENSTRO   9   m   30   99      Am:USA  1960  2003    Pr   Inc    COPD:mort   1 Current  Cigs only   Nev any  20  20
  ENSTRO  18   f   30   99      Am:USA  1960  2003    Pr   Inc    COPD:mort   1 Current  Cigs only   Nev any  20  20
  FERRI2  27   m   25   80      Am:USA  1967  1971    CS  Prev     COPD:oth   1 Current       Cigs   Nev any  15  24
  FERRI2  44   f   25   80      Am:USA  1967  1971    CS  Prev     COPD:oth   1 Current       Cigs   Nev any  15  24
  HOZAWA   5   b   45   64      Am:USA  1987  2006    CS  Prev      COPD:LF   0 Current       Cigs  Nev cigs  15  29
  HUHTI1  27   m   40   64    Eu:Scand  1961  1965    CS  Prev    CB/EM/Ast   1 Current       Cigs   Nev any  15  24
  HUHTI3  13   m   25   69    Eu:Scand  1968  1978    CS  Prev      COPD:LF   1 Current        Any   Nev any  15  24
  JACOBS  20   m   40   84       Multi  1957  1999    Pr   Inc    COPD:mort   2 Current       Cigs  Nev cigs  20  29
   KAHN2 100   m   31   84      Am:USA  1954  1966    Pr   Inc    COPD:mort   1 Current       Cigs   Nev any  10  20
     LEE  24   m   35   82       Eu:UK  1964  1979    Pr   Inc    COPD:mort   1 Current  Cigs only   Nev any  20  20
     LEE  45   f   35   82       Eu:UK  1964  1979    Pr   Inc    COPD:mort   0 Current  Cigs only   Nev any  20  20
  MUELLE  26   m   20   69      Am:USA  1967  1971    CS  Prev      COPD:LF   1 Current       Cigs   Nev any  15  24
    TODD  24   m   35   81       Eu:UK  1965  1978    Pr   Inc    COPD:mort   1 Current  Cigs only   Nev any  20  20
  VONHER  11   m   30   99    Eu:Scand  1978  2000    CS  Prev        CB/EM   0 Current       Cigs   Nev any  20  29
  VONHER  18   f   30   99    Eu:Scand  1978  2000    CS  Prev        CB/EM   0 Current       Cigs   Nev any  20  29
   WEISS   7   m   50   69      Am:USA  1961  1963    CS  Prev      COPD:LF   0 Current  Cigs only   Nev any  10  20
     WEN   3   m   35   99   Asia:FarE  1982  2004    Pr   Inc    COPD:mort   1 Current       Cigs  Nev cigs  11  20


  ________________________________________________________________________________________________________________________
                                            International Evidence on Smoking and COPD, Phase 3, Analysis run on 28-SEP-10

                                                   Table 1 - E - 2 - 2

                               IESCOPD - Meta-analysis of amount smoked : key value (1) 20
                            Any COPD, cigarettes (or any product if cigarettes not available)
                                                      Most-adjusted


                        Number Exposed  Non-exposed
 REF    NRR SEX ADJ     Case    Cont    Case    Cont      RR        95.00%CI
 ANDER1 12  m   1          8       -       2       -      5.93 (  1.14-  30.72)
*BEST   39  m   1         70       -       6       -      9.97 (  4.33-  22.94)
 DEAN1  25  m   3         75       -      47       -      2.56 (  1.64-   4.01)
 DEAN1  65  f   3         21       -     120       -      2.23 (  1.26-   3.95)
 Subtotal DEAN1                                           2.43 (  1.71-   3.45)
*DOLL1  8   m   2         61       -      12       -     11.20 (  6.06-  20.68)
*DOLL2  5   f   1          4       -       1       -     28.50 (  3.19- 255.00)
*ENSTRO 9   m   1        326       -     103       -      8.30 (  6.62-  10.40)
*ENSTRO 18  f   1        309       -     296       -      9.32 (  7.85-  11.06)
 Subtotal ENSTRO                                          8.93 (  7.79-  10.24)
 FERRI2 27  m   1         55       -      11       -      4.99 (  2.42-  10.31)
 FERRI2 44  f   1         28       -      54       -      2.89 (  1.72-   4.87)
 Subtotal FERRI2                                          3.48 (  2.28-   5.31)
 HOZAWA 5   b   0        822    1020     872    5019      4.64 (  4.13-   5.21)
 HUHTI1 27  m   1         86       -       7       -     11.82 (  5.14-  27.19)
 HUHTI3 13  m   1         14       -       3       -      5.92 (  1.80-  19.47)
*JACOBS 20  m   2         61       -      28       -      4.00 (  2.43-   6.58)
*KAHN2  100 m   1        162       -      31       -      8.73 (  5.94-  12.82)
*LEE    24  m   1          7       -       1       -      7.54 (  0.93-  60.98)
*LEE    45  f   0          0     212       5    1694      0.72~(  0.04-  13.06)
 Subtotal LEE                                             3.37 (  0.62-  18.37)
 MUELLE 26  m   1         14       -       2       -      6.82 (  1.41-  33.04)
*TODD   24  m   1          8       -       1       -      9.31 (  1.17-  74.18)
 VONHER 11  m   0        217     174      63     911     18.03 ( 13.04-  24.93)
 VONHER 18  f   0         40      65     137    2888     12.97 (  8.44-  19.93)
 Subtotal VONHER                                         16.00 ( 12.36-  20.73)
 WEISS  7   m   0          8      42       2      34      3.24 (  0.64-  16.27)
*WEN    3   m   1         62       -      52       -      2.65 (  1.68-   4.18)
Partial Totals          2458    1513    1856   10546
*prospective study                                        ~ With 0.5 adjustment for zero


 REF    NRR SEX ADJ             Ys       Ws       Qs       Ps
 ANDER1 12  m   1              1.78     1.42     0.00       0.03
*BEST   39  m   1              2.30     5.53     1.21       0.00
 DEAN1  25  m   3              0.94    19.22    15.27       0.00
 DEAN1  65  f   3              0.80    11.77    12.47       0.01
 Subtotal DEAN1                0.89    30.99    27.75
*DOLL1  8   m   2              2.42    10.20     3.48       0.00
*DOLL2  5   f   1              3.35     0.80     1.85       0.00
*ENSTRO 9   m   1              2.12    75.31     6.11       0.00
*ENSTRO 18  f   1              2.23   130.74    21.00       0.00
 Subtotal ENSTRO               2.19   206.05    27.11
 FERRI2 27  m   1              1.61     7.31     0.37       0.00
 FERRI2 44  f   1              1.06    14.19     8.41       0.00
 Subtotal FERRI2               1.25    21.50     8.78
 HOZAWA 5   b   0              1.53   282.25    24.90       0.00
 HUHTI1 27  m   1              2.47     5.54     2.26       0.00
 HUHTI3 13  m   1              1.78     2.71     0.01       0.00
*JACOBS 20  m   2              1.39    15.49     3.07       0.00
*KAHN2  100 m   1              2.17    25.96     2.92       0.00
*LEE    24  m   1              2.02     0.88     0.03       0.06
*LEE    45  f   0             -0.32     0.46     2.13       0.83
 Subtotal LEE                  1.22     1.34     2.16
 MUELLE 26  m   1              1.92     1.54     0.01       0.02
*TODD   24  m   1              2.23     0.89     0.14       0.04
 VONHER 11  m   0              2.89    36.60    41.18       0.00
 VONHER 18  f   0              2.56    20.82    11.14       0.00
 Subtotal VONHER               2.77    57.42    52.32
 WEISS  7   m   0              1.17     1.47     0.64       0.15
*WEN    3   m   1              0.97    18.49    13.58       0.00


  ________________________________________________________________________________________________________________________
                                            International Evidence on Smoking and COPD, Phase 3, Analysis run on 28-SEP-10

                                                   Table 1 - E - 2 - 2

                               IESCOPD - Meta-analysis of amount smoked : key value (1) 20
                            Any COPD, cigarettes (or any product if cigarettes not available)
                                                      Most-adjusted


                       N       23
                      NS       18


                      Wt   689.59
                 Het Chi   172.19
                 Het  df       22
                 Het  P       ***
               Fixed  RR     6.24
                     RRl     5.79
                     RRu     6.73
                      P       +++
              Random  RR     6.21
                     RRl     4.72
                     RRu     8.17
                      P       +++
               Asymm  P      N.S.


  ________________________________________________________________________________________________________________________
                                            International Evidence on Smoking and COPD, Phase 3, Analysis run on 28-SEP-10

                                                   Table 1 - E - 2 - 3

                               IESCOPD - Meta-analysis of amount smoked : key value (1) 20
                            Any COPD, cigarettes (or any product if cigarettes not available)
                                                      Most-adjusted


                       N       23
                      NS       18


                      Wt   689.59
                 Het Chi   172.19
                 Het  df       22
                 Het  P       ***
               Fixed  RR     6.24
                     RRl     5.79
                     RRu     6.73
                      P       +++
              Random  RR     6.21
                     RRl     4.72
                     RRu     8.17
                      P       +++
               Asymm  P      N.S.

                                   Sex
                             both      male    female     Total


                       N        1        16         6        23
                      NS        1        16         6        23


                      Wt   282.25    228.56    178.78    689.59
                 Het Chi     0.00     83.62     45.77    172.19
                 Het  df        0        15         5        22
                 Het  P      N.S.       ***       ***       ***
               Fixed  RR     4.64      7.41      8.02      6.24
                     RRl     4.13      6.50      6.93      5.79
                     RRu     5.21      8.43      9.29      6.73
                      P       +++       +++       +++       +++
              Random  RR     4.64      6.62      5.57      6.21
                     RRl     4.13      4.58      2.79      4.72
                     RRu     5.21      9.56     11.16      8.17
                      P       +++       +++       +++       +++
             Between Chi                                  42.80
             Between  df                                      2
             Between  P                                     ***
             Btwn(F)  P                                     (*)

                                        Continent
                            NAmer    Europe      Asia  oth/mult     Total


                       N       10        11         1         1        23
                      NS        8         8         1         1        18


                      Wt   545.73    109.88     18.49     15.49    689.59
                 Het Chi    65.30     78.88      0.00      0.00    172.19
                 Het  df        9        10         0         0        22
                 Het  P       ***       ***      N.S.      N.S.       ***
               Fixed  RR     6.11      8.58      2.65      4.00      6.24
                     RRl     5.61      7.11      1.68      2.43      5.79
                     RRu     6.64     10.34      4.18      6.58      6.73
                      P       +++       +++       +++       +++       +++
              Random  RR     6.32      7.32      2.65      4.00      6.21
                     RRl     4.59      3.90      1.68      2.43      4.72
                     RRu     8.71     13.74      4.18      6.58      8.17
                      P       +++       +++       +++       +++       +++
             Between Chi                                            28.00
             Between  df                                                3
             Between  P                                               ***
             Btwn(F)  P                                              N.S.


  ________________________________________________________________________________________________________________________
                                            International Evidence on Smoking and COPD, Phase 3, Analysis run on 28-SEP-10

                                                   Table 1 - E - 2 - 3

                               IESCOPD - Meta-analysis of amount smoked : key value (1) 20
                            Any COPD, cigarettes (or any product if cigarettes not available)
                                                      Most-adjusted
                        National cigarette tobacco type (excluding mixed/unkown)
                          blended  virginia     Total


                       N       13         9        22
                      NS       10         7        17


                      Wt   619.93     51.16    671.09
                 Het Chi   119.94     28.76    158.23
                 Het  df       12         8        21
                 Het  P       ***       ***       ***
               Fixed  RR     6.61      4.22      6.39
                     RRl     6.11      3.21      5.93
                     RRu     7.16      5.55      6.89
                      P       +++       +++       +++
              Random  RR     7.21      5.46      6.55
                     RRl     5.25      2.86      4.96
                     RRu     9.90     10.43      8.65
                      P       +++       +++       +++
             Between Chi                         9.54
             Between  df                            1
             Between  P                            **
             Btwn(F)  P                          N.S.

                                        Start year of study
                            <1970   1970-79   1980-89   1990-99     2000+   unknown     Total


                       N       19         2         2                                      23
                      NS       15         1         2                                      18


                      Wt   331.43     57.42    300.74                                  689.59
                 Het Chi    75.10      1.44      5.44                                  172.19
                 Het  df       18         1         1                                      22
                 Het  P       ***      N.S.         *                                     ***
               Fixed  RR     7.16     16.00      4.48                                    6.24
                     RRl     6.43     12.36      4.00                                    5.79
                     RRu     7.98     20.73      5.02                                    6.73
                      P       +++       +++       +++                                     +++
              Random  RR     5.86     15.78      3.67                                    6.21
                     RRl     4.37     11.50      2.13                                    4.72
                     RRu     7.86     21.67      6.30                                    8.17
                      P       +++       +++       +++                                     +++
             Between Chi                                                                90.21
             Between  df                                                                    2
             Between  P                                                                   ***
             Btwn(F)  P                                                                   ***

                                Publication year
                            <1980   1980-89   1990-99     2000+     Total


                       N       14         1         2         6        23
                      NS       11         1         2         4        18


                      Wt    98.90      0.80     25.68    564.21    689.59
                 Het Chi    37.67      0.00      6.52    116.50    172.19
                 Het  df       13         0         1         5        22
                 Het  P       ***      N.S.         *       ***       ***
               Fixed  RR     4.68     28.50      6.02      6.56      6.24
                     RRl     3.84      3.19      4.09      6.04      5.79
                     RRu     5.70    254.81      8.86      7.13      6.73
                      P       +++        ++       +++       +++       +++
              Random  RR     4.92     28.50      6.59      7.82      6.21
                     RRl     3.30      3.19      2.40      4.98      4.72
                     RRu     7.33    254.81     18.05     12.28      8.17
                      P       +++        ++       +++       +++       +++
             Between Chi                                            11.50
             Between  df                                                3
             Between  P                                                **
             Btwn(F)  P                                              N.S.
  ________________________________________________________________________________________________________________________
                                            International Evidence on Smoking and COPD, Phase 3, Analysis run on 28-SEP-10

                                                   Table 1 - E - 2 - 3

                               IESCOPD - Meta-analysis of amount smoked : key value (1) 20
                            Any COPD, cigarettes (or any product if cigarettes not available)
                                                      Most-adjusted
                               Study type
                               CC        Pr        CS     Total


                       N        2        11        10        23
                      NS        1         9         8        18


                      Wt    30.99    284.75    373.85    689.59
                 Het Chi     0.14     38.74     84.67    172.19
                 Het  df        1        10         9        22
                 Het  P      N.S.       ***       ***       ***
               Fixed  RR     2.43      7.96      5.61      6.24
                     RRl     1.71      7.08      5.07      5.79
                     RRu     3.45      8.94      6.21      6.73
                      P       +++       +++       +++       +++
              Random  RR     2.43      6.99      6.90      6.21
                     RRl     1.71      5.09      4.12      4.72
                     RRu     3.45      9.59     11.55      8.17
                      P       +++       +++       +++       +++
             Between Chi                                  48.63
             Between  df                                      2
             Between  P                                     ***
             Btwn(F)  P                                       *

                                    Lowest age in RR
                        <25/unlim     25-39       40+   unknown     Total


                       N        3        16         4                  23
                      NS        3        11         4                  18


                      Wt    12.54    372.30    304.75              689.59
                 Het Chi     1.08    118.42      5.36              172.19
                 Het  df        2        15         3                  22
                 Het  P      N.S.       ***      N.S.                 ***
               Fixed  RR    11.18      7.76      4.67                6.24
                     RRl     6.43      7.01      4.18                5.79
                     RRu    19.45      8.59      5.23                6.73
                      P       +++       +++       +++                 +++
              Random  RR    11.18      5.99      4.99                6.21
                     RRl     6.43      4.23      3.50                4.72
                     RRu    19.45      8.50      7.10                8.17
                      P       +++       +++       +++                 +++
             Between Chi                                            47.33
             Between  df                                                2
             Between  P                                               ***
             Btwn(F)  P                                                 *

                                         Highest age in RR
                              <65     65-74     75-84 85+/unlim   unknown     Total


                       N        2         4         7        10                  23
                      NS        2         4         5         7                  18


                      Wt   287.79      7.15     65.18    329.48              689.59
                 Het Chi     4.75      0.51     15.14    101.29              172.19
                 Het  df        1         3         6         9                  22
                 Het  P         *      N.S.         *       ***                 ***
               Fixed  RR     4.72      5.39      5.26      8.27                6.24
                     RRl     4.21      2.59      4.12      7.42                5.79
                     RRu     5.30     11.22      6.70      9.21                6.73
                      P       +++       +++       +++       +++                 +++
              Random  RR     6.74      5.39      4.83      7.12                6.21
                     RRl     2.74      2.59      3.00      4.68                4.72
                     RRu    16.53     11.22      7.78     10.84                8.17
                      P       +++       +++       +++       +++                 +++
             Between Chi                                                      50.50
             Between  df                                                          3
             Between  P                                                         ***
             Btwn(F)  P                                                         (*)
  ________________________________________________________________________________________________________________________
                                            International Evidence on Smoking and COPD, Phase 3, Analysis run on 28-SEP-10

                                                   Table 1 - E - 2 - 3

                               IESCOPD - Meta-analysis of amount smoked : key value (1) 20
                            Any COPD, cigarettes (or any product if cigarettes not available)
                                                      Most-adjusted
                           Study weakness
                              Yes        No     Total


                       N        2        21        23
                      NS        1        17        18


                      Wt    30.99    658.60    689.59
                 Het Chi     0.14    143.14    172.19
                 Het  df        1        20        22
                 Het  P      N.S.       ***       ***
               Fixed  RR     2.43      6.53      6.24
                     RRl     1.71      6.05      5.79
                     RRu     3.45      7.04      6.73
                      P       +++       +++       +++
              Random  RR     2.43      7.02      6.21
                     RRl     1.71      5.31      4.72
                     RRu     3.45      9.27      8.17
                      P       +++       +++       +++
             Between Chi                        28.91
             Between  df                            1
             Between  P                           ***
             Btwn(F)  P                           (*)

                          COPD subtype
                             mort        LF     other     Total


                       N       13         4         6        23
                      NS       10         4         4        18


                      Wt   315.74    287.98     85.87    689.59
                 Het Chi    78.23      0.58     39.83    172.19
                 Het  df       12         3         5        22
                 Het  P       ***      N.S.       ***       ***
               Fixed  RR     7.08      4.65     10.54      6.24
                     RRl     6.34      4.14      8.53      5.79
                     RRu     7.91      5.22     13.02      6.73
                      P       +++       +++       +++       +++
              Random  RR     5.66      4.65      8.17      6.21
                     RRl     3.93      4.14      4.17      4.72
                     RRu     8.14      5.22     16.01      8.17
                      P       +++       +++       +++       +++
             Between Chi                                  53.54
             Between  df                                      2
             Between  P                                     ***
             Btwn(F)  P                                       *

                               Asthma analysis type (COPD)
                        inc-irres  excl-all defn-incl     other     Total


                       N       12                   8         3        23
                      NS       10                   5         3        18


                      Wt   397.61              265.50     26.48    689.59
                 Het Chi    94.67               61.15      8.40    172.19
                 Het  df       11                   7         2        22
                 Het  P       ***                 ***         *       ***
               Fixed  RR     5.71                7.14      6.31      6.24
                     RRl     5.17                6.33      4.31      5.79
                     RRu     6.29                8.05      9.23      6.73
                      P       +++                 +++       +++       +++
              Random  RR     6.95                5.03      8.07      6.21
                     RRl     4.31                3.23      3.10      4.72
                     RRu    11.19                7.83     21.06      8.17
                      P       +++                 +++       +++       +++
             Between Chi                                             7.97
             Between  df                                                2
             Between  P                                                 *
             Btwn(F)  P                                              N.S.
  ________________________________________________________________________________________________________________________
                                            International Evidence on Smoking and COPD, Phase 3, Analysis run on 28-SEP-10

                                                   Table 1 - E - 2 - 3

                               IESCOPD - Meta-analysis of amount smoked : key value (1) 20
                            Any COPD, cigarettes (or any product if cigarettes not available)
                                                      Most-adjusted
                        Bronchodilator/reversibility (LF only)
                          no/unkn  yes/revs     Total


                       N        4                   4
                      NS        4                   4


                      Wt   287.98              287.98
                 Het Chi     0.58                0.58
                 Het  df        3                   3
                 Het  P      N.S.                N.S.
               Fixed  RR     4.65                4.65
                     RRl     4.14                4.14
                     RRu     5.22                5.22
                      P       +++                 +++
              Random  RR     4.65                4.65
                     RRl     4.14                4.14
                     RRu     5.22                5.22
                      P       +++                 +++
             Between Chi
             Between  df
             Between  P                          N.S.
             Btwn(F)  P                          N.S.

                                  Number of COPD cases
                             1-50    51-100   101-200      201+     Total


                       N        3         5         2        13        23
                      NS        3         4         2         9        18


                      Wt     3.82      6.36     24.02    655.39    689.59
                 Het Chi     2.45      2.24      7.47    152.31    172.19
                 Het  df        2         4         1        12        22
                 Het  P      N.S.      N.S.        **       ***       ***
               Fixed  RR     6.90      5.61      3.59      6.37      6.24
                     RRl     2.53      2.58      2.41      5.90      5.79
                     RRu    18.82     12.20      5.36      6.88      6.73
                      P       +++       +++       +++       +++       +++
              Random  RR     7.13      5.61      4.90      6.48      6.21
                     RRl     2.32      2.58      1.34      4.72      4.72
                     RRu    21.86     12.20     17.89      8.90      8.17
                      P       +++       +++         +       +++       +++
             Between Chi                                             7.71
             Between  df                                                3
             Between  P                                               (*)
             Btwn(F)  P                                              N.S.

                        Number of COPD cases (excluding unknown)


                       N        3         5         2        13        23
                      NS        3         4         2         9        18


                      Wt     3.82      6.36     24.02    655.39    689.59
                 Het Chi     2.45      2.24      7.47    152.31    172.19
                 Het  df        2         4         1        12        22
                 Het  P      N.S.      N.S.        **       ***       ***
               Fixed  RR     6.90      5.61      3.59      6.37      6.24
                     RRl     2.53      2.58      2.41      5.90      5.79
                     RRu    18.82     12.20      5.36      6.88      6.73
                      P       +++       +++       +++       +++       +++
              Random  RR     7.13      5.61      4.90      6.48      6.21
                     RRl     2.32      2.58      1.34      4.72      4.72
                     RRu    21.86     12.20     17.89      8.90      8.17
                      P       +++       +++         +       +++       +++
             Between Chi                                             7.71
             Between  df                                                3
             Between  P                                               (*)
             Btwn(F)  P                                              N.S.

  ________________________________________________________________________________________________________________________
                                            International Evidence on Smoking and COPD, Phase 3, Analysis run on 28-SEP-10

                                                   Table 1 - E - 2 - 3

                               IESCOPD - Meta-analysis of amount smoked : key value (1) 20
                            Any COPD, cigarettes (or any product if cigarettes not available)
                                                      Most-adjusted
                            Analysis type
                         prevlnce     onset     Total


                       N       12        11        23
                      NS        9         9        18


                      Wt   404.84    284.75    689.59
                 Het Chi   104.87     38.74    172.19
                 Het  df       11        10        22
                 Het  P       ***       ***       ***
               Fixed  RR     5.26      7.96      6.24
                     RRl     4.77      7.08      5.79
                     RRu     5.80      8.94      6.73
                      P       +++       +++       +++
              Random  RR     5.59      6.99      6.21
                     RRl     3.56      5.09      4.72
                     RRu     8.79      9.59      8.17
                      P       +++       +++       +++
             Between Chi                        28.57
             Between  df                            1
             Between  P                           ***
             Btwn(F)  P                           (*)

                             Smoking product
                              any      cigs  cigsonly     Total


                       N        1        11        11        23
                      NS        1         9         8        18


                      Wt     2.71    429.61    257.27    689.59
                 Het Chi     0.00    101.36     53.45    172.19
                 Het  df        0        10        10        22
                 Het  P      N.S.       ***       ***       ***
               Fixed  RR     5.92      5.52      7.67      6.24
                     RRl     1.80      5.02      6.79      5.79
                     RRu    19.47      6.07      8.67      6.73
                      P        ++       +++       +++       +++
              Random  RR     5.92      6.36      6.03      6.21
                     RRl     1.80      4.18      3.97      4.72
                     RRu    19.47      9.66      9.16      8.17
                      P        ++       +++       +++       +++
             Between Chi                                  17.38
             Between  df                                      2
             Between  P                                     ***
             Btwn(F)  P                                    N.S.

                                     Unexposed group
                          nev any   nev cig  nev+ any  nev+ cig     Total


                       N       20         3                            23
                      NS       15         3                            18


                      Wt   373.36    316.23                        689.59
                 Het Chi   100.22      5.63                        172.19
                 Het  df       19         2                            22
                 Het  P       ***       (*)                           ***
               Fixed  RR     8.31      4.46                          6.24
                     RRl     7.50      3.99                          5.79
                     RRu     9.19      4.98                          6.73
                      P       +++       +++                           +++
              Random  RR     6.98      3.85                          6.21
                     RRl     5.18      2.74                          4.72
                     RRu     9.42      5.41                          8.17
                      P       +++       +++                           +++
             Between Chi                                            66.34
             Between  df                                                1
             Between  P                                               ***
             Btwn(F)  P                                                **
  ________________________________________________________________________________________________________________________
                                            International Evidence on Smoking and COPD, Phase 3, Analysis run on 28-SEP-10

                                                   Table 1 - E - 2 - 3

                               IESCOPD - Meta-analysis of amount smoked : key value (1) 20
                            Any COPD, cigarettes (or any product if cigarettes not available)
                                                      Most-adjusted
                        Unexposed group (combining nev+ with main levels)
                          nev any   nev cig     Total


                       N       20         3        23
                      NS       15         3        18


                      Wt   373.36    316.23    689.59
                 Het Chi   100.22      5.63    172.19
                 Het  df       19         2        22
                 Het  P       ***       (*)       ***
               Fixed  RR     8.31      4.46      6.24
                     RRl     7.50      3.99      5.79
                     RRu     9.19      4.98      6.73
                      P       +++       +++       +++
              Random  RR     6.98      3.85      6.21
                     RRl     5.18      2.74      4.72
                     RRu     9.42      5.41      8.17
                      P       +++       +++       +++
             Between Chi                        66.34
             Between  df                            1
             Between  P                           ***
             Btwn(F)  P                            **

                        Number of adjustment variables
                                0         1        2+     Total


                       N        5        14         4        23
                      NS        4        12         3        19


                      Wt   341.60    291.32     56.67    689.59
                 Het Chi    77.04     44.73     18.23    172.19
                 Het  df        4        13         3        22
                 Het  P       ***       ***       ***       ***
               Fixed  RR     5.69      7.72      3.67      6.24
                     RRl     5.12      6.88      2.83      5.79
                     RRu     6.33      8.66      4.76      6.73
                      P       +++       +++       +++       +++
              Random  RR     7.25      6.60      3.94      6.21
                     RRl     3.06      4.90      2.06      4.72
                     RRu    17.19      8.91      7.54      8.17
                      P       +++       +++       +++       +++
             Between Chi                                  32.19
             Between  df                                      2
             Between  P                                     ***
             Btwn(F)  P                                    N.S.


  ________________________________________________________________________________________________________________________
                                            International Evidence on Smoking and COPD, Phase 3, Analysis run on 28-SEP-10

                                                   Table 1 - E - 2 - 4

                               IESCOPD - Meta-analysis of amount smoked : key value (1) 20
                            Any COPD, cigarettes (or any product if cigarettes not available)
                                                      Least-adjusted


     REF|NRR|X|SEX|AGEL|AGEH|     REGION|BEGYR|PUBYR|STTYP|ONSET|      DISEAS|ADJ|SMOKSTA|   PRODUCT|    UNEXP|LOW| HI|

  ANDER1  12     m   25   74   Am:Canada  1963  1965    CS  Prev     COPD:oth   1 Current       Cigs   Nev any  15  24
    BEST  39     m   30   97   Am:Canada  1955  1967    Pr   Inc    COPD:mort   1 Current  Cigs only   Nev any  10  20
   DEAN1  20 x   m   35   99       Eu:UK  1969  1977    CC  Prev    COPD:mort   0 Current MCigs only   Nev any  13  22
   DEAN1  60 x   f   35   99       Eu:UK  1969  1977    CC  Prev    COPD:mort   0 Current MCigs only   Nev any  13  22
   DOLL1   8     m   20   99       Eu:UK  1951  1994    Pr   Inc    COPD:mort   2 Current  Cigs only   Nev any  15  24
   DOLL2   5     f   20   99       Eu:UK  1951  1980    Pr   Inc    COPD:mort   1 Current  Cigs only   Nev any  15  24
  ENSTRO   9     m   30   99      Am:USA  1960  2003    Pr   Inc    COPD:mort   1 Current  Cigs only   Nev any  20  20
  ENSTRO  18     f   30   99      Am:USA  1960  2003    Pr   Inc    COPD:mort   1 Current  Cigs only   Nev any  20  20
  FERRI2  27     m   25   80      Am:USA  1967  1971    CS  Prev     COPD:oth   1 Current       Cigs   Nev any  15  24
  FERRI2  44     f   25   80      Am:USA  1967  1971    CS  Prev     COPD:oth   1 Current       Cigs   Nev any  15  24
  HOZAWA   5     b   45   64      Am:USA  1987  2006    CS  Prev      COPD:LF   0 Current       Cigs  Nev cigs  15  29
  HUHTI1  22 x   m   40   64    Eu:Scand  1961  1965    CS  Prev    CB/EM/Ast   0 Current       Cigs   Nev any  15  24
  HUHTI3   8 x   m   25   69    Eu:Scand  1968  1978    CS  Prev      COPD:LF   0 Current        Any   Nev any  15  24
  JACOBS  20     m   40   84       Multi  1957  1999    Pr   Inc    COPD:mort   2 Current       Cigs  Nev cigs  20  29
   KAHN2 100     m   31   84      Am:USA  1954  1966    Pr   Inc    COPD:mort   1 Current       Cigs   Nev any  10  20
     LEE  10 x   m   35   82       Eu:UK  1964  1979    Pr   Inc    COPD:mort   0 Current  Cigs only   Nev any  20  20
     LEE  45     f   35   82       Eu:UK  1964  1979    Pr   Inc    COPD:mort   0 Current  Cigs only   Nev any  20  20
  MUELLE  21 x   m   20   69      Am:USA  1967  1971    CS  Prev      COPD:LF   0 Current       Cigs   Nev any  15  24
    TODD  10 x   m   35   81       Eu:UK  1965  1978    Pr   Inc    COPD:mort   0 Current  Cigs only   Nev any  20  20
  VONHER  11     m   30   99    Eu:Scand  1978  2000    CS  Prev        CB/EM   0 Current       Cigs   Nev any  20  29
  VONHER  18     f   30   99    Eu:Scand  1978  2000    CS  Prev        CB/EM   0 Current       Cigs   Nev any  20  29
   WEISS   7     m   50   69      Am:USA  1961  1963    CS  Prev      COPD:LF   0 Current  Cigs only   Nev any  10  20
     WEN   3     m   35   99   Asia:FarE  1982  2004    Pr   Inc    COPD:mort   1 Current       Cigs  Nev cigs  11  20


  ________________________________________________________________________________________________________________________
                                            International Evidence on Smoking and COPD, Phase 3, Analysis run on 28-SEP-10

                                                   Table 1 - E - 2 - 5

                               IESCOPD - Meta-analysis of amount smoked : key value (1) 20
                            Any COPD, cigarettes (or any product if cigarettes not available)
                                                      Least-adjusted


                        Number Exposed  Non-exposed
 REF    NRR SEX ADJ     Case    Cont    Case    Cont      RR        95.00%CI
 ANDER1 12  m   1          8       -       2       -      5.93 (  1.14-  30.72)
*BEST   39  m   1         70       -       6       -      9.97 (  4.33-  22.94)
 DEAN1  20  m   0         75     429      47     510      1.90 (  1.29-   2.79)
 DEAN1  60  f   0         21     521     120    1538      0.52 (  0.32-   0.83)
 Subtotal DEAN1                                           1.13 (  0.84-   1.52)
*DOLL1  8   m   2         61       -      12       -     11.20 (  6.06-  20.68)
*DOLL2  5   f   1          4       -       1       -     28.50 (  3.19- 255.00)
*ENSTRO 9   m   1        326       -     103       -      8.30 (  6.62-  10.40)
*ENSTRO 18  f   1        309       -     296       -      9.32 (  7.85-  11.06)
 Subtotal ENSTRO                                          8.93 (  7.79-  10.24)
 FERRI2 27  m   1         55       -      11       -      4.99 (  2.42-  10.31)
 FERRI2 44  f   1         28       -      54       -      2.89 (  1.72-   4.87)
 Subtotal FERRI2                                          3.48 (  2.28-   5.31)
 HOZAWA 5   b   0        822    1020     872    5019      4.64 (  4.13-   5.21)
 HUHTI1 22  m   0         86     105       7     115     13.46 (  5.96-  30.38)
 HUHTI3 8   m   0         14     187       3     236      5.89 (  1.67-  20.80)
*JACOBS 20  m   2         61       -      28       -      4.00 (  2.43-   6.58)
*KAHN2  100 m   1        162       -      31       -      8.73 (  5.94-  12.82)
*LEE    10  m   0          7     364       1     347      6.67 (  0.83-  53.96)
*LEE    45  f   0          0     212       5    1694      0.72~(  0.04-  13.06)
 Subtotal LEE                                             3.12 (  0.57-  16.95)
 MUELLE 21  m   0         14      50       2      57      7.98 (  1.73-  36.83)
*TODD   10  m   0          8     487       1     520      8.54 (  1.07-  68.05)
 VONHER 11  m   0        217     174      63     911     18.03 ( 13.04-  24.93)
 VONHER 18  f   0         40      65     137    2888     12.97 (  8.44-  19.93)
 Subtotal VONHER                                         16.00 ( 12.36-  20.73)
 WEISS  7   m   0          8      42       2      34      3.24 (  0.64-  16.27)
*WEN    3   m   1         62       -      52       -      2.65 (  1.68-   4.18)
Partial Totals          2458    3656    1856   13869
*prospective study                                        ~ With 0.5 adjustment for zero


 REF    NRR SEX ADJ             Ys       Ws       Qs       Ps
 ANDER1 12  m   1              1.78     1.42     0.00       0.03
*BEST   39  m   1              2.30     5.53     1.55       0.00
 DEAN1  20  m   0              0.64    25.71    32.82       0.00
 DEAN1  60  f   0             -0.66    17.09   100.96       0.01
 Subtotal DEAN1                0.12    42.79   133.77
*DOLL1  8   m   2              2.42    10.20     4.25       0.00
*DOLL2  5   f   1              3.35     0.80     2.00       0.00
*ENSTRO 9   m   1              2.12    75.31     9.02       0.00
*ENSTRO 18  f   1              2.23   130.74    27.90       0.00
 Subtotal ENSTRO               2.19   206.05    36.92
 FERRI2 27  m   1              1.61     7.31     0.19       0.00
 FERRI2 44  f   1              1.06    14.19     7.13       0.00
 Subtotal FERRI2               1.25    21.50     7.32
 HOZAWA 5   b   0              1.53   282.25    15.69       0.00
 HUHTI1 22  m   0              2.60     5.79     3.98       0.00
 HUHTI3 8   m   0              1.77     2.41     0.00       0.01
*JACOBS 20  m   2              1.39    15.49     2.28       0.00
*KAHN2  100 m   1              2.17    25.96     4.08       0.00
*LEE    10  m   0              1.90     0.88     0.01       0.08
*LEE    45  f   0             -0.32     0.46     2.01       0.83
 Subtotal LEE                  1.14     1.34     2.02
 MUELLE 21  m   0              2.08     1.64     0.15       0.01
*TODD   10  m   0              2.15     0.89     0.13       0.04
 VONHER 11  m   0              2.89    36.60    46.07       0.00
 VONHER 18  f   0              2.56    20.82    13.08       0.00
 Subtotal VONHER               2.77    57.42    59.16
 WEISS  7   m   0              1.17     1.47     0.52       0.15
*WEN    3   m   1              0.97    18.49    11.71       0.00


  ________________________________________________________________________________________________________________________
                                            International Evidence on Smoking and COPD, Phase 3, Analysis run on 28-SEP-10

                                                   Table 1 - E - 2 - 5

                               IESCOPD - Meta-analysis of amount smoked : key value (1) 20
                            Any COPD, cigarettes (or any product if cigarettes not available)
                                                      Least-adjusted


                       N       23
                      NS       18


                      Wt   701.45
                 Het Chi   285.55
                 Het  df       22
                 Het  P       ***
               Fixed  RR     5.87
                     RRl     5.45
                     RRu     6.32
                      P       +++
              Random  RR     5.63
                     RRl     4.02
                     RRu     7.90
                      P       +++
               Asymm  P      N.S.


  ________________________________________________________________________________________________________________________
                                            International Evidence on Smoking and COPD, Phase 3, Analysis run on 28-SEP-10

                                                   Table 1 - E - 2 - 6

                               IESCOPD - Meta-analysis of amount smoked : key value (1) 20
                            Any COPD, cigarettes (or any product if cigarettes not available)
                                                      Least-adjusted


                       N       23
                      NS       18


                      Wt   701.45
                 Het Chi   285.55
                 Het  df       22
                 Het  P       ***
               Fixed  RR     5.87
                     RRl     5.45
                     RRu     6.32
                      P       +++
              Random  RR     5.63
                     RRl     4.02
                     RRu     7.90
                      P       +++
               Asymm  P      N.S.

                                   Sex
                             both      male    female     Total


                       N        1        16         6        23
                      NS        1        16         6        23


                      Wt   282.25    235.10    184.10    701.45
                 Het Chi     0.00    109.65    149.51    285.55
                 Het  df        0        15         5        22
                 Het  P      N.S.       ***       ***       ***
               Fixed  RR     4.64      6.99      6.75      5.87
                     RRl     4.13      6.15      5.84      5.45
                     RRu     5.21      7.94      7.80      6.32
                      P       +++       +++       +++       +++
              Random  RR     4.64      6.50      4.06      5.63
                     RRl     4.13      4.31      1.29      4.02
                     RRu     5.21      9.79     12.79      7.90
                      P       +++       +++         +       +++
             Between Chi                                  26.39
             Between  df                                      2
             Between  P                                     ***
             Btwn(F)  P                                    N.S.

                                        Continent
                            NAmer    Europe      Asia  oth/mult     Total


                       N       10        11         1         1        23
                      NS        8         8         1         1        18


                      Wt   545.82    121.64     18.49     15.49    701.45
                 Het Chi    65.40    205.30      0.00      0.00    285.55
                 Het  df        9        10         0         0        22
                 Het  P       ***       ***      N.S.      N.S.       ***
               Fixed  RR     6.11      5.83      2.65      4.00      5.87
                     RRl     5.62      4.88      1.68      2.43      5.45
                     RRu     6.64      6.96      4.18      6.58      6.32
                      P       +++       +++       +++       +++       +++
              Random  RR     6.36      5.93      2.65      4.00      5.63
                     RRl     4.61      2.36      1.68      2.43      4.02
                     RRu     8.75     14.92      4.18      6.58      7.90
                      P       +++       +++       +++       +++       +++
             Between Chi                                            14.84
             Between  df                                                3
             Between  P                                                **
             Btwn(F)  P                                              N.S.


  ________________________________________________________________________________________________________________________
                                            International Evidence on Smoking and COPD, Phase 3, Analysis run on 28-SEP-10

                                                   Table 1 - E - 2 - 6

                               IESCOPD - Meta-analysis of amount smoked : key value (1) 20
                            Any COPD, cigarettes (or any product if cigarettes not available)
                                                      Least-adjusted
                               Study type
                               CC        Pr        CS     Total


                       N        2        11        10        23
                      NS        1         9         8        18


                      Wt    42.79    284.75    373.90    701.45
                 Het Chi    17.37     38.75     86.16    285.55
                 Het  df        1        10         9        22
                 Het  P       ***       ***       ***       ***
               Fixed  RR     1.13      7.95      5.63      5.87
                     RRl     0.84      7.08      5.09      5.45
                     RRu     1.52      8.93      6.23      6.32
                      P      N.S.       +++       +++       +++
              Random  RR     1.00      6.96      7.07      5.63
                     RRl     0.28      5.07      4.20      4.02
                     RRu     3.57      9.55     11.87      7.90
                      P      N.S.       +++       +++       +++
             Between Chi                                 143.27
             Between  df                                      2
             Between  P                                     ***
             Btwn(F)  P                                     ***

                          COPD subtype
                             mort        LF     other     Total


                       N       13         4         6        23
                      NS       10         4         4        18


                      Wt   327.54    287.78     86.12    701.45
                 Het Chi   197.96      0.81     40.10    285.55
                 Het  df       12         3         5        22
                 Het  P       ***      N.S.       ***       ***
               Fixed  RR     6.16      4.65     10.63      5.87
                     RRl     5.53      4.15      8.61      5.45
                     RRu     6.87      5.22     13.13      6.32
                      P       +++       +++       +++       +++
              Random  RR     4.79      4.65      8.34      5.63
                     RRl     2.79      4.15      4.25      4.02
                     RRu     8.22      5.22     16.36      7.90
                      P       +++       +++       +++       +++
             Between Chi                                  46.68
             Between  df                                      2
             Between  P                                     ***
             Btwn(F)  P                                    N.S.

                             Smoking product
                              any      cigs  cigsonly     Total


                       N        1        11        11        23
                      NS        1         9         8        18


                      Wt     2.41    429.96    269.08    701.45
                 Het Chi     0.00    102.89    178.80    285.55
                 Het  df        0        10        10        22
                 Het  P      N.S.       ***       ***       ***
               Fixed  RR     5.89      5.54      6.45      5.87
                     RRl     1.67      5.04      5.72      5.45
                     RRu    20.80      6.09      7.27      6.32
                      P        ++       +++       +++       +++
              Random  RR     5.89      6.48      4.78      5.63
                     RRl     1.67      4.25      2.43      4.02
                     RRu    20.80      9.87      9.41      7.90
                      P        ++       +++       +++       +++
             Between Chi                                   3.86
             Between  df                                      2
             Between  P                                    N.S.
             Btwn(F)  P                                    N.S.
  ________________________________________________________________________________________________________________________
                                            International Evidence on Smoking and COPD, Phase 3, Analysis run on 28-SEP-10

                                                   Table 1 - E - 2 - 6

                               IESCOPD - Meta-analysis of amount smoked : key value (1) 20
                            Any COPD, cigarettes (or any product if cigarettes not available)
                                                      Least-adjusted
                                     Unexposed group
                          nev any   nev cig  nev+ any  nev+ cig     Total


                       N       20         3                            23
                      NS       15         3                            18


                      Wt   385.22    316.23                        701.45
                 Het Chi   236.12      5.63                        285.55
                 Het  df       19         2                            22
                 Het  P       ***       (*)                           ***
               Fixed  RR     7.36      4.46                          5.87
                     RRl     6.66      3.99                          5.45
                     RRu     8.14      4.98                          6.32
                      P       +++       +++                           +++
              Random  RR     6.15      3.85                          5.63
                     RRl     4.01      2.74                          4.02
                     RRu     9.44      5.41                          7.90
                      P       +++       +++                           +++
             Between Chi                                            43.80
             Between  df                                                1
             Between  P                                               ***
             Btwn(F)  P                                               (*)

                        Unexposed group (combining nev+ with main levels)
                          nev any   nev cig     Total


                       N       20         3        23
                      NS       15         3        18


                      Wt   385.22    316.23    701.45
                 Het Chi   236.12      5.63    285.55
                 Het  df       19         2        22
                 Het  P       ***       (*)       ***
               Fixed  RR     7.36      4.46      5.87
                     RRl     6.66      3.99      5.45
                     RRu     8.14      4.98      6.32
                      P       +++       +++       +++
              Random  RR     6.15      3.85      5.63
                     RRl     4.01      2.74      4.02
                     RRu     9.44      5.41      7.90
                      P       +++       +++       +++
             Between Chi                        43.80
             Between  df                            1
             Between  P                           ***
             Btwn(F)  P                           (*)


  ________________________________________________________________________________________________________________________
                                            International Evidence on Smoking and COPD, Phase 3, Analysis run on 28-SEP-10

                                                   Table 1 - E - 2 - 7

                               IESCOPD - Meta-analysis of amount smoked : key value (1) 20
                            Any COPD, cigarettes (or any product if cigarettes not available)
                                 Excluded studies (and stage at which they were excluded)


1       CLARK COTTON  MEYER REMYJA RUTGER SNYDER SOBRAX     SU TAKEMU  WANG4   WEIR WHICKE ZALACA
2      ALDERS ANDER2 AUERBA   BANG  BECK1  BECK2 BJORNS  BROWN CERVER CHAPMA COATES COLLEG  DEAN2  DEANE DONTA2 DOPICO
       EHRLIC ENRIGH FINKLE FLETCH FOXMAN GOLDBE HAENSZ HARRIS  HAYES HIGGI2 HIGGI3 HIGGI6 HIRAYA HOLLA2 HOLLNA  HOUSE
       HRUBEC HUCHON JENSEN JINDA2  JOSHI JOUSI1   KATO KOTAN1  KUBIK LAMBER LANGE2 LANGHA LAVECC LUNDB2 MAGNUS MANFRE
       MELLST MENEZ1  MEREN MILLER  MILNE MOLLER   NAWA NEJJAR OGILVI  OMORI OSWAL1 OSWAL2 PANDEY  PRATT   REID RIMING
        RYDER SCHWAR  SHARP SHIMUR SOBRAD STJERN SUADIC SUTINE TAGER2 TROISI URRUTI VIEGI1 VIKGRE WAGEN2  WANG2    WIG
       WILHEL WILSO2  WOODS  WOOLF   ZOIA
3      VINEIS
4      ALESSA  AMIGO ANDER3 BEDNAR BROGGE  CHEN3  CHENG  COCCI DEJONG DEMARC DETORR DICKIN EKBERG  FIDAN FORAST FUKUCH
       GEIJER GULSVI HAMMO2 HARDIE HARIKK HEDMAN HIGGI4     HO HUHTI2 ITABAS JAENDI JOHANN KACHEL KARAKA KATANC KHOURY
          KIM  KIRAZ KLAYTO KOJIMA KOTAN2    LAI   LAM1   LAM2   LAM3  LANGE LINDBE   LIU2  MADOR MANNI1 MANNI2 MANNI3
       MARAN1 MARAN2 MARCUS MATHES MENEZ2 MENEZ3 MENEZ4 MENEZ5 MENEZ6 MONTNE NIEPSU NIHLEN   PEAT PELKON PEREZP  PRICE
       RENWIC RICCIO SARGEA SAWICK SHAHAB   SHIN SICHLE  SILVA STERLI  STROM  TAGER   TANG   THUN TRUPIN TSUSHI VESTBO
       VIEGI2 VOLLM1 VOLLM2   WALD WATSON WILSO1 WOJTYN   XIAO     XU ZIELI1 ZIELI2 ZIETKO
5       CHEN1 HAWTHO KULLER   LIU1
6       CHEN2 CLEMEN FERRI3 GODTFR KRZYZA LEBOWI LINDST LUNDB1 NILSSO   PETO SPEIZE TVERDA YAMAGU   YUAN
7        KAHN
11     DONTA1 FERRI1   LIAW


  ________________________________________________________________________________________________________________________
                                            International Evidence on Smoking and COPD, Phase 3, Analysis run on 28-SEP-10

                                                   Table 1 - E - 2 - 8

                               IESCOPD - Meta-analysis of amount smoked : key value (1) 20
                            Any COPD, cigarettes (or any product if cigarettes not available)
                                             Potentially overlapping studies


     REF| REFGP|PRINC|                     OVERLAP|

  HOZAWA HOZAWA     1         ENRIGH/HOZAWA/HARIKK
  JACOBS JACOBS     1  JACOBS/DONTA1/DONTA2/PELKON
  HUHTI1 HUHTI1     1                HUHTI1/HUHTI2
  ENSTRO HAMMO2     2                HAMMO2/ENSTRO
  FERRI2 FERRIS     1         FERRI1/FERRI2/FERRI3
     WEN    WEN     1                     WEN/LIAW
    TODD   TODD     1                  LAMBER/TODD
   KAHN2   KAHN     2                   KAHN/KAHN2

                                    Most-adjusted - insufficient data for meta-analysis
     REF|NRR|SEX|AGEL|AGEH|     REGION|BEGYR|PUBYR|STTYP|ONSET|      DISEAS|ADJ|SMOKSTA|   PRODUCT|    UNEXP|LOW| HI|

    KAHN   4   m   31   99      Am:USA  1954  1966    Pr   Inc    COPD:mort   2 Current       Cigs   Nev any  10  20
          RR|SIG|

       11.23   ?


  ________________________________________________________________________________________________________________________
                                            International Evidence on Smoking and COPD, Phase 3, Analysis run on 28-SEP-10

                                                    Table 1 - E - 3 -

                               IESCOPD - Meta-analysis of amount smoked : key value (1) 45
                            Any COPD, cigarettes (or any product if cigarettes not available)


This analysis is restricted to results for:
1) Eligible study on database
2) Outcome COPD
3) Current or ever smoking
4) Categorical dose-response data for amount smoked
5) vs never smoking base
6) Key value (scheme 1) = 45
7) Results complete enough for use in meta-analysis

Within each study, results are then selected (in the following order of preference, within each sex) for:
8) SMKSTA  : current, ever
9) UNEXP   : never any, never cigarettes
10) PROD    : cigarettes, cigarettes only, any product
11) For overlapping studies: principal rather than subsidiary studies
and then for single sex results (m, f) in preference to results for both sexes combined (b).

Results adjusted for the most potential confounders are then chosen in Sections -1 to -3
and results adjusted for the least confounders in Sections -4 to -6. (Those least-adjusted results which
actually differ from the most-adjusted are marked 'x' in column X in Section -4)

Section -7 shows excluded studies, together with the stage (as above) at which no qualifying
results were found.

Section -8 lists the potentially overlapping studies which have been included (1=principal, 2=subsidiary),
and any results which would have been included in preference except that they had data not complete enough
for use in meta-analysis. It also lists their significance (yes/no), if known.


  ________________________________________________________________________________________________________________________
                                            International Evidence on Smoking and COPD, Phase 3, Analysis run on 28-SEP-10

                                                   Table 1 - E - 3 - 1

                               IESCOPD - Meta-analysis of amount smoked : key value (1) 45
                            Any COPD, cigarettes (or any product if cigarettes not available)
                                                      Most-adjusted


     REF|NRR|SEX|AGEL|AGEH|     REGION|BEGYR|PUBYR|STTYP|ONSET|      DISEAS|ADJ|SMOKSTA|   PRODUCT|    UNEXP|LOW| HI|

  ANDER1  13   m   25   74   Am:Canada  1963  1965    CS  Prev     COPD:oth   1 Current       Cigs   Nev any  25   +
    BEST  40   m   30   97   Am:Canada  1955  1967    Pr   Inc    COPD:mort   1 Current  Cigs only   Nev any  21   +
  CLEMEN   5   m   20   60     Eu:West  1960  1982    Pr  Prev      COPD:LF   0 Current       Cigs   Nev any  21   +
   DEAN1  26   m   35   99       Eu:UK  1969  1977    CC  Prev    COPD:mort   3 Current MCigs only   Nev any  23   +
   DEAN1  66   f   35   99       Eu:UK  1969  1977    CC  Prev    COPD:mort   3 Current MCigs only   Nev any  23   +
   DOLL1   9   m   20   99       Eu:UK  1951  1994    Pr   Inc    COPD:mort   2 Current  Cigs only   Nev any  25   +
   DOLL2   6   f   20   99       Eu:UK  1951  1980    Pr   Inc    COPD:mort   1 Current  Cigs only   Nev any  25   +
  ENSTRO  11   m   30   99      Am:USA  1960  2003    Pr   Inc    COPD:mort   1 Current  Cigs only   Nev any  40  80
  ENSTRO  20   f   30   99      Am:USA  1960  2003    Pr   Inc    COPD:mort   1 Current  Cigs only   Nev any  40  80
  FERRI2  30   m   25   80      Am:USA  1967  1971    CS  Prev     COPD:oth   1 Current       Cigs   Nev any  45   +
  FERRI2  47   f   25   80      Am:USA  1967  1971    CS  Prev     COPD:oth   1 Current       Cigs   Nev any  45   +
  HOZAWA   6   b   45   64      Am:USA  1987  2006    CS  Prev      COPD:LF   0 Current       Cigs  Nev cigs  30   +
  HUHTI1  28   m   40   64    Eu:Scand  1961  1965    CS  Prev    CB/EM/Ast   1 Current       Cigs   Nev any  25   +
  HUHTI3  14   m   25   69    Eu:Scand  1968  1978    CS  Prev      COPD:LF   1 Current        Any   Nev any  25   +
  JACOBS  21   m   40   84       Multi  1957  1999    Pr   Inc    COPD:mort   2 Current       Cigs  Nev cigs  30   +
   KAHN2 102   m   31   84      Am:USA  1954  1966    Pr   Inc    COPD:mort   1 Current       Cigs   Nev any  40   +
  LEBOWI  11   m   15   96      Am:USA  1972  1977    CS  Prev      COPD:LF   1 Current       Cigs  Nev cigs  21   +
  LEBOWI  23   f   15   96      Am:USA  1972  1977    CS  Prev      COPD:LF   1 Current       Cigs  Nev cigs  21   +
     LEE  25   m   35   82       Eu:UK  1964  1979    Pr   Inc    COPD:mort   1 Current  Cigs only   Nev any  21   +
     LEE  46   f   35   82       Eu:UK  1964  1979    Pr   Inc    COPD:mort   0 Current  Cigs only   Nev any  21   +
  MUELLE  27   m   20   69      Am:USA  1967  1971    CS  Prev      COPD:LF   1 Current       Cigs   Nev any  25   +
    TODD  25   m   35   81       Eu:UK  1965  1978    Pr   Inc    COPD:mort   1 Current  Cigs only   Nev any  21   +
  VONHER  12   m   30   99    Eu:Scand  1978  2000    CS  Prev        CB/EM   0 Current       Cigs   Nev any  30   +
  VONHER  19   f   30   99    Eu:Scand  1978  2000    CS  Prev        CB/EM   0 Current       Cigs   Nev any  30   +
   WEISS   8   m   50   69      Am:USA  1961  1963    CS  Prev      COPD:LF   0 Current  Cigs only   Nev any  21   +
     WEN   4   m   35   99   Asia:FarE  1982  2004    Pr   Inc    COPD:mort   1 Current       Cigs  Nev cigs  21   +


  ________________________________________________________________________________________________________________________
                                            International Evidence on Smoking and COPD, Phase 3, Analysis run on 28-SEP-10

                                                   Table 1 - E - 3 - 2

                               IESCOPD - Meta-analysis of amount smoked : key value (1) 45
                            Any COPD, cigarettes (or any product if cigarettes not available)
                                                      Most-adjusted


                        Number Exposed  Non-exposed
 REF    NRR SEX ADJ     Case    Cont    Case    Cont      RR        95.00%CI
 ANDER1 13  m   1         22       -       2       -     10.15 (  2.00-  51.42)
*BEST   40  m   1         19       -       6       -     10.38 (  4.15-  25.98)
 CLEMEN 5   m   0         37     645      11     709      3.70 (  1.87-   7.31)
 DEAN1  26  m   3         66       -      47       -      7.87 (  4.70-  13.17)
 DEAN1  66  f   3         24       -     120       -     18.53 (  9.39-  36.56)
 Subtotal DEAN1                                          10.76 (  7.13-  16.22)
*DOLL1  9   m   2         76       -      12       -     22.50 ( 12.30-  41.15)
*DOLL2  6   f   1          2       -       1       -     32.00 (  2.90- 352.92)
*ENSTRO 11  m   1        148       -     103       -     13.54 ( 10.33-  17.75)
*ENSTRO 20  f   1         46       -     296       -     15.33 ( 11.06-  21.23)
 Subtotal ENSTRO                                         14.24 ( 11.57-  17.54)
 FERRI2 30  m   1         19       -      11       -     17.09 (  5.90-  49.47)
 FERRI2 47  f   1          5       -      54       -     65.73 (  4.45- 971.21)
 Subtotal FERRI2                                         20.49 (  7.62-  55.10)
 HOZAWA 6   b   0        382     284     872    5019      7.74 (  6.53-   9.17)
 HUHTI1 28  m   1         39       -       7       -     11.35 (  4.57-  28.15)
 HUHTI3 14  m   1         12       -       3       -     15.33 (  4.45-  52.84)
*JACOBS 21  m   2         12       -      28       -      5.29 (  2.73-  10.25)
*KAHN2  102 m   1         26       -      31       -     15.02 (  8.92-  25.29)
 LEBOWI 11  m   1          6       -       4       -      4.37 (  1.14-  16.80)
 LEBOWI 23  f   1         14       -      36       -      3.19 (  1.65-   6.17)
 Subtotal LEBOWI                                          3.39 (  1.88-   6.13)
*LEE    25  m   1          5       -       1       -      7.88 (  0.93-  67.01)
*LEE    46  f   0          1      90       5    1694      3.76 (  0.44-  31.89)
 Subtotal LEE                                             5.44 (  1.20-  24.68)
 MUELLE 27  m   1         10       -       2       -      5.54 (  1.12-  27.29)
*TODD   25  m   1          6       -       1       -     10.25 (  1.24-  84.76)
 VONHER 12  m   0         73      65      63     911     16.24 ( 10.66-  24.73)
 VONHER 19  f   0          2       5     137    2888      8.43 (  1.62-  43.85)
 Subtotal VONHER                                         15.60 ( 10.38-  23.45)
 WEISS  8   m   0         12      32       2      34      6.38 (  1.32-  30.73)
*WEN    4   m   1          6       -      52       -      1.46 (  0.51-   4.19)
Partial Totals          1070    1121    1907   11255
*prospective study


 REF    NRR SEX ADJ             Ys       Ws       Qs       Ps
 ANDER1 13  m   1              2.32     1.46     0.00       0.01
*BEST   40  m   1              2.34     4.57     0.01       0.00
 CLEMEN 5   m   0              1.31     8.27     7.90       0.00
 DEAN1  26  m   3              2.06    14.47     0.71       0.00
 DEAN1  66  f   3              2.92     8.32     3.35       0.00
 Subtotal DEAN1                2.38    22.79     4.06
*DOLL1  9   m   2              3.11    10.54     7.23       0.00
*DOLL2  6   f   1              3.47     0.67     0.93       0.00
*ENSTRO 11  m   1              2.61    52.44     5.39       0.00
*ENSTRO 20  f   1              2.73    36.14     7.15       0.00
 Subtotal ENSTRO               2.66    88.57    12.54
 FERRI2 30  m   1              2.84     3.40     1.04       0.00
 FERRI2 47  f   1              4.19     0.53     1.91       0.00
 Subtotal FERRI2               3.02     3.93     2.95
 HOZAWA 6   b   0              2.05   133.60     7.59       0.00
 HUHTI1 28  m   1              2.43     4.65     0.10       0.00
 HUHTI3 14  m   1              2.73     2.51     0.50       0.00
*JACOBS 21  m   2              1.67     8.78     3.37       0.00
*KAHN2  102 m   1              2.71    14.15     2.55       0.00
 LEBOWI 11  m   1              1.47     2.12     1.39       0.03
 LEBOWI 23  f   1              1.16     8.83    11.18       0.00
 Subtotal LEBOWI               1.22    10.96    12.57
*LEE    25  m   1              2.06     0.84     0.04       0.06
*LEE    46  f   0              1.33     0.84     0.77       0.22
 Subtotal LEE                  1.69     1.68     0.82
 MUELLE 27  m   1              1.71     1.51     0.49       0.04
*TODD   25  m   1              2.33     0.86     0.00       0.03
 VONHER 12  m   0              2.79    21.71     5.48       0.00
 VONHER 19  f   0              2.13     1.41     0.03       0.01
 Subtotal VONHER               2.75    23.13     5.51
 WEISS  8   m   0              1.85     1.55     0.29       0.02
*WEN    4   m   1              0.38     3.46    12.59       0.48


  ________________________________________________________________________________________________________________________
                                            International Evidence on Smoking and COPD, Phase 3, Analysis run on 28-SEP-10

                                                   Table 1 - E - 3 - 2

                               IESCOPD - Meta-analysis of amount smoked : key value (1) 45
                            Any COPD, cigarettes (or any product if cigarettes not available)
                                                      Most-adjusted


                       N       26
                      NS       20


                      Wt   347.63
                 Het Chi    82.02
                 Het  df       25
                 Het  P       ***
               Fixed  RR     9.83
                     RRl     8.85
                     RRu    10.92
                      P       +++
              Random  RR     9.50
                     RRl     7.38
                     RRu    12.22
                      P       +++
               Asymm  P      N.S.


  ________________________________________________________________________________________________________________________
                                            International Evidence on Smoking and COPD, Phase 3, Analysis run on 28-SEP-10

                                                   Table 1 - E - 3 - 3

                               IESCOPD - Meta-analysis of amount smoked : key value (1) 45
                            Any COPD, cigarettes (or any product if cigarettes not available)
                                                      Most-adjusted


                       N       26
                      NS       20


                      Wt   347.63
                 Het Chi    82.02
                 Het  df       25
                 Het  P       ***
               Fixed  RR     9.83
                     RRl     8.85
                     RRu    10.92
                      P       +++
              Random  RR     9.50
                     RRl     7.38
                     RRu    12.22
                      P       +++
               Asymm  P      N.S.

                                   Sex
                             both      male    female     Total


                       N        1        18         7        26
                      NS        1        18         7        26


                      Wt   133.60    157.29     56.74    347.63
                 Het Chi     0.00     46.64     22.71     82.02
                 Het  df        0        17         6        25
                 Het  P      N.S.       ***       ***       ***
               Fixed  RR     7.74     11.14     12.18      9.83
                     RRl     6.53      9.52      9.39      8.85
                     RRu     9.17     13.02     15.80     10.92
                      P       +++       +++       +++       +++
              Random  RR     7.74      9.32     10.85      9.50
                     RRl     6.53      6.83      5.20      7.38
                     RRu     9.17     12.72     22.64     12.22
                      P       +++       +++       +++       +++
             Between Chi                                  12.67
             Between  df                                      2
             Between  P                                      **
             Btwn(F)  P                                    N.S.

                                        Continent
                            NAmer    Europe      Asia  oth/mult     Total


                       N       12        12         1         1        26
                      NS        9         9         1         1        20


                      Wt   260.29     75.09      3.46      8.78    347.63
                 Het Chi    38.97     24.04      0.00      0.00     82.02
                 Het  df       11        11         0         0        25
                 Het  P       ***         *      N.S.      N.S.       ***
               Fixed  RR     9.71     12.00      1.46      5.29      9.83
                     RRl     8.60      9.57      0.51      2.73      8.85
                     RRu    10.97     15.05      4.18     10.25     10.92
                      P       +++       +++      N.S.       +++       +++
              Random  RR     9.90     11.39      1.46      5.29      9.50
                     RRl     7.12      7.67      0.51      2.73      7.38
                     RRu    13.76     16.89      4.18     10.25     12.22
                      P       +++       +++      N.S.       +++       +++
             Between Chi                                            19.00
             Between  df                                                3
             Between  P                                               ***
             Btwn(F)  P                                              N.S.


  ________________________________________________________________________________________________________________________
                                            International Evidence on Smoking and COPD, Phase 3, Analysis run on 28-SEP-10

                                                   Table 1 - E - 3 - 3

                               IESCOPD - Meta-analysis of amount smoked : key value (1) 45
                            Any COPD, cigarettes (or any product if cigarettes not available)
                                                      Most-adjusted
                        National cigarette tobacco type (excluding mixed/unkown)
                          blended  virginia     Total


                       N       16         9        25
                      NS       12         7        19


                      Wt   301.60     42.56    344.16
                 Het Chi    56.31     10.25     69.29
                 Het  df       15         8        24
                 Het  P       ***      N.S.       ***
               Fixed  RR     9.69     12.70     10.02
                     RRl     8.65      9.40      9.01
                     RRu    10.84     17.15     11.13
                      P       +++       +++       +++
              Random  RR     9.36     12.74     10.17
                     RRl     6.99      8.71      8.01
                     RRu    12.53     18.65     12.93
                      P       +++       +++       +++
             Between Chi                         2.74
             Between  df                            1
             Between  P                           (*)
             Btwn(F)  P                          N.S.

                                        Start year of study
                            <1970   1970-79   1980-89   1990-99     2000+   unknown     Total


                       N       20         4         2                                      26
                      NS       16         2         2                                      20


                      Wt   176.48     34.08    137.07                                  347.63
                 Het Chi    34.93     18.06      9.40                                   82.02
                 Het  df       19         3         1                                      25
                 Het  P         *       ***        **                                     ***
               Fixed  RR    12.29      9.55      7.42                                    9.83
                     RRl    10.60      6.83      6.28                                    8.85
                     RRu    14.24     13.36      8.77                                   10.92
                      P       +++       +++       +++                                     +++
              Random  RR    11.37      6.78      3.66                                    9.50
                     RRl     8.88      2.40      0.72                                    7.38
                     RRu    14.56     19.15     18.60                                   12.22
                      P       +++       +++      N.S.                                     +++
             Between Chi                                                                19.62
             Between  df                                                                    2
             Between  P                                                                   ***
             Btwn(F)  P                                                                     *

                                Publication year
                            <1980   1980-89   1990-99     2000+     Total


                       N       16         2         2         6        26
                      NS       12         2         2         4        20


                      Wt    70.61      8.94     19.32    248.77    347.63
                 Het Chi    24.33      2.87     10.04     38.13     82.02
                 Het  df       15         1         1         5        25
                 Het  P       (*)       (*)        **       ***       ***
               Fixed  RR     9.67      4.34     11.65     10.03      9.83
                     RRl     7.66      2.25      7.46      8.86      8.85
                     RRu    12.21      8.37     18.20     11.36     10.92
                      P       +++       +++       +++       +++       +++
              Random  RR     9.58      7.90     10.98      9.90      9.50
                     RRl     6.85      1.05      2.66      6.41      7.38
                     RRu    13.41     59.57     45.37     15.30     12.22
                      P       +++         +       +++       +++       +++
             Between Chi                                             6.64
             Between  df                                                3
             Between  P                                               (*)
             Btwn(F)  P                                              N.S.
  ________________________________________________________________________________________________________________________
                                            International Evidence on Smoking and COPD, Phase 3, Analysis run on 28-SEP-10

                                                   Table 1 - E - 3 - 3

                               IESCOPD - Meta-analysis of amount smoked : key value (1) 45
                            Any COPD, cigarettes (or any product if cigarettes not available)
                                                      Most-adjusted
                               Study type
                               CC        Pr        CS     Total


                       N        2        12        12        26
                      NS        1        10         9        20


                      Wt    22.79    141.55    183.29    347.63
                 Het Chi     3.87     42.57     25.23     82.02
                 Het  df        1        11        11        25
                 Het  P         *       ***        **       ***
               Fixed  RR    10.76     11.94      8.36      9.83
                     RRl     7.13     10.13      7.23      8.85
                     RRu    16.22     14.08      9.66     10.92
                      P       +++       +++       +++       +++
              Random  RR    11.72      9.23      9.01      9.50
                     RRl     5.07      6.14      6.18      7.38
                     RRu    27.07     13.89     13.16     12.22
                      P       +++       +++       +++       +++
             Between Chi                                  10.35
             Between  df                                      2
             Between  P                                      **
             Btwn(F)  P                                    N.S.

                                    Lowest age in RR
                        <25/unlim     25-39       40+   unknown     Total


                       N        6        16         4                  26
                      NS        5        11         4                  20


                      Wt    31.94    167.11    148.58              347.63
                 Het Chi    25.28     27.53      1.99               82.02
                 Het  df        5        15         3                  25
                 Het  P       ***         *      N.S.                 ***
               Fixed  RR     6.94     13.13      7.65                9.83
                     RRl     4.91     11.28      6.51                8.85
                     RRu     9.82     15.27      8.98               10.92
                      P       +++       +++       +++                 +++
              Random  RR     6.86     12.08      7.65                9.50
                     RRl     2.84      9.34      6.51                7.38
                     RRu    16.57     15.64      8.98               12.22
                      P       +++       +++       +++                 +++
             Between Chi                                            27.22
             Between  df                                                2
             Between  P                                               ***
             Btwn(F)  P                                                **

                                         Highest age in RR
                              <65     65-74     75-84 85+/unlim   unknown     Total


                       N        3         4         7        12                  26
                      NS        3         4         5         8                  20


                      Wt   146.52      7.03     29.40    164.68              347.63
                 Het Chi     5.07      1.26      9.47     47.08               82.02
                 Het  df        2         3         6        11                  25
                 Het  P       (*)      N.S.      N.S.       ***                 ***
               Fixed  RR     7.52      9.32     10.70     12.31                9.83
                     RRl     6.39      4.45      7.45     10.57                8.85
                     RRu     8.84     19.52     15.36     14.34               10.92
                      P       +++       +++       +++       +++                 +++
              Random  RR     6.80      9.32     10.53     10.05                9.50
                     RRl     4.05      4.45      6.01      6.92                7.38
                     RRu    11.41     19.52     18.43     14.58               12.22
                      P       +++       +++       +++       +++                 +++
             Between Chi                                                      19.13
             Between  df                                                          3
             Between  P                                                         ***
             Btwn(F)  P                                                        N.S.
  ________________________________________________________________________________________________________________________
                                            International Evidence on Smoking and COPD, Phase 3, Analysis run on 28-SEP-10

                                                   Table 1 - E - 3 - 3

                               IESCOPD - Meta-analysis of amount smoked : key value (1) 45
                            Any COPD, cigarettes (or any product if cigarettes not available)
                                                      Most-adjusted
                           Study weakness
                              Yes        No     Total


                       N        2        24        26
                      NS        1        19        20


                      Wt    22.79    324.84    347.63
                 Het Chi     3.87     77.94     82.02
                 Het  df        1        23        25
                 Het  P         *       ***       ***
               Fixed  RR    10.76      9.76      9.83
                     RRl     7.13      8.76      8.85
                     RRu    16.22     10.89     10.92
                      P       +++       +++       +++
              Random  RR    11.72      9.24      9.50
                     RRl     5.07      7.03      7.38
                     RRu    27.07     12.13     12.22
                      P       +++       +++       +++
             Between Chi                         0.20
             Between  df                            1
             Between  P                          N.S.
             Btwn(F)  P                          N.S.

                          COPD subtype
                             mort        LF     other     Total


                       N       13         7         6        26
                      NS       10         6         4        20


                      Wt   156.07    158.40     33.16    347.63
                 Het Chi    34.98     12.27      2.40     82.02
                 Het  df       12         6         5        25
                 Het  P       ***       (*)      N.S.       ***
               Fixed  RR    12.51      7.08     15.12      9.83
                     RRl    10.70      6.06     10.76      8.85
                     RRu    14.64      8.27     21.25     10.92
                      P       +++       +++       +++       +++
              Random  RR    10.83      5.60     15.12      9.50
                     RRl     7.79      3.63     10.76      7.38
                     RRu    15.07      8.63     21.25     12.22
                      P       +++       +++       +++       +++
             Between Chi                                  32.36
             Between  df                                      2
             Between  P                                     ***
             Btwn(F)  P                                      **

                               Asthma analysis type (COPD)
                        inc-irres  excl-all defn-incl     other     Total


                       N       15                   8         3        26
                      NS       12                   5         3        20


                      Wt   206.25              121.40     19.98    347.63
                 Het Chi    42.03                7.41     10.69     82.02
                 Het  df       14                   7         2        25
                 Het  P       ***                N.S.        **       ***
               Fixed  RR     7.99               13.50     12.05      9.83
                     RRl     6.97               11.30      7.77      8.85
                     RRu     9.16               16.13     18.68     10.92
                      P       +++                 +++       +++       +++
              Random  RR     7.00               13.46     13.13      9.50
                     RRl     4.85               11.11      3.88      7.38
                     RRu    10.10               16.31     44.45     12.22
                      P       +++                 +++       +++       +++
             Between Chi                                            21.89
             Between  df                                                2
             Between  P                                               ***
             Btwn(F)  P                                                 *
  ________________________________________________________________________________________________________________________
                                            International Evidence on Smoking and COPD, Phase 3, Analysis run on 28-SEP-10

                                                   Table 1 - E - 3 - 3

                               IESCOPD - Meta-analysis of amount smoked : key value (1) 45
                            Any COPD, cigarettes (or any product if cigarettes not available)
                                                      Most-adjusted
                        Bronchodilator/reversibility (LF only)
                          no/unkn  yes/revs     Total


                       N        7                   7
                      NS        6                   6


                      Wt   158.40              158.40
                 Het Chi    12.27               12.27
                 Het  df        6                   6
                 Het  P       (*)                 (*)
               Fixed  RR     7.08                7.08
                     RRl     6.06                6.06
                     RRu     8.27                8.27
                      P       +++                 +++
              Random  RR     5.60                5.60
                     RRl     3.63                3.63
                     RRu     8.63                8.63
                      P       +++                 +++
             Between Chi
             Between  df
             Between  P                          N.S.
             Btwn(F)  P                          N.S.

                                  Number of COPD cases
                             1-50    51-100   101-200      201+     Total


                       N        3         6         4        13        26
                      NS        3         5         3         9        20


                      Wt     3.73     14.78     18.99    310.13    347.63
                 Het Chi     1.56      5.02      8.09     43.61     82.02
                 Het  df        2         5         3        12        25
                 Het  P      N.S.      N.S.         *       ***       ***
               Fixed  RR     8.04      5.77      3.81     10.71      9.83
                     RRl     2.91      3.46      2.43      9.58      8.85
                     RRu    22.19      9.60      5.97     11.97     10.92
                      P       +++       +++       +++       +++       +++
              Random  RR     8.04      5.78      3.83     12.48      9.50
                     RRl     2.91      3.46      1.75      9.57      7.38
                     RRu    22.19      9.66      8.41     16.29     12.22
                      P       +++       +++       +++       +++       +++
             Between Chi                                            23.72
             Between  df                                                3
             Between  P                                               ***
             Btwn(F)  P                                               (*)

                        Number of COPD cases (excluding unknown)


                       N        3         6         4        13        26
                      NS        3         5         3         9        20


                      Wt     3.73     14.78     18.99    310.13    347.63
                 Het Chi     1.56      5.02      8.09     43.61     82.02
                 Het  df        2         5         3        12        25
                 Het  P      N.S.      N.S.         *       ***       ***
               Fixed  RR     8.04      5.77      3.81     10.71      9.83
                     RRl     2.91      3.46      2.43      9.58      8.85
                     RRu    22.19      9.60      5.97     11.97     10.92
                      P       +++       +++       +++       +++       +++
              Random  RR     8.04      5.78      3.83     12.48      9.50
                     RRl     2.91      3.46      1.75      9.57      7.38
                     RRu    22.19      9.66      8.41     16.29     12.22
                      P       +++       +++       +++       +++       +++
             Between Chi                                            23.72
             Between  df                                                3
             Between  P                                               ***
             Btwn(F)  P                                               (*)

  ________________________________________________________________________________________________________________________
                                            International Evidence on Smoking and COPD, Phase 3, Analysis run on 28-SEP-10

                                                   Table 1 - E - 3 - 3

                               IESCOPD - Meta-analysis of amount smoked : key value (1) 45
                            Any COPD, cigarettes (or any product if cigarettes not available)
                                                      Most-adjusted
                            Analysis type
                         prevlnce     onset     Total


                       N       15        11        26
                      NS       11         9        20


                      Wt   214.35    133.28    347.63
                 Het Chi    36.05     30.50     82.02
                 Het  df       14        10        25
                 Het  P        **       ***       ***
               Fixed  RR     8.32     12.84      9.83
                     RRl     7.28     10.84      8.85
                     RRu     9.51     15.22     10.92
                      P       +++       +++       +++
              Random  RR     8.75     10.55      9.50
                     RRl     6.36      7.18      7.38
                     RRu    12.04     15.50     12.22
                      P       +++       +++       +++
             Between Chi                        15.47
             Between  df                            1
             Between  P                           ***
             Btwn(F)  P                             *

                             Smoking product
                              any      cigs  cigsonly     Total


                       N        1        14        11        26
                      NS        1        11         8        20


                      Wt     2.51    213.89    131.23    347.63
                 Het Chi     0.00     46.51     11.73     82.02
                 Het  df        0        13        10        25
                 Het  P      N.S.       ***      N.S.       ***
               Fixed  RR    15.33      7.99     13.64      9.83
                     RRl     4.45      6.99     11.50      8.85
                     RRu    52.83      9.14     16.19     10.92
                      P       +++       +++       +++       +++
              Random  RR    15.33      7.35     13.51      9.50
                     RRl     4.45      5.08     10.93      7.38
                     RRu    52.83     10.62     16.70     12.22
                      P       +++       +++       +++       +++
             Between Chi                                  23.77
             Between  df                                      2
             Between  P                                     ***
             Btwn(F)  P                                       *

                                     Unexposed group
                          nev any   nev cig  nev+ any  nev+ cig     Total


                       N       21         5                            26
                      NS       16         4                            20


                      Wt   190.83    156.80                        347.63
                 Het Chi    29.71     16.45                         82.02
                 Het  df       20         4                            25
                 Het  P       (*)        **                           ***
               Fixed  RR    13.15      6.89                          9.83
                     RRl    11.41      5.90                          8.85
                     RRu    15.15      8.06                         10.92
                      P       +++       +++                           +++
              Random  RR    12.54      4.26                          9.50
                     RRl    10.18      2.37                          7.38
                     RRu    15.46      7.65                         12.22
                      P       +++       +++                           +++
             Between Chi                                            35.85
             Between  df                                                1
             Between  P                                               ***
             Btwn(F)  P                                               ***
  ________________________________________________________________________________________________________________________
                                            International Evidence on Smoking and COPD, Phase 3, Analysis run on 28-SEP-10

                                                   Table 1 - E - 3 - 3

                               IESCOPD - Meta-analysis of amount smoked : key value (1) 45
                            Any COPD, cigarettes (or any product if cigarettes not available)
                                                      Most-adjusted
                        Unexposed group (combining nev+ with main levels)
                          nev any   nev cig     Total


                       N       21         5        26
                      NS       16         4        20


                      Wt   190.83    156.80    347.63
                 Het Chi    29.71     16.45     82.02
                 Het  df       20         4        25
                 Het  P       (*)        **       ***
               Fixed  RR    13.15      6.89      9.83
                     RRl    11.41      5.90      8.85
                     RRu    15.15      8.06     10.92
                      P       +++       +++       +++
              Random  RR    12.54      4.26      9.50
                     RRl    10.18      2.37      7.38
                     RRu    15.46      7.65     12.22
                      P       +++       +++       +++
             Between Chi                        35.85
             Between  df                            1
             Between  P                           ***
             Btwn(F)  P                           ***

                        Number of adjustment variables
                                0         1        2+     Total


                       N        6        16         4        26
                      NS        5        13         3        21


                      Wt   167.39    138.13     42.10    347.63
                 Het Chi    16.44     40.62     13.98     82.02
                 Het  df        5        15         3        25
                 Het  P        **       ***        **       ***
               Fixed  RR     8.18     11.81     11.16      9.83
                     RRl     7.03      9.99      8.25      8.85
                     RRu     9.52     13.95     15.09     10.92
                      P       +++       +++       +++       +++
              Random  RR     7.77      9.61     11.43      9.50
                     RRl     4.69      6.69      5.91      7.38
                     RRu    12.88     13.80     22.09     12.22
                      P       +++       +++       +++       +++
             Between Chi                                  10.98
             Between  df                                      2
             Between  P                                      **
             Btwn(F)  P                                    N.S.


  ________________________________________________________________________________________________________________________
                                            International Evidence on Smoking and COPD, Phase 3, Analysis run on 28-SEP-10

                                                   Table 1 - E - 3 - 4

                               IESCOPD - Meta-analysis of amount smoked : key value (1) 45
                            Any COPD, cigarettes (or any product if cigarettes not available)
                                                      Least-adjusted


     REF|NRR|X|SEX|AGEL|AGEH|     REGION|BEGYR|PUBYR|STTYP|ONSET|      DISEAS|ADJ|SMOKSTA|   PRODUCT|    UNEXP|LOW| HI|

  ANDER1  13     m   25   74   Am:Canada  1963  1965    CS  Prev     COPD:oth   1 Current       Cigs   Nev any  25   +
    BEST  40     m   30   97   Am:Canada  1955  1967    Pr   Inc    COPD:mort   1 Current  Cigs only   Nev any  21   +
  CLEMEN   5     m   20   60     Eu:West  1960  1982    Pr  Prev      COPD:LF   0 Current       Cigs   Nev any  21   +
   DEAN1  21 x   m   35   99       Eu:UK  1969  1977    CC  Prev    COPD:mort   0 Current MCigs only   Nev any  23   +
   DEAN1  61 x   f   35   99       Eu:UK  1969  1977    CC  Prev    COPD:mort   0 Current MCigs only   Nev any  23   +
   DOLL1   9     m   20   99       Eu:UK  1951  1994    Pr   Inc    COPD:mort   2 Current  Cigs only   Nev any  25   +
   DOLL2   6     f   20   99       Eu:UK  1951  1980    Pr   Inc    COPD:mort   1 Current  Cigs only   Nev any  25   +
  ENSTRO  11     m   30   99      Am:USA  1960  2003    Pr   Inc    COPD:mort   1 Current  Cigs only   Nev any  40  80
  ENSTRO  20     f   30   99      Am:USA  1960  2003    Pr   Inc    COPD:mort   1 Current  Cigs only   Nev any  40  80
  FERRI2  30     m   25   80      Am:USA  1967  1971    CS  Prev     COPD:oth   1 Current       Cigs   Nev any  45   +
  FERRI2  47     f   25   80      Am:USA  1967  1971    CS  Prev     COPD:oth   1 Current       Cigs   Nev any  45   +
  HOZAWA   6     b   45   64      Am:USA  1987  2006    CS  Prev      COPD:LF   0 Current       Cigs  Nev cigs  30   +
  HUHTI1  23 x   m   40   64    Eu:Scand  1961  1965    CS  Prev    CB/EM/Ast   0 Current       Cigs   Nev any  25   +
  HUHTI3   9 x   m   25   69    Eu:Scand  1968  1978    CS  Prev      COPD:LF   0 Current        Any   Nev any  25   +
  JACOBS  21     m   40   84       Multi  1957  1999    Pr   Inc    COPD:mort   2 Current       Cigs  Nev cigs  30   +
   KAHN2 102     m   31   84      Am:USA  1954  1966    Pr   Inc    COPD:mort   1 Current       Cigs   Nev any  40   +
  LEBOWI   8 x   m   15   96      Am:USA  1972  1977    CS  Prev      COPD:LF   0 Current       Cigs  Nev cigs  21   +
  LEBOWI  20 x   f   15   96      Am:USA  1972  1977    CS  Prev      COPD:LF   0 Current       Cigs  Nev cigs  21   +
     LEE  11 x   m   35   82       Eu:UK  1964  1979    Pr   Inc    COPD:mort   0 Current  Cigs only   Nev any  21   +
     LEE  46     f   35   82       Eu:UK  1964  1979    Pr   Inc    COPD:mort   0 Current  Cigs only   Nev any  21   +
  MUELLE  22 x   m   20   69      Am:USA  1967  1971    CS  Prev      COPD:LF   0 Current       Cigs   Nev any  25   +
    TODD  11 x   m   35   81       Eu:UK  1965  1978    Pr   Inc    COPD:mort   0 Current  Cigs only   Nev any  21   +
  VONHER  12     m   30   99    Eu:Scand  1978  2000    CS  Prev        CB/EM   0 Current       Cigs   Nev any  30   +
  VONHER  19     f   30   99    Eu:Scand  1978  2000    CS  Prev        CB/EM   0 Current       Cigs   Nev any  30   +
   WEISS   8     m   50   69      Am:USA  1961  1963    CS  Prev      COPD:LF   0 Current  Cigs only   Nev any  21   +
     WEN   4     m   35   99   Asia:FarE  1982  2004    Pr   Inc    COPD:mort   1 Current       Cigs  Nev cigs  21   +


  ________________________________________________________________________________________________________________________
                                            International Evidence on Smoking and COPD, Phase 3, Analysis run on 28-SEP-10

                                                   Table 1 - E - 3 - 5

                               IESCOPD - Meta-analysis of amount smoked : key value (1) 45
                            Any COPD, cigarettes (or any product if cigarettes not available)
                                                      Least-adjusted


                        Number Exposed  Non-exposed
 REF    NRR SEX ADJ     Case    Cont    Case    Cont      RR        95.00%CI
 ANDER1 13  m   1         22       -       2       -     10.15 (  2.00-  51.42)
*BEST   40  m   1         19       -       6       -     10.38 (  4.15-  25.98)
 CLEMEN 5   m   0         37     645      11     709      3.70 (  1.87-   7.31)
 DEAN1  21  m   0         66     237      47     510      3.02 (  2.02-   4.53)
 DEAN1  61  f   0         24     151     120    1538      2.04 (  1.27-   3.26)
 Subtotal DEAN1                                           2.55 (  1.88-   3.47)
*DOLL1  9   m   2         76       -      12       -     22.50 ( 12.30-  41.15)
*DOLL2  6   f   1          2       -       1       -     32.00 (  2.90- 352.92)
*ENSTRO 11  m   1        148       -     103       -     13.54 ( 10.33-  17.75)
*ENSTRO 20  f   1         46       -     296       -     15.33 ( 11.06-  21.23)
 Subtotal ENSTRO                                         14.24 ( 11.57-  17.54)
 FERRI2 30  m   1         19       -      11       -     17.09 (  5.90-  49.47)
 FERRI2 47  f   1          5       -      54       -     65.73 (  4.45- 971.21)
 Subtotal FERRI2                                         20.49 (  7.62-  55.10)
 HOZAWA 6   b   0        382     284     872    5019      7.74 (  6.53-   9.17)
 HUHTI1 23  m   0         39      46       7     115     13.93 (  5.81-  33.39)
 HUHTI3 9   m   0         12      64       3     236     14.75 (  4.04-  53.85)
*JACOBS 21  m   2         12       -      28       -      5.29 (  2.73-  10.25)
*KAHN2  102 m   1         26       -      31       -     15.02 (  8.92-  25.29)
 LEBOWI 8   m   0          6      54       4     172      4.78 (  1.30-  17.56)
 LEBOWI 20  f   0         14     104      36     766      2.86 (  1.49-   5.49)
 Subtotal LEBOWI                                          3.17 (  1.77-   5.68)
*LEE    11  m   0          5     266       1     347      6.52 (  0.77-  55.50)
*LEE    46  f   0          1      90       5    1694      3.76 (  0.44-  31.89)
 Subtotal LEE                                             4.95 (  1.09-  22.47)
 MUELLE 22  m   0         10      46       2      57      6.20 (  1.29-  29.69)
*TODD   11  m   0          6     338       1     520      9.23 (  1.12-  76.33)
 VONHER 12  m   0         73      65      63     911     16.24 ( 10.66-  24.73)
 VONHER 19  f   0          2       5     137    2888      8.43 (  1.62-  43.85)
 Subtotal VONHER                                         15.60 ( 10.38-  23.45)
 WEISS  8   m   0         12      32       2      34      6.38 (  1.32-  30.73)
*WEN    4   m   1          6       -      52       -      1.46 (  0.51-   4.19)
Partial Totals          1070    2427    1907   15516
*prospective study


 REF    NRR SEX ADJ             Ys       Ws       Qs       Ps
 ANDER1 13  m   1              2.32     1.46     0.05       0.01
*BEST   40  m   1              2.34     4.57     0.21       0.00
 CLEMEN 5   m   0              1.31     8.27     5.57       0.00
 DEAN1  21  m   0              1.11    23.47    24.51       0.00
 DEAN1  61  f   0              0.71    17.46    35.03       0.00
 Subtotal DEAN1                0.94    40.93    59.54
*DOLL1  9   m   2              3.11    10.54    10.24       0.00
*DOLL2  6   f   1              3.47     0.67     1.19       0.00
*ENSTRO 11  m   1              2.61    52.44    11.97       0.00
*ENSTRO 20  f   1              2.73    36.14    13.09       0.00
 Subtotal ENSTRO               2.66    88.57    25.06
 FERRI2 30  m   1              2.84     3.40     1.72       0.00
 FERRI2 47  f   1              4.19     0.53     2.24       0.00
 Subtotal FERRI2               3.02     3.93     3.96
 HOZAWA 6   b   0              2.05   133.60     0.88       0.00
 HUHTI1 23  m   0              2.63     5.03     1.29       0.00
 HUHTI3 9   m   0              2.69     2.29     0.73       0.00
*JACOBS 21  m   2              1.67     8.78     1.87       0.00
*KAHN2  102 m   1              2.71    14.15     4.78       0.00
 LEBOWI 8   m   0              1.56     2.27     0.72       0.02
 LEBOWI 20  f   0              1.05     9.08    10.50       0.00
 Subtotal LEBOWI               1.15    11.35    11.23
*LEE    11  m   0              1.88     0.84     0.05       0.09
*LEE    46  f   0              1.33     0.84     0.54       0.22
 Subtotal LEE                  1.60     1.68     0.60
 MUELLE 22  m   0              1.82     1.56     0.14       0.02
*TODD   11  m   0              2.22     0.86     0.01       0.04
 VONHER 12  m   0              2.79    21.71     9.45       0.00
 VONHER 19  f   0              2.13     1.41     0.00       0.01
 Subtotal VONHER               2.75    23.13     9.45
 WEISS  8   m   0              1.85     1.55     0.12       0.02
*WEN    4   m   1              0.38     3.46    10.60       0.48


  ________________________________________________________________________________________________________________________
                                            International Evidence on Smoking and COPD, Phase 3, Analysis run on 28-SEP-10

                                                   Table 1 - E - 3 - 5

                               IESCOPD - Meta-analysis of amount smoked : key value (1) 45
                            Any COPD, cigarettes (or any product if cigarettes not available)
                                                      Least-adjusted


                       N       26
                      NS       20


                      Wt   366.37
                 Het Chi   147.51
                 Het  df       25
                 Het  P       ***
               Fixed  RR     8.40
                     RRl     7.58
                     RRu     9.30
                      P       +++
              Random  RR     7.85
                     RRl     5.72
                     RRu    10.76
                      P       +++
               Asymm  P      N.S.


  ________________________________________________________________________________________________________________________
                                            International Evidence on Smoking and COPD, Phase 3, Analysis run on 28-SEP-10

                                                   Table 1 - E - 3 - 6

                               IESCOPD - Meta-analysis of amount smoked : key value (1) 45
                            Any COPD, cigarettes (or any product if cigarettes not available)
                                                      Least-adjusted


                       N       26
                      NS       20


                      Wt   366.37
                 Het Chi   147.51
                 Het  df       25
                 Het  P       ***
               Fixed  RR     8.40
                     RRl     7.58
                     RRu     9.30
                      P       +++
              Random  RR     7.85
                     RRl     5.72
                     RRu    10.76
                      P       +++
               Asymm  P      N.S.

                                   Sex
                             both      male    female     Total


                       N        1        18         7        26
                      NS        1        18         7        26


                      Wt   133.60    166.64     66.13    366.37
                 Het Chi     0.00     81.05     60.63    147.51
                 Het  df        0        17         6        25
                 Het  P      N.S.       ***       ***       ***
               Fixed  RR     7.74      9.60      7.06      8.40
                     RRl     6.53      8.25      5.55      7.58
                     RRu     9.17     11.17      8.99      9.30
                      P       +++       +++       +++       +++
              Random  RR     7.74      8.45      7.29      7.85
                     RRl     6.53      5.72      2.62      5.72
                     RRu     9.17     12.49     20.32     10.76
                      P       +++       +++       +++       +++
             Between Chi                                   5.84
             Between  df                                      2
             Between  P                                     (*)
             Btwn(F)  P                                    N.S.

                                        Continent
                            NAmer    Europe      Asia  oth/mult     Total


                       N       12        12         1         1        26
                      NS        9         9         1         1        20


                      Wt   260.74     93.39      3.46      8.78    366.37
                 Het Chi    41.19     80.87      0.00      0.00    147.51
                 Het  df       11        11         0         0        25
                 Het  P       ***       ***      N.S.      N.S.       ***
               Fixed  RR     9.68      6.30      1.46      5.29      8.40
                     RRl     8.57      5.14      0.51      2.73      7.58
                     RRu    10.93      7.71      4.18     10.25      9.30
                      P       +++       +++      N.S.       +++       +++
              Random  RR     9.82      7.79      1.46      5.29      7.85
                     RRl     7.01      4.09      0.51      2.73      5.72
                     RRu    13.74     14.86      4.18     10.25     10.76
                      P       +++       +++      N.S.       +++       +++
             Between Chi                                            25.45
             Between  df                                                3
             Between  P                                               ***
             Btwn(F)  P                                              N.S.


  ________________________________________________________________________________________________________________________
                                            International Evidence on Smoking and COPD, Phase 3, Analysis run on 28-SEP-10

                                                   Table 1 - E - 3 - 6

                               IESCOPD - Meta-analysis of amount smoked : key value (1) 45
                            Any COPD, cigarettes (or any product if cigarettes not available)
                                                      Least-adjusted
                               Study type
                               CC        Pr        CS     Total


                       N        2        12        12        26
                      NS        1        10         9        20


                      Wt    40.93    141.55    183.90    366.37
                 Het Chi     1.56     42.77     27.84    147.51
                 Het  df        1        11        11        25
                 Het  P      N.S.       ***        **       ***
               Fixed  RR     2.55     11.92      8.36      8.40
                     RRl     1.88     10.11      7.23      7.58
                     RRu     3.47     14.05      9.66      9.30
                      P       +++       +++       +++       +++
              Random  RR     2.53      9.15      9.12      7.85
                     RRl     1.72      6.08      6.15      5.72
                     RRu     3.71     13.77     13.52     10.76
                      P       +++       +++       +++       +++
             Between Chi                                  75.35
             Between  df                                      2
             Between  P                                     ***
             Btwn(F)  P                                     ***

                          COPD subtype
                             mort        LF     other     Total


                       N       13         7         6        26
                      NS       10         6         4        20


                      Wt   174.21    158.63     33.54    366.37
                 Het Chi   113.77     13.61      2.03    147.51
                 Het  df       12         6         5        25
                 Het  P       ***         *      N.S.       ***
               Fixed  RR     8.77      7.03     15.54      8.40
                     RRl     7.56      6.01     11.08      7.58
                     RRu    10.18      8.21     21.80      9.30
                      P       +++       +++       +++       +++
              Random  RR     7.36      5.49     15.54      7.85
                     RRl     4.29      3.48     11.08      5.72
                     RRu    12.61      8.66     21.80     10.76
                      P       +++       +++       +++       +++
             Between Chi                                  18.10
             Between  df                                      2
             Between  P                                     ***
             Btwn(F)  P                                    N.S.

                             Smoking product
                              any      cigs  cigsonly     Total


                       N        1        14        11        26
                      NS        1        11         8        20


                      Wt     2.29    214.72    149.37    366.37
                 Het Chi     0.00     49.32     96.40    147.51
                 Het  df        0        13        10        25
                 Het  P      N.S.       ***       ***       ***
               Fixed  RR    14.75      8.00      8.92      8.40
                     RRl     4.04      7.00      7.60      7.58
                     RRu    53.85      9.15     10.48      9.30
                      P       +++       +++       +++       +++
              Random  RR    14.75      7.44      8.10      7.85
                     RRl     4.04      5.11      4.36      5.72
                     RRu    53.85     10.84     15.03     10.76
                      P       +++       +++       +++       +++
             Between Chi                                   1.78
             Between  df                                      2
             Between  P                                    N.S.
             Btwn(F)  P                                    N.S.
  ________________________________________________________________________________________________________________________
                                            International Evidence on Smoking and COPD, Phase 3, Analysis run on 28-SEP-10

                                                   Table 1 - E - 3 - 6

                               IESCOPD - Meta-analysis of amount smoked : key value (1) 45
                            Any COPD, cigarettes (or any product if cigarettes not available)
                                                      Least-adjusted
                                     Unexposed group
                          nev any   nev cig  nev+ any  nev+ cig     Total


                       N       21         5                            26
                      NS       16         4                            20


                      Wt   209.18    157.19                        366.37
                 Het Chi   118.03     18.06                        147.51
                 Het  df       20         4                            25
                 Het  P       ***        **                           ***
               Fixed  RR     9.79      6.85                          8.40
                     RRl     8.55      5.86                          7.58
                     RRu    11.21      8.01                          9.30
                      P       +++       +++                           +++
              Random  RR     9.58      4.17                          7.85
                     RRl     6.43      2.27                          5.72
                     RRu    14.27      7.66                         10.76
                      P       +++       +++                           +++
             Between Chi                                            11.42
             Between  df                                                1
             Between  P                                               ***
             Btwn(F)  P                                              N.S.

                        Unexposed group (combining nev+ with main levels)
                          nev any   nev cig     Total


                       N       21         5        26
                      NS       16         4        20


                      Wt   209.18    157.19    366.37
                 Het Chi   118.03     18.06    147.51
                 Het  df       20         4        25
                 Het  P       ***        **       ***
               Fixed  RR     9.79      6.85      8.40
                     RRl     8.55      5.86      7.58
                     RRu    11.21      8.01      9.30
                      P       +++       +++       +++
              Random  RR     9.58      4.17      7.85
                     RRl     6.43      2.27      5.72
                     RRu    14.27      7.66     10.76
                      P       +++       +++       +++
             Between Chi                        11.42
             Between  df                            1
             Between  P                           ***
             Btwn(F)  P                          N.S.


  ________________________________________________________________________________________________________________________
                                            International Evidence on Smoking and COPD, Phase 3, Analysis run on 28-SEP-10

                                                   Table 1 - E - 3 - 7

                               IESCOPD - Meta-analysis of amount smoked : key value (1) 45
                            Any COPD, cigarettes (or any product if cigarettes not available)
                                 Excluded studies (and stage at which they were excluded)


1       CLARK COTTON  MEYER REMYJA RUTGER SNYDER SOBRAX     SU TAKEMU  WANG4   WEIR WHICKE ZALACA
2      ALDERS ANDER2 AUERBA   BANG  BECK1  BECK2 BJORNS  BROWN CERVER CHAPMA COATES COLLEG  DEAN2  DEANE DONTA2 DOPICO
       EHRLIC ENRIGH FINKLE FLETCH FOXMAN GOLDBE HAENSZ HARRIS  HAYES HIGGI2 HIGGI3 HIGGI6 HIRAYA HOLLA2 HOLLNA  HOUSE
       HRUBEC HUCHON JENSEN JINDA2  JOSHI JOUSI1   KATO KOTAN1  KUBIK LAMBER LANGE2 LANGHA LAVECC LUNDB2 MAGNUS MANFRE
       MELLST MENEZ1  MEREN MILLER  MILNE MOLLER   NAWA NEJJAR OGILVI  OMORI OSWAL1 OSWAL2 PANDEY  PRATT   REID RIMING
        RYDER SCHWAR  SHARP SHIMUR SOBRAD STJERN SUADIC SUTINE TAGER2 TROISI URRUTI VIEGI1 VIKGRE WAGEN2  WANG2    WIG
       WILHEL WILSO2  WOODS  WOOLF   ZOIA
3      VINEIS
4      ALESSA  AMIGO ANDER3 BEDNAR BROGGE  CHEN3  CHENG  COCCI DEJONG DEMARC DETORR DICKIN EKBERG  FIDAN FORAST FUKUCH
       GEIJER GULSVI HAMMO2 HARDIE HARIKK HEDMAN HIGGI4     HO HUHTI2 ITABAS JAENDI JOHANN KACHEL KARAKA KATANC KHOURY
          KIM  KIRAZ KLAYTO KOJIMA KOTAN2    LAI   LAM1   LAM2   LAM3  LANGE LINDBE   LIU2  MADOR MANNI1 MANNI2 MANNI3
       MARAN1 MARAN2 MARCUS MATHES MENEZ2 MENEZ3 MENEZ4 MENEZ5 MENEZ6 MONTNE NIEPSU NIHLEN   PEAT PELKON PEREZP  PRICE
       RENWIC RICCIO SARGEA SAWICK SHAHAB   SHIN SICHLE  SILVA STERLI  STROM  TAGER   TANG   THUN TRUPIN TSUSHI VESTBO
       VIEGI2 VOLLM1 VOLLM2   WALD WATSON WILSO1 WOJTYN   XIAO     XU ZIELI1 ZIELI2 ZIETKO
5       CHEN1 HAWTHO KULLER   LIU1
6       CHEN2 GODTFR KRZYZA LINDST LUNDB1 NILSSO   PETO SPEIZE TVERDA YAMAGU   YUAN
7        KAHN
11     DONTA1 FERRI1 FERRI3   LIAW


  ________________________________________________________________________________________________________________________
                                            International Evidence on Smoking and COPD, Phase 3, Analysis run on 28-SEP-10

                                                   Table 1 - E - 3 - 8

                               IESCOPD - Meta-analysis of amount smoked : key value (1) 45
                            Any COPD, cigarettes (or any product if cigarettes not available)
                                             Potentially overlapping studies


     REF| REFGP|PRINC|                     OVERLAP|

  HOZAWA HOZAWA     1         ENRIGH/HOZAWA/HARIKK
  JACOBS JACOBS     1  JACOBS/DONTA1/DONTA2/PELKON
  HUHTI1 HUHTI1     1                HUHTI1/HUHTI2
  ENSTRO HAMMO2     2                HAMMO2/ENSTRO
  LEBOWI LEBOWI     1                 LEBOWI/SILVA
  FERRI2 FERRIS     1         FERRI1/FERRI2/FERRI3
     WEN    WEN     1                     WEN/LIAW
    TODD   TODD     1                  LAMBER/TODD
   KAHN2   KAHN     2                   KAHN/KAHN2

                                    Most-adjusted - insufficient data for meta-analysis
     REF|NRR|SEX|AGEL|AGEH|     REGION|BEGYR|PUBYR|STTYP|ONSET|      DISEAS|ADJ|SMOKSTA|   PRODUCT|    UNEXP|LOW| HI|

    KAHN   6   m   31   99      Am:USA  1954  1966    Pr   Inc    COPD:mort   2 Current       Cigs   Nev any  40   +
          RR|SIG|

       21.98   ?


  ________________________________________________________________________________________________________________________
                                            International Evidence on Smoking and COPD, Phase 3, Analysis run on 28-SEP-10

                                                    Table 1 - E - 4 -

                                IESCOPD - Meta-analysis of amount smoked : key value (2) 1
                            Any COPD, cigarettes (or any product if cigarettes not available)


This analysis is restricted to results for:
1) Eligible study on database
2) Outcome COPD
3) Current or ever smoking
4) Categorical dose-response data for amount smoked
5) vs never smoking base
6) Key value (scheme 2) = 1
7) Results complete enough for use in meta-analysis

Within each study, results are then selected (in the following order of preference, within each sex) for:
8) SMKSTA  : current, ever
9) UNEXP   : never any, never cigarettes
10) PROD    : cigarettes, cigarettes only, any product
11) For overlapping studies: principal rather than subsidiary studies
and then for single sex results (m, f) in preference to results for both sexes combined (b).

Results adjusted for the most potential confounders are then chosen in Sections -1 to -3
and results adjusted for the least confounders in Sections -4 to -6. (Those least-adjusted results which
actually differ from the most-adjusted are marked 'x' in column X in Section -4)

Section -7 shows excluded studies, together with the stage (as above) at which no qualifying
results were found.

Section -8 lists the potentially overlapping studies which have been included (1=principal, 2=subsidiary),
and any results which would have been included in preference except that they had data not complete enough
for use in meta-analysis. It also lists their significance (yes/no), if known.


  ________________________________________________________________________________________________________________________
                                            International Evidence on Smoking and COPD, Phase 3, Analysis run on 28-SEP-10

                                                   Table 1 - E - 4 - 1

                                IESCOPD - Meta-analysis of amount smoked : key value (2) 1
                            Any COPD, cigarettes (or any product if cigarettes not available)
                                                      Most-adjusted


     REF|NRR|SEX|AGEL|AGEH|     REGION|BEGYR|PUBYR|STTYP|ONSET|      DISEAS|ADJ|SMOKSTA|   PRODUCT|    UNEXP|LOW| HI|

    BEST  38   m   30   97   Am:Canada  1955  1967    Pr   Inc    COPD:mort   1 Current  Cigs only   Nev any   1   9
  ENSTRO   7   m   30   99      Am:USA  1960  2003    Pr   Inc    COPD:mort   1 Current  Cigs only   Nev any   1   9
  ENSTRO  16   f   30   99      Am:USA  1960  2003    Pr   Inc    COPD:mort   1 Current  Cigs only   Nev any   1   9
  FERRI2  25   m   25   80      Am:USA  1967  1971    CS  Prev     COPD:oth   1 Current       Cigs   Nev any   1   4
  FERRI2  42   f   25   80      Am:USA  1967  1971    CS  Prev     COPD:oth   1 Current       Cigs   Nev any   1   4
  JACOBS  26   m   40   84       Multi  1957  1999    Pr   Inc    COPD:mort   6 Current       Cigs  Nev cigs   1   9
   KAHN2  99   m   31   84      Am:USA  1954  1966    Pr   Inc    COPD:mort   1 Current       Cigs   Nev any   1   9
     LEE  22   m   35   82       Eu:UK  1964  1979    Pr   Inc    COPD:mort   1 Current  Cigs only   Nev any   1   9
     LEE  43   f   35   82       Eu:UK  1964  1979    Pr   Inc    COPD:mort   0 Current  Cigs only   Nev any   1   9
  LINDST  10   b   20   69    Eu:Scand     *  2001    CS  Prev        CB/EM   5 Current       Cigs  Nev cigs   1   4
  LUNDB1  10   b   46   77    Eu:Scand  1996  2003    CS  Prev      COPD:LF   4 Current       Cigs  Nev cigs   1   4
  NILSSO  10   m   18   99    Eu:Scand  1963  2001    Pr   Inc    COPD:mort   2 Current  Cigs only   Nev any   1   7
  NILSSO  15   f   18   99    Eu:Scand  1963  2001    Pr   Inc    COPD:mort   2 Current  Cigs only   Nev any   1   7
    TODD  22   m   35   81       Eu:UK  1965  1978    Pr   Inc    COPD:mort   1 Current  Cigs only   Nev any   1   9
  TVERDA   5   m   35   65    Eu:Scand  1972  1993    Pr   Inc    COPD:mort   2 Current  Cigs only  Nev cigs   1   9
  VONHER   9   m   30   99    Eu:Scand  1978  2000    CS  Prev        CB/EM   0 Current       Cigs   Nev any   1   9
  VONHER  16   f   30   99    Eu:Scand  1978  2000    CS  Prev        CB/EM   0 Current       Cigs   Nev any   1   9
   WEISS   6   m   50   69      Am:USA  1961  1963    CS  Prev      COPD:LF   0 Current  Cigs only   Nev any   1   9
  YAMAGU   4   b   40   99   Asia:FarE  1986  1988    CS  Prev      COPD:LF   6 Current       Cigs  Nev cigs   1   9


  ________________________________________________________________________________________________________________________
                                            International Evidence on Smoking and COPD, Phase 3, Analysis run on 28-SEP-10

                                                   Table 1 - E - 4 - 2

                                IESCOPD - Meta-analysis of amount smoked : key value (2) 1
                            Any COPD, cigarettes (or any product if cigarettes not available)
                                                      Most-adjusted


                        Number Exposed  Non-exposed
 REF    NRR SEX ADJ     Case    Cont    Case    Cont      RR        95.00%CI
*BEST   38  m   1         26       -       6       -      6.06 (  2.50-  14.71)
*ENSTRO 7   m   1         35       -     103       -      2.84 (  1.94-   4.17)
*ENSTRO 16  f   1         50       -     296       -      1.64 (  1.21-   2.22)
 Subtotal ENSTRO                                          2.03 (  1.60-   2.57)
 FERRI2 25  m   1          2       -      11       -      1.73 (  0.30-   9.90)
 FERRI2 42  f   1         10       -      54       -      2.08 (  0.98-   4.41)
 Subtotal FERRI2                                          2.02 (  1.01-   4.03)
*JACOBS 26  m   6         39       -      28       -      2.90 (  1.75-   4.85)
*KAHN2  99  m   1         28       -      31       -      4.14 (  2.48-   6.90)
*LEE    22  m   1          4       -       1       -      7.75 (  0.87-  68.85)
*LEE    43  f   0          2     342       5    1694      1.98 (  0.39-  10.17)
 Subtotal LEE                                             3.23 (  0.87-  11.98)
 LINDST 10  b   5          -       -     166       -      0.97 (  0.53-   1.77)
 LUNDB1 10  b   4          -       -      39       -      4.39 (  1.66-  11.60)
*NILSSO 10  m   2         19       -      31       -      3.30 (  1.86-   5.85)
*NILSSO 15  f   2         18       -      57       -      3.80 (  2.20-   6.57)
 Subtotal NILSSO                                          3.55 (  2.39-   5.28)
*TODD   22  m   1          6       -       1       -      8.94 (  1.08-  73.89)
*TVERDA 5   m   2          5       -       7       -      1.86 (  0.59-   5.88)
 VONHER 9   m   0         50     121      63     911      5.98 (  3.94-   9.07)
 VONHER 16  f   0         28     172     137    2888      3.43 (  2.22-   5.30)
 Subtotal VONHER                                          4.58 (  3.39-   6.19)
 WEISS  6   m   0          2      13       2      34      2.62 (  0.33-  20.55)
 YAMAGU 4   b   6          -       -     144       -      1.96 (  1.53-   2.53)
Partial Totals           324     648    1182    5527
*prospective study


 REF    NRR SEX ADJ             Ys       Ws       Qs       Ps
*BEST   38  m   1              1.80     4.89     3.49       0.00
*ENSTRO 7   m   1              1.04    26.24     0.20       0.00
*ENSTRO 16  f   1              0.49    41.72     8.93       0.00
 Subtotal ENSTRO               0.71    67.96     9.13
 FERRI2 25  m   1              0.55     1.26     0.21       0.54
 FERRI2 42  f   1              0.73     6.79     0.34       0.06
 Subtotal FERRI2               0.70     8.05     0.55
*JACOBS 26  m   6              1.06    14.79     0.17       0.00
*KAHN2  99  m   1              1.42    14.68     3.15       0.00
*LEE    22  m   1              2.05     0.80     0.96       0.07
*LEE    43  f   0              0.68     1.44     0.11       0.41
 Subtotal LEE                  1.17     2.24     1.06
 LINDST 10  b   5             -0.03    10.57    10.31       0.92
 LUNDB1 10  b   4              1.48     4.07     1.11       0.00
*NILSSO 10  m   2              1.19    11.70     0.66       0.00
*NILSSO 15  f   2              1.34    12.84     1.83       0.00
 Subtotal NILSSO               1.27    24.54     2.49
*TODD   22  m   1              2.19     0.86     1.31       0.04
*TVERDA 5   m   2              0.62     2.91     0.33       0.29
 VONHER 9   m   0              1.79    22.11    15.24       0.00
 VONHER 16  f   0              1.23    20.34     1.55       0.00
 Subtotal VONHER               1.52    42.44    16.79
 WEISS  6   m   0              0.96     0.90     0.00       0.36
 YAMAGU 4   b   6              0.67    60.74     4.91       0.00


  ________________________________________________________________________________________________________________________
                                            International Evidence on Smoking and COPD, Phase 3, Analysis run on 28-SEP-10

                                                   Table 1 - E - 4 - 2

                                IESCOPD - Meta-analysis of amount smoked : key value (2) 1
                            Any COPD, cigarettes (or any product if cigarettes not available)
                                                      Most-adjusted


                       N       19
                      NS       14


                      Wt   259.63
                 Het Chi    54.80
                 Het  df       18
                 Het  P       ***
               Fixed  RR     2.60
                     RRl     2.31
                     RRu     2.94
                      P       +++
              Random  RR     2.87
                     RRl     2.24
                     RRu     3.69
                      P       +++
               Asymm  P      N.S.


  ________________________________________________________________________________________________________________________
                                            International Evidence on Smoking and COPD, Phase 3, Analysis run on 28-SEP-10

                                                   Table 1 - E - 4 - 3

                                IESCOPD - Meta-analysis of amount smoked : key value (2) 1
                            Any COPD, cigarettes (or any product if cigarettes not available)
                                                      Most-adjusted


                       N       19
                      NS       14


                      Wt   259.63
                 Het Chi    54.80
                 Het  df       18
                 Het  P       ***
               Fixed  RR     2.60
                     RRl     2.31
                     RRu     2.94
                      P       +++
              Random  RR     2.87
                     RRl     2.24
                     RRu     3.69
                      P       +++
               Asymm  P      N.S.

                                   Sex
                             both      male    female     Total


                       N        3        11         5        19
                      NS        3        11         5        19


                      Wt    75.38    101.14     83.12    259.63
                 Het Chi     7.64     12.63     11.36     54.80
                 Het  df        2        10         4        18
                 Het  P         *      N.S.         *       ***
               Fixed  RR     1.85      3.73      2.29      2.60
                     RRl     1.48      3.07      1.85      2.31
                     RRu     2.32      4.53      2.84      2.94
                      P       +++       +++       +++       +++
              Random  RR     1.86      3.73      2.51      2.87
                     RRl     0.98      2.93      1.64      2.24
                     RRu     3.54      4.74      3.85      3.69
                      P       (+)       +++       +++       +++
             Between Chi                                  23.16
             Between  df                                      2
             Between  P                                     ***
             Btwn(F)  P                                       *

                                        Continent
                            NAmer    Europe      Asia  oth/mult     Total


                       N        7        10         1         1        19
                      NS        5         7         1         1        14


                      Wt    96.48     87.62     60.74     14.79    259.63
                 Het Chi    15.63     26.91      0.00      0.00     54.80
                 Het  df        6         9         0         0        18
                 Het  P         *        **      N.S.      N.S.       ***
               Fixed  RR     2.39      3.42      1.96      2.90      2.60
                     RRl     1.96      2.77      1.52      1.74      2.31
                     RRu     2.92      4.22      2.52      4.83      2.94
                      P       +++       +++       +++       +++       +++
              Random  RR     2.73      3.21      1.96      2.90      2.87
                     RRl     1.84      2.11      1.52      1.74      2.24
                     RRu     4.05      4.87      2.52      4.83      3.69
                      P       +++       +++       +++       +++       +++
             Between Chi                                            12.25
             Between  df                                                3
             Between  P                                                **
             Btwn(F)  P                                              N.S.


  ________________________________________________________________________________________________________________________
                                            International Evidence on Smoking and COPD, Phase 3, Analysis run on 28-SEP-10

                                                   Table 1 - E - 4 - 3

                                IESCOPD - Meta-analysis of amount smoked : key value (2) 1
                            Any COPD, cigarettes (or any product if cigarettes not available)
                                                      Most-adjusted
                               Study type
                               CC        Pr        CS     Total


                       N                 11         8        19
                      NS                  8         6        14


                      Wt             132.86    126.77    259.63
                 Het Chi              21.02     33.57     54.80
                 Het  df                 10         7        18
                 Het  P                   *       ***       ***
               Fixed  RR               2.68      2.53      2.60
                     RRl               2.26      2.13      2.31
                     RRu               3.17      3.01      2.94
                      P                 +++       +++       +++
              Random  RR               3.04      2.59      2.87
                     RRl               2.28      1.61      2.24
                     RRu               4.05      4.15      3.69
                      P                 +++       +++       +++
             Between Chi                                   0.21
             Between  df                                      1
             Between  P                                    N.S.
             Btwn(F)  P                                    N.S.

                          COPD subtype
                             mort        LF     other     Total


                       N       11         3         5        19
                      NS        8         3         3        14


                      Wt   132.86     65.71     61.06    259.63
                 Het Chi    21.02      2.53     25.49     54.80
                 Het  df       10         2         4        18
                 Het  P         *      N.S.       ***       ***
               Fixed  RR     2.68      2.07      3.14      2.60
                     RRl     2.26      1.62      2.45      2.31
                     RRu     3.17      2.63      4.04      2.94
                      P       +++       +++       +++       +++
              Random  RR     3.04      2.31      2.51      2.87
                     RRl     2.28      1.45      1.24      2.24
                     RRu     4.05      3.67      5.07      3.69
                      P       +++       +++         +       +++
             Between Chi                                   5.75
             Between  df                                      2
             Between  P                                     (*)
             Btwn(F)  P                                    N.S.

                             Smoking product
                              any      cigs  cigsonly     Total


                       N                  9        10        19
                      NS                  7         7        14


                      Wt             155.33    104.30    259.63
                 Het Chi              36.85     17.59     54.80
                 Het  df                  8         9        18
                 Het  P                 ***         *       ***
               Fixed  RR               2.69      2.49      2.60
                     RRl               2.29      2.05      2.31
                     RRu               3.14      3.02      2.94
                      P                 +++       +++       +++
              Random  RR               2.79      2.91      2.87
                     RRl               1.90      2.08      2.24
                     RRu               4.09      4.08      3.69
                      P                 +++       +++       +++
             Between Chi                                   0.36
             Between  df                                      1
             Between  P                                    N.S.
             Btwn(F)  P                                    N.S.
  ________________________________________________________________________________________________________________________
                                            International Evidence on Smoking and COPD, Phase 3, Analysis run on 28-SEP-10

                                                   Table 1 - E - 4 - 3

                                IESCOPD - Meta-analysis of amount smoked : key value (2) 1
                            Any COPD, cigarettes (or any product if cigarettes not available)
                                                      Most-adjusted
                                     Unexposed group
                          nev any   nev cig  nev+ any  nev+ cig     Total


                       N       14         5                            19
                      NS        9         5                            14


                      Wt   166.56     93.07                        259.63
                 Het Chi    34.22     10.13                         54.80
                 Het  df       13         4                            18
                 Het  P        **         *                           ***
               Fixed  RR     3.03      1.99                          2.60
                     RRl     2.60      1.63                          2.31
                     RRu     3.52      2.44                          2.94
                      P       +++       +++                           +++
              Random  RR     3.31      2.04                          2.87
                     RRl     2.47      1.34                          2.24
                     RRu     4.43      3.11                          3.69
                      P       +++       +++                           +++
             Between Chi                                            10.45
             Between  df                                                1
             Between  P                                                **
             Btwn(F)  P                                               (*)

                        Unexposed group (combining nev+ with main levels)
                          nev any   nev cig     Total


                       N       14         5        19
                      NS        9         5        14


                      Wt   166.56     93.07    259.63
                 Het Chi    34.22     10.13     54.80
                 Het  df       13         4        18
                 Het  P        **         *       ***
               Fixed  RR     3.03      1.99      2.60
                     RRl     2.60      1.63      2.31
                     RRu     3.52      2.44      2.94
                      P       +++       +++       +++
              Random  RR     3.31      2.04      2.87
                     RRl     2.47      1.34      2.24
                     RRu     4.43      3.11      3.69
                      P       +++       +++       +++
             Between Chi                        10.45
             Between  df                            1
             Between  P                            **
             Btwn(F)  P                           (*)


  ________________________________________________________________________________________________________________________
                                            International Evidence on Smoking and COPD, Phase 3, Analysis run on 28-SEP-10

                                                   Table 1 - E - 4 - 4

                                IESCOPD - Meta-analysis of amount smoked : key value (2) 1
                            Any COPD, cigarettes (or any product if cigarettes not available)
                                                      Least-adjusted


     REF|NRR|X|SEX|AGEL|AGEH|     REGION|BEGYR|PUBYR|STTYP|ONSET|      DISEAS|ADJ|SMOKSTA|   PRODUCT|    UNEXP|LOW| HI|

    BEST  38     m   30   97   Am:Canada  1955  1967    Pr   Inc    COPD:mort   1 Current  Cigs only   Nev any   1   9
  ENSTRO   7     m   30   99      Am:USA  1960  2003    Pr   Inc    COPD:mort   1 Current  Cigs only   Nev any   1   9
  ENSTRO  16     f   30   99      Am:USA  1960  2003    Pr   Inc    COPD:mort   1 Current  Cigs only   Nev any   1   9
  FERRI2  25     m   25   80      Am:USA  1967  1971    CS  Prev     COPD:oth   1 Current       Cigs   Nev any   1   4
  FERRI2  42     f   25   80      Am:USA  1967  1971    CS  Prev     COPD:oth   1 Current       Cigs   Nev any   1   4
  JACOBS  17 x   m   40   84       Multi  1957  1999    Pr   Inc    COPD:mort   2 Current       Cigs  Nev cigs   1   4
   KAHN2  99     m   31   84      Am:USA  1954  1966    Pr   Inc    COPD:mort   1 Current       Cigs   Nev any   1   9
     LEE   8 x   m   35   82       Eu:UK  1964  1979    Pr   Inc    COPD:mort   0 Current  Cigs only   Nev any   1   9
     LEE  43     f   35   82       Eu:UK  1964  1979    Pr   Inc    COPD:mort   0 Current  Cigs only   Nev any   1   9
  LINDST  10     b   20   69    Eu:Scand     *  2001    CS  Prev        CB/EM   5 Current       Cigs  Nev cigs   1   4
  LUNDB1  10     b   46   77    Eu:Scand  1996  2003    CS  Prev      COPD:LF   4 Current       Cigs  Nev cigs   1   4
  NILSSO  10     m   18   99    Eu:Scand  1963  2001    Pr   Inc    COPD:mort   2 Current  Cigs only   Nev any   1   7
  NILSSO  15     f   18   99    Eu:Scand  1963  2001    Pr   Inc    COPD:mort   2 Current  Cigs only   Nev any   1   7
    TODD   8 x   m   35   81       Eu:UK  1965  1978    Pr   Inc    COPD:mort   0 Current  Cigs only   Nev any   1   9
  TVERDA   5     m   35   65    Eu:Scand  1972  1993    Pr   Inc    COPD:mort   2 Current  Cigs only  Nev cigs   1   9
  VONHER   9     m   30   99    Eu:Scand  1978  2000    CS  Prev        CB/EM   0 Current       Cigs   Nev any   1   9
  VONHER  16     f   30   99    Eu:Scand  1978  2000    CS  Prev        CB/EM   0 Current       Cigs   Nev any   1   9
   WEISS   6     m   50   69      Am:USA  1961  1963    CS  Prev      COPD:LF   0 Current  Cigs only   Nev any   1   9
  YAMAGU   4     b   40   99   Asia:FarE  1986  1988    CS  Prev      COPD:LF   6 Current       Cigs  Nev cigs   1   9


  ________________________________________________________________________________________________________________________
                                            International Evidence on Smoking and COPD, Phase 3, Analysis run on 28-SEP-10

                                                   Table 1 - E - 4 - 5

                                IESCOPD - Meta-analysis of amount smoked : key value (2) 1
                            Any COPD, cigarettes (or any product if cigarettes not available)
                                                      Least-adjusted


                        Number Exposed  Non-exposed
 REF    NRR SEX ADJ     Case    Cont    Case    Cont      RR        95.00%CI
*BEST   38  m   1         26       -       6       -      6.06 (  2.50-  14.71)
*ENSTRO 7   m   1         35       -     103       -      2.84 (  1.94-   4.17)
*ENSTRO 16  f   1         50       -     296       -      1.64 (  1.21-   2.22)
 Subtotal ENSTRO                                          2.03 (  1.60-   2.57)
 FERRI2 25  m   1          2       -      11       -      1.73 (  0.30-   9.90)
 FERRI2 42  f   1         10       -      54       -      2.08 (  0.98-   4.41)
 Subtotal FERRI2                                          2.02 (  1.01-   4.03)
*JACOBS 17  m   2          9       -      28       -      2.14 (  0.97-   4.71)
*KAHN2  99  m   1         28       -      31       -      4.14 (  2.48-   6.90)
*LEE    8   m   0          4     192       1     347      7.23 (  0.81-  64.22)
*LEE    43  f   0          2     342       5    1694      1.98 (  0.39-  10.17)
 Subtotal LEE                                             3.15 (  0.85-  11.68)
 LINDST 10  b   5          -       -     166       -      0.97 (  0.53-   1.77)
 LUNDB1 10  b   4          -       -      39       -      4.39 (  1.66-  11.60)
*NILSSO 10  m   2         19       -      31       -      3.30 (  1.86-   5.85)
*NILSSO 15  f   2         18       -      57       -      3.80 (  2.20-   6.57)
 Subtotal NILSSO                                          3.55 (  2.39-   5.28)
*TODD   8   m   0          6     316       1     520      9.87 (  1.19-  81.63)
*TVERDA 5   m   2          5       -       7       -      1.86 (  0.59-   5.88)
 VONHER 9   m   0         50     121      63     911      5.98 (  3.94-   9.07)
 VONHER 16  f   0         28     172     137    2888      3.43 (  2.22-   5.30)
 Subtotal VONHER                                          4.58 (  3.39-   6.19)
 WEISS  6   m   0          2      13       2      34      2.62 (  0.33-  20.55)
 YAMAGU 4   b   6          -       -     144       -      1.96 (  1.53-   2.53)
Partial Totals           294    1156    1182    6394
*prospective study


 REF    NRR SEX ADJ             Ys       Ws       Qs       Ps
*BEST   38  m   1              1.80     4.89     3.58       0.00
*ENSTRO 7   m   1              1.04    26.24     0.25       0.00
*ENSTRO 16  f   1              0.49    41.72     8.51       0.00
 Subtotal ENSTRO               0.71    67.96     8.76
 FERRI2 25  m   1              0.55     1.26     0.20       0.54
 FERRI2 42  f   1              0.73     6.79     0.31       0.06
 Subtotal FERRI2               0.70     8.05     0.51
*JACOBS 17  m   2              0.76     6.15     0.21       0.06
*KAHN2  99  m   1              1.42    14.68     3.30       0.00
*LEE    8   m   0              1.98     0.81     0.86       0.08
*LEE    43  f   0              0.68     1.44     0.10       0.41
 Subtotal LEE                  1.15     2.24     0.96
 LINDST 10  b   5             -0.03    10.57    10.08       0.92
 LUNDB1 10  b   4              1.48     4.07     1.15       0.00
*NILSSO 10  m   2              1.19    11.70     0.72       0.00
*NILSSO 15  f   2              1.34    12.84     1.94       0.00
 Subtotal NILSSO               1.27    24.54     2.66
*TODD   8   m   0              2.29     0.86     1.55       0.03
*TVERDA 5   m   2              0.62     2.91     0.31       0.29
 VONHER 9   m   0              1.79    22.11    15.65       0.00
 VONHER 16  f   0              1.23    20.34     1.67       0.00
 Subtotal VONHER               1.52    42.44    17.32
 WEISS  6   m   0              0.96     0.90     0.00       0.36
 YAMAGU 4   b   6              0.67    60.74     4.54       0.00


  ________________________________________________________________________________________________________________________
                                            International Evidence on Smoking and COPD, Phase 3, Analysis run on 28-SEP-10

                                                   Table 1 - E - 4 - 5

                                IESCOPD - Meta-analysis of amount smoked : key value (2) 1
                            Any COPD, cigarettes (or any product if cigarettes not available)
                                                      Least-adjusted


                       N       19
                      NS       14


                      Wt   251.00
                 Het Chi    54.93
                 Het  df       18
                 Het  P       ***
               Fixed  RR     2.58
                     RRl     2.28
                     RRu     2.92
                      P       +++
              Random  RR     2.83
                     RRl     2.19
                     RRu     3.66
                      P       +++
               Asymm  P      N.S.


  ________________________________________________________________________________________________________________________
                                            International Evidence on Smoking and COPD, Phase 3, Analysis run on 28-SEP-10

                                                   Table 1 - E - 4 - 6

                                IESCOPD - Meta-analysis of amount smoked : key value (2) 1
                            Any COPD, cigarettes (or any product if cigarettes not available)
                                                      Least-adjusted


                       N       19
                      NS       14


                      Wt   251.00
                 Het Chi    54.93
                 Het  df       18
                 Het  P       ***
               Fixed  RR     2.58
                     RRl     2.28
                     RRu     2.92
                      P       +++
              Random  RR     2.83
                     RRl     2.19
                     RRu     3.66
                      P       +++
               Asymm  P      N.S.

                                   Sex
                             both      male    female     Total


                       N        3        11         5        19
                      NS        3        11         5        19


                      Wt    75.38     92.51     83.12    251.00
                 Het Chi     7.64     13.68     11.36     54.93
                 Het  df        2        10         4        18
                 Het  P         *      N.S.         *       ***
               Fixed  RR     1.85      3.75      2.29      2.58
                     RRl     1.48      3.05      1.85      2.28
                     RRu     2.32      4.59      2.84      2.92
                      P       +++       +++       +++       +++
              Random  RR     1.86      3.69      2.51      2.83
                     RRl     0.98      2.82      1.64      2.19
                     RRu     3.54      4.83      3.85      3.66
                      P       (+)       +++       +++       +++
             Between Chi                                  22.25
             Between  df                                      2
             Between  P                                     ***
             Btwn(F)  P                                       *

                                        Continent
                            NAmer    Europe      Asia  oth/mult     Total


                       N        7        10         1         1        19
                      NS        5         7         1         1        14


                      Wt    96.48     87.62     60.74      6.15    251.00
                 Het Chi    15.63     27.00      0.00      0.00     54.93
                 Het  df        6         9         0         0        18
                 Het  P         *        **      N.S.      N.S.       ***
               Fixed  RR     2.39      3.42      1.96      2.14      2.58
                     RRl     1.96      2.77      1.52      0.97      2.28
                     RRu     2.92      4.22      2.52      4.72      2.92
                      P       +++       +++       +++       (+)       +++
              Random  RR     2.73      3.21      1.96      2.14      2.83
                     RRl     1.84      2.11      1.52      0.97      2.19
                     RRu     4.05      4.88      2.52      4.72      3.66
                      P       +++       +++       +++       (+)       +++
             Between Chi                                            12.30
             Between  df                                                3
             Between  P                                                **
             Btwn(F)  P                                              N.S.


  ________________________________________________________________________________________________________________________
                                            International Evidence on Smoking and COPD, Phase 3, Analysis run on 28-SEP-10

                                                   Table 1 - E - 4 - 6

                                IESCOPD - Meta-analysis of amount smoked : key value (2) 1
                            Any COPD, cigarettes (or any product if cigarettes not available)
                                                      Least-adjusted
                               Study type
                               CC        Pr        CS     Total


                       N                 11         8        19
                      NS                  8         6        14


                      Wt             124.23    126.77    251.00
                 Het Chi              21.29     33.57     54.93
                 Het  df                 10         7        18
                 Het  P                   *       ***       ***
               Fixed  RR               2.62      2.53      2.58
                     RRl               2.20      2.13      2.28
                     RRu               3.13      3.01      2.92
                      P                 +++       +++       +++
              Random  RR               2.98      2.59      2.83
                     RRl               2.20      1.61      2.19
                     RRu               4.04      4.15      3.66
                      P                 +++       +++       +++
             Between Chi                                   0.08
             Between  df                                      1
             Between  P                                    N.S.
             Btwn(F)  P                                    N.S.

                          COPD subtype
                             mort        LF     other     Total


                       N       11         3         5        19
                      NS        8         3         3        14


                      Wt   124.23     65.71     61.06    251.00
                 Het Chi    21.29      2.53     25.49     54.93
                 Het  df       10         2         4        18
                 Het  P         *      N.S.       ***       ***
               Fixed  RR     2.62      2.07      3.14      2.58
                     RRl     2.20      1.62      2.45      2.28
                     RRu     3.13      2.63      4.04      2.92
                      P       +++       +++       +++       +++
              Random  RR     2.98      2.31      2.51      2.83
                     RRl     2.20      1.45      1.24      2.19
                     RRu     4.04      3.67      5.07      3.66
                      P       +++       +++         +       +++
             Between Chi                                   5.63
             Between  df                                      2
             Between  P                                     (*)
             Btwn(F)  P                                    N.S.

                             Smoking product
                              any      cigs  cigsonly     Total


                       N                  9        10        19
                      NS                  7         7        14


                      Wt             146.70    104.31    251.00
                 Het Chi              37.04     17.69     54.93
                 Het  df                  8         9        18
                 Het  P                 ***         *       ***
               Fixed  RR               2.64      2.49      2.58
                     RRl               2.24      2.06      2.28
                     RRu               3.10      3.02      2.92
                      P                 +++       +++       +++
              Random  RR               2.70      2.92      2.83
                     RRl               1.81      2.08      2.19
                     RRu               4.03      4.09      3.66
                      P                 +++       +++       +++
             Between Chi                                   0.20
             Between  df                                      1
             Between  P                                    N.S.
             Btwn(F)  P                                    N.S.
  ________________________________________________________________________________________________________________________
                                            International Evidence on Smoking and COPD, Phase 3, Analysis run on 28-SEP-10

                                                   Table 1 - E - 4 - 6

                                IESCOPD - Meta-analysis of amount smoked : key value (2) 1
                            Any COPD, cigarettes (or any product if cigarettes not available)
                                                      Least-adjusted
                                     Unexposed group
                          nev any   nev cig  nev+ any  nev+ cig     Total


                       N       14         5                            19
                      NS        9         5                            14


                      Wt   166.57     84.44                        251.00
                 Het Chi    34.31      7.76                         54.93
                 Het  df       13         4                            18
                 Het  P        **      N.S.                           ***
               Fixed  RR     3.03      1.87                          2.58
                     RRl     2.60      1.51                          2.28
                     RRu     3.52      2.32                          2.92
                      P       +++       +++                           +++
              Random  RR     3.31      1.87                          2.83
                     RRl     2.48      1.24                          2.19
                     RRu     4.43      2.81                          3.66
                      P       +++        ++                           +++
             Between Chi                                            12.87
             Between  df                                                1
             Between  P                                               ***
             Btwn(F)  P                                                 *

                        Unexposed group (combining nev+ with main levels)
                          nev any   nev cig     Total


                       N       14         5        19
                      NS        9         5        14


                      Wt   166.57     84.44    251.00
                 Het Chi    34.31      7.76     54.93
                 Het  df       13         4        18
                 Het  P        **      N.S.       ***
               Fixed  RR     3.03      1.87      2.58
                     RRl     2.60      1.51      2.28
                     RRu     3.52      2.32      2.92
                      P       +++       +++       +++
              Random  RR     3.31      1.87      2.83
                     RRl     2.48      1.24      2.19
                     RRu     4.43      2.81      3.66
                      P       +++        ++       +++
             Between Chi                        12.87
             Between  df                            1
             Between  P                           ***
             Btwn(F)  P                             *


  ________________________________________________________________________________________________________________________
                                            International Evidence on Smoking and COPD, Phase 3, Analysis run on 28-SEP-10

                                                   Table 1 - E - 4 - 7

                                IESCOPD - Meta-analysis of amount smoked : key value (2) 1
                            Any COPD, cigarettes (or any product if cigarettes not available)
                                 Excluded studies (and stage at which they were excluded)


1       CLARK COTTON  MEYER REMYJA RUTGER SNYDER SOBRAX     SU TAKEMU  WANG4   WEIR WHICKE ZALACA
2      ALDERS ANDER2 AUERBA   BANG  BECK1  BECK2 BJORNS  BROWN CERVER CHAPMA COATES COLLEG  DEAN2  DEANE DONTA2 DOPICO
       EHRLIC ENRIGH FINKLE FLETCH FOXMAN GOLDBE HAENSZ HARRIS  HAYES HIGGI2 HIGGI3 HIGGI6 HIRAYA HOLLA2 HOLLNA  HOUSE
       HRUBEC HUCHON JENSEN JINDA2  JOSHI JOUSI1   KATO KOTAN1  KUBIK LAMBER LANGE2 LANGHA LAVECC LUNDB2 MAGNUS MANFRE
       MELLST MENEZ1  MEREN MILLER  MILNE MOLLER   NAWA NEJJAR OGILVI  OMORI OSWAL1 OSWAL2 PANDEY  PRATT   REID RIMING
        RYDER SCHWAR  SHARP SHIMUR SOBRAD STJERN SUADIC SUTINE TAGER2 TROISI URRUTI VIEGI1 VIKGRE WAGEN2  WANG2    WIG
       WILHEL WILSO2  WOODS  WOOLF   ZOIA
3      VINEIS
4      ALESSA  AMIGO ANDER3 BEDNAR BROGGE  CHEN3  CHENG  COCCI DEJONG DEMARC DETORR DICKIN EKBERG  FIDAN FORAST FUKUCH
       GEIJER GULSVI HAMMO2 HARDIE HARIKK HEDMAN HIGGI4     HO HUHTI2 ITABAS JAENDI JOHANN KACHEL KARAKA KATANC KHOURY
          KIM  KIRAZ KLAYTO KOJIMA KOTAN2    LAI   LAM1   LAM2   LAM3  LANGE LINDBE   LIU2  MADOR MANNI1 MANNI2 MANNI3
       MARAN1 MARAN2 MARCUS MATHES MENEZ2 MENEZ3 MENEZ4 MENEZ5 MENEZ6 MONTNE NIEPSU NIHLEN   PEAT PELKON PEREZP  PRICE
       RENWIC RICCIO SARGEA SAWICK SHAHAB   SHIN SICHLE  SILVA STERLI  STROM  TAGER   TANG   THUN TRUPIN TSUSHI VESTBO
       VIEGI2 VOLLM1 VOLLM2   WALD WATSON WILSO1 WOJTYN   XIAO     XU ZIELI1 ZIELI2 ZIETKO
5       CHEN1 HAWTHO KULLER   LIU1
6      ANDER1  CHEN2 CLEMEN  DEAN1  DOLL1  DOLL2 FERRI3 GODTFR HOZAWA HUHTI1 HUHTI3 KRZYZA LEBOWI   LIAW MUELLE   PETO
       SPEIZE    WEN   YUAN
7        KAHN
11     DONTA1 FERRI1


  ________________________________________________________________________________________________________________________
                                            International Evidence on Smoking and COPD, Phase 3, Analysis run on 28-SEP-10

                                                   Table 1 - E - 4 - 8

                                IESCOPD - Meta-analysis of amount smoked : key value (2) 1
                            Any COPD, cigarettes (or any product if cigarettes not available)
                                             Potentially overlapping studies


     REF| REFGP|PRINC|                     OVERLAP|

  JACOBS JACOBS     1  JACOBS/DONTA1/DONTA2/PELKON
  LUNDB1 LUNDBA     1  LINDBE/LUNDB1/LUNDB2/HEDLUN
  ENSTRO HAMMO2     2                HAMMO2/ENSTRO
  FERRI2 FERRIS     1         FERRI1/FERRI2/FERRI3
    TODD   TODD     1                  LAMBER/TODD
   KAHN2   KAHN     2                   KAHN/KAHN2

                                    Most-adjusted - insufficient data for meta-analysis
     REF|NRR|SEX|AGEL|AGEH|     REGION|BEGYR|PUBYR|STTYP|ONSET|      DISEAS|ADJ|SMOKSTA|   PRODUCT|    UNEXP|LOW| HI|

    KAHN   3   m   31   99      Am:USA  1954  1966    Pr   Inc    COPD:mort   2 Current       Cigs   Nev any   1   9
          RR|SIG|

        4.84   ?


  ________________________________________________________________________________________________________________________
                                            International Evidence on Smoking and COPD, Phase 3, Analysis run on 28-SEP-10

                                                    Table 1 - E - 5 -

                               IESCOPD - Meta-analysis of amount smoked : key value (2) 10
                            Any COPD, cigarettes (or any product if cigarettes not available)


This analysis is restricted to results for:
1) Eligible study on database
2) Outcome COPD
3) Current or ever smoking
4) Categorical dose-response data for amount smoked
5) vs never smoking base
6) Key value (scheme 2) = 10
7) Results complete enough for use in meta-analysis

Within each study, results are then selected (in the following order of preference, within each sex) for:
8) SMKSTA  : current, ever
9) UNEXP   : never any, never cigarettes
10) PROD    : cigarettes, cigarettes only, any product
11) For overlapping studies: principal rather than subsidiary studies
and then for single sex results (m, f) in preference to results for both sexes combined (b).

Results adjusted for the most potential confounders are then chosen in Sections -1 to -3
and results adjusted for the least confounders in Sections -4 to -6. (Those least-adjusted results which
actually differ from the most-adjusted are marked 'x' in column X in Section -4)

Section -7 shows excluded studies, together with the stage (as above) at which no qualifying
results were found.

Section -8 lists the potentially overlapping studies which have been included (1=principal, 2=subsidiary),
and any results which would have been included in preference except that they had data not complete enough
for use in meta-analysis. It also lists their significance (yes/no), if known.


  ________________________________________________________________________________________________________________________
                                            International Evidence on Smoking and COPD, Phase 3, Analysis run on 28-SEP-10

                                                   Table 1 - E - 5 - 1

                               IESCOPD - Meta-analysis of amount smoked : key value (2) 10
                            Any COPD, cigarettes (or any product if cigarettes not available)
                                                      Most-adjusted


     REF|NRR|SEX|AGEL|AGEH|     REGION|BEGYR|PUBYR|STTYP|ONSET|      DISEAS|ADJ|SMOKSTA|   PRODUCT|    UNEXP|LOW| HI|

  ENSTRO   8   m   30   99      Am:USA  1960  2003    Pr   Inc    COPD:mort   1 Current  Cigs only   Nev any  10  19
  ENSTRO  17   f   30   99      Am:USA  1960  2003    Pr   Inc    COPD:mort   1 Current  Cigs only   Nev any  10  19
  FERRI2  26   m   25   80      Am:USA  1967  1971    CS  Prev     COPD:oth   1 Current       Cigs   Nev any   5  14
  FERRI2  43   f   25   80      Am:USA  1967  1971    CS  Prev     COPD:oth   1 Current       Cigs   Nev any   5  14
  JACOBS  19   m   40   84       Multi  1957  1999    Pr   Inc    COPD:mort   2 Current       Cigs  Nev cigs  10  19
     LEE  23   m   35   82       Eu:UK  1964  1979    Pr   Inc    COPD:mort   1 Current  Cigs only   Nev any  10  19
     LEE  44   f   35   82       Eu:UK  1964  1979    Pr   Inc    COPD:mort   0 Current  Cigs only   Nev any  10  19
  LINDST  11   b   20   69    Eu:Scand     *  2001    CS  Prev        CB/EM   5 Current       Cigs  Nev cigs   5  14
  LUNDB1  11   b   46   77    Eu:Scand  1996  2003    CS  Prev      COPD:LF   4 Current       Cigs  Nev cigs   5  14
  NILSSO  11   m   18   99    Eu:Scand  1963  2001    Pr   Inc    COPD:mort   2 Current  Cigs only   Nev any   8  15
  NILSSO  16   f   18   99    Eu:Scand  1963  2001    Pr   Inc    COPD:mort   2 Current  Cigs only   Nev any   8  15
    TODD  23   m   35   81       Eu:UK  1965  1978    Pr   Inc    COPD:mort   1 Current  Cigs only   Nev any  10  19
  TVERDA   6   m   35   65    Eu:Scand  1972  1993    Pr   Inc    COPD:mort   2 Current  Cigs only  Nev cigs  10  19
  VONHER  10   m   30   99    Eu:Scand  1978  2000    CS  Prev        CB/EM   0 Current       Cigs   Nev any  10  19
  VONHER  17   f   30   99    Eu:Scand  1978  2000    CS  Prev        CB/EM   0 Current       Cigs   Nev any  10  19


  ________________________________________________________________________________________________________________________
                                            International Evidence on Smoking and COPD, Phase 3, Analysis run on 28-SEP-10

                                                   Table 1 - E - 5 - 2

                               IESCOPD - Meta-analysis of amount smoked : key value (2) 10
                            Any COPD, cigarettes (or any product if cigarettes not available)
                                                      Most-adjusted


                        Number Exposed  Non-exposed
 REF    NRR SEX ADJ     Case    Cont    Case    Cont      RR        95.00%CI
*ENSTRO 8   m   1        125       -     103       -      5.46 (  4.19-   7.11)
*ENSTRO 17  f   1        214       -     296       -      5.69 (  4.73-   6.85)
 Subtotal ENSTRO                                          5.61 (  4.82-   6.53)
 FERRI2 26  m   1         16       -      11       -      2.55 (  1.08-   6.04)
 FERRI2 43  f   1         13       -      54       -      1.59 (  0.82-   3.09)
 Subtotal FERRI2                                          1.90 (  1.12-   3.21)
*JACOBS 19  m   2        104       -      28       -      4.29 (  2.66-   6.92)
*LEE    23  m   1         10       -       1       -      5.54 (  0.71-  43.11)
*LEE    44  f   0          2     606       5    1694      1.12 (  0.22-   5.75)
 Subtotal LEE                                             2.08 (  0.58-   7.49)
 LINDST 11  b   5          -       -     166       -      1.94 (  1.47-   2.57)
 LUNDB1 11  b   4          -       -      39       -      6.44 (  3.37-  11.60)
*NILSSO 11  m   2         36       -      31       -      8.46 (  5.15-  13.90)
*NILSSO 16  f   2         31       -      57       -     11.80 (  7.36-  18.90)
 Subtotal NILSSO                                         10.08 (  7.16-  14.18)
*TODD   23  m   1         12       -       1       -      6.56 (  0.86-  50.33)
*TVERDA 6   m   2         29       -       7       -      4.85 (  2.13-  11.08)
 VONHER 10  m   0        155     266      63     911      8.43 (  6.10-  11.64)
 VONHER 17  f   0         50     132     137    2888      7.98 (  5.53-  11.54)
 Subtotal VONHER                                          8.23 (  6.46-  10.49)
Partial Totals           797    1004     999    5493
*prospective study


 REF    NRR SEX ADJ             Ys       Ws       Qs       Ps
*ENSTRO 8   m   1              1.70    54.95     0.15       0.00
*ENSTRO 17  f   1              1.74   112.05     1.00       0.00
 Subtotal ENSTRO               1.73   167.00     1.15
 FERRI2 26  m   1              0.94     5.19     2.60       0.03
 FERRI2 43  f   1              0.46     8.73    12.17       0.17
 Subtotal FERRI2               0.64    13.92    14.77
*JACOBS 19  m   2              1.46    16.81     0.59       0.00
*LEE    23  m   1              1.71     0.91     0.00       0.10
*LEE    44  f   0              0.11     1.43     3.37       0.89
 Subtotal LEE                  0.73     2.34     3.37
 LINDST 11  b   5              0.66    49.24    47.45       0.00
 LUNDB1 11  b   4              1.86    10.06     0.48       0.00
*NILSSO 11  m   2              2.14    15.59     3.76       0.00
*NILSSO 16  f   2              2.47    17.28    11.72       0.00
 Subtotal NILSSO               2.31    32.86    15.48
*TODD   23  m   1              1.88     0.93     0.05       0.07
*TVERDA 6   m   2              1.58     5.65     0.02       0.00
 VONHER 10  m   0              2.13    36.79     8.72       0.00
 VONHER 17  f   0              2.08    28.39     5.33       0.00
 Subtotal VONHER               2.11    65.18    14.05

                       N       15
                      NS       10


                      Wt   363.98
                 Het Chi    97.42
                 Het  df       14
                 Het  P       ***
               Fixed  RR     5.18
                     RRl     4.67
                     RRu     5.74
                      P       +++
              Random  RR     4.93
                     RRl     3.59
                     RRu     6.77
                      P       +++
               Asymm  P      N.S.


  ________________________________________________________________________________________________________________________
                                            International Evidence on Smoking and COPD, Phase 3, Analysis run on 28-SEP-10

                                                   Table 1 - E - 5 - 3

                               IESCOPD - Meta-analysis of amount smoked : key value (2) 10
                            Any COPD, cigarettes (or any product if cigarettes not available)
                                                      Most-adjusted


                       N       15
                      NS       10


                      Wt   363.98
                 Het Chi    97.42
                 Het  df       14
                 Het  P       ***
               Fixed  RR     5.18
                     RRl     4.67
                     RRu     5.74
                      P       +++
              Random  RR     4.93
                     RRl     3.59
                     RRu     6.77
                      P       +++
               Asymm  P      N.S.

                                   Sex
                             both      male    female     Total


                       N        2         8         5        15
                      NS        2         8         5        15


                      Wt    59.29    136.81    167.88    363.98
                 Het Chi    12.02     12.51     29.98     97.42
                 Het  df        1         7         4        14
                 Het  P       ***       (*)       ***       ***
               Fixed  RR     2.38      6.06      5.99      5.18
                     RRl     1.84      5.13      5.15      4.67
                     RRu     3.07      7.17      6.97      5.74
                      P       +++       +++       +++       +++
              Random  RR     3.42      5.83      4.98      4.93
                     RRl     1.06      4.45      2.85      3.59
                     RRu    11.06      7.62      8.69      6.77
                      P         +       +++       +++       +++
             Between Chi                                  42.91
             Between  df                                      2
             Between  P                                     ***
             Btwn(F)  P                                       *

                                        Continent
                            NAmer    Europe      Asia  oth/mult     Total


                       N        4        10                   1        15
                      NS        2         7                   1        10


                      Wt   180.91    166.26               16.81    363.98
                 Het Chi    15.92     80.83                0.00     97.42
                 Het  df        3         9                   0        14
                 Het  P        **       ***                N.S.       ***
               Fixed  RR     5.16      5.29                4.29      5.18
                     RRl     4.46      4.55                2.66      4.67
                     RRu     5.97      6.16                6.92      5.74
                      P       +++       +++                 +++       +++
              Random  RR     3.88      5.68                4.29      4.93
                     RRl     2.51      3.35                2.66      3.59
                     RRu     6.00      9.63                6.92      6.77
                      P       +++       +++                 +++       +++
             Between Chi                                             0.68
             Between  df                                                2
             Between  P                                              N.S.
             Btwn(F)  P                                              N.S.


  ________________________________________________________________________________________________________________________
                                            International Evidence on Smoking and COPD, Phase 3, Analysis run on 28-SEP-10

                                                   Table 1 - E - 5 - 3

                               IESCOPD - Meta-analysis of amount smoked : key value (2) 10
                            Any COPD, cigarettes (or any product if cigarettes not available)
                                                      Most-adjusted
                               Study type
                               CC        Pr        CS     Total


                       N                  9         6        15
                      NS                  6         4        10


                      Wt             225.59    138.39    363.98
                 Het Chi              16.70     70.29     97.42
                 Het  df                  8         5        14
                 Het  P                   *       ***       ***
               Fixed  RR               5.91      4.17      5.18
                     RRl               5.19      3.53      4.67
                     RRu               6.74      4.93      5.74
                      P                 +++       +++       +++
              Random  RR               6.06      3.97      4.93
                     RRl               4.72      2.03      3.59
                     RRu               7.79      7.77      6.77
                      P                 +++       +++       +++
             Between Chi                                  10.43
             Between  df                                      1
             Between  P                                      **
             Btwn(F)  P                                    N.S.

                          COPD subtype
                             mort        LF     other     Total


                       N        9         1         5        15
                      NS        6         1         3        10


                      Wt   225.59     10.06    128.33    363.98
                 Het Chi    16.70      0.00     68.24     97.42
                 Het  df        8         0         4        14
                 Het  P         *      N.S.       ***       ***
               Fixed  RR     5.91      6.44      4.03      5.18
                     RRl     5.19      3.47      3.39      4.67
                     RRu     6.74     11.95      4.79      5.74
                      P       +++       +++       +++       +++
              Random  RR     6.06      6.44      3.60      4.93
                     RRl     4.72      3.47      1.67      3.59
                     RRu     7.79     11.95      7.78      6.77
                      P       +++       +++        ++       +++
             Between Chi                                  12.48
             Between  df                                      2
             Between  P                                      **
             Btwn(F)  P                                    N.S.

                             Smoking product
                              any      cigs  cigsonly     Total


                       N                  7         8        15
                      NS                  5         5        10


                      Wt             155.20    208.78    363.98
                 Het Chi              70.30     14.84     97.42
                 Het  df                  6         7        14
                 Het  P                 ***         *       ***
               Fixed  RR               4.18      6.07      5.18
                     RRl               3.58      5.30      4.67
                     RRu               4.90      6.95      5.74
                      P                 +++       +++       +++
              Random  RR               4.02      6.42      4.93
                     RRl               2.27      4.88      3.59
                     RRu               7.14      8.43      6.77
                      P                 +++       +++       +++
             Between Chi                                  12.29
             Between  df                                      1
             Between  P                                     ***
             Btwn(F)  P                                    N.S.
  ________________________________________________________________________________________________________________________
                                            International Evidence on Smoking and COPD, Phase 3, Analysis run on 28-SEP-10

                                                   Table 1 - E - 5 - 3

                               IESCOPD - Meta-analysis of amount smoked : key value (2) 10
                            Any COPD, cigarettes (or any product if cigarettes not available)
                                                      Most-adjusted
                                     Unexposed group
                          nev any   nev cig  nev+ any  nev+ cig     Total


                       N       11         4                            15
                      NS        6         4                            10


                      Wt   282.23     81.75                        363.98
                 Het Chi    40.13     18.37                         97.42
                 Het  df       10         3                            14
                 Het  P       ***       ***                           ***
               Fixed  RR     6.17      2.82                          5.18
                     RRl     5.49      2.27                          4.67
                     RRu     6.94      3.50                          5.74
                      P       +++       +++                           +++
              Random  RR     5.74      3.84                          4.93
                     RRl     4.28      2.05                          3.59
                     RRu     7.70      7.18                          6.77
                      P       +++       +++                           +++
             Between Chi                                            38.93
             Between  df                                                1
             Between  P                                               ***
             Btwn(F)  P                                                 *

                        Unexposed group (combining nev+ with main levels)
                          nev any   nev cig     Total


                       N       11         4        15
                      NS        6         4        10


                      Wt   282.23     81.75    363.98
                 Het Chi    40.13     18.37     97.42
                 Het  df       10         3        14
                 Het  P       ***       ***       ***
               Fixed  RR     6.17      2.82      5.18
                     RRl     5.49      2.27      4.67
                     RRu     6.94      3.50      5.74
                      P       +++       +++       +++
              Random  RR     5.74      3.84      4.93
                     RRl     4.28      2.05      3.59
                     RRu     7.70      7.18      6.77
                      P       +++       +++       +++
             Between Chi                        38.93
             Between  df                            1
             Between  P                           ***
             Btwn(F)  P                             *


  ________________________________________________________________________________________________________________________
                                            International Evidence on Smoking and COPD, Phase 3, Analysis run on 28-SEP-10

                                                   Table 1 - E - 5 - 4

                               IESCOPD - Meta-analysis of amount smoked : key value (2) 10
                            Any COPD, cigarettes (or any product if cigarettes not available)
                                                      Least-adjusted


     REF|NRR|X|SEX|AGEL|AGEH|     REGION|BEGYR|PUBYR|STTYP|ONSET|      DISEAS|ADJ|SMOKSTA|   PRODUCT|    UNEXP|LOW| HI|

  ENSTRO   8     m   30   99      Am:USA  1960  2003    Pr   Inc    COPD:mort   1 Current  Cigs only   Nev any  10  19
  ENSTRO  17     f   30   99      Am:USA  1960  2003    Pr   Inc    COPD:mort   1 Current  Cigs only   Nev any  10  19
  FERRI2  26     m   25   80      Am:USA  1967  1971    CS  Prev     COPD:oth   1 Current       Cigs   Nev any   5  14
  FERRI2  43     f   25   80      Am:USA  1967  1971    CS  Prev     COPD:oth   1 Current       Cigs   Nev any   5  14
  JACOBS  19     m   40   84       Multi  1957  1999    Pr   Inc    COPD:mort   2 Current       Cigs  Nev cigs  10  19
     LEE   9 x   m   35   82       Eu:UK  1964  1979    Pr   Inc    COPD:mort   0 Current  Cigs only   Nev any  10  19
     LEE  44     f   35   82       Eu:UK  1964  1979    Pr   Inc    COPD:mort   0 Current  Cigs only   Nev any  10  19
  LINDST  11     b   20   69    Eu:Scand     *  2001    CS  Prev        CB/EM   5 Current       Cigs  Nev cigs   5  14
  LUNDB1  11     b   46   77    Eu:Scand  1996  2003    CS  Prev      COPD:LF   4 Current       Cigs  Nev cigs   5  14
  NILSSO  11     m   18   99    Eu:Scand  1963  2001    Pr   Inc    COPD:mort   2 Current  Cigs only   Nev any   8  15
  NILSSO  16     f   18   99    Eu:Scand  1963  2001    Pr   Inc    COPD:mort   2 Current  Cigs only   Nev any   8  15
    TODD   9 x   m   35   81       Eu:UK  1965  1978    Pr   Inc    COPD:mort   0 Current  Cigs only   Nev any  10  19
  TVERDA   6     m   35   65    Eu:Scand  1972  1993    Pr   Inc    COPD:mort   2 Current  Cigs only  Nev cigs  10  19
  VONHER  10     m   30   99    Eu:Scand  1978  2000    CS  Prev        CB/EM   0 Current       Cigs   Nev any  10  19
  VONHER  17     f   30   99    Eu:Scand  1978  2000    CS  Prev        CB/EM   0 Current       Cigs   Nev any  10  19


  ________________________________________________________________________________________________________________________
                                            International Evidence on Smoking and COPD, Phase 3, Analysis run on 28-SEP-10

                                                   Table 1 - E - 5 - 5

                               IESCOPD - Meta-analysis of amount smoked : key value (2) 10
                            Any COPD, cigarettes (or any product if cigarettes not available)
                                                      Least-adjusted


                        Number Exposed  Non-exposed
 REF    NRR SEX ADJ     Case    Cont    Case    Cont      RR        95.00%CI
*ENSTRO 8   m   1        125       -     103       -      5.46 (  4.19-   7.11)
*ENSTRO 17  f   1        214       -     296       -      5.69 (  4.73-   6.85)
 Subtotal ENSTRO                                          5.61 (  4.82-   6.53)
 FERRI2 26  m   1         16       -      11       -      2.55 (  1.08-   6.04)
 FERRI2 43  f   1         13       -      54       -      1.59 (  0.82-   3.09)
 Subtotal FERRI2                                          1.90 (  1.12-   3.21)
*JACOBS 19  m   2        104       -      28       -      4.29 (  2.66-   6.92)
*LEE    9   m   0         10     673       1     347      5.16 (  0.66-  40.11)
*LEE    44  f   0          2     606       5    1694      1.12 (  0.22-   5.75)
 Subtotal LEE                                             2.03 (  0.56-   7.29)
 LINDST 11  b   5          -       -     166       -      1.94 (  1.47-   2.57)
 LUNDB1 11  b   4          -       -      39       -      6.44 (  3.37-  11.60)
*NILSSO 11  m   2         36       -      31       -      8.46 (  5.15-  13.90)
*NILSSO 16  f   2         31       -      57       -     11.80 (  7.36-  18.90)
 Subtotal NILSSO                                         10.08 (  7.16-  14.18)
*TODD   9   m   0         12    1023       1     520      6.10 (  0.80-  46.78)
*TVERDA 6   m   2         29       -       7       -      4.85 (  2.13-  11.08)
 VONHER 10  m   0        155     266      63     911      8.43 (  6.10-  11.64)
 VONHER 17  f   0         50     132     137    2888      7.98 (  5.53-  11.54)
 Subtotal VONHER                                          8.23 (  6.46-  10.49)
Partial Totals           797    2700     999    6360
*prospective study


 REF    NRR SEX ADJ             Ys       Ws       Qs       Ps
*ENSTRO 8   m   1              1.70    54.95     0.16       0.00
*ENSTRO 17  f   1              1.74   112.05     1.00       0.00
 Subtotal ENSTRO               1.73   167.00     1.16
 FERRI2 26  m   1              0.94     5.19     2.60       0.03
 FERRI2 43  f   1              0.46     8.73    12.16       0.17
 Subtotal FERRI2               0.64    13.92    14.76
*JACOBS 19  m   2              1.46    16.81     0.59       0.00
*LEE    9   m   0              1.64     0.91     0.00       0.12
*LEE    44  f   0              0.11     1.43     3.37       0.89
 Subtotal LEE                  0.71     2.35     3.37
 LINDST 11  b   5              0.66    49.24    47.42       0.00
 LUNDB1 11  b   4              1.86    10.06     0.48       0.00
*NILSSO 11  m   2              2.14    15.59     3.76       0.00
*NILSSO 16  f   2              2.47    17.28    11.73       0.00
 Subtotal NILSSO               2.31    32.86    15.49
*TODD   9   m   0              1.81     0.93     0.02       0.08
*TVERDA 6   m   2              1.58     5.65     0.02       0.00
 VONHER 10  m   0              2.13    36.79     8.74       0.00
 VONHER 17  f   0              2.08    28.39     5.34       0.00
 Subtotal VONHER               2.11    65.18    14.07

                       N       15
                      NS       10


                      Wt   363.98
                 Het Chi    97.39
                 Het  df       14
                 Het  P       ***
               Fixed  RR     5.18
                     RRl     4.67
                     RRu     5.74
                      P       +++
              Random  RR     4.91
                     RRl     3.58
                     RRu     6.75
                      P       +++
               Asymm  P      N.S.


  ________________________________________________________________________________________________________________________
                                            International Evidence on Smoking and COPD, Phase 3, Analysis run on 28-SEP-10

                                                   Table 1 - E - 5 - 6

                               IESCOPD - Meta-analysis of amount smoked : key value (2) 10
                            Any COPD, cigarettes (or any product if cigarettes not available)
                                                      Least-adjusted


                       N       15
                      NS       10


                      Wt   363.98
                 Het Chi    97.39
                 Het  df       14
                 Het  P       ***
               Fixed  RR     5.18
                     RRl     4.67
                     RRu     5.74
                      P       +++
              Random  RR     4.91
                     RRl     3.58
                     RRu     6.75
                      P       +++
               Asymm  P      N.S.

                                   Sex
                             both      male    female     Total


                       N        2         8         5        15
                      NS        2         8         5        15


                      Wt    59.29    136.81    167.88    363.98
                 Het Chi    12.02     12.52     29.98     97.39
                 Het  df        1         7         4        14
                 Het  P       ***       (*)       ***       ***
               Fixed  RR     2.38      6.06      5.99      5.18
                     RRl     1.84      5.12      5.15      4.67
                     RRu     3.07      7.16      6.97      5.74
                      P       +++       +++       +++       +++
              Random  RR     3.42      5.81      4.98      4.91
                     RRl     1.06      4.44      2.85      3.58
                     RRu    11.06      7.61      8.69      6.75
                      P         +       +++       +++       +++
             Between Chi                                  42.87
             Between  df                                      2
             Between  P                                     ***
             Btwn(F)  P                                       *

                                        Continent
                            NAmer    Europe      Asia  oth/mult     Total


                       N        4        10                   1        15
                      NS        2         7                   1        10


                      Wt   180.91    166.26               16.81    363.98
                 Het Chi    15.92     80.80                0.00     97.39
                 Het  df        3         9                   0        14
                 Het  P        **       ***                N.S.       ***
               Fixed  RR     5.16      5.29                4.29      5.18
                     RRl     4.46      4.54                2.66      4.67
                     RRu     5.97      6.16                6.92      5.74
                      P       +++       +++                 +++       +++
              Random  RR     3.88      5.64                4.29      4.91
                     RRl     2.51      3.33                2.66      3.58
                     RRu     6.00      9.57                6.92      6.75
                      P       +++       +++                 +++       +++
             Between Chi                                             0.67
             Between  df                                                2
             Between  P                                              N.S.
             Btwn(F)  P                                              N.S.


  ________________________________________________________________________________________________________________________
                                            International Evidence on Smoking and COPD, Phase 3, Analysis run on 28-SEP-10

                                                   Table 1 - E - 5 - 6

                               IESCOPD - Meta-analysis of amount smoked : key value (2) 10
                            Any COPD, cigarettes (or any product if cigarettes not available)
                                                      Least-adjusted
                               Study type
                               CC        Pr        CS     Total


                       N                  9         6        15
                      NS                  6         4        10


                      Wt             225.59    138.39    363.98
                 Het Chi              16.71     70.29     97.39
                 Het  df                  8         5        14
                 Het  P                   *       ***       ***
               Fixed  RR               5.91      4.17      5.18
                     RRl               5.19      3.53      4.67
                     RRu               6.73      4.93      5.74
                      P                 +++       +++       +++
              Random  RR               6.05      3.97      4.91
                     RRl               4.71      2.03      3.58
                     RRu               7.77      7.77      6.75
                      P                 +++       +++       +++
             Between Chi                                  10.40
             Between  df                                      1
             Between  P                                      **
             Btwn(F)  P                                    N.S.

                          COPD subtype
                             mort        LF     other     Total


                       N        9         1         5        15
                      NS        6         1         3        10


                      Wt   225.59     10.06    128.33    363.98
                 Het Chi    16.71      0.00     68.24     97.39
                 Het  df        8         0         4        14
                 Het  P         *      N.S.       ***       ***
               Fixed  RR     5.91      6.44      4.03      5.18
                     RRl     5.19      3.47      3.39      4.67
                     RRu     6.73     11.95      4.79      5.74
                      P       +++       +++       +++       +++
              Random  RR     6.05      6.44      3.60      4.91
                     RRl     4.71      3.47      1.67      3.58
                     RRu     7.77     11.95      7.78      6.75
                      P       +++       +++        ++       +++
             Between Chi                                  12.44
             Between  df                                      2
             Between  P                                      **
             Btwn(F)  P                                    N.S.

                             Smoking product
                              any      cigs  cigsonly     Total


                       N                  7         8        15
                      NS                  5         5        10


                      Wt             155.20    208.78    363.98
                 Het Chi              70.30     14.85     97.39
                 Het  df                  6         7        14
                 Het  P                 ***         *       ***
               Fixed  RR               4.18      6.06      5.18
                     RRl               3.58      5.29      4.67
                     RRu               4.90      6.94      5.74
                      P                 +++       +++       +++
              Random  RR               4.02      6.40      4.91
                     RRl               2.27      4.87      3.58
                     RRu               7.14      8.41      6.75
                      P                 +++       +++       +++
             Between Chi                                  12.25
             Between  df                                      1
             Between  P                                     ***
             Btwn(F)  P                                    N.S.
  ________________________________________________________________________________________________________________________
                                            International Evidence on Smoking and COPD, Phase 3, Analysis run on 28-SEP-10

                                                   Table 1 - E - 5 - 6

                               IESCOPD - Meta-analysis of amount smoked : key value (2) 10
                            Any COPD, cigarettes (or any product if cigarettes not available)
                                                      Least-adjusted
                                     Unexposed group
                          nev any   nev cig  nev+ any  nev+ cig     Total


                       N       11         4                            15
                      NS        6         4                            10


                      Wt   282.23     81.75                        363.98
                 Het Chi    40.15     18.37                         97.39
                 Het  df       10         3                            14
                 Het  P       ***       ***                           ***
               Fixed  RR     6.17      2.82                          5.18
                     RRl     5.49      2.27                          4.67
                     RRu     6.94      3.50                          5.74
                      P       +++       +++                           +++
              Random  RR     5.73      3.84                          4.91
                     RRl     4.27      2.05                          3.58
                     RRu     7.68      7.18                          6.75
                      P       +++       +++                           +++
             Between Chi                                            38.88
             Between  df                                                1
             Between  P                                               ***
             Btwn(F)  P                                                 *

                        Unexposed group (combining nev+ with main levels)
                          nev any   nev cig     Total


                       N       11         4        15
                      NS        6         4        10


                      Wt   282.23     81.75    363.98
                 Het Chi    40.15     18.37     97.39
                 Het  df       10         3        14
                 Het  P       ***       ***       ***
               Fixed  RR     6.17      2.82      5.18
                     RRl     5.49      2.27      4.67
                     RRu     6.94      3.50      5.74
                      P       +++       +++       +++
              Random  RR     5.73      3.84      4.91
                     RRl     4.27      2.05      3.58
                     RRu     7.68      7.18      6.75
                      P       +++       +++       +++
             Between Chi                        38.88
             Between  df                            1
             Between  P                           ***
             Btwn(F)  P                             *


  ________________________________________________________________________________________________________________________
                                            International Evidence on Smoking and COPD, Phase 3, Analysis run on 28-SEP-10

                                                   Table 1 - E - 5 - 7

                               IESCOPD - Meta-analysis of amount smoked : key value (2) 10
                            Any COPD, cigarettes (or any product if cigarettes not available)
                                 Excluded studies (and stage at which they were excluded)


1       CLARK COTTON  MEYER REMYJA RUTGER SNYDER SOBRAX     SU TAKEMU  WANG4   WEIR WHICKE ZALACA
2      ALDERS ANDER2 AUERBA   BANG  BECK1  BECK2 BJORNS  BROWN CERVER CHAPMA COATES COLLEG  DEAN2  DEANE DONTA2 DOPICO
       EHRLIC ENRIGH FINKLE FLETCH FOXMAN GOLDBE HAENSZ HARRIS  HAYES HIGGI2 HIGGI3 HIGGI6 HIRAYA HOLLA2 HOLLNA  HOUSE
       HRUBEC HUCHON JENSEN JINDA2  JOSHI JOUSI1   KATO KOTAN1  KUBIK LAMBER LANGE2 LANGHA LAVECC LUNDB2 MAGNUS MANFRE
       MELLST MENEZ1  MEREN MILLER  MILNE MOLLER   NAWA NEJJAR OGILVI  OMORI OSWAL1 OSWAL2 PANDEY  PRATT   REID RIMING
        RYDER SCHWAR  SHARP SHIMUR SOBRAD STJERN SUADIC SUTINE TAGER2 TROISI URRUTI VIEGI1 VIKGRE WAGEN2  WANG2    WIG
       WILHEL WILSO2  WOODS  WOOLF   ZOIA
3      VINEIS
4      ALESSA  AMIGO ANDER3 BEDNAR BROGGE  CHEN3  CHENG  COCCI DEJONG DEMARC DETORR DICKIN EKBERG  FIDAN FORAST FUKUCH
       GEIJER GULSVI HAMMO2 HARDIE HARIKK HEDMAN HIGGI4     HO HUHTI2 ITABAS JAENDI JOHANN KACHEL KARAKA KATANC KHOURY
          KIM  KIRAZ KLAYTO KOJIMA KOTAN2    LAI   LAM1   LAM2   LAM3  LANGE LINDBE   LIU2  MADOR MANNI1 MANNI2 MANNI3
       MARAN1 MARAN2 MARCUS MATHES MENEZ2 MENEZ3 MENEZ4 MENEZ5 MENEZ6 MONTNE NIEPSU NIHLEN   PEAT PELKON PEREZP  PRICE
       RENWIC RICCIO SARGEA SAWICK SHAHAB   SHIN SICHLE  SILVA STERLI  STROM  TAGER   TANG   THUN TRUPIN TSUSHI VESTBO
       VIEGI2 VOLLM1 VOLLM2   WALD WATSON WILSO1 WOJTYN   XIAO     XU ZIELI1 ZIELI2 ZIETKO
5       CHEN1 HAWTHO KULLER   LIU1
6      ANDER1   BEST  CHEN2 CLEMEN  DEAN1  DOLL1  DOLL2 FERRI3 GODTFR HOZAWA HUHTI1 HUHTI3   KAHN  KAHN2 KRZYZA LEBOWI
         LIAW MUELLE   PETO SPEIZE  WEISS    WEN YAMAGU   YUAN
11     DONTA1 FERRI1


  ________________________________________________________________________________________________________________________
                                            International Evidence on Smoking and COPD, Phase 3, Analysis run on 28-SEP-10

                                                   Table 1 - E - 5 - 8

                               IESCOPD - Meta-analysis of amount smoked : key value (2) 10
                            Any COPD, cigarettes (or any product if cigarettes not available)
                                             Potentially overlapping studies


     REF| REFGP|PRINC|                     OVERLAP|

  JACOBS JACOBS     1  JACOBS/DONTA1/DONTA2/PELKON
  LUNDB1 LUNDBA     1  LINDBE/LUNDB1/LUNDB2/HEDLUN
  ENSTRO HAMMO2     2                HAMMO2/ENSTRO
  FERRI2 FERRIS     1         FERRI1/FERRI2/FERRI3
    TODD   TODD     1                  LAMBER/TODD


  ________________________________________________________________________________________________________________________
                                            International Evidence on Smoking and COPD, Phase 3, Analysis run on 28-SEP-10

                                                    Table 1 - E - 6 -

                               IESCOPD - Meta-analysis of amount smoked : key value (2) 20
                            Any COPD, cigarettes (or any product if cigarettes not available)


This analysis is restricted to results for:
1) Eligible study on database
2) Outcome COPD
3) Current or ever smoking
4) Categorical dose-response data for amount smoked
5) vs never smoking base
6) Key value (scheme 2) = 20
7) Results complete enough for use in meta-analysis

Within each study, results are then selected (in the following order of preference, within each sex) for:
8) SMKSTA  : current, ever
9) UNEXP   : never any, never cigarettes
10) PROD    : cigarettes, cigarettes only, any product
11) For overlapping studies: principal rather than subsidiary studies
and then for single sex results (m, f) in preference to results for both sexes combined (b).

Results adjusted for the most potential confounders are then chosen in Sections -1 to -3
and results adjusted for the least confounders in Sections -4 to -6. (Those least-adjusted results which
actually differ from the most-adjusted are marked 'x' in column X in Section -4)

Section -7 shows excluded studies, together with the stage (as above) at which no qualifying
results were found.

Section -8 lists the potentially overlapping studies which have been included (1=principal, 2=subsidiary),
and any results which would have been included in preference except that they had data not complete enough
for use in meta-analysis. It also lists their significance (yes/no), if known.


  ________________________________________________________________________________________________________________________
                                            International Evidence on Smoking and COPD, Phase 3, Analysis run on 28-SEP-10

                                                   Table 1 - E - 6 - 1

                               IESCOPD - Meta-analysis of amount smoked : key value (2) 20
                            Any COPD, cigarettes (or any product if cigarettes not available)
                                                      Most-adjusted


     REF|NRR|SEX|AGEL|AGEH|     REGION|BEGYR|PUBYR|STTYP|ONSET|      DISEAS|ADJ|SMOKSTA|   PRODUCT|    UNEXP|LOW| HI|

  ANDER1  12   m   25   74   Am:Canada  1963  1965    CS  Prev     COPD:oth   1 Current       Cigs   Nev any  15  24
   DEAN1  25   m   35   99       Eu:UK  1969  1977    CC  Prev    COPD:mort   3 Current MCigs only   Nev any  13  22
   DEAN1  65   f   35   99       Eu:UK  1969  1977    CC  Prev    COPD:mort   3 Current MCigs only   Nev any  13  22
   DOLL1   8   m   20   99       Eu:UK  1951  1994    Pr   Inc    COPD:mort   2 Current  Cigs only   Nev any  15  24
   DOLL2   5   f   20   99       Eu:UK  1951  1980    Pr   Inc    COPD:mort   1 Current  Cigs only   Nev any  15  24
  ENSTRO   9   m   30   99      Am:USA  1960  2003    Pr   Inc    COPD:mort   1 Current  Cigs only   Nev any  20  20
  ENSTRO  18   f   30   99      Am:USA  1960  2003    Pr   Inc    COPD:mort   1 Current  Cigs only   Nev any  20  20
  FERRI2  27   m   25   80      Am:USA  1967  1971    CS  Prev     COPD:oth   1 Current       Cigs   Nev any  15  24
  FERRI2  44   f   25   80      Am:USA  1967  1971    CS  Prev     COPD:oth   1 Current       Cigs   Nev any  15  24
  HOZAWA   5   b   45   64      Am:USA  1987  2006    CS  Prev      COPD:LF   0 Current       Cigs  Nev cigs  15  29
  HUHTI1  27   m   40   64    Eu:Scand  1961  1965    CS  Prev    CB/EM/Ast   1 Current       Cigs   Nev any  15  24
  HUHTI3  13   m   25   69    Eu:Scand  1968  1978    CS  Prev      COPD:LF   1 Current        Any   Nev any  15  24
  JACOBS  20   m   40   84       Multi  1957  1999    Pr   Inc    COPD:mort   2 Current       Cigs  Nev cigs  20  29
     LEE  24   m   35   82       Eu:UK  1964  1979    Pr   Inc    COPD:mort   1 Current  Cigs only   Nev any  20  20
     LEE  45   f   35   82       Eu:UK  1964  1979    Pr   Inc    COPD:mort   0 Current  Cigs only   Nev any  20  20
  MUELLE  26   m   20   69      Am:USA  1967  1971    CS  Prev      COPD:LF   1 Current       Cigs   Nev any  15  24
    TODD  24   m   35   81       Eu:UK  1965  1978    Pr   Inc    COPD:mort   1 Current  Cigs only   Nev any  20  20
  VONHER  11   m   30   99    Eu:Scand  1978  2000    CS  Prev        CB/EM   0 Current       Cigs   Nev any  20  29
  VONHER  18   f   30   99    Eu:Scand  1978  2000    CS  Prev        CB/EM   0 Current       Cigs   Nev any  20  29
     WEN   3   m   35   99   Asia:FarE  1982  2004    Pr   Inc    COPD:mort   1 Current       Cigs  Nev cigs  11  20


  ________________________________________________________________________________________________________________________
                                            International Evidence on Smoking and COPD, Phase 3, Analysis run on 28-SEP-10

                                                   Table 1 - E - 6 - 2

                               IESCOPD - Meta-analysis of amount smoked : key value (2) 20
                            Any COPD, cigarettes (or any product if cigarettes not available)
                                                      Most-adjusted


                        Number Exposed  Non-exposed
 REF    NRR SEX ADJ     Case    Cont    Case    Cont      RR        95.00%CI
 ANDER1 12  m   1          8       -       2       -      5.93 (  1.14-  30.72)
 DEAN1  25  m   3         75       -      47       -      2.56 (  1.64-   4.01)
 DEAN1  65  f   3         21       -     120       -      2.23 (  1.26-   3.95)
 Subtotal DEAN1                                           2.43 (  1.71-   3.45)
*DOLL1  8   m   2         61       -      12       -     11.20 (  6.06-  20.68)
*DOLL2  5   f   1          4       -       1       -     28.50 (  3.19- 255.00)
*ENSTRO 9   m   1        326       -     103       -      8.30 (  6.62-  10.40)
*ENSTRO 18  f   1        309       -     296       -      9.32 (  7.85-  11.06)
 Subtotal ENSTRO                                          8.93 (  7.79-  10.24)
 FERRI2 27  m   1         55       -      11       -      4.99 (  2.42-  10.31)
 FERRI2 44  f   1         28       -      54       -      2.89 (  1.72-   4.87)
 Subtotal FERRI2                                          3.48 (  2.28-   5.31)
 HOZAWA 5   b   0        822    1020     872    5019      4.64 (  4.13-   5.21)
 HUHTI1 27  m   1         86       -       7       -     11.82 (  5.14-  27.19)
 HUHTI3 13  m   1         14       -       3       -      5.92 (  1.80-  19.47)
*JACOBS 20  m   2         61       -      28       -      4.00 (  2.43-   6.58)
*LEE    24  m   1          7       -       1       -      7.54 (  0.93-  60.98)
*LEE    45  f   0          0     212       5    1694      0.72~(  0.04-  13.06)
 Subtotal LEE                                             3.37 (  0.62-  18.37)
 MUELLE 26  m   1         14       -       2       -      6.82 (  1.41-  33.04)
*TODD   24  m   1          8       -       1       -      9.31 (  1.17-  74.18)
 VONHER 11  m   0        217     174      63     911     18.03 ( 13.04-  24.93)
 VONHER 18  f   0         40      65     137    2888     12.97 (  8.44-  19.93)
 Subtotal VONHER                                         16.00 ( 12.36-  20.73)
*WEN    3   m   1         62       -      52       -      2.65 (  1.68-   4.18)
Partial Totals          2218    1471    1817   10512
*prospective study                                        ~ With 0.5 adjustment for zero


 REF    NRR SEX ADJ             Ys       Ws       Qs       Ps
 ANDER1 12  m   1              1.78     1.42     0.00       0.03
 DEAN1  25  m   3              0.94    19.22    14.74       0.00
 DEAN1  65  f   3              0.80    11.77    12.09       0.01
 Subtotal DEAN1                0.89    30.99    26.83
*DOLL1  8   m   2              2.42    10.20     3.67       0.00
*DOLL2  5   f   1              3.35     0.80     1.88       0.00
*ENSTRO 9   m   1              2.12    75.31     6.80       0.00
*ENSTRO 18  f   1              2.23   130.74    22.68       0.00
 Subtotal ENSTRO               2.19   206.05    29.48
 FERRI2 27  m   1              1.61     7.31     0.32       0.00
 FERRI2 44  f   1              1.06    14.19     8.07       0.00
 Subtotal FERRI2               1.25    21.50     8.39
 HOZAWA 5   b   0              1.53   282.25    22.33       0.00
 HUHTI1 27  m   1              2.47     5.54     2.37       0.00
 HUHTI3 13  m   1              1.78     2.71     0.00       0.00
*JACOBS 20  m   2              1.39    15.49     2.86       0.00
*LEE    24  m   1              2.02     0.88     0.04       0.06
*LEE    45  f   0             -0.32     0.46     2.10       0.83
 Subtotal LEE                  1.22     1.34     2.14
 MUELLE 26  m   1              1.92     1.54     0.02       0.02
*TODD   24  m   1              2.23     0.89     0.15       0.04
 VONHER 11  m   0              2.89    36.60    42.41       0.00
 VONHER 18  f   0              2.56    20.82    11.62       0.00
 Subtotal VONHER               2.77    57.42    54.04
*WEN    3   m   1              0.97    18.49    13.08       0.00


  ________________________________________________________________________________________________________________________
                                            International Evidence on Smoking and COPD, Phase 3, Analysis run on 28-SEP-10

                                                   Table 1 - E - 6 - 2

                               IESCOPD - Meta-analysis of amount smoked : key value (2) 20
                            Any COPD, cigarettes (or any product if cigarettes not available)
                                                      Most-adjusted


                       N       20
                      NS       15


                      Wt   656.62
                 Het Chi   167.26
                 Het  df       19
                 Het  P       ***
               Fixed  RR     6.15
                     RRl     5.69
                     RRu     6.63
                      P       +++
              Random  RR     6.01
                     RRl     4.45
                     RRu     8.13
                      P       +++
               Asymm  P      N.S.


  ________________________________________________________________________________________________________________________
                                            International Evidence on Smoking and COPD, Phase 3, Analysis run on 28-SEP-10

                                                   Table 1 - E - 6 - 3

                               IESCOPD - Meta-analysis of amount smoked : key value (2) 20
                            Any COPD, cigarettes (or any product if cigarettes not available)
                                                      Most-adjusted


                       N       20
                      NS       15


                      Wt   656.62
                 Het Chi   167.26
                 Het  df       19
                 Het  P       ***
               Fixed  RR     6.15
                     RRl     5.69
                     RRu     6.63
                      P       +++
              Random  RR     6.01
                     RRl     4.45
                     RRu     8.13
                      P       +++
               Asymm  P      N.S.

                                   Sex
                             both      male    female     Total


                       N        1        13         6        20
                      NS        1        13         6        20


                      Wt   282.25    195.59    178.78    656.62
                 Het Chi     0.00     81.31     45.77    167.26
                 Het  df        0        12         5        19
                 Het  P      N.S.       ***       ***       ***
               Fixed  RR     4.64      7.23      8.02      6.15
                     RRl     4.13      6.28      6.93      5.69
                     RRu     5.21      8.32      9.29      6.63
                      P       +++       +++       +++       +++
              Random  RR     4.64      6.42      5.57      6.01
                     RRl     4.13      4.12      2.79      4.45
                     RRu     5.21     10.00     11.16      8.13
                      P       +++       +++       +++       +++
             Between Chi                                  40.18
             Between  df                                      2
             Between  P                                     ***
             Btwn(F)  P                                     (*)

                                        Continent
                            NAmer    Europe      Asia  oth/mult     Total


                       N        7        11         1         1        20
                      NS        5         8         1         1        15


                      Wt   512.76    109.88     18.49     15.49    656.62
                 Het Chi    59.82     78.88      0.00      0.00    167.26
                 Het  df        6        10         0         0        19
                 Het  P       ***       ***      N.S.      N.S.       ***
               Fixed  RR     5.97      8.58      2.65      4.00      6.15
                     RRl     5.48      7.11      1.68      2.43      5.69
                     RRu     6.51     10.34      4.18      6.58      6.63
                      P       +++       +++       +++       +++       +++
              Random  RR     5.84      7.32      2.65      4.00      6.01
                     RRl     3.98      3.90      1.68      2.43      4.45
                     RRu     8.55     13.74      4.18      6.58      8.13
                      P       +++       +++       +++       +++       +++
             Between Chi                                            28.56
             Between  df                                                3
             Between  P                                               ***
             Btwn(F)  P                                              N.S.


  ________________________________________________________________________________________________________________________
                                            International Evidence on Smoking and COPD, Phase 3, Analysis run on 28-SEP-10

                                                   Table 1 - E - 6 - 3

                               IESCOPD - Meta-analysis of amount smoked : key value (2) 20
                            Any COPD, cigarettes (or any product if cigarettes not available)
                                                      Most-adjusted
                               Study type
                               CC        Pr        CS     Total


                       N        2         9         9        20
                      NS        1         7         7        15


                      Wt    30.99    253.26    372.37    656.62
                 Het Chi     0.14     38.19     84.22    167.26
                 Het  df        1         8         8        19
                 Het  P      N.S.       ***       ***       ***
               Fixed  RR     2.43      7.84      5.62      6.15
                     RRl     1.71      6.93      5.08      5.69
                     RRu     3.45      8.87      6.22      6.63
                      P       +++       +++       +++       +++
              Random  RR     2.43      6.45      7.23      6.01
                     RRl     1.71      4.34      4.23      4.45
                     RRu     3.45      9.60     12.36      8.13
                      P       +++       +++       +++       +++
             Between Chi                                  44.71
             Between  df                                      2
             Between  P                                     ***
             Btwn(F)  P                                     (*)

                          COPD subtype
                             mort        LF     other     Total


                       N       11         3         6        20
                      NS        8         3         4        15


                      Wt   284.25    286.50     85.87    656.62
                 Het Chi    76.26      0.39     39.83    167.26
                 Het  df       10         2         5        19
                 Het  P       ***      N.S.       ***       ***
               Fixed  RR     6.90      4.66     10.54      6.15
                     RRl     6.15      4.15      8.53      5.69
                     RRu     7.75      5.23     13.02      6.63
                      P       +++       +++       +++       +++
              Random  RR     5.08      4.66      8.17      6.01
                     RRl     3.30      4.15      4.17      4.45
                     RRu     7.81      5.23     16.01      8.13
                      P       +++       +++       +++       +++
             Between Chi                                  50.77
             Between  df                                      2
             Between  P                                     ***
             Btwn(F)  P                                       *

                             Smoking product
                              any      cigs  cigsonly     Total


                       N        1        10         9        20
                      NS        1         8         6        15


                      Wt     2.71    403.64    250.27    656.62
                 Het Chi     0.00     95.56     51.97    167.26
                 Het  df        0         9         8        19
                 Het  P      N.S.       ***       ***       ***
               Fixed  RR     5.92      5.36      7.66      6.15
                     RRl     1.80      4.86      6.77      5.69
                     RRu    19.47      5.91      8.67      6.63
                      P        ++       +++       +++       +++
              Random  RR     5.92      6.12      5.88      6.01
                     RRl     1.80      3.84      3.69      4.45
                     RRu    19.47      9.74      9.37      8.13
                      P        ++       +++       +++       +++
             Between Chi                                  19.73
             Between  df                                      2
             Between  P                                     ***
             Btwn(F)  P                                    N.S.
  ________________________________________________________________________________________________________________________
                                            International Evidence on Smoking and COPD, Phase 3, Analysis run on 28-SEP-10

                                                   Table 1 - E - 6 - 3

                               IESCOPD - Meta-analysis of amount smoked : key value (2) 20
                            Any COPD, cigarettes (or any product if cigarettes not available)
                                                      Most-adjusted
                                     Unexposed group
                          nev any   nev cig  nev+ any  nev+ cig     Total


                       N       17         3                            20
                      NS       12         3                            15


                      Wt   340.39    316.23                        656.62
                 Het Chi    98.66      5.63                        167.26
                 Het  df       16         2                            19
                 Het  P       ***       (*)                           ***
               Fixed  RR     8.28      4.46                          6.15
                     RRl     7.45      3.99                          5.69
                     RRu     9.21      4.98                          6.63
                      P       +++       +++                           +++
              Random  RR     6.83      3.85                          6.01
                     RRl     4.85      2.74                          4.45
                     RRu     9.64      5.41                          8.13
                      P       +++       +++                           +++
             Between Chi                                            62.97
             Between  df                                                1
             Between  P                                               ***
             Btwn(F)  P                                                **

                        Unexposed group (combining nev+ with main levels)
                          nev any   nev cig     Total


                       N       17         3        20
                      NS       12         3        15


                      Wt   340.39    316.23    656.62
                 Het Chi    98.66      5.63    167.26
                 Het  df       16         2        19
                 Het  P       ***       (*)       ***
               Fixed  RR     8.28      4.46      6.15
                     RRl     7.45      3.99      5.69
                     RRu     9.21      4.98      6.63
                      P       +++       +++       +++
              Random  RR     6.83      3.85      6.01
                     RRl     4.85      2.74      4.45
                     RRu     9.64      5.41      8.13
                      P       +++       +++       +++
             Between Chi                        62.97
             Between  df                            1
             Between  P                           ***
             Btwn(F)  P                            **


  ________________________________________________________________________________________________________________________
                                            International Evidence on Smoking and COPD, Phase 3, Analysis run on 28-SEP-10

                                                   Table 1 - E - 6 - 4

                               IESCOPD - Meta-analysis of amount smoked : key value (2) 20
                            Any COPD, cigarettes (or any product if cigarettes not available)
                                                      Least-adjusted


     REF|NRR|X|SEX|AGEL|AGEH|     REGION|BEGYR|PUBYR|STTYP|ONSET|      DISEAS|ADJ|SMOKSTA|   PRODUCT|    UNEXP|LOW| HI|

  ANDER1  12     m   25   74   Am:Canada  1963  1965    CS  Prev     COPD:oth   1 Current       Cigs   Nev any  15  24
   DEAN1  20 x   m   35   99       Eu:UK  1969  1977    CC  Prev    COPD:mort   0 Current MCigs only   Nev any  13  22
   DEAN1  60 x   f   35   99       Eu:UK  1969  1977    CC  Prev    COPD:mort   0 Current MCigs only   Nev any  13  22
   DOLL1   8     m   20   99       Eu:UK  1951  1994    Pr   Inc    COPD:mort   2 Current  Cigs only   Nev any  15  24
   DOLL2   5     f   20   99       Eu:UK  1951  1980    Pr   Inc    COPD:mort   1 Current  Cigs only   Nev any  15  24
  ENSTRO   9     m   30   99      Am:USA  1960  2003    Pr   Inc    COPD:mort   1 Current  Cigs only   Nev any  20  20
  ENSTRO  18     f   30   99      Am:USA  1960  2003    Pr   Inc    COPD:mort   1 Current  Cigs only   Nev any  20  20
  FERRI2  27     m   25   80      Am:USA  1967  1971    CS  Prev     COPD:oth   1 Current       Cigs   Nev any  15  24
  FERRI2  44     f   25   80      Am:USA  1967  1971    CS  Prev     COPD:oth   1 Current       Cigs   Nev any  15  24
  HOZAWA   5     b   45   64      Am:USA  1987  2006    CS  Prev      COPD:LF   0 Current       Cigs  Nev cigs  15  29
  HUHTI1  22 x   m   40   64    Eu:Scand  1961  1965    CS  Prev    CB/EM/Ast   0 Current       Cigs   Nev any  15  24
  HUHTI3   8 x   m   25   69    Eu:Scand  1968  1978    CS  Prev      COPD:LF   0 Current        Any   Nev any  15  24
  JACOBS  20     m   40   84       Multi  1957  1999    Pr   Inc    COPD:mort   2 Current       Cigs  Nev cigs  20  29
     LEE  10 x   m   35   82       Eu:UK  1964  1979    Pr   Inc    COPD:mort   0 Current  Cigs only   Nev any  20  20
     LEE  45     f   35   82       Eu:UK  1964  1979    Pr   Inc    COPD:mort   0 Current  Cigs only   Nev any  20  20
  MUELLE  21 x   m   20   69      Am:USA  1967  1971    CS  Prev      COPD:LF   0 Current       Cigs   Nev any  15  24
    TODD  10 x   m   35   81       Eu:UK  1965  1978    Pr   Inc    COPD:mort   0 Current  Cigs only   Nev any  20  20
  VONHER  11     m   30   99    Eu:Scand  1978  2000    CS  Prev        CB/EM   0 Current       Cigs   Nev any  20  29
  VONHER  18     f   30   99    Eu:Scand  1978  2000    CS  Prev        CB/EM   0 Current       Cigs   Nev any  20  29
     WEN   3     m   35   99   Asia:FarE  1982  2004    Pr   Inc    COPD:mort   1 Current       Cigs  Nev cigs  11  20


  ________________________________________________________________________________________________________________________
                                            International Evidence on Smoking and COPD, Phase 3, Analysis run on 28-SEP-10

                                                   Table 1 - E - 6 - 5

                               IESCOPD - Meta-analysis of amount smoked : key value (2) 20
                            Any COPD, cigarettes (or any product if cigarettes not available)
                                                      Least-adjusted


                        Number Exposed  Non-exposed
 REF    NRR SEX ADJ     Case    Cont    Case    Cont      RR        95.00%CI
 ANDER1 12  m   1          8       -       2       -      5.93 (  1.14-  30.72)
 DEAN1  20  m   0         75     429      47     510      1.90 (  1.29-   2.79)
 DEAN1  60  f   0         21     521     120    1538      0.52 (  0.32-   0.83)
 Subtotal DEAN1                                           1.13 (  0.84-   1.52)
*DOLL1  8   m   2         61       -      12       -     11.20 (  6.06-  20.68)
*DOLL2  5   f   1          4       -       1       -     28.50 (  3.19- 255.00)
*ENSTRO 9   m   1        326       -     103       -      8.30 (  6.62-  10.40)
*ENSTRO 18  f   1        309       -     296       -      9.32 (  7.85-  11.06)
 Subtotal ENSTRO                                          8.93 (  7.79-  10.24)
 FERRI2 27  m   1         55       -      11       -      4.99 (  2.42-  10.31)
 FERRI2 44  f   1         28       -      54       -      2.89 (  1.72-   4.87)
 Subtotal FERRI2                                          3.48 (  2.28-   5.31)
 HOZAWA 5   b   0        822    1020     872    5019      4.64 (  4.13-   5.21)
 HUHTI1 22  m   0         86     105       7     115     13.46 (  5.96-  30.38)
 HUHTI3 8   m   0         14     187       3     236      5.89 (  1.67-  20.80)
*JACOBS 20  m   2         61       -      28       -      4.00 (  2.43-   6.58)
*LEE    10  m   0          7     364       1     347      6.67 (  0.83-  53.96)
*LEE    45  f   0          0     212       5    1694      0.72~(  0.04-  13.06)
 Subtotal LEE                                             3.12 (  0.57-  16.95)
 MUELLE 21  m   0         14      50       2      57      7.98 (  1.73-  36.83)
*TODD   10  m   0          8     487       1     520      8.54 (  1.07-  68.05)
 VONHER 11  m   0        217     174      63     911     18.03 ( 13.04-  24.93)
 VONHER 18  f   0         40      65     137    2888     12.97 (  8.44-  19.93)
 Subtotal VONHER                                         16.00 ( 12.36-  20.73)
*WEN    3   m   1         62       -      52       -      2.65 (  1.68-   4.18)
Partial Totals          2218    3614    1817   13835
*prospective study                                        ~ With 0.5 adjustment for zero


 REF    NRR SEX ADJ             Ys       Ws       Qs       Ps
 ANDER1 12  m   1              1.78     1.42     0.00       0.03
 DEAN1  20  m   0              0.64    25.71    31.75       0.00
 DEAN1  60  f   0             -0.66    17.09    99.43       0.01
 Subtotal DEAN1                0.12    42.79   131.18
*DOLL1  8   m   2              2.42    10.20     4.50       0.00
*DOLL2  5   f   1              3.35     0.80     2.04       0.00
*ENSTRO 9   m   1              2.12    75.31    10.01       0.00
*ENSTRO 18  f   1              2.23   130.74    30.18       0.00
 Subtotal ENSTRO               2.19   206.05    40.19
 FERRI2 27  m   1              1.61     7.31     0.15       0.00
 FERRI2 44  f   1              1.06    14.19     6.76       0.00
 Subtotal FERRI2               1.25    21.50     6.91
 HOZAWA 5   b   0              1.53   282.25    13.33       0.00
 HUHTI1 22  m   0              2.60     5.79     4.16       0.00
 HUHTI3 8   m   0              1.77     2.41     0.00       0.01
*JACOBS 20  m   2              1.39    15.49     2.07       0.00
*LEE    10  m   0              1.90     0.88     0.02       0.08
*LEE    45  f   0             -0.32     0.46     1.98       0.83
 Subtotal LEE                  1.14     1.34     1.99
 MUELLE 21  m   0              2.08     1.64     0.17       0.01
*TODD   10  m   0              2.15     0.89     0.14       0.04
 VONHER 11  m   0              2.89    36.60    47.60       0.00
 VONHER 18  f   0              2.56    20.82    13.70       0.00
 Subtotal VONHER               2.77    57.42    61.30
*WEN    3   m   1              0.97    18.49    11.17       0.00


  ________________________________________________________________________________________________________________________
                                            International Evidence on Smoking and COPD, Phase 3, Analysis run on 28-SEP-10

                                                   Table 1 - E - 6 - 5

                               IESCOPD - Meta-analysis of amount smoked : key value (2) 20
                            Any COPD, cigarettes (or any product if cigarettes not available)
                                                      Least-adjusted


                       N       20
                      NS       15


                      Wt   668.48
                 Het Chi   279.17
                 Het  df       19
                 Het  P       ***
               Fixed  RR     5.76
                     RRl     5.34
                     RRu     6.22
                      P       +++
              Random  RR     5.41
                     RRl     3.73
                     RRu     7.84
                      P       +++
               Asymm  P      N.S.


  ________________________________________________________________________________________________________________________
                                            International Evidence on Smoking and COPD, Phase 3, Analysis run on 28-SEP-10

                                                   Table 1 - E - 6 - 6

                               IESCOPD - Meta-analysis of amount smoked : key value (2) 20
                            Any COPD, cigarettes (or any product if cigarettes not available)
                                                      Least-adjusted


                       N       20
                      NS       15


                      Wt   668.48
                 Het Chi   279.17
                 Het  df       19
                 Het  P       ***
               Fixed  RR     5.76
                     RRl     5.34
                     RRu     6.22
                      P       +++
              Random  RR     5.41
                     RRl     3.73
                     RRu     7.84
                      P       +++
               Asymm  P      N.S.

                                   Sex
                             both      male    female     Total


                       N        1        13         6        20
                      NS        1        13         6        20


                      Wt   282.25    202.13    184.10    668.48
                 Het Chi     0.00    106.58    149.51    279.17
                 Het  df        0        12         5        19
                 Het  P      N.S.       ***       ***       ***
               Fixed  RR     4.64      6.76      6.75      5.76
                     RRl     4.13      5.89      5.84      5.34
                     RRu     5.21      7.76      7.80      6.22
                      P       +++       +++       +++       +++
              Random  RR     4.64      6.31      4.06      5.41
                     RRl     4.13      3.86      1.29      3.73
                     RRu     5.21     10.32     12.79      7.84
                      P       +++       +++         +       +++
             Between Chi                                  23.07
             Between  df                                      2
             Between  P                                     ***
             Btwn(F)  P                                    N.S.

                                        Continent
                            NAmer    Europe      Asia  oth/mult     Total


                       N        7        11         1         1        20
                      NS        5         8         1         1        15


                      Wt   512.86    121.64     18.49     15.49    668.48
                 Het Chi    59.93    205.30      0.00      0.00    279.17
                 Het  df        6        10         0         0        19
                 Het  P       ***       ***      N.S.      N.S.       ***
               Fixed  RR     5.98      5.83      2.65      4.00      5.76
                     RRl     5.48      4.88      1.68      2.43      5.34
                     RRu     6.52      6.96      4.18      6.58      6.22
                      P       +++       +++       +++       +++       +++
              Random  RR     5.88      5.93      2.65      4.00      5.41
                     RRl     4.02      2.36      1.68      2.43      3.73
                     RRu     8.62     14.92      4.18      6.58      7.84
                      P       +++       +++       +++       +++       +++
             Between Chi                                            13.93
             Between  df                                                3
             Between  P                                                **
             Btwn(F)  P                                              N.S.


  ________________________________________________________________________________________________________________________
                                            International Evidence on Smoking and COPD, Phase 3, Analysis run on 28-SEP-10

                                                   Table 1 - E - 6 - 6

                               IESCOPD - Meta-analysis of amount smoked : key value (2) 20
                            Any COPD, cigarettes (or any product if cigarettes not available)
                                                      Least-adjusted
                               Study type
                               CC        Pr        CS     Total


                       N        2         9         9        20
                      NS        1         7         7        15


                      Wt    42.79    253.26    372.43    668.48
                 Het Chi    17.37     38.19     85.71    279.17
                 Het  df        1         8         8        19
                 Het  P       ***       ***       ***       ***
               Fixed  RR     1.13      7.84      5.64      5.76
                     RRl     0.84      6.93      5.10      5.34
                     RRu     1.52      8.87      6.24      6.22
                      P      N.S.       +++       +++       +++
              Random  RR     1.00      6.41      7.42      5.41
                     RRl     0.28      4.31      4.33      3.73
                     RRu     3.57      9.54     12.74      7.84
                      P      N.S.       +++       +++       +++
             Between Chi                                 137.90
             Between  df                                      2
             Between  P                                     ***
             Btwn(F)  P                                      **

                          COPD subtype
                             mort        LF     other     Total


                       N       11         3         6        20
                      NS        8         3         4        15


                      Wt   296.05    286.30     86.12    668.48
                 Het Chi   193.06      0.61     40.10    279.17
                 Het  df       10         2         5        19
                 Het  P       ***      N.S.       ***       ***
               Fixed  RR     5.92      4.66     10.63      5.76
                     RRl     5.29      4.15      8.61      5.34
                     RRu     6.64      5.23     13.13      6.22
                      P       +++       +++       +++       +++
              Random  RR     4.16      4.66      8.34      5.41
                     RRl     2.21      4.15      4.25      3.73
                     RRu     7.85      5.23     16.36      7.84
                      P       +++       +++       +++       +++
             Between Chi                                  45.39
             Between  df                                      2
             Between  P                                     ***
             Btwn(F)  P                                    N.S.

                             Smoking product
                              any      cigs  cigsonly     Total


                       N        1        10         9        20
                      NS        1         8         6        15


                      Wt     2.41    403.99    262.07    668.48
                 Het Chi     0.00     97.16    177.04    279.17
                 Het  df        0         9         8        19
                 Het  P      N.S.       ***       ***       ***
               Fixed  RR     5.89      5.38      6.42      5.76
                     RRl     1.67      4.88      5.68      5.34
                     RRu    20.80      5.93      7.24      6.22
                      P        ++       +++       +++       +++
              Random  RR     5.89      6.25      4.51      5.41
                     RRl     1.67      3.92      2.10      3.73
                     RRu    20.80      9.98      9.69      7.84
                      P        ++       +++       +++       +++
             Between Chi                                   4.96
             Between  df                                      2
             Between  P                                     (*)
             Btwn(F)  P                                    N.S.
  ________________________________________________________________________________________________________________________
                                            International Evidence on Smoking and COPD, Phase 3, Analysis run on 28-SEP-10

                                                   Table 1 - E - 6 - 6

                               IESCOPD - Meta-analysis of amount smoked : key value (2) 20
                            Any COPD, cigarettes (or any product if cigarettes not available)
                                                      Least-adjusted
                                     Unexposed group
                          nev any   nev cig  nev+ any  nev+ cig     Total


                       N       17         3                            20
                      NS       12         3                            15


                      Wt   352.25    316.23                        668.48
                 Het Chi   233.80      5.63                        279.17
                 Het  df       16         2                            19
                 Het  P       ***       (*)                           ***
               Fixed  RR     7.26      4.46                          5.76
                     RRl     6.54      3.99                          5.34
                     RRu     8.06      4.98                          6.22
                      P       +++       +++                           +++
              Random  RR     5.97      3.85                          5.41
                     RRl     3.65      2.74                          3.73
                     RRu     9.75      5.41                          7.84
                      P       +++       +++                           +++
             Between Chi                                            39.74
             Between  df                                                1
             Between  P                                               ***
             Btwn(F)  P                                              N.S.

                        Unexposed group (combining nev+ with main levels)
                          nev any   nev cig     Total


                       N       17         3        20
                      NS       12         3        15


                      Wt   352.25    316.23    668.48
                 Het Chi   233.80      5.63    279.17
                 Het  df       16         2        19
                 Het  P       ***       (*)       ***
               Fixed  RR     7.26      4.46      5.76
                     RRl     6.54      3.99      5.34
                     RRu     8.06      4.98      6.22
                      P       +++       +++       +++
              Random  RR     5.97      3.85      5.41
                     RRl     3.65      2.74      3.73
                     RRu     9.75      5.41      7.84
                      P       +++       +++       +++
             Between Chi                        39.74
             Between  df                            1
             Between  P                           ***
             Btwn(F)  P                          N.S.


  ________________________________________________________________________________________________________________________
                                            International Evidence on Smoking and COPD, Phase 3, Analysis run on 28-SEP-10

                                                   Table 1 - E - 6 - 7

                               IESCOPD - Meta-analysis of amount smoked : key value (2) 20
                            Any COPD, cigarettes (or any product if cigarettes not available)
                                 Excluded studies (and stage at which they were excluded)


1       CLARK COTTON  MEYER REMYJA RUTGER SNYDER SOBRAX     SU TAKEMU  WANG4   WEIR WHICKE ZALACA
2      ALDERS ANDER2 AUERBA   BANG  BECK1  BECK2 BJORNS  BROWN CERVER CHAPMA COATES COLLEG  DEAN2  DEANE DONTA2 DOPICO
       EHRLIC ENRIGH FINKLE FLETCH FOXMAN GOLDBE HAENSZ HARRIS  HAYES HIGGI2 HIGGI3 HIGGI6 HIRAYA HOLLA2 HOLLNA  HOUSE
       HRUBEC HUCHON JENSEN JINDA2  JOSHI JOUSI1   KATO KOTAN1  KUBIK LAMBER LANGE2 LANGHA LAVECC LUNDB2 MAGNUS MANFRE
       MELLST MENEZ1  MEREN MILLER  MILNE MOLLER   NAWA NEJJAR OGILVI  OMORI OSWAL1 OSWAL2 PANDEY  PRATT   REID RIMING
        RYDER SCHWAR  SHARP SHIMUR SOBRAD STJERN SUADIC SUTINE TAGER2 TROISI URRUTI VIEGI1 VIKGRE WAGEN2  WANG2    WIG
       WILHEL WILSO2  WOODS  WOOLF   ZOIA
3      VINEIS
4      ALESSA  AMIGO ANDER3 BEDNAR BROGGE  CHEN3  CHENG  COCCI DEJONG DEMARC DETORR DICKIN EKBERG  FIDAN FORAST FUKUCH
       GEIJER GULSVI HAMMO2 HARDIE HARIKK HEDMAN HIGGI4     HO HUHTI2 ITABAS JAENDI JOHANN KACHEL KARAKA KATANC KHOURY
          KIM  KIRAZ KLAYTO KOJIMA KOTAN2    LAI   LAM1   LAM2   LAM3  LANGE LINDBE   LIU2  MADOR MANNI1 MANNI2 MANNI3
       MARAN1 MARAN2 MARCUS MATHES MENEZ2 MENEZ3 MENEZ4 MENEZ5 MENEZ6 MONTNE NIEPSU NIHLEN   PEAT PELKON PEREZP  PRICE
       RENWIC RICCIO SARGEA SAWICK SHAHAB   SHIN SICHLE  SILVA STERLI  STROM  TAGER   TANG   THUN TRUPIN TSUSHI VESTBO
       VIEGI2 VOLLM1 VOLLM2   WALD WATSON WILSO1 WOJTYN   XIAO     XU ZIELI1 ZIELI2 ZIETKO
5       CHEN1 HAWTHO KULLER   LIU1
6        BEST  CHEN2 CLEMEN FERRI3 GODTFR   KAHN  KAHN2 KRZYZA LEBOWI LINDST LUNDB1 NILSSO   PETO SPEIZE TVERDA  WEISS
       YAMAGU   YUAN
11     DONTA1 FERRI1   LIAW


  ________________________________________________________________________________________________________________________
                                            International Evidence on Smoking and COPD, Phase 3, Analysis run on 28-SEP-10

                                                   Table 1 - E - 6 - 8

                               IESCOPD - Meta-analysis of amount smoked : key value (2) 20
                            Any COPD, cigarettes (or any product if cigarettes not available)
                                             Potentially overlapping studies


     REF| REFGP|PRINC|                     OVERLAP|

  HOZAWA HOZAWA     1         ENRIGH/HOZAWA/HARIKK
  JACOBS JACOBS     1  JACOBS/DONTA1/DONTA2/PELKON
  HUHTI1 HUHTI1     1                HUHTI1/HUHTI2
  ENSTRO HAMMO2     2                HAMMO2/ENSTRO
  FERRI2 FERRIS     1         FERRI1/FERRI2/FERRI3
     WEN    WEN     1                     WEN/LIAW
    TODD   TODD     1                  LAMBER/TODD


  ________________________________________________________________________________________________________________________
                                            International Evidence on Smoking and COPD, Phase 3, Analysis run on 28-SEP-10

                                                    Table 1 - E - 7 -

                               IESCOPD - Meta-analysis of amount smoked : key value (2) 30
                            Any COPD, cigarettes (or any product if cigarettes not available)


This analysis is restricted to results for:
1) Eligible study on database
2) Outcome COPD
3) Current or ever smoking
4) Categorical dose-response data for amount smoked
5) vs never smoking base
6) Key value (scheme 2) = 30
7) Results complete enough for use in meta-analysis

Within each study, results are then selected (in the following order of preference, within each sex) for:
8) SMKSTA  : current, ever
9) UNEXP   : never any, never cigarettes
10) PROD    : cigarettes, cigarettes only, any product
11) For overlapping studies: principal rather than subsidiary studies
and then for single sex results (m, f) in preference to results for both sexes combined (b).

Results adjusted for the most potential confounders are then chosen in Sections -1 to -3
and results adjusted for the least confounders in Sections -4 to -6. (Those least-adjusted results which
actually differ from the most-adjusted are marked 'x' in column X in Section -4)

Section -7 shows excluded studies, together with the stage (as above) at which no qualifying
results were found.

Section -8 lists the potentially overlapping studies which have been included (1=principal, 2=subsidiary),
and any results which would have been included in preference except that they had data not complete enough
for use in meta-analysis. It also lists their significance (yes/no), if known.


  ________________________________________________________________________________________________________________________
                                            International Evidence on Smoking and COPD, Phase 3, Analysis run on 28-SEP-10

                                                   Table 1 - E - 7 - 1

                               IESCOPD - Meta-analysis of amount smoked : key value (2) 30
                            Any COPD, cigarettes (or any product if cigarettes not available)
                                                      Most-adjusted


     REF|NRR|SEX|AGEL|AGEH|     REGION|BEGYR|PUBYR|STTYP|ONSET|      DISEAS|ADJ|SMOKSTA|   PRODUCT|    UNEXP|LOW| HI|

  ENSTRO  10   m   30   99      Am:USA  1960  2003    Pr   Inc    COPD:mort   1 Current  Cigs only   Nev any  21  39
  ENSTRO  19   f   30   99      Am:USA  1960  2003    Pr   Inc    COPD:mort   1 Current  Cigs only   Nev any  21  39
  FERRI2  28   m   25   80      Am:USA  1967  1971    CS  Prev     COPD:oth   1 Current       Cigs   Nev any  25  34
  FERRI2  45   f   25   80      Am:USA  1967  1971    CS  Prev     COPD:oth   1 Current       Cigs   Nev any  25  34
   KAHN2 101   m   31   84      Am:USA  1954  1966    Pr   Inc    COPD:mort   1 Current       Cigs   Nev any  21  39


  ________________________________________________________________________________________________________________________
                                            International Evidence on Smoking and COPD, Phase 3, Analysis run on 28-SEP-10

                                                   Table 1 - E - 7 - 2

                               IESCOPD - Meta-analysis of amount smoked : key value (2) 30
                            Any COPD, cigarettes (or any product if cigarettes not available)
                                                      Most-adjusted


                        Number Exposed  Non-exposed
 REF    NRR SEX ADJ     Case    Cont    Case    Cont      RR        95.00%CI
*ENSTRO 10  m   1        258       -     103       -     11.99 (  9.39-  15.31)
*ENSTRO 19  f   1        106       -     296       -     12.87 ( 10.13-  16.35)
 Subtotal ENSTRO                                         12.43 ( 10.48-  14.75)
 FERRI2 28  m   1         35       -      11       -      4.91 (  2.27-  10.60)
 FERRI2 45  f   1         13       -      54       -      4.00 (  1.91-   8.37)
 Subtotal FERRI2                                          4.41 (  2.59-   7.52)
*KAHN2  101 m   1        106       -      31       -     11.13 (  7.46-  16.61)
Partial Totals           518       0     495       0
*prospective study


 REF    NRR SEX ADJ             Ys       Ws       Qs       Ps
*ENSTRO 10  m   1              2.48    64.30     0.25       0.00
*ENSTRO 19  f   1              2.55    67.05     1.19       0.00
 Subtotal ENSTRO               2.52   131.34     1.44
 FERRI2 28  m   1              1.59     6.47     4.46       0.00
 FERRI2 45  f   1              1.39     7.04     7.54       0.00
 Subtotal FERRI2               1.48    13.51    12.01
*KAHN2  101 m   1              2.41    23.98     0.00       0.00

                       N        5
                      NS        3


                      Wt   168.83
                 Het Chi    13.45
                 Het  df        4
                 Het  P        **
               Fixed  RR    11.26
                     RRl     9.69
                     RRu    13.10
                      P       +++
              Random  RR     9.53
                     RRl     6.90
                     RRu    13.15
                      P       +++
               Asymm  P         *


  ________________________________________________________________________________________________________________________
                                            International Evidence on Smoking and COPD, Phase 3, Analysis run on 28-SEP-10

                                                   Table 1 - E - 7 - 3

                               IESCOPD - Meta-analysis of amount smoked : key value (2) 30
                            Any COPD, cigarettes (or any product if cigarettes not available)
                                                      Most-adjusted


                       N        5
                      NS        3


                      Wt   168.83
                 Het Chi    13.45
                 Het  df        4
                 Het  P        **
               Fixed  RR    11.26
                     RRl     9.69
                     RRu    13.10
                      P       +++
              Random  RR     9.53
                     RRl     6.90
                     RRu    13.15
                      P       +++
               Asymm  P         *

                                   Sex
                             both      male    female     Total


                       N                  3         2         5
                      NS                  3         2         5


                      Wt              94.75     74.09    168.83
                 Het Chi               4.69      8.70     13.45
                 Het  df                  2         1         4
                 Het  P                 (*)        **        **
               Fixed  RR              11.07     11.52     11.26
                     RRl               9.05      9.17      9.69
                     RRu              13.54     14.46     13.10
                      P                 +++       +++       +++
              Random  RR              10.05      7.58      9.53
                     RRl               6.92      2.42      6.90
                     RRu              14.59     23.69     13.15
                      P                 +++       +++       +++
             Between Chi                                   0.07
             Between  df                                      1
             Between  P                                    N.S.
             Btwn(F)  P                                    N.S.

                                        Continent
                            NAmer    Europe      Asia  oth/mult     Total


                       N        5                                       5
                      NS        3                                       3


                      Wt   168.83                                  168.83
                 Het Chi    13.45                                   13.45
                 Het  df        4                                       4
                 Het  P        **                                      **
               Fixed  RR    11.26                                   11.26
                     RRl     9.69                                    9.69
                     RRu    13.10                                   13.10
                      P       +++                                     +++
              Random  RR     9.53                                    9.53
                     RRl     6.90                                    6.90
                     RRu    13.15                                   13.15
                      P       +++                                     +++
             Between Chi
             Between  df
             Between  P                                              N.S.
             Btwn(F)  P                                              N.S.


  ________________________________________________________________________________________________________________________
                                            International Evidence on Smoking and COPD, Phase 3, Analysis run on 28-SEP-10

                                                   Table 1 - E - 7 - 3

                               IESCOPD - Meta-analysis of amount smoked : key value (2) 30
                            Any COPD, cigarettes (or any product if cigarettes not available)
                                                      Most-adjusted
                               Study type
                               CC        Pr        CS     Total


                       N                  3         2         5
                      NS                  2         1         3


                      Wt             155.33     13.51    168.83
                 Het Chi               0.41      0.14     13.45
                 Het  df                  2         1         4
                 Het  P                N.S.      N.S.        **
               Fixed  RR              12.22      4.41     11.26
                     RRl              10.44      2.59      9.69
                     RRu              14.30      7.52     13.10
                      P                 +++       +++       +++
              Random  RR              12.22      4.41      9.53
                     RRl              10.44      2.59      6.90
                     RRu              14.30      7.52     13.15
                      P                 +++       +++       +++
             Between Chi                                  12.90
             Between  df                                      1
             Between  P                                     ***
             Btwn(F)  P                                      **

                          COPD subtype
                             mort        LF     other     Total


                       N        3                   2         5
                      NS        2                   1         3


                      Wt   155.33               13.51    168.83
                 Het Chi     0.41                0.14     13.45
                 Het  df        2                   1         4
                 Het  P      N.S.                N.S.        **
               Fixed  RR    12.22                4.41     11.26
                     RRl    10.44                2.59      9.69
                     RRu    14.30                7.52     13.10
                      P       +++                 +++       +++
              Random  RR    12.22                4.41      9.53
                     RRl    10.44                2.59      6.90
                     RRu    14.30                7.52     13.15
                      P       +++                 +++       +++
             Between Chi                                  12.90
             Between  df                                      1
             Between  P                                     ***
             Btwn(F)  P                                      **

                             Smoking product
                              any      cigs  cigsonly     Total


                       N                  3         2         5
                      NS                  2         1         3


                      Wt              37.49    131.34    168.83
                 Het Chi               7.54      0.16     13.45
                 Het  df                  2         1         4
                 Het  P                   *      N.S.        **
               Fixed  RR               7.97     12.43     11.26
                     RRl               5.79     10.48      9.69
                     RRu              10.98     14.75     13.10
                      P                 +++       +++       +++
              Random  RR               6.40     12.43      9.53
                     RRl               3.16     10.48      6.90
                     RRu              12.93     14.75     13.15
                      P                 +++       +++       +++
             Between Chi                                   5.75
             Between  df                                      1
             Between  P                                       *
             Btwn(F)  P                                    N.S.
  ________________________________________________________________________________________________________________________
                                            International Evidence on Smoking and COPD, Phase 3, Analysis run on 28-SEP-10

                                                   Table 1 - E - 7 - 3

                               IESCOPD - Meta-analysis of amount smoked : key value (2) 30
                            Any COPD, cigarettes (or any product if cigarettes not available)
                                                      Most-adjusted
                                     Unexposed group
                          nev any   nev cig  nev+ any  nev+ cig     Total


                       N        5                                       5
                      NS        3                                       3


                      Wt   168.83                                  168.83
                 Het Chi    13.45                                   13.45
                 Het  df        4                                       4
                 Het  P        **                                      **
               Fixed  RR    11.26                                   11.26
                     RRl     9.69                                    9.69
                     RRu    13.10                                   13.10
                      P       +++                                     +++
              Random  RR     9.53                                    9.53
                     RRl     6.90                                    6.90
                     RRu    13.15                                   13.15
                      P       +++                                     +++
             Between Chi
             Between  df
             Between  P                                              N.S.
             Btwn(F)  P                                              N.S.

                        Unexposed group (combining nev+ with main levels)
                          nev any   nev cig     Total


                       N        5                   5
                      NS        3                   3


                      Wt   168.83              168.83
                 Het Chi    13.45               13.45
                 Het  df        4                   4
                 Het  P        **                  **
               Fixed  RR    11.26               11.26
                     RRl     9.69                9.69
                     RRu    13.10               13.10
                      P       +++                 +++
              Random  RR     9.53                9.53
                     RRl     6.90                6.90
                     RRu    13.15               13.15
                      P       +++                 +++
             Between Chi
             Between  df
             Between  P                          N.S.
             Btwn(F)  P                          N.S.


  ________________________________________________________________________________________________________________________
                                            International Evidence on Smoking and COPD, Phase 3, Analysis run on 28-SEP-10

                                                   Table 1 - E - 7 - 4

                               IESCOPD - Meta-analysis of amount smoked : key value (2) 30
                            Any COPD, cigarettes (or any product if cigarettes not available)
                                                      Least-adjusted


     REF|NRR|X|SEX|AGEL|AGEH|     REGION|BEGYR|PUBYR|STTYP|ONSET|      DISEAS|ADJ|SMOKSTA|   PRODUCT|    UNEXP|LOW| HI|

  ENSTRO  10     m   30   99      Am:USA  1960  2003    Pr   Inc    COPD:mort   1 Current  Cigs only   Nev any  21  39
  ENSTRO  19     f   30   99      Am:USA  1960  2003    Pr   Inc    COPD:mort   1 Current  Cigs only   Nev any  21  39
  FERRI2  28     m   25   80      Am:USA  1967  1971    CS  Prev     COPD:oth   1 Current       Cigs   Nev any  25  34
  FERRI2  45     f   25   80      Am:USA  1967  1971    CS  Prev     COPD:oth   1 Current       Cigs   Nev any  25  34
   KAHN2 101     m   31   84      Am:USA  1954  1966    Pr   Inc    COPD:mort   1 Current       Cigs   Nev any  21  39


  ________________________________________________________________________________________________________________________
                                            International Evidence on Smoking and COPD, Phase 3, Analysis run on 28-SEP-10

                                                   Table 1 - E - 7 - 5

                               IESCOPD - Meta-analysis of amount smoked : key value (2) 30
                            Any COPD, cigarettes (or any product if cigarettes not available)
                                                      Least-adjusted


                        Number Exposed  Non-exposed
 REF    NRR SEX ADJ     Case    Cont    Case    Cont      RR        95.00%CI
*ENSTRO 10  m   1        258       -     103       -     11.99 (  9.39-  15.31)
*ENSTRO 19  f   1        106       -     296       -     12.87 ( 10.13-  16.35)
 Subtotal ENSTRO                                         12.43 ( 10.48-  14.75)
 FERRI2 28  m   1         35       -      11       -      4.91 (  2.27-  10.60)
 FERRI2 45  f   1         13       -      54       -      4.00 (  1.91-   8.37)
 Subtotal FERRI2                                          4.41 (  2.59-   7.52)
*KAHN2  101 m   1        106       -      31       -     11.13 (  7.46-  16.61)
Partial Totals           518       0     495       0
*prospective study


 REF    NRR SEX ADJ             Ys       Ws       Qs       Ps
*ENSTRO 10  m   1              2.48    64.30     0.25       0.00
*ENSTRO 19  f   1              2.55    67.05     1.19       0.00
 Subtotal ENSTRO               2.52   131.34     1.44
 FERRI2 28  m   1              1.59     6.47     4.46       0.00
 FERRI2 45  f   1              1.39     7.04     7.54       0.00
 Subtotal FERRI2               1.48    13.51    12.01
*KAHN2  101 m   1              2.41    23.98     0.00       0.00

                       N        5
                      NS        3


                      Wt   168.83
                 Het Chi    13.45
                 Het  df        4
                 Het  P        **
               Fixed  RR    11.26
                     RRl     9.69
                     RRu    13.10
                      P       +++
              Random  RR     9.53
                     RRl     6.90
                     RRu    13.15
                      P       +++
               Asymm  P         *


  ________________________________________________________________________________________________________________________
                                            International Evidence on Smoking and COPD, Phase 3, Analysis run on 28-SEP-10

                                                   Table 1 - E - 7 - 6

                               IESCOPD - Meta-analysis of amount smoked : key value (2) 30
                            Any COPD, cigarettes (or any product if cigarettes not available)
                                                      Least-adjusted


                       N        5
                      NS        3


                      Wt   168.83
                 Het Chi    13.45
                 Het  df        4
                 Het  P        **
               Fixed  RR    11.26
                     RRl     9.69
                     RRu    13.10
                      P       +++
              Random  RR     9.53
                     RRl     6.90
                     RRu    13.15
                      P       +++
               Asymm  P         *

                                   Sex
                             both      male    female     Total


                       N                  3         2         5
                      NS                  3         2         5


                      Wt              94.75     74.09    168.83
                 Het Chi               4.69      8.70     13.45
                 Het  df                  2         1         4
                 Het  P                 (*)        **        **
               Fixed  RR              11.07     11.52     11.26
                     RRl               9.05      9.17      9.69
                     RRu              13.54     14.46     13.10
                      P                 +++       +++       +++
              Random  RR              10.05      7.58      9.53
                     RRl               6.92      2.42      6.90
                     RRu              14.59     23.69     13.15
                      P                 +++       +++       +++
             Between Chi                                   0.07
             Between  df                                      1
             Between  P                                    N.S.
             Btwn(F)  P                                    N.S.

                                        Continent
                            NAmer    Europe      Asia  oth/mult     Total


                       N        5                                       5
                      NS        3                                       3


                      Wt   168.83                                  168.83
                 Het Chi    13.45                                   13.45
                 Het  df        4                                       4
                 Het  P        **                                      **
               Fixed  RR    11.26                                   11.26
                     RRl     9.69                                    9.69
                     RRu    13.10                                   13.10
                      P       +++                                     +++
              Random  RR     9.53                                    9.53
                     RRl     6.90                                    6.90
                     RRu    13.15                                   13.15
                      P       +++                                     +++
             Between Chi
             Between  df
             Between  P                                              N.S.
             Btwn(F)  P                                              N.S.


  ________________________________________________________________________________________________________________________
                                            International Evidence on Smoking and COPD, Phase 3, Analysis run on 28-SEP-10

                                                   Table 1 - E - 7 - 6

                               IESCOPD - Meta-analysis of amount smoked : key value (2) 30
                            Any COPD, cigarettes (or any product if cigarettes not available)
                                                      Least-adjusted
                               Study type
                               CC        Pr        CS     Total


                       N                  3         2         5
                      NS                  2         1         3


                      Wt             155.33     13.51    168.83
                 Het Chi               0.41      0.14     13.45
                 Het  df                  2         1         4
                 Het  P                N.S.      N.S.        **
               Fixed  RR              12.22      4.41     11.26
                     RRl              10.44      2.59      9.69
                     RRu              14.30      7.52     13.10
                      P                 +++       +++       +++
              Random  RR              12.22      4.41      9.53
                     RRl              10.44      2.59      6.90
                     RRu              14.30      7.52     13.15
                      P                 +++       +++       +++
             Between Chi                                  12.90
             Between  df                                      1
             Between  P                                     ***
             Btwn(F)  P                                      **

                          COPD subtype
                             mort        LF     other     Total


                       N        3                   2         5
                      NS        2                   1         3


                      Wt   155.33               13.51    168.83
                 Het Chi     0.41                0.14     13.45
                 Het  df        2                   1         4
                 Het  P      N.S.                N.S.        **
               Fixed  RR    12.22                4.41     11.26
                     RRl    10.44                2.59      9.69
                     RRu    14.30                7.52     13.10
                      P       +++                 +++       +++
              Random  RR    12.22                4.41      9.53
                     RRl    10.44                2.59      6.90
                     RRu    14.30                7.52     13.15
                      P       +++                 +++       +++
             Between Chi                                  12.90
             Between  df                                      1
             Between  P                                     ***
             Btwn(F)  P                                      **

                             Smoking product
                              any      cigs  cigsonly     Total


                       N                  3         2         5
                      NS                  2         1         3


                      Wt              37.49    131.34    168.83
                 Het Chi               7.54      0.16     13.45
                 Het  df                  2         1         4
                 Het  P                   *      N.S.        **
               Fixed  RR               7.97     12.43     11.26
                     RRl               5.79     10.48      9.69
                     RRu              10.98     14.75     13.10
                      P                 +++       +++       +++
              Random  RR               6.40     12.43      9.53
                     RRl               3.16     10.48      6.90
                     RRu              12.93     14.75     13.15
                      P                 +++       +++       +++
             Between Chi                                   5.75
             Between  df                                      1
             Between  P                                       *
             Btwn(F)  P                                    N.S.
  ________________________________________________________________________________________________________________________
                                            International Evidence on Smoking and COPD, Phase 3, Analysis run on 28-SEP-10

                                                   Table 1 - E - 7 - 6

                               IESCOPD - Meta-analysis of amount smoked : key value (2) 30
                            Any COPD, cigarettes (or any product if cigarettes not available)
                                                      Least-adjusted
                                     Unexposed group
                          nev any   nev cig  nev+ any  nev+ cig     Total


                       N        5                                       5
                      NS        3                                       3


                      Wt   168.83                                  168.83
                 Het Chi    13.45                                   13.45
                 Het  df        4                                       4
                 Het  P        **                                      **
               Fixed  RR    11.26                                   11.26
                     RRl     9.69                                    9.69
                     RRu    13.10                                   13.10
                      P       +++                                     +++
              Random  RR     9.53                                    9.53
                     RRl     6.90                                    6.90
                     RRu    13.15                                   13.15
                      P       +++                                     +++
             Between Chi
             Between  df
             Between  P                                              N.S.
             Btwn(F)  P                                              N.S.

                        Unexposed group (combining nev+ with main levels)
                          nev any   nev cig     Total


                       N        5                   5
                      NS        3                   3


                      Wt   168.83              168.83
                 Het Chi    13.45               13.45
                 Het  df        4                   4
                 Het  P        **                  **
               Fixed  RR    11.26               11.26
                     RRl     9.69                9.69
                     RRu    13.10               13.10
                      P       +++                 +++
              Random  RR     9.53                9.53
                     RRl     6.90                6.90
                     RRu    13.15               13.15
                      P       +++                 +++
             Between Chi
             Between  df
             Between  P                          N.S.
             Btwn(F)  P                          N.S.


  ________________________________________________________________________________________________________________________
                                            International Evidence on Smoking and COPD, Phase 3, Analysis run on 28-SEP-10

                                                   Table 1 - E - 7 - 7

                               IESCOPD - Meta-analysis of amount smoked : key value (2) 30
                            Any COPD, cigarettes (or any product if cigarettes not available)
                                 Excluded studies (and stage at which they were excluded)


1       CLARK COTTON  MEYER REMYJA RUTGER SNYDER SOBRAX     SU TAKEMU  WANG4   WEIR WHICKE ZALACA
2      ALDERS ANDER2 AUERBA   BANG  BECK1  BECK2 BJORNS  BROWN CERVER CHAPMA COATES COLLEG  DEAN2  DEANE DONTA2 DOPICO
       EHRLIC ENRIGH FINKLE FLETCH FOXMAN GOLDBE HAENSZ HARRIS  HAYES HIGGI2 HIGGI3 HIGGI6 HIRAYA HOLLA2 HOLLNA  HOUSE
       HRUBEC HUCHON JENSEN JINDA2  JOSHI JOUSI1   KATO KOTAN1  KUBIK LAMBER LANGE2 LANGHA LAVECC LUNDB2 MAGNUS MANFRE
       MELLST MENEZ1  MEREN MILLER  MILNE MOLLER   NAWA NEJJAR OGILVI  OMORI OSWAL1 OSWAL2 PANDEY  PRATT   REID RIMING
        RYDER SCHWAR  SHARP SHIMUR SOBRAD STJERN SUADIC SUTINE TAGER2 TROISI URRUTI VIEGI1 VIKGRE WAGEN2  WANG2    WIG
       WILHEL WILSO2  WOODS  WOOLF   ZOIA
3      VINEIS
4      ALESSA  AMIGO ANDER3 BEDNAR BROGGE  CHEN3  CHENG  COCCI DEJONG DEMARC DETORR DICKIN EKBERG  FIDAN FORAST FUKUCH
       GEIJER GULSVI HAMMO2 HARDIE HARIKK HEDMAN HIGGI4     HO HUHTI2 ITABAS JAENDI JOHANN KACHEL KARAKA KATANC KHOURY
          KIM  KIRAZ KLAYTO KOJIMA KOTAN2    LAI   LAM1   LAM2   LAM3  LANGE LINDBE   LIU2  MADOR MANNI1 MANNI2 MANNI3
       MARAN1 MARAN2 MARCUS MATHES MENEZ2 MENEZ3 MENEZ4 MENEZ5 MENEZ6 MONTNE NIEPSU NIHLEN   PEAT PELKON PEREZP  PRICE
       RENWIC RICCIO SARGEA SAWICK SHAHAB   SHIN SICHLE  SILVA STERLI  STROM  TAGER   TANG   THUN TRUPIN TSUSHI VESTBO
       VIEGI2 VOLLM1 VOLLM2   WALD WATSON WILSO1 WOJTYN   XIAO     XU ZIELI1 ZIELI2 ZIETKO
5       CHEN1 HAWTHO KULLER   LIU1
6      ANDER1   BEST  CHEN2 CLEMEN  DEAN1  DOLL1  DOLL2 DONTA1 FERRI3 GODTFR HOZAWA HUHTI1 HUHTI3 JACOBS KRZYZA LEBOWI
          LEE   LIAW LINDST LUNDB1 MUELLE NILSSO   PETO SPEIZE   TODD TVERDA VONHER  WEISS    WEN YAMAGU   YUAN
7        KAHN
11     FERRI1


  ________________________________________________________________________________________________________________________
                                            International Evidence on Smoking and COPD, Phase 3, Analysis run on 28-SEP-10

                                                   Table 1 - E - 7 - 8

                               IESCOPD - Meta-analysis of amount smoked : key value (2) 30
                            Any COPD, cigarettes (or any product if cigarettes not available)
                                             Potentially overlapping studies


     REF| REFGP|PRINC|                     OVERLAP|

  ENSTRO HAMMO2     2                HAMMO2/ENSTRO
  FERRI2 FERRIS     1         FERRI1/FERRI2/FERRI3
   KAHN2   KAHN     2                   KAHN/KAHN2

                                    Most-adjusted - insufficient data for meta-analysis
     REF|NRR|SEX|AGEL|AGEH|     REGION|BEGYR|PUBYR|STTYP|ONSET|      DISEAS|ADJ|SMOKSTA|   PRODUCT|    UNEXP|LOW| HI|

    KAHN   5   m   31   99      Am:USA  1954  1966    Pr   Inc    COPD:mort   2 Current       Cigs   Nev any  21  39
          RR|SIG|

       17.45   ?


  ________________________________________________________________________________________________________________________
                                            International Evidence on Smoking and COPD, Phase 3, Analysis run on 28-SEP-10

                                                    Table 1 - E - 8 -

                               IESCOPD - Meta-analysis of amount smoked : key value (2) 40
                            Any COPD, cigarettes (or any product if cigarettes not available)


This analysis is restricted to results for:
1) Eligible study on database
2) Outcome COPD
3) Current or ever smoking
4) Categorical dose-response data for amount smoked
5) vs never smoking base
6) Key value (scheme 2) = 40
7) Results complete enough for use in meta-analysis

Within each study, results are then selected (in the following order of preference, within each sex) for:
8) SMKSTA  : current, ever
9) UNEXP   : never any, never cigarettes
10) PROD    : cigarettes, cigarettes only, any product
11) For overlapping studies: principal rather than subsidiary studies
and then for single sex results (m, f) in preference to results for both sexes combined (b).

Results adjusted for the most potential confounders are then chosen in Sections -1 to -3
and results adjusted for the least confounders in Sections -4 to -6. (Those least-adjusted results which
actually differ from the most-adjusted are marked 'x' in column X in Section -4)

Section -7 shows excluded studies, together with the stage (as above) at which no qualifying
results were found.

Section -8 lists the potentially overlapping studies which have been included (1=principal, 2=subsidiary),
and any results which would have been included in preference except that they had data not complete enough
for use in meta-analysis. It also lists their significance (yes/no), if known.


  ________________________________________________________________________________________________________________________
                                            International Evidence on Smoking and COPD, Phase 3, Analysis run on 28-SEP-10

                                                   Table 1 - E - 8 - 1

                               IESCOPD - Meta-analysis of amount smoked : key value (2) 40
                            Any COPD, cigarettes (or any product if cigarettes not available)
                                                      Most-adjusted


     REF|NRR|SEX|AGEL|AGEH|     REGION|BEGYR|PUBYR|STTYP|ONSET|      DISEAS|ADJ|SMOKSTA|   PRODUCT|    UNEXP|LOW| HI|

  ENSTRO  11   m   30   99      Am:USA  1960  2003    Pr   Inc    COPD:mort   1 Current  Cigs only   Nev any  40  80
  ENSTRO  20   f   30   99      Am:USA  1960  2003    Pr   Inc    COPD:mort   1 Current  Cigs only   Nev any  40  80
  FERRI2  29   m   25   80      Am:USA  1967  1971    CS  Prev     COPD:oth   1 Current       Cigs   Nev any  35  44
  FERRI2  46   f   25   80      Am:USA  1967  1971    CS  Prev     COPD:oth   1 Current       Cigs   Nev any  35  44


  ________________________________________________________________________________________________________________________
                                            International Evidence on Smoking and COPD, Phase 3, Analysis run on 28-SEP-10

                                                   Table 1 - E - 8 - 2

                               IESCOPD - Meta-analysis of amount smoked : key value (2) 40
                            Any COPD, cigarettes (or any product if cigarettes not available)
                                                      Most-adjusted


                        Number Exposed  Non-exposed
 REF    NRR SEX ADJ     Case    Cont    Case    Cont      RR        95.00%CI
*ENSTRO 11  m   1        148       -     103       -     13.54 ( 10.33-  17.75)
*ENSTRO 20  f   1         46       -     296       -     15.33 ( 11.06-  21.23)
 Subtotal ENSTRO                                         14.24 ( 11.57-  17.54)
 FERRI2 29  m   1         34       -      11       -     14.96 (  6.25-  35.81)
 FERRI2 46  f   1          7       -      54       -      3.98 (  1.56-  10.17)
 Subtotal FERRI2                                          8.09 (  4.27-  15.32)
Partial Totals           235       0     464       0
*prospective study


 REF    NRR SEX ADJ             Ys       Ws       Qs       Ps
*ENSTRO 11  m   1              2.61    52.44     0.00       0.00
*ENSTRO 20  f   1              2.73    36.14     0.59       0.00
 Subtotal ENSTRO               2.66    88.57     0.59
 FERRI2 29  m   1              2.71     5.04     0.05       0.00
 FERRI2 46  f   1              1.38     4.37     6.51       0.00
 Subtotal FERRI2               2.09     9.41     6.57

                       N        4
                      NS        2


                      Wt    97.99
                 Het Chi     7.16
                 Het  df        3
                 Het  P       (*)
               Fixed  RR    13.49
                     RRl    11.07
                     RRu    16.44
                      P       +++
              Random  RR    12.38
                     RRl     8.52
                     RRu    17.99
                      P       +++
               Asymm  P      N.S.


  ________________________________________________________________________________________________________________________
                                            International Evidence on Smoking and COPD, Phase 3, Analysis run on 28-SEP-10

                                                   Table 1 - E - 8 - 3

                               IESCOPD - Meta-analysis of amount smoked : key value (2) 40
                            Any COPD, cigarettes (or any product if cigarettes not available)
                                                      Most-adjusted


                       N        4
                      NS        2


                      Wt    97.99
                 Het Chi     7.16
                 Het  df        3
                 Het  P       (*)
               Fixed  RR    13.49
                     RRl    11.07
                     RRu    16.44
                      P       +++
              Random  RR    12.38
                     RRl     8.52
                     RRu    17.99
                      P       +++
               Asymm  P      N.S.

                                   Sex
                             both      male    female     Total


                       N                  2         2         4
                      NS                  2         2         4


                      Wt              57.48     40.51     97.99
                 Het Chi               0.05      7.09      7.16
                 Het  df                  1         1         3
                 Het  P                N.S.        **       (*)
               Fixed  RR              13.66     13.25     13.49
                     RRl              10.55      9.74     11.07
                     RRu              17.69     18.03     16.44
                      P                 +++       +++       +++
              Random  RR              13.66      8.42     12.38
                     RRl              10.55      2.26      8.52
                     RRu              17.69     31.30     17.99
                      P                 +++        ++       +++
             Between Chi                                   0.02
             Between  df                                      1
             Between  P                                    N.S.
             Btwn(F)  P                                    N.S.

                                        Continent
                            NAmer    Europe      Asia  oth/mult     Total


                       N        4                                       4
                      NS        2                                       2


                      Wt    97.99                                   97.99
                 Het Chi     7.16                                    7.16
                 Het  df        3                                       3
                 Het  P       (*)                                     (*)
               Fixed  RR    13.49                                   13.49
                     RRl    11.07                                   11.07
                     RRu    16.44                                   16.44
                      P       +++                                     +++
              Random  RR    12.38                                   12.38
                     RRl     8.52                                    8.52
                     RRu    17.99                                   17.99
                      P       +++                                     +++
             Between Chi
             Between  df
             Between  P                                              N.S.
             Btwn(F)  P                                              N.S.


  ________________________________________________________________________________________________________________________
                                            International Evidence on Smoking and COPD, Phase 3, Analysis run on 28-SEP-10

                                                   Table 1 - E - 8 - 3

                               IESCOPD - Meta-analysis of amount smoked : key value (2) 40
                            Any COPD, cigarettes (or any product if cigarettes not available)
                                                      Most-adjusted
                               Study type
                               CC        Pr        CS     Total


                       N                  2         2         4
                      NS                  1         1         2


                      Wt              88.57      9.41     97.99
                 Het Chi               0.33      4.11      7.16
                 Het  df                  1         1         3
                 Het  P                N.S.         *       (*)
               Fixed  RR              14.24      8.09     13.49
                     RRl              11.57      4.27     11.07
                     RRu              17.54     15.32     16.44
                      P                 +++       +++       +++
              Random  RR              14.24      7.81     12.38
                     RRl              11.57      2.13      8.52
                     RRu              17.54     28.57     17.99
                      P                 +++        ++       +++
             Between Chi                                   2.72
             Between  df                                      1
             Between  P                                     (*)
             Btwn(F)  P                                    N.S.

                          COPD subtype
                             mort        LF     other     Total


                       N        2                   2         4
                      NS        1                   1         2


                      Wt    88.57                9.41     97.99
                 Het Chi     0.33                4.11      7.16
                 Het  df        1                   1         3
                 Het  P      N.S.                   *       (*)
               Fixed  RR    14.24                8.09     13.49
                     RRl    11.57                4.27     11.07
                     RRu    17.54               15.32     16.44
                      P       +++                 +++       +++
              Random  RR    14.24                7.81     12.38
                     RRl    11.57                2.13      8.52
                     RRu    17.54               28.57     17.99
                      P       +++                  ++       +++
             Between Chi                                   2.72
             Between  df                                      1
             Between  P                                     (*)
             Btwn(F)  P                                    N.S.

                             Smoking product
                              any      cigs  cigsonly     Total


                       N                  2         2         4
                      NS                  1         1         2


                      Wt               9.41     88.57     97.99
                 Het Chi               4.11      0.33      7.16
                 Het  df                  1         1         3
                 Het  P                   *      N.S.       (*)
               Fixed  RR               8.09     14.24     13.49
                     RRl               4.27     11.57     11.07
                     RRu              15.32     17.54     16.44
                      P                 +++       +++       +++
              Random  RR               7.81     14.24     12.38
                     RRl               2.13     11.57      8.52
                     RRu              28.57     17.54     17.99
                      P                  ++       +++       +++
             Between Chi                                   2.72
             Between  df                                      1
             Between  P                                     (*)
             Btwn(F)  P                                    N.S.
  ________________________________________________________________________________________________________________________
                                            International Evidence on Smoking and COPD, Phase 3, Analysis run on 28-SEP-10

                                                   Table 1 - E - 8 - 3

                               IESCOPD - Meta-analysis of amount smoked : key value (2) 40
                            Any COPD, cigarettes (or any product if cigarettes not available)
                                                      Most-adjusted
                                     Unexposed group
                          nev any   nev cig  nev+ any  nev+ cig     Total


                       N        4                                       4
                      NS        2                                       2


                      Wt    97.99                                   97.99
                 Het Chi     7.16                                    7.16
                 Het  df        3                                       3
                 Het  P       (*)                                     (*)
               Fixed  RR    13.49                                   13.49
                     RRl    11.07                                   11.07
                     RRu    16.44                                   16.44
                      P       +++                                     +++
              Random  RR    12.38                                   12.38
                     RRl     8.52                                    8.52
                     RRu    17.99                                   17.99
                      P       +++                                     +++
             Between Chi
             Between  df
             Between  P                                              N.S.
             Btwn(F)  P                                              N.S.

                        Unexposed group (combining nev+ with main levels)
                          nev any   nev cig     Total


                       N        4                   4
                      NS        2                   2


                      Wt    97.99               97.99
                 Het Chi     7.16                7.16
                 Het  df        3                   3
                 Het  P       (*)                 (*)
               Fixed  RR    13.49               13.49
                     RRl    11.07               11.07
                     RRu    16.44               16.44
                      P       +++                 +++
              Random  RR    12.38               12.38
                     RRl     8.52                8.52
                     RRu    17.99               17.99
                      P       +++                 +++
             Between Chi
             Between  df
             Between  P                          N.S.
             Btwn(F)  P                          N.S.


  ________________________________________________________________________________________________________________________
                                            International Evidence on Smoking and COPD, Phase 3, Analysis run on 28-SEP-10

                                                   Table 1 - E - 8 - 4

                               IESCOPD - Meta-analysis of amount smoked : key value (2) 40
                            Any COPD, cigarettes (or any product if cigarettes not available)
                                                      Least-adjusted


     REF|NRR|X|SEX|AGEL|AGEH|     REGION|BEGYR|PUBYR|STTYP|ONSET|      DISEAS|ADJ|SMOKSTA|   PRODUCT|    UNEXP|LOW| HI|

  ENSTRO  11     m   30   99      Am:USA  1960  2003    Pr   Inc    COPD:mort   1 Current  Cigs only   Nev any  40  80
  ENSTRO  20     f   30   99      Am:USA  1960  2003    Pr   Inc    COPD:mort   1 Current  Cigs only   Nev any  40  80
  FERRI2  29     m   25   80      Am:USA  1967  1971    CS  Prev     COPD:oth   1 Current       Cigs   Nev any  35  44
  FERRI2  46     f   25   80      Am:USA  1967  1971    CS  Prev     COPD:oth   1 Current       Cigs   Nev any  35  44


  ________________________________________________________________________________________________________________________
                                            International Evidence on Smoking and COPD, Phase 3, Analysis run on 28-SEP-10

                                                   Table 1 - E - 8 - 5

                               IESCOPD - Meta-analysis of amount smoked : key value (2) 40
                            Any COPD, cigarettes (or any product if cigarettes not available)
                                                      Least-adjusted


                        Number Exposed  Non-exposed
 REF    NRR SEX ADJ     Case    Cont    Case    Cont      RR        95.00%CI
*ENSTRO 11  m   1        148       -     103       -     13.54 ( 10.33-  17.75)
*ENSTRO 20  f   1         46       -     296       -     15.33 ( 11.06-  21.23)
 Subtotal ENSTRO                                         14.24 ( 11.57-  17.54)
 FERRI2 29  m   1         34       -      11       -     14.96 (  6.25-  35.81)
 FERRI2 46  f   1          7       -      54       -      3.98 (  1.56-  10.17)
 Subtotal FERRI2                                          8.09 (  4.27-  15.32)
Partial Totals           235       0     464       0
*prospective study


 REF    NRR SEX ADJ             Ys       Ws       Qs       Ps
*ENSTRO 11  m   1              2.61    52.44     0.00       0.00
*ENSTRO 20  f   1              2.73    36.14     0.59       0.00
 Subtotal ENSTRO               2.66    88.57     0.59
 FERRI2 29  m   1              2.71     5.04     0.05       0.00
 FERRI2 46  f   1              1.38     4.37     6.51       0.00
 Subtotal FERRI2               2.09     9.41     6.57

                       N        4
                      NS        2


                      Wt    97.99
                 Het Chi     7.16
                 Het  df        3
                 Het  P       (*)
               Fixed  RR    13.49
                     RRl    11.07
                     RRu    16.44
                      P       +++
              Random  RR    12.38
                     RRl     8.52
                     RRu    17.99
                      P       +++
               Asymm  P      N.S.


  ________________________________________________________________________________________________________________________
                                            International Evidence on Smoking and COPD, Phase 3, Analysis run on 28-SEP-10

                                                   Table 1 - E - 8 - 6

                               IESCOPD - Meta-analysis of amount smoked : key value (2) 40
                            Any COPD, cigarettes (or any product if cigarettes not available)
                                                      Least-adjusted


                       N        4
                      NS        2


                      Wt    97.99
                 Het Chi     7.16
                 Het  df        3
                 Het  P       (*)
               Fixed  RR    13.49
                     RRl    11.07
                     RRu    16.44
                      P       +++
              Random  RR    12.38
                     RRl     8.52
                     RRu    17.99
                      P       +++
               Asymm  P      N.S.

                                   Sex
                             both      male    female     Total


                       N                  2         2         4
                      NS                  2         2         4


                      Wt              57.48     40.51     97.99
                 Het Chi               0.05      7.09      7.16
                 Het  df                  1         1         3
                 Het  P                N.S.        **       (*)
               Fixed  RR              13.66     13.25     13.49
                     RRl              10.55      9.74     11.07
                     RRu              17.69     18.03     16.44
                      P                 +++       +++       +++
              Random  RR              13.66      8.42     12.38
                     RRl              10.55      2.26      8.52
                     RRu              17.69     31.30     17.99
                      P                 +++        ++       +++
             Between Chi                                   0.02
             Between  df                                      1
             Between  P                                    N.S.
             Btwn(F)  P                                    N.S.

                                        Continent
                            NAmer    Europe      Asia  oth/mult     Total


                       N        4                                       4
                      NS        2                                       2


                      Wt    97.99                                   97.99
                 Het Chi     7.16                                    7.16
                 Het  df        3                                       3
                 Het  P       (*)                                     (*)
               Fixed  RR    13.49                                   13.49
                     RRl    11.07                                   11.07
                     RRu    16.44                                   16.44
                      P       +++                                     +++
              Random  RR    12.38                                   12.38
                     RRl     8.52                                    8.52
                     RRu    17.99                                   17.99
                      P       +++                                     +++
             Between Chi
             Between  df
             Between  P                                              N.S.
             Btwn(F)  P                                              N.S.


  ________________________________________________________________________________________________________________________
                                            International Evidence on Smoking and COPD, Phase 3, Analysis run on 28-SEP-10

                                                   Table 1 - E - 8 - 6

                               IESCOPD - Meta-analysis of amount smoked : key value (2) 40
                            Any COPD, cigarettes (or any product if cigarettes not available)
                                                      Least-adjusted
                               Study type
                               CC        Pr        CS     Total


                       N                  2         2         4
                      NS                  1         1         2


                      Wt              88.57      9.41     97.99
                 Het Chi               0.33      4.11      7.16
                 Het  df                  1         1         3
                 Het  P                N.S.         *       (*)
               Fixed  RR              14.24      8.09     13.49
                     RRl              11.57      4.27     11.07
                     RRu              17.54     15.32     16.44
                      P                 +++       +++       +++
              Random  RR              14.24      7.81     12.38
                     RRl              11.57      2.13      8.52
                     RRu              17.54     28.57     17.99
                      P                 +++        ++       +++
             Between Chi                                   2.72
             Between  df                                      1
             Between  P                                     (*)
             Btwn(F)  P                                    N.S.

                          COPD subtype
                             mort        LF     other     Total


                       N        2                   2         4
                      NS        1                   1         2


                      Wt    88.57                9.41     97.99
                 Het Chi     0.33                4.11      7.16
                 Het  df        1                   1         3
                 Het  P      N.S.                   *       (*)
               Fixed  RR    14.24                8.09     13.49
[truncated: 1,185,661 more chars]
