# Supplementary material for: Systematic review with meta-analysis of the epidemiological evidence relating smoking to COPD, chronic bronchitis and emphysema
Source: BMC Pulm Med. 2011 Jun 14;11:36. doi: 10.1186/1471-2466-11-36 (PMC3128042; doi:10.1186/1471-2466-11-36)
Supplement: Additional file 8 — MetaDoseCB. .RTF file giving the full results of the meta-analyses for the dose-related smoking variables for CB. [file 1471-2466-11-36-S8.RTF]

Systematic review with meta-analysis of the epidemiological evidence relating smoking to COPD, chronic bronchitis and emphysema

Barbara A Forey, Alison J Thornton and Peter N Lee

Additional file 8 : MetaDoseCB

See Additional file 10 (Intro sheet) for list of tables and page numbers


                                                    Table 2 - E - 1 -

                                IESCOPD - Meta-analysis of amount smoked : key value (1) 5
                             Any CB, cigarettes (or any product if cigarettes not available)


This analysis is restricted to results for:
1) Eligible study on database
2) Outcome CB
3) Current or ever smoking
4) Categorical dose-response data for amount smoked
5) vs never smoking base
6) Key value (scheme 1) = 5
7) Results complete enough for use in meta-analysis

Within each study, results are then selected (in the following order of preference, within each sex) for:
8) SMKSTA  : current, ever
9) UNEXP   : never any, never cigarettes
10) PROD    : cigarettes, cigarettes only, any product
11) For overlapping studies: principal rather than subsidiary studies
and then for single sex results (m, f) in preference to results for both sexes combined (b).

Results adjusted for the most potential confounders are then chosen in Sections -1 to -3
and results adjusted for the least confounders in Sections -4 to -6. (Those least-adjusted results which
actually differ from the most-adjusted are marked 'x' in column X in Section -4)

Section -7 shows excluded studies, together with the stage (as above) at which no qualifying
results were found.

Section -8 lists the potentially overlapping studies which have been included (1=principal, 2=subsidiary),
and any results which would have been included in preference except that they had data not complete enough
for use in meta-analysis. It also lists their significance (yes/no), if known.


  ________________________________________________________________________________________________________________________
                                            International Evidence on Smoking and COPD, Phase 3, Analysis run on 28-SEP-10

                                                   Table 2 - E - 1 - 1

                                IESCOPD - Meta-analysis of amount smoked : key value (1) 5
                             Any CB, cigarettes (or any product if cigarettes not available)
                                                      Most-adjusted


     REF|NRR|SEX|AGEL|AGEH|     REGION|BEGYR|PUBYR|STTYP|ONSET|      DISEAS|ADJ|SMOKSTA|   PRODUCT|    UNEXP|LOW| HI|

  ALDERS  11   m   35   74       Eu:UK  1977  1985    CC  Prev CB:diagnosed   1    Ever MCigs only   Nev any   1  17
  ALDERS  16   f   35   74       Eu:UK  1977  1985    CC  Prev CB:diagnosed   1    Ever MCigs only   Nev any   1  17
  ANDER1  40   m   25   74   Am:Canada  1963  1965    CS  Prev  CB:symptoms   1 Current       Cigs   Nev any   1  14
  ANDER1  45   f   25   74   Am:Canada  1963  1965    CS  Prev  CB:symptoms   1 Current       Cigs   Nev any   1  14
    BEST   4   m   30   97   Am:Canada  1955  1967    Pr   Inc CB:mortality   1 Current  Cigs only   Nev any   1   9
   BROWN   9   m   60   69       Eu:UK  1956  1957    CS  Prev CB:diagnosed   0 Current  Cigs only   Nev any   1   9
  CHAPMA  20   m   15   99      Am:USA  1976  1985    CS  Prev  CB:symptoms   1 Current       Cigs  Nev cigs   1  19
  CHAPMA  28   f   15   99      Am:USA  1976  1985    CS  Prev  CB:symptoms   1 Current       Cigs  Nev cigs   1  19
  COATES   9   b   40   64      Am:USA  1962  1965    CS  Prev  CB:symptoms   0 Current       Cigs  Nev cigs   1  14
   DEAN2   9   m   37   67       Eu:UK  1972  1978    CS  Prev  CB:symptoms   1 Current MCigs only   Nev any   1   7
   DEAN2  23   f   37   67       Eu:UK  1972  1978    CS  Prev  CB:symptoms   1 Current MCigs only   Nev any   1   7
   DOLL1  16   m   20   99       Eu:UK  1951  1994    Pr   Inc CB:mortality   1 Current  Cigs only   Nev any   1  14
  DONTA2   4   m   25   84 Eu:SE/Balkn  1960  1984    Pr   Inc CB:diagnosed   0 Current       Cigs  Nev cigs   1   9
  EHRLIC  16   m   15   99      Africa  1998  2004    CS  Prev  CB:symptoms   6 Current       Cigs   Nev any   1  14
  EHRLIC  22   f   15   99      Africa  1998  2004    CS  Prev  CB:symptoms   6 Current       Cigs   Nev any   1  14
  FERRI1 141   m   25   74      Am:USA  1961  1971    CS  Prev  CB:symptoms   1 Current       Cigs   Nev any   1  10
  FERRI1 100   f   25   74      Am:USA  1961  1971    CS  Prev  CB:symptoms   1 Current       Cigs   Nev any   1  10
  HAENSZ  48   m   35   74    Eu:Scand  1964  1972    CS  Prev  CB:symptoms   1 Current       Cigs   Nev any   1  19
  HAENSZ  74   f   35   74    Eu:Scand  1964  1972    CS  Prev  CB:symptoms   1 Current       Cigs   Nev any   1   9
  HARRIS   4   m   15   60      Africa     *  1993    CS  Prev  CB:symptoms   0 Current        Any   Nev any   1  14
  HIGGI2   4   m   25   74       Eu:UK  1956  1957    CS  Prev  CB:symptoms   0 Current       Cigs  Nev cigs   1  14
  HIGGI2  16   f   25   74       Eu:UK  1956  1957    CS  Prev  CB:symptoms   1 Current       Cigs  Nev cigs   1  14
  HIGGI3   4   m   55   64       Eu:UK  1956  1958    CS  Prev  CB:symptoms   0 Current        Any   Nev any   1  14
  HIGGI6   5   m   25   64      Am:USA  1962  1977    CS  Prev  CB:symptoms   1 Current       Cigs   Nev any   1  19
  HIGGI6  26   f   25   64      Am:USA  1962  1977    CS  Prev  CB:symptoms   1 Current       Cigs   Nev any   1  19
  HOLLA2   4   m   40   59      Am:USA  1962  1965    CS  Prev  CB:symptoms   0 Current        Any   Nev any   1  14
  HOLLNA   4   m   40   40    Eu:Scand  1976  1983    CS  Prev  CB:symptoms   0 Current        Any   Nev any   1  14
  HOLLNA  12   f   40   40    Eu:Scand  1976  1983    CS  Prev  CB:symptoms   0 Current        Any   Nev any   1  14
  HUHTI1  50   m   40   64    Eu:Scand  1961  1965    CS  Prev  CB:symptoms   1 Current       Cigs   Nev any   1  14
  HUHTI1 107   f   40   64    Eu:Scand  1961  1965    CS  Prev  CB:symptoms   1 Current       Cigs   Nev any   1  14
  HUHTI3  28   m   25   69    Eu:Scand  1968  1978    CS  Prev  CB:symptoms   1 Current        Any   Nev any   1  14
   KAHN2   3   m   31   84      Am:USA  1954  1966    Pr   Inc CB:mortality   1 Current       Cigs   Nev any   1   9
  LAMBER  12   m   35   69       Eu:UK  1965  1970    CS  Prev  CB:symptoms   1 Current       Cigs  Nev cigs   1  19
  LAMBER  58   f   35   69       Eu:UK  1965  1970    CS  Prev  CB:symptoms   1 Current       Cigs   Nev any   1  19
  LAVECC  19   b   15   99     Eu:West  1983  1988    CS  Prev  CB:self-rep   6 Current       Cigs   Nev any   1  14
  LEBOWI  37   b   15   96      Am:USA  1972  1977    CS  Prev CB:diagnosed   0 Current       Cigs  Nev cigs   1  19
  LINDST  25   b   20   69    Eu:Scand     *  2001    CS  Prev  CB:symptoms   5 Current       Cigs  Nev cigs   5  14
  MENEZ1  10   b   40   99 Am:Sth/Cent  1990  1995    CS  Prev  CB:symptoms   8 Current       Cigs  Nev cigs   1  19
   MEREN   3   b   15   64     Eu:East  1995  2001    CS  Prev  CB:symptoms   4 Current       Cigs  Nev cigs   5  14
   MILNE   4   m   62   90       Eu:UK  1968  1972    CS  Prev  CB:symptoms   0 Current       Cigs  Nev cigs   1  14
   MILNE  10   f   62   90       Eu:UK  1968  1972    CS  Prev  CB:symptoms   0 Current       Cigs  Nev cigs   1  14
  MUELLE  71   f   20   69      Am:USA  1967  1971    CS  Prev  CB:symptoms   1 Current       Cigs   Nev any   1  14
  OGILVI  15   m   30   99       Eu:UK  1955  1957    CC  Prev  CB:symptoms   0 Current       Cigs   Nev any   1   5
  OGILVI  27   f   30   99       Eu:UK  1955  1957    CC  Prev  CB:symptoms   0 Current       Cigs   Nev any   1   5
    REID  12   m   35   74      Am:USA  1962  1966    CS  Prev  CB:symptoms   1 Current       Cigs  Nev cigs   1  19
    REID  28   f   35   74      Am:USA  1962  1966    CS  Prev  CB:symptoms   1 Current       Cigs  Nev cigs   1  19
  TROISI   4   f   34   69      Am:USA  1980  1995    Pr   Inc CB:diagnosed   1 Current       Cigs  Nev cigs   1  14
  URRUTI   4   b   20   44     Eu:West     *  2005    CS  Prev  CB:symptoms   0 Current       Cigs  Nev cigs   1   9
  WAGEN2   4   b   21   68     Eu:West  2001  2004    CC  Prev  CB:symptoms   0    Ever       Cigs  Nev cigs   1  10
     WEN   8   m   35   99   Asia:FarE  1982  2004    Pr   Inc CB:mortality   1 Current       Cigs  Nev cigs   1  10
  WILHEL   4   m   54   54    Eu:Scand  1967  1969    CS  Prev  CB:symptoms   0 Current        Any   Nev any   1  14
   WOOLF   4   f   25   54   Am:Canada     *  1974    CS  Prev  CB:symptoms   0 Current       Cigs  Nev cigs   1  10
  YAMAGU  10   b   40   99   Asia:FarE  1986  1988    CS  Prev  CB:symptoms   6 Current       Cigs  Nev cigs   1   9


  ________________________________________________________________________________________________________________________
                                            International Evidence on Smoking and COPD, Phase 3, Analysis run on 28-SEP-10

                                                   Table 2 - E - 1 - 2

                                IESCOPD - Meta-analysis of amount smoked : key value (1) 5
                             Any CB, cigarettes (or any product if cigarettes not available)
                                                      Most-adjusted


                        Number Exposed  Non-exposed
 REF    NRR SEX ADJ     Case    Cont    Case    Cont      RR        95.00%CI
 ALDERS 11  m   1         50       -      31       -      2.16 (  1.21-   3.86)
 ALDERS 16  f   1         98       -     111       -      1.93 (  1.35-   2.77)
 Subtotal ALDERS                                          1.99 (  1.47-   2.70)
 ANDER1 40  m   1          7       -       4       -      3.72 (  0.97-  14.24)
 ANDER1 45  f   1          5       -      15       -      1.32 (  0.45-   3.87)
 Subtotal ANDER1                                          1.98 (  0.85-   4.58)
*BEST   4   m   1         17       -       3       -      7.02 (  2.06-  23.94)
 BROWN  9   m   0         36      97      15      76      1.88 (  0.96-   3.69)
 CHAPMA 20  m   1         12       -      35       -      1.81 (  0.89-   3.64)
 CHAPMA 28  f   1         25       -      48       -      4.08 (  2.45-   6.80)
 Subtotal CHAPMA                                          3.08 (  2.04-   4.66)
 COATES 9   b   0         14     252      21     515      1.36 (  0.68-   2.72)
 DEAN2  9   m   1         11       -      39       -      1.97 (  1.00-   3.88)
 DEAN2  23  f   1         17       -      80       -      1.39 (  0.82-   2.35)
 Subtotal DEAN2                                           1.58 (  1.05-   2.40)
*DOLL1  16  m   1         15       -       3       -      6.80 (  1.87-  24.72)
*DONTA2 4   m   0         18     118       8     127      2.42 (  1.09-   5.36)
 EHRLIC 16  m   6         54       -      33       -      1.50 (  0.80-   3.00)
 EHRLIC 22  f   6         34       -     138       -      2.30 (  1.40-   3.80)
 Subtotal EHRLIC                                          1.97 (  1.32-   2.93)
 FERRI1 141 m   1         16       -      10       -      2.65 (  1.10-   6.38)
 FERRI1 100 f   1         10       -      35       -      1.45 (  0.69-   3.07)
 Subtotal FERRI1                                          1.87 (  1.06-   3.30)
 HAENSZ 48  m   1         83       -      27       -      2.61 (  1.67-   4.09)
 HAENSZ 74  f   1         31       -      97       -      2.43 (  1.59-   3.70)
 Subtotal HAENSZ                                          2.51 (  1.85-   3.42)
 HARRIS 4   m   0          4     116       6     518      2.98 (  0.83-  10.72)
 HIGGI2 4   m   0          7      93       1      27      2.03 (  0.24-  17.25)
 HIGGI2 16  f   1          4       -       6       -      1.48 (  0.30-   7.19)
 Subtotal HIGGI2                                          1.66 (  0.46-   5.93)
 HIGGI3 4   m   0          2      36       0       6      0.89~(  0.04-  20.76)
 HIGGI6 5   m   1         32       -      17       -      2.88 (  1.56-   5.32)
 HIGGI6 26  f   1         16       -      40       -      1.39 (  0.77-   2.52)
 Subtotal HIGGI6                                          1.98 (  1.29-   3.03)
 HOLLA2 4   m   0          9      51       5      84      2.96 (  0.94-   9.34)
 HOLLNA 4   m   0         28      62      10     101      4.56 (  2.07-  10.03)
 HOLLNA 12  f   0         24     101      14     217      3.68 (  1.83-   7.42)
 Subtotal HOLLNA                                          4.05 (  2.40-   6.83)
 HUHTI1 50  m   1         41       -       7       -      9.18 (  3.76-  22.43)
 HUHTI1 107 f   1          8       -      32       -      2.44 (  1.02-   5.81)
 Subtotal HUHTI1                                          4.65 (  2.49-   8.67)
 HUHTI3 28  m   1        112       -      28       -      3.23 (  2.00-   5.24)
*KAHN2  3   m   1          6       -      13       -      2.13 (  0.81-   5.60)
 LAMBER 12  m   1        116       -      17       -      2.73 (  1.62-   4.60)
 LAMBER 58  f   1         68       -      54       -      2.66 (  1.82-   3.90)
 Subtotal LAMBER                                          2.68 (  1.97-   3.65)
 LAVECC 19  b   6        553       -    1630       -      1.84 (  1.65-   2.06)
 LEBOWI 37  b   0         37     521      47    1123      1.70 (  1.09-   2.64)
 LINDST 25  b   5          -       -     494       -      1.69 (  1.42-   2.02)
 MENEZ1 10  b   8         24       -      29       -      2.36 (  1.24-   4.46)
 MEREN  3   b   4          -       -       -       -      2.47 (  1.79-   3.56)
 MILNE  4   m   0         15      39       3      40      5.13 (  1.38-  19.12)
 MILNE  10  f   0          5      32      12     186      2.42 (  0.80-   7.34)
 Subtotal MILNE                                           3.31 (  1.42-   7.72)
 MUELLE 71  f   1          7       -       3       -     11.61 (  2.46-  54.91)
 OGILVI 15  m   0         23       8      11      29      7.58 (  2.62-  21.93)
 OGILVI 27  f   0         27      31      86     160      1.62 (  0.91-   2.89)
 Subtotal OGILVI                                          2.31 (  1.39-   3.83)
 REID   12  m   1         62       -      16       -      5.54 (  3.15-   9.75)
 REID   28  f   1         22       -      37       -      2.23 (  1.27-   3.92)
 Subtotal REID                                            3.51 (  2.36-   5.23)
*TROISI 4   f   1          -       -       -       -      1.50 (  1.13-   2.00)
 URRUTI 4   b   0         12     222      16     707      2.39 (  1.11-   5.13)
 WAGEN2 4   b   0         76    1179     111    2136      1.24 (  0.92-   1.68)
*WEN    8   m   1          -       -       7       -      3.99 (  1.20-  13.30)
 WILHEL 4   m   0          5      89       1      87      4.89 (  0.56-  42.69)
 WOOLF  4   f   0          9      31      10     173      5.02 (  1.89-  13.36)
 YAMAGU 10  b   6          -       -     128       -      2.34 (  1.81-   3.02)
Partial Totals          1907    3078    3644    6312
*prospective study                                        ~ With 0.5 adjustment for zero

  ________________________________________________________________________________________________________________________
                                            International Evidence on Smoking and COPD, Phase 3, Analysis run on 28-SEP-10

                                                   Table 2 - E - 1 - 2

                                IESCOPD - Meta-analysis of amount smoked : key value (1) 5
                             Any CB, cigarettes (or any product if cigarettes not available)
                                                      Most-adjusted


 REF    NRR SEX ADJ             Ys       Ws       Qs       Ps
 ALDERS 11  m   1              0.77    11.42     0.04       0.01
 ALDERS 16  f   1              0.66    29.74     0.08       0.00
 Subtotal ALDERS               0.69    41.16     0.12
 ANDER1 40  m   1              1.31     2.13     0.77       0.06
 ANDER1 45  f   1              0.28     3.32     0.62       0.61
 Subtotal ANDER1               0.68     5.45     1.40
*BEST   4   m   1              1.95     2.55     3.92       0.00
 BROWN  9   m   0              0.63     8.48     0.05       0.07
 CHAPMA 20  m   1              0.59     7.75     0.11       0.10
 CHAPMA 28  f   1              1.41    14.75     7.13       0.00
 Subtotal CHAPMA               1.13    22.49     7.24
 COATES 9   b   0              0.31     8.00     1.29       0.38
 DEAN2  9   m   1              0.68     8.36     0.01       0.05
 DEAN2  23  f   1              0.33    13.86     2.02       0.22
 Subtotal DEAN2                0.46    22.22     2.02
*DOLL1  16  m   1              1.92     2.31     3.35       0.00
*DONTA2 4   m   0              0.88     6.09     0.18       0.03
 EHRLIC 16  m   6              0.41     8.80     0.82       0.23
 EHRLIC 22  f   6              0.83    15.41     0.23       0.00
 Subtotal EHRLIC               0.68    24.21     1.05
 FERRI1 141 m   1              0.97     4.97     0.35       0.03
 FERRI1 100 f   1              0.37     6.90     0.79       0.33
 Subtotal FERRI1               0.62    11.87     1.14
 HAENSZ 48  m   1              0.96    19.15     1.18       0.00
 HAENSZ 74  f   1              0.89    21.54     0.68       0.00
 Subtotal HAENSZ               0.92    40.69     1.86
 HARRIS 4   m   0              1.09     2.34     0.34       0.10
 HIGGI2 4   m   0              0.71     0.84     0.00       0.52
 HIGGI2 16  f   1              0.39     1.52     0.15       0.63
 Subtotal HIGGI2               0.50     2.36     0.15
 HIGGI3 4   m   0             -0.12     0.39     0.26       0.94
 HIGGI6 5   m   1              1.06    10.21     1.23       0.00
 HIGGI6 26  f   1              0.33    10.93     1.59       0.28
 Subtotal HIGGI6               0.68    21.14     2.82
 HOLLA2 4   m   0              1.09     2.92     0.41       0.06
 HOLLNA 4   m   0              1.52     6.18     4.03       0.00
 HOLLNA 12  f   0              1.30     7.84     2.76       0.00
 Subtotal HOLLNA               1.40    14.02     6.78
 HUHTI1 50  m   1              2.22     4.82    10.93       0.00
 HUHTI1 107 f   1              0.89     5.08     0.17       0.04
 Subtotal HUHTI1               1.54     9.89    11.10
 HUHTI3 28  m   1              1.17    16.56     3.53       0.00
*KAHN2  3   m   1              0.76     4.11     0.01       0.13
 LAMBER 12  m   1              1.00    14.11     1.22       0.00
 LAMBER 58  f   1              0.98    26.45     1.90       0.00
 Subtotal LAMBER               0.99    40.56     3.11
 LAVECC 19  b   6              0.61   311.98     3.17       0.00
 LEBOWI 37  b   0              0.53    19.56     0.65       0.02
 LINDST 25  b   5              0.52   123.70     4.27       0.00
 MENEZ1 10  b   8              0.86     9.38     0.21       0.01
 MEREN  3   b   4              0.90    32.51     1.22       0.00
 MILNE  4   m   0              1.63     2.22     1.90       0.01
 MILNE  10  f   0              0.88     3.13     0.09       0.12
 Subtotal MILNE                1.20     5.34     1.99
 MUELLE 71  f   1              2.45     1.59     4.83       0.00
 OGILVI 15  m   0              2.03     3.40     5.88       0.00
 OGILVI 27  f   0              0.48    11.47     0.60       0.10
 Subtotal OGILVI               0.84    14.87     6.48
 REID   12  m   1              1.71    12.04    12.07       0.00
 REID   28  f   1              0.80    12.10     0.10       0.01
 Subtotal REID                 1.26    24.13    12.17
*TROISI 4   f   1              0.41    47.14     4.39       0.01
 URRUTI 4   b   0              0.87     6.59     0.17       0.03
 WAGEN2 4   b   0              0.22    42.58    10.44       0.16
*WEN    8   m   1              1.38     2.66     1.20       0.02
 WILHEL 4   m   0              1.59     0.82     0.63       0.15
 WOOLF  4   f   0              1.61     4.01     3.28       0.00
 YAMAGU 10  b   6              0.85    58.63     1.14       0.00


  ________________________________________________________________________________________________________________________
                                            International Evidence on Smoking and COPD, Phase 3, Analysis run on 28-SEP-10

                                                   Table 2 - E - 1 - 2

                                IESCOPD - Meta-analysis of amount smoked : key value (1) 5
                             Any CB, cigarettes (or any product if cigarettes not available)
                                                      Most-adjusted


                       N       53
                      NS       38


                      Wt  1015.32
                 Het Chi   108.39
                 Het  df       52
                 Het  P       ***
               Fixed  RR     2.04
                     RRl     1.91
                     RRu     2.16
                      P       +++
              Random  RR     2.32
                     RRl     2.07
                     RRu     2.60
                      P       +++
               Asymm  P       ***


  ________________________________________________________________________________________________________________________
                                            International Evidence on Smoking and COPD, Phase 3, Analysis run on 28-SEP-10

                                                   Table 2 - E - 1 - 3

                                IESCOPD - Meta-analysis of amount smoked : key value (1) 5
                             Any CB, cigarettes (or any product if cigarettes not available)
                                                      Most-adjusted


                       N       53
                      NS       38


                      Wt  1015.32
                 Het Chi   108.39
                 Het  df       52
                 Het  P       ***
               Fixed  RR     2.04
                     RRl     1.91
                     RRu     2.16
                      P       +++
              Random  RR     2.32
                     RRl     2.07
                     RRu     2.60
                      P       +++
               Asymm  P       ***

                                   Sex
                             both      male    female     Total


                       N        9        26        18        53
                      NS        9        26        18        53


                      Wt   612.94    165.61    236.78   1015.32
                 Het Chi    15.58     31.94     31.32    108.39
                 Het  df        8        25        17        52
                 Het  P         *      N.S.         *       ***
               Fixed  RR     1.83      2.94      2.07      2.04
                     RRl     1.69      2.53      1.83      1.91
                     RRu     1.98      3.43      2.35      2.16
                      P       +++       +++       +++       +++
              Random  RR     1.84      2.99      2.16      2.32
                     RRl     1.60      2.49      1.79      2.07
                     RRu     2.12      3.59      2.62      2.60
                      P       +++       +++       +++       +++
             Between Chi                                  29.54
             Between  df                                      2
             Between  P                                     ***
             Btwn(F)  P                                     ***

                                        Continent
                            NAmer    Europe      Asia  oth/mult     Total


                       N       18        29         2         4        53
                      NS       13        20         2         3        38


                      Wt   174.98    743.13     61.29     35.93   1015.32
                 Het Chi    42.92     60.28      0.72      1.53    108.39
                 Het  df       17        28         1         3        52
                 Het  P       ***       ***      N.S.      N.S.       ***
               Fixed  RR     2.16      1.98      2.39      2.12      2.04
                     RRl     1.86      1.84      1.86      1.53      1.91
                     RRu     2.50      2.12      3.08      2.94      2.16
                      P       +++       +++       +++       +++       +++
              Random  RR     2.43      2.29      2.39      2.12      2.32
                     RRl     1.87      1.99      1.86      1.53      2.07
                     RRu     3.16      2.65      3.08      2.94      2.60
                      P       +++       +++       +++       +++       +++
             Between Chi                                             2.94
             Between  df                                                3
             Between  P                                              N.S.
             Btwn(F)  P                                              N.S.


  ________________________________________________________________________________________________________________________
                                            International Evidence on Smoking and COPD, Phase 3, Analysis run on 28-SEP-10

                                                   Table 2 - E - 1 - 3

                                IESCOPD - Meta-analysis of amount smoked : key value (1) 5
                             Any CB, cigarettes (or any product if cigarettes not available)
                                                      Most-adjusted
                        National cigarette tobacco type (excluding mixed/unkown)
                          blended  virginia     Total


                       N       29        22        51
                      NS       22        14        36


                      Wt   777.77    176.26    954.04
                 Het Chi    77.56     25.84    105.94
                 Het  df       28        21        50
                 Het  P       ***      N.S.       ***
               Fixed  RR     1.97      2.24      2.01
                     RRl     1.83      1.94      1.89
                     RRu     2.11      2.60      2.15
                      P       +++       +++       +++
              Random  RR     2.32      2.29      2.31
                     RRl     2.00      1.92      2.06
                     RRu     2.70      2.73      2.60
                      P       +++       +++       +++
             Between Chi                         2.54
             Between  df                            1
             Between  P                          N.S.
             Btwn(F)  P                          N.S.

                                        Start year of study
                            <1970   1970-79   1980-89   1990-99     2000+   unknown     Total


                       N       31         9         4         4         1         4        53
                      NS       21         5         4         3         1         4        38


                      Wt   230.14    119.46    420.40     66.09     42.58    136.65   1015.32
                 Het Chi    45.81     16.14      6.85      1.76      0.00      5.84    108.39
                 Het  df       30         8         3         3         0         3        52
                 Het  P         *         *       (*)      N.S.      N.S.      N.S.       ***
               Fixed  RR     2.62      2.19      1.87      2.26      1.24      1.79      2.04
                     RRl     2.31      1.83      1.70      1.77      0.92      1.52      1.91
                     RRu     2.99      2.62      2.06      2.87      1.68      2.12      2.16
                      P       +++       +++       +++       +++      N.S.       +++       +++
              Random  RR     2.67      2.28      1.91      2.26      1.24      2.34      2.32
                     RRl     2.25      1.75      1.56      1.77      0.92      1.44      2.07
                     RRu     3.18      2.97      2.34      2.87      1.68      3.81      2.60
                      P       +++       +++       +++       +++      N.S.       +++       +++
             Between Chi                                                                32.00
             Between  df                                                                    5
             Between  P                                                                   ***
             Btwn(F)  P                                                                    **

                                Publication year
                            <1980   1980-89   1990-99     2000+     Total


                       N       33         9         4         7        53
                      NS       22         6         4         6        38


                      Wt   267.55    454.37     61.16    232.24   1015.32
                 Het Chi    54.06     18.61      6.87     12.85    108.39
                 Het  df       32         8         3         6        52
                 Het  P        **         *       (*)         *       ***
               Fixed  RR     2.45      2.02      1.75      1.74      2.04
                     RRl     2.17      1.84      1.36      1.53      1.91
                     RRu     2.76      2.21      2.25      1.98      2.16
                      P       +++       +++       +++       +++       +++
              Random  RR     2.52      2.38      2.31      1.85      2.32
                     RRl     2.13      1.93      1.32      1.46      2.07
                     RRu     2.98      2.94      4.04      2.34      2.60
                      P       +++       +++        ++       +++       +++
             Between Chi                                            16.01
             Between  df                                                3
             Between  P                                                **
             Btwn(F)  P                                                 *
  ________________________________________________________________________________________________________________________
                                            International Evidence on Smoking and COPD, Phase 3, Analysis run on 28-SEP-10

                                                   Table 2 - E - 1 - 3

                                IESCOPD - Meta-analysis of amount smoked : key value (1) 5
                             Any CB, cigarettes (or any product if cigarettes not available)
                                                      Most-adjusted
                               Study type
                               CC        Pr        CS     Total


                       N        5         6        42        53
                      NS        3         6        29        38


                      Wt    98.62     64.86    851.85   1015.32
                 Het Chi    12.94     12.60     77.53    108.39
                 Het  df        4         5        41        52
                 Het  P         *         *       ***       ***
               Fixed  RR     1.66      1.87      2.10      2.04
                     RRl     1.36      1.47      1.96      1.91
                     RRu     2.02      2.39      2.24      2.16
                      P       +++       +++       +++       +++
              Random  RR     1.94      2.83      2.38      2.32
                     RRl     1.30      1.63      2.11      2.07
                     RRu     2.88      4.93      2.69      2.60
                      P        ++       +++       +++       +++
             Between Chi                                   5.32
             Between  df                                      2
             Between  P                                     (*)
             Btwn(F)  P                                    N.S.

                                    Lowest age in RR
                        <25/unlim     25-39       40+   unknown     Total


                       N       13        27        13                  53
                      NS       11        17        10                  38


                      Wt   589.86    307.59    117.88             1015.32
                 Het Chi    31.27     45.03     17.41              108.39
                 Het  df       12        26        12                  52
                 Het  P        **         *      N.S.                 ***
               Fixed  RR     1.85      2.24      2.57                2.04
                     RRl     1.71      2.00      2.15                1.91
                     RRu     2.00      2.50      3.08                2.16
                      P       +++       +++       +++                 +++
              Random  RR     2.03      2.35      2.76                2.32
                     RRl     1.69      2.00      2.11                2.07
                     RRu     2.44      2.76      3.59                2.60
                      P       +++       +++       +++                 +++
             Between Chi                                            14.69
             Between  df                                                2
             Between  P                                               ***
             Btwn(F)  P                                                 *

                                         Highest age in RR
                              <65     65-74     75-84 85+/unlim   unknown     Total


                       N       14        22         2        15                  53
                      NS       11        14         2        11                  38


                      Wt   102.63    428.51     10.20    473.98             1015.32
                 Het Chi    21.02     48.18      0.04     30.35              108.39
                 Het  df       13        21         1        14                  52
                 Het  P       (*)       ***      N.S.        **                 ***
               Fixed  RR     2.66      1.92      2.30      2.02                2.04
                     RRl     2.19      1.75      1.24      1.84                1.91
                     RRu     3.22      2.12      4.25      2.20                2.16
                      P       +++       +++        ++       +++                 +++
              Random  RR     2.80      2.12      2.30      2.40                2.32
                     RRl     2.13      1.80      1.24      1.95                2.07
                     RRu     3.68      2.51      4.25      2.95                2.60
                      P       +++       +++        ++       +++                 +++
             Between Chi                                                       8.81
             Between  df                                                          3
             Between  P                                                           *
             Btwn(F)  P                                                        N.S.
  ________________________________________________________________________________________________________________________
                                            International Evidence on Smoking and COPD, Phase 3, Analysis run on 28-SEP-10

                                                   Table 2 - E - 1 - 3

                                IESCOPD - Meta-analysis of amount smoked : key value (1) 5
                             Any CB, cigarettes (or any product if cigarettes not available)
                                                      Most-adjusted
                           Study weakness
                              Yes        No     Total


                       N        1        52        53
                      NS        1        37        38


                      Wt     6.09   1009.23   1015.32
                 Het Chi     0.00    108.21    108.39
                 Het  df        0        51        52
                 Het  P      N.S.       ***       ***
               Fixed  RR     2.42      2.03      2.04
                     RRl     1.09      1.91      1.91
                     RRu     5.36      2.16      2.16
                      P         +       +++       +++
              Random  RR     2.42      2.32      2.32
                     RRl     1.09      2.07      2.07
                     RRu     5.36      2.60      2.60
                      P         +       +++       +++
             Between Chi                         0.19
             Between  df                            1
             Between  P                          N.S.
             Btwn(F)  P                          N.S.

                           CB subtype
                             mort     sympt     other     Total


                       N        4        42         7        53
                      NS        4        28         6        38


                      Wt    11.63    569.29    434.41   1015.32
                 Het Chi     3.09     88.24      2.83    108.39
                 Het  df        3        41         6        52
                 Het  P      N.S.       ***      N.S.       ***
               Fixed  RR     4.02      2.19      1.81      2.04
                     RRl     2.26      2.02      1.65      1.91
                     RRu     7.15      2.38      1.99      2.16
                      P       +++       +++       +++       +++
              Random  RR     4.04      2.44      1.81      2.32
                     RRl     2.25      2.12      1.65      2.07
                     RRu     7.24      2.81      1.99      2.60
                      P       +++       +++       +++       +++
             Between Chi                                  14.23
             Between  df                                      2
             Between  P                                     ***
             Btwn(F)  P                                       *

                           Asthma analysis type (CB)
                        inc-irres  excl-all excl-cntr     Total


                       N       47         6                  53
                      NS       32         6                  38


                      Wt   906.57    108.76             1015.32
                 Het Chi    88.68     11.15              108.39
                 Het  df       46         5                  52
                 Het  P       ***         *                 ***
               Fixed  RR     2.10      1.56                2.04
                     RRl     1.97      1.29                1.91
                     RRu     2.24      1.88                2.16
                      P       +++       +++                 +++
              Random  RR     2.39      1.88                2.32
                     RRl     2.13      1.32                2.07
                     RRu     2.68      2.67                2.60
                      P       +++       +++                 +++
             Between Chi                                   8.57
             Between  df                                      1
             Between  P                                      **
             Btwn(F)  P                                       *
  ________________________________________________________________________________________________________________________
                                            International Evidence on Smoking and COPD, Phase 3, Analysis run on 28-SEP-10

                                                   Table 2 - E - 1 - 3

                                IESCOPD - Meta-analysis of amount smoked : key value (1) 5
                             Any CB, cigarettes (or any product if cigarettes not available)
                                                      Most-adjusted
                                   Number of CB cases
                             1-50    51-100   101-200      201+     Total


                       N        6         7         8        32        53
                      NS        5         5         8        20        38


                      Wt     8.56     21.03     56.88    928.85   1015.32
                 Het Chi     1.81      8.08      9.16     83.87    108.39
                 Het  df        5         6         7        31        52
                 Het  P      N.S.      N.S.      N.S.       ***       ***
               Fixed  RR     2.75      3.19      2.16      2.00      2.04
                     RRl     1.41      2.08      1.67      1.88      1.91
                     RRu     5.38      4.89      2.80      2.13      2.16
                      P        ++       +++       +++       +++       +++
              Random  RR     2.75      3.34      2.27      2.26      2.32
                     RRl     1.41      2.01      1.66      1.99      2.07
                     RRu     5.38      5.54      3.11      2.57      2.60
                      P        ++       +++       +++       +++       +++
             Between Chi                                             5.47
             Between  df                                                3
             Between  P                                              N.S.
             Btwn(F)  P                                              N.S.

                         Number of CB cases (excluding unknown)


                       N        6         7         8        31        52
                      NS        5         5         8        19        37


                      Wt     8.56     21.03     56.88    896.34    982.82
                 Het Chi     1.81      8.08      9.16     82.38    107.13
                 Het  df        5         6         7        30        51
                 Het  P      N.S.      N.S.      N.S.       ***       ***
               Fixed  RR     2.75      3.19      2.16      1.99      2.02
                     RRl     1.41      2.08      1.67      1.86      1.90
                     RRu     5.38      4.89      2.80      2.12      2.15
                      P        ++       +++       +++       +++       +++
              Random  RR     2.75      3.34      2.27      2.25      2.32
                     RRl     1.41      2.01      1.66      1.97      2.06
                     RRu     5.38      5.54      3.11      2.57      2.60
                      P        ++       +++       +++       +++       +++
             Between Chi                                             5.70
             Between  df                                                3
             Between  P                                              N.S.
             Btwn(F)  P                                              N.S.

                            Analysis type
                         prevlnce     onset     Total


                       N       47         6        53
                      NS       32         6        38


                      Wt   950.47     64.86   1015.32
                 Het Chi    95.31     12.60    108.39
                 Het  df       46         5        52
                 Het  P       ***         *       ***
               Fixed  RR     2.05      1.87      2.04
                     RRl     1.92      1.47      1.91
                     RRu     2.18      2.39      2.16
                      P       +++       +++       +++
              Random  RR     2.31      2.83      2.32
                     RRl     2.06      1.63      2.07
                     RRu     2.59      4.93      2.60
                      P       +++       +++       +++
             Between Chi                         0.48
             Between  df                            1
             Between  P                          N.S.
             Btwn(F)  P                          N.S.

  ________________________________________________________________________________________________________________________
                                            International Evidence on Smoking and COPD, Phase 3, Analysis run on 28-SEP-10

                                                   Table 2 - E - 1 - 3

                                IESCOPD - Meta-analysis of amount smoked : key value (1) 5
                             Any CB, cigarettes (or any product if cigarettes not available)
                                                      Most-adjusted
                             Smoking product
                              any      cigs  cigsonly     Total


                       N        7        39         7        53
                      NS        6        27         5        38


                      Wt    37.05    901.55     76.72   1015.32
                 Het Chi     1.52     86.58      9.45    108.39
                 Het  df        6        38         6        52
                 Het  P      N.S.       ***      N.S.       ***
               Fixed  RR     3.46      1.99      2.00      2.04
                     RRl     2.51      1.87      1.60      1.91
                     RRu     4.78      2.13      2.51      2.16
                      P       +++       +++       +++       +++
              Random  RR     3.46      2.26      2.11      2.32
                     RRl     2.51      1.99      1.55      2.07
                     RRu     4.78      2.57      2.87      2.60
                      P       +++       +++       +++       +++
             Between Chi                                  10.84
             Between  df                                      2
             Between  P                                      **
             Btwn(F)  P                                     (*)

                                     Unexposed group
                          nev any   nev cig  nev+ any  nev+ cig     Total


                       N       32        21                            53
                      NS       22        17                            39


                      Wt   586.03    429.30                       1015.32
                 Het Chi    56.89     50.81                        108.39
                 Het  df       31        20                            52
                 Het  P        **       ***                           ***
               Fixed  RR     2.08      1.97                          2.04
                     RRl     1.92      1.80                          1.91
                     RRu     2.26      2.17                          2.16
                      P       +++       +++                           +++
              Random  RR     2.41      2.23                          2.32
                     RRl     2.07      1.86                          2.07
                     RRu     2.81      2.68                          2.60
                      P       +++       +++                           +++
             Between Chi                                             0.69
             Between  df                                                1
             Between  P                                              N.S.
             Btwn(F)  P                                              N.S.

                        Unexposed group (combining nev+ with main levels)
                          nev any   nev cig     Total


                       N       32        21        53
                      NS       22        17        39


                      Wt   586.03    429.30   1015.32
                 Het Chi    56.89     50.81    108.39
                 Het  df       31        20        52
                 Het  P        **       ***       ***
               Fixed  RR     2.08      1.97      2.04
                     RRl     1.92      1.80      1.91
                     RRu     2.26      2.17      2.16
                      P       +++       +++       +++
              Random  RR     2.41      2.23      2.32
                     RRl     2.07      1.86      2.07
                     RRu     2.81      2.68      2.60
                      P       +++       +++       +++
             Between Chi                         0.69
             Between  df                            1
             Between  P                          N.S.
             Btwn(F)  P                          N.S.
  ________________________________________________________________________________________________________________________
                                            International Evidence on Smoking and COPD, Phase 3, Analysis run on 28-SEP-10

                                                   Table 2 - E - 1 - 3

                                IESCOPD - Meta-analysis of amount smoked : key value (1) 5
                             Any CB, cigarettes (or any product if cigarettes not available)
                                                      Most-adjusted
                        Number of adjustment variables
                                0         1        2+     Total


                       N       18        28         7        53
                      NS       15        18         6        39


                      Wt   136.87    318.06    560.40   1015.32
                 Het Chi    32.66     57.81      8.29    108.39
                 Het  df       17        27         6        52
                 Het  P         *       ***      N.S.       ***
               Fixed  RR     1.94      2.35      1.90      2.04
                     RRl     1.64      2.11      1.75      1.91
                     RRu     2.30      2.62      2.06      2.16
                      P       +++       +++       +++       +++
              Random  RR     2.38      2.53      1.95      2.32
                     RRl     1.82      2.12      1.73      2.07
                     RRu     3.12      3.02      2.20      2.60
                      P       +++       +++       +++       +++
             Between Chi                                   9.63
             Between  df                                      2
             Between  P                                      **
             Btwn(F)  P                                     (*)


  ________________________________________________________________________________________________________________________
                                            International Evidence on Smoking and COPD, Phase 3, Analysis run on 28-SEP-10

                                                   Table 2 - E - 1 - 4

                                IESCOPD - Meta-analysis of amount smoked : key value (1) 5
                             Any CB, cigarettes (or any product if cigarettes not available)
                                                      Least-adjusted


     REF|NRR|X|SEX|AGEL|AGEH|     REGION|BEGYR|PUBYR|STTYP|ONSET|      DISEAS|ADJ|SMOKSTA|   PRODUCT|    UNEXP|LOW| HI|

  ALDERS  11     m   35   74       Eu:UK  1977  1985    CC  Prev CB:diagnosed   1    Ever MCigs only   Nev any   1  17
  ALDERS  16     f   35   74       Eu:UK  1977  1985    CC  Prev CB:diagnosed   1    Ever MCigs only   Nev any   1  17
  ANDER1  40     m   25   74   Am:Canada  1963  1965    CS  Prev  CB:symptoms   1 Current       Cigs   Nev any   1  14
  ANDER1  45     f   25   74   Am:Canada  1963  1965    CS  Prev  CB:symptoms   1 Current       Cigs   Nev any   1  14
    BEST   4     m   30   97   Am:Canada  1955  1967    Pr   Inc CB:mortality   1 Current  Cigs only   Nev any   1   9
   BROWN   9     m   60   69       Eu:UK  1956  1957    CS  Prev CB:diagnosed   0 Current  Cigs only   Nev any   1   9
  CHAPMA   4 x   m   15   99      Am:USA  1976  1985    CS  Prev  CB:symptoms   0 Current       Cigs  Nev cigs   1  19
  CHAPMA  12 x   f   15   99      Am:USA  1976  1985    CS  Prev  CB:symptoms   0 Current       Cigs  Nev cigs   1  19
  COATES   9     b   40   64      Am:USA  1962  1965    CS  Prev  CB:symptoms   0 Current       Cigs  Nev cigs   1  14
   DEAN2   9     m   37   67       Eu:UK  1972  1978    CS  Prev  CB:symptoms   1 Current MCigs only   Nev any   1   7
   DEAN2  23     f   37   67       Eu:UK  1972  1978    CS  Prev  CB:symptoms   1 Current MCigs only   Nev any   1   7
   DOLL1  16     m   20   99       Eu:UK  1951  1994    Pr   Inc CB:mortality   1 Current  Cigs only   Nev any   1  14
  DONTA2   4     m   25   84 Eu:SE/Balkn  1960  1984    Pr   Inc CB:diagnosed   0 Current       Cigs  Nev cigs   1   9
  EHRLIC   4 x   m   15   99      Africa  1998  2004    CS  Prev  CB:symptoms   0 Current       Cigs   Nev any   1  14
  EHRLIC  10 x   f   15   99      Africa  1998  2004    CS  Prev  CB:symptoms   0 Current       Cigs   Nev any   1  14
  FERRI1 141     m   25   74      Am:USA  1961  1971    CS  Prev  CB:symptoms   1 Current       Cigs   Nev any   1  10
  FERRI1 100     f   25   74      Am:USA  1961  1971    CS  Prev  CB:symptoms   1 Current       Cigs   Nev any   1  10
  HAENSZ  45 x   m   35   74    Eu:Scand  1964  1972    CS  Prev  CB:symptoms   0 Current       Cigs   Nev any   1  19
  HAENSZ  71 x   f   35   74    Eu:Scand  1964  1972    CS  Prev  CB:symptoms   0 Current       Cigs   Nev any   1   9
  HARRIS   4     m   15   60      Africa     *  1993    CS  Prev  CB:symptoms   0 Current        Any   Nev any   1  14
  HIGGI2   4     m   25   74       Eu:UK  1956  1957    CS  Prev  CB:symptoms   0 Current       Cigs  Nev cigs   1  14
  HIGGI2  10 x   f   25   74       Eu:UK  1956  1957    CS  Prev  CB:symptoms   0 Current       Cigs  Nev cigs   1  14
  HIGGI3   4     m   55   64       Eu:UK  1956  1958    CS  Prev  CB:symptoms   0 Current        Any   Nev any   1  14
  HIGGI6   5     m   25   64      Am:USA  1962  1977    CS  Prev  CB:symptoms   1 Current       Cigs   Nev any   1  19
  HIGGI6  26     f   25   64      Am:USA  1962  1977    CS  Prev  CB:symptoms   1 Current       Cigs   Nev any   1  19
  HOLLA2   4     m   40   59      Am:USA  1962  1965    CS  Prev  CB:symptoms   0 Current        Any   Nev any   1  14
  HOLLNA   4     m   40   40    Eu:Scand  1976  1983    CS  Prev  CB:symptoms   0 Current        Any   Nev any   1  14
  HOLLNA  12     f   40   40    Eu:Scand  1976  1983    CS  Prev  CB:symptoms   0 Current        Any   Nev any   1  14
  HUHTI1  45 x   m   40   64    Eu:Scand  1961  1965    CS  Prev  CB:symptoms   0 Current       Cigs   Nev any   1  14
  HUHTI1 104 x   f   40   64    Eu:Scand  1961  1965    CS  Prev  CB:symptoms   0 Current       Cigs   Nev any   1  14
  HUHTI3  23 x   m   25   69    Eu:Scand  1968  1978    CS  Prev  CB:symptoms   0 Current        Any   Nev any   1  14
   KAHN2   3     m   31   84      Am:USA  1954  1966    Pr   Inc CB:mortality   1 Current       Cigs   Nev any   1   9
  LAMBER   7 x   m   35   69       Eu:UK  1965  1970    CS  Prev  CB:symptoms   0 Current       Cigs  Nev cigs   1  19
  LAMBER  55 x   f   35   69       Eu:UK  1965  1970    CS  Prev  CB:symptoms   0 Current       Cigs   Nev any   1  19
  LAVECC  13 x   m   15   99     Eu:West  1983  1988    CS  Prev  CB:self-rep   0 Current       Cigs   Nev any   1  14
  LAVECC  16 x   f   15   99     Eu:West  1983  1988    CS  Prev  CB:self-rep   0 Current       Cigs   Nev any   1  14
  LEBOWI  37     b   15   96      Am:USA  1972  1977    CS  Prev CB:diagnosed   0 Current       Cigs  Nev cigs   1  19
  LINDST  25     b   20   69    Eu:Scand     *  2001    CS  Prev  CB:symptoms   5 Current       Cigs  Nev cigs   5  14
  MENEZ1   7 x   b   40   99 Am:Sth/Cent  1990  1995    CS  Prev  CB:symptoms   0 Current       Cigs  Nev cigs   1  19
   MEREN   3     b   15   64     Eu:East  1995  2001    CS  Prev  CB:symptoms   4 Current       Cigs  Nev cigs   5  14
   MILNE   4     m   62   90       Eu:UK  1968  1972    CS  Prev  CB:symptoms   0 Current       Cigs  Nev cigs   1  14
   MILNE  10     f   62   90       Eu:UK  1968  1972    CS  Prev  CB:symptoms   0 Current       Cigs  Nev cigs   1  14
  MUELLE  66 x   f   20   69      Am:USA  1967  1971    CS  Prev  CB:symptoms   0 Current       Cigs   Nev any   1  14
  OGILVI  15     m   30   99       Eu:UK  1955  1957    CC  Prev  CB:symptoms   0 Current       Cigs   Nev any   1   5
  OGILVI  27     f   30   99       Eu:UK  1955  1957    CC  Prev  CB:symptoms   0 Current       Cigs   Nev any   1   5
    REID   7 x   m   35   74      Am:USA  1962  1966    CS  Prev  CB:symptoms   0 Current       Cigs  Nev cigs   1  19
    REID  23 x   f   35   74      Am:USA  1962  1966    CS  Prev  CB:symptoms   0 Current       Cigs  Nev cigs   1  19
  TROISI   4     f   34   69      Am:USA  1980  1995    Pr   Inc CB:diagnosed   1 Current       Cigs  Nev cigs   1  14
  URRUTI   4     b   20   44     Eu:West     *  2005    CS  Prev  CB:symptoms   0 Current       Cigs  Nev cigs   1   9
  WAGEN2   4     b   21   68     Eu:West  2001  2004    CC  Prev  CB:symptoms   0    Ever       Cigs  Nev cigs   1  10
     WEN   8     m   35   99   Asia:FarE  1982  2004    Pr   Inc CB:mortality   1 Current       Cigs  Nev cigs   1  10
  WILHEL   4     m   54   54    Eu:Scand  1967  1969    CS  Prev  CB:symptoms   0 Current        Any   Nev any   1  14
   WOOLF   4     f   25   54   Am:Canada     *  1974    CS  Prev  CB:symptoms   0 Current       Cigs  Nev cigs   1  10
  YAMAGU  10     b   40   99   Asia:FarE  1986  1988    CS  Prev  CB:symptoms   6 Current       Cigs  Nev cigs   1   9


  ________________________________________________________________________________________________________________________
                                            International Evidence on Smoking and COPD, Phase 3, Analysis run on 28-SEP-10

                                                   Table 2 - E - 1 - 5

                                IESCOPD - Meta-analysis of amount smoked : key value (1) 5
                             Any CB, cigarettes (or any product if cigarettes not available)
                                                      Least-adjusted


                        Number Exposed  Non-exposed
 REF    NRR SEX ADJ     Case    Cont    Case    Cont      RR        95.00%CI
 ALDERS 11  m   1         50       -      31       -      2.16 (  1.21-   3.86)
 ALDERS 16  f   1         98       -     111       -      1.93 (  1.35-   2.77)
 Subtotal ALDERS                                          1.99 (  1.47-   2.70)
 ANDER1 40  m   1          7       -       4       -      3.72 (  0.97-  14.24)
 ANDER1 45  f   1          5       -      15       -      1.32 (  0.45-   3.87)
 Subtotal ANDER1                                          1.98 (  0.85-   4.58)
*BEST   4   m   1         17       -       3       -      7.02 (  2.06-  23.94)
 BROWN  9   m   0         36      97      15      76      1.88 (  0.96-   3.69)
 CHAPMA 4   m   0         12     194      35     989      1.75 (  0.89-   3.43)
 CHAPMA 12  f   0         25     245      48    1857      3.95 (  2.39-   6.52)
 Subtotal CHAPMA                                          2.95 (  1.97-   4.41)
 COATES 9   b   0         14     252      21     515      1.36 (  0.68-   2.72)
 DEAN2  9   m   1         11       -      39       -      1.97 (  1.00-   3.88)
 DEAN2  23  f   1         17       -      80       -      1.39 (  0.82-   2.35)
 Subtotal DEAN2                                           1.58 (  1.05-   2.40)
*DOLL1  16  m   1         15       -       3       -      6.80 (  1.87-  24.72)
*DONTA2 4   m   0         18     118       8     127      2.42 (  1.09-   5.36)
 EHRLIC 4   m   0         54    1686      33    2672      2.59 (  1.67-   4.02)
 EHRLIC 10  f   0         34     591     138    6208      2.59 (  1.76-   3.80)
 Subtotal EHRLIC                                          2.59 (  1.94-   3.46)
 FERRI1 141 m   1         16       -      10       -      2.65 (  1.10-   6.38)
 FERRI1 100 f   1         10       -      35       -      1.45 (  0.69-   3.07)
 Subtotal FERRI1                                          1.87 (  1.06-   3.30)
 HAENSZ 45  m   0         83    3711      27    2742      2.27 (  1.47-   3.52)
 HAENSZ 71  f   0         31    1734      97   11536      2.13 (  1.41-   3.20)
 Subtotal HAENSZ                                          2.19 (  1.63-   2.95)
 HARRIS 4   m   0          4     116       6     518      2.98 (  0.83-  10.72)
 HIGGI2 4   m   0          7      93       1      27      2.03 (  0.24-  17.25)
 HIGGI2 10  f   0          4      73       6     170      1.55 (  0.43-   5.67)
 Subtotal HIGGI2                                          1.67 (  0.55-   5.05)
 HIGGI3 4   m   0          2      36       0       6      0.89~(  0.04-  20.76)
 HIGGI6 5   m   1         32       -      17       -      2.88 (  1.56-   5.32)
 HIGGI6 26  f   1         16       -      40       -      1.39 (  0.77-   2.52)
 Subtotal HIGGI6                                          1.98 (  1.29-   3.03)
 HOLLA2 4   m   0          9      51       5      84      2.96 (  0.94-   9.34)
 HOLLNA 4   m   0         28      62      10     101      4.56 (  2.07-  10.03)
 HOLLNA 12  f   0         24     101      14     217      3.68 (  1.83-   7.42)
 Subtotal HOLLNA                                          4.05 (  2.40-   6.83)
 HUHTI1 45  m   0         41      67       7     115     10.05 (  4.27-  23.67)
 HUHTI1 104 f   0          8      69      32     677      2.45 (  1.09-   5.53)
 Subtotal HUHTI1                                          4.79 (  2.66-   8.64)
 HUHTI3 23  m   0        112     203      28     211      4.16 (  2.63-   6.56)
*KAHN2  3   m   1          6       -      13       -      2.13 (  0.81-   5.60)
 LAMBER 7   m   0        116    1522      17     642      2.88 (  1.72-   4.83)
 LAMBER 55  f   0         68    1377      54    2390      2.19 (  1.52-   3.14)
 Subtotal LAMBER                                          2.39 (  1.78-   3.22)
 LAVECC 13  m   0        441    5070     588   13724      2.03 (  1.79-   2.31)
 LAVECC 16  f   0        112    4395    1042   28818      0.70 (  0.58-   0.86)
 Subtotal LAVECC                                          1.49 (  1.34-   1.65)
 LEBOWI 37  b   0         37     521      47    1123      1.70 (  1.09-   2.64)
 LINDST 25  b   5          -       -     494       -      1.69 (  1.42-   2.02)
 MENEZ1 7   b   0         24     161      29     435      2.24 (  1.26-   3.95)
 MEREN  3   b   4          -       -       -       -      2.47 (  1.79-   3.56)
 MILNE  4   m   0         15      39       3      40      5.13 (  1.38-  19.12)
 MILNE  10  f   0          5      32      12     186      2.42 (  0.80-   7.34)
 Subtotal MILNE                                           3.31 (  1.42-   7.72)
 MUELLE 66  f   0          7      44       3     168      8.91 (  2.21-  35.86)
 OGILVI 15  m   0         23       8      11      29      7.58 (  2.62-  21.93)
 OGILVI 27  f   0         27      31      86     160      1.62 (  0.91-   2.89)
 Subtotal OGILVI                                          2.31 (  1.39-   3.83)
 REID   7   m   0         62    2890      16    4127      5.53 (  3.19-   9.61)
 REID   23  f   0         22    3545      37   10430      1.75 (  1.03-   2.97)
 Subtotal REID                                            3.04 (  2.07-   4.45)
*TROISI 4   f   1          -       -       -       -      1.50 (  1.13-   2.00)
 URRUTI 4   b   0         12     222      16     707      2.39 (  1.11-   5.13)
 WAGEN2 4   b   0         76    1179     111    2136      1.24 (  0.92-   1.68)
*WEN    8   m   1          -       -       7       -      3.99 (  1.20-  13.30)
 WILHEL 4   m   0          5      89       1      87      4.89 (  0.56-  42.69)
 WOOLF  4   f   0          9      31      10     173      5.02 (  1.89-  13.36)
 YAMAGU 10  b   6          -       -     128       -      2.34 (  1.81-   3.02)
Partial Totals          1907   30655    3644   94223
  ________________________________________________________________________________________________________________________
                                            International Evidence on Smoking and COPD, Phase 3, Analysis run on 28-SEP-10

                                                   Table 2 - E - 1 - 5

                                IESCOPD - Meta-analysis of amount smoked : key value (1) 5
                             Any CB, cigarettes (or any product if cigarettes not available)
                                                      Least-adjusted


                        Number Exposed  Non-exposed
 REF    NRR SEX ADJ     Case    Cont    Case    Cont      RR        95.00%CI
*prospective study                                        ~ With 0.5 adjustment for zero


 REF    NRR SEX ADJ             Ys       Ws       Qs       Ps
 ALDERS 11  m   1              0.77    11.42     0.17       0.01
 ALDERS 16  f   1              0.66    29.74     0.00       0.00
 Subtotal ALDERS               0.69    41.16     0.17
 ANDER1 40  m   1              1.31     2.13     0.94       0.06
 ANDER1 45  f   1              0.28     3.32     0.45       0.61
 Subtotal ANDER1               0.68     5.45     1.40
*BEST   4   m   1              1.95     2.55     4.32       0.00
 BROWN  9   m   0              0.63     8.48     0.00       0.07
 CHAPMA 4   m   0              0.56     8.47     0.07       0.10
 CHAPMA 12  f   0              1.37    15.28     8.04       0.00
 Subtotal CHAPMA               1.08    23.75     8.11
 COATES 9   b   0              0.31     8.00     0.92       0.38
 DEAN2  9   m   1              0.68     8.36     0.01       0.05
 DEAN2  23  f   1              0.33    13.86     1.40       0.22
 Subtotal DEAN2                0.46    22.22     1.41
*DOLL1  16  m   1              1.92     2.31     3.71       0.00
*DONTA2 4   m   0              0.88     6.09     0.34       0.03
 EHRLIC 4   m   0              0.95    20.08     1.87       0.00
 EHRLIC 10  f   0              0.95    25.97     2.39       0.00
 Subtotal EHRLIC               0.95    46.05     4.26
 FERRI1 141 m   1              0.97     4.97     0.53       0.03
 FERRI1 100 f   1              0.37     6.90     0.53       0.33
 Subtotal FERRI1               0.62    11.87     1.06
 HAENSZ 45  m   0              0.82    20.11     0.60       0.00
 HAENSZ 71  f   0              0.75    23.13     0.26       0.00
 Subtotal HAENSZ               0.79    43.24     0.86
 HARRIS 4   m   0              1.09     2.34     0.46       0.10
 HIGGI2 4   m   0              0.71     0.84     0.00       0.52
 HIGGI2 10  f   0              0.44     2.29     0.10       0.51
 Subtotal HIGGI2               0.51     3.13     0.10
 HIGGI3 4   m   0             -0.12     0.39     0.23       0.94
 HIGGI6 5   m   1              1.06    10.21     1.72       0.00
 HIGGI6 26  f   1              0.33    10.93     1.11       0.28
 Subtotal HIGGI6               0.68    21.14     2.83
 HOLLA2 4   m   0              1.09     2.92     0.56       0.06
 HOLLNA 4   m   0              1.52     6.18     4.68       0.00
 HOLLNA 12  f   0              1.30     7.84     3.38       0.00
 Subtotal HOLLNA               1.40    14.02     8.06
 HUHTI1 45  m   0              2.31     5.24    14.44       0.00
 HUHTI1 104 f   0              0.90     5.81     0.36       0.03
 Subtotal HUHTI1               1.57    11.05    14.81
 HUHTI3 23  m   0              1.42    18.41    11.13       0.00
*KAHN2  3   m   1              0.76     4.11     0.05       0.13
 LAMBER 7   m   0              1.06    14.36     2.41       0.00
 LAMBER 55  f   0              0.78    29.10     0.53       0.00
 Subtotal LAMBER               0.87    43.45     2.93
 LAVECC 13  m   0              0.71   235.94     0.87       0.00
 LAVECC 16  f   0             -0.35    98.52    98.00       0.00
 Subtotal LAVECC               0.40   334.46    98.87
 LEBOWI 37  b   0              0.53    19.56     0.28       0.02
 LINDST 25  b   5              0.52   123.70     1.86       0.00
 MENEZ1 7   b   0              0.80    11.81     0.29       0.01
 MEREN  3   b   4              0.90    32.51     2.14       0.00
 MILNE  4   m   0              1.63     2.22     2.16       0.01
 MILNE  10  f   0              0.88     3.13     0.18       0.12
 Subtotal MILNE                1.20     5.34     2.34
 MUELLE 66  f   0              2.19     1.98     4.69       0.00
 OGILVI 15  m   0              2.03     3.40     6.46       0.00
 OGILVI 27  f   0              0.48    11.47     0.31       0.10
 Subtotal OGILVI               0.84    14.87     6.77
 REID   7   m   0              1.71    12.62    14.27       0.00
 REID   23  f   0              0.56    13.73     0.11       0.04
 Subtotal REID                 1.11    26.35    14.38
*TROISI 4   f   1              0.41    47.14     2.76       0.01
 URRUTI 4   b   0              0.87     6.59     0.33       0.03
 WAGEN2 4   b   0              0.22    42.58     7.95       0.16
*WEN    8   m   1              1.38     2.66     1.44       0.02
  ________________________________________________________________________________________________________________________
                                            International Evidence on Smoking and COPD, Phase 3, Analysis run on 28-SEP-10

                                                   Table 2 - E - 1 - 5

                                IESCOPD - Meta-analysis of amount smoked : key value (1) 5
                             Any CB, cigarettes (or any product if cigarettes not available)
                                                      Least-adjusted


 REF    NRR SEX ADJ             Ys       Ws       Qs       Ps
 WILHEL 4   m   0              1.59     0.82     0.72       0.15
 WOOLF  4   f   0              1.61     4.01     3.75       0.00
 YAMAGU 10  b   6              0.85    58.63     2.41       0.00

                       N       54
                      NS       38


                      Wt  1075.16
                 Het Chi   218.72
                 Het  df       53
                 Het  P       ***
               Fixed  RR     1.91
                     RRl     1.80
                     RRu     2.03
                      P       +++
              Random  RR     2.34
                     RRl     2.02
                     RRu     2.70
                      P       +++
               Asymm  P        **


  ________________________________________________________________________________________________________________________
                                            International Evidence on Smoking and COPD, Phase 3, Analysis run on 28-SEP-10

                                                   Table 2 - E - 1 - 6

                                IESCOPD - Meta-analysis of amount smoked : key value (1) 5
                             Any CB, cigarettes (or any product if cigarettes not available)
                                                      Least-adjusted


                       N       54
                      NS       38


                      Wt  1075.16
                 Het Chi   218.72
                 Het  df       53
                 Het  P       ***
               Fixed  RR     1.91
                     RRl     1.80
                     RRu     2.03
                      P       +++
              Random  RR     2.34
                     RRl     2.02
                     RRu     2.70
                      P       +++
               Asymm  P        **

                                   Sex
                             both      male    female     Total


                       N        8        27        19        54
                      NS        8        27        19        54


                      Wt   303.39    417.63    354.13   1075.16
                 Het Chi    15.43     50.52    108.26    218.72
                 Het  df        7        26        18        53
                 Het  P         *        **       ***       ***
               Fixed  RR     1.82      2.42      1.51      1.91
                     RRl     1.62      2.20      1.36      1.80
                     RRu     2.03      2.67      1.67      2.03
                      P       +++       +++       +++       +++
              Random  RR     1.85      3.01      1.99      2.34
                     RRl     1.53      2.49      1.49      2.02
                     RRu     2.24      3.64      2.65      2.70
                      P       +++       +++       +++       +++
             Between Chi                                  44.52
             Between  df                                      2
             Between  P                                     ***
             Btwn(F)  P                                      **

                                        Continent
                            NAmer    Europe      Asia  oth/mult     Total


                       N       18        30         2         4        54
                      NS       13        20         2         3        38


                      Wt   178.84    774.83     61.29     60.20   1075.16
                 Het Chi    43.15    161.59      0.72      0.27    218.72
                 Het  df       17        29         1         3        53
                 Het  P       ***       ***      N.S.      N.S.       ***
               Fixed  RR     2.12      1.79      2.39      2.53      1.91
                     RRl     1.83      1.67      1.86      1.97      1.80
                     RRu     2.46      1.92      3.08      3.26      2.03
                      P       +++       +++       +++       +++       +++
              Random  RR     2.38      2.29      2.39      2.53      2.34
                     RRl     1.83      1.87      1.86      1.97      2.02
                     RRu     3.08      2.81      3.08      3.26      2.70
                      P       +++       +++       +++       +++       +++
             Between Chi                                            12.98
             Between  df                                                3
             Between  P                                                **
             Btwn(F)  P                                              N.S.


  ________________________________________________________________________________________________________________________
                                            International Evidence on Smoking and COPD, Phase 3, Analysis run on 28-SEP-10

                                                   Table 2 - E - 1 - 6

                                IESCOPD - Meta-analysis of amount smoked : key value (1) 5
                             Any CB, cigarettes (or any product if cigarettes not available)
                                                      Least-adjusted
                               Study type
                               CC        Pr        CS     Total


                       N        5         6        43        54
                      NS        3         6        29        38


                      Wt    98.62     64.86    911.68   1075.16
                 Het Chi    12.94     12.60    190.94    218.72
                 Het  df        4         5        42        53
                 Het  P         *         *       ***       ***
               Fixed  RR     1.66      1.87      1.94      1.91
                     RRl     1.36      1.47      1.82      1.80
                     RRu     2.02      2.39      2.07      2.03
                      P       +++       +++       +++       +++
              Random  RR     1.94      2.83      2.37      2.34
                     RRl     1.30      1.63      2.00      2.02
                     RRu     2.88      4.93      2.80      2.70
                      P        ++       +++       +++       +++
             Between Chi                                   2.24
             Between  df                                      2
             Between  P                                    N.S.
             Btwn(F)  P                                    N.S.

                           CB subtype
                             mort     sympt     other     Total


                       N        4        42         8        54
                      NS        4        28         6        38


                      Wt    11.63    606.64    456.90   1075.16
                 Het Chi     3.09     94.56     82.80    218.72
                 Het  df        3        41         7        53
                 Het  P      N.S.       ***       ***       ***
               Fixed  RR     4.02      2.20      1.55      1.91
                     RRl     2.26      2.03      1.42      1.80
                     RRu     7.15      2.38      1.70      2.03
                      P       +++       +++       +++       +++
              Random  RR     4.04      2.45      1.64      2.34
                     RRl     2.25      2.13      1.12      2.02
                     RRu     7.24      2.82      2.41      2.70
                      P       +++       +++         +       +++
             Between Chi                                  38.26
             Between  df                                      2
             Between  P                                     ***
             Btwn(F)  P                                      **

                             Smoking product
                              any      cigs  cigsonly     Total


                       N        7        40         7        54
                      NS        6        27         5        38


                      Wt    38.90    959.54     76.72   1075.16
                 Het Chi     1.53    186.91      9.45    218.72
                 Het  df        6        39         6        53
                 Het  P      N.S.       ***      N.S.       ***
               Fixed  RR     3.89      1.85      2.00      1.91
                     RRl     2.84      1.74      1.60      1.80
                     RRu     5.32      1.97      2.51      2.03
                      P       +++       +++       +++       +++
              Random  RR     3.89      2.24      2.11      2.34
                     RRl     2.84      1.90      1.55      2.02
                     RRu     5.32      2.65      2.87      2.70
                      P       +++       +++       +++       +++
             Between Chi                                  20.82
             Between  df                                      2
             Between  P                                     ***
             Btwn(F)  P                                     (*)
  ________________________________________________________________________________________________________________________
                                            International Evidence on Smoking and COPD, Phase 3, Analysis run on 28-SEP-10

                                                   Table 2 - E - 1 - 6

                                IESCOPD - Meta-analysis of amount smoked : key value (1) 5
                             Any CB, cigarettes (or any product if cigarettes not available)
                                                      Least-adjusted
                                     Unexposed group
                          nev any   nev cig  nev+ any  nev+ cig     Total


                       N       33        21                            54
                      NS       22        17                            39


                      Wt   638.94    436.22                       1075.16
                 Het Chi   166.69     51.49                        218.72
                 Het  df       32        20                            53
                 Het  P       ***       ***                           ***
               Fixed  RR     1.88      1.96                          1.91
                     RRl     1.74      1.79                          1.80
                     RRu     2.03      2.16                          2.03
                      P       +++       +++                           +++
              Random  RR     2.46      2.20                          2.34
                     RRl     1.98      1.84                          2.02
                     RRu     3.06      2.63                          2.70
                      P       +++       +++                           +++
             Between Chi                                             0.53
             Between  df                                                1
             Between  P                                              N.S.
             Btwn(F)  P                                              N.S.

                        Unexposed group (combining nev+ with main levels)
                          nev any   nev cig     Total


                       N       33        21        54
                      NS       22        17        39


                      Wt   638.94    436.22   1075.16
                 Het Chi   166.69     51.49    218.72
                 Het  df       32        20        53
                 Het  P       ***       ***       ***
               Fixed  RR     1.88      1.96      1.91
                     RRl     1.74      1.79      1.80
                     RRu     2.03      2.16      2.03
                      P       +++       +++       +++
              Random  RR     2.46      2.20      2.34
                     RRl     1.98      1.84      2.02
                     RRu     3.06      2.63      2.70
                      P       +++       +++       +++
             Between Chi                         0.53
             Between  df                            1
             Between  P                          N.S.
             Btwn(F)  P                          N.S.


  ________________________________________________________________________________________________________________________
                                            International Evidence on Smoking and COPD, Phase 3, Analysis run on 28-SEP-10

                                                   Table 2 - E - 1 - 7

                                IESCOPD - Meta-analysis of amount smoked : key value (1) 5
                             Any CB, cigarettes (or any product if cigarettes not available)
                                 Excluded studies (and stage at which they were excluded)


1       CLARK COTTON  MEYER REMYJA RUTGER SNYDER SOBRAX     SU TAKEMU  WANG4   WEIR WHICKE ZALACA
2      ALESSA  AMIGO ANDER2 ANDER3 AUERBA BEDNAR BROGGE  CHEN1  CHEN2  CHEN3  CHENG CLEMEN  COCCI  DEAN1 DEJONG DETORR
       DICKIN DONTA1 EKBERG ENSTRO FERRI2 FERRI3  FIDAN FORAST FUKUCH GEIJER GODTFR GULSVI HAMMO2 HARIKK HEDMAN HIGGI4
       HOZAWA ITABAS JACOBS JAENDI JOHANN KACHEL KARAKA KATANC KHOURY    KIM KLAYTO KOJIMA KOTAN2 KRZYZA KULLER    LAI
         LAM1   LAM2   LAM3  LANGE    LEE   LIAW LINDBE   LIU1   LIU2 LUNDB1  MADOR MANNI1 MANNI2 MANNI3 MARAN1 MARAN2
       MARCUS MATHES MENEZ2 MENEZ3 MENEZ4 MENEZ5 MENEZ6 MONTNE   NAWA NIEPSU NIHLEN NILSSO  OMORI   PEAT   PETO  PRATT
        PRICE RENWIC RICCIO  RYDER SARGEA SHAHAB   SHIN SICHLE SPEIZE STERLI  STROM SUTINE  TAGER   TANG   THUN   TODD
       TRUPIN TSUSHI TVERDA VESTBO VIEGI2 VIKGRE VINEIS VOLLM1 VOLLM2 VONHER   WALD  WANG2 WATSON  WEISS WILSO1   XIAO
           XU   YUAN ZIELI1 ZIELI2 ZIETKO
3        KAHN
4        BANG  BECK1  BECK2 BJORNS CERVER COLLEG  DEANE DEMARC DOPICO ENRIGH FINKLE FOXMAN GOLDBE HARDIE  HAYES HIRAYA
           HO  HOUSE HUHTI2 JENSEN JINDA2  JOSHI JOUSI1   KATO  KIRAZ KOTAN1  KUBIK LANGE2 LANGHA LUNDB2 MAGNUS MANFRE
       MELLST MILLER MOLLER NEJJAR OSWAL1 OSWAL2 PANDEY PELKON PEREZP SAWICK SCHWAR SHIMUR  SILVA SOBRAD STJERN SUADIC
       TAGER2 VIEGI1 WOJTYN  WOODS   ZOIA
5       DOLL2 FLETCH HAWTHO HRUBEC RIMING  SHARP    WIG WILSO2
6      HUCHON


  ________________________________________________________________________________________________________________________
                                            International Evidence on Smoking and COPD, Phase 3, Analysis run on 28-SEP-10

                                                   Table 2 - E - 1 - 8

                                IESCOPD - Meta-analysis of amount smoked : key value (1) 5
                             Any CB, cigarettes (or any product if cigarettes not available)
                                             Potentially overlapping studies


     REF| REFGP|PRINC|                     OVERLAP|

  URRUTI DEMARC     2         DEMARC/URRUTI/DEMEER
  DONTA2 JACOBS     2  JACOBS/DONTA1/DONTA2/PELKON
  HIGGI2   PETO     2    PETO/HIGGI1/HIGGI2/HIGGI5
  HUHTI1 HUHTI1     1                HUHTI1/HUHTI2
  LEBOWI LEBOWI     1                 LEBOWI/SILVA
  FERRI1 FERRIS     2         FERRI1/FERRI2/FERRI3
     WEN    WEN     1                     WEN/LIAW
  LAMBER   TODD     2                  LAMBER/TODD
  CHAPMA  HOUSE     2                 HOUSE/CHAPMA
  HOLLNA HOLLNA     1 GODT/VEST/LANG1+2/SUAD/HOLLN
  HIGGI6 HIGGI4     2                HIGGI4/HIGGI6
   KAHN2   KAHN     2                   KAHN/KAHN2


  ________________________________________________________________________________________________________________________
                                            International Evidence on Smoking and COPD, Phase 3, Analysis run on 28-SEP-10

                                                    Table 2 - E - 2 -

                               IESCOPD - Meta-analysis of amount smoked : key value (1) 20
                             Any CB, cigarettes (or any product if cigarettes not available)


This analysis is restricted to results for:
1) Eligible study on database
2) Outcome CB
3) Current or ever smoking
4) Categorical dose-response data for amount smoked
5) vs never smoking base
6) Key value (scheme 1) = 20
7) Results complete enough for use in meta-analysis

Within each study, results are then selected (in the following order of preference, within each sex) for:
8) SMKSTA  : current, ever
9) UNEXP   : never any, never cigarettes
10) PROD    : cigarettes, cigarettes only, any product
11) For overlapping studies: principal rather than subsidiary studies
and then for single sex results (m, f) in preference to results for both sexes combined (b).

Results adjusted for the most potential confounders are then chosen in Sections -1 to -3
and results adjusted for the least confounders in Sections -4 to -6. (Those least-adjusted results which
actually differ from the most-adjusted are marked 'x' in column X in Section -4)

Section -7 shows excluded studies, together with the stage (as above) at which no qualifying
results were found.

Section -8 lists the potentially overlapping studies which have been included (1=principal, 2=subsidiary),
and any results which would have been included in preference except that they had data not complete enough
for use in meta-analysis. It also lists their significance (yes/no), if known.


  ________________________________________________________________________________________________________________________
                                            International Evidence on Smoking and COPD, Phase 3, Analysis run on 28-SEP-10

                                                   Table 2 - E - 2 - 1

                               IESCOPD - Meta-analysis of amount smoked : key value (1) 20
                             Any CB, cigarettes (or any product if cigarettes not available)
                                                      Most-adjusted


     REF|NRR|SEX|AGEL|AGEH|     REGION|BEGYR|PUBYR|STTYP|ONSET|      DISEAS|ADJ|SMOKSTA|   PRODUCT|    UNEXP|LOW| HI|

  ALDERS  12   m   35   74       Eu:UK  1977  1985    CC  Prev CB:diagnosed   1    Ever MCigs only   Nev any  18  27
  ALDERS  17   f   35   74       Eu:UK  1977  1985    CC  Prev CB:diagnosed   1    Ever MCigs only   Nev any  18  27
  ANDER1  41   m   25   74   Am:Canada  1963  1965    CS  Prev  CB:symptoms   1 Current       Cigs   Nev any  15  24
  ANDER1  46   f   25   74   Am:Canada  1963  1965    CS  Prev  CB:symptoms   1 Current       Cigs   Nev any  15  24
    BEST   5   m   30   97   Am:Canada  1955  1967    Pr   Inc CB:mortality   1 Current  Cigs only   Nev any  10  20
  CHAPMA  21   m   15   99      Am:USA  1976  1985    CS  Prev  CB:symptoms   1 Current       Cigs  Nev cigs  20  20
  CHAPMA  29   f   15   99      Am:USA  1976  1985    CS  Prev  CB:symptoms   1 Current       Cigs  Nev cigs  20  20
  COATES  10   b   40   64      Am:USA  1962  1965    CS  Prev  CB:symptoms   0 Current       Cigs  Nev cigs  15  24
   DEAN2  12   m   37   67       Eu:UK  1972  1978    CS  Prev  CB:symptoms   1 Current MCigs only   Nev any  18  22
   DEAN2  26   f   37   67       Eu:UK  1972  1978    CS  Prev  CB:symptoms   1 Current MCigs only   Nev any  18  22
   DOLL1  17   m   20   99       Eu:UK  1951  1994    Pr   Inc CB:mortality   1 Current  Cigs only   Nev any  15  24
  DONTA2   6   m   25   84 Eu:SE/Balkn  1960  1984    Pr   Inc CB:diagnosed   0 Current       Cigs  Nev cigs  20  29
  FERRI1 142   m   25   74      Am:USA  1961  1971    CS  Prev  CB:symptoms   1 Current       Cigs   Nev any  11  20
  FERRI1 101   f   25   74      Am:USA  1961  1971    CS  Prev  CB:symptoms   1 Current       Cigs   Nev any  11  20
  HARRIS   5   m   15   60      Africa     *  1993    CS  Prev  CB:symptoms   0 Current        Any   Nev any  15  24
  HOLLA2   5   m   40   59      Am:USA  1962  1965    CS  Prev  CB:symptoms   0 Current        Any   Nev any  15  24
  HOLLNA   5   m   40   40    Eu:Scand  1976  1983    CS  Prev  CB:symptoms   0 Current        Any   Nev any  15  24
  HOLLNA  13   f   40   40    Eu:Scand  1976  1983    CS  Prev  CB:symptoms   0 Current        Any   Nev any  15  24
  HUHTI1  51   m   40   64    Eu:Scand  1961  1965    CS  Prev  CB:symptoms   1 Current       Cigs   Nev any  15  24
  HUHTI3  29   m   25   69    Eu:Scand  1968  1978    CS  Prev  CB:symptoms   1 Current        Any   Nev any  15  24
   KAHN2   4   m   31   84      Am:USA  1954  1966    Pr   Inc CB:mortality   1 Current       Cigs   Nev any  10  20
  LAMBER  13   m   35   69       Eu:UK  1965  1970    CS  Prev  CB:symptoms   1 Current       Cigs  Nev cigs  20  20
  LAMBER  59   f   35   69       Eu:UK  1965  1970    CS  Prev  CB:symptoms   1 Current       Cigs   Nev any  20  20
  MUELLE  72   f   20   69      Am:USA  1967  1971    CS  Prev  CB:symptoms   1 Current       Cigs   Nev any  15  24
  OGILVI  17   m   30   99       Eu:UK  1955  1957    CC  Prev  CB:symptoms   0 Current       Cigs   Nev any  16  25
  OGILVI  51   f   30   99       Eu:UK  1955  1957    CC  Prev  CB:symptoms   0    Ever       Cigs   Nev any  16  25
    REID  13   m   35   74      Am:USA  1962  1966    CS  Prev  CB:symptoms   1 Current       Cigs  Nev cigs  20  20
    REID  29   f   35   74      Am:USA  1962  1966    CS  Prev  CB:symptoms   1 Current       Cigs  Nev cigs  20  20
  TROISI   5   f   34   69      Am:USA  1980  1995    Pr   Inc CB:diagnosed   1 Current       Cigs  Nev cigs  15  25
  URRUTI   5   b   20   44     Eu:West     *  2005    CS  Prev  CB:symptoms   0 Current       Cigs  Nev cigs  10  20
  WAGEN2   5   b   21   68     Eu:West  2001  2004    CC  Prev  CB:symptoms   0    Ever       Cigs  Nev cigs  11  20
     WEN   9   m   35   99   Asia:FarE  1982  2004    Pr   Inc CB:mortality   1 Current       Cigs  Nev cigs  11  20
   WOOLF   5   f   25   54   Am:Canada     *  1974    CS  Prev  CB:symptoms   0 Current       Cigs  Nev cigs  11  20


  ________________________________________________________________________________________________________________________
                                            International Evidence on Smoking and COPD, Phase 3, Analysis run on 28-SEP-10

                                                   Table 2 - E - 2 - 2

                               IESCOPD - Meta-analysis of amount smoked : key value (1) 20
                             Any CB, cigarettes (or any product if cigarettes not available)
                                                      Most-adjusted


                        Number Exposed  Non-exposed
 REF    NRR SEX ADJ     Case    Cont    Case    Cont      RR        95.00%CI
 ALDERS 12  m   1         72       -      31       -      1.96 (  1.15-   3.34)
 ALDERS 17  f   1        125       -     111       -      3.12 (  2.17-   4.49)
 Subtotal ALDERS                                          2.69 (  1.99-   3.64)
 ANDER1 41  m   1         16       -       4       -      7.03 (  2.11-  23.42)
 ANDER1 46  f   1          5       -      15       -      2.56 (  0.83-   7.87)
 Subtotal ANDER1                                          4.10 (  1.80-   9.32)
*BEST   5   m   1         49       -       3       -     13.65 (  4.26-  43.78)
 CHAPMA 21  m   1         72       -      35       -      5.52 (  3.58-   8.51)
 CHAPMA 29  f   1         48       -      48       -      6.07 (  3.98-   9.27)
 Subtotal CHAPMA                                          5.80 (  4.28-   7.84)
 COATES 10  b   0         69     333      21     515      5.08 (  3.06-   8.44)
 DEAN2  12  m   1         61       -      39       -      2.57 (  1.70-   3.89)
 DEAN2  26  f   1         69       -      80       -      4.59 (  3.29-   6.42)
 Subtotal DEAN2                                           3.65 (  2.81-   4.73)
*DOLL1  17  m   1         37       -       3       -     12.80 (  3.74-  43.85)
*DONTA2 6   m   0         19      67       8     127      4.50 (  2.08-   9.73)
 FERRI1 142 m   1         44       -      10       -      3.25 (  1.53-   6.91)
 FERRI1 101 f   1         17       -      35       -      2.75 (  1.45-   5.22)
 Subtotal FERRI1                                          2.95 (  1.81-   4.81)
 HARRIS 5   m   0          3       2       6     518    129.50 ( 18.21- 921.07)
 HOLLA2 5   m   0         38     131       5      84      4.87 (  1.84-  12.88)
 HOLLNA 5   m   0         72      89      10     101      8.17 (  3.98-  16.79)
 HOLLNA 13  f   0         42      86      14     217      7.57 (  3.93-  14.56)
 Subtotal HOLLNA                                          7.84 (  4.83-  12.72)
 HUHTI1 51  m   1         79       -       7       -     10.12 (  4.39-  23.29)
 HUHTI3 29  m   1         79       -      28       -      4.33 (  2.49-   7.53)
*KAHN2  4   m   1         34       -      13       -      4.34 (  2.29-   8.22)
 LAMBER 13  m   1         41       -      17       -      2.99 (  1.67-   5.36)
 LAMBER 59  f   1         29       -      54       -      5.14 (  3.12-   8.47)
 Subtotal LAMBER                                          4.09 (  2.80-   5.97)
 MUELLE 72  f   1         14       -       3       -     15.14 (  3.04-  75.48)
 OGILVI 17  m   0         66      44      11      29      3.95 (  1.79-   8.73)
 OGILVI 51  f   0         24      13      86     160      3.43 (  1.67-   7.08)
 Subtotal OGILVI                                          3.66 (  2.15-   6.25)
 REID   13  m   1         66       -      16       -      6.07 (  3.46-  10.62)
 REID   29  f   1         19       -      37       -      4.30 (  2.38-   7.75)
 Subtotal REID                                            5.15 (  3.43-   7.74)
*TROISI 5   f   1          -       -       -       -      2.63 (  2.13-   3.13)
 URRUTI 5   b   0         67     540      16     707      5.48 (  3.14-   9.57)
 WAGEN2 5   b   0        111    1084     111    2136      1.97 (  1.50-   2.59)
*WEN    9   m   1          -       -       7       -      2.03 (  0.57-   7.23)
 WOOLF  5   f   0         25      67      10     173      6.46 (  2.94-  14.16)
Partial Totals          1512    2456     894    4767
*prospective study


 REF    NRR SEX ADJ             Ys       Ws       Qs       Ps
 ALDERS 12  m   1              0.67    13.52     5.16       0.01
 ALDERS 17  f   1              1.14    29.06     0.68       0.00
 Subtotal ALDERS               0.99    42.58     5.84
 ANDER1 41  m   1              1.95     2.65     1.15       0.00
 ANDER1 46  f   1              0.94     3.04     0.37       0.10
 Subtotal ANDER1               1.41     5.69     1.53
*BEST   5   m   1              2.61     2.83     4.95       0.00
 CHAPMA 21  m   1              1.71    20.49     3.57       0.00
 CHAPMA 29  f   1              1.80    21.49     5.64       0.00
 Subtotal CHAPMA               1.76    41.99     9.22
 COATES 10  b   0              1.63    14.91     1.67       0.00
 DEAN2  12  m   1              0.94    22.42     2.70       0.00
 DEAN2  26  f   1              1.52    34.38     1.87       0.00
 Subtotal DEAN2                1.29    56.81     4.57
*DOLL1  17  m   1              2.55     2.54     4.02       0.00
*DONTA2 6   m   0              1.50     6.46     0.29       0.00
 FERRI1 142 m   1              1.18     6.76     0.09       0.00
 FERRI1 101 f   1              1.01     9.36     0.73       0.00
 Subtotal FERRI1               1.08    16.12     0.82
 HARRIS 5   m   0              4.86     1.00    12.74       0.00
 HOLLA2 5   m   0              1.58     4.07     0.35       0.00
 HOLLNA 5   m   0              2.10     7.41     4.85       0.00
 HOLLNA 13  f   0              2.02     8.97     4.82       0.00
 Subtotal HOLLNA               2.06    16.38     9.68
  ________________________________________________________________________________________________________________________
                                            International Evidence on Smoking and COPD, Phase 3, Analysis run on 28-SEP-10

                                                   Table 2 - E - 2 - 2

                               IESCOPD - Meta-analysis of amount smoked : key value (1) 20
                             Any CB, cigarettes (or any product if cigarettes not available)
                                                      Most-adjusted


 REF    NRR SEX ADJ             Ys       Ws       Qs       Ps
 HUHTI1 51  m   1              2.31     5.52     5.78       0.00
 HUHTI3 29  m   1              1.47    12.55     0.38       0.00
*KAHN2  4   m   1              1.47     9.41     0.29       0.00
 LAMBER 13  m   1              1.10    11.30     0.43       0.00
 LAMBER 59  f   1              1.64    15.41     1.85       0.00
 Subtotal LAMBER               1.41    26.71     2.28
 MUELLE 72  f   1              2.72     1.49     3.03       0.00
 OGILVI 17  m   0              1.37     6.12     0.04       0.00
 OGILVI 51  f   0              1.23     7.33     0.02       0.00
 Subtotal OGILVI               1.30    13.45     0.07
 REID   13  m   1              1.80    12.22     3.21       0.00
 REID   29  f   1              1.46    11.02     0.31       0.00
 Subtotal REID                 1.64    23.24     3.52
*TROISI 5   f   1              0.97   103.71    10.88       0.00
 URRUTI 5   b   0              1.70    12.39     2.09       0.00
 WAGEN2 5   b   0              0.68    51.52    19.34       0.00
*WEN    9   m   1              0.71     2.38     0.81       0.27
 WOOLF  5   f   0              1.86     6.22     2.05       0.00

                       N       33
                      NS       24


                      Wt   479.96
                 Het Chi   106.19
                 Het  df       32
                 Het  P       ***
               Fixed  RR     3.64
                     RRl     3.33
                     RRu     3.98
                      P       +++
              Random  RR     4.43
                     RRl     3.68
                     RRu     5.32
                      P       +++
               Asymm  P       ***


  ________________________________________________________________________________________________________________________
                                            International Evidence on Smoking and COPD, Phase 3, Analysis run on 28-SEP-10

                                                   Table 2 - E - 2 - 3

                               IESCOPD - Meta-analysis of amount smoked : key value (1) 20
                             Any CB, cigarettes (or any product if cigarettes not available)
                                                      Most-adjusted


                       N       33
                      NS       24


                      Wt   479.96
                 Het Chi   106.19
                 Het  df       32
                 Het  P       ***
               Fixed  RR     3.64
                     RRl     3.33
                     RRu     3.98
                      P       +++
              Random  RR     4.43
                     RRl     3.68
                     RRu     5.32
                      P       +++
               Asymm  P       ***

                                   Sex
                             both      male    female     Total


                       N        3        18        12        33
                      NS        3        18        12        33


                      Wt    78.83    149.64    251.49    479.96
                 Het Chi    17.24     46.60     32.21    106.19
                 Het  df        2        17        11        32
                 Het  P       ***       ***       ***       ***
               Fixed  RR     2.77      4.30      3.58      3.64
                     RRl     2.22      3.67      3.17      3.33
                     RRu     3.45      5.05      4.05      3.98
                      P       +++       +++       +++       +++
              Random  RR     3.69      4.87      4.22      4.43
                     RRl     1.76      3.67      3.28      3.68
                     RRu     7.76      6.48      5.42      5.32
                      P       +++       +++       +++       +++
             Between Chi                                  10.14
             Between  df                                      2
             Between  P                                      **
             Btwn(F)  P                                    N.S.

                                        Continent
                            NAmer    Europe      Asia  oth/mult     Total


                       N       15        16         1         1        33
                      NS       11        11         1         1        24


                      Wt   229.69    246.90      2.38      1.00    479.96
                 Het Chi    37.98     53.86      0.00      0.00    106.19
                 Het  df       14        15         0         0        32
                 Het  P       ***       ***      N.S.      N.S.       ***
               Fixed  RR     3.78      3.48      2.03    129.50      3.64
                     RRl     3.32      3.07      0.57     18.21      3.33
                     RRu     4.30      3.94      7.23    921.07      3.98
                      P       +++       +++      N.S.       +++       +++
              Random  RR     4.69      4.10      2.03    129.50      4.43
                     RRl     3.62      3.18      0.57     18.21      3.68
                     RRu     6.09      5.29      7.23    921.07      5.32
                      P       +++       +++      N.S.       +++       +++
             Between Chi                                            14.35
             Between  df                                                3
             Between  P                                                **
             Btwn(F)  P                                              N.S.


  ________________________________________________________________________________________________________________________
                                            International Evidence on Smoking and COPD, Phase 3, Analysis run on 28-SEP-10

                                                   Table 2 - E - 2 - 3

                               IESCOPD - Meta-analysis of amount smoked : key value (1) 20
                             Any CB, cigarettes (or any product if cigarettes not available)
                                                      Most-adjusted
                        National cigarette tobacco type (excluding mixed/unkown)
                          blended  virginia     Total


                       N       18        14        32
                      NS       14         9        23


                      Wt   319.76    157.82    477.58
                 Het Chi    67.29     37.86    105.38
                 Het  df       17        13        31
                 Het  P       ***       ***       ***
               Fixed  RR     3.59      3.76      3.65
                     RRl     3.22      3.22      3.33
                     RRu     4.01      4.40      3.99
                      P       +++       +++       +++
              Random  RR     4.65      4.29      4.48
                     RRl     3.63      3.18      3.72
                     RRu     5.95      5.79      5.40
                      P       +++       +++       +++
             Between Chi                         0.23
             Between  df                            1
             Between  P                          N.S.
             Btwn(F)  P                          N.S.

                                        Start year of study
                            <1970   1970-79   1980-89   1990-99     2000+   unknown     Total


                       N       19         8         2                   1         3        33
                      NS       14         4         2                   1         3        24


                      Wt   144.98    157.75    106.09               51.52     19.61    479.96
                 Het Chi    20.64     26.88      0.16                0.00      9.26    106.19
                 Het  df       18         7         1                   0         2        32
                 Het  P      N.S.       ***      N.S.                N.S.        **       ***
               Fixed  RR     4.62      4.12      2.61                1.97      6.78      3.64
                     RRl     3.93      3.52      2.16                1.50      4.36      3.33
                     RRu     5.44      4.81      3.16                2.59     10.56      3.98
                      P       +++       +++       +++                 +++       +++       +++
              Random  RR     4.65      4.29      2.61                1.97     10.92      4.43
                     RRl     3.89      3.12      2.16                1.50      3.45      3.68
                     RRu     5.55      5.88      3.16                2.59     34.58      5.32
                      P       +++       +++       +++                 +++       +++       +++
             Between Chi                                                                49.26
             Between  df                                                                    4
             Between  P                                                                   ***
             Btwn(F)  P                                                                    **

                                Publication year
                            <1980   1980-89   1990-99     2000+     Total


                       N       20         7         3         3        33
                      NS       14         4         3         3        24


                      Wt   199.02    107.40    107.25     66.30    479.96
                 Het Chi    25.48     21.03     20.92     10.53    106.19
                 Het  df       19         6         2         2        32
                 Het  P      N.S.        **       ***        **       ***
               Fixed  RR     4.31      4.41      2.83      2.39      3.64
                     RRl     3.75      3.65      2.34      1.88      3.33
                     RRu     4.96      5.33      3.42      3.04      3.98
                      P       +++       +++       +++       +++       +++
              Random  RR     4.39      4.65     13.40      2.89      4.43
                     RRl     3.70      3.21      1.79      1.32      3.68
                     RRu     5.20      6.74    100.41      6.33      5.32
                      P       +++       +++         +        ++       +++
             Between Chi                                            28.23
             Between  df                                                3
             Between  P                                               ***
             Btwn(F)  P                                                 *
  ________________________________________________________________________________________________________________________
                                            International Evidence on Smoking and COPD, Phase 3, Analysis run on 28-SEP-10

                                                   Table 2 - E - 2 - 3

                               IESCOPD - Meta-analysis of amount smoked : key value (1) 20
                             Any CB, cigarettes (or any product if cigarettes not available)
                                                      Most-adjusted
                               Study type
                               CC        Pr        CS     Total


                       N        5         6        22        33
                      NS        3         6        15        24


                      Wt   107.56    127.33    245.08    479.96
                 Het Chi     7.03     16.34     40.11    106.19
                 Het  df        4         5        21        32
                 Het  P      N.S.        **        **       ***
               Fixed  RR     2.41      2.99      4.82      3.64
                     RRl     1.99      2.51      4.26      3.33
                     RRu     2.91      3.55      5.47      3.98
                      P       +++       +++       +++       +++
              Random  RR     2.55      4.57      5.01      4.43
                     RRl     1.92      2.66      4.16      3.68
                     RRu     3.38      7.85      6.03      5.32
                      P       +++       +++       +++       +++
             Between Chi                                  42.72
             Between  df                                      2
             Between  P                                     ***
             Btwn(F)  P                                     ***

                                    Lowest age in RR
                        <25/unlim     25-39       40+   unknown     Total


                       N        7        21         5                  33
                      NS        6        14         4                  24


                      Wt   110.93    328.16     40.88              479.96
                 Het Chi    50.40     36.16      2.91              106.19
                 Het  df        6        20         4                  32
                 Het  P       ***         *      N.S.                 ***
               Fixed  RR     3.70      3.36      6.61                3.64
                     RRl     3.07      3.01      4.86                3.33
                     RRu     4.46      3.74      8.98                3.98
                      P       +++       +++       +++                 +++
              Random  RR     6.98      3.66      6.61                4.43
                     RRl     3.62      3.09      4.86                3.68
                     RRu    13.44      4.32      8.98                5.32
                      P       +++       +++       +++                 +++
             Between Chi                                            16.73
             Between  df                                                2
             Between  P                                               ***
             Btwn(F)  P                                               (*)

                                         Highest age in RR
                              <65     65-74     75-84 85+/unlim   unknown     Total


                       N        8        16         2         7                  33
                      NS        7        10         2         5                  24


                      Wt    60.49    340.42     15.87     63.19              479.96
                 Het Chi    12.18     39.82      0.01      9.00              106.19
                 Het  df        7        15         1         6                  32
                 Het  P       (*)       ***      N.S.      N.S.                 ***
               Fixed  RR     6.66      3.01      4.41      5.42                3.64
                     RRl     5.18      2.70      2.69      4.24                3.33
                     RRu     8.57      3.34      7.21      6.93                3.98
                      P       +++       +++       +++       +++                 +++
              Random  RR     7.09      3.34      4.41      5.35                4.43
                     RRl     4.99      2.73      2.69      3.83                3.68
                     RRu    10.06      4.08      7.21      7.49                5.32
                      P       +++       +++       +++       +++                 +++
             Between Chi                                                      45.19
             Between  df                                                          3
             Between  P                                                         ***
             Btwn(F)  P                                                         ***
  ________________________________________________________________________________________________________________________
                                            International Evidence on Smoking and COPD, Phase 3, Analysis run on 28-SEP-10

                                                   Table 2 - E - 2 - 3

                               IESCOPD - Meta-analysis of amount smoked : key value (1) 20
                             Any CB, cigarettes (or any product if cigarettes not available)
                                                      Most-adjusted
                           Study weakness
                              Yes        No     Total


                       N        1        32        33
                      NS        1        23        24


                      Wt     6.46    473.50    479.96
                 Het Chi     0.00    105.89    106.19
                 Het  df        0        31        32
                 Het  P      N.S.       ***       ***
               Fixed  RR     4.50      3.63      3.64
                     RRl     2.08      3.31      3.33
                     RRu     9.73      3.97      3.98
                      P       +++       +++       +++
              Random  RR     4.50      4.43      4.43
                     RRl     2.08      3.67      3.68
                     RRu     9.73      5.35      5.32
                      P       +++       +++       +++
             Between Chi                         0.30
             Between  df                            1
             Between  P                          N.S.
             Btwn(F)  P                          N.S.

                           CB subtype
                             mort     sympt     other     Total


                       N        4        25         4        33
                      NS        4        17         3        24


                      Wt    17.15    310.06    152.75    479.96
                 Het Chi     7.04     74.49      3.75    106.19
                 Het  df        3        24         3        32
                 Het  P       (*)       ***      N.S.       ***
               Fixed  RR     5.54      4.11      2.71      3.64
                     RRl     3.45      3.68      2.31      3.33
                     RRu     8.89      4.59      3.17      3.98
                      P       +++       +++       +++       +++
              Random  RR     6.08      4.71      2.74      4.43
                     RRl     2.73      3.81      2.23      3.68
                     RRu    13.50      5.83      3.36      5.32
                      P       +++       +++       +++       +++
             Between Chi                                  20.91
             Between  df                                      2
             Between  P                                     ***
             Btwn(F)  P                                       *

                           Asthma analysis type (CB)
                        inc-irres  excl-all excl-cntr     Total


                       N       27         6                  33
                      NS       18         6                  24


                      Wt   298.65    181.31              479.96
                 Het Chi    49.89     32.74              106.19
                 Het  df       26         5                  32
                 Het  P        **       ***                 ***
               Fixed  RR     4.32      2.74                3.64
                     RRl     3.86      2.37                3.33
                     RRu     4.84      3.16                3.98
                      P       +++       +++                 +++
              Random  RR     4.47      4.27                4.43
                     RRl     3.78      2.60                3.68
                     RRu     5.29      7.00                5.32
                      P       +++       +++                 +++
             Between Chi                                  23.56
             Between  df                                      1
             Between  P                                     ***
             Btwn(F)  P                                      **
  ________________________________________________________________________________________________________________________
                                            International Evidence on Smoking and COPD, Phase 3, Analysis run on 28-SEP-10

                                                   Table 2 - E - 2 - 3

                               IESCOPD - Meta-analysis of amount smoked : key value (1) 20
                             Any CB, cigarettes (or any product if cigarettes not available)
                                                      Most-adjusted
                                   Number of CB cases
                             1-50    51-100   101-200      201+     Total


                       N        2         5         6        20        33
                      NS        2         4         6        12        24


                      Wt     3.38     16.47     49.54    410.58    479.96
                 Het Chi    12.15      5.99      2.64     70.21    106.19
                 Het  df        1         4         5        19        32
                 Het  P       ***      N.S.      N.S.       ***       ***
               Fixed  RR     6.93      5.89      5.41      3.38      3.64
                     RRl     2.39      3.63      4.10      3.07      3.33
                     RRu    20.12      9.54      7.15      3.73      3.98
                      P       +++       +++       +++       +++       +++
              Random  RR    15.12      6.21      5.41      3.92      4.43
                     RRl     0.26      3.36      4.10      3.20      3.68
                     RRu   885.35     11.48      7.15      4.80      5.32
                      P      N.S.       +++       +++       +++       +++
             Between Chi                                            15.22
             Between  df                                                3
             Between  P                                                **
             Btwn(F)  P                                              N.S.

                         Number of CB cases (excluding unknown)


                       N        2         5         6        20        33
                      NS        2         4         6        12        24


                      Wt     3.38     16.47     49.54    410.58    479.96
                 Het Chi    12.15      5.99      2.64     70.21    106.19
                 Het  df        1         4         5        19        32
                 Het  P       ***      N.S.      N.S.       ***       ***
               Fixed  RR     6.93      5.89      5.41      3.38      3.64
                     RRl     2.39      3.63      4.10      3.07      3.33
                     RRu    20.12      9.54      7.15      3.73      3.98
                      P       +++       +++       +++       +++       +++
              Random  RR    15.12      6.21      5.41      3.92      4.43
                     RRl     0.26      3.36      4.10      3.20      3.68
                     RRu   885.35     11.48      7.15      4.80      5.32
                      P      N.S.       +++       +++       +++       +++
             Between Chi                                            15.22
             Between  df                                                3
             Between  P                                                **
             Btwn(F)  P                                              N.S.

                            Analysis type
                         prevlnce     onset     Total


                       N       27         6        33
                      NS       18         6        24


                      Wt   352.64    127.33    479.96
                 Het Chi    83.17     16.34    106.19
                 Het  df       26         5        32
                 Het  P       ***        **       ***
               Fixed  RR     3.90      2.99      3.64
                     RRl     3.52      2.51      3.33
                     RRu     4.33      3.55      3.98
                      P       +++       +++       +++
              Random  RR     4.44      4.57      4.43
                     RRl     3.63      2.66      3.68
                     RRu     5.44      7.85      5.32
                      P       +++       +++       +++
             Between Chi                         6.68
             Between  df                            1
             Between  P                            **
             Btwn(F)  P                          N.S.

  ________________________________________________________________________________________________________________________
                                            International Evidence on Smoking and COPD, Phase 3, Analysis run on 28-SEP-10

                                                   Table 2 - E - 2 - 3

                               IESCOPD - Meta-analysis of amount smoked : key value (1) 20
                             Any CB, cigarettes (or any product if cigarettes not available)
                                                      Most-adjusted
                             Smoking product
                              any      cigs  cigsonly     Total


                       N        5        22         6        33
                      NS        4        16         4        24


                      Wt    33.99    341.22    104.75    479.96
                 Het Chi    11.94     63.10     19.07    106.19
                 Het  df        4        21         5        32
                 Het  P         *       ***        **       ***
               Fixed  RR     6.46      3.49      3.45      3.64
                     RRl     4.61      3.14      2.85      3.33
                     RRu     9.04      3.88      4.17      3.98
                      P       +++       +++       +++       +++
              Random  RR     7.68      4.21      3.82      4.43
                     RRl     4.07      3.41      2.51      3.68
                     RRu    14.48      5.20      5.83      5.32
                      P       +++       +++       +++       +++
             Between Chi                                  12.08
             Between  df                                      2
             Between  P                                      **
             Btwn(F)  P                                    N.S.

                                     Unexposed group
                          nev any   nev cig  nev+ any  nev+ cig     Total


                       N       21        12                            33
                      NS       15        10                            25


                      Wt   205.83    274.13                        479.96
                 Het Chi    52.59     47.83                        106.19
                 Het  df       20        11                            32
                 Het  P       ***       ***                           ***
               Fixed  RR     4.13      3.31                          3.64
                     RRl     3.60      2.94                          3.33
                     RRu     4.73      3.72                          3.98
                      P       +++       +++                           +++
              Random  RR     4.70      4.09                          4.43
                     RRl     3.68      3.08                          3.68
                     RRu     6.00      5.43                          5.32
                      P       +++       +++                           +++
             Between Chi                                             5.77
             Between  df                                                1
             Between  P                                                 *
             Btwn(F)  P                                              N.S.

                        Unexposed group (combining nev+ with main levels)
                          nev any   nev cig     Total


                       N       21        12        33
                      NS       15        10        25


                      Wt   205.83    274.13    479.96
                 Het Chi    52.59     47.83    106.19
                 Het  df       20        11        32
                 Het  P       ***       ***       ***
               Fixed  RR     4.13      3.31      3.64
                     RRl     3.60      2.94      3.33
                     RRu     4.73      3.72      3.98
                      P       +++       +++       +++
              Random  RR     4.70      4.09      4.43
                     RRl     3.68      3.08      3.68
                     RRu     6.00      5.43      5.32
                      P       +++       +++       +++
             Between Chi                         5.77
             Between  df                            1
             Between  P                             *
             Btwn(F)  P                          N.S.
  ________________________________________________________________________________________________________________________
                                            International Evidence on Smoking and COPD, Phase 3, Analysis run on 28-SEP-10

                                                   Table 2 - E - 2 - 3

                               IESCOPD - Meta-analysis of amount smoked : key value (1) 20
                             Any CB, cigarettes (or any product if cigarettes not available)
                                                      Most-adjusted
                        Number of adjustment variables
                                0         1        2+     Total


                       N       11        22                  33
                      NS        9        15                  24


                      Wt   126.40    353.56              479.96
                 Het Chi    48.27     57.91              106.19
                 Het  df       10        21                  32
                 Het  P       ***       ***                 ***
               Fixed  RR     3.66      3.63                3.64
                     RRl     3.08      3.27                3.33
                     RRu     4.36      4.03                3.98
                      P       +++       +++                 +++
              Random  RR     5.28      4.14                4.43
                     RRl     3.43      3.39                3.68
                     RRu     8.14      5.05                5.32
                      P       +++       +++                 +++
             Between Chi                                   0.01
             Between  df                                      1
             Between  P                                    N.S.
             Btwn(F)  P                                    N.S.


  ________________________________________________________________________________________________________________________
                                            International Evidence on Smoking and COPD, Phase 3, Analysis run on 28-SEP-10

                                                   Table 2 - E - 2 - 4

                               IESCOPD - Meta-analysis of amount smoked : key value (1) 20
                             Any CB, cigarettes (or any product if cigarettes not available)
                                                      Least-adjusted


     REF|NRR|X|SEX|AGEL|AGEH|     REGION|BEGYR|PUBYR|STTYP|ONSET|      DISEAS|ADJ|SMOKSTA|   PRODUCT|    UNEXP|LOW| HI|

  ALDERS  12     m   35   74       Eu:UK  1977  1985    CC  Prev CB:diagnosed   1    Ever MCigs only   Nev any  18  27
  ALDERS  17     f   35   74       Eu:UK  1977  1985    CC  Prev CB:diagnosed   1    Ever MCigs only   Nev any  18  27
  ANDER1  41     m   25   74   Am:Canada  1963  1965    CS  Prev  CB:symptoms   1 Current       Cigs   Nev any  15  24
  ANDER1  46     f   25   74   Am:Canada  1963  1965    CS  Prev  CB:symptoms   1 Current       Cigs   Nev any  15  24
    BEST   5     m   30   97   Am:Canada  1955  1967    Pr   Inc CB:mortality   1 Current  Cigs only   Nev any  10  20
  CHAPMA   5 x   m   15   99      Am:USA  1976  1985    CS  Prev  CB:symptoms   0 Current       Cigs  Nev cigs  20  20
  CHAPMA  13 x   f   15   99      Am:USA  1976  1985    CS  Prev  CB:symptoms   0 Current       Cigs  Nev cigs  20  20
  COATES  10     b   40   64      Am:USA  1962  1965    CS  Prev  CB:symptoms   0 Current       Cigs  Nev cigs  15  24
   DEAN2  12     m   37   67       Eu:UK  1972  1978    CS  Prev  CB:symptoms   1 Current MCigs only   Nev any  18  22
   DEAN2  26     f   37   67       Eu:UK  1972  1978    CS  Prev  CB:symptoms   1 Current MCigs only   Nev any  18  22
   DOLL1  17     m   20   99       Eu:UK  1951  1994    Pr   Inc CB:mortality   1 Current  Cigs only   Nev any  15  24
  DONTA2   6     m   25   84 Eu:SE/Balkn  1960  1984    Pr   Inc CB:diagnosed   0 Current       Cigs  Nev cigs  20  29
  FERRI1 142     m   25   74      Am:USA  1961  1971    CS  Prev  CB:symptoms   1 Current       Cigs   Nev any  11  20
  FERRI1 101     f   25   74      Am:USA  1961  1971    CS  Prev  CB:symptoms   1 Current       Cigs   Nev any  11  20
  HARRIS   5     m   15   60      Africa     *  1993    CS  Prev  CB:symptoms   0 Current        Any   Nev any  15  24
  HOLLA2   5     m   40   59      Am:USA  1962  1965    CS  Prev  CB:symptoms   0 Current        Any   Nev any  15  24
  HOLLNA   5     m   40   40    Eu:Scand  1976  1983    CS  Prev  CB:symptoms   0 Current        Any   Nev any  15  24
  HOLLNA  13     f   40   40    Eu:Scand  1976  1983    CS  Prev  CB:symptoms   0 Current        Any   Nev any  15  24
  HUHTI1  46 x   m   40   64    Eu:Scand  1961  1965    CS  Prev  CB:symptoms   0 Current       Cigs   Nev any  15  24
  HUHTI3  24 x   m   25   69    Eu:Scand  1968  1978    CS  Prev  CB:symptoms   0 Current        Any   Nev any  15  24
   KAHN2   4     m   31   84      Am:USA  1954  1966    Pr   Inc CB:mortality   1 Current       Cigs   Nev any  10  20
  LAMBER   8 x   m   35   69       Eu:UK  1965  1970    CS  Prev  CB:symptoms   0 Current       Cigs  Nev cigs  20  20
  LAMBER  56 x   f   35   69       Eu:UK  1965  1970    CS  Prev  CB:symptoms   0 Current       Cigs   Nev any  20  20
  MUELLE  67 x   f   20   69      Am:USA  1967  1971    CS  Prev  CB:symptoms   0 Current       Cigs   Nev any  15  24
  OGILVI  17     m   30   99       Eu:UK  1955  1957    CC  Prev  CB:symptoms   0 Current       Cigs   Nev any  16  25
  OGILVI  51     f   30   99       Eu:UK  1955  1957    CC  Prev  CB:symptoms   0    Ever       Cigs   Nev any  16  25
    REID   8 x   m   35   74      Am:USA  1962  1966    CS  Prev  CB:symptoms   0 Current       Cigs  Nev cigs  20  20
    REID  24 x   f   35   74      Am:USA  1962  1966    CS  Prev  CB:symptoms   0 Current       Cigs  Nev cigs  20  20
  TROISI   5     f   34   69      Am:USA  1980  1995    Pr   Inc CB:diagnosed   1 Current       Cigs  Nev cigs  15  25
  URRUTI   5     b   20   44     Eu:West     *  2005    CS  Prev  CB:symptoms   0 Current       Cigs  Nev cigs  10  20
  WAGEN2   5     b   21   68     Eu:West  2001  2004    CC  Prev  CB:symptoms   0    Ever       Cigs  Nev cigs  11  20
     WEN   9     m   35   99   Asia:FarE  1982  2004    Pr   Inc CB:mortality   1 Current       Cigs  Nev cigs  11  20
   WOOLF   5     f   25   54   Am:Canada     *  1974    CS  Prev  CB:symptoms   0 Current       Cigs  Nev cigs  11  20


  ________________________________________________________________________________________________________________________
                                            International Evidence on Smoking and COPD, Phase 3, Analysis run on 28-SEP-10

                                                   Table 2 - E - 2 - 5

                               IESCOPD - Meta-analysis of amount smoked : key value (1) 20
                             Any CB, cigarettes (or any product if cigarettes not available)
                                                      Least-adjusted


                        Number Exposed  Non-exposed
 REF    NRR SEX ADJ     Case    Cont    Case    Cont      RR        95.00%CI
 ALDERS 12  m   1         72       -      31       -      1.96 (  1.15-   3.34)
 ALDERS 17  f   1        125       -     111       -      3.12 (  2.17-   4.49)
 Subtotal ALDERS                                          2.69 (  1.99-   3.64)
 ANDER1 41  m   1         16       -       4       -      7.03 (  2.11-  23.42)
 ANDER1 46  f   1          5       -      15       -      2.56 (  0.83-   7.87)
 Subtotal ANDER1                                          4.10 (  1.80-   9.32)
*BEST   5   m   1         49       -       3       -     13.65 (  4.26-  43.78)
 CHAPMA 5   m   0         72     361      35     989      5.64 (  3.70-   8.59)
 CHAPMA 13  f   0         48     313      48    1857      5.93 (  3.91-   9.01)
 Subtotal CHAPMA                                          5.78 (  4.30-   7.78)
 COATES 10  b   0         69     333      21     515      5.08 (  3.06-   8.44)
 DEAN2  12  m   1         61       -      39       -      2.57 (  1.70-   3.89)
 DEAN2  26  f   1         69       -      80       -      4.59 (  3.29-   6.42)
 Subtotal DEAN2                                           3.65 (  2.81-   4.73)
*DOLL1  17  m   1         37       -       3       -     12.80 (  3.74-  43.85)
*DONTA2 6   m   0         19      67       8     127      4.50 (  2.08-   9.73)
 FERRI1 142 m   1         44       -      10       -      3.25 (  1.53-   6.91)
 FERRI1 101 f   1         17       -      35       -      2.75 (  1.45-   5.22)
 Subtotal FERRI1                                          2.95 (  1.81-   4.81)
 HARRIS 5   m   0          3       2       6     518    129.50 ( 18.21- 921.07)
 HOLLA2 5   m   0         38     131       5      84      4.87 (  1.84-  12.88)
 HOLLNA 5   m   0         72      89      10     101      8.17 (  3.98-  16.79)
 HOLLNA 13  f   0         42      86      14     217      7.57 (  3.93-  14.56)
 Subtotal HOLLNA                                          7.84 (  4.83-  12.72)
 HUHTI1 46  m   0         79     112       7     115     11.59 (  5.13-  26.19)
 HUHTI3 24  m   0         79     122      28     211      4.88 (  3.00-   7.93)
*KAHN2  4   m   1         34       -      13       -      4.34 (  2.29-   8.22)
 LAMBER 8   m   0         41     540      17     642      2.87 (  1.61-   5.11)
 LAMBER 56  f   0         29     305      54    2390      4.21 (  2.64-   6.71)
 Subtotal LAMBER                                          3.62 (  2.52-   5.20)
 MUELLE 67  f   0         14      43       3     168     18.23 (  5.01-  66.31)
 OGILVI 17  m   0         66      44      11      29      3.95 (  1.79-   8.73)
 OGILVI 51  f   0         24      13      86     160      3.43 (  1.67-   7.08)
 Subtotal OGILVI                                          3.66 (  2.15-   6.25)
 REID   8   m   0         66    3484      16    4127      4.89 (  2.82-   8.45)
 REID   24  f   0         19    1881      37   10430      2.85 (  1.63-   4.96)
 Subtotal REID                                            3.74 (  2.53-   5.53)
*TROISI 5   f   1          -       -       -       -      2.63 (  2.13-   3.13)
 URRUTI 5   b   0         67     540      16     707      5.48 (  3.14-   9.57)
 WAGEN2 5   b   0        111    1084     111    2136      1.97 (  1.50-   2.59)
*WEN    9   m   1          -       -       7       -      2.03 (  0.57-   7.23)
 WOOLF  5   f   0         25      67      10     173      6.46 (  2.94-  14.16)
Partial Totals          1512    9617     894   25696
*prospective study


 REF    NRR SEX ADJ             Ys       Ws       Qs       Ps
 ALDERS 12  m   1              0.67    13.52     5.00       0.01
 ALDERS 17  f   1              1.14    29.06     0.60       0.00
 Subtotal ALDERS               0.99    42.58     5.60
 ANDER1 41  m   1              1.95     2.65     1.19       0.00
 ANDER1 46  f   1              0.94     3.04     0.35       0.10
 Subtotal ANDER1               1.41     5.69     1.54
*BEST   5   m   1              2.61     2.83     5.03       0.00
 CHAPMA 5   m   0              1.73    21.63     4.34       0.00
 CHAPMA 13  f   0              1.78    22.03     5.49       0.00
 Subtotal CHAPMA               1.76    43.65     9.83
 COATES 10  b   0              1.63    14.91     1.77       0.00
 DEAN2  12  m   1              0.94    22.42     2.55       0.00
 DEAN2  26  f   1              1.52    34.38     2.02       0.00
 Subtotal DEAN2                1.29    56.81     4.58
*DOLL1  17  m   1              2.55     2.54     4.08       0.00
*DONTA2 6   m   0              1.50     6.46     0.32       0.00
 FERRI1 142 m   1              1.18     6.76     0.07       0.00
 FERRI1 101 f   1              1.01     9.36     0.68       0.00
 Subtotal FERRI1               1.08    16.12     0.75
 HARRIS 5   m   0              4.86     1.00    12.81       0.00
 HOLLA2 5   m   0              1.58     4.07     0.37       0.00
 HOLLNA 5   m   0              2.10     7.41     4.97       0.00
 HOLLNA 13  f   0              2.02     8.97     4.95       0.00
 Subtotal HOLLNA               2.06    16.38     9.92
  ________________________________________________________________________________________________________________________
                                            International Evidence on Smoking and COPD, Phase 3, Analysis run on 28-SEP-10

                                                   Table 2 - E - 2 - 5

                               IESCOPD - Meta-analysis of amount smoked : key value (1) 20
                             Any CB, cigarettes (or any product if cigarettes not available)
                                                      Least-adjusted


 REF    NRR SEX ADJ             Ys       Ws       Qs       Ps
 HUHTI1 46  m   0              2.45     5.78     7.89       0.00
 HUHTI3 24  m   0              1.59    16.31     1.51       0.00
*KAHN2  4   m   1              1.47     9.41     0.33       0.00
 LAMBER 8   m   0              1.05    11.54     0.60       0.00
 LAMBER 56  f   0              1.44    17.64     0.43       0.00
 Subtotal LAMBER               1.29    29.18     1.03
 MUELLE 67  f   0              2.90     2.30     6.06       0.00
 OGILVI 17  m   0              1.37     6.12     0.05       0.00
 OGILVI 51  f   0              1.23     7.33     0.02       0.00
 Subtotal OGILVI               1.30    13.45     0.07
 REID   8   m   0              1.59    12.79     1.19       0.00
 REID   24  f   0              1.05    12.46     0.69       0.00
 Subtotal REID                 1.32    25.25     1.88
*TROISI 5   f   1              0.97   103.71    10.25       0.00
 URRUTI 5   b   0              1.70    12.39     2.19       0.00
 WAGEN2 5   b   0              0.68    51.52    18.74       0.00
*WEN    9   m   1              0.71     2.38     0.78       0.27
 WOOLF  5   f   0              1.86     6.22     2.12       0.00

                       N       33
                      NS       24


                      Wt   490.94
                 Het Chi   109.42
                 Het  df       32
                 Het  P       ***
               Fixed  RR     3.60
                     RRl     3.30
                     RRu     3.93
                      P       +++
              Random  RR     4.37
                     RRl     3.63
                     RRu     5.25
                      P       +++
               Asymm  P       ***


  ________________________________________________________________________________________________________________________
                                            International Evidence on Smoking and COPD, Phase 3, Analysis run on 28-SEP-10

                                                   Table 2 - E - 2 - 6

                               IESCOPD - Meta-analysis of amount smoked : key value (1) 20
                             Any CB, cigarettes (or any product if cigarettes not available)
                                                      Least-adjusted


                       N       33
                      NS       24


                      Wt   490.94
                 Het Chi   109.42
                 Het  df       32
                 Het  P       ***
               Fixed  RR     3.60
                     RRl     3.30
                     RRu     3.93
                      P       +++
              Random  RR     4.37
                     RRl     3.63
                     RRu     5.25
                      P       +++
               Asymm  P       ***

                                   Sex
                             both      male    female     Total


                       N        3        18        12        33
                      NS        3        18        12        33


                      Wt    78.83    155.61    256.50    490.94
                 Het Chi    17.24     47.96     33.44    109.42
                 Het  df        2        17        11        32
                 Het  P       ***       ***       ***       ***
               Fixed  RR     2.77      4.32      3.50      3.60
                     RRl     2.22      3.69      3.09      3.30
                     RRu     3.45      5.05      3.95      3.93
                      P       +++       +++       +++       +++
              Random  RR     3.69      4.88      4.06      4.37
                     RRl     1.76      3.67      3.16      3.63
                     RRu     7.76      6.48      5.22      5.25
                      P       +++       +++       +++       +++
             Between Chi                                  10.79
             Between  df                                      2
             Between  P                                      **
             Btwn(F)  P                                    N.S.

                                        Continent
                            NAmer    Europe      Asia  oth/mult     Total


                       N       15        16         1         1        33
                      NS       11        11         1         1        24


                      Wt   234.17    253.39      2.38      1.00    490.94
                 Het Chi    39.77     55.66      0.00      0.00    109.42
                 Het  df       14        15         0         0        32
                 Het  P       ***       ***      N.S.      N.S.       ***
               Fixed  RR     3.69      3.49      2.03    129.50      3.60
                     RRl     3.25      3.08      0.57     18.21      3.30
                     RRu     4.20      3.95      7.23    921.07      3.93
                      P       +++       +++      N.S.       +++       +++
              Random  RR     4.54      4.10      2.03    129.50      4.37
                     RRl     3.50      3.18      0.57     18.21      3.63
                     RRu     5.90      5.29      7.23    921.07      5.25
                      P       +++       +++      N.S.       +++       +++
             Between Chi                                            13.99
             Between  df                                                3
             Between  P                                                **
             Btwn(F)  P                                              N.S.


  ________________________________________________________________________________________________________________________
                                            International Evidence on Smoking and COPD, Phase 3, Analysis run on 28-SEP-10

                                                   Table 2 - E - 2 - 6

                               IESCOPD - Meta-analysis of amount smoked : key value (1) 20
                             Any CB, cigarettes (or any product if cigarettes not available)
                                                      Least-adjusted
                               Study type
                               CC        Pr        CS     Total


                       N        5         6        22        33
                      NS        3         6        15        24


                      Wt   107.56    127.33    256.06    490.94
                 Het Chi     7.03     16.34     46.69    109.42
                 Het  df        4         5        21        32
                 Het  P      N.S.        **        **       ***
               Fixed  RR     2.41      2.99      4.68      3.60
                     RRl     1.99      2.51      4.14      3.30
                     RRu     2.91      3.55      5.29      3.93
                      P       +++       +++       +++       +++
              Random  RR     2.55      4.57      4.91      4.37
                     RRl     1.92      2.66      4.03      3.63
                     RRu     3.38      7.85      5.97      5.25
                      P       +++       +++       +++       +++
             Between Chi                                  39.36
             Between  df                                      2
             Between  P                                     ***
             Btwn(F)  P                                      **

                           CB subtype
                             mort     sympt     other     Total


                       N        4        25         4        33
                      NS        4        17         3        24


                      Wt    17.15    321.04    152.75    490.94
                 Het Chi     7.04     78.96      3.75    109.42
                 Het  df        3        24         3        32
                 Het  P       (*)       ***      N.S.       ***
               Fixed  RR     5.54      4.03      2.71      3.60
                     RRl     3.45      3.61      2.31      3.30
                     RRu     8.89      4.50      3.17      3.93
                      P       +++       +++       +++       +++
              Random  RR     6.08      4.63      2.74      4.37
                     RRl     2.73      3.74      2.23      3.63
                     RRu    13.50      5.74      3.36      5.25
                      P       +++       +++       +++       +++
             Between Chi                                  19.67
             Between  df                                      2
             Between  P                                     ***
             Btwn(F)  P                                     (*)

                             Smoking product
                              any      cigs  cigsonly     Total


                       N        5        22         6        33
                      NS        4        16         4        24


                      Wt    37.75    348.44    104.75    490.94
                 Het Chi    11.21     64.62     19.07    109.42
                 Het  df        4        21         5        32
                 Het  P         *       ***        **       ***
               Fixed  RR     6.53      3.42      3.45      3.60
                     RRl     4.75      3.08      2.85      3.30
                     RRu     8.99      3.80      4.17      3.93
                      P       +++       +++       +++       +++
              Random  RR     7.74      4.09      3.82      4.37
                     RRl     4.27      3.31      2.51      3.63
                     RRu    14.05      5.06      5.83      5.25
                      P       +++       +++       +++       +++
             Between Chi                                  14.52
             Between  df                                      2
             Between  P                                     ***
             Btwn(F)  P                                    N.S.
  ________________________________________________________________________________________________________________________
                                            International Evidence on Smoking and COPD, Phase 3, Analysis run on 28-SEP-10

                                                   Table 2 - E - 2 - 6

                               IESCOPD - Meta-analysis of amount smoked : key value (1) 20
                             Any CB, cigarettes (or any product if cigarettes not available)
                                                      Least-adjusted
                                     Unexposed group
                          nev any   nev cig  nev+ any  nev+ cig     Total


                       N       21        12                            33
                      NS       15        10                            25


                      Wt   212.90    278.05                        490.94
                 Het Chi    56.56     45.10                        109.42
                 Het  df       20        11                            32
                 Het  P       ***       ***                           ***
               Fixed  RR     4.16      3.23                          3.60
                     RRl     3.64      2.87                          3.30
                     RRu     4.76      3.63                          3.93
                      P       +++       +++                           +++
              Random  RR     4.79      3.86                          4.37
                     RRl     3.74      2.94                          3.63
                     RRu     6.15      5.07                          5.25
                      P       +++       +++                           +++
             Between Chi                                             7.76
             Between  df                                                1
             Between  P                                                **
             Btwn(F)  P                                              N.S.

                        Unexposed group (combining nev+ with main levels)
                          nev any   nev cig     Total


                       N       21        12        33
                      NS       15        10        25


                      Wt   212.90    278.05    490.94
                 Het Chi    56.56     45.10    109.42
                 Het  df       20        11        32
                 Het  P       ***       ***       ***
               Fixed  RR     4.16      3.23      3.60
                     RRl     3.64      2.87      3.30
                     RRu     4.76      3.63      3.93
                      P       +++       +++       +++
              Random  RR     4.79      3.86      4.37
                     RRl     3.74      2.94      3.63
                     RRu     6.15      5.07      5.25
                      P       +++       +++       +++
             Between Chi                         7.76
             Between  df                            1
             Between  P                            **
             Btwn(F)  P                          N.S.


  ________________________________________________________________________________________________________________________
                                            International Evidence on Smoking and COPD, Phase 3, Analysis run on 28-SEP-10

                                                   Table 2 - E - 2 - 7

                               IESCOPD - Meta-analysis of amount smoked : key value (1) 20
                             Any CB, cigarettes (or any product if cigarettes not available)
                                 Excluded studies (and stage at which they were excluded)


1       CLARK COTTON  MEYER REMYJA RUTGER SNYDER SOBRAX     SU TAKEMU  WANG4   WEIR WHICKE ZALACA
2      ALESSA  AMIGO ANDER2 ANDER3 AUERBA BEDNAR BROGGE  CHEN1  CHEN2  CHEN3  CHENG CLEMEN  COCCI  DEAN1 DEJONG DETORR
       DICKIN DONTA1 EKBERG ENSTRO FERRI2 FERRI3  FIDAN FORAST FUKUCH GEIJER GODTFR GULSVI HAMMO2 HARIKK HEDMAN HIGGI4
       HOZAWA ITABAS JACOBS JAENDI JOHANN KACHEL KARAKA KATANC KHOURY    KIM KLAYTO KOJIMA KOTAN2 KRZYZA KULLER    LAI
         LAM1   LAM2   LAM3  LANGE    LEE   LIAW LINDBE   LIU1   LIU2 LUNDB1  MADOR MANNI1 MANNI2 MANNI3 MARAN1 MARAN2
       MARCUS MATHES MENEZ2 MENEZ3 MENEZ4 MENEZ5 MENEZ6 MONTNE   NAWA NIEPSU NIHLEN NILSSO  OMORI   PEAT   PETO  PRATT
        PRICE RENWIC RICCIO  RYDER SARGEA SHAHAB   SHIN SICHLE SPEIZE STERLI  STROM SUTINE  TAGER   TANG   THUN   TODD
       TRUPIN TSUSHI TVERDA VESTBO VIEGI2 VIKGRE VINEIS VOLLM1 VOLLM2 VONHER   WALD  WANG2 WATSON  WEISS WILSO1   XIAO
           XU   YUAN ZIELI1 ZIELI2 ZIETKO
3        KAHN
4        BANG  BECK1  BECK2 BJORNS CERVER COLLEG  DEANE DEMARC DOPICO ENRIGH FINKLE FOXMAN GOLDBE HARDIE  HAYES HIRAYA
           HO  HOUSE HUHTI2 JENSEN JINDA2  JOSHI JOUSI1   KATO  KIRAZ KOTAN1  KUBIK LANGE2 LANGHA LUNDB2 MAGNUS MANFRE
       MELLST MILLER MOLLER NEJJAR OSWAL1 OSWAL2 PANDEY PELKON PEREZP SAWICK SCHWAR SHIMUR  SILVA SOBRAD STJERN SUADIC
       TAGER2 VIEGI1 WOJTYN  WOODS   ZOIA
5       DOLL2 FLETCH HAWTHO HRUBEC RIMING  SHARP    WIG WILSO2
6       BROWN EHRLIC HAENSZ HIGGI2 HIGGI3 HIGGI6 HUCHON LAVECC LEBOWI LINDST MENEZ1  MEREN  MILNE WILHEL YAMAGU


  ________________________________________________________________________________________________________________________
                                            International Evidence on Smoking and COPD, Phase 3, Analysis run on 28-SEP-10

                                                   Table 2 - E - 2 - 8

                               IESCOPD - Meta-analysis of amount smoked : key value (1) 20
                             Any CB, cigarettes (or any product if cigarettes not available)
                                             Potentially overlapping studies


     REF| REFGP|PRINC|                     OVERLAP|

  URRUTI DEMARC     2         DEMARC/URRUTI/DEMEER
  DONTA2 JACOBS     2  JACOBS/DONTA1/DONTA2/PELKON
  HUHTI1 HUHTI1     1                HUHTI1/HUHTI2
  FERRI1 FERRIS     2         FERRI1/FERRI2/FERRI3
     WEN    WEN     1                     WEN/LIAW
  LAMBER   TODD     2                  LAMBER/TODD
  CHAPMA  HOUSE     2                 HOUSE/CHAPMA
  HOLLNA HOLLNA     1 GODT/VEST/LANG1+2/SUAD/HOLLN
   KAHN2   KAHN     2                   KAHN/KAHN2


  ________________________________________________________________________________________________________________________
                                            International Evidence on Smoking and COPD, Phase 3, Analysis run on 28-SEP-10

                                                    Table 2 - E - 3 -

                               IESCOPD - Meta-analysis of amount smoked : key value (1) 45
                             Any CB, cigarettes (or any product if cigarettes not available)


This analysis is restricted to results for:
1) Eligible study on database
2) Outcome CB
3) Current or ever smoking
4) Categorical dose-response data for amount smoked
5) vs never smoking base
6) Key value (scheme 1) = 45
7) Results complete enough for use in meta-analysis

Within each study, results are then selected (in the following order of preference, within each sex) for:
8) SMKSTA  : current, ever
9) UNEXP   : never any, never cigarettes
10) PROD    : cigarettes, cigarettes only, any product
11) For overlapping studies: principal rather than subsidiary studies
and then for single sex results (m, f) in preference to results for both sexes combined (b).

Results adjusted for the most potential confounders are then chosen in Sections -1 to -3
and results adjusted for the least confounders in Sections -4 to -6. (Those least-adjusted results which
actually differ from the most-adjusted are marked 'x' in column X in Section -4)

Section -7 shows excluded studies, together with the stage (as above) at which no qualifying
results were found.

Section -8 lists the potentially overlapping studies which have been included (1=principal, 2=subsidiary),
and any results which would have been included in preference except that they had data not complete enough
for use in meta-analysis. It also lists their significance (yes/no), if known.


  ________________________________________________________________________________________________________________________
                                            International Evidence on Smoking and COPD, Phase 3, Analysis run on 28-SEP-10

                                                   Table 2 - E - 3 - 1

                               IESCOPD - Meta-analysis of amount smoked : key value (1) 45
                             Any CB, cigarettes (or any product if cigarettes not available)
                                                      Most-adjusted


     REF|NRR|SEX|AGEL|AGEH|     REGION|BEGYR|PUBYR|STTYP|ONSET|      DISEAS|ADJ|SMOKSTA|   PRODUCT|    UNEXP|LOW| HI|

  ALDERS  13   m   35   74       Eu:UK  1977  1985    CC  Prev CB:diagnosed   1    Ever MCigs only   Nev any  28   +
  ALDERS  18   f   35   74       Eu:UK  1977  1985    CC  Prev CB:diagnosed   1    Ever MCigs only   Nev any  28   +
  ANDER1  42   m   25   74   Am:Canada  1963  1965    CS  Prev  CB:symptoms   1 Current       Cigs   Nev any  25   +
  ANDER1  47   f   25   74   Am:Canada  1963  1965    CS  Prev  CB:symptoms   1 Current       Cigs   Nev any  25   +
    BEST   6   m   30   97   Am:Canada  1955  1967    Pr   Inc CB:mortality   1 Current  Cigs only   Nev any  21   +
  CHAPMA  22   m   15   99      Am:USA  1976  1985    CS  Prev  CB:symptoms   1 Current       Cigs  Nev cigs  21   +
  CHAPMA  30   f   15   99      Am:USA  1976  1985    CS  Prev  CB:symptoms   1 Current       Cigs  Nev cigs  21   +
  COATES  11   b   40   64      Am:USA  1962  1965    CS  Prev  CB:symptoms   0 Current       Cigs  Nev cigs  25   +
   DEAN2  15   m   37   67       Eu:UK  1972  1978    CS  Prev  CB:symptoms   1 Current MCigs only   Nev any  33   +
   DEAN2  27   f   37   67       Eu:UK  1972  1978    CS  Prev  CB:symptoms   1 Current MCigs only   Nev any  23   +
   DOLL1  18   m   20   99       Eu:UK  1951  1994    Pr   Inc CB:mortality   1 Current  Cigs only   Nev any  25   +
  DONTA2   7   m   25   84 Eu:SE/Balkn  1960  1984    Pr   Inc CB:diagnosed   0 Current       Cigs  Nev cigs  30   +
  FERRI1 145   m   25   74      Am:USA  1961  1971    CS  Prev  CB:symptoms   1 Current       Cigs   Nev any  41   +
  FERRI1 102   f   25   74      Am:USA  1961  1971    CS  Prev  CB:symptoms   1 Current       Cigs   Nev any  21   +
  HARRIS   6   m   15   60      Africa     *  1993    CS  Prev  CB:symptoms   0 Current        Any   Nev any  25   +
  HOLLA2   6   m   40   59      Am:USA  1962  1965    CS  Prev  CB:symptoms   0 Current        Any   Nev any  25   +
  HOLLNA   6   m   40   40    Eu:Scand  1976  1983    CS  Prev  CB:symptoms   0 Current        Any   Nev any  25   +
  HOLLNA  14   f   40   40    Eu:Scand  1976  1983    CS  Prev  CB:symptoms   0 Current        Any   Nev any  25   +
  HUCHON  24   b   25   99     Eu:West     *  2002    CS  Prev  CB:symptoms   1 Current       Cigs  Nev cigs  21   +
  HUHTI1  52   m   40   64    Eu:Scand  1961  1965    CS  Prev  CB:symptoms   1 Current       Cigs   Nev any  25   +
  HUHTI3  30   m   25   69    Eu:Scand  1968  1978    CS  Prev  CB:symptoms   1 Current        Any   Nev any  25   +
   KAHN2   6   m   31   84      Am:USA  1954  1966    Pr   Inc CB:mortality   1 Current       Cigs   Nev any  40   +
  LAMBER  14   m   35   69       Eu:UK  1965  1970    CS  Prev  CB:symptoms   1 Current       Cigs  Nev cigs  21   +
  LAMBER  60   f   35   69       Eu:UK  1965  1970    CS  Prev  CB:symptoms   1 Current       Cigs   Nev any  21   +
  MUELLE  45   m   20   69      Am:USA  1967  1971    CS  Prev  CB:symptoms   1 Current       Cigs   Nev any  25   +
  MUELLE  73   f   20   69      Am:USA  1967  1971    CS  Prev  CB:symptoms   1 Current       Cigs   Nev any  25   +
  OGILVI  18   m   30   99       Eu:UK  1955  1957    CC  Prev  CB:symptoms   0 Current       Cigs   Nev any  26   +
  OGILVI  52   f   30   99       Eu:UK  1955  1957    CC  Prev  CB:symptoms   0    Ever       Cigs   Nev any  26   +
    REID  14   m   35   74      Am:USA  1962  1966    CS  Prev  CB:symptoms   1 Current       Cigs  Nev cigs  21   +
    REID  30   f   35   74      Am:USA  1962  1966    CS  Prev  CB:symptoms   1 Current       Cigs  Nev cigs  21   +
  TROISI   6   f   34   69      Am:USA  1980  1995    Pr   Inc CB:diagnosed   1 Current       Cigs  Nev cigs  25   +
  URRUTI   6   b   20   44     Eu:West     *  2005    CS  Prev  CB:symptoms   0 Current       Cigs  Nev cigs  21   +
  WAGEN2   6   b   21   68     Eu:West  2001  2004    CC  Prev  CB:symptoms   0    Ever       Cigs  Nev cigs  21   +
     WEN  10   m   35   99   Asia:FarE  1982  2004    Pr   Inc CB:mortality   1 Current       Cigs  Nev cigs  21   +
   WOOLF   6   f   25   54   Am:Canada     *  1974    CS  Prev  CB:symptoms   0 Current       Cigs  Nev cigs  21   +


  ________________________________________________________________________________________________________________________
                                            International Evidence on Smoking and COPD, Phase 3, Analysis run on 28-SEP-10

                                                   Table 2 - E - 3 - 2

                               IESCOPD - Meta-analysis of amount smoked : key value (1) 45
                             Any CB, cigarettes (or any product if cigarettes not available)
                                                      Most-adjusted


                        Number Exposed  Non-exposed
 REF    NRR SEX ADJ     Case    Cont    Case    Cont      RR        95.00%CI
 ALDERS 13  m   1        148       -      31       -      2.75 (  1.68-   4.50)
 ALDERS 18  f   1        104       -     111       -      4.53 (  2.98-   6.88)
 Subtotal ALDERS                                          3.68 (  2.67-   5.06)
 ANDER1 42  m   1         14       -       4       -      7.54 (  2.21-  25.76)
 ANDER1 47  f   1          7       -      15       -      7.99 (  2.60-  24.59)
 Subtotal ANDER1                                          7.78 (  3.40-  17.83)
*BEST   6   m   1         12       -       3       -     14.63 (  4.13-  51.82)
 CHAPMA 22  m   1        102       -      35       -     12.31 (  8.07-  18.78)
 CHAPMA 30  f   1         61       -      48       -     17.79 ( 11.67-  27.13)
 Subtotal CHAPMA                                         14.80 ( 10.98-  19.95)
 COATES 11  b   0         43     127      21     515      8.30 (  4.76-  14.49)
 DEAN2  15  m   1         23       -      39       -      3.80 (  2.21-   6.53)
 DEAN2  27  f   1         34       -      80       -      5.72 (  3.75-   8.73)
 Subtotal DEAN2                                           4.90 (  3.51-   6.84)
*DOLL1  18  m   1         33       -       3       -     21.20 (  6.16-  72.97)
*DONTA2 7   m   0          5      14       8     127      5.67 (  2.15-  14.98)
 FERRI1 145 m   1         10       -      10       -     19.04 (  4.59-  79.02)
 FERRI1 102 f   1         13       -      35       -      3.62 (  1.73-   7.56)
 Subtotal FERRI1                                          5.14 (  2.67-   9.90)
 HARRIS 6   m   0          2       1       6     518    172.67 ( 13.73-2171.40)
 HOLLA2 6   m   0         88     130       5      84     11.37 (  4.43-  29.17)
 HOLLNA 6   m   0         25      33      10     101      7.65 (  3.33-  17.58)
 HOLLNA 14  f   0          5       9      14     217      8.61 (  2.54-  29.15)
 Subtotal HOLLNA                                          7.94 (  4.00-  15.80)
 HUCHON 24  b   1        145       -     177       -      7.15 (  5.60-   9.14)
 HUHTI1 52  m   1         34       -       7       -      9.52 (  3.83-  23.66)
 HUHTI3 30  m   1         44       -      28       -      8.86 (  4.47-  17.55)
*KAHN2  6   m   1          5       -      13       -      6.91 (  2.46-  19.38)
 LAMBER 14  m   1         31       -      17       -      2.87 (  1.50-   5.48)
 LAMBER 60  f   1          7       -      54       -      6.33 (  2.63-  15.23)
 Subtotal LAMBER                                          3.79 (  2.25-   6.39)
 MUELLE 45  m   1         21       -       2       -     16.01 (  3.52-  72.95)
 MUELLE 73  f   1          9       -       3       -     40.86 (  7.00- 238.48)
 Subtotal MUELLE                                         23.83 (  7.55-  75.24)
 OGILVI 18  m   0         21      12      11      29      4.61 (  1.71-  12.45)
 OGILVI 52  f   0          9       3      86     160      5.58 (  1.47-  21.16)
 Subtotal OGILVI                                          4.94 (  2.23-  10.94)
 REID   14  m   1         61       -      16       -      8.44 (  4.65-  15.29)
 REID   30  f   1         25       -      37       -     10.56 (  5.95-  18.73)
 Subtotal REID                                            9.48 (  6.27-  14.33)
*TROISI 6   f   1          -       -       -       -      4.63 (  3.88-   5.50)
 URRUTI 6   b   0         54     160      16     707     14.91 (  8.32-  26.73)
 WAGEN2 6   b   0         53     521     111    2136      1.96 (  1.39-   2.75)
*WEN    10  m   1          -       -       7       -      3.01 (  0.33-  27.60)
 WOOLF  6   f   0         57      66      10     173     14.94 (  7.20-  30.98)
Partial Totals          1305    1076    1073    4767
*prospective study


  ________________________________________________________________________________________________________________________
                                            International Evidence on Smoking and COPD, Phase 3, Analysis run on 28-SEP-10

                                                   Table 2 - E - 3 - 2

                               IESCOPD - Meta-analysis of amount smoked : key value (1) 45
                             Any CB, cigarettes (or any product if cigarettes not available)
                                                      Most-adjusted


 REF    NRR SEX ADJ             Ys       Ws       Qs       Ps
 ALDERS 13  m   1              1.01    15.83     9.63       0.00
 ALDERS 18  f   1              1.51    21.95     1.73       0.00
 Subtotal ALDERS               1.30    37.78    11.37
 ANDER1 42  m   1              2.02     2.55     0.13       0.00
 ANDER1 47  f   1              2.08     3.04     0.25       0.00
 Subtotal ANDER1               2.05     5.59     0.38
*BEST   6   m   1              2.68     2.40     1.91       0.00
 CHAPMA 22  m   1              2.51    21.54    11.12       0.00
 CHAPMA 30  f   1              2.88    21.59    25.51       0.00
 Subtotal CHAPMA               2.69    43.13    36.63
 COATES 11  b   0              2.12    12.39     1.31       0.00
 DEAN2  15  m   1              1.34    13.09     2.73       0.00
 DEAN2  27  f   1              1.74    21.52     0.05       0.00
 Subtotal DEAN2                1.59    34.61     2.78
*DOLL1  18  m   1              3.05     2.51     4.01       0.00
*DONTA2 7   m   0              1.74     4.07     0.01       0.00
 FERRI1 145 m   1              2.95     1.90     2.53       0.00
 FERRI1 102 f   1              1.29     7.07     1.80       0.00
 Subtotal FERRI1               1.64     8.96     4.33
 HARRIS 6   m   0              5.15     0.60     6.76       0.00
 HOLLA2 6   m   0              2.43     4.33     1.77       0.00
 HOLLNA 6   m   0              2.03     5.55     0.33       0.00
 HOLLNA 14  f   0              2.15     2.58     0.34       0.00
 Subtotal HOLLNA               2.07     8.13     0.67
 HUCHON 24  b   1              1.97    64.03     1.97       0.00
 HUHTI1 52  m   1              2.25     4.63     0.99       0.00
 HUHTI3 30  m   1              2.18     8.21     1.25       0.00
*KAHN2  6   m   1              1.93     3.61     0.07       0.00
 LAMBER 14  m   1              1.05     9.15     4.98       0.00
 LAMBER 60  f   1              1.85     4.98     0.01       0.00
 Subtotal LAMBER               1.33    14.14     4.99
 MUELLE 45  m   1              2.77     1.67     1.61       0.00
 MUELLE 73  f   1              3.71     1.23     4.54       0.00
 Subtotal MUELLE               3.17     2.91     6.15
 OGILVI 18  m   0              1.53     3.90     0.27       0.00
 OGILVI 52  f   0              1.72     2.16     0.01       0.01
 Subtotal OGILVI               1.60     6.06     0.28
 REID   14  m   1              2.13    10.84     1.26       0.00
 REID   30  f   1              2.36    11.69     3.73       0.00
 Subtotal REID                 2.25    22.53     5.00
*TROISI 6   f   1              1.53   126.22     8.48       0.00
 URRUTI 6   b   0              2.70    11.28     9.35       0.00
 WAGEN2 6   b   0              0.67    33.04    41.45       0.00
*WEN    10  m   1              1.10     0.78     0.37       0.33
 WOOLF  6   f   0              2.70     7.22     6.01       0.00

                       N       35
                      NS       25


                      Wt   469.17
                 Het Chi   158.29
                 Het  df       34
                 Het  P       ***
               Fixed  RR     6.00
                     RRl     5.48
                     RRu     6.57
                      P       +++
              Random  RR     7.37
                     RRl     5.86
                     RRu     9.28
                      P       +++
               Asymm  P         *


  ________________________________________________________________________________________________________________________
                                            International Evidence on Smoking and COPD, Phase 3, Analysis run on 28-SEP-10

                                                   Table 2 - E - 3 - 3

                               IESCOPD - Meta-analysis of amount smoked : key value (1) 45
                             Any CB, cigarettes (or any product if cigarettes not available)
                                                      Most-adjusted


                       N       35
                      NS       25


                      Wt   469.17
                 Het Chi   158.29
                 Het  df       34
                 Het  P       ***
               Fixed  RR     6.00
                     RRl     5.48
                     RRu     6.57
                      P       +++
              Random  RR     7.37
                     RRl     5.86
                     RRu     9.28
                      P       +++
               Asymm  P         *

                                   Sex
                             both      male    female     Total


                       N        4        19        12        35
                      NS        4        19        12        35


                      Wt   120.74    117.18    231.25    469.17
                 Het Chi    52.98     50.07     52.44    158.29
                 Het  df        3        18        11        34
                 Het  P       ***       ***       ***       ***
               Fixed  RR     5.46      6.76      5.93      6.00
                     RRl     4.56      5.64      5.22      5.48
                     RRu     6.52      8.10      6.75      6.57
                      P       +++       +++       +++       +++
              Random  RR     6.32      7.59      7.64      7.37
                     RRl     2.76      5.43      5.30      5.86
                     RRu    14.48     10.59     11.03      9.28
                      P       +++       +++       +++       +++
             Between Chi                                   2.80
             Between  df                                      2
             Between  P                                    N.S.
             Btwn(F)  P                                    N.S.

                                        Continent
                            NAmer    Europe      Asia  oth/mult     Total


                       N       16        17         1         1        35
                      NS       11        12         1         1        25


                      Wt   239.29    228.50      0.78      0.60    469.17
                 Het Chi    66.43     72.74      0.00      0.00    158.29
                 Het  df       15        16         0         0        34
                 Het  P       ***       ***      N.S.      N.S.       ***
               Fixed  RR     6.99      5.08      3.01    172.67      6.00
                     RRl     6.16      4.46      0.33     13.73      5.48
                     RRu     7.94      5.78     27.53   2171.40      6.57
                      P       +++       +++      N.S.       +++       +++
              Random  RR     9.87      5.61      3.01    172.67      7.37
                     RRl     6.98      4.11      0.33     13.73      5.86
                     RRu    13.96      7.65     27.53   2171.40      9.28
                      P       +++       +++      N.S.       +++       +++
             Between Chi                                            19.12
             Between  df                                                3
             Between  P                                               ***
             Btwn(F)  P                                              N.S.


  ________________________________________________________________________________________________________________________
                                            International Evidence on Smoking and COPD, Phase 3, Analysis run on 28-SEP-10

                                                   Table 2 - E - 3 - 3

                               IESCOPD - Meta-analysis of amount smoked : key value (1) 45
                             Any CB, cigarettes (or any product if cigarettes not available)
                                                      Most-adjusted
                        National cigarette tobacco type (excluding mixed/unkown)
                          blended  virginia     Total


                       N       20        14        34
                      NS       15         9        24


                      Wt   357.47    110.92    468.39
                 Het Chi   118.48     35.63    157.92
                 Het  df       19        13        33
                 Het  P       ***       ***       ***
               Fixed  RR     6.32      5.11      6.01
                     RRl     5.69      4.24      5.49
                     RRu     7.01      6.15      6.58
                      P       +++       +++       +++
              Random  RR     8.33      6.10      7.44
                     RRl     6.13      4.32      5.90
                     RRu    11.34      8.62      9.37
                      P       +++       +++       +++
             Between Chi                         3.80
             Between  df                            1
             Between  P                           (*)
             Btwn(F)  P                          N.S.

                                        Start year of study
                            <1970   1970-79   1980-89   1990-99     2000+   unknown     Total


                       N       20         8         2                   1         4        35
                      NS       14         4         2                   1         4        25


                      Wt   102.35    123.65    127.00               33.04     83.12    469.17
                 Het Chi    26.61     49.45      0.14                0.00     13.20    158.29
                 Het  df       19         7         1                   0         3        34
                 Het  P      N.S.       ***      N.S.                N.S.        **       ***
               Fixed  RR     7.62      6.81      4.62                1.96      8.62      6.00
                     RRl     6.28      5.71      3.88                1.39      6.95      5.48
                     RRu     9.25      8.13      5.49                2.75     10.68      6.57
                      P       +++       +++       +++                 +++       +++       +++
              Random  RR     7.79      6.60      4.62                1.96     13.27      7.37
                     RRl     6.13      4.05      3.88                1.39      6.66      5.86
                     RRu     9.91     10.76      5.49                2.75     26.46      9.28
                      P       +++       +++       +++                 +++       +++       +++
             Between Chi                                                                68.89
             Between  df                                                                    4
             Between  P                                                                   ***
             Btwn(F)  P                                                                    **

                                Publication year
                            <1980   1980-89   1990-99     2000+     Total


                       N       21         7         3         4        35
                      NS       14         4         3         4        25


                      Wt   137.60     93.11    129.33    109.13    469.17
                 Het Chi    33.99     43.25     13.39     50.78    158.29
                 Het  df       20         6         2         3        34
                 Het  P         *       ***        **       ***       ***
               Fixed  RR     6.99      7.64      4.85      5.18      6.00
                     RRl     5.92      6.23      4.08      4.29      5.48
                     RRu     8.27      9.36      5.76      6.25      6.57
                      P       +++       +++       +++       +++       +++
              Random  RR     7.38      7.23     17.82      5.38      7.37
                     RRl     5.84      4.03      3.10      2.04      5.86
                     RRu     9.32     12.98    102.32     14.20      9.28
                      P       +++       +++        ++       +++       +++
             Between Chi                                            16.88
             Between  df                                                3
             Between  P                                               ***
             Btwn(F)  P                                              N.S.
  ________________________________________________________________________________________________________________________
                                            International Evidence on Smoking and COPD, Phase 3, Analysis run on 28-SEP-10

                                                   Table 2 - E - 3 - 3

                               IESCOPD - Meta-analysis of amount smoked : key value (1) 45
                             Any CB, cigarettes (or any product if cigarettes not available)
                                                      Most-adjusted
                               Study type
                               CC        Pr        CS     Total


                       N        5         6        24        35
                      NS        3         6        16        25


                      Wt    76.88    139.60    252.69    469.17
                 Het Chi    11.27      9.37     62.18    158.29
                 Het  df        4         5        23        34
                 Het  P         *       (*)       ***       ***
               Fixed  RR     2.87      4.92      8.38      6.00
                     RRl     2.29      4.17      7.41      5.48
                     RRu     3.59      5.81      9.48      6.57
                      P       +++       +++       +++       +++
              Random  RR     3.21      6.90      8.76      7.37
                     RRl     2.09      4.11      6.96      5.86
                     RRu     4.94     11.60     11.04      9.28
                      P       +++       +++       +++       +++
             Between Chi                                  75.47
             Between  df                                      2
             Between  P                                     ***
             Btwn(F)  P                                     ***

                                    Lowest age in RR
                        <25/unlim     25-39       40+   unknown     Total


                       N        8        22         5                  35
                      NS        6        15         4                  25


                      Wt    93.47    346.21     29.49              469.17
                 Het Chi    99.45     46.13      0.46              158.29
                 Het  df        7        21         4                  34
                 Het  P       ***        **      N.S.                 ***
               Fixed  RR     7.54      5.46      8.78                6.00
                     RRl     6.16      4.91      6.12                5.48
                     RRu     9.24      6.07     12.59                6.57
                      P       +++       +++       +++                 +++
              Random  RR    14.96      5.85      8.78                7.37
                     RRl     6.14      4.81      6.12                5.86
                     RRu    36.47      7.12     12.59                9.28
                      P       +++       +++       +++                 +++
             Between Chi                                            12.24
             Between  df                                                2
             Between  P                                                **
             Btwn(F)  P                                              N.S.

                                         Highest age in RR
                              <65     65-74     75-84 85+/unlim   unknown     Total


                       N        8        17         2         8                  35
                      NS        7        10         2         6                  25


                      Wt    48.59    293.99      7.68    118.92              469.17
                 Het Chi     8.22     62.11      0.07     20.86              158.29
                 Het  df        7        16         1         7                  34
                 Het  P      N.S.       ***      N.S.        **                 ***
               Fixed  RR    11.14      4.51      6.22      9.43                6.00
                     RRl     8.41      4.02      3.07      7.88                5.48
                     RRu    14.76      5.05     12.62     11.29                6.57
                      P       +++       +++       +++       +++                 +++
              Random  RR    11.20      5.35      6.22     10.08                7.37
                     RRl     8.18      4.06      3.07      6.62                5.86
                     RRu    15.33      7.05     12.62     15.34                9.28
                      P       +++       +++       +++       +++                 +++
             Between Chi                                                      67.03
             Between  df                                                          3
             Between  P                                                         ***
             Btwn(F)  P                                                         ***
  ________________________________________________________________________________________________________________________
                                            International Evidence on Smoking and COPD, Phase 3, Analysis run on 28-SEP-10

                                                   Table 2 - E - 3 - 3

                               IESCOPD - Meta-analysis of amount smoked : key value (1) 45
                             Any CB, cigarettes (or any product if cigarettes not available)
                                                      Most-adjusted
                           Study weakness
                              Yes        No     Total


                       N        1        34        35
                      NS        1        24        25


                      Wt     4.07    465.10    469.17
                 Het Chi     0.00    158.28    158.29
                 Het  df        0        33        34
                 Het  P      N.S.       ***       ***
               Fixed  RR     5.67      6.00      6.00
                     RRl     2.15      5.48      5.48
                     RRu    14.98      6.57      6.57
                      P       +++       +++       +++
              Random  RR     5.67      7.43      7.37
                     RRl     2.15      5.88      5.86
                     RRu    14.98      9.39      9.28
                      P       +++       +++       +++
             Between Chi                         0.01
             Between  df                            1
             Between  P                          N.S.
             Btwn(F)  P                          N.S.

                           CB subtype
                             mort     sympt     other     Total


                       N        4        27         4        35
                      NS        4        18         3        25


                      Wt     9.31    291.80    168.07    469.17
                 Het Chi     3.36    124.77      4.10    158.29
                 Het  df        3        26         3        34
                 Het  P      N.S.       ***      N.S.       ***
               Fixed  RR    10.58      7.03      4.42      6.00
                     RRl     5.57      6.27      3.80      5.48
                     RRu    20.12      7.88      5.14      6.57
                      P       +++       +++       +++       +++
              Random  RR    10.57      8.04      4.26      7.37
                     RRl     5.31      6.07      3.40      5.86
                     RRu    21.06     10.66      5.35      9.28
                      P       +++       +++       +++       +++
             Between Chi                                  26.06
             Between  df                                      2
             Between  P                                     ***
             Btwn(F)  P                                     (*)

                           Asthma analysis type (CB)
                        inc-irres  excl-all excl-cntr     Total


                       N       29         6                  35
                      NS       19         6                  25


                      Wt   286.74    182.43              469.17
                 Het Chi    77.13     57.76              158.29
                 Het  df       28         5                  34
                 Het  P       ***       ***                 ***
               Fixed  RR     7.17      4.53                6.00
                     RRl     6.39      3.92                5.48
                     RRu     8.05      5.24                6.57
                      P       +++       +++                 +++
              Random  RR     7.40      7.56                7.37
                     RRl     5.94      3.72                5.86
                     RRu     9.21     15.34                9.28
                      P       +++       +++                 +++
             Between Chi                                  23.40
             Between  df                                      1
             Between  P                                     ***
             Btwn(F)  P                                       *
  ________________________________________________________________________________________________________________________
                                            International Evidence on Smoking and COPD, Phase 3, Analysis run on 28-SEP-10

                                                   Table 2 - E - 3 - 3

                               IESCOPD - Meta-analysis of amount smoked : key value (1) 45
                             Any CB, cigarettes (or any product if cigarettes not available)
                                                      Most-adjusted
                                   Number of CB cases
                             1-50    51-100   101-200      201+     Total


                       N        2         6         6        21        35
                      NS        2         4         6        13        25


                      Wt     1.38     14.97     41.34    411.47    469.17
                 Het Chi     5.57      4.83      4.44    117.00    158.29
                 Het  df        1         5         5        20        34
                 Het  P         *      N.S.      N.S.       ***       ***
               Fixed  RR    17.39      9.82     11.62      5.49      6.00
                     RRl     3.29      5.92      8.57      4.99      5.48
                     RRu    92.05     16.30     15.76      6.05      6.57
                      P       +++       +++       +++       +++       +++
              Random  RR    21.72      9.82     11.62      6.04      7.37
                     RRl     0.41      5.92      8.57      4.62      5.86
                     RRu  1147.44     16.30     15.76      7.88      9.28
                      P      N.S.       +++       +++       +++       +++
             Between Chi                                            26.45
             Between  df                                                3
             Between  P                                               ***
             Btwn(F)  P                                              N.S.

                         Number of CB cases (excluding unknown)


                       N        2         6         6        21        35
                      NS        2         4         6        13        25


                      Wt     1.38     14.97     41.34    411.47    469.17
                 Het Chi     5.57      4.83      4.44    117.00    158.29
                 Het  df        1         5         5        20        34
                 Het  P         *      N.S.      N.S.       ***       ***
               Fixed  RR    17.39      9.82     11.62      5.49      6.00
                     RRl     3.29      5.92      8.57      4.99      5.48
                     RRu    92.05     16.30     15.76      6.05      6.57
                      P       +++       +++       +++       +++       +++
              Random  RR    21.72      9.82     11.62      6.04      7.37
                     RRl     0.41      5.92      8.57      4.62      5.86
                     RRu  1147.44     16.30     15.76      7.88      9.28
                      P      N.S.       +++       +++       +++       +++
             Between Chi                                            26.45
             Between  df                                                3
             Between  P                                               ***
             Btwn(F)  P                                              N.S.

                            Analysis type
                         prevlnce     onset     Total


                       N       29         6        35
                      NS       19         6        25


                      Wt   329.57    139.60    469.17
                 Het Chi   141.12      9.37    158.29
                 Het  df       28         5        34
                 Het  P       ***       (*)       ***
               Fixed  RR     6.53      4.92      6.00
                     RRl     5.86      4.17      5.48
                     RRu     7.27      5.81      6.57
                      P       +++       +++       +++
              Random  RR     7.48      6.90      7.37
                     RRl     5.72      4.11      5.86
                     RRu     9.79     11.60      9.28
                      P       +++       +++       +++
             Between Chi                         7.80
             Between  df                            1
             Between  P                            **
             Btwn(F)  P                          N.S.

  ________________________________________________________________________________________________________________________
                                            International Evidence on Smoking and COPD, Phase 3, Analysis run on 28-SEP-10

                                                   Table 2 - E - 3 - 3

                               IESCOPD - Meta-analysis of amount smoked : key value (1) 45
                             Any CB, cigarettes (or any product if cigarettes not available)
                                                      Most-adjusted
                             Smoking product
                              any      cigs  cigsonly     Total


                       N        5        24         6        35
                      NS        4        17         4        25


                      Wt    21.28    370.59     77.30    469.17
                 Het Chi     5.49    127.52     14.78    158.29
                 Het  df        4        23         5        34
                 Het  P      N.S.       ***         *       ***
               Fixed  RR     9.72      6.16      4.62      6.00
                     RRl     6.36      5.57      3.70      5.48
                     RRu    14.87      6.82      5.77      6.57
                      P       +++       +++       +++       +++
              Random  RR    10.13      7.55      5.19      7.37
                     RRl     5.98      5.65      3.41      5.86
                     RRu    17.14     10.08      7.92      9.28
                      P       +++       +++       +++       +++
             Between Chi                                  10.50
             Between  df                                      2
             Between  P                                      **
             Btwn(F)  P                                    N.S.

                                     Unexposed group
                          nev any   nev cig  nev+ any  nev+ cig     Total


                       N       22        13                            35
                      NS       15        11                            26


                      Wt   135.33    333.84                        469.17
                 Het Chi    42.61    115.51                        158.29
                 Het  df       21        12                            34
                 Het  P        **       ***                           ***
               Fixed  RR     5.82      6.07                          6.00
                     RRl     4.92      5.46                          5.48
                     RRu     6.89      6.76                          6.57
                      P       +++       +++                           +++
              Random  RR     7.01      7.39                          7.37
                     RRl     5.36      5.04                          5.86
                     RRu     9.16     10.81                          9.28
                      P       +++       +++                           +++
             Between Chi                                             0.18
             Between  df                                                1
             Between  P                                              N.S.
             Btwn(F)  P                                              N.S.

                        Unexposed group (combining nev+ with main levels)
                          nev any   nev cig     Total


                       N       22        13        35
                      NS       15        11        26


                      Wt   135.33    333.84    469.17
                 Het Chi    42.61    115.51    158.29
                 Het  df       21        12        34
                 Het  P        **       ***       ***
               Fixed  RR     5.82      6.07      6.00
                     RRl     4.92      5.46      5.48
                     RRu     6.89      6.76      6.57
                      P       +++       +++       +++
              Random  RR     7.01      7.39      7.37
                     RRl     5.36      5.04      5.86
                     RRu     9.16     10.81      9.28
                      P       +++       +++       +++
             Between Chi                         0.18
             Between  df                            1
             Between  P                          N.S.
             Btwn(F)  P                          N.S.
  ________________________________________________________________________________________________________________________
                                            International Evidence on Smoking and COPD, Phase 3, Analysis run on 28-SEP-10

                                                   Table 2 - E - 3 - 3

                               IESCOPD - Meta-analysis of amount smoked : key value (1) 45
                             Any CB, cigarettes (or any product if cigarettes not available)
                                                      Most-adjusted
                        Number of adjustment variables
                                0         1        2+     Total


                       N       11        24                  35
                      NS        9        16                  25


                      Wt    87.13    382.04              469.17
                 Het Chi    66.35     90.39              158.29
                 Het  df       10        23                  34
                 Het  P       ***       ***                 ***
               Fixed  RR     5.32      6.17                6.00
                     RRl     4.31      5.58                5.48
                     RRu     6.56      6.82                6.57
                      P       +++       +++                 +++
              Random  RR     8.14      7.15                7.37
                     RRl     4.43      5.61                5.86
                     RRu    14.97      9.11                9.28
                      P       +++       +++                 +++
             Between Chi                                   1.55
             Between  df                                      1
             Between  P                                    N.S.
             Btwn(F)  P                                    N.S.


  ________________________________________________________________________________________________________________________
                                            International Evidence on Smoking and COPD, Phase 3, Analysis run on 28-SEP-10

                                                   Table 2 - E - 3 - 4

                               IESCOPD - Meta-analysis of amount smoked : key value (1) 45
                             Any CB, cigarettes (or any product if cigarettes not available)
                                                      Least-adjusted


     REF|NRR|X|SEX|AGEL|AGEH|     REGION|BEGYR|PUBYR|STTYP|ONSET|      DISEAS|ADJ|SMOKSTA|   PRODUCT|    UNEXP|LOW| HI|

  ALDERS  13     m   35   74       Eu:UK  1977  1985    CC  Prev CB:diagnosed   1    Ever MCigs only   Nev any  28   +
  ALDERS  18     f   35   74       Eu:UK  1977  1985    CC  Prev CB:diagnosed   1    Ever MCigs only   Nev any  28   +
  ANDER1  42     m   25   74   Am:Canada  1963  1965    CS  Prev  CB:symptoms   1 Current       Cigs   Nev any  25   +
  ANDER1  47     f   25   74   Am:Canada  1963  1965    CS  Prev  CB:symptoms   1 Current       Cigs   Nev any  25   +
    BEST   6     m   30   97   Am:Canada  1955  1967    Pr   Inc CB:mortality   1 Current  Cigs only   Nev any  21   +
  CHAPMA   6 x   m   15   99      Am:USA  1976  1985    CS  Prev  CB:symptoms   0 Current       Cigs  Nev cigs  21   +
  CHAPMA  14 x   f   15   99      Am:USA  1976  1985    CS  Prev  CB:symptoms   0 Current       Cigs  Nev cigs  21   +
  COATES  11     b   40   64      Am:USA  1962  1965    CS  Prev  CB:symptoms   0 Current       Cigs  Nev cigs  25   +
   DEAN2  15     m   37   67       Eu:UK  1972  1978    CS  Prev  CB:symptoms   1 Current MCigs only   Nev any  33   +
   DEAN2  27     f   37   67       Eu:UK  1972  1978    CS  Prev  CB:symptoms   1 Current MCigs only   Nev any  23   +
   DOLL1  18     m   20   99       Eu:UK  1951  1994    Pr   Inc CB:mortality   1 Current  Cigs only   Nev any  25   +
  DONTA2   7     m   25   84 Eu:SE/Balkn  1960  1984    Pr   Inc CB:diagnosed   0 Current       Cigs  Nev cigs  30   +
  FERRI1 145     m   25   74      Am:USA  1961  1971    CS  Prev  CB:symptoms   1 Current       Cigs   Nev any  41   +
  FERRI1 102     f   25   74      Am:USA  1961  1971    CS  Prev  CB:symptoms   1 Current       Cigs   Nev any  21   +
  HARRIS   6     m   15   60      Africa     *  1993    CS  Prev  CB:symptoms   0 Current        Any   Nev any  25   +
  HOLLA2   6     m   40   59      Am:USA  1962  1965    CS  Prev  CB:symptoms   0 Current        Any   Nev any  25   +
  HOLLNA   6     m   40   40    Eu:Scand  1976  1983    CS  Prev  CB:symptoms   0 Current        Any   Nev any  25   +
  HOLLNA  14     f   40   40    Eu:Scand  1976  1983    CS  Prev  CB:symptoms   0 Current        Any   Nev any  25   +
  HUCHON  14 x   b   25   99     Eu:West     *  2002    CS  Prev  CB:symptoms   0 Current       Cigs  Nev cigs  21   +
  HUHTI1  47 x   m   40   64    Eu:Scand  1961  1965    CS  Prev  CB:symptoms   0 Current       Cigs   Nev any  25   +
  HUHTI3  25 x   m   25   69    Eu:Scand  1968  1978    CS  Prev  CB:symptoms   0 Current        Any   Nev any  25   +
   KAHN2   6     m   31   84      Am:USA  1954  1966    Pr   Inc CB:mortality   1 Current       Cigs   Nev any  40   +
  LAMBER   9 x   m   35   69       Eu:UK  1965  1970    CS  Prev  CB:symptoms   0 Current       Cigs  Nev cigs  21   +
  LAMBER  57 x   f   35   69       Eu:UK  1965  1970    CS  Prev  CB:symptoms   0 Current       Cigs   Nev any  21   +
  MUELLE  42 x   m   20   69      Am:USA  1967  1971    CS  Prev  CB:symptoms   0 Current       Cigs   Nev any  25   +
  MUELLE  68 x   f   20   69      Am:USA  1967  1971    CS  Prev  CB:symptoms   0 Current       Cigs   Nev any  25   +
  OGILVI  18     m   30   99       Eu:UK  1955  1957    CC  Prev  CB:symptoms   0 Current       Cigs   Nev any  26   +
  OGILVI  52     f   30   99       Eu:UK  1955  1957    CC  Prev  CB:symptoms   0    Ever       Cigs   Nev any  26   +
    REID   9 x   m   35   74      Am:USA  1962  1966    CS  Prev  CB:symptoms   0 Current       Cigs  Nev cigs  21   +
    REID  25 x   f   35   74      Am:USA  1962  1966    CS  Prev  CB:symptoms   0 Current       Cigs  Nev cigs  21   +
  TROISI   6     f   34   69      Am:USA  1980  1995    Pr   Inc CB:diagnosed   1 Current       Cigs  Nev cigs  25   +
  URRUTI   6     b   20   44     Eu:West     *  2005    CS  Prev  CB:symptoms   0 Current       Cigs  Nev cigs  21   +
  WAGEN2   6     b   21   68     Eu:West  2001  2004    CC  Prev  CB:symptoms   0    Ever       Cigs  Nev cigs  21   +
     WEN  10     m   35   99   Asia:FarE  1982  2004    Pr   Inc CB:mortality   1 Current       Cigs  Nev cigs  21   +
   WOOLF   6     f   25   54   Am:Canada     *  1974    CS  Prev  CB:symptoms   0 Current       Cigs  Nev cigs  21   +


  ________________________________________________________________________________________________________________________
                                            International Evidence on Smoking and COPD, Phase 3, Analysis run on 28-SEP-10

                                                   Table 2 - E - 3 - 5

                               IESCOPD - Meta-analysis of amount smoked : key value (1) 45
                             Any CB, cigarettes (or any product if cigarettes not available)
                                                      Least-adjusted


                        Number Exposed  Non-exposed
 REF    NRR SEX ADJ     Case    Cont    Case    Cont      RR        95.00%CI
 ALDERS 13  m   1        148       -      31       -      2.75 (  1.68-   4.50)
 ALDERS 18  f   1        104       -     111       -      4.53 (  2.98-   6.88)
 Subtotal ALDERS                                          3.68 (  2.67-   5.06)
 ANDER1 42  m   1         14       -       4       -      7.54 (  2.21-  25.76)
 ANDER1 47  f   1          7       -      15       -      7.99 (  2.60-  24.59)
 Subtotal ANDER1                                          7.78 (  3.40-  17.83)
*BEST   6   m   1         12       -       3       -     14.63 (  4.13-  51.82)
 CHAPMA 6   m   0        102     226      35     989     12.75 (  8.46-  19.22)
 CHAPMA 14  f   0         61     129      48    1857     18.29 ( 12.04-  27.79)
 Subtotal CHAPMA                                         15.22 ( 11.36-  20.40)
 COATES 11  b   0         43     127      21     515      8.30 (  4.76-  14.49)
 DEAN2  15  m   1         23       -      39       -      3.80 (  2.21-   6.53)
 DEAN2  27  f   1         34       -      80       -      5.72 (  3.75-   8.73)
 Subtotal DEAN2                                           4.90 (  3.51-   6.84)
*DOLL1  18  m   1         33       -       3       -     21.20 (  6.16-  72.97)
*DONTA2 7   m   0          5      14       8     127      5.67 (  2.15-  14.98)
 FERRI1 145 m   1         10       -      10       -     19.04 (  4.59-  79.02)
 FERRI1 102 f   1         13       -      35       -      3.62 (  1.73-   7.56)
 Subtotal FERRI1                                          5.14 (  2.67-   9.90)
 HARRIS 6   m   0          2       1       6     518    172.67 ( 13.73-2171.40)
 HOLLA2 6   m   0         88     130       5      84     11.37 (  4.43-  29.17)
 HOLLNA 6   m   0         25      33      10     101      7.65 (  3.33-  17.58)
 HOLLNA 14  f   0          5       9      14     217      8.61 (  2.54-  29.15)
 Subtotal HOLLNA                                          7.94 (  4.00-  15.80)
 HUCHON 14  b   0        145     948     177    7183      6.21 (  4.93-   7.81)
 HUHTI1 47  m   0         34      51       7     115     10.95 (  4.55-  26.35)
 HUHTI3 25  m   0         44      32      28     211     10.36 (  5.67-  18.92)
*KAHN2  6   m   1          5       -      13       -      6.91 (  2.46-  19.38)
 LAMBER 9   m   0         31     386      17     642      3.03 (  1.66-   5.55)
 LAMBER 57  f   0          7      62      54    2390      5.00 (  2.19-  11.42)
 Subtotal LAMBER                                          3.61 (  2.22-   5.88)
 MUELLE 42  m   0         21      35       2      57     17.10 (  3.78-  77.43)
 MUELLE 68  f   0          9      18       3     168     28.00 (  6.95- 112.88)
 Subtotal MUELLE                                         22.32 (  8.01-  62.16)
 OGILVI 18  m   0         21      12      11      29      4.61 (  1.71-  12.45)
 OGILVI 52  f   0          9       3      86     160      5.58 (  1.47-  21.16)
 Subtotal OGILVI                                          4.94 (  2.23-  10.94)
 REID   9   m   0         61    2866      16    4127      5.49 (  3.16-   9.54)
 REID   25  f   0         25     806      37   10430      8.74 (  5.24-  14.60)
 Subtotal REID                                            7.05 (  4.84-  10.27)
*TROISI 6   f   1          -       -       -       -      4.63 (  3.88-   5.50)
 URRUTI 6   b   0         54     160      16     707     14.91 (  8.32-  26.73)
 WAGEN2 6   b   0         53     521     111    2136      1.96 (  1.39-   2.75)
*WEN    10  m   1          -       -       7       -      3.01 (  0.33-  27.60)
 WOOLF  6   f   0         57      66      10     173     14.94 (  7.20-  30.98)
Partial Totals          1305    6635    1073   32936
*prospective study


  ________________________________________________________________________________________________________________________
                                            International Evidence on Smoking and COPD, Phase 3, Analysis run on 28-SEP-10

                                                   Table 2 - E - 3 - 5

                               IESCOPD - Meta-analysis of amount smoked : key value (1) 45
                             Any CB, cigarettes (or any product if cigarettes not available)
                                                      Least-adjusted


 REF    NRR SEX ADJ             Ys       Ws       Qs       Ps
 ALDERS 13  m   1              1.01    15.83     9.17       0.00
 ALDERS 18  f   1              1.51    21.95     1.51       0.00
 Subtotal ALDERS               1.30    37.78    10.67
 ANDER1 42  m   1              2.02     2.55     0.16       0.00
 ANDER1 47  f   1              2.08     3.04     0.28       0.00
 Subtotal ANDER1               2.05     5.59     0.44
*BEST   6   m   1              2.68     2.40     1.99       0.00
 CHAPMA 6   m   0              2.55    22.83    13.65       0.00
 CHAPMA 14  f   0              2.91    21.97    28.25       0.00
 Subtotal CHAPMA               2.72    44.79    41.90
 COATES 11  b   0              2.12    12.39     1.47       0.00
 DEAN2  15  m   1              1.34    13.09     2.51       0.00
 DEAN2  27  f   1              1.74    21.52     0.02       0.00
 Subtotal DEAN2                1.59    34.61     2.52
*DOLL1  18  m   1              3.05     2.51     4.13       0.00
*DONTA2 7   m   0              1.74     4.07     0.01       0.00
 FERRI1 145 m   1              2.95     1.90     2.61       0.00
 FERRI1 102 f   1              1.29     7.07     1.67       0.00
 Subtotal FERRI1               1.64     8.96     4.28
 HARRIS 6   m   0              5.15     0.60     6.84       0.00
 HOLLA2 6   m   0              2.43     4.33     1.88       0.00
 HOLLNA 6   m   0              2.03     5.55     0.38       0.00
 HOLLNA 14  f   0              2.15     2.58     0.37       0.00
 Subtotal HOLLNA               2.07     8.13     0.76
 HUCHON 14  b   0              1.83    72.78     0.21       0.00
 HUHTI1 47  m   0              2.39     4.99     1.92       0.00
 HUHTI3 25  m   0              2.34    10.59     3.39       0.00
*KAHN2  6   m   1              1.93     3.61     0.09       0.00
 LAMBER 9   m   0              1.11    10.50     4.62       0.00
 LAMBER 57  f   0              1.61     5.62     0.15       0.00
 Subtotal LAMBER               1.28    16.12     4.77
 MUELLE 42  m   0              2.84     1.68     1.92       0.00
 MUELLE 68  f   0              3.33     1.98     4.81       0.00
 Subtotal MUELLE               3.11     3.66     6.72
 OGILVI 18  m   0              1.53     3.90     0.23       0.00
 OGILVI 52  f   0              1.72     2.16     0.01       0.01
 Subtotal OGILVI               1.60     6.06     0.24
 REID   9   m   0              1.70    12.58     0.06       0.00
 REID   25  f   0              2.17    14.63     2.29       0.00
 Subtotal REID                 1.95    27.21     2.35
*TROISI 6   f   1              1.53   126.22     7.27       0.00
 URRUTI 6   b   0              2.70    11.28     9.75       0.00
 WAGEN2 6   b   0              0.67    33.04    40.04       0.00
*WEN    10  m   1              1.10     0.78     0.35       0.33
 WOOLF  6   f   0              2.70     7.22     6.27       0.00

                       N       35
                      NS       25


                      Wt   489.73
                 Het Chi   160.26
                 Het  df       34
                 Het  P       ***
               Fixed  RR     5.89
                     RRl     5.39
                     RRu     6.43
                      P       +++
              Random  RR     7.22
                     RRl     5.76
                     RRu     9.06
                      P       +++
               Asymm  P         *


  ________________________________________________________________________________________________________________________
                                            International Evidence on Smoking and COPD, Phase 3, Analysis run on 28-SEP-10

                                                   Table 2 - E - 3 - 6

                               IESCOPD - Meta-analysis of amount smoked : key value (1) 45
                             Any CB, cigarettes (or any product if cigarettes not available)
                                                      Least-adjusted


                       N       35
                      NS       25


                      Wt   489.73
                 Het Chi   160.26
                 Het  df       34
                 Het  P       ***
               Fixed  RR     5.89
                     RRl     5.39
                     RRu     6.43
                      P       +++
              Random  RR     7.22
                     RRl     5.76
                     RRu     9.06
                      P       +++
               Asymm  P         *

                                   Sex
                             both      male    female     Total


                       N        4        19        12        35
                      NS        4        19        12        35


                      Wt   129.49    124.29    235.96    489.73
                 Het Chi    49.03     53.71     52.89    160.26
                 Het  df        3        18        11        34
                 Het  P       ***       ***       ***       ***
               Fixed  RR     5.13      6.72      5.92      5.89
                     RRl     4.32      5.64      5.21      5.39
                     RRu     6.10      8.01      6.72      6.43
                      P       +++       +++       +++       +++
              Random  RR     6.07      7.58      7.42      7.22
                     RRl     2.77      5.41      5.18      5.76
                     RRu    13.32     10.61     10.62      9.06
                      P       +++       +++       +++       +++
             Between Chi                                   4.63
             Between  df                                      2
             Between  P                                     (*)
             Btwn(F)  P                                    N.S.

                                        Continent
                            NAmer    Europe      Asia  oth/mult     Total


                       N       16        17         1         1        35
                      NS       11        12         1         1        25


                      Wt   246.39    241.96      0.78      0.60    489.73
                 Het Chi    68.49     71.61      0.00      0.00    160.26
                 Het  df       15        16         0         0        34
                 Het  P       ***       ***      N.S.      N.S.       ***
               Fixed  RR     6.90      4.98      3.01    172.67      5.89
                     RRl     6.09      4.39      0.33     13.73      5.39
                     RRu     7.81      5.65     27.53   2171.40      6.43
                      P       +++       +++      N.S.       +++       +++
              Random  RR     9.47      5.59      3.01    172.67      7.22
                     RRl     6.72      4.13      0.33     13.73      5.76
                     RRu    13.34      7.55     27.53   2171.40      9.06
                      P       +++       +++      N.S.       +++       +++
             Between Chi                                            20.16
             Between  df                                                3
             Between  P                                               ***
             Btwn(F)  P                                              N.S.


  ________________________________________________________________________________________________________________________
                                            International Evidence on Smoking and COPD, Phase 3, Analysis run on 28-SEP-10

                                                   Table 2 - E - 3 - 6

                               IESCOPD - Meta-analysis of amount smoked : key value (1) 45
                             Any CB, cigarettes (or any product if cigarettes not available)
                                                      Least-adjusted
                               Study type
                               CC        Pr        CS     Total


                       N        5         6        24        35
                      NS        3         6        16        25


                      Wt    76.88    139.60    273.26    489.73
                 Het Chi    11.27      9.37     71.91    160.26
                 Het  df        4         5        23        34
                 Het  P         *       (*)       ***       ***
               Fixed  RR     2.87      4.92      7.89      5.89
                     RRl     2.29      4.17      7.01      5.39
                     RRu     3.59      5.81      8.89      6.43
                      P       +++       +++       +++       +++
              Random  RR     3.21      6.90      8.52      7.22
                     RRl     2.09      4.11      6.71      5.76
                     RRu     4.94     11.60     10.82      9.06
                      P       +++       +++       +++       +++
             Between Chi                                  67.70
             Between  df                                      2
             Between  P                                     ***
             Btwn(F)  P                                     ***

                           CB subtype
                             mort     sympt     other     Total


                       N        4        27         4        35
                      NS        4        18         3        25


                      Wt     9.31    312.36    168.07    489.73
                 Het Chi     3.36    129.88      4.10    160.26
                 Het  df        3        26         3        34
                 Het  P      N.S.       ***      N.S.       ***
               Fixed  RR    10.58      6.75      4.42      5.89
                     RRl     5.57      6.04      3.80      5.39
                     RRu    20.12      7.54      5.14      6.43
                      P       +++       +++       +++       +++
              Random  RR    10.57      7.84      4.26      7.22
                     RRl     5.31      5.94      3.40      5.76
                     RRu    21.06     10.35      5.35      9.06
                      P       +++       +++       +++       +++
             Between Chi                                  22.91
             Between  df                                      2
             Between  P                                     ***
             Btwn(F)  P                                     (*)

                             Smoking product
                              any      cigs  cigsonly     Total


                       N        5        24         6        35
                      NS        4        17         4        25


                      Wt    23.65    388.78     77.30    489.73
                 Het Chi     5.38    128.00     14.78    160.26
                 Het  df        4        23         5        34
                 Het  P      N.S.       ***         *       ***
               Fixed  RR    10.33      5.97      4.62      5.89
                     RRl     6.90      5.40      3.70      5.39
                     RRu    15.46      6.59      5.77      6.43
                      P       +++       +++       +++       +++
              Random  RR    10.61      7.27      5.19      7.22
                     RRl     6.43      5.49      3.41      5.76
                     RRu    17.51      9.64      7.92      9.06
                      P       +++       +++       +++       +++
             Between Chi                                  12.10
             Between  df                                      2
             Between  P                                      **
             Btwn(F)  P                                    N.S.
  ________________________________________________________________________________________________________________________
                                            International Evidence on Smoking and COPD, Phase 3, Analysis run on 28-SEP-10

                                                   Table 2 - E - 3 - 6

                               IESCOPD - Meta-analysis of amount smoked : key value (1) 45
                             Any CB, cigarettes (or any product if cigarettes not available)
                                                      Least-adjusted
                                     Unexposed group
                          nev any   nev cig  nev+ any  nev+ cig     Total


                       N       22        13                            35
                      NS       15        11                            26


                      Wt   139.45    350.29                        489.73
                 Het Chi    46.01    114.22                        160.26
                 Het  df       21        12                            34
                 Het  P        **       ***                           ***
               Fixed  RR     5.95      5.86                          5.89
                     RRl     5.04      5.28                          5.39
                     RRu     7.02      6.51                          6.43
                      P       +++       +++                           +++
              Random  RR     7.17      7.00                          7.22
                     RRl     5.46      4.84                          5.76
                     RRu     9.43     10.12                          9.06
                      P       +++       +++                           +++
             Between Chi                                             0.02
             Between  df                                                1
             Between  P                                              N.S.
             Btwn(F)  P                                              N.S.

                        Unexposed group (combining nev+ with main levels)
                          nev any   nev cig     Total


                       N       22        13        35
                      NS       15        11        26


                      Wt   139.45    350.29    489.73
                 Het Chi    46.01    114.22    160.26
                 Het  df       21        12        34
                 Het  P        **       ***       ***
               Fixed  RR     5.95      5.86      5.89
                     RRl     5.04      5.28      5.39
                     RRu     7.02      6.51      6.43
                      P       +++       +++       +++
              Random  RR     7.17      7.00      7.22
                     RRl     5.46      4.84      5.76
                     RRu     9.43     10.12      9.06
                      P       +++       +++       +++
             Between Chi                         0.02
             Between  df                            1
             Between  P                          N.S.
             Btwn(F)  P                          N.S.


  ________________________________________________________________________________________________________________________
                                            International Evidence on Smoking and COPD, Phase 3, Analysis run on 28-SEP-10

                                                   Table 2 - E - 3 - 7

                               IESCOPD - Meta-analysis of amount smoked : key value (1) 45
                             Any CB, cigarettes (or any product if cigarettes not available)
                                 Excluded studies (and stage at which they were excluded)


1       CLARK COTTON  MEYER REMYJA RUTGER SNYDER SOBRAX     SU TAKEMU  WANG4   WEIR WHICKE ZALACA
2      ALESSA  AMIGO ANDER2 ANDER3 AUERBA BEDNAR BROGGE  CHEN1  CHEN2  CHEN3  CHENG CLEMEN  COCCI  DEAN1 DEJONG DETORR
       DICKIN DONTA1 EKBERG ENSTRO FERRI2 FERRI3  FIDAN FORAST FUKUCH GEIJER GODTFR GULSVI HAMMO2 HARIKK HEDMAN HIGGI4
       HOZAWA ITABAS JACOBS JAENDI JOHANN KACHEL KARAKA KATANC KHOURY    KIM KLAYTO KOJIMA KOTAN2 KRZYZA KULLER    LAI
         LAM1   LAM2   LAM3  LANGE    LEE   LIAW LINDBE   LIU1   LIU2 LUNDB1  MADOR MANNI1 MANNI2 MANNI3 MARAN1 MARAN2
       MARCUS MATHES MENEZ2 MENEZ3 MENEZ4 MENEZ5 MENEZ6 MONTNE   NAWA NIEPSU NIHLEN NILSSO  OMORI   PEAT   PETO  PRATT
        PRICE RENWIC RICCIO  RYDER SARGEA SHAHAB   SHIN SICHLE SPEIZE STERLI  STROM SUTINE  TAGER   TANG   THUN   TODD
       TRUPIN TSUSHI TVERDA VESTBO VIEGI2 VIKGRE VINEIS VOLLM1 VOLLM2 VONHER   WALD  WANG2 WATSON  WEISS WILSO1   XIAO
           XU   YUAN ZIELI1 ZIELI2 ZIETKO
3        KAHN
4        BANG  BECK1  BECK2 BJORNS CERVER COLLEG  DEANE DEMARC DOPICO ENRIGH FINKLE FOXMAN GOLDBE HARDIE  HAYES HIRAYA
           HO  HOUSE HUHTI2 JENSEN JINDA2  JOSHI JOUSI1   KATO  KIRAZ KOTAN1  KUBIK LANGE2 LANGHA LUNDB2 MAGNUS MANFRE
       MELLST MILLER MOLLER NEJJAR OSWAL1 OSWAL2 PANDEY PELKON PEREZP SAWICK SCHWAR SHIMUR  SILVA SOBRAD STJERN SUADIC
       TAGER2 VIEGI1 WOJTYN  WOODS   ZOIA
5       DOLL2 FLETCH HAWTHO HRUBEC RIMING  SHARP    WIG WILSO2
6       BROWN EHRLIC HAENSZ HIGGI2 HIGGI3 HIGGI6 LAVECC LEBOWI LINDST MENEZ1  MEREN  MILNE WILHEL YAMAGU


  ________________________________________________________________________________________________________________________
                                            International Evidence on Smoking and COPD, Phase 3, Analysis run on 28-SEP-10

                                                   Table 2 - E - 3 - 8

                               IESCOPD - Meta-analysis of amount smoked : key value (1) 45
                             Any CB, cigarettes (or any product if cigarettes not available)
                                             Potentially overlapping studies


     REF| REFGP|PRINC|                     OVERLAP|

  URRUTI DEMARC     2         DEMARC/URRUTI/DEMEER
  DONTA2 JACOBS     2  JACOBS/DONTA1/DONTA2/PELKON
  HUHTI1 HUHTI1     1                HUHTI1/HUHTI2
  FERRI1 FERRIS     2         FERRI1/FERRI2/FERRI3
     WEN    WEN     1                     WEN/LIAW
  LAMBER   TODD     2                  LAMBER/TODD
  CHAPMA  HOUSE     2                 HOUSE/CHAPMA
  HOLLNA HOLLNA     1 GODT/VEST/LANG1+2/SUAD/HOLLN
   KAHN2   KAHN     2                   KAHN/KAHN2


  ________________________________________________________________________________________________________________________
                                            International Evidence on Smoking and COPD, Phase 3, Analysis run on 28-SEP-10

                                                    Table 2 - E - 4 -

                                IESCOPD - Meta-analysis of amount smoked : key value (2) 1
                             Any CB, cigarettes (or any product if cigarettes not available)


This analysis is restricted to results for:
1) Eligible study on database
2) Outcome CB
3) Current or ever smoking
4) Categorical dose-response data for amount smoked
5) vs never smoking base
6) Key value (scheme 2) = 1
7) Results complete enough for use in meta-analysis

Within each study, results are then selected (in the following order of preference, within each sex) for:
8) SMKSTA  : current, ever
9) UNEXP   : never any, never cigarettes
10) PROD    : cigarettes, cigarettes only, any product
11) For overlapping studies: principal rather than subsidiary studies
and then for single sex results (m, f) in preference to results for both sexes combined (b).

Results adjusted for the most potential confounders are then chosen in Sections -1 to -3
and results adjusted for the least confounders in Sections -4 to -6. (Those least-adjusted results which
actually differ from the most-adjusted are marked 'x' in column X in Section -4)

Section -7 shows excluded studies, together with the stage (as above) at which no qualifying
results were found.

Section -8 lists the potentially overlapping studies which have been included (1=principal, 2=subsidiary),
and any results which would have been included in preference except that they had data not complete enough
for use in meta-analysis. It also lists their significance (yes/no), if known.


  ________________________________________________________________________________________________________________________
                                            International Evidence on Smoking and COPD, Phase 3, Analysis run on 28-SEP-10

                                                   Table 2 - E - 4 - 1

                                IESCOPD - Meta-analysis of amount smoked : key value (2) 1
                             Any CB, cigarettes (or any product if cigarettes not available)
                                                      Most-adjusted


     REF|NRR|SEX|AGEL|AGEH|     REGION|BEGYR|PUBYR|STTYP|ONSET|      DISEAS|ADJ|SMOKSTA|   PRODUCT|    UNEXP|LOW| HI|

    BEST   4   m   30   97   Am:Canada  1955  1967    Pr   Inc CB:mortality   1 Current  Cigs only   Nev any   1   9
   BROWN   9   m   60   69       Eu:UK  1956  1957    CS  Prev CB:diagnosed   0 Current  Cigs only   Nev any   1   9
   DEAN2   9   m   37   67       Eu:UK  1972  1978    CS  Prev  CB:symptoms   1 Current MCigs only   Nev any   1   7
   DEAN2  23   f   37   67       Eu:UK  1972  1978    CS  Prev  CB:symptoms   1 Current MCigs only   Nev any   1   7
  DONTA2   4   m   25   84 Eu:SE/Balkn  1960  1984    Pr   Inc CB:diagnosed   0 Current       Cigs  Nev cigs   1   9
  HAENSZ  56   m   35   74    Eu:Scand  1964  1972    CS  Prev  CB:symptoms   1 Current  Cigs only   Nev any   1   9
  HAENSZ  74   f   35   74    Eu:Scand  1964  1972    CS  Prev  CB:symptoms   1 Current       Cigs   Nev any   1   9
  HUCHON  22   b   25   99     Eu:West     *  2002    CS  Prev  CB:symptoms   1 Current       Cigs  Nev cigs   1   1
   KAHN2   3   m   31   84      Am:USA  1954  1966    Pr   Inc CB:mortality   1 Current       Cigs   Nev any   1   9
  LINDST  24   b   20   69    Eu:Scand     *  2001    CS  Prev  CB:symptoms   5 Current       Cigs  Nev cigs   1   4
   MEREN   2   b   15   64     Eu:East  1995  2001    CS  Prev  CB:symptoms   4 Current       Cigs  Nev cigs   1   4
  OGILVI  15   m   30   99       Eu:UK  1955  1957    CC  Prev  CB:symptoms   0 Current       Cigs   Nev any   1   5
  OGILVI  27   f   30   99       Eu:UK  1955  1957    CC  Prev  CB:symptoms   0 Current       Cigs   Nev any   1   5
  URRUTI   4   b   20   44     Eu:West     *  2005    CS  Prev  CB:symptoms   0 Current       Cigs  Nev cigs   1   9
  YAMAGU  10   b   40   99   Asia:FarE  1986  1988    CS  Prev  CB:symptoms   6 Current       Cigs  Nev cigs   1   9


  ________________________________________________________________________________________________________________________
                                            International Evidence on Smoking and COPD, Phase 3, Analysis run on 28-SEP-10

                                                   Table 2 - E - 4 - 2

                                IESCOPD - Meta-analysis of amount smoked : key value (2) 1
                             Any CB, cigarettes (or any product if cigarettes not available)
                                                      Most-adjusted


                        Number Exposed  Non-exposed
 REF    NRR SEX ADJ     Case    Cont    Case    Cont      RR        95.00%CI
*BEST   4   m   1         17       -       3       -      7.02 (  2.06-  23.94)
 BROWN  9   m   0         36      97      15      76      1.88 (  0.96-   3.69)
 DEAN2  9   m   1         11       -      39       -      1.97 (  1.00-   3.88)
 DEAN2  23  f   1         17       -      80       -      1.39 (  0.82-   2.35)
 Subtotal DEAN2                                           1.58 (  1.05-   2.40)
*DONTA2 4   m   0         18     118       8     127      2.42 (  1.09-   5.36)
 HAENSZ 56  m   1         22       -      27       -      1.82 (  1.02-   3.24)
 HAENSZ 74  f   1         31       -      97       -      2.43 (  1.59-   3.70)
 Subtotal HAENSZ                                          2.20 (  1.56-   3.09)
 HUCHON 22  b   1          3       -     177       -      1.81 (  0.61-   5.34)
*KAHN2  3   m   1          6       -      13       -      2.13 (  0.81-   5.60)
 LINDST 24  b   5          -       -     494       -      0.84 (  0.59-   1.20)
 MEREN  2   b   4          -       -       -       -      1.57 (  1.20-   2.13)
 OGILVI 15  m   0         23       8      11      29      7.58 (  2.62-  21.93)
 OGILVI 27  f   0         27      31      86     160      1.62 (  0.91-   2.89)
 Subtotal OGILVI                                          2.31 (  1.39-   3.83)
 URRUTI 4   b   0         12     222      16     707      2.39 (  1.11-   5.13)
 YAMAGU 10  b   6          -       -     128       -      2.34 (  1.81-   3.02)
Partial Totals           223     476    1194    1099
*prospective study


 REF    NRR SEX ADJ             Ys       Ws       Qs       Ps
*BEST   4   m   1              1.95     2.55     4.69       0.00
 BROWN  9   m   0              0.63     8.48     0.01       0.07
 DEAN2  9   m   1              0.68     8.36     0.06       0.05
 DEAN2  23  f   1              0.33    13.86     0.97       0.22
 Subtotal DEAN2                0.46    22.22     1.03
*DONTA2 4   m   0              0.88     6.09     0.51       0.03
 HAENSZ 56  m   1              0.60    11.50     0.00       0.04
 HAENSZ 74  f   1              0.89    21.54     1.86       0.00
 Subtotal HAENSZ               0.79    33.04     1.86
 HUCHON 22  b   1              0.59     3.26     0.00       0.28
*KAHN2  3   m   1              0.76     4.11     0.11       0.13
 LINDST 24  b   5             -0.17    30.49    17.99       0.34
 MEREN  2   b   4              0.45    46.67     0.95       0.00
 OGILVI 15  m   0              2.03     3.40     6.97       0.00
 OGILVI 27  f   0              0.48    11.47     0.14       0.10
 Subtotal OGILVI               0.84    14.87     7.12
 URRUTI 4   b   0              0.87     6.59     0.50       0.03
 YAMAGU 10  b   6              0.85    58.63     3.85       0.00

                       N       15
                      NS       12


                      Wt   237.01
                 Het Chi    38.63
                 Het  df       14
                 Het  P       ***
               Fixed  RR     1.81
                     RRl     1.59
                     RRu     2.06
                      P       +++
              Random  RR     1.95
                     RRl     1.53
                     RRu     2.47
                      P       +++
               Asymm  P      N.S.


  ________________________________________________________________________________________________________________________
                                            International Evidence on Smoking and COPD, Phase 3, Analysis run on 28-SEP-10

                                                   Table 2 - E - 4 - 3

                                IESCOPD - Meta-analysis of amount smoked : key value (2) 1
                             Any CB, cigarettes (or any product if cigarettes not available)
                                                      Most-adjusted


                       N       15
                      NS       12


                      Wt   237.01
                 Het Chi    38.63
                 Het  df       14
                 Het  P       ***
               Fixed  RR     1.81
                     RRl     1.59
                     RRu     2.06
                      P       +++
              Random  RR     1.95
                     RRl     1.53
                     RRu     2.47
                      P       +++
               Asymm  P      N.S.

                                   Sex
                             both      male    female     Total


                       N        5         7         3        15
                      NS        5         7         3        15


                      Wt   145.64     44.50     46.87    237.01
                 Het Chi    22.10      9.20      2.93     38.63
                 Het  df        4         6         2        14
                 Het  P       ***      N.S.      N.S.       ***
               Fixed  RR     1.65      2.36      1.87      1.81
                     RRl     1.41      1.76      1.40      1.59
                     RRu     1.95      3.17      2.48      2.06
                      P       +++       +++       +++       +++
              Random  RR     1.62      2.51      1.83      1.95
                     RRl     1.04      1.72      1.29      1.53
                     RRu     2.52      3.65      2.60      2.47
                      P         +       +++       +++       +++
             Between Chi                                   4.40
             Between  df                                      2
             Between  P                                    N.S.
             Btwn(F)  P                                    N.S.

                                        Continent
                            NAmer    Europe      Asia  oth/mult     Total


                       N        2        12         1                  15
                      NS        2         9         1                  12


                      Wt     6.66    171.72     58.63              237.01
                 Het Chi     2.24     27.85      0.00               38.63
                 Het  df        1        11         0                  14
                 Het  P      N.S.        **      N.S.                 ***
               Fixed  RR     3.36      1.62      2.34                1.81
                     RRl     1.57      1.39      1.81                1.59
                     RRu     7.19      1.88      3.02                2.06
                      P        ++       +++       +++                 +++
              Random  RR     3.63      1.80      2.34                1.95
                     RRl     1.14      1.39      1.81                1.53
                     RRu    11.62      2.34      3.02                2.47
                      P         +       +++       +++                 +++
             Between Chi                                             8.54
             Between  df                                                2
             Between  P                                                 *
             Btwn(F)  P                                              N.S.


  ________________________________________________________________________________________________________________________
                                            International Evidence on Smoking and COPD, Phase 3, Analysis run on 28-SEP-10

                                                   Table 2 - E - 4 - 3

                                IESCOPD - Meta-analysis of amount smoked : key value (2) 1
                             Any CB, cigarettes (or any product if cigarettes not available)
                                                      Most-adjusted
                               Study type
                               CC        Pr        CS     Total


                       N        2         3        10        15
                      NS        1         3         8        12


                      Wt    14.87     12.75    209.39    237.01
                 Het Chi     6.25      2.58     25.77     38.63
                 Het  df        1         2         9        14
                 Het  P         *      N.S.        **       ***
               Fixed  RR     2.31      2.88      1.73      1.81
                     RRl     1.39      1.66      1.51      1.59
                     RRu     3.83      4.98      1.98      2.06
                      P        ++       +++       +++       +++
              Random  RR     3.28      2.95      1.73      1.95
                     RRl     0.73      1.56      1.34      1.53
                     RRu    14.78      5.57      2.23      2.47
                      P      N.S.       +++       +++       +++
             Between Chi                                   4.03
             Between  df                                      2
             Between  P                                    N.S.
             Btwn(F)  P                                    N.S.

                           CB subtype
                             mort     sympt     other     Total


                       N        2        11         2        15
                      NS        2         8         2        12


                      Wt     6.66    215.78     14.57    237.01
                 Het Chi     2.24     33.13      0.23     38.63
                 Het  df        1        10         1        14
                 Het  P      N.S.       ***      N.S.       ***
               Fixed  RR     3.36      1.76      2.09      1.81
                     RRl     1.57      1.54      1.25      1.59
                     RRu     7.19      2.01      3.49      2.06
                      P        ++       +++        ++       +++
              Random  RR     3.63      1.83      2.09      1.95
                     RRl     1.14      1.40      1.25      1.53
                     RRu    11.62      2.40      3.49      2.47
                      P         +       +++        ++       +++
             Between Chi                                   3.03
             Between  df                                      2
             Between  P                                    N.S.
             Btwn(F)  P                                    N.S.

                             Smoking product
                              any      cigs  cigsonly     Total


                       N                 10         5        15
                      NS                  9         4        13


                      Wt             192.26     44.76    237.01
                 Het Chi              32.90      5.71     38.63
                 Het  df                  9         4        14
                 Het  P                 ***      N.S.       ***
               Fixed  RR               1.80      1.85      1.81
                     RRl               1.57      1.38      1.59
                     RRu               2.08      2.48      2.06
                      P                 +++       +++       +++
              Random  RR               1.95      1.92      1.95
                     RRl               1.43      1.34      1.53
                     RRu               2.67      2.75      2.47
                      P                 +++       +++       +++
             Between Chi                                   0.02
             Between  df                                      1
             Between  P                                    N.S.
             Btwn(F)  P                                    N.S.
  ________________________________________________________________________________________________________________________
                                            International Evidence on Smoking and COPD, Phase 3, Analysis run on 28-SEP-10

                                                   Table 2 - E - 4 - 3

                                IESCOPD - Meta-analysis of amount smoked : key value (2) 1
                             Any CB, cigarettes (or any product if cigarettes not available)
                                                      Most-adjusted
                                     Unexposed group
                          nev any   nev cig  nev+ any  nev+ cig     Total


                       N        9         6                            15
                      NS        6         6                            12


                      Wt    85.28    151.73                        237.01
                 Het Chi    13.27     22.95                         38.63
                 Het  df        8         5                            14
                 Het  P      N.S.       ***                           ***
               Fixed  RR     2.07      1.68                          1.81
                     RRl     1.68      1.43                          1.59
                     RRu     2.56      1.97                          2.06
                      P       +++       +++                           +++
              Random  RR     2.15      1.70                          1.95
                     RRl     1.61      1.14                          1.53
                     RRu     2.87      2.54                          2.47
                      P       +++        ++                           +++
             Between Chi                                             2.41
             Between  df                                                1
             Between  P                                              N.S.
             Btwn(F)  P                                              N.S.

                        Unexposed group (combining nev+ with main levels)
                          nev any   nev cig     Total


                       N        9         6        15
                      NS        6         6        12


                      Wt    85.28    151.73    237.01
                 Het Chi    13.27     22.95     38.63
                 Het  df        8         5        14
                 Het  P      N.S.       ***       ***
               Fixed  RR     2.07      1.68      1.81
                     RRl     1.68      1.43      1.59
                     RRu     2.56      1.97      2.06
                      P       +++       +++       +++
              Random  RR     2.15      1.70      1.95
                     RRl     1.61      1.14      1.53
                     RRu     2.87      2.54      2.47
                      P       +++        ++       +++
             Between Chi                         2.41
             Between  df                            1
             Between  P                          N.S.
             Btwn(F)  P                          N.S.


  ________________________________________________________________________________________________________________________
                                            International Evidence on Smoking and COPD, Phase 3, Analysis run on 28-SEP-10

                                                   Table 2 - E - 4 - 4

                                IESCOPD - Meta-analysis of amount smoked : key value (2) 1
                             Any CB, cigarettes (or any product if cigarettes not available)
                                                      Least-adjusted


     REF|NRR|X|SEX|AGEL|AGEH|     REGION|BEGYR|PUBYR|STTYP|ONSET|      DISEAS|ADJ|SMOKSTA|   PRODUCT|    UNEXP|LOW| HI|

    BEST   4     m   30   97   Am:Canada  1955  1967    Pr   Inc CB:mortality   1 Current  Cigs only   Nev any   1   9
   BROWN   9     m   60   69       Eu:UK  1956  1957    CS  Prev CB:diagnosed   0 Current  Cigs only   Nev any   1   9
   DEAN2   9     m   37   67       Eu:UK  1972  1978    CS  Prev  CB:symptoms   1 Current MCigs only   Nev any   1   7
   DEAN2  23     f   37   67       Eu:UK  1972  1978    CS  Prev  CB:symptoms   1 Current MCigs only   Nev any   1   7
  DONTA2   4     m   25   84 Eu:SE/Balkn  1960  1984    Pr   Inc CB:diagnosed   0 Current       Cigs  Nev cigs   1   9
  HAENSZ  51 x   m   35   74    Eu:Scand  1964  1972    CS  Prev  CB:symptoms   0 Current  Cigs only   Nev any   1   9
  HAENSZ  71 x   f   35   74    Eu:Scand  1964  1972    CS  Prev  CB:symptoms   0 Current       Cigs   Nev any   1   9
  HUCHON  12 x   b   25   99     Eu:West     *  2002    CS  Prev  CB:symptoms   0 Current       Cigs  Nev cigs   1   1
   KAHN2   3     m   31   84      Am:USA  1954  1966    Pr   Inc CB:mortality   1 Current       Cigs   Nev any   1   9
  LINDST  24     b   20   69    Eu:Scand     *  2001    CS  Prev  CB:symptoms   5 Current       Cigs  Nev cigs   1   4
   MEREN   2     b   15   64     Eu:East  1995  2001    CS  Prev  CB:symptoms   4 Current       Cigs  Nev cigs   1   4
  OGILVI  15     m   30   99       Eu:UK  1955  1957    CC  Prev  CB:symptoms   0 Current       Cigs   Nev any   1   5
  OGILVI  27     f   30   99       Eu:UK  1955  1957    CC  Prev  CB:symptoms   0 Current       Cigs   Nev any   1   5
  URRUTI   4     b   20   44     Eu:West     *  2005    CS  Prev  CB:symptoms   0 Current       Cigs  Nev cigs   1   9
  YAMAGU  10     b   40   99   Asia:FarE  1986  1988    CS  Prev  CB:symptoms   6 Current       Cigs  Nev cigs   1   9


  ________________________________________________________________________________________________________________________
                                            International Evidence on Smoking and COPD, Phase 3, Analysis run on 28-SEP-10

                                                   Table 2 - E - 4 - 5

                                IESCOPD - Meta-analysis of amount smoked : key value (2) 1
                             Any CB, cigarettes (or any product if cigarettes not available)
                                                      Least-adjusted


                        Number Exposed  Non-exposed
 REF    NRR SEX ADJ     Case    Cont    Case    Cont      RR        95.00%CI
*BEST   4   m   1         17       -       3       -      7.02 (  2.06-  23.94)
 BROWN  9   m   0         36      97      15      76      1.88 (  0.96-   3.69)
 DEAN2  9   m   1         11       -      39       -      1.97 (  1.00-   3.88)
 DEAN2  23  f   1         17       -      80       -      1.39 (  0.82-   2.35)
 Subtotal DEAN2                                           1.58 (  1.05-   2.40)
*DONTA2 4   m   0         18     118       8     127      2.42 (  1.09-   5.36)
 HAENSZ 51  m   0         22    1438      27    2742      1.55 (  0.88-   2.74)
 HAENSZ 71  f   0         31    1734      97   11536      2.13 (  1.41-   3.20)
 Subtotal HAENSZ                                          1.91 (  1.37-   2.66)
 HUCHON 12  b   0          3     117     177    7183      1.04 (  0.33-   3.30)
*KAHN2  3   m   1          6       -      13       -      2.13 (  0.81-   5.60)
 LINDST 24  b   5          -       -     494       -      0.84 (  0.59-   1.20)
 MEREN  2   b   4          -       -       -       -      1.57 (  1.20-   2.13)
 OGILVI 15  m   0         23       8      11      29      7.58 (  2.62-  21.93)
 OGILVI 27  f   0         27      31      86     160      1.62 (  0.91-   2.89)
 Subtotal OGILVI                                          2.31 (  1.39-   3.83)
 URRUTI 4   b   0         12     222      16     707      2.39 (  1.11-   5.13)
 YAMAGU 10  b   6          -       -     128       -      2.34 (  1.81-   3.02)
Partial Totals           223    3765    1194   22560
*prospective study


 REF    NRR SEX ADJ             Ys       Ws       Qs       Ps
*BEST   4   m   1              1.95     2.55     4.87       0.00
 BROWN  9   m   0              0.63     8.48     0.03       0.07
 DEAN2  9   m   1              0.68     8.36     0.10       0.05
 DEAN2  23  f   1              0.33    13.86     0.79       0.22
 Subtotal DEAN2                0.46    22.22     0.89
*DONTA2 4   m   0              0.88     6.09     0.61       0.03
 HAENSZ 51  m   0              0.44    11.97     0.20       0.13
 HAENSZ 71  f   0              0.75    23.13     0.80       0.00
 Subtotal HAENSZ               0.65    35.10     1.00
 HUCHON 12  b   0              0.04     2.88     0.80       0.95
*KAHN2  3   m   1              0.76     4.11     0.14       0.13
 LINDST 24  b   5             -0.17    30.49    16.81       0.34
 MEREN  2   b   4              0.45    46.67     0.64       0.00
 OGILVI 15  m   0              2.03     3.40     7.23       0.00
 OGILVI 27  f   0              0.48    11.47     0.08       0.10
 Subtotal OGILVI               0.84    14.87     7.31
 URRUTI 4   b   0              0.87     6.59     0.60       0.03
 YAMAGU 10  b   6              0.85    58.63     4.66       0.00

                       N       15
                      NS       12


                      Wt   238.68
                 Het Chi    38.37
                 Het  df       14
                 Het  P       ***
               Fixed  RR     1.77
                     RRl     1.55
                     RRu     2.00
                      P       +++
              Random  RR     1.87
                     RRl     1.47
                     RRu     2.37
                      P       +++
               Asymm  P      N.S.


  ________________________________________________________________________________________________________________________
                                            International Evidence on Smoking and COPD, Phase 3, Analysis run on 28-SEP-10

                                                   Table 2 - E - 4 - 6

                                IESCOPD - Meta-analysis of amount smoked : key value (2) 1
                             Any CB, cigarettes (or any product if cigarettes not available)
                                                      Least-adjusted


                       N       15
                      NS       12


                      Wt   238.68
                 Het Chi    38.37
                 Het  df       14
                 Het  P       ***
               Fixed  RR     1.77
                     RRl     1.55
                     RRu     2.00
                      P       +++
              Random  RR     1.87
                     RRl     1.47
                     RRu     2.37
                      P       +++
               Asymm  P      N.S.

                                   Sex
                             both      male    female     Total


                       N        5         7         3        15
                      NS        5         7         3        15


                      Wt   145.25     44.97     48.46    238.68
                 Het Chi    22.67     10.43      1.68     38.37
                 Het  df        4         6         2        14
                 Het  P       ***      N.S.      N.S.       ***
               Fixed  RR     1.64      2.26      1.77      1.77
                     RRl     1.39      1.69      1.33      1.55
                     RRu     1.92      3.03      2.34      2.00
                      P       +++       +++       +++       +++
              Random  RR     1.54      2.46      1.77      1.87
                     RRl     0.98      1.65      1.33      1.47
                     RRu     2.41      3.67      2.34      2.37
                      P       (+)       +++       +++       +++
             Between Chi                                   3.59
             Between  df                                      2
             Between  P                                    N.S.
             Btwn(F)  P                                    N.S.

                                        Continent
                            NAmer    Europe      Asia  oth/mult     Total


                       N        2        12         1                  15
                      NS        2         9         1                  12


                      Wt     6.66    173.39     58.63              238.68
                 Het Chi     2.24     26.20      0.00               38.37
                 Het  df        1        11         0                  14
                 Het  P      N.S.        **      N.S.                 ***
               Fixed  RR     3.36      1.57      2.34                1.77
                     RRl     1.57      1.35      1.81                1.55
                     RRu     7.19      1.82      3.02                2.00
                      P        ++       +++       +++                 +++
              Random  RR     3.63      1.71      2.34                1.87
                     RRl     1.14      1.33      1.81                1.47
                     RRu    11.62      2.20      3.02                2.37
                      P         +       +++       +++                 +++
             Between Chi                                             9.93
             Between  df                                                2
             Between  P                                                **
             Btwn(F)  P                                              N.S.


  ________________________________________________________________________________________________________________________
                                            International Evidence on Smoking and COPD, Phase 3, Analysis run on 28-SEP-10

                                                   Table 2 - E - 4 - 6

                                IESCOPD - Meta-analysis of amount smoked : key value (2) 1
                             Any CB, cigarettes (or any product if cigarettes not available)
                                                      Least-adjusted
                               Study type
                               CC        Pr        CS     Total


                       N        2         3        10        15
                      NS        1         3         8        12


                      Wt    14.87     12.75    211.05    238.68
                 Het Chi     6.25      2.58     24.95     38.37
                 Het  df        1         2         9        14
                 Het  P         *      N.S.        **       ***
               Fixed  RR     2.31      2.88      1.68      1.77
                     RRl     1.39      1.66      1.47      1.55
                     RRu     3.83      4.98      1.92      2.00
                      P        ++       +++       +++       +++
              Random  RR     3.28      2.95      1.64      1.87
                     RRl     0.73      1.56      1.28      1.47
                     RRu    14.78      5.57      2.11      2.37
                      P      N.S.       +++       +++       +++
             Between Chi                                   4.59
             Between  df                                      2
             Between  P                                    N.S.
             Btwn(F)  P                                    N.S.

                           CB subtype
                             mort     sympt     other     Total


                       N        2        11         2        15
                      NS        2         8         2        12


                      Wt     6.66    217.45     14.57    238.68
                 Het Chi     2.24     32.51      0.23     38.37
                 Het  df        1        10         1        14
                 Het  P      N.S.       ***      N.S.       ***
               Fixed  RR     3.36      1.71      2.09      1.77
                     RRl     1.57      1.50      1.25      1.55
                     RRu     7.19      1.95      3.49      2.00
                      P        ++       +++        ++       +++
              Random  RR     3.63      1.74      2.09      1.87
                     RRl     1.14      1.33      1.25      1.47
                     RRu    11.62      2.28      3.49      2.37
                      P         +       +++        ++       +++
             Between Chi                                   3.40
             Between  df                                      2
             Between  P                                    N.S.
             Btwn(F)  P                                    N.S.

                             Smoking product
                              any      cigs  cigsonly     Total


                       N                 10         5        15
                      NS                  9         4        13


                      Wt             193.46     45.22    238.68
                 Het Chi              32.38      5.99     38.37
                 Het  df                  9         4        14
                 Het  P                 ***      N.S.       ***
               Fixed  RR               1.76      1.77      1.77
                     RRl               1.53      1.32      1.55
                     RRu               2.03      2.37      2.00
                      P                 +++       +++       +++
              Random  RR               1.87      1.85      1.87
                     RRl               1.37      1.28      1.47
                     RRu               2.55      2.67      2.37
                      P                 +++        ++       +++
             Between Chi                                   0.00
             Between  df                                      1
             Between  P                                    N.S.
             Btwn(F)  P                                    N.S.
  ________________________________________________________________________________________________________________________
                                            International Evidence on Smoking and COPD, Phase 3, Analysis run on 28-SEP-10

                                                   Table 2 - E - 4 - 6

                                IESCOPD - Meta-analysis of amount smoked : key value (2) 1
                             Any CB, cigarettes (or any product if cigarettes not available)
                                                      Least-adjusted
                                     Unexposed group
                          nev any   nev cig  nev+ any  nev+ cig     Total


                       N        9         6                            15
                      NS        6         6                            12


                      Wt    87.34    151.34                        238.68
                 Het Chi    13.28     23.57                         38.37
                 Het  df        8         5                            14
                 Het  P      N.S.       ***                           ***
               Fixed  RR     1.96      1.66                          1.77
                     RRl     1.59      1.42                          1.55
                     RRu     2.42      1.95                          2.00
                      P       +++       +++                           +++
              Random  RR     2.05      1.63                          1.87
                     RRl     1.54      1.08                          1.47
                     RRu     2.73      2.44                          2.37
                      P       +++         +                           +++
             Between Chi                                             1.52
             Between  df                                                1
             Between  P                                              N.S.
             Btwn(F)  P                                              N.S.

                        Unexposed group (combining nev+ with main levels)
                          nev any   nev cig     Total


                       N        9         6        15
                      NS        6         6        12


                      Wt    87.34    151.34    238.68
                 Het Chi    13.28     23.57     38.37
                 Het  df        8         5        14
                 Het  P      N.S.       ***       ***
               Fixed  RR     1.96      1.66      1.77
                     RRl     1.59      1.42      1.55
                     RRu     2.42      1.95      2.00
                      P       +++       +++       +++
              Random  RR     2.05      1.63      1.87
                     RRl     1.54      1.08      1.47
                     RRu     2.73      2.44      2.37
                      P       +++         +       +++
             Between Chi                         1.52
             Between  df                            1
             Between  P                          N.S.
             Btwn(F)  P                          N.S.


  ________________________________________________________________________________________________________________________
                                            International Evidence on Smoking and COPD, Phase 3, Analysis run on 28-SEP-10

                                                   Table 2 - E - 4 - 7

                                IESCOPD - Meta-analysis of amount smoked : key value (2) 1
                             Any CB, cigarettes (or any product if cigarettes not available)
                                 Excluded studies (and stage at which they were excluded)


1       CLARK COTTON  MEYER REMYJA RUTGER SNYDER SOBRAX     SU TAKEMU  WANG4   WEIR WHICKE ZALACA
2      ALESSA  AMIGO ANDER2 ANDER3 AUERBA BEDNAR BROGGE  CHEN1  CHEN2  CHEN3  CHENG CLEMEN  COCCI  DEAN1 DEJONG DETORR
       DICKIN DONTA1 EKBERG ENSTRO FERRI2 FERRI3  FIDAN FORAST FUKUCH GEIJER GODTFR GULSVI HAMMO2 HARIKK HEDMAN HIGGI4
       HOZAWA ITABAS JACOBS JAENDI JOHANN KACHEL KARAKA KATANC KHOURY    KIM KLAYTO KOJIMA KOTAN2 KRZYZA KULLER    LAI
         LAM1   LAM2   LAM3  LANGE    LEE   LIAW LINDBE   LIU1   LIU2 LUNDB1  MADOR MANNI1 MANNI2 MANNI3 MARAN1 MARAN2
       MARCUS MATHES MENEZ2 MENEZ3 MENEZ4 MENEZ5 MENEZ6 MONTNE   NAWA NIEPSU NIHLEN NILSSO  OMORI   PEAT   PETO  PRATT
        PRICE RENWIC RICCIO  RYDER SARGEA SHAHAB   SHIN SICHLE SPEIZE STERLI  STROM SUTINE  TAGER   TANG   THUN   TODD
       TRUPIN TSUSHI TVERDA VESTBO VIEGI2 VIKGRE VINEIS VOLLM1 VOLLM2 VONHER   WALD  WANG2 WATSON  WEISS WILSO1   XIAO
           XU   YUAN ZIELI1 ZIELI2 ZIETKO
3        KAHN
4        BANG  BECK1  BECK2 BJORNS CERVER COLLEG  DEANE DEMARC DOPICO ENRIGH FINKLE FOXMAN GOLDBE HARDIE  HAYES HIRAYA
           HO  HOUSE HUHTI2 JENSEN JINDA2  JOSHI JOUSI1   KATO  KIRAZ KOTAN1  KUBIK LANGE2 LANGHA LUNDB2 MAGNUS MANFRE
       MELLST MILLER MOLLER NEJJAR OSWAL1 OSWAL2 PANDEY PELKON PEREZP SAWICK SCHWAR SHIMUR  SILVA SOBRAD STJERN SUADIC
       TAGER2 VIEGI1 WOJTYN  WOODS   ZOIA
5       DOLL2 FLETCH HAWTHO HRUBEC RIMING  SHARP    WIG WILSO2
6      ALDERS ANDER1 CHAPMA COATES  DOLL1 EHRLIC FERRI1 HARRIS HIGGI2 HIGGI3 HIGGI6 HOLLA2 HOLLNA HUHTI1 HUHTI3 LAMBER
       LAVECC LEBOWI MENEZ1  MILNE MUELLE   REID TROISI WAGEN2    WEN WILHEL  WOOLF


  ________________________________________________________________________________________________________________________
                                            International Evidence on Smoking and COPD, Phase 3, Analysis run on 28-SEP-10

                                                   Table 2 - E - 4 - 8

                                IESCOPD - Meta-analysis of amount smoked : key value (2) 1
                             Any CB, cigarettes (or any product if cigarettes not available)
                                             Potentially overlapping studies


     REF| REFGP|PRINC|                     OVERLAP|

  URRUTI DEMARC     2         DEMARC/URRUTI/DEMEER
  DONTA2 JACOBS     2  JACOBS/DONTA1/DONTA2/PELKON
   KAHN2   KAHN     2                   KAHN/KAHN2


  ________________________________________________________________________________________________________________________
                                            International Evidence on Smoking and COPD, Phase 3, Analysis run on 28-SEP-10

                                                    Table 2 - E - 5 -

                               IESCOPD - Meta-analysis of amount smoked : key value (2) 10
                             Any CB, cigarettes (or any product if cigarettes not available)


This analysis is restricted to results for:
1) Eligible study on database
2) Outcome CB
3) Current or ever smoking
4) Categorical dose-response data for amount smoked
5) vs never smoking base
6) Key value (scheme 2) = 10
7) Results complete enough for use in meta-analysis

Within each study, results are then selected (in the following order of preference, within each sex) for:
8) SMKSTA  : current, ever
9) UNEXP   : never any, never cigarettes
10) PROD    : cigarettes, cigarettes only, any product
11) For overlapping studies: principal rather than subsidiary studies
and then for single sex results (m, f) in preference to results for both sexes combined (b).

Results adjusted for the most potential confounders are then chosen in Sections -1 to -3
and results adjusted for the least confounders in Sections -4 to -6. (Those least-adjusted results which
actually differ from the most-adjusted are marked 'x' in column X in Section -4)

Section -7 shows excluded studies, together with the stage (as above) at which no qualifying
results were found.

Section -8 lists the potentially overlapping studies which have been included (1=principal, 2=subsidiary),
and any results which would have been included in preference except that they had data not complete enough
for use in meta-analysis. It also lists their significance (yes/no), if known.


  ________________________________________________________________________________________________________________________
                                            International Evidence on Smoking and COPD, Phase 3, Analysis run on 28-SEP-10

                                                   Table 2 - E - 5 - 1

                               IESCOPD - Meta-analysis of amount smoked : key value (2) 10
                             Any CB, cigarettes (or any product if cigarettes not available)
                                                      Most-adjusted


     REF|NRR|SEX|AGEL|AGEH|     REGION|BEGYR|PUBYR|STTYP|ONSET|      DISEAS|ADJ|SMOKSTA|   PRODUCT|    UNEXP|LOW| HI|

   BROWN  10   m   60   69       Eu:UK  1956  1957    CS  Prev CB:diagnosed   0 Current  Cigs only   Nev any  10  19
   DEAN2  10   m   37   67       Eu:UK  1972  1978    CS  Prev  CB:symptoms   1 Current MCigs only   Nev any   8  12
   DEAN2  24   f   37   67       Eu:UK  1972  1978    CS  Prev  CB:symptoms   1 Current MCigs only   Nev any   8  12
  DONTA2   5   m   25   84 Eu:SE/Balkn  1960  1984    Pr   Inc CB:diagnosed   0 Current       Cigs  Nev cigs  10  19
  HAENSZ  57   m   35   74    Eu:Scand  1964  1972    CS  Prev  CB:symptoms   1 Current  Cigs only   Nev any  10  19
  HAENSZ  75   f   35   74    Eu:Scand  1964  1972    CS  Prev  CB:symptoms   1 Current       Cigs   Nev any  10  19
  LINDST  25   b   20   69    Eu:Scand     *  2001    CS  Prev  CB:symptoms   5 Current       Cigs  Nev cigs   5  14
   MEREN   3   b   15   64     Eu:East  1995  2001    CS  Prev  CB:symptoms   4 Current       Cigs  Nev cigs   5  14
  OGILVI  16   m   30   99       Eu:UK  1955  1957    CC  Prev  CB:symptoms   0 Current       Cigs   Nev any   6  15
  OGILVI  28   f   30   99       Eu:UK  1955  1957    CC  Prev  CB:symptoms   0 Current       Cigs   Nev any   6  15


  ________________________________________________________________________________________________________________________
                                            International Evidence on Smoking and COPD, Phase 3, Analysis run on 28-SEP-10

                                                   Table 2 - E - 5 - 2

                               IESCOPD - Meta-analysis of amount smoked : key value (2) 10
                             Any CB, cigarettes (or any product if cigarettes not available)
                                                      Most-adjusted


                        Number Exposed  Non-exposed
 REF    NRR SEX ADJ     Case    Cont    Case    Cont      RR        95.00%CI
 BROWN  10  m   0         83     188      15      76      2.24 (  1.21-   4.12)
 DEAN2  10  m   1         37       -      39       -      3.09 (  1.94-   4.92)
 DEAN2  24  f   1         28       -      80       -      1.87 (  1.21-   2.90)
 Subtotal DEAN2                                           2.37 (  1.72-   3.25)
*DONTA2 5   m   0         15      76       8     127      3.13 (  1.39-   7.04)
 HAENSZ 57  m   1         53       -      27       -      3.80 (  2.32-   6.23)
 HAENSZ 75  f   1         26       -      97       -      4.75 (  3.01-   7.50)
 Subtotal HAENSZ                                          4.29 (  3.07-   5.99)
 LINDST 25  b   5          -       -     494       -      1.69 (  1.42-   2.02)
 MEREN  3   b   4          -       -       -       -      2.47 (  1.79-   3.56)
 OGILVI 16  m   0         96      69      11      29      3.67 (  1.72-   7.84)
 OGILVI 28  f   0         54      28      86     160      3.59 (  2.12-   6.07)
 Subtotal OGILVI                                          3.61 (  2.34-   5.57)
Partial Totals           392     361     857     392
*prospective study


 REF    NRR SEX ADJ             Ys       Ws       Qs       Ps
 BROWN  10  m   0              0.81    10.29     0.00       0.01
 DEAN2  10  m   1              1.13    17.74     1.65       0.00
 DEAN2  24  f   1              0.63    20.11     0.78       0.00
 Subtotal DEAN2                0.86    37.85     2.43
*DONTA2 5   m   0              1.14     5.86     0.60       0.01
 HAENSZ 57  m   1              1.34    15.75     4.13       0.00
 HAENSZ 75  f   1              1.56    18.44     9.97       0.00
 Subtotal HAENSZ               1.46    34.18    14.10
 LINDST 25  b   5              0.52   123.70    11.00       0.00
 MEREN  3   b   4              0.90    32.51     0.22       0.00
 OGILVI 16  m   0              1.30     6.65     1.51       0.00
 OGILVI 28  f   0              1.28    13.87     2.87       0.00
 Subtotal OGILVI               1.28    20.52     4.38

                       N       10
                      NS        7


                      Wt   264.92
                 Het Chi    32.72
                 Het  df        9
                 Het  P       ***
               Fixed  RR     2.28
                     RRl     2.02
                     RRu     2.57
                      P       +++
              Random  RR     2.79
                     RRl     2.13
                     RRu     3.64
                      P       +++
               Asymm  P        **


  ________________________________________________________________________________________________________________________
                                            International Evidence on Smoking and COPD, Phase 3, Analysis run on 28-SEP-10

                                                   Table 2 - E - 5 - 3

                               IESCOPD - Meta-analysis of amount smoked : key value (2) 10
                             Any CB, cigarettes (or any product if cigarettes not available)
                                                      Most-adjusted


                       N       10
                      NS        7


                      Wt   264.92
                 Het Chi    32.72
                 Het  df        9
                 Het  P       ***
               Fixed  RR     2.28
                     RRl     2.02
                     RRu     2.57
                      P       +++
              Random  RR     2.79
                     RRl     2.13
                     RRu     3.64
                      P       +++
               Asymm  P        **

                                   Sex
                             both      male    female     Total


                       N        2         5         3        10
                      NS        2         5         3        10


                      Wt   156.21     56.29     52.41    264.92
                 Het Chi     3.71      1.92      8.79     32.72
                 Het  df        1         4         2         9
                 Het  P       (*)      N.S.         *       ***
               Fixed  RR     1.83      3.15      3.08      2.28
                     RRl     1.56      2.43      2.35      2.02
                     RRu     2.14      4.10      4.04      2.57
                      P       +++       +++       +++       +++
              Random  RR     1.98      3.15      3.15      2.79
                     RRl     1.37      2.43      1.78      2.13
                     RRu     2.86      4.10      5.58      3.64
                      P       +++       +++       +++       +++
             Between Chi                                  18.30
             Between  df                                      2
             Between  P                                     ***
             Btwn(F)  P                                     (*)

                                        Continent
                            NAmer    Europe      Asia  oth/mult     Total


                       N                 10                            10
                      NS                  7                             7


                      Wt             264.92                        264.92
                 Het Chi              32.72                         32.72
                 Het  df                  9                             9
                 Het  P                 ***                           ***
               Fixed  RR               2.28                          2.28
                     RRl               2.02                          2.02
                     RRu               2.57                          2.57
                      P                 +++                           +++
              Random  RR               2.79                          2.79
                     RRl               2.13                          2.13
                     RRu               3.64                          3.64
                      P                 +++                           +++
             Between Chi
             Between  df
             Between  P                                              N.S.
             Btwn(F)  P                                              N.S.


  ________________________________________________________________________________________________________________________
                                            International Evidence on Smoking and COPD, Phase 3, Analysis run on 28-SEP-10

                                                   Table 2 - E - 5 - 3

                               IESCOPD - Meta-analysis of amount smoked : key value (2) 10
                             Any CB, cigarettes (or any product if cigarettes not available)
                                                      Most-adjusted
                               Study type
                               CC        Pr        CS     Total


                       N        2         1         7        10
                      NS        1         1         5         7


                      Wt    20.52      5.86    238.53    264.92
                 Het Chi     0.00      0.00     27.21     32.72
                 Het  df        1         0         6         9
                 Het  P      N.S.      N.S.       ***       ***
               Fixed  RR     3.61      3.13      2.17      2.28
                     RRl     2.34      1.39      1.91      2.02
                     RRu     5.57      7.04      2.47      2.57
                      P       +++        ++       +++       +++
              Random  RR     3.61      3.13      2.61      2.79
                     RRl     2.34      1.39      1.91      2.13
                     RRu     5.57      7.04      3.57      3.64
                      P       +++        ++       +++       +++
             Between Chi                                   5.51
             Between  df                                      2
             Between  P                                     (*)
             Btwn(F)  P                                    N.S.

                           CB subtype
                             mort     sympt     other     Total


                       N                  8         2        10
                      NS                  5         2         7


                      Wt             248.77     16.15    264.92
                 Het Chi              32.11      0.42     32.72
                 Het  df                  7         1         9
                 Het  P                 ***      N.S.       ***
               Fixed  RR               2.26      2.53      2.28
                     RRl               2.00      1.55      2.02
                     RRu               2.56      4.12      2.57
                      P                 +++       +++       +++
              Random  RR               2.84      2.53      2.79
                     RRl               2.09      1.55      2.13
                     RRu               3.86      4.12      3.64
                      P                 +++       +++       +++
             Between Chi                                   0.19
             Between  df                                      1
             Between  P                                    N.S.
             Btwn(F)  P                                    N.S.

                             Smoking product
                              any      cigs  cigsonly     Total


                       N                  6         4        10
                      NS                  5         3         8


                      Wt             201.03     63.89    264.92
                 Het Chi              25.72      5.20     32.72
                 Het  df                  5         3         9
                 Het  P                 ***      N.S.       ***
               Fixed  RR               2.17      2.64      2.28
                     RRl               1.89      2.06      2.02
                     RRu               2.50      3.37      2.57
                      P                 +++       +++       +++
              Random  RR               2.93      2.65      2.79
                     RRl               1.97      1.91      2.13
                     RRu               4.35      3.67      3.64
                      P                 +++       +++       +++
             Between Chi                                   1.80
             Between  df                                      1
             Between  P                                    N.S.
             Btwn(F)  P                                    N.S.
  ________________________________________________________________________________________________________________________
                                            International Evidence on Smoking and COPD, Phase 3, Analysis run on 28-SEP-10

                                                   Table 2 - E - 5 - 3

                               IESCOPD - Meta-analysis of amount smoked : key value (2) 10
                             Any CB, cigarettes (or any product if cigarettes not available)
                                                      Most-adjusted
                                     Unexposed group
                          nev any   nev cig  nev+ any  nev+ cig     Total


                       N        7         3                            10
                      NS        4         3                             7


                      Wt   102.85    162.07                        264.92
                 Het Chi    10.72      5.34                         32.72
                 Het  df        6         2                             9
                 Het  P       (*)       (*)                           ***
               Fixed  RR     3.12      1.86                          2.28
                     RRl     2.57      1.60                          2.02
                     RRu     3.78      2.18                          2.57
                      P       +++       +++                           +++
              Random  RR     3.13      2.11                          2.79
                     RRl     2.41      1.50                          2.13
                     RRu     4.07      2.99                          3.64
                      P       +++       +++                           +++
             Between Chi                                            16.65
             Between  df                                                1
             Between  P                                               ***
             Btwn(F)  P                                                 *

                        Unexposed group (combining nev+ with main levels)
                          nev any   nev cig     Total


                       N        7         3        10
                      NS        4         3         7


                      Wt   102.85    162.07    264.92
                 Het Chi    10.72      5.34     32.72
                 Het  df        6         2         9
                 Het  P       (*)       (*)       ***
               Fixed  RR     3.12      1.86      2.28
                     RRl     2.57      1.60      2.02
                     RRu     3.78      2.18      2.57
                      P       +++       +++       +++
              Random  RR     3.13      2.11      2.79
                     RRl     2.41      1.50      2.13
                     RRu     4.07      2.99      3.64
                      P       +++       +++       +++
             Between Chi                        16.65
             Between  df                            1
             Between  P                           ***
             Btwn(F)  P                             *


  ________________________________________________________________________________________________________________________
                                            International Evidence on Smoking and COPD, Phase 3, Analysis run on 28-SEP-10

                                                   Table 2 - E - 5 - 4

                               IESCOPD - Meta-analysis of amount smoked : key value (2) 10
                             Any CB, cigarettes (or any product if cigarettes not available)
                                                      Least-adjusted


     REF|NRR|X|SEX|AGEL|AGEH|     REGION|BEGYR|PUBYR|STTYP|ONSET|      DISEAS|ADJ|SMOKSTA|   PRODUCT|    UNEXP|LOW| HI|

   BROWN  10     m   60   69       Eu:UK  1956  1957    CS  Prev CB:diagnosed   0 Current  Cigs only   Nev any  10  19
   DEAN2  10     m   37   67       Eu:UK  1972  1978    CS  Prev  CB:symptoms   1 Current MCigs only   Nev any   8  12
   DEAN2  24     f   37   67       Eu:UK  1972  1978    CS  Prev  CB:symptoms   1 Current MCigs only   Nev any   8  12
  DONTA2   5     m   25   84 Eu:SE/Balkn  1960  1984    Pr   Inc CB:diagnosed   0 Current       Cigs  Nev cigs  10  19
  HAENSZ  52 x   m   35   74    Eu:Scand  1964  1972    CS  Prev  CB:symptoms   0 Current  Cigs only   Nev any  10  19
  HAENSZ  72 x   f   35   74    Eu:Scand  1964  1972    CS  Prev  CB:symptoms   0 Current       Cigs   Nev any  10  19
  LINDST  25     b   20   69    Eu:Scand     *  2001    CS  Prev  CB:symptoms   5 Current       Cigs  Nev cigs   5  14
   MEREN   3     b   15   64     Eu:East  1995  2001    CS  Prev  CB:symptoms   4 Current       Cigs  Nev cigs   5  14
  OGILVI  16     m   30   99       Eu:UK  1955  1957    CC  Prev  CB:symptoms   0 Current       Cigs   Nev any   6  15
  OGILVI  28     f   30   99       Eu:UK  1955  1957    CC  Prev  CB:symptoms   0 Current       Cigs   Nev any   6  15


  ________________________________________________________________________________________________________________________
                                            International Evidence on Smoking and COPD, Phase 3, Analysis run on 28-SEP-10

                                                   Table 2 - E - 5 - 5

                               IESCOPD - Meta-analysis of amount smoked : key value (2) 10
                             Any CB, cigarettes (or any product if cigarettes not available)
                                                      Least-adjusted


                        Number Exposed  Non-exposed
 REF    NRR SEX ADJ     Case    Cont    Case    Cont      RR        95.00%CI
 BROWN  10  m   0         83     188      15      76      2.24 (  1.21-   4.12)
 DEAN2  10  m   1         37       -      39       -      3.09 (  1.94-   4.92)
 DEAN2  24  f   1         28       -      80       -      1.87 (  1.21-   2.90)
 Subtotal DEAN2                                           2.37 (  1.72-   3.25)
*DONTA2 5   m   0         15      76       8     127      3.13 (  1.39-   7.04)
 HAENSZ 52  m   0         53    1656      27    2742      3.25 (  2.04-   5.19)
 HAENSZ 72  f   0         26     745      97   11536      4.15 (  2.68-   6.44)
 Subtotal HAENSZ                                          3.70 (  2.69-   5.10)
 LINDST 25  b   5          -       -     494       -      1.69 (  1.42-   2.02)
 MEREN  3   b   4          -       -       -       -      2.47 (  1.79-   3.56)
 OGILVI 16  m   0         96      69      11      29      3.67 (  1.72-   7.84)
 OGILVI 28  f   0         54      28      86     160      3.59 (  2.12-   6.07)
 Subtotal OGILVI                                          3.61 (  2.34-   5.57)
Partial Totals           392    2762     857   14670
*prospective study


 REF    NRR SEX ADJ             Ys       Ws       Qs       Ps
 BROWN  10  m   0              0.81    10.29     0.00       0.01
 DEAN2  10  m   1              1.13    17.74     1.79       0.00
 DEAN2  24  f   1              0.63    20.11     0.68       0.00
 Subtotal DEAN2                0.86    37.85     2.48
*DONTA2 5   m   0              1.14     5.86     0.65       0.01
 HAENSZ 52  m   0              1.18    17.58     2.39       0.00
 HAENSZ 72  f   0              1.42    19.92     7.49       0.00
 Subtotal HAENSZ               1.31    37.50     9.87
 LINDST 25  b   5              0.52   123.70    10.08       0.00
 MEREN  3   b   4              0.90    32.51     0.29       0.00
 OGILVI 16  m   0              1.30     6.65     1.59       0.00
 OGILVI 28  f   0              1.28    13.87     3.03       0.00
 Subtotal OGILVI               1.28    20.52     4.62

                       N       10
                      NS        7


                      Wt   268.24
                 Het Chi    27.99
                 Het  df        9
                 Het  P       ***
               Fixed  RR     2.25
                     RRl     1.99
                     RRu     2.53
                      P       +++
              Random  RR     2.69
                     RRl     2.11
                     RRu     3.44
                      P       +++
               Asymm  P        **


  ________________________________________________________________________________________________________________________
                                            International Evidence on Smoking and COPD, Phase 3, Analysis run on 28-SEP-10

                                                   Table 2 - E - 5 - 6

                               IESCOPD - Meta-analysis of amount smoked : key value (2) 10
                             Any CB, cigarettes (or any product if cigarettes not available)
                                                      Least-adjusted


                       N       10
                      NS        7


                      Wt   268.24
                 Het Chi    27.99
                 Het  df        9
                 Het  P       ***
               Fixed  RR     2.25
                     RRl     1.99
                     RRu     2.53
                      P       +++
              Random  RR     2.69
                     RRl     2.11
                     RRu     3.44
                      P       +++
               Asymm  P        **

                                   Sex
                             both      male    female     Total


                       N        2         5         3        10
                      NS        2         5         3        10


                      Wt   156.21     58.13     53.90    268.24
                 Het Chi     3.71      1.29      7.03     27.99
                 Het  df        1         4         2         9
                 Het  P       (*)      N.S.         *       ***
               Fixed  RR     1.83      3.03      2.97      2.25
                     RRl     1.56      2.34      2.27      1.99
                     RRu     2.14      3.91      3.88      2.53
                      P       +++       +++       +++       +++
              Random  RR     1.98      3.03      3.01      2.69
                     RRl     1.37      2.34      1.82      2.11
                     RRu     2.86      3.91      4.99      3.44
                      P       +++       +++       +++       +++
             Between Chi                                  15.96
             Between  df                                      2
             Between  P                                     ***
             Btwn(F)  P                                     (*)

                                        Continent
                            NAmer    Europe      Asia  oth/mult     Total


                       N                 10                            10
                      NS                  7                             7


                      Wt             268.24                        268.24
                 Het Chi              27.99                         27.99
                 Het  df                  9                             9
                 Het  P                 ***                           ***
               Fixed  RR               2.25                          2.25
                     RRl               1.99                          1.99
                     RRu               2.53                          2.53
                      P                 +++                           +++
              Random  RR               2.69                          2.69
                     RRl               2.11                          2.11
                     RRu               3.44                          3.44
                      P                 +++                           +++
             Between Chi
             Between  df
             Between  P                                              N.S.
             Btwn(F)  P                                              N.S.


  ________________________________________________________________________________________________________________________
                                            International Evidence on Smoking and COPD, Phase 3, Analysis run on 28-SEP-10

                                                   Table 2 - E - 5 - 6

                               IESCOPD - Meta-analysis of amount smoked : key value (2) 10
                             Any CB, cigarettes (or any product if cigarettes not available)
                                                      Least-adjusted
                               Study type
                               CC        Pr        CS     Total


                       N        2         1         7        10
                      NS        1         1         5         7


                      Wt    20.52      5.86    241.86    268.24
                 Het Chi     0.00      0.00     22.16     27.99
                 Het  df        1         0         6         9
                 Het  P      N.S.      N.S.        **       ***
               Fixed  RR     3.61      3.13      2.14      2.25
                     RRl     2.34      1.39      1.89      1.99
                     RRu     5.57      7.04      2.43      2.53
                      P       +++        ++       +++       +++
              Random  RR     3.61      3.13      2.51      2.69
                     RRl     2.34      1.39      1.90      2.11
                     RRu     5.57      7.04      3.31      3.44
                      P       +++        ++       +++       +++
             Between Chi                                   5.83
             Between  df                                      2
             Between  P                                     (*)
             Btwn(F)  P                                    N.S.

                           CB subtype
                             mort     sympt     other     Total


                       N                  8         2        10
                      NS                  5         2         7


                      Wt             252.09     16.15    268.24
                 Het Chi              27.33      0.42     27.99
                 Het  df                  7         1         9
                 Het  P                 ***      N.S.       ***
               Fixed  RR               2.23      2.53      2.25
                     RRl               1.97      1.55      1.99
                     RRu               2.52      4.12      2.53
                      P                 +++       +++       +++
              Random  RR               2.73      2.53      2.69
                     RRl               2.06      1.55      2.11
                     RRu               3.61      4.12      3.44
                      P                 +++       +++       +++
             Between Chi                                   0.24
             Between  df                                      1
             Between  P                                    N.S.
             Btwn(F)  P                                    N.S.

                             Smoking product
                              any      cigs  cigsonly     Total


                       N                  6         4        10
                      NS                  5         3         8


                      Wt             202.51     65.73    268.24
                 Het Chi              22.78      3.80     27.99
                 Het  df                  5         3         9
                 Het  P                 ***      N.S.       ***
               Fixed  RR               2.16      2.55      2.25
                     RRl               1.88      2.01      1.99
                     RRu               2.48      3.25      2.53
                      P                 +++       +++       +++
              Random  RR               2.85      2.56      2.69
                     RRl               1.96      1.94      2.11
                     RRu               4.13      3.36      3.44
                      P                 +++       +++       +++
             Between Chi                                   1.41
             Between  df                                      1
             Between  P                                    N.S.
             Btwn(F)  P                                    N.S.
  ________________________________________________________________________________________________________________________
                                            International Evidence on Smoking and COPD, Phase 3, Analysis run on 28-SEP-10

                                                   Table 2 - E - 5 - 6

                               IESCOPD - Meta-analysis of amount smoked : key value (2) 10
                             Any CB, cigarettes (or any product if cigarettes not available)
                                                      Least-adjusted
                                     Unexposed group
                          nev any   nev cig  nev+ any  nev+ cig     Total


                       N        7         3                            10
                      NS        4         3                             7


                      Wt   106.17    162.07                        268.24
                 Het Chi     8.32      5.34                         27.99
                 Het  df        6         2                             9
                 Het  P      N.S.       (*)                           ***
               Fixed  RR     2.99      1.86                          2.25
                     RRl     2.47      1.60                          1.99
                     RRu     3.62      2.18                          2.53
                      P       +++       +++                           +++
              Random  RR     3.00      2.11                          2.69
                     RRl     2.39      1.50                          2.11
                     RRu     3.76      2.99                          3.44
                      P       +++       +++                           +++
             Between Chi                                            14.32
             Between  df                                                1
             Between  P                                               ***
             Btwn(F)  P                                                 *

                        Unexposed group (combining nev+ with main levels)
                          nev any   nev cig     Total


                       N        7         3        10
                      NS        4         3         7


                      Wt   106.17    162.07    268.24
                 Het Chi     8.32      5.34     27.99
                 Het  df        6         2         9
                 Het  P      N.S.       (*)       ***
               Fixed  RR     2.99      1.86      2.25
                     RRl     2.47      1.60      1.99
                     RRu     3.62      2.18      2.53
                      P       +++       +++       +++
              Random  RR     3.00      2.11      2.69
                     RRl     2.39      1.50      2.11
                     RRu     3.76      2.99      3.44
                      P       +++       +++       +++
             Between Chi                        14.32
             Between  df                            1
             Between  P                           ***
             Btwn(F)  P                             *


  ________________________________________________________________________________________________________________________
                                            International Evidence on Smoking and COPD, Phase 3, Analysis run on 28-SEP-10

                                                   Table 2 - E - 5 - 7

                               IESCOPD - Meta-analysis of amount smoked : key value (2) 10
                             Any CB, cigarettes (or any product if cigarettes not available)
                                 Excluded studies (and stage at which they were excluded)


1       CLARK COTTON  MEYER REMYJA RUTGER SNYDER SOBRAX     SU TAKEMU  WANG4   WEIR WHICKE ZALACA
2      ALESSA  AMIGO ANDER2 ANDER3 AUERBA BEDNAR BROGGE  CHEN1  CHEN2  CHEN3  CHENG CLEMEN  COCCI  DEAN1 DEJONG DETORR
       DICKIN DONTA1 EKBERG ENSTRO FERRI2 FERRI3  FIDAN FORAST FUKUCH GEIJER GODTFR GULSVI HAMMO2 HARIKK HEDMAN HIGGI4
       HOZAWA ITABAS JACOBS JAENDI JOHANN KACHEL KARAKA KATANC KHOURY    KIM KLAYTO KOJIMA KOTAN2 KRZYZA KULLER    LAI
         LAM1   LAM2   LAM3  LANGE    LEE   LIAW LINDBE   LIU1   LIU2 LUNDB1  MADOR MANNI1 MANNI2 MANNI3 MARAN1 MARAN2
       MARCUS MATHES MENEZ2 MENEZ3 MENEZ4 MENEZ5 MENEZ6 MONTNE   NAWA NIEPSU NIHLEN NILSSO  OMORI   PEAT   PETO  PRATT
        PRICE RENWIC RICCIO  RYDER SARGEA SHAHAB   SHIN SICHLE SPEIZE STERLI  STROM SUTINE  TAGER   TANG   THUN   TODD
       TRUPIN TSUSHI TVERDA VESTBO VIEGI2 VIKGRE VINEIS VOLLM1 VOLLM2 VONHER   WALD  WANG2 WATSON  WEISS WILSO1   XIAO
           XU   YUAN ZIELI1 ZIELI2 ZIETKO
3        KAHN
4        BANG  BECK1  BECK2 BJORNS CERVER COLLEG  DEANE DEMARC DOPICO ENRIGH FINKLE FOXMAN GOLDBE HARDIE  HAYES HIRAYA
           HO  HOUSE HUHTI2 JENSEN JINDA2  JOSHI JOUSI1   KATO  KIRAZ KOTAN1  KUBIK LANGE2 LANGHA LUNDB2 MAGNUS MANFRE
       MELLST MILLER MOLLER NEJJAR OSWAL1 OSWAL2 PANDEY PELKON PEREZP SAWICK SCHWAR SHIMUR  SILVA SOBRAD STJERN SUADIC
       TAGER2 VIEGI1 WOJTYN  WOODS   ZOIA
5       DOLL2 FLETCH HAWTHO HRUBEC RIMING  SHARP    WIG WILSO2
6      ALDERS ANDER1   BEST CHAPMA COATES  DOLL1 EHRLIC FERRI1 HARRIS HIGGI2 HIGGI3 HIGGI6 HOLLA2 HOLLNA HUCHON HUHTI1
       HUHTI3  KAHN2 LAMBER LAVECC LEBOWI MENEZ1  MILNE MUELLE   REID TROISI URRUTI WAGEN2    WEN WILHEL  WOOLF YAMAGU


  ________________________________________________________________________________________________________________________
                                            International Evidence on Smoking and COPD, Phase 3, Analysis run on 28-SEP-10

                                                   Table 2 - E - 5 - 8

                               IESCOPD - Meta-analysis of amount smoked : key value (2) 10
                             Any CB, cigarettes (or any product if cigarettes not available)
                                             Potentially overlapping studies


     REF| REFGP|PRINC|                     OVERLAP|

  DONTA2 JACOBS     2  JACOBS/DONTA1/DONTA2/PELKON


  ________________________________________________________________________________________________________________________
                                            International Evidence on Smoking and COPD, Phase 3, Analysis run on 28-SEP-10

                                                    Table 2 - E - 6 -

                               IESCOPD - Meta-analysis of amount smoked : key value (2) 20
                             Any CB, cigarettes (or any product if cigarettes not available)


This analysis is restricted to results for:
1) Eligible study on database
2) Outcome CB
3) Current or ever smoking
4) Categorical dose-response data for amount smoked
5) vs never smoking base
6) Key value (scheme 2) = 20
7) Results complete enough for use in meta-analysis

Within each study, results are then selected (in the following order of preference, within each sex) for:
8) SMKSTA  : current, ever
9) UNEXP   : never any, never cigarettes
10) PROD    : cigarettes, cigarettes only, any product
11) For overlapping studies: principal rather than subsidiary studies
and then for single sex results (m, f) in preference to results for both sexes combined (b).

Results adjusted for the most potential confounders are then chosen in Sections -1 to -3
and results adjusted for the least confounders in Sections -4 to -6. (Those least-adjusted results which
actually differ from the most-adjusted are marked 'x' in column X in Section -4)

Section -7 shows excluded studies, together with the stage (as above) at which no qualifying
results were found.

Section -8 lists the potentially overlapping studies which have been included (1=principal, 2=subsidiary),
and any results which would have been included in preference except that they had data not complete enough
for use in meta-analysis. It also lists their significance (yes/no), if known.


  ________________________________________________________________________________________________________________________
                                            International Evidence on Smoking and COPD, Phase 3, Analysis run on 28-SEP-10

                                                   Table 2 - E - 6 - 1

                               IESCOPD - Meta-analysis of amount smoked : key value (2) 20
                             Any CB, cigarettes (or any product if cigarettes not available)
                                                      Most-adjusted


     REF|NRR|SEX|AGEL|AGEH|     REGION|BEGYR|PUBYR|STTYP|ONSET|      DISEAS|ADJ|SMOKSTA|   PRODUCT|    UNEXP|LOW| HI|

  ALDERS  12   m   35   74       Eu:UK  1977  1985    CC  Prev CB:diagnosed   1    Ever MCigs only   Nev any  18  27
  ALDERS  17   f   35   74       Eu:UK  1977  1985    CC  Prev CB:diagnosed   1    Ever MCigs only   Nev any  18  27
  ANDER1  41   m   25   74   Am:Canada  1963  1965    CS  Prev  CB:symptoms   1 Current       Cigs   Nev any  15  24
  ANDER1  46   f   25   74   Am:Canada  1963  1965    CS  Prev  CB:symptoms   1 Current       Cigs   Nev any  15  24
  CHAPMA  21   m   15   99      Am:USA  1976  1985    CS  Prev  CB:symptoms   1 Current       Cigs  Nev cigs  20  20
  CHAPMA  29   f   15   99      Am:USA  1976  1985    CS  Prev  CB:symptoms   1 Current       Cigs  Nev cigs  20  20
  COATES  10   b   40   64      Am:USA  1962  1965    CS  Prev  CB:symptoms   0 Current       Cigs  Nev cigs  15  24
   DEAN2  12   m   37   67       Eu:UK  1972  1978    CS  Prev  CB:symptoms   1 Current MCigs only   Nev any  18  22
   DEAN2  26   f   37   67       Eu:UK  1972  1978    CS  Prev  CB:symptoms   1 Current MCigs only   Nev any  18  22
   DOLL1  17   m   20   99       Eu:UK  1951  1994    Pr   Inc CB:mortality   1 Current  Cigs only   Nev any  15  24
  DONTA2   6   m   25   84 Eu:SE/Balkn  1960  1984    Pr   Inc CB:diagnosed   0 Current       Cigs  Nev cigs  20  29
  FERRI1 142   m   25   74      Am:USA  1961  1971    CS  Prev  CB:symptoms   1 Current       Cigs   Nev any  11  20
  FERRI1 101   f   25   74      Am:USA  1961  1971    CS  Prev  CB:symptoms   1 Current       Cigs   Nev any  11  20
  HARRIS   5   m   15   60      Africa     *  1993    CS  Prev  CB:symptoms   0 Current        Any   Nev any  15  24
  HOLLA2   5   m   40   59      Am:USA  1962  1965    CS  Prev  CB:symptoms   0 Current        Any   Nev any  15  24
  HOLLNA   5   m   40   40    Eu:Scand  1976  1983    CS  Prev  CB:symptoms   0 Current        Any   Nev any  15  24
  HOLLNA  13   f   40   40    Eu:Scand  1976  1983    CS  Prev  CB:symptoms   0 Current        Any   Nev any  15  24
  HUHTI1  51   m   40   64    Eu:Scand  1961  1965    CS  Prev  CB:symptoms   1 Current       Cigs   Nev any  15  24
  HUHTI3  29   m   25   69    Eu:Scand  1968  1978    CS  Prev  CB:symptoms   1 Current        Any   Nev any  15  24
  LAMBER  13   m   35   69       Eu:UK  1965  1970    CS  Prev  CB:symptoms   1 Current       Cigs  Nev cigs  20  20
  LAMBER  59   f   35   69       Eu:UK  1965  1970    CS  Prev  CB:symptoms   1 Current       Cigs   Nev any  20  20
  MUELLE  72   f   20   69      Am:USA  1967  1971    CS  Prev  CB:symptoms   1 Current       Cigs   Nev any  15  24
  OGILVI  17   m   30   99       Eu:UK  1955  1957    CC  Prev  CB:symptoms   0 Current       Cigs   Nev any  16  25
  OGILVI  51   f   30   99       Eu:UK  1955  1957    CC  Prev  CB:symptoms   0    Ever       Cigs   Nev any  16  25
    REID  13   m   35   74      Am:USA  1962  1966    CS  Prev  CB:symptoms   1 Current       Cigs  Nev cigs  20  20
    REID  29   f   35   74      Am:USA  1962  1966    CS  Prev  CB:symptoms   1 Current       Cigs  Nev cigs  20  20
  TROISI   5   f   34   69      Am:USA  1980  1995    Pr   Inc CB:diagnosed   1 Current       Cigs  Nev cigs  15  25
  WAGEN2   5   b   21   68     Eu:West  2001  2004    CC  Prev  CB:symptoms   0    Ever       Cigs  Nev cigs  11  20
     WEN   9   m   35   99   Asia:FarE  1982  2004    Pr   Inc CB:mortality   1 Current       Cigs  Nev cigs  11  20
   WOOLF   5   f   25   54   Am:Canada     *  1974    CS  Prev  CB:symptoms   0 Current       Cigs  Nev cigs  11  20


  ________________________________________________________________________________________________________________________
                                            International Evidence on Smoking and COPD, Phase 3, Analysis run on 28-SEP-10

                                                   Table 2 - E - 6 - 2

                               IESCOPD - Meta-analysis of amount smoked : key value (2) 20
                             Any CB, cigarettes (or any product if cigarettes not available)
                                                      Most-adjusted


                        Number Exposed  Non-exposed
 REF    NRR SEX ADJ     Case    Cont    Case    Cont      RR        95.00%CI
 ALDERS 12  m   1         72       -      31       -      1.96 (  1.15-   3.34)
 ALDERS 17  f   1        125       -     111       -      3.12 (  2.17-   4.49)
 Subtotal ALDERS                                          2.69 (  1.99-   3.64)
 ANDER1 41  m   1         16       -       4       -      7.03 (  2.11-  23.42)
 ANDER1 46  f   1          5       -      15       -      2.56 (  0.83-   7.87)
 Subtotal ANDER1                                          4.10 (  1.80-   9.32)
 CHAPMA 21  m   1         72       -      35       -      5.52 (  3.58-   8.51)
 CHAPMA 29  f   1         48       -      48       -      6.07 (  3.98-   9.27)
 Subtotal CHAPMA                                          5.80 (  4.28-   7.84)
 COATES 10  b   0         69     333      21     515      5.08 (  3.06-   8.44)
 DEAN2  12  m   1         61       -      39       -      2.57 (  1.70-   3.89)
 DEAN2  26  f   1         69       -      80       -      4.59 (  3.29-   6.42)
 Subtotal DEAN2                                           3.65 (  2.81-   4.73)
*DOLL1  17  m   1         37       -       3       -     12.80 (  3.74-  43.85)
*DONTA2 6   m   0         19      67       8     127      4.50 (  2.08-   9.73)
 FERRI1 142 m   1         44       -      10       -      3.25 (  1.53-   6.91)
 FERRI1 101 f   1         17       -      35       -      2.75 (  1.45-   5.22)
 Subtotal FERRI1                                          2.95 (  1.81-   4.81)
 HARRIS 5   m   0          3       2       6     518    129.50 ( 18.21- 921.07)
 HOLLA2 5   m   0         38     131       5      84      4.87 (  1.84-  12.88)
 HOLLNA 5   m   0         72      89      10     101      8.17 (  3.98-  16.79)
 HOLLNA 13  f   0         42      86      14     217      7.57 (  3.93-  14.56)
 Subtotal HOLLNA                                          7.84 (  4.83-  12.72)
 HUHTI1 51  m   1         79       -       7       -     10.12 (  4.39-  23.29)
 HUHTI3 29  m   1         79       -      28       -      4.33 (  2.49-   7.53)
 LAMBER 13  m   1         41       -      17       -      2.99 (  1.67-   5.36)
 LAMBER 59  f   1         29       -      54       -      5.14 (  3.12-   8.47)
 Subtotal LAMBER                                          4.09 (  2.80-   5.97)
 MUELLE 72  f   1         14       -       3       -     15.14 (  3.04-  75.48)
 OGILVI 17  m   0         66      44      11      29      3.95 (  1.79-   8.73)
 OGILVI 51  f   0         24      13      86     160      3.43 (  1.67-   7.08)
 Subtotal OGILVI                                          3.66 (  2.15-   6.25)
 REID   13  m   1         66       -      16       -      6.07 (  3.46-  10.62)
 REID   29  f   1         19       -      37       -      4.30 (  2.38-   7.75)
 Subtotal REID                                            5.15 (  3.43-   7.74)
*TROISI 5   f   1          -       -       -       -      2.63 (  2.13-   3.13)
 WAGEN2 5   b   0        111    1084     111    2136      1.97 (  1.50-   2.59)
*WEN    9   m   1          -       -       7       -      2.03 (  0.57-   7.23)
 WOOLF  5   f   0         25      67      10     173      6.46 (  2.94-  14.16)
Partial Totals          1362    1916     862    4060
*prospective study


 REF    NRR SEX ADJ             Ys       Ws       Qs       Ps
 ALDERS 12  m   1              0.67    13.52     4.78       0.01
 ALDERS 17  f   1              1.14    29.06     0.49       0.00
 Subtotal ALDERS               0.99    42.58     5.28
 ANDER1 41  m   1              1.95     2.65     1.23       0.00
 ANDER1 46  f   1              0.94     3.04     0.33       0.10
 Subtotal ANDER1               1.41     5.69     1.56
 CHAPMA 21  m   1              1.71    20.49     3.98       0.00
 CHAPMA 29  f   1              1.80    21.49     6.16       0.00
 Subtotal CHAPMA               1.76    41.99    10.14
 COATES 10  b   0              1.63    14.91     1.91       0.00
 DEAN2  12  m   1              0.94    22.42     2.35       0.00
 DEAN2  26  f   1              1.52    34.38     2.25       0.00
 Subtotal DEAN2                1.29    56.81     4.61
*DOLL1  17  m   1              2.55     2.54     4.16       0.00
*DONTA2 6   m   0              1.50     6.46     0.36       0.00
 FERRI1 142 m   1              1.18     6.76     0.05       0.00
 FERRI1 101 f   1              1.01     9.36     0.62       0.00
 Subtotal FERRI1               1.08    16.12     0.67
 HARRIS 5   m   0              4.86     1.00    12.90       0.00
 HOLLA2 5   m   0              1.58     4.07     0.41       0.00
 HOLLNA 5   m   0              2.10     7.41     5.14       0.00
 HOLLNA 13  f   0              2.02     8.97     5.13       0.00
 Subtotal HOLLNA               2.06    16.38    10.27
 HUHTI1 51  m   1              2.31     5.52     6.04       0.00
 HUHTI3 29  m   1              1.47    12.55     0.49       0.00
 LAMBER 13  m   1              1.10    11.30     0.34       0.00
 LAMBER 59  f   1              1.64    15.41     2.10       0.00
  ________________________________________________________________________________________________________________________
                                            International Evidence on Smoking and COPD, Phase 3, Analysis run on 28-SEP-10

                                                   Table 2 - E - 6 - 2

                               IESCOPD - Meta-analysis of amount smoked : key value (2) 20
                             Any CB, cigarettes (or any product if cigarettes not available)
                                                      Most-adjusted


 REF    NRR SEX ADJ             Ys       Ws       Qs       Ps
 Subtotal LAMBER               1.41    26.71     2.44
 MUELLE 72  f   1              2.72     1.49     3.13       0.00
 OGILVI 17  m   0              1.37     6.12     0.07       0.00
 OGILVI 51  f   0              1.23     7.33     0.01       0.00
 Subtotal OGILVI               1.30    13.45     0.08
 REID   13  m   1              1.80    12.22     3.50       0.00
 REID   29  f   1              1.46    11.02     0.40       0.00
 Subtotal REID                 1.64    23.24     3.90
*TROISI 5   f   1              0.97   103.71     9.39       0.00
 WAGEN2 5   b   0              0.68    51.52    17.91       0.00
*WEN    9   m   1              0.71     2.38     0.75       0.27
 WOOLF  5   f   0              1.86     6.22     2.22       0.00

                       N       30
                      NS       21


                      Wt   455.33
                 Het Chi    98.61
                 Het  df       29
                 Het  P       ***
               Fixed  RR     3.55
                     RRl     3.24
                     RRu     3.90
                      P       +++
              Random  RR     4.30
                     RRl     3.55
                     RRu     5.21
                      P       +++
               Asymm  P       ***


  ________________________________________________________________________________________________________________________
                                            International Evidence on Smoking and COPD, Phase 3, Analysis run on 28-SEP-10

                                                   Table 2 - E - 6 - 3

                               IESCOPD - Meta-analysis of amount smoked : key value (2) 20
                             Any CB, cigarettes (or any product if cigarettes not available)
                                                      Most-adjusted


                       N       30
                      NS       21


                      Wt   455.33
                 Het Chi    98.61
                 Het  df       29
                 Het  P       ***
               Fixed  RR     3.55
                     RRl     3.24
                     RRu     3.90
                      P       +++
              Random  RR     4.30
                     RRl     3.55
                     RRu     5.21
                      P       +++
               Asymm  P       ***

                                   Sex
                             both      male    female     Total


                       N        2        16        12        30
                      NS        2        16        12        30


                      Wt    66.44    137.40    251.49    455.33
                 Het Chi    10.38     42.74     32.21     98.61
                 Het  df        1        15        11        29
                 Het  P        **       ***       ***       ***
               Fixed  RR     2.44      4.20      3.58      3.55
                     RRl     1.92      3.55      3.17      3.24
                     RRu     3.10      4.96      4.05      3.90
                      P       +++       +++       +++       +++
              Random  RR     3.09      4.72      4.22      4.30
                     RRl     1.22      3.48      3.28      3.55
                     RRu     7.80      6.40      5.42      5.21
                      P         +       +++       +++       +++
             Between Chi                                  13.28
             Between  df                                      2
             Between  P                                      **
             Btwn(F)  P                                    N.S.

                                        Continent
                            NAmer    Europe      Asia  oth/mult     Total


                       N       13        15         1         1        30
                      NS        9        10         1         1        21


                      Wt   217.45    234.50      2.38      1.00    455.33
                 Het Chi    33.01     51.16      0.00      0.00     98.61
                 Het  df       12        14         0         0        29
                 Het  P       ***       ***      N.S.      N.S.       ***
               Fixed  RR     3.69      3.40      2.03    129.50      3.55
                     RRl     3.23      2.99      0.57     18.21      3.24
                     RRu     4.22      3.86      7.23    921.07      3.90
                      P       +++       +++      N.S.       +++       +++
              Random  RR     4.52      4.02      2.03    129.50      4.30
                     RRl     3.44      3.09      0.57     18.21      3.55
                     RRu     5.93      5.24      7.23    921.07      5.21
                      P       +++       +++      N.S.       +++       +++
             Between Chi                                            14.44
             Between  df                                                3
             Between  P                                                **
             Btwn(F)  P                                              N.S.


  ________________________________________________________________________________________________________________________
                                            International Evidence on Smoking and COPD, Phase 3, Analysis run on 28-SEP-10

                                                   Table 2 - E - 6 - 3

                               IESCOPD - Meta-analysis of amount smoked : key value (2) 20
                             Any CB, cigarettes (or any product if cigarettes not available)
                                                      Most-adjusted
                               Study type
                               CC        Pr        CS     Total


                       N        5         4        21        30
                      NS        3         4        14        21


                      Wt   107.56    115.09    232.69    455.33
                 Het Chi     7.03      7.97     39.89     98.61
                 Het  df        4         3        20        29
                 Het  P      N.S.         *        **       ***
               Fixed  RR     2.41      2.79      4.79      3.55
                     RRl     1.99      2.33      4.21      3.24
                     RRu     2.91      3.35      5.45      3.90
                      P       +++       +++       +++       +++
              Random  RR     2.55      3.76      4.99      4.30
                     RRl     1.92      2.01      4.11      3.55
                     RRu     3.38      7.04      6.07      5.21
                      P       +++       +++       +++       +++
             Between Chi                                  43.73
             Between  df                                      2
             Between  P                                     ***
             Btwn(F)  P                                     ***

                           CB subtype
                             mort     sympt     other     Total


                       N        2        24         4        30
                      NS        2        16         3        21


                      Wt     4.92    297.66    152.75    455.33
                 Het Chi     4.16     73.41      3.75     98.61
                 Het  df        1        23         3        29
                 Het  P         *       ***      N.S.       ***
               Fixed  RR     5.25      4.06      2.71      3.55
                     RRl     2.17      3.62      2.31      3.24
                     RRu    12.70      4.55      3.17      3.90
                      P       +++       +++       +++       +++
              Random  RR     5.13      4.69      2.74      4.30
                     RRl     0.84      3.76      2.23      3.55
                     RRu    31.19      5.85      3.36      5.21
                      P       (+)       +++       +++       +++
             Between Chi                                  17.29
             Between  df                                      2
             Between  P                                     ***
             Btwn(F)  P                                     (*)

                             Smoking product
                              any      cigs  cigsonly     Total


                       N        5        20         5        30
                      NS        4        14         3        21


                      Wt    33.99    319.42    101.92    455.33
                 Het Chi    11.94     59.95     13.56     98.61
                 Het  df        4        19         4        29
                 Het  P         *       ***        **       ***
               Fixed  RR     6.46      3.41      3.32      3.55
                     RRl     4.61      3.05      2.73      3.24
                     RRu     9.04      3.80      4.03      3.90
                      P       +++       +++       +++       +++
              Random  RR     7.68      4.15      3.34      4.30
                     RRl     4.07      3.30      2.26      3.55
                     RRu    14.48      5.21      4.93      5.21
                      P       +++       +++       +++       +++
             Between Chi                                  13.17
             Between  df                                      2
             Between  P                                      **
             Btwn(F)  P                                    N.S.
  ________________________________________________________________________________________________________________________
                                            International Evidence on Smoking and COPD, Phase 3, Analysis run on 28-SEP-10

                                                   Table 2 - E - 6 - 3

                               IESCOPD - Meta-analysis of amount smoked : key value (2) 20
                             Any CB, cigarettes (or any product if cigarettes not available)
                                                      Most-adjusted
                                     Unexposed group
                          nev any   nev cig  nev+ any  nev+ cig     Total


                       N       19        11                            30
                      NS       13         9                            22


                      Wt   193.59    261.74                        455.33
                 Het Chi    48.44     44.51                         98.61
                 Het  df       18        10                            29
                 Het  P       ***       ***                           ***
               Fixed  RR     4.05      3.23                          3.55
                     RRl     3.51      2.86                          3.24
                     RRu     4.66      3.64                          3.90
                      P       +++       +++                           +++
              Random  RR     4.57      3.98                          4.30
                     RRl     3.54      2.96                          3.55
                     RRu     5.89      5.35                          5.21
                      P       +++       +++                           +++
             Between Chi                                             5.67
             Between  df                                                1
             Between  P                                                 *
             Btwn(F)  P                                              N.S.

                        Unexposed group (combining nev+ with main levels)
                          nev any   nev cig     Total


                       N       19        11        30
                      NS       13         9        22


                      Wt   193.59    261.74    455.33
                 Het Chi    48.44     44.51     98.61
                 Het  df       18        10        29
                 Het  P       ***       ***       ***
               Fixed  RR     4.05      3.23      3.55
                     RRl     3.51      2.86      3.24
                     RRu     4.66      3.64      3.90
                      P       +++       +++       +++
              Random  RR     4.57      3.98      4.30
                     RRl     3.54      2.96      3.55
                     RRu     5.89      5.35      5.21
                      P       +++       +++       +++
             Between Chi                         5.67
             Between  df                            1
             Between  P                             *
             Btwn(F)  P                          N.S.


  ________________________________________________________________________________________________________________________
                                            International Evidence on Smoking and COPD, Phase 3, Analysis run on 28-SEP-10

                                                   Table 2 - E - 6 - 4

                               IESCOPD - Meta-analysis of amount smoked : key value (2) 20
                             Any CB, cigarettes (or any product if cigarettes not available)
                                                      Least-adjusted


     REF|NRR|X|SEX|AGEL|AGEH|     REGION|BEGYR|PUBYR|STTYP|ONSET|      DISEAS|ADJ|SMOKSTA|   PRODUCT|    UNEXP|LOW| HI|

  ALDERS  12     m   35   74       Eu:UK  1977  1985    CC  Prev CB:diagnosed   1    Ever MCigs only   Nev any  18  27
  ALDERS  17     f   35   74       Eu:UK  1977  1985    CC  Prev CB:diagnosed   1    Ever MCigs only   Nev any  18  27
  ANDER1  41     m   25   74   Am:Canada  1963  1965    CS  Prev  CB:symptoms   1 Current       Cigs   Nev any  15  24
  ANDER1  46     f   25   74   Am:Canada  1963  1965    CS  Prev  CB:symptoms   1 Current       Cigs   Nev any  15  24
  CHAPMA   5 x   m   15   99      Am:USA  1976  1985    CS  Prev  CB:symptoms   0 Current       Cigs  Nev cigs  20  20
  CHAPMA  13 x   f   15   99      Am:USA  1976  1985    CS  Prev  CB:symptoms   0 Current       Cigs  Nev cigs  20  20
  COATES  10     b   40   64      Am:USA  1962  1965    CS  Prev  CB:symptoms   0 Current       Cigs  Nev cigs  15  24
   DEAN2  12     m   37   67       Eu:UK  1972  1978    CS  Prev  CB:symptoms   1 Current MCigs only   Nev any  18  22
   DEAN2  26     f   37   67       Eu:UK  1972  1978    CS  Prev  CB:symptoms   1 Current MCigs only   Nev any  18  22
   DOLL1  17     m   20   99       Eu:UK  1951  1994    Pr   Inc CB:mortality   1 Current  Cigs only   Nev any  15  24
  DONTA2   6     m   25   84 Eu:SE/Balkn  1960  1984    Pr   Inc CB:diagnosed   0 Current       Cigs  Nev cigs  20  29
  FERRI1 142     m   25   74      Am:USA  1961  1971    CS  Prev  CB:symptoms   1 Current       Cigs   Nev any  11  20
  FERRI1 101     f   25   74      Am:USA  1961  1971    CS  Prev  CB:symptoms   1 Current       Cigs   Nev any  11  20
  HARRIS   5     m   15   60      Africa     *  1993    CS  Prev  CB:symptoms   0 Current        Any   Nev any  15  24
  HOLLA2   5     m   40   59      Am:USA  1962  1965    CS  Prev  CB:symptoms   0 Current        Any   Nev any  15  24
  HOLLNA   5     m   40   40    Eu:Scand  1976  1983    CS  Prev  CB:symptoms   0 Current        Any   Nev any  15  24
  HOLLNA  13     f   40   40    Eu:Scand  1976  1983    CS  Prev  CB:symptoms   0 Current        Any   Nev any  15  24
  HUHTI1  46 x   m   40   64    Eu:Scand  1961  1965    CS  Prev  CB:symptoms   0 Current       Cigs   Nev any  15  24
  HUHTI3  24 x   m   25   69    Eu:Scand  1968  1978    CS  Prev  CB:symptoms   0 Current        Any   Nev any  15  24
  LAMBER   8 x   m   35   69       Eu:UK  1965  1970    CS  Prev  CB:symptoms   0 Current       Cigs  Nev cigs  20  20
  LAMBER  56 x   f   35   69       Eu:UK  1965  1970    CS  Prev  CB:symptoms   0 Current       Cigs   Nev any  20  20
  MUELLE  67 x   f   20   69      Am:USA  1967  1971    CS  Prev  CB:symptoms   0 Current       Cigs   Nev any  15  24
  OGILVI  17     m   30   99       Eu:UK  1955  1957    CC  Prev  CB:symptoms   0 Current       Cigs   Nev any  16  25
  OGILVI  51     f   30   99       Eu:UK  1955  1957    CC  Prev  CB:symptoms   0    Ever       Cigs   Nev any  16  25
    REID   8 x   m   35   74      Am:USA  1962  1966    CS  Prev  CB:symptoms   0 Current       Cigs  Nev cigs  20  20
    REID  24 x   f   35   74      Am:USA  1962  1966    CS  Prev  CB:symptoms   0 Current       Cigs  Nev cigs  20  20
  TROISI   5     f   34   69      Am:USA  1980  1995    Pr   Inc CB:diagnosed   1 Current       Cigs  Nev cigs  15  25
  WAGEN2   5     b   21   68     Eu:West  2001  2004    CC  Prev  CB:symptoms   0    Ever       Cigs  Nev cigs  11  20
     WEN   9     m   35   99   Asia:FarE  1982  2004    Pr   Inc CB:mortality   1 Current       Cigs  Nev cigs  11  20
   WOOLF   5     f   25   54   Am:Canada     *  1974    CS  Prev  CB:symptoms   0 Current       Cigs  Nev cigs  11  20


  ________________________________________________________________________________________________________________________
                                            International Evidence on Smoking and COPD, Phase 3, Analysis run on 28-SEP-10

                                                   Table 2 - E - 6 - 5

                               IESCOPD - Meta-analysis of amount smoked : key value (2) 20
                             Any CB, cigarettes (or any product if cigarettes not available)
                                                      Least-adjusted


                        Number Exposed  Non-exposed
 REF    NRR SEX ADJ     Case    Cont    Case    Cont      RR        95.00%CI
 ALDERS 12  m   1         72       -      31       -      1.96 (  1.15-   3.34)
 ALDERS 17  f   1        125       -     111       -      3.12 (  2.17-   4.49)
 Subtotal ALDERS                                          2.69 (  1.99-   3.64)
 ANDER1 41  m   1         16       -       4       -      7.03 (  2.11-  23.42)
 ANDER1 46  f   1          5       -      15       -      2.56 (  0.83-   7.87)
 Subtotal ANDER1                                          4.10 (  1.80-   9.32)
 CHAPMA 5   m   0         72     361      35     989      5.64 (  3.70-   8.59)
 CHAPMA 13  f   0         48     313      48    1857      5.93 (  3.91-   9.01)
 Subtotal CHAPMA                                          5.78 (  4.30-   7.78)
 COATES 10  b   0         69     333      21     515      5.08 (  3.06-   8.44)
 DEAN2  12  m   1         61       -      39       -      2.57 (  1.70-   3.89)
 DEAN2  26  f   1         69       -      80       -      4.59 (  3.29-   6.42)
 Subtotal DEAN2                                           3.65 (  2.81-   4.73)
*DOLL1  17  m   1         37       -       3       -     12.80 (  3.74-  43.85)
*DONTA2 6   m   0         19      67       8     127      4.50 (  2.08-   9.73)
 FERRI1 142 m   1         44       -      10       -      3.25 (  1.53-   6.91)
 FERRI1 101 f   1         17       -      35       -      2.75 (  1.45-   5.22)
 Subtotal FERRI1                                          2.95 (  1.81-   4.81)
 HARRIS 5   m   0          3       2       6     518    129.50 ( 18.21- 921.07)
 HOLLA2 5   m   0         38     131       5      84      4.87 (  1.84-  12.88)
 HOLLNA 5   m   0         72      89      10     101      8.17 (  3.98-  16.79)
 HOLLNA 13  f   0         42      86      14     217      7.57 (  3.93-  14.56)
 Subtotal HOLLNA                                          7.84 (  4.83-  12.72)
 HUHTI1 46  m   0         79     112       7     115     11.59 (  5.13-  26.19)
 HUHTI3 24  m   0         79     122      28     211      4.88 (  3.00-   7.93)
 LAMBER 8   m   0         41     540      17     642      2.87 (  1.61-   5.11)
 LAMBER 56  f   0         29     305      54    2390      4.21 (  2.64-   6.71)
 Subtotal LAMBER                                          3.62 (  2.52-   5.20)
 MUELLE 67  f   0         14      43       3     168     18.23 (  5.01-  66.31)
 OGILVI 17  m   0         66      44      11      29      3.95 (  1.79-   8.73)
 OGILVI 51  f   0         24      13      86     160      3.43 (  1.67-   7.08)
 Subtotal OGILVI                                          3.66 (  2.15-   6.25)
 REID   8   m   0         66    3484      16    4127      4.89 (  2.82-   8.45)
 REID   24  f   0         19    1881      37   10430      2.85 (  1.63-   4.96)
 Subtotal REID                                            3.74 (  2.53-   5.53)
*TROISI 5   f   1          -       -       -       -      2.63 (  2.13-   3.13)
 WAGEN2 5   b   0        111    1084     111    2136      1.97 (  1.50-   2.59)
*WEN    9   m   1          -       -       7       -      2.03 (  0.57-   7.23)
 WOOLF  5   f   0         25      67      10     173      6.46 (  2.94-  14.16)
Partial Totals          1362    9077     862   24989
*prospective study


 REF    NRR SEX ADJ             Ys       Ws       Qs       Ps
 ALDERS 12  m   1              0.67    13.52     4.63       0.01
 ALDERS 17  f   1              1.14    29.06     0.42       0.00
 Subtotal ALDERS               0.99    42.58     5.05
 ANDER1 41  m   1              1.95     2.65     1.27       0.00
 ANDER1 46  f   1              0.94     3.04     0.31       0.10
 Subtotal ANDER1               1.41     5.69     1.58
 CHAPMA 5   m   0              1.73    21.63     4.79       0.00
 CHAPMA 13  f   0              1.78    22.03     6.01       0.00
 Subtotal CHAPMA               1.76    43.65    10.80
 COATES 10  b   0              1.63    14.91     2.01       0.00
 DEAN2  12  m   1              0.94    22.42     2.22       0.00
 DEAN2  26  f   1              1.52    34.38     2.43       0.00
 Subtotal DEAN2                1.29    56.81     4.64
*DOLL1  17  m   1              2.55     2.54     4.23       0.00
*DONTA2 6   m   0              1.50     6.46     0.39       0.00
 FERRI1 142 m   1              1.18     6.76     0.04       0.00
 FERRI1 101 f   1              1.01     9.36     0.57       0.00
 Subtotal FERRI1               1.08    16.12     0.61
 HARRIS 5   m   0              4.86     1.00    12.97       0.00
 HOLLA2 5   m   0              1.58     4.07     0.43       0.00
 HOLLNA 5   m   0              2.10     7.41     5.25       0.00
 HOLLNA 13  f   0              2.02     8.97     5.26       0.00
 Subtotal HOLLNA               2.06    16.38    10.52
 HUHTI1 46  m   0              2.45     5.78     8.20       0.00
 HUHTI3 24  m   0              1.59    16.31     1.74       0.00
 LAMBER 8   m   0              1.05    11.54     0.48       0.00
 LAMBER 56  f   0              1.44    17.64     0.56       0.00
  ________________________________________________________________________________________________________________________
                                            International Evidence on Smoking and COPD, Phase 3, Analysis run on 28-SEP-10

                                                   Table 2 - E - 6 - 5

                               IESCOPD - Meta-analysis of amount smoked : key value (2) 20
                             Any CB, cigarettes (or any product if cigarettes not available)
                                                      Least-adjusted


 REF    NRR SEX ADJ             Ys       Ws       Qs       Ps
 Subtotal LAMBER               1.29    29.18     1.05
 MUELLE 67  f   0              2.90     2.30     6.23       0.00
 OGILVI 17  m   0              1.37     6.12     0.08       0.00
 OGILVI 51  f   0              1.23     7.33     0.00       0.00
 Subtotal OGILVI               1.30    13.45     0.09
 REID   8   m   0              1.59    12.79     1.38       0.00
 REID   24  f   0              1.05    12.46     0.56       0.00
 Subtotal REID                 1.32    25.25     1.94
*TROISI 5   f   1              0.97   103.71     8.80       0.00
 WAGEN2 5   b   0              0.68    51.52    17.33       0.00
*WEN    9   m   1              0.71     2.38     0.72       0.27
 WOOLF  5   f   0              1.86     6.22     2.29       0.00

                       N       30
                      NS       21


                      Wt   466.31
                 Het Chi   101.63
                 Het  df       29
                 Het  P       ***
               Fixed  RR     3.52
                     RRl     3.21
                     RRu     3.85
                      P       +++
              Random  RR     4.24
                     RRl     3.50
                     RRu     5.13
                      P       +++
               Asymm  P       ***


  ________________________________________________________________________________________________________________________
                                            International Evidence on Smoking and COPD, Phase 3, Analysis run on 28-SEP-10

                                                   Table 2 - E - 6 - 6

                               IESCOPD - Meta-analysis of amount smoked : key value (2) 20
                             Any CB, cigarettes (or any product if cigarettes not available)
                                                      Least-adjusted


                       N       30
                      NS       21


                      Wt   466.31
                 Het Chi   101.63
                 Het  df       29
                 Het  P       ***
               Fixed  RR     3.52
                     RRl     3.21
                     RRu     3.85
                      P       +++
              Random  RR     4.24
                     RRl     3.50
                     RRu     5.13
                      P       +++
               Asymm  P       ***

                                   Sex
                             both      male    female     Total


                       N        2        16        12        30
                      NS        2        16        12        30


                      Wt    66.44    143.37    256.50    466.31
                 Het Chi    10.38     44.13     33.44    101.63
                 Het  df        1        15        11        29
                 Het  P        **       ***       ***       ***
               Fixed  RR     2.44      4.22      3.50      3.52
                     RRl     1.92      3.58      3.09      3.21
                     RRu     3.10      4.97      3.95      3.85
                      P       +++       +++       +++       +++
              Random  RR     3.09      4.72      4.06      4.24
                     RRl     1.22      3.49      3.16      3.50
                     RRu     7.80      6.40      5.22      5.13
                      P         +       +++       +++       +++
             Between Chi                                  13.69
             Between  df                                      2
             Between  P                                      **
             Btwn(F)  P                                    N.S.

                                        Continent
                            NAmer    Europe      Asia  oth/mult     Total


                       N       13        15         1         1        30
                      NS        9        10         1         1        21


                      Wt   221.93    241.00      2.38      1.00    466.31
                 Het Chi    34.56     53.00      0.00      0.00    101.63
                 Het  df       12        14         0         0        29
                 Het  P       ***       ***      N.S.      N.S.       ***
               Fixed  RR     3.61      3.41      2.03    129.50      3.52
                     RRl     3.16      3.00      0.57     18.21      3.21
                     RRu     4.11      3.87      7.23    921.07      3.85
                      P       +++       +++      N.S.       +++       +++
              Random  RR     4.35      4.02      2.03    129.50      4.24
                     RRl     3.31      3.09      0.57     18.21      3.50
                     RRu     5.72      5.24      7.23    921.07      5.13
                      P       +++       +++      N.S.       +++       +++
             Between Chi                                            14.07
             Between  df                                                3
             Between  P                                                **
             Btwn(F)  P                                              N.S.


  ________________________________________________________________________________________________________________________
                                            International Evidence on Smoking and COPD, Phase 3, Analysis run on 28-SEP-10

                                                   Table 2 - E - 6 - 6

                               IESCOPD - Meta-analysis of amount smoked : key value (2) 20
                             Any CB, cigarettes (or any product if cigarettes not available)
                                                      Least-adjusted
                               Study type
                               CC        Pr        CS     Total


                       N        5         4        21        30
                      NS        3         4        14        21


                      Wt   107.56    115.09    243.67    466.31
                 Het Chi     7.03      7.97     46.37    101.63
                 Het  df        4         3        20        29
                 Het  P      N.S.         *       ***       ***
               Fixed  RR     2.41      2.79      4.64      3.52
                     RRl     1.99      2.33      4.09      3.21
                     RRu     2.91      3.35      5.26      3.85
                      P       +++       +++       +++       +++
              Random  RR     2.55      3.76      4.89      4.24
                     RRl     1.92      2.01      3.98      3.50
                     RRu     3.38      7.04      6.01      5.13
                      P       +++       +++       +++       +++
             Between Chi                                  40.28
             Between  df                                      2
             Between  P                                     ***
             Btwn(F)  P                                      **

                           CB subtype
                             mort     sympt     other     Total


                       N        2        24         4        30
                      NS        2        16         3        21


                      Wt     4.92    308.64    152.75    466.31
                 Het Chi     4.16     77.74      3.75    101.63
                 Het  df        1        23         3        29
                 Het  P         *       ***      N.S.       ***
               Fixed  RR     5.25      3.98      2.71      3.52
                     RRl     2.17      3.56      2.31      3.21
                     RRu    12.70      4.45      3.17      3.85
                      P       +++       +++       +++       +++
              Random  RR     5.13      4.60      2.74      4.24
                     RRl     0.84      3.68      2.23      3.50
                     RRu    31.19      5.75      3.36      5.13
                      P       (+)       +++       +++       +++
             Between Chi                                  15.97
             Between  df                                      2
             Between  P                                     ***
             Btwn(F)  P                                     (*)

                             Smoking product
                              any      cigs  cigsonly     Total


                       N        5        20         5        30
                      NS        4        14         3        21


                      Wt    37.75    326.64    101.92    466.31
                 Het Chi    11.21     61.13     13.56    101.63
                 Het  df        4        19         4        29
                 Het  P         *       ***        **       ***
               Fixed  RR     6.53      3.34      3.32      3.52
                     RRl     4.75      2.99      2.73      3.21
                     RRu     8.99      3.72      4.03      3.85
                      P       +++       +++       +++       +++
              Random  RR     7.74      4.02      3.34      4.24
                     RRl     4.27      3.21      2.26      3.50
                     RRu    14.05      5.04      4.93      5.13
                      P       +++       +++       +++       +++
             Between Chi                                  15.73
             Between  df                                      2
             Between  P                                     ***
             Btwn(F)  P                                    N.S.
  ________________________________________________________________________________________________________________________
                                            International Evidence on Smoking and COPD, Phase 3, Analysis run on 28-SEP-10

                                                   Table 2 - E - 6 - 6

                               IESCOPD - Meta-analysis of amount smoked : key value (2) 20
                             Any CB, cigarettes (or any product if cigarettes not available)
                                                      Least-adjusted
                                     Unexposed group
                          nev any   nev cig  nev+ any  nev+ cig     Total


                       N       19        11                            30
                      NS       13         9                            22


                      Wt   200.66    265.65                        466.31
                 Het Chi    52.47     41.45                        101.63
                 Het  df       18        10                            29
                 Het  P       ***       ***                           ***
               Fixed  RR     4.08      3.15                          3.52
                     RRl     3.55      2.79                          3.21
                     RRu     4.69      3.55                          3.85
                      P       +++       +++                           +++
              Random  RR     4.67      3.74                          4.24
                     RRl     3.60      2.82                          3.50
                     RRu     6.05      4.97                          5.13
                      P       +++       +++                           +++
             Between Chi                                             7.71
             Between  df                                                1
             Between  P                                                **
             Btwn(F)  P                                              N.S.

                        Unexposed group (combining nev+ with main levels)
                          nev any   nev cig     Total


                       N       19        11        30
                      NS       13         9        22


                      Wt   200.66    265.65    466.31
                 Het Chi    52.47     41.45    101.63
                 Het  df       18        10        29
                 Het  P       ***       ***       ***
               Fixed  RR     4.08      3.15      3.52
                     RRl     3.55      2.79      3.21
                     RRu     4.69      3.55      3.85
                      P       +++       +++       +++
              Random  RR     4.67      3.74      4.24
                     RRl     3.60      2.82      3.50
                     RRu     6.05      4.97      5.13
                      P       +++       +++       +++
             Between Chi                         7.71
             Between  df                            1
             Between  P                            **
             Btwn(F)  P                          N.S.


  ________________________________________________________________________________________________________________________
                                            International Evidence on Smoking and COPD, Phase 3, Analysis run on 28-SEP-10

                                                   Table 2 - E - 6 - 7

                               IESCOPD - Meta-analysis of amount smoked : key value (2) 20
                             Any CB, cigarettes (or any product if cigarettes not available)
                                 Excluded studies (and stage at which they were excluded)


1       CLARK COTTON  MEYER REMYJA RUTGER SNYDER SOBRAX     SU TAKEMU  WANG4   WEIR WHICKE ZALACA
2      ALESSA  AMIGO ANDER2 ANDER3 AUERBA BEDNAR BROGGE  CHEN1  CHEN2  CHEN3  CHENG CLEMEN  COCCI  DEAN1 DEJONG DETORR
       DICKIN DONTA1 EKBERG ENSTRO FERRI2 FERRI3  FIDAN FORAST FUKUCH GEIJER GODTFR GULSVI HAMMO2 HARIKK HEDMAN HIGGI4
       HOZAWA ITABAS JACOBS JAENDI JOHANN KACHEL KARAKA KATANC KHOURY    KIM KLAYTO KOJIMA KOTAN2 KRZYZA KULLER    LAI
         LAM1   LAM2   LAM3  LANGE    LEE   LIAW LINDBE   LIU1   LIU2 LUNDB1  MADOR MANNI1 MANNI2 MANNI3 MARAN1 MARAN2
       MARCUS MATHES MENEZ2 MENEZ3 MENEZ4 MENEZ5 MENEZ6 MONTNE   NAWA NIEPSU NIHLEN NILSSO  OMORI   PEAT   PETO  PRATT
        PRICE RENWIC RICCIO  RYDER SARGEA SHAHAB   SHIN SICHLE SPEIZE STERLI  STROM SUTINE  TAGER   TANG   THUN   TODD
       TRUPIN TSUSHI TVERDA VESTBO VIEGI2 VIKGRE VINEIS VOLLM1 VOLLM2 VONHER   WALD  WANG2 WATSON  WEISS WILSO1   XIAO
           XU   YUAN ZIELI1 ZIELI2 ZIETKO
3        KAHN
4        BANG  BECK1  BECK2 BJORNS CERVER COLLEG  DEANE DEMARC DOPICO ENRIGH FINKLE FOXMAN GOLDBE HARDIE  HAYES HIRAYA
           HO  HOUSE HUHTI2 JENSEN JINDA2  JOSHI JOUSI1   KATO  KIRAZ KOTAN1  KUBIK LANGE2 LANGHA LUNDB2 MAGNUS MANFRE
       MELLST MILLER MOLLER NEJJAR OSWAL1 OSWAL2 PANDEY PELKON PEREZP SAWICK SCHWAR SHIMUR  SILVA SOBRAD STJERN SUADIC
       TAGER2 VIEGI1 WOJTYN  WOODS   ZOIA
5       DOLL2 FLETCH HAWTHO HRUBEC RIMING  SHARP    WIG WILSO2
6        BEST  BROWN EHRLIC HAENSZ HIGGI2 HIGGI3 HIGGI6 HUCHON  KAHN2 LAVECC LEBOWI LINDST MENEZ1  MEREN  MILNE URRUTI
       WILHEL YAMAGU


  ________________________________________________________________________________________________________________________
                                            International Evidence on Smoking and COPD, Phase 3, Analysis run on 28-SEP-10

                                                   Table 2 - E - 6 - 8

                               IESCOPD - Meta-analysis of amount smoked : key value (2) 20
                             Any CB, cigarettes (or any product if cigarettes not available)
                                             Potentially overlapping studies


     REF| REFGP|PRINC|                     OVERLAP|

  DONTA2 JACOBS     2  JACOBS/DONTA1/DONTA2/PELKON
  HUHTI1 HUHTI1     1                HUHTI1/HUHTI2
  FERRI1 FERRIS     2         FERRI1/FERRI2/FERRI3
     WEN    WEN     1                     WEN/LIAW
  LAMBER   TODD     2                  LAMBER/TODD
  CHAPMA  HOUSE     2                 HOUSE/CHAPMA
  HOLLNA HOLLNA     1 GODT/VEST/LANG1+2/SUAD/HOLLN


  ________________________________________________________________________________________________________________________
                                            International Evidence on Smoking and COPD, Phase 3, Analysis run on 28-SEP-10

                                                    Table 2 - E - 7 -

                               IESCOPD - Meta-analysis of amount smoked : key value (2) 30
                             Any CB, cigarettes (or any product if cigarettes not available)


This analysis is restricted to results for:
1) Eligible study on database
2) Outcome CB
3) Current or ever smoking
4) Categorical dose-response data for amount smoked
5) vs never smoking base
6) Key value (scheme 2) = 30
7) Results complete enough for use in meta-analysis

Within each study, results are then selected (in the following order of preference, within each sex) for:
8) SMKSTA  : current, ever
9) UNEXP   : never any, never cigarettes
10) PROD    : cigarettes, cigarettes only, any product
11) For overlapping studies: principal rather than subsidiary studies
and then for single sex results (m, f) in preference to results for both sexes combined (b).

Results adjusted for the most potential confounders are then chosen in Sections -1 to -3
and results adjusted for the least confounders in Sections -4 to -6. (Those least-adjusted results which
actually differ from the most-adjusted are marked 'x' in column X in Section -4)

Section -7 shows excluded studies, together with the stage (as above) at which no qualifying
results were found.

Section -8 lists the potentially overlapping studies which have been included (1=principal, 2=subsidiary),
and any results which would have been included in preference except that they had data not complete enough
for use in meta-analysis. It also lists their significance (yes/no), if known.


  ________________________________________________________________________________________________________________________
                                            International Evidence on Smoking and COPD, Phase 3, Analysis run on 28-SEP-10

                                                   Table 2 - E - 7 - 1

                               IESCOPD - Meta-analysis of amount smoked : key value (2) 30
                             Any CB, cigarettes (or any product if cigarettes not available)
                                                      Most-adjusted


     REF|NRR|SEX|AGEL|AGEH|     REGION|BEGYR|PUBYR|STTYP|ONSET|      DISEAS|ADJ|SMOKSTA|   PRODUCT|    UNEXP|LOW| HI|

   DEAN2  14   m   37   67       Eu:UK  1972  1978    CS  Prev  CB:symptoms   1 Current MCigs only   Nev any  28  32
  FERRI1 143   m   25   74      Am:USA  1961  1971    CS  Prev  CB:symptoms   1 Current       Cigs   Nev any  21  30
   KAHN2   5   m   31   84      Am:USA  1954  1966    Pr   Inc CB:mortality   1 Current       Cigs   Nev any  21  39


  ________________________________________________________________________________________________________________________
                                            International Evidence on Smoking and COPD, Phase 3, Analysis run on 28-SEP-10

                                                   Table 2 - E - 7 - 2

                               IESCOPD - Meta-analysis of amount smoked : key value (2) 30
                             Any CB, cigarettes (or any product if cigarettes not available)
                                                      Most-adjusted


                        Number Exposed  Non-exposed
 REF    NRR SEX ADJ     Case    Cont    Case    Cont      RR        95.00%CI
 DEAN2  14  m   1         20       -      39       -      2.91 (  1.66-   5.11)
 FERRI1 143 m   1         44       -      10       -      4.58 (  2.13-   9.87)
*KAHN2  5   m   1         16       -      13       -      4.01 (  1.93-   8.34)
Partial Totals            80       0      62       0
*prospective study


 REF    NRR SEX ADJ             Ys       Ws       Qs       Ps
 DEAN2  14  m   1              1.07    12.15     0.50       0.00
 FERRI1 143 m   1              1.52     6.54     0.41       0.00
*KAHN2  5   m   1              1.39     7.17     0.10       0.00

                       N        3
                      NS        3


                      Wt    25.86
                 Het Chi     1.01
                 Het  df        2
                 Het  P      N.S.
               Fixed  RR     3.57
                     RRl     2.43
                     RRu     5.24
                      P       +++
              Random  RR     3.57
                     RRl     2.43
                     RRu     5.24
                      P       +++
               Asymm  P       (*)


  ________________________________________________________________________________________________________________________
                                            International Evidence on Smoking and COPD, Phase 3, Analysis run on 28-SEP-10

                                                   Table 2 - E - 7 - 3

                               IESCOPD - Meta-analysis of amount smoked : key value (2) 30
                             Any CB, cigarettes (or any product if cigarettes not available)
                                                      Most-adjusted


                       N        3
                      NS        3


                      Wt    25.86
                 Het Chi     1.01
                 Het  df        2
                 Het  P      N.S.
               Fixed  RR     3.57
                     RRl     2.43
                     RRu     5.24
                      P       +++
              Random  RR     3.57
                     RRl     2.43
                     RRu     5.24
                      P       +++
               Asymm  P       (*)

                                   Sex
                             both      male    female     Total


                       N                  3                   3
                      NS                  3                   3


                      Wt              25.86               25.86
                 Het Chi               1.01                1.01
                 Het  df                  2                   2
                 Het  P                N.S.                N.S.
               Fixed  RR               3.57                3.57
                     RRl               2.43                2.43
                     RRu               5.24                5.24
                      P                 +++                 +++
              Random  RR               3.57                3.57
                     RRl               2.43                2.43
                     RRu               5.24                5.24
                      P                 +++                 +++
             Between Chi
             Between  df
             Between  P                                    N.S.
             Btwn(F)  P                                    N.S.

                                        Continent
                            NAmer    Europe      Asia  oth/mult     Total


                       N        2         1                             3
                      NS        2         1                             3


                      Wt    13.71     12.15                         25.86
                 Het Chi     0.06      0.00                          1.01
                 Het  df        1         0                             2
                 Het  P      N.S.      N.S.                          N.S.
               Fixed  RR     4.27      2.91                          3.57
                     RRl     2.52      1.66                          2.43
                     RRu     7.25      5.11                          5.24
                      P       +++       +++                           +++
              Random  RR     4.27      2.91                          3.57
                     RRl     2.52      1.66                          2.43
                     RRu     7.25      5.11                          5.24
                      P       +++       +++                           +++
             Between Chi                                             0.95
             Between  df                                                1
             Between  P                                              N.S.
             Btwn(F)  P                                              N.S.


  ________________________________________________________________________________________________________________________
                                            International Evidence on Smoking and COPD, Phase 3, Analysis run on 28-SEP-10

                                                   Table 2 - E - 7 - 3

                               IESCOPD - Meta-analysis of amount smoked : key value (2) 30
                             Any CB, cigarettes (or any product if cigarettes not available)
                                                      Most-adjusted
                               Study type
                               CC        Pr        CS     Total


                       N                  1         2         3
                      NS                  1         2         3


                      Wt               7.17     18.69     25.86
                 Het Chi               0.00      0.87      1.01
                 Het  df                  0         1         2
                 Het  P                N.S.      N.S.      N.S.
               Fixed  RR               4.01      3.41      3.57
                     RRl               1.93      2.17      2.43
                     RRu               8.34      5.37      5.24
                      P                 +++       +++       +++
              Random  RR               4.01      3.41      3.57
                     RRl               1.93      2.17      2.43
                     RRu               8.34      5.37      5.24
                      P                 +++       +++       +++
             Between Chi                                   0.14
             Between  df                                      1
             Between  P                                    N.S.
             Btwn(F)  P                                    N.S.

                           CB subtype
                             mort     sympt     other     Total


                       N        1         2                   3
                      NS        1         2                   3


                      Wt     7.17     18.69               25.86
                 Het Chi     0.00      0.87                1.01
                 Het  df        0         1                   2
                 Het  P      N.S.      N.S.                N.S.
               Fixed  RR     4.01      3.41                3.57
                     RRl     1.93      2.17                2.43
                     RRu     8.34      5.37                5.24
                      P       +++       +++                 +++
              Random  RR     4.01      3.41                3.57
                     RRl     1.93      2.17                2.43
                     RRu     8.34      5.37                5.24
                      P       +++       +++                 +++
             Between Chi                                   0.14
             Between  df                                      1
             Between  P                                    N.S.
             Btwn(F)  P                                    N.S.

                             Smoking product
                              any      cigs  cigsonly     Total


                       N                  2         1         3
                      NS                  2         1         3


                      Wt              13.71     12.15     25.86
                 Het Chi               0.06      0.00      1.01
                 Het  df                  1         0         2
                 Het  P                N.S.      N.S.      N.S.
               Fixed  RR               4.27      2.91      3.57
                     RRl               2.52      1.66      2.43
                     RRu               7.25      5.11      5.24
                      P                 +++       +++       +++
              Random  RR               4.27      2.91      3.57
                     RRl               2.52      1.66      2.43
                     RRu               7.25      5.11      5.24
                      P                 +++       +++       +++
             Between Chi                                   0.95
             Between  df                                      1
             Between  P                                    N.S.
             Btwn(F)  P                                    N.S.
  ________________________________________________________________________________________________________________________
                                            International Evidence on Smoking and COPD, Phase 3, Analysis run on 28-SEP-10

                                                   Table 2 - E - 7 - 3

                               IESCOPD - Meta-analysis of amount smoked : key value (2) 30
                             Any CB, cigarettes (or any product if cigarettes not available)
                                                      Most-adjusted
                                     Unexposed group
                          nev any   nev cig  nev+ any  nev+ cig     Total


                       N        3                                       3
                      NS        3                                       3


                      Wt    25.86                                   25.86
                 Het Chi     1.01                                    1.01
                 Het  df        2                                       2
                 Het  P      N.S.                                    N.S.
               Fixed  RR     3.57                                    3.57
                     RRl     2.43                                    2.43
                     RRu     5.24                                    5.24
                      P       +++                                     +++
              Random  RR     3.57                                    3.57
                     RRl     2.43                                    2.43
                     RRu     5.24                                    5.24
                      P       +++                                     +++
             Between Chi
             Between  df
             Between  P                                              N.S.
             Btwn(F)  P                                              N.S.

                        Unexposed group (combining nev+ with main levels)
                          nev any   nev cig     Total


                       N        3                   3
                      NS        3                   3


                      Wt    25.86               25.86
                 Het Chi     1.01                1.01
                 Het  df        2                   2
                 Het  P      N.S.                N.S.
               Fixed  RR     3.57                3.57
                     RRl     2.43                2.43
                     RRu     5.24                5.24
                      P       +++                 +++
              Random  RR     3.57                3.57
                     RRl     2.43                2.43
                     RRu     5.24                5.24
                      P       +++                 +++
             Between Chi
             Between  df
             Between  P                          N.S.
             Btwn(F)  P                          N.S.


  ________________________________________________________________________________________________________________________
                                            International Evidence on Smoking and COPD, Phase 3, Analysis run on 28-SEP-10

                                                   Table 2 - E - 7 - 4

                               IESCOPD - Meta-analysis of amount smoked : key value (2) 30
                             Any CB, cigarettes (or any product if cigarettes not available)
                                                      Least-adjusted


     REF|NRR|X|SEX|AGEL|AGEH|     REGION|BEGYR|PUBYR|STTYP|ONSET|      DISEAS|ADJ|SMOKSTA|   PRODUCT|    UNEXP|LOW| HI|

   DEAN2  14     m   37   67       Eu:UK  1972  1978    CS  Prev  CB:symptoms   1 Current MCigs only   Nev any  28  32
  FERRI1 143     m   25   74      Am:USA  1961  1971    CS  Prev  CB:symptoms   1 Current       Cigs   Nev any  21  30
   KAHN2   5     m   31   84      Am:USA  1954  1966    Pr   Inc CB:mortality   1 Current       Cigs   Nev any  21  39


  ________________________________________________________________________________________________________________________
                                            International Evidence on Smoking and COPD, Phase 3, Analysis run on 28-SEP-10

                                                   Table 2 - E - 7 - 5

                               IESCOPD - Meta-analysis of amount smoked : key value (2) 30
                             Any CB, cigarettes (or any product if cigarettes not available)
                                                      Least-adjusted


                        Number Exposed  Non-exposed
 REF    NRR SEX ADJ     Case    Cont    Case    Cont      RR        95.00%CI
 DEAN2  14  m   1         20       -      39       -      2.91 (  1.66-   5.11)
 FERRI1 143 m   1         44       -      10       -      4.58 (  2.13-   9.87)
*KAHN2  5   m   1         16       -      13       -      4.01 (  1.93-   8.34)
Partial Totals            80       0      62       0
*prospective study


 REF    NRR SEX ADJ             Ys       Ws       Qs       Ps
 DEAN2  14  m   1              1.07    12.15     0.50       0.00
 FERRI1 143 m   1              1.52     6.54     0.41       0.00
*KAHN2  5   m   1              1.39     7.17     0.10       0.00

                       N        3
                      NS        3


                      Wt    25.86
                 Het Chi     1.01
                 Het  df        2
                 Het  P      N.S.
               Fixed  RR     3.57
                     RRl     2.43
                     RRu     5.24
                      P       +++
              Random  RR     3.57
                     RRl     2.43
                     RRu     5.24
                      P       +++
               Asymm  P       (*)


  ________________________________________________________________________________________________________________________
                                            International Evidence on Smoking and COPD, Phase 3, Analysis run on 28-SEP-10

                                                   Table 2 - E - 7 - 6

                               IESCOPD - Meta-analysis of amount smoked : key value (2) 30
                             Any CB, cigarettes (or any product if cigarettes not available)
                                                      Least-adjusted


                       N        3
                      NS        3


                      Wt    25.86
                 Het Chi     1.01
                 Het  df        2
                 Het  P      N.S.
               Fixed  RR     3.57
                     RRl     2.43
                     RRu     5.24
                      P       +++
              Random  RR     3.57
                     RRl     2.43
                     RRu     5.24
                      P       +++
               Asymm  P       (*)

                                   Sex
                             both      male    female     Total


                       N                  3                   3
                      NS                  3                   3


                      Wt              25.86               25.86
                 Het Chi               1.01                1.01
                 Het  df                  2                   2
                 Het  P                N.S.                N.S.
               Fixed  RR               3.57                3.57
                     RRl               2.43                2.43
                     RRu               5.24                5.24
                      P                 +++                 +++
              Random  RR               3.57                3.57
                     RRl               2.43                2.43
                     RRu               5.24                5.24
                      P                 +++                 +++
             Between Chi
             Between  df
             Between  P                                    N.S.
             Btwn(F)  P                                    N.S.

                                        Continent
                            NAmer    Europe      Asia  oth/mult     Total


                       N        2         1                             3
                      NS        2         1                             3


                      Wt    13.71     12.15                         25.86
                 Het Chi     0.06      0.00                          1.01
                 Het  df        1         0                             2
                 Het  P      N.S.      N.S.                          N.S.
               Fixed  RR     4.27      2.91                          3.57
                     RRl     2.52      1.66                          2.43
                     RRu     7.25      5.11                          5.24
                      P       +++       +++                           +++
              Random  RR     4.27      2.91                          3.57
                     RRl     2.52      1.66                          2.43
                     RRu     7.25      5.11                          5.24
                      P       +++       +++                           +++
             Between Chi                                             0.95
             Between  df                                                1
             Between  P                                              N.S.
             Btwn(F)  P                                              N.S.


  ________________________________________________________________________________________________________________________
                                            International Evidence on Smoking and COPD, Phase 3, Analysis run on 28-SEP-10

                                                   Table 2 - E - 7 - 6

                               IESCOPD - Meta-analysis of amount smoked : key value (2) 30
                             Any CB, cigarettes (or any product if cigarettes not available)
                                                      Least-adjusted
                               Study type
                               CC        Pr        CS     Total


                       N                  1         2         3
                      NS                  1         2         3


                      Wt               7.17     18.69     25.86
                 Het Chi               0.00      0.87      1.01
                 Het  df                  0         1         2
                 Het  P                N.S.      N.S.      N.S.
               Fixed  RR               4.01      3.41      3.57
                     RRl               1.93      2.17      2.43
                     RRu               8.34      5.37      5.24
                      P                 +++       +++       +++
              Random  RR               4.01      3.41      3.57
                     RRl               1.93      2.17      2.43
                     RRu               8.34      5.37      5.24
                      P                 +++       +++       +++
             Between Chi                                   0.14
             Between  df                                      1
             Between  P                                    N.S.
             Btwn(F)  P                                    N.S.

                           CB subtype
                             mort     sympt     other     Total


                       N        1         2                   3
                      NS        1         2                   3


                      Wt     7.17     18.69               25.86
                 Het Chi     0.00      0.87                1.01
                 Het  df        0         1                   2
                 Het  P      N.S.      N.S.                N.S.
               Fixed  RR     4.01      3.41                3.57
                     RRl     1.93      2.17                2.43
                     RRu     8.34      5.37                5.24
                      P       +++       +++                 +++
              Random  RR     4.01      3.41                3.57
                     RRl     1.93      2.17                2.43
                     RRu     8.34      5.37                5.24
                      P       +++       +++                 +++
             Between Chi                                   0.14
             Between  df                                      1
             Between  P                                    N.S.
             Btwn(F)  P                                    N.S.

                             Smoking product
                              any      cigs  cigsonly     Total


                       N                  2         1         3
                      NS                  2         1         3


                      Wt              13.71     12.15     25.86
                 Het Chi               0.06      0.00      1.01
                 Het  df                  1         0         2
                 Het  P                N.S.      N.S.      N.S.
               Fixed  RR               4.27      2.91      3.57
                     RRl               2.52      1.66      2.43
                     RRu               7.25      5.11      5.24
                      P                 +++       +++       +++
              Random  RR               4.27      2.91      3.57
                     RRl               2.52      1.66      2.43
                     RRu               7.25      5.11      5.24
                      P                 +++       +++       +++
             Between Chi                                   0.95
             Between  df                                      1
             Between  P                                    N.S.
             Btwn(F)  P                                    N.S.
  ________________________________________________________________________________________________________________________
                                            International Evidence on Smoking and COPD, Phase 3, Analysis run on 28-SEP-10

                                                   Table 2 - E - 7 - 6

                               IESCOPD - Meta-analysis of amount smoked : key value (2) 30
                             Any CB, cigarettes (or any product if cigarettes not available)
                                                      Least-adjusted
                                     Unexposed group
                          nev any   nev cig  nev+ any  nev+ cig     Total


                       N        3                                       3
                      NS        3                                       3


                      Wt    25.86                                   25.86
                 Het Chi     1.01                                    1.01
                 Het  df        2                                       2
                 Het  P      N.S.                                    N.S.
               Fixed  RR     3.57                                    3.57
                     RRl     2.43                                    2.43
                     RRu     5.24                                    5.24
                      P       +++                                     +++
              Random  RR     3.57                                    3.57
                     RRl     2.43                                    2.43
                     RRu     5.24                                    5.24
                      P       +++                                     +++
             Between Chi
             Between  df
             Between  P                                              N.S.
             Btwn(F)  P                                              N.S.

                        Unexposed group (combining nev+ with main levels)
                          nev any   nev cig     Total


                       N        3                   3
                      NS        3                   3


                      Wt    25.86               25.86
                 Het Chi     1.01                1.01
                 Het  df        2                   2
                 Het  P      N.S.                N.S.
               Fixed  RR     3.57                3.57
                     RRl     2.43                2.43
                     RRu     5.24                5.24
                      P       +++                 +++
              Random  RR     3.57                3.57
                     RRl     2.43                2.43
                     RRu     5.24                5.24
                      P       +++                 +++
             Between Chi
             Between  df
             Between  P                          N.S.
             Btwn(F)  P                          N.S.


  ________________________________________________________________________________________________________________________
                                            International Evidence on Smoking and COPD, Phase 3, Analysis run on 28-SEP-10

                                                   Table 2 - E - 7 - 7

                               IESCOPD - Meta-analysis of amount smoked : key value (2) 30
                             Any CB, cigarettes (or any product if cigarettes not available)
                                 Excluded studies (and stage at which they were excluded)


1       CLARK COTTON  MEYER REMYJA RUTGER SNYDER SOBRAX     SU TAKEMU  WANG4   WEIR WHICKE ZALACA
2      ALESSA  AMIGO ANDER2 ANDER3 AUERBA BEDNAR BROGGE  CHEN1  CHEN2  CHEN3  CHENG CLEMEN  COCCI  DEAN1 DEJONG DETORR
       DICKIN DONTA1 EKBERG ENSTRO FERRI2 FERRI3  FIDAN FORAST FUKUCH GEIJER GODTFR GULSVI HAMMO2 HARIKK HEDMAN HIGGI4
       HOZAWA ITABAS JACOBS JAENDI JOHANN KACHEL KARAKA KATANC KHOURY    KIM KLAYTO KOJIMA KOTAN2 KRZYZA KULLER    LAI
         LAM1   LAM2   LAM3  LANGE    LEE   LIAW LINDBE   LIU1   LIU2 LUNDB1  MADOR MANNI1 MANNI2 MANNI3 MARAN1 MARAN2
       MARCUS MATHES MENEZ2 MENEZ3 MENEZ4 MENEZ5 MENEZ6 MONTNE   NAWA NIEPSU NIHLEN NILSSO  OMORI   PEAT   PETO  PRATT
        PRICE RENWIC RICCIO  RYDER SARGEA SHAHAB   SHIN SICHLE SPEIZE STERLI  STROM SUTINE  TAGER   TANG   THUN   TODD
       TRUPIN TSUSHI TVERDA VESTBO VIEGI2 VIKGRE VINEIS VOLLM1 VOLLM2 VONHER   WALD  WANG2 WATSON  WEISS WILSO1   XIAO
           XU   YUAN ZIELI1 ZIELI2 ZIETKO
3        KAHN
4        BANG  BECK1  BECK2 BJORNS CERVER COLLEG  DEANE DEMARC DOPICO ENRIGH FINKLE FOXMAN GOLDBE HARDIE  HAYES HIRAYA
           HO  HOUSE HUHTI2 JENSEN JINDA2  JOSHI JOUSI1   KATO  KIRAZ KOTAN1  KUBIK LANGE2 LANGHA LUNDB2 MAGNUS MANFRE
       MELLST MILLER MOLLER NEJJAR OSWAL1 OSWAL2 PANDEY PELKON PEREZP SAWICK SCHWAR SHIMUR  SILVA SOBRAD STJERN SUADIC
       TAGER2 VIEGI1 WOJTYN  WOODS   ZOIA
5       DOLL2 FLETCH HAWTHO HRUBEC RIMING  SHARP    WIG WILSO2
6      ALDERS ANDER1   BEST  BROWN CHAPMA COATES  DOLL1 DONTA2 EHRLIC HAENSZ HARRIS HIGGI2 HIGGI3 HIGGI6 HOLLA2 HOLLNA
       HUCHON HUHTI1 HUHTI3 LAMBER LAVECC LEBOWI LINDST MENEZ1  MEREN  MILNE MUELLE OGILVI   REID TROISI URRUTI WAGEN2
          WEN WILHEL  WOOLF YAMAGU


  ________________________________________________________________________________________________________________________
                                            International Evidence on Smoking and COPD, Phase 3, Analysis run on 28-SEP-10

                                                   Table 2 - E - 7 - 8

                               IESCOPD - Meta-analysis of amount smoked : key value (2) 30
                             Any CB, cigarettes (or any product if cigarettes not available)
                                             Potentially overlapping studies


     REF| REFGP|PRINC|                     OVERLAP|

  FERRI1 FERRIS     2         FERRI1/FERRI2/FERRI3
   KAHN2   KAHN     2                   KAHN/KAHN2


  ________________________________________________________________________________________________________________________
                                            International Evidence on Smoking and COPD, Phase 3, Analysis run on 28-SEP-10

                                                    Table 2 - E - 8 -

                               IESCOPD - Meta-analysis of amount smoked : key value (2) 40
                             Any CB, cigarettes (or any product if cigarettes not available)


This analysis is restricted to results for:
1) Eligible study on database
2) Outcome CB
3) Current or ever smoking
4) Categorical dose-response data for amount smoked
5) vs never smoking base
6) Key value (scheme 2) = 40
7) Results complete enough for use in meta-analysis

Within each study, results are then selected (in the following order of preference, within each sex) for:
8) SMKSTA  : current, ever
9) UNEXP   : never any, never cigarettes
10) PROD    : cigarettes, cigarettes only, any product
11) For overlapping studies: principal rather than subsidiary studies
and then for single sex results (m, f) in preference to results for both sexes combined (b).

Results adjusted for the most potential confounders are then chosen in Sections -1 to -3
and results adjusted for the least confounders in Sections -4 to -6. (Those least-adjusted results which
actually differ from the most-adjusted are marked 'x' in column X in Section -4)

Section -7 shows excluded studies, together with the stage (as above) at which no qualifying
results were found.

Section -8 lists the potentially overlapping studies which have been included (1=principal, 2=subsidiary),
and any results which would have been included in preference except that they had data not complete enough
for use in meta-analysis. It also lists their significance (yes/no), if known.


  ________________________________________________________________________________________________________________________
                                            International Evidence on Smoking and COPD, Phase 3, Analysis run on 28-SEP-10

                                                   Table 2 - E - 8 - 1

                               IESCOPD - Meta-analysis of amount smoked : key value (2) 40
                             Any CB, cigarettes (or any product if cigarettes not available)
                                                      Most-adjusted


     REF|NRR|SEX|AGEL|AGEH|     REGION|BEGYR|PUBYR|STTYP|ONSET|      DISEAS|ADJ|SMOKSTA|   PRODUCT|    UNEXP|LOW| HI|

  FERRI1 144   m   25   74      Am:USA  1961  1971    CS  Prev  CB:symptoms   1 Current       Cigs   Nev any  31  40


  ________________________________________________________________________________________________________________________
                                            International Evidence on Smoking and COPD, Phase 3, Analysis run on 28-SEP-10

                                                   Table 2 - E - 8 - 2

                               IESCOPD - Meta-analysis of amount smoked : key value (2) 40
                             Any CB, cigarettes (or any product if cigarettes not available)
                                                      Most-adjusted


                        Number Exposed  Non-exposed
 REF    NRR SEX ADJ     Case    Cont    Case    Cont      RR        95.00%CI
 FERRI1 144 m   1         20       -      10       -      9.81 (  3.75-  25.68)
Partial Totals            20       0      10       0
*prospective study


 REF    NRR SEX ADJ             Ys       Ws       Qs       Ps
 FERRI1 144 m   1              2.28     4.15     0.00       0.00

                       N        1
                      NS        1


                      Wt     4.15
                 Het Chi     0.00
                 Het  df        0
                 Het  P      N.S.
               Fixed  RR     9.81
                     RRl     3.75
                     RRu    25.67
                      P       +++
              Random  RR     9.81
                     RRl     3.75
                     RRu    25.67
                      P       +++
               Asymm  P


  ________________________________________________________________________________________________________________________
                                            International Evidence on Smoking and COPD, Phase 3, Analysis run on 28-SEP-10

                                                   Table 2 - E - 8 - 3

                               IESCOPD - Meta-analysis of amount smoked : key value (2) 40
                             Any CB, cigarettes (or any product if cigarettes not available)
                                                      Most-adjusted


                       N        1
                      NS        1


                      Wt     4.15
                 Het Chi     0.00
                 Het  df        0
                 Het  P      N.S.
               Fixed  RR     9.81
                     RRl     3.75
                     RRu    25.67
                      P       +++
              Random  RR     9.81
                     RRl     3.75
                     RRu    25.67
                      P       +++
               Asymm  P

                                   Sex
                             both      male    female     Total


                       N                  1                   1
                      NS                  1                   1


                      Wt               4.15                4.15
                 Het Chi               0.00                0.00
                 Het  df                  0                   0
                 Het  P                N.S.                N.S.
               Fixed  RR               9.81                9.81
                     RRl               3.75                3.75
                     RRu              25.67               25.67
                      P                 +++                 +++
              Random  RR               9.81                9.81
                     RRl               3.75                3.75
                     RRu              25.67               25.67
                      P                 +++                 +++
             Between Chi
             Between  df
             Between  P                                    N.S.
             Btwn(F)  P                                    N.S.

                                        Continent
                            NAmer    Europe      Asia  oth/mult     Total


                       N        1                                       1
                      NS        1                                       1


                      Wt     4.15                                    4.15
                 Het Chi     0.00                                    0.00
                 Het  df        0                                       0
                 Het  P      N.S.                                    N.S.
               Fixed  RR     9.81                                    9.81
                     RRl     3.75                                    3.75
                     RRu    25.67                                   25.67
                      P       +++                                     +++
              Random  RR     9.81                                    9.81
                     RRl     3.75                                    3.75
                     RRu    25.67                                   25.67
                      P       +++                                     +++
             Between Chi
             Between  df
             Between  P                                              N.S.
             Btwn(F)  P                                              N.S.


  ________________________________________________________________________________________________________________________
                                            International Evidence on Smoking and COPD, Phase 3, Analysis run on 28-SEP-10

                                                   Table 2 - E - 8 - 3

                               IESCOPD - Meta-analysis of amount smoked : key value (2) 40
                             Any CB, cigarettes (or any product if cigarettes not available)
                                                      Most-adjusted
                               Study type
                               CC        Pr        CS     Total


                       N                            1         1
                      NS                            1         1


                      Wt                         4.15      4.15
                 Het Chi                         0.00      0.00
                 Het  df                            0         0
                 Het  P                          N.S.      N.S.
               Fixed  RR                         9.81      9.81
                     RRl                         3.75      3.75
                     RRu                        25.67     25.67
                      P                           +++       +++
              Random  RR                         9.81      9.81
                     RRl                         3.75      3.75
                     RRu                        25.67     25.67
                      P                           +++       +++
             Between Chi
             Between  df
             Between  P                                    N.S.
             Btwn(F)  P                                    N.S.

                           CB subtype
                             mort     sympt     other     Total


                       N                  1                   1
                      NS                  1                   1


                      Wt               4.15                4.15
                 Het Chi               0.00                0.00
                 Het  df                  0                   0
                 Het  P                N.S.                N.S.
               Fixed  RR               9.81                9.81
                     RRl               3.75                3.75
                     RRu              25.67               25.67
                      P                 +++                 +++
              Random  RR               9.81                9.81
                     RRl               3.75                3.75
                     RRu              25.67               25.67
                      P                 +++                 +++
             Between Chi
             Between  df
             Between  P                                    N.S.
             Btwn(F)  P                                    N.S.

                             Smoking product
                              any      cigs  cigsonly     Total


                       N                  1                   1
                      NS                  1                   1


                      Wt               4.15                4.15
                 Het Chi               0.00                0.00
                 Het  df                  0                   0
                 Het  P                N.S.                N.S.
               Fixed  RR               9.81                9.81
                     RRl               3.75                3.75
                     RRu              25.67               25.67
                      P                 +++                 +++
              Random  RR               9.81                9.81
                     RRl               3.75                3.75
                     RRu              25.67               25.67
                      P                 +++                 +++
             Between Chi
             Between  df
             Between  P                                    N.S.
             Btwn(F)  P                                    N.S.
  ________________________________________________________________________________________________________________________
                                            International Evidence on Smoking and COPD, Phase 3, Analysis run on 28-SEP-10

                                                   Table 2 - E - 8 - 3

                               IESCOPD - Meta-analysis of amount smoked : key value (2) 40
[truncated: 915,646 more chars]
