# Supplementary material for: Systematic review with meta-analysis of the epidemiological evidence relating smoking to COPD, chronic bronchitis and emphysema
Source: BMC Pulm Med. 2011 Jun 14;11:36. doi: 10.1186/1471-2466-11-36 (PMC3128042; doi:10.1186/1471-2466-11-36)
Supplement: Additional file 9 — MetaDoseEMP. .RTF file giving the full results of the meta-analyses for the dose-related smoking variables for EMP. [file 1471-2466-11-36-S9.RTF]

Systematic review with meta-analysis of the epidemiological evidence relating smoking to COPD, chronic bronchitis and emphysema

Barbara A Forey, Alison J Thornton and Peter N Lee

Additional file 9 : MetaDoseEMP

See Additional file 10 (Intro sheet) for list of tables and page numbers

                                                    Table 3 - E - 1 -

                                IESCOPD - Meta-analysis of amount smoked : key value (1) 5
                          Any Emphysema, cigarettes (or any product if cigarettes not available)


This analysis is restricted to results for:
1) Eligible study on database
2) Outcome Emphysema
3) Current or ever smoking
4) Categorical dose-response data for amount smoked
5) vs never smoking base
6) Key value (scheme 1) = 5
7) Results complete enough for use in meta-analysis

Within each study, results are then selected (in the following order of preference, within each sex) for:
8) SMKSTA  : current, ever
9) UNEXP   : never any, never cigarettes
10) PROD    : cigarettes, cigarettes only, any product
11) For overlapping studies: principal rather than subsidiary studies
and then for single sex results (m, f) in preference to results for both sexes combined (b).

Results adjusted for the most potential confounders are then chosen in Sections -1 to -3
and results adjusted for the least confounders in Sections -4 to -6. (Those least-adjusted results which
actually differ from the most-adjusted are marked 'x' in column X in Section -4)

Section -7 shows excluded studies, together with the stage (as above) at which no qualifying
results were found.

Section -8 lists the potentially overlapping studies which have been included (1=principal, 2=subsidiary),
and any results which would have been included in preference except that they had data not complete enough
for use in meta-analysis. It also lists their significance (yes/no), if known.


  ________________________________________________________________________________________________________________________
                                            International Evidence on Smoking and COPD, Phase 3, Analysis run on 28-SEP-10

                                                   Table 3 - E - 1 - 1

                                IESCOPD - Meta-analysis of amount smoked : key value (1) 5
                          Any Emphysema, cigarettes (or any product if cigarettes not available)
                                                      Most-adjusted


     REF|NRR|SEX|AGEL|AGEH|     REGION|BEGYR|PUBYR|STTYP|ONSET|      DISEAS|ADJ|SMOKSTA|   PRODUCT|    UNEXP|LOW| HI|

  AUERBA   6   m   15   99      Am:USA  1963  1972    CS  Prev  Emp:viscomp   1 Current       Cigs   Nev any   1   9
    BEST  21   m   30   97   Am:Canada  1955  1967    Pr   Inc     Emp:mort   1 Current  Cigs only   Nev any   1   9
  DONTA2  14   m   25   84 Eu:SE/Balkn  1960  1984    Pr   Inc Emp:diagnosd   0 Current       Cigs  Nev cigs   1   9
  HAMMO2  13   f   35   99      Am:USA  1959  1966    Pr   Inc     Emp:mort   1    Ever       Cigs   Nev any   1   9
  HUHTI1  71   m   40   64    Eu:Scand  1961  1965    CS  Prev    Emp:other   0 Current       Cigs   Nev any   1  14
   KAHN2  48   m   31   84      Am:USA  1954  1966    Pr   Inc     Emp:mort   1 Current       Cigs   Nev any   1   9
  LAVECC  40   b   15   99     Eu:West  1983  1988    CS  Prev Emp:self-rep   6 Current       Cigs   Nev any   1  14
   WEISS  19   m   50   69      Am:USA  1961  1963    CS  Prev  Emp:viscomp   0 Current  Cigs only   Nev any   1   9
     WEN  19   m   35   99   Asia:FarE  1982  2004    Pr   Inc     Emp:mort   1 Current       Cigs  Nev cigs   1  10


  ________________________________________________________________________________________________________________________
                                            International Evidence on Smoking and COPD, Phase 3, Analysis run on 28-SEP-10

                                                   Table 3 - E - 1 - 2

                                IESCOPD - Meta-analysis of amount smoked : key value (1) 5
                          Any Emphysema, cigarettes (or any product if cigarettes not available)
                                                      Most-adjusted


                        Number Exposed  Non-exposed
 REF    NRR SEX ADJ     Case    Cont    Case    Cont      RR        95.00%CI
 AUERBA 6   m   1         48       -      20       -     29.46 ( 11.92-  72.77)
*BEST   21  m   1          9       -       3       -      4.81 (  1.30-  17.76)
*DONTA2 14  m   0          1     118       4     127      0.27 (  0.03-   2.37)
*HAMMO2 13  f   1          9       -      21       -      3.69 (  1.69-   8.07)
 HUHTI1 71  m   0         20      88       1     121     27.50 (  3.62- 208.77)
*KAHN2  48  m   1         22       -      18       -      5.58 (  2.99-  10.40)
 LAVECC 40  b   6        202       -     595       -      1.77 (  1.48-   2.12)
 WEISS  19  m   0          4      11       1      35     12.73 (  1.28- 126.14)
*WEN    19  m   1          -       -      13       -      0.85 (  0.22-   3.31)
Partial Totals           315     217     676     283
*prospective study


 REF    NRR SEX ADJ             Ys       Ws       Qs       Ps
 AUERBA 6   m   1              3.38     4.70    31.53       0.00
*BEST   21  m   1              1.57     2.25     1.36       0.02
*DONTA2 14  m   0             -1.31     0.81     3.59       0.24
*HAMMO2 13  f   1              1.31     6.29     1.66       0.00
 HUHTI1 71  m   0              3.31     0.93     5.95       0.00
*KAHN2  48  m   1              1.72     9.89     8.51       0.00
 LAVECC 40  b   6              0.57   118.98     5.79       0.00
 WEISS  19  m   0              2.54     0.73     2.24       0.03
*WEN    19  m   1             -0.16     2.09     1.90       0.81

                       N        9
                      NS        9


                      Wt   146.66
                 Het Chi    62.54
                 Het  df        8
                 Het  P       ***
               Fixed  RR     2.21
                     RRl     1.88
                     RRu     2.59
                      P       +++
              Random  RR     4.24
                     RRl     1.88
                     RRu     9.55
                      P       +++
               Asymm  P      N.S.


  ________________________________________________________________________________________________________________________
                                            International Evidence on Smoking and COPD, Phase 3, Analysis run on 28-SEP-10

                                                   Table 3 - E - 1 - 3

                                IESCOPD - Meta-analysis of amount smoked : key value (1) 5
                          Any Emphysema, cigarettes (or any product if cigarettes not available)
                                                      Most-adjusted


                       N        9
                      NS        9


                      Wt   146.66
                 Het Chi    62.54
                 Het  df        8
                 Het  P       ***
               Fixed  RR     2.21
                     RRl     1.88
                     RRu     2.59
                      P       +++
              Random  RR     4.24
                     RRl     1.88
                     RRu     9.55
                      P       +++
               Asymm  P      N.S.

                                   Sex
                             both      male    female     Total


                       N        1         7         1         9
                      NS        1         7         1         9


                      Wt   118.98     21.40      6.29    146.66
                 Het Chi     0.00     30.30      0.00     62.54
                 Het  df        0         6         0         8
                 Het  P      N.S.       ***      N.S.       ***
               Fixed  RR     1.77      6.47      3.69      2.21
                     RRl     1.48      4.24      1.69      1.88
                     RRu     2.12      9.89      8.06      2.59
                      P       +++       +++        ++       +++
              Random  RR     1.77      5.15      3.69      4.24
                     RRl     1.48      1.72      1.69      1.88
                     RRu     2.12     15.44      8.06      9.55
                      P       +++        ++        ++       +++
             Between Chi                                  32.23
             Between  df                                      2
             Between  P                                     ***
             Btwn(F)  P                                    N.S.

                                        Continent
                            NAmer    Europe      Asia  oth/mult     Total


                       N        5         3         1                   9
                      NS        5         3         1                   9


                      Wt    23.85    120.72      2.09              146.66
                 Het Chi    13.36      9.90      0.00               62.54
                 Het  df        4         2         0                   8
                 Het  P        **        **      N.S.                 ***
               Fixed  RR     7.02      1.79      0.85                2.21
                     RRl     4.70      1.49      0.22                1.88
                     RRu    10.49      2.13      3.30                2.59
                      P       +++       +++      N.S.                 +++
              Random  RR     7.72      2.34      0.85                4.24
                     RRl     3.41      0.33      0.22                1.88
                     RRu    17.47     16.31      3.30                9.55
                      P       +++      N.S.      N.S.                 +++
             Between Chi                                            39.28
             Between  df                                                2
             Between  P                                               ***
             Btwn(F)  P                                               (*)


  ________________________________________________________________________________________________________________________
                                            International Evidence on Smoking and COPD, Phase 3, Analysis run on 28-SEP-10

                                                   Table 3 - E - 1 - 3

                                IESCOPD - Meta-analysis of amount smoked : key value (1) 5
                          Any Emphysema, cigarettes (or any product if cigarettes not available)
                                                      Most-adjusted
                        National cigarette tobacco type (excluding mixed/unkown)
                          blended  virginia     Total


                       N        7         1         8
                      NS        7         1         8


                      Wt   142.32      2.25    144.57
                 Het Chi    59.27      0.00     60.61
                 Het  df        6         0         7
                 Het  P       ***      N.S.       ***
               Fixed  RR     2.21      4.81      2.24
                     RRl     1.88      1.30      1.90
                     RRu     2.61     17.78      2.63
                      P       +++         +       +++
              Random  RR     5.21      4.81      5.15
                     RRl     1.97      1.30      2.14
                     RRu    13.75     17.78     12.42
                      P       +++         +       +++
             Between Chi                         1.34
             Between  df                            1
             Between  P                          N.S.
             Btwn(F)  P                          N.S.

                                        Start year of study
                            <1970   1970-79   1980-89   1990-99     2000+   unknown     Total


                       N        7                   2                                       9
                      NS        7                   2                                       9


                      Wt    25.59              121.07                                  146.66
                 Het Chi    23.65                1.11                                   62.54
                 Het  df        6                   1                                       8
                 Het  P       ***                N.S.                                     ***
               Fixed  RR     6.66                1.75                                    2.21
                     RRl     4.52                1.46                                    1.88
                     RRu     9.81                2.09                                    2.59
                      P       +++                 +++                                     +++
              Random  RR     6.47                1.69                                    4.24
                     RRl     2.67                1.19                                    1.88
                     RRu    15.68                2.40                                    9.55
                      P       +++                  ++                                     +++
             Between Chi                                                                37.78
             Between  df                                                                    1
             Between  P                                                                   ***
             Btwn(F)  P                                                                     *

                                Publication year
                            <1980   1980-89   1990-99     2000+     Total


                       N        6         2                   1         9
                      NS        6         2                   1         9


                      Wt    24.78    119.79                2.09    146.66
                 Het Chi    15.04      2.86                0.00     62.54
                 Het  df        5         1                   0         8
                 Het  P         *       (*)                N.S.       ***
               Fixed  RR     7.39      1.75                0.85      2.21
                     RRl     4.99      1.46                0.22      1.88
                     RRu    10.96      2.09                3.30      2.59
                      P       +++       +++                N.S.       +++
              Random  RR     8.73      0.96                0.85      4.24
                     RRl     4.02      0.17                0.22      1.88
                     RRu    18.99      5.40                3.30      9.55
                      P       +++      N.S.                N.S.       +++
             Between Chi                                            44.65
             Between  df                                                2
             Between  P                                               ***
             Btwn(F)  P                                                 *
  ________________________________________________________________________________________________________________________
                                            International Evidence on Smoking and COPD, Phase 3, Analysis run on 28-SEP-10

                                                   Table 3 - E - 1 - 3

                                IESCOPD - Meta-analysis of amount smoked : key value (1) 5
                          Any Emphysema, cigarettes (or any product if cigarettes not available)
                                                      Most-adjusted
                               Study type
                               CC        Pr        CS     Total


                       N                  5         4         9
                      NS                  5         4         9


                      Wt              21.32    125.34    146.66
                 Het Chi              11.90     44.64     62.54
                 Het  df                  4         3         8
                 Het  P                   *       ***       ***
               Fixed  RR               3.60      2.03      2.21
                     RRl               2.36      1.70      1.88
                     RRu               5.51      2.42      2.59
                      P                 +++       +++       +++
              Random  RR               2.57     10.70      4.24
                     RRl               1.11      1.51      1.88
                     RRu               5.92     75.91      9.55
                      P                   +         +       +++
             Between Chi                                   6.00
             Between  df                                      1
             Between  P                                       *
             Btwn(F)  P                                    N.S.

                                    Lowest age in RR
                        <25/unlim     25-39       40+   unknown     Total


                       N        2         5         2                   9
                      NS        2         5         2                   9


                      Wt   123.67     21.32      1.67              146.66
                 Het Chi    35.72     11.90      0.24               62.54
                 Het  df        1         4         1                   8
                 Het  P       ***         *      N.S.                 ***
               Fixed  RR     1.97      3.60     19.62                2.21
                     RRl     1.65      2.36      4.29                1.88
                     RRu     2.35      5.51     89.59                2.59
                      P       +++       +++       +++                 +++
              Random  RR     6.96      2.57     19.62                4.24
                     RRl     0.44      1.11      4.29                1.88
                     RRu   109.45      5.92     89.59                9.55
                      P      N.S.         +       +++                 +++
             Between Chi                                            14.68
             Between  df                                                2
             Between  P                                               ***
             Btwn(F)  P                                              N.S.

                                         Highest age in RR
                              <65     65-74     75-84 85+/unlim   unknown     Total


                       N        1         1         2         5                   9
                      NS        1         1         2         5                   9


                      Wt     0.93      0.73     10.70    134.30              146.66
                 Het Chi     0.00      0.00      6.89     41.33               62.54
                 Het  df        0         0         1         4                   8
                 Het  P      N.S.      N.S.        **       ***                 ***
               Fixed  RR    27.50     12.73      4.43      2.03                2.21
                     RRl     3.62      1.28      2.44      1.72                1.88
                     RRu   208.77    126.14      8.07      2.41                2.59
                      P        ++         +       +++       +++                 +++
              Random  RR    27.50     12.73      1.48      3.85                4.24
                     RRl     3.62      1.28      0.08      1.29                1.88
                     RRu   208.77    126.14     28.18     11.49                9.55
                      P        ++         +      N.S.         +                 +++
             Between Chi                                                      14.32
             Between  df                                                          3
             Between  P                                                          **
             Btwn(F)  P                                                        N.S.
  ________________________________________________________________________________________________________________________
                                            International Evidence on Smoking and COPD, Phase 3, Analysis run on 28-SEP-10

                                                   Table 3 - E - 1 - 3

                                IESCOPD - Meta-analysis of amount smoked : key value (1) 5
                          Any Emphysema, cigarettes (or any product if cigarettes not available)
                                                      Most-adjusted
                           Study weakness
                              Yes        No     Total


                       N        1         8         9
                      NS        1         8         9


                      Wt     0.81    145.85    146.66
                 Het Chi     0.00     58.93     62.54
                 Het  df        0         7         8
                 Het  P      N.S.       ***       ***
               Fixed  RR     0.27      2.23      2.21
                     RRl     0.03      1.90      1.88
                     RRu     2.37      2.63      2.59
                      P      N.S.       +++       +++
              Random  RR     0.27      5.26      4.24
                     RRl     0.03      2.28      1.88
                     RRu     2.37     12.16      9.55
                      P      N.S.       +++       +++
             Between Chi                         3.61
             Between  df                            1
             Between  P                           (*)
             Btwn(F)  P                          N.S.

                           Type of outcome
                        prevalnce mortality incidence     Total


                       N        4         4         1         9
                      NS        4         4         1         9


                      Wt   125.34     20.51      0.81    146.66
                 Het Chi    44.64      6.23      0.00     62.54
                 Het  df        3         3         0         8
                 Het  P       ***      N.S.      N.S.       ***
               Fixed  RR     2.03      3.99      0.27      2.21
                     RRl     1.70      2.59      0.03      1.88
                     RRu     2.42      6.15      2.37      2.59
                      P       +++       +++      N.S.       +++
              Random  RR    10.70      3.50      0.27      4.24
                     RRl     1.51      1.77      0.03      1.88
                     RRu    75.91      6.91      2.37      9.55
                      P         +       +++      N.S.       +++
             Between Chi                                  11.67
             Between  df                                      2
             Between  P                                      **
             Btwn(F)  P                                    N.S.

                        Emp subtype
                             mort     other     Total


                       N        4         5         9
                      NS        4         5         9


                      Wt    20.51    126.15    146.66
                 Het Chi     6.23     47.93     62.54
                 Het  df        3         4         8
                 Het  P      N.S.       ***       ***
               Fixed  RR     3.99      2.00      2.21
                     RRl     2.59      1.68      1.88
                     RRu     6.15      2.39      2.59
                      P       +++       +++       +++
              Random  RR     3.50      5.58      4.24
                     RRl     1.77      0.99      1.88
                     RRu     6.91     31.45      9.55
                      P       +++       (+)       +++
             Between Chi                         8.38
             Between  df                            1
             Between  P                            **
             Btwn(F)  P                          N.S.
  ________________________________________________________________________________________________________________________
                                            International Evidence on Smoking and COPD, Phase 3, Analysis run on 28-SEP-10

                                                   Table 3 - E - 1 - 3

                                IESCOPD - Meta-analysis of amount smoked : key value (1) 5
                          Any Emphysema, cigarettes (or any product if cigarettes not available)
                                                      Most-adjusted
                        Asthma analysis type (Emphysema)
                        inc-irres  excl-all     Total


                       N        8         1         9
                      NS        8         1         9


                      Wt   145.85      0.81    146.66
                 Het Chi    58.93      0.00     62.54
                 Het  df        7         0         8
                 Het  P       ***      N.S.       ***
               Fixed  RR     2.23      0.27      2.21
                     RRl     1.90      0.03      1.88
                     RRu     2.63      2.37      2.59
                      P       +++      N.S.       +++
              Random  RR     5.26      0.27      4.24
                     RRl     2.28      0.03      1.88
                     RRu    12.16      2.37      9.55
                      P       +++      N.S.       +++
             Between Chi                         3.61
             Between  df                            1
             Between  P                           (*)
             Btwn(F)  P                          N.S.

                                Number of emphysema cases
                             1-50    51-100   101-200      201+     Total


                       N        3         2                   4         9
                      NS        3         2                   4         9


                      Wt     3.63      3.18              139.85    146.66
                 Het Chi     6.12      2.01               47.47     62.54
                 Het  df        2         1                   3         8
                 Het  P         *      N.S.                 ***       ***
               Fixed  RR     1.13      8.03                2.18      2.21
                     RRl     0.41      2.68                1.85      1.88
                     RRu     3.17     24.08                2.57      2.59
                      P      N.S.       +++                 +++       +++
              Random  RR     1.32      9.61                5.44      4.24
                     RRl     0.19      1.81                1.78      1.88
                     RRu     9.19     51.18               16.64      9.55
                      P      N.S.        ++                  ++       +++
             Between Chi                                             6.94
             Between  df                                                2
             Between  P                                                 *
             Btwn(F)  P                                              N.S.

                        Number of emphysma cases (excluding unknown)


                       N        3         2                   4         9
                      NS        3         2                   4         9


                      Wt     3.63      3.18              139.85    146.66
                 Het Chi     6.12      2.01               47.47     62.54
                 Het  df        2         1                   3         8
                 Het  P         *      N.S.                 ***       ***
               Fixed  RR     1.13      8.03                2.18      2.21
                     RRl     0.41      2.68                1.85      1.88
                     RRu     3.17     24.08                2.57      2.59
                      P      N.S.       +++                 +++       +++
              Random  RR     1.32      9.61                5.44      4.24
                     RRl     0.19      1.81                1.78      1.88
                     RRu     9.19     51.18               16.64      9.55
                      P      N.S.        ++                  ++       +++
             Between Chi                                             6.94
             Between  df                                                2
             Between  P                                                 *
             Btwn(F)  P                                              N.S.

  ________________________________________________________________________________________________________________________
                                            International Evidence on Smoking and COPD, Phase 3, Analysis run on 28-SEP-10

                                                   Table 3 - E - 1 - 3

                                IESCOPD - Meta-analysis of amount smoked : key value (1) 5
                          Any Emphysema, cigarettes (or any product if cigarettes not available)
                                                      Most-adjusted
                            Analysis type
                         prevlnce     onset     Total


                       N        4         5         9
                      NS        4         5         9


                      Wt   125.34     21.32    146.66
                 Het Chi    44.64     11.90     62.54
                 Het  df        3         4         8
                 Het  P       ***         *       ***
               Fixed  RR     2.03      3.60      2.21
                     RRl     1.70      2.36      1.88
                     RRu     2.42      5.51      2.59
                      P       +++       +++       +++
              Random  RR    10.70      2.57      4.24
                     RRl     1.51      1.11      1.88
                     RRu    75.91      5.92      9.55
                      P         +         +       +++
             Between Chi                         6.00
             Between  df                            1
             Between  P                             *
             Btwn(F)  P                          N.S.

                             Smoking product
                              any      cigs  cigsonly     Total


                       N                  7         2         9
                      NS                  7         2         9


                      Wt             143.68      2.98    146.66
                 Het Chi              58.87      0.52     62.54
                 Het  df                  6         1         8
                 Het  P                 ***      N.S.       ***
               Fixed  RR               2.16      6.11      2.21
                     RRl               1.84      1.96      1.88
                     RRu               2.54     19.01      2.59
                      P                 +++        ++       +++
              Random  RR               3.79      6.11      4.24
                     RRl               1.50      1.96      1.88
                     RRu               9.55     19.01      9.55
                      P                  ++        ++       +++
             Between Chi                                   3.15
             Between  df                                      1
             Between  P                                     (*)
             Btwn(F)  P                                    N.S.

                                     Unexposed group
                          nev any   nev cig  nev+ any  nev+ cig     Total


                       N        7         2                             9
                      NS        7         2                             9


                      Wt   143.76      2.90                        146.66
                 Het Chi    56.95      0.77                         62.54
                 Het  df        6         1                             8
                 Het  P       ***      N.S.                           ***
               Fixed  RR     2.26      0.62                          2.21
                     RRl     1.92      0.20                          1.88
                     RRu     2.67      1.95                          2.59
                      P       +++      N.S.                           +++
              Random  RR     6.73      0.62                          4.24
                     RRl     2.70      0.20                          1.88
                     RRu    16.75      1.95                          9.55
                      P       +++      N.S.                           +++
             Between Chi                                             4.82
             Between  df                                                1
             Between  P                                                 *
             Btwn(F)  P                                              N.S.
  ________________________________________________________________________________________________________________________
                                            International Evidence on Smoking and COPD, Phase 3, Analysis run on 28-SEP-10

                                                   Table 3 - E - 1 - 3

                                IESCOPD - Meta-analysis of amount smoked : key value (1) 5
                          Any Emphysema, cigarettes (or any product if cigarettes not available)
                                                      Most-adjusted
                        Unexposed group (combining nev+ with main levels)
                          nev any   nev cig     Total


                       N        7         2         9
                      NS        7         2         9


                      Wt   143.76      2.90    146.66
                 Het Chi    56.95      0.77     62.54
                 Het  df        6         1         8
                 Het  P       ***      N.S.       ***
               Fixed  RR     2.26      0.62      2.21
                     RRl     1.92      0.20      1.88
                     RRu     2.67      1.95      2.59
                      P       +++      N.S.       +++
              Random  RR     6.73      0.62      4.24
                     RRl     2.70      0.20      1.88
                     RRu    16.75      1.95      9.55
                      P       +++      N.S.       +++
             Between Chi                         4.82
             Between  df                            1
             Between  P                             *
             Btwn(F)  P                          N.S.

                        Number of adjustment variables
                                0         1        2+     Total


                       N        3         5         1         9
                      NS        3         5         1         9


                      Wt     2.48     25.21    118.98    146.66
                 Het Chi    10.27     21.49      0.00     62.54
                 Het  df        2         4         0         8
                 Het  P        **       ***      N.S.       ***
               Fixed  RR     4.82      5.79      1.77      2.21
                     RRl     1.39      3.92      1.48      1.88
                     RRu    16.74      8.56      2.12      2.59
                      P         +       +++       +++       +++
              Random  RR     4.59      5.11      1.77      4.24
                     RRl     0.27      1.95      1.48      1.88
                     RRu    77.88     13.37      2.12      9.55
                      P      N.S.       +++       +++       +++
             Between Chi                                  30.78
             Between  df                                      2
             Between  P                                     ***
             Btwn(F)  P                                    N.S.


  ________________________________________________________________________________________________________________________
                                            International Evidence on Smoking and COPD, Phase 3, Analysis run on 28-SEP-10

                                                   Table 3 - E - 1 - 4

                                IESCOPD - Meta-analysis of amount smoked : key value (1) 5
                          Any Emphysema, cigarettes (or any product if cigarettes not available)
                                                      Least-adjusted


     REF|NRR|X|SEX|AGEL|AGEH|     REGION|BEGYR|PUBYR|STTYP|ONSET|      DISEAS|ADJ|SMOKSTA|   PRODUCT|    UNEXP|LOW| HI|

  AUERBA   3 x   m   15   99      Am:USA  1963  1972    CS  Prev  Emp:viscomp   0 Current       Cigs   Nev any   1   9
    BEST  21     m   30   97   Am:Canada  1955  1967    Pr   Inc     Emp:mort   1 Current  Cigs only   Nev any   1   9
  DONTA2  14     m   25   84 Eu:SE/Balkn  1960  1984    Pr   Inc Emp:diagnosd   0 Current       Cigs  Nev cigs   1   9
  HAMMO2  13     f   35   99      Am:USA  1959  1966    Pr   Inc     Emp:mort   1    Ever       Cigs   Nev any   1   9
  HUHTI1  71     m   40   64    Eu:Scand  1961  1965    CS  Prev    Emp:other   0 Current       Cigs   Nev any   1  14
   KAHN2  48     m   31   84      Am:USA  1954  1966    Pr   Inc     Emp:mort   1 Current       Cigs   Nev any   1   9
  LAVECC  34 x   m   15   99     Eu:West  1983  1988    CS  Prev Emp:self-rep   0 Current       Cigs   Nev any   1  14
  LAVECC  37 x   f   15   99     Eu:West  1983  1988    CS  Prev Emp:self-rep   0 Current       Cigs   Nev any   1  14
   WEISS  19     m   50   69      Am:USA  1961  1963    CS  Prev  Emp:viscomp   0 Current  Cigs only   Nev any   1   9
     WEN  19     m   35   99   Asia:FarE  1982  2004    Pr   Inc     Emp:mort   1 Current       Cigs  Nev cigs   1  10


  ________________________________________________________________________________________________________________________
                                            International Evidence on Smoking and COPD, Phase 3, Analysis run on 28-SEP-10

                                                   Table 3 - E - 1 - 5

                                IESCOPD - Meta-analysis of amount smoked : key value (1) 5
                          Any Emphysema, cigarettes (or any product if cigarettes not available)
                                                      Least-adjusted


                        Number Exposed  Non-exposed
 REF    NRR SEX ADJ     Case    Cont    Case    Cont      RR        95.00%CI
 AUERBA 3   m   0         48      18      20     156     20.80 ( 10.18-  42.49)
*BEST   21  m   1          9       -       3       -      4.81 (  1.30-  17.76)
*DONTA2 14  m   0          1     118       4     127      0.27 (  0.03-   2.37)
*HAMMO2 13  f   1          9       -      21       -      3.69 (  1.69-   8.07)
 HUHTI1 71  m   0         20      88       1     121     27.50 (  3.62- 208.77)
*KAHN2  48  m   1         22       -      18       -      5.58 (  2.99-  10.40)
 LAVECC 34  m   0        170    5341     183   14129      2.46 (  1.99-   3.04)
 LAVECC 37  f   0         32    4475     412   29448      0.51 (  0.36-   0.73)
 Subtotal LAVECC                                          1.65 (  1.37-   1.98)
 WEISS  19  m   0          4      11       1      35     12.73 (  1.28- 126.14)
*WEN    19  m   1          -       -      13       -      0.85 (  0.22-   3.31)
Partial Totals           315   10051     676   44016
*prospective study


 REF    NRR SEX ADJ             Ys       Ws       Qs       Ps
 AUERBA 3   m   0              3.03     7.53    38.56       0.00
*BEST   21  m   1              1.57     2.25     1.43       0.02
*DONTA2 14  m   0             -1.31     0.81     3.52       0.24
*HAMMO2 13  f   1              1.31     6.29     1.79       0.00
 HUHTI1 71  m   0              3.31     0.93     6.04       0.00
*KAHN2  48  m   1              1.72     9.89     8.87       0.00
 LAVECC 34  m   0              0.90    86.17     1.39       0.00
 LAVECC 37  f   0             -0.67    29.47    61.38       0.00
 Subtotal LAVECC               0.50   115.64    62.77
 WEISS  19  m   0              2.54     0.73     2.29       0.03
*WEN    19  m   1             -0.16     2.09     1.83       0.81

                       N       10
                      NS        9


                      Wt   146.16
                 Het Chi   127.11
                 Het  df        9
                 Het  P       ***
               Fixed  RR     2.16
                     RRl     1.84
                     RRu     2.55
                      P       +++
              Random  RR     3.29
                     RRl     1.46
                     RRu     7.43
                      P        ++
               Asymm  P      N.S.


  ________________________________________________________________________________________________________________________
                                            International Evidence on Smoking and COPD, Phase 3, Analysis run on 28-SEP-10

                                                   Table 3 - E - 1 - 6

                                IESCOPD - Meta-analysis of amount smoked : key value (1) 5
                          Any Emphysema, cigarettes (or any product if cigarettes not available)
                                                      Least-adjusted


                       N       10
                      NS        9


                      Wt   146.16
                 Het Chi   127.11
                 Het  df        9
                 Het  P       ***
               Fixed  RR     2.16
                     RRl     1.84
                     RRu     2.55
                      P       +++
              Random  RR     3.29
                     RRl     1.46
                     RRu     7.43
                      P        ++
               Asymm  P      N.S.

                                   Sex
                             both      male    female     Total


                       N                  8         2        10
                      NS                  8         2        10


                      Wt             110.40     35.75    146.16
                 Het Chi              50.04     20.25    127.11
                 Het  df                  7         1         9
                 Het  P                 ***       ***       ***
               Fixed  RR               3.09      0.72      2.16
                     RRl               2.56      0.52      1.84
                     RRu               3.72      1.00      2.55
                      P                 +++       (-)       +++
              Random  RR               4.43      1.33      3.29
                     RRl               1.90      0.19      1.46
                     RRu              10.33      9.22      7.43
                      P                 +++      N.S.        ++
             Between Chi                                  56.83
             Between  df                                      1
             Between  P                                     ***
             Btwn(F)  P                                       *

                                        Continent
                            NAmer    Europe      Asia  oth/mult     Total


                       N        5         4         1                  10
                      NS        5         3         1                   9


                      Wt    26.68    117.38      2.09              146.16
                 Het Chi    12.51     64.21      0.00              127.11
                 Het  df        4         3         0                   9
                 Het  P         *       ***      N.S.                 ***
               Fixed  RR     7.41      1.66      0.85                2.16
                     RRl     5.07      1.39      0.22                1.84
                     RRu    10.83      1.99      3.30                2.55
                      P       +++       +++      N.S.                 +++
              Random  RR     7.33      1.58      0.85                3.29
                     RRl     3.48      0.44      0.22                1.46
                     RRu    15.45      5.73      3.30                7.43
                      P       +++      N.S.      N.S.                  ++
             Between Chi                                            50.40
             Between  df                                                2
             Between  P                                               ***
             Btwn(F)  P                                              N.S.


  ________________________________________________________________________________________________________________________
                                            International Evidence on Smoking and COPD, Phase 3, Analysis run on 28-SEP-10

                                                   Table 3 - E - 1 - 6

                                IESCOPD - Meta-analysis of amount smoked : key value (1) 5
                          Any Emphysema, cigarettes (or any product if cigarettes not available)
                                                      Least-adjusted
                               Study type
                               CC        Pr        CS     Total


                       N                  5         5        10
                      NS                  5         4         9


                      Wt              21.32    124.83    146.16
                 Het Chi              11.90    108.72    127.11
                 Het  df                  4         4         9
                 Het  P                   *       ***       ***
               Fixed  RR               3.60      1.98      2.16
                     RRl               2.36      1.66      1.84
                     RRu               5.51      2.36      2.55
                      P                 +++       +++       +++
              Random  RR               2.57      4.98      3.29
                     RRl               1.11      1.36      1.46
                     RRu               5.92     18.30      7.43
                      P                   +         +        ++
             Between Chi                                   6.49
             Between  df                                      1
             Between  P                                       *
             Btwn(F)  P                                    N.S.

                        Emp subtype
                             mort     other     Total


                       N        4         6        10
                      NS        4         5         9


                      Wt    20.51    125.65    146.16
                 Het Chi     6.23    111.93    127.11
                 Het  df        3         5         9
                 Het  P      N.S.       ***       ***
               Fixed  RR     3.99      1.96      2.16
                     RRl     2.59      1.64      1.84
                     RRu     6.15      2.33      2.55
                      P       +++       +++       +++
              Random  RR     3.50      3.44      3.29
                     RRl     1.77      1.02      1.46
                     RRu     6.91     11.62      7.43
                      P       +++         +        ++
             Between Chi                         8.95
             Between  df                            1
             Between  P                            **
             Btwn(F)  P                          N.S.

                             Smoking product
                              any      cigs  cigsonly     Total


                       N                  8         2        10
                      NS                  7         2         9


                      Wt             143.18      2.98    146.16
                 Het Chi             123.32      0.52    127.11
                 Het  df                  7         1         9
                 Het  P                 ***      N.S.       ***
               Fixed  RR               2.12      6.11      2.16
                     RRl               1.80      1.96      1.84
                     RRu               2.50     19.01      2.55
                      P                 +++        ++       +++
              Random  RR               2.84      6.11      3.29
                     RRl               1.15      1.96      1.46
                     RRu               7.00     19.01      7.43
                      P                   +        ++        ++
             Between Chi                                   3.27
             Between  df                                      1
             Between  P                                     (*)
             Btwn(F)  P                                    N.S.
  ________________________________________________________________________________________________________________________
                                            International Evidence on Smoking and COPD, Phase 3, Analysis run on 28-SEP-10

                                                   Table 3 - E - 1 - 6

                                IESCOPD - Meta-analysis of amount smoked : key value (1) 5
                          Any Emphysema, cigarettes (or any product if cigarettes not available)
                                                      Least-adjusted
                                     Unexposed group
                          nev any   nev cig  nev+ any  nev+ cig     Total


                       N        8         2                            10
                      NS        7         2                             9


                      Wt   143.26      2.90                        146.16
                 Het Chi   121.67      0.77                        127.11
                 Het  df        7         1                             9
                 Het  P       ***      N.S.                           ***
               Fixed  RR     2.22      0.62                          2.16
                     RRl     1.88      0.20                          1.84
                     RRu     2.61      1.95                          2.55
                      P       +++      N.S.                           +++
              Random  RR     4.70      0.62                          3.29
                     RRl     1.92      0.20                          1.46
                     RRu    11.52      1.95                          7.43
                      P       +++      N.S.                            ++
             Between Chi                                             4.67
             Between  df                                                1
             Between  P                                                 *
             Btwn(F)  P                                              N.S.

                        Unexposed group (combining nev+ with main levels)
                          nev any   nev cig     Total


                       N        8         2        10
                      NS        7         2         9


                      Wt   143.26      2.90    146.16
                 Het Chi   121.67      0.77    127.11
                 Het  df        7         1         9
                 Het  P       ***      N.S.       ***
               Fixed  RR     2.22      0.62      2.16
                     RRl     1.88      0.20      1.84
                     RRu     2.61      1.95      2.55
                      P       +++      N.S.       +++
              Random  RR     4.70      0.62      3.29
                     RRl     1.92      0.20      1.46
                     RRu    11.52      1.95      7.43
                      P       +++      N.S.        ++
             Between Chi                         4.67
             Between  df                            1
             Between  P                             *
             Btwn(F)  P                          N.S.


  ________________________________________________________________________________________________________________________
                                            International Evidence on Smoking and COPD, Phase 3, Analysis run on 28-SEP-10

                                                   Table 3 - E - 1 - 7

                                IESCOPD - Meta-analysis of amount smoked : key value (1) 5
                          Any Emphysema, cigarettes (or any product if cigarettes not available)
                                 Excluded studies (and stage at which they were excluded)


1       CLARK COTTON  MEYER REMYJA RUTGER SNYDER SOBRAX     SU TAKEMU  WANG4   WEIR WHICKE ZALACA
2      ALDERS ALESSA  AMIGO ANDER1 ANDER3   BANG  BECK1  BECK2 BEDNAR BJORNS BROGGE  BROWN CERVER CHAPMA  CHEN1  CHEN2
        CHEN3  CHENG CLEMEN COATES  COCCI COLLEG  DEAN1  DEAN2  DEANE DEJONG DEMARC DETORR DICKIN  DOLL1  DOLL2 DONTA1
       DOPICO EHRLIC EKBERG ENSTRO FERRI1 FERRI2 FERRI3  FIDAN FINKLE FLETCH FORAST FOXMAN FUKUCH GEIJER GODTFR GOLDBE
       HAENSZ HARIKK HARRIS HAWTHO  HAYES HEDMAN HIGGI2 HIGGI3 HIGGI4 HIGGI6 HOLLA2 HOLLNA  HOUSE HRUBEC HUCHON HUHTI2
       HUHTI3 ITABAS JACOBS JAENDI JENSEN JINDA2 JOHANN  JOSHI JOUSI1 KACHEL KARAKA KATANC   KATO KHOURY    KIM  KIRAZ
       KLAYTO KOJIMA KOTAN1 KOTAN2 KRZYZA  KUBIK KULLER    LAI   LAM1   LAM2   LAM3 LAMBER  LANGE LANGE2 LANGHA    LEE
         LIAW LINDBE LINDST   LIU1   LIU2 LUNDB1 LUNDB2  MADOR MAGNUS MANFRE MANNI1 MANNI2 MANNI3 MARAN1 MARAN2 MARCUS
       MATHES MELLST MENEZ1 MENEZ2 MENEZ3 MENEZ4 MENEZ5 MENEZ6  MEREN  MILNE MOLLER MONTNE MUELLE NEJJAR NIEPSU NIHLEN
       NILSSO OGILVI OSWAL1 OSWAL2 PANDEY   PEAT PELKON PEREZP   PETO  PRICE   REID RENWIC RICCIO RIMING SARGEA SAWICK
       SCHWAR SHAHAB  SHARP SHIMUR   SHIN SICHLE SOBRAD SPEIZE STERLI STJERN  STROM SUADIC  TAGER TAGER2   TANG   THUN
         TODD TROISI TRUPIN TSUSHI TVERDA URRUTI VESTBO VIEGI1 VIEGI2 VINEIS VOLLM1 VOLLM2 VONHER WAGEN2   WALD WATSON
          WIG WILHEL WILSO1 WOJTYN  WOODS  WOOLF   XIAO     XU YAMAGU   YUAN ZIELI1 ZIELI2 ZIETKO   ZOIA
3        KAHN
4      ANDER2 ENRIGH GULSVI HARDIE     HO HOZAWA LEBOWI MILLER   NAWA  OMORI  PRATT  RYDER  SILVA SUTINE VIKGRE  WANG2
5      WILSO2
7      HIRAYA


  ________________________________________________________________________________________________________________________
                                            International Evidence on Smoking and COPD, Phase 3, Analysis run on 28-SEP-10

                                                   Table 3 - E - 1 - 8

                                IESCOPD - Meta-analysis of amount smoked : key value (1) 5
                          Any Emphysema, cigarettes (or any product if cigarettes not available)
                                             Potentially overlapping studies


     REF| REFGP|PRINC|                     OVERLAP|

  DONTA2 JACOBS     2  JACOBS/DONTA1/DONTA2/PELKON
  HUHTI1 HUHTI1     1                HUHTI1/HUHTI2
  HAMMO2 HAMMO2     1                HAMMO2/ENSTRO
     WEN    WEN     1                     WEN/LIAW
   KAHN2   KAHN     2                   KAHN/KAHN2

                                    Most-adjusted - insufficient data for meta-analysis
     REF|NRR|SEX|AGEL|AGEH|     REGION|BEGYR|PUBYR|STTYP|ONSET|      DISEAS|ADJ|SMOKSTA|   PRODUCT|    UNEXP|LOW| HI|

  HIRAYA  19   m   40   99   Asia:FarE  1965  1982    Pr   Inc     Emp:mort   1 Current       Cigs  Nev cigs   1   9
          RR|SIG|

        1.80   ?


  ________________________________________________________________________________________________________________________
                                            International Evidence on Smoking and COPD, Phase 3, Analysis run on 28-SEP-10

                                                    Table 3 - E - 2 -

                               IESCOPD - Meta-analysis of amount smoked : key value (1) 20
                          Any Emphysema, cigarettes (or any product if cigarettes not available)


This analysis is restricted to results for:
1) Eligible study on database
2) Outcome Emphysema
3) Current or ever smoking
4) Categorical dose-response data for amount smoked
5) vs never smoking base
6) Key value (scheme 1) = 20
7) Results complete enough for use in meta-analysis

Within each study, results are then selected (in the following order of preference, within each sex) for:
8) SMKSTA  : current, ever
9) UNEXP   : never any, never cigarettes
10) PROD    : cigarettes, cigarettes only, any product
11) For overlapping studies: principal rather than subsidiary studies
and then for single sex results (m, f) in preference to results for both sexes combined (b).

Results adjusted for the most potential confounders are then chosen in Sections -1 to -3
and results adjusted for the least confounders in Sections -4 to -6. (Those least-adjusted results which
actually differ from the most-adjusted are marked 'x' in column X in Section -4)

Section -7 shows excluded studies, together with the stage (as above) at which no qualifying
results were found.

Section -8 lists the potentially overlapping studies which have been included (1=principal, 2=subsidiary),
and any results which would have been included in preference except that they had data not complete enough
for use in meta-analysis. It also lists their significance (yes/no), if known.


  ________________________________________________________________________________________________________________________
                                            International Evidence on Smoking and COPD, Phase 3, Analysis run on 28-SEP-10

                                                   Table 3 - E - 2 - 1

                               IESCOPD - Meta-analysis of amount smoked : key value (1) 20
                          Any Emphysema, cigarettes (or any product if cigarettes not available)
                                                      Most-adjusted


     REF|NRR|SEX|AGEL|AGEH|     REGION|BEGYR|PUBYR|STTYP|ONSET|      DISEAS|ADJ|SMOKSTA|   PRODUCT|    UNEXP|LOW| HI|

    BEST  22   m   30   97   Am:Canada  1955  1967    Pr   Inc     Emp:mort   1 Current  Cigs only   Nev any  10  20
  DONTA2  16   m   25   84 Eu:SE/Balkn  1960  1984    Pr   Inc Emp:diagnosd   0 Current       Cigs  Nev cigs  20  29
  HUHTI1  72   m   40   64    Eu:Scand  1961  1965    CS  Prev    Emp:other   0 Current       Cigs   Nev any  15  24
   KAHN2  49   m   31   84      Am:USA  1954  1966    Pr   Inc     Emp:mort   1 Current       Cigs   Nev any  10  20
   WEISS  20   m   50   69      Am:USA  1961  1963    CS  Prev  Emp:viscomp   0 Current  Cigs only   Nev any  10  20
     WEN  20   m   35   99   Asia:FarE  1982  2004    Pr   Inc     Emp:mort   1 Current       Cigs  Nev cigs  11  20


  ________________________________________________________________________________________________________________________
                                            International Evidence on Smoking and COPD, Phase 3, Analysis run on 28-SEP-10

                                                   Table 3 - E - 2 - 2

                               IESCOPD - Meta-analysis of amount smoked : key value (1) 20
                          Any Emphysema, cigarettes (or any product if cigarettes not available)
                                                      Most-adjusted


                        Number Exposed  Non-exposed
 REF    NRR SEX ADJ     Case    Cont    Case    Cont      RR        95.00%CI
*BEST   22  m   1         21       -       3       -      6.12 (  1.83-  20.51)
*DONTA2 16  m   0          4      67       4     127      1.90 (  0.49-   7.34)
 HUHTI1 72  m   0         21     170       1     121     14.95 (  1.98- 112.63)
*KAHN2  49  m   1        128       -      18       -     11.94 (  7.29-  19.56)
 WEISS  20  m   0          9      41       1      35      7.68 (  0.93-  63.67)
*WEN    20  m   1          -       -      13       -      1.43 (  0.51-   4.00)
Partial Totals           183     278      40     283
*prospective study


 REF    NRR SEX ADJ             Ys       Ws       Qs       Ps
*BEST   22  m   1              1.81     2.63     0.06       0.00
*DONTA2 16  m   0              0.64     2.10     3.66       0.35
 HUHTI1 72  m   0              2.70     0.94     0.52       0.01
*KAHN2  49  m   1              2.48    15.77     4.26       0.00
 WEISS  20  m   0              2.04     0.86     0.01       0.06
*WEN    20  m   1              0.36     3.62     9.30       0.50

                       N        6
                      NS        6


                      Wt    25.92
                 Het Chi    17.80
                 Het  df        5
                 Het  P        **
               Fixed  RR     7.10
                     RRl     4.83
                     RRu    10.44
                      P       +++
              Random  RR     5.07
                     RRl     2.04
                     RRu    12.61
                      P       +++
               Asymm  P      N.S.


  ________________________________________________________________________________________________________________________
                                            International Evidence on Smoking and COPD, Phase 3, Analysis run on 28-SEP-10

                                                   Table 3 - E - 2 - 3

                               IESCOPD - Meta-analysis of amount smoked : key value (1) 20
                          Any Emphysema, cigarettes (or any product if cigarettes not available)
                                                      Most-adjusted


                       N        6
                      NS        6


                      Wt    25.92
                 Het Chi    17.80
                 Het  df        5
                 Het  P        **
               Fixed  RR     7.10
                     RRl     4.83
                     RRu    10.44
                      P       +++
              Random  RR     5.07
                     RRl     2.04
                     RRu    12.61
                      P       +++
               Asymm  P      N.S.

                                   Sex
                             both      male    female     Total


                       N                  6                   6
                      NS                  6                   6


                      Wt              25.92               25.92
                 Het Chi              17.80               17.80
                 Het  df                  5                   5
                 Het  P                  **                  **
               Fixed  RR               7.10                7.10
                     RRl               4.83                4.83
                     RRu              10.44               10.44
                      P                 +++                 +++
              Random  RR               5.07                5.07
                     RRl               2.04                2.04
                     RRu              12.61               12.61
                      P                 +++                 +++
             Between Chi
             Between  df
             Between  P                                    N.S.
             Btwn(F)  P                                    N.S.

                                        Continent
                            NAmer    Europe      Asia  oth/mult     Total


                       N        3         2         1                   6
                      NS        3         2         1                   6


                      Wt    19.26      3.04      3.62               25.92
                 Het Chi     1.11      2.77      0.00               17.80
                 Het  df        2         1         0                   5
                 Het  P      N.S.       (*)      N.S.                  **
               Fixed  RR    10.69      3.60      1.43                7.10
                     RRl     6.84      1.17      0.51                4.83
                     RRu    16.70     11.07      4.00               10.44
                      P       +++         +      N.S.                 +++
              Random  RR    10.69      4.62      1.43                5.07
                     RRl     6.84      0.62      0.51                2.04
                     RRu    16.70     34.30      4.00               12.61
                      P       +++      N.S.      N.S.                 +++
             Between Chi                                            13.93
             Between  df                                                2
             Between  P                                               ***
             Btwn(F)  P                                              N.S.


  ________________________________________________________________________________________________________________________
                                            International Evidence on Smoking and COPD, Phase 3, Analysis run on 28-SEP-10

                                                   Table 3 - E - 2 - 3

                               IESCOPD - Meta-analysis of amount smoked : key value (1) 20
                          Any Emphysema, cigarettes (or any product if cigarettes not available)
                                                      Most-adjusted
                        National cigarette tobacco type (excluding mixed/unkown)
                          blended  virginia     Total


                       N        4         1         5
                      NS        4         1         5


                      Wt    19.67      2.63     22.30
                 Het Chi     6.49      0.00      6.99
                 Het  df        3         0         4
                 Het  P       (*)      N.S.      N.S.
               Fixed  RR     9.73      6.12      9.21
                     RRl     6.26      1.83      6.08
                     RRu    15.14     20.49     13.95
                      P       +++        ++       +++
              Random  RR     7.25      6.12      7.25
                     RRl     2.70      1.83      3.48
                     RRu    19.46     20.49     15.09
                      P       +++        ++       +++
             Between Chi                         0.50
             Between  df                            1
             Between  P                          N.S.
             Btwn(F)  P                          N.S.

                                        Start year of study
                            <1970   1970-79   1980-89   1990-99     2000+   unknown     Total


                       N        5                   1                                       6
                      NS        5                   1                                       6


                      Wt    22.30                3.62                                   25.92
                 Het Chi     6.99                0.00                                   17.80
                 Het  df        4                   0                                       5
                 Het  P      N.S.                N.S.                                      **
               Fixed  RR     9.21                1.43                                    7.10
                     RRl     6.08                0.51                                    4.83
                     RRu    13.95                4.00                                   10.44
                      P       +++                N.S.                                     +++
              Random  RR     7.25                1.43                                    5.07
                     RRl     3.48                0.51                                    2.04
                     RRu    15.09                4.00                                   12.61
                      P       +++                N.S.                                     +++
             Between Chi                                                                10.81
             Between  df                                                                    1
             Between  P                                                                    **
             Btwn(F)  P                                                                   (*)

                                Publication year
                            <1980   1980-89   1990-99     2000+     Total


                       N        4         1                   1         6
                      NS        4         1                   1         6


                      Wt    20.21      2.10                3.62     25.92
                 Het Chi     1.21      0.00                0.00     17.80
                 Het  df        3         0                   0         5
                 Het  P      N.S.      N.S.                N.S.        **
               Fixed  RR    10.85      1.90                1.43      7.10
                     RRl     7.02      0.49                0.51      4.83
                     RRu    16.79      7.34                4.00     10.44
                      P       +++      N.S.                N.S.       +++
              Random  RR    10.85      1.90                1.43      5.07
                     RRl     7.02      0.49                0.51      2.04
                     RRu    16.79      7.34                4.00     12.61
                      P       +++      N.S.                N.S.       +++
             Between Chi                                            16.60
             Between  df                                                2
             Between  P                                               ***
             Btwn(F)  P                                                 *
  ________________________________________________________________________________________________________________________
                                            International Evidence on Smoking and COPD, Phase 3, Analysis run on 28-SEP-10

                                                   Table 3 - E - 2 - 3

                               IESCOPD - Meta-analysis of amount smoked : key value (1) 20
                          Any Emphysema, cigarettes (or any product if cigarettes not available)
                                                      Most-adjusted
                               Study type
                               CC        Pr        CS     Total


                       N                  4         2         6
                      NS                  4         2         6


                      Wt              24.12      1.80     25.92
                 Het Chi              17.25      0.20     17.80
                 Het  df                  3         1         5
                 Het  P                 ***      N.S.        **
               Fixed  RR               6.88     10.88      7.10
                     RRl               4.62      2.53      4.83
                     RRu              10.25     46.88     10.44
                      P                 +++        ++       +++
              Random  RR               4.01     10.88      5.07
                     RRl               1.26      2.53      2.04
                     RRu              12.75     46.88     12.61
                      P                   +        ++       +++
             Between Chi                                   0.35
             Between  df                                      1
             Between  P                                    N.S.
             Btwn(F)  P                                    N.S.

                                    Lowest age in RR
                        <25/unlim     25-39       40+   unknown     Total


                       N                  4         2                   6
                      NS                  4         2                   6


                      Wt              24.12      1.80               25.92
                 Het Chi              17.25      0.20               17.80
                 Het  df                  3         1                   5
                 Het  P                 ***      N.S.                  **
               Fixed  RR               6.88     10.88                7.10
                     RRl               4.62      2.53                4.83
                     RRu              10.25     46.88               10.44
                      P                 +++        ++                 +++
              Random  RR               4.01     10.88                5.07
                     RRl               1.26      2.53                2.04
                     RRu              12.75     46.88               12.61
                      P                   +        ++                 +++
             Between Chi                                             0.35
             Between  df                                                1
             Between  P                                              N.S.
             Btwn(F)  P                                              N.S.

                                         Highest age in RR
                              <65     65-74     75-84 85+/unlim   unknown     Total


                       N        1         1         2         2                   6
                      NS        1         1         2         2                   6


                      Wt     0.94      0.86     17.87      6.25               25.92
                 Het Chi     0.00      0.00      6.27      3.22               17.80
                 Het  df        0         0         1         1                   5
                 Het  P      N.S.      N.S.         *       (*)                  **
               Fixed  RR    14.95      7.68      9.62      2.64                7.10
                     RRl     1.98      0.93      6.05      1.20                4.83
                     RRu   112.63     63.67     15.30      5.77               10.44
                      P        ++       (+)       +++         +                 +++
              Random  RR    14.95      7.68      5.32      2.85                5.07
                     RRl     1.98      0.93      0.89      0.69                2.04
                     RRu   112.63     63.67     31.89     11.85               12.61
                      P        ++       (+)       (+)      N.S.                 +++
             Between Chi                                                       8.32
             Between  df                                                          3
             Between  P                                                           *
             Btwn(F)  P                                                        N.S.
  ________________________________________________________________________________________________________________________
                                            International Evidence on Smoking and COPD, Phase 3, Analysis run on 28-SEP-10

                                                   Table 3 - E - 2 - 3

                               IESCOPD - Meta-analysis of amount smoked : key value (1) 20
                          Any Emphysema, cigarettes (or any product if cigarettes not available)
                                                      Most-adjusted
                           Study weakness
                              Yes        No     Total


                       N        1         5         6
                      NS        1         5         6


                      Wt     2.10     23.83     25.92
                 Het Chi     0.00     13.83     17.80
                 Het  df        0         4         5
                 Het  P      N.S.        **        **
               Fixed  RR     1.90      7.98      7.10
                     RRl     0.49      5.34      4.83
                     RRu     7.34     11.92     10.44
                      P      N.S.       +++       +++
              Random  RR     1.90      6.15      5.07
                     RRl     0.49      2.31      2.04
                     RRu     7.34     16.33     12.61
                      P      N.S.       +++       +++
             Between Chi                         3.98
             Between  df                            1
             Between  P                             *
             Btwn(F)  P                          N.S.

                           Type of outcome
                        prevalnce mortality incidence     Total


                       N        2         3         1         6
                      NS        2         3         1         6


                      Wt     1.80     22.03      2.10     25.92
                 Het Chi     0.20     13.44      0.00     17.80
                 Het  df        1         2         0         5
                 Het  P      N.S.        **      N.S.        **
               Fixed  RR    10.88      7.78      1.90      7.10
                     RRl     2.53      5.12      0.49      4.83
                     RRu    46.88     11.81      7.34     10.44
                      P        ++       +++      N.S.       +++
              Random  RR    10.88      4.93      1.90      5.07
                     RRl     2.53      1.30      0.49      2.04
                     RRu    46.88     18.74      7.34     12.61
                      P        ++         +      N.S.       +++
             Between Chi                                   4.17
             Between  df                                      2
             Between  P                                    N.S.
             Btwn(F)  P                                    N.S.

                        Emp subtype
                             mort     other     Total


                       N        3         3         6
                      NS        3         3         6


                      Wt    22.03      3.90     25.92
                 Het Chi    13.44      3.16     17.80
                 Het  df        2         2         5
                 Het  P        **      N.S.        **
               Fixed  RR     7.78      4.25      7.10
                     RRl     5.12      1.58      4.83
                     RRu    11.81     11.47     10.44
                      P       +++        ++       +++
              Random  RR     4.93      4.92      5.07
                     RRl     1.30      1.34      2.04
                     RRu    18.74     18.02     12.61
                      P         +         +       +++
             Between Chi                         1.21
             Between  df                            1
             Between  P                          N.S.
             Btwn(F)  P                          N.S.
  ________________________________________________________________________________________________________________________
                                            International Evidence on Smoking and COPD, Phase 3, Analysis run on 28-SEP-10

                                                   Table 3 - E - 2 - 3

                               IESCOPD - Meta-analysis of amount smoked : key value (1) 20
                          Any Emphysema, cigarettes (or any product if cigarettes not available)
                                                      Most-adjusted
                        Asthma analysis type (Emphysema)
                        inc-irres  excl-all     Total


                       N        5         1         6
                      NS        5         1         6


                      Wt    23.83      2.10     25.92
                 Het Chi    13.83      0.00     17.80
                 Het  df        4         0         5
                 Het  P        **      N.S.        **
               Fixed  RR     7.98      1.90      7.10
                     RRl     5.34      0.49      4.83
                     RRu    11.92      7.34     10.44
                      P       +++      N.S.       +++
              Random  RR     6.15      1.90      5.07
                     RRl     2.31      0.49      2.04
                     RRu    16.33      7.34     12.61
                      P       +++      N.S.       +++
             Between Chi                         3.98
             Between  df                            1
             Between  P                             *
             Btwn(F)  P                          N.S.

                                Number of emphysema cases
                             1-50    51-100   101-200      201+     Total


                       N        3         2                   1         6
                      NS        3         2                   1         6


                      Wt     6.58      3.57               15.77     25.92
                 Het Chi     1.97      0.55                0.00     17.80
                 Het  df        2         1                   0         5
                 Het  P      N.S.      N.S.                N.S.        **
               Fixed  RR     1.95      7.74               11.94      7.10
                     RRl     0.91      2.75                7.29      4.83
                     RRu     4.18     21.84               19.56     10.44
                      P       (+)       +++                 +++       +++
              Random  RR     1.95      7.74               11.94      5.07
                     RRl     0.91      2.75                7.29      2.04
                     RRu     4.18     21.84               19.56     12.61
                      P       (+)       +++                 +++       +++
             Between Chi                                            15.28
             Between  df                                                2
             Between  P                                               ***
             Btwn(F)  P                                               (*)

                        Number of emphysma cases (excluding unknown)


                       N        3         2                   1         6
                      NS        3         2                   1         6


                      Wt     6.58      3.57               15.77     25.92
                 Het Chi     1.97      0.55                0.00     17.80
                 Het  df        2         1                   0         5
                 Het  P      N.S.      N.S.                N.S.        **
               Fixed  RR     1.95      7.74               11.94      7.10
                     RRl     0.91      2.75                7.29      4.83
                     RRu     4.18     21.84               19.56     10.44
                      P       (+)       +++                 +++       +++
              Random  RR     1.95      7.74               11.94      5.07
                     RRl     0.91      2.75                7.29      2.04
                     RRu     4.18     21.84               19.56     12.61
                      P       (+)       +++                 +++       +++
             Between Chi                                            15.28
             Between  df                                                2
             Between  P                                               ***
             Btwn(F)  P                                               (*)

  ________________________________________________________________________________________________________________________
                                            International Evidence on Smoking and COPD, Phase 3, Analysis run on 28-SEP-10

                                                   Table 3 - E - 2 - 3

                               IESCOPD - Meta-analysis of amount smoked : key value (1) 20
                          Any Emphysema, cigarettes (or any product if cigarettes not available)
                                                      Most-adjusted
                            Analysis type
                         prevlnce     onset     Total


                       N        2         4         6
                      NS        2         4         6


                      Wt     1.80     24.12     25.92
                 Het Chi     0.20     17.25     17.80
                 Het  df        1         3         5
                 Het  P      N.S.       ***        **
               Fixed  RR    10.88      6.88      7.10
                     RRl     2.53      4.62      4.83
                     RRu    46.88     10.25     10.44
                      P        ++       +++       +++
              Random  RR    10.88      4.01      5.07
                     RRl     2.53      1.26      2.04
                     RRu    46.88     12.75     12.61
                      P        ++         +       +++
             Between Chi                         0.35
             Between  df                            1
             Between  P                          N.S.
             Btwn(F)  P                          N.S.

                             Smoking product
                              any      cigs  cigsonly     Total


                       N                  4         2         6
                      NS                  4         2         6


                      Wt              22.43      3.49     25.92
                 Het Chi              17.73      0.03     17.80
                 Het  df                  3         1         5
                 Het  P                 ***      N.S.        **
               Fixed  RR               7.20      6.47      7.10
                     RRl               4.76      2.27      4.83
                     RRu              10.90     18.48     10.44
                      P                 +++       +++       +++
              Random  RR               4.52      6.47      5.07
                     RRl               1.21      2.27      2.04
                     RRu              16.85     18.48     12.61
                      P                   +       +++       +++
             Between Chi                                   0.03
             Between  df                                      1
             Between  P                                    N.S.
             Btwn(F)  P                                    N.S.

                                     Unexposed group
                          nev any   nev cig  nev+ any  nev+ cig     Total


                       N        4         2                             6
                      NS        4         2                             6


                      Wt    20.21      5.72                         25.92
                 Het Chi     1.21      0.11                         17.80
                 Het  df        3         1                             5
                 Het  P      N.S.      N.S.                            **
               Fixed  RR    10.85      1.59                          7.10
                     RRl     7.02      0.70                          4.83
                     RRu    16.79      3.60                         10.44
                      P       +++      N.S.                           +++
              Random  RR    10.85      1.59                          5.07
                     RRl     7.02      0.70                          2.04
                     RRu    16.79      3.60                         12.61
                      P       +++      N.S.                           +++
             Between Chi                                            16.49
             Between  df                                                1
             Between  P                                               ***
             Btwn(F)  P                                                **
  ________________________________________________________________________________________________________________________
                                            International Evidence on Smoking and COPD, Phase 3, Analysis run on 28-SEP-10

                                                   Table 3 - E - 2 - 3

                               IESCOPD - Meta-analysis of amount smoked : key value (1) 20
                          Any Emphysema, cigarettes (or any product if cigarettes not available)
                                                      Most-adjusted
                        Unexposed group (combining nev+ with main levels)
                          nev any   nev cig     Total


                       N        4         2         6
                      NS        4         2         6


                      Wt    20.21      5.72     25.92
                 Het Chi     1.21      0.11     17.80
                 Het  df        3         1         5
                 Het  P      N.S.      N.S.        **
               Fixed  RR    10.85      1.59      7.10
                     RRl     7.02      0.70      4.83
                     RRu    16.79      3.60     10.44
                      P       +++      N.S.       +++
              Random  RR    10.85      1.59      5.07
                     RRl     7.02      0.70      2.04
                     RRu    16.79      3.60     12.61
                      P       +++      N.S.       +++
             Between Chi                        16.49
             Between  df                            1
             Between  P                           ***
             Btwn(F)  P                            **

                        Number of adjustment variables
                                0         1        2+     Total


                       N        3         3                   6
                      NS        3         3                   6


                      Wt     3.90     22.03               25.92
                 Het Chi     3.16     13.44               17.80
                 Het  df        2         2                   5
                 Het  P      N.S.        **                  **
               Fixed  RR     4.25      7.78                7.10
                     RRl     1.58      5.12                4.83
                     RRu    11.47     11.81               10.44
                      P        ++       +++                 +++
              Random  RR     4.92      4.93                5.07
                     RRl     1.34      1.30                2.04
                     RRu    18.02     18.74               12.61
                      P         +         +                 +++
             Between Chi                                   1.21
             Between  df                                      1
             Between  P                                    N.S.
             Btwn(F)  P                                    N.S.


  ________________________________________________________________________________________________________________________
                                            International Evidence on Smoking and COPD, Phase 3, Analysis run on 28-SEP-10

                                                   Table 3 - E - 2 - 4

                               IESCOPD - Meta-analysis of amount smoked : key value (1) 20
                          Any Emphysema, cigarettes (or any product if cigarettes not available)
                                                      Least-adjusted


     REF|NRR|X|SEX|AGEL|AGEH|     REGION|BEGYR|PUBYR|STTYP|ONSET|      DISEAS|ADJ|SMOKSTA|   PRODUCT|    UNEXP|LOW| HI|

    BEST  22     m   30   97   Am:Canada  1955  1967    Pr   Inc     Emp:mort   1 Current  Cigs only   Nev any  10  20
  DONTA2  16     m   25   84 Eu:SE/Balkn  1960  1984    Pr   Inc Emp:diagnosd   0 Current       Cigs  Nev cigs  20  29
  HUHTI1  72     m   40   64    Eu:Scand  1961  1965    CS  Prev    Emp:other   0 Current       Cigs   Nev any  15  24
   KAHN2  49     m   31   84      Am:USA  1954  1966    Pr   Inc     Emp:mort   1 Current       Cigs   Nev any  10  20
   WEISS  20     m   50   69      Am:USA  1961  1963    CS  Prev  Emp:viscomp   0 Current  Cigs only   Nev any  10  20
     WEN  20     m   35   99   Asia:FarE  1982  2004    Pr   Inc     Emp:mort   1 Current       Cigs  Nev cigs  11  20


  ________________________________________________________________________________________________________________________
                                            International Evidence on Smoking and COPD, Phase 3, Analysis run on 28-SEP-10

                                                   Table 3 - E - 2 - 5

                               IESCOPD - Meta-analysis of amount smoked : key value (1) 20
                          Any Emphysema, cigarettes (or any product if cigarettes not available)
                                                      Least-adjusted


                        Number Exposed  Non-exposed
 REF    NRR SEX ADJ     Case    Cont    Case    Cont      RR        95.00%CI
*BEST   22  m   1         21       -       3       -      6.12 (  1.83-  20.51)
*DONTA2 16  m   0          4      67       4     127      1.90 (  0.49-   7.34)
 HUHTI1 72  m   0         21     170       1     121     14.95 (  1.98- 112.63)
*KAHN2  49  m   1        128       -      18       -     11.94 (  7.29-  19.56)
 WEISS  20  m   0          9      41       1      35      7.68 (  0.93-  63.67)
*WEN    20  m   1          -       -      13       -      1.43 (  0.51-   4.00)
Partial Totals           183     278      40     283
*prospective study


 REF    NRR SEX ADJ             Ys       Ws       Qs       Ps
*BEST   22  m   1              1.81     2.63     0.06       0.00
*DONTA2 16  m   0              0.64     2.10     3.66       0.35
 HUHTI1 72  m   0              2.70     0.94     0.52       0.01
*KAHN2  49  m   1              2.48    15.77     4.26       0.00
 WEISS  20  m   0              2.04     0.86     0.01       0.06
*WEN    20  m   1              0.36     3.62     9.30       0.50

                       N        6
                      NS        6


                      Wt    25.92
                 Het Chi    17.80
                 Het  df        5
                 Het  P        **
               Fixed  RR     7.10
                     RRl     4.83
                     RRu    10.44
                      P       +++
              Random  RR     5.07
                     RRl     2.04
                     RRu    12.61
                      P       +++
               Asymm  P      N.S.


  ________________________________________________________________________________________________________________________
                                            International Evidence on Smoking and COPD, Phase 3, Analysis run on 28-SEP-10

                                                   Table 3 - E - 2 - 6

                               IESCOPD - Meta-analysis of amount smoked : key value (1) 20
                          Any Emphysema, cigarettes (or any product if cigarettes not available)
                                                      Least-adjusted


                       N        6
                      NS        6


                      Wt    25.92
                 Het Chi    17.80
                 Het  df        5
                 Het  P        **
               Fixed  RR     7.10
                     RRl     4.83
                     RRu    10.44
                      P       +++
              Random  RR     5.07
                     RRl     2.04
                     RRu    12.61
                      P       +++
               Asymm  P      N.S.

                                   Sex
                             both      male    female     Total


                       N                  6                   6
                      NS                  6                   6


                      Wt              25.92               25.92
                 Het Chi              17.80               17.80
                 Het  df                  5                   5
                 Het  P                  **                  **
               Fixed  RR               7.10                7.10
                     RRl               4.83                4.83
                     RRu              10.44               10.44
                      P                 +++                 +++
              Random  RR               5.07                5.07
                     RRl               2.04                2.04
                     RRu              12.61               12.61
                      P                 +++                 +++
             Between Chi
             Between  df
             Between  P                                    N.S.
             Btwn(F)  P                                    N.S.

                                        Continent
                            NAmer    Europe      Asia  oth/mult     Total


                       N        3         2         1                   6
                      NS        3         2         1                   6


                      Wt    19.26      3.04      3.62               25.92
                 Het Chi     1.11      2.77      0.00               17.80
                 Het  df        2         1         0                   5
                 Het  P      N.S.       (*)      N.S.                  **
               Fixed  RR    10.69      3.60      1.43                7.10
                     RRl     6.84      1.17      0.51                4.83
                     RRu    16.70     11.07      4.00               10.44
                      P       +++         +      N.S.                 +++
              Random  RR    10.69      4.62      1.43                5.07
                     RRl     6.84      0.62      0.51                2.04
                     RRu    16.70     34.30      4.00               12.61
                      P       +++      N.S.      N.S.                 +++
             Between Chi                                            13.93
             Between  df                                                2
             Between  P                                               ***
             Btwn(F)  P                                              N.S.


  ________________________________________________________________________________________________________________________
                                            International Evidence on Smoking and COPD, Phase 3, Analysis run on 28-SEP-10

                                                   Table 3 - E - 2 - 6

                               IESCOPD - Meta-analysis of amount smoked : key value (1) 20
                          Any Emphysema, cigarettes (or any product if cigarettes not available)
                                                      Least-adjusted
                               Study type
                               CC        Pr        CS     Total


                       N                  4         2         6
                      NS                  4         2         6


                      Wt              24.12      1.80     25.92
                 Het Chi              17.25      0.20     17.80
                 Het  df                  3         1         5
                 Het  P                 ***      N.S.        **
               Fixed  RR               6.88     10.88      7.10
                     RRl               4.62      2.53      4.83
                     RRu              10.25     46.88     10.44
                      P                 +++        ++       +++
              Random  RR               4.01     10.88      5.07
                     RRl               1.26      2.53      2.04
                     RRu              12.75     46.88     12.61
                      P                   +        ++       +++
             Between Chi                                   0.35
             Between  df                                      1
             Between  P                                    N.S.
             Btwn(F)  P                                    N.S.

                        Emp subtype
                             mort     other     Total


                       N        3         3         6
                      NS        3         3         6


                      Wt    22.03      3.90     25.92
                 Het Chi    13.44      3.16     17.80
                 Het  df        2         2         5
                 Het  P        **      N.S.        **
               Fixed  RR     7.78      4.25      7.10
                     RRl     5.12      1.58      4.83
                     RRu    11.81     11.47     10.44
                      P       +++        ++       +++
              Random  RR     4.93      4.92      5.07
                     RRl     1.30      1.34      2.04
                     RRu    18.74     18.02     12.61
                      P         +         +       +++
             Between Chi                         1.21
             Between  df                            1
             Between  P                          N.S.
             Btwn(F)  P                          N.S.

                             Smoking product
                              any      cigs  cigsonly     Total


                       N                  4         2         6
                      NS                  4         2         6


                      Wt              22.43      3.49     25.92
                 Het Chi              17.73      0.03     17.80
                 Het  df                  3         1         5
                 Het  P                 ***      N.S.        **
               Fixed  RR               7.20      6.47      7.10
                     RRl               4.76      2.27      4.83
                     RRu              10.90     18.48     10.44
                      P                 +++       +++       +++
              Random  RR               4.52      6.47      5.07
                     RRl               1.21      2.27      2.04
                     RRu              16.85     18.48     12.61
                      P                   +       +++       +++
             Between Chi                                   0.03
             Between  df                                      1
             Between  P                                    N.S.
             Btwn(F)  P                                    N.S.
  ________________________________________________________________________________________________________________________
                                            International Evidence on Smoking and COPD, Phase 3, Analysis run on 28-SEP-10

                                                   Table 3 - E - 2 - 6

                               IESCOPD - Meta-analysis of amount smoked : key value (1) 20
                          Any Emphysema, cigarettes (or any product if cigarettes not available)
                                                      Least-adjusted
                                     Unexposed group
                          nev any   nev cig  nev+ any  nev+ cig     Total


                       N        4         2                             6
                      NS        4         2                             6


                      Wt    20.21      5.72                         25.92
                 Het Chi     1.21      0.11                         17.80
                 Het  df        3         1                             5
                 Het  P      N.S.      N.S.                            **
               Fixed  RR    10.85      1.59                          7.10
                     RRl     7.02      0.70                          4.83
                     RRu    16.79      3.60                         10.44
                      P       +++      N.S.                           +++
              Random  RR    10.85      1.59                          5.07
                     RRl     7.02      0.70                          2.04
                     RRu    16.79      3.60                         12.61
                      P       +++      N.S.                           +++
             Between Chi                                            16.49
             Between  df                                                1
             Between  P                                               ***
             Btwn(F)  P                                                **

                        Unexposed group (combining nev+ with main levels)
                          nev any   nev cig     Total


                       N        4         2         6
                      NS        4         2         6


                      Wt    20.21      5.72     25.92
                 Het Chi     1.21      0.11     17.80
                 Het  df        3         1         5
                 Het  P      N.S.      N.S.        **
               Fixed  RR    10.85      1.59      7.10
                     RRl     7.02      0.70      4.83
                     RRu    16.79      3.60     10.44
                      P       +++      N.S.       +++
              Random  RR    10.85      1.59      5.07
                     RRl     7.02      0.70      2.04
                     RRu    16.79      3.60     12.61
                      P       +++      N.S.       +++
             Between Chi                        16.49
             Between  df                            1
             Between  P                           ***
             Btwn(F)  P                            **


  ________________________________________________________________________________________________________________________
                                            International Evidence on Smoking and COPD, Phase 3, Analysis run on 28-SEP-10

                                                   Table 3 - E - 2 - 7

                               IESCOPD - Meta-analysis of amount smoked : key value (1) 20
                          Any Emphysema, cigarettes (or any product if cigarettes not available)
                                 Excluded studies (and stage at which they were excluded)


1       CLARK COTTON  MEYER REMYJA RUTGER SNYDER SOBRAX     SU TAKEMU  WANG4   WEIR WHICKE ZALACA
2      ALDERS ALESSA  AMIGO ANDER1 ANDER3   BANG  BECK1  BECK2 BEDNAR BJORNS BROGGE  BROWN CERVER CHAPMA  CHEN1  CHEN2
        CHEN3  CHENG CLEMEN COATES  COCCI COLLEG  DEAN1  DEAN2  DEANE DEJONG DEMARC DETORR DICKIN  DOLL1  DOLL2 DONTA1
       DOPICO EHRLIC EKBERG ENSTRO FERRI1 FERRI2 FERRI3  FIDAN FINKLE FLETCH FORAST FOXMAN FUKUCH GEIJER GODTFR GOLDBE
       HAENSZ HARIKK HARRIS HAWTHO  HAYES HEDMAN HIGGI2 HIGGI3 HIGGI4 HIGGI6 HOLLA2 HOLLNA  HOUSE HRUBEC HUCHON HUHTI2
       HUHTI3 ITABAS JACOBS JAENDI JENSEN JINDA2 JOHANN  JOSHI JOUSI1 KACHEL KARAKA KATANC   KATO KHOURY    KIM  KIRAZ
       KLAYTO KOJIMA KOTAN1 KOTAN2 KRZYZA  KUBIK KULLER    LAI   LAM1   LAM2   LAM3 LAMBER  LANGE LANGE2 LANGHA    LEE
         LIAW LINDBE LINDST   LIU1   LIU2 LUNDB1 LUNDB2  MADOR MAGNUS MANFRE MANNI1 MANNI2 MANNI3 MARAN1 MARAN2 MARCUS
       MATHES MELLST MENEZ1 MENEZ2 MENEZ3 MENEZ4 MENEZ5 MENEZ6  MEREN  MILNE MOLLER MONTNE MUELLE NEJJAR NIEPSU NIHLEN
       NILSSO OGILVI OSWAL1 OSWAL2 PANDEY   PEAT PELKON PEREZP   PETO  PRICE   REID RENWIC RICCIO RIMING SARGEA SAWICK
       SCHWAR SHAHAB  SHARP SHIMUR   SHIN SICHLE SOBRAD SPEIZE STERLI STJERN  STROM SUADIC  TAGER TAGER2   TANG   THUN
         TODD TROISI TRUPIN TSUSHI TVERDA URRUTI VESTBO VIEGI1 VIEGI2 VINEIS VOLLM1 VOLLM2 VONHER WAGEN2   WALD WATSON
          WIG WILHEL WILSO1 WOJTYN  WOODS  WOOLF   XIAO     XU YAMAGU   YUAN ZIELI1 ZIELI2 ZIETKO   ZOIA
3        KAHN
4      ANDER2 ENRIGH GULSVI HARDIE     HO HOZAWA LEBOWI MILLER   NAWA  OMORI  PRATT  RYDER  SILVA SUTINE VIKGRE  WANG2
5      WILSO2
6      AUERBA HAMMO2 LAVECC
7      HIRAYA


  ________________________________________________________________________________________________________________________
                                            International Evidence on Smoking and COPD, Phase 3, Analysis run on 28-SEP-10

                                                   Table 3 - E - 2 - 8

                               IESCOPD - Meta-analysis of amount smoked : key value (1) 20
                          Any Emphysema, cigarettes (or any product if cigarettes not available)
                                             Potentially overlapping studies


     REF| REFGP|PRINC|                     OVERLAP|

  DONTA2 JACOBS     2  JACOBS/DONTA1/DONTA2/PELKON
  HUHTI1 HUHTI1     1                HUHTI1/HUHTI2
     WEN    WEN     1                     WEN/LIAW
   KAHN2   KAHN     2                   KAHN/KAHN2

                                    Most-adjusted - insufficient data for meta-analysis
     REF|NRR|SEX|AGEL|AGEH|     REGION|BEGYR|PUBYR|STTYP|ONSET|      DISEAS|ADJ|SMOKSTA|   PRODUCT|    UNEXP|LOW| HI|

  HIRAYA  21   m   40   99   Asia:FarE  1965  1982    Pr   Inc     Emp:mort   1 Current       Cigs  Nev cigs  20  29
          RR|SIG|

        2.50   ?


  ________________________________________________________________________________________________________________________
                                            International Evidence on Smoking and COPD, Phase 3, Analysis run on 28-SEP-10

                                                    Table 3 - E - 3 -

                               IESCOPD - Meta-analysis of amount smoked : key value (1) 45
                          Any Emphysema, cigarettes (or any product if cigarettes not available)


This analysis is restricted to results for:
1) Eligible study on database
2) Outcome Emphysema
3) Current or ever smoking
4) Categorical dose-response data for amount smoked
5) vs never smoking base
6) Key value (scheme 1) = 45
7) Results complete enough for use in meta-analysis

Within each study, results are then selected (in the following order of preference, within each sex) for:
8) SMKSTA  : current, ever
9) UNEXP   : never any, never cigarettes
10) PROD    : cigarettes, cigarettes only, any product
11) For overlapping studies: principal rather than subsidiary studies
and then for single sex results (m, f) in preference to results for both sexes combined (b).

Results adjusted for the most potential confounders are then chosen in Sections -1 to -3
and results adjusted for the least confounders in Sections -4 to -6. (Those least-adjusted results which
actually differ from the most-adjusted are marked 'x' in column X in Section -4)

Section -7 shows excluded studies, together with the stage (as above) at which no qualifying
results were found.

Section -8 lists the potentially overlapping studies which have been included (1=principal, 2=subsidiary),
and any results which would have been included in preference except that they had data not complete enough
for use in meta-analysis. It also lists their significance (yes/no), if known.


  ________________________________________________________________________________________________________________________
                                            International Evidence on Smoking and COPD, Phase 3, Analysis run on 28-SEP-10

                                                   Table 3 - E - 3 - 1

                               IESCOPD - Meta-analysis of amount smoked : key value (1) 45
                          Any Emphysema, cigarettes (or any product if cigarettes not available)
                                                      Most-adjusted


     REF|NRR|SEX|AGEL|AGEH|     REGION|BEGYR|PUBYR|STTYP|ONSET|      DISEAS|ADJ|SMOKSTA|   PRODUCT|    UNEXP|LOW| HI|

    BEST  23   m   30   97   Am:Canada  1955  1967    Pr   Inc     Emp:mort   1 Current  Cigs only   Nev any  21   +
  DONTA2  17   m   25   84 Eu:SE/Balkn  1960  1984    Pr   Inc Emp:diagnosd   0 Current       Cigs  Nev cigs  30   +
  HUHTI1  73   m   40   64    Eu:Scand  1961  1965    CS  Prev    Emp:other   0 Current       Cigs   Nev any  25   +
   KAHN2  51   m   31   84      Am:USA  1954  1966    Pr   Inc     Emp:mort   1 Current       Cigs   Nev any  40   +
   WEISS  21   m   50   69      Am:USA  1961  1963    CS  Prev  Emp:viscomp   0 Current  Cigs only   Nev any  21   +
     WEN  21   m   35   99   Asia:FarE  1982  2004    Pr   Inc     Emp:mort   1 Current       Cigs  Nev cigs  21   +


  ________________________________________________________________________________________________________________________
                                            International Evidence on Smoking and COPD, Phase 3, Analysis run on 28-SEP-10

                                                   Table 3 - E - 3 - 2

                               IESCOPD - Meta-analysis of amount smoked : key value (1) 45
                          Any Emphysema, cigarettes (or any product if cigarettes not available)
                                                      Most-adjusted


                        Number Exposed  Non-exposed
 REF    NRR SEX ADJ     Case    Cont    Case    Cont      RR        95.00%CI
*BEST   23  m   1          7       -       3       -      6.93 (  1.79-  26.79)
*DONTA2 17  m   0          1      14       4     127      2.27 (  0.27-  18.91)
 HUHTI1 73  m   0         12      73       1     121     19.89 (  2.53- 156.15)
*KAHN2  51  m   1         21       -      18       -     20.86 ( 11.11-  39.15)
 WEISS  21  m   0          4      40       1      35      3.50 (  0.37-  32.80)
*WEN    21  m   1          -       -      13       -      1.44 (  0.17-  11.90)
Partial Totals            45     127      40     283
*prospective study


 REF    NRR SEX ADJ             Ys       Ws       Qs       Ps
*BEST   23  m   1              1.94     2.10     0.71       0.01
*DONTA2 17  m   0              0.82     0.85     2.46       0.45
 HUHTI1 73  m   0              2.99     0.90     0.20       0.00
*KAHN2  51  m   1              3.04     9.69     2.63       0.00
 WEISS  21  m   0              1.25     0.77     1.23       0.27
*WEN    21  m   1              0.36     0.85     3.94       0.74

                       N        6
                      NS        6


                      Wt    15.16
                 Het Chi    11.17
                 Het  df        5
                 Het  P         *
               Fixed  RR    12.39
                     RRl     7.49
                     RRu    20.50
                      P       +++
              Random  RR     7.19
                     RRl     2.74
                     RRu    18.87
                      P       +++
               Asymm  P         *


  ________________________________________________________________________________________________________________________
                                            International Evidence on Smoking and COPD, Phase 3, Analysis run on 28-SEP-10

                                                   Table 3 - E - 3 - 3

                               IESCOPD - Meta-analysis of amount smoked : key value (1) 45
                          Any Emphysema, cigarettes (or any product if cigarettes not available)
                                                      Most-adjusted


                       N        6
                      NS        6


                      Wt    15.16
                 Het Chi    11.17
                 Het  df        5
                 Het  P         *
               Fixed  RR    12.39
                     RRl     7.49
                     RRu    20.50
                      P       +++
              Random  RR     7.19
                     RRl     2.74
                     RRu    18.87
                      P       +++
               Asymm  P         *

                                   Sex
                             both      male    female     Total


                       N                  6                   6
                      NS                  6                   6


                      Wt              15.16               15.16
                 Het Chi              11.17               11.17
                 Het  df                  5                   5
                 Het  P                   *                   *
               Fixed  RR              12.39               12.39
                     RRl               7.49                7.49
                     RRu              20.50               20.50
                      P                 +++                 +++
              Random  RR               7.19                7.19
                     RRl               2.74                2.74
                     RRu              18.87               18.87
                      P                 +++                 +++
             Between Chi
             Between  df
             Between  P                                    N.S.
             Btwn(F)  P                                    N.S.

                                        Continent
                            NAmer    Europe      Asia  oth/mult     Total


                       N        3         2         1                   6
                      NS        3         2         1                   6


                      Wt    12.55      1.76      0.85               15.16
                 Het Chi     3.91      2.07      0.00               11.17
                 Het  df        2         1         0                   5
                 Het  P      N.S.      N.S.      N.S.                   *
               Fixed  RR    15.56      6.93      1.44               12.39
                     RRl     8.95      1.58      0.17                7.49
                     RRu    27.05     30.37     12.05               20.50
                      P       +++         +      N.S.                 +++
              Random  RR    11.18      6.82      1.44                7.19
                     RRl     4.02      0.81      0.17                2.74
                     RRu    31.11     57.24     12.05               18.87
                      P       +++       (+)      N.S.                 +++
             Between Chi                                             5.19
             Between  df                                                2
             Between  P                                               (*)
             Btwn(F)  P                                              N.S.


  ________________________________________________________________________________________________________________________
                                            International Evidence on Smoking and COPD, Phase 3, Analysis run on 28-SEP-10

                                                   Table 3 - E - 3 - 3

                               IESCOPD - Meta-analysis of amount smoked : key value (1) 45
                          Any Emphysema, cigarettes (or any product if cigarettes not available)
                                                      Most-adjusted
                        National cigarette tobacco type (excluding mixed/unkown)
                          blended  virginia     Total


                       N        4         1         5
                      NS        4         1         5


                      Wt    12.21      2.10     14.31
                 Het Chi     5.76      0.00      6.99
                 Het  df        3         0         4
                 Het  P      N.S.      N.S.      N.S.
               Fixed  RR    15.91      6.93     14.08
                     RRl     9.08      1.79      8.39
                     RRu    27.88     26.81     23.65
                      P       +++        ++       +++
              Random  RR    10.10      6.93      9.84
                     RRl     3.26      1.79      4.08
                     RRu    31.29     26.81     23.75
                      P       +++        ++       +++
             Between Chi                         1.24
             Between  df                            1
             Between  P                          N.S.
             Btwn(F)  P                          N.S.

                                        Start year of study
                            <1970   1970-79   1980-89   1990-99     2000+   unknown     Total


                       N        5                   1                                       6
                      NS        5                   1                                       6


                      Wt    14.31                0.85                                   15.16
                 Het Chi     6.99                0.00                                   11.17
                 Het  df        4                   0                                       5
                 Het  P      N.S.                N.S.                                       *
               Fixed  RR    14.08                1.44                                   12.39
                     RRl     8.39                0.17                                    7.49
                     RRu    23.65               12.05                                   20.50
                      P       +++                N.S.                                     +++
              Random  RR     9.84                1.44                                    7.19
                     RRl     4.08                0.17                                    2.74
                     RRu    23.75               12.05                                   18.87
                      P       +++                N.S.                                     +++
             Between Chi                                                                 4.18
             Between  df                                                                    1
             Between  P                                                                     *
             Btwn(F)  P                                                                  N.S.

                                Publication year
                            <1980   1980-89   1990-99     2000+     Total


                       N        4         1                   1         6
                      NS        4         1                   1         6


                      Wt    13.46      0.85                0.85     15.16
                 Het Chi     3.96      0.00                0.00     11.17
                 Het  df        3         0                   0         5
                 Het  P      N.S.      N.S.                N.S.         *
               Fixed  RR    15.82      2.27                1.44     12.39
                     RRl     9.27      0.27                0.17      7.49
                     RRu    26.99     18.91               12.05     20.50
                      P       +++      N.S.                N.S.       +++
              Random  RR    13.49      2.27                1.44      7.19
                     RRl     6.38      0.27                0.17      2.74
                     RRu    28.50     18.91               12.05     18.87
                      P       +++      N.S.                N.S.       +++
             Between Chi                                             7.21
             Between  df                                                2
             Between  P                                                 *
             Btwn(F)  P                                              N.S.
  ________________________________________________________________________________________________________________________
                                            International Evidence on Smoking and COPD, Phase 3, Analysis run on 28-SEP-10

                                                   Table 3 - E - 3 - 3

                               IESCOPD - Meta-analysis of amount smoked : key value (1) 45
                          Any Emphysema, cigarettes (or any product if cigarettes not available)
                                                      Most-adjusted
                               Study type
                               CC        Pr        CS     Total


                       N                  4         2         6
                      NS                  4         2         6


                      Wt              13.49      1.67     15.16
                 Het Chi               9.72      1.25     11.17
                 Het  df                  3         1         5
                 Het  P                   *      N.S.         *
               Fixed  RR              12.90      8.96     12.39
                     RRl               7.57      1.97      7.49
                     RRu              22.00     40.81     20.50
                      P                 +++        ++       +++
              Random  RR               6.18      8.83      7.19
                     RRl               1.72      1.62      2.74
                     RRu              22.25     48.31     18.87
                      P                  ++         +       +++
             Between Chi                                   0.20
             Between  df                                      1
             Between  P                                    N.S.
             Btwn(F)  P                                    N.S.

                                    Lowest age in RR
                        <25/unlim     25-39       40+   unknown     Total


                       N                  4         2                   6
                      NS                  4         2                   6


                      Wt              13.49      1.67               15.16
                 Het Chi               9.72      1.25               11.17
                 Het  df                  3         1                   5
                 Het  P                   *      N.S.                   *
               Fixed  RR              12.90      8.96               12.39
                     RRl               7.57      1.97                7.49
                     RRu              22.00     40.81               20.50
                      P                 +++        ++                 +++
              Random  RR               6.18      8.83                7.19
                     RRl               1.72      1.62                2.74
                     RRu              22.25     48.31               18.87
                      P                  ++         +                 +++
             Between Chi                                             0.20
             Between  df                                                1
             Between  P                                              N.S.
             Btwn(F)  P                                              N.S.

                                         Highest age in RR
                              <65     65-74     75-84 85+/unlim   unknown     Total


                       N        1         1         2         2                   6
                      NS        1         1         2         2                   6


                      Wt     0.90      0.77     10.54      2.95               15.16
                 Het Chi     0.00      0.00      3.87      1.50               11.17
                 Het  df        0         0         1         1                   5
                 Het  P      N.S.      N.S.         *      N.S.                   *
               Fixed  RR    19.89      3.50     17.43      4.40               12.39
                     RRl     2.53      0.37      9.53      1.41                7.49
                     RRu   156.15     32.80     31.87     13.78               20.50
                      P        ++      N.S.       +++         +                 +++
              Random  RR    19.89      3.50      8.75      3.94                7.19
                     RRl     2.53      0.37      1.05      0.90                2.74
                     RRu   156.15     32.80     73.09     17.28               18.87
                      P        ++      N.S.         +       (+)                 +++
             Between Chi                                                       5.81
             Between  df                                                          3
             Between  P                                                        N.S.
             Btwn(F)  P                                                        N.S.
  ________________________________________________________________________________________________________________________
                                            International Evidence on Smoking and COPD, Phase 3, Analysis run on 28-SEP-10

                                                   Table 3 - E - 3 - 3

                               IESCOPD - Meta-analysis of amount smoked : key value (1) 45
                          Any Emphysema, cigarettes (or any product if cigarettes not available)
                                                      Most-adjusted
                           Study weakness
                              Yes        No     Total


                       N        1         5         6
                      NS        1         5         6


                      Wt     0.85     14.31     15.16
                 Het Chi     0.00      8.56     11.17
                 Het  df        0         4         5
                 Het  P      N.S.       (*)         *
               Fixed  RR     2.27     13.71     12.39
                     RRl     0.27      8.17      7.49
                     RRu    18.91     23.02     20.50
                      P      N.S.       +++       +++
              Random  RR     2.27      8.70      7.19
                     RRl     0.27      3.24      2.74
                     RRu    18.91     23.38     18.87
                      P      N.S.       +++       +++
             Between Chi                         2.61
             Between  df                            1
             Between  P                          N.S.
             Btwn(F)  P                          N.S.

                           Type of outcome
                        prevalnce mortality incidence     Total


                       N        2         3         1         6
                      NS        2         3         1         6


                      Wt     1.67     12.64      0.85     15.16
                 Het Chi     1.25      6.97      0.00     11.17
                 Het  df        1         2         0         5
                 Het  P      N.S.         *      N.S.         *
               Fixed  RR     8.96     14.51      2.27     12.39
                     RRl     1.97      8.36      0.27      7.49
                     RRu    40.81     25.18     18.91     20.50
                      P        ++       +++      N.S.       +++
              Random  RR     8.83      7.89      2.27      7.19
                     RRl     1.62      1.97      0.27      2.74
                     RRu    48.31     31.59     18.91     18.87
                      P         +        ++      N.S.       +++
             Between Chi                                   2.95
             Between  df                                      2
             Between  P                                    N.S.
             Btwn(F)  P                                    N.S.

                        Emp subtype
                             mort     other     Total


                       N        3         3         6
                      NS        3         3         6


                      Wt    12.64      2.53     15.16
                 Het Chi     6.97      2.32     11.17
                 Het  df        2         2         5
                 Het  P         *      N.S.         *
               Fixed  RR    14.51      5.63     12.39
                     RRl     8.36      1.64      7.49
                     RRu    25.18     19.33     20.50
                      P       +++        ++       +++
              Random  RR     7.89      5.60      7.19
                     RRl     1.97      1.48      2.74
                     RRu    31.59     21.15     18.87
                      P        ++         +       +++
             Between Chi                         1.89
             Between  df                            1
             Between  P                          N.S.
             Btwn(F)  P                          N.S.
  ________________________________________________________________________________________________________________________
                                            International Evidence on Smoking and COPD, Phase 3, Analysis run on 28-SEP-10

                                                   Table 3 - E - 3 - 3

                               IESCOPD - Meta-analysis of amount smoked : key value (1) 45
                          Any Emphysema, cigarettes (or any product if cigarettes not available)
                                                      Most-adjusted
                        Asthma analysis type (Emphysema)
                        inc-irres  excl-all     Total


                       N        5         1         6
                      NS        5         1         6


                      Wt    14.31      0.85     15.16
                 Het Chi     8.56      0.00     11.17
                 Het  df        4         0         5
                 Het  P       (*)      N.S.         *
               Fixed  RR    13.71      2.27     12.39
                     RRl     8.17      0.27      7.49
                     RRu    23.02     18.91     20.50
                      P       +++      N.S.       +++
              Random  RR     8.70      2.27      7.19
                     RRl     3.24      0.27      2.74
                     RRu    23.38     18.91     18.87
                      P       +++      N.S.       +++
             Between Chi                         2.61
             Between  df                            1
             Between  P                          N.S.
             Btwn(F)  P                          N.S.

                                Number of emphysema cases
                             1-50    51-100   101-200      201+     Total


                       N        3         2                   1         6
                      NS        3         2                   1         6


                      Wt     2.47      3.00                9.69     15.16
                 Het Chi     0.32      0.70                0.00     11.17
                 Het  df        2         1                   0         5
                 Het  P      N.S.      N.S.                N.S.         *
               Fixed  RR     2.22      9.52               20.86     12.39
                     RRl     0.64      3.07               11.11      7.49
                     RRu     7.72     29.50               39.16     20.50
                      P      N.S.       +++                 +++       +++
              Random  RR     2.22      9.52               20.86      7.19
                     RRl     0.64      3.07               11.11      2.74
                     RRu     7.72     29.50               39.16     18.87
                      P      N.S.       +++                 +++       +++
             Between Chi                                            10.15
             Between  df                                                2
             Between  P                                                **
             Btwn(F)  P                                                 *

                        Number of emphysma cases (excluding unknown)


                       N        3         2                   1         6
                      NS        3         2                   1         6


                      Wt     2.47      3.00                9.69     15.16
                 Het Chi     0.32      0.70                0.00     11.17
                 Het  df        2         1                   0         5
                 Het  P      N.S.      N.S.                N.S.         *
               Fixed  RR     2.22      9.52               20.86     12.39
                     RRl     0.64      3.07               11.11      7.49
                     RRu     7.72     29.50               39.16     20.50
                      P      N.S.       +++                 +++       +++
              Random  RR     2.22      9.52               20.86      7.19
                     RRl     0.64      3.07               11.11      2.74
                     RRu     7.72     29.50               39.16     18.87
                      P      N.S.       +++                 +++       +++
             Between Chi                                            10.15
             Between  df                                                2
             Between  P                                                **
             Btwn(F)  P                                                 *

  ________________________________________________________________________________________________________________________
                                            International Evidence on Smoking and COPD, Phase 3, Analysis run on 28-SEP-10

                                                   Table 3 - E - 3 - 3

                               IESCOPD - Meta-analysis of amount smoked : key value (1) 45
                          Any Emphysema, cigarettes (or any product if cigarettes not available)
                                                      Most-adjusted
                            Analysis type
                         prevlnce     onset     Total


                       N        2         4         6
                      NS        2         4         6


                      Wt     1.67     13.49     15.16
                 Het Chi     1.25      9.72     11.17
                 Het  df        1         3         5
                 Het  P      N.S.         *         *
               Fixed  RR     8.96     12.90     12.39
                     RRl     1.97      7.57      7.49
                     RRu    40.81     22.00     20.50
                      P        ++       +++       +++
              Random  RR     8.83      6.18      7.19
                     RRl     1.62      1.72      2.74
                     RRu    48.31     22.25     18.87
                      P         +        ++       +++
             Between Chi                         0.20
             Between  df                            1
             Between  P                          N.S.
             Btwn(F)  P                          N.S.

                             Smoking product
                              any      cigs  cigsonly     Total


                       N                  4         2         6
                      NS                  4         2         6


                      Wt              12.30      2.87     15.16
                 Het Chi               8.85      0.26     11.17
                 Het  df                  3         1         5
                 Het  P                   *      N.S.         *
               Fixed  RR              14.81      5.77     12.39
                     RRl               8.47      1.81      7.49
                     RRu              25.89     18.37     20.50
                      P                 +++        ++       +++
              Random  RR               7.43      5.77      7.19
                     RRl               1.81      1.81      2.74
                     RRu              30.44     18.37     18.87
                      P                  ++        ++       +++
             Between Chi                                   2.06
             Between  df                                      1
             Between  P                                    N.S.
             Btwn(F)  P                                    N.S.

                                     Unexposed group
                          nev any   nev cig  nev+ any  nev+ cig     Total


                       N        4         2                             6
                      NS        4         2                             6


                      Wt    13.46      1.71                         15.16
                 Het Chi     3.96      0.09                         11.17
                 Het  df        3         1                             5
                 Het  P      N.S.      N.S.                             *
               Fixed  RR    15.82      1.81                         12.39
                     RRl     9.27      0.40                          7.49
                     RRu    26.99      8.11                         20.50
                      P       +++      N.S.                           +++
              Random  RR    13.49      1.81                          7.19
                     RRl     6.38      0.40                          2.74
                     RRu    28.50      8.11                         18.87
                      P       +++      N.S.                           +++
             Between Chi                                             7.12
             Between  df                                                1
             Between  P                                                **
             Btwn(F)  P                                               (*)
  ________________________________________________________________________________________________________________________
                                            International Evidence on Smoking and COPD, Phase 3, Analysis run on 28-SEP-10

                                                   Table 3 - E - 3 - 3

                               IESCOPD - Meta-analysis of amount smoked : key value (1) 45
                          Any Emphysema, cigarettes (or any product if cigarettes not available)
                                                      Most-adjusted
                        Unexposed group (combining nev+ with main levels)
                          nev any   nev cig     Total


                       N        4         2         6
                      NS        4         2         6


                      Wt    13.46      1.71     15.16
                 Het Chi     3.96      0.09     11.17
                 Het  df        3         1         5
                 Het  P      N.S.      N.S.         *
               Fixed  RR    15.82      1.81     12.39
                     RRl     9.27      0.40      7.49
                     RRu    26.99      8.11     20.50
                      P       +++      N.S.       +++
              Random  RR    13.49      1.81      7.19
                     RRl     6.38      0.40      2.74
                     RRu    28.50      8.11     18.87
                      P       +++      N.S.       +++
             Between Chi                         7.12
             Between  df                            1
             Between  P                            **
             Btwn(F)  P                           (*)

                        Number of adjustment variables
                                0         1        2+     Total


                       N        3         3                   6
                      NS        3         3                   6


                      Wt     2.53     12.64               15.16
                 Het Chi     2.32      6.97               11.17
                 Het  df        2         2                   5
                 Het  P      N.S.         *                   *
               Fixed  RR     5.63     14.51               12.39
                     RRl     1.64      8.36                7.49
                     RRu    19.33     25.18               20.50
                      P        ++       +++                 +++
              Random  RR     5.60      7.89                7.19
                     RRl     1.48      1.97                2.74
                     RRu    21.15     31.59               18.87
                      P         +        ++                 +++
             Between Chi                                   1.89
             Between  df                                      1
             Between  P                                    N.S.
             Btwn(F)  P                                    N.S.


  ________________________________________________________________________________________________________________________
                                            International Evidence on Smoking and COPD, Phase 3, Analysis run on 28-SEP-10

                                                   Table 3 - E - 3 - 4

                               IESCOPD - Meta-analysis of amount smoked : key value (1) 45
                          Any Emphysema, cigarettes (or any product if cigarettes not available)
                                                      Least-adjusted


     REF|NRR|X|SEX|AGEL|AGEH|     REGION|BEGYR|PUBYR|STTYP|ONSET|      DISEAS|ADJ|SMOKSTA|   PRODUCT|    UNEXP|LOW| HI|

    BEST  23     m   30   97   Am:Canada  1955  1967    Pr   Inc     Emp:mort   1 Current  Cigs only   Nev any  21   +
  DONTA2  17     m   25   84 Eu:SE/Balkn  1960  1984    Pr   Inc Emp:diagnosd   0 Current       Cigs  Nev cigs  30   +
  HUHTI1  73     m   40   64    Eu:Scand  1961  1965    CS  Prev    Emp:other   0 Current       Cigs   Nev any  25   +
   KAHN2  51     m   31   84      Am:USA  1954  1966    Pr   Inc     Emp:mort   1 Current       Cigs   Nev any  40   +
   WEISS  21     m   50   69      Am:USA  1961  1963    CS  Prev  Emp:viscomp   0 Current  Cigs only   Nev any  21   +
     WEN  21     m   35   99   Asia:FarE  1982  2004    Pr   Inc     Emp:mort   1 Current       Cigs  Nev cigs  21   +


  ________________________________________________________________________________________________________________________
                                            International Evidence on Smoking and COPD, Phase 3, Analysis run on 28-SEP-10

                                                   Table 3 - E - 3 - 5

                               IESCOPD - Meta-analysis of amount smoked : key value (1) 45
                          Any Emphysema, cigarettes (or any product if cigarettes not available)
                                                      Least-adjusted


                        Number Exposed  Non-exposed
 REF    NRR SEX ADJ     Case    Cont    Case    Cont      RR        95.00%CI
*BEST   23  m   1          7       -       3       -      6.93 (  1.79-  26.79)
*DONTA2 17  m   0          1      14       4     127      2.27 (  0.27-  18.91)
 HUHTI1 73  m   0         12      73       1     121     19.89 (  2.53- 156.15)
*KAHN2  51  m   1         21       -      18       -     20.86 ( 11.11-  39.15)
 WEISS  21  m   0          4      40       1      35      3.50 (  0.37-  32.80)
*WEN    21  m   1          -       -      13       -      1.44 (  0.17-  11.90)
Partial Totals            45     127      40     283
*prospective study


 REF    NRR SEX ADJ             Ys       Ws       Qs       Ps
*BEST   23  m   1              1.94     2.10     0.71       0.01
*DONTA2 17  m   0              0.82     0.85     2.46       0.45
 HUHTI1 73  m   0              2.99     0.90     0.20       0.00
*KAHN2  51  m   1              3.04     9.69     2.63       0.00
 WEISS  21  m   0              1.25     0.77     1.23       0.27
*WEN    21  m   1              0.36     0.85     3.94       0.74

                       N        6
                      NS        6


                      Wt    15.16
                 Het Chi    11.17
                 Het  df        5
                 Het  P         *
               Fixed  RR    12.39
                     RRl     7.49
                     RRu    20.50
                      P       +++
              Random  RR     7.19
                     RRl     2.74
                     RRu    18.87
                      P       +++
               Asymm  P         *


  ________________________________________________________________________________________________________________________
                                            International Evidence on Smoking and COPD, Phase 3, Analysis run on 28-SEP-10

                                                   Table 3 - E - 3 - 6

                               IESCOPD - Meta-analysis of amount smoked : key value (1) 45
                          Any Emphysema, cigarettes (or any product if cigarettes not available)
                                                      Least-adjusted


                       N        6
                      NS        6


                      Wt    15.16
                 Het Chi    11.17
                 Het  df        5
                 Het  P         *
               Fixed  RR    12.39
                     RRl     7.49
                     RRu    20.50
                      P       +++
              Random  RR     7.19
                     RRl     2.74
                     RRu    18.87
                      P       +++
               Asymm  P         *

                                   Sex
                             both      male    female     Total


                       N                  6                   6
                      NS                  6                   6


                      Wt              15.16               15.16
                 Het Chi              11.17               11.17
                 Het  df                  5                   5
                 Het  P                   *                   *
               Fixed  RR              12.39               12.39
                     RRl               7.49                7.49
                     RRu              20.50               20.50
                      P                 +++                 +++
              Random  RR               7.19                7.19
                     RRl               2.74                2.74
                     RRu              18.87               18.87
                      P                 +++                 +++
             Between Chi
             Between  df
             Between  P                                    N.S.
             Btwn(F)  P                                    N.S.

                                        Continent
                            NAmer    Europe      Asia  oth/mult     Total


                       N        3         2         1                   6
                      NS        3         2         1                   6


                      Wt    12.55      1.76      0.85               15.16
                 Het Chi     3.91      2.07      0.00               11.17
                 Het  df        2         1         0                   5
                 Het  P      N.S.      N.S.      N.S.                   *
               Fixed  RR    15.56      6.93      1.44               12.39
                     RRl     8.95      1.58      0.17                7.49
                     RRu    27.05     30.37     12.05               20.50
                      P       +++         +      N.S.                 +++
              Random  RR    11.18      6.82      1.44                7.19
                     RRl     4.02      0.81      0.17                2.74
                     RRu    31.11     57.24     12.05               18.87
                      P       +++       (+)      N.S.                 +++
             Between Chi                                             5.19
             Between  df                                                2
             Between  P                                               (*)
             Btwn(F)  P                                              N.S.


  ________________________________________________________________________________________________________________________
                                            International Evidence on Smoking and COPD, Phase 3, Analysis run on 28-SEP-10

                                                   Table 3 - E - 3 - 6

                               IESCOPD - Meta-analysis of amount smoked : key value (1) 45
                          Any Emphysema, cigarettes (or any product if cigarettes not available)
                                                      Least-adjusted
                               Study type
                               CC        Pr        CS     Total


                       N                  4         2         6
                      NS                  4         2         6


                      Wt              13.49      1.67     15.16
                 Het Chi               9.72      1.25     11.17
                 Het  df                  3         1         5
                 Het  P                   *      N.S.         *
               Fixed  RR              12.90      8.96     12.39
                     RRl               7.57      1.97      7.49
                     RRu              22.00     40.81     20.50
                      P                 +++        ++       +++
              Random  RR               6.18      8.83      7.19
                     RRl               1.72      1.62      2.74
                     RRu              22.25     48.31     18.87
                      P                  ++         +       +++
             Between Chi                                   0.20
             Between  df                                      1
             Between  P                                    N.S.
             Btwn(F)  P                                    N.S.

                        Emp subtype
                             mort     other     Total


                       N        3         3         6
                      NS        3         3         6


                      Wt    12.64      2.53     15.16
                 Het Chi     6.97      2.32     11.17
                 Het  df        2         2         5
                 Het  P         *      N.S.         *
               Fixed  RR    14.51      5.63     12.39
                     RRl     8.36      1.64      7.49
                     RRu    25.18     19.33     20.50
                      P       +++        ++       +++
              Random  RR     7.89      5.60      7.19
                     RRl     1.97      1.48      2.74
                     RRu    31.59     21.15     18.87
                      P        ++         +       +++
             Between Chi                         1.89
             Between  df                            1
             Between  P                          N.S.
             Btwn(F)  P                          N.S.

                             Smoking product
                              any      cigs  cigsonly     Total


                       N                  4         2         6
                      NS                  4         2         6


                      Wt              12.30      2.87     15.16
                 Het Chi               8.85      0.26     11.17
                 Het  df                  3         1         5
                 Het  P                   *      N.S.         *
               Fixed  RR              14.81      5.77     12.39
                     RRl               8.47      1.81      7.49
                     RRu              25.89     18.37     20.50
                      P                 +++        ++       +++
              Random  RR               7.43      5.77      7.19
                     RRl               1.81      1.81      2.74
                     RRu              30.44     18.37     18.87
                      P                  ++        ++       +++
             Between Chi                                   2.06
             Between  df                                      1
             Between  P                                    N.S.
             Btwn(F)  P                                    N.S.
  ________________________________________________________________________________________________________________________
                                            International Evidence on Smoking and COPD, Phase 3, Analysis run on 28-SEP-10

                                                   Table 3 - E - 3 - 6

                               IESCOPD - Meta-analysis of amount smoked : key value (1) 45
                          Any Emphysema, cigarettes (or any product if cigarettes not available)
                                                      Least-adjusted
                                     Unexposed group
                          nev any   nev cig  nev+ any  nev+ cig     Total


                       N        4         2                             6
                      NS        4         2                             6


                      Wt    13.46      1.71                         15.16
                 Het Chi     3.96      0.09                         11.17
                 Het  df        3         1                             5
                 Het  P      N.S.      N.S.                             *
               Fixed  RR    15.82      1.81                         12.39
                     RRl     9.27      0.40                          7.49
                     RRu    26.99      8.11                         20.50
                      P       +++      N.S.                           +++
              Random  RR    13.49      1.81                          7.19
                     RRl     6.38      0.40                          2.74
                     RRu    28.50      8.11                         18.87
                      P       +++      N.S.                           +++
             Between Chi                                             7.12
             Between  df                                                1
             Between  P                                                **
             Btwn(F)  P                                               (*)

                        Unexposed group (combining nev+ with main levels)
                          nev any   nev cig     Total


                       N        4         2         6
                      NS        4         2         6


                      Wt    13.46      1.71     15.16
                 Het Chi     3.96      0.09     11.17
                 Het  df        3         1         5
                 Het  P      N.S.      N.S.         *
               Fixed  RR    15.82      1.81     12.39
                     RRl     9.27      0.40      7.49
                     RRu    26.99      8.11     20.50
                      P       +++      N.S.       +++
              Random  RR    13.49      1.81      7.19
                     RRl     6.38      0.40      2.74
                     RRu    28.50      8.11     18.87
                      P       +++      N.S.       +++
             Between Chi                         7.12
             Between  df                            1
             Between  P                            **
             Btwn(F)  P                           (*)


  ________________________________________________________________________________________________________________________
                                            International Evidence on Smoking and COPD, Phase 3, Analysis run on 28-SEP-10

                                                   Table 3 - E - 3 - 7

                               IESCOPD - Meta-analysis of amount smoked : key value (1) 45
                          Any Emphysema, cigarettes (or any product if cigarettes not available)
                                 Excluded studies (and stage at which they were excluded)


1       CLARK COTTON  MEYER REMYJA RUTGER SNYDER SOBRAX     SU TAKEMU  WANG4   WEIR WHICKE ZALACA
2      ALDERS ALESSA  AMIGO ANDER1 ANDER3   BANG  BECK1  BECK2 BEDNAR BJORNS BROGGE  BROWN CERVER CHAPMA  CHEN1  CHEN2
        CHEN3  CHENG CLEMEN COATES  COCCI COLLEG  DEAN1  DEAN2  DEANE DEJONG DEMARC DETORR DICKIN  DOLL1  DOLL2 DONTA1
       DOPICO EHRLIC EKBERG ENSTRO FERRI1 FERRI2 FERRI3  FIDAN FINKLE FLETCH FORAST FOXMAN FUKUCH GEIJER GODTFR GOLDBE
       HAENSZ HARIKK HARRIS HAWTHO  HAYES HEDMAN HIGGI2 HIGGI3 HIGGI4 HIGGI6 HOLLA2 HOLLNA  HOUSE HRUBEC HUCHON HUHTI2
       HUHTI3 ITABAS JACOBS JAENDI JENSEN JINDA2 JOHANN  JOSHI JOUSI1 KACHEL KARAKA KATANC   KATO KHOURY    KIM  KIRAZ
       KLAYTO KOJIMA KOTAN1 KOTAN2 KRZYZA  KUBIK KULLER    LAI   LAM1   LAM2   LAM3 LAMBER  LANGE LANGE2 LANGHA    LEE
         LIAW LINDBE LINDST   LIU1   LIU2 LUNDB1 LUNDB2  MADOR MAGNUS MANFRE MANNI1 MANNI2 MANNI3 MARAN1 MARAN2 MARCUS
       MATHES MELLST MENEZ1 MENEZ2 MENEZ3 MENEZ4 MENEZ5 MENEZ6  MEREN  MILNE MOLLER MONTNE MUELLE NEJJAR NIEPSU NIHLEN
       NILSSO OGILVI OSWAL1 OSWAL2 PANDEY   PEAT PELKON PEREZP   PETO  PRICE   REID RENWIC RICCIO RIMING SARGEA SAWICK
       SCHWAR SHAHAB  SHARP SHIMUR   SHIN SICHLE SOBRAD SPEIZE STERLI STJERN  STROM SUADIC  TAGER TAGER2   TANG   THUN
         TODD TROISI TRUPIN TSUSHI TVERDA URRUTI VESTBO VIEGI1 VIEGI2 VINEIS VOLLM1 VOLLM2 VONHER WAGEN2   WALD WATSON
          WIG WILHEL WILSO1 WOJTYN  WOODS  WOOLF   XIAO     XU YAMAGU   YUAN ZIELI1 ZIELI2 ZIETKO   ZOIA
3        KAHN
4      ANDER2 ENRIGH GULSVI HARDIE     HO HOZAWA LEBOWI MILLER   NAWA  OMORI  PRATT  RYDER  SILVA SUTINE VIKGRE  WANG2
5      WILSO2
6      AUERBA HAMMO2 LAVECC
7      HIRAYA


  ________________________________________________________________________________________________________________________
                                            International Evidence on Smoking and COPD, Phase 3, Analysis run on 28-SEP-10

                                                   Table 3 - E - 3 - 8

                               IESCOPD - Meta-analysis of amount smoked : key value (1) 45
                          Any Emphysema, cigarettes (or any product if cigarettes not available)
                                             Potentially overlapping studies


     REF| REFGP|PRINC|                     OVERLAP|

  DONTA2 JACOBS     2  JACOBS/DONTA1/DONTA2/PELKON
  HUHTI1 HUHTI1     1                HUHTI1/HUHTI2
     WEN    WEN     1                     WEN/LIAW
   KAHN2   KAHN     2                   KAHN/KAHN2

                                    Most-adjusted - insufficient data for meta-analysis
     REF|NRR|SEX|AGEL|AGEH|     REGION|BEGYR|PUBYR|STTYP|ONSET|      DISEAS|ADJ|SMOKSTA|   PRODUCT|    UNEXP|LOW| HI|

  HIRAYA  22   m   40   99   Asia:FarE  1965  1982    Pr   Inc     Emp:mort   1 Current       Cigs  Nev cigs  30   +
          RR|SIG|

        1.90   ?


  ________________________________________________________________________________________________________________________
                                            International Evidence on Smoking and COPD, Phase 3, Analysis run on 28-SEP-10

                                                    Table 3 - E - 10 -

                               IESCOPD - Meta-analysis of amount smoked : highest vs lowest
                          Any Emphysema, cigarettes (or any product if cigarettes not available)


This analysis is restricted to results for:
1) Eligible study on database
2) Outcome Emphysema
3) Current or ever smoking
4) Categorical dose-response data for amount smoked
5) High vs low base
6) Results complete enough for use in meta-analysis

Within each study, results are then selected (in the following order of preference, within each sex) for:
7) SMKSTA  : current, ever
8) PROD    : cigarettes, cigarettes only, any product
9) The highest vs lowest category
10) For overlapping studies: principal rather than subsidiary studies
and then for single sex results (m, f) in preference to results for both sexes combined (b).

Results adjusted for the most potential confounders are then chosen in Sections -1 to -3
and results adjusted for the least confounders in Sections -4 to -6. (Those least-adjusted results which
actually differ from the most-adjusted are marked 'x' in column X in Section -4)

Section -7 shows excluded studies, together with the stage (as above) at which no qualifying
results were found.

Section -8 lists the potentially overlapping studies which have been included (1=principal, 2=subsidiary),
and any results which would have been included in preference except that they had data not complete enough
for use in meta-analysis. It also lists their significance (yes/no), if known.


  ________________________________________________________________________________________________________________________
                                            International Evidence on Smoking and COPD, Phase 3, Analysis run on 28-SEP-10

                                                   Table 3 - E - 10 - 1

                               IESCOPD - Meta-analysis of amount smoked : highest vs lowest
                          Any Emphysema, cigarettes (or any product if cigarettes not available)
                                                      Most-adjusted


     REF|NRR|SEX|AGEL|AGEH|     REGION|BEGYR|PUBYR|STTYP|ONSET|      DISEAS|ADJ|SMOKSTA|   PRODUCT|BASE-HI|LOW| HI|

  AUERBA  12   m   15   99      Am:USA  1963  1972    CS  Prev  Emp:viscomp   1 Current       Cigs       9  20   +
    BEST  25   m   30   97   Am:Canada  1955  1967    Pr   Inc     Emp:mort   1 Current  Cigs only       9  21   +
  DONTA2  20   m   25   84 Eu:SE/Balkn  1960  1984    Pr   Inc Emp:diagnosd   0 Current       Cigs       9  30   +
  HAMMO2  15   f   35   99      Am:USA  1959  1966    Pr   Inc     Emp:mort   1    Ever       Cigs       9  10   +
  HUHTI1  77   m   40   64    Eu:Scand  1961  1965    CS  Prev    Emp:other   1 Current       Cigs      14  25   +
   KAHN2  54   m   31   84      Am:USA  1954  1966    Pr   Inc     Emp:mort   1 Current       Cigs       9  40   +
  LAVECC  42   b   15   99     Eu:West  1983  1988    CS  Prev Emp:self-rep   6 Current       Cigs      14  15   +
   WEISS  23   m   50   69      Am:USA  1961  1963    CS  Prev  Emp:viscomp   0 Current  Cigs only       9  21   +
     WEN  23   m   35   99   Asia:FarE  1982  2004    Pr   Inc     Emp:mort   1 Current       Cigs      10  21   +
  WILSO2   3   m   15   99      Aus/NZ  1998  2004    CS  Prev Emp:diagnosd   0 Current       Cigs      24  25   +
  WILSO2   4   f   15   99      Aus/NZ  1998  2004    CS  Prev Emp:diagnosd   0 Current       Cigs      24  25   +


  ________________________________________________________________________________________________________________________
                                            International Evidence on Smoking and COPD, Phase 3, Analysis run on 28-SEP-10

                                                   Table 3 - E - 10 - 2

                               IESCOPD - Meta-analysis of amount smoked : highest vs lowest
                          Any Emphysema, cigarettes (or any product if cigarettes not available)
                                                      Most-adjusted


                        Number Exposed  Non-exposed
 REF    NRR SEX ADJ     Case    Cont    Case    Cont      RR        95.00%CI
 AUERBA 12  m   1        656       -      48       -    161.53 ( 39.47- 661.09)
*BEST   25  m   1          7       -       9       -      1.44 (  0.61-   3.42)
*DONTA2 20  m   0          1      14       1     118      8.43 (  0.56- 127.42)
*HAMMO2 15  f   1         15       -       9       -      3.45 (  1.50-   7.95)
 HUHTI1 77  m   1         12       -      20       -      0.71 (  0.24-   2.09)
*KAHN2  54  m   1         21       -      22       -      3.74 (  2.08-   6.73)
 LAVECC 42  b   6        230       -     202       -      1.05 (  0.84-   1.31)
 WEISS  23  m   0          4      40       4      11      0.28 (  0.06-   1.28)
*WEN    23  m   1          -       -       -       -      1.69 (  0.16-  17.93)
 WILSO2 3   m   0          2      85       2     304      3.58 (  0.50-  25.77)
 WILSO2 4   f   0          1      42       1     296      7.05 (  0.43- 114.81)
 Subtotal WILSO2                                          4.48 (  0.89-  22.48)
Partial Totals           949     181     318     729
*prospective study


 REF    NRR SEX ADJ             Ys       Ws       Qs       Ps
 AUERBA 12  m   1              5.08     1.93    43.48       0.00
*BEST   25  m   1              0.36     5.17     0.00       0.41
*DONTA2 20  m   0              2.13     0.52     1.66       0.12
*HAMMO2 15  f   1              1.24     5.52     4.42       0.00
 HUHTI1 77  m   1             -0.34     3.28     1.54       0.54
*KAHN2  54  m   1              1.32    11.14    10.60       0.00
 LAVECC 42  b   6              0.05    77.81     6.77       0.67
 WEISS  23  m   0             -1.29     1.62     4.34       0.10
*WEN    23  m   1              0.52     0.69     0.02       0.66
 WILSO2 3   m   0              1.27     0.99     0.85       0.21
 WILSO2 4   f   0              1.95     0.49     1.28       0.17
 Subtotal WILSO2               1.50     1.48     2.13

                       N       11
                      NS       10


                      Wt   109.18
                 Het Chi    74.98
                 Het  df       10
                 Het  P       ***
               Fixed  RR     1.41
                     RRl     1.17
                     RRu     1.70
                      P       +++
              Random  RR     2.73
                     RRl     1.23
                     RRu     6.04
                      P         +
               Asymm  P      N.S.


  ________________________________________________________________________________________________________________________
                                            International Evidence on Smoking and COPD, Phase 3, Analysis run on 28-SEP-10

                                                   Table 3 - E - 10 - 3

                               IESCOPD - Meta-analysis of amount smoked : highest vs lowest
                          Any Emphysema, cigarettes (or any product if cigarettes not available)
                                                      Most-adjusted


                       N       11
                      NS       10


                      Wt   109.18
                 Het Chi    74.98
                 Het  df       10
                 Het  P       ***
               Fixed  RR     1.41
                     RRl     1.17
                     RRu     1.70
                      P       +++
              Random  RR     2.73
                     RRl     1.23
                     RRu     6.04
                      P         +
               Asymm  P      N.S.

                                   Sex
                             both      male    female     Total


                       N        1         8         2        11
                      NS        1         8         2        11


                      Wt    77.81     25.35      6.02    109.18
                 Het Chi     0.00     50.82      0.23     74.98
                 Het  df        0         7         1        10
                 Het  P      N.S.       ***      N.S.       ***
               Fixed  RR     1.05      2.78      3.66      1.41
                     RRl     0.84      1.88      1.65      1.17
                     RRu     1.31      4.10      8.13      1.70
                      P      N.S.       +++        ++       +++
              Random  RR     1.05      2.99      3.66      2.73
                     RRl     0.84      0.90      1.65      1.23
                     RRu     1.31      9.93      8.13      6.04
                      P      N.S.       (+)        ++         +
             Between Chi                                  23.93
             Between  df                                      2
             Between  P                                     ***
             Btwn(F)  P                                    N.S.

                                        Continent
                            NAmer    Europe      Asia  oth/mult     Total


                       N        5         3         1         2        11
                      NS        5         3         1         1        10


                      Wt    25.40     81.61      0.69      1.48    109.18
                 Het Chi    43.02      2.76      0.00      0.15     74.98
                 Het  df        4         2         0         1        10
                 Het  P       ***      N.S.      N.S.      N.S.       ***
               Fixed  RR     3.41      1.05      1.69      4.48      1.41
                     RRl     2.31      0.84      0.16      0.89      1.17
                     RRu     5.03      1.30     17.89     22.48      1.70
                      P       +++      N.S.      N.S.       (+)       +++
              Random  RR     3.79      1.06      1.69      4.48      2.73
                     RRl     0.96      0.59      0.16      0.89      1.23
                     RRu    15.03      1.89     17.89     22.48      6.04
                      P       (+)      N.S.      N.S.       (+)         +
             Between Chi                                            29.04
             Between  df                                                3
             Between  P                                               ***
             Btwn(F)  P                                              N.S.


  ________________________________________________________________________________________________________________________
                                            International Evidence on Smoking and COPD, Phase 3, Analysis run on 28-SEP-10

                                                   Table 3 - E - 10 - 3

                               IESCOPD - Meta-analysis of amount smoked : highest vs lowest
                          Any Emphysema, cigarettes (or any product if cigarettes not available)
                                                      Most-adjusted
                        National cigarette tobacco type (excluding mixed/unkown)
                          blended  virginia     Total


                       N        7         3        10
                      NS        7         2         9


                      Wt   101.84      6.65    108.49
                 Het Chi    72.79      1.64     74.95
                 Het  df        6         2         9
                 Het  P       ***      N.S.       ***
               Fixed  RR     1.38      1.85      1.41
                     RRl     1.14      0.87      1.17
                     RRu     1.68      3.96      1.70
                      P        ++      N.S.       +++
              Random  RR     2.92      1.85      2.82
                     RRl     1.01      0.87      1.23
                     RRu     8.41      3.96      6.48
                      P         +      N.S.         +
             Between Chi                         0.53
             Between  df                            1
             Between  P                          N.S.
             Btwn(F)  P                          N.S.

                                        Start year of study
                            <1970   1970-79   1980-89   1990-99     2000+   unknown     Total


                       N        7                   2         2                            11
                      NS        7                   2         1                            10


                      Wt    29.20               78.50      1.48                        109.18
                 Het Chi    50.78                0.15      0.15                         74.98
                 Het  df        6                   1         1                            10
                 Het  P       ***                N.S.      N.S.                           ***
               Fixed  RR     2.91                1.05      4.48                          1.41
                     RRl     2.02                0.85      0.89                          1.17
                     RRu     4.18                1.32     22.48                          1.70
                      P       +++                N.S.       (+)                           +++
              Random  RR     3.15                1.05      4.48                          2.73
                     RRl     0.99                0.85      0.89                          1.23
                     RRu    10.05                1.32     22.48                          6.04
                      P       (+)                N.S.       (+)                             +
             Between Chi                                                                23.89
             Between  df                                                                    2
             Between  P                                                                   ***
             Btwn(F)  P                                                                  N.S.

                                Publication year
                            <1980   1980-89   1990-99     2000+     Total


                       N        6         2                   3        11
                      NS        6         2                   2        10


                      Wt    28.68     78.33                2.17    109.18
                 Het Chi    50.18      2.24                0.60     74.98
                 Het  df        5         1                   2        10
                 Het  P       ***      N.S.                N.S.       ***
               Fixed  RR     2.85      1.06                3.29      1.41
                     RRl     1.98      0.85                0.87      1.17
                     RRu     4.11      1.33               12.44      1.70
                      P       +++      N.S.                 (+)       +++
              Random  RR     2.87      1.88                3.29      2.73
                     RRl     0.83      0.30                0.87      1.23
                     RRu     9.85     11.77               12.44      6.04
                      P       (+)      N.S.                 (+)         +
             Between Chi                                            21.95
             Between  df                                                2
             Between  P                                               ***
             Btwn(F)  P                                              N.S.
  ________________________________________________________________________________________________________________________
                                            International Evidence on Smoking and COPD, Phase 3, Analysis run on 28-SEP-10

                                                   Table 3 - E - 10 - 3

                               IESCOPD - Meta-analysis of amount smoked : highest vs lowest
                          Any Emphysema, cigarettes (or any product if cigarettes not available)
                                                      Most-adjusted
                               Study type
                               CC        Pr        CS     Total


                       N                  5         6        11
                      NS                  5         5        10


                      Wt              23.05     86.13    109.18
                 Het Chi               4.21     54.92     74.98
                 Het  df                  4         5        10
                 Het  P                N.S.       ***       ***
               Fixed  RR               2.95      1.16      1.41
                     RRl               1.96      0.94      1.17
                     RRu               4.43      1.43      1.70
                      P                 +++      N.S.       +++
              Random  RR               2.92      2.84      2.73
                     RRl               1.90      0.59      1.23
                     RRu               4.49     13.64      6.04
                      P                 +++      N.S.         +
             Between Chi                                  15.85
             Between  df                                      1
             Between  P                                     ***
             Btwn(F)  P                                    N.S.

                                    Lowest age in RR
                        <25/unlim     25-39       40+   unknown     Total


                       N        4         5         2                  11
                      NS        3         5         2                  10


                      Wt    81.22     23.05      4.90              109.18
                 Het Chi    50.59      4.21      0.98               74.98
                 Het  df        3         4         1                  10
                 Het  P       ***      N.S.      N.S.                 ***
               Fixed  RR     1.22      2.95      0.52                1.41
                     RRl     0.98      1.96      0.21                1.17
                     RRu     1.51      4.43      1.26                1.70
                      P       (+)       +++      N.S.                 +++
              Random  RR     7.94      2.92      0.52                2.73
                     RRl     0.50      1.90      0.21                1.23
                     RRu   127.21      4.49      1.26                6.04
                      P      N.S.       +++      N.S.                   +
             Between Chi                                            19.20
             Between  df                                                2
             Between  P                                               ***
             Btwn(F)  P                                              N.S.

                                         Highest age in RR
                              <65     65-74     75-84 85+/unlim   unknown     Total


                       N        1         1         2         7                  11
                      NS        1         1         2         6                  10


                      Wt     3.28      1.62     11.67     92.61              109.18
                 Het Chi     0.00      0.00      0.33     56.31               74.98
                 Het  df        0         0         1         6                  10
                 Het  P      N.S.      N.S.      N.S.       ***                 ***
               Fixed  RR     0.71      0.28      3.88      1.31                1.41
                     RRl     0.24      0.06      2.18      1.07                1.17
                     RRu     2.10      1.28      6.88      1.60                1.70
                      P      N.S.       (-)       +++        ++                 +++
              Random  RR     0.71      0.28      3.88      4.18                2.73
                     RRl     0.24      0.06      2.18      1.29                1.23
                     RRu     2.10      1.28      6.88     13.48                6.04
                      P      N.S.       (-)       +++         +                   +
             Between Chi                                                      18.33
             Between  df                                                          3
             Between  P                                                         ***
             Btwn(F)  P                                                        N.S.
  ________________________________________________________________________________________________________________________
                                            International Evidence on Smoking and COPD, Phase 3, Analysis run on 28-SEP-10

                                                   Table 3 - E - 10 - 3

                               IESCOPD - Meta-analysis of amount smoked : highest vs lowest
                          Any Emphysema, cigarettes (or any product if cigarettes not available)
                                                      Most-adjusted
                           Study weakness
                              Yes        No     Total


                       N        1        10        11
                      NS        1         9        10


                      Wt     0.52    108.66    109.18
                 Het Chi     0.00     73.30     74.98
                 Het  df        0         9        10
                 Het  P      N.S.       ***       ***
               Fixed  RR     8.43      1.40      1.41
                     RRl     0.56      1.16      1.17
                     RRu   127.42      1.69      1.70
                      P      N.S.       +++       +++
              Random  RR     8.43      2.57      2.73
                     RRl     0.56      1.13      1.23
                     RRu   127.42      5.81      6.04
                      P      N.S.         +         +
             Between Chi                         1.67
             Between  df                            1
             Between  P                          N.S.
             Btwn(F)  P                          N.S.

                           Type of outcome
                        prevalnce mortality incidence     Total


                       N        6         4         1        11
                      NS        5         4         1        10


                      Wt    86.13     22.53      0.52    109.18
                 Het Chi    54.92      3.62      0.00     74.98
                 Het  df        5         3         0        10
                 Het  P       ***      N.S.      N.S.       ***
               Fixed  RR     1.16      2.87      8.43      1.41
                     RRl     0.94      1.90      0.56      1.17
                     RRu     1.43      4.34    127.42      1.70
                      P      N.S.       +++      N.S.       +++
              Random  RR     2.84      2.79      8.43      2.73
                     RRl     0.59      1.73      0.56      1.23
                     RRu    13.64      4.50    127.42      6.04
                      P      N.S.       +++      N.S.         +
             Between Chi                                  16.44
             Between  df                                      2
             Between  P                                     ***
             Btwn(F)  P                                    N.S.

                        Emp subtype
                             mort     other     Total


                       N        4         7        11
                      NS        4         6        10


                      Wt    22.53     86.65    109.18
                 Het Chi     3.62     56.96     74.98
                 Het  df        3         6        10
                 Het  P      N.S.       ***       ***
               Fixed  RR     2.87      1.17      1.41
                     RRl     1.90      0.95      1.17
                     RRu     4.34      1.45      1.70
                      P       +++      N.S.       +++
              Random  RR     2.79      3.19      2.73
                     RRl     1.73      0.74      1.23
                     RRu     4.50     13.72      6.04
                      P       +++      N.S.         +
             Between Chi                        14.40
             Between  df                            1
             Between  P                           ***
             Btwn(F)  P                          N.S.
  ________________________________________________________________________________________________________________________
                                            International Evidence on Smoking and COPD, Phase 3, Analysis run on 28-SEP-10

                                                   Table 3 - E - 10 - 3

                               IESCOPD - Meta-analysis of amount smoked : highest vs lowest
                          Any Emphysema, cigarettes (or any product if cigarettes not available)
                                                      Most-adjusted
                        Asthma analysis type (Emphysema)
                        inc-irres  excl-all     Total


                       N       10         1        11
                      NS        9         1        10


                      Wt   108.66      0.52    109.18
                 Het Chi    73.30      0.00     74.98
                 Het  df        9         0        10
                 Het  P       ***      N.S.       ***
               Fixed  RR     1.40      8.43      1.41
                     RRl     1.16      0.56      1.17
                     RRu     1.69    127.42      1.70
                      P       +++      N.S.       +++
              Random  RR     2.57      8.43      2.73
                     RRl     1.13      0.56      1.23
                     RRu     5.81    127.42      6.04
                      P         +      N.S.         +
             Between Chi                         1.67
             Between  df                            1
             Between  P                          N.S.
             Btwn(F)  P                          N.S.

                                Number of emphysema cases
                             1-50    51-100   101-200      201+     Total


                       N        5         2                   4        11
                      NS        4         2                   4        10


                      Wt     4.31      8.45               96.42    109.18
                 Het Chi     8.15      1.00               65.23     74.98
                 Het  df        4         1                   3        10
                 Het  P       (*)      N.S.                 ***       ***
               Fixed  RR     1.45      1.09                1.44      1.41
                     RRl     0.56      0.56                1.18      1.17
                     RRu     3.72      2.15                1.76      1.70
                      P      N.S.      N.S.                 +++       +++
              Random  RR     1.97      1.09                5.86      2.73
                     RRl     0.48      0.56                1.40      1.23
                     RRu     8.05      2.15               24.49      6.04
                      P      N.S.      N.S.                   +         +
             Between Chi                                             0.59
             Between  df                                                2
             Between  P                                              N.S.
             Btwn(F)  P                                              N.S.

                        Number of emphysma cases (excluding unknown)


                       N        5         2                   4        11
                      NS        4         2                   4        10


                      Wt     4.31      8.45               96.42    109.18
                 Het Chi     8.15      1.00               65.23     74.98
                 Het  df        4         1                   3        10
                 Het  P       (*)      N.S.                 ***       ***
               Fixed  RR     1.45      1.09                1.44      1.41
                     RRl     0.56      0.56                1.18      1.17
                     RRu     3.72      2.15                1.76      1.70
                      P      N.S.      N.S.                 +++       +++
              Random  RR     1.97      1.09                5.86      2.73
                     RRl     0.48      0.56                1.40      1.23
                     RRu     8.05      2.15               24.49      6.04
                      P      N.S.      N.S.                   +         +
             Between Chi                                             0.59
             Between  df                                                2
             Between  P                                              N.S.
             Btwn(F)  P                                              N.S.

  ________________________________________________________________________________________________________________________
                                            International Evidence on Smoking and COPD, Phase 3, Analysis run on 28-SEP-10

                                                   Table 3 - E - 10 - 3

                               IESCOPD - Meta-analysis of amount smoked : highest vs lowest
                          Any Emphysema, cigarettes (or any product if cigarettes not available)
                                                      Most-adjusted
                            Analysis type
                         prevlnce     onset     Total


                       N        6         5        11
                      NS        5         5        10


                      Wt    86.13     23.05    109.18
                 Het Chi    54.92      4.21     74.98
                 Het  df        5         4        10
                 Het  P       ***      N.S.       ***
               Fixed  RR     1.16      2.95      1.41
                     RRl     0.94      1.96      1.17
                     RRu     1.43      4.43      1.70
                      P      N.S.       +++       +++
              Random  RR     2.84      2.92      2.73
                     RRl     0.59      1.90      1.23
                     RRu    13.64      4.49      6.04
                      P      N.S.       +++         +
             Between Chi                        15.85
             Between  df                            1
             Between  P                           ***
             Btwn(F)  P                          N.S.

                             Smoking product
                              any      cigs  cigsonly     Total


                       N                  9         2        11
                      NS                  8         2        10


                      Wt             102.39      6.79    109.18
                 Het Chi              70.57      3.39     74.98
                 Het  df                  8         1        10
                 Het  P                 ***       (*)       ***
               Fixed  RR               1.45      0.97      1.41
                     RRl               1.19      0.46      1.17
                     RRu               1.75      2.06      1.70
                      P                 +++      N.S.       +++
              Random  RR               3.91      0.71      2.73
                     RRl               1.51      0.14      1.23
                     RRu              10.14      3.55      6.04
                      P                  ++      N.S.         +
             Between Chi                                   1.02
             Between  df                                      1
             Between  P                                    N.S.
             Btwn(F)  P                                    N.S.

                        Number of adjustment variables
                                0         1        2+     Total


                       N        4         6         1        11
                      NS        3         6         1        10


                      Wt     3.62     27.74     77.81    109.18
                 Het Chi     8.13     41.06      0.00     74.98
                 Het  df        3         5         0        10
                 Het  P         *       ***      N.S.       ***
               Fixed  RR     1.41      3.23      1.05      1.41
                     RRl     0.50      2.22      0.84      1.17
                     RRu     3.94      4.68      1.31      1.70
                      P      N.S.       +++      N.S.       +++
              Random  RR     2.21      3.84      1.05      2.73
                     RRl     0.37      1.19      0.84      1.23
                     RRu    13.28     12.32      1.31      6.04
                      P      N.S.         +      N.S.         +
             Between Chi                                  25.78
             Between  df                                      2
             Between  P                                     ***
             Btwn(F)  P                                    N.S.
  ________________________________________________________________________________________________________________________
                                            International Evidence on Smoking and COPD, Phase 3, Analysis run on 28-SEP-10

                                                   Table 3 - E - 10 - 4

                               IESCOPD - Meta-analysis of amount smoked : highest vs lowest
                          Any Emphysema, cigarettes (or any product if cigarettes not available)
                                                      Least-adjusted


     REF|NRR|X|SEX|AGEL|AGEH|     REGION|BEGYR|PUBYR|STTYP|ONSET|      DISEAS|ADJ|SMOKSTA|   PRODUCT|BASE-HI|LOW| HI|

  AUERBA  10 x   m   15   99      Am:USA  1963  1972    CS  Prev  Emp:viscomp   0 Current       Cigs       9  20   +
    BEST  25     m   30   97   Am:Canada  1955  1967    Pr   Inc     Emp:mort   1 Current  Cigs only       9  21   +
  DONTA2  20     m   25   84 Eu:SE/Balkn  1960  1984    Pr   Inc Emp:diagnosd   0 Current       Cigs       9  30   +
  HAMMO2  15     f   35   99      Am:USA  1959  1966    Pr   Inc     Emp:mort   1    Ever       Cigs       9  10   +
  HUHTI1  75 x   m   40   64    Eu:Scand  1961  1965    CS  Prev    Emp:other   0 Current       Cigs      14  25   +
   KAHN2  54     m   31   84      Am:USA  1954  1966    Pr   Inc     Emp:mort   1 Current       Cigs       9  40   +
  LAVECC  36 x   m   15   99     Eu:West  1983  1988    CS  Prev Emp:self-rep   0 Current       Cigs      14  15   +
  LAVECC  39 x   f   15   99     Eu:West  1983  1988    CS  Prev Emp:self-rep   0 Current       Cigs      14  15   +
   WEISS  23     m   50   69      Am:USA  1961  1963    CS  Prev  Emp:viscomp   0 Current  Cigs only       9  21   +
     WEN  23     m   35   99   Asia:FarE  1982  2004    Pr   Inc     Emp:mort   1 Current       Cigs      10  21   +
  WILSO2   3     m   15   99      Aus/NZ  1998  2004    CS  Prev Emp:diagnosd   0 Current       Cigs      24  25   +
  WILSO2   4     f   15   99      Aus/NZ  1998  2004    CS  Prev Emp:diagnosd   0 Current       Cigs      24  25   +


  ________________________________________________________________________________________________________________________
                                            International Evidence on Smoking and COPD, Phase 3, Analysis run on 28-SEP-10

                                                   Table 3 - E - 10 - 5

                               IESCOPD - Meta-analysis of amount smoked : highest vs lowest
                          Any Emphysema, cigarettes (or any product if cigarettes not available)
                                                      Least-adjusted


                        Number Exposed  Non-exposed
 REF    NRR SEX ADJ     Case    Cont    Case    Cont      RR        95.00%CI
 AUERBA 10  m   0        656       2      48      18    123.00 ( 27.72- 545.75)
*BEST   25  m   1          7       -       9       -      1.44 (  0.61-   3.42)
*DONTA2 20  m   0          1      14       1     118      8.43 (  0.56- 127.42)
*HAMMO2 15  f   1         15       -       9       -      3.45 (  1.50-   7.95)
 HUHTI1 75  m   0         12      73      20      88      0.72 (  0.33-   1.58)
*KAHN2  54  m   1         21       -      22       -      3.74 (  2.08-   6.73)
 LAVECC 36  m   0        201    9593     170    5341      0.66 (  0.54-   0.81)
 LAVECC 39  f   0         29    2090      32    4475      1.94 (  1.17-   3.22)
 Subtotal LAVECC                                          0.77 (  0.63-   0.93)
 WEISS  23  m   0          4      40       4      11      0.28 (  0.06-   1.28)
*WEN    23  m   1          -       -       -       -      1.69 (  0.16-  17.93)
 WILSO2 3   m   0          2      85       2     304      3.58 (  0.50-  25.77)
 WILSO2 4   f   0          1      42       1     296      7.05 (  0.43- 114.81)
 Subtotal WILSO2                                          4.48 (  0.89-  22.48)
Partial Totals           949   11939     318   10651
*prospective study


 REF    NRR SEX ADJ             Ys       Ws       Qs       Ps
 AUERBA 10  m   0              4.81     1.73    39.62       0.00
*BEST   25  m   1              0.36     5.17     0.59       0.41
*DONTA2 20  m   0              2.13     0.52     2.31       0.12
*HAMMO2 15  f   1              1.24     5.52     8.10       0.00
 HUHTI1 75  m   0             -0.32     6.31     0.78       0.42
*KAHN2  54  m   1              1.32    11.14    18.60       0.00
 LAVECC 36  m   0             -0.42    89.69    17.79       0.00
 LAVECC 39  f   0              0.66    15.05     6.08       0.01
 Subtotal LAVECC              -0.26   104.75    23.87
 WEISS  23  m   0             -1.29     1.62     2.82       0.10
*WEN    23  m   1              0.52     0.69     0.17       0.66
 WILSO2 3   m   0              1.27     0.99     1.53       0.21
 WILSO2 4   f   0              1.95     0.49     1.83       0.17
 Subtotal WILSO2               1.50     1.48     3.36

                       N       12
                      NS       10


                      Wt   138.94
                 Het Chi   100.22
                 Het  df       11
                 Het  P       ***
               Fixed  RR     1.03
                     RRl     0.87
                     RRu     1.21
                      P      N.S.
              Random  RR     2.37
                     RRl     1.15
                     RRu     4.86
                      P         +
               Asymm  P         *


  ________________________________________________________________________________________________________________________
                                            International Evidence on Smoking and COPD, Phase 3, Analysis run on 28-SEP-10

                                                   Table 3 - E - 10 - 6

                               IESCOPD - Meta-analysis of amount smoked : highest vs lowest
                          Any Emphysema, cigarettes (or any product if cigarettes not available)
                                                      Least-adjusted


                       N       12
                      NS       10


                      Wt   138.94
                 Het Chi   100.22
                 Het  df       11
                 Het  P       ***
               Fixed  RR     1.03
                     RRl     0.87
                     RRu     1.21
                      P      N.S.
              Random  RR     2.37
                     RRl     1.15
                     RRu     4.86
                      P         +
               Asymm  P         *

                                   Sex
                             both      male    female     Total


                       N                  9         3        12
                      NS                  9         3        12


                      Wt             117.87     21.07    138.94
                 Het Chi              81.69      1.96    100.22
                 Het  df                  8         2        11
                 Het  P                 ***      N.S.       ***
               Fixed  RR               0.89      2.33      1.03
                     RRl               0.74      1.52      0.87
                     RRu               1.06      3.56      1.21
                      P                N.S.       +++      N.S.
              Random  RR               2.24      2.33      2.37
                     RRl               0.88      1.52      1.15
                     RRu               5.71      3.56      4.86
                      P                 (+)       +++         +
             Between Chi                                  16.57
             Between  df                                      1
             Between  P                                     ***
             Btwn(F)  P                                    N.S.

                                        Continent
                            NAmer    Europe      Asia  oth/mult     Total


                       N        5         4         1         2        12
                      NS        5         3         1         1        10


                      Wt    25.19    111.58      0.69      1.48    138.94
                 Het Chi    36.41     18.07      0.00      0.15    100.22
                 Het  df        4         3         0         1        11
                 Het  P       ***       ***      N.S.      N.S.       ***
               Fixed  RR     3.25      0.77      1.69      4.48      1.03
                     RRl     2.20      0.64      0.16      0.89      0.87
                     RRu     4.80      0.93     17.89     22.48      1.21
                      P       +++        --      N.S.       (+)      N.S.
              Random  RR     3.54      1.12      1.69      4.48      2.37
                     RRl     0.98      0.52      0.16      0.89      1.15
                     RRu    12.74      2.38     17.89     22.48      4.86
                      P       (+)      N.S.      N.S.       (+)         +
             Between Chi                                            45.59
             Between  df                                                3
             Between  P                                               ***
             Btwn(F)  P                                              N.S.


  ________________________________________________________________________________________________________________________
                                            International Evidence on Smoking and COPD, Phase 3, Analysis run on 28-SEP-10

                                                   Table 3 - E - 10 - 6

                               IESCOPD - Meta-analysis of amount smoked : highest vs lowest
                          Any Emphysema, cigarettes (or any product if cigarettes not available)
                                                      Least-adjusted
                               Study type
                               CC        Pr        CS     Total


                       N                  5         7        12
                      NS                  5         5        10


                      Wt              23.05    115.89    138.94
                 Het Chi               4.21     65.37    100.22
                 Het  df                  4         6        11
                 Het  P                N.S.       ***       ***
               Fixed  RR               2.95      0.83      1.03
                     RRl               1.96      0.69      0.87
                     RRu               4.43      1.00      1.21
                      P                 +++         -      N.S.
              Random  RR               2.92      2.12      2.37
                     RRl               1.90      0.78      1.15
                     RRu               4.49      5.76      4.86
                      P                 +++      N.S.         +
             Between Chi                                  30.64
             Between  df                                      1
             Between  P                                     ***
             Btwn(F)  P                                     (*)

                        Emp subtype
                             mort     other     Total


                       N        4         8        12
                      NS        4         6        10


                      Wt    22.53    116.41    138.94
                 Het Chi     3.62     68.14    100.22
                 Het  df        3         7        11
                 Het  P      N.S.       ***       ***
               Fixed  RR     2.87      0.84      1.03
                     RRl     1.90      0.70      0.87
                     RRu     4.34      1.01      1.21
                      P       +++       (-)      N.S.
              Random  RR     2.79      2.35      2.37
                     RRl     1.73      0.90      1.15
                     RRu     4.50      6.18      4.86
                      P       +++       (+)         +
             Between Chi                        28.46
             Between  df                            1
             Between  P                           ***
             Btwn(F)  P                           (*)

                             Smoking product
                              any      cigs  cigsonly     Total


                       N                 10         2        12
                      NS                  8         2        10


                      Wt             132.15      6.79    138.94
                 Het Chi              96.81      3.39    100.22
                 Het  df                  9         1        11
                 Het  P                 ***       (*)       ***
               Fixed  RR               1.03      0.97      1.03
                     RRl               0.87      0.46      0.87
                     RRu               1.22      2.06      1.21
                      P                N.S.      N.S.      N.S.
              Random  RR               3.12      0.71      2.37
                     RRl               1.36      0.14      1.15
                     RRu               7.17      3.55      4.86
                      P                  ++      N.S.         +
             Between Chi                                   0.02
             Between  df                                      1
             Between  P                                    N.S.
             Btwn(F)  P                                    N.S.
  ________________________________________________________________________________________________________________________
                                            International Evidence on Smoking and COPD, Phase 3, Analysis run on 28-SEP-10

                                                   Table 3 - E - 10 - 7

                               IESCOPD - Meta-analysis of amount smoked : highest vs lowest
                          Any Emphysema, cigarettes (or any product if cigarettes not available)
                                 Excluded studies (and stage at which they were excluded)


1       CLARK COTTON  MEYER REMYJA RUTGER SNYDER SOBRAX     SU TAKEMU  WANG4   WEIR WHICKE ZALACA
2      ALDERS ALESSA  AMIGO ANDER1 ANDER3   BANG  BECK1  BECK2 BEDNAR BJORNS BROGGE  BROWN CERVER CHAPMA  CHEN1  CHEN2
        CHEN3  CHENG CLEMEN COATES  COCCI COLLEG  DEAN1  DEAN2  DEANE DEJONG DEMARC DETORR DICKIN  DOLL1  DOLL2 DONTA1
       DOPICO EHRLIC EKBERG ENSTRO FERRI1 FERRI2 FERRI3  FIDAN FINKLE FLETCH FORAST FOXMAN FUKUCH GEIJER GODTFR GOLDBE
       HAENSZ HARIKK HARRIS HAWTHO  HAYES HEDMAN HIGGI2 HIGGI3 HIGGI4 HIGGI6 HOLLA2 HOLLNA  HOUSE HRUBEC HUCHON HUHTI2
       HUHTI3 ITABAS JACOBS JAENDI JENSEN JINDA2 JOHANN  JOSHI JOUSI1 KACHEL KARAKA KATANC   KATO KHOURY    KIM  KIRAZ
       KLAYTO KOJIMA KOTAN1 KOTAN2 KRZYZA  KUBIK KULLER    LAI   LAM1   LAM2   LAM3 LAMBER  LANGE LANGE2 LANGHA    LEE
         LIAW LINDBE LINDST   LIU1   LIU2 LUNDB1 LUNDB2  MADOR MAGNUS MANFRE MANNI1 MANNI2 MANNI3 MARAN1 MARAN2 MARCUS
       MATHES MELLST MENEZ1 MENEZ2 MENEZ3 MENEZ4 MENEZ5 MENEZ6  MEREN  MILNE MOLLER MONTNE MUELLE NEJJAR NIEPSU NIHLEN
       NILSSO OGILVI OSWAL1 OSWAL2 PANDEY   PEAT PELKON PEREZP   PETO  PRICE   REID RENWIC RICCIO RIMING SARGEA SAWICK
       SCHWAR SHAHAB  SHARP SHIMUR   SHIN SICHLE SOBRAD SPEIZE STERLI STJERN  STROM SUADIC  TAGER TAGER2   TANG   THUN
         TODD TROISI TRUPIN TSUSHI TVERDA URRUTI VESTBO VIEGI1 VIEGI2 VINEIS VOLLM1 VOLLM2 VONHER WAGEN2   WALD WATSON
          WIG WILHEL WILSO1 WOJTYN  WOODS  WOOLF   XIAO     XU YAMAGU   YUAN ZIELI1 ZIELI2 ZIETKO   ZOIA
3        KAHN
4      ANDER2 ENRIGH GULSVI HARDIE     HO HOZAWA LEBOWI MILLER   NAWA  OMORI  PRATT  RYDER  SILVA SUTINE VIKGRE  WANG2
5      HIRAYA


  ________________________________________________________________________________________________________________________
                                            International Evidence on Smoking and COPD, Phase 3, Analysis run on 28-SEP-10

                                                   Table 3 - E - 10 - 8

                               IESCOPD - Meta-analysis of amount smoked : highest vs lowest
                          Any Emphysema, cigarettes (or any product if cigarettes not available)
                                             Potentially overlapping studies


     REF| REFGP|PRINC|                     OVERLAP|

  DONTA2 JACOBS     2  JACOBS/DONTA1/DONTA2/PELKON
  HUHTI1 HUHTI1     1                HUHTI1/HUHTI2
  HAMMO2 HAMMO2     1                HAMMO2/ENSTRO
     WEN    WEN     1                     WEN/LIAW
  WILSO2 WILSO2     1                WILSO1/WILSO2
   KAHN2   KAHN     2                   KAHN/KAHN2


  ________________________________________________________________________________________________________________________
                                            International Evidence on Smoking and COPD, Phase 3, Analysis run on 28-SEP-10

                                                    Table 3 - F - 4 -

                     IESCOPD - Meta-analysis of age started : highest (=youngest) vs lowest (=oldest)
                          Any Emphysema, cigarettes (or any product if cigarettes not available)


This analysis is restricted to results for:
1) Eligible study on database
2) Outcome Emphysema
3) Current or ever smoking
4) Categorical dose-response data for age started
5) Young vs old base
6) Results complete enough for use in meta-analysis

Within each study, results are then selected (in the following order of preference, within each sex) for:
7) SMKSTA  : current, ever
8) PROD    : cigarettes, cigarettes only, any product
9) The youngest vs oldest category
10) For overlapping studies: principal rather than subsidiary studies
and then for single sex results (m, f) in preference to results for both sexes combined (b).

Results adjusted for the most potential confounders are then chosen in Sections -1 to -3
and results adjusted for the least confounders in Sections -4 to -6. (Those least-adjusted results which
actually differ from the most-adjusted are marked 'x' in column X in Section -4)

Section -7 shows excluded studies, together with the stage (as above) at which no qualifying
results were found.

Section -8 lists the potentially overlapping studies which have been included (1=principal, 2=subsidiary),
and any results which would have been included in preference except that they had data not complete enough
for use in meta-analysis. It also lists their significance (yes/no), if known.


  ________________________________________________________________________________________________________________________
                                            International Evidence on Smoking and COPD, Phase 3, Analysis run on 28-SEP-10

                                                   Table 3 - F - 4 - 1

                     IESCOPD - Meta-analysis of age started : highest (=youngest) vs lowest (=oldest)
                          Any Emphysema, cigarettes (or any product if cigarettes not available)
                                                      Most-adjusted


     REF|NRR|SEX|AGEL|AGEH|     REGION|BEGYR|PUBYR|STTYP|ONSET|      DISEAS|ADJ|SMOKSTA|   PRODUCT|BASE-LO|LOW| HI|

  HIRAYA  15   m   40   99   Asia:FarE  1965  1982    Pr   Inc     Emp:mort   1 Current       Cigs      20   1  19
  HIRAYA  18   f   40   99   Asia:FarE  1965  1982    Pr   Inc     Emp:mort   1 Current       Cigs      20   1  19


  ________________________________________________________________________________________________________________________
                                            International Evidence on Smoking and COPD, Phase 3, Analysis run on 28-SEP-10

                                                   Table 3 - F - 4 - 2

                     IESCOPD - Meta-analysis of age started : highest (=youngest) vs lowest (=oldest)
                          Any Emphysema, cigarettes (or any product if cigarettes not available)
                                                      Most-adjusted


                        Number Exposed  Non-exposed
 REF    NRR SEX ADJ     Case    Cont    Case    Cont      RR        95.00%CI
*HIRAYA 15  m   1         18       -     115       -      1.14 (  0.69-   1.89)
*HIRAYA 18  f   1          0       -      10       -      1.28 (  0.08-  21.85)
 Subtotal HIRAYA                                          1.14 (  0.70-   1.88)
Partial Totals            18       0     125       0
*prospective study


 REF    NRR SEX ADJ             Ys       Ws       Qs       Ps
*HIRAYA 15  m   1              0.13    15.13     0.00       0.61
*HIRAYA 18  f   1              0.25     0.49     0.01       0.86
 Subtotal HIRAYA               0.13    15.62     0.01

                       N        2
                      NS        1


                      Wt    15.62
                 Het Chi     0.01
                 Het  df        1
                 Het  P      N.S.
               Fixed  RR     1.14
                     RRl     0.70
                     RRu     1.88
                      P      N.S.
              Random  RR     1.14
                     RRl     0.70
                     RRu     1.88
                      P      N.S.
               Asymm  P


  ________________________________________________________________________________________________________________________
                                            International Evidence on Smoking and COPD, Phase 3, Analysis run on 28-SEP-10

                                                   Table 3 - F - 4 - 3

                     IESCOPD - Meta-analysis of age started : highest (=youngest) vs lowest (=oldest)
                          Any Emphysema, cigarettes (or any product if cigarettes not available)
                                                      Most-adjusted


                       N        2
                      NS        1


                      Wt    15.62
                 Het Chi     0.01
                 Het  df        1
                 Het  P      N.S.
               Fixed  RR     1.14
                     RRl     0.70
                     RRu     1.88
                      P      N.S.
              Random  RR     1.14
                     RRl     0.70
                     RRu     1.88
                      P      N.S.
               Asymm  P

                                   Sex
                             both      male    female     Total


                       N                  1         1         2
                      NS                  1         1         1


                      Wt              15.13      0.49     15.62
                 Het Chi               0.00      0.00      0.01
                 Het  df                  0         0         1
                 Het  P                N.S.      N.S.      N.S.
               Fixed  RR               1.14      1.28      1.14
                     RRl               0.69      0.08      0.70
                     RRu               1.89     21.15      1.88
                      P                N.S.      N.S.      N.S.
              Random  RR               1.14      1.28      1.14
                     RRl               0.69      0.08      0.70
                     RRu               1.89     21.15      1.88
                      P                N.S.      N.S.      N.S.
             Between Chi                                   0.01
             Between  df                                      1
             Between  P                                    N.S.
             Btwn(F)  P                                    N.S.

                                        Continent
                            NAmer    Europe      Asia  oth/mult     Total


                       N                            2                   2
                      NS                            1                   1


                      Wt                        15.62               15.62
                 Het Chi                         0.01                0.01
                 Het  df                            1                   1
                 Het  P                          N.S.                N.S.
               Fixed  RR                         1.14                1.14
                     RRl                         0.70                0.70
                     RRu                         1.88                1.88
                      P                          N.S.                N.S.
              Random  RR                         1.14                1.14
                     RRl                         0.70                0.70
                     RRu                         1.88                1.88
                      P                          N.S.                N.S.
             Between Chi
             Between  df
             Between  P                                              N.S.
             Btwn(F)  P                                              N.S.


  ________________________________________________________________________________________________________________________
                                            International Evidence on Smoking and COPD, Phase 3, Analysis run on 28-SEP-10

                                                   Table 3 - F - 4 - 3

                     IESCOPD - Meta-analysis of age started : highest (=youngest) vs lowest (=oldest)
                          Any Emphysema, cigarettes (or any product if cigarettes not available)
                                                      Most-adjusted
                        National cigarette tobacco type (excluding mixed/unkown)
                          blended  virginia     Total


                       N        2                   2
                      NS        1                   1


                      Wt    15.62               15.62
                 Het Chi     0.01                0.01
                 Het  df        1                   1
                 Het  P      N.S.                N.S.
               Fixed  RR     1.14                1.14
                     RRl     0.70                0.70
                     RRu     1.88                1.88
                      P      N.S.                N.S.
              Random  RR     1.14                1.14
                     RRl     0.70                0.70
                     RRu     1.88                1.88
                      P      N.S.                N.S.
             Between Chi
             Between  df
             Between  P                          N.S.
             Btwn(F)  P                          N.S.

                                        Start year of study
                            <1970   1970-79   1980-89   1990-99     2000+   unknown     Total


                       N        2                                                           2
                      NS        1                                                           1


                      Wt    15.62                                                       15.62
                 Het Chi     0.01                                                        0.01
                 Het  df        1                                                           1
                 Het  P      N.S.                                                        N.S.
               Fixed  RR     1.14                                                        1.14
                     RRl     0.70                                                        0.70
                     RRu     1.88                                                        1.88
                      P      N.S.                                                        N.S.
              Random  RR     1.14                                                        1.14
                     RRl     0.70                                                        0.70
                     RRu     1.88                                                        1.88
                      P      N.S.                                                        N.S.
             Between Chi
             Between  df
             Between  P                                                                  N.S.
             Btwn(F)  P                                                                  N.S.

                                Publication year
                            <1980   1980-89   1990-99     2000+     Total


                       N                  2                             2
                      NS                  1                             1


                      Wt              15.62                         15.62
                 Het Chi               0.01                          0.01
                 Het  df                  1                             1
                 Het  P                N.S.                          N.S.
               Fixed  RR               1.14                          1.14
                     RRl               0.70                          0.70
                     RRu               1.88                          1.88
                      P                N.S.                          N.S.
              Random  RR               1.14                          1.14
                     RRl               0.70                          0.70
                     RRu               1.88                          1.88
                      P                N.S.                          N.S.
             Between Chi
             Between  df
             Between  P                                              N.S.
             Btwn(F)  P                                              N.S.
  ________________________________________________________________________________________________________________________
                                            International Evidence on Smoking and COPD, Phase 3, Analysis run on 28-SEP-10

                                                   Table 3 - F - 4 - 3

                     IESCOPD - Meta-analysis of age started : highest (=youngest) vs lowest (=oldest)
                          Any Emphysema, cigarettes (or any product if cigarettes not available)
                                                      Most-adjusted
                               Study type
                               CC        Pr        CS     Total


                       N                  2                   2
                      NS                  1                   1


                      Wt              15.62               15.62
                 Het Chi               0.01                0.01
                 Het  df                  1                   1
                 Het  P                N.S.                N.S.
               Fixed  RR               1.14                1.14
                     RRl               0.70                0.70
                     RRu               1.88                1.88
                      P                N.S.                N.S.
              Random  RR               1.14                1.14
                     RRl               0.70                0.70
                     RRu               1.88                1.88
                      P                N.S.                N.S.
             Between Chi
             Between  df
             Between  P                                    N.S.
             Btwn(F)  P                                    N.S.

                                    Lowest age in RR
                        <25/unlim     25-39       40+   unknown     Total


                       N                            2                   2
                      NS                            1                   1


                      Wt                        15.62               15.62
                 Het Chi                         0.01                0.01
                 Het  df                            1                   1
                 Het  P                          N.S.                N.S.
               Fixed  RR                         1.14                1.14
                     RRl                         0.70                0.70
                     RRu                         1.88                1.88
                      P                          N.S.                N.S.
              Random  RR                         1.14                1.14
                     RRl                         0.70                0.70
                     RRu                         1.88                1.88
                      P                          N.S.                N.S.
             Between Chi
             Between  df
             Between  P                                              N.S.
             Btwn(F)  P                                              N.S.

                                         Highest age in RR
                              <65     65-74     75-84 85+/unlim   unknown     Total


                       N                                      2                   2
                      NS                                      1                   1


                      Wt                                  15.62               15.62
                 Het Chi                                   0.01                0.01
                 Het  df                                      1                   1
                 Het  P                                    N.S.                N.S.
               Fixed  RR                                   1.14                1.14
                     RRl                                   0.70                0.70
                     RRu                                   1.88                1.88
                      P                                    N.S.                N.S.
              Random  RR                                   1.14                1.14
                     RRl                                   0.70                0.70
                     RRu                                   1.88                1.88
                      P                                    N.S.                N.S.
             Between Chi
             Between  df
             Between  P                                                        N.S.
             Btwn(F)  P                                                        N.S.
  ________________________________________________________________________________________________________________________
                                            International Evidence on Smoking and COPD, Phase 3, Analysis run on 28-SEP-10

                                                   Table 3 - F - 4 - 3

                     IESCOPD - Meta-analysis of age started : highest (=youngest) vs lowest (=oldest)
                          Any Emphysema, cigarettes (or any product if cigarettes not available)
                                                      Most-adjusted
                           Study weakness
                              Yes        No     Total


                       N                  2         2
                      NS                  1         1


                      Wt              15.62     15.62
                 Het Chi               0.01      0.01
                 Het  df                  1         1
                 Het  P                N.S.      N.S.
               Fixed  RR               1.14      1.14
                     RRl               0.70      0.70
                     RRu               1.88      1.88
                      P                N.S.      N.S.
              Random  RR               1.14      1.14
                     RRl               0.70      0.70
                     RRu               1.88      1.88
                      P                N.S.      N.S.
             Between Chi
             Between  df
             Between  P                          N.S.
             Btwn(F)  P                          N.S.

                           Type of outcome
                        prevalnce mortality incidence     Total


                       N                  2                   2
                      NS                  1                   1


                      Wt              15.62               15.62
                 Het Chi               0.01                0.01
                 Het  df                  1                   1
                 Het  P                N.S.                N.S.
               Fixed  RR               1.14                1.14
                     RRl               0.70                0.70
                     RRu               1.88                1.88
                      P                N.S.                N.S.
              Random  RR               1.14                1.14
                     RRl               0.70                0.70
                     RRu               1.88                1.88
                      P                N.S.                N.S.
             Between Chi
             Between  df
             Between  P                                    N.S.
             Btwn(F)  P                                    N.S.

                        Emp subtype
                             mort     other     Total


                       N        2                   2
                      NS        1                   1


                      Wt    15.62               15.62
                 Het Chi     0.01                0.01
                 Het  df        1                   1
                 Het  P      N.S.                N.S.
               Fixed  RR     1.14                1.14
                     RRl     0.70                0.70
                     RRu     1.88                1.88
                      P      N.S.                N.S.
              Random  RR     1.14                1.14
                     RRl     0.70                0.70
                     RRu     1.88                1.88
                      P      N.S.                N.S.
             Between Chi
             Between  df
             Between  P                          N.S.
             Btwn(F)  P                          N.S.
  ________________________________________________________________________________________________________________________
                                            International Evidence on Smoking and COPD, Phase 3, Analysis run on 28-SEP-10

                                                   Table 3 - F - 4 - 3

                     IESCOPD - Meta-analysis of age started : highest (=youngest) vs lowest (=oldest)
                          Any Emphysema, cigarettes (or any product if cigarettes not available)
                                                      Most-adjusted
                        Asthma analysis type (Emphysema)
                        inc-irres  excl-all     Total


                       N        2                   2
                      NS        1                   1


                      Wt    15.62               15.62
                 Het Chi     0.01                0.01
                 Het  df        1                   1
                 Het  P      N.S.                N.S.
               Fixed  RR     1.14                1.14
                     RRl     0.70                0.70
                     RRu     1.88                1.88
                      P      N.S.                N.S.
              Random  RR     1.14                1.14
                     RRl     0.70                0.70
                     RRu     1.88                1.88
                      P      N.S.                N.S.
             Between Chi
             Between  df
             Between  P                          N.S.
             Btwn(F)  P                          N.S.

                                Number of emphysema cases
                             1-50    51-100   101-200      201+     Total


                       N                                      2         2
                      NS                                      1         1


                      Wt                                  15.62     15.62
                 Het Chi                                   0.01      0.01
                 Het  df                                      1         1
                 Het  P                                    N.S.      N.S.
               Fixed  RR                                   1.14      1.14
                     RRl                                   0.70      0.70
                     RRu                                   1.88      1.88
                      P                                    N.S.      N.S.
              Random  RR                                   1.14      1.14
                     RRl                                   0.70      0.70
                     RRu                                   1.88      1.88
                      P                                    N.S.      N.S.
             Between Chi
             Between  df
             Between  P                                              N.S.
             Btwn(F)  P                                              N.S.

                        Number of emphysma cases (excluding unknown)


                       N                                      2         2
                      NS                                      1         1


                      Wt                                  15.62     15.62
                 Het Chi                                   0.01      0.01
                 Het  df                                      1         1
                 Het  P                                    N.S.      N.S.
               Fixed  RR                                   1.14      1.14
                     RRl                                   0.70      0.70
                     RRu                                   1.88      1.88
                      P                                    N.S.      N.S.
              Random  RR                                   1.14      1.14
                     RRl                                   0.70      0.70
                     RRu                                   1.88      1.88
                      P                                    N.S.      N.S.
             Between Chi
             Between  df
             Between  P                                              N.S.
             Btwn(F)  P                                              N.S.

  ________________________________________________________________________________________________________________________
                                            International Evidence on Smoking and COPD, Phase 3, Analysis run on 28-SEP-10

                                                   Table 3 - F - 4 - 3

                     IESCOPD - Meta-analysis of age started : highest (=youngest) vs lowest (=oldest)
                          Any Emphysema, cigarettes (or any product if cigarettes not available)
                                                      Most-adjusted
                            Analysis type
                         prevlnce     onset     Total


                       N                  2         2
                      NS                  1         1


                      Wt              15.62     15.62
                 Het Chi               0.01      0.01
                 Het  df                  1         1
                 Het  P                N.S.      N.S.
               Fixed  RR               1.14      1.14
                     RRl               0.70      0.70
                     RRu               1.88      1.88
                      P                N.S.      N.S.
              Random  RR               1.14      1.14
                     RRl               0.70      0.70
                     RRu               1.88      1.88
                      P                N.S.      N.S.
             Between Chi
             Between  df
             Between  P                          N.S.
             Btwn(F)  P                          N.S.

                             Smoking product
                              any      cigs  cigsonly     Total


                       N                  2                   2
                      NS                  1                   1


                      Wt              15.62               15.62
                 Het Chi               0.01                0.01
                 Het  df                  1                   1
                 Het  P                N.S.                N.S.
               Fixed  RR               1.14                1.14
                     RRl               0.70                0.70
                     RRu               1.88                1.88
                      P                N.S.                N.S.
              Random  RR               1.14                1.14
                     RRl               0.70                0.70
                     RRu               1.88                1.88
                      P                N.S.                N.S.
             Between Chi
             Between  df
             Between  P                                    N.S.
             Btwn(F)  P                                    N.S.

                        Number of adjustment variables
                                0         1        2+     Total


                       N                  2                   2
                      NS                  1                   1


                      Wt              15.62               15.62
                 Het Chi               0.01                0.01
                 Het  df                  1                   1
                 Het  P                N.S.                N.S.
               Fixed  RR               1.14                1.14
                     RRl               0.70                0.70
                     RRu               1.88                1.88
                      P                N.S.                N.S.
              Random  RR               1.14                1.14
                     RRl               0.70                0.70
                     RRu               1.88                1.88
                      P                N.S.                N.S.
             Between Chi
             Between  df
             Between  P                                    N.S.
             Btwn(F)  P                                    N.S.
  ________________________________________________________________________________________________________________________
                                            International Evidence on Smoking and COPD, Phase 3, Analysis run on 28-SEP-10

                                                   Table 3 - F - 4 - 4

                     IESCOPD - Meta-analysis of age started : highest (=youngest) vs lowest (=oldest)
                          Any Emphysema, cigarettes (or any product if cigarettes not available)
                                                      Least-adjusted


     REF|NRR|X|SEX|AGEL|AGEH|     REGION|BEGYR|PUBYR|STTYP|ONSET|      DISEAS|ADJ|SMOKSTA|   PRODUCT|BASE-LO|LOW| HI|

  HIRAYA  15     m   40   99   Asia:FarE  1965  1982    Pr   Inc     Emp:mort   1 Current       Cigs      20   1  19
  HIRAYA  18     f   40   99   Asia:FarE  1965  1982    Pr   Inc     Emp:mort   1 Current       Cigs      20   1  19


  ________________________________________________________________________________________________________________________
                                            International Evidence on Smoking and COPD, Phase 3, Analysis run on 28-SEP-10

                                                   Table 3 - F - 4 - 5

                     IESCOPD - Meta-analysis of age started : highest (=youngest) vs lowest (=oldest)
                          Any Emphysema, cigarettes (or any product if cigarettes not available)
                                                      Least-adjusted


                        Number Exposed  Non-exposed
 REF    NRR SEX ADJ     Case    Cont    Case    Cont      RR        95.00%CI
*HIRAYA 15  m   1         18       -     115       -      1.14 (  0.69-   1.89)
*HIRAYA 18  f   1          0       -      10       -      1.28 (  0.08-  21.85)
 Subtotal HIRAYA                                          1.14 (  0.70-   1.88)
Partial Totals            18       0     125       0
*prospective study


 REF    NRR SEX ADJ             Ys       Ws       Qs       Ps
*HIRAYA 15  m   1              0.13    15.13     0.00       0.61
*HIRAYA 18  f   1              0.25     0.49     0.01       0.86
 Subtotal HIRAYA               0.13    15.62     0.01

                       N        2
                      NS        1


                      Wt    15.62
                 Het Chi     0.01
                 Het  df        1
                 Het  P      N.S.
               Fixed  RR     1.14
                     RRl     0.70
                     RRu     1.88
                      P      N.S.
              Random  RR     1.14
                     RRl     0.70
                     RRu     1.88
                      P      N.S.
               Asymm  P


  ________________________________________________________________________________________________________________________
                                            International Evidence on Smoking and COPD, Phase 3, Analysis run on 28-SEP-10

                                                   Table 3 - F - 4 - 6

                     IESCOPD - Meta-analysis of age started : highest (=youngest) vs lowest (=oldest)
                          Any Emphysema, cigarettes (or any product if cigarettes not available)
                                                      Least-adjusted


                       N        2
                      NS        1


                      Wt    15.62
                 Het Chi     0.01
                 Het  df        1
                 Het  P      N.S.
               Fixed  RR     1.14
                     RRl     0.70
                     RRu     1.88
                      P      N.S.
              Random  RR     1.14
                     RRl     0.70
                     RRu     1.88
                      P      N.S.
               Asymm  P

                                   Sex
                             both      male    female     Total


                       N                  1         1         2
                      NS                  1         1         1


                      Wt              15.13      0.49     15.62
                 Het Chi               0.00      0.00      0.01
                 Het  df                  0         0         1
                 Het  P                N.S.      N.S.      N.S.
               Fixed  RR               1.14      1.28      1.14
                     RRl               0.69      0.08      0.70
                     RRu               1.89     21.15      1.88
                      P                N.S.      N.S.      N.S.
              Random  RR               1.14      1.28      1.14
                     RRl               0.69      0.08      0.70
                     RRu               1.89     21.15      1.88
                      P                N.S.      N.S.      N.S.
             Between Chi                                   0.01
             Between  df                                      1
             Between  P                                    N.S.
             Btwn(F)  P                                    N.S.

                                        Continent
                            NAmer    Europe      Asia  oth/mult     Total


                       N                            2                   2
                      NS                            1                   1


                      Wt                        15.62               15.62
                 Het Chi                         0.01                0.01
                 Het  df                            1                   1
                 Het  P                          N.S.                N.S.
               Fixed  RR                         1.14                1.14
                     RRl                         0.70                0.70
                     RRu                         1.88                1.88
                      P                          N.S.                N.S.
              Random  RR                         1.14                1.14
                     RRl                         0.70                0.70
                     RRu                         1.88                1.88
                      P                          N.S.                N.S.
             Between Chi
             Between  df
             Between  P                                              N.S.
             Btwn(F)  P                                              N.S.


  ________________________________________________________________________________________________________________________
                                            International Evidence on Smoking and COPD, Phase 3, Analysis run on 28-SEP-10

                                                   Table 3 - F - 4 - 6

                     IESCOPD - Meta-analysis of age started : highest (=youngest) vs lowest (=oldest)
                          Any Emphysema, cigarettes (or any product if cigarettes not available)
                                                      Least-adjusted
                               Study type
                               CC        Pr        CS     Total


                       N                  2                   2
                      NS                  1                   1


                      Wt              15.62               15.62
                 Het Chi               0.01                0.01
                 Het  df                  1                   1
                 Het  P                N.S.                N.S.
               Fixed  RR               1.14                1.14
                     RRl               0.70                0.70
                     RRu               1.88                1.88
                      P                N.S.                N.S.
              Random  RR               1.14                1.14
                     RRl               0.70                0.70
                     RRu               1.88                1.88
                      P                N.S.                N.S.
             Between Chi
             Between  df
             Between  P                                    N.S.
             Btwn(F)  P                                    N.S.

                        Emp subtype
                             mort     other     Total


                       N        2                   2
                      NS        1                   1


                      Wt    15.62               15.62
                 Het Chi     0.01                0.01
                 Het  df        1                   1
                 Het  P      N.S.                N.S.
               Fixed  RR     1.14                1.14
                     RRl     0.70                0.70
                     RRu     1.88                1.88
                      P      N.S.                N.S.
              Random  RR     1.14                1.14
                     RRl     0.70                0.70
                     RRu     1.88                1.88
                      P      N.S.                N.S.
             Between Chi
             Between  df
             Between  P                          N.S.
             Btwn(F)  P                          N.S.

                             Smoking product
                              any      cigs  cigsonly     Total


                       N                  2                   2
                      NS                  1                   1


                      Wt              15.62               15.62
                 Het Chi               0.01                0.01
                 Het  df                  1                   1
                 Het  P                N.S.                N.S.
               Fixed  RR               1.14                1.14
                     RRl               0.70                0.70
                     RRu               1.88                1.88
                      P                N.S.                N.S.
              Random  RR               1.14                1.14
                     RRl               0.70                0.70
                     RRu               1.88                1.88
                      P                N.S.                N.S.
             Between Chi
             Between  df
             Between  P                                    N.S.
             Btwn(F)  P                                    N.S.
  ________________________________________________________________________________________________________________________
                                            International Evidence on Smoking and COPD, Phase 3, Analysis run on 28-SEP-10

                                                   Table 3 - F - 4 - 7

                     IESCOPD - Meta-analysis of age started : highest (=youngest) vs lowest (=oldest)
                          Any Emphysema, cigarettes (or any product if cigarettes not available)
                                 Excluded studies (and stage at which they were excluded)


1       CLARK COTTON  MEYER REMYJA RUTGER SNYDER SOBRAX     SU TAKEMU  WANG4   WEIR WHICKE ZALACA
2      ALDERS ALESSA  AMIGO ANDER1 ANDER3   BANG  BECK1  BECK2 BEDNAR BJORNS BROGGE  BROWN CERVER CHAPMA  CHEN1  CHEN2
        CHEN3  CHENG CLEMEN COATES  COCCI COLLEG  DEAN1  DEAN2  DEANE DEJONG DEMARC DETORR DICKIN  DOLL1  DOLL2 DONTA1
       DOPICO EHRLIC EKBERG ENSTRO FERRI1 FERRI2 FERRI3  FIDAN FINKLE FLETCH FORAST FOXMAN FUKUCH GEIJER GODTFR GOLDBE
       HAENSZ HARIKK HARRIS HAWTHO  HAYES HEDMAN HIGGI2 HIGGI3 HIGGI4 HIGGI6 HOLLA2 HOLLNA  HOUSE HRUBEC HUCHON HUHTI2
       HUHTI3 ITABAS JACOBS JAENDI JENSEN JINDA2 JOHANN  JOSHI JOUSI1 KACHEL KARAKA KATANC   KATO KHOURY    KIM  KIRAZ
       KLAYTO KOJIMA KOTAN1 KOTAN2 KRZYZA  KUBIK KULLER    LAI   LAM1   LAM2   LAM3 LAMBER  LANGE LANGE2 LANGHA    LEE
         LIAW LINDBE LINDST   LIU1   LIU2 LUNDB1 LUNDB2  MADOR MAGNUS MANFRE MANNI1 MANNI2 MANNI3 MARAN1 MARAN2 MARCUS
       MATHES MELLST MENEZ1 MENEZ2 MENEZ3 MENEZ4 MENEZ5 MENEZ6  MEREN  MILNE MOLLER MONTNE MUELLE NEJJAR NIEPSU NIHLEN
       NILSSO OGILVI OSWAL1 OSWAL2 PANDEY   PEAT PELKON PEREZP   PETO  PRICE   REID RENWIC RICCIO RIMING SARGEA SAWICK
       SCHWAR SHAHAB  SHARP SHIMUR   SHIN SICHLE SOBRAD SPEIZE STERLI STJERN  STROM SUADIC  TAGER TAGER2   TANG   THUN
         TODD TROISI TRUPIN TSUSHI TVERDA URRUTI VESTBO VIEGI1 VIEGI2 VINEIS VOLLM1 VOLLM2 VONHER WAGEN2   WALD WATSON
          WIG WILHEL WILSO1 WOJTYN  WOODS  WOOLF   XIAO     XU YAMAGU   YUAN ZIELI1 ZIELI2 ZIETKO   ZOIA
3        KAHN
4      ANDER2 AUERBA   BEST DONTA2 ENRIGH GULSVI HAMMO2 HARDIE     HO HOZAWA HUHTI1  KAHN2 LAVECC LEBOWI MILLER   NAWA
        OMORI  PRATT  RYDER  SILVA SUTINE VIKGRE  WANG2  WEISS    WEN WILSO2


  ________________________________________________________________________________________________________________________
                                            International Evidence on Smoking and COPD, Phase 3, Analysis run on 28-SEP-10

                                                    Table 3 - G - 9 -

                                IESCOPD - Meta-analysis of pack-years : highest vs lowest
                          Any Emphysema, cigarettes (or any product if cigarettes not available)


This analysis is restricted to results for:
1) Eligible study on database
2) Outcome Emphysema
3) Current or ever smoking
4) Categorical dose-response data for pack-years
5) High vs low base
6) Results complete enough for use in meta-analysis

Within each study, results are then selected (in the following order of preference, within each sex) for:
7) SMKSTA  : current, ever
8) PROD    : cigarettes, cigarettes only, any product
9) The highest vs lowest category
10) For overlapping studies: principal rather than subsidiary studies
and then for single sex results (m, f) in preference to results for both sexes combined (b).

Results adjusted for the most potential confounders are then chosen in Sections -1 to -3
and results adjusted for the least confounders in Sections -4 to -6. (Those least-adjusted results which
actually differ from the most-adjusted are marked 'x' in column X in Section -4)

Section -7 shows excluded studies, together with the stage (as above) at which no qualifying
results were found.

Section -8 lists the potentially overlapping studies which have been included (1=principal, 2=subsidiary),
and any results which would have been included in preference except that they had data not complete enough
for use in meta-analysis. It also lists their significance (yes/no), if known.


  ________________________________________________________________________________________________________________________
                                            International Evidence on Smoking and COPD, Phase 3, Analysis run on 28-SEP-10

                                                   Table 3 - G - 9 - 1

                                IESCOPD - Meta-analysis of pack-years : highest vs lowest
                          Any Emphysema, cigarettes (or any product if cigarettes not available)
                                                      Most-adjusted


     REF|NRR|SEX|AGEL|AGEH|     REGION|BEGYR|PUBYR|STTYP|ONSET|      DISEAS|ADJ|SMOKSTA|   PRODUCT|BASE-HI|LOW| HI|

  HIRAYA   7   m   40   99   Asia:FarE  1965  1982    Pr   Inc     Emp:mort   1 Current       Cigs      13  28   +
  HIRAYA  12   f   40   99   Asia:FarE  1965  1982    Pr   Inc     Emp:mort   1 Current       Cigs      13  28   +
   WANG2   4   b   19   92   Asia:FarE  1996  2001    CS  Prev  Emp:viscomp   0    Ever       Cigs      30  31   +


  ________________________________________________________________________________________________________________________
                                            International Evidence on Smoking and COPD, Phase 3, Analysis run on 28-SEP-10

                                                   Table 3 - G - 9 - 2

                                IESCOPD - Meta-analysis of pack-years : highest vs lowest
                          Any Emphysema, cigarettes (or any product if cigarettes not available)
                                                      Most-adjusted


                        Number Exposed  Non-exposed
 REF    NRR SEX ADJ     Case    Cont    Case    Cont      RR        95.00%CI
*HIRAYA 7   m   1         29       -       4       -      3.78 (  1.34-  10.70)
*HIRAYA 12  f   1          2       -       6       -      4.79 (  1.02-  22.60)
 Subtotal HIRAYA                                          4.07 (  1.72-   9.64)
 WANG2  4   b   0        113    1603      75    1805      1.70 (  1.26-   2.29)
Partial Totals           144    1603      85    1805
*prospective study


 REF    NRR SEX ADJ             Ys       Ws       Qs       Ps
*HIRAYA 7   m   1              1.33     3.56     1.78       0.01
*HIRAYA 12  f   1              1.57     1.60     1.43       0.05
 Subtotal HIRAYA               1.40     5.16     3.21
 WANG2  4   b   0              0.53    42.81     0.38       0.00

                       N        3
                      NS        2


                      Wt    47.97
                 Het Chi     3.58
                 Het  df        2
                 Het  P      N.S.
               Fixed  RR     1.86
                     RRl     1.40
                     RRu     2.47
                      P       +++
              Random  RR     2.42
                     RRl     1.25
                     RRu     4.70
                      P        ++
               Asymm  P       (*)


  ________________________________________________________________________________________________________________________
                                            International Evidence on Smoking and COPD, Phase 3, Analysis run on 28-SEP-10

                                                   Table 3 - G - 9 - 3

                                IESCOPD - Meta-analysis of pack-years : highest vs lowest
                          Any Emphysema, cigarettes (or any product if cigarettes not available)
                                                      Most-adjusted


                       N        3
                      NS        2


                      Wt    47.97
                 Het Chi     3.58
                 Het  df        2
                 Het  P      N.S.
               Fixed  RR     1.86
                     RRl     1.40
                     RRu     2.47
                      P       +++
              Random  RR     2.42
                     RRl     1.25
                     RRu     4.70
                      P        ++
               Asymm  P       (*)

                                   Sex
                             both      male    female     Total


                       N        1         1         1         3
                      NS        1         1         1         2


                      Wt    42.81      3.56      1.60     47.97
                 Het Chi     0.00      0.00      0.00      3.58
                 Het  df        0         0         0         2
                 Het  P      N.S.      N.S.      N.S.      N.S.
               Fixed  RR     1.70      3.78      4.79      1.86
                     RRl     1.26      1.34      1.02      1.40
                     RRu     2.29     10.68     22.55      2.47
                      P       +++         +         +       +++
              Random  RR     1.70      3.78      4.79      2.42
                     RRl     1.26      1.34      1.02      1.25
                     RRu     2.29     10.68     22.55      4.70
                      P       +++         +         +        ++
             Between Chi                                   3.58
             Between  df                                      2
             Between  P                                    N.S.
             Btwn(F)  P                                    N.S.

                                        Continent
                            NAmer    Europe      Asia  oth/mult     Total


                       N                            3                   3
                      NS                            2                   2


                      Wt                        47.97               47.97
                 Het Chi                         3.58                3.58
                 Het  df                            2                   2
                 Het  P                          N.S.                N.S.
               Fixed  RR                         1.86                1.86
                     RRl                         1.40                1.40
                     RRu                         2.47                2.47
                      P                           +++                 +++
              Random  RR                         2.42                2.42
                     RRl                         1.25                1.25
                     RRu                         4.70                4.70
                      P                            ++                  ++
             Between Chi
             Between  df
             Between  P                                              N.S.
             Btwn(F)  P                                              N.S.


  ________________________________________________________________________________________________________________________
                                            International Evidence on Smoking and COPD, Phase 3, Analysis run on 28-SEP-10

                                                   Table 3 - G - 9 - 3

                                IESCOPD - Meta-analysis of pack-years : highest vs lowest
                          Any Emphysema, cigarettes (or any product if cigarettes not available)
                                                      Most-adjusted
                        National cigarette tobacco type (excluding mixed/unkown)
                          blended  virginia     Total


                       N        3                   3
                      NS        2                   2


                      Wt    47.97               47.97
                 Het Chi     3.58                3.58
                 Het  df        2                   2
                 Het  P      N.S.                N.S.
               Fixed  RR     1.86                1.86
                     RRl     1.40                1.40
                     RRu     2.47                2.47
                      P       +++                 +++
              Random  RR     2.42                2.42
                     RRl     1.25                1.25
                     RRu     4.70                4.70
                      P        ++                  ++
             Between Chi
             Between  df
             Between  P                          N.S.
             Btwn(F)  P                          N.S.

                                        Start year of study
                            <1970   1970-79   1980-89   1990-99     2000+   unknown     Total


                       N        2                             1                             3
                      NS        1                             1                             2


                      Wt     5.16                         42.81                         47.97
                 Het Chi     0.06                          0.00                          3.58
                 Het  df        1                             0                             2
                 Het  P      N.S.                          N.S.                          N.S.
               Fixed  RR     4.07                          1.70                          1.86
                     RRl     1.72                          1.26                          1.40
                     RRu     9.64                          2.29                          2.47
                      P        ++                           +++                           +++
              Random  RR     4.07                          1.70                          2.42
                     RRl     1.72                          1.26                          1.25
                     RRu     9.64                          2.29                          4.70
                      P        ++                           +++                            ++
             Between Chi                                                                 3.52
             Between  df                                                                    1
             Between  P                                                                   (*)
             Btwn(F)  P                                                                   (*)

                                Publication year
                            <1980   1980-89   1990-99     2000+     Total


                       N                  2                   1         3
                      NS                  1                   1         2


                      Wt               5.16               42.81     47.97
                 Het Chi               0.06                0.00      3.58
                 Het  df                  1                   0         2
                 Het  P                N.S.                N.S.      N.S.
               Fixed  RR               4.07                1.70      1.86
                     RRl               1.72                1.26      1.40
                     RRu               9.64                2.29      2.47
                      P                  ++                 +++       +++
              Random  RR               4.07                1.70      2.42
                     RRl               1.72                1.26      1.25
                     RRu               9.64                2.29      4.70
                      P                  ++                 +++        ++
             Between Chi                                             3.52
             Between  df                                                1
             Between  P                                               (*)
             Btwn(F)  P                                               (*)
  ________________________________________________________________________________________________________________________
                                            International Evidence on Smoking and COPD, Phase 3, Analysis run on 28-SEP-10

                                                   Table 3 - G - 9 - 3

                                IESCOPD - Meta-analysis of pack-years : highest vs lowest
                          Any Emphysema, cigarettes (or any product if cigarettes not available)
                                                      Most-adjusted
                               Study type
                               CC        Pr        CS     Total


                       N                  2         1         3
                      NS                  1         1         2


                      Wt               5.16     42.81     47.97
                 Het Chi               0.06      0.00      3.58
                 Het  df                  1         0         2
                 Het  P                N.S.      N.S.      N.S.
               Fixed  RR               4.07      1.70      1.86
                     RRl               1.72      1.26      1.40
                     RRu               9.64      2.29      2.47
                      P                  ++       +++       +++
              Random  RR               4.07      1.70      2.42
                     RRl               1.72      1.26      1.25
                     RRu               9.64      2.29      4.70
                      P                  ++       +++        ++
             Between Chi                                   3.52
             Between  df                                      1
             Between  P                                     (*)
             Btwn(F)  P                                     (*)

                                    Lowest age in RR
                        <25/unlim     25-39       40+   unknown     Total


                       N        1                   2                   3
                      NS        1                   1                   2


                      Wt    42.81                5.16               47.97
                 Het Chi     0.00                0.06                3.58
                 Het  df        0                   1                   2
                 Het  P      N.S.                N.S.                N.S.
               Fixed  RR     1.70                4.07                1.86
                     RRl     1.26                1.72                1.40
                     RRu     2.29                9.64                2.47
                      P       +++                  ++                 +++
              Random  RR     1.70                4.07                2.42
                     RRl     1.26                1.72                1.25
                     RRu     2.29                9.64                4.70
                      P       +++                  ++                  ++
             Between Chi                                             3.52
             Between  df                                                1
             Between  P                                               (*)
             Btwn(F)  P                                               (*)

                                         Highest age in RR
                              <65     65-74     75-84 85+/unlim   unknown     Total


                       N                                      3                   3
                      NS                                      2                   2


                      Wt                                  47.97               47.97
                 Het Chi                                   3.58                3.58
                 Het  df                                      2                   2
                 Het  P                                    N.S.                N.S.
               Fixed  RR                                   1.86                1.86
                     RRl                                   1.40                1.40
                     RRu                                   2.47                2.47
                      P                                     +++                 +++
              Random  RR                                   2.42                2.42
                     RRl                                   1.25                1.25
                     RRu                                   4.70                4.70
                      P                                      ++                  ++
             Between Chi
             Between  df
             Between  P                                                        N.S.
             Btwn(F)  P                                                        N.S.
  ________________________________________________________________________________________________________________________
                                            International Evidence on Smoking and COPD, Phase 3, Analysis run on 28-SEP-10

                                                   Table 3 - G - 9 - 3

                                IESCOPD - Meta-analysis of pack-years : highest vs lowest
                          Any Emphysema, cigarettes (or any product if cigarettes not available)
                                                      Most-adjusted
                           Study weakness
                              Yes        No     Total


                       N                  3         3
                      NS                  2         2


                      Wt              47.97     47.97
                 Het Chi               3.58      3.58
                 Het  df                  2         2
                 Het  P                N.S.      N.S.
               Fixed  RR               1.86      1.86
                     RRl               1.40      1.40
                     RRu               2.47      2.47
                      P                 +++       +++
              Random  RR               2.42      2.42
                     RRl               1.25      1.25
                     RRu               4.70      4.70
                      P                  ++        ++
             Between Chi
             Between  df
             Between  P                          N.S.
             Btwn(F)  P                          N.S.

                           Type of outcome
                        prevalnce mortality incidence     Total


                       N        1         2                   3
                      NS        1         1                   2


                      Wt    42.81      5.16               47.97
                 Het Chi     0.00      0.06                3.58
                 Het  df        0         1                   2
                 Het  P      N.S.      N.S.                N.S.
               Fixed  RR     1.70      4.07                1.86
                     RRl     1.26      1.72                1.40
                     RRu     2.29      9.64                2.47
                      P       +++        ++                 +++
              Random  RR     1.70      4.07                2.42
                     RRl     1.26      1.72                1.25
                     RRu     2.29      9.64                4.70
                      P       +++        ++                  ++
             Between Chi                                   3.52
             Between  df                                      1
             Between  P                                     (*)
             Btwn(F)  P                                     (*)

                        Emp subtype
                             mort     other     Total


                       N        2         1         3
                      NS        1         1         2


                      Wt     5.16     42.81     47.97
                 Het Chi     0.06      0.00      3.58
                 Het  df        1         0         2
                 Het  P      N.S.      N.S.      N.S.
               Fixed  RR     4.07      1.70      1.86
                     RRl     1.72      1.26      1.40
                     RRu     9.64      2.29      2.47
                      P        ++       +++       +++
              Random  RR     4.07      1.70      2.42
                     RRl     1.72      1.26      1.25
                     RRu     9.64      2.29      4.70
                      P        ++       +++        ++
             Between Chi                         3.52
             Between  df                            1
             Between  P                           (*)
             Btwn(F)  P                           (*)
  ________________________________________________________________________________________________________________________
                                            International Evidence on Smoking and COPD, Phase 3, Analysis run on 28-SEP-10

                                                   Table 3 - G - 9 - 3

                                IESCOPD - Meta-analysis of pack-years : highest vs lowest
                          Any Emphysema, cigarettes (or any product if cigarettes not available)
                                                      Most-adjusted
                        Asthma analysis type (Emphysema)
                        inc-irres  excl-all     Total


                       N        3                   3
                      NS        2                   2


                      Wt    47.97               47.97
                 Het Chi     3.58                3.58
                 Het  df        2                   2
                 Het  P      N.S.                N.S.
               Fixed  RR     1.86                1.86
                     RRl     1.40                1.40
                     RRu     2.47                2.47
                      P       +++                 +++
              Random  RR     2.42                2.42
                     RRl     1.25                1.25
                     RRu     4.70                4.70
                      P        ++                  ++
             Between Chi
             Between  df
             Between  P                          N.S.
             Btwn(F)  P                          N.S.

                                Number of emphysema cases
                             1-50    51-100   101-200      201+     Total


                       N                                      3         3
                      NS                                      2         2


                      Wt                                  47.97     47.97
                 Het Chi                                   3.58      3.58
                 Het  df                                      2         2
                 Het  P                                    N.S.      N.S.
               Fixed  RR                                   1.86      1.86
                     RRl                                   1.40      1.40
                     RRu                                   2.47      2.47
                      P                                     +++       +++
              Random  RR                                   2.42      2.42
                     RRl                                   1.25      1.25
                     RRu                                   4.70      4.70
                      P                                      ++        ++
             Between Chi
             Between  df
             Between  P                                              N.S.
             Btwn(F)  P                                              N.S.

                        Number of emphysma cases (excluding unknown)


                       N                                      3         3
                      NS                                      2         2


                      Wt                                  47.97     47.97
                 Het Chi                                   3.58      3.58
                 Het  df                                      2         2
                 Het  P                                    N.S.      N.S.
               Fixed  RR                                   1.86      1.86
                     RRl                                   1.40      1.40
                     RRu                                   2.47      2.47
                      P                                     +++       +++
              Random  RR                                   2.42      2.42
                     RRl                                   1.25      1.25
                     RRu                                   4.70      4.70
                      P                                      ++        ++
             Between Chi
             Between  df
             Between  P                                              N.S.
             Btwn(F)  P                                              N.S.

  ________________________________________________________________________________________________________________________
                                            International Evidence on Smoking and COPD, Phase 3, Analysis run on 28-SEP-10

                                                   Table 3 - G - 9 - 3

                                IESCOPD - Meta-analysis of pack-years : highest vs lowest
                          Any Emphysema, cigarettes (or any product if cigarettes not available)
                                                      Most-adjusted
                            Analysis type
                         prevlnce     onset     Total


                       N        1         2         3
                      NS        1         1         2


                      Wt    42.81      5.16     47.97
                 Het Chi     0.00      0.06      3.58
                 Het  df        0         1         2
                 Het  P      N.S.      N.S.      N.S.
               Fixed  RR     1.70      4.07      1.86
                     RRl     1.26      1.72      1.40
                     RRu     2.29      9.64      2.47
                      P       +++        ++       +++
              Random  RR     1.70      4.07      2.42
                     RRl     1.26      1.72      1.25
                     RRu     2.29      9.64      4.70
                      P       +++        ++        ++
             Between Chi                         3.52
             Between  df                            1
             Between  P                           (*)
             Btwn(F)  P                           (*)

                             Smoking product
                              any      cigs  cigsonly     Total


                       N                  3                   3
                      NS                  2                   2


                      Wt              47.97               47.97
                 Het Chi               3.58                3.58
                 Het  df                  2                   2
                 Het  P                N.S.                N.S.
               Fixed  RR               1.86                1.86
                     RRl               1.40                1.40
                     RRu               2.47                2.47
                      P                 +++                 +++
              Random  RR               2.42                2.42
                     RRl               1.25                1.25
                     RRu               4.70                4.70
                      P                  ++                  ++
             Between Chi
             Between  df
             Between  P                                    N.S.
             Btwn(F)  P                                    N.S.

                        Number of adjustment variables
                                0         1        2+     Total


                       N        1         2                   3
                      NS        1         1                   2


                      Wt    42.81      5.16               47.97
                 Het Chi     0.00      0.06                3.58
                 Het  df        0         1                   2
                 Het  P      N.S.      N.S.                N.S.
               Fixed  RR     1.70      4.07                1.86
                     RRl     1.26      1.72                1.40
                     RRu     2.29      9.64                2.47
                      P       +++        ++                 +++
              Random  RR     1.70      4.07                2.42
                     RRl     1.26      1.72                1.25
                     RRu     2.29      9.64                4.70
                      P       +++        ++                  ++
             Between Chi                                   3.52
             Between  df                                      1
             Between  P                                     (*)
             Btwn(F)  P                                     (*)
  ________________________________________________________________________________________________________________________
                                            International Evidence on Smoking and COPD, Phase 3, Analysis run on 28-SEP-10

                                                   Table 3 - G - 9 - 4

                                IESCOPD - Meta-analysis of pack-years : highest vs lowest
                          Any Emphysema, cigarettes (or any product if cigarettes not available)
                                                      Least-adjusted


     REF|NRR|X|SEX|AGEL|AGEH|     REGION|BEGYR|PUBYR|STTYP|ONSET|      DISEAS|ADJ|SMOKSTA|   PRODUCT|BASE-HI|LOW| HI|

  HIRAYA   7     m   40   99   Asia:FarE  1965  1982    Pr   Inc     Emp:mort   1 Current       Cigs      13  28   +
  HIRAYA  12     f   40   99   Asia:FarE  1965  1982    Pr   Inc     Emp:mort   1 Current       Cigs      13  28   +
   WANG2   4     b   19   92   Asia:FarE  1996  2001    CS  Prev  Emp:viscomp   0    Ever       Cigs      30  31   +


  ________________________________________________________________________________________________________________________
                                            International Evidence on Smoking and COPD, Phase 3, Analysis run on 28-SEP-10

                                                   Table 3 - G - 9 - 5

                                IESCOPD - Meta-analysis of pack-years : highest vs lowest
                          Any Emphysema, cigarettes (or any product if cigarettes not available)
                                                      Least-adjusted


                        Number Exposed  Non-exposed
 REF    NRR SEX ADJ     Case    Cont    Case    Cont      RR        95.00%CI
*HIRAYA 7   m   1         29       -       4       -      3.78 (  1.34-  10.70)
*HIRAYA 12  f   1          2       -       6       -      4.79 (  1.02-  22.60)
 Subtotal HIRAYA                                          4.07 (  1.72-   9.64)
 WANG2  4   b   0        113    1603      75    1805      1.70 (  1.26-   2.29)
Partial Totals           144    1603      85    1805
*prospective study


 REF    NRR SEX ADJ             Ys       Ws       Qs       Ps
*HIRAYA 7   m   1              1.33     3.56     1.78       0.01
*HIRAYA 12  f   1              1.57     1.60     1.43       0.05
 Subtotal HIRAYA               1.40     5.16     3.21
 WANG2  4   b   0              0.53    42.81     0.38       0.00

                       N        3
                      NS        2


                      Wt    47.97
                 Het Chi     3.58
                 Het  df        2
                 Het  P      N.S.
               Fixed  RR     1.86
                     RRl     1.40
                     RRu     2.47
                      P       +++
              Random  RR     2.42
                     RRl     1.25
                     RRu     4.70
                      P        ++
               Asymm  P       (*)


  ________________________________________________________________________________________________________________________
                                            International Evidence on Smoking and COPD, Phase 3, Analysis run on 28-SEP-10

                                                   Table 3 - G - 9 - 6

                                IESCOPD - Meta-analysis of pack-years : highest vs lowest
                          Any Emphysema, cigarettes (or any product if cigarettes not available)
                                                      Least-adjusted


                       N        3
                      NS        2


                      Wt    47.97
                 Het Chi     3.58
                 Het  df        2
                 Het  P      N.S.
               Fixed  RR     1.86
                     RRl     1.40
                     RRu     2.47
                      P       +++
              Random  RR     2.42
                     RRl     1.25
                     RRu     4.70
                      P        ++
               Asymm  P       (*)

                                   Sex
                             both      male    female     Total


                       N        1         1         1         3
                      NS        1         1         1         2


                      Wt    42.81      3.56      1.60     47.97
                 Het Chi     0.00      0.00      0.00      3.58
                 Het  df        0         0         0         2
                 Het  P      N.S.      N.S.      N.S.      N.S.
               Fixed  RR     1.70      3.78      4.79      1.86
                     RRl     1.26      1.34      1.02      1.40
                     RRu     2.29     10.68     22.55      2.47
                      P       +++         +         +       +++
              Random  RR     1.70      3.78      4.79      2.42
                     RRl     1.26      1.34      1.02      1.25
                     RRu     2.29     10.68     22.55      4.70
                      P       +++         +         +        ++
             Between Chi                                   3.58
             Between  df                                      2
             Between  P                                    N.S.
             Btwn(F)  P                                    N.S.

                                        Continent
                            NAmer    Europe      Asia  oth/mult     Total


                       N                            3                   3
                      NS                            2                   2


                      Wt                        47.97               47.97
                 Het Chi                         3.58                3.58
                 Het  df                            2                   2
                 Het  P                          N.S.                N.S.
               Fixed  RR                         1.86                1.86
                     RRl                         1.40                1.40
                     RRu                         2.47                2.47
                      P                           +++                 +++
              Random  RR                         2.42                2.42
                     RRl                         1.25                1.25
                     RRu                         4.70                4.70
                      P                            ++                  ++
             Between Chi
             Between  df
             Between  P                                              N.S.
             Btwn(F)  P                                              N.S.


  ________________________________________________________________________________________________________________________
                                            International Evidence on Smoking and COPD, Phase 3, Analysis run on 28-SEP-10

                                                   Table 3 - G - 9 - 6

                                IESCOPD - Meta-analysis of pack-years : highest vs lowest
                          Any Emphysema, cigarettes (or any product if cigarettes not available)
                                                      Least-adjusted
                               Study type
                               CC        Pr        CS     Total


                       N                  2         1         3
                      NS                  1         1         2


                      Wt               5.16     42.81     47.97
                 Het Chi               0.06      0.00      3.58
                 Het  df                  1         0         2
                 Het  P                N.S.      N.S.      N.S.
               Fixed  RR               4.07      1.70      1.86
                     RRl               1.72      1.26      1.40
                     RRu               9.64      2.29      2.47
                      P                  ++       +++       +++
              Random  RR               4.07      1.70      2.42
                     RRl               1.72      1.26      1.25
                     RRu               9.64      2.29      4.70
                      P                  ++       +++        ++
             Between Chi                                   3.52
             Between  df                                      1
             Between  P                                     (*)
             Btwn(F)  P                                     (*)

                        Emp subtype
                             mort     other     Total


                       N        2         1         3
                      NS        1         1         2


                      Wt     5.16     42.81     47.97
                 Het Chi     0.06      0.00      3.58
                 Het  df        1         0         2
                 Het  P      N.S.      N.S.      N.S.
               Fixed  RR     4.07      1.70      1.86
                     RRl     1.72      1.26      1.40
                     RRu     9.64      2.29      2.47
                      P        ++       +++       +++
              Random  RR     4.07      1.70      2.42
                     RRl     1.72      1.26      1.25
                     RRu     9.64      2.29      4.70
                      P        ++       +++        ++
             Between Chi                         3.52
             Between  df                            1
             Between  P                           (*)
             Btwn(F)  P                           (*)

                             Smoking product
                              any      cigs  cigsonly     Total


                       N                  3                   3
                      NS                  2                   2


                      Wt              47.97               47.97
                 Het Chi               3.58                3.58
                 Het  df                  2                   2
                 Het  P                N.S.                N.S.
               Fixed  RR               1.86                1.86
                     RRl               1.40                1.40
                     RRu               2.47                2.47
                      P                 +++                 +++
              Random  RR               2.42                2.42
                     RRl               1.25                1.25
                     RRu               4.70                4.70
                      P                  ++                  ++
             Between Chi
             Between  df
             Between  P                                    N.S.
             Btwn(F)  P                                    N.S.
  ________________________________________________________________________________________________________________________
                                            International Evidence on Smoking and COPD, Phase 3, Analysis run on 28-SEP-10

                                                   Table 3 - G - 9 - 7

                                IESCOPD - Meta-analysis of pack-years : highest vs lowest
                          Any Emphysema, cigarettes (or any product if cigarettes not available)
                                 Excluded studies (and stage at which they were excluded)


1       CLARK COTTON  MEYER REMYJA RUTGER SNYDER SOBRAX     SU TAKEMU  WANG4   WEIR WHICKE ZALACA
2      ALDERS ALESSA  AMIGO ANDER1 ANDER3   BANG  BECK1  BECK2 BEDNAR BJORNS BROGGE  BROWN CERVER CHAPMA  CHEN1  CHEN2
        CHEN3  CHENG CLEMEN COATES  COCCI COLLEG  DEAN1  DEAN2  DEANE DEJONG DEMARC DETORR DICKIN  DOLL1  DOLL2 DONTA1
       DOPICO EHRLIC EKBERG ENSTRO FERRI1 FERRI2 FERRI3  FIDAN FINKLE FLETCH FORAST FOXMAN FUKUCH GEIJER GODTFR GOLDBE
       HAENSZ HARIKK HARRIS HAWTHO  HAYES HEDMAN HIGGI2 HIGGI3 HIGGI4 HIGGI6 HOLLA2 HOLLNA  HOUSE HRUBEC HUCHON HUHTI2
       HUHTI3 ITABAS JACOBS JAENDI JENSEN JINDA2 JOHANN  JOSHI JOUSI1 KACHEL KARAKA KATANC   KATO KHOURY    KIM  KIRAZ
       KLAYTO KOJIMA KOTAN1 KOTAN2 KRZYZA  KUBIK KULLER    LAI   LAM1   LAM2   LAM3 LAMBER  LANGE LANGE2 LANGHA    LEE
         LIAW LINDBE LINDST   LIU1   LIU2 LUNDB1 LUNDB2  MADOR MAGNUS MANFRE MANNI1 MANNI2 MANNI3 MARAN1 MARAN2 MARCUS
       MATHES MELLST MENEZ1 MENEZ2 MENEZ3 MENEZ4 MENEZ5 MENEZ6  MEREN  MILNE MOLLER MONTNE MUELLE NEJJAR NIEPSU NIHLEN
       NILSSO OGILVI OSWAL1 OSWAL2 PANDEY   PEAT PELKON PEREZP   PETO  PRICE   REID RENWIC RICCIO RIMING SARGEA SAWICK
       SCHWAR SHAHAB  SHARP SHIMUR   SHIN SICHLE SOBRAD SPEIZE STERLI STJERN  STROM SUADIC  TAGER TAGER2   TANG   THUN
         TODD TROISI TRUPIN TSUSHI TVERDA URRUTI VESTBO VIEGI1 VIEGI2 VINEIS VOLLM1 VOLLM2 VONHER WAGEN2   WALD WATSON
          WIG WILHEL WILSO1 WOJTYN  WOODS  WOOLF   XIAO     XU YAMAGU   YUAN ZIELI1 ZIELI2 ZIETKO   ZOIA
3        KAHN
4      ANDER2 AUERBA   BEST DONTA2 ENRIGH GULSVI HAMMO2 HARDIE     HO HOZAWA HUHTI1  KAHN2 LAVECC LEBOWI MILLER   NAWA
        OMORI  PRATT  RYDER  SILVA SUTINE VIKGRE  WEISS    WEN WILSO2


  ________________________________________________________________________________________________________________________
                                            International Evidence on Smoking and COPD, Phase 3, Analysis run on 28-SEP-10

                                                    Table 3 - H - 1 -

                            IESCOPD - Meta-analysis of duration of smoking : highest vs lowest
                          Any Emphysema, cigarettes (or any product if cigarettes not available)


This analysis is restricted to results for:
1) Eligible study on database
2) Outcome Emphysema
3) Current or ever smoking
4) Categorical dose-response data for duration of smoking
5) High vs low base
6) Results complete enough for use in meta-analysis

Within each study, results are then selected (in the following order of preference, within each sex) for:
7) SMKSTA  : current, ever
8) PROD    : cigarettes, cigarettes only, any product
9) The highest vs lowest category
10) For overlapping studies: principal rather than subsidiary studies
and then for single sex results (m, f) in preference to results for both sexes combined (b).

Results adjusted for the most potential confounders are then chosen in Sections -1 to -3
and results adjusted for the least confounders in Sections -4 to -6. (Those least-adjusted results which
actually differ from the most-adjusted are marked 'x' in column X in Section -4)

Section -7 shows excluded studies, together with the stage (as above) at which no qualifying
results were found.

Section -8 lists the potentially overlapping studies which have been included (1=principal, 2=subsidiary),
and any results which would have been included in preference except that they had data not complete enough
for use in meta-analysis. It also lists their significance (yes/no), if known.


  ________________________________________________________________________________________________________________________
                                            International Evidence on Smoking and COPD, Phase 3, Analysis run on 28-SEP-10

                                                   Table 3 - H - 1 - 1

                            IESCOPD - Meta-analysis of duration of smoking : highest vs lowest
                          Any Emphysema, cigarettes (or any product if cigarettes not available)
                                                      Most-adjusted


     REF|NRR|SEX|AGEL|AGEH|     REGION|BEGYR|PUBYR|STTYP|ONSET|      DISEAS|ADJ|SMOKSTA|   PRODUCT|BASE-HI|LOW| HI|

    BEST  34   m   30   97   Am:Canada  1955  1967    Pr   Inc     Emp:mort   1 Current  Cigs only       9  40   +
  SUTINE   6   b   10   99    Eu:Scand  1971  1978    CS  Prev  Emp:viscomp   0    Ever        Any      19  40   +


  ________________________________________________________________________________________________________________________
                                            International Evidence on Smoking and COPD, Phase 3, Analysis run on 28-SEP-10

                                                   Table 3 - H - 1 - 2

                            IESCOPD - Meta-analysis of duration of smoking : highest vs lowest
                          Any Emphysema, cigarettes (or any product if cigarettes not available)
                                                      Most-adjusted


                        Number Exposed  Non-exposed
 REF    NRR SEX ADJ     Case    Cont    Case    Cont      RR        95.00%CI
*BEST   34  m   1         14       -       1       -      1.03 (  0.15-   7.24)
 SUTINE 6   b   0         31       3       2      11     56.83 (  8.36- 386.44)
Partial Totals            45       3       3      11
*prospective study


 REF    NRR SEX ADJ             Ys       Ws       Qs       Ps
*BEST   34  m   1              0.03     1.02     4.20       0.98
 SUTINE 6   b   0              4.04     1.05     4.11       0.00

                       N        2
                      NS        2


                      Wt     2.07
                 Het Chi     8.31
                 Het  df        1
                 Het  P        **
               Fixed  RR     7.82
                     RRl     2.00
                     RRu    30.58
                      P        ++
              Random  RR     7.67
                     RRl     0.15
                     RRu   390.65
                      P      N.S.
               Asymm  P


  ________________________________________________________________________________________________________________________
                                            International Evidence on Smoking and COPD, Phase 3, Analysis run on 28-SEP-10

                                                   Table 3 - H - 1 - 3

                            IESCOPD - Meta-analysis of duration of smoking : highest vs lowest
                          Any Emphysema, cigarettes (or any product if cigarettes not available)
                                                      Most-adjusted


                       N        2
                      NS        2


                      Wt     2.07
                 Het Chi     8.31
                 Het  df        1
                 Het  P        **
               Fixed  RR     7.82
                     RRl     2.00
                     RRu    30.58
                      P        ++
              Random  RR     7.67
                     RRl     0.15
                     RRu   390.65
                      P      N.S.
               Asymm  P

                                   Sex
                             both      male    female     Total


                       N        1         1                   2
                      NS        1         1                   2


                      Wt     1.05      1.02                2.07
                 Het Chi     0.00      0.00                8.31
                 Het  df        0         0                   1
                 Het  P      N.S.      N.S.                  **
               Fixed  RR    56.83      1.03                7.82
                     RRl     8.36      0.15                2.00
                     RRu   386.44      7.16               30.58
                      P       +++      N.S.                  ++
              Random  RR    56.83      1.03                7.67
                     RRl     8.36      0.15                0.15
                     RRu   386.44      7.16              390.65
                      P       +++      N.S.                N.S.
             Between Chi                                   8.31
             Between  df                                      1
             Between  P                                      **
             Btwn(F)  P                                    N.S.

                                        Continent
                            NAmer    Europe      Asia  oth/mult     Total


                       N        1         1                             2
                      NS        1         1                             2


                      Wt     1.02      1.05                          2.07
                 Het Chi     0.00      0.00                          8.31
                 Het  df        0         0                             1
                 Het  P      N.S.      N.S.                            **
               Fixed  RR     1.03     56.83                          7.82
                     RRl     0.15      8.36                          2.00
                     RRu     7.16    386.44                         30.58
                      P      N.S.       +++                            ++
              Random  RR     1.03     56.83                          7.67
                     RRl     0.15      8.36                          0.15
                     RRu     7.16    386.44                        390.65
                      P      N.S.       +++                          N.S.
             Between Chi                                             8.31
             Between  df                                                1
             Between  P                                                **
             Btwn(F)  P                                              N.S.


  ________________________________________________________________________________________________________________________
                                            International Evidence on Smoking and COPD, Phase 3, Analysis run on 28-SEP-10

                                                   Table 3 - H - 1 - 3

                            IESCOPD - Meta-analysis of duration of smoking : highest vs lowest
                          Any Emphysema, cigarettes (or any product if cigarettes not available)
                                                      Most-adjusted
                        National cigarette tobacco type (excluding mixed/unkown)
                          blended  virginia     Total


                       N        1         1         2
                      NS        1         1         2


                      Wt     1.05      1.02      2.07
                 Het Chi     0.00      0.00      8.31
                 Het  df        0         0         1
                 Het  P      N.S.      N.S.        **
               Fixed  RR    56.83      1.03      7.82
                     RRl     8.36      0.15      2.00
                     RRu   386.44      7.16     30.58
                      P       +++      N.S.        ++
              Random  RR    56.83      1.03      7.67
                     RRl     8.36      0.15      0.15
                     RRu   386.44      7.16    390.65
                      P       +++      N.S.      N.S.
             Between Chi                         8.31
             Between  df                            1
             Between  P                            **
             Btwn(F)  P                          N.S.

                                        Start year of study
                            <1970   1970-79   1980-89   1990-99     2000+   unknown     Total


                       N        1         1                                                 2
                      NS        1         1                                                 2


                      Wt     1.02      1.05                                              2.07
                 Het Chi     0.00      0.00                                              8.31
                 Het  df        0         0                                                 1
                 Het  P      N.S.      N.S.                                                **
               Fixed  RR     1.03     56.83                                              7.82
                     RRl     0.15      8.36                                              2.00
                     RRu     7.16    386.44                                             30.58
                      P      N.S.       +++                                                ++
              Random  RR     1.03     56.83                                              7.67
                     RRl     0.15      8.36                                              0.15
                     RRu     7.16    386.44                                            390.65
                      P      N.S.       +++                                              N.S.
             Between Chi                                                                 8.31
             Between  df                                                                    1
             Between  P                                                                    **
             Btwn(F)  P                                                                  N.S.

                                Publication year
                            <1980   1980-89   1990-99     2000+     Total


                       N        2                                       2
                      NS        2                                       2


                      Wt     2.07                                    2.07
                 Het Chi     8.31                                    8.31
                 Het  df        1                                       1
                 Het  P        **                                      **
               Fixed  RR     7.82                                    7.82
                     RRl     2.00                                    2.00
                     RRu    30.58                                   30.58
                      P        ++                                      ++
              Random  RR     7.67                                    7.67
                     RRl     0.15                                    0.15
                     RRu   390.65                                  390.65
                      P      N.S.                                    N.S.
             Between Chi
             Between  df
             Between  P                                              N.S.
             Btwn(F)  P                                              N.S.
  ________________________________________________________________________________________________________________________
                                            International Evidence on Smoking and COPD, Phase 3, Analysis run on 28-SEP-10

                                                   Table 3 - H - 1 - 3

                            IESCOPD - Meta-analysis of duration of smoking : highest vs lowest
                          Any Emphysema, cigarettes (or any product if cigarettes not available)
                                                      Most-adjusted
                               Study type
                               CC        Pr        CS     Total


                       N                  1         1         2
                      NS                  1         1         2


                      Wt               1.02      1.05      2.07
                 Het Chi               0.00      0.00      8.31
                 Het  df                  0         0         1
                 Het  P                N.S.      N.S.        **
               Fixed  RR               1.03     56.83      7.82
                     RRl               0.15      8.36      2.00
                     RRu               7.16    386.44     30.58
                      P                N.S.       +++        ++
              Random  RR               1.03     56.83      7.67
                     RRl               0.15      8.36      0.15
                     RRu               7.16    386.44    390.65
                      P                N.S.       +++      N.S.
             Between Chi                                   8.31
             Between  df                                      1
             Between  P                                      **
             Btwn(F)  P                                    N.S.

                                    Lowest age in RR
                        <25/unlim     25-39       40+   unknown     Total


                       N        1         1                             2
                      NS        1         1                             2


                      Wt     1.05      1.02                          2.07
                 Het Chi     0.00      0.00                          8.31
                 Het  df        0         0                             1
                 Het  P      N.S.      N.S.                            **
               Fixed  RR    56.83      1.03                          7.82
                     RRl     8.36      0.15                          2.00
                     RRu   386.44      7.16                         30.58
                      P       +++      N.S.                            ++
              Random  RR    56.83      1.03                          7.67
                     RRl     8.36      0.15                          0.15
                     RRu   386.44      7.16                        390.65
                      P       +++      N.S.                          N.S.
             Between Chi                                             8.31
             Between  df                                                1
             Between  P                                                **
             Btwn(F)  P                                              N.S.

                                         Highest age in RR
                              <65     65-74     75-84 85+/unlim   unknown     Total


                       N                                      2                   2
                      NS                                      2                   2


                      Wt                                   2.07                2.07
                 Het Chi                                   8.31                8.31
                 Het  df                                      1                   1
                 Het  P                                      **                  **
               Fixed  RR                                   7.82                7.82
                     RRl                                   2.00                2.00
                     RRu                                  30.58               30.58
                      P                                      ++                  ++
              Random  RR                                   7.67                7.67
                     RRl                                   0.15                0.15
                     RRu                                 390.65              390.65
                      P                                    N.S.                N.S.
             Between Chi
             Between  df
             Between  P                                                        N.S.
             Btwn(F)  P                                                        N.S.
  ________________________________________________________________________________________________________________________
                                            International Evidence on Smoking and COPD, Phase 3, Analysis run on 28-SEP-10

                                                   Table 3 - H - 1 - 3

                            IESCOPD - Meta-analysis of duration of smoking : highest vs lowest
                          Any Emphysema, cigarettes (or any product if cigarettes not available)
                                                      Most-adjusted
                           Study weakness
                              Yes        No     Total


                       N                  2         2
                      NS                  2         2


                      Wt               2.07      2.07
                 Het Chi               8.31      8.31
                 Het  df                  1         1
                 Het  P                  **        **
               Fixed  RR               7.82      7.82
                     RRl               2.00      2.00
                     RRu              30.58     30.58
                      P                  ++        ++
              Random  RR               7.67      7.67
                     RRl               0.15      0.15
                     RRu             390.65    390.65
                      P                N.S.      N.S.
             Between Chi
             Between  df
             Between  P                          N.S.
             Btwn(F)  P                          N.S.

                           Type of outcome
                        prevalnce mortality incidence     Total


                       N        1         1                   2
                      NS        1         1                   2


                      Wt     1.05      1.02                2.07
                 Het Chi     0.00      0.00                8.31
                 Het  df        0         0                   1
                 Het  P      N.S.      N.S.                  **
               Fixed  RR    56.83      1.03                7.82
                     RRl     8.36      0.15                2.00
                     RRu   386.44      7.16               30.58
                      P       +++      N.S.                  ++
              Random  RR    56.83      1.03                7.67
                     RRl     8.36      0.15                0.15
                     RRu   386.44      7.16              390.65
                      P       +++      N.S.                N.S.
             Between Chi                                   8.31
             Between  df                                      1
             Between  P                                      **
             Btwn(F)  P                                    N.S.

                        Emp subtype
                             mort     other     Total


                       N        1         1         2
                      NS        1         1         2


                      Wt     1.02      1.05      2.07
                 Het Chi     0.00      0.00      8.31
                 Het  df        0         0         1
                 Het  P      N.S.      N.S.        **
               Fixed  RR     1.03     56.83      7.82
                     RRl     0.15      8.36      2.00
                     RRu     7.16    386.44     30.58
                      P      N.S.       +++        ++
              Random  RR     1.03     56.83      7.67
                     RRl     0.15      8.36      0.15
                     RRu     7.16    386.44    390.65
                      P      N.S.       +++      N.S.
             Between Chi                         8.31
             Between  df                            1
             Between  P                            **
             Btwn(F)  P                          N.S.
  ________________________________________________________________________________________________________________________
                                            International Evidence on Smoking and COPD, Phase 3, Analysis run on 28-SEP-10

                                                   Table 3 - H - 1 - 3

                            IESCOPD - Meta-analysis of duration of smoking : highest vs lowest
                          Any Emphysema, cigarettes (or any product if cigarettes not available)
                                                      Most-adjusted
                        Asthma analysis type (Emphysema)
                        inc-irres  excl-all     Total


                       N        2                   2
                      NS        2                   2


                      Wt     2.07                2.07
                 Het Chi     8.31                8.31
                 Het  df        1                   1
                 Het  P        **                  **
               Fixed  RR     7.82                7.82
                     RRl     2.00                2.00
                     RRu    30.58               30.58
                      P        ++                  ++
              Random  RR     7.67                7.67
                     RRl     0.15                0.15
                     RRu   390.65              390.65
                      P      N.S.                N.S.
             Between Chi
             Between  df
             Between  P                          N.S.
             Btwn(F)  P                          N.S.

                                Number of emphysema cases
                             1-50    51-100   101-200      201+     Total


                       N                  2                             2
                      NS                  2                             2


                      Wt               2.07                          2.07
                 Het Chi               8.31                          8.31
                 Het  df                  1                             1
                 Het  P                  **                            **
               Fixed  RR               7.82                          7.82
                     RRl               2.00                          2.00
                     RRu              30.58                         30.58
                      P                  ++                            ++
              Random  RR               7.67                          7.67
                     RRl               0.15                          0.15
                     RRu             390.65                        390.65
                      P                N.S.                          N.S.
             Between Chi
             Between  df
             Between  P                                              N.S.
             Btwn(F)  P                                              N.S.

                        Number of emphysma cases (excluding unknown)


                       N                  2                             2
                      NS                  2                             2


                      Wt               2.07                          2.07
                 Het Chi               8.31                          8.31
                 Het  df                  1                             1
                 Het  P                  **                            **
               Fixed  RR               7.82                          7.82
                     RRl               2.00                          2.00
                     RRu              30.58                         30.58
                      P                  ++                            ++
              Random  RR               7.67                          7.67
                     RRl               0.15                          0.15
                     RRu             390.65                        390.65
                      P                N.S.                          N.S.
             Between Chi
             Between  df
             Between  P                                              N.S.
             Btwn(F)  P                                              N.S.

  ________________________________________________________________________________________________________________________
                                            International Evidence on Smoking and COPD, Phase 3, Analysis run on 28-SEP-10

                                                   Table 3 - H - 1 - 3

                            IESCOPD - Meta-analysis of duration of smoking : highest vs lowest
                          Any Emphysema, cigarettes (or any product if cigarettes not available)
                                                      Most-adjusted
                            Analysis type
                         prevlnce     onset     Total


                       N        1         1         2
                      NS        1         1         2


                      Wt     1.05      1.02      2.07
                 Het Chi     0.00      0.00      8.31
                 Het  df        0         0         1
                 Het  P      N.S.      N.S.        **
               Fixed  RR    56.83      1.03      7.82
                     RRl     8.36      0.15      2.00
                     RRu   386.44      7.16     30.58
                      P       +++      N.S.        ++
              Random  RR    56.83      1.03      7.67
                     RRl     8.36      0.15      0.15
                     RRu   386.44      7.16    390.65
                      P       +++      N.S.      N.S.
             Between Chi                         8.31
             Between  df                            1
             Between  P                            **
             Btwn(F)  P                          N.S.

                             Smoking product
                              any      cigs  cigsonly     Total


                       N        1                   1         2
                      NS        1                   1         2


                      Wt     1.05                1.02      2.07
                 Het Chi     0.00                0.00      8.31
                 Het  df        0                   0         1
                 Het  P      N.S.                N.S.        **
               Fixed  RR    56.83                1.03      7.82
                     RRl     8.36                0.15      2.00
                     RRu   386.44                7.16     30.58
                      P       +++                N.S.        ++
              Random  RR    56.83                1.03      7.67
                     RRl     8.36                0.15      0.15
                     RRu   386.44                7.16    390.65
                      P       +++                N.S.      N.S.
             Between Chi                                   8.31
             Between  df                                      1
             Between  P                                      **
             Btwn(F)  P                                    N.S.

                        Number of adjustment variables
                                0         1        2+     Total


                       N        1         1                   2
                      NS        1         1                   2


                      Wt     1.05      1.02                2.07
                 Het Chi     0.00      0.00                8.31
                 Het  df        0         0                   1
                 Het  P      N.S.      N.S.                  **
               Fixed  RR    56.83      1.03                7.82
                     RRl     8.36      0.15                2.00
                     RRu   386.44      7.16               30.58
                      P       +++      N.S.                  ++
              Random  RR    56.83      1.03                7.67
                     RRl     8.36      0.15                0.15
                     RRu   386.44      7.16              390.65
                      P       +++      N.S.                N.S.
             Between Chi                                   8.31
             Between  df                                      1
             Between  P                                      **
             Btwn(F)  P                                    N.S.
  ________________________________________________________________________________________________________________________
                                            International Evidence on Smoking and COPD, Phase 3, Analysis run on 28-SEP-10

                                                   Table 3 - H - 1 - 4

                            IESCOPD - Meta-analysis of duration of smoking : highest vs lowest
                          Any Emphysema, cigarettes (or any product if cigarettes not available)
                                                      Least-adjusted


     REF|NRR|X|SEX|AGEL|AGEH|     REGION|BEGYR|PUBYR|STTYP|ONSET|      DISEAS|ADJ|SMOKSTA|   PRODUCT|BASE-HI|LOW| HI|

    BEST  34     m   30   97   Am:Canada  1955  1967    Pr   Inc     Emp:mort   1 Current  Cigs only       9  40   +
  SUTINE   6     b   10   99    Eu:Scand  1971  1978    CS  Prev  Emp:viscomp   0    Ever        Any      19  40   +


  ________________________________________________________________________________________________________________________
                                            International Evidence on Smoking and COPD, Phase 3, Analysis run on 28-SEP-10

                                                   Table 3 - H - 1 - 5

                            IESCOPD - Meta-analysis of duration of smoking : highest vs lowest
                          Any Emphysema, cigarettes (or any product if cigarettes not available)
                                                      Least-adjusted


                        Number Exposed  Non-exposed
 REF    NRR SEX ADJ     Case    Cont    Case    Cont      RR        95.00%CI
*BEST   34  m   1         14       -       1       -      1.03 (  0.15-   7.24)
 SUTINE 6   b   0         31       3       2      11     56.83 (  8.36- 386.44)
Partial Totals            45       3       3      11
*prospective study


 REF    NRR SEX ADJ             Ys       Ws       Qs       Ps
*BEST   34  m   1              0.03     1.02     4.20       0.98
 SUTINE 6   b   0              4.04     1.05     4.11       0.00

                       N        2
                      NS        2


                      Wt     2.07
                 Het Chi     8.31
                 Het  df        1
                 Het  P        **
               Fixed  RR     7.82
                     RRl     2.00
                     RRu    30.58
                      P        ++
              Random  RR     7.67
                     RRl     0.15
                     RRu   390.65
                      P      N.S.
               Asymm  P


  ________________________________________________________________________________________________________________________
                                            International Evidence on Smoking and COPD, Phase 3, Analysis run on 28-SEP-10

                                                   Table 3 - H - 1 - 6

                            IESCOPD - Meta-analysis of duration of smoking : highest vs lowest
                          Any Emphysema, cigarettes (or any product if cigarettes not available)
                                                      Least-adjusted


                       N        2
                      NS        2


                      Wt     2.07
                 Het Chi     8.31
                 Het  df        1
                 Het  P        **
               Fixed  RR     7.82
                     RRl     2.00
                     RRu    30.58
                      P        ++
              Random  RR     7.67
                     RRl     0.15
                     RRu   390.65
                      P      N.S.
               Asymm  P

                                   Sex
                             both      male    female     Total


                       N        1         1                   2
                      NS        1         1                   2


                      Wt     1.05      1.02                2.07
                 Het Chi     0.00      0.00                8.31
                 Het  df        0         0                   1
                 Het  P      N.S.      N.S.                  **
               Fixed  RR    56.83      1.03                7.82
                     RRl     8.36      0.15                2.00
                     RRu   386.44      7.16               30.58
                      P       +++      N.S.                  ++
              Random  RR    56.83      1.03                7.67
                     RRl     8.36      0.15                0.15
                     RRu   386.44      7.16              390.65
                      P       +++      N.S.                N.S.
             Between Chi                                   8.31
             Between  df                                      1
             Between  P                                      **
             Btwn(F)  P                                    N.S.

                                        Continent
                            NAmer    Europe      Asia  oth/mult     Total


                       N        1         1                             2
                      NS        1         1                             2


                      Wt     1.02      1.05                          2.07
                 Het Chi     0.00      0.00                          8.31
                 Het  df        0         0                             1
                 Het  P      N.S.      N.S.                            **
               Fixed  RR     1.03     56.83                          7.82
                     RRl     0.15      8.36                          2.00
                     RRu     7.16    386.44                         30.58
                      P      N.S.       +++                            ++
              Random  RR     1.03     56.83                          7.67
                     RRl     0.15      8.36                          0.15
                     RRu     7.16    386.44                        390.65
                      P      N.S.       +++                          N.S.
             Between Chi                                             8.31
             Between  df                                                1
             Between  P                                                **
             Btwn(F)  P                                              N.S.


  ________________________________________________________________________________________________________________________
                                            International Evidence on Smoking and COPD, Phase 3, Analysis run on 28-SEP-10

                                                   Table 3 - H - 1 - 6

                            IESCOPD - Meta-analysis of duration of smoking : highest vs lowest
                          Any Emphysema, cigarettes (or any product if cigarettes not available)
                                                      Least-adjusted
                               Study type
                               CC        Pr        CS     Total


                       N                  1         1         2
                      NS                  1         1         2


                      Wt               1.02      1.05      2.07
                 Het Chi               0.00      0.00      8.31
                 Het  df                  0         0         1
                 Het  P                N.S.      N.S.        **
               Fixed  RR               1.03     56.83      7.82
                     RRl               0.15      8.36      2.00
                     RRu               7.16    386.44     30.58
                      P                N.S.       +++        ++
              Random  RR               1.03     56.83      7.67
                     RRl               0.15      8.36      0.15
                     RRu               7.16    386.44    390.65
                      P                N.S.       +++      N.S.
             Between Chi                                   8.31
             Between  df                                      1
             Between  P                                      **
             Btwn(F)  P                                    N.S.

                        Emp subtype
                             mort     other     Total


                       N        1         1         2
                      NS        1         1         2


                      Wt     1.02      1.05      2.07
                 Het Chi     0.00      0.00      8.31
                 Het  df        0         0         1
                 Het  P      N.S.      N.S.        **
               Fixed  RR     1.03     56.83      7.82
                     RRl     0.15      8.36      2.00
                     RRu     7.16    386.44     30.58
                      P      N.S.       +++        ++
              Random  RR     1.03     56.83      7.67
                     RRl     0.15      8.36      0.15
                     RRu     7.16    386.44    390.65
                      P      N.S.       +++      N.S.
             Between Chi                         8.31
             Between  df                            1
             Between  P                            **
             Btwn(F)  P                          N.S.

                             Smoking product
                              any      cigs  cigsonly     Total


                       N        1                   1         2
                      NS        1                   1         2


                      Wt     1.05                1.02      2.07
                 Het Chi     0.00                0.00      8.31
                 Het  df        0                   0         1
                 Het  P      N.S.                N.S.        **
               Fixed  RR    56.83                1.03      7.82
                     RRl     8.36                0.15      2.00
                     RRu   386.44                7.16     30.58
                      P       +++                N.S.        ++
              Random  RR    56.83                1.03      7.67
                     RRl     8.36                0.15      0.15
                     RRu   386.44                7.16    390.65
                      P       +++                N.S.      N.S.
             Between Chi                                   8.31
             Between  df                                      1
             Between  P                                      **
             Btwn(F)  P                                    N.S.
  ________________________________________________________________________________________________________________________
                                            International Evidence on Smoking and COPD, Phase 3, Analysis run on 28-SEP-10

                                                   Table 3 - H - 1 - 7

                            IESCOPD - Meta-analysis of duration of smoking : highest vs lowest
                          Any Emphysema, cigarettes (or any product if cigarettes not available)
                                 Excluded studies (and stage at which they were excluded)


1       CLARK COTTON  MEYER REMYJA RUTGER SNYDER SOBRAX     SU TAKEMU  WANG4   WEIR WHICKE ZALACA
2      ALDERS ALESSA  AMIGO ANDER1 ANDER3   BANG  BECK1  BECK2 BEDNAR BJORNS BROGGE  BROWN CERVER CHAPMA  CHEN1  CHEN2
        CHEN3  CHENG CLEMEN COATES  COCCI COLLEG  DEAN1  DEAN2  DEANE DEJONG DEMARC DETORR DICKIN  DOLL1  DOLL2 DONTA1
       DOPICO EHRLIC EKBERG ENSTRO FERRI1 FERRI2 FERRI3  FIDAN FINKLE FLETCH FORAST FOXMAN FUKUCH GEIJER GODTFR GOLDBE
       HAENSZ HARIKK HARRIS HAWTHO  HAYES HEDMAN HIGGI2 HIGGI3 HIGGI4 HIGGI6 HOLLA2 HOLLNA  HOUSE HRUBEC HUCHON HUHTI2
       HUHTI3 ITABAS JACOBS JAENDI JENSEN JINDA2 JOHANN  JOSHI JOUSI1 KACHEL KARAKA KATANC   KATO KHOURY    KIM  KIRAZ
       KLAYTO KOJIMA KOTAN1 KOTAN2 KRZYZA  KUBIK KULLER    LAI   LAM1   LAM2   LAM3 LAMBER  LANGE LANGE2 LANGHA    LEE
         LIAW LINDBE LINDST   LIU1   LIU2 LUNDB1 LUNDB2  MADOR MAGNUS MANFRE MANNI1 MANNI2 MANNI3 MARAN1 MARAN2 MARCUS
       MATHES MELLST MENEZ1 MENEZ2 MENEZ3 MENEZ4 MENEZ5 MENEZ6  MEREN  MILNE MOLLER MONTNE MUELLE NEJJAR NIEPSU NIHLEN
       NILSSO OGILVI OSWAL1 OSWAL2 PANDEY   PEAT PELKON PEREZP   PETO  PRICE   REID RENWIC RICCIO RIMING SARGEA SAWICK
       SCHWAR SHAHAB  SHARP SHIMUR   SHIN SICHLE SOBRAD SPEIZE STERLI STJERN  STROM SUADIC  TAGER TAGER2   TANG   THUN
         TODD TROISI TRUPIN TSUSHI TVERDA URRUTI VESTBO VIEGI1 VIEGI2 VINEIS VOLLM1 VOLLM2 VONHER WAGEN2   WALD WATSON
          WIG WILHEL WILSO1 WOJTYN  WOODS  WOOLF   XIAO     XU YAMAGU   YUAN ZIELI1 ZIELI2 ZIETKO   ZOIA
3        KAHN
4      ANDER2 AUERBA DONTA2 ENRIGH GULSVI HAMMO2 HARDIE HIRAYA     HO HOZAWA HUHTI1  KAHN2 LAVECC LEBOWI MILLER   NAWA
        OMORI  PRATT  RYDER  SILVA VIKGRE  WANG2  WEISS WILSO2
5         WEN


  ________________________________________________________________________________________________________________________
                                            International Evidence on Smoking and COPD, Phase 3, Analysis run on 28-SEP-10
