# Supplementary material for: Systematic review with meta-analysis of the epidemiological evidence relating smoking to COPD, chronic bronchitis and emphysema
Source: BMC Pulm Med. 2011 Jun 14;11:36. doi: 10.1186/1471-2466-11-36 (PMC3128042; doi:10.1186/1471-2466-11-36)
Supplement: Additional file 11 — MetaRegressionTables. .DOC file giving the full results of the meta-regressions carried out for ever smoking and current smoking. [file 1471-2466-11-36-S11.DOC]

**Systematic review with meta-analysis of the epidemiological evidence relating smoking to COPD, chronic bronchitis and emphysema**

Barbara A Forey, Alison J Thornton and Peter N Lee

**Additional File 11 : MetaRegression Tables**

***Contents***

Table 1 - A - 6 [2](#__RefHeading___Toc275782825)

Table 1 - B - 6 [22](#__RefHeading___Toc275782826)

Table 2 - A - 6 [41](#__RefHeading___Toc275782827)

Table 2 - B - 6 [59](#__RefHeading___Toc275782828)

Table 3 - A - 6 [77](#__RefHeading___Toc275782829)

Table 3 - B - 6 [92](#__RefHeading___Toc275782830)

## Table 1 - A - 6

IESCOPD - Meta-regression of ever smoking, any product (or cigarettes if all product not available)

Multiple regression of data from Table 1 - A - 1 (most-adjusted RRs)

Any COPD

Fixed model

|  |  | **Deviance** | **(DF)** |  |  |  |  |
| --- | --- | --- | --- | --- | --- | --- | --- |
|  |  |  |  |  |  |  |  |
| **Fixed model** |  | 421.765 | (112) |  |  |  |  |
|  |  | **Estimate** | **S.E.** | **P** | **RR** | **95%CIl** | **95%CIu** |
| **Constant** |  | 1.149 | 0.141 | +++ | 3.155 | 2.394 | 4.159 |
| **Sex(RR)** |  |  |  |  |  |  |  |
| Male | 49 | Aliased |  |  | 2.462 | 2.195 | 2.763 |
| Female | 35 | -0.212 | 0.044 | --- | 1.993 | 1.761 | 2.256 |
| Combined | 45 | -0.006 | 0.039 | N.S. | 2.447 | 2.253 | 2.658 |
| **Continent** |  |  |  |  |  |  |  |
| NAmer | 35 | Aliased |  |  | 2.766 | 2.442 | 3.132 |
| Europe | 59 | -0.200 | 0.040 | --- | 2.264 | 2.097 | 2.443 |
| Asia | 23 | -0.295 | 0.067 | --- | 2.059 | 1.675 | 2.531 |
| oth/mult | 12 | -0.355 | 0.072 | --- | 1.940 | 1.521 | 2.475 |
| **Asthma analysis type (COPD)** |  |  |  |  |  |  |  |
| inc-irresp | 76 | Aliased |  |  | 2.500 | 2.323 | 2.690 |
| excl-all | 21 | -0.143 | 0.043 | -- | 2.167 | 1.891 | 2.484 |
| defn-incl | 19 | -0.461 | 0.060 | --- | 1.576 | 1.274 | 1.949 |
| other | 13 | 0.283 | 0.093 | ++ | 3.318 | 2.359 | 4.667 |
| **Smoking product (3)** |  |  |  |  |  |  |  |
| any | 48 | Aliased |  |  | 1.712 | 1.233 | 2.376 |
| cigs | 73 | 0.428 | 0.128 | ++ | 2.627 | 2.212 | 3.119 |
| cigsonly | 8 | 0.589 | 0.107 | +++ | 3.085 | 1.897 | 5.018 |
| **Unexposed group** |  |  |  |  |  |  |  |
| nev any | 57 | Aliased |  |  | 3.443 | 2.533 | 4.680 |
| nev cig | 72 | -0.603 | 0.123 | --- | 1.884 | 1.581 | 2.246 |
| **COPD subtype** |  |  |  |  |  |  |  |
| mort | 29 | Aliased |  |  | 3.155 | 2.311 | 4.309 |
| LF | 60 | -0.404 | 0.092 | --- | 2.107 | 1.943 | 2.285 |
| other | 40 | -0.114 | 0.085 | N.S. | 2.816 | 2.453 | 3.232 |
| **Midpoint age in RR** |  | 0.003 | 0.002 | N.S. | 1.925 | 1.203 | 3.080 |
| **RR adjusted for age** |  |  |  |  |  |  |  |
| Yes | 69 | Aliased |  |  | 2.273 | 2.028 | 2.548 |
| No | 60 | 0.046 | 0.045 | N.S. | 2.380 | 2.181 | 2.597 |
| **RR adjusted for factor other than sex, age** |  |  |  |  |  |  |  |
| Yes | 34 | Aliased |  |  | 2.025 | 1.733 | 2.366 |
| No | 95 | 0.195 | 0.052 | +++ | 2.460 | 2.282 | 2.652 |

Table 1 - A - 6

IESCOPD - Meta-regression of ever smoking, any product (or cigarettes if all product not available)

Multiple regression of data from Table 1 - A - 1 (most-adjusted RRs)

Any COPD

Test removing variables one at a time

|  |  | **Deviance** | **(DF)** | **Drop Dev** | **P** |  |  |
| --- | --- | --- | --- | --- | --- | --- | --- |
| **Omitting Sex (RR)** |  | 451.322 | (114) | -29.557 | * |  |  |
|  |  | **Estimate** | **S.E.** | **P** | **RR** | **95%CIl** | **95%CIu** |
| **Constant** |  | 1.150 | 0.140 | +++ | 3.158 | 2.399 | 4.157 |
| **RR adjusted for factor other than sex, age** |  |  |  |  |  |  |  |
| Yes | 34 | Aliased |  |  | 2.090 | 1.794 | 2.434 |
| No | 95 | 0.152 | 0.052 | ++ | 2.432 | 2.259 | 2.619 |
| **Continent** |  |  |  |  |  |  |  |
| NAmer | 35 | Aliased |  |  | 2.808 | 2.485 | 3.172 |
| Europe | 59 | -0.221 | 0.040 | --- | 2.251 | 2.087 | 2.428 |
| Asia | 23 | -0.314 | 0.066 | --- | 2.050 | 1.676 | 2.507 |
| oth/mult | 12 | -0.373 | 0.072 | --- | 1.934 | 1.523 | 2.457 |
| **Asthma analysis type (COPD)** |  |  |  |  |  |  |  |
| inc-irresp | 76 | Aliased |  |  | 2.538 | 2.362 | 2.727 |
| excl-all | 21 | -0.205 | 0.041 | --- | 2.068 | 1.818 | 2.352 |
| defn-incl | 19 | -0.474 | 0.060 | --- | 1.580 | 1.283 | 1.947 |
| other | 13 | 0.306 | 0.093 | ++ | 3.446 | 2.460 | 4.827 |
| **Smoking product (3)** |  |  |  |  |  |  |  |
| any | 48 | Aliased |  |  | 2.009 | 1.481 | 2.726 |
| cigs | 73 | 0.194 | 0.120 | N.S. | 2.440 | 2.075 | 2.869 |
| cigsonly | 8 | 0.475 | 0.104 | +++ | 3.231 | 2.004 | 5.207 |
| **Unexposed group** |  |  |  |  |  |  |  |
| nev any | 57 | Aliased |  |  | 2.973 | 2.237 | 3.950 |
| nev cig | 72 | -0.375 | 0.115 | -- | 2.044 | 1.736 | 2.407 |
| **COPD subtype** |  |  |  |  |  |  |  |
| mort | 29 | Aliased |  |  | 3.163 | 2.347 | 4.262 |
| LF | 60 | -0.400 | 0.089 | --- | 2.120 | 1.958 | 2.296 |
| other | 40 | -0.135 | 0.082 | N.S. | 2.764 | 2.412 | 3.167 |
| **Midpoint age in RR** |  | 0.004 | 0.002 | (+) | 1.891 | 1.189 | 3.008 |
| **RR adjusted for age** |  |  |  |  |  |  |  |
| Yes | 69 | Aliased |  |  | 2.260 | 2.018 | 2.531 |
| No | 60 | 0.055 | 0.045 | N.S. | 2.389 | 2.191 | 2.605 |

Table 1 - A - 6

IESCOPD - Meta-regression of ever smoking, any product (or cigarettes if all product not available)

Multiple regression of data from Table 1 - A - 1 (most-adjusted RRs)

Any COPD

Test by removing variables one at a time

|  |  | **Deviance** | **(DF)** | **Drop Dev** | **P** |  |  |
| --- | --- | --- | --- | --- | --- | --- | --- |
| **Omitting Continent** |  | 460.282 | (115) | -38.517 | * |  |  |
|  |  | **Estimate** | **S.E.** | **P** | **RR** | **95%CIl** | **95%CIu** |
| **Constant** |  | 1.152 | 0.136 | +++ | 3.165 | 2.424 | 4.133 |
| **RR adjusted for factor other than sex, age** |  |  |  |  |  |  |  |
| Yes | 34 | Aliased |  |  | 1.838 | 1.601 | 2.111 |
| No | 95 | 0.327 | 0.046 | +++ | 2.548 | 2.376 | 2.733 |
| **Sex(RR)** |  |  |  |  |  |  |  |
| Male | 49 | Aliased |  |  | 2.441 | 2.184 | 2.729 |
| Female | 35 | -0.220 | 0.044 | --- | 1.959 | 1.735 | 2.213 |
| Combined | 45 | 0.015 | 0.038 | N.S. | 2.477 | 2.287 | 2.684 |
| **Asthma analysis type (COPD)** |  |  |  |  |  |  |  |
| inc-irresp | 76 | Aliased |  |  | 2.530 | 2.358 | 2.714 |
| excl-all | 21 | -0.176 | 0.041 | --- | 2.122 | 1.867 | 2.412 |
| defn-incl | 19 | -0.462 | 0.058 | --- | 1.593 | 1.298 | 1.955 |
| other | 13 | 0.178 | 0.089 | + | 3.022 | 2.196 | 4.160 |
| **Smoking product (3)** |  |  |  |  |  |  |  |
| any | 48 | Aliased |  |  | 1.432 | 1.056 | 1.943 |
| cigs | 73 | 0.694 | 0.120 | +++ | 2.867 | 2.441 | 3.368 |
| cigsonly | 8 | 0.632 | 0.106 | +++ | 2.696 | 1.680 | 4.326 |
| **Unexposed group** |  |  |  |  |  |  |  |
| nev any | 57 | Aliased |  |  | 4.041 | 3.035 | 5.380 |
| nev cig | 72 | -0.852 | 0.116 | --- | 1.724 | 1.463 | 2.032 |
| **COPD subtype** |  |  |  |  |  |  |  |
| mort | 29 | Aliased |  |  | 3.407 | 2.520 | 4.608 |
| LF | 60 | -0.494 | 0.091 | --- | 2.080 | 1.922 | 2.251 |
| other | 40 | -0.181 | 0.082 | - | 2.842 | 2.490 | 3.244 |
| **Midpoint age in RR** |  | 0.000 | 0.002 | N.S. | 2.281 | 1.484 | 3.505 |
| **RR adjusted for age** |  |  |  |  |  |  |  |
| Yes | 69 | Aliased |  |  | 2.352 | 2.108 | 2.625 |
| No | 60 | -0.012 | 0.043 | N.S. | 2.324 | 2.136 | 2.529 |
|  |  | **Deviance** | **(DF)** | **Drop Dev** | **P** |  |  |
| **Omitting Asthma analysis type (COPD)** |  | 507.204 | (115) | -85.439 | *** |  |  |
|  |  | **Estimate** | **S.E.** | **P** | **RR** | **95%CIl** | **95%CIu** |
| **Constant** |  | 1.014 | 0.140 | +++ | 2.755 | 2.094 | 3.626 |
| **RR adjusted for factor other than sex, age** |  |  |  |  |  |  |  |
| Yes | 34 | Aliased |  |  | 1.894 | 1.630 | 2.201 |
| No | 95 | 0.286 | 0.051 | +++ | 2.521 | 2.343 | 2.712 |
| **Sex(RR)** |  |  |  |  |  |  |  |
| Male | 49 | Aliased |  |  | 2.513 | 2.246 | 2.813 |
| Female | 35 | -0.267 | 0.044 | --- | 1.925 | 1.711 | 2.166 |
| Combined | 45 | -0.021 | 0.038 | N.S. | 2.461 | 2.274 | 2.664 |
| **Continent** |  |  |  |  |  |  |  |
| NAmer | 35 | Aliased |  |  | 2.823 | 2.505 | 3.182 |
| Europe | 59 | -0.271 | 0.038 | --- | 2.153 | 2.005 | 2.313 |
| Asia | 23 | -0.153 | 0.065 | - | 2.423 | 2.001 | 2.933 |
| oth/mult | 12 | -0.274 | 0.071 | --- | 2.147 | 1.699 | 2.712 |
| **Smoking product (3)** |  |  |  |  |  |  |  |
| any | 48 | Aliased |  |  | 1.572 | 1.141 | 2.165 |
| cigs | 73 | 0.551 | 0.126 | +++ | 2.728 | 2.308 | 3.225 |
| cigsonly | 8 | 0.668 | 0.102 | +++ | 3.067 | 1.939 | 4.850 |

Table 1 - A - 6

IESCOPD - Meta-regression of ever smoking, any product (or cigarettes if all product not available)

Multiple regression of data from Table 1 - A - 1 (most-adjusted RRs)

Any COPD

Test by removing variables one at a time

|  |  |  |  |  |  |  |  |
| --- | --- | --- | --- | --- | --- | --- | --- |
|  |  | **Estimate** | **S.E.** | **P** | **RR** | **95%CIl** | **95%CIu** |
| **Unexposed group** |  |  |  |  |  |  |  |
| nev any | 57 | Aliased |  |  | 3.812 | 2.828 | 5.138 |
| nev cig | 72 | -0.761 | 0.122 | --- | 1.781 | 1.501 | 2.113 |
| **COPD subtype** |  |  |  |  |  |  |  |
| mort | 29 | Aliased |  |  | 2.934 | 2.217 | 3.883 |
| LF | 60 | -0.314 | 0.082 | --- | 2.143 | 1.993 | 2.305 |
| other | 40 | -0.064 | 0.082 | N.S. | 2.754 | 2.426 | 3.125 |
| **Midpoint age in RR** |  | 0.003 | 0.002 | N.S. | 1.968 | 1.249 | 3.099 |
| **RR adjusted for age** |  |  |  |  |  |  |  |
| Yes | 69 | Aliased |  |  | 2.255 | 2.022 | 2.515 |
| No | 60 | 0.059 | 0.043 | N.S. | 2.393 | 2.200 | 2.603 |
|  |  | **Deviance** | **(DF)** | **Drop Dev** | **P** |  |  |
| **Omitting Smoking product (3)** |  | 461.008 | (114) | -39.243 | ** |  |  |
|  |  | **Estimate** | **S.E.** | **P** | **RR** | **95%CIl** | **95%CIu** |
| **Constant** |  | 1.336 | 0.135 | +++ | 3.803 | 2.916 | 4.959 |
| **RR adjusted for factor other than sex, age** |  |  |  |  |  |  |  |
| Yes | 34 | Aliased |  |  | 2.072 | 1.793 | 2.393 |
| No | 95 | 0.164 | 0.049 | ++ | 2.440 | 2.271 | 2.622 |
| **Sex(RR)** |  |  |  |  |  |  |  |
| Male | 49 | Aliased |  |  | 2.385 | 2.132 | 2.669 |
| Female | 35 | -0.122 | 0.042 | -- | 2.112 | 1.881 | 2.370 |
| Combined | 45 | 0.015 | 0.039 | N.S. | 2.421 | 2.234 | 2.625 |
| **Continent** |  |  |  |  |  |  |  |
| NAmer | 35 | Aliased |  |  | 2.880 | 2.560 | 3.240 |
| Europe | 59 | -0.251 | 0.039 | --- | 2.240 | 2.077 | 2.415 |
| Asia | 23 | -0.363 | 0.065 | --- | 2.003 | 1.636 | 2.453 |
| oth/mult | 12 | -0.415 | 0.070 | --- | 1.901 | 1.496 | 2.416 |
| **Asthma analysis type (COPD)** |  |  |  |  |  |  |  |
| inc-irresp | 76 | Aliased |  |  | 2.527 | 2.351 | 2.717 |
| excl-all | 21 | -0.146 | 0.043 | -- | 2.183 | 1.907 | 2.498 |
| defn-incl | 19 | -0.537 | 0.059 | --- | 1.477 | 1.201 | 1.816 |
| other | 13 | 0.210 | 0.089 | + | 3.116 | 2.249 | 4.317 |
| **Unexposed group** |  |  |  |  |  |  |  |
| nev any | 57 | Aliased |  |  | 2.698 | 2.440 | 2.983 |
| nev cig | 72 | -0.224 | 0.035 | --- | 2.156 | 2.009 | 2.315 |
| **COPD subtype** |  |  |  |  |  |  |  |
| mort | 29 | Aliased |  |  | 3.961 | 3.149 | 4.983 |
| LF | 60 | -0.652 | 0.068 | --- | 2.064 | 1.917 | 2.223 |
| other | 40 | -0.364 | 0.067 | --- | 2.751 | 2.407 | 3.145 |
| **Midpoint age in RR** |  | 0.006 | 0.002 | ++ | 1.637 | 1.039 | 2.579 |
| **RR adjusted for age** |  |  |  |  |  |  |  |
| Yes | 69 | Aliased |  |  | 2.307 | 2.061 | 2.582 |
| No | 60 | 0.021 | 0.045 | N.S. | 2.356 | 2.161 | 2.568 |
|  |  | **Deviance** | **(DF)** | **Drop Dev** | **P** |  |  |
| **Omitting Unexposed group** |  | 445.621 | (113) | -23.855 | * |  |  |
|  |  | **Estimate** | **S.E.** | **P** | **RR** | **95%CIl** | **95%CIu** |
| **Constant** |  | 1.156 | 0.141 | +++ | 3.178 | 2.411 | 4.188 |
| **RR adjusted for factor other than sex, age** |  |  |  |  |  |  |  |
| Yes | 34 | Aliased |  |  | 2.160 | 1.866 | 2.501 |
| No | 95 | 0.106 | 0.049 | + | 2.403 | 2.235 | 2.584 |
| **Sex(RR)** |  |  |  |  |  |  |  |
| Male | 49 | Aliased |  |  | 2.460 | 2.193 | 2.758 |
| Female | 35 | -0.160 | 0.043 | --- | 2.097 | 1.865 | 2.357 |
| Combined | 45 | -0.028 | 0.039 | N.S. | 2.392 | 2.208 | 2.592 |
| **Continent** |  |  |  |  |  |  |  |
| NAmer | 35 | Aliased |  |  | 2.891 | 2.567 | 3.256 |
| Europe | 59 | -0.252 | 0.039 | --- | 2.248 | 2.084 | 2.425 |
| Asia | 23 | -0.387 | 0.065 | --- | 1.962 | 1.603 | 2.402 |
| oth/mult | 12 | -0.433 | 0.071 | --- | 1.874 | 1.473 | 2.385 |

Table 1 - A - 6

IESCOPD - Meta-regression of ever smoking, any product (or cigarettes if all product not available)

Multiple regression of data from Table 1 - A - 1 (most-adjusted RRs)

Any COPD

Test by removing variables one at a time

|  |  |  |  |  |  |  |  |
| --- | --- | --- | --- | --- | --- | --- | --- |
|  |  | **Estimate** | **S.E.** | **P** | **RR** | **95%CIl** | **95%CIu** |
| **Asthma analysis type (COPD)** |  |  |  |  |  |  |  |
| inc-irresp | 76 | Aliased |  |  | 2.512 | 2.335 | 2.702 |
| excl-all | 21 | -0.164 | 0.043 | --- | 2.131 | 1.862 | 2.440 |
| defn-incl | 19 | -0.479 | 0.060 | --- | 1.556 | 1.260 | 1.923 |
| other | 13 | 0.334 | 0.092 | +++ | 3.509 | 2.505 | 4.914 |
| **Smoking product (3)** |  |  |  |  |  |  |  |
| any | 48 | Aliased |  |  | 2.545 | 2.281 | 2.839 |
| cigs | 73 | -0.169 | 0.037 | --- | 2.150 | 2.001 | 2.310 |
| cigsonly | 8 | 0.565 | 0.106 | +++ | 4.479 | 3.037 | 6.606 |
| **COPD subtype** |  |  |  |  |  |  |  |
| mort | 29 | Aliased |  |  | 2.985 | 2.196 | 4.059 |
| LF | 60 | -0.329 | 0.091 | --- | 2.148 | 1.984 | 2.324 |
| other | 40 | -0.093 | 0.085 | N.S. | 2.720 | 2.377 | 3.113 |
| **Midpoint age in RR** |  | 0.004 | 0.002 | + | 1.816 | 1.140 | 2.892 |
| **RR adjusted for age** |  |  |  |  |  |  |  |
| Yes | 69 | Aliased |  |  | 2.274 | 2.030 | 2.548 |
| No | 60 | 0.045 | 0.045 | N.S. | 2.379 | 2.181 | 2.595 |
|  |  | **Deviance** | **(DF)** | **Drop Dev** | **P** |  |  |
| **Omitting COPD subtype** |  | 463.993 | (114) | -42.228 | ** |  |  |
|  |  | **Estimate** | **S.E.** | **P** | **RR** | **95%CIl** | **95%CIu** |
| **Constant** |  | 0.936 | 0.122 | +++ | 2.550 | 2.009 | 3.237 |
| **RR adjusted for factor other than sex, age** |  |  |  |  |  |  |  |
| Yes | 34 | Aliased |  |  | 2.192 | 1.893 | 2.539 |
| No | 95 | 0.086 | 0.050 | (+) | 2.390 | 2.223 | 2.570 |
| **Sex(RR)** |  |  |  |  |  |  |  |
| Male | 49 | Aliased |  |  | 2.496 | 2.234 | 2.789 |
| Female | 35 | -0.207 | 0.044 | --- | 2.029 | 1.796 | 2.293 |
| Combined | 45 | -0.035 | 0.038 | N.S. | 2.410 | 2.224 | 2.613 |
| **Continent** |  |  |  |  |  |  |  |
| NAmer | 35 | Aliased |  |  | 2.796 | 2.475 | 3.157 |
| Europe | 59 | -0.208 | 0.040 | --- | 2.271 | 2.106 | 2.449 |
| Asia | 23 | -0.306 | 0.066 | --- | 2.058 | 1.681 | 2.520 |
| oth/mult | 12 | -0.441 | 0.071 | --- | 1.799 | 1.423 | 2.274 |
| **Asthma analysis type (COPD)** |  |  |  |  |  |  |  |
| inc-irresp | 76 | Aliased |  |  | 2.454 | 2.284 | 2.636 |
| excl-all | 21 | -0.164 | 0.043 | --- | 2.083 | 1.823 | 2.379 |
| defn-incl | 19 | -0.312 | 0.056 | --- | 1.796 | 1.477 | 2.185 |
| other | 13 | 0.529 | 0.084 | +++ | 4.166 | 3.058 | 5.673 |
| **Smoking product (3)** |  |  |  |  |  |  |  |
| any | 48 | Aliased |  |  | 1.981 | 1.448 | 2.712 |
| cigs | 73 | 0.192 | 0.122 | N.S. | 2.400 | 2.043 | 2.821 |
| cigsonly | 8 | 0.814 | 0.082 | +++ | 4.472 | 3.086 | 6.479 |
| **Unexposed group** |  |  |  |  |  |  |  |
| nev any | 57 | Aliased |  |  | 3.034 | 2.258 | 4.075 |
| nev cig | 72 | -0.406 | 0.120 | --- | 2.021 | 1.706 | 2.394 |
| **Midpoint age in RR** |  | 0.004 | 0.002 | (+) | 1.848 | 1.161 | 2.941 |
| **RR adjusted for age** |  |  |  |  |  |  |  |
| Yes | 69 | Aliased |  |  | 2.329 | 2.083 | 2.605 |
| No | 60 | 0.005 | 0.044 | N.S. | 2.340 | 2.148 | 2.550 |
|  |  | **Deviance** | **(DF)** | **Drop Dev** | **P** |  |  |
| **Omitting Midpoint age in RR** |  | 424.246 | (113) | -2.480 | N.S. |  |  |
|  |  | **Estimate** | **S.E.** | **P** | **RR** | **95%CIl** | **95%CIu** |
| **Constant** |  | 1.325 | 0.086 | +++ | 3.764 | 3.183 | 4.451 |
| **RR adjusted for factor other than sex, age** |  |  |  |  |  |  |  |
| Yes | 34 | Aliased |  |  | 2.000 | 1.718 | 2.327 |
| No | 95 | 0.212 | 0.051 | +++ | 2.472 | 2.295 | 2.661 |

Table 1 - A - 6

IESCOPD - Meta-regression of ever smoking, any product (or cigarettes if all product not available)

Multiple regression of data from Table 1 - A - 1 (most-adjusted RRs)

Any COPD

Test by removing variables one at a time

|  |  |  |  |  |  |  |  |
| --- | --- | --- | --- | --- | --- | --- | --- |
|  |  | **Estimate** | **S.E.** | **P** | **RR** | **95%CIl** | **95%CIu** |
| **Sex(RR)** |  |  |  |  |  |  |  |
| Male | 49 | Aliased |  |  | 2.471 | 2.205 | 2.770 |
| Female | 35 | -0.216 | 0.044 | --- | 1.992 | 1.760 | 2.253 |
| Combined | 45 | -0.011 | 0.039 | N.S. | 2.444 | 2.251 | 2.653 |
| **Continent** |  |  |  |  |  |  |  |
| NAmer | 35 | Aliased |  |  | 2.738 | 2.425 | 3.092 |
| Europe | 59 | -0.191 | 0.040 | --- | 2.262 | 2.096 | 2.440 |
| Asia | 23 | -0.256 | 0.063 | --- | 2.120 | 1.748 | 2.571 |
| oth/mult | 12 | -0.347 | 0.072 | --- | 1.935 | 1.519 | 2.466 |
| **Asthma analysis type (COPD)** |  |  |  |  |  |  |  |
| inc-irresp | 76 | Aliased |  |  | 2.496 | 2.320 | 2.685 |
| excl-all | 21 | -0.145 | 0.043 | -- | 2.158 | 1.885 | 2.471 |
| defn-incl | 19 | -0.449 | 0.060 | --- | 1.593 | 1.291 | 1.965 |
| other | 13 | 0.307 | 0.092 | ++ | 3.393 | 2.426 | 4.745 |
| **Smoking product (3)** |  |  |  |  |  |  |  |
| any | 48 | Aliased |  |  | 1.672 | 1.212 | 2.307 |
| cigs | 73 | 0.461 | 0.126 | +++ | 2.653 | 2.240 | 3.142 |
| cigsonly | 8 | 0.616 | 0.105 | +++ | 3.095 | 1.907 | 5.023 |
| **Unexposed group** |  |  |  |  |  |  |  |
| nev any | 57 | Aliased |  |  | 3.486 | 2.572 | 4.725 |
| nev cig | 72 | -0.622 | 0.123 | --- | 1.871 | 1.572 | 2.227 |
| **COPD subtype** |  |  |  |  |  |  |  |
| mort | 29 | Aliased |  |  | 3.183 | 2.336 | 4.338 |
| LF | 60 | -0.414 | 0.092 | --- | 2.104 | 1.941 | 2.280 |
| other | 40 | -0.122 | 0.085 | N.S. | 2.818 | 2.457 | 3.233 |
| **RR adjusted for age** |  |  |  |  |  |  |  |
| Yes | 69 | Aliased |  |  | 2.278 | 2.033 | 2.552 |
| No | 60 | 0.042 | 0.045 | N.S. | 2.376 | 2.179 | 2.592 |
|  |  | **Deviance** | **(DF)** | **Drop Dev** | **P** |  |  |
| **Omitting RR adjusted for age** |  | 422.825 | (113) | -1.060 | N.S. |  |  |
|  |  | **Estimate** | **S.E.** | **P** | **RR** | **95%CIl** | **95%CIu** |
| **Constant** |  | 1.146 | 0.141 | +++ | 3.145 | 2.386 | 4.144 |
| **RR adjusted for factor other than sex, age** |  |  |  |  |  |  |  |
| Yes | 34 | Aliased |  |  | 1.985 | 1.732 | 2.275 |
| No | 95 | 0.222 | 0.045 | +++ | 2.478 | 2.311 | 2.658 |
| **Sex(RR)** |  |  |  |  |  |  |  |
| Male | 49 | Aliased |  |  | 2.463 | 2.196 | 2.761 |
| Female | 35 | -0.213 | 0.044 | --- | 1.991 | 1.760 | 2.252 |
| Combined | 45 | -0.006 | 0.039 | N.S. | 2.448 | 2.255 | 2.658 |
| **Continent** |  |  |  |  |  |  |  |
| NAmer | 35 | Aliased |  |  | 2.754 | 2.435 | 3.113 |
| Europe | 59 | -0.196 | 0.040 | --- | 2.264 | 2.098 | 2.443 |
| Asia | 23 | -0.290 | 0.067 | --- | 2.061 | 1.678 | 2.531 |
| oth/mult | 12 | -0.335 | 0.070 | --- | 1.969 | 1.555 | 2.493 |
| **Asthma analysis type (COPD)** |  |  |  |  |  |  |  |
| inc-irresp | 76 | Aliased |  |  | 2.493 | 2.319 | 2.680 |
| excl-all | 21 | -0.132 | 0.042 | -- | 2.185 | 1.914 | 2.494 |
| defn-incl | 19 | -0.465 | 0.060 | --- | 1.566 | 1.269 | 1.934 |
| other | 13 | 0.295 | 0.092 | ++ | 3.348 | 2.387 | 4.694 |
| **Smoking product (3)** |  |  |  |  |  |  |  |
| any | 48 | Aliased |  |  | 1.718 | 1.240 | 2.380 |
| cigs | 73 | 0.423 | 0.128 | ++ | 2.623 | 2.211 | 3.113 |
| cigsonly | 8 | 0.580 | 0.106 | +++ | 3.068 | 1.891 | 4.977 |
| **Unexposed group** |  |  |  |  |  |  |  |
| nev any | 57 | Aliased |  |  | 3.442 | 2.536 | 4.672 |
| nev cig | 72 | -0.602 | 0.123 | --- | 1.884 | 1.582 | 2.244 |
| **COPD subtype** |  |  |  |  |  |  |  |
| mort | 29 | Aliased |  |  | 3.112 | 2.292 | 4.226 |
| LF | 60 | -0.387 | 0.091 | --- | 2.113 | 1.950 | 2.288 |
| other | 40 | -0.102 | 0.084 | N.S. | 2.809 | 2.449 | 3.222 |
| **Midpoint age in RR** |  | 0.003 | 0.002 | N.S. | 1.938 | 1.215 | 3.093 |

Table 1 - A - 6

IESCOPD - Meta-regression of ever smoking, any product (or cigarettes if all product not available)

Multiple regression of data from Table 1 - A - 1 (most-adjusted RRs)

Any COPD

Test by removing variables one at a time

|  |  |  |  |  |  |  |  |
| --- | --- | --- | --- | --- | --- | --- | --- |
|  |  | **Deviance** | **(DF)** | **Drop Dev** | **P** |  |  |
| **Omitting RR adjusted for factor other than sex, age** |  | 435.549 | (113) | -13.783 | (*) |  |  |
|  |  | **Estimate** | **S.E.** | **P** | **RR** | **95%CIl** | **95%CIu** |
| **Constant** |  | 1.151 | 0.141 | +++ | 3.162 | 2.399 | 4.168 |
| **RR adjusted for age** |  |  |  |  |  |  |  |
| Yes | 69 | Aliased |  |  | 2.162 | 1.953 | 2.393 |
| No | 60 | 0.130 | 0.039 | ++ | 2.463 | 2.274 | 2.667 |
| **Sex(RR)** |  |  |  |  |  |  |  |
| Male | 49 | Aliased |  |  | 2.457 | 2.191 | 2.756 |
| Female | 35 | -0.193 | 0.044 | --- | 2.027 | 1.794 | 2.290 |
| Combined | 45 | -0.011 | 0.039 | N.S. | 2.431 | 2.240 | 2.638 |
| **Continent** |  |  |  |  |  |  |  |
| NAmer | 35 | Aliased |  |  | 2.856 | 2.534 | 3.218 |
| Europe | 59 | -0.229 | 0.040 | --- | 2.272 | 2.105 | 2.451 |
| Asia | 23 | -0.379 | 0.064 | --- | 1.955 | 1.603 | 2.385 |
| oth/mult | 12 | -0.464 | 0.066 | --- | 1.796 | 1.428 | 2.259 |
| **Asthma analysis type (COPD)** |  |  |  |  |  |  |  |
| inc-irresp | 76 | Aliased |  |  | 2.503 | 2.327 | 2.693 |
| excl-all | 21 | -0.149 | 0.043 | --- | 2.157 | 1.883 | 2.470 |
| defn-incl | 19 | -0.485 | 0.060 | --- | 1.542 | 1.249 | 1.903 |
| other | 13 | 0.342 | 0.092 | +++ | 3.526 | 2.525 | 4.924 |
| **Smoking product (3)** |  |  |  |  |  |  |  |
| any | 48 | Aliased |  |  | 1.911 | 1.407 | 2.597 |
| cigs | 73 | 0.259 | 0.119 | + | 2.477 | 2.110 | 2.908 |
| cigsonly | 8 | 0.624 | 0.106 | +++ | 3.567 | 2.249 | 5.656 |
| **Unexposed group** |  |  |  |  |  |  |  |
| nev any | 57 | Aliased |  |  | 3.111 | 2.334 | 4.147 |
| nev cig | 72 | -0.445 | 0.116 | --- | 1.993 | 1.690 | 2.351 |
| **COPD subtype** |  |  |  |  |  |  |  |
| mort | 29 | Aliased |  |  | 3.051 | 2.242 | 4.153 |
| LF | 60 | -0.352 | 0.091 | --- | 2.145 | 1.983 | 2.320 |
| other | 40 | -0.120 | 0.085 | N.S. | 2.707 | 2.374 | 3.087 |
| **Midpoint age in RR** |  | 0.005 | 0.002 | + | 1.749 | 1.107 | 2.764 |

Table 1 - A - 6

IESCOPD - Meta-regression of ever smoking, any product (or cigarettes if all product not available)

Multiple regression of data from Table 1 - A - 1 (most-adjusted RRs)

Any COPD

Test reduction to 2-level product

|  |  | **Deviance** | **(DF)** | **Drop Dev** | **P** |  |  |
| --- | --- | --- | --- | --- | --- | --- | --- |
| **Reducing Smoking Product to 2 levels** |  | 422.780 | (113) | -1.015 | N.S. |  |  |
|  |  | **Estimate** | **S.E.** | **P** | **RR** | **95%CIl** | **95%CIu** |
| **Constant** |  | 1.179 | 0.138 | +++ | 3.250 | 2.481 | 4.258 |
| **Sex(RR)** |  |  |  |  |  |  |  |
| Male | 49 | Aliased |  |  | 2.459 | 2.193 | 2.757 |
| Female | 35 | -0.214 | 0.044 | --- | 1.984 | 1.756 | 2.242 |
| Combined | 45 | -0.002 | 0.039 | N.S. | 2.454 | 2.262 | 2.662 |
| **Continent** |  |  |  |  |  |  |  |
| NAmer | 35 | Aliased |  |  | 2.749 | 2.434 | 3.104 |
| Europe | 59 | -0.193 | 0.040 | --- | 2.266 | 2.100 | 2.445 |
| Asia | 23 | -0.284 | 0.066 | --- | 2.070 | 1.687 | 2.540 |
| oth/mult | 12 | -0.342 | 0.071 | --- | 1.952 | 1.534 | 2.485 |
| **Asthma analysis type (COPD)** |  |  |  |  |  |  |  |
| inc-irresp | 76 | Aliased |  |  | 2.503 | 2.328 | 2.693 |
| excl-all | 21 | -0.142 | 0.043 | -- | 2.172 | 1.896 | 2.487 |
| defn-incl | 19 | -0.469 | 0.060 | --- | 1.567 | 1.269 | 1.934 |
| other | 13 | 0.258 | 0.090 | ++ | 3.242 | 2.335 | 4.501 |
| **Smoking product (2)** |  |  |  |  |  |  |  |
| any | 48 | Aliased |  |  | 1.611 | 1.275 | 2.035 |
| cigs | 81 | 0.524 | 0.085 | +++ | 2.721 | 2.444 | 3.030 |
| **Unexposed group** |  |  |  |  |  |  |  |
| nev any | 57 | Aliased |  |  | 3.652 | 2.957 | 4.510 |
| nev cig | 72 | -0.694 | 0.084 | --- | 1.824 | 1.610 | 2.066 |
| **COPD subtype** |  |  |  |  |  |  |  |
| mort | 29 | Aliased |  |  | 3.306 | 2.561 | 4.269 |
| LF | 60 | -0.459 | 0.075 | --- | 2.090 | 1.940 | 2.252 |
| other | 40 | -0.154 | 0.075 | - | 2.833 | 2.474 | 3.244 |
| **Midpoint age in RR** |  | 0.003 | 0.002 | N.S. | 1.932 | 1.210 | 3.083 |
| **RR adjusted for age** |  |  |  |  |  |  |  |
| Yes | 69 | Aliased |  |  | 2.274 | 2.030 | 2.548 |
| No | 60 | 0.045 | 0.045 | N.S. | 2.379 | 2.181 | 2.595 |
| **RR adjusted for factor other than sex, age** |  |  |  |  |  |  |  |
| Yes | 34 | Aliased |  |  | 1.998 | 1.726 | 2.314 |
| No | 95 | 0.213 | 0.049 | +++ | 2.472 | 2.299 | 2.658 |

Table 1 - A - 6

IESCOPD - Meta-regression of ever smoking, any product (or cigarettes if all product not available)

Multiple regression of data from Table 1 - A - 1 (most-adjusted RRs)

Any COPD

Test reduction to 2-level outcome subtype

|  |  | **Deviance** | **(DF)** | **Drop Dev** | **P** |  |  |
| --- | --- | --- | --- | --- | --- | --- | --- |
| **Reducing COPD subtype to 2 levels** |  | 423.557 | (113) | -1.792 | N.S. |  |  |
|  |  | **Estimate** | **S.E.** | **P** | **RR** | **95%CIl** | **95%CIu** |
| **Constant** |  | 0.760 | 0.125 | +++ | 2.139 | 1.675 | 2.732 |
| **Sex(RR)** |  |  |  |  |  |  |  |
| Male | 49 | Aliased |  |  | 2.485 | 2.223 | 2.779 |
| Female | 35 | -0.221 | 0.044 | --- | 1.992 | 1.761 | 2.254 |
| Combined | 45 | -0.020 | 0.038 | N.S. | 2.436 | 2.246 | 2.642 |
| **Continent** |  |  |  |  |  |  |  |
| NAmer | 35 | Aliased |  |  | 2.785 | 2.465 | 3.148 |
| Europe | 59 | -0.210 | 0.040 | --- | 2.259 | 2.094 | 2.437 |
| Asia | 23 | -0.313 | 0.066 | --- | 2.037 | 1.662 | 2.497 |
| oth/mult | 12 | -0.352 | 0.072 | --- | 1.959 | 1.540 | 2.492 |
| **Asthma analysis type (COPD)** |  |  |  |  |  |  |  |
| inc-irresp | 76 | Aliased |  |  | 2.491 | 2.317 | 2.679 |
| excl-all | 21 | -0.139 | 0.043 | -- | 2.169 | 1.894 | 2.484 |
| defn-incl | 19 | -0.448 | 0.060 | --- | 1.592 | 1.291 | 1.963 |
| other | 13 | 0.310 | 0.091 | +++ | 3.396 | 2.433 | 4.739 |
| **Smoking product (3)** |  |  |  |  |  |  |  |
| any | 48 | Aliased |  |  | 1.722 | 1.243 | 2.386 |
| cigs | 73 | 0.413 | 0.127 | ++ | 2.603 | 2.198 | 3.082 |
| cigsonly | 8 | 0.676 | 0.085 | +++ | 3.384 | 2.251 | 5.088 |
| **Unexposed group** |  |  |  |  |  |  |  |
| nev any | 57 | Aliased |  |  | 3.425 | 2.524 | 4.647 |
| nev cig | 72 | -0.595 | 0.123 | --- | 1.890 | 1.587 | 2.250 |
| **COPD subtype (2)** |  |  |  |  |  |  |  |
| LF | 60 | Aliased |  |  | 2.123 | 1.964 | 2.294 |
| other | 69 | 0.297 | 0.047 | +++ | 2.857 | 2.506 | 3.257 |
| **Midpoint age in RR** |  | 0.004 | 0.002 | N.S. | 1.907 | 1.195 | 3.041 |
| **RR adjusted for age** |  |  |  |  |  |  |  |
| Yes | 69 | Aliased |  |  | 2.283 | 2.040 | 2.556 |
| No | 60 | 0.038 | 0.044 | N.S. | 2.372 | 2.176 | 2.587 |
| **RR adjusted for factor other than sex, age** |  |  |  |  |  |  |  |
| Yes | 34 | Aliased |  |  | 2.023 | 1.733 | 2.362 |
| No | 95 | 0.196 | 0.052 | +++ | 2.461 | 2.284 | 2.652 |

Table 1 - A - 6

IESCOPD - Meta-regression of ever smoking, any product (or cigarettes if all product not available)

Multiple regression of data from Table 1 - A - 1 (most-adjusted RRs)

Any COPD

Test by adding extra variables one at a time

|  |  | **Deviance** | **(DF)** | **Drop Dev** | **P** |  |  |
| --- | --- | --- | --- | --- | --- | --- | --- |
| **Increasing COPD subtype to 4 levels** |  | 407.599 | (111) | 14.166 | (*) |  |  |
|  |  | **Estimate** | **S.E.** | **P** | **RR** | **95%CIl** | **95%CIu** |
| **Constant** |  | 1.164 | 0.141 | +++ | 3.201 | 2.429 | 4.220 |
| **Sex(RR)** |  |  |  |  |  |  |  |
| Male | 49 | Aliased |  |  | 2.495 | 2.226 | 2.798 |
| Female | 35 | -0.217 | 0.044 | --- | 2.008 | 1.776 | 2.270 |
| Combined | 45 | -0.030 | 0.040 | N.S. | 2.423 | 2.232 | 2.630 |
| **Continent** |  |  |  |  |  |  |  |
| NAmer | 35 | Aliased |  |  | 2.755 | 2.437 | 3.115 |
| Europe | 59 | -0.199 | 0.040 | --- | 2.258 | 2.094 | 2.435 |
| Asia | 23 | -0.273 | 0.068 | --- | 2.097 | 1.709 | 2.573 |
| oth/mult | 12 | -0.345 | 0.072 | --- | 1.952 | 1.535 | 2.482 |
| **Asthma analysis type (COPD)** |  |  |  |  |  |  |  |
| inc-irresp | 76 | Aliased |  |  | 2.496 | 2.321 | 2.683 |
| excl-all | 21 | -0.155 | 0.044 | --- | 2.138 | 1.867 | 2.448 |
| defn-incl | 19 | -0.412 | 0.062 | --- | 1.654 | 1.333 | 2.051 |
| other | 13 | 0.274 | 0.093 | ++ | 3.282 | 2.343 | 4.597 |
| **Smoking product (3)** |  |  |  |  |  |  |  |
| any | 48 | Aliased |  |  | 1.683 | 1.217 | 2.327 |
| cigs | 73 | 0.452 | 0.128 | +++ | 2.645 | 2.232 | 3.134 |
| cigsonly | 8 | 0.612 | 0.107 | +++ | 3.105 | 1.921 | 5.019 |
| **Unexposed group** |  |  |  |  |  |  |  |
| nev any | 57 | Aliased |  |  | 3.441 | 2.541 | 4.658 |
| nev cig | 72 | -0.602 | 0.123 | --- | 1.885 | 1.585 | 2.242 |
| **COPD subtype** |  |  |  |  |  |  |  |
| mort | 29 | Aliased |  |  |  |  |  |
| LF | 60 | Aliased |  |  |  |  |  |
| other | 40 | Aliased |  |  |  |  |  |
|  |  | **Estimate** | **S.E.** | **P** | **RR** | **95%CIl** | **95%CIu** |
| **Midpoint age in RR** |  | 0.003 | 0.002 | N.S. | 2.018 | 1.266 | 3.218 |
| **RR adjusted for age** |  |  |  |  |  |  |  |
| Yes | 69 | Aliased |  |  | 2.271 | 2.029 | 2.542 |
| No | 60 | 0.048 | 0.045 | N.S. | 2.381 | 2.185 | 2.596 |
| **RR adjusted for factor other than sex, age** |  |  |  |  |  |  |  |
| Yes | 34 | Aliased |  |  | 2.002 | 1.716 | 2.336 |
| No | 95 | 0.210 | 0.053 | +++ | 2.470 | 2.293 | 2.661 |
| **COPD subtype (4)** |  |  |  |  |  |  |  |
| mort | 29 | Aliased |  |  | 3.119 | 2.293 | 4.243 |
| LF | 60 | -0.389 | 0.093 | --- | 2.115 | 1.952 | 2.291 |
| oth-prev | 32 | -0.145 | 0.085 | (-) | 2.697 | 2.338 | 3.112 |
| oth-inc | 8 | 0.245 | 0.128 | (+) | 3.984 | 2.746 | 5.780 |

Table 1 - A - 6

IESCOPD - Meta-regression of ever smoking, any product (or cigarettes if all product not available)

Multiple regression of data from Table 1 - A - 1 (most-adjusted RRs)

Any COPD

Test by adding extra variables one at a time

|  |  | **Deviance** | **(DF)** | **Drop Dev** | **P** |  |  |
| --- | --- | --- | --- | --- | --- | --- | --- |
| **Adding National cigarette tobacco type** |  | 404.486 | (110) | 17.279 | N.S. |  |  |
|  |  | **Estimate** | **S.E.** | **P** | **RR** | **95%CIl** | **95%CIu** |
| **Constant** |  | 1.346 | 0.153 | +++ | 3.841 | 2.843 | 5.189 |
| **Sex(RR)** |  |  |  |  |  |  |  |
| Male | 49 | Aliased |  |  | 2.487 | 2.217 | 2.790 |
| Female | 35 | -0.215 | 0.044 | --- | 2.005 | 1.772 | 2.268 |
| Combined | 45 | -0.024 | 0.040 | N.S. | 2.428 | 2.235 | 2.638 |
| **Continent** |  |  |  |  |  |  |  |
| NAmer | 35 | Aliased |  |  | 2.676 | 2.356 | 3.040 |
| Europe | 59 | -0.196 | 0.042 | --- | 2.201 | 2.022 | 2.395 |
| Asia | 23 | -0.061 | 0.090 | N.S. | 2.517 | 1.886 | 3.359 |
| oth/mult | 12 | -0.272 | 0.075 | --- | 2.039 | 1.592 | 2.612 |
| **Asthma analysis type (COPD)** |  |  |  |  |  |  |  |
| inc-irresp | 76 | Aliased |  |  | 2.513 | 2.337 | 2.703 |
| excl-all | 21 | -0.161 | 0.045 | --- | 2.139 | 1.854 | 2.467 |
| defn-incl | 19 | -0.452 | 0.068 | --- | 1.600 | 1.259 | 2.033 |
| other | 13 | 0.230 | 0.094 | + | 3.164 | 2.249 | 4.451 |
| **Smoking product (3)** |  |  |  |  |  |  |  |
| any | 48 | Aliased |  |  | 1.785 | 1.288 | 2.474 |
| cigs | 73 | 0.364 | 0.129 | ++ | 2.568 | 2.165 | 3.047 |
| cigsonly | 8 | 0.604 | 0.107 | +++ | 3.267 | 2.013 | 5.302 |
| **Unexposed group** |  |  |  |  |  |  |  |
| nev any | 57 | Aliased |  |  | 3.310 | 2.439 | 4.493 |
| nev cig | 72 | -0.542 | 0.124 | --- | 1.926 | 1.617 | 2.293 |
| **COPD subtype** |  |  |  |  |  |  |  |
| mort | 29 | Aliased |  |  | 3.165 | 2.326 | 4.305 |
| LF | 60 | -0.405 | 0.092 | --- | 2.112 | 1.949 | 2.288 |
| other | 40 | -0.124 | 0.085 | N.S. | 2.795 | 2.437 | 3.206 |
| **Midpoint age in RR** |  | 0.001 | 0.002 | N.S. | 2.184 | 1.331 | 3.585 |
| **RR adjusted for age** |  |  |  |  |  |  |  |
| Yes | 69 | Aliased |  |  | 2.234 | 1.991 | 2.507 |
| No | 60 | 0.075 | 0.046 | N.S. | 2.408 | 2.206 | 2.629 |
| **RR adjusted for factor other than sex, age** |  |  |  |  |  |  |  |
| Yes | 34 | Aliased |  |  | 2.151 | 1.823 | 2.539 |
| No | 95 | 0.112 | 0.057 | (+) | 2.407 | 2.227 | 2.601 |
| **National cigarette tobacco type** |  |  |  |  |  |  |  |
| bl | 96 | Aliased |  |  | 2.423 | 2.267 | 2.590 |
| vir | 21 | -0.047 | 0.060 | N.S. | 2.312 | 1.880 | 2.843 |
| m/u | 12 | -0.350 | 0.086 | --- | 1.707 | 1.265 | 2.303 |

Table 1 - A - 6

IESCOPD - Meta-regression of ever smoking, any product (or cigarettes if all product not available)

Multiple regression of data from Table 1 - A - 1 (most-adjusted RRs)

Any COPD

Test by adding extra variables one at a time

|  |  | **Deviance** | **(DF)** | **Drop Dev** | **P** |  |  |
| --- | --- | --- | --- | --- | --- | --- | --- |
| **Adding Publication year** |  | 417.391 | (109) | 4.375 | N.S. |  |  |
|  |  | **Estimate** | **S.E.** | **P** | **RR** | **95%CIl** | **95%CIu** |
| **Constant** |  | 1.240 | 0.151 | +++ | 3.454 | 2.570 | 4.643 |
| **Sex(RR)** |  |  |  |  |  |  |  |
| Male | 49 | Aliased |  |  | 2.459 | 2.189 | 2.764 |
| Female | 35 | -0.220 | 0.045 | --- | 1.974 | 1.740 | 2.240 |
| Combined | 45 | 0.000 | 0.040 | N.S. | 2.460 | 2.261 | 2.675 |
| **Continent** |  |  |  |  |  |  |  |
| NAmer | 35 | Aliased |  |  | 2.753 | 2.426 | 3.124 |
| Europe | 59 | -0.190 | 0.041 | --- | 2.277 | 2.106 | 2.461 |
| Asia | 23 | -0.308 | 0.069 | --- | 2.024 | 1.632 | 2.510 |
| oth/mult | 12 | -0.356 | 0.073 | --- | 1.929 | 1.503 | 2.474 |
| **Asthma analysis type (COPD)** |  |  |  |  |  |  |  |
| inc-irresp | 76 | Aliased |  |  | 2.506 | 2.327 | 2.699 |
| excl-all | 21 | -0.142 | 0.043 | -- | 2.174 | 1.894 | 2.495 |
| defn-incl | 19 | -0.493 | 0.063 | --- | 1.531 | 1.226 | 1.912 |
| other | 13 | 0.293 | 0.093 | ++ | 3.360 | 2.378 | 4.749 |
| **Smoking product (3)** |  |  |  |  |  |  |  |
| any | 48 | Aliased |  |  | 1.682 | 1.206 | 2.347 |
| cigs | 73 | 0.454 | 0.129 | +++ | 2.650 | 2.226 | 3.154 |
| cigsonly | 8 | 0.587 | 0.108 | +++ | 3.027 | 1.849 | 4.955 |
| **Unexposed group** |  |  |  |  |  |  |  |
| nev any | 57 | Aliased |  |  | 3.476 | 2.547 | 4.746 |
| nev cig | 72 | -0.618 | 0.124 | --- | 1.874 | 1.568 | 2.239 |
| **COPD subtype** |  |  |  |  |  |  |  |
| mort | 29 | Aliased |  |  | 3.049 | 2.175 | 4.274 |
| LF | 60 | -0.367 | 0.100 | --- | 2.112 | 1.939 | 2.301 |
| other | 40 | -0.074 | 0.090 | N.S. | 2.832 | 2.447 | 3.278 |
| **Midpoint age in RR** |  | 0.004 | 0.002 | N.S. | 1.907 | 1.185 | 3.069 |
| **RR adjusted for age** |  |  |  |  |  |  |  |
| Yes | 69 | Aliased |  |  | 2.243 | 1.993 | 2.524 |
| No | 60 | 0.068 | 0.046 | N.S. | 2.402 | 2.195 | 2.628 |
| **RR adjusted for factor other than sex, age** |  |  |  |  |  |  |  |
| Yes | 34 | Aliased |  |  | 2.058 | 1.747 | 2.425 |
| No | 95 | 0.173 | 0.055 | ++ | 2.446 | 2.263 | 2.644 |
| **Publication year** |  |  |  |  |  |  |  |
| <1980 | 25 | Aliased |  |  | 2.694 | 1.999 | 3.630 |
| 1980-89 | 10 | -0.069 | 0.121 | N.S. | 2.514 | 1.765 | 3.582 |
| 1990-99 | 19 | -0.141 | 0.088 | N.S. | 2.340 | 1.807 | 3.029 |
| 2000+ | 75 | -0.154 | 0.083 | (-) | 2.308 | 2.170 | 2.456 |
|  |  | **Deviance** | **(DF)** | **Drop Dev** | **P** |  |  |
| **Adding Study type** |  | 411.557 | (110) | 10.208 | N.S. |  |  |
|  |  | **Estimate** | **S.E.** | **P** | **RR** | **95%CIl** | **95%CIu** |
| **Constant** |  | 1.049 | 0.177 | +++ | 2.855 | 2.017 | 4.043 |
| **Sex(RR)** |  |  |  |  |  |  |  |
| Male | 49 | Aliased |  |  | 2.486 | 2.215 | 2.790 |
| Female | 35 | -0.216 | 0.044 | --- | 2.004 | 1.771 | 2.268 |
| Combined | 45 | -0.023 | 0.040 | N.S. | 2.429 | 2.236 | 2.639 |
| **Continent** |  |  |  |  |  |  |  |
| NAmer | 35 | Aliased |  |  | 2.767 | 2.444 | 3.133 |
| Europe | 59 | -0.203 | 0.040 | --- | 2.259 | 2.093 | 2.438 |
| Asia | 23 | -0.284 | 0.068 | --- | 2.084 | 1.695 | 2.562 |
| oth/mult | 12 | -0.360 | 0.073 | --- | 1.931 | 1.514 | 2.463 |
| **Asthma analysis type (COPD)** |  |  |  |  |  |  |  |
| inc-irresp | 76 | Aliased |  |  | 2.496 | 2.318 | 2.687 |
| excl-all | 21 | -0.151 | 0.044 | --- | 2.146 | 1.872 | 2.460 |
| defn-incl | 19 | -0.413 | 0.064 | --- | 1.651 | 1.320 | 2.065 |
| other | 13 | 0.255 | 0.094 | ++ | 3.220 | 2.285 | 4.537 |

Table 1 - A - 6

IESCOPD - Meta-regression of ever smoking, any product (or cigarettes if all product not available)

Multiple regression of data from Table 1 - A - 1 (most-adjusted RRs)

Any COPD

Fixed model, test by adding extra variables one at a time

|  |  | **Estimate** | **S.E.** | **P** | **RR** | **95%CIl** | **95%CIu** |
| --- | --- | --- | --- | --- | --- | --- | --- |
| **Smoking product (3)** |  |  |  |  |  |  |  |
| any | 48 | Aliased |  |  | 1.694 | 1.221 | 2.349 |
| cigs | 73 | 0.443 | 0.128 | +++ | 2.637 | 2.222 | 3.130 |
| cigsonly | 8 | 0.607 | 0.107 | +++ | 3.108 | 1.912 | 5.052 |
| **Unexposed group** |  |  |  |  |  |  |  |
| nev any | 57 | Aliased |  |  | 3.437 | 2.531 | 4.667 |
| nev cig | 72 | -0.600 | 0.123 | --- | 1.886 | 1.583 | 2.247 |
| **COPD subtype** |  |  |  |  |  |  |  |
| mort | 29 | Aliased |  |  | 2.521 | 1.675 | 3.795 |
| LF | 60 | -0.150 | 0.122 | N.S. | 2.171 | 1.988 | 2.371 |
| other | 40 | 0.106 | 0.109 | N.S. | 2.803 | 2.442 | 3.219 |
| **Midpoint age in RR** |  | 0.003 | 0.002 | N.S. | 1.949 | 1.219 | 3.115 |
| **RR adjusted for age** |  |  |  |  |  |  |  |
| Yes | 69 | Aliased |  |  | 2.254 | 2.010 | 2.528 |
| No | 60 | 0.060 | 0.045 | N.S. | 2.394 | 2.193 | 2.612 |
| **RR adjusted for factor other than sex, age** |  |  |  |  |  |  |  |
| Yes | 34 | Aliased |  |  | 2.030 | 1.737 | 2.372 |
| No | 95 | 0.191 | 0.053 | +++ | 2.458 | 2.280 | 2.650 |
| **Study type** |  |  |  |  |  |  |  |
| CC | 16 | Aliased |  |  | 2.638 | 1.798 | 3.871 |
| Pr | 37 | 0.111 | 0.109 | N.S. | 2.947 | 2.219 | 3.914 |
| CS | 76 | -0.152 | 0.105 | N.S. | 2.265 | 2.124 | 2.416 |
|  |  | **Deviance** | **(DF)** | **Drop Dev** | **P** |  |  |
| **Adding Study weakness** |  | 421.763 | (111) | 0.002 | N.S. |  |  |
|  |  | **Estimate** | **S.E.** | **P** | **RR** | **95%CIl** | **95%CIu** |
| **Constant** |  | 1.145 | 0.163 | +++ | 3.144 | 2.285 | 4.326 |
| **Sex(RR)** |  |  |  |  |  |  |  |
| Male | 49 | Aliased |  |  | 2.462 | 2.193 | 2.765 |
| Female | 35 | -0.212 | 0.044 | --- | 1.993 | 1.759 | 2.258 |
| Combined | 45 | -0.006 | 0.039 | N.S. | 2.447 | 2.252 | 2.660 |
| **Continent** |  |  |  |  |  |  |  |
| NAmer | 35 | Aliased |  |  | 2.766 | 2.441 | 3.134 |
| Europe | 59 | -0.200 | 0.040 | --- | 2.264 | 2.096 | 2.444 |
| Asia | 23 | -0.295 | 0.067 | --- | 2.058 | 1.673 | 2.533 |
| oth/mult | 12 | -0.355 | 0.073 | --- | 1.940 | 1.519 | 2.478 |
| **Asthma analysis type (COPD)** |  |  |  |  |  |  |  |
| inc-irresp | 76 | Aliased |  |  | 2.499 | 2.320 | 2.692 |
| excl-all | 21 | -0.142 | 0.044 | -- | 2.168 | 1.888 | 2.489 |
| defn-incl | 19 | -0.461 | 0.061 | --- | 1.576 | 1.272 | 1.952 |
| other | 13 | 0.283 | 0.093 | ++ | 3.318 | 2.355 | 4.674 |
| **Smoking product (3)** |  |  |  |  |  |  |  |
| any | 48 | Aliased |  |  | 1.712 | 1.231 | 2.379 |
| cigs | 73 | 0.428 | 0.128 | ++ | 2.627 | 2.210 | 3.121 |
| cigsonly | 8 | 0.589 | 0.107 | +++ | 3.085 | 1.892 | 5.030 |
| **Unexposed group** |  |  |  |  |  |  |  |
| nev any | 57 | Aliased |  |  | 3.443 | 2.530 | 4.687 |
| nev cig | 72 | -0.603 | 0.123 | --- | 1.884 | 1.579 | 2.247 |
| **COPD subtype** |  |  |  |  |  |  |  |
| mort | 29 | Aliased |  |  | 3.157 | 2.297 | 4.340 |
| LF | 60 | -0.404 | 0.094 | --- | 2.107 | 1.943 | 2.285 |
| other | 40 | -0.115 | 0.087 | N.S. | 2.815 | 2.449 | 3.236 |
| **Midpoint age in RR** |  | 0.003 | 0.002 | N.S. | 1.924 | 1.195 | 3.098 |
| **RR adjusted for age** |  |  |  |  |  |  |  |
| Yes | 69 | Aliased |  |  | 2.273 | 2.026 | 2.549 |
| No | 60 | 0.046 | 0.045 | N.S. | 2.380 | 2.180 | 2.599 |
| **RR adjusted for factor other than sex, age** |  |  |  |  |  |  |  |
| Yes | 34 | Aliased |  |  | 2.025 | 1.732 | 2.369 |
| No | 95 | 0.194 | 0.053 | +++ | 2.460 | 2.281 | 2.653 |

Table 1 - A - 6

IESCOPD - Meta-regression of ever smoking, any product (or cigarettes if all product not available)

Multiple regression of data from Table 1 - A - 1 (most-adjusted RRs)

Any COPD

Fixed model, test by adding extra variables one at a time

|  |  | **Estimate** | **S.E.** | **P** | **RR** | **95%CIl** | **95%CIu** |
| --- | --- | --- | --- | --- | --- | --- | --- |
| **Study weakness** |  |  |  |  |  |  |  |
| Yes | 16 | Aliased |  |  | 2.328 | 1.733 | 3.128 |
| No | 113 | 0.004 | 0.079 | N.S. | 2.336 | 2.212 | 2.467 |
|  |  | **Deviance** | **(DF)** | **Drop Dev** | **P** |  |  |
| **Adding Bronchodiliator/**  **reversibility (LF only)** |  | 416.132 | (111) | 5.633 | N.S. |  |  |
|  |  | **Estimate** | **S.E.** | **P** | **RR** | **95%CIl** | **95%CIu** |
| **Constant** |  | 1.276 | 0.151 | +++ | 3.582 | 2.666 | 4.813 |
| **Sex(RR)** |  |  |  |  |  |  |  |
| Male | 49 | Aliased |  |  | 2.470 | 2.202 | 2.771 |
| Female | 35 | -0.209 | 0.044 | --- | 2.005 | 1.771 | 2.270 |
| Combined | 45 | -0.014 | 0.039 | N.S. | 2.436 | 2.243 | 2.646 |
| **Continent** |  |  |  |  |  |  |  |
| NAmer | 35 | Aliased |  |  | 2.801 | 2.470 | 3.176 |
| Europe | 59 | -0.208 | 0.040 | --- | 2.275 | 2.107 | 2.456 |
| Asia | 23 | -0.292 | 0.067 | --- | 2.092 | 1.700 | 2.574 |
| oth/mult | 12 | -0.499 | 0.095 | --- | 1.701 | 1.233 | 2.345 |
| **Asthma analysis type (COPD)** |  |  |  |  |  |  |  |
| inc-irresp | 76 | Aliased |  |  | 2.487 | 2.310 | 2.677 |
| excl-all | 21 | -0.135 | 0.044 | -- | 2.174 | 1.897 | 2.491 |
| defn-incl | 19 | -0.452 | 0.061 | --- | 1.583 | 1.280 | 1.957 |
| other | 13 | 0.341 | 0.096 | +++ | 3.497 | 2.463 | 4.966 |
| **Smoking product (3)** |  |  |  |  |  |  |  |
| any | 48 | Aliased |  |  | 1.735 | 1.250 | 2.409 |
| cigs | 73 | 0.407 | 0.128 | ++ | 2.607 | 2.195 | 3.095 |
| cigsonly | 8 | 0.598 | 0.107 | +++ | 3.155 | 1.939 | 5.132 |
| **Unexposed group** |  |  |  |  |  |  |  |
| nev any | 57 | Aliased |  |  | 3.415 | 2.514 | 4.640 |
| nev cig | 72 | -0.590 | 0.124 | --- | 1.893 | 1.588 | 2.255 |
| **COPD subtype** |  |  |  |  |  |  |  |
| mort | 29 | Aliased |  |  | 3.157 | 2.314 | 4.308 |
| LF | 60 | -0.403 | 0.092 | --- | 2.109 | 1.946 | 2.287 |
| other | 40 | -0.118 | 0.085 | N.S. | 2.806 | 2.445 | 3.221 |
| **Midpoint age in RR** |  | 0.001 | 0.002 | N.S. | 2.144 | 1.301 | 3.534 |
| **RR adjusted for age** |  |  |  |  |  |  |  |
| Yes | 69 | Aliased |  |  | 2.261 | 2.017 | 2.534 |
| No | 60 | 0.055 | 0.045 | N.S. | 2.389 | 2.189 | 2.607 |
| **RR adjusted for factor other than sex, age** |  |  |  |  |  |  |  |
| Yes | 34 | Aliased |  |  | 2.056 | 1.757 | 2.406 |
| No | 95 | 0.174 | 0.053 | ++ | 2.447 | 2.269 | 2.639 |
| **Bronchodilator/**  **reversibility (LF only)** |  |  |  |  |  |  |  |
| no/na | 117 | Aliased |  |  | 2.313 | 2.188 | 2.444 |
| yes/revs | 12 | 0.245 | 0.103 | + | 2.956 | 2.021 | 4.323 |
|  |  | **Deviance** | **(DF)** | **Drop Dev** | **P** |  |  |
| **Adding Number of cases (COPD)** |  | 404.917 | (109) | 16.848 | N.S. |  |  |
|  |  | **Estimate** | **S.E.** | **P** | **RR** | **95%CIl** | **95%CIu** |
| **Constant** |  | 1.637 | 0.188 | +++ | 5.139 | 3.553 | 7.435 |
| **Sex(RR)** |  |  |  |  |  |  |  |
| Male | 49 | Aliased |  |  | 2.465 | 2.198 | 2.764 |
| Female | 35 | -0.217 | 0.044 | --- | 1.985 | 1.754 | 2.246 |
| Combined | 45 | -0.006 | 0.039 | N.S. | 2.450 | 2.257 | 2.660 |
| **Continent** |  |  |  |  |  |  |  |
| NAmer | 35 | Aliased |  |  | 2.771 | 2.448 | 3.137 |
| Europe | 59 | -0.196 | 0.041 | --- | 2.279 | 2.110 | 2.462 |
| Asia | 23 | -0.315 | 0.068 | --- | 2.022 | 1.646 | 2.484 |
| oth/mult | 12 | -0.397 | 0.079 | --- | 1.863 | 1.426 | 2.433 |

Table 1 - A - 6

IESCOPD - Meta-regression of ever smoking, any product (or cigarettes if all product not available)

Multiple regression of data from Table 1 - A - 1 (most-adjusted RRs)

Any COPD

Test by adding extra variables one at a time

|  |  | **Estimate** | **S.E.** | **P** | **RR** | **95%CIl** | **95%CIu** |
| --- | --- | --- | --- | --- | --- | --- | --- |
| **Asthma analysis type (COPD)** |  |  |  |  |  |  |  |
| inc-irresp | 76 | Aliased |  |  | 2.496 | 2.320 | 2.686 |
| excl-all | 21 | -0.137 | 0.044 | -- | 2.176 | 1.898 | 2.495 |
| defn-incl | 19 | -0.466 | 0.060 | --- | 1.566 | 1.268 | 1.935 |
| other | 13 | 0.296 | 0.093 | ++ | 3.357 | 2.390 | 4.715 |
| **Smoking product (3)** |  |  |  |  |  |  |  |
| any | 48 | Aliased |  |  | 1.716 | 1.239 | 2.376 |
| cigs | 73 | 0.421 | 0.128 | ++ | 2.614 | 2.204 | 3.100 |
| cigsonly | 8 | 0.645 | 0.109 | +++ | 3.269 | 2.002 | 5.336 |
| **Unexposed group** |  |  |  |  |  |  |  |
| nev any | 57 | Aliased |  |  | 3.443 | 2.539 | 4.670 |
| nev cig | 72 | -0.603 | 0.123 | --- | 1.884 | 1.582 | 2.243 |
| **COPD subtype** |  |  |  |  |  |  |  |
| mort | 29 | Aliased |  |  | 3.013 | 2.201 | 4.124 |
| LF | 60 | -0.350 | 0.094 | --- | 2.123 | 1.957 | 2.302 |
| other | 40 | -0.072 | 0.086 | N.S. | 2.803 | 2.442 | 3.217 |
| **Midpoint age in RR** |  | 0.003 | 0.002 | N.S. | 1.939 | 1.207 | 3.117 |
| **RR adjusted for age** |  |  |  |  |  |  |  |
| Yes | 69 | Aliased |  |  | 2.275 | 2.030 | 2.549 |
| No | 60 | 0.045 | 0.045 | N.S. | 2.379 | 2.180 | 2.595 |
| **RR adjusted for factor other than sex, age** |  |  |  |  |  |  |  |
| Yes | 34 | Aliased |  |  | 2.055 | 1.755 | 2.407 |
| No | 95 | 0.174 | 0.054 | ++ | 2.447 | 2.269 | 2.639 |
| **Number of cases (COPD)** |  |  |  |  |  |  |  |
| 1-50 | 23 | Aliased |  |  | 3.877 | 2.317 | 6.488 |
| 51-100 | 29 | -0.506 | 0.152 | -- | 2.337 | 1.798 | 3.038 |
| 101-200 | 30 | -0.424 | 0.146 | -- | 2.538 | 2.074 | 3.106 |
| 201+ | 47 | -0.522 | 0.137 | --- | 2.302 | 2.171 | 2.440 |
|  |  | **Deviance** | **(DF)** | **Drop Dev** | **P** |  |  |
| **Adding Smoking results reported in study (COPD)** |  | 420.119 | (111) | 1.647 | N.S. |  |  |
|  |  | **Estimate** | **S.E.** | **P** | **RR** | **95%CIl** | **95%CIu** |
| **Constant** |  | 1.091 | 0.148 | +++ | 2.976 | 2.227 | 3.978 |
| **Sex(RR)** |  |  |  |  |  |  |  |
| Male | 49 | Aliased |  |  | 2.465 | 2.197 | 2.767 |
| Female | 35 | -0.216 | 0.044 | --- | 1.987 | 1.754 | 2.250 |
| Combined | 45 | -0.007 | 0.039 | N.S. | 2.449 | 2.255 | 2.660 |
| **Continent** |  |  |  |  |  |  |  |
| NAmer | 35 | Aliased |  |  | 2.728 | 2.392 | 3.111 |
| Europe | 59 | -0.186 | 0.042 | --- | 2.265 | 2.098 | 2.446 |
| Asia | 23 | -0.260 | 0.073 | --- | 2.104 | 1.694 | 2.614 |
| oth/mult | 12 | -0.329 | 0.075 | --- | 1.963 | 1.534 | 2.511 |
| **Asthma analysis type (COPD)** |  |  |  |  |  |  |  |
| inc-irresp | 76 | Aliased |  |  | 2.509 | 2.329 | 2.703 |
| excl-all | 21 | -0.145 | 0.043 | -- | 2.170 | 1.893 | 2.489 |
| defn-incl | 19 | -0.483 | 0.063 | --- | 1.548 | 1.243 | 1.928 |
| other | 13 | 0.252 | 0.096 | ++ | 3.229 | 2.272 | 4.589 |
| **Smoking product (3)** |  |  |  |  |  |  |  |
| any | 48 | Aliased |  |  | 1.699 | 1.222 | 2.362 |
| cigs | 73 | 0.441 | 0.128 | +++ | 2.639 | 2.220 | 3.136 |
| cigsonly | 8 | 0.578 | 0.107 | +++ | 3.029 | 1.854 | 4.948 |
| **Unexposed group** |  |  |  |  |  |  |  |
| nev any | 57 | Aliased |  |  | 3.451 | 2.537 | 4.695 |
| nev cig | 72 | -0.607 | 0.124 | --- | 1.882 | 1.578 | 2.244 |
| **COPD subtype** |  |  |  |  |  |  |  |
| mort | 29 | Aliased |  |  | 3.170 | 2.319 | 4.333 |
| LF | 60 | -0.412 | 0.093 | --- | 2.100 | 1.936 | 2.279 |
| other | 40 | -0.111 | 0.085 | N.S. | 2.837 | 2.466 | 3.264 |
| **Midpoint age in RR** |  | 0.003 | 0.002 | N.S. | 1.957 | 1.219 | 3.143 |

Table 1 - A - 6

IESCOPD - Meta-regression of ever smoking, any product (or cigarettes if all product not available)

Multiple regression of data from Table 1 - A - 1 (most-adjusted RRs)

Any COPD

Test by adding extra variables one at a time

|  |  | **Estimate** | **S.E.** | **P** | **RR** | **95%CIl** | **95%CIu** |
| --- | --- | --- | --- | --- | --- | --- | --- |
| **RR adjusted for age** |  |  |  |  |  |  |  |
| Yes | 69 | Aliased |  |  | 2.310 | 2.040 | 2.615 |
| No | 60 | 0.019 | 0.050 | N.S. | 2.354 | 2.143 | 2.585 |
| **RR adjusted for factor other than sex, age** |  |  |  |  |  |  |  |
| Yes | 34 | Aliased |  |  | 1.995 | 1.697 | 2.346 |
| No | 95 | 0.215 | 0.055 | +++ | 2.473 | 2.290 | 2.672 |
| **Smoking results reported in study (COPD)** |  |  |  |  |  |  |  |
| Ever Only | 18 | Aliased |  |  | 2.184 | 1.777 | 2.685 |
| Current Only | 0 | Aliased |  |  | 2.184 | 1.777 | 2.685 |
| Both | 111 | 0.075 | 0.058 | N.S. | 2.354 | 2.221 | 2.495 |
|  |  | **Deviance** | **(DF)** | **Drop Dev** | **P** |  |  |
| **Adding Derivation of RR/CI** |  | 421.749 | (111) | 0.016 | N.S. |  |  |
|  |  | **Estimate** | **S.E.** | **P** | **RR** | **95%CIl** | **95%CIu** |
| **Constant** |  | 1.160 | 0.165 | +++ | 3.190 | 2.307 | 4.411 |
| **Sex(RR)** |  |  |  |  |  |  |  |
| Male | 49 | Aliased |  |  | 2.463 | 2.193 | 2.766 |
| Female | 35 | -0.211 | 0.044 | --- | 1.994 | 1.759 | 2.260 |
| Combined | 45 | -0.007 | 0.040 | N.S. | 2.446 | 2.250 | 2.660 |
| **Continent** |  |  |  |  |  |  |  |
| NAmer | 35 | Aliased |  |  | 2.768 | 2.439 | 3.140 |
| Europe | 59 | -0.201 | 0.041 | --- | 2.263 | 2.093 | 2.446 |
| Asia | 23 | -0.295 | 0.067 | --- | 2.060 | 1.673 | 2.536 |
| oth/mult | 12 | -0.355 | 0.072 | --- | 1.941 | 1.520 | 2.479 |
| **Asthma analysis type (COPD)** |  |  |  |  |  |  |  |
| inc-irresp | 76 | Aliased |  |  | 2.499 | 2.320 | 2.691 |
| excl-all | 21 | -0.142 | 0.044 | -- | 2.169 | 1.889 | 2.489 |
| defn-incl | 19 | -0.460 | 0.061 | --- | 1.577 | 1.272 | 1.954 |
| other | 13 | 0.283 | 0.093 | ++ | 3.317 | 2.354 | 4.673 |
| **Smoking product (3)** |  |  |  |  |  |  |  |
| any | 48 | Aliased |  |  | 1.713 | 1.231 | 2.383 |
| cigs | 73 | 0.427 | 0.128 | ++ | 2.626 | 2.209 | 3.121 |
| cigsonly | 8 | 0.589 | 0.107 | +++ | 3.088 | 1.893 | 5.036 |
| **Unexposed group** |  |  |  |  |  |  |  |
| nev any | 57 | Aliased |  |  | 3.442 | 2.529 | 4.686 |
| nev cig | 72 | -0.602 | 0.124 | --- | 1.884 | 1.579 | 2.248 |
| **COPD subtype** |  |  |  |  |  |  |  |
| mort | 29 | Aliased |  |  | 3.154 | 2.305 | 4.314 |
| LF | 60 | -0.403 | 0.093 | --- | 2.107 | 1.943 | 2.286 |
| other | 40 | -0.113 | 0.085 | N.S. | 2.815 | 2.451 | 3.234 |
| **Midpoint age in RR** |  | 0.003 | 0.002 | N.S. | 1.931 | 1.194 | 3.123 |
| **RR adjusted for age** |  |  |  |  |  |  |  |
| Yes | 69 | Aliased |  |  | 2.274 | 2.026 | 2.551 |
| No | 60 | 0.046 | 0.045 | N.S. | 2.379 | 2.179 | 2.599 |
| **RR adjusted for factor other than sex, age** |  |  |  |  |  |  |  |
| Yes | 34 | Aliased |  |  | 2.025 | 1.732 | 2.368 |
| No | 95 | 0.195 | 0.052 | +++ | 2.460 | 2.281 | 2.653 |
| **Derivation of RR/CI** |  |  |  |  |  |  |  |
| Orig/2x2 | 18 | Aliased |  |  | 2.352 | 1.892 | 2.924 |
| Other | 111 | -0.008 | 0.059 | N.S. | 2.335 | 2.208 | 2.468 |
|  |  | **Deviance** | **(DF)** | **Drop Dev** | **P** |  |  |
| **Adding Analysis type** |  | 412.717 | (111) | 9.049 | N.S. |  |  |
|  |  | **Estimate** | **S.E.** | **P** | **RR** | **95%CIl** | **95%CIu** |
| **Constant** |  | 0.926 | 0.159 | +++ | 2.526 | 1.849 | 3.450 |
| **Sex(RR)** |  |  |  |  |  |  |  |
| Male | 49 | Aliased |  |  | 2.482 | 2.213 | 2.784 |
| Female | 35 | -0.214 | 0.044 | --- | 2.004 | 1.772 | 2.267 |
| Combined | 45 | -0.021 | 0.040 | N.S. | 2.431 | 2.239 | 2.640 |

Table 1 - A - 6

IESCOPD - Meta-regression of ever smoking, any product (or cigarettes if all product not available)

Multiple regression of data from Table 1 - A - 1 (most-adjusted RRs)

Any COPD

Test by adding extra variables one at a time

|  |  | **Estimate** | **S.E.** | **P** | **RR** | **95%CIl** | **95%CIu** |
| --- | --- | --- | --- | --- | --- | --- | --- |
| **Continent** |  |  |  |  |  |  |  |
| NAmer | 35 | Aliased |  |  | 2.756 | 2.435 | 3.119 |
| Europe | 59 | -0.198 | 0.040 | --- | 2.261 | 2.096 | 2.439 |
| Asia | 23 | -0.278 | 0.068 | --- | 2.088 | 1.699 | 2.565 |
| oth/mult | 12 | -0.349 | 0.072 | --- | 1.944 | 1.526 | 2.475 |
| **Asthma analysis type (COPD)** |  |  |  |  |  |  |  |
| inc-irresp | 76 | Aliased |  |  | 2.488 | 2.313 | 2.677 |
| excl-all | 21 | -0.147 | 0.043 | --- | 2.148 | 1.874 | 2.460 |
| defn-incl | 19 | -0.399 | 0.064 | --- | 1.670 | 1.335 | 2.088 |
| other | 13 | 0.273 | 0.093 | ++ | 3.270 | 2.328 | 4.591 |
| **Smoking product (3)** |  |  |  |  |  |  |  |
| any | 48 | Aliased |  |  | 1.696 | 1.224 | 2.350 |
| cigs | 73 | 0.440 | 0.128 | +++ | 2.634 | 2.220 | 3.124 |
| cigsonly | 8 | 0.618 | 0.107 | +++ | 3.146 | 1.939 | 5.104 |
| **Unexposed group** |  |  |  |  |  |  |  |
| nev any | 57 | Aliased |  |  | 3.445 | 2.539 | 4.673 |
| nev cig | 72 | -0.604 | 0.123 | --- | 1.884 | 1.582 | 2.243 |
| **COPD subtype** |  |  |  |  |  |  |  |
| mort | 29 | Aliased |  |  | 2.591 | 1.743 | 3.851 |
| LF | 60 | -0.179 | 0.119 | N.S. | 2.167 | 1.985 | 2.365 |
| other | 40 | 0.074 | 0.106 | N.S. | 2.791 | 2.433 | 3.203 |
| **Midpoint age in RR** |  | 0.003 | 0.002 | N.S. | 1.948 | 1.221 | 3.108 |
| **RR adjusted for age** |  |  |  |  |  |  |  |
| Yes | 69 | Aliased |  |  | 2.249 | 2.006 | 2.521 |
| No | 60 | 0.064 | 0.045 | N.S. | 2.397 | 2.197 | 2.616 |
| **RR adjusted for factor other than sex, age** |  |  |  |  |  |  |  |
| Yes | 34 | Aliased |  |  | 2.038 | 1.745 | 2.379 |
| No | 95 | 0.186 | 0.052 | +++ | 2.455 | 2.278 | 2.645 |
| **Analysis type** |  |  |  |  |  |  |  |
| prevlnce | 93 | Aliased |  |  | 2.280 | 2.145 | 2.423 |
| onset | 36 | 0.240 | 0.080 | ++ | 2.898 | 2.199 | 3.821 |

Table 1 - A - 6

IESCOPD - Meta-regression of ever smoking, any product (or cigarettes if all product not available)

Multiple regression of data from Table 1 - A - 1 (most-adjusted RRs)

Any COPD

Fitted values and residuals

|  | **Study Ref** | **NRR** | **SEX** | **LOGRR** | **FITVAL** | **SEFITV** | **STDRES** |
| --- | --- | --- | --- | --- | --- | --- | --- |
|  |  |  |  |  |  |  |  |
| #42 | XU | 2 | m | 0.231 | 0.790 | 0.160 | -3.487 |
| #413 | EKBERG | 3 | m | 0.743 | 0.977 | 0.114 | -2.050 |
| #292 | MANNI3 | 3 | b | 0.384 | 1.124 | 0.387 | -1.912 |
| #1168 | MENEZ3 | 3 | b | 0.104 | 0.682 | 0.324 | -1.786 |
| #330 | MONTNE | 3 | b | 0.873 | 1.200 | 0.236 | -1.390 |
| #2983 | VESTBO | 3 | b | 0.645 | 0.835 | 0.143 | -1.331 |
| #475 | DEAN1 | 17 | f | 0.652 | 1.090 | 0.333 | -1.316 |
| #2253 | ZIELI2 | 6 | f | 0.338 | 0.448 | 0.085 | -1.306 |
| #97 | HO | 3 | m | 0.720 | 1.371 | 0.499 | -1.305 |
| #3548 | SILVA | 3 | b | 0.464 | 1.052 | 0.498 | -1.182 |
| #327 | LAM2 | 3 | m | 0.987 | 1.354 | 0.329 | -1.114 |
| #534 | HIGGI4 | 3 | m | 1.125 | 1.677 | 0.574 | -0.961 |
| #57 | LINDST | 9 | b | 0.610 | 0.802 | 0.200 | -0.957 |
| #2246 | TODD | 46 | f | -0.117 | 1.127 | 1.374 | -0.905 |
| #147 | VIEGI2 | 12 | f | 0.182 | 0.518 | 0.401 | -0.837 |
| #466 | HARIKK | 3 | m | 0.720 | 1.365 | 0.780 | -0.827 |
| #37 | LAM3 | 3 | m | 0.593 | 1.228 | 0.772 | -0.821 |
| #141 | VIEGI2 | 6 | m | 0.329 | 0.729 | 0.508 | -0.788 |
| #184 | MANNI2 | 3 | b | 0.971 | 1.098 | 0.164 | -0.778 |
| #379 | BEDNAR | 3 | b | 0.455 | 0.852 | 0.535 | -0.741 |
| #182 | ANDER1 | 6 | f | 0.166 | 0.721 | 0.792 | -0.702 |
| #93 | KARAKA | 3 | b | 0.787 | 1.118 | 0.550 | -0.601 |
| #531 | MARCUS | 3 | m | 0.775 | 1.101 | 0.581 | -0.560 |
| #1184 | MENEZ5 | 3 | b | 0.495 | 0.682 | 0.345 | -0.544 |
| #2318 | LEE | 52 | f | 0.554 | 1.127 | 1.054 | -0.543 |
| #2823 | LEBOWI | 18 | f | 0.507 | 0.738 | 0.437 | -0.530 |
| #2350 | HAMMO2 | 6 | f | 1.770 | 1.946 | 0.338 | -0.523 |
| #1440 | SHAHAB | 3 | b | 0.762 | 0.830 | 0.137 | -0.492 |
| #490 | SARGEA | 9 | b | 0.336 | 0.390 | 0.116 | -0.466 |
| #1176 | MENEZ4 | 3 | b | 0.501 | 0.682 | 0.458 | -0.396 |
| #103 | ALESSA | 3 | b | 0.727 | 1.106 | 0.994 | -0.382 |
| #390 | KRZYZA | 6 | f | 0.344 | 0.525 | 0.533 | -0.340 |
| #508 | KATANC | 3 | b | 0.891 | 1.054 | 0.517 | -0.314 |
| #100 | HO | 6 | f | 0.999 | 1.160 | 0.533 | -0.302 |
| #561 | MUELLE | 13 | f | 0.470 | 0.876 | 1.799 | -0.226 |
| #456 | FORAST | 6 | f | 0.859 | 0.960 | 0.461 | -0.219 |
| #394 | ZIELI1 | 2 | b | 0.723 | 0.750 | 0.129 | -0.211 |
| #422 | NILSSO | 9 | m | 1.022 | 1.144 | 0.608 | -0.199 |
| #197 | CLEMEN | 3 | m | 0.813 | 0.920 | 0.644 | -0.166 |
| #362 | YUAN | 5 | m | 0.336 | 0.412 | 0.552 | -0.137 |
| #428 | NIHLEN | 3 | b | 1.160 | 1.214 | 0.465 | -0.115 |
| #176 | PEREZP | 1 | f | 0.264 | 0.334 | 0.621 | -0.113 |
| #1453 | LAI | 2 | f | 0.570 | 0.644 | 0.850 | -0.087 |
| #15 | MENEZ2 | 6 | b | 0.432 | 0.441 | 0.345 | -0.028 |
| #376 | ANDER3 | 6 | f | 0.693 | 0.766 | 2.853 | -0.025 |
| #530 | MANNI1 | 10 | f | 1.157 | 1.157 | 0.195 | -0.000 |
| #34 | WEISS | 3 | m | 1.229 | 1.184 | 1.448 | 0.031 |
| #371 | JACOBS | 6 | m | 1.125 | 1.111 | 0.408 | 0.034 |
| #434 | STROM | 3 | m | 1.035 | 1.014 | 0.560 | 0.037 |
| #154 | ITABAS | 1 | b | 0.675 | 0.632 | 0.759 | 0.056 |
| #169 | KIM | 6 | f | 0.554 | 0.500 | 0.709 | 0.076 |
| #2286 | LEE | 20 | m | 1.554 | 1.338 | 1.957 | 0.110 |
| #425 | LINDBE | 3 | b | 0.824 | 0.757 | 0.525 | 0.128 |
| #348 | DEMARC | 6 | b | 0.565 | 0.538 | 0.198 | 0.138 |
| #1335 | THUN | 5 | m | 2.193 | 2.148 | 0.293 | 0.153 |
| #135 | RICCIO | 3 | b | 0.706 | 0.670 | 0.218 | 0.164 |
| #450 | DOLL2 | 3 | f | 2.400 | 2.002 | 2.007 | 0.198 |
| #3622 | BEST | 37 | m | 2.305 | 2.145 | 0.808 | 0.198 |
| #2349 | HAMMO2 | 5 | m | 2.227 | 2.158 | 0.311 | 0.221 |
| #521 | MANNI1 | 1 | m | 0.990 | 0.940 | 0.215 | 0.230 |
| #481 | RENWIC | 3 | b | 1.196 | 1.021 | 0.759 | 0.230 |
| #599 | FERRI2 | 10 | f | 0.824 | 0.731 | 0.368 | 0.252 |

Table 1 - A - 6

IESCOPD - Meta-regression of ever smoking, any product (or cigarettes if all product not available)

Multiple regression of data from Table 1 - A - 1 (most-adjusted RRs)

Any COPD

Fitted values and residuals

|  | **Study Ref** | **NRR** | **SEX** | **LOGRR** | **FITVAL** | **SEFITV** | **STDRES** |
| --- | --- | --- | --- | --- | --- | --- | --- |
|  |  |  |  |  |  |  |  |
| #387 | KRZYZA | 3 | m | 0.967 | 0.736 | 0.873 | 0.264 |
| #166 | KIM | 3 | m | 0.837 | 0.712 | 0.469 | 0.268 |
| #553 | MUELLE | 6 | m | 1.484 | 1.088 | 1.455 | 0.272 |
| #24 | CHEN2 | 9 | m | 1.233 | 1.025 | 0.755 | 0.275 |
| #27 | CHEN2 | 12 | f | 0.944 | 0.814 | 0.455 | 0.287 |
| #2220 | TODD | 20 | m | 1.929 | 1.338 | 1.946 | 0.303 |
| #175 | SHIN | 5 | b | 0.626 | 0.464 | 0.493 | 0.329 |
| #94 | SICHLE | 1 | b | 0.775 | 0.679 | 0.286 | 0.334 |
| #1347 | HOZAWA | 3 | b | 1.019 | 0.986 | 0.085 | 0.384 |
| #1431 | MATHES | 3 | b | 1.143 | 0.925 | 0.555 | 0.392 |
| #124 | GULSVI | 2 | f | 1.258 | 1.005 | 0.619 | 0.409 |
| #2326 | TANG | 4 | m | 1.416 | 1.174 | 0.562 | 0.431 |
| #179 | ANDER1 | 3 | m | 1.635 | 0.933 | 1.483 | 0.473 |
| #40 | XIAO | 3 | b | 1.566 | 1.274 | 0.614 | 0.475 |
| #105 | COCCI | 2 | b | 2.983 | 1.373 | 2.916 | 0.552 |
| #511 | SPEIZE | 3 | m | 2.434 | 0.892 | 2.764 | 0.558 |
| #1336 | THUN | 6 | f | 2.086 | 1.936 | 0.263 | 0.568 |
| #520 | TRUPIN | 6 | b | 1.238 | 1.072 | 0.283 | 0.587 |
| #491 | HARIKK | 6 | f | 2.812 | 1.154 | 2.811 | 0.590 |
| #471 | DEAN1 | 13 | m | 0.932 | 0.712 | 0.361 | 0.609 |
| #592 | FERRI2 | 3 | m | 1.356 | 0.943 | 0.649 | 0.636 |
| #1407 | AMIGO | 11 | m | 1.537 | 0.555 | 1.535 | 0.640 |
| #571 | LEBOWI | 6 | m | 1.613 | 0.950 | 1.037 | 0.640 |
| #1427 | MADOR | 3 | m | 2.901 | 0.860 | 3.045 | 0.670 |
| #410 | NILSSO | 6 | f | 1.780 | 1.521 | 0.384 | 0.674 |
| #437 | MARAN2 | 3 | b | 1.699 | 1.348 | 0.503 | 0.699 |
| #439 | FIDAN | 2 | m | 1.940 | 0.852 | 1.548 | 0.703 |
| #160 | KOJIMA | 6 | f | 1.031 | 0.326 | 0.975 | 0.723 |
| #498 | PETO | 3 | m | 3.238 | 1.209 | 2.738 | 0.741 |
| #193 | TVERDA | 3 | m | 1.058 | 0.480 | 0.778 | 0.742 |
| #514 | SPEIZE | 6 | f | 1.435 | 0.681 | 1.008 | 0.748 |
| #579 | NIEPSU | 3 | b | 1.371 | 0.752 | 0.788 | 0.784 |
| #307 | DEJONG | 3 | b | 3.210 | 0.996 | 2.779 | 0.797 |
| #540 | DOLL1 | 3 | m | 1.878 | 1.430 | 0.558 | 0.802 |
| #373 | ANDER3 | 3 | m | 3.434 | 0.977 | 2.940 | 0.836 |
| #1192 | MENEZ6 | 3 | b | 1.004 | 0.682 | 0.385 | 0.837 |
| #382 | KACHEL | 3 | b | 1.454 | 0.769 | 0.801 | 0.855 |
| #612 | YAMAGU | 3 | b | 0.678 | 0.501 | 0.206 | 0.859 |
| #220 | LAM1 | 1 | m | 1.399 | 0.443 | 1.112 | 0.860 |
| #123 | GULSVI | 1 | m | 2.923 | 1.217 | 1.975 | 0.864 |
| #492 | KLAYTO | 1 | b | 1.750 | 1.037 | 0.824 | 0.865 |
| #319 | HUHTI1 | 14 | f | 1.058 | 0.531 | 0.569 | 0.926 |
| #416 | EKBERG | 6 | f | 0.945 | 0.766 | 0.179 | 1.001 |
| #3370 | KAHN2 | 131 | m | 1.901 | 1.535 | 0.363 | 1.007 |
| #84 | VONHER | 6 | f | 1.333 | 1.079 | 0.249 | 1.017 |
| #78 | HUHTI3 | 6 | m | 2.046 | 0.897 | 1.071 | 1.073 |
| #59 | XU | 4 | f | 0.959 | 0.579 | 0.340 | 1.120 |
| #398 | ZIETKO | 4 | f | 3.789 | 0.448 | 2.954 | 1.131 |
| #130 | HARDIE | 6 | f | 1.012 | 0.464 | 0.463 | 1.184 |
| #150 | FUKUCH | 3 | b | 1.090 | 0.741 | 0.292 | 1.193 |
| #478 | DICKIN | 3 | b | 3.237 | 0.862 | 1.979 | 1.200 |
| #157 | KOJIMA | 3 | m | 1.104 | 0.538 | 0.471 | 1.202 |
| #441 | KIRAZ | 2 | f | 1.926 | 0.703 | 1.009 | 1.212 |
| #221 | LAM1 | 2 | f | 3.281 | 0.231 | 2.509 | 1.216 |
| #6 | WILSO1 | 1 | b | 1.368 | 0.820 | 0.445 | 1.232 |
| #396 | ZIETKO | 2 | m | 4.346 | 0.660 | 2.873 | 1.283 |
| #359 | LUNDB1 | 9 | b | 1.151 | 0.569 | 0.448 | 1.300 |
| #127 | HARDIE | 3 | m | 1.692 | 0.675 | 0.747 | 1.361 |
| #188 | JOHANN | 3 | b | 1.422 | 0.786 | 0.454 | 1.400 |
| #1445 | TSUSHI | 3 | b | 1.689 | 0.559 | 0.796 | 1.421 |
| #324 | MARAN1 | 3 | b | 1.800 | 1.348 | 0.313 | 1.446 |
| #1408 | AMIGO | 12 | f | 2.064 | 0.343 | 1.113 | 1.547 |

Table 1 - A - 6

IESCOPD - Meta-regression of ever smoking, any product (or cigarettes if all product not available)

Multiple regression of data from Table 1 - A - 1 (most-adjusted RRs)

Any COPD

Fitted values and residuals

|  | **Study Ref** | **NRR** | **SEX** | **LOGRR** | **FITVAL** | **SEFITV** | **STDRES** |
| --- | --- | --- | --- | --- | --- | --- | --- |
|  |  |  |  |  |  |  |  |
| #81 | VONHER | 3 | m | 1.725 | 1.291 | 0.268 | 1.624 |
| #66 | HUHTI1 | 6 | m | 2.102 | 0.743 | 0.788 | 1.724 |
| #1452 | LAI | 1 | m | 1.727 | 0.855 | 0.488 | 1.787 |
| #2250 | ZIELI2 | 3 | m | 0.895 | 0.660 | 0.114 | 2.068 |
| #365 | GODTFR | 3 | b | 1.697 | 1.027 | 0.260 | 2.584 |

## Table 1 - B - 6

IESCOPD - Meta-regression of current smoking, any product (or cigarettes if all product not available)

Multiple regression of data from Table 1 - B - 1 (most-adjusted RRs)

Any COPD

Fixed model

|  |  | **Deviance** | **(DF)** |  |  |  |  |
| --- | --- | --- | --- | --- | --- | --- | --- |
|  |  |  |  |  |  |  |  |
| **Fixed model** |  | 433.281 | (103) |  |  |  |  |
|  |  | **Estimate** | **S.E.** | **P** | **RR** | **95%CIl** | **95%CIu** |
| **Constant** |  | 1.011 | 0.156 | +++ | 2.748 | 2.024 | 3.731 |
| **Sex(RR)** |  |  |  |  |  |  |  |
| Male | 48 | Aliased |  |  | 3.190 | 2.812 | 3.619 |
| Female | 31 | -0.218 | 0.045 | --- | 2.566 | 2.255 | 2.920 |
| Combined | 41 | -0.007 | 0.045 | N.S. | 3.169 | 2.841 | 3.534 |
| **Continent** |  |  |  |  |  |  |  |
| NAmer | 39 | Aliased |  |  | 3.870 | 3.345 | 4.478 |
| Europe | 55 | -0.347 | 0.051 | --- | 2.735 | 2.466 | 3.034 |
| Asia | 17 | -0.499 | 0.079 | --- | 2.351 | 1.787 | 3.092 |
| oth/mult | 9 | -0.510 | 0.081 | --- | 2.324 | 1.736 | 3.112 |
| **Asthma analysis type (COPD)** |  |  |  |  |  |  |  |
| inc-irresp | 72 | Aliased |  |  | 3.446 | 3.162 | 3.756 |
| excl-all | 17 | -0.334 | 0.051 | --- | 2.468 | 2.089 | 2.915 |
| defn-incl | 18 | -0.721 | 0.072 | --- | 1.676 | 1.271 | 2.212 |
| other | 13 | -0.055 | 0.103 | N.S. | 3.260 | 2.178 | 4.880 |
| **Smoking product (3)** |  |  |  |  |  |  |  |
| any | 46 | Aliased |  |  | 2.471 | 1.952 | 3.128 |
| cigs | 66 | 0.255 | 0.084 | ++ | 3.190 | 2.800 | 3.633 |
| cigsonly | 8 | 0.505 | 0.099 | +++ | 4.095 | 2.904 | 5.773 |
| **Unexposed group** |  |  |  |  |  |  |  |
| nev any | 58 | Aliased |  |  | 3.839 | 3.202 | 4.603 |
| nev cig | 62 | -0.446 | 0.077 | --- | 2.459 | 2.113 | 2.861 |
| **COPD subtype** |  |  |  |  |  |  |  |
| mort | 31 | Aliased |  |  | 3.975 | 3.119 | 5.067 |
| LF | 56 | -0.435 | 0.071 | --- | 2.574 | 2.355 | 2.813 |
| other | 33 | 0.049 | 0.076 | N.S. | 4.174 | 3.513 | 4.959 |
| **Midpoint age in RR** |  | 0.012 | 0.002 | +++ | 1.485 | 0.846 | 2.606 |
| **RR adjusted for age** |  |  |  |  |  |  |  |
| Yes | 63 | Aliased |  |  | 2.860 | 2.480 | 3.298 |
| No | 57 | 0.076 | 0.050 | N.S. | 3.086 | 2.803 | 3.398 |
| **RR adjusted for factor other than sex, age** |  |  |  |  |  |  |  |
| Yes | 27 | Aliased |  |  | 2.689 | 2.245 | 3.220 |
| No | 93 | 0.148 | 0.057 | + | 3.117 | 2.863 | 3.393 |

Table 1 - B - 6

IESCOPD - Meta-regression of current smoking, any product (or cigarettes if all product not available)

Multiple regression of data from Table 1 - B - 1 (most-adjusted RRs)

Any COPD

Test by removing variables one at a time

|  |  | **Deviance** | **(DF)** | **Drop Dev** | **P** |  |  |
| --- | --- | --- | --- | --- | --- | --- | --- |
| **Omitting Sex (RR)** |  | 464.558 | (105) | -31.278 | * |  |  |
|  |  | **Estimate** | **S.E.** | **P** | **RR** | **95%CIl** | **95%CIu** |
| **Constant** |  | 0.985 | 0.155 | +++ | 2.679 | 1.978 | 3.628 |
| **RR adjusted for factor other than sex, age** |  |  |  |  |  |  |  |
| Yes | 27 | Aliased |  |  | 2.722 | 2.283 | 3.245 |
| No | 93 | 0.131 | 0.056 | + | 3.104 | 2.856 | 3.374 |
| **Continent** |  |  |  |  |  |  |  |
| NAmer | 39 | Aliased |  |  | 3.898 | 3.386 | 4.486 |
| Europe | 55 | -0.365 | 0.049 | --- | 2.705 | 2.450 | 2.988 |
| Asia | 17 | -0.480 | 0.079 | --- | 2.412 | 1.840 | 3.163 |
| oth/mult | 9 | -0.485 | 0.081 | --- | 2.399 | 1.807 | 3.186 |
| **Asthma analysis type (COPD)** |  |  |  |  |  |  |  |
| inc-irresp | 72 | Aliased |  |  | 3.497 | 3.213 | 3.806 |
| excl-all | 17 | -0.386 | 0.050 | --- | 2.378 | 2.020 | 2.798 |
| defn-incl | 18 | -0.736 | 0.072 | --- | 1.675 | 1.273 | 2.203 |
| other | 13 | -0.063 | 0.103 | N.S. | 3.285 | 2.207 | 4.888 |
| **Smoking product (3)** |  |  |  |  |  |  |  |
| any | 46 | Aliased |  |  | 2.586 | 2.068 | 3.234 |
| cigs | 66 | 0.198 | 0.080 | + | 3.153 | 2.784 | 3.570 |
| cigsonly | 8 | 0.361 | 0.095 | +++ | 3.710 | 2.670 | 5.155 |
| **Unexposed group** |  |  |  |  |  |  |  |
| nev any | 58 | Aliased |  |  | 3.759 | 3.168 | 4.461 |
| nev cig | 62 | -0.407 | 0.073 | --- | 2.501 | 2.167 | 2.886 |
| **COPD subtype** |  |  |  |  |  |  |  |
| mort | 31 | Aliased |  |  | 4.109 | 3.268 | 5.166 |
| LF | 56 | -0.470 | 0.068 | --- | 2.567 | 2.353 | 2.801 |
| other | 33 | -0.003 | 0.072 | N.S. | 4.097 | 3.459 | 4.854 |
| **Midpoint age in RR** |  | 0.013 | 0.002 | +++ | 1.414 | 0.811 | 2.463 |
| **RR adjusted for age** |  |  |  |  |  |  |  |
| Yes | 63 | Aliased |  |  | 2.843 | 2.469 | 3.274 |
| No | 57 | 0.086 | 0.050 | (+) | 3.096 | 2.815 | 3.406 |

Table 1 - B - 6

IESCOPD - Meta-regression of current smoking, any product (or cigarettes if all product not available)

Multiple regression of data from Table 1 - B - 1 (most-adjusted RRs)

Any COPD

Test by removing variables one at a time

|  |  | **Deviance** | **(DF)** | **Drop Dev** | **P** |  |  |
| --- | --- | --- | --- | --- | --- | --- | --- |
| **Omitting Continent** |  | 507.328 | (106) | -74.048 | *** |  |  |
|  |  | **Estimate** | **S.E.** | **P** | **RR** | **95%CIl** | **95%CIu** |
| **Constant** |  | 1.000 | 0.150 | +++ | 2.720 | 2.028 | 3.647 |
| **RR adjusted for factor other than sex, age** |  |  |  |  |  |  |  |
| Yes | 27 | Aliased |  |  | 2.312 | 1.969 | 2.715 |
| No | 93 | 0.350 | 0.050 | +++ | 3.283 | 3.032 | 3.554 |
| **Sex(RR)** |  |  |  |  |  |  |  |
| Male | 48 | Aliased |  |  | 3.087 | 2.736 | 3.484 |
| Female | 31 | -0.185 | 0.044 | --- | 2.566 | 2.262 | 2.911 |
| Combined | 41 | 0.050 | 0.043 | N.S. | 3.245 | 2.926 | 3.599 |
| **Asthma analysis type (COPD)** |  |  |  |  |  |  |  |
| inc-irresp | 72 | Aliased |  |  | 3.587 | 3.307 | 3.890 |
| excl-all | 17 | -0.455 | 0.045 | --- | 2.275 | 1.964 | 2.637 |
| defn-incl | 18 | -0.752 | 0.071 | --- | 1.691 | 1.287 | 2.221 |
| other | 13 | -0.234 | 0.096 | - | 2.837 | 1.969 | 4.088 |
| **Smoking product (3)** |  |  |  |  |  |  |  |
| any | 46 | Aliased |  |  | 1.989 | 1.629 | 2.429 |
| cigs | 66 | 0.582 | 0.071 | +++ | 3.561 | 3.179 | 3.989 |
| cigsonly | 8 | 0.646 | 0.097 | +++ | 3.797 | 2.713 | 5.314 |
| **Unexposed group** |  |  |  |  |  |  |  |
| nev any | 58 | Aliased |  |  | 4.388 | 3.720 | 5.175 |
| nev cig | 62 | -0.687 | 0.070 | --- | 2.207 | 1.922 | 2.534 |
| **COPD subtype** |  |  |  |  |  |  |  |
| mort | 31 | Aliased |  |  | 4.244 | 3.354 | 5.372 |
| LF | 56 | -0.523 | 0.070 | --- | 2.516 | 2.307 | 2.744 |
| other | 33 | 0.012 | 0.074 | N.S. | 4.294 | 3.639 | 5.067 |
| **Midpoint age in RR** |  | 0.006 | 0.002 | ++ | 2.083 | 1.253 | 3.464 |
| **RR adjusted for age** |  |  |  |  |  |  |  |
| Yes | 63 | Aliased |  |  | 3.039 | 2.651 | 3.483 |
| No | 57 | -0.019 | 0.049 | N.S. | 2.980 | 2.716 | 3.271 |
|  |  | **Deviance** | **(DF)** | **Drop Dev** | **P** |  |  |
| **Omitting Asthma analysis type (COPD)** |  | 576.666 | (106) | -143.385 | *** |  |  |
|  |  | **Estimate** | **S.E.** | **P** | **RR** | **95%CIl** | **95%CIu** |
| **Constant** |  | 0.909 | 0.153 | +++ | 2.481 | 1.839 | 3.346 |
| **RR adjusted for factor other than sex, age** |  |  |  |  |  |  |  |
| Yes | 27 | Aliased |  |  | 2.866 | 2.410 | 3.407 |
| No | 93 | 0.062 | 0.055 | N.S. | 3.049 | 2.808 | 3.312 |
| **Sex(RR)** |  |  |  |  |  |  |  |
| Male | 48 | Aliased |  |  | 3.264 | 2.884 | 3.693 |
| Female | 31 | -0.283 | 0.044 | --- | 2.460 | 2.169 | 2.789 |
| Combined | 41 | -0.019 | 0.045 | N.S. | 3.201 | 2.876 | 3.563 |
| **Continent** |  |  |  |  |  |  |  |
| NAmer | 39 | Aliased |  |  | 4.322 | 3.784 | 4.936 |
| Europe | 55 | -0.544 | 0.046 | --- | 2.508 | 2.286 | 2.753 |
| Asia | 17 | -0.497 | 0.076 | --- | 2.628 | 2.038 | 3.390 |
| oth/mult | 9 | -0.566 | 0.079 | --- | 2.454 | 1.854 | 3.249 |
| **Smoking product (3)** |  |  |  |  |  |  |  |
| any | 46 | Aliased |  |  | 2.457 | 1.962 | 3.076 |
| cigs | 66 | 0.251 | 0.082 | ++ | 3.158 | 2.784 | 3.581 |
| cigsonly | 8 | 0.639 | 0.096 | +++ | 4.656 | 3.333 | 6.505 |

Table 1 - B - 6

IESCOPD - Meta-regression of current smoking, any product (or cigarettes if all product not available)

Multiple regression of data from Table 1 - B - 1 (most-adjusted RRs)

Any COPD

Test by removing variables one at a time

|  |  | **Estimate** | **S.E.** | **P** | **RR** | **95%CIl** | **95%CIu** |
| --- | --- | --- | --- | --- | --- | --- | --- |
| **Unexposed group** |  |  |  |  |  |  |  |
| nev any | 58 | Aliased |  |  | 4.070 | 3.414 | 4.852 |
| nev cig | 62 | -0.551 | 0.075 | --- | 2.345 | 2.025 | 2.716 |
| **COPD subtype** |  |  |  |  |  |  |  |
| mort | 31 | Aliased |  |  | 3.477 | 2.763 | 4.376 |
| LF | 56 | -0.258 | 0.067 | --- | 2.686 | 2.475 | 2.915 |
| other | 33 | 0.140 | 0.075 | (+) | 3.999 | 3.415 | 4.683 |
| **Midpoint age in RR** |  | 0.013 | 0.002 | +++ | 1.435 | 0.836 | 2.461 |
| **RR adjusted for age** |  |  |  |  |  |  |  |
| Yes | 63 | Aliased |  |  | 2.735 | 2.383 | 3.138 |
| No | 57 | 0.146 | 0.049 | ++ | 3.166 | 2.884 | 3.476 |
|  |  | **Deviance** | **(DF)** | **Drop Dev** | **P** |  |  |
| **Omitting Smoking product (3)** |  | 460.137 | (105) | -26.856 | * |  |  |
|  |  | **Estimate** | **S.E.** | **P** | **RR** | **95%CIl** | **95%CIu** |
| **Constant** |  | 1.189 | 0.152 | +++ | 3.284 | 2.437 | 4.425 |
| **RR adjusted for factor other than sex, age** |  |  |  |  |  |  |  |
| Yes | 27 | Aliased |  |  | 2.731 | 2.319 | 3.215 |
| No | 93 | 0.127 | 0.051 | + | 3.100 | 2.861 | 3.360 |
| **Sex(RR)** |  |  |  |  |  |  |  |
| Male | 48 | Aliased |  |  | 3.173 | 2.811 | 3.581 |
| Female | 31 | -0.174 | 0.043 | --- | 2.667 | 2.355 | 3.021 |
| Combined | 41 | -0.022 | 0.043 | N.S. | 3.105 | 2.796 | 3.448 |
| **Continent** |  |  |  |  |  |  |  |
| NAmer | 39 | Aliased |  |  | 4.062 | 3.580 | 4.610 |
| Europe | 55 | -0.424 | 0.043 | --- | 2.660 | 2.420 | 2.924 |
| Asia | 17 | -0.564 | 0.076 | --- | 2.312 | 1.763 | 3.032 |
| oth/mult | 9 | -0.546 | 0.079 | --- | 2.353 | 1.763 | 3.140 |
| **Asthma analysis type (COPD)** |  |  |  |  |  |  |  |
| inc-irresp | 72 | Aliased |  |  | 3.447 | 3.166 | 3.753 |
| excl-all | 17 | -0.302 | 0.050 | --- | 2.548 | 2.168 | 2.994 |
| defn-incl | 18 | -0.800 | 0.070 | --- | 1.548 | 1.185 | 2.023 |
| other | 13 | -0.132 | 0.102 | N.S. | 3.020 | 2.035 | 4.481 |
| **Unexposed group** |  |  |  |  |  |  |  |
| nev any | 58 | Aliased |  |  | 3.453 | 3.112 | 3.832 |
| nev cig | 62 | -0.254 | 0.038 | --- | 2.679 | 2.444 | 2.936 |
| **COPD subtype** |  |  |  |  |  |  |  |
| mort | 31 | Aliased |  |  | 4.625 | 3.757 | 5.694 |
| LF | 56 | -0.615 | 0.061 | --- | 2.501 | 2.298 | 2.722 |
| other | 33 | -0.126 | 0.068 | (-) | 4.076 | 3.440 | 4.829 |
| **Midpoint age in RR** |  | 0.015 | 0.002 | +++ | 1.281 | 0.753 | 2.180 |
| **RR adjusted for age** |  |  |  |  |  |  |  |
| Yes | 63 | Aliased |  |  | 2.945 | 2.573 | 3.370 |
| No | 57 | 0.030 | 0.048 | N.S. | 3.034 | 2.767 | 3.328 |
|  |  | **Deviance** | **(DF)** | **Drop Dev** | **P** |  |  |
| **Omitting Unexposed group** |  | 466.883 | (104) | -33.602 | ** |  |  |
|  |  | **Estimate** | **S.E.** | **P** | **RR** | **95%CIl** | **95%CIu** |
| **Constant** |  | 1.163 | 0.154 | +++ | 3.201 | 2.368 | 4.326 |
| **RR adjusted for factor other than sex, age** |  |  |  |  |  |  |  |
| Yes | 27 | Aliased |  |  | 2.864 | 2.407 | 3.409 |
| No | 93 | 0.063 | 0.055 | N.S. | 3.050 | 2.807 | 3.314 |
| **Sex(RR)** |  |  |  |  |  |  |  |
| Male | 48 | Aliased |  |  | 3.320 | 2.937 | 3.752 |
| Female | 31 | -0.237 | 0.044 | --- | 2.619 | 2.305 | 2.975 |
| Combined | 41 | -0.088 | 0.043 | - | 3.039 | 2.737 | 3.375 |
| **Continent** |  |  |  |  |  |  |  |
| NAmer | 39 | Aliased |  |  | 4.180 | 3.651 | 4.784 |
| Europe | 55 | -0.463 | 0.047 | --- | 2.631 | 2.381 | 2.906 |
| Asia | 17 | -0.629 | 0.076 | --- | 2.229 | 1.701 | 2.921 |
| oth/mult | 9 | -0.583 | 0.080 | --- | 2.332 | 1.745 | 3.117 |

Table 1 - B - 6

IESCOPD - Meta-regression of current smoking, any product (or cigarettes if all product not available)

Multiple regression of data from Table 1 - B - 1 (most-adjusted RRs)

Any COPD

Test by removing variables one at a time

|  |  | **Estimate** | **S.E.** | **P** | **RR** | **95%CIl** | **95%CIu** |
| --- | --- | --- | --- | --- | --- | --- | --- |
| **Asthma analysis type (COPD)** |  |  |  |  |  |  |  |
| inc-irresp | 72 | Aliased |  |  | 3.471 | 3.186 | 3.781 |
| excl-all | 17 | -0.331 | 0.051 | --- | 2.493 | 2.113 | 2.942 |
| defn-incl | 18 | -0.788 | 0.071 | --- | 1.579 | 1.202 | 2.074 |
| other | 13 | -0.142 | 0.102 | N.S. | 3.012 | 2.024 | 4.482 |
| **Smoking product (3)** |  |  |  |  |  |  |  |
| any | 46 | Aliased |  |  | 3.276 | 2.874 | 3.735 |
| cigs | 66 | -0.168 | 0.042 | --- | 2.769 | 2.543 | 3.015 |
| cigsonly | 8 | 0.302 | 0.092 | ++ | 4.433 | 3.164 | 6.213 |
| **COPD subtype** |  |  |  |  |  |  |  |
| mort | 31 | Aliased |  |  | 4.350 | 3.445 | 5.493 |
| LF | 56 | -0.540 | 0.069 | --- | 2.535 | 2.322 | 2.768 |
| other | 33 | -0.063 | 0.073 | N.S. | 4.084 | 3.443 | 4.846 |
| **Midpoint age in RR** |  | 0.015 | 0.002 | +++ | 1.286 | 0.741 | 2.230 |
| **RR adjusted for age** |  |  |  |  |  |  |  |
| Yes | 63 | Aliased |  |  | 2.819 | 2.447 | 3.248 |
| No | 57 | 0.098 | 0.050 | (+) | 3.111 | 2.827 | 3.423 |
|  |  | **Deviance** | **(DF)** | **Drop Dev** | **P** |  |  |
| **Omitting COPD subtype** |  | 540.045 | (105) | -106.764 | *** |  |  |
|  |  | **Estimate** | **S.E.** | **P** | **RR** | **95%CIl** | **95%CIu** |
| **Constant** |  | 0.789 | 0.140 | +++ | 2.201 | 1.674 | 2.894 |
| **RR adjusted for factor other than sex, age** |  |  |  |  |  |  |  |
| Yes | 27 | Aliased |  |  | 2.867 | 2.403 | 3.420 |
| No | 93 | 0.062 | 0.056 | N.S. | 3.049 | 2.805 | 3.315 |
| **Sex(RR)** |  |  |  |  |  |  |  |
| Male | 48 | Aliased |  |  | 3.263 | 2.894 | 3.678 |
| Female | 31 | -0.234 | 0.043 | --- | 2.583 | 2.274 | 2.934 |
| Combined | 41 | -0.050 | 0.043 | N.S. | 3.105 | 2.791 | 3.453 |
| **Continent** |  |  |  |  |  |  |  |
| NAmer | 39 | Aliased |  |  | 3.996 | 3.464 | 4.610 |
| Europe | 55 | -0.380 | 0.051 | --- | 2.732 | 2.468 | 3.024 |
| Asia | 17 | -0.564 | 0.079 | --- | 2.273 | 1.734 | 2.981 |
| oth/mult | 9 | -0.675 | 0.079 | --- | 2.035 | 1.537 | 2.694 |
| **Asthma analysis type (COPD)** |  |  |  |  |  |  |  |
| inc-irresp | 72 | Aliased |  |  | 3.413 | 3.134 | 3.716 |
| excl-all | 17 | -0.428 | 0.050 | --- | 2.225 | 1.896 | 2.610 |
| defn-incl | 18 | -0.431 | 0.066 | --- | 2.218 | 1.725 | 2.852 |
| other | 13 | 0.312 | 0.097 | ++ | 4.663 | 3.206 | 6.783 |
| **Smoking product (3)** |  |  |  |  |  |  |  |
| any | 46 | Aliased |  |  | 2.415 | 1.924 | 3.030 |
| cigs | 66 | 0.269 | 0.083 | ++ | 3.159 | 2.779 | 3.592 |
| cigsonly | 8 | 0.732 | 0.085 | +++ | 5.023 | 3.704 | 6.811 |
| **Unexposed group** |  |  |  |  |  |  |  |
| nev any | 58 | Aliased |  |  | 3.975 | 3.341 | 4.730 |
| nev cig | 62 | -0.509 | 0.074 | --- | 2.391 | 2.067 | 2.764 |
| **Midpoint age in RR** |  | 0.013 | 0.002 | +++ | 1.423 | 0.821 | 2.467 |
| **RR adjusted for age** |  |  |  |  |  |  |  |
| Yes | 63 | Aliased |  |  | 2.807 | 2.438 | 3.232 |
| No | 57 | 0.105 | 0.050 | + | 3.119 | 2.836 | 3.430 |
|  |  | **Deviance** | **(DF)** | **Drop Dev** | **P** |  |  |
| **Omitting Midpoint age in RR** |  | 458.902 | (104) | -25.621 | * |  |  |
|  |  | **Estimate** | **S.E.** | **P** | **RR** | **95%CIl** | **95%CIu** |
| **Constant** |  | 1.652 | 0.091 | +++ | 5.220 | 4.368 | 6.239 |
| **RR adjusted for factor other than sex, age** |  |  |  |  |  |  |  |
| Yes | 27 | Aliased |  |  | 2.563 | 2.150 | 3.054 |
| No | 93 | 0.212 | 0.055 | +++ | 3.169 | 2.915 | 3.445 |

Table 1 - B - 6

IESCOPD - Meta-regression of current smoking, any product (or cigarettes if all product not available)

Multiple regression of data from Table 1 - B - 1 (most-adjusted RRs)

Any COPD

Test by removing variables one at a time

|  |  | **Estimate** | **S.E.** | **P** | **RR** | **95%CIl** | **95%CIu** |
| --- | --- | --- | --- | --- | --- | --- | --- |
| **Sex(RR)** |  |  |  |  |  |  |  |
| Male | 48 | Aliased |  |  | 3.182 | 2.807 | 3.607 |
| Female | 31 | -0.225 | 0.045 | --- | 2.541 | 2.235 | 2.889 |
| Combined | 41 | 0.004 | 0.045 | N.S. | 3.194 | 2.866 | 3.560 |
| **Continent** |  |  |  |  |  |  |  |
| NAmer | 39 | Aliased |  |  | 3.683 | 3.202 | 4.235 |
| Europe | 55 | -0.276 | 0.050 | --- | 2.794 | 2.523 | 3.093 |
| Asia | 17 | -0.355 | 0.074 | --- | 2.581 | 1.985 | 3.356 |
| oth/mult | 9 | -0.502 | 0.081 | --- | 2.230 | 1.671 | 2.975 |
| **Asthma analysis type (COPD)** |  |  |  |  |  |  |  |
| inc-irresp | 72 | Aliased |  |  | 3.446 | 3.162 | 3.754 |
| excl-all | 17 | -0.355 | 0.051 | --- | 2.416 | 2.049 | 2.850 |
| defn-incl | 18 | -0.700 | 0.072 | --- | 1.711 | 1.299 | 2.253 |
| other | 13 | 0.055 | 0.101 | N.S. | 3.641 | 2.461 | 5.387 |
| **Smoking product (3)** |  |  |  |  |  |  |  |
| any | 46 | Aliased |  |  | 2.268 | 1.811 | 2.839 |
| cigs | 66 | 0.382 | 0.081 | +++ | 3.323 | 2.931 | 3.767 |
| cigsonly | 8 | 0.588 | 0.097 | +++ | 4.083 | 2.901 | 5.746 |
| **Unexposed group** |  |  |  |  |  |  |  |
| nev any | 58 | Aliased |  |  | 3.989 | 3.339 | 4.767 |
| nev cig | 62 | -0.515 | 0.076 | --- | 2.384 | 2.054 | 2.766 |
| **COPD subtype** |  |  |  |  |  |  |  |
| mort | 31 | Aliased |  |  | 4.155 | 3.272 | 5.276 |
| LF | 56 | -0.485 | 0.070 | --- | 2.557 | 2.341 | 2.793 |
| other | 33 | -0.009 | 0.075 | N.S. | 4.116 | 3.468 | 4.884 |
| **RR adjusted for age** |  |  |  |  |  |  |  |
| Yes | 63 | Aliased |  |  | 2.944 | 2.560 | 3.387 |
| No | 57 | 0.030 | 0.049 | N.S. | 3.035 | 2.760 | 3.337 |
|  |  | **Deviance** | **(DF)** | **Drop Dev** | **P** |  |  |
| **Omitting RR adjusted for age** |  | 435.562 | (104) | -2.281 | N.S. |  |  |
|  |  | **Estimate** | **S.E.** | **P** | **RR** | **95%CIl** | **95%CIu** |
| **Constant** |  | 1.045 | 0.154 | +++ | 2.844 | 2.102 | 3.849 |
| **RR adjusted for factor other than sex, age** |  |  |  |  |  |  |  |
| Yes | 27 | Aliased |  |  | 2.587 | 2.232 | 2.999 |
| No | 93 | 0.200 | 0.045 | +++ | 3.158 | 2.925 | 3.411 |
| **Sex(RR)** |  |  |  |  |  |  |  |
| Male | 48 | Aliased |  |  | 3.192 | 2.816 | 3.619 |
| Female | 31 | -0.220 | 0.045 | --- | 2.563 | 2.254 | 2.914 |
| Combined | 41 | -0.007 | 0.045 | N.S. | 3.170 | 2.843 | 3.534 |
| **Continent** |  |  |  |  |  |  |  |
| NAmer | 39 | Aliased |  |  | 3.832 | 3.322 | 4.420 |
| Europe | 55 | -0.333 | 0.051 | --- | 2.747 | 2.480 | 3.043 |
| Asia | 17 | -0.491 | 0.079 | --- | 2.346 | 1.786 | 3.081 |
| oth/mult | 9 | -0.481 | 0.079 | --- | 2.369 | 1.780 | 3.153 |
| **Asthma analysis type (COPD)** |  |  |  |  |  |  |  |
| inc-irresp | 72 | Aliased |  |  | 3.444 | 3.161 | 3.752 |
| excl-all | 17 | -0.327 | 0.051 | --- | 2.485 | 2.107 | 2.930 |
| defn-incl | 18 | -0.741 | 0.070 | --- | 1.641 | 1.253 | 2.149 |
| other | 13 | -0.050 | 0.103 | N.S. | 3.276 | 2.194 | 4.893 |
| **Smoking product (3)** |  |  |  |  |  |  |  |
| any | 46 | Aliased |  |  | 2.452 | 1.941 | 3.098 |
| cigs | 66 | 0.271 | 0.084 | ++ | 3.214 | 2.828 | 3.653 |
| cigsonly | 8 | 0.472 | 0.096 | +++ | 3.932 | 2.842 | 5.440 |
| **Unexposed group** |  |  |  |  |  |  |  |
| nev any | 58 | Aliased |  |  | 3.858 | 3.222 | 4.620 |
| nev cig | 62 | -0.455 | 0.077 | --- | 2.449 | 2.107 | 2.846 |
| **COPD subtype** |  |  |  |  |  |  |  |
| mort | 31 | Aliased |  |  | 3.970 | 3.119 | 5.055 |
| LF | 56 | -0.434 | 0.071 | --- | 2.572 | 2.354 | 2.809 |
| other | 33 | 0.054 | 0.076 | N.S. | 4.192 | 3.533 | 4.975 |
| **Midpoint age in RR** |  | 0.012 | 0.002 | +++ | 1.542 | 0.889 | 2.674 |

Table 1 - B - 6

IESCOPD - Meta-regression of current smoking, any product (or cigarettes if all product not available)

Multiple regression of data from Table 1 - B - 1 (most-adjusted RRs)

Any COPD

Test by removing variables one at a time

|  |  | **Deviance** | **(DF)** | **Drop Dev** | **P** |  |  |
| --- | --- | --- | --- | --- | --- | --- | --- |
| **Omitting RR adjusted for factor other than sex, age** |  | 440.098 | (104) | -6.817 | N.S. |  |  |
|  |  | **Estimate** | **S.E.** | **P** | **RR** | **95%CIl** | **95%CIu** |
| **Constant** |  | 1.052 | 0.155 | +++ | 2.863 | 2.112 | 3.880 |
| **RR adjusted for age** |  |  |  |  |  |  |  |
| Yes | 63 | Aliased |  |  | 2.719 | 2.414 | 3.063 |
| No | 57 | 0.155 | 0.040 | +++ | 3.176 | 2.918 | 3.458 |
| **Sex(RR)** |  |  |  |  |  |  |  |
| Male | 48 | Aliased |  |  | 3.224 | 2.847 | 3.651 |
| Female | 31 | -0.223 | 0.045 | --- | 2.580 | 2.269 | 2.933 |
| Combined | 41 | -0.028 | 0.044 | N.S. | 3.134 | 2.815 | 3.489 |
| **Continent** |  |  |  |  |  |  |  |
| NAmer | 39 | Aliased |  |  | 4.013 | 3.510 | 4.590 |
| Europe | 55 | -0.401 | 0.047 | --- | 2.687 | 2.433 | 2.967 |
| Asia | 17 | -0.536 | 0.078 | --- | 2.348 | 1.787 | 3.084 |
| oth/mult | 9 | -0.583 | 0.076 | --- | 2.241 | 1.686 | 2.979 |
| **Asthma analysis type (COPD)** |  |  |  |  |  |  |  |
| inc-irresp | 72 | Aliased |  |  | 3.417 | 3.139 | 3.719 |
| excl-all | 17 | -0.301 | 0.050 | --- | 2.528 | 2.150 | 2.971 |
| defn-incl | 18 | -0.721 | 0.072 | --- | 1.662 | 1.262 | 2.189 |
| other | 13 | -0.049 | 0.103 | N.S. | 3.253 | 2.178 | 4.859 |
| **Smoking product (3)** |  |  |  |  |  |  |  |
| any | 46 | Aliased |  |  | 2.608 | 2.094 | 3.249 |
| cigs | 66 | 0.170 | 0.078 | + | 3.092 | 2.741 | 3.488 |
| cigsonly | 8 | 0.505 | 0.099 | +++ | 4.324 | 3.104 | 6.023 |
| **Unexposed group** |  |  |  |  |  |  |  |
| nev any | 58 | Aliased |  |  | 3.730 | 3.131 | 4.445 |
| nev cig | 62 | -0.394 | 0.074 | --- | 2.517 | 2.174 | 2.914 |
| **COPD subtype** |  |  |  |  |  |  |  |
| mort | 31 | Aliased |  |  | 3.977 | 3.124 | 5.063 |
| LF | 56 | -0.430 | 0.071 | --- | 2.586 | 2.368 | 2.824 |
| other | 33 | 0.031 | 0.076 | N.S. | 4.102 | 3.463 | 4.860 |
| **Midpoint age in RR** |  | 0.014 | 0.002 | +++ | 1.369 | 0.793 | 2.362 |

Table 1 - B - 6

IESCOPD - Meta-regression of current smoking, any product (or cigarettes if all product not available)

Multiple regression of data from Table 1 - B - 1 (most-adjusted RRs)

Any COPD

Test reduction to 2-level product

|  |  | **Deviance** | **(DF)** | **Drop Dev** | **P** |  |  |
| --- | --- | --- | --- | --- | --- | --- | --- |
| **Reducing Smoking product to 2 levels** |  | 439.823 | (104) | -6.543 | N.S. |  |  |
|  |  | **Estimate** | **S.E.** | **P** | **RR** | **95%CIl** | **95%CIu** |
| **Constant** |  | 1.048 | 0.155 | +++ | 2.852 | 2.103 | 3.867 |
| **Sex(RR)** |  |  |  |  |  |  |  |
| Male | 48 | Aliased |  |  | 3.132 | 2.772 | 3.539 |
| Female | 31 | -0.190 | 0.043 | --- | 2.591 | 2.281 | 2.944 |
| Combined | 41 | 0.019 | 0.044 | N.S. | 3.192 | 2.865 | 3.556 |
| **Continent** |  |  |  |  |  |  |  |
| NAmer | 39 | Aliased |  |  | 3.789 | 3.289 | 4.364 |
| Europe | 55 | -0.314 | 0.050 | --- | 2.768 | 2.501 | 3.063 |
| Asia | 17 | -0.476 | 0.079 | --- | 2.353 | 1.792 | 3.091 |
| oth/mult | 9 | -0.482 | 0.080 | --- | 2.341 | 1.751 | 3.128 |
| **Asthma analysis type (COPD)** |  |  |  |  |  |  |  |
| inc-irresp | 72 | Aliased |  |  | 3.461 | 3.177 | 3.770 |
| excl-all | 17 | -0.344 | 0.051 | --- | 2.454 | 2.080 | 2.896 |
| defn-incl | 18 | -0.739 | 0.071 | --- | 1.653 | 1.256 | 2.177 |
| other | 13 | -0.068 | 0.103 | N.S. | 3.234 | 2.166 | 4.831 |
| **Smoking product (2)** |  |  |  |  |  |  |  |
| any | 46 | Aliased |  |  | 2.357 | 1.886 | 2.946 |
| cigs | 74 | 0.345 | 0.077 | +++ | 3.330 | 2.980 | 3.720 |
| **Unexposed group** |  |  |  |  |  |  |  |
| nev any | 58 | Aliased |  |  | 4.005 | 3.386 | 4.739 |
| nev cig | 62 | -0.522 | 0.071 | --- | 2.376 | 2.064 | 2.735 |
| **COPD subtype** |  |  |  |  |  |  |  |
| mort | 31 | Aliased |  |  | 4.198 | 3.349 | 5.262 |
| LF | 56 | -0.501 | 0.066 | --- | 2.543 | 2.332 | 2.772 |
| other | 33 | -0.007 | 0.073 | N.S. | 4.168 | 3.511 | 4.948 |
| **Midpoint age in RR** |  | 0.012 | 0.002 | +++ | 1.532 | 0.878 | 2.676 |
| **RR adjusted for age** |  |  |  |  |  |  |  |
| Yes | 63 | Aliased |  |  | 2.937 | 2.565 | 3.364 |
| No | 57 | 0.034 | 0.048 | N.S. | 3.039 | 2.770 | 3.334 |
| **RR adjusted for factor other than sex, age** |  |  |  |  |  |  |  |
| Yes | 27 | Aliased |  |  | 2.593 | 2.187 | 3.075 |
| No | 93 | 0.196 | 0.053 | +++ | 3.156 | 2.907 | 3.426 |

Table 1 - B - 6

IESCOPD - Meta-regression of current smoking, any product (or cigarettes if all product not available)

Multiple regression of data from Table 1 - B - 1 (most-adjusted RRs)

Any COPD

Test reduction to 2-level outcome subtype

|  |  | **Deviance** | **(DF)** | **Drop Dev** | **P** |  |  |
| --- | --- | --- | --- | --- | --- | --- | --- |
| **Reducing COPD subtype to 2 levels** |  | 433.694 | (104) | -0.413 | N.S. |  |  |
|  |  | **Estimate** | **S.E.** | **P** | **RR** | **95%CIl** | **95%CIu** |
| **Constant** |  | 0.583 | 0.141 | +++ | 1.791 | 1.358 | 2.362 |
| **Sex(RR)** |  |  |  |  |  |  |  |
| Male | 48 | Aliased |  |  | 3.173 | 2.812 | 3.581 |
| Female | 31 | -0.211 | 0.043 | --- | 2.570 | 2.261 | 2.922 |
| Combined | 41 | 0.001 | 0.043 | N.S. | 3.178 | 2.855 | 3.537 |
| **Continent** |  |  |  |  |  |  |  |
| NAmer | 39 | Aliased |  |  | 3.860 | 3.341 | 4.458 |
| Europe | 55 | -0.342 | 0.051 | --- | 2.741 | 2.475 | 3.036 |
| Asia | 17 | -0.495 | 0.079 | --- | 2.354 | 1.792 | 3.092 |
| oth/mult | 9 | -0.515 | 0.081 | --- | 2.307 | 1.732 | 3.071 |
| **Asthma analysis type (COPD)** |  |  |  |  |  |  |  |
| inc-irresp | 72 | Aliased |  |  | 3.449 | 3.166 | 3.757 |
| excl-all | 17 | -0.336 | 0.051 | --- | 2.464 | 2.088 | 2.907 |
| defn-incl | 18 | -0.724 | 0.072 | --- | 1.672 | 1.270 | 2.202 |
| other | 13 | -0.053 | 0.103 | N.S. | 3.270 | 2.190 | 4.883 |
| **Smoking product (3)** |  |  |  |  |  |  |  |
| any | 46 | Aliased |  |  | 2.492 | 1.983 | 3.132 |
| cigs | 66 | 0.245 | 0.083 | ++ | 3.183 | 2.798 | 3.621 |
| cigsonly | 8 | 0.476 | 0.088 | +++ | 4.013 | 2.920 | 5.516 |
| **Unexposed group** |  |  |  |  |  |  |  |
| nev any | 58 | Aliased |  |  | 3.813 | 3.199 | 4.544 |
| nev cig | 62 | -0.433 | 0.074 | --- | 2.473 | 2.135 | 2.863 |
| **COPD subtype (2)** |  |  |  |  |  |  |  |
| LF | 56 | Aliased |  |  | 2.566 | 2.354 | 2.798 |
| other | 64 | 0.470 | 0.046 | +++ | 4.104 | 3.582 | 4.703 |
| **Midpoint age in RR** |  | 0.012 | 0.002 | +++ | 1.505 | 0.866 | 2.617 |
| **RR adjusted for age** |  |  |  |  |  |  |  |
| Yes | 63 | Aliased |  |  | 2.857 | 2.480 | 3.292 |
| No | 57 | 0.078 | 0.050 | N.S. | 3.087 | 2.806 | 3.398 |
| **RR adjusted for factor other than sex, age** |  |  |  |  |  |  |  |
| Yes | 27 | Aliased |  |  | 2.695 | 2.254 | 3.223 |
| No | 93 | 0.145 | 0.056 | + | 3.114 | 2.862 | 3.388 |

Table 1 - B - 6

IESCOPD - Meta-regression of current smoking, any product (or cigarettes if all product not available)

Multiple regression of data from Table 1 - B - 1 (most-adjusted RRs)

Any COPD

Test by adding extra variables one at a time

|  |  | **Deviance** | **(DF)** | **Drop Dev** | **P** |  |  |
| --- | --- | --- | --- | --- | --- | --- | --- |
| **Increasing COPD subtype to 4 levels** |  | 430.421 | (102) | 2.859 | N.S. |  |  |
|  |  | **Estimate** | **S.E.** | **P** | **RR** | **95%CIl** | **95%CIu** |
| **Constant** |  | 1.012 | 0.156 | +++ | 2.752 | 2.027 | 3.736 |
| **Sex(RR)** |  |  |  |  |  |  |  |
| Male | 48 | Aliased |  |  | 3.203 | 2.822 | 3.635 |
| Female | 31 | -0.217 | 0.045 | --- | 2.578 | 2.264 | 2.935 |
| Combined | 41 | -0.016 | 0.046 | N.S. | 3.151 | 2.822 | 3.518 |
| **Continent** |  |  |  |  |  |  |  |
| NAmer | 39 | Aliased |  |  | 3.868 | 3.342 | 4.477 |
| Europe | 55 | -0.347 | 0.051 | --- | 2.735 | 2.465 | 3.034 |
| Asia | 17 | -0.497 | 0.079 | --- | 2.353 | 1.788 | 3.096 |
| oth/mult | 9 | -0.505 | 0.081 | --- | 2.334 | 1.742 | 3.126 |
| **Asthma analysis type (COPD)** |  |  |  |  |  |  |  |
| inc-irresp | 72 | Aliased |  |  | 3.448 | 3.163 | 3.759 |
| excl-all | 17 | -0.344 | 0.051 | --- | 2.445 | 2.067 | 2.893 |
| defn-incl | 18 | -0.694 | 0.073 | --- | 1.723 | 1.295 | 2.290 |
| other | 13 | -0.052 | 0.103 | N.S. | 3.274 | 2.186 | 4.904 |
| **Smoking product (3)** |  |  |  |  |  |  |  |
| any | 46 | Aliased |  |  | 2.453 | 1.935 | 3.108 |
| cigs | 66 | 0.266 | 0.085 | ++ | 3.200 | 2.808 | 3.646 |
| cigsonly | 8 | 0.517 | 0.099 | +++ | 4.113 | 2.915 | 5.803 |
| **Unexposed group** |  |  |  |  |  |  |  |
| nev any | 58 | Aliased |  |  | 3.834 | 3.197 | 4.599 |
| nev cig | 62 | -0.443 | 0.077 | --- | 2.461 | 2.115 | 2.865 |
| **COPD subtype** |  |  |  |  |  |  |  |
| mort | 31 | Aliased |  |  |  |  |  |
| LF | 56 | Aliased |  |  |  |  |  |
| other | 33 | Aliased |  |  |  |  |  |
|  |  | **Estimate** | **S.E.** | **P** | **RR** | **95%CIl** | **95%CIu** |
| **Midpoint age in RR** |  | 0.012 | 0.002 | +++ | 1.515 | 0.861 | 2.666 |
| **RR adjusted for age** |  |  |  |  |  |  |  |
| Yes | 63 | Aliased |  |  | 2.853 | 2.473 | 3.291 |
| No | 57 | 0.080 | 0.050 | N.S. | 3.090 | 2.806 | 3.403 |
| **RR adjusted for factor other than sex, age** |  |  |  |  |  |  |  |
| Yes | 27 | Aliased |  |  | 2.674 | 2.231 | 3.205 |
| No | 93 | 0.155 | 0.057 | ++ | 3.123 | 2.868 | 3.400 |
| **COPD subtype (4)** |  |  |  |  |  |  |  |
| mort | 31 | Aliased |  |  | 3.950 | 3.096 | 5.039 |
| LF | 56 | -0.426 | 0.071 | --- | 2.579 | 2.360 | 2.820 |
| oth-prev | 27 | 0.027 | 0.077 | N.S. | 4.058 | 3.372 | 4.884 |
| oth-inc | 6 | 0.216 | 0.125 | (+) | 4.905 | 3.219 | 7.474 |

Table 1 - B - 6

IESCOPD - Meta-regression of current smoking, any product (or cigarettes if all product not available)

Multiple regression of data from Table 1 - B - 1 (most-adjusted RRs)

Any COPD

Test by adding extra variables one at a time

|  |  | **Deviance** | **(DF)** | **Drop Dev** | **P** |  |  |
| --- | --- | --- | --- | --- | --- | --- | --- |
| **Adding National cigarette tobacco type** |  | 429.588 | (101) | 3.693 | N.S. |  |  |
|  |  | **Estimate** | **S.E.** | **P** | **RR** | **95%CIl** | **95%CIu** |
| **Constant** |  | 1.147 | 0.172 | +++ | 3.150 | 2.251 | 4.409 |
| **Sex(RR)** |  |  |  |  |  |  |  |
| Male | 48 | Aliased |  |  | 3.197 | 2.816 | 3.629 |
| Female | 31 | -0.219 | 0.045 | --- | 2.568 | 2.255 | 2.924 |
| Combined | 41 | -0.011 | 0.045 | N.S. | 3.163 | 2.833 | 3.531 |
| **Continent** |  |  |  |  |  |  |  |
| NAmer | 39 | Aliased |  |  | 3.842 | 3.315 | 4.453 |
| Europe | 55 | -0.355 | 0.052 | --- | 2.695 | 2.414 | 3.009 |
| Asia | 17 | -0.382 | 0.101 | --- | 2.623 | 1.816 | 3.790 |
| oth/mult | 9 | -0.461 | 0.085 | --- | 2.422 | 1.780 | 3.297 |
| **Asthma analysis type (COPD)** |  |  |  |  |  |  |  |
| inc-irresp | 72 | Aliased |  |  | 3.441 | 3.155 | 3.753 |
| excl-all | 17 | -0.327 | 0.052 | --- | 2.481 | 2.094 | 2.940 |
| defn-incl | 18 | -0.715 | 0.075 | --- | 1.683 | 1.258 | 2.253 |
| other | 13 | -0.071 | 0.104 | N.S. | 3.205 | 2.132 | 4.818 |
| **Smoking product (3)** |  |  |  |  |  |  |  |
| any | 46 | Aliased |  |  | 2.521 | 1.980 | 3.209 |
| cigs | 66 | 0.225 | 0.086 | + | 3.157 | 2.764 | 3.606 |
| cigsonly | 8 | 0.493 | 0.099 | +++ | 4.129 | 2.920 | 5.838 |
| **Unexposed group** |  |  |  |  |  |  |  |
| nev any | 58 | Aliased |  |  | 3.803 | 3.158 | 4.580 |
| nev cig | 62 | -0.429 | 0.078 | --- | 2.478 | 2.122 | 2.893 |
| **COPD subtype** |  |  |  |  |  |  |  |
| mort | 31 | Aliased |  |  | 4.046 | 3.160 | 5.179 |
| LF | 56 | -0.453 | 0.072 | --- | 2.572 | 2.352 | 2.813 |
| other | 33 | 0.019 | 0.078 | N.S. | 4.122 | 3.459 | 4.913 |
| **Midpoint age in RR** |  | 0.011 | 0.003 | +++ | 1.599 | 0.881 | 2.901 |
| **RR adjusted for age** |  |  |  |  |  |  |  |
| Yes | 63 | Aliased |  |  | 2.835 | 2.453 | 3.277 |
| No | 57 | 0.090 | 0.051 | (+) | 3.101 | 2.813 | 3.419 |
| **RR adjusted for factor other than sex, age** |  |  |  |  |  |  |  |
| Yes | 27 | Aliased |  |  | 2.785 | 2.256 | 3.438 |
| No | 93 | 0.101 | 0.067 | N.S. | 3.080 | 2.806 | 3.379 |
| **National cigarette tobacco type** |  |  |  |  |  |  |  |
| bl | 90 | Aliased |  |  | 3.042 | 2.821 | 3.281 |
| vir | 23 | -0.018 | 0.067 | N.S. | 2.987 | 2.337 | 3.818 |
| m/u | 7 | -0.199 | 0.104 | (-) | 2.492 | 1.676 | 3.705 |

Table 1 - B - 6

IESCOPD - Meta-regression of current smoking, any product (or cigarettes if all product not available)

Multiple regression of data from Table 1 - B - 1 (most-adjusted RRs)

Any COPD

Fixed model, test by adding extra variables one at a time

|  |  | **Deviance** | **(DF)** | **Drop Dev** | **P** |  |  |
| --- | --- | --- | --- | --- | --- | --- | --- |
| **Adding Publication year** |  | 424.646 | (100) | 8.634 | N.S. |  |  |
|  |  | **Estimate** | **S.E.** | **P** | **RR** | **95%CIl** | **95%CIu** |
| **Constant** |  | 0.922 | 0.167 | +++ | 2.515 | 1.813 | 3.487 |
| **Sex(RR)** |  |  |  |  |  |  |  |
| Male | 48 | Aliased |  |  | 3.193 | 2.813 | 3.624 |
| Female | 31 | -0.217 | 0.045 | --- | 2.569 | 2.256 | 2.925 |
| Combined | 41 | -0.009 | 0.045 | N.S. | 3.164 | 2.835 | 3.532 |
| **Continent** |  |  |  |  |  |  |  |
| NAmer | 39 | Aliased |  |  | 3.881 | 3.351 | 4.496 |
| Europe | 55 | -0.352 | 0.052 | --- | 2.730 | 2.460 | 3.031 |
| Asia | 17 | -0.478 | 0.084 | --- | 2.406 | 1.793 | 3.229 |
| oth/mult | 9 | -0.543 | 0.082 | --- | 2.255 | 1.675 | 3.035 |
| **Asthma analysis type (COPD)** |  |  |  |  |  |  |  |
| inc-irresp | 72 | Aliased |  |  | 3.452 | 3.165 | 3.765 |
| excl-all | 17 | -0.344 | 0.051 | --- | 2.447 | 2.068 | 2.896 |
| defn-incl | 18 | -0.683 | 0.075 | --- | 1.744 | 1.304 | 2.331 |
| other | 13 | -0.102 | 0.105 | N.S. | 3.116 | 2.066 | 4.699 |
| **Smoking product (3)** |  |  |  |  |  |  |  |
| any | 46 | Aliased |  |  | 2.455 | 1.918 | 3.142 |
| cigs | 66 | 0.270 | 0.088 | ++ | 3.216 | 2.806 | 3.686 |
| cigsonly | 8 | 0.460 | 0.100 | +++ | 3.888 | 2.726 | 5.546 |
| **Unexposed group** |  |  |  |  |  |  |  |
| nev any | 58 | Aliased |  |  | 3.857 | 3.181 | 4.675 |
| nev cig | 62 | -0.454 | 0.082 | --- | 2.450 | 2.087 | 2.875 |
| **COPD subtype** |  |  |  |  |  |  |  |
| mort | 31 | Aliased |  |  | 3.850 | 2.911 | 5.092 |
| LF | 56 | -0.390 | 0.083 | --- | 2.607 | 2.368 | 2.871 |
| other | 33 | 0.060 | 0.082 | N.S. | 4.090 | 3.426 | 4.882 |
| **Midpoint age in RR** |  | 0.013 | 0.002 | +++ | 1.427 | 0.808 | 2.523 |
| **RR adjusted for age** |  |  |  |  |  |  |  |
| Yes | 63 | Aliased |  |  | 2.818 | 2.430 | 3.269 |
| No | 57 | 0.099 | 0.052 | (+) | 3.112 | 2.818 | 3.436 |
| **RR adjusted for factor other than sex, age** |  |  |  |  |  |  |  |
| Yes | 27 | Aliased |  |  | 2.740 | 2.267 | 3.312 |
| No | 93 | 0.122 | 0.060 | + | 3.097 | 2.838 | 3.379 |
| **Publication year** |  |  |  |  |  |  |  |
| <1980 | 24 | Aliased |  |  | 2.928 | 2.195 | 3.906 |
| 1980-89 | 11 | -0.080 | 0.125 | N.S. | 2.702 | 1.815 | 4.022 |
| 1990-99 | 18 | 0.195 | 0.083 | + | 3.559 | 2.696 | 4.700 |
| 2000+ | 67 | 0.014 | 0.080 | N.S. | 2.969 | 2.739 | 3.218 |
|  |  | **Deviance** | **(DF)** | **Drop Dev** | **P** |  |  |
| **Adding Study type** |  | 422.497 | (101) | 10.783 | N.S. |  |  |
|  |  | **Estimate** | **S.E.** | **P** | **RR** | **95%CIl** | **95%CIu** |
| **Constant** |  | 1.077 | 0.183 | +++ | 2.936 | 2.050 | 4.203 |
| **Sex(RR)** |  |  |  |  |  |  |  |
| Male | 48 | Aliased |  |  | 3.225 | 2.842 | 3.660 |
| Female | 31 | -0.220 | 0.045 | --- | 2.587 | 2.272 | 2.946 |
| Combined | 41 | -0.031 | 0.046 | N.S. | 3.127 | 2.801 | 3.492 |
| **Continent** |  |  |  |  |  |  |  |
| NAmer | 39 | Aliased |  |  | 3.919 | 3.384 | 4.537 |
| Europe | 55 | -0.366 | 0.052 | --- | 2.718 | 2.451 | 3.015 |
| Asia | 17 | -0.511 | 0.079 | --- | 2.350 | 1.788 | 3.089 |
| oth/mult | 9 | -0.535 | 0.082 | --- | 2.296 | 1.715 | 3.074 |
| **Asthma analysis type (COPD)** |  |  |  |  |  |  |  |
| inc-irresp | 72 | Aliased |  |  | 3.457 | 3.171 | 3.769 |
| excl-all | 17 | -0.349 | 0.051 | --- | 2.438 | 2.063 | 2.880 |
| defn-incl | 18 | -0.694 | 0.076 | --- | 1.727 | 1.291 | 2.309 |
| other | 13 | -0.086 | 0.104 | N.S. | 3.174 | 2.120 | 4.752 |

Table 1 - B - 6

IESCOPD - Meta-regression of current smoking, any product (or cigarettes if all product not available)

Multiple regression of data from Table 1 - B - 1 (most-adjusted RRs)

Any COPD

Test by adding extra variables one at a time

|  |  | **Estimate** | **S.E.** | **P** | **RR** | **95%CIl** | **95%CIu** |
| --- | --- | --- | --- | --- | --- | --- | --- |
| **Smoking product (3)** |  |  |  |  |  |  |  |
| any | 46 | Aliased |  |  | 2.486 | 1.957 | 3.158 |
| cigs | 66 | 0.244 | 0.086 | ++ | 3.174 | 2.782 | 3.621 |
| cigsonly | 8 | 0.518 | 0.099 | +++ | 4.173 | 2.960 | 5.884 |
| **Unexposed group** |  |  |  |  |  |  |  |
| nev any | 58 | Aliased |  |  | 3.775 | 3.140 | 4.538 |
| nev cig | 62 | -0.415 | 0.078 | --- | 2.493 | 2.138 | 2.906 |
| **COPD subtype** |  |  |  |  |  |  |  |
| mort | 31 | Aliased |  |  | 3.192 | 2.203 | 4.624 |
| LF | 56 | -0.166 | 0.112 | N.S. | 2.703 | 2.422 | 3.017 |
| other | 33 | 0.276 | 0.104 | ++ | 4.206 | 3.536 | 5.002 |
| **Midpoint age in RR** |  | 0.012 | 0.002 | +++ | 1.472 | 0.839 | 2.582 |
| **RR adjusted for age** |  |  |  |  |  |  |  |
| Yes | 63 | Aliased |  |  | 2.814 | 2.435 | 3.252 |
| No | 57 | 0.102 | 0.051 | (+) | 3.115 | 2.826 | 3.433 |
| **RR adjusted for factor other than sex, age** |  |  |  |  |  |  |  |
| Yes | 27 | Aliased |  |  | 2.712 | 2.260 | 3.255 |
| No | 93 | 0.136 | 0.058 | + | 3.108 | 2.853 | 3.384 |
| **Study type** |  |  |  |  |  |  |  |
| CC | 14 | Aliased |  |  | 3.995 | 2.539 | 6.285 |
| Pr | 38 | -0.086 | 0.109 | N.S. | 3.667 | 2.744 | 4.901 |
| CS | 68 | -0.338 | 0.122 | -- | 2.848 | 2.600 | 3.121 |
|  |  | **Deviance** | **(DF)** | **Drop Dev** | **P** |  |  |
| **Adding Study weakness** |  | 433.166 | (102) | 0.114 | N.S. |  |  |
|  |  | **Estimate** | **S.E.** | **P** | **RR** | **95%CIl** | **95%CIu** |
| **Constant** |  | 0.993 | 0.165 | +++ | 2.698 | 1.952 | 3.729 |
| **Sex(RR)** |  |  |  |  |  |  |  |
| Male | 48 | Aliased |  |  | 3.191 | 2.811 | 3.622 |
| Female | 31 | -0.217 | 0.045 | --- | 2.570 | 2.255 | 2.928 |
| Combined | 41 | -0.008 | 0.045 | N.S. | 3.165 | 2.835 | 3.535 |
| **Continent** |  |  |  |  |  |  |  |
| NAmer | 39 | Aliased |  |  | 3.881 | 3.340 | 4.509 |
| Europe | 55 | -0.351 | 0.053 | --- | 2.732 | 2.460 | 3.034 |
| Asia | 17 | -0.503 | 0.080 | --- | 2.346 | 1.779 | 3.093 |
| oth/mult | 9 | -0.514 | 0.082 | --- | 2.320 | 1.730 | 3.113 |
| **Asthma analysis type (COPD)** |  |  |  |  |  |  |  |
| inc-irresp | 72 | Aliased |  |  | 3.444 | 3.157 | 3.757 |
| excl-all | 17 | -0.332 | 0.052 | --- | 2.471 | 2.089 | 2.924 |
| defn-incl | 18 | -0.718 | 0.072 | --- | 1.679 | 1.270 | 2.220 |
| other | 13 | -0.056 | 0.103 | N.S. | 3.257 | 2.171 | 4.885 |
| **Smoking product (3)** |  |  |  |  |  |  |  |
| any | 46 | Aliased |  |  | 2.484 | 1.944 | 3.174 |
| cigs | 66 | 0.248 | 0.087 | ++ | 3.182 | 2.782 | 3.639 |
| cigsonly | 8 | 0.500 | 0.100 | +++ | 4.097 | 2.901 | 5.785 |
| **Unexposed group** |  |  |  |  |  |  |  |
| nev any | 58 | Aliased |  |  | 3.824 | 3.166 | 4.617 |
| nev cig | 62 | -0.438 | 0.080 | --- | 2.467 | 2.108 | 2.886 |
| **COPD subtype** |  |  |  |  |  |  |  |
| mort | 31 | Aliased |  |  | 3.993 | 3.110 | 5.127 |
| LF | 56 | -0.440 | 0.073 | --- | 2.571 | 2.350 | 2.814 |
| other | 33 | 0.044 | 0.077 | N.S. | 4.173 | 3.510 | 4.962 |
| **Midpoint age in RR** |  | 0.012 | 0.002 | +++ | 1.479 | 0.838 | 2.607 |
| **RR adjusted for age** |  |  |  |  |  |  |  |
| Yes | 63 | Aliased |  |  | 2.854 | 2.467 | 3.301 |
| No | 57 | 0.079 | 0.051 | N.S. | 3.090 | 2.802 | 3.407 |
| **RR adjusted for factor other than sex, age** |  |  |  |  |  |  |  |
| Yes | 27 | Aliased |  |  | 2.701 | 2.235 | 3.263 |
| No | 93 | 0.142 | 0.059 | + | 3.112 | 2.852 | 3.396 |

Table 1 - B - 6

IESCOPD - Meta-regression of current smoking, any product (or cigarettes if all product not available)

Multiple regression of data from Table 1 - B - 1 (most-adjusted RRs)

Any COPD

Test by adding extra variables one at a time

|  |  | **Estimate** | **S.E.** | **P** | **RR** | **95%CIl** | **95%CIu** |
| --- | --- | --- | --- | --- | --- | --- | --- |
| **Study weakness** |  |  |  |  |  |  |  |
| Yes | 20 | Aliased |  |  | 2.929 | 2.175 | 3.945 |
| No | 100 | 0.026 | 0.076 | N.S. | 3.006 | 2.818 | 3.205 |
|  |  | **Deviance** | **(DF)** | **Drop Dev** | **P** |  |  |
| **Adding Bronchodilator/**  **reversibility (LF only)** |  | 433.094 | (102) | 0.187 | N.S. |  |  |
|  |  | **Estimate** | **S.E.** | **P** | **RR** | **95%CIl** | **95%CIu** |
| **Constant** |  | 0.980 | 0.171 | +++ | 2.665 | 1.906 | 3.728 |
| **Sex(RR)** |  |  |  |  |  |  |  |
| Male | 48 | Aliased |  |  | 3.187 | 2.807 | 3.619 |
| Female | 31 | -0.217 | 0.045 | --- | 2.565 | 2.253 | 2.920 |
| Combined | 41 | -0.005 | 0.045 | N.S. | 3.172 | 2.841 | 3.541 |
| **Continent** |  |  |  |  |  |  |  |
| NAmer | 39 | Aliased |  |  | 3.859 | 3.324 | 4.480 |
| Europe | 55 | -0.344 | 0.052 | --- | 2.735 | 2.465 | 3.035 |
| Asia | 17 | -0.500 | 0.079 | --- | 2.340 | 1.771 | 3.092 |
| oth/mult | 9 | -0.482 | 0.103 | --- | 2.383 | 1.641 | 3.459 |
| **Asthma analysis type (COPD)** |  |  |  |  |  |  |  |
| inc-irresp | 72 | Aliased |  |  | 3.452 | 3.161 | 3.770 |
| excl-all | 17 | -0.339 | 0.052 | --- | 2.461 | 2.077 | 2.915 |
| defn-incl | 18 | -0.722 | 0.072 | --- | 1.677 | 1.269 | 2.215 |
| other | 13 | -0.067 | 0.106 | N.S. | 3.229 | 2.131 | 4.891 |
| **Smoking product (3)** |  |  |  |  |  |  |  |
| any | 46 | Aliased |  |  | 2.467 | 1.946 | 3.128 |
| cigs | 66 | 0.258 | 0.084 | ++ | 3.192 | 2.800 | 3.639 |
| cigsonly | 8 | 0.506 | 0.099 | +++ | 4.090 | 2.896 | 5.777 |
| **Unexposed group** |  |  |  |  |  |  |  |
| nev any | 58 | Aliased |  |  | 3.835 | 3.194 | 4.604 |
| nev cig | 62 | -0.443 | 0.077 | --- | 2.461 | 2.113 | 2.866 |
| **COPD subtype** |  |  |  |  |  |  |  |
| mort | 31 | Aliased |  |  | 3.970 | 3.110 | 5.067 |
| LF | 56 | -0.433 | 0.071 | --- | 2.574 | 2.354 | 2.815 |
| other | 33 | 0.051 | 0.076 | N.S. | 4.178 | 3.513 | 4.969 |
| **Midpoint age in RR** |  | 0.013 | 0.003 | +++ | 1.452 | 0.795 | 2.653 |
| **RR adjusted for age** |  |  |  |  |  |  |  |
| Yes | 63 | Aliased |  |  | 2.862 | 2.480 | 3.303 |
| No | 57 | 0.075 | 0.050 | N.S. | 3.084 | 2.800 | 3.398 |
| **RR adjusted for factor other than sex, age** |  |  |  |  |  |  |  |
| Yes | 27 | Aliased |  |  | 2.678 | 2.225 | 3.222 |
| No | 93 | 0.153 | 0.058 | ++ | 3.121 | 2.863 | 3.402 |
| **Bronchodilator/reversibility (LF only)** |  |  |  |  |  |  |  |
| no/na | 109 | Aliased |  |  | 3.007 | 2.820 | 3.206 |
| yes/revs | 11 | -0.052 | 0.120 | N.S. | 2.854 | 1.777 | 4.583 |
|  |  | **Deviance** | **(DF)** | **Drop Dev** | **P** |  |  |
| **Adding Number of cases (COPD)** |  | 421.740 | (100) | 11.540 | N.S. |  |  |
|  |  | **Estimate** | **S.E.** | **P** | **RR** | **95%CIl** | **95%CIu** |
| **Constant** |  | 1.424 | 0.214 | +++ | 4.154 | 2.730 | 6.322 |
| **Sex(RR)** |  |  |  |  |  |  |  |
| Male | 48 | Aliased |  |  | 3.196 | 2.817 | 3.626 |
| Female | 31 | -0.215 | 0.045 | --- | 2.578 | 2.265 | 2.934 |
| Combined | 41 | -0.013 | 0.045 | N.S. | 3.156 | 2.828 | 3.521 |
| **Continent** |  |  |  |  |  |  |  |
| NAmer | 39 | Aliased |  |  | 3.834 | 3.308 | 4.444 |
| Europe | 55 | -0.337 | 0.052 | --- | 2.736 | 2.466 | 3.037 |
| Asia | 17 | -0.492 | 0.080 | --- | 2.344 | 1.778 | 3.088 |
| oth/mult | 9 | -0.446 | 0.090 | --- | 2.456 | 1.777 | 3.393 |

Table 1 - B - 6

IESCOPD - Meta-regression of current smoking, any product (or cigarettes if all product not available)

Multiple regression of data from Table 1 - B - 1 (most-adjusted RRs)

Any COPD

Test by adding extra variables one at a time

|  |  | **Estimate** | **S.E.** | **P** | **RR** | **95%CIl** | **95%CIu** |
| --- | --- | --- | --- | --- | --- | --- | --- |
| **Asthma analysis type (COPD)** |  |  |  |  |  |  |  |
| inc-irresp | 72 | Aliased |  |  | 3.466 | 3.177 | 3.781 |
| excl-all | 17 | -0.350 | 0.052 | --- | 2.442 | 2.063 | 2.889 |
| defn-incl | 18 | -0.725 | 0.072 | --- | 1.678 | 1.271 | 2.216 |
| other | 13 | -0.087 | 0.104 | N.S. | 3.178 | 2.116 | 4.772 |
| **Smoking product (3)** |  |  |  |  |  |  |  |
| any | 46 | Aliased |  |  | 2.453 | 1.934 | 3.111 |
| cigs | 66 | 0.267 | 0.085 | ++ | 3.204 | 2.812 | 3.652 |
| cigsonly | 8 | 0.501 | 0.100 | +++ | 4.047 | 2.855 | 5.735 |
| **Unexposed group** |  |  |  |  |  |  |  |
| nev any | 58 | Aliased |  |  | 3.839 | 3.198 | 4.608 |
| nev cig | 62 | -0.445 | 0.077 | --- | 2.459 | 2.112 | 2.864 |
| **COPD subtype** |  |  |  |  |  |  |  |
| mort | 31 | Aliased |  |  | 3.950 | 3.095 | 5.041 |
| LF | 56 | -0.429 | 0.071 | --- | 2.573 | 2.353 | 2.814 |
| other | 33 | 0.062 | 0.076 | N.S. | 4.203 | 3.534 | 4.999 |
| **Midpoint age in RR** |  | 0.013 | 0.002 | +++ | 1.435 | 0.814 | 2.530 |
| **RR adjusted for age** |  |  |  |  |  |  |  |
| Yes | 63 | Aliased |  |  | 2.888 | 2.496 | 3.342 |
| No | 57 | 0.061 | 0.052 | N.S. | 3.068 | 2.782 | 3.384 |
| **RR adjusted for factor other than sex, age** |  |  |  |  |  |  |  |
| Yes | 27 | Aliased |  |  | 2.644 | 2.190 | 3.192 |
| No | 93 | 0.170 | 0.059 | ++ | 3.135 | 2.874 | 3.420 |
| **Number of cases (COPD)** |  |  |  |  |  |  |  |
| 1-50 | 21 | Aliased |  |  | 4.803 | 2.575 | 8.958 |
| 51-100 | 23 | -0.455 | 0.174 | - | 3.046 | 2.186 | 4.244 |
| 101-200 | 29 | -0.562 | 0.166 | -- | 2.738 | 2.140 | 3.502 |
| 201+ | 47 | -0.467 | 0.156 | -- | 3.010 | 2.812 | 3.222 |
|  |  | **Deviance** | **(DF)** | **Drop Dev** | **P** |  |  |
| **Adding Smoking results reported in study (COPD)** |  | 433.277 | (102) | 0.003 | N.S. |  |  |
|  |  | **Estimate** | **S.E.** | **P** | **RR** | **95%CIl** | **95%CIu** |
| **Constant** |  | 1.014 | 0.163 | +++ | 2.755 | 2.003 | 3.791 |
| **Sex(RR)** |  |  |  |  |  |  |  |
| Male | 48 | Aliased |  |  | 3.190 | 2.811 | 3.621 |
| Female | 31 | -0.218 | 0.045 | --- | 2.566 | 2.252 | 2.923 |
| Combined | 41 | -0.007 | 0.045 | N.S. | 3.169 | 2.839 | 3.537 |
| **Continent** |  |  |  |  |  |  |  |
| NAmer | 39 | Aliased |  |  | 3.870 | 3.341 | 4.482 |
| Europe | 55 | -0.347 | 0.052 | --- | 2.736 | 2.464 | 3.038 |
| Asia | 17 | -0.499 | 0.079 | --- | 2.350 | 1.781 | 3.100 |
| oth/mult | 9 | -0.510 | 0.081 | --- | 2.324 | 1.732 | 3.117 |
| **Asthma analysis type (COPD)** |  |  |  |  |  |  |  |
| inc-irresp | 72 | Aliased |  |  | 3.446 | 3.156 | 3.761 |
| excl-all | 17 | -0.334 | 0.051 | --- | 2.468 | 2.087 | 2.917 |
| defn-incl | 18 | -0.719 | 0.075 | --- | 1.678 | 1.260 | 2.236 |
| other | 13 | -0.053 | 0.110 | N.S. | 3.267 | 2.130 | 5.009 |
| **Smoking product (3)** |  |  |  |  |  |  |  |
| any | 46 | Aliased |  |  | 2.473 | 1.937 | 3.157 |
| cigs | 66 | 0.254 | 0.089 | ++ | 3.187 | 2.769 | 3.669 |
| cigsonly | 8 | 0.508 | 0.108 | +++ | 4.109 | 2.702 | 6.247 |
| **Unexposed group** |  |  |  |  |  |  |  |
| nev any | 58 | Aliased |  |  | 3.836 | 3.177 | 4.634 |
| nev cig | 62 | -0.444 | 0.080 | --- | 2.460 | 2.102 | 2.879 |
| **COPD subtype** |  |  |  |  |  |  |  |
| mort | 31 | Aliased |  |  | 3.965 | 2.940 | 5.347 |
| LF | 56 | -0.431 | 0.090 | --- | 2.576 | 2.325 | 2.853 |
| other | 33 | 0.051 | 0.083 | N.S. | 4.172 | 3.495 | 4.979 |
| **Midpoint age in RR** |  | 0.012 | 0.002 | +++ | 1.485 | 0.844 | 2.613 |

Table 1 - B - 6

IESCOPD - Meta-regression of current smoking, any product (or cigarettes if all product not available)

Multiple regression of data from Table 1 - B - 1 (most-adjusted RRs)

Any COPD

Test by adding extra variables one at a time

|  |  | **Estimate** | **S.E.** | **P** | **RR** | **95%CIl** | **95%CIu** |
| --- | --- | --- | --- | --- | --- | --- | --- |
| **RR adjusted for age** |  |  |  |  |  |  |  |
| Yes | 63 | Aliased |  |  | 2.861 | 2.473 | 3.311 |
| No | 57 | 0.075 | 0.051 | N.S. | 3.085 | 2.797 | 3.403 |
| **RR adjusted for factor other than sex, age** |  |  |  |  |  |  |  |
| Yes | 27 | Aliased |  |  | 2.689 | 2.243 | 3.223 |
| No | 93 | 0.148 | 0.057 | + | 3.117 | 2.862 | 3.395 |
| **Smoking results reported in study (COPD)** |  |  |  |  |  |  |  |
| Current Only | 10 | Aliased |  |  | 3.017 | 2.107 | 4.319 |
| Both | 110 | -0.005 | 0.095 | N.S. | 3.000 | 2.801 | 3.213 |
|  |  | **Deviance** | **(DF)** | **Drop Dev** | **P** |  |  |
| **Adding Derivation of RR/CI** |  | 428.892 | (102) | 4.389 | N.S. |  |  |
|  |  | **Estimate** | **S.E.** | **P** | **RR** | **95%CIl** | **95%CIu** |
| **Constant** |  | 1.119 | 0.164 | +++ | 3.063 | 2.219 | 4.227 |
| **Sex(RR)** |  |  |  |  |  |  |  |
| Male | 48 | Aliased |  |  | 3.183 | 2.806 | 3.611 |
| Female | 31 | -0.219 | 0.045 | --- | 2.558 | 2.248 | 2.911 |
| Combined | 41 | -0.001 | 0.045 | N.S. | 3.180 | 2.851 | 3.548 |
| **Continent** |  |  |  |  |  |  |  |
| NAmer | 39 | Aliased |  |  | 3.936 | 3.390 | 4.571 |
| Europe | 55 | -0.376 | 0.053 | --- | 2.702 | 2.430 | 3.005 |
| Asia | 17 | -0.508 | 0.079 | --- | 2.369 | 1.801 | 3.117 |
| oth/mult | 9 | -0.518 | 0.081 | --- | 2.344 | 1.750 | 3.139 |
| **Asthma analysis type (COPD)** |  |  |  |  |  |  |  |
| inc-irresp | 72 | Aliased |  |  | 3.454 | 3.169 | 3.766 |
| excl-all | 17 | -0.364 | 0.053 | --- | 2.400 | 2.014 | 2.858 |
| defn-incl | 18 | -0.658 | 0.078 | --- | 1.789 | 1.320 | 2.425 |
| other | 13 | -0.028 | 0.104 | N.S. | 3.361 | 2.236 | 5.051 |
| **Smoking product (3)** |  |  |  |  |  |  |  |
| any | 46 | Aliased |  |  | 2.544 | 1.996 | 3.242 |
| cigs | 66 | 0.214 | 0.087 | + | 3.151 | 2.761 | 3.597 |
| cigsonly | 8 | 0.460 | 0.101 | +++ | 4.030 | 2.855 | 5.689 |
| **Unexposed group** |  |  |  |  |  |  |  |
| nev any | 58 | Aliased |  |  | 3.824 | 3.188 | 4.586 |
| nev cig | 62 | -0.438 | 0.077 | --- | 2.467 | 2.120 | 2.871 |
| **COPD subtype** |  |  |  |  |  |  |  |
| mort | 31 | Aliased |  |  | 3.852 | 3.000 | 4.946 |
| LF | 56 | -0.389 | 0.074 | --- | 2.610 | 2.379 | 2.864 |
| other | 33 | 0.055 | 0.076 | N.S. | 4.072 | 3.405 | 4.869 |
| **Midpoint age in RR** |  | 0.012 | 0.002 | +++ | 1.536 | 0.872 | 2.704 |
| **RR adjusted for age** |  |  |  |  |  |  |  |
| Yes | 63 | Aliased |  |  | 2.861 | 2.481 | 3.299 |
| No | 57 | 0.076 | 0.050 | N.S. | 3.085 | 2.802 | 3.397 |
| **RR adjusted for factor other than sex, age** |  |  |  |  |  |  |  |
| Yes | 27 | Aliased |  |  | 2.723 | 2.270 | 3.267 |
| No | 93 | 0.131 | 0.057 | + | 3.103 | 2.850 | 3.380 |
| **Derivation of RR/CI** |  |  |  |  |  |  |  |
| Orig/2x2 | 48 | Aliased |  |  | 3.157 | 2.814 | 3.542 |
| Other | 72 | -0.094 | 0.045 | - | 2.874 | 2.590 | 3.188 |
|  |  | **Deviance** | **(DF)** | **Drop Dev** | **P** |  |  |
| **Adding Analysis type** |  | 432.181 | (102) | 1.099 | N.S. |  |  |
|  |  | **Estimate** | **S.E.** | **P** | **RR** | **95%CIl** | **95%CIu** |
| **Constant** |  | 0.937 | 0.171 | +++ | 2.551 | 1.824 | 3.570 |
| **Sex(RR)** |  |  |  |  |  |  |  |
| Male | 48 | Aliased |  |  | 3.195 | 2.815 | 3.626 |
| Female | 31 | -0.215 | 0.045 | --- | 2.576 | 2.261 | 2.935 |
| Combined | 41 | -0.012 | 0.045 | N.S. | 3.157 | 2.827 | 3.526 |

Table 1 - B - 6

IESCOPD - Meta-regression of current smoking, any product (or cigarettes if all product not available)

Multiple regression of data from Table 1 - B - 1 (most-adjusted RRs)

Any COPD

Test by adding extra variables one at a time

|  |  | **Estimate** | **S.E.** | **P** | **RR** | **95%CIl** | **95%CIu** |
| --- | --- | --- | --- | --- | --- | --- | --- |
| **Continent** |  |  |  |  |  |  |  |
| NAmer | 39 | Aliased |  |  | 3.880 | 3.350 | 4.493 |
| Europe | 55 | -0.351 | 0.052 | --- | 2.731 | 2.461 | 3.030 |
| Asia | 17 | -0.500 | 0.079 | --- | 2.353 | 1.787 | 3.097 |
| oth/mult | 9 | -0.512 | 0.081 | --- | 2.325 | 1.735 | 3.116 |
| **Asthma analysis type (COPD)** |  |  |  |  |  |  |  |
| inc-irresp | 72 | Aliased |  |  | 3.441 | 3.155 | 3.753 |
| excl-all | 17 | -0.335 | 0.051 | --- | 2.462 | 2.082 | 2.910 |
| defn-incl | 18 | -0.695 | 0.076 | --- | 1.717 | 1.281 | 2.303 |
| other | 13 | -0.055 | 0.103 | N.S. | 3.255 | 2.172 | 4.879 |
| **Smoking product (3)** |  |  |  |  |  |  |  |
| any | 46 | Aliased |  |  | 2.485 | 1.959 | 3.153 |
| cigs | 66 | 0.246 | 0.085 | ++ | 3.180 | 2.789 | 3.626 |
| cigsonly | 8 | 0.503 | 0.099 | +++ | 4.110 | 2.911 | 5.803 |
| **Unexposed group** |  |  |  |  |  |  |  |
| nev any | 58 | Aliased |  |  | 3.820 | 3.181 | 4.588 |
| nev cig | 62 | -0.437 | 0.077 | --- | 2.469 | 2.119 | 2.876 |
| **COPD subtype** |  |  |  |  |  |  |  |
| mort | 31 | Aliased |  |  | 3.743 | 2.674 | 5.238 |
| LF | 56 | -0.360 | 0.101 | --- | 2.612 | 2.351 | 2.903 |
| other | 33 | 0.107 | 0.094 | N.S. | 4.163 | 3.501 | 4.951 |
| **Midpoint age in RR** |  | 0.012 | 0.002 | +++ | 1.480 | 0.841 | 2.602 |
| **RR adjusted for age** |  |  |  |  |  |  |  |
| Yes | 63 | Aliased |  |  | 2.847 | 2.465 | 3.288 |
| No | 57 | 0.083 | 0.051 | N.S. | 3.094 | 2.807 | 3.409 |
| **RR adjusted for factor other than sex, age** |  |  |  |  |  |  |  |
| Yes | 27 | Aliased |  |  | 2.701 | 2.252 | 3.240 |
| No | 93 | 0.142 | 0.057 | + | 3.112 | 2.857 | 3.390 |
| **Analysis type** |  |  |  |  |  |  |  |
| prevlnce | 84 | Aliased |  |  | 2.960 | 2.729 | 3.212 |
| onset | 36 | 0.083 | 0.079 | N.S. | 3.216 | 2.448 | 4.224 |

Table 1 - B - 6

Table 1 - B - 6

IESCOPD - Meta-regression of current smoking, any product (or cigarettes if all product not available)

Multiple regression of data from Table 1 - B - 1 (most-adjusted RRs)

Any COPD

Fitted values and residuals

|  | **Study Ref** | **NRR** | **SEX** | **LOGRR** | **FITVAL** | **SEFITV** | **STDRES** |
| --- | --- | --- | --- | --- | --- | --- | --- |
|  |  |  |  |  |  |  |  |
| #189 | MANNI3 | 1 | b | 0.331 | 1.242 | 0.445 | -2.046 |
| #1166 | MENEZ3 | 1 | b | 0.199 | 0.938 | 0.374 | -1.977 |
| #411 | EKBERG | 1 | m | 0.926 | 1.151 | 0.125 | -1.802 |
| #328 | MONTNE | 1 | b | 0.940 | 1.407 | 0.268 | -1.744 |
| #325 | LAM2 | 1 | m | 0.851 | 1.506 | 0.388 | -1.690 |
| #55 | LINDST | 7 | b | 0.693 | 1.054 | 0.238 | -1.514 |
| #2251 | ZIELI2 | 4 | f | 0.276 | 0.409 | 0.092 | -1.447 |
| #95 | HO | 1 | m | 0.772 | 1.567 | 0.608 | -1.309 |
| #522 | MANNI1 | 2 | m | 1.247 | 1.542 | 0.241 | -1.223 |
| #503 | HAMMO2 | 2 | f | 1.773 | 2.266 | 0.413 | -1.193 |
| #1174 | MENEZ4 | 1 | b | 0.278 | 0.938 | 0.599 | -1.103 |
| #473 | DEAN1 | 15 | f | 0.637 | 1.051 | 0.378 | -1.094 |
| #1182 | MENEZ5 | 1 | b | 0.476 | 0.938 | 0.426 | -1.084 |
| #131 | MANNI2 | 1 | b | 1.122 | 1.317 | 0.181 | -1.079 |
| #1337 | WEN | 1 | m | 0.829 | 1.290 | 0.441 | -1.046 |
| #532 | HIGGI4 | 1 | m | 1.075 | 1.733 | 0.634 | -1.038 |
| #2348 | SAWICK | 2 | f | 0.159 | 0.774 | 0.630 | -0.976 |
| #572 | SILVA | 1 | b | 1.065 | 1.585 | 0.557 | -0.934 |
| #502 | HAMMO2 | 1 | m | 2.176 | 2.484 | 0.334 | -0.923 |
| #35 | LAM3 | 1 | m | 0.378 | 1.234 | 0.962 | -0.889 |
| #180 | ANDER1 | 4 | f | 0.077 | 0.869 | 0.936 | -0.846 |
| #139 | VIEGI2 | 4 | m | 0.322 | 0.787 | 0.567 | -0.820 |
| #101 | ALESSA | 1 | b | 0.395 | 1.511 | 1.406 | -0.793 |
| #493 | MARCUS | 1 | m | 0.747 | 1.234 | 0.631 | -0.772 |
| #2981 | VESTBO | 1 | b | 0.719 | 0.834 | 0.153 | -0.755 |
| #2244 | TODD | 44 | f | -0.301 | 1.304 | 2.299 | -0.698 |
| #31 | CHEN3 | 4 | f | 1.135 | 1.328 | 0.288 | -0.670 |
| #30 | CHEN3 | 3 | m | 1.169 | 1.545 | 0.565 | -0.665 |
| #2821 | LEBOWI | 16 | f | 0.663 | 0.990 | 0.501 | -0.653 |
| #426 | NIHLEN | 1 | b | 1.124 | 1.456 | 0.527 | -0.630 |
| #374 | ANDER3 | 4 | f | -1.099 | 0.933 | 3.686 | -0.551 |
| #2316 | LEE | 50 | f | 0.658 | 1.304 | 1.199 | -0.539 |
| #518 | TRUPIN | 4 | b | 1.482 | 1.658 | 0.337 | -0.525 |
| #464 | HARIKK | 1 | m | 1.500 | 1.794 | 0.891 | -0.329 |
| #420 | NILSSO | 7 | m | 1.179 | 1.374 | 0.655 | -0.298 |
| #91 | KARAKA | 1 | b | 1.107 | 1.293 | 0.642 | -0.291 |
| #377 | BEDNAR | 1 | b | 0.720 | 0.896 | 0.623 | -0.282 |
| #488 | SARGEA | 7 | b | 0.470 | 0.517 | 0.192 | -0.246 |
| #388 | KRZYZA | 4 | f | 0.445 | 0.594 | 0.610 | -0.244 |
| #559 | MUELLE | 12 | f | 0.621 | 1.045 | 1.908 | -0.223 |
| #145 | VIEGI2 | 10 | f | 0.457 | 0.569 | 0.516 | -0.216 |
| #133 | RICCIO | 1 | b | 0.618 | 0.681 | 0.301 | -0.209 |
| #3620 | BEST | 35 | m | 2.259 | 2.435 | 0.859 | -0.205 |
| #610 | YAMAGU | 1 | b | 0.683 | 0.725 | 0.226 | -0.187 |
| #22 | CHEN2 | 7 | m | 1.314 | 1.469 | 0.872 | -0.178 |
| #167 | KIM | 4 | f | 0.554 | 0.699 | 0.820 | -0.177 |
| #32 | WEISS | 1 | m | 1.282 | 1.522 | 1.542 | -0.156 |
| #171 | SHIN | 1 | m | 0.728 | 0.821 | 0.664 | -0.140 |
| #195 | CLEMEN | 1 | m | 0.852 | 0.943 | 0.685 | -0.132 |
| #164 | KIM | 1 | m | 0.850 | 0.917 | 0.509 | -0.131 |
| #1441 | SHAHAB | 4 | b | 0.837 | 0.852 | 0.156 | -0.097 |
| #98 | HO | 4 | f | 1.290 | 1.350 | 0.762 | -0.079 |
| #1190 | MENEZ6 | 1 | b | 0.948 | 0.938 | 0.459 | 0.021 |
| #2281 | LEE | 15 | m | 1.575 | 1.522 | 2.076 | 0.026 |
| #361 | YUAN | 4 | m | 0.329 | 0.311 | 0.600 | 0.030 |
| #504 | THUN | 1 | m | 2.460 | 2.447 | 0.261 | 0.047 |
| #299 | STERLI | 1 | m | 1.991 | 1.918 | 0.597 | 0.122 |
| #432 | STROM | 1 | m | 1.370 | 1.285 | 0.638 | 0.132 |
| #104 | COCCI | 1 | b | 1.834 | 1.394 | 3.146 | 0.140 |
| #580 | KAHN | 1 | m | 2.309 | 2.286 | 0.153 | 0.153 |
| #479 | RENWIC | 1 | b | 1.482 | 1.328 | 0.865 | 0.178 |
| #2215 | TODD | 15 | m | 1.901 | 1.522 | 2.069 | 0.183 |

Table 1 - B - 6

IESCOPD - Meta-regression of current smoking, any product (or cigarettes if all product not available)

Multiple regression of data from Table 1 - B - 1 (most-adjusted RRs)

Any COPD

Fitted values and residuals

|  | **Study Ref** | **NRR** | **SEX** | **LOGRR** | **FITVAL** | **SEFITV** | **STDRES** |
| --- | --- | --- | --- | --- | --- | --- | --- |
|  |  |  |  |  |  |  |  |
| #454 | FORAST | 4 | f | 1.539 | 1.408 | 0.687 | 0.190 |
| #506 | KATANC | 1 | b | 1.666 | 1.509 | 0.741 | 0.212 |
| #597 | FERRI2 | 8 | f | 0.997 | 0.906 | 0.405 | 0.225 |
| #385 | KRZYZA | 1 | m | 1.044 | 0.811 | 0.943 | 0.247 |
| #551 | MUELLE | 4 | m | 1.651 | 1.263 | 1.552 | 0.250 |
| #576 | VOLLM2 | 1 | b | 1.814 | 1.266 | 2.106 | 0.260 |
| #38 | XIAO | 1 | b | 1.451 | 1.230 | 0.706 | 0.313 |
| #569 | LEBOWI | 4 | m | 1.583 | 1.207 | 1.164 | 0.323 |
| #528 | MANNI1 | 8 | f | 1.399 | 1.324 | 0.222 | 0.336 |
| #2324 | TANG | 2 | m | 1.690 | 1.484 | 0.600 | 0.344 |
| #25 | CHEN2 | 10 | f | 1.437 | 1.252 | 0.515 | 0.361 |
| #13 | MENEZ2 | 4 | b | 0.875 | 0.714 | 0.442 | 0.365 |
| #148 | FUKUCH | 1 | b | 1.085 | 0.949 | 0.339 | 0.401 |
| #194 | ANDER3 | 1 | m | 2.633 | 1.151 | 3.254 | 0.455 |
| #469 | DEAN1 | 11 | m | 0.944 | 0.763 | 0.387 | 0.467 |
| #177 | ANDER1 | 1 | m | 1.845 | 1.087 | 1.586 | 0.478 |
| #369 | JACOBS | 4 | m | 1.221 | 1.014 | 0.432 | 0.479 |
| #317 | HUHTI1 | 12 | f | 0.896 | 0.559 | 0.703 | 0.480 |
| #448 | DOLL2 | 1 | f | 2.913 | 1.766 | 2.144 | 0.535 |
| #509 | SPEIZE | 1 | m | 2.500 | 0.921 | 2.946 | 0.536 |
| #435 | MARAN2 | 1 | b | 1.827 | 1.499 | 0.563 | 0.583 |
| #304 | DEJONG | 1 | b | 3.113 | 1.301 | 2.951 | 0.614 |
| #590 | FERRI2 | 1 | m | 1.554 | 1.123 | 0.690 | 0.624 |
| #496 | PETO | 1 | m | 3.290 | 1.407 | 2.897 | 0.650 |
| #2347 | SAWICK | 1 | m | 1.708 | 0.992 | 1.065 | 0.673 |
| #1425 | MADOR | 1 | m | 3.281 | 0.974 | 3.419 | 0.675 |
| #185 | JOHANN | 1 | b | 1.270 | 0.917 | 0.517 | 0.682 |
| #512 | SPEIZE | 4 | f | 1.495 | 0.704 | 1.138 | 0.696 |
| #467 | HARIKK | 4 | f | 3.749 | 1.576 | 3.003 | 0.724 |
| #128 | HARDIE | 4 | f | 1.227 | 0.760 | 0.640 | 0.729 |
| #408 | NILSSO | 4 | f | 1.969 | 1.661 | 0.410 | 0.750 |
| #346 | DEMARC | 4 | b | 0.703 | 0.527 | 0.223 | 0.788 |
| #423 | LINDBE | 1 | b | 1.515 | 1.018 | 0.625 | 0.796 |
| #577 | NIEPSU | 1 | b | 1.507 | 0.795 | 0.887 | 0.802 |
| #321 | MARAN1 | 1 | b | 1.820 | 1.499 | 0.357 | 0.898 |
| #397 | ZIETKO | 3 | f | 3.279 | 0.409 | 3.158 | 0.909 |
| #380 | KACHEL | 1 | b | 1.672 | 0.856 | 0.856 | 0.954 |
| #395 | ZIETKO | 1 | m | 3.845 | 0.627 | 3.049 | 1.056 |
| #76 | HUHTI3 | 4 | m | 2.193 | 0.952 | 1.137 | 1.091 |
| #414 | EKBERG | 4 | f | 1.149 | 0.933 | 0.194 | 1.110 |
| #190 | TVERDA | 1 | m | 1.295 | 0.365 | 0.829 | 1.122 |
| #125 | HARDIE | 1 | m | 2.050 | 0.978 | 0.853 | 1.257 |
| #446 | DOLL1 | 1 | m | 2.093 | 1.330 | 0.594 | 1.284 |
| #158 | KOJIMA | 4 | f | 1.499 | 0.160 | 1.033 | 1.296 |
| #476 | DICKIN | 1 | b | 3.782 | 0.932 | 2.159 | 1.320 |
| #9 | PEAT | 3 | b | 1.579 | 0.839 | 0.535 | 1.382 |
| #82 | VONHER | 4 | f | 1.904 | 1.502 | 0.279 | 1.437 |
| #505 | THUN | 2 | f | 2.549 | 2.230 | 0.222 | 1.443 |
| #1429 | MATHES | 1 | b | 2.008 | 0.736 | 0.881 | 1.445 |
| #2248 | ZIELI2 | 1 | m | 0.802 | 0.627 | 0.121 | 1.447 |
| #1443 | TSUSHI | 1 | b | 1.757 | 0.468 | 0.873 | 1.476 |
| #363 | GODTFR | 1 | b | 1.848 | 1.428 | 0.275 | 1.530 |
| #300 | STERLI | 2 | f | 2.536 | 1.700 | 0.524 | 1.595 |
| #155 | KOJIMA | 1 | m | 1.216 | 0.377 | 0.509 | 1.648 |
| #1345 | HOZAWA | 1 | b | 1.437 | 1.264 | 0.102 | 1.694 |
| #64 | HUHTI1 | 4 | m | 2.394 | 0.776 | 0.839 | 1.928 |
| #357 | LUNDB1 | 7 | b | 1.876 | 0.779 | 0.546 | 2.009 |
| #79 | VONHER | 1 | m | 2.391 | 1.720 | 0.292 | 2.298 |

## Table 2 - A - 6

IESCOPD - Meta-regression of ever smoking, any product (or cigarettes if all product not available)

Multiple regression of data from Table 2 - A - 1 (most-adjusted RRs)

Any CB

Fixed model

|  |  | **Deviance** | **(DF)** |  |  |  |  |
| --- | --- | --- | --- | --- | --- | --- | --- |
| **Fixed model** |  | 402.328 | (98) |  |  |  |  |
|  |  | **Estimate** | **S.E.** | **P** | **RR** | **95%CIl** | **95%CIu** |
| **Constant** |  | 1.316 | 0.266 | +++ | 3.729 | 2.214 | 6.282 |
| **Sex(RR)** |  |  |  |  |  |  |  |
| Male | 51 | Aliased |  |  | 2.659 | 2.286 | 3.092 |
| Female | 39 | -0.171 | 0.036 | --- | 2.242 | 1.939 | 2.592 |
| Combined | 24 | -0.121 | 0.077 | N.S. | 2.357 | 1.971 | 2.818 |
| **Continent** |  |  |  |  |  |  |  |
| NAmer | 38 | Aliased |  |  | 2.997 | 2.628 | 3.418 |
| Europe | 60 | -0.278 | 0.039 | --- | 2.270 | 2.123 | 2.427 |
| Asia | 9 | -0.281 | 0.060 | --- | 2.263 | 1.895 | 2.703 |
| oth/mult | 7 | -0.177 | 0.074 | - | 2.512 | 1.929 | 3.270 |
| **Asthma analysis type (CB)** |  |  |  |  |  |  |  |
| inc-irresp | 103 | Aliased |  |  | 2.420 | 2.296 | 2.551 |
| excl-all | 7 | -0.109 | 0.066 | (-) | 2.170 | 1.687 | 2.791 |
| defn-incl | 0 | Aliased |  |  | 2.420 | 2.296 | 2.551 |
| other | 4 | 0.996 | 0.214 | +++ | 6.551 | 2.804 | 15.306 |
| **Smoking product (3)** |  |  |  |  |  |  |  |
| any | 61 | Aliased |  |  | 2.467 | 1.985 | 3.068 |
| cigs | 51 | -0.060 | 0.128 | N.S. | 2.324 | 1.719 | 3.142 |
| cigsonly | 2 | 0.449 | 0.149 | ++ | 3.868 | 2.066 | 7.240 |
| **Unexposed group** |  |  |  |  |  |  |  |
| nev any | 70 | Aliased |  |  | 2.526 | 2.052 | 3.111 |
| nev cig | 44 | -0.111 | 0.126 | N.S. | 2.260 | 1.668 | 3.061 |
| **CB subtype** |  |  |  |  |  |  |  |
| mort | 3 | Aliased |  |  | 3.046 | 1.140 | 8.142 |
| sympt | 83 | -0.212 | 0.249 | N.S. | 2.464 | 2.318 | 2.620 |
| other | 28 | -0.302 | 0.248 | N.S. | 2.252 | 1.985 | 2.554 |
| **Midpoint age in RR** |  | 0.003 | 0.001 | + | 2.090 | 1.641 | 2.661 |
| **RR adjusted for age** |  |  |  |  |  |  |  |
| Yes | 63 | Aliased |  |  | 2.201 | 2.043 | 2.372 |
| No | 51 | 0.214 | 0.032 | +++ | 2.726 | 2.497 | 2.976 |
| **RR adjusted for factor other than sex, age** |  |  |  |  |  |  |  |
| Yes | 30 | Aliased |  |  | 2.572 | 2.186 | 3.026 |
| No | 84 | -0.113 | 0.070 | N.S. | 2.298 | 2.012 | 2.624 |

Table 2 - A - 6

IESCOPD - Meta-regression of ever smoking, any product (or cigarettes if all product not available)

Multiple regression of data from Table 2 - A - 1 (most-adjusted RRs)

Any CB

Test by removing variables one at a time

|  |  | **Deviance** | **(DF)** | **Drop Dev** | **P** |  |  |
| --- | --- | --- | --- | --- | --- | --- | --- |
| **Omitting Sex (RR)** |  | 425.010 | (100) | -22.681 | (*) |  |  |
|  |  | **Estimate** | **S.E.** | **P** | **RR** | **95%CIl** | **95%CIu** |
| **Constant** |  | 1.347 | 0.254 | +++ | 3.847 | 2.340 | 6.323 |
| **RR adjusted for factor other than sex, age** |  |  |  |  |  |  |  |
| Yes | 30 | Aliased |  |  | 2.522 | 2.318 | 2.744 |
| No | 84 | -0.077 | 0.031 | - | 2.334 | 2.168 | 2.512 |
| **Continent** |  |  |  |  |  |  |  |
| NAmer | 38 | Aliased |  |  | 3.026 | 2.669 | 3.431 |
| Europe | 60 | -0.292 | 0.037 | --- | 2.261 | 2.119 | 2.412 |
| Asia | 9 | -0.284 | 0.059 | --- | 2.279 | 1.911 | 2.716 |
| oth/mult | 7 | -0.188 | 0.074 | - | 2.507 | 1.936 | 3.245 |
| **Asthma analysis type (CB)** |  |  |  |  |  |  |  |
| inc-irresp | 103 | Aliased |  |  | 2.425 | 2.302 | 2.555 |
| excl-all | 7 | -0.146 | 0.065 | - | 2.095 | 1.635 | 2.683 |
| defn-incl | 0 | Aliased |  |  | 2.425 | 2.302 | 2.555 |
| other | 4 | 0.983 | 0.214 | +++ | 6.478 | 2.797 | 15.005 |
| **Smoking product (3)** |  |  |  |  |  |  |  |
| any | 61 | Aliased |  |  | 2.504 | 2.023 | 3.100 |
| cigs | 51 | -0.094 | 0.127 | N.S. | 2.280 | 1.696 | 3.064 |
| cigsonly | 2 | 0.369 | 0.148 | + | 3.620 | 1.956 | 6.700 |
| **Unexposed group** |  |  |  |  |  |  |  |
| nev any | 70 | Aliased |  |  | 2.507 | 2.043 | 3.077 |
| nev cig | 44 | -0.092 | 0.125 | N.S. | 2.286 | 1.695 | 3.082 |
| **CB subtype** |  |  |  |  |  |  |  |
| mort | 3 | Aliased |  |  | 3.344 | 1.268 | 8.819 |
| sympt | 83 | -0.306 | 0.248 | N.S. | 2.462 | 2.317 | 2.616 |
| other | 28 | -0.394 | 0.247 | N.S. | 2.256 | 1.992 | 2.554 |
| **Midpoint age in RR** |  | 0.002 | 0.001 | (+) | 2.180 | 1.734 | 2.739 |
| **RR adjusted for age** |  |  |  |  |  |  |  |
| Yes | 63 | Aliased |  |  | 2.184 | 2.029 | 2.349 |
| No | 51 | 0.232 | 0.031 | +++ | 2.755 | 2.529 | 3.001 |

Table 2 - A - 6

IESCOPD - Meta-regression of ever smoking, any product (or cigarettes if all product not available)

Multiple regression of data from Table 2 - A - 1 (most-adjusted RRs)

Any CB

Test by removing variables one at a time

|  |  | **Deviance** | **(DF)** | **Drop Dev** | **P** |  |  |
| --- | --- | --- | --- | --- | --- | --- | --- |
| **Omitting Continent** |  | 453.998 | (101) | -51.669 | ** |  |  |
|  |  | **Estimate** | **S.E.** | **P** | **RR** | **95%CIl** | **95%CIu** |
| **Constant** |  | 1.553 | 0.263 | +++ | 4.723 | 2.823 | 7.903 |
| **RR adjusted for factor other than sex, age** |  |  |  |  |  |  |  |
| Yes | 30 | Aliased |  |  | 2.671 | 2.286 | 3.121 |
| No | 84 | -0.181 | 0.068 | -- | 2.230 | 1.963 | 2.533 |
| **Sex(RR)** |  |  |  |  |  |  |  |
| Male | 51 | Aliased |  |  | 2.867 | 2.488 | 3.304 |
| Female | 39 | -0.187 | 0.036 | --- | 2.378 | 2.071 | 2.731 |
| Combined | 24 | -0.293 | 0.072 | --- | 2.139 | 1.811 | 2.526 |
| **Asthma analysis type (CB)** |  |  |  |  |  |  |  |
| inc-irresp | 103 | Aliased |  |  | 2.415 | 2.293 | 2.543 |
| excl-all | 7 | -0.058 | 0.065 | N.S. | 2.279 | 1.783 | 2.912 |
| defn-incl | 0 | Aliased |  |  | 2.415 | 2.293 | 2.543 |
| other | 4 | 0.901 | 0.214 | +++ | 5.944 | 2.582 | 13.685 |
| **Smoking product (3)** |  |  |  |  |  |  |  |
| any | 61 | Aliased |  |  | 2.379 | 1.961 | 2.887 |
| cigs | 51 | 0.029 | 0.115 | N.S. | 2.450 | 1.876 | 3.199 |
| cigsonly | 2 | 0.367 | 0.148 | + | 3.433 | 1.870 | 6.303 |
| **Unexposed group** |  |  |  |  |  |  |  |
| nev any | 70 | Aliased |  |  | 2.574 | 2.134 | 3.106 |
| nev cig | 44 | -0.158 | 0.115 | N.S. | 2.197 | 1.673 | 2.887 |
| **CB subtype** |  |  |  |  |  |  |  |
| mort | 3 | Aliased |  |  | 3.645 | 1.391 | 9.548 |
| sympt | 83 | -0.399 | 0.247 | N.S. | 2.445 | 2.303 | 2.597 |
| other | 28 | -0.458 | 0.247 | (-) | 2.305 | 2.039 | 2.606 |
| **Midpoint age in RR** |  | -0.000 | 0.001 | N.S. | 2.464 | 2.003 | 3.031 |
| **RR adjusted for age** |  |  |  |  |  |  |  |
| Yes | 63 | Aliased |  |  | 2.226 | 2.071 | 2.392 |
| No | 51 | 0.188 | 0.030 | +++ | 2.687 | 2.471 | 2.922 |
|  |  | **Deviance** | **(DF)** | **Drop Dev** | **P** |  |  |
| **Omitting Asthma analysis type (CB)** |  | 427.535 | (100) | -25.206 | * |  |  |
|  |  | **Estimate** | **S.E.** | **P** | **RR** | **95%CIl** | **95%CIu** |
| **Constant** |  | 1.329 | 0.266 | +++ | 3.776 | 2.242 | 6.360 |
| **RR adjusted for factor other than sex, age** |  |  |  |  |  |  |  |
| Yes | 30 | Aliased |  |  | 2.573 | 2.192 | 3.020 |
| No | 84 | -0.114 | 0.069 | N.S. | 2.297 | 2.015 | 2.618 |
| **Sex(RR)** |  |  |  |  |  |  |  |
| Male | 51 | Aliased |  |  | 2.672 | 2.301 | 3.101 |
| Female | 39 | -0.176 | 0.036 | --- | 2.239 | 1.940 | 2.585 |
| Combined | 24 | -0.128 | 0.077 | (-) | 2.350 | 1.968 | 2.805 |
| **Continent** |  |  |  |  |  |  |  |
| NAmer | 38 | Aliased |  |  | 2.951 | 2.594 | 3.358 |
| Europe | 60 | -0.259 | 0.038 | --- | 2.279 | 2.133 | 2.434 |
| Asia | 9 | -0.257 | 0.059 | --- | 2.281 | 1.916 | 2.716 |
| oth/mult | 7 | -0.162 | 0.074 | - | 2.509 | 1.932 | 3.258 |
| **Smoking product (3)** |  |  |  |  |  |  |  |
| any | 61 | Aliased |  |  | 2.468 | 1.989 | 3.062 |
| cigs | 51 | -0.060 | 0.128 | N.S. | 2.324 | 1.724 | 3.132 |
| cigsonly | 2 | 0.441 | 0.149 | ++ | 3.836 | 2.067 | 7.120 |

Table 2 - A - 6

IESCOPD - Meta-regression of ever smoking, any product (or cigarettes if all product not available)

Multiple regression of data from Table 2 - A - 1 (most-adjusted RRs)

Any CB

Test by removing variables one at a time

|  |  | **Estimate** | **S.E.** | **P** | **RR** | **95%CIl** | **95%CIu** |
| --- | --- | --- | --- | --- | --- | --- | --- |
| **Unexposed group** |  |  |  |  |  |  |  |
| nev any | 70 | Aliased |  |  | 2.543 | 2.070 | 3.123 |
| nev cig | 44 | -0.128 | 0.126 | N.S. | 2.238 | 1.658 | 3.021 |
| **CB subtype** |  |  |  |  |  |  |  |
| mort | 3 | Aliased |  |  | 3.060 | 1.156 | 8.099 |
| sympt | 83 | -0.217 | 0.248 | N.S. | 2.463 | 2.320 | 2.615 |
| other | 28 | -0.306 | 0.248 | N.S. | 2.254 | 2.000 | 2.540 |
| **Midpoint age in RR** |  | 0.003 | 0.001 | + | 2.113 | 1.665 | 2.682 |
| **RR adjusted for age** |  |  |  |  |  |  |  |
| Yes | 63 | Aliased |  |  | 2.206 | 2.049 | 2.375 |
| No | 51 | 0.209 | 0.032 | +++ | 2.719 | 2.492 | 2.965 |
|  |  | **Deviance** | **(DF)** | **Drop Dev** | **P** |  |  |
| **Omitting Smoking product (3)** |  | 411.624 | (100) | -9.296 | N.S. |  |  |
|  |  | **Estimate** | **S.E.** | **P** | **RR** | **95%CIl** | **95%CIu** |
| **Constant** |  | 1.410 | 0.264 | +++ | 4.096 | 2.440 | 6.875 |
| **RR adjusted for factor other than sex, age** |  |  |  |  |  |  |  |
| Yes | 30 | Aliased |  |  | 2.571 | 2.190 | 3.017 |
| No | 84 | -0.112 | 0.069 | N.S. | 2.299 | 2.017 | 2.620 |
| **Sex(RR)** |  |  |  |  |  |  |  |
| Male | 51 | Aliased |  |  | 2.659 | 2.293 | 3.084 |
| Female | 39 | -0.159 | 0.036 | --- | 2.268 | 1.967 | 2.616 |
| Combined | 24 | -0.129 | 0.076 | (-) | 2.338 | 1.962 | 2.787 |
| **Continent** |  |  |  |  |  |  |  |
| NAmer | 38 | Aliased |  |  | 2.976 | 2.614 | 3.388 |
| Europe | 60 | -0.267 | 0.038 | --- | 2.279 | 2.135 | 2.433 |
| Asia | 9 | -0.278 | 0.060 | --- | 2.253 | 1.890 | 2.685 |
| oth/mult | 7 | -0.181 | 0.068 | -- | 2.483 | 1.961 | 3.145 |
| **Asthma analysis type (CB)** |  |  |  |  |  |  |  |
| inc-irresp | 103 | Aliased |  |  | 2.423 | 2.300 | 2.552 |
| excl-all | 7 | -0.125 | 0.065 | (-) | 2.137 | 1.667 | 2.740 |
| defn-incl | 0 | Aliased |  |  | 2.423 | 2.300 | 2.552 |
| other | 4 | 0.967 | 0.214 | +++ | 6.373 | 2.753 | 14.752 |
| **Unexposed group** |  |  |  |  |  |  |  |
| nev any | 70 | Aliased |  |  | 2.587 | 2.416 | 2.770 |
| nev cig | 44 | -0.171 | 0.029 | --- | 2.181 | 2.003 | 2.375 |
| **CB subtype** |  |  |  |  |  |  |  |
| mort | 3 | Aliased |  |  | 3.292 | 1.250 | 8.666 |
| sympt | 83 | -0.294 | 0.247 | N.S. | 2.453 | 2.309 | 2.606 |
| other | 28 | -0.366 | 0.247 | N.S. | 2.284 | 2.019 | 2.583 |
| **Midpoint age in RR** |  | 0.003 | 0.001 | + | 2.124 | 1.674 | 2.696 |
| **RR adjusted for age** |  |  |  |  |  |  |  |
| Yes | 63 | Aliased |  |  | 2.209 | 2.054 | 2.376 |
| No | 51 | 0.206 | 0.031 | +++ | 2.713 | 2.492 | 2.955 |
|  |  | **Deviance** | **(DF)** | **Drop Dev** | **P** |  |  |
| **Omitting Unexposed group** |  | 403.108 | (99) | -0.780 | N.S. |  |  |
|  |  | **Estimate** | **S.E.** | **P** | **RR** | **95%CIl** | **95%CIu** |
| **Constant** |  | 1.311 | 0.266 | +++ | 3.709 | 2.202 | 6.246 |
| **RR adjusted for factor other than sex, age** |  |  |  |  |  |  |  |
| Yes | 30 | Aliased |  |  | 2.564 | 2.183 | 3.012 |
| No | 84 | -0.107 | 0.069 | N.S. | 2.303 | 2.020 | 2.627 |
| **Sex(RR)** |  |  |  |  |  |  |  |
| Male | 51 | Aliased |  |  | 2.650 | 2.282 | 3.077 |
| Female | 39 | -0.170 | 0.036 | --- | 2.236 | 1.936 | 2.583 |
| Combined | 24 | -0.113 | 0.076 | N.S. | 2.366 | 1.982 | 2.825 |
| **Continent** |  |  |  |  |  |  |  |
| NAmer | 38 | Aliased |  |  | 2.996 | 2.629 | 3.414 |
| Europe | 60 | -0.279 | 0.039 | --- | 2.266 | 2.121 | 2.421 |
| Asia | 9 | -0.281 | 0.060 | --- | 2.263 | 1.897 | 2.701 |
| oth/mult | 7 | -0.153 | 0.068 | - | 2.572 | 2.023 | 3.270 |

Table 2 - A - 6

IESCOPD - Meta-regression of ever smoking, any product (or cigarettes if all product not available)

Multiple regression of data from Table 2 - A - 1 (most-adjusted RRs)

Any CB

Test by removing variables one at a time

|  |  | **Estimate** | **S.E.** | **P** | **RR** | **95%CIl** | **95%CIu** |
| --- | --- | --- | --- | --- | --- | --- | --- |
| **Asthma analysis type (CB)** |  |  |  |  |  |  |  |
| inc-irresp | 103 | Aliased |  |  | 2.421 | 2.297 | 2.551 |
| excl-all | 7 | -0.113 | 0.065 | (-) | 2.162 | 1.684 | 2.777 |
| defn-incl | 0 | Aliased |  |  | 2.421 | 2.297 | 2.551 |
| other | 4 | 0.996 | 0.214 | +++ | 6.555 | 2.818 | 15.250 |
| **Smoking product (3)** |  |  |  |  |  |  |  |
| any | 61 | Aliased |  |  | 2.583 | 2.407 | 2.772 |
| cigs | 51 | -0.170 | 0.030 | --- | 2.179 | 2.001 | 2.373 |
| cigsonly | 2 | 0.450 | 0.149 | ++ | 4.053 | 2.252 | 7.295 |
| **CB subtype** |  |  |  |  |  |  |  |
| mort | 3 | Aliased |  |  | 3.054 | 1.148 | 8.123 |
| sympt | 83 | -0.215 | 0.248 | N.S. | 2.463 | 2.317 | 2.617 |
| other | 28 | -0.303 | 0.248 | N.S. | 2.257 | 1.991 | 2.557 |
| **Midpoint age in RR** |  | 0.003 | 0.001 | + | 2.090 | 1.643 | 2.658 |
| **RR adjusted for age** |  |  |  |  |  |  |  |
| Yes | 63 | Aliased |  |  | 2.195 | 2.040 | 2.363 |
| No | 51 | 0.220 | 0.031 | +++ | 2.735 | 2.510 | 2.981 |
|  |  | **Deviance** | **(DF)** | **Drop Dev** | **P** |  |  |
| **Omitting CB subtype** |  | 409.146 | (100) | -6.818 | N.S. |  |  |
|  |  | **Estimate** | **S.E.** | **P** | **RR** | **95%CIl** | **95%CIu** |
| **Constant** |  | 1.122 | 0.095 | +++ | 3.070 | 2.549 | 3.697 |
| **RR adjusted for factor other than sex, age** |  |  |  |  |  |  |  |
| Yes | 30 | Aliased |  |  | 2.577 | 2.194 | 3.026 |
| No | 84 | -0.116 | 0.070 | (-) | 2.294 | 2.012 | 2.616 |
| **Sex(RR)** |  |  |  |  |  |  |  |
| Male | 51 | Aliased |  |  | 2.679 | 2.308 | 3.109 |
| Female | 39 | -0.172 | 0.036 | --- | 2.255 | 1.955 | 2.602 |
| Combined | 24 | -0.138 | 0.076 | (-) | 2.334 | 1.957 | 2.783 |
| **Continent** |  |  |  |  |  |  |  |
| NAmer | 38 | Aliased |  |  | 2.977 | 2.618 | 3.386 |
| Europe | 60 | -0.271 | 0.038 | --- | 2.271 | 2.126 | 2.426 |
| Asia | 9 | -0.274 | 0.059 | --- | 2.263 | 1.899 | 2.698 |
| oth/mult | 7 | -0.149 | 0.073 | - | 2.565 | 1.979 | 3.323 |
| **Asthma analysis type (CB)** |  |  |  |  |  |  |  |
| inc-irresp | 103 | Aliased |  |  | 2.427 | 2.304 | 2.556 |
| excl-all | 7 | -0.156 | 0.063 | - | 2.076 | 1.635 | 2.637 |
| defn-incl | 0 | Aliased |  |  | 2.427 | 2.304 | 2.556 |
| other | 4 | 0.932 | 0.213 | +++ | 6.163 | 2.676 | 14.191 |
| **Smoking product (3)** |  |  |  |  |  |  |  |
| any | 61 | Aliased |  |  | 2.487 | 2.006 | 3.084 |
| cigs | 51 | -0.078 | 0.128 | N.S. | 2.300 | 1.707 | 3.099 |
| cigsonly | 2 | 0.403 | 0.146 | ++ | 3.721 | 2.018 | 6.862 |
| **Unexposed group** |  |  |  |  |  |  |  |
| nev any | 70 | Aliased |  |  | 2.502 | 2.038 | 3.072 |
| nev cig | 44 | -0.088 | 0.126 | N.S. | 2.292 | 1.699 | 3.093 |
| **Midpoint age in RR** |  | 0.002 | 0.001 | (+) | 2.176 | 1.733 | 2.731 |
| **RR adjusted for age** |  |  |  |  |  |  |  |
| Yes | 63 | Aliased |  |  | 2.185 | 2.031 | 2.350 |
| No | 51 | 0.231 | 0.031 | +++ | 2.753 | 2.528 | 2.997 |
|  |  | **Deviance** | **(DF)** | **Drop Dev** | **P** |  |  |
| **Omitting Midpoint age in RR** |  | 408.241 | (99) | -5.912 | N.S. |  |  |
|  |  | **Estimate** | **S.E.** | **P** | **RR** | **95%CIl** | **95%CIu** |
| **Constant** |  | 1.509 | 0.254 | +++ | 4.521 | 2.748 | 7.438 |
| **RR adjusted for factor other than sex, age** |  |  |  |  |  |  |  |
| Yes | 30 | Aliased |  |  | 2.599 | 2.213 | 3.052 |
| No | 84 | -0.131 | 0.069 | (-) | 2.279 | 1.998 | 2.599 |

Table 2 - A - 6

IESCOPD - Meta-regression of ever smoking, any product (or cigarettes if all product not available)

Multiple regression of data from Table 2 - A - 1 (most-adjusted RRs)

Any CB

Test by removing variables one at a time

|  |  | **Estimate** | **S.E.** | **P** | **RR** | **95%CIl** | **95%CIu** |
| --- | --- | --- | --- | --- | --- | --- | --- |
| **Sex(RR)** |  |  |  |  |  |  |  |
| Male | 51 | Aliased |  |  | 2.690 | 2.318 | 3.122 |
| Female | 39 | -0.157 | 0.035 | --- | 2.299 | 2.001 | 2.641 |
| Combined | 24 | -0.158 | 0.075 | - | 2.297 | 1.932 | 2.731 |
| **Continent** |  |  |  |  |  |  |  |
| NAmer | 38 | Aliased |  |  | 2.910 | 2.577 | 3.287 |
| Europe | 60 | -0.244 | 0.036 | --- | 2.281 | 2.135 | 2.437 |
| Asia | 9 | -0.212 | 0.053 | --- | 2.355 | 1.999 | 2.776 |
| oth/mult | 7 | -0.172 | 0.074 | - | 2.450 | 1.890 | 3.176 |
| **Asthma analysis type (CB)** |  |  |  |  |  |  |  |
| inc-irresp | 103 | Aliased |  |  | 2.422 | 2.298 | 2.552 |
| excl-all | 7 | -0.119 | 0.065 | (-) | 2.150 | 1.674 | 2.761 |
| defn-incl | 0 | Aliased |  |  | 2.422 | 2.298 | 2.552 |
| other | 4 | 0.963 | 0.214 | +++ | 6.342 | 2.731 | 14.730 |
| **Smoking product (3)** |  |  |  |  |  |  |  |
| any | 61 | Aliased |  |  | 2.458 | 1.979 | 3.052 |
| cigs | 51 | -0.050 | 0.128 | N.S. | 2.338 | 1.732 | 3.156 |
| cigsonly | 2 | 0.418 | 0.149 | ++ | 3.735 | 2.006 | 6.951 |
| **Unexposed group** |  |  |  |  |  |  |  |
| nev any | 70 | Aliased |  |  | 2.526 | 2.054 | 3.107 |
| nev cig | 44 | -0.112 | 0.126 | N.S. | 2.260 | 1.671 | 3.057 |
| **CB subtype** |  |  |  |  |  |  |  |
| mort | 3 | Aliased |  |  | 3.197 | 1.206 | 8.477 |
| sympt | 83 | -0.267 | 0.247 | N.S. | 2.448 | 2.305 | 2.599 |
| other | 28 | -0.329 | 0.248 | N.S. | 2.302 | 2.041 | 2.596 |
| **RR adjusted for age** |  |  |  |  |  |  |  |
| Yes | 63 | Aliased |  |  | 2.216 | 2.059 | 2.385 |
| No | 51 | 0.198 | 0.031 | +++ | 2.702 | 2.479 | 2.945 |
|  |  | **Deviance** | **(DF)** | **Drop Dev** | **P** |  |  |
| **Omitting RR adjusted for age** |  | 447.378 | (99) | -45.049 | ** |  |  |
|  |  | **Estimate** | **S.E.** | **P** | **RR** | **95%CIl** | **95%CIu** |
| **Constant** |  | 1.442 | 0.265 | +++ | 4.229 | 2.514 | 7.115 |
| **RR adjusted for factor other than sex, age** |  |  |  |  |  |  |  |
| Yes | 30 | Aliased |  |  | 2.606 | 2.218 | 3.062 |
| No | 84 | -0.136 | 0.070 | (-) | 2.274 | 1.993 | 2.594 |
| **Sex(RR)** |  |  |  |  |  |  |  |
| Male | 51 | Aliased |  |  | 2.789 | 2.406 | 3.232 |
| Female | 39 | -0.199 | 0.036 | --- | 2.286 | 1.979 | 2.640 |
| Combined | 24 | -0.218 | 0.075 | -- | 2.242 | 1.881 | 2.673 |
| **Continent** |  |  |  |  |  |  |  |
| NAmer | 38 | Aliased |  |  | 2.959 | 2.597 | 3.372 |
| Europe | 60 | -0.247 | 0.038 | --- | 2.312 | 2.165 | 2.469 |
| Asia | 9 | -0.309 | 0.060 | --- | 2.173 | 1.824 | 2.589 |
| oth/mult | 7 | -0.255 | 0.073 | --- | 2.294 | 1.774 | 2.967 |
| **Asthma analysis type (CB)** |  |  |  |  |  |  |  |
| inc-irresp | 103 | Aliased |  |  | 2.421 | 2.298 | 2.551 |
| excl-all | 7 | -0.112 | 0.066 | (-) | 2.164 | 1.684 | 2.780 |
| defn-incl | 0 | Aliased |  |  | 2.421 | 2.298 | 2.551 |
| other | 4 | 0.943 | 0.214 | +++ | 6.219 | 2.675 | 14.460 |
| **Smoking product (3)** |  |  |  |  |  |  |  |
| any | 61 | Aliased |  |  | 2.273 | 1.840 | 2.807 |
| cigs | 51 | 0.139 | 0.125 | N.S. | 2.611 | 1.950 | 3.497 |
| cigsonly | 2 | 0.398 | 0.149 | ++ | 3.382 | 1.822 | 6.281 |
| **Unexposed group** |  |  |  |  |  |  |  |
| nev any | 70 | Aliased |  |  | 2.713 | 2.216 | 3.323 |
| nev cig | 44 | -0.289 | 0.123 | - | 2.032 | 1.512 | 2.731 |
| **CB subtype** |  |  |  |  |  |  |  |
| mort | 3 | Aliased |  |  | 2.788 | 1.050 | 7.406 |
| sympt | 83 | -0.109 | 0.248 | N.S. | 2.500 | 2.353 | 2.655 |
| other | 28 | -0.261 | 0.248 | N.S. | 2.148 | 1.901 | 2.428 |
| **Midpoint age in RR** |  | 0.001 | 0.001 | N.S. | 2.268 | 1.792 | 2.871 |

Table 2 - A - 6

IESCOPD - Meta-regression of ever smoking, any product (or cigarettes if all product not available)

Multiple regression of data from Table 2 - A - 1 (most-adjusted RRs)

Any CB

Test by removing variables one at a time

|  |  | **Deviance** | **(DF)** | **Drop Dev** | **P** |  |  |
| --- | --- | --- | --- | --- | --- | --- | --- |
| **Omitting adjusted for factor other than sex, age** |  | 404.942 | (99) | -2.614 | N.S. |  |  |
|  |  | **Estimate** | **S.E.** | **P** | **RR** | **95%CIl** | **95%CIu** |
| **Constant** |  | 1.193 | 0.255 | +++ | 3.296 | 2.000 | 5.431 |
| **RR adjusted for age** |  |  |  |  |  |  |  |
| Yes | 63 | Aliased |  |  | 2.199 | 2.041 | 2.368 |
| No | 51 | 0.217 | 0.032 | +++ | 2.730 | 2.502 | 2.979 |
| **Sex(RR)** |  |  |  |  |  |  |  |
| Male | 51 | Aliased |  |  | 2.539 | 2.299 | 2.805 |
| Female | 39 | -0.166 | 0.036 | --- | 2.151 | 1.939 | 2.387 |
| Combined | 24 | -0.012 | 0.037 | N.S. | 2.509 | 2.291 | 2.748 |
| **Continent** |  |  |  |  |  |  |  |
| NAmer | 38 | Aliased |  |  | 3.012 | 2.644 | 3.430 |
| Europe | 60 | -0.286 | 0.038 | --- | 2.262 | 2.118 | 2.416 |
| Asia | 9 | -0.284 | 0.060 | --- | 2.266 | 1.899 | 2.704 |
| oth/mult | 7 | -0.161 | 0.073 | - | 2.564 | 1.982 | 3.318 |
| **Asthma analysis type (CB)** |  |  |  |  |  |  |  |
| inc-irresp | 103 | Aliased |  |  | 2.421 | 2.298 | 2.551 |
| excl-all | 7 | -0.118 | 0.065 | (-) | 2.152 | 1.676 | 2.762 |
| defn-incl | 0 | Aliased |  |  | 2.421 | 2.298 | 2.551 |
| other | 4 | 0.985 | 0.214 | +++ | 6.486 | 2.789 | 15.083 |
| **Smoking product (3)** |  |  |  |  |  |  |  |
| any | 61 | Aliased |  |  | 2.486 | 2.003 | 3.085 |
| cigs | 51 | -0.078 | 0.128 | N.S. | 2.300 | 1.706 | 3.102 |
| cigsonly | 2 | 0.445 | 0.149 | ++ | 3.880 | 2.079 | 7.240 |
| **Unexposed group** |  |  |  |  |  |  |  |
| nev any | 70 | Aliased |  |  | 2.507 | 2.040 | 3.082 |
| nev cig | 44 | -0.093 | 0.126 | N.S. | 2.285 | 1.691 | 3.087 |
| **CB subtype** |  |  |  |  |  |  |  |
| mort | 3 | Aliased |  |  | 3.022 | 1.136 | 8.036 |
| sympt | 83 | -0.204 | 0.248 | N.S. | 2.465 | 2.319 | 2.620 |
| other | 28 | -0.296 | 0.248 | N.S. | 2.249 | 1.984 | 2.549 |
| **Midpoint age in RR** |  | 0.003 | 0.001 | + | 2.068 | 1.628 | 2.626 |

Table 2 - A - 6

IESCOPD - Meta-regression of ever smoking, any product (or cigarettes if all product not available)

Multiple regression of data from Table 2 - A - 1 (most-adjusted RRs)

Any CB

Test reduction to 2-level product

|  |  | **Deviance** | **(DF)** | **Drop Dev** | **P** |  |  |
| --- | --- | --- | --- | --- | --- | --- | --- |
| **Reducing smoking product to 2 levels** |  | 409.019 | (99) | -6.691 | N.S. |  |  |
|  |  | **Estimate** | **S.E.** | **P** | **RR** | **95%CIl** | **95%CIu** |
| **Constant** |  | 1.388 | 0.265 | +++ | 4.006 | 2.385 | 6.729 |
| **Sex(RR)** |  |  |  |  |  |  |  |
| Male | 51 | Aliased |  |  | 2.677 | 2.305 | 3.110 |
| Female | 39 | -0.166 | 0.036 | --- | 2.268 | 1.965 | 2.618 |
| Combined | 24 | -0.141 | 0.076 | (-) | 2.326 | 1.949 | 2.776 |
| **Continent** |  |  |  |  |  |  |  |
| NAmer | 38 | Aliased |  |  | 2.980 | 2.616 | 3.395 |
| Europe | 60 | -0.266 | 0.038 | --- | 2.284 | 2.139 | 2.440 |
| Asia | 9 | -0.279 | 0.060 | --- | 2.254 | 1.889 | 2.689 |
| oth/mult | 7 | -0.218 | 0.072 | -- | 2.397 | 1.861 | 3.086 |
| **Asthma analysis type (CB)** |  |  |  |  |  |  |  |
| inc-irresp | 103 | Aliased |  |  | 2.422 | 2.298 | 2.552 |
| excl-all | 7 | -0.120 | 0.065 | (-) | 2.147 | 1.672 | 2.758 |
| defn-incl | 0 | Aliased |  |  | 2.422 | 2.298 | 2.552 |
| other | 4 | 0.977 | 0.214 | +++ | 6.431 | 2.766 | 14.954 |
| **Smoking product (2)** |  |  |  |  |  |  |  |
| any | 61 | Aliased |  |  | 2.260 | 1.906 | 2.680 |
| cigs | 53 | 0.157 | 0.097 | N.S. | 2.643 | 2.107 | 3.316 |
| **Unexposed group** |  |  |  |  |  |  |  |
| nev any | 70 | Aliased |  |  | 2.747 | 2.335 | 3.232 |
| nev cig | 44 | -0.320 | 0.097 | -- | 1.995 | 1.578 | 2.523 |
| **CB subtype** |  |  |  |  |  |  |  |
| mort | 3 | Aliased |  |  | 3.199 | 1.206 | 8.485 |
| sympt | 83 | -0.263 | 0.248 | N.S. | 2.459 | 2.313 | 2.613 |
| other | 28 | -0.344 | 0.248 | N.S. | 2.267 | 2.001 | 2.569 |
| **Midpoint age in RR** |  | 0.003 | 0.001 | + | 2.118 | 1.667 | 2.691 |
| **RR adjusted for age** |  |  |  |  |  |  |  |
| Yes | 63 | Aliased |  |  | 2.216 | 2.059 | 2.385 |
| No | 51 | 0.198 | 0.031 | +++ | 2.702 | 2.479 | 2.946 |
| **RR adjusted for factor other than sex, age** |  |  |  |  |  |  |  |
| Yes | 30 | Aliased |  |  | 2.583 | 2.198 | 3.036 |
| No | 84 | -0.120 | 0.070 | (-) | 2.290 | 2.007 | 2.613 |

Table 2 - A - 6

IESCOPD - Meta-regression of ever smoking, any product (or cigarettes if all product not available)

Multiple regression of data from Table 2 - A - 1 (most-adjusted RRs)

Any CB

Test reduction to 2-level outcome subtype

|  |  | **Deviance** | **(DF)** | **Drop Dev** | **P** |  |  |
| --- | --- | --- | --- | --- | --- | --- | --- |
| **Reducing CB subtype to 2 levels** |  | 403.810 | (99) | -1.482 | N.S. |  |  |
|  |  | **Estimate** | **S.E.** | **P** | **RR** | **95%CIl** | **95%CIu** |
| **Constant** |  | 1.105 | 0.095 | +++ | 3.021 | 2.507 | 3.639 |
| **Sex(RR)** |  |  |  |  |  |  |  |
| Male | 51 | Aliased |  |  | 2.662 | 2.291 | 3.093 |
| Female | 39 | -0.174 | 0.036 | --- | 2.237 | 1.936 | 2.584 |
| Combined | 24 | -0.121 | 0.077 | N.S. | 2.358 | 1.974 | 2.817 |
| **Continent** |  |  |  |  |  |  |  |
| NAmer | 38 | Aliased |  |  | 3.007 | 2.639 | 3.425 |
| Europe | 60 | -0.282 | 0.039 | --- | 2.268 | 2.123 | 2.424 |
| Asia | 9 | -0.287 | 0.060 | --- | 2.257 | 1.892 | 2.693 |
| oth/mult | 7 | -0.179 | 0.074 | - | 2.515 | 1.934 | 3.270 |
| **Asthma analysis type (CB)** |  |  |  |  |  |  |  |
| inc-irresp | 103 | Aliased |  |  | 2.421 | 2.297 | 2.551 |
| excl-all | 7 | -0.112 | 0.066 | (-) | 2.164 | 1.684 | 2.779 |
| defn-incl | 0 | Aliased |  |  | 2.421 | 2.297 | 2.551 |
| other | 4 | 0.993 | 0.214 | +++ | 6.532 | 2.808 | 15.196 |
| **Smoking product (3)** |  |  |  |  |  |  |  |
| any | 61 | Aliased |  |  | 2.468 | 1.987 | 3.065 |
| cigs | 51 | -0.060 | 0.128 | N.S. | 2.323 | 1.721 | 3.136 |
| cigsonly | 2 | 0.465 | 0.149 | ++ | 3.927 | 2.109 | 7.314 |
| **Unexposed group** |  |  |  |  |  |  |  |
| nev any | 70 | Aliased |  |  | 2.527 | 2.054 | 3.108 |
| nev cig | 44 | -0.112 | 0.126 | N.S. | 2.259 | 1.670 | 3.056 |
| **CB subtype (2)** |  |  |  |  |  |  |  |
| sympt | 83 | Aliased |  |  | 2.464 | 2.319 | 2.619 |
| other | 31 | -0.087 | 0.038 | - | 2.259 | 1.994 | 2.560 |
| **Midpoint age in RR** |  | 0.003 | 0.001 | + | 2.083 | 1.639 | 2.649 |
| **RR adjusted for age** |  |  |  |  |  |  |  |
| Yes | 63 | Aliased |  |  | 2.202 | 2.044 | 2.372 |
| No | 51 | 0.213 | 0.032 | +++ | 2.725 | 2.497 | 2.973 |
| **RR adjusted for factor other than sex, age** |  |  |  |  |  |  |  |
| Yes | 30 | Aliased |  |  | 2.570 | 2.187 | 3.021 |
| No | 84 | -0.111 | 0.070 | N.S. | 2.299 | 2.015 | 2.624 |

Table 2 - A - 6

IESCOPD - Meta-regression of ever smoking, any product (or cigarettes if all product not available)

Multiple regression of data from Table 2 - A - 1 (most-adjusted RRs)

Any CB

Test by adding extra variables one at a time

|  |  | **Deviance** | **(DF)** | **Drop Dev** | **P** |  |  |
| --- | --- | --- | --- | --- | --- | --- | --- |
| **Increasing CB subtype to 4 levels** |  | 400.422 | (97) | 1.907 | N.S. |  |  |
|  |  | **Estimate** | **S.E.** | **P** | **RR** | **95%CIl** | **95%CIu** |
| **Constant** |  | 1.307 | 0.266 | +++ | 3.694 | 2.192 | 6.223 |
| **Sex(RR)** |  |  |  |  |  |  |  |
| Male | 51 | Aliased |  |  | 2.654 | 2.281 | 3.088 |
| Female | 39 | -0.172 | 0.036 | --- | 2.234 | 1.931 | 2.585 |
| Combined | 24 | -0.115 | 0.077 | N.S. | 2.365 | 1.976 | 2.831 |
| **Continent** |  |  |  |  |  |  |  |
| NAmer | 38 | Aliased |  |  | 2.979 | 2.608 | 3.402 |
| Europe | 60 | -0.271 | 0.039 | --- | 2.271 | 2.124 | 2.429 |
| Asia | 9 | -0.270 | 0.060 | --- | 2.275 | 1.902 | 2.720 |
| oth/mult | 7 | -0.163 | 0.074 | - | 2.530 | 1.940 | 3.300 |
| **Asthma analysis type (CB)** |  |  |  |  |  |  |  |
| inc-irresp | 103 | Aliased |  |  | 2.433 | 2.303 | 2.571 |
| excl-all | 7 | -0.215 | 0.101 | - | 1.963 | 1.338 | 2.880 |
| defn-incl | 0 | Aliased |  |  | 2.433 | 2.303 | 2.571 |
| other | 4 | 1.011 | 0.215 | +++ | 6.687 | 2.850 | 15.694 |
| **Smoking product (3)** |  |  |  |  |  |  |  |
| any | 61 | Aliased |  |  | 2.483 | 1.994 | 3.091 |
| cigs | 51 | -0.075 | 0.129 | N.S. | 2.303 | 1.700 | 3.120 |
| cigsonly | 2 | 0.466 | 0.150 | ++ | 3.957 | 2.103 | 7.445 |
| **Unexposed group** |  |  |  |  |  |  |  |
| nev any | 70 | Aliased |  |  | 2.515 | 2.041 | 3.100 |
| nev cig | 44 | -0.100 | 0.126 | N.S. | 2.275 | 1.677 | 3.086 |
| **CB subtype** |  |  |  |  |  |  |  |
| mort | 3 | Aliased |  |  |  |  |  |
| sympt | 83 | Aliased |  |  |  |  |  |
| other | 28 | Aliased |  |  |  |  |  |
|  |  | **Estimate** | **S.E.** | **P** | **RR** | **95%CIl** | **95%CIu** |
| **Midpoint age in RR** |  | 0.003 | 0.001 | + | 2.076 | 1.628 | 2.647 |
| **RR adjusted for age** |  |  |  |  |  |  |  |
| Yes | 63 | Aliased |  |  | 2.192 | 2.032 | 2.365 |
| No | 51 | 0.223 | 0.033 | +++ | 2.741 | 2.507 | 2.997 |
| **RR adjusted for factor other than sex, age** |  |  |  |  |  |  |  |
| Yes | 30 | Aliased |  |  | 2.576 | 2.188 | 3.031 |
| No | 84 | -0.115 | 0.070 | N.S. | 2.295 | 2.009 | 2.622 |
| **CB subtype (4)** |  |  |  |  |  |  |  |
| mort | 3 | Aliased |  |  | 3.048 | 1.137 | 8.170 |
| sympt | 83 | -0.216 | 0.249 | N.S. | 2.455 | 2.307 | 2.613 |
| oth-prev | 24 | -0.320 | 0.249 | N.S. | 2.214 | 1.933 | 2.535 |
| oth-inc | 4 | -0.147 | 0.272 | N.S. | 2.631 | 1.650 | 4.196 |

Table 2 - A - 6

IESCOPD - Meta-regression of ever smoking, any product (or cigarettes if all product not available)

Multiple regression of data from Table 2 - A - 1 (most-adjusted RRs)

Any CB

Test by adding extra variables one at a time

|  |  | **Deviance** | **(DF)** | **Drop Dev** | **P** |  |  |
| --- | --- | --- | --- | --- | --- | --- | --- |
| **Adding National cigarette tobacco type** |  | 395.377 | (96) | 6.952 | N.S. |  |  |
|  |  | **Estimate** | **S.E.** | **P** | **RR** | **95%CIl** | **95%CIu** |
| **Constant** |  | 1.303 | 0.266 | +++ | 3.680 | 2.183 | 6.204 |
| **Sex(RR)** |  |  |  |  |  |  |  |
| Male | 51 | Aliased |  |  | 2.652 | 2.279 | 3.087 |
| Female | 39 | -0.173 | 0.036 | --- | 2.231 | 1.926 | 2.584 |
| Combined | 24 | -0.113 | 0.077 | N.S. | 2.368 | 1.977 | 2.838 |
| **Continent** |  |  |  |  |  |  |  |
| NAmer | 38 | Aliased |  |  | 3.046 | 2.664 | 3.484 |
| Europe | 60 | -0.285 | 0.039 | --- | 2.290 | 2.139 | 2.452 |
| Asia | 9 | -0.346 | 0.065 | --- | 2.154 | 1.775 | 2.615 |
| oth/mult | 7 | -0.263 | 0.081 | -- | 2.341 | 1.757 | 3.120 |
| **Asthma analysis type (CB)** |  |  |  |  |  |  |  |
| inc-irresp | 103 | Aliased |  |  | 2.421 | 2.297 | 2.552 |
| excl-all | 7 | -0.116 | 0.066 | (-) | 2.156 | 1.675 | 2.775 |
| defn-incl | 0 | Aliased |  |  | 2.421 | 2.297 | 2.552 |
| other | 4 | 0.988 | 0.214 | +++ | 6.500 | 2.777 | 15.214 |
| **Smoking product (3)** |  |  |  |  |  |  |  |
| any | 61 | Aliased |  |  | 2.546 | 1.953 | 3.319 |
| cigs | 51 | -0.133 | 0.157 | N.S. | 2.229 | 1.542 | 3.222 |
| cigsonly | 2 | 0.332 | 0.156 | + | 3.549 | 1.825 | 6.902 |
| **Unexposed group** |  |  |  |  |  |  |  |
| nev any | 70 | Aliased |  |  | 2.454 | 1.905 | 3.160 |
| nev cig | 44 | -0.039 | 0.155 | N.S. | 2.360 | 1.628 | 3.420 |
| **CB subtype** |  |  |  |  |  |  |  |
| mort | 3 | Aliased |  |  | 3.023 | 1.129 | 8.095 |
| sympt | 83 | -0.212 | 0.249 | N.S. | 2.446 | 2.298 | 2.604 |
| other | 28 | -0.270 | 0.249 | N.S. | 2.309 | 2.023 | 2.634 |
| **Midpoint age in RR** |  | 0.003 | 0.001 | + | 2.108 | 1.653 | 2.688 |
| **RR adjusted for age** |  |  |  |  |  |  |  |
| Yes | 63 | Aliased |  |  | 2.191 | 2.033 | 2.363 |
| No | 51 | 0.224 | 0.032 | +++ | 2.742 | 2.510 | 2.995 |
| **RR adjusted for factor other than sex, age** |  |  |  |  |  |  |  |
| Yes | 30 | Aliased |  |  | 2.560 | 2.174 | 3.013 |
| No | 84 | -0.104 | 0.070 | N.S. | 2.307 | 2.019 | 2.635 |
| **National cigarette tobacco type** |  |  |  |  |  |  |  |
| bl | 78 | Aliased |  |  | 2.363 | 2.225 | 2.511 |
| vir | 34 | 0.110 | 0.045 | + | 2.639 | 2.257 | 3.086 |
| m/u | 2 | 0.175 | 0.117 | N.S. | 2.815 | 1.795 | 4.416 |

Table 2 - A - 6

IESCOPD - Meta-regression of ever smoking, any product (or cigarettes if all product not available)

Multiple regression of data from Table 2 - A - 1 (most-adjusted RRs)

Any CB

Test by adding extra variables one at a time

|  |  | **Deviance** | **(DF)** | **Drop Dev** | **P** |  |  |
| --- | --- | --- | --- | --- | --- | --- | --- |
| **Adding Publication year** |  | 352.351 | (95) | 49.977 | ** |  |  |
|  |  | **Estimate** | **S.E.** | **P** | **RR** | **95%CIl** | **95%CIu** |
| **Constant** |  | 1.331 | 0.267 | +++ | 3.784 | 2.244 | 6.381 |
| **Sex(RR)** |  |  |  |  |  |  |  |
| Male | 51 | Aliased |  |  | 2.549 | 2.204 | 2.949 |
| Female | 39 | -0.142 | 0.036 | --- | 2.211 | 1.924 | 2.541 |
| Combined | 24 | -0.037 | 0.078 | N.S. | 2.456 | 2.068 | 2.918 |
| **Continent** |  |  |  |  |  |  |  |
| NAmer | 38 | Aliased |  |  | 2.764 | 2.395 | 3.189 |
| Europe | 60 | -0.178 | 0.045 | --- | 2.313 | 2.167 | 2.468 |
| Asia | 9 | -0.185 | 0.066 | -- | 2.298 | 1.934 | 2.730 |
| oth/mult | 7 | -0.034 | 0.080 | N.S. | 2.672 | 2.070 | 3.449 |
| **Asthma analysis type (CB)** |  |  |  |  |  |  |  |
| inc-irresp | 103 | Aliased |  |  | 2.394 | 2.277 | 2.518 |
| excl-all | 7 | 0.087 | 0.072 | N.S. | 2.612 | 2.014 | 3.388 |
| defn-incl | 0 | Aliased |  |  | 2.394 | 2.277 | 2.518 |
| other | 4 | 1.186 | 0.217 | +++ | 7.839 | 3.465 | 17.736 |
| **Smoking product (3)** |  |  |  |  |  |  |  |
| any | 61 | Aliased |  |  | 2.494 | 2.025 | 3.070 |
| cigs | 51 | -0.085 | 0.129 | N.S. | 2.290 | 1.717 | 3.056 |
| cigsonly | 2 | 0.427 | 0.150 | ++ | 3.823 | 2.103 | 6.947 |
| **Unexposed group** |  |  |  |  |  |  |  |
| nev any | 70 | Aliased |  |  | 2.522 | 2.069 | 3.074 |
| nev cig | 44 | -0.107 | 0.126 | N.S. | 2.266 | 1.697 | 3.025 |
| **CB subtype** |  |  |  |  |  |  |  |
| mort | 3 | Aliased |  |  | 3.053 | 1.197 | 7.784 |
| sympt | 83 | -0.207 | 0.249 | N.S. | 2.481 | 2.333 | 2.640 |
| other | 28 | -0.328 | 0.250 | N.S. | 2.199 | 1.914 | 2.527 |
| **Midpoint age in RR** |  | 0.003 | 0.001 | + | 2.065 | 1.632 | 2.613 |
| **RR adjusted for age** |  |  |  |  |  |  |  |
| Yes | 63 | Aliased |  |  | 2.191 | 2.038 | 2.356 |
| No | 51 | 0.224 | 0.033 | +++ | 2.742 | 2.518 | 2.986 |
| **RR adjusted for factor other than sex, age** |  |  |  |  |  |  |  |
| Yes | 30 | Aliased |  |  | 2.557 | 2.190 | 2.985 |
| No | 84 | -0.102 | 0.070 | N.S. | 2.309 | 2.034 | 2.620 |
| **Publication year** |  |  |  |  |  |  |  |
| <1980 | 49 | Aliased |  |  | 2.835 | 2.471 | 3.253 |
| 1980-89 | 28 | -0.120 | 0.052 | - | 2.516 | 2.242 | 2.823 |
| 1990-99 | 17 | -0.330 | 0.050 | --- | 2.038 | 1.794 | 2.314 |
| 2000+ | 20 | -0.228 | 0.051 | --- | 2.258 | 2.024 | 2.519 |
|  |  | **Deviance** | **(DF)** | **Drop Dev** | **P** |  |  |
| **Adding Study type** |  | 389.435 | (96) | 12.894 | N.S. |  |  |
|  |  | **Estimate** | **S.E.** | **P** | **RR** | **95%CIl** | **95%CIu** |
| **Constant** |  | 0.943 | 0.295 | ++ | 2.568 | 1.441 | 4.578 |
| **Sex(RR)** |  |  |  |  |  |  |  |
| Male | 51 | Aliased |  |  | 2.651 | 2.282 | 3.081 |
| Female | 39 | -0.177 | 0.036 | --- | 2.221 | 1.922 | 2.567 |
| Combined | 24 | -0.110 | 0.077 | N.S. | 2.376 | 1.988 | 2.839 |
| **Continent** |  |  |  |  |  |  |  |
| NAmer | 38 | Aliased |  |  | 2.977 | 2.611 | 3.395 |
| Europe | 60 | -0.273 | 0.039 | --- | 2.267 | 2.120 | 2.423 |
| Asia | 9 | -0.260 | 0.060 | --- | 2.296 | 1.922 | 2.743 |
| oth/mult | 7 | -0.154 | 0.074 | - | 2.552 | 1.962 | 3.320 |
| **Asthma analysis type (CB)** |  |  |  |  |  |  |  |
| inc-irresp | 103 | Aliased |  |  | 2.428 | 2.299 | 2.563 |
| excl-all | 7 | -0.181 | 0.094 | (-) | 2.025 | 1.423 | 2.883 |
| defn-incl | 0 | Aliased |  |  | 2.428 | 2.299 | 2.563 |
| other | 4 | 1.164 | 0.241 | +++ | 7.772 | 3.026 | 19.960 |

Table 2 - A - 6

IESCOPD - Meta-regression of ever smoking, any product (or cigarettes if all product not available)

Multiple regression of data from Table 2 - A - 1 (most-adjusted RRs)

Any CB

Test by adding extra variables one at a time

|  |  | **Estimate** | **S.E.** | **P** | **RR** | **95%CIl** | **95%CIu** |
| --- | --- | --- | --- | --- | --- | --- | --- |
| **Smoking product (3)** |  |  |  |  |  |  |  |
| any | 61 | Aliased |  |  | 2.511 | 2.021 | 3.121 |
| cigs | 51 | -0.106 | 0.129 | N.S. | 2.260 | 1.672 | 3.055 |
| cigsonly | 2 | 0.614 | 0.182 | ++ | 4.639 | 2.189 | 9.830 |
| **Unexposed group** |  |  |  |  |  |  |  |
| nev any | 70 | Aliased |  |  | 2.505 | 2.037 | 3.082 |
| nev cig | 44 | -0.091 | 0.126 | N.S. | 2.288 | 1.691 | 3.095 |
| **CB subtype** |  |  |  |  |  |  |  |
| mort | 3 | Aliased |  |  | 2.427 | 0.873 | 6.747 |
| sympt | 83 | 0.021 | 0.260 | N.S. | 2.478 | 2.331 | 2.634 |
| other | 28 | -0.091 | 0.259 | N.S. | 2.216 | 1.953 | 2.516 |
| **Midpoint age in RR** |  | 0.003 | 0.001 | + | 2.084 | 1.634 | 2.657 |
| **RR adjusted for age** |  |  |  |  |  |  |  |
| Yes | 63 | Aliased |  |  | 2.191 | 2.032 | 2.361 |
| No | 51 | 0.225 | 0.032 | +++ | 2.743 | 2.512 | 2.997 |
| **RR adjusted for factor other than sex, age** |  |  |  |  |  |  |  |
| Yes | 30 | Aliased |  |  | 2.591 | 2.204 | 3.046 |
| No | 84 | -0.126 | 0.070 | (-) | 2.284 | 2.001 | 2.607 |
| **Study type** |  |  |  |  |  |  |  |
| CC | 10 | Aliased |  |  | 2.083 | 1.388 | 3.125 |
| Pr | 11 | 0.354 | 0.107 | ++ | 2.967 | 2.185 | 4.028 |
| CS | 93 | 0.140 | 0.107 | N.S. | 2.395 | 2.261 | 2.537 |
|  |  | **Deviance** | **(DF)** | **Drop Dev** | **P** |  |  |
| **Adding Study weakness** |  | 401.162 | (97) | 1.166 | N.S. |  |  |
|  |  | **Estimate** | **S.E.** | **P** | **RR** | **95%CIl** | **95%CIu** |
| **Constant** |  | 1.712 | 0.453 | +++ | 5.542 | 2.280 | 13.472 |
| **Sex(RR)** |  |  |  |  |  |  |  |
| Male | 51 | Aliased |  |  | 2.653 | 2.280 | 3.088 |
| Female | 39 | -0.168 | 0.036 | --- | 2.243 | 1.939 | 2.595 |
| Combined | 24 | -0.117 | 0.077 | N.S. | 2.359 | 1.972 | 2.824 |
| **Continent** |  |  |  |  |  |  |  |
| NAmer | 38 | Aliased |  |  | 3.004 | 2.632 | 3.428 |
| Europe | 60 | -0.281 | 0.039 | --- | 2.269 | 2.121 | 2.426 |
| Asia | 9 | -0.284 | 0.060 | --- | 2.261 | 1.892 | 2.703 |
| oth/mult | 7 | -0.179 | 0.074 | - | 2.510 | 1.926 | 3.272 |
| **Asthma analysis type (CB)** |  |  |  |  |  |  |  |
| inc-irresp | 103 | Aliased |  |  | 2.425 | 2.299 | 2.558 |
| excl-all | 7 | -0.119 | 0.066 | (-) | 2.153 | 1.669 | 2.777 |
| defn-incl | 0 | Aliased |  |  | 2.425 | 2.299 | 2.558 |
| other | 4 | 0.600 | 0.424 | N.S. | 4.419 | 0.820 | 23.812 |
| **Smoking product (3)** |  |  |  |  |  |  |  |
| any | 61 | Aliased |  |  | 2.467 | 1.983 | 3.070 |
| cigs | 51 | -0.060 | 0.128 | N.S. | 2.324 | 1.717 | 3.146 |
| cigsonly | 2 | 0.450 | 0.149 | ++ | 3.868 | 2.061 | 7.258 |
| **Unexposed group** |  |  |  |  |  |  |  |
| nev any | 70 | Aliased |  |  | 2.527 | 2.051 | 3.114 |
| nev cig | 44 | -0.112 | 0.126 | N.S. | 2.259 | 1.665 | 3.063 |
| **CB subtype** |  |  |  |  |  |  |  |
| mort | 3 | Aliased |  |  | 3.042 | 1.134 | 8.162 |
| sympt | 83 | -0.210 | 0.249 | N.S. | 2.465 | 2.318 | 2.622 |
| other | 28 | -0.303 | 0.248 | N.S. | 2.248 | 1.981 | 2.552 |
| **Midpoint age in RR** |  | 0.003 | 0.001 | + | 2.089 | 1.639 | 2.663 |
| **RR adjusted for age** |  |  |  |  |  |  |  |
| Yes | 63 | Aliased |  |  | 2.203 | 2.043 | 2.374 |
| No | 51 | 0.212 | 0.032 | +++ | 2.724 | 2.494 | 2.975 |
| **RR adjusted for factor other than sex, age** |  |  |  |  |  |  |  |
| Yes | 30 | Aliased |  |  | 2.571 | 2.184 | 3.026 |
| No | 84 | -0.112 | 0.070 | N.S. | 2.299 | 2.012 | 2.626 |

Table 2 - A - 6

IESCOPD - Meta-regression of ever smoking, any product (or cigarettes if all product not available)

Multiple regression of data from Table 2 - A - 1 (most-adjusted RRs)

Any CB

Test by adding extra variables one at a time

|  |  | **Estimate** | **S.E.** | **P** | **RR** | **95%CIl** | **95%CIu** |
| --- | --- | --- | --- | --- | --- | --- | --- |
| **Study weakness** |  |  |  |  |  |  |  |
| Yes | 5 | Aliased |  |  | 3.585 | 0.834 | 15.421 |
| No | 109 | -0.397 | 0.368 | N.S. | 2.411 | 2.290 | 2.538 |
|  |  | **Deviance** | **(DF)** | **Drop Dev** | **P** |  |  |
| **Adding Number of cases (CB)** |  | 383.482 | (95) | 18.846 | N.S. |  |  |
|  |  | **Estimate** | **S.E.** | **P** | **RR** | **95%CIl** | **95%CIu** |
| **Constant** |  | 1.098 | 0.320 | +++ | 2.997 | 1.601 | 5.612 |
| **Sex(RR)** |  |  |  |  |  |  |  |
| Male | 51 | Aliased |  |  | 2.683 | 2.307 | 3.119 |
| Female | 39 | -0.169 | 0.036 | --- | 2.265 | 1.959 | 2.620 |
| Combined | 24 | -0.143 | 0.078 | (-) | 2.324 | 1.943 | 2.780 |
| **Continent** |  |  |  |  |  |  |  |
| NAmer | 38 | Aliased |  |  | 2.937 | 2.574 | 3.352 |
| Europe | 60 | -0.251 | 0.039 | --- | 2.286 | 2.138 | 2.443 |
| Asia | 9 | -0.248 | 0.060 | --- | 2.293 | 1.922 | 2.736 |
| oth/mult | 7 | -0.189 | 0.074 | - | 2.433 | 1.865 | 3.173 |
| **Asthma analysis type (CB)** |  |  |  |  |  |  |  |
| inc-irresp | 103 | Aliased |  |  | 2.420 | 2.297 | 2.549 |
| excl-all | 7 | -0.108 | 0.066 | N.S. | 2.173 | 1.693 | 2.790 |
| defn-incl | 0 | Aliased |  |  | 2.420 | 2.297 | 2.549 |
| other | 4 | 1.006 | 0.215 | +++ | 6.616 | 2.847 | 15.374 |
| **Smoking product (3)** |  |  |  |  |  |  |  |
| any | 61 | Aliased |  |  | 2.427 | 1.943 | 3.033 |
| cigs | 51 | -0.021 | 0.132 | N.S. | 2.378 | 1.746 | 3.238 |
| cigsonly | 2 | 0.448 | 0.149 | ++ | 3.799 | 2.035 | 7.090 |
| **Unexposed group** |  |  |  |  |  |  |  |
| nev any | 70 | Aliased |  |  | 2.568 | 2.076 | 3.177 |
| nev cig | 44 | -0.153 | 0.130 | N.S. | 2.205 | 1.616 | 3.009 |
| **CB subtype** |  |  |  |  |  |  |  |
| mort | 3 | Aliased |  |  | 2.410 | 0.881 | 6.594 |
| sympt | 83 | 0.026 | 0.257 | N.S. | 2.472 | 2.326 | 2.628 |
| other | 28 | -0.076 | 0.256 | N.S. | 2.233 | 1.970 | 2.531 |
| **Midpoint age in RR** |  | 0.002 | 0.001 | N.S. | 2.193 | 1.716 | 2.803 |
| **RR adjusted for age** |  |  |  |  |  |  |  |
| Yes | 63 | Aliased |  |  | 2.215 | 2.056 | 2.386 |
| No | 51 | 0.199 | 0.032 | +++ | 2.704 | 2.477 | 2.951 |
| **RR adjusted for factor other than sex, age** |  |  |  |  |  |  |  |
| Yes | 30 | Aliased |  |  | 2.596 | 2.207 | 3.053 |
| No | 84 | -0.129 | 0.070 | (-) | 2.281 | 1.998 | 2.604 |
| **Number of cases (CB)** |  |  |  |  |  |  |  |
| 1-50 | 17 | Aliased |  |  | 2.325 | 1.220 | 4.433 |
| 51-100 | 13 | 0.454 | 0.206 | + | 3.661 | 2.241 | 5.981 |
| 101-200 | 19 | 0.255 | 0.182 | N.S. | 3.001 | 2.211 | 4.073 |
| 201+ | 65 | 0.026 | 0.164 | N.S. | 2.388 | 2.267 | 2.514 |
|  |  | **Deviance** | **(DF)** | **Drop Dev** | **P** |  |  |
| **Adding Smoking results reported in study (CB)** |  | 402.219 | (97) | 0.110 | N.S. |  |  |
|  |  | **Estimate** | **S.E.** | **P** | **RR** | **95%CIl** | **95%CIu** |
| **Constant** |  | 1.300 | 0.270 | +++ | 3.670 | 2.160 | 6.235 |
| **Sex(RR)** |  |  |  |  |  |  |  |
| Male | 51 | Aliased |  |  | 2.658 | 2.284 | 3.093 |
| Female | 39 | -0.173 | 0.036 | --- | 2.237 | 1.928 | 2.595 |
| Combined | 24 | -0.118 | 0.077 | N.S. | 2.361 | 1.970 | 2.829 |
| **Continent** |  |  |  |  |  |  |  |
| NAmer | 38 | Aliased |  |  | 3.001 | 2.627 | 3.428 |
| Europe | 60 | -0.280 | 0.040 | --- | 2.267 | 2.116 | 2.429 |
| Asia | 9 | -0.277 | 0.061 | --- | 2.274 | 1.885 | 2.743 |
| oth/mult | 7 | -0.177 | 0.074 | - | 2.513 | 1.927 | 3.277 |

Table 2 - A - 6

IESCOPD - Meta-regression of ever smoking, any product (or cigarettes if all product not available)

Multiple regression of data from Table 2 - A - 1 (most-adjusted RRs)

Any CB

Test by adding extra variables one at a time

|  |  | **Estimate** | **S.E.** | **P** | **RR** | **95%CIl** | **95%CIu** |
| --- | --- | --- | --- | --- | --- | --- | --- |
| **Asthma analysis type (CB)** |  |  |  |  |  |  |  |
| inc-irresp | 103 | Aliased |  |  | 2.420 | 2.296 | 2.551 |
| excl-all | 7 | -0.108 | 0.066 | N.S. | 2.173 | 1.686 | 2.800 |
| defn-incl | 0 | Aliased |  |  | 2.420 | 2.296 | 2.551 |
| other | 4 | 0.995 | 0.214 | +++ | 6.545 | 2.789 | 15.358 |
| **Smoking product (3)** |  |  |  |  |  |  |  |
| any | 61 | Aliased |  |  | 2.475 | 1.982 | 3.090 |
| cigs | 51 | -0.067 | 0.130 | N.S. | 2.314 | 1.701 | 3.147 |
| cigsonly | 2 | 0.450 | 0.149 | ++ | 3.882 | 2.064 | 7.302 |
| **Unexposed group** |  |  |  |  |  |  |  |
| nev any | 70 | Aliased |  |  | 2.523 | 2.045 | 3.112 |
| nev cig | 44 | -0.108 | 0.127 | N.S. | 2.264 | 1.667 | 3.075 |
| **CB subtype** |  |  |  |  |  |  |  |
| mort | 3 | Aliased |  |  | 3.029 | 1.125 | 8.156 |
| sympt | 83 | -0.206 | 0.249 | N.S. | 2.466 | 2.317 | 2.623 |
| other | 28 | -0.299 | 0.248 | N.S. | 2.247 | 1.975 | 2.557 |
| **Midpoint age in RR** |  | 0.003 | 0.001 | + | 2.091 | 1.640 | 2.666 |
| **RR adjusted for age** |  |  |  |  |  |  |  |
| Yes | 63 | Aliased |  |  | 2.198 | 2.035 | 2.374 |
| No | 51 | 0.217 | 0.033 | +++ | 2.731 | 2.494 | 2.991 |
| **RR adjusted for factor other than sex, age** |  |  |  |  |  |  |  |
| Yes | 30 | Aliased |  |  | 2.567 | 2.176 | 3.028 |
| No | 84 | -0.109 | 0.071 | N.S. | 2.302 | 2.011 | 2.634 |
| **Smoking results reported in study (CB)** |  |  |  |  |  |  |  |
| Ever Only | 8 | Aliased |  |  | 2.391 | 2.094 | 2.729 |
| Current Only | 0 | Aliased |  |  | 2.391 | 2.094 | 2.729 |
| Both | 106 | 0.013 | 0.040 | N.S. | 2.423 | 2.276 | 2.579 |
|  |  | **Deviance** | **(DF)** | **Drop Dev** | **P** |  |  |
| **Adding Derivation of RR/CI** |  | 393.546 | (97) | 8.783 | N.S. |  |  |
|  |  | **Estimate** | **S.E.** | **P** | **RR** | **95%CIl** | **95%CIu** |
| **Constant** |  | 1.575 | 0.280 | +++ | 4.832 | 2.791 | 8.365 |
| **Sex(RR)** |  |  |  |  |  |  |  |
| Male | 51 | Aliased |  |  | 2.661 | 2.291 | 3.092 |
| Female | 39 | -0.165 | 0.036 | --- | 2.256 | 1.952 | 2.607 |
| Combined | 24 | -0.127 | 0.077 | N.S. | 2.345 | 1.963 | 2.801 |
| **Continent** |  |  |  |  |  |  |  |
| NAmer | 38 | Aliased |  |  | 3.042 | 2.665 | 3.472 |
| Europe | 60 | -0.299 | 0.039 | --- | 2.257 | 2.110 | 2.413 |
| Asia | 9 | -0.279 | 0.060 | --- | 2.301 | 1.926 | 2.750 |
| oth/mult | 7 | -0.213 | 0.075 | -- | 2.459 | 1.889 | 3.202 |
| **Asthma analysis type (CB)** |  |  |  |  |  |  |  |
| inc-irresp | 103 | Aliased |  |  | 2.421 | 2.298 | 2.551 |
| excl-all | 7 | -0.117 | 0.066 | (-) | 2.153 | 1.676 | 2.766 |
| defn-incl | 0 | Aliased |  |  | 2.421 | 2.298 | 2.551 |
| other | 4 | 1.008 | 0.214 | +++ | 6.635 | 2.854 | 15.428 |
| **Smoking product (3)** |  |  |  |  |  |  |  |
| any | 61 | Aliased |  |  | 2.430 | 1.955 | 3.020 |
| cigs | 51 | -0.023 | 0.129 | N.S. | 2.374 | 1.757 | 3.209 |
| cigsonly | 2 | 0.465 | 0.149 | ++ | 3.868 | 2.074 | 7.214 |
| **Unexposed group** |  |  |  |  |  |  |  |
| nev any | 70 | Aliased |  |  | 2.542 | 2.066 | 3.126 |
| nev cig | 44 | -0.126 | 0.126 | N.S. | 2.240 | 1.656 | 3.029 |
| **CB subtype** |  |  |  |  |  |  |  |
| mort | 3 | Aliased |  |  | 3.106 | 1.168 | 8.257 |
| sympt | 83 | -0.235 | 0.249 | N.S. | 2.455 | 2.309 | 2.610 |
| other | 28 | -0.309 | 0.248 | N.S. | 2.280 | 2.009 | 2.587 |
| **Midpoint age in RR** |  | 0.002 | 0.001 | N.S. | 2.227 | 1.726 | 2.873 |

Table 2 - A - 6

IESCOPD - Meta-regression of ever smoking, any product (or cigarettes if all product not available)

Multiple regression of data from Table 2 - A - 1 (most-adjusted RRs)

Any CB

Test by adding extra variables one at a time

|  |  | **Estimate** | **S.E.** | **P** | **RR** | **95%CIl** | **95%CIu** |
| --- | --- | --- | --- | --- | --- | --- | --- |
| **RR adjusted for age** |  |  |  |  |  |  |  |
| Yes | 63 | Aliased |  |  | 2.231 | 2.067 | 2.408 |
| No | 51 | 0.183 | 0.034 | +++ | 2.679 | 2.447 | 2.932 |
| **RR adjusted for factor other than sex, age** |  |  |  |  |  |  |  |
| Yes | 30 | Aliased |  |  | 2.610 | 2.218 | 3.070 |
| No | 84 | -0.139 | 0.070 | (-) | 2.271 | 1.989 | 2.594 |
| **Derivation of RR/CI** |  |  |  |  |  |  |  |
| Orig/2x2 | 8 | Aliased |  |  | 2.777 | 2.290 | 3.368 |
| Other | 106 | -0.156 | 0.053 | -- | 2.377 | 2.250 | 2.510 |
|  |  | **Deviance** | **(DF)** | **Drop Dev** | **P** |  |  |
| **Adding Analysis type** |  | 400.991 | (97) | 1.337 | N.S. |  |  |
|  |  | **Estimate** | **S.E.** | **P** | **RR** | **95%CIl** | **95%CIu** |
| **Constant** |  | 1.167 | 0.296 | +++ | 3.212 | 1.799 | 5.734 |
| **Sex(RR)** |  |  |  |  |  |  |  |
| Male | 51 | Aliased |  |  | 2.655 | 2.281 | 3.089 |
| Female | 39 | -0.172 | 0.036 | --- | 2.235 | 1.932 | 2.587 |
| Combined | 24 | -0.116 | 0.077 | N.S. | 2.364 | 1.975 | 2.829 |
| **Continent** |  |  |  |  |  |  |  |
| NAmer | 38 | Aliased |  |  | 2.981 | 2.609 | 3.405 |
| Europe | 60 | -0.272 | 0.039 | --- | 2.271 | 2.124 | 2.429 |
| Asia | 9 | -0.271 | 0.060 | --- | 2.273 | 1.901 | 2.718 |
| oth/mult | 7 | -0.164 | 0.075 | - | 2.531 | 1.939 | 3.302 |
| **Asthma analysis type (CB)** |  |  |  |  |  |  |  |
| inc-irresp | 103 | Aliased |  |  | 2.431 | 2.301 | 2.568 |
| excl-all | 7 | -0.196 | 0.099 | (-) | 1.999 | 1.368 | 2.921 |
| defn-incl | 0 | Aliased |  |  | 2.431 | 2.301 | 2.568 |
| other | 4 | 1.008 | 0.214 | +++ | 6.662 | 2.837 | 15.641 |
| **Smoking product (3)** |  |  |  |  |  |  |  |
| any | 61 | Aliased |  |  | 2.487 | 1.996 | 3.100 |
| cigs | 51 | -0.080 | 0.129 | N.S. | 2.297 | 1.693 | 3.117 |
| cigsonly | 2 | 0.463 | 0.150 | ++ | 3.952 | 2.097 | 7.446 |
| **Unexposed group** |  |  |  |  |  |  |  |
| nev any | 70 | Aliased |  |  | 2.510 | 2.035 | 3.096 |
| nev cig | 44 | -0.095 | 0.127 | N.S. | 2.281 | 1.679 | 3.100 |
| **CB subtype** |  |  |  |  |  |  |  |
| mort | 3 | Aliased |  |  | 2.661 | 0.894 | 7.923 |
| sympt | 83 | -0.074 | 0.276 | N.S. | 2.471 | 2.322 | 2.630 |
| other | 28 | -0.175 | 0.271 | N.S. | 2.234 | 1.963 | 2.543 |
| **Midpoint age in RR** |  | 0.003 | 0.001 | + | 2.079 | 1.631 | 2.652 |
| **RR adjusted for age** |  |  |  |  |  |  |  |
| Yes | 63 | Aliased |  |  | 2.193 | 2.033 | 2.367 |
| No | 51 | 0.222 | 0.033 | +++ | 2.739 | 2.504 | 2.995 |
| **RR adjusted for factor other than sex, age** |  |  |  |  |  |  |  |
| Yes | 30 | Aliased |  |  | 2.575 | 2.188 | 3.031 |
| No | 84 | -0.115 | 0.070 | N.S. | 2.296 | 2.009 | 2.623 |
| **Analysis type** |  |  |  |  |  |  |  |
| prevlnce | 105 | Aliased |  |  | 2.401 | 2.273 | 2.536 |
| onset | 9 | 0.142 | 0.123 | N.S. | 2.768 | 1.727 | 4.436 |

Table 2 - A - 6

IESCOPD - Meta-regression of ever smoking, any product (or cigarettes if all product not available)

Multiple regression of data from Table 2 - A - 1 (most-adjusted RRs)

Any CB

Fitted values and residuals

|  | **Study Ref** | **NRR** | **SEX** | **LOGRR** | **FITVAL** | **SEFITV** | **STDRES** |
| --- | --- | --- | --- | --- | --- | --- | --- |
|  |  |  |  |  |  |  |  |
| #2175 | GOLDBE | 6 | m | 0.392 | 0.990 | 0.218 | -2.744 |
| #231 | JOUSI1 | 6 | f | 0.506 | 0.888 | 0.151 | -2.534 |
| #3422 | LINDST | 23 | b | 0.419 | 0.666 | 0.129 | -1.914 |
| #286 | KATO | 3 | m | 0.285 | 0.655 | 0.208 | -1.779 |
| #1730 | ENRIGH | 6 | f | 0.482 | 1.018 | 0.307 | -1.746 |
| #1715 | EHRLIC | 15 | m | 0.239 | 1.097 | 0.516 | -1.662 |
| #2658 | KUBIK | 4 | f | 0.705 | 0.927 | 0.141 | -1.574 |
| #2835 | LEBOWI | 30 | b | 0.122 | 0.886 | 0.522 | -1.463 |
| #228 | JOUSI1 | 3 | m | 0.885 | 1.059 | 0.128 | -1.361 |
| #2723 | OSWAL2 | 2 | m | 0.344 | 0.661 | 0.247 | -1.288 |
| #3486 | PEREZP | 5 | f | 0.079 | 0.783 | 0.564 | -1.249 |
| #1721 | EHRLIC | 21 | f | 0.495 | 0.927 | 0.366 | -1.181 |
| #253 | NEJJAR | 5 | b | 0.445 | 0.779 | 0.313 | -1.067 |
| #3119 | WAGEN2 | 11 | b | 0.542 | 0.770 | 0.241 | -0.946 |
| #278 | VIEGI1 | 6 | f | -1.292 | 0.732 | 2.149 | -0.942 |
| #1558 | BROWN | 1 | m | 0.509 | 1.028 | 0.591 | -0.878 |
| #1727 | ENRIGH | 3 | m | 0.712 | 1.188 | 0.554 | -0.860 |
| #2557 | PANDEY | 12 | f | 0.565 | 0.828 | 0.312 | -0.844 |
| #341 | SHIMUR | 3 | b | 0.000 | 1.873 | 2.545 | -0.736 |
| #1820 | HUCHON | 6 | b | 0.793 | 0.933 | 0.193 | -0.726 |
| #3226 | KAHN2 | 32 | m | 1.012 | 1.373 | 0.599 | -0.604 |
| #240 | KOTAN1 | 9 | b | 0.560 | 0.747 | 0.323 | -0.581 |
| #1742 | FOXMAN | 3 | m | 0.908 | 1.123 | 0.370 | -0.579 |
| #3127 | ZOIA | 3 | b | 0.372 | 0.834 | 0.809 | -0.572 |
| #3551 | SILVA | 6 | b | 0.565 | 0.898 | 0.599 | -0.555 |
| #1553 | BECK2 | 3 | m | 0.665 | 1.315 | 1.670 | -0.390 |
| #2134 | FERRI1 | 82 | f | 0.775 | 0.967 | 0.493 | -0.389 |
| #2769 | MEREN | 6 | b | 0.560 | 0.651 | 0.242 | -0.376 |
| #2168 | FINKLE | 1 | m | 1.234 | 1.268 | 0.093 | -0.364 |
| #1959 | LANGHA | 12 | f | 0.668 | 0.719 | 0.142 | -0.358 |
| #1791 | HIGGI3 | 3 | m | 0.024 | 1.103 | 3.084 | -0.350 |
| #1973 | LUNDB2 | 12 | f | 1.421 | 1.598 | 0.593 | -0.298 |
| #2022 | OGILVI | 8 | f | 0.830 | 0.948 | 0.400 | -0.295 |
| #1556 | BECK2 | 6 | f | 0.756 | 1.145 | 1.524 | -0.255 |
| #2664 | ANDER1 | 35 | f | 0.788 | 0.967 | 0.738 | -0.242 |
| #1680 | DOPICO | 3 | m | 0.928 | 1.153 | 0.946 | -0.238 |
| #2700 | BJORNS | 6 | b | 0.779 | 0.801 | 0.142 | -0.154 |
| #1550 | BECK1 | 6 | f | 0.985 | 1.145 | 1.053 | -0.152 |
| #2368 | HAENSZ | 10 | m | 0.820 | 0.875 | 0.422 | -0.130 |
| #545 | ALDERS | 6 | f | 1.026 | 1.063 | 0.295 | -0.126 |
| #214 | MANFRE | 6 | f | 1.102 | 1.160 | 0.581 | -0.100 |
| #2720 | JINDA2 | 3 | b | 0.896 | 0.902 | 0.132 | -0.047 |
| #1748 | FOXMAN | 6 | f | 0.948 | 0.952 | 0.325 | -0.013 |
| #2058 | FERRI1 | 61 | m | 1.169 | 1.137 | 0.711 | 0.045 |
| #1976 | MELLST | 3 | m | 1.072 | 1.046 | 0.578 | 0.045 |
| #1773 | HIGGI2 | 3 | m | 1.001 | 0.902 | 2.105 | 0.047 |
| #2016 | OGILVI | 3 | m | 1.187 | 1.118 | 0.741 | 0.092 |
| #2706 | DEMARC | 20 | b | 1.085 | 1.056 | 0.244 | 0.118 |
| #2725 | OSWAL2 | 4 | f | 0.525 | 0.491 | 0.272 | 0.125 |
| #1547 | BECK1 | 3 | m | 1.483 | 1.315 | 1.273 | 0.132 |
| #1631 | COLLEG | 6 | m | 1.058 | 0.891 | 1.111 | 0.150 |
| #259 | MOLLER | 3 | b | 2.072 | 1.798 | 1.742 | 0.157 |
| #3485 | PELKON | 6 | m | 0.565 | 0.513 | 0.300 | 0.175 |
| #208 | MANFRE | 3 | m | 1.687 | 1.331 | 1.284 | 0.277 |
| #1751 | HARRIS | 3 | m | 1.352 | 1.030 | 1.019 | 0.316 |
| #1785 | HIGGI2 | 15 | f | 0.900 | 0.518 | 1.198 | 0.319 |
| #2772 | MILNE | 15 | f | 1.263 | 0.984 | 0.866 | 0.322 |
| #2661 | ANDER1 | 32 | m | 1.506 | 1.137 | 1.116 | 0.330 |
| #3570 | YAMAGU | 9 | b | 0.815 | 0.737 | 0.224 | 0.350 |
| #2894 | HIGGI6 | 10 | f | 1.095 | 0.952 | 0.399 | 0.359 |
| #2594 | TROISI | 3 | f | 0.658 | 0.602 | 0.155 | 0.359 |
| #3295 | HO | 11 | b | 0.842 | 0.692 | 0.415 | 0.362 |

Table 2 - A - 6

IESCOPD - Meta-regression of ever smoking, any product (or cigarettes if all product not available)

Multiple regression of data from Table 2 - A - 1 (most-adjusted RRs)

Any CB

Fitted values and residuals

|  | **Study Ref** | **NRR** | **SEX** | **LOGRR** | **FITVAL** | **SEFITV** | **STDRES** |
| --- | --- | --- | --- | --- | --- | --- | --- |
|  |  |  |  |  |  |  |  |
| #1812 | HOUSE | 6 | m | 1.437 | 1.317 | 0.322 | 0.376 |
| #114 | LAVECC | 9 | b | 0.820 | 0.786 | 0.082 | 0.418 |
| #275 | VIEGI1 | 3 | m | 1.355 | 0.903 | 1.071 | 0.422 |
| #2580 | STJERN | 3 | b | 1.369 | 0.938 | 0.982 | 0.439 |
| #2640 | WILHEL | 3 | m | 2.024 | 1.089 | 2.097 | 0.446 |
| #1967 | LUNDB2 | 6 | m | 2.059 | 1.768 | 0.645 | 0.451 |
| #1615 | COATES | 3 | b | 1.288 | 1.068 | 0.486 | 0.452 |
| #203 | MENEZ1 | 6 | b | 1.075 | 0.841 | 0.508 | 0.460 |
| #3106 | HUHTI3 | 22 | m | 1.068 | 0.854 | 0.455 | 0.472 |
| #1801 | HOLLA2 | 3 | m | 1.801 | 1.351 | 0.953 | 0.472 |
| #1797 | HIGGI3 | 9 | f | 2.499 | 0.933 | 3.172 | 0.494 |
| #1921 | LAMBER | 6 | m | 0.956 | 0.697 | 0.517 | 0.500 |
| #544 | ALDERS | 5 | m | 1.033 | 0.784 | 0.495 | 0.502 |
| #3588 | BEST | 3 | m | 2.437 | 1.841 | 1.173 | 0.509 |
| #90 | DONTA2 | 3 | m | 1.100 | 0.718 | 0.729 | 0.524 |
| #1675 | DEANE | 6 | m | 1.816 | 1.137 | 1.251 | 0.543 |
| #1953 | LANGHA | 6 | m | 0.978 | 0.889 | 0.162 | 0.548 |
| #3433 | MUELLE | 36 | m | 1.957 | 1.123 | 1.498 | 0.557 |
| #1989 | MILLER | 3 | m | 1.656 | 1.227 | 0.683 | 0.629 |
| #1634 | COLLEG | 9 | f | 0.971 | 0.608 | 0.556 | 0.653 |
| #3294 | KIRAZ | 4 | f | 1.386 | 0.793 | 0.845 | 0.702 |
| #1559 | DOLL1 | 13 | m | 2.001 | 1.102 | 1.240 | 0.726 |
| #2887 | HIGGI6 | 3 | m | 1.524 | 1.123 | 0.519 | 0.774 |
| #2181 | GOLDBE | 12 | f | 1.054 | 0.819 | 0.298 | 0.788 |
| #2873 | HAWTHO | 10 | m | 1.179 | 0.935 | 0.304 | 0.803 |
| #3012 | HUHTI1 | 91 | f | 1.289 | 0.698 | 0.736 | 0.803 |
| #272 | CERVER | 6 | b | 0.924 | 0.844 | 0.096 | 0.834 |
| #2551 | PANDEY | 6 | m | 1.681 | 0.999 | 0.780 | 0.874 |
| #2864 | HARDIE | 12 | f | 1.203 | 0.532 | 0.764 | 0.879 |
| #265 | MAGNUS | 6 | m | 1.430 | 0.907 | 0.589 | 0.888 |
| #3511 | REID | 22 | f | 1.200 | 0.810 | 0.438 | 0.889 |
| #2001 | MILNE | 3 | m | 2.111 | 0.983 | 1.253 | 0.900 |
| #243 | HOLLNA | 3 | m | 1.710 | 1.047 | 0.705 | 0.941 |
| #2861 | HARDIE | 9 | m | 2.389 | 0.703 | 1.719 | 0.981 |
| #3448 | MUELLE | 51 | f | 2.350 | 0.952 | 1.387 | 1.008 |
| #3133 | LAMBER | 38 | f | 1.058 | 0.698 | 0.356 | 1.012 |
| #2604 | HOLLNA | 11 | f | 1.516 | 0.876 | 0.621 | 1.031 |
| #3495 | REID | 6 | m | 1.528 | 0.981 | 0.528 | 1.037 |
| #2652 | JOSHI | 6 | m | 1.841 | 1.044 | 0.759 | 1.050 |
| #289 | KATO | 6 | f | 0.652 | 0.484 | 0.155 | 1.083 |
| #5 | WOODS | 3 | b | 0.900 | 0.731 | 0.156 | 1.084 |
| #3524 | SAWICK | 5 | m | 1.735 | 1.059 | 0.568 | 1.191 |
| #2932 | HUHTI1 | 42 | m | 1.953 | 0.869 | 0.825 | 1.314 |
| #1992 | MILLER | 6 | f | 3.009 | 1.056 | 1.465 | 1.333 |
| #2880 | HAWTHO | 17 | f | 1.080 | 0.764 | 0.234 | 1.348 |
| #1818 | HOUSE | 12 | f | 1.497 | 1.146 | 0.259 | 1.357 |
| #3537 | SAWICK | 18 | f | 1.428 | 0.888 | 0.391 | 1.382 |
| #2394 | HAENSZ | 32 | f | 1.200 | 0.704 | 0.322 | 1.542 |
| #224 | WOOLF | 3 | f | 1.956 | 0.870 | 0.704 | 1.544 |
| #2921 | SOBRAD | 4 | b | 1.899 | 0.867 | 0.574 | 1.799 |
| #2657 | KUBIK | 3 | m | 1.327 | 1.098 | 0.108 | 2.116 |
| #2167 | HAYES | 9 | b | 1.740 | 0.982 | 0.346 | 2.194 |

## Table 2 - B - 6

IESCOPD - Meta-regression of current smoking, any product (or cigarettes if all product not available)

Multiple regression of data from Table 2 - B - 1 (most-adjusted RRs)

Any CB

Fixed model

|  |  | **Deviance** | **(DF)** |  |  |  |  |
| --- | --- | --- | --- | --- | --- | --- | --- |
| **Fixed model** |  | 407.697 | (97) |  |  |  |  |
|  |  | **Estimate** | **S.E.** | **P** | **RR** | **95%CIl** | **95%CIu** |
| **Constant** |  | 1.335 | 0.258 | +++ | 3.802 | 2.293 | 6.303 |
| **Sex(RR)** |  |  |  |  |  |  |  |
| Male | 51 | Aliased |  |  | 2.847 | 2.363 | 3.431 |
| Female | 37 | -0.097 | 0.043 | - | 2.583 | 2.163 | 3.085 |
| Combined | 25 | 0.039 | 0.082 | N.S. | 2.959 | 2.476 | 3.537 |
| **Continent** |  |  |  |  |  |  |  |
| NAmer | 40 | Aliased |  |  | 3.773 | 3.175 | 4.484 |
| Europe | 58 | -0.353 | 0.052 | --- | 2.651 | 2.436 | 2.885 |
| Asia | 9 | -0.493 | 0.070 | --- | 2.304 | 1.780 | 2.984 |
| oth/mult | 6 | -0.144 | 0.086 | (-) | 3.268 | 2.416 | 4.420 |
| **Asthma analysis type (CB)** |  |  |  |  |  |  |  |
| inc-irresp | 103 | Aliased |  |  | 2.775 | 2.603 | 2.959 |
| excl-all | 6 | 0.159 | 0.076 | + | 3.253 | 2.431 | 4.353 |
| defn-incl | 0 | Aliased |  |  | 2.775 | 2.603 | 2.959 |
| other | 4 | 1.225 | 0.237 | +++ | 9.446 | 3.661 | 24.368 |
| **Smoking product (3)** |  |  |  |  |  |  |  |
| any | 57 | Aliased |  |  | 2.833 | 2.143 | 3.746 |
| cigs | 52 | -0.023 | 0.136 | N.S. | 2.770 | 2.088 | 3.675 |
| cigsonly | 4 | 0.410 | 0.136 | ++ | 4.270 | 2.333 | 7.818 |
| **Unexposed group** |  |  |  |  |  |  |  |
| nev any | 69 | Aliased |  |  | 2.994 | 2.315 | 3.871 |
| nev cig | 44 | -0.127 | 0.130 | N.S. | 2.638 | 1.994 | 3.488 |
| **CB subtype** |  |  |  |  |  |  |  |
| mort | 4 | Aliased |  |  | 3.390 | 1.341 | 8.572 |
| sympt | 81 | -0.104 | 0.232 | N.S. | 3.056 | 2.823 | 3.308 |
| other | 28 | -0.406 | 0.232 | (-) | 2.259 | 1.948 | 2.620 |
| **Midpoint age in RR** |  | 0.003 | 0.002 | + | 2.399 | 1.723 | 3.340 |
| **RR adjusted for age** |  |  |  |  |  |  |  |
| Yes | 65 | Aliased |  |  | 2.608 | 2.403 | 2.831 |
| No | 48 | 0.219 | 0.039 | +++ | 3.247 | 2.887 | 3.652 |
| **RR adjusted for factor other than sex, age** |  |  |  |  |  |  |  |
| Yes | 29 | Aliased |  |  | 2.827 | 2.419 | 3.305 |
| No | 84 | -0.007 | 0.072 | N.S. | 2.808 | 2.398 | 3.289 |

Table 2 - B - 6

IESCOPD - Meta-regression of current smoking, any product (or cigarettes if all product not available)

Multiple regression of data from Table 2 - B - 1 (most-adjusted RRs)

Any CB

Test by removing variables one at a time

|  |  | **Deviance** | **(DF)** | **Drop Dev** | **P** |  |  |
| --- | --- | --- | --- | --- | --- | --- | --- |
| **Omitting Sex (RR)** |  | 414.288 | (99) | -6.590 | N.S. |  |  |
|  |  | **Estimate** | **S.E.** | **P** | **RR** | **95%CIl** | **95%CIu** |
| **Constant** |  | 1.420 | 0.247 | +++ | 4.137 | 2.552 | 6.706 |
| **RR adjusted for factor other than sex, age** |  |  |  |  |  |  |  |
| Yes | 29 | Aliased |  |  | 2.923 | 2.657 | 3.217 |
| No | 84 | -0.074 | 0.038 | (-) | 2.715 | 2.465 | 2.990 |
| **Continent** |  |  |  |  |  |  |  |
| NAmer | 40 | Aliased |  |  | 3.689 | 3.137 | 4.338 |
| Europe | 58 | -0.321 | 0.048 | --- | 2.676 | 2.470 | 2.899 |
| Asia | 9 | -0.494 | 0.070 | --- | 2.251 | 1.754 | 2.888 |
| oth/mult | 6 | -0.130 | 0.086 | N.S. | 3.238 | 2.407 | 4.357 |
| **Asthma analysis type (CB)** |  |  |  |  |  |  |  |
| inc-irresp | 103 | Aliased |  |  | 2.780 | 2.609 | 2.962 |
| excl-all | 6 | 0.134 | 0.075 | (+) | 3.179 | 2.389 | 4.229 |
| defn-incl | 0 | Aliased |  |  | 2.780 | 2.609 | 2.962 |
| other | 4 | 1.213 | 0.236 | +++ | 9.348 | 3.661 | 23.867 |
| **Smoking product (3)** |  |  |  |  |  |  |  |
| any | 57 | Aliased |  |  | 2.795 | 2.128 | 3.672 |
| cigs | 52 | 0.005 | 0.134 | N.S. | 2.809 | 2.132 | 3.702 |
| cigsonly | 4 | 0.390 | 0.135 | ++ | 4.128 | 2.285 | 7.457 |
| **Unexposed group** |  |  |  |  |  |  |  |
| nev any | 69 | Aliased |  |  | 3.009 | 2.336 | 3.878 |
| nev cig | 44 | -0.138 | 0.130 | N.S. | 2.622 | 1.991 | 3.455 |
| **CB subtype** |  |  |  |  |  |  |  |
| mort | 4 | Aliased |  |  | 3.628 | 1.457 | 9.030 |
| sympt | 81 | -0.175 | 0.231 | N.S. | 3.045 | 2.817 | 3.291 |
| other | 28 | -0.465 | 0.231 | - | 2.279 | 1.973 | 2.632 |
| **Midpoint age in RR** |  | 0.003 | 0.002 | (+) | 2.449 | 1.777 | 3.375 |
| **RR adjusted for age** |  |  |  |  |  |  |  |
| Yes | 65 | Aliased |  |  | 2.613 | 2.410 | 2.832 |
| No | 48 | 0.214 | 0.038 | +++ | 3.237 | 2.886 | 3.631 |

Table 2 - B - 6

IESCOPD - Meta-regression of current smoking, any product (or cigarettes if all product not available)

Multiple regression of data from Table 2 - B - 1 (most-adjusted RRs)

Any CB

Test by removing variables one at a time

|  |  | **Deviance** | **(DF)** | **Drop Dev** | **P** |  |  |
| --- | --- | --- | --- | --- | --- | --- | --- |
| **Omitting Continient** |  | 482.363 | (100) | -74.666 | *** |  |  |
|  |  | **Estimate** | **S.E.** | **P** | **RR** | **95%CIl** | **95%CIu** |
| **Constant** |  | 1.404 | 0.257 | +++ | 4.071 | 2.460 | 6.737 |
| **RR adjusted for factor other than sex, age** |  |  |  |  |  |  |  |
| Yes | 29 | Aliased |  |  | 2.940 | 2.532 | 3.414 |
| No | 84 | -0.086 | 0.070 | N.S. | 2.699 | 2.320 | 3.140 |
| **Sex(RR)** |  |  |  |  |  |  |  |
| Male | 51 | Aliased |  |  | 3.035 | 2.546 | 3.619 |
| Female | 37 | -0.082 | 0.043 | (-) | 2.797 | 2.379 | 3.289 |
| Combined | 25 | -0.107 | 0.076 | N.S. | 2.726 | 2.318 | 3.205 |
| **Asthma analysis type (CB)** |  |  |  |  |  |  |  |
| inc-irresp | 103 | Aliased |  |  | 2.757 | 2.589 | 2.937 |
| excl-all | 6 | 0.266 | 0.073 | +++ | 3.598 | 2.726 | 4.747 |
| defn-incl | 0 | Aliased |  |  | 2.757 | 2.589 | 2.937 |
| other | 4 | 1.175 | 0.236 | +++ | 8.929 | 3.520 | 22.652 |
| **Smoking product (3)** |  |  |  |  |  |  |  |
| any | 57 | Aliased |  |  | 2.560 | 2.017 | 3.248 |
| cigs | 52 | 0.182 | 0.117 | N.S. | 3.072 | 2.415 | 3.908 |
| cigsonly | 4 | 0.386 | 0.134 | ++ | 3.767 | 2.129 | 6.665 |
| **Unexposed group** |  |  |  |  |  |  |  |
| nev any | 69 | Aliased |  |  | 3.200 | 2.548 | 4.019 |
| nev cig | 44 | -0.266 | 0.116 | - | 2.453 | 1.915 | 3.142 |
| **CB subtype** |  |  |  |  |  |  |  |
| mort | 4 | Aliased |  |  | 3.938 | 1.589 | 9.762 |
| sympt | 81 | -0.249 | 0.231 | N.S. | 3.069 | 2.841 | 3.315 |
| other | 28 | -0.570 | 0.231 | - | 2.227 | 1.931 | 2.568 |
| **Midpoint age in RR** |  | 0.001 | 0.002 | N.S. | 2.724 | 1.994 | 3.721 |
| **RR adjusted for age** |  |  |  |  |  |  |  |
| Yes | 65 | Aliased |  |  | 2.658 | 2.458 | 2.874 |
| No | 48 | 0.166 | 0.036 | +++ | 3.137 | 2.812 | 3.498 |
|  |  | **Deviance** | **(DF)** | **Drop Dev** | **P** |  |  |
| **Omitting Asthma analysis type (CB)** |  | 437.373 | (99) | -29.676 | * |  |  |
|  |  | **Estimate** | **S.E.** | **P** | **RR** | **95%CIl** | **95%CIu** |
| **Constant** |  | 1.394 | 0.257 | +++ | 4.029 | 2.433 | 6.673 |
| **RR adjusted for factor other than sex, age** |  |  |  |  |  |  |  |
| Yes | 29 | Aliased |  |  | 2.805 | 2.404 | 3.272 |
| No | 84 | 0.009 | 0.072 | N.S. | 2.831 | 2.422 | 3.309 |
| **Sex(RR)** |  |  |  |  |  |  |  |
| Male | 51 | Aliased |  |  | 2.879 | 2.394 | 3.462 |
| Female | 37 | -0.091 | 0.043 | - | 2.629 | 2.208 | 3.129 |
| Combined | 25 | 0.011 | 0.082 | N.S. | 2.911 | 2.442 | 3.469 |
| **Continent** |  |  |  |  |  |  |  |
| NAmer | 40 | Aliased |  |  | 3.745 | 3.159 | 4.441 |
| Europe | 58 | -0.338 | 0.052 | --- | 2.670 | 2.456 | 2.903 |
| Asia | 9 | -0.529 | 0.068 | --- | 2.207 | 1.721 | 2.829 |
| oth/mult | 6 | -0.136 | 0.086 | N.S. | 3.269 | 2.424 | 4.408 |
| **Smoking product (3)** |  |  |  |  |  |  |  |
| any | 57 | Aliased |  |  | 2.812 | 2.134 | 3.707 |
| cigs | 52 | -0.006 | 0.136 | N.S. | 2.796 | 2.115 | 3.697 |
| cigsonly | 4 | 0.339 | 0.134 | + | 3.947 | 2.183 | 7.138 |

Table 2 - B - 6

IESCOPD - Meta-regression of current smoking, any product (or cigarettes if all product not available)

Multiple regression of data from Table 2 - B - 1 (most-adjusted RRs)

Any CB

Test by removing variables one at a time

|  |  | **Estimate** | **S.E.** | **P** | **RR** | **95%CIl** | **95%CIu** |
| --- | --- | --- | --- | --- | --- | --- | --- |
| **Unexposed group** |  |  |  |  |  |  |  |
| nev any | 69 | Aliased |  |  | 2.974 | 2.306 | 3.834 |
| nev cig | 44 | -0.113 | 0.130 | N.S. | 2.657 | 2.016 | 3.503 |
| **CB subtype** |  |  |  |  |  |  |  |
| mort | 4 | Aliased |  |  | 3.397 | 1.356 | 8.508 |
| sympt | 81 | -0.124 | 0.232 | N.S. | 3.001 | 2.783 | 3.238 |
| other | 28 | -0.360 | 0.232 | N.S. | 2.371 | 2.070 | 2.715 |
| **Midpoint age in RR** |  | 0.002 | 0.002 | N.S. | 2.536 | 1.838 | 3.499 |
| **RR adjusted for age** |  |  |  |  |  |  |  |
| Yes | 65 | Aliased |  |  | 2.614 | 2.411 | 2.836 |
| No | 48 | 0.212 | 0.039 | +++ | 3.233 | 2.878 | 3.631 |
|  |  | **Deviance** | **(DF)** | **Drop Dev** | **P** |  |  |
| **Omitting Smoking product (3)** |  | 416.868 | (99) | -9.171 | N.S. |  |  |
|  |  | **Estimate** | **S.E.** | **P** | **RR** | **95%CIl** | **95%CIu** |
| **Constant** |  | 1.408 | 0.256 | +++ | 4.090 | 2.475 | 6.759 |
| **RR adjusted for factor other than sex, age** |  |  |  |  |  |  |  |
| Yes | 29 | Aliased |  |  | 2.825 | 2.423 | 3.294 |
| No | 84 | -0.005 | 0.072 | N.S. | 2.810 | 2.405 | 3.284 |
| **Sex(RR)** |  |  |  |  |  |  |  |
| Male | 51 | Aliased |  |  | 2.874 | 2.394 | 3.450 |
| Female | 37 | -0.095 | 0.043 | - | 2.614 | 2.198 | 3.109 |
| Combined | 25 | 0.017 | 0.081 | N.S. | 2.923 | 2.457 | 3.477 |
| **Continent** |  |  |  |  |  |  |  |
| NAmer | 40 | Aliased |  |  | 3.727 | 3.148 | 4.413 |
| Europe | 58 | -0.335 | 0.051 | --- | 2.667 | 2.460 | 2.891 |
| Asia | 9 | -0.505 | 0.070 | --- | 2.250 | 1.749 | 2.895 |
| oth/mult | 6 | -0.130 | 0.081 | N.S. | 3.273 | 2.507 | 4.272 |
| **Asthma analysis type (CB)** |  |  |  |  |  |  |  |
| inc-irresp | 103 | Aliased |  |  | 2.781 | 2.610 | 2.963 |
| excl-all | 6 | 0.129 | 0.075 | (+) | 3.165 | 2.379 | 4.212 |
| defn-incl | 0 | Aliased |  |  | 2.781 | 2.610 | 2.963 |
| other | 4 | 1.180 | 0.236 | +++ | 9.053 | 3.549 | 23.094 |
| **Unexposed group** |  |  |  |  |  |  |  |
| nev any | 69 | Aliased |  |  | 3.017 | 2.739 | 3.322 |
| nev cig | 44 | -0.143 | 0.040 | --- | 2.616 | 2.362 | 2.897 |
| **CB subtype** |  |  |  |  |  |  |  |
| mort | 4 | Aliased |  |  | 3.583 | 1.437 | 8.930 |
| sympt | 81 | -0.169 | 0.231 | N.S. | 3.026 | 2.800 | 3.269 |
| other | 28 | -0.436 | 0.231 | (-) | 2.318 | 2.010 | 2.673 |
| **Midpoint age in RR** |  | 0.003 | 0.002 | (+) | 2.437 | 1.757 | 3.380 |
| **RR adjusted for age** |  |  |  |  |  |  |  |
| Yes | 65 | Aliased |  |  | 2.610 | 2.411 | 2.825 |
| No | 48 | 0.217 | 0.037 | +++ | 3.243 | 2.900 | 3.627 |
|  |  | **Deviance** | **(DF)** | **Drop Dev** | **P** |  |  |
| **Omitting Unexposed group** |  | 408.647 | (98) | -0.950 | N.S. |  |  |
|  |  | **Estimate** | **S.E.** | **P** | **RR** | **95%CIl** | **95%CIu** |
| **Constant** |  | 1.350 | 0.258 | +++ | 3.856 | 2.328 | 6.388 |
| **RR adjusted for factor other than sex, age** |  |  |  |  |  |  |  |
| Yes | 29 | Aliased |  |  | 2.818 | 2.414 | 3.289 |
| No | 84 | 0.000 | 0.072 | N.S. | 2.818 | 2.410 | 3.295 |
| **Sex(RR)** |  |  |  |  |  |  |  |
| Male | 51 | Aliased |  |  | 2.838 | 2.358 | 3.414 |
| Female | 37 | -0.097 | 0.043 | - | 2.575 | 2.159 | 3.072 |
| Combined | 25 | 0.046 | 0.082 | N.S. | 2.970 | 2.489 | 3.544 |
| **Continent** |  |  |  |  |  |  |  |
| NAmer | 40 | Aliased |  |  | 3.775 | 3.180 | 4.483 |
| Europe | 58 | -0.357 | 0.052 | --- | 2.643 | 2.432 | 2.872 |
| Asia | 9 | -0.493 | 0.070 | --- | 2.305 | 1.782 | 2.981 |
| oth/mult | 6 | -0.113 | 0.080 | N.S. | 3.373 | 2.574 | 4.422 |

Table 2 - B - 6

IESCOPD - Meta-regression of current smoking, any product (or cigarettes if all product not available)

Multiple regression of data from Table 2 - B - 1 (most-adjusted RRs)

Any CB

Test by removing variables one at a time

|  |  | **Estimate** | **S.E.** | **P** | **RR** | **95%CIl** | **95%CIu** |
| --- | --- | --- | --- | --- | --- | --- | --- |
| **Asthma analysis type (CB)** |  |  |  |  |  |  |  |
| inc-irresp | 103 | Aliased |  |  | 2.776 | 2.604 | 2.959 |
| excl-all | 6 | 0.155 | 0.076 | + | 3.241 | 2.427 | 4.328 |
| defn-incl | 0 | Aliased |  |  | 2.776 | 2.604 | 2.959 |
| other | 4 | 1.224 | 0.237 | +++ | 9.441 | 3.677 | 24.239 |
| **Smoking product (3)** |  |  |  |  |  |  |  |
| any | 57 | Aliased |  |  | 3.017 | 2.723 | 3.343 |
| cigs | 52 | -0.149 | 0.042 | --- | 2.599 | 2.343 | 2.883 |
| cigsonly | 4 | 0.408 | 0.136 | ++ | 4.538 | 2.625 | 7.846 |
| **CB subtype** |  |  |  |  |  |  |  |
| mort | 4 | Aliased |  |  | 3.452 | 1.376 | 8.662 |
| sympt | 81 | -0.123 | 0.232 | N.S. | 3.052 | 2.821 | 3.302 |
| other | 28 | -0.421 | 0.232 | (-) | 2.266 | 1.957 | 2.624 |
| **Midpoint age in RR** |  | 0.003 | 0.002 | (+) | 2.405 | 1.731 | 3.343 |
| **RR adjusted for age** |  |  |  |  |  |  |  |
| Yes | 65 | Aliased |  |  | 2.600 | 2.399 | 2.818 |
| No | 48 | 0.228 | 0.037 | +++ | 3.267 | 2.913 | 3.662 |
|  |  | **Deviance** | **(DF)** | **Drop Dev** | **P** |  |  |
| **Omitting CB subtype** |  | 451.929 | (99) | -44.232 | ** |  |  |
|  |  | **Estimate** | **S.E.** | **P** | **RR** | **95%CIl** | **95%CIu** |
| **Constant** |  | 1.356 | 0.119 | +++ | 3.880 | 3.074 | 4.898 |
| **RR adjusted for factor other than sex, age** |  |  |  |  |  |  |  |
| Yes | 29 | Aliased |  |  | 2.845 | 2.438 | 3.319 |
| No | 84 | -0.019 | 0.072 | N.S. | 2.791 | 2.387 | 3.263 |
| **Sex(RR)** |  |  |  |  |  |  |  |
| Male | 51 | Aliased |  |  | 2.992 | 2.494 | 3.590 |
| Female | 37 | -0.100 | 0.042 | - | 2.708 | 2.279 | 3.218 |
| Combined | 25 | -0.066 | 0.081 | N.S. | 2.802 | 2.356 | 3.331 |
| **Continent** |  |  |  |  |  |  |  |
| NAmer | 40 | Aliased |  |  | 3.703 | 3.126 | 4.387 |
| Europe | 58 | -0.323 | 0.051 | --- | 2.680 | 2.466 | 2.913 |
| Asia | 9 | -0.561 | 0.069 | --- | 2.114 | 1.646 | 2.715 |
| oth/mult | 6 | -0.072 | 0.085 | N.S. | 3.445 | 2.560 | 4.635 |
| **Asthma analysis type (CB)** |  |  |  |  |  |  |  |
| inc-irresp | 103 | Aliased |  |  | 2.813 | 2.641 | 2.996 |
| excl-all | 6 | -0.041 | 0.069 | N.S. | 2.699 | 2.070 | 3.519 |
| defn-incl | 0 | Aliased |  |  | 2.813 | 2.641 | 2.996 |
| other | 4 | 0.993 | 0.234 | +++ | 7.592 | 2.999 | 19.218 |
| **Smoking product (3)** |  |  |  |  |  |  |  |
| any | 57 | Aliased |  |  | 2.869 | 2.178 | 3.780 |
| cigs | 52 | -0.042 | 0.136 | N.S. | 2.752 | 2.081 | 3.638 |
| cigsonly | 4 | 0.194 | 0.131 | N.S. | 3.482 | 1.941 | 6.248 |
| **Unexposed group** |  |  |  |  |  |  |  |
| nev any | 69 | Aliased |  |  | 2.895 | 2.249 | 3.727 |
| nev cig | 44 | -0.056 | 0.129 | N.S. | 2.736 | 2.079 | 3.601 |
| **Midpoint age in RR** |  | -0.001 | 0.002 | N.S. | 2.899 | 2.133 | 3.939 |
| **RR adjusted for age** |  |  |  |  |  |  |  |
| Yes | 65 | Aliased |  |  | 2.562 | 2.364 | 2.777 |
| No | 48 | 0.270 | 0.038 | +++ | 3.355 | 2.992 | 3.762 |
|  |  | **Deviance** | **(DF)** | **Drop Dev** | **P** |  |  |
| **Omitting Midpoint age in RR** |  | 411.651 | (98) | -3.954 | N.S. |  |  |
|  |  | **Estimate** | **S.E.** | **P** | **RR** | **95%CIl** | **95%CIu** |
| **Constant** |  | 1.532 | 0.238 | +++ | 4.626 | 2.899 | 7.380 |
| **RR adjusted for factor other than sex, age** |  |  |  |  |  |  |  |
| Yes | 29 | Aliased |  |  | 2.835 | 2.428 | 3.311 |
| No | 84 | -0.012 | 0.072 | N.S. | 2.800 | 2.393 | 3.277 |

Table 2 - B - 6

IESCOPD - Meta-regression of current smoking, any product (or cigarettes if all product not available)

Multiple regression of data from Table 2 - B - 1 (most-adjusted RRs)

Any CB

Test by removing variables one at a time

|  |  | **Estimate** | **S.E.** | **P** | **RR** | **95%CIl** | **95%CIu** |
| --- | --- | --- | --- | --- | --- | --- | --- |
| **Sex(RR)** |  |  |  |  |  |  |  |
| Male | 51 | Aliased |  |  | 2.890 | 2.407 | 3.471 |
| Female | 37 | -0.097 | 0.043 | - | 2.623 | 2.204 | 3.121 |
| Combined | 25 | 0.006 | 0.081 | N.S. | 2.909 | 2.444 | 3.462 |
| **Continent** |  |  |  |  |  |  |  |
| NAmer | 40 | Aliased |  |  | 3.765 | 3.171 | 4.470 |
| Europe | 58 | -0.352 | 0.052 | --- | 2.648 | 2.434 | 2.881 |
| Asia | 9 | -0.461 | 0.068 | --- | 2.374 | 1.849 | 3.048 |
| oth/mult | 6 | -0.168 | 0.085 | (-) | 3.181 | 2.367 | 4.276 |
| **Asthma analysis type (CB)** |  |  |  |  |  |  |  |
| inc-irresp | 103 | Aliased |  |  | 2.781 | 2.609 | 2.964 |
| excl-all | 6 | 0.131 | 0.074 | (+) | 3.171 | 2.384 | 4.217 |
| defn-incl | 0 | Aliased |  |  | 2.781 | 2.609 | 2.964 |
| other | 4 | 1.190 | 0.236 | +++ | 9.136 | 3.567 | 23.399 |
| **Smoking product (3)** |  |  |  |  |  |  |  |
| any | 57 | Aliased |  |  | 2.839 | 2.150 | 3.748 |
| cigs | 52 | -0.026 | 0.136 | N.S. | 2.766 | 2.088 | 3.664 |
| cigsonly | 4 | 0.393 | 0.135 | ++ | 4.204 | 2.305 | 7.666 |
| **Unexposed group** |  |  |  |  |  |  |  |
| nev any | 69 | Aliased |  |  | 2.981 | 2.309 | 3.849 |
| nev cig | 44 | -0.118 | 0.130 | N.S. | 2.650 | 2.007 | 3.499 |
| **CB subtype** |  |  |  |  |  |  |  |
| mort | 4 | Aliased |  |  | 3.436 | 1.366 | 8.645 |
| sympt | 81 | -0.126 | 0.232 | N.S. | 3.028 | 2.804 | 3.270 |
| other | 28 | -0.396 | 0.232 | (-) | 2.314 | 2.013 | 2.659 |
| **RR adjusted for age** |  |  |  |  |  |  |  |
| Yes | 65 | Aliased |  |  | 2.616 | 2.412 | 2.838 |
| No | 48 | 0.211 | 0.038 | +++ | 3.229 | 2.875 | 3.628 |
|  |  | **Deviance** | **(DF)** | **Drop Dev** | **P** |  |  |
| **Omitting RR adjusted for age** |  | 439.945 | (98) | -32.247 | ** |  |  |
|  |  | **Estimate** | **S.E.** | **P** | **RR** | **95%CIl** | **95%CIu** |
| **Constant** |  | 1.387 | 0.258 | +++ | 4.001 | 2.414 | 6.632 |
| **RR adjusted for factor other than sex, age** |  |  |  |  |  |  |  |
| Yes | 29 | Aliased |  |  | 2.886 | 2.473 | 3.368 |
| No | 84 | -0.048 | 0.072 | N.S. | 2.750 | 2.352 | 3.216 |
| **Sex(RR)** |  |  |  |  |  |  |  |
| Male | 51 | Aliased |  |  | 2.976 | 2.479 | 3.574 |
| Female | 37 | -0.107 | 0.043 | - | 2.673 | 2.244 | 3.184 |
| Combined | 25 | -0.050 | 0.081 | N.S. | 2.832 | 2.378 | 3.372 |
| **Continent** |  |  |  |  |  |  |  |
| NAmer | 40 | Aliased |  |  | 3.596 | 3.039 | 4.256 |
| Europe | 58 | -0.275 | 0.050 | --- | 2.732 | 2.518 | 2.964 |
| Asia | 9 | -0.516 | 0.070 | --- | 2.148 | 1.669 | 2.764 |
| oth/mult | 6 | -0.199 | 0.085 | - | 2.946 | 2.201 | 3.943 |
| **Asthma analysis type (CB)** |  |  |  |  |  |  |  |
| inc-irresp | 103 | Aliased |  |  | 2.778 | 2.606 | 2.961 |
| excl-all | 6 | 0.146 | 0.076 | (+) | 3.214 | 2.406 | 4.294 |
| defn-incl | 0 | Aliased |  |  | 2.778 | 2.606 | 2.961 |
| other | 4 | 1.191 | 0.237 | +++ | 9.139 | 3.561 | 23.456 |
| **Smoking product (3)** |  |  |  |  |  |  |  |
| any | 57 | Aliased |  |  | 2.502 | 1.922 | 3.257 |
| cigs | 52 | 0.227 | 0.129 | (+) | 3.141 | 2.405 | 4.102 |
| cigsonly | 4 | 0.412 | 0.136 | ++ | 3.779 | 2.083 | 6.854 |
| **Unexposed group** |  |  |  |  |  |  |  |
| nev any | 69 | Aliased |  |  | 3.260 | 2.543 | 4.181 |
| nev cig | 44 | -0.305 | 0.126 | - | 2.403 | 1.834 | 3.148 |
| **CB subtype** |  |  |  |  |  |  |  |
| mort | 4 | Aliased |  |  | 3.199 | 1.272 | 8.043 |
| sympt | 81 | -0.031 | 0.232 | N.S. | 3.101 | 2.868 | 3.354 |
| other | 28 | -0.387 | 0.232 | (-) | 2.173 | 1.880 | 2.511 |
| **Midpoint age in RR** |  | 0.002 | 0.002 | N.S. | 2.526 | 1.821 | 3.504 |

Table 2 - B - 6

IESCOPD - Meta-regression of current smoking, any product (or cigarettes if all product not available)

Multiple regression of data from Table 2 - B - 1 (most-adjusted RRs)

Any CB

Test by removing variables one at a time

|  |  | **Deviance** | **(DF)** | **Drop Dev** | **P** |  |  |
| --- | --- | --- | --- | --- | --- | --- | --- |
| **Omitting RR adjusted for factor other than sex, age** |  | 407.706 | (98) | -0.009 | N.S. |  |  |
|  |  | **Estimate** | **S.E.** | **P** | **RR** | **95%CIl** | **95%CIu** |
| **Constant** |  | 1.329 | 0.248 | +++ | 3.776 | 2.324 | 6.135 |
| **RR adjusted for age** |  |  |  |  |  |  |  |
| Yes | 65 | Aliased |  |  | 2.608 | 2.404 | 2.829 |
| No | 48 | 0.220 | 0.038 | +++ | 3.248 | 2.891 | 3.649 |
| **Sex(RR)** |  |  |  |  |  |  |  |
| Male | 51 | Aliased |  |  | 2.838 | 2.483 | 3.244 |
| Female | 37 | -0.097 | 0.043 | - | 2.575 | 2.269 | 2.923 |
| Combined | 25 | 0.045 | 0.046 | N.S. | 2.969 | 2.670 | 3.302 |
| **Continent** |  |  |  |  |  |  |  |
| NAmer | 40 | Aliased |  |  | 3.775 | 3.184 | 4.475 |
| Europe | 58 | -0.354 | 0.051 | --- | 2.650 | 2.438 | 2.879 |
| Asia | 9 | -0.493 | 0.070 | --- | 2.306 | 1.785 | 2.978 |
| oth/mult | 6 | -0.143 | 0.086 | (-) | 3.272 | 2.433 | 4.399 |
| **Asthma analysis type (CB)** |  |  |  |  |  |  |  |
| inc-irresp | 103 | Aliased |  |  | 2.775 | 2.604 | 2.958 |
| excl-all | 6 | 0.158 | 0.076 | + | 3.252 | 2.435 | 4.342 |
| defn-incl | 0 | Aliased |  |  | 2.775 | 2.604 | 2.958 |
| other | 4 | 1.224 | 0.237 | +++ | 9.441 | 3.678 | 24.233 |
| **Smoking product (3)** |  |  |  |  |  |  |  |
| any | 57 | Aliased |  |  | 2.835 | 2.151 | 3.737 |
| cigs | 52 | -0.024 | 0.136 | N.S. | 2.768 | 2.093 | 3.661 |
| cigsonly | 4 | 0.410 | 0.136 | ++ | 4.273 | 2.342 | 7.795 |
| **Unexposed group** |  |  |  |  |  |  |  |
| nev any | 69 | Aliased |  |  | 2.992 | 2.320 | 3.859 |
| nev cig | 44 | -0.125 | 0.129 | N.S. | 2.639 | 2.001 | 3.481 |
| **CB subtype** |  |  |  |  |  |  |  |
| mort | 4 | Aliased |  |  | 3.388 | 1.347 | 8.523 |
| sympt | 81 | -0.103 | 0.232 | N.S. | 3.056 | 2.824 | 3.307 |
| other | 28 | -0.405 | 0.232 | (-) | 2.259 | 1.950 | 2.617 |
| **Midpoint age in RR** |  | 0.003 | 0.002 | + | 2.398 | 1.725 | 3.333 |

Table 2 - B - 6

IESCOPD - Meta-regression of current smoking, any product (or cigarettes if all product not available)

Multiple regression of data from Table 2 - B - 1 (most-adjusted RRs)

Any CB

Test reduction to 2-level product

|  |  | **Deviance** | **(DF)** | **Drop Dev** | **P** |  |  |
| --- | --- | --- | --- | --- | --- | --- | --- |
| **Reducing Smoking Product to 2 levels** |  | 412.779 | (98) | -5.082 | N.S. |  |  |
|  |  | **Estimate** | **S.E.** | **P** | **RR** | **95%CIl** | **95%CIu** |
| **Constant** |  | 1.351 | 0.258 | +++ | 3.863 | 2.330 | 6.403 |
| **Sex(RR)** |  |  |  |  |  |  |  |
| Male | 51 | Aliased |  |  | 2.888 | 2.403 | 3.471 |
| Female | 37 | -0.096 | 0.043 | - | 2.624 | 2.204 | 3.124 |
| Combined | 25 | 0.007 | 0.081 | N.S. | 2.909 | 2.443 | 3.465 |
| **Continent** |  |  |  |  |  |  |  |
| NAmer | 40 | Aliased |  |  | 3.719 | 3.138 | 4.407 |
| Europe | 58 | -0.327 | 0.051 | --- | 2.681 | 2.470 | 2.909 |
| Asia | 9 | -0.504 | 0.070 | --- | 2.248 | 1.745 | 2.895 |
| oth/mult | 6 | -0.180 | 0.085 | - | 3.107 | 2.332 | 4.139 |
| **Asthma analysis type (CB)** |  |  |  |  |  |  |  |
| inc-irresp | 103 | Aliased |  |  | 2.779 | 2.608 | 2.963 |
| excl-all | 6 | 0.137 | 0.075 | (+) | 3.188 | 2.392 | 4.250 |
| defn-incl | 0 | Aliased |  |  | 2.779 | 2.608 | 2.963 |
| other | 4 | 1.199 | 0.236 | +++ | 9.220 | 3.595 | 23.647 |
| **Smoking product (2)** |  |  |  |  |  |  |  |
| any | 57 | Aliased |  |  | 2.551 | 2.076 | 3.134 |
| cigs | 56 | 0.195 | 0.096 | + | 3.100 | 2.543 | 3.779 |
| **Unexposed group** |  |  |  |  |  |  |  |
| nev any | 69 | Aliased |  |  | 3.286 | 2.704 | 3.994 |
| nev cig | 44 | -0.322 | 0.097 | -- | 2.382 | 1.928 | 2.943 |
| **CB subtype** |  |  |  |  |  |  |  |
| mort | 4 | Aliased |  |  | 3.414 | 1.357 | 8.591 |
| sympt | 81 | -0.115 | 0.232 | N.S. | 3.042 | 2.812 | 3.291 |
| other | 28 | -0.401 | 0.232 | (-) | 2.286 | 1.976 | 2.645 |
| **Midpoint age in RR** |  | 0.003 | 0.002 | (+) | 2.415 | 1.738 | 3.357 |
| **RR adjusted for age** |  |  |  |  |  |  |  |
| Yes | 65 | Aliased |  |  | 2.626 | 2.423 | 2.847 |
| No | 48 | 0.199 | 0.038 | +++ | 3.206 | 2.859 | 3.596 |
| **RR adjusted for factor other than sex, age** |  |  |  |  |  |  |  |
| Yes | 29 | Aliased |  |  | 2.843 | 2.435 | 3.319 |
| No | 84 | -0.018 | 0.072 | N.S. | 2.793 | 2.387 | 3.267 |

Table 2 - B - 6

IESCOPD - Meta-regression of current smoking, any product (or cigarettes if all product not available)

Multiple regression of data from Table 2 - B - 1 (most-adjusted RRs)

Any CB

Test reduction to 2-level outcome subtype

|  |  | **Deviance** | **(DF)** | **Drop Dev** | **P** |  |  |
| --- | --- | --- | --- | --- | --- | --- | --- |
| **Reducing CB subtype to 2 levels** |  | 410.755 | (98) | -3.057 | N.S. |  |  |
|  |  | **Estimate** | **S.E.** | **P** | **RR** | **95%CIl** | **95%CIu** |
| **Constant** |  | 1.245 | 0.120 | +++ | 3.474 | 2.745 | 4.395 |
| **Sex(RR)** |  |  |  |  |  |  |  |
| Male | 51 | Aliased |  |  | 2.860 | 2.376 | 3.442 |
| Female | 37 | -0.106 | 0.042 | - | 2.573 | 2.157 | 3.069 |
| Combined | 25 | 0.034 | 0.082 | N.S. | 2.960 | 2.479 | 3.534 |
| **Continent** |  |  |  |  |  |  |  |
| NAmer | 40 | Aliased |  |  | 3.798 | 3.201 | 4.507 |
| Europe | 58 | -0.361 | 0.052 | --- | 2.647 | 2.433 | 2.879 |
| Asia | 9 | -0.500 | 0.070 | --- | 2.305 | 1.782 | 2.980 |
| oth/mult | 6 | -0.153 | 0.086 | (-) | 3.259 | 2.413 | 4.400 |
| **Asthma analysis type (CB)** |  |  |  |  |  |  |  |
| inc-irresp | 103 | Aliased |  |  | 2.777 | 2.605 | 2.960 |
| excl-all | 6 | 0.151 | 0.076 | + | 3.230 | 2.419 | 4.313 |
| defn-incl | 0 | Aliased |  |  | 2.777 | 2.605 | 2.960 |
| other | 4 | 1.217 | 0.237 | +++ | 9.374 | 3.652 | 24.063 |
| **Smoking product (3)** |  |  |  |  |  |  |  |
| any | 57 | Aliased |  |  | 2.813 | 2.132 | 3.712 |
| cigs | 52 | -0.009 | 0.136 | N.S. | 2.789 | 2.106 | 3.693 |
| cigsonly | 4 | 0.421 | 0.136 | ++ | 4.287 | 2.349 | 7.823 |
| **Unexposed group** |  |  |  |  |  |  |  |
| nev any | 69 | Aliased |  |  | 3.016 | 2.336 | 3.892 |
| nev cig | 44 | -0.142 | 0.130 | N.S. | 2.617 | 1.983 | 3.454 |
| **CB subtype (2)** |  |  |  |  |  |  |  |
| sympt | 81 | Aliased |  |  | 3.055 | 2.823 | 3.306 |
| other | 32 | -0.295 | 0.046 | --- | 2.276 | 1.966 | 2.634 |
| **Midpoint age in RR** |  | 0.003 | 0.002 | (+) | 2.406 | 1.731 | 3.345 |
| **RR adjusted for age** |  |  |  |  |  |  |  |
| Yes | 65 | Aliased |  |  | 2.609 | 2.405 | 2.831 |
| No | 48 | 0.218 | 0.039 | +++ | 3.245 | 2.887 | 3.648 |
| **RR adjusted for factor other than sex, age** |  |  |  |  |  |  |  |
| Yes | 29 | Aliased |  |  | 2.823 | 2.418 | 3.297 |
| No | 84 | -0.004 | 0.072 | N.S. | 2.813 | 2.403 | 3.291 |

Table 2 - B - 6

IESCOPD - Meta-regression of current smoking, any product (or cigarettes if all product not available)

Multiple regression of data from Table 2 - B - 1 (most-adjusted RRs)

Any CB

Test by adding extra variables one at a time

|  |  | **Deviance** | **(DF)** | **Drop Dev** | **P** |  |  |
| --- | --- | --- | --- | --- | --- | --- | --- |
| **Increasing CB subtype to 4 levels** |  | 407.196 | (96) | 0.501 | N.S. |  |  |
|  |  | **Estimate** | **S.E.** | **P** | **RR** | **95%CIl** | **95%CIu** |
| **Constant** |  | 1.327 | 0.258 | +++ | 3.771 | 2.274 | 6.256 |
| **Sex(RR)** |  |  |  |  |  |  |  |
| Male | 51 | Aliased |  |  | 2.841 | 2.354 | 3.428 |
| Female | 37 | -0.098 | 0.043 | - | 2.575 | 2.152 | 3.080 |
| Combined | 25 | 0.044 | 0.083 | N.S. | 2.969 | 2.480 | 3.554 |
| **Continent** |  |  |  |  |  |  |  |
| NAmer | 40 | Aliased |  |  | 3.763 | 3.161 | 4.478 |
| Europe | 58 | -0.351 | 0.052 | --- | 2.649 | 2.434 | 2.885 |
| Asia | 9 | -0.482 | 0.072 | --- | 2.323 | 1.785 | 3.025 |
| oth/mult | 6 | -0.141 | 0.086 | N.S. | 3.269 | 2.414 | 4.429 |
| **Asthma analysis type (CB)** |  |  |  |  |  |  |  |
| inc-irresp | 103 | Aliased |  |  | 2.786 | 2.603 | 2.982 |
| excl-all | 6 | 0.098 | 0.114 | N.S. | 3.073 | 1.985 | 4.757 |
| defn-incl | 0 | Aliased |  |  | 2.786 | 2.603 | 2.982 |
| other | 4 | 1.236 | 0.237 | +++ | 9.591 | 3.687 | 24.950 |
| **Smoking product (3)** |  |  |  |  |  |  |  |
| any | 57 | Aliased |  |  | 2.844 | 2.147 | 3.768 |
| cigs | 52 | -0.030 | 0.137 | N.S. | 2.759 | 2.075 | 3.668 |
| cigsonly | 4 | 0.421 | 0.137 | ++ | 4.332 | 2.347 | 7.997 |
| **Unexposed group** |  |  |  |  |  |  |  |
| nev any | 69 | Aliased |  |  | 2.990 | 2.309 | 3.871 |
| nev cig | 44 | -0.124 | 0.130 | N.S. | 2.641 | 1.994 | 3.498 |
| **CB subtype** |  |  |  |  |  |  |  |
| mort | 4 | Aliased |  |  |  |  |  |
| sympt | 81 | Aliased |  |  |  |  |  |
| other | 28 | Aliased |  |  |  |  |  |
|  |  | **Estimate** | **S.E.** | **P** | **RR** | **95%CIl** | **95%CIu** |
| **Midpoint age in RR** |  | 0.003 | 0.002 | + | 2.382 | 1.704 | 3.330 |
| **RR adjusted for age** |  |  |  |  |  |  |  |
| Yes | 65 | Aliased |  |  | 2.604 | 2.396 | 2.829 |
| No | 48 | 0.224 | 0.039 | +++ | 3.258 | 2.891 | 3.672 |
| **RR adjusted for factor other than sex, age** |  |  |  |  |  |  |  |
| Yes | 29 | Aliased |  |  | 2.830 | 2.420 | 3.311 |
| No | 84 | -0.009 | 0.072 | N.S. | 2.805 | 2.393 | 3.288 |
| **CB subtype (4)** |  |  |  |  |  |  |  |
| mort | 4 | Aliased |  |  | 3.385 | 1.333 | 8.595 |
| sympt | 81 | -0.104 | 0.232 | N.S. | 3.050 | 2.814 | 3.305 |
| oth-prev | 24 | -0.417 | 0.233 | (-) | 2.230 | 1.888 | 2.633 |
| oth-inc | 4 | -0.317 | 0.264 | N.S. | 2.466 | 1.464 | 4.152 |

Table 2 - B - 6

IESCOPD - Meta-regression of current smoking, any product (or cigarettes if all product not available)

Multiple regression of data from Table 2 - B - 1 (most-adjusted RRs)

Any CB

Test by adding extra variables one at a time

|  |  | **Deviance** | **(DF)** | **Drop Dev** | **P** |  |  |
| --- | --- | --- | --- | --- | --- | --- | --- |
| **Adding National cigarette tobacco type** |  | 403.609 | (95) | 4.089 | N.S. |  |  |
|  |  | **Estimate** | **S.E.** | **P** | **RR** | **95%CIl** | **95%CIu** |
| **Constant** |  | 1.319 | 0.259 | +++ | 3.738 | 2.249 | 6.215 |
| **Sex(RR)** |  |  |  |  |  |  |  |
| Male | 51 | Aliased |  |  | 2.826 | 2.331 | 3.426 |
| Female | 37 | -0.109 | 0.043 | - | 2.534 | 2.097 | 3.062 |
| Combined | 25 | 0.062 | 0.086 | N.S. | 3.006 | 2.488 | 3.633 |
| **Continent** |  |  |  |  |  |  |  |
| NAmer | 40 | Aliased |  |  | 3.854 | 3.221 | 4.611 |
| Europe | 58 | -0.372 | 0.053 | --- | 2.658 | 2.434 | 2.902 |
| Asia | 9 | -0.518 | 0.078 | --- | 2.295 | 1.707 | 3.086 |
| oth/mult | 6 | -0.251 | 0.101 | - | 2.998 | 2.115 | 4.251 |
| **Asthma analysis type (CB)** |  |  |  |  |  |  |  |
| inc-irresp | 103 | Aliased |  |  | 2.775 | 2.601 | 2.961 |
| excl-all | 6 | 0.158 | 0.078 | + | 3.252 | 2.410 | 4.388 |
| defn-incl | 0 | Aliased |  |  | 2.775 | 2.601 | 2.961 |
| other | 4 | 1.232 | 0.238 | +++ | 9.515 | 3.658 | 24.755 |
| **Smoking product (3)** |  |  |  |  |  |  |  |
| any | 57 | Aliased |  |  | 2.798 | 1.988 | 3.940 |
| cigs | 52 | 0.005 | 0.168 | N.S. | 2.813 | 1.990 | 3.976 |
| cigsonly | 4 | 0.307 | 0.146 | + | 3.806 | 1.958 | 7.397 |
| **Unexposed group** |  |  |  |  |  |  |  |
| nev any | 69 | Aliased |  |  | 3.039 | 2.188 | 4.221 |
| nev cig | 44 | -0.158 | 0.167 | N.S. | 2.595 | 1.814 | 3.712 |
| **CB subtype** |  |  |  |  |  |  |  |
| mort | 4 | Aliased |  |  | 3.313 | 1.302 | 8.430 |
| sympt | 81 | -0.086 | 0.233 | N.S. | 3.042 | 2.800 | 3.304 |
| other | 28 | -0.370 | 0.233 | N.S. | 2.288 | 1.949 | 2.687 |
| **Midpoint age in RR** |  | 0.003 | 0.002 | (+) | 2.434 | 1.730 | 3.426 |
| **RR adjusted for age** |  |  |  |  |  |  |  |
| Yes | 65 | Aliased |  |  | 2.606 | 2.400 | 2.830 |
| No | 48 | 0.222 | 0.039 | +++ | 3.252 | 2.890 | 3.660 |
| **RR adjusted for factor other than sex, age** |  |  |  |  |  |  |  |
| Yes | 29 | Aliased |  |  | 2.810 | 2.400 | 3.290 |
| No | 84 | 0.005 | 0.072 | N.S. | 2.826 | 2.408 | 3.315 |
| **National cigarette tobacco type** |  |  |  |  |  |  |  |
| bl | 77 | Aliased |  |  | 2.766 | 2.571 | 2.976 |
| vir | 33 | 0.114 | 0.057 | + | 3.100 | 2.528 | 3.801 |
| m/u | 3 | 0.054 | 0.131 | N.S. | 2.919 | 1.751 | 4.866 |

Table 2 - B - 6

IESCOPD - Meta-regression of current smoking, any product (or cigarettes if all product not available)

Multiple regression of data from Table 2 - B - 1 (most-adjusted RRs)

Any CB

Test by adding extra variables one at a time

|  |  | **Deviance** | **(DF)** | **Drop Dev** | **P** |  |  |
| --- | --- | --- | --- | --- | --- | --- | --- |
| **Adding Publication year** |  | 378.676 | (94) | 29.021 | (*) |  |  |
|  |  | **Estimate** | **S.E.** | **P** | **RR** | **95%CIl** | **95%CIu** |
| **Constant** |  | 1.511 | 0.260 | +++ | 4.529 | 2.719 | 7.543 |
| **Sex(RR)** |  |  |  |  |  |  |  |
| Male | 51 | Aliased |  |  | 2.775 | 2.308 | 3.336 |
| Female | 37 | -0.099 | 0.043 | - | 2.512 | 2.107 | 2.997 |
| Combined | 25 | 0.095 | 0.083 | N.S. | 3.050 | 2.555 | 3.642 |
| **Continent** |  |  |  |  |  |  |  |
| NAmer | 40 | Aliased |  |  | 3.614 | 2.994 | 4.362 |
| Europe | 58 | -0.309 | 0.059 | --- | 2.652 | 2.428 | 2.897 |
| Asia | 9 | -0.394 | 0.084 | --- | 2.437 | 1.783 | 3.331 |
| oth/mult | 6 | -0.066 | 0.093 | N.S. | 3.385 | 2.504 | 4.576 |
| **Asthma analysis type (CB)** |  |  |  |  |  |  |  |
| inc-irresp | 103 | Aliased |  |  | 2.751 | 2.582 | 2.930 |
| excl-all | 6 | 0.293 | 0.080 | +++ | 3.688 | 2.727 | 4.988 |
| defn-incl | 0 | Aliased |  |  | 2.751 | 2.582 | 2.930 |
| other | 4 | 1.346 | 0.240 | +++ | 10.568 | 4.135 | 27.010 |
| **Smoking product (3)** |  |  |  |  |  |  |  |
| any | 57 | Aliased |  |  | 2.942 | 2.226 | 3.888 |
| cigs | 52 | -0.097 | 0.139 | N.S. | 2.670 | 2.014 | 3.541 |
| cigsonly | 4 | 0.359 | 0.137 | + | 4.212 | 2.321 | 7.645 |
| **Unexposed group** |  |  |  |  |  |  |  |
| nev any | 69 | Aliased |  |  | 2.955 | 2.294 | 3.807 |
| nev cig | 44 | -0.100 | 0.131 | N.S. | 2.675 | 2.031 | 3.523 |
| **CB subtype** |  |  |  |  |  |  |  |
| mort | 4 | Aliased |  |  | 3.340 | 1.337 | 8.339 |
| sympt | 81 | -0.095 | 0.233 | N.S. | 3.037 | 2.786 | 3.310 |
| other | 28 | -0.374 | 0.238 | N.S. | 2.298 | 1.924 | 2.744 |
| **Midpoint age in RR** |  | 0.001 | 0.002 | N.S. | 2.663 | 1.906 | 3.720 |
| **RR adjusted for age** |  |  |  |  |  |  |  |
| Yes | 65 | Aliased |  |  | 2.578 | 2.376 | 2.797 |
| No | 48 | 0.252 | 0.040 | +++ | 3.317 | 2.949 | 3.732 |
| **RR adjusted for factor other than sex, age** |  |  |  |  |  |  |  |
| Yes | 29 | Aliased |  |  | 2.793 | 2.395 | 3.257 |
| No | 84 | 0.018 | 0.073 | N.S. | 2.843 | 2.433 | 3.322 |
| **Publication year** |  |  |  |  |  |  |  |
| <1980 | 50 | Aliased |  |  | 3.308 | 2.787 | 3.927 |
| 1980-89 | 26 | -0.139 | 0.076 | (-) | 2.877 | 2.352 | 3.520 |
| 1990-99 | 17 | -0.299 | 0.058 | --- | 2.454 | 2.112 | 2.851 |
| 2000+ | 20 | -0.188 | 0.056 | -- | 2.742 | 2.393 | 3.143 |
|  |  | **Deviance** | **(DF)** | **Drop Dev** | **P** |  |  |
| **Adding Study type** |  | 403.776 | (95) | 3.921 | N.S. |  |  |
|  |  | **Estimate** | **S.E.** | **P** | **RR** | **95%CIl** | **95%CIu** |
| **Constant** |  | 1.117 | 0.296 | +++ | 3.057 | 1.711 | 5.461 |
| **Sex(RR)** |  |  |  |  |  |  |  |
| Male | 51 | Aliased |  |  | 2.824 | 2.339 | 3.410 |
| Female | 37 | -0.102 | 0.043 | - | 2.550 | 2.128 | 3.055 |
| Combined | 25 | 0.059 | 0.083 | N.S. | 2.996 | 2.500 | 3.590 |
| **Continent** |  |  |  |  |  |  |  |
| NAmer | 40 | Aliased |  |  | 3.794 | 3.181 | 4.525 |
| Europe | 58 | -0.364 | 0.053 | --- | 2.635 | 2.417 | 2.874 |
| Asia | 9 | -0.464 | 0.071 | --- | 2.386 | 1.819 | 3.130 |
| oth/mult | 6 | -0.150 | 0.086 | (-) | 3.264 | 2.409 | 4.422 |
| **Asthma analysis type (CB)** |  |  |  |  |  |  |  |
| inc-irresp | 103 | Aliased |  |  | 2.781 | 2.600 | 2.975 |
| excl-all | 6 | 0.116 | 0.103 | N.S. | 3.125 | 2.106 | 4.636 |
| defn-incl | 0 | Aliased |  |  | 2.781 | 2.600 | 2.975 |
| other | 4 | 1.336 | 0.269 | +++ | 10.584 | 3.600 | 31.111 |

Table 2 - B - 6

IESCOPD - Meta-regression of current smoking, any product (or cigarettes if all product not available)

Multiple regression of data from Table 2 - B - 1 (most-adjusted RRs)

Any CB

Test by adding extra variables one at a time

|  |  | **Estimate** | **S.E.** | **P** | **RR** | **95%CIl** | **95%CIu** |
| --- | --- | --- | --- | --- | --- | --- | --- |
| **Smoking product (3)** |  |  |  |  |  |  |  |
| any | 57 | Aliased |  |  | 2.874 | 2.167 | 3.814 |
| cigs | 52 | -0.054 | 0.137 | N.S. | 2.722 | 2.044 | 3.625 |
| cigsonly | 4 | 0.504 | 0.168 | ++ | 4.756 | 2.295 | 9.858 |
| **Unexposed group** |  |  |  |  |  |  |  |
| nev any | 69 | Aliased |  |  | 2.991 | 2.310 | 3.874 |
| nev cig | 44 | -0.125 | 0.130 | N.S. | 2.640 | 1.993 | 3.497 |
| **CB subtype** |  |  |  |  |  |  |  |
| mort | 4 | Aliased |  |  | 2.933 | 1.080 | 7.963 |
| sympt | 81 | 0.048 | 0.250 | N.S. | 3.078 | 2.838 | 3.339 |
| other | 28 | -0.278 | 0.247 | N.S. | 2.220 | 1.904 | 2.589 |
| **Midpoint age in RR** |  | 0.003 | 0.002 | (+) | 2.399 | 1.707 | 3.371 |
| **RR adjusted for age** |  |  |  |  |  |  |  |
| Yes | 65 | Aliased |  |  | 2.603 | 2.396 | 2.828 |
| No | 48 | 0.224 | 0.039 | +++ | 3.258 | 2.892 | 3.671 |
| **RR adjusted for factor other than sex, age** |  |  |  |  |  |  |  |
| Yes | 29 | Aliased |  |  | 2.841 | 2.428 | 3.324 |
| No | 84 | -0.016 | 0.072 | N.S. | 2.795 | 2.384 | 3.277 |
| **Study type** |  |  |  |  |  |  |  |
| CC | 9 | Aliased |  |  | 2.577 | 1.610 | 4.123 |
| Pr | 12 | 0.220 | 0.124 | (+) | 3.210 | 2.288 | 4.503 |
| CS | 92 | 0.081 | 0.122 | N.S. | 2.795 | 2.598 | 3.007 |
|  |  | **Deviance** | **(DF)** | **Drop Dev** | **P** |  |  |
| **Adding Study weakness** |  | 404.503 | (96) | 3.195 | N.S. |  |  |
|  |  | **Estimate** | **S.E.** | **P** | **RR** | **95%CIl** | **95%CIu** |
| **Constant** |  | 1.808 | 0.369 | +++ | 6.095 | 2.956 | 12.568 |
| **Sex(RR)** |  |  |  |  |  |  |  |
| Male | 51 | Aliased |  |  | 2.835 | 2.352 | 3.418 |
| Female | 37 | -0.092 | 0.043 | - | 2.586 | 2.165 | 3.090 |
| Combined | 25 | 0.044 | 0.082 | N.S. | 2.963 | 2.479 | 3.542 |
| **Continent** |  |  |  |  |  |  |  |
| NAmer | 40 | Aliased |  |  | 3.774 | 3.175 | 4.486 |
| Europe | 58 | -0.354 | 0.052 | --- | 2.649 | 2.434 | 2.883 |
| Asia | 9 | -0.489 | 0.070 | --- | 2.314 | 1.786 | 2.998 |
| oth/mult | 6 | -0.145 | 0.086 | (-) | 3.266 | 2.414 | 4.419 |
| **Asthma analysis type (CB)** |  |  |  |  |  |  |  |
| inc-irresp | 103 | Aliased |  |  | 2.782 | 2.608 | 2.968 |
| excl-all | 6 | 0.150 | 0.076 | (+) | 3.233 | 2.414 | 4.329 |
| defn-incl | 0 | Aliased |  |  | 2.782 | 2.608 | 2.968 |
| other | 4 | 0.760 | 0.352 | + | 5.949 | 1.455 | 24.326 |
| **Smoking product (3)** |  |  |  |  |  |  |  |
| any | 57 | Aliased |  |  | 2.836 | 2.145 | 3.751 |
| cigs | 52 | -0.025 | 0.136 | N.S. | 2.767 | 2.085 | 3.672 |
| cigsonly | 4 | 0.413 | 0.136 | ++ | 4.288 | 2.340 | 7.857 |
| **Unexposed group** |  |  |  |  |  |  |  |
| nev any | 69 | Aliased |  |  | 2.996 | 2.316 | 3.876 |
| nev cig | 44 | -0.128 | 0.130 | N.S. | 2.635 | 1.992 | 3.486 |
| **CB subtype** |  |  |  |  |  |  |  |
| mort | 4 | Aliased |  |  | 3.403 | 1.344 | 8.616 |
| sympt | 81 | -0.107 | 0.232 | N.S. | 3.057 | 2.824 | 3.310 |
| other | 28 | -0.411 | 0.232 | (-) | 2.256 | 1.945 | 2.616 |
| **Midpoint age in RR** |  | 0.003 | 0.002 | (+) | 2.404 | 1.725 | 3.349 |
| **RR adjusted for age** |  |  |  |  |  |  |  |
| Yes | 65 | Aliased |  |  | 2.609 | 2.403 | 2.832 |
| No | 48 | 0.219 | 0.039 | +++ | 3.246 | 2.886 | 3.652 |
| **RR adjusted for factor other than sex, age** |  |  |  |  |  |  |  |
| Yes | 29 | Aliased |  |  | 2.830 | 2.421 | 3.308 |
| No | 84 | -0.008 | 0.072 | N.S. | 2.806 | 2.395 | 3.287 |

Table 2 - B - 6

IESCOPD - Meta-regression of current smoking, any product (or cigarettes if all product not available)

Multiple regression of data from Table 2 - B - 1 (most-adjusted RRs)

Any CB

Test by adding extra variables one at a time

|  |  | **Estimate** | **S.E.** | **P** | **RR** | **95%CIl** | **95%CIu** |
| --- | --- | --- | --- | --- | --- | --- | --- |
| **Study weakness** |  |  |  |  |  |  |  |
| Yes | 7 | Aliased |  |  | 4.483 | 1.574 | 12.772 |
| No | 106 | -0.468 | 0.262 | (-) | 2.808 | 2.640 | 2.986 |
|  |  | **Deviance** | **(DF)** | **Drop Dev** | **P** |  |  |
| **Adding Number of cases (CB)** |  | 377.346 | (94) | 30.352 | (*) |  |  |
|  |  | **Estimate** | **S.E.** | **P** | **RR** | **95%CIl** | **95%CIu** |
| **Constant** |  | 1.236 | 0.300 | +++ | 3.443 | 1.912 | 6.199 |
| **Sex(RR)** |  |  |  |  |  |  |  |
| Male | 51 | Aliased |  |  | 2.953 | 2.453 | 3.554 |
| Female | 37 | -0.081 | 0.043 | (-) | 2.723 | 2.277 | 3.256 |
| Combined | 25 | -0.049 | 0.084 | N.S. | 2.811 | 2.349 | 3.364 |
| **Continent** |  |  |  |  |  |  |  |
| NAmer | 40 | Aliased |  |  | 3.516 | 2.944 | 4.199 |
| Europe | 58 | -0.261 | 0.055 | --- | 2.709 | 2.489 | 2.947 |
| Asia | 9 | -0.427 | 0.071 | --- | 2.293 | 1.781 | 2.952 |
| oth/mult | 6 | -0.109 | 0.086 | N.S. | 3.153 | 2.341 | 4.246 |
| **Asthma analysis type (CB)** |  |  |  |  |  |  |  |
| inc-irresp | 103 | Aliased |  |  | 2.771 | 2.602 | 2.950 |
| excl-all | 6 | 0.185 | 0.076 | + | 3.333 | 2.505 | 4.435 |
| defn-incl | 0 | Aliased |  |  | 2.771 | 2.602 | 2.950 |
| other | 4 | 1.236 | 0.237 | +++ | 9.535 | 3.772 | 24.098 |
| **Smoking product (3)** |  |  |  |  |  |  |  |
| any | 57 | Aliased |  |  | 2.747 | 2.077 | 3.633 |
| cigs | 52 | 0.039 | 0.140 | N.S. | 2.857 | 2.152 | 3.792 |
| cigsonly | 4 | 0.426 | 0.136 | ++ | 4.205 | 2.321 | 7.619 |
| **Unexposed group** |  |  |  |  |  |  |  |
| nev any | 69 | Aliased |  |  | 3.055 | 2.362 | 3.951 |
| nev cig | 44 | -0.169 | 0.133 | N.S. | 2.580 | 1.950 | 3.413 |
| **CB subtype** |  |  |  |  |  |  |  |
| mort | 4 | Aliased |  |  | 2.608 | 1.026 | 6.631 |
| sympt | 81 | 0.166 | 0.239 | N.S. | 3.080 | 2.849 | 3.329 |
| other | 28 | -0.160 | 0.238 | N.S. | 2.222 | 1.921 | 2.570 |
| **Midpoint age in RR** |  | 0.002 | 0.002 | N.S. | 2.506 | 1.810 | 3.469 |
| **RR adjusted for age** |  |  |  |  |  |  |  |
| Yes | 65 | Aliased |  |  | 2.644 | 2.439 | 2.867 |
| No | 48 | 0.180 | 0.039 | +++ | 3.166 | 2.817 | 3.558 |
| **RR adjusted for factor other than sex, age** |  |  |  |  |  |  |  |
| Yes | 29 | Aliased |  |  | 2.942 | 2.517 | 3.439 |
| No | 84 | -0.087 | 0.074 | N.S. | 2.697 | 2.302 | 3.159 |
| **Number of cases (CB)** |  |  |  |  |  |  |  |
| 1-50 | 18 | Aliased |  |  | 3.150 | 1.630 | 6.087 |
| 51-100 | 14 | 0.397 | 0.210 | (+) | 4.683 | 2.823 | 7.767 |
| 101-200 | 19 | 0.199 | 0.183 | N.S. | 3.842 | 2.807 | 5.259 |
| 201+ | 62 | -0.135 | 0.169 | N.S. | 2.752 | 2.585 | 2.929 |
|  |  | **Deviance** | **(DF)** | **Drop Dev** | **P** |  |  |
| **Adding Smoking results reported in study (CB)** |  | 401.089 | (96) | 6.608 | N.S. |  |  |
|  |  | **Estimate** | **S.E.** | **P** | **RR** | **95%CIl** | **95%CIu** |
| **Constant** |  | 1.021 | 0.285 | +++ | 2.776 | 1.586 | 4.857 |
| **Sex(RR)** |  |  |  |  |  |  |  |
| Male | 51 | Aliased |  |  | 2.804 | 2.325 | 3.382 |
| Female | 37 | -0.099 | 0.043 | - | 2.539 | 2.123 | 3.037 |
| Combined | 25 | 0.072 | 0.083 | N.S. | 3.014 | 2.517 | 3.609 |
| **Continent** |  |  |  |  |  |  |  |
| NAmer | 40 | Aliased |  |  | 3.854 | 3.234 | 4.592 |
| Europe | 58 | -0.380 | 0.053 | --- | 2.636 | 2.422 | 2.869 |
| Asia | 9 | -0.518 | 0.071 | --- | 2.296 | 1.775 | 2.971 |
| oth/mult | 6 | -0.159 | 0.086 | (-) | 3.287 | 2.432 | 4.443 |

Table 2 - B - 6

IESCOPD - Meta-regression of current smoking, any product (or cigarettes if all product not available)

Multiple regression of data from Table 2 - B - 1 (most-adjusted RRs)

Any CB

Test by adding extra variables one at a time

|  |  | **Estimate** | **S.E.** | **P** | **RR** | **95%CIl** | **95%CIu** |
| --- | --- | --- | --- | --- | --- | --- | --- |
| **Asthma analysis type (CB)** |  |  |  |  |  |  |  |
| inc-irresp | 103 | Aliased |  |  | 2.775 | 2.603 | 2.958 |
| excl-all | 6 | 0.162 | 0.076 | + | 3.263 | 2.440 | 4.362 |
| defn-incl | 0 | Aliased |  |  | 2.775 | 2.603 | 2.958 |
| other | 4 | 1.239 | 0.237 | +++ | 9.581 | 3.723 | 24.655 |
| **Smoking product (3)** |  |  |  |  |  |  |  |
| any | 57 | Aliased |  |  | 2.867 | 2.169 | 3.790 |
| cigs | 52 | -0.047 | 0.137 | N.S. | 2.736 | 2.063 | 3.629 |
| cigsonly | 4 | 0.415 | 0.136 | ++ | 4.343 | 2.375 | 7.942 |
| **Unexposed group** |  |  |  |  |  |  |  |
| nev any | 69 | Aliased |  |  | 2.978 | 2.304 | 3.848 |
| nev cig | 44 | -0.115 | 0.130 | N.S. | 2.653 | 2.007 | 3.506 |
| **CB subtype** |  |  |  |  |  |  |  |
| mort | 4 | Aliased |  |  | 3.512 | 1.391 | 8.872 |
| sympt | 81 | -0.137 | 0.233 | N.S. | 3.062 | 2.829 | 3.314 |
| other | 28 | -0.447 | 0.233 | (-) | 2.246 | 1.937 | 2.604 |
| **Midpoint age in RR** |  | 0.004 | 0.002 | + | 2.265 | 1.609 | 3.189 |
| **RR adjusted for age** |  |  |  |  |  |  |  |
| Yes | 65 | Aliased |  |  | 2.594 | 2.389 | 2.816 |
| No | 48 | 0.235 | 0.039 | +++ | 3.280 | 2.914 | 3.691 |
| **RR adjusted for factor other than sex, age** |  |  |  |  |  |  |  |
| Yes | 29 | Aliased |  |  | 2.819 | 2.413 | 3.294 |
| No | 84 | -0.001 | 0.072 | N.S. | 2.817 | 2.406 | 3.297 |
| **Smoking results reported in study (CB)** |  |  |  |  |  |  |  |
| Current Only | 5 | Aliased |  |  | 2.097 | 1.317 | 3.337 |
| Both | 108 | 0.302 | 0.117 | + | 2.835 | 2.665 | 3.015 |
|  |  | **Deviance** | **(DF)** | **Drop Dev** | **P** |  |  |
| **Adding Derivation of RR/CI** |  | 397.315 | (96) | 10.382 | N.S. |  |  |
|  |  | **Estimate** | **S.E.** | **P** | **RR** | **95%CIl** | **95%CIu** |
| **Constant** |  | 1.507 | 0.263 | +++ | 4.512 | 2.693 | 7.560 |
| **Sex(RR)** |  |  |  |  |  |  |  |
| Male | 51 | Aliased |  |  | 2.865 | 2.381 | 3.448 |
| Female | 37 | -0.104 | 0.043 | - | 2.581 | 2.164 | 3.078 |
| Combined | 25 | 0.030 | 0.082 | N.S. | 2.951 | 2.473 | 3.522 |
| **Continent** |  |  |  |  |  |  |  |
| NAmer | 40 | Aliased |  |  | 3.680 | 3.093 | 4.380 |
| Europe | 58 | -0.323 | 0.053 | --- | 2.665 | 2.450 | 2.899 |
| Asia | 9 | -0.426 | 0.073 | --- | 2.405 | 1.851 | 3.125 |
| oth/mult | 6 | -0.171 | 0.086 | (-) | 3.103 | 2.284 | 4.216 |
| **Asthma analysis type (CB)** |  |  |  |  |  |  |  |
| inc-irresp | 103 | Aliased |  |  | 2.786 | 2.614 | 2.970 |
| excl-all | 6 | 0.097 | 0.078 | N.S. | 3.070 | 2.280 | 4.135 |
| defn-incl | 0 | Aliased |  |  | 2.786 | 2.614 | 2.970 |
| other | 4 | 1.235 | 0.237 | +++ | 9.580 | 3.740 | 24.539 |
| **Smoking product (3)** |  |  |  |  |  |  |  |
| any | 57 | Aliased |  |  | 2.884 | 2.185 | 3.809 |
| cigs | 52 | -0.059 | 0.137 | N.S. | 2.718 | 2.052 | 3.602 |
| cigsonly | 4 | 0.431 | 0.136 | ++ | 4.437 | 2.430 | 8.101 |
| **Unexposed group** |  |  |  |  |  |  |  |
| nev any | 69 | Aliased |  |  | 2.929 | 2.266 | 3.786 |
| nev cig | 44 | -0.081 | 0.131 | N.S. | 2.701 | 2.044 | 3.570 |
| **CB subtype** |  |  |  |  |  |  |  |
| mort | 4 | Aliased |  |  | 3.579 | 1.422 | 9.008 |
| sympt | 81 | -0.171 | 0.233 | N.S. | 3.015 | 2.782 | 3.268 |
| other | 28 | -0.425 | 0.232 | (-) | 2.340 | 2.007 | 2.727 |
| **Midpoint age in RR** |  | 0.002 | 0.002 | N.S. | 2.546 | 1.818 | 3.566 |
| **RR adjusted for age** |  |  |  |  |  |  |  |
| Yes | 65 | Aliased |  |  | 2.590 | 2.386 | 2.811 |
| No | 48 | 0.239 | 0.039 | +++ | 3.289 | 2.924 | 3.700 |

Table 2 - B - 6

IESCOPD - Meta-regression of current smoking, any product (or cigarettes if all product not available)

Multiple regression of data from Table 2 - B - 1 (most-adjusted RRs)

Any CB

Test by adding extra variables one at a time

|  |  | **Estimate** | **S.E.** | **P** | **RR** | **95%CIl** | **95%CIu** |
| --- | --- | --- | --- | --- | --- | --- | --- |
| **RR adjusted for factor other than sex, age** |  |  |  |  |  |  |  |
| Yes | 29 | Aliased |  |  | 2.845 | 2.436 | 3.321 |
| No | 84 | -0.019 | 0.072 | N.S. | 2.791 | 2.385 | 3.265 |
| **Derivation of RR/CI** |  |  |  |  |  |  |  |
| Orig/2x2 | 40 | Aliased |  |  | 3.039 | 2.719 | 3.397 |
| Other | 73 | -0.122 | 0.038 | -- | 2.690 | 2.475 | 2.924 |
|  |  | **Deviance** | **(DF)** | **Drop Dev** | **P** |  |  |
| **Adding Analysis type** |  | 407.462 | (96) | 0.235 | N.S. |  |  |
|  |  | **Estimate** | **S.E.** | **P** | **RR** | **95%CIl** | **95%CIu** |
| **Constant** |  | 1.263 | 0.298 | +++ | 3.538 | 1.974 | 6.339 |
| **Sex(RR)** |  |  |  |  |  |  |  |
| Male | 51 | Aliased |  |  | 2.843 | 2.356 | 3.430 |
| Female | 37 | -0.098 | 0.043 | - | 2.577 | 2.154 | 3.084 |
| Combined | 25 | 0.042 | 0.082 | N.S. | 2.966 | 2.477 | 3.551 |
| **Continent** |  |  |  |  |  |  |  |
| NAmer | 40 | Aliased |  |  | 3.765 | 3.163 | 4.482 |
| Europe | 58 | -0.351 | 0.052 | --- | 2.650 | 2.434 | 2.885 |
| Asia | 9 | -0.485 | 0.072 | --- | 2.317 | 1.780 | 3.017 |
| oth/mult | 6 | -0.141 | 0.086 | N.S. | 3.271 | 2.415 | 4.432 |
| **Asthma analysis type (CB)** |  |  |  |  |  |  |  |
| inc-irresp | 103 | Aliased |  |  | 2.782 | 2.600 | 2.978 |
| excl-all | 6 | 0.118 | 0.113 | N.S. | 3.132 | 2.036 | 4.818 |
| defn-incl | 0 | Aliased |  |  | 2.782 | 2.600 | 2.978 |
| other | 4 | 1.232 | 0.237 | +++ | 9.542 | 3.668 | 24.824 |
| **Smoking product (3)** |  |  |  |  |  |  |  |
| any | 57 | Aliased |  |  | 2.845 | 2.144 | 3.774 |
| cigs | 52 | -0.031 | 0.138 | N.S. | 2.758 | 2.071 | 3.672 |
| cigsonly | 4 | 0.417 | 0.137 | ++ | 4.318 | 2.335 | 7.985 |
| **Unexposed group** |  |  |  |  |  |  |  |
| nev any | 69 | Aliased |  |  | 2.987 | 2.305 | 3.870 |
| nev cig | 44 | -0.122 | 0.130 | N.S. | 2.644 | 1.995 | 3.504 |
| **CB subtype** |  |  |  |  |  |  |  |
| mort | 4 | Aliased |  |  | 3.179 | 1.086 | 9.308 |
| sympt | 81 | -0.037 | 0.270 | N.S. | 3.063 | 2.822 | 3.324 |
| other | 28 | -0.347 | 0.262 | N.S. | 2.248 | 1.926 | 2.624 |
| **Midpoint age in RR** |  | 0.003 | 0.002 | + | 2.388 | 1.709 | 3.338 |
| **RR adjusted for age** |  |  |  |  |  |  |  |
| Yes | 65 | Aliased |  |  | 2.605 | 2.397 | 2.831 |
| No | 48 | 0.223 | 0.039 | +++ | 3.255 | 2.888 | 3.668 |
| **RR adjusted for factor other than sex, age** |  |  |  |  |  |  |  |
| Yes | 29 | Aliased |  |  | 2.829 | 2.419 | 3.310 |
| No | 84 | -0.008 | 0.072 | N.S. | 2.806 | 2.394 | 3.289 |
| **Analysis type** |  |  |  |  |  |  |  |
| prevlnce | 103 | Aliased |  |  | 2.808 | 2.622 | 3.006 |
| onset | 10 | 0.067 | 0.139 | N.S. | 3.003 | 1.762 | 5.120 |

Table 2 - B - 6

IESCOPD - Meta-regression of current smoking, any product (or cigarettes if all product not available)

Multiple regression of data from Table 2 - B - 1 (most-adjusted RRs)

Any CB

Fitted values and residuals

|  | **Study Ref** | **NRR** | **SEX** | **LOGRR** | **FITVAL** | **SEFITV** | **STDRES** |
| --- | --- | --- | --- | --- | --- | --- | --- |
|  |  |  |  |  |  |  |  |
| #2173 | GOLDBE | 4 | m | 0.457 | 1.260 | 0.233 | -3.440 |
| #229 | JOUSI1 | 4 | f | 0.669 | 1.136 | 0.162 | -2.883 |
| #3420 | LINDST | 21 | b | 0.637 | 0.911 | 0.146 | -1.883 |
| #1728 | ENRIGH | 4 | f | 0.446 | 1.161 | 0.451 | -1.587 |
| #284 | KATO | 1 | m | 0.182 | 0.504 | 0.215 | -1.496 |
| #2543 | EHRLIC | 27 | m | 0.457 | 1.273 | 0.564 | -1.445 |
| #2833 | LEBOWI | 28 | b | 0.215 | 0.997 | 0.596 | -1.313 |
| #251 | NEJJAR | 3 | b | 0.470 | 1.034 | 0.463 | -1.219 |
| #2740 | LANGE2 | 1 | b | 1.030 | 1.402 | 0.309 | -1.205 |
| #2544 | EHRLIC | 28 | f | 0.693 | 1.175 | 0.455 | -1.061 |
| #226 | JOUSI1 | 1 | m | 1.094 | 1.234 | 0.133 | -1.050 |
| #2555 | PANDEY | 10 | f | 0.501 | 0.833 | 0.321 | -1.035 |
| #1544 | BANG | 1 | b | 0.588 | 0.962 | 0.368 | -1.016 |
| #1562 | BROWN | 4 | m | 0.805 | 1.407 | 0.604 | -0.995 |
| #339 | SHIMUR | 1 | b | -1.435 | 2.091 | 3.619 | -0.974 |
| #276 | VIEGI1 | 4 | f | -1.822 | 0.805 | 2.966 | -0.886 |
| #257 | MOLLER | 1 | b | 0.606 | 2.387 | 2.020 | -0.881 |
| #2105 | FERRI1 | 80 | f | 0.867 | 1.286 | 0.517 | -0.811 |
| #2768 | MEREN | 5 | b | 0.703 | 0.895 | 0.253 | -0.757 |
| #3194 | KAHN2 | 1 | m | 1.051 | 1.513 | 0.619 | -0.748 |
| #3117 | WAGEN2 | 9 | b | 1.109 | 1.289 | 0.264 | -0.683 |
| #1635 | DEAN2 | 1 | m | 0.824 | 1.040 | 0.357 | -0.605 |
| #2020 | OGILVI | 6 | f | 0.961 | 1.201 | 0.423 | -0.567 |
| #1740 | FOXMAN | 1 | m | 1.191 | 1.367 | 0.381 | -0.463 |
| #2662 | ANDER1 | 33 | f | 0.940 | 1.286 | 0.782 | -0.443 |
| #1551 | BECK2 | 1 | m | 0.835 | 1.606 | 1.840 | -0.419 |
| #1789 | HIGGI3 | 1 | m | 0.014 | 1.282 | 3.140 | -0.404 |
| #238 | KOTAN1 | 7 | b | 0.610 | 0.758 | 0.372 | -0.398 |
| #3483 | PELKON | 4 | m | 0.647 | 0.764 | 0.307 | -0.381 |
| #1957 | LANGHA | 10 | f | 0.908 | 0.966 | 0.153 | -0.375 |
| #1746 | FOXMAN | 4 | f | 1.144 | 1.270 | 0.338 | -0.372 |
| #2366 | HAENSZ | 8 | m | 0.916 | 1.047 | 0.436 | -0.299 |
| #1725 | ENRIGH | 1 | m | 1.049 | 1.259 | 0.741 | -0.282 |
| #1548 | BECK1 | 4 | f | 1.206 | 1.509 | 1.118 | -0.271 |
| #1554 | BECK2 | 4 | f | 1.198 | 1.509 | 1.537 | -0.202 |
| #2892 | HIGGI6 | 8 | f | 1.206 | 1.270 | 0.411 | -0.156 |
| #212 | MANFRE | 4 | f | 1.411 | 1.483 | 0.612 | -0.117 |
| #2704 | DEMARC | 18 | b | 1.404 | 1.427 | 0.250 | -0.093 |
| #548 | ALDERS | 9 | m | 1.109 | 1.155 | 0.553 | -0.084 |
| #1613 | COATES | 1 | b | 1.468 | 1.502 | 0.495 | -0.069 |
| #2056 | FERRI1 | 59 | m | 1.351 | 1.384 | 0.723 | -0.046 |
| #2014 | OGILVI | 1 | m | 1.282 | 1.298 | 0.753 | -0.022 |
| #2770 | MILNE | 13 | f | 1.224 | 1.240 | 0.969 | -0.016 |
| #1678 | DOPICO | 1 | m | 1.413 | 1.424 | 0.979 | -0.012 |
| #1771 | HIGGI2 | 1 | m | 1.089 | 1.101 | 2.135 | -0.006 |
| #1971 | LUNDB2 | 10 | f | 1.937 | 1.859 | 0.673 | 0.116 |
| #3568 | YAMAGU | 7 | b | 0.884 | 0.852 | 0.231 | 0.138 |
| #112 | LAVECC | 7 | b | 0.815 | 0.800 | 0.092 | 0.166 |
| #3409 | WEN | 7 | m | 1.141 | 0.904 | 1.131 | 0.210 |
| #1783 | HIGGI2 | 13 | f | 1.040 | 0.784 | 1.211 | 0.212 |
| #3549 | SILVA | 4 | b | 1.163 | 1.010 | 0.641 | 0.238 |
| #1749 | HARRIS | 1 | m | 1.876 | 1.579 | 1.092 | 0.272 |
| #1919 | LAMBER | 4 | m | 1.037 | 0.891 | 0.530 | 0.275 |
| #2592 | TROISI | 1 | f | 1.047 | 1.000 | 0.168 | 0.279 |
| #88 | DONTA2 | 1 | m | 1.191 | 0.974 | 0.740 | 0.294 |
| #270 | CERVER | 4 | b | 1.122 | 1.091 | 0.102 | 0.300 |
| #273 | VIEGI1 | 1 | m | 1.251 | 0.903 | 1.113 | 0.313 |
| #1629 | COLLEG | 4 | m | 1.115 | 0.745 | 1.129 | 0.328 |
| #1795 | HIGGI3 | 7 | f | 2.295 | 1.185 | 3.386 | 0.328 |
| #3509 | REID | 20 | f | 1.306 | 1.153 | 0.462 | 0.330 |
| #2659 | ANDER1 | 30 | m | 1.768 | 1.384 | 1.150 | 0.334 |
| #1965 | LUNDB2 | 4 | m | 2.202 | 1.957 | 0.683 | 0.359 |

Table 2 - B - 6

IESCOPD - Meta-regression of current smoking, any product (or cigarettes if all product not available)

Multiple regression of data from Table 2 - B - 1 (most-adjusted RRs)

Any CB

Fitted values and residuals

|  | **Study Ref** | **NRR** | **SEX** | **LOGRR** | **FITVAL** | **SEFITV** | **STDRES** |
| --- | --- | --- | --- | --- | --- | --- | --- |
|  |  |  |  |  |  |  |  |
| #3010 | HUHTI1 | 89 | f | 1.250 | 0.943 | 0.810 | 0.379 |
| #1545 | BECK1 | 1 | m | 2.104 | 1.606 | 1.300 | 0.383 |
| #2578 | STJERN | 1 | b | 1.673 | 1.272 | 1.017 | 0.394 |
| #1799 | HOLLA2 | 1 | m | 1.984 | 1.603 | 0.965 | 0.395 |
| #3586 | BEST | 1 | m | 2.420 | 1.943 | 1.191 | 0.401 |
| #3390 | TAGER2 | 2 | m | 1.647 | 1.260 | 0.935 | 0.413 |
| #282 | ZOIA | 1 | b | 1.482 | 1.057 | 1.008 | 0.421 |
| #2878 | HAWTHO | 15 | f | 1.146 | 1.039 | 0.241 | 0.444 |
| #2179 | GOLDBE | 10 | f | 1.303 | 1.163 | 0.305 | 0.459 |
| #206 | MANFRE | 1 | m | 2.211 | 1.580 | 1.331 | 0.474 |
| #201 | MENEZ1 | 4 | b | 1.461 | 1.201 | 0.544 | 0.478 |
| #2638 | WILHEL | 1 | m | 2.322 | 1.266 | 2.135 | 0.495 |
| #3431 | MUELLE | 34 | m | 2.123 | 1.367 | 1.525 | 0.496 |
| #549 | ALDERS | 10 | f | 1.238 | 1.058 | 0.351 | 0.514 |
| #1632 | COLLEG | 7 | f | 0.952 | 0.641 | 0.590 | 0.527 |
| #1671 | DEANE | 2 | m | 2.069 | 1.384 | 1.272 | 0.539 |
| #3131 | LAMBER | 36 | f | 1.157 | 0.943 | 0.368 | 0.581 |
| #1638 | DEAN2 | 3 | f | 1.112 | 0.943 | 0.281 | 0.601 |
| #3104 | HUHTI3 | 20 | m | 1.316 | 1.024 | 0.472 | 0.619 |
| #1974 | MELLST | 1 | m | 1.418 | 1.016 | 0.601 | 0.669 |
| #2885 | HIGGI6 | 1 | m | 1.725 | 1.367 | 0.526 | 0.678 |
| #2922 | HO | 7 | b | 0.963 | 0.598 | 0.523 | 0.698 |
| #3 | WOODS | 1 | b | 1.203 | 1.081 | 0.172 | 0.709 |
| #2698 | BJORNS | 4 | b | 1.131 | 1.021 | 0.150 | 0.734 |
| #248 | HUCHON | 4 | b | 1.351 | 1.188 | 0.218 | 0.745 |
| #2871 | HAWTHO | 8 | m | 1.380 | 1.136 | 0.311 | 0.785 |
| #1999 | MILNE | 1 | m | 2.308 | 1.188 | 1.285 | 0.871 |
| #1575 | DOLL1 | 14 | m | 2.242 | 1.144 | 1.244 | 0.882 |
| #3447 | MUELLE | 50 | f | 2.592 | 1.270 | 1.416 | 0.933 |
| #2549 | PANDEY | 4 | m | 1.673 | 0.930 | 0.791 | 0.940 |
| #2055 | HOLLNA | 9 | f | 1.725 | 1.123 | 0.632 | 0.953 |
| #2392 | HAENSZ | 30 | f | 1.273 | 0.950 | 0.337 | 0.957 |
| #241 | HOLLNA | 1 | m | 1.926 | 1.221 | 0.719 | 0.980 |
| #266 | JOSHI | 1 | m | 1.841 | 1.081 | 0.766 | 0.992 |
| #2862 | HARDIE | 10 | f | 1.526 | 0.596 | 0.933 | 0.998 |
| #3399 | TAGER2 | 11 | f | 2.455 | 1.163 | 1.278 | 1.011 |
| #3535 | SAWICK | 16 | f | 1.548 | 1.136 | 0.406 | 1.016 |
| #287 | KATO | 4 | f | 0.588 | 0.407 | 0.176 | 1.027 |
| #3493 | REID | 4 | m | 1.833 | 1.251 | 0.543 | 1.072 |
| #222 | WOOLF | 1 | f | 2.262 | 1.483 | 0.717 | 1.087 |
| #1987 | MILLER | 1 | m | 1.963 | 1.184 | 0.707 | 1.101 |
| #3522 | SAWICK | 3 | m | 1.868 | 1.234 | 0.576 | 1.102 |
| #263 | MAGNUS | 4 | m | 1.921 | 1.083 | 0.707 | 1.186 |
| #1816 | HOUSE | 10 | f | 1.739 | 1.389 | 0.272 | 1.285 |
| #2859 | HARDIE | 7 | m | 2.898 | 0.693 | 1.710 | 1.290 |
| #1810 | HOUSE | 4 | m | 1.918 | 1.486 | 0.332 | 1.302 |
| #2930 | HUHTI1 | 40 | m | 2.244 | 1.040 | 0.841 | 1.431 |
| #1990 | MILLER | 4 | f | 3.297 | 1.087 | 1.484 | 1.488 |
| #1951 | LANGHA | 4 | m | 1.364 | 1.063 | 0.170 | 1.763 |
| #2165 | HAYES | 7 | b | 2.067 | 1.306 | 0.353 | 2.158 |
| #2853 | SOBRAD | 1 | b | 2.561 | 1.305 | 0.501 | 2.507 |

## Table 3 - A - 6

IESCOPD - Meta-regression of ever smoking, any product (or cigarettes if all product not available)

Multiple regression of data from Table 3 - A - 1 (most-adjusted RRs)

Any emphysema

Fixed model

|  |  | **Deviance** | **(DF)** |  |  |  |  |
| --- | --- | --- | --- | --- | --- | --- | --- |
| **Fixed model** |  | 33.651 | (15) |  |  |  |  |
|  |  | **Estimate** | **S.E.** | **P** | **RR** | **95%CIl** | **95%CIu** |
| **Constant** |  | 1.388 | 0.621 | + | 4.005 | 1.186 | 13.521 |
| **Sex(RR)** |  |  |  |  |  |  |  |
| Male | 13 | Aliased |  |  | 2.713 | 1.809 | 4.067 |
| Female | 6 | -0.370 | 0.207 | (-) | 1.874 | 1.021 | 3.439 |
| Combined | 9 | 0.310 | 0.185 | N.S. | 3.699 | 2.965 | 4.614 |
| **Continent** |  |  |  |  |  |  |  |
| NAmer | 14 | Aliased |  |  | 3.936 | 2.421 | 6.401 |
| Europe | 10 | -0.377 | 0.265 | N.S. | 2.699 | 1.894 | 3.846 |
| Asia | 4 | 0.121 | 0.192 | N.S. | 4.444 | 2.344 | 8.424 |
| **Asthma analysis type (Emp)** |  |  |  |  |  |  |  |
| inc-irresp | 26 | Aliased |  |  | 3.236 | 2.830 | 3.699 |
| excl-all | 2 | -0.404 | 0.456 | N.S. | 2.161 | 0.571 | 8.169 |
| **Smoking product (3)** |  |  |  |  |  |  |  |
| any | 11 | Aliased |  |  | 3.264 | 2.321 | 4.591 |
| cigs | 15 | -0.040 | 0.310 | N.S. | 3.136 | 1.703 | 5.773 |
| cigsonly | 2 | -0.005 | 0.393 | N.S. | 3.248 | 1.042 | 10.118 |
| **Unexposed group** |  |  |  |  |  |  |  |
| nev any | 14 | Aliased |  |  | 3.094 | 2.133 | 4.488 |
| nev cig | 14 | 0.131 | 0.391 | N.S. | 3.528 | 1.567 | 7.941 |
| **Emp subtype** |  |  |  |  |  |  |  |
| mort | 4 | Aliased |  |  | 4.907 | 1.773 | 13.578 |
| other | 24 | -0.470 | 0.383 | N.S. | 3.068 | 2.574 | 3.658 |
| **Midpoint age in RR** |  | -0.003 | 0.008 | N.S. | 3.732 | 0.971 | 14.347 |
| **RR adjusted for age** |  |  |  |  |  |  |  |
| Yes | 15 | Aliased |  |  | 3.523 | 2.715 | 4.571 |
| No | 13 | -0.306 | 0.260 | N.S. | 2.594 | 1.491 | 4.514 |
| **RR adjusted for factor other than sex, age** |  |  |  |  |  |  |  |
| Yes | 6 | Aliased |  |  | 2.048 | 1.403 | 2.990 |
| No | 22 | 0.977 | 0.261 | ++ | 5.442 | 3.533 | 8.382 |

Table 3 - A - 6

IESCOPD - Meta-regression of ever smoking, any product (or cigarettes if all product not available)

Multiple regression of data from Table 3 - A - 1 (most-adjusted RRs)

Any emphysema

Test by removing variables one at a time

|  |  | **Deviance** | **(DF)** | **Drop Dev** | **P** |  |  |
| --- | --- | --- | --- | --- | --- | --- | --- |
| **Omitting Sex (RR)** |  | 41.171 | (17) | -7.521 | N.S. |  |  |
|  |  | **Estimate** | **S.E.** | **P** | **RR** | **95%CIl** | **95%CIu** |
| **Constant** |  | 2.241 | 0.522 | +++ | 9.405 | 3.379 | 26.179 |
| **RR adjusted for factor other than sex, age** |  |  |  |  |  |  |  |
| Yes | 6 | Aliased |  |  | 2.548 | 1.933 | 3.360 |
| No | 22 | 0.505 | 0.193 | + | 4.223 | 3.090 | 5.771 |
| **Continent** |  |  |  |  |  |  |  |
| NAmer | 14 | Aliased |  |  | 3.718 | 2.377 | 5.816 |
| Europe | 10 | -0.306 | 0.259 | N.S. | 2.739 | 1.977 | 3.795 |
| Asia | 4 | 0.222 | 0.187 | N.S. | 4.644 | 2.583 | 8.350 |
| **Asthma analysis type (Emp)** |  |  |  |  |  |  |  |
| inc-irresp | 26 | Aliased |  |  | 3.239 | 2.856 | 3.672 |
| excl-all | 2 | -0.476 | 0.437 | N.S. | 2.012 | 0.608 | 6.663 |
| **Smoking product (3)** |  |  |  |  |  |  |  |
| any | 11 | Aliased |  |  | 3.029 | 2.225 | 4.124 |
| cigs | 15 | 0.166 | 0.297 | N.S. | 3.575 | 2.061 | 6.203 |
| cigsonly | 2 | 0.246 | 0.379 | N.S. | 3.874 | 1.373 | 10.932 |
| **Unexposed group** |  |  |  |  |  |  |  |
| nev any | 14 | Aliased |  |  | 3.228 | 2.306 | 4.520 |
| nev cig | 14 | -0.009 | 0.375 | N.S. | 3.199 | 1.540 | 6.645 |
| **Emp subtype** |  |  |  |  |  |  |  |
| mort | 4 | Aliased |  |  | 5.429 | 2.100 | 14.039 |
| other | 24 | -0.582 | 0.381 | N.S. | 3.033 | 2.572 | 3.576 |
| **Midpoint age in RR** |  | -0.011 | 0.007 | N.S. | 6.253 | 1.984 | 19.704 |
| **RR adjusted for age** |  |  |  |  |  |  |  |
| Yes | 15 | Aliased |  |  | 3.273 | 2.593 | 4.130 |
| No | 13 | -0.056 | 0.242 | N.S. | 3.095 | 1.901 | 5.041 |

Table 3 - A - 6

IESCOPD - Meta-regression of ever smoking, any product (or cigarettes if all product not available)

Multiple regression of data from Table 3 - A - 1 (most-adjusted RRs)

Any emphysema

Fixed model, test by removing variables one at a time

|  |  | **Deviance** | **(DF)** | **Drop Dev** | **P** |  |  |
| --- | --- | --- | --- | --- | --- | --- | --- |
| **Omitting Continent** |  | 36.278 | (17) | -2.628 | N.S. |  |  |
|  |  | **Estimate** | **S.E.** | **P** | **RR** | **95%CIl** | **95%CIu** |
| **Constant** |  | 1.670 | 0.589 | + | 5.312 | 1.675 | 16.842 |
| **RR adjusted for factor other than sex, age** |  |  |  |  |  |  |  |
| Yes | 6 | Aliased |  |  | 2.115 | 1.494 | 2.995 |
| No | 22 | 0.908 | 0.254 | ++ | 5.242 | 3.525 | 7.797 |
| **Sex(RR)** |  |  |  |  |  |  |  |
| Male | 13 | Aliased |  |  | 2.824 | 1.945 | 4.100 |
| Female | 6 | -0.425 | 0.202 | (-) | 1.846 | 1.057 | 3.226 |
| Combined | 9 | 0.258 | 0.181 | N.S. | 3.657 | 2.980 | 4.488 |
| **Asthma analysis type (Emp)** |  |  |  |  |  |  |  |
| inc-irresp | 26 | Aliased |  |  | 3.243 | 2.860 | 3.677 |
| excl-all | 2 | -0.581 | 0.443 | N.S. | 1.813 | 0.539 | 6.097 |
| **Smoking product (3)** |  |  |  |  |  |  |  |
| any | 11 | Aliased |  |  | 3.236 | 2.350 | 4.456 |
| cigs | 15 | -0.024 | 0.309 | N.S. | 3.161 | 1.782 | 5.606 |
| cigsonly | 2 | 0.169 | 0.371 | N.S. | 3.831 | 1.392 | 10.539 |
| **Unexposed group** |  |  |  |  |  |  |  |
| nev any | 14 | Aliased |  |  | 2.808 | 2.066 | 3.816 |
| nev cig | 14 | 0.452 | 0.336 | N.S. | 4.413 | 2.285 | 8.519 |
| **Emp subtype** |  |  |  |  |  |  |  |
| mort | 4 | Aliased |  |  | 7.051 | 3.584 | 13.870 |
| other | 24 | -0.873 | 0.269 | -- | 2.944 | 2.544 | 3.407 |
| **Midpoint age in RR** |  | -0.006 | 0.007 | N.S. | 4.566 | 1.402 | 14.868 |
| **RR adjusted for age** |  |  |  |  |  |  |  |
| Yes | 15 | Aliased |  |  | 3.341 | 2.673 | 4.176 |
| No | 13 | -0.126 | 0.228 | N.S. | 2.945 | 1.858 | 4.667 |
|  |  | **Deviance** | **(DF)** | **Drop Dev** | **P** |  |  |
| **Omitting Asthma analysis type (Emp)** |  | 34.433 | (16) | -0.783 | N.S. |  |  |
|  |  | **Estimate** | **S.E.** | **P** | **RR** | **95%CIl** | **95%CIu** |
| **Constant** |  | 1.288 | 0.611 | (+) | 3.626 | 1.096 | 12.000 |
| **RR adjusted for factor other than sex, age** |  |  |  |  |  |  |  |
| Yes | 6 | Aliased |  |  | 2.050 | 1.421 | 2.956 |
| No | 22 | 0.976 | 0.261 | ++ | 5.437 | 3.578 | 8.261 |
| **Sex(RR)** |  |  |  |  |  |  |  |
| Male | 13 | Aliased |  |  | 2.628 | 1.800 | 3.838 |
| Female | 6 | -0.338 | 0.204 | N.S. | 1.875 | 1.042 | 3.375 |
| Combined | 9 | 0.352 | 0.179 | (+) | 3.739 | 3.027 | 4.618 |
| **Continent** |  |  |  |  |  |  |  |
| NAmer | 14 | Aliased |  |  | 4.053 | 2.556 | 6.429 |
| Europe | 10 | -0.431 | 0.258 | N.S. | 2.635 | 1.886 | 3.681 |
| Asia | 4 | 0.132 | 0.191 | N.S. | 4.624 | 2.523 | 8.476 |
| **Smoking product (3)** |  |  |  |  |  |  |  |
| any | 11 | Aliased |  |  | 3.268 | 2.349 | 4.547 |
| cigs | 15 | -0.042 | 0.310 | N.S. | 3.135 | 1.736 | 5.661 |
| cigsonly | 2 | -0.048 | 0.390 | N.S. | 3.115 | 1.045 | 9.284 |
| **Unexposed group** |  |  |  |  |  |  |  |
| nev any | 14 | Aliased |  |  | 3.166 | 2.225 | 4.504 |
| nev cig | 14 | 0.055 | 0.382 | N.S. | 3.346 | 1.554 | 7.207 |
| **Emp subtype** |  |  |  |  |  |  |  |
| mort | 4 | Aliased |  |  | 4.805 | 1.798 | 12.844 |
| other | 24 | -0.446 | 0.382 | N.S. | 3.076 | 2.595 | 3.646 |
| **Midpoint age in RR** |  | -0.001 | 0.008 | N.S. | 3.411 | 0.957 | 12.160 |
| **RR adjusted for age** |  |  |  |  |  |  |  |
| Yes | 15 | Aliased |  |  | 3.507 | 2.726 | 4.512 |
| No | 13 | -0.291 | 0.259 | N.S. | 2.621 | 1.535 | 4.477 |

Table 3 - A - 6

IESCOPD - Meta-regression of ever smoking, any product (or cigarettes if all product not available)

Multiple regression of data from Table 3 - A - 1 (most-adjusted RRs)

Any emphysema

Test by removing variables one at a time

|  |  | **Deviance** | **(DF)** | **Drop Dev** | **P** |  |  |
| --- | --- | --- | --- | --- | --- | --- | --- |
| **Omitting Smoking product (3)** |  | 33.667 | (17) | -0.017 | N.S. |  |  |
|  |  | **Estimate** | **S.E.** | **P** | **RR** | **95%CIl** | **95%CIu** |
| **Constant** |  | 1.389 | 0.612 | + | 4.010 | 1.208 | 13.307 |
| **RR adjusted for factor other than sex, age** |  |  |  |  |  |  |  |
| Yes | 6 | Aliased |  |  | 2.053 | 1.456 | 2.895 |
| No | 22 | 0.972 | 0.251 | ++ | 5.427 | 3.666 | 8.033 |
| **Sex(RR)** |  |  |  |  |  |  |  |
| Male | 13 | Aliased |  |  | 2.711 | 1.870 | 3.931 |
| Female | 6 | -0.362 | 0.199 | (-) | 1.887 | 1.096 | 3.247 |
| Combined | 9 | 0.310 | 0.180 | N.S. | 3.696 | 3.018 | 4.525 |
| **Continent** |  |  |  |  |  |  |  |
| NAmer | 14 | Aliased |  |  | 3.935 | 2.551 | 6.069 |
| Europe | 10 | -0.377 | 0.250 | N.S. | 2.700 | 1.962 | 3.713 |
| Asia | 4 | 0.121 | 0.191 | N.S. | 4.442 | 2.461 | 8.018 |
| **Asthma analysis type (Emp)** |  |  |  |  |  |  |  |
| inc-irresp | 26 | Aliased |  |  | 3.236 | 2.853 | 3.669 |
| excl-all | 2 | -0.404 | 0.453 | N.S. | 2.161 | 0.626 | 7.465 |
| **Unexposed group** |  |  |  |  |  |  |  |
| nev any | 14 | Aliased |  |  | 3.129 | 2.449 | 3.999 |
| nev cig | 14 | 0.094 | 0.253 | N.S. | 3.437 | 2.080 | 5.678 |
| **Emp subtype** |  |  |  |  |  |  |  |
| mort | 4 | Aliased |  |  | 4.862 | 1.946 | 12.144 |
| other | 24 | -0.459 | 0.366 | N.S. | 3.071 | 2.612 | 3.612 |
| **Midpoint age in RR** |  | -0.003 | 0.007 | N.S. | 3.778 | 1.113 | 12.832 |
| **RR adjusted for age** |  |  |  |  |  |  |  |
| Yes | 15 | Aliased |  |  | 3.519 | 2.760 | 4.488 |
| No | 13 | -0.303 | 0.257 | N.S. | 2.600 | 1.552 | 4.356 |
|  |  | **Deviance** | **(DF)** | **Drop Dev** | **P** |  |  |
| **Omitting Unexposed group** |  | 33.763 | (16) | -0.112 | N.S. |  |  |
|  |  | **Estimate** | **S.E.** | **P** | **RR** | **95%CIl** | **95%CIu** |
| **Constant** |  | 1.319 | 0.587 | + | 3.741 | 1.185 | 11.809 |
| **RR adjusted for factor other than sex, age** |  |  |  |  |  |  |  |
| Yes | 6 | Aliased |  |  | 2.043 | 1.417 | 2.945 |
| No | 22 | 0.982 | 0.260 | ++ | 5.457 | 3.594 | 8.286 |
| **Sex(RR)** |  |  |  |  |  |  |  |
| Male | 13 | Aliased |  |  | 2.693 | 1.828 | 3.968 |
| Female | 6 | -0.350 | 0.199 | (-) | 1.897 | 1.064 | 3.384 |
| Combined | 9 | 0.318 | 0.184 | N.S. | 3.701 | 2.988 | 4.584 |
| **Continent** |  |  |  |  |  |  |  |
| NAmer | 14 | Aliased |  |  | 4.010 | 2.574 | 6.249 |
| Europe | 10 | -0.414 | 0.241 | N.S. | 2.651 | 1.949 | 3.607 |
| Asia | 4 | 0.138 | 0.185 | N.S. | 4.605 | 2.682 | 7.906 |
| **Asthma analysis type (Emp)** |  |  |  |  |  |  |  |
| inc-irresp | 26 | Aliased |  |  | 3.234 | 2.841 | 3.682 |
| excl-all | 2 | -0.370 | 0.445 | N.S. | 2.233 | 0.635 | 7.849 |
| **Smoking product (3)** |  |  |  |  |  |  |  |
| any | 11 | Aliased |  |  | 3.182 | 2.478 | 4.087 |
| cigs | 15 | 0.035 | 0.213 | N.S. | 3.296 | 2.181 | 4.982 |
| cigsonly | 2 | -0.021 | 0.390 | N.S. | 3.116 | 1.098 | 8.843 |
| **Emp subtype** |  |  |  |  |  |  |  |
| mort | 4 | Aliased |  |  | 4.609 | 2.009 | 10.576 |
| other | 24 | -0.400 | 0.322 | N.S. | 3.090 | 2.636 | 3.623 |
| **Midpoint age in RR** |  | -0.002 | 0.008 | N.S. | 3.626 | 1.008 | 13.049 |
| **RR adjusted for age** |  |  |  |  |  |  |  |
| Yes | 15 | Aliased |  |  | 3.525 | 2.740 | 4.537 |
| No | 13 | -0.309 | 0.259 | N.S. | 2.589 | 1.515 | 4.426 |

Table 3 - A - 6

IESCOPD - Meta-regression of ever smoking, any product (or cigarettes if all product not available)

Multiple regression of data from Table 3 - A - 1 (most-adjusted RRs)

Any emphysema

Test by removing variables one at a time

|  |  | **Deviance** | **(DF)** | **Drop Dev** | **P** |  |  |
| --- | --- | --- | --- | --- | --- | --- | --- |
| **Omitting Emp subtype** |  | 35.153 | (16) | -1.503 | N.S. |  |  |
|  |  | **Estimate** | **S.E.** | **P** | **RR** | **95%CIl** | **95%CIu** |
| **Constant** |  | 1.020 | 0.544 | (+) | 2.773 | 0.956 | 8.048 |
| **RR adjusted for factor other than sex, age** |  |  |  |  |  |  |  |
| Yes | 6 | Aliased |  |  | 1.904 | 1.376 | 2.635 |
| No | 22 | 1.135 | 0.227 | +++ | 5.922 | 4.093 | 8.569 |
| **Sex(RR)** |  |  |  |  |  |  |  |
| Male | 13 | Aliased |  |  | 2.704 | 1.827 | 4.001 |
| Female | 6 | -0.394 | 0.206 | (-) | 1.823 | 1.016 | 3.270 |
| Combined | 9 | 0.319 | 0.185 | N.S. | 3.718 | 3.003 | 4.604 |
| **Continent** |  |  |  |  |  |  |  |
| NAmer | 14 | Aliased |  |  | 4.503 | 3.166 | 6.406 |
| Europe | 10 | -0.608 | 0.186 | -- | 2.452 | 1.889 | 3.182 |
| Asia | 4 | 0.118 | 0.192 | N.S. | 5.067 | 2.955 | 8.689 |
| **Asthma analysis type (Emp)** |  |  |  |  |  |  |  |
| inc-irresp | 26 | Aliased |  |  | 3.234 | 2.841 | 3.682 |
| excl-all | 2 | -0.365 | 0.455 | N.S. | 2.244 | 0.621 | 8.111 |
| **Smoking product (3)** |  |  |  |  |  |  |  |
| any | 11 | Aliased |  |  | 3.187 | 2.301 | 4.414 |
| cigs | 15 | 0.034 | 0.304 | N.S. | 3.298 | 1.848 | 5.886 |
| cigsonly | 2 | -0.093 | 0.387 | N.S. | 2.905 | 0.997 | 8.466 |
| **Unexposed group** |  |  |  |  |  |  |  |
| nev any | 14 | Aliased |  |  | 3.348 | 2.455 | 4.566 |
| nev cig | 14 | -0.129 | 0.328 | N.S. | 2.942 | 1.515 | 5.712 |
| **Midpoint age in RR** |  | -0.000 | 0.008 | N.S. | 3.313 | 0.927 | 11.847 |
| **RR adjusted for age** |  |  |  |  |  |  |  |
| Yes | 15 | Aliased |  |  | 3.749 | 3.049 | 4.611 |
| No | 13 | -0.518 | 0.194 | - | 2.234 | 1.484 | 3.364 |
|  |  | **Deviance** | **(DF)** | **Drop Dev** | **P** |  |  |
| **Omitting Midpoint age in RR** |  | 33.756 | (16) | -0.105 | N.S. |  |  |
|  |  | **Estimate** | **S.E.** | **P** | **RR** | **95%CIl** | **95%CIu** |
| **Constant** |  | 1.210 | 0.290 | +++ | 3.352 | 1.900 | 5.916 |
| **RR adjusted for factor other than sex, age** |  |  |  |  |  |  |  |
| Yes | 6 | Aliased |  |  | 2.019 | 1.431 | 2.847 |
| No | 22 | 1.008 | 0.242 | +++ | 5.533 | 3.740 | 8.187 |
| **Sex(RR)** |  |  |  |  |  |  |  |
| Male | 13 | Aliased |  |  | 2.681 | 1.836 | 3.914 |
| Female | 6 | -0.383 | 0.203 | (-) | 1.828 | 1.060 | 3.152 |
| Combined | 9 | 0.330 | 0.175 | (+) | 3.728 | 3.043 | 4.567 |
| **Continent** |  |  |  |  |  |  |  |
| NAmer | 14 | Aliased |  |  | 3.949 | 2.469 | 6.318 |
| Europe | 10 | -0.388 | 0.263 | N.S. | 2.678 | 1.913 | 3.749 |
| Asia | 4 | 0.141 | 0.182 | N.S. | 4.549 | 2.536 | 8.159 |
| **Asthma analysis type (Emp)** |  |  |  |  |  |  |  |
| inc-irresp | 26 | Aliased |  |  | 3.234 | 2.841 | 3.682 |
| excl-all | 2 | -0.371 | 0.445 | N.S. | 2.232 | 0.636 | 7.835 |
| **Smoking product (3)** |  |  |  |  |  |  |  |
| any | 11 | Aliased |  |  | 3.289 | 2.381 | 4.544 |
| cigs | 15 | -0.062 | 0.302 | N.S. | 3.093 | 1.735 | 5.515 |
| cigsonly | 2 | -0.027 | 0.387 | N.S. | 3.202 | 1.073 | 9.554 |
| **Unexposed group** |  |  |  |  |  |  |  |
| nev any | 14 | Aliased |  |  | 3.117 | 2.186 | 4.443 |
| nev cig | 14 | 0.107 | 0.384 | N.S. | 3.469 | 1.603 | 7.507 |
| **Emp subtype** |  |  |  |  |  |  |  |
| mort | 4 | Aliased |  |  | 4.792 | 1.829 | 12.556 |
| other | 24 | -0.443 | 0.374 | N.S. | 3.077 | 2.599 | 3.641 |
| **RR adjusted for age** |  |  |  |  |  |  |  |
| Yes | 15 | Aliased |  |  | 3.538 | 2.758 | 4.540 |
| No | 13 | -0.321 | 0.255 | N.S. | 2.567 | 1.514 | 4.352 |

Table 3 - A - 6

IESCOPD - Meta-regression of ever smoking, any product (or cigarettes if all product not available)

Multiple regression of data from Table 3 - A - 1 (most-adjusted RRs)

Any emphysema

Test by removing variables one at a time

|  |  | **Deviance** | **(DF)** | **Drop Dev** | **P** |  |  |
| --- | --- | --- | --- | --- | --- | --- | --- |
| **Omitting RR adjusted for age** |  | 35.040 | (16) | -1.389 | N.S. |  |  |
|  |  | **Estimate** | **S.E.** | **P** | **RR** | **95%CIl** | **95%CIu** |
| **Constant** |  | 1.675 | 0.571 | ++ | 5.339 | 1.744 | 16.344 |
| **RR adjusted for factor other than sex, age** |  |  |  |  |  |  |  |
| Yes | 6 | Aliased |  |  | 2.263 | 1.716 | 2.983 |
| No | 22 | 0.762 | 0.186 | +++ | 4.847 | 3.549 | 6.621 |
| **Sex(RR)** |  |  |  |  |  |  |  |
| Male | 13 | Aliased |  |  | 2.756 | 1.866 | 4.072 |
| Female | 6 | -0.300 | 0.198 | N.S. | 2.041 | 1.177 | 3.540 |
| Combined | 9 | 0.276 | 0.183 | N.S. | 3.632 | 2.946 | 4.479 |
| **Continent** |  |  |  |  |  |  |  |
| NAmer | 14 | Aliased |  |  | 3.605 | 2.368 | 5.487 |
| Europe | 10 | -0.228 | 0.232 | N.S. | 2.870 | 2.106 | 3.910 |
| Asia | 4 | 0.129 | 0.192 | N.S. | 4.101 | 2.277 | 7.384 |
| **Asthma analysis type (Emp)** |  |  |  |  |  |  |  |
| inc-irresp | 26 | Aliased |  |  | 3.234 | 2.841 | 3.682 |
| excl-all | 2 | -0.369 | 0.456 | N.S. | 2.235 | 0.618 | 8.081 |
| **Smoking product (3)** |  |  |  |  |  |  |  |
| any | 11 | Aliased |  |  | 3.219 | 2.318 | 4.471 |
| cigs | 15 | -0.002 | 0.308 | N.S. | 3.214 | 1.785 | 5.786 |
| cigsonly | 2 | 0.039 | 0.391 | N.S. | 3.348 | 1.117 | 10.037 |
| **Unexposed group** |  |  |  |  |  |  |  |
| nev any | 14 | Aliased |  |  | 3.081 | 2.149 | 4.415 |
| nev cig | 14 | 0.146 | 0.391 | N.S. | 3.563 | 1.625 | 7.814 |
| **Emp subtype** |  |  |  |  |  |  |  |
| mort | 4 | Aliased |  |  | 6.426 | 3.064 | 13.473 |
| other | 24 | -0.770 | 0.286 | - | 2.975 | 2.553 | 3.468 |
| **Midpoint age in RR** |  | -0.004 | 0.008 | N.S. | 4.113 | 1.141 | 14.831 |
|  |  | **Deviance** | **(DF)** | **Drop Dev** | **P** |  |  |
| **Omitting RR adjusted for factor other than sex, age** |  | 47.697 | (16) | -14.047 | * |  |  |
|  |  | **Estimate** | **S.E.** | **P** | **RR** | **95%CIl** | **95%CIu** |
| **Constant** |  | 2.893 | 0.473 | +++ | 18.054 | 7.141 | 45.643 |
| **RR adjusted for age** |  |  |  |  |  |  |  |
| Yes | 15 | Aliased |  |  | 2.883 | 2.357 | 3.526 |
| No | 13 | 0.375 | 0.185 | (+) | 4.196 | 2.832 | 6.217 |
| **Sex(RR)** |  |  |  |  |  |  |  |
| Male | 13 | Aliased |  |  | 3.332 | 2.325 | 4.774 |
| Female | 6 | -0.106 | 0.194 | N.S. | 2.997 | 1.877 | 4.784 |
| Combined | 9 | -0.035 | 0.161 | N.S. | 3.216 | 2.671 | 3.873 |
| **Continent** |  |  |  |  |  |  |  |
| NAmer | 14 | Aliased |  |  | 3.456 | 2.181 | 5.475 |
| Europe | 10 | -0.166 | 0.259 | N.S. | 2.926 | 2.088 | 4.101 |
| Asia | 4 | 0.170 | 0.191 | N.S. | 4.096 | 2.212 | 7.586 |
| **Asthma analysis type (Emp)** |  |  |  |  |  |  |  |
| inc-irresp | 26 | Aliased |  |  | 3.235 | 2.842 | 3.683 |
| excl-all | 2 | -0.391 | 0.456 | N.S. | 2.187 | 0.603 | 7.928 |
| **Smoking product (3)** |  |  |  |  |  |  |  |
| any | 11 | Aliased |  |  | 3.041 | 2.195 | 4.213 |
| cigs | 15 | 0.149 | 0.305 | N.S. | 3.529 | 1.968 | 6.329 |
| cigsonly | 2 | 0.347 | 0.382 | N.S. | 4.301 | 1.461 | 12.658 |
| **Unexposed group** |  |  |  |  |  |  |  |
| nev any | 14 | Aliased |  |  | 3.013 | 2.103 | 4.317 |
| nev cig | 14 | 0.219 | 0.390 | N.S. | 3.750 | 1.712 | 8.215 |
| **Emp subtype** |  |  |  |  |  |  |  |
| mort | 4 | Aliased |  |  | 9.259 | 3.919 | 21.876 |
| other | 24 | -1.177 | 0.333 | -- | 2.854 | 2.429 | 3.353 |
| **Midpoint age in RR** |  | -0.013 | 0.007 | (-) | 7.011 | 2.085 | 23.578 |

Table 3 - A - 6

IESCOPD - Meta-regression of ever smoking, any product (or cigarettes if all product not available)

Multiple regression of data from Table 3 - A - 1 (most-adjusted RRs)

Any emphysema

Test reduction to 2-level product

|  |  | **Deviance** | **(DF)** | **Drop Dev** | **P** |  |  |
| --- | --- | --- | --- | --- | --- | --- | --- |
| **Reducing Smoking Product to 2 levels** |  | 33.656 | (16) | -0.006 | N.S. |  |  |
|  |  | **Estimate** | **S.E.** | **P** | **RR** | **95%CIl** | **95%CIu** |
| **Constant** |  | 1.382 | 0.616 | + | 3.981 | 1.191 | 13.310 |
| **Sex(RR)** |  |  |  |  |  |  |  |
| Male | 13 | Aliased |  |  | 2.707 | 1.842 | 3.979 |
| Female | 6 | -0.368 | 0.205 | (-) | 1.874 | 1.041 | 3.374 |
| Combined | 9 | 0.313 | 0.182 | N.S. | 3.701 | 2.992 | 4.578 |
| **Continent** |  |  |  |  |  |  |  |
| NAmer | 14 | Aliased |  |  | 3.948 | 2.501 | 6.233 |
| Europe | 10 | -0.383 | 0.256 | N.S. | 2.693 | 1.928 | 3.763 |
| Asia | 4 | 0.120 | 0.192 | N.S. | 4.454 | 2.413 | 8.222 |
| **Asthma analysis type (Emp)** |  |  |  |  |  |  |  |
| inc-irresp | 26 | Aliased |  |  | 3.236 | 2.842 | 3.683 |
| excl-all | 2 | -0.400 | 0.454 | N.S. | 2.168 | 0.602 | 7.807 |
| **Smoking product (2)** |  |  |  |  |  |  |  |
| any | 11 | Aliased |  |  | 3.251 | 2.419 | 4.369 |
| cigs | 17 | -0.027 | 0.261 | N.S. | 3.163 | 1.934 | 5.175 |
| **Unexposed group** |  |  |  |  |  |  |  |
| nev any | 14 | Aliased |  |  | 3.111 | 2.303 | 4.201 |
| nev cig | 14 | 0.114 | 0.316 | N.S. | 3.485 | 1.839 | 6.603 |
| **Emp subtype** |  |  |  |  |  |  |  |
| mort | 4 | Aliased |  |  | 4.870 | 1.893 | 12.529 |
| other | 24 | -0.461 | 0.367 | N.S. | 3.071 | 2.598 | 3.630 |
| **Midpoint age in RR** |  | -0.003 | 0.008 | N.S. | 3.732 | 1.013 | 13.745 |
| **RR adjusted for age** |  |  |  |  |  |  |  |
| Yes | 15 | Aliased |  |  | 3.523 | 2.737 | 4.534 |
| No | 13 | -0.306 | 0.260 | N.S. | 2.594 | 1.517 | 4.434 |
| **RR adjusted for factor other than sex, age** |  |  |  |  |  |  |  |
| Yes | 6 | Aliased |  |  | 2.046 | 1.421 | 2.947 |
| No | 22 | 0.979 | 0.260 | ++ | 5.447 | 3.591 | 8.262 |

Table 3 - A - 6

IESCOPD - Meta-regression of ever smoking, any product (or cigarettes if all product not available)

Multiple regression of data from Table 3 - A - 1 (most-adjusted RRs)

Any emphysema

Test by adding extra variables one at a time

|  |  | **Deviance** | **(DF)** | **Drop Dev** | **P** |  |  |
| --- | --- | --- | --- | --- | --- | --- | --- |
| **Increasing Emp subtype to 3 levels** |  | 28.309 | (14) | 5.341 | N.S. |  |  |
|  |  | **Estimate** | **S.E.** | **P** | **RR** | **95%CIl** | **95%CIu** |
| **Constant** |  | 1.949 | 0.667 | + | 7.024 | 1.902 | 25.947 |
| **Sex(RR)** |  |  |  |  |  |  |  |
| Male | 13 | Aliased |  |  | 2.749 | 1.871 | 4.039 |
| Female | 6 | -0.377 | 0.207 | (-) | 1.886 | 1.060 | 3.356 |
| Combined | 9 | 0.291 | 0.186 | N.S. | 3.679 | 2.982 | 4.538 |
| **Continent** |  |  |  |  |  |  |  |
| NAmer | 14 | Aliased |  |  | 4.189 | 2.624 | 6.685 |
| Europe | 10 | -0.426 | 0.266 | N.S. | 2.735 | 1.953 | 3.830 |
| Asia | 4 | -0.102 | 0.215 | N.S. | 3.783 | 2.000 | 7.157 |
| **Asthma analysis type (Emp)** |  |  |  |  |  |  |  |
| inc-irresp | 26 | Aliased |  |  | 3.223 | 2.838 | 3.660 |
| excl-all | 2 | -0.079 | 0.478 | N.S. | 2.979 | 0.795 | 11.161 |
| **Smoking product (3)** |  |  |  |  |  |  |  |
| any | 11 | Aliased |  |  | 3.210 | 2.320 | 4.440 |
| cigs | 15 | 0.006 | 0.310 | N.S. | 3.228 | 1.806 | 5.767 |
| cigsonly | 2 | 0.066 | 0.394 | N.S. | 3.427 | 1.163 | 10.101 |
| **Unexposed group** |  |  |  |  |  |  |  |
| nev any | 14 | Aliased |  |  | 2.866 | 1.990 | 4.128 |
| nev cig | 14 | 0.384 | 0.406 | N.S. | 4.209 | 1.893 | 9.359 |
|  |  | **Estimate** |  |  |  |  |  |
| **Emp subtype** |  |  |  |  |  |  |  |
| mort | 4 | Aliased |  |  |  |  |  |
| other | 24 | Aliased |  |  |  |  |  |
| **Midpoint age in RR** |  | -0.010 | 0.008 | N.S. | 5.659 | 1.433 | 22.341 |
| **RR adjusted for age** |  |  |  |  |  |  |  |
| Yes | 15 | Aliased |  |  | 3.550 | 2.771 | 4.547 |
| No | 13 | -0.332 | 0.260 | N.S. | 2.547 | 1.505 | 4.311 |
| **RR adjusted for factor other than sex, age** |  |  |  |  |  |  |  |
| Yes | 6 | Aliased |  |  | 2.179 | 1.510 | 3.145 |
| No | 22 | 0.843 | 0.267 | ++ | 5.064 | 3.330 | 7.702 |
| **Emp subtype (3)** |  |  |  |  |  |  |  |
| mort | 4 | Aliased |  |  | 5.375 | 2.032 | 14.215 |
| oth-prev | 22 | -0.547 | 0.384 | N.S. | 3.111 | 2.631 | 3.679 |
| oth-inc | 2 | -1.440 | 0.568 | - | 1.274 | 0.436 | 3.725 |

Table 3 - A - 6

IESCOPD - Meta-regression of ever smoking, any product (or cigarettes if all product not available)

Multiple regression of data from Table 3 - A - 1 (most-adjusted RRs)

Any emphysema

Test by adding extra variables one at a time

|  |  | **Deviance** | **(DF)** | **Drop Dev** | **P** |  |  |
| --- | --- | --- | --- | --- | --- | --- | --- |
| **Adding National cigarette type** |  | 30.382 | (14) | 3.269 | N.S. |  |  |
|  |  | **Estimate** | **S.E.** | **P** | **RR** | **95%CIl** | **95%CIu** |
| **Constant** |  | 1.736 | 0.650 | + | 5.673 | 1.587 | 20.280 |
| **Sex(RR)** |  |  |  |  |  |  |  |
| Male | 13 | Aliased |  |  | 2.809 | 1.879 | 4.199 |
| Female | 6 | -0.263 | 0.215 | N.S. | 2.160 | 1.140 | 4.090 |
| Combined | 9 | 0.242 | 0.189 | N.S. | 3.579 | 2.862 | 4.476 |
| **Continent** |  |  |  |  |  |  |  |
| NAmer | 14 | Aliased |  |  | 3.962 | 2.456 | 6.391 |
| Europe | 10 | -0.390 | 0.265 | N.S. | 2.683 | 1.893 | 3.801 |
| Asia | 4 | 0.126 | 0.192 | N.S. | 4.496 | 2.396 | 8.435 |
| **Asthma analysis type (Emp)** |  |  |  |  |  |  |  |
| inc-irresp | 26 | Aliased |  |  | 3.234 | 2.835 | 3.689 |
| excl-all | 2 | -0.351 | 0.457 | N.S. | 2.276 | 0.614 | 8.442 |
| **Smoking product (3)** |  |  |  |  |  |  |  |
| any | 11 | Aliased |  |  | 3.156 | 2.247 | 4.433 |
| cigs | 15 | 0.068 | 0.315 | N.S. | 3.379 | 1.832 | 6.232 |
| cigsonly | 2 | -0.195 | 0.407 | N.S. | 2.597 | 0.803 | 8.395 |
| **Unexposed group** |  |  |  |  |  |  |  |
| nev any | 14 | Aliased |  |  | 3.178 | 2.199 | 4.594 |
| nev cig | 14 | 0.042 | 0.394 | N.S. | 3.316 | 1.484 | 7.410 |
| **Emp subtype** |  |  |  |  |  |  |  |
| mort | 4 | Aliased |  |  | 5.353 | 1.948 | 14.707 |
| other | 24 | -0.567 | 0.387 | N.S. | 3.038 | 2.554 | 3.614 |
| **Midpoint age in RR** |  | -0.006 | 0.008 | N.S. | 4.459 | 1.151 | 17.274 |
| **RR adjusted for age** |  |  |  |  |  |  |  |
| Yes | 15 | Aliased |  |  | 3.325 | 2.533 | 4.366 |
| No | 13 | -0.110 | 0.281 | N.S. | 2.979 | 1.655 | 5.362 |
| **RR adjusted for factor other than sex, age** |  |  |  |  |  |  |  |
| Yes | 6 | Aliased |  |  | 2.304 | 1.518 | 3.495 |
| No | 22 | 0.723 | 0.296 | + | 4.747 | 2.944 | 7.653 |
| **National cigarette tobacco type** |  |  |  |  |  |  |  |
| bl | 26 | Aliased |  |  | 3.176 | 2.783 | 3.626 |
| vir | 2 | 0.765 | 0.423 | (+) | 6.829 | 2.041 | 22.855 |
|  |  | **Deviance** | **(DF)** | **Drop Dev** | **P** |  |  |
| **Adding Publication year** |  | 29.477 | (12) | 4.174 | N.S. |  |  |
|  |  | **Estimate** | **S.E.** | **P** | **RR** | **95%CIl** | **95%CIu** |
| **Constant** |  | 0.421 | 0.862 | N.S. | 1.523 | 0.281 | 8.253 |
| **Sex(RR)** |  |  |  |  |  |  |  |
| Male | 13 | Aliased |  |  | 2.801 | 1.781 | 4.406 |
| Female | 6 | -0.329 | 0.208 | N.S. | 2.017 | 1.042 | 3.904 |
| Combined | 9 | 0.256 | 0.201 | N.S. | 3.619 | 2.834 | 4.621 |
| **Continent** |  |  |  |  |  |  |  |
| NAmer | 14 | Aliased |  |  | 4.404 | 2.556 | 7.586 |
| Europe | 10 | -0.556 | 0.285 | (-) | 2.526 | 1.700 | 3.755 |
| Asia | 4 | 0.065 | 0.209 | N.S. | 4.698 | 2.268 | 9.731 |
| **Asthma analysis type (Emp)** |  |  |  |  |  |  |  |
| inc-irresp | 26 | Aliased |  |  | 3.226 | 2.803 | 3.713 |
| excl-all | 2 | -0.174 | 0.558 | N.S. | 2.712 | 0.496 | 14.837 |
| **Smoking product (3)** |  |  |  |  |  |  |  |
| any | 11 | Aliased |  |  | 3.417 | 2.372 | 4.921 |
| cigs | 15 | -0.162 | 0.316 | N.S. | 2.905 | 1.516 | 5.569 |
| cigsonly | 2 | -0.232 | 0.421 | N.S. | 2.708 | 0.775 | 9.467 |

Table 3 - A - 6

IESCOPD - Meta-regression of ever smoking, any product (or cigarettes if all product not available)

Multiple regression of data from Table 3 - A - 1 (most-adjusted RRs)

Any emphysema

Test by adding extra variables one at a time

|  |  | **Estimate** | **S.E.** | **P** | **RR** | **95%CIl** | **95%CIu** |
| --- | --- | --- | --- | --- | --- | --- | --- |
| **Unexposed group** |  |  |  |  |  |  |  |
| nev any | 14 | Aliased |  |  | 3.181 | 1.827 | 5.539 |
| nev cig | 14 | 0.039 | 0.577 | N.S. | 3.309 | 0.953 | 11.487 |
| **Emp subtype** |  |  |  |  |  |  |  |
| mort | 4 | Aliased |  |  | 3.752 | 1.177 | 11.961 |
| other | 24 | -0.170 | 0.417 | N.S. | 3.164 | 2.613 | 3.830 |
| **Midpoint age in RR** |  | 0.017 | 0.013 | N.S. | 1.177 | 0.121 | 11.405 |
| **RR adjusted for age** |  |  |  |  |  |  |  |
| Yes | 15 | Aliased |  |  | 3.373 | 2.411 | 4.719 |
| No | 13 | -0.158 | 0.338 | N.S. | 2.879 | 1.365 | 6.073 |
| **RR adjusted for factor other than sex, age** |  |  |  |  |  |  |  |
| Yes | 6 | Aliased |  |  | 2.273 | 1.194 | 4.328 |
| No | 22 | 0.752 | 0.442 | N.S. | 4.822 | 2.294 | 10.137 |
| **Publication year** |  |  |  |  |  |  |  |
| <1980 | 13 | Aliased |  |  | 3.931 | 1.952 | 7.917 |
| 1980-89 | 5 | -0.222 | 0.342 | N.S. | 3.148 | 1.610 | 6.154 |
| 1990-99 | 3 | -0.798 | 0.575 | N.S. | 1.771 | 0.437 | 7.167 |
| 2000+ | 7 | -0.057 | 0.467 | N.S. | 3.714 | 1.061 | 13.001 |
|  |  | **Deviance** | **(DF)** | **Drop Dev** | **P** |  |  |
| **Adding Study type** |  | 28.309 | (14) | 5.341 | N.S. |  |  |
|  |  | **Estimate** | **S.E.** | **P** | **RR** | **95%CIl** | **95%CIu** |
| **Constant** |  | 1.949 | 0.667 | + | 7.024 | 1.902 | 25.947 |
| **Sex(RR)** |  |  |  |  |  |  |  |
| Male | 13 | Aliased |  |  | 2.749 | 1.871 | 4.039 |
| Female | 6 | -0.377 | 0.207 | (-) | 1.886 | 1.060 | 3.356 |
| Combined | 9 | 0.291 | 0.186 | N.S. | 3.679 | 2.982 | 4.538 |
| **Continent** |  |  |  |  |  |  |  |
| NAmer | 14 | Aliased |  |  | 4.189 | 2.624 | 6.685 |
| Europe | 10 | -0.426 | 0.266 | N.S. | 2.735 | 1.953 | 3.830 |
| Asia | 4 | -0.102 | 0.215 | N.S. | 3.783 | 2.000 | 7.157 |
| **Asthma analysis type (Emp)** |  |  |  |  |  |  |  |
| inc-irresp | 26 | Aliased |  |  | 3.223 | 2.838 | 3.660 |
| excl-all | 2 | -0.079 | 0.478 | N.S. | 2.979 | 0.795 | 11.161 |
| **Smoking product (3)** |  |  |  |  |  |  |  |
| any | 11 | Aliased |  |  | 3.210 | 2.320 | 4.440 |
| cigs | 15 | 0.006 | 0.310 | N.S. | 3.228 | 1.806 | 5.767 |
| cigsonly | 2 | 0.066 | 0.394 | N.S. | 3.427 | 1.163 | 10.101 |
| **Unexposed group** |  |  |  |  |  |  |  |
| nev any | 14 | Aliased |  |  | 2.866 | 1.990 | 4.128 |
| nev cig | 14 | 0.384 | 0.406 | N.S. | 4.209 | 1.893 | 9.359 |
| **Emp subtype** |  |  |  |  |  |  |  |
| mort | 4 | Aliased |  |  | 11.722 | 2.813 | 48.841 |
| other | 24 | -1.440 | 0.568 | - | 2.778 | 2.262 | 3.412 |
| **Midpoint age in RR** |  | -0.010 | 0.008 | N.S. | 5.659 | 1.433 | 22.341 |
| **RR adjusted for age** |  |  |  |  |  |  |  |
| Yes | 15 | Aliased |  |  | 3.550 | 2.771 | 4.547 |
| No | 13 | -0.332 | 0.260 | N.S. | 2.547 | 1.505 | 4.311 |
| **RR adjusted for factor other than sex, age** |  |  |  |  |  |  |  |
| Yes | 6 | Aliased |  |  | 2.179 | 1.510 | 3.145 |
| No | 22 | 0.843 | 0.267 | ++ | 5.064 | 3.330 | 7.702 |
| **Study type** |  |  |  |  |  |  |  |
| Pr | 6 | Aliased |  |  | 1.476 | 0.572 | 3.812 |
| CS | 22 | 0.893 | 0.386 | + | 3.605 | 2.994 | 4.342 |

Table 3 - A - 6

IESCOPD - Meta-regression of ever smoking, any product (or cigarettes if all product not available)

Multiple regression of data from Table 3 - A - 1 (most-adjusted RRs)

Any emphysema

Test by adding extra variables one at a time

|  |  | **Deviance** | **(DF)** | **Drop Dev** | **P** |  |  |
| --- | --- | --- | --- | --- | --- | --- | --- |
| **Adding Study weakness** |  | 33.513 | (14) | 0.137 | N.S. |  |  |
|  |  | **Estimate** | **S.E.** | **P** | **RR** | **95%CIl** | **95%CIu** |
| **Constant** |  | 1.190 | 0.818 | N.S. | 3.288 | 0.662 | 16.335 |
| **Sex(RR)** |  |  |  |  |  |  |  |
| Male | 13 | Aliased |  |  | 2.744 | 1.787 | 4.214 |
| Female | 6 | -0.383 | 0.210 | (-) | 1.872 | 1.000 | 3.505 |
| Combined | 9 | 0.295 | 0.190 | N.S. | 3.685 | 2.927 | 4.640 |
| **Continent** |  |  |  |  |  |  |  |
| NAmer | 14 | Aliased |  |  | 4.121 | 2.201 | 7.718 |
| Europe | 10 | -0.454 | 0.336 | N.S. | 2.617 | 1.678 | 4.081 |
| Asia | 4 | 0.112 | 0.193 | N.S. | 4.612 | 2.228 | 9.546 |
| **Asthma analysis type (Emp)** |  |  |  |  |  |  |  |
| inc-irresp | 26 | Aliased |  |  | 3.228 | 2.807 | 3.712 |
| excl-all | 2 | -0.208 | 0.697 | N.S. | 2.620 | 0.323 | 21.245 |
| **Smoking product (3)** |  |  |  |  |  |  |  |
| any | 11 | Aliased |  |  | 3.281 | 2.301 | 4.679 |
| cigs | 15 | -0.052 | 0.311 | N.S. | 3.114 | 1.654 | 5.865 |
| cigsonly | 2 | -0.065 | 0.426 | N.S. | 3.074 | 0.874 | 10.808 |
| **Unexposed group** |  |  |  |  |  |  |  |
| nev any | 14 | Aliased |  |  | 3.162 | 2.070 | 4.831 |
| nev cig | 14 | 0.059 | 0.437 | N.S. | 3.355 | 1.319 | 8.533 |
| **Emp subtype** |  |  |  |  |  |  |  |
| mort | 4 | Aliased |  |  | 4.492 | 1.254 | 16.093 |
| other | 24 | -0.371 | 0.466 | N.S. | 3.099 | 2.539 | 3.784 |
| **Midpoint age in RR** |  | -0.002 | 0.008 | N.S. | 3.655 | 0.900 | 14.843 |
| **RR adjusted for age** |  |  |  |  |  |  |  |
| Yes | 15 | Aliased |  |  | 3.530 | 2.695 | 4.623 |
| No | 13 | -0.313 | 0.260 | N.S. | 2.582 | 1.455 | 4.581 |
| **RR adjusted for factor other than sex, age** |  |  |  |  |  |  |  |
| Yes | 6 | Aliased |  |  | 2.034 | 1.371 | 3.019 |
| No | 22 | 0.992 | 0.264 | ++ | 5.484 | 3.494 | 8.608 |
| **Study weakness** |  |  |  |  |  |  |  |
| Yes | 3 | Aliased |  |  | 2.730 | 0.702 | 10.609 |
| No | 25 | 0.171 | 0.462 | N.S. | 3.239 | 2.799 | 3.748 |
|  |  | **Deviance** | **(DF)** | **Drop Dev** | **P** |  |  |
| **Adding Number of cases (Emp)** |  | 19.608 | (12) | 14.042 | (*) |  |  |
|  |  | **Estimate** | **S.E.** | **P** | **RR** | **95%CIl** | **95%CIu** |
| **Constant** |  | 2.238 | 0.742 | + | 9.370 | 2.188 | 40.137 |
| **Sex(RR)** |  |  |  |  |  |  |  |
| Male | 13 | Aliased |  |  | 3.334 | 2.293 | 4.848 |
| Female | 6 | -0.161 | 0.215 | N.S. | 2.839 | 1.566 | 5.150 |
| Combined | 9 | -0.028 | 0.208 | N.S. | 3.241 | 2.627 | 3.999 |
| **Continent** |  |  |  |  |  |  |  |
| NAmer | 14 | Aliased |  |  | 2.199 | 1.220 | 3.961 |
| Europe | 10 | 0.533 | 0.368 | N.S. | 3.748 | 2.564 | 5.477 |
| Asia | 4 | 0.470 | 0.245 | (+) | 3.517 | 1.922 | 6.435 |
| **Asthma analysis type (Emp)** |  |  |  |  |  |  |  |
| inc-irresp | 26 | Aliased |  |  | 3.268 | 2.915 | 3.665 |
| excl-all | 2 | -1.208 | 0.534 | - | 0.976 | 0.259 | 3.675 |
| **Smoking product (3)** |  |  |  |  |  |  |  |
| any | 11 | Aliased |  |  | 3.153 | 2.355 | 4.223 |
| cigs | 15 | 0.054 | 0.311 | N.S. | 3.329 | 1.974 | 5.614 |
| cigsonly | 2 | 0.126 | 0.410 | N.S. | 3.576 | 1.303 | 9.813 |
| **Unexposed group** |  |  |  |  |  |  |  |
| nev any | 14 | Aliased |  |  | 2.610 | 1.861 | 3.660 |
| nev cig | 14 | 0.694 | 0.420 | N.S. | 5.222 | 2.485 | 10.976 |

Table 3 - A - 6

IESCOPD - Meta-regression of ever smoking, any product (or cigarettes if all product not available)

Multiple regression of data from Table 3 - A - 1 (most-adjusted RRs)

Any emphysema

Test by adding extra variables one at a time

|  |  | **Estimate** | **S.E.** | **P** | **RR** | **95%CIl** | **95%CIu** |
| --- | --- | --- | --- | --- | --- | --- | --- |
| **Emp subtype** |  |  |  |  |  |  |  |
| mort | 4 | Aliased |  |  | 14.338 | 4.052 | 50.740 |
| other | 24 | -1.664 | 0.560 | - | 2.715 | 2.261 | 3.260 |
| **Midpoint age in RR** |  | -0.006 | 0.008 | N.S. | 4.503 | 1.325 | 15.304 |
| **RR adjusted for age** |  |  |  |  |  |  |  |
| Yes | 15 | Aliased |  |  | 3.110 | 2.359 | 4.101 |
| No | 13 | 0.118 | 0.342 | N.S. | 3.498 | 1.890 | 6.473 |
| **RR adjusted for factor other than sex, age** |  |  |  |  |  |  |  |
| Yes | 6 | Aliased |  |  | 2.829 | 1.745 | 4.587 |
| No | 22 | 0.279 | 0.405 | N.S. | 3.740 | 2.143 | 6.528 |
| **Number of cases (Emp)** |  |  |  |  |  |  |  |
| 1-50 | 6 | Aliased |  |  | 3.090 | 1.486 | 6.426 |
| 51-100 | 9 | -0.150 | 0.397 | N.S. | 2.659 | 1.554 | 4.550 |
| 101-200 | 5 | 1.043 | 0.437 | + | 8.765 | 4.101 | 18.734 |
| 201+ | 8 | -0.032 | 0.295 | N.S. | 2.991 | 2.565 | 3.489 |
|  |  | **Deviance** | **(DF)** | **Drop Dev** | **P** |  |  |
| **Adding Smoking results reported in study (Emp)** |  | 33.298 | (14) | 0.353 | N.S. |  |  |
|  |  | **Estimate** | **S.E.** | **P** | **RR** | **95%CIl** | **95%CIu** |
| **Constant** |  | 1.452 | 0.630 | + | 4.270 | 1.242 | 14.681 |
| **Sex(RR)** |  |  |  |  |  |  |  |
| Male | 13 | Aliased |  |  | 2.798 | 1.792 | 4.369 |
| Female | 6 | -0.404 | 0.215 | (-) | 1.867 | 0.999 | 3.490 |
| Combined | 9 | 0.269 | 0.198 | N.S. | 3.662 | 2.901 | 4.624 |
| **Continent** |  |  |  |  |  |  |  |
| NAmer | 14 | Aliased |  |  | 3.889 | 2.348 | 6.439 |
| Europe | 10 | -0.341 | 0.272 | N.S. | 2.766 | 1.881 | 4.066 |
| Asia | 4 | 0.061 | 0.217 | N.S. | 4.134 | 1.945 | 8.788 |
| **Asthma analysis type (Emp)** |  |  |  |  |  |  |  |
| inc-irresp | 26 | Aliased |  |  | 3.232 | 2.816 | 3.710 |
| excl-all | 2 | -0.313 | 0.481 | N.S. | 2.363 | 0.558 | 10.007 |
| **Smoking product (3)** |  |  |  |  |  |  |  |
| any | 11 | Aliased |  |  | 3.408 | 2.253 | 5.154 |
| cigs | 15 | -0.167 | 0.376 | N.S. | 2.883 | 1.348 | 6.165 |
| cigsonly | 2 | 0.016 | 0.395 | N.S. | 3.461 | 1.028 | 11.654 |
| **Unexposed group** |  |  |  |  |  |  |  |
| nev any | 14 | Aliased |  |  | 2.932 | 1.830 | 4.697 |
| nev cig | 14 | 0.310 | 0.493 | N.S. | 3.995 | 1.401 | 11.395 |
| **Emp subtype** |  |  |  |  |  |  |  |
| mort | 4 | Aliased |  |  | 5.187 | 1.752 | 15.357 |
| other | 24 | -0.531 | 0.397 | N.S. | 3.049 | 2.537 | 3.664 |
| **Midpoint age in RR** |  | 0.000 | 0.009 | N.S. | 3.177 | 0.635 | 15.902 |
| **RR adjusted for age** |  |  |  |  |  |  |  |
| Yes | 15 | Aliased |  |  | 3.531 | 2.699 | 4.619 |
| No | 13 | -0.314 | 0.260 | N.S. | 2.579 | 1.457 | 4.566 |
| **RR adjusted for factor other than sex, age** |  |  |  |  |  |  |  |
| Yes | 6 | Aliased |  |  | 2.165 | 1.338 | 3.505 |
| No | 22 | 0.857 | 0.330 | + | 5.101 | 2.934 | 8.870 |
| **Smoking results reported in study (Emp)** |  |  |  |  |  |  |  |
| Ever Only | 11 | Aliased |  |  | 3.585 | 2.039 | 6.302 |
| Current Only | 0 | Aliased |  |  | 3.585 | 2.039 | 6.302 |
| Both | 17 | -0.156 | 0.262 | N.S. | 3.068 | 2.318 | 4.061 |

Table 3 - A - 6

IESCOPD - Meta-regression of ever smoking, any product (or cigarettes if all product not available)

Multiple regression of data from Table 3 - A - 1 (most-adjusted RRs)

Any emphysema

Test by adding extra variables one at a time

|  |  | **Deviance** | **(DF)** | **Drop Dev** | **P** |  |  |
| --- | --- | --- | --- | --- | --- | --- | --- |
| **Adding Derivation of RR/CI** |  | 28.354 | (14) | 5.296 | N.S. |  |  |
|  |  | **Estimate** | **S.E.** | **P** | **RR** | **95%CIl** | **95%CIu** |
| **Constant** |  | 1.940 | 0.666 | + | 6.957 | 1.888 | 25.639 |
| **Sex(RR)** |  |  |  |  |  |  |  |
| Male | 13 | Aliased |  |  | 2.368 | 1.558 | 3.599 |
| Female | 6 | -0.202 | 0.219 | N.S. | 1.935 | 1.085 | 3.450 |
| Combined | 9 | 0.487 | 0.201 | + | 3.855 | 3.107 | 4.784 |
| **Continent** |  |  |  |  |  |  |  |
| NAmer | 14 | Aliased |  |  | 3.994 | 2.515 | 6.340 |
| Europe | 10 | -0.360 | 0.265 | N.S. | 2.785 | 1.985 | 3.908 |
| Asia | 4 | -0.040 | 0.204 | N.S. | 3.839 | 2.038 | 7.229 |
| **Asthma analysis type (Emp)** |  |  |  |  |  |  |  |
| inc-irresp | 26 | Aliased |  |  | 3.228 | 2.842 | 3.666 |
| excl-all | 2 | -0.212 | 0.464 | N.S. | 2.612 | 0.723 | 9.438 |
| **Smoking product (3)** |  |  |  |  |  |  |  |
| any | 11 | Aliased |  |  | 3.169 | 2.287 | 4.391 |
| cigs | 15 | 0.038 | 0.311 | N.S. | 3.292 | 1.838 | 5.897 |
| cigsonly | 2 | 0.158 | 0.400 | N.S. | 3.713 | 1.246 | 11.064 |
| **Unexposed group** |  |  |  |  |  |  |  |
| nev any | 14 | Aliased |  |  | 2.961 | 2.072 | 4.233 |
| nev cig | 14 | 0.276 | 0.396 | N.S. | 3.903 | 1.788 | 8.519 |
| **Emp subtype** |  |  |  |  |  |  |  |
| mort | 4 | Aliased |  |  | 6.056 | 2.228 | 16.464 |
| other | 24 | -0.704 | 0.396 | (-) | 2.995 | 2.528 | 3.549 |
| **Midpoint age in RR** |  | -0.003 | 0.008 | N.S. | 3.875 | 1.077 | 13.938 |
| **RR adjusted for age** |  |  |  |  |  |  |  |
| Yes | 15 | Aliased |  |  | 3.604 | 2.809 | 4.624 |
| No | 13 | -0.384 | 0.262 | N.S. | 2.456 | 1.445 | 4.174 |
| **RR adjusted for factor other than sex, age** |  |  |  |  |  |  |  |
| Yes | 6 | Aliased |  |  | 2.146 | 1.491 | 3.088 |
| No | 22 | 0.876 | 0.264 | ++ | 5.155 | 3.401 | 7.812 |
| **Derivation of RR/CI** |  |  |  |  |  |  |  |
| Orig/2x2 | 4 | Aliased |  |  | 4.956 | 2.894 | 8.485 |
| Other | 24 | -0.487 | 0.212 | - | 3.045 | 2.639 | 3.514 |
|  |  | **Deviance** | **(DF)** | **Drop Dev** | **P** |  |  |
| **Adding Analysis type** |  | 28.309 | (14) | 5.341 | N.S. |  |  |
|  |  | **Estimate** | **S.E.** | **P** | **RR** | **95%CIl** | **95%CIu** |
| **Constant** |  | 2.842 | 0.884 | ++ | 17.156 | 3.033 | 97.039 |
| **Sex(RR)** |  |  |  |  |  |  |  |
| Male | 13 | Aliased |  |  | 2.749 | 1.871 | 4.039 |
| Female | 6 | -0.377 | 0.207 | (-) | 1.886 | 1.060 | 3.356 |
| Combined | 9 | 0.291 | 0.186 | N.S. | 3.679 | 2.982 | 4.538 |
| **Continent** |  |  |  |  |  |  |  |
| NAmer | 14 | Aliased |  |  | 4.189 | 2.624 | 6.685 |
| Europe | 10 | -0.426 | 0.266 | N.S. | 2.735 | 1.953 | 3.830 |
| Asia | 4 | -0.102 | 0.215 | N.S. | 3.783 | 2.000 | 7.157 |
| **Asthma analysis type (Emp)** |  |  |  |  |  |  |  |
| inc-irresp | 26 | Aliased |  |  | 3.223 | 2.838 | 3.660 |
| excl-all | 2 | -0.079 | 0.478 | N.S. | 2.979 | 0.795 | 11.161 |
| **Smoking product (3)** |  |  |  |  |  |  |  |
| any | 11 | Aliased |  |  | 3.210 | 2.320 | 4.440 |
| cigs | 15 | 0.006 | 0.310 | N.S. | 3.228 | 1.806 | 5.767 |
| cigsonly | 2 | 0.066 | 0.394 | N.S. | 3.427 | 1.163 | 10.101 |
| **Unexposed group** |  |  |  |  |  |  |  |
| nev any | 14 | Aliased |  |  | 2.866 | 1.990 | 4.128 |
| nev cig | 14 | 0.384 | 0.406 | N.S. | 4.209 | 1.893 | 9.359 |
| **Emp subtype** |  |  |  |  |  |  |  |
| mort | 4 | Aliased |  |  | 11.722 | 2.813 | 48.841 |
| other | 24 | -1.440 | 0.568 | - | 2.778 | 2.262 | 3.412 |
| **Midpoint age in RR** |  | -0.010 | 0.008 | N.S. | 5.659 | 1.433 | 22.341 |

Table 3 - A - 6

IESCOPD - Meta-regression of ever smoking, any product (or cigarettes if all product not available)

Multiple regression of data from Table 3 - A - 1 (most-adjusted RRs)

Any emphysema

Test by adding extra variables one at a time

|  |  | **Estimate** | **S.E.** | **P** | **RR** | **95%CIl** | **95%CIu** |
| --- | --- | --- | --- | --- | --- | --- | --- |
| **RR adjusted for age** |  |  |  |  |  |  |  |
| Yes | 15 | Aliased |  |  | 3.550 | 2.771 | 4.547 |
| No | 13 | -0.332 | 0.260 | N.S. | 2.547 | 1.505 | 4.311 |
| **RR adjusted for factor other than sex, age** |  |  |  |  |  |  |  |
| Yes | 6 | Aliased |  |  | 2.179 | 1.510 | 3.145 |
| No | 22 | 0.843 | 0.267 | ++ | 5.064 | 3.330 | 7.702 |
| **Analysis type** |  |  |  |  |  |  |  |
| prevlnce | 22 | Aliased |  |  | 3.605 | 2.994 | 4.342 |
| onset | 6 | -0.893 | 0.386 | - | 1.476 | 0.572 | 3.812 |

Table 3 - A - 6

IESCOPD - Meta-regression of ever smoking, any product (or cigarettes if all product not available)

Multiple regression of data from Table 3 - A - 1 (most-adjusted RRs)

Any emphysema

Fitted values and residuals

|  | **Study Ref** | **NRR** | **SEX** | **LOGRR** | **FITVAL** | **SEFITV** | **STDRES** |
| --- | --- | --- | --- | --- | --- | --- | --- |
|  |  |  |  |  |  |  |  |
| #1733 | ENRIGH | 9 | m | 0.942 | 1.474 | 0.351 | -1.517 |
| #342 | WANG2 | 1 | b | 1.710 | 1.974 | 0.255 | -1.033 |
| #2858 | GULSVI | 6 | f | 0.599 | 1.043 | 0.460 | -0.964 |
| #1693 | DONTA2 | 13 | m | 0.048 | 0.763 | 0.764 | -0.937 |
| #3576 | SILVA | 9 | b | 0.742 | 1.171 | 0.492 | -0.872 |
| #2856 | GULSVI | 4 | m | 0.863 | 1.412 | 0.769 | -0.715 |
| #3066 | HUHTI1 | 145 | f | 0.678 | 1.017 | 0.966 | -0.351 |
| #3296 | HO | 12 | b | 1.054 | 1.229 | 0.526 | -0.333 |
| #244 | SUTINE | 1 | b | 1.285 | 1.379 | 0.486 | -0.192 |
| #2558 | PRATT | 1 | m | 1.375 | 1.446 | 0.434 | -0.164 |
| #2870 | HARDIE | 18 | f | 0.936 | 1.028 | 0.569 | -0.162 |
| #3605 | BEST | 20 | m | 2.137 | 2.201 | 0.817 | -0.079 |
| #3277 | KAHN2 | 80 | m | 2.209 | 2.222 | 0.355 | -0.034 |
| #2818 | HAMMO2 | 7 | m | 2.156 | 2.156 | 0.335 | -0.000 |
| #2630 | WEISS | 16 | m | 1.480 | 1.441 | 1.508 | 0.026 |
| #1460 | ANDER2 | 1 | b | 1.783 | 1.751 | 0.584 | 0.055 |
| #2837 | HAMMO2 | 8 | f | 1.863 | 1.827 | 0.440 | 0.081 |
| #1526 | LAVECC | 30 | b | 0.718 | 0.707 | 0.096 | 0.107 |
| #2852 | LEBOWI | 36 | b | 1.581 | 1.181 | 0.720 | 0.556 |
| #1998 | MILLER | 12 | f | 1.797 | 0.190 | 2.873 | 0.559 |
| #1995 | MILLER | 9 | m | 1.881 | 0.560 | 2.142 | 0.617 |
| #1736 | ENRIGH | 12 | f | 1.373 | 1.105 | 0.340 | 0.789 |
| #338 | OMORI | 6 | m | 2.275 | 1.568 | 0.760 | 0.931 |
| #332 | NAWA | 1 | m | 1.926 | 1.653 | 0.272 | 1.002 |
| #2867 | HARDIE | 15 | m | 2.594 | 1.398 | 1.057 | 1.132 |
| #2959 | HUHTI1 | 69 | m | 2.808 | 1.081 | 1.486 | 1.163 |
| #2926 | HOZAWA | 9 | b | 2.360 | 1.855 | 0.423 | 1.196 |
| #2571 | RYDER | 3 | b | 2.459 | 1.682 | 0.614 | 1.265 |

## Table 3 - B - 6

IESCOPD - Meta-regression of current smoking, any product (or cigarettes if all product not available)

Multiple regression of data from Table 3 - B - 1 (most-adjusted RRs)

Any emphysema

Fixed model

|  |  | **Deviance** | **(DF)** |  |  |  |  |
| --- | --- | --- | --- | --- | --- | --- | --- |
| **Fixed model** |  | 12.949 | (9) |  |  |  |  |
|  |  | **Estimate** | **S.E.** | **P** | **RR** | **95%CIl** | **95%CIu** |
| **Constant** |  | 2.730 | 1.371 | (+) | 15.333 | 1.043 | 225.344 |
| **Sex(RR)** |  |  |  |  |  |  |  |
| Male | 13 | Aliased |  |  | 14.799 | 1.896 | 115.519 |
| Female | 5 | -0.264 | 0.265 | N.S. | 11.368 | 1.419 | 91.073 |
| Combined | 4 | -2.534 | 1.288 | (-) | 1.174 | 0.436 | 3.161 |
| **Continent** |  |  |  |  |  |  |  |
| NAmer | 10 | Aliased |  |  | 7.148 | 4.370 | 11.694 |
| Europe | 7 | -1.395 | 0.307 | -- | 1.771 | 1.285 | 2.441 |
| Asia | 5 | -0.681 | 0.383 | N.S. | 3.617 | 1.351 | 9.682 |
| **Asthma analysis type (Emp)** |  |  |  |  |  |  |  |
| inc-irresp | 19 | Aliased |  |  | 2.602 | 2.252 | 3.006 |
| excl-all | 3 | 0.151 | 0.768 | N.S. | 3.025 | 0.515 | 17.774 |
| **Smoking product (3)** |  |  |  |  |  |  |  |
| any | 5 | Aliased |  |  | 1.289 | 0.726 | 2.287 |
| cigs | 16 | 2.337 | 0.782 | + | 13.337 | 3.670 | 48.465 |
| cigsonly | 1 | -0.138 | 0.663 | N.S. | 1.123 | 0.215 | 5.873 |
| **Unexposed group** |  |  |  |  |  |  |  |
| nev any | 7 | Aliased |  |  | 6.055 | 3.572 | 10.265 |
| nev cig | 15 | -2.966 | 0.764 | -- | 0.312 | 0.086 | 1.137 |
| **Emp subtype** |  |  |  |  |  |  |  |
| mort | 5 | Aliased |  |  | 0.648 | 0.181 | 2.327 |
| other | 17 | 1.684 | 0.653 | + | 3.493 | 2.589 | 4.714 |
| **Midpoint age in RR** |  | 0.002 | 0.019 | N.S. | 2.381 | 0.153 | 37.122 |
| **RR adjusted for age** |  |  |  |  |  |  |  |
| Yes | 13 | Aliased |  |  | 3.111 | 2.619 | 3.694 |
| No | 9 | -1.720 | 0.424 | -- | 0.557 | 0.225 | 1.378 |
| **RR adjusted for factor other than sex, age** |  |  |  |  |  |  |  |
| Yes | 6 | Aliased |  |  | 3.214 | 1.197 | 8.633 |
| No | 16 | -0.645 | 1.291 | N.S. | 1.686 | 0.215 | 13.243 |

Table 3 - B - 6

IESCOPD - Meta-regression of current smoking, any product (or cigarettes if all product not available)

Multiple regression of data from Table 3 - B - 1 (most-adjusted RRs)

Any emphysema

Test by removing variables one at a time

|  |  | **Deviance** | **(DF)** | **Drop Dev** | **P** |  |  |
| --- | --- | --- | --- | --- | --- | --- | --- |
| **Omitting Sex (RR)** |  | 17.520 | (11) | -4.571 | N.S. |  |  |
|  |  | **Estimate** | **S.E.** | **P** | **RR** | **95%CIl** | **95%CIu** |
| **Constant** |  | 0.678 | 0.832 | N.S. | 1.971 | 0.386 | 10.063 |
| **RR adjusted for factor other than sex, age** |  |  |  |  |  |  |  |
| Yes | 6 | Aliased |  |  | 1.586 | 1.078 | 2.334 |
| No | 16 | 1.546 | 0.533 | + | 7.445 | 3.418 | 16.216 |
| **Continent** |  |  |  |  |  |  |  |
| NAmer | 10 | Aliased |  |  | 7.390 | 4.790 | 11.401 |
| Europe | 7 | -1.435 | 0.300 | --- | 1.760 | 1.321 | 2.346 |
| Asia | 5 | -0.738 | 0.382 | (-) | 3.534 | 1.454 | 8.588 |
| **Asthma analysis type (Emp)** |  |  |  |  |  |  |  |
| inc-irresp | 19 | Aliased |  |  | 2.593 | 2.276 | 2.954 |
| excl-all | 3 | 0.303 | 0.746 | N.S. | 3.513 | 0.740 | 16.671 |
| **Smoking product (3)** |  |  |  |  |  |  |  |
| any | 5 | Aliased |  |  | 1.176 | 0.706 | 1.960 |
| cigs | 16 | 2.637 | 0.770 | ++ | 16.434 | 5.212 | 51.822 |
| cigsonly | 1 | -0.084 | 0.662 | N.S. | 1.082 | 0.242 | 4.827 |
| **Unexposed group** |  |  |  |  |  |  |  |
| nev any | 7 | Aliased |  |  | 6.488 | 4.059 | 10.370 |
| nev cig | 15 | -3.210 | 0.749 | -- | 0.262 | 0.083 | 0.826 |
| **Emp subtype** |  |  |  |  |  |  |  |
| mort | 5 | Aliased |  |  | 0.807 | 0.259 | 2.511 |
| other | 17 | 1.420 | 0.641 | + | 3.337 | 2.555 | 4.359 |
| **Midpoint age in RR** |  | -0.002 | 0.019 | N.S. | 2.865 | 0.242 | 33.989 |
|  |  | **Estimate** | **S.E.** | **P** | **RR** | **95%CIl** | **95%CIu** |
| **RR adjusted for age** |  |  |  |  |  |  |  |
| Yes | 13 | Aliased |  |  | 3.034 | 2.603 | 3.536 |
| No | 9 | -1.475 | 0.406 | -- | 0.694 | 0.316 | 1.521 |

Table 3 - B - 6

IESCOPD - Meta-regression of current smoking, any product (or cigarettes if all product not available)

Multiple regression of data from Table 3 - B - 1 (most-adjusted RRs)

Any emphysema

Test by removing variables one at a time

|  |  | **Deviance** | **(DF)** | **Drop Dev** | **P** |  |  |
| --- | --- | --- | --- | --- | --- | --- | --- |
| **Omitting Continent** |  | 39.738 | (11) | -26.790 | ** |  |  |
|  |  | **Estimate** | **S.E.** | **P** | **RR** | **95%CIl** | **95%CIu** |
| **Constant** |  | 2.989 | 1.360 | (+) | 19.870 | 1.382 | 285.669 |
| **RR adjusted for factor other than sex, age** |  |  |  |  |  |  |  |
| Yes | 6 | Aliased |  |  | 2.777 | 1.148 | 6.721 |
| No | 16 | -0.192 | 1.276 | N.S. | 2.293 | 0.363 | 14.481 |
| **Sex(RR)** |  |  |  |  |  |  |  |
| Male | 13 | Aliased |  |  | 15.029 | 2.349 | 96.170 |
| Female | 5 | -0.588 | 0.257 | - | 8.350 | 1.281 | 54.444 |
| Combined | 4 | -2.513 | 1.286 | (-) | 1.217 | 0.498 | 2.978 |
| **Asthma analysis type (Emp)** |  |  |  |  |  |  |  |
| inc-irresp | 19 | Aliased |  |  | 2.681 | 2.356 | 3.051 |
| excl-all | 3 | -1.206 | 0.663 | (-) | 0.802 | 0.201 | 3.205 |
| **Smoking product (3)** |  |  |  |  |  |  |  |
| any | 5 | Aliased |  |  | 0.971 | 0.625 | 1.508 |
| cigs | 16 | 3.247 | 0.656 | +++ | 24.956 | 9.358 | 66.550 |
| cigsonly | 1 | 0.679 | 0.637 | N.S. | 1.915 | 0.467 | 7.853 |
| **Unexposed group** |  |  |  |  |  |  |  |
| nev any | 7 | Aliased |  |  | 6.304 | 3.966 | 10.019 |
| nev cig | 15 | -3.108 | 0.740 | -- | 0.282 | 0.091 | 0.875 |
| **Emp subtype** |  |  |  |  |  |  |  |
| mort | 5 | Aliased |  |  | 0.790 | 0.336 | 1.855 |
| other | 17 | 1.446 | 0.480 | + | 3.352 | 2.699 | 4.163 |
| **Midpoint age in RR** |  | -0.023 | 0.018 | N.S. | 10.264 | 1.088 | 96.797 |
| **RR adjusted for age** |  |  |  |  |  |  |  |
| Yes | 13 | Aliased |  |  | 2.893 | 2.484 | 3.370 |
| No | 9 | -1.009 | 0.401 | - | 1.054 | 0.485 | 2.291 |
|  |  | **Deviance** | **(DF)** | **Drop Dev** | **P** |  |  |
| **Omitting Asthma analysis type (Emp)** |  | 12.987 | (10) | -0.039 | N.S. |  |  |
|  |  | **Estimate** | **S.E.** | **P** | **RR** | **95%CIl** | **95%CIu** |
| **Constant** |  | 2.809 | 1.310 | (+) | 16.600 | 1.273 | 216.466 |
| **RR adjusted for factor other than sex, age** |  |  |  |  |  |  |  |
| Yes | 6 | Aliased |  |  | 3.138 | 1.280 | 7.694 |
| No | 16 | -0.571 | 1.234 | N.S. | 1.774 | 0.274 | 11.500 |
| **Sex(RR)** |  |  |  |  |  |  |  |
| Male | 13 | Aliased |  |  | 14.819 | 2.110 | 104.090 |
| Female | 5 | -0.276 | 0.257 | N.S. | 11.246 | 1.568 | 80.658 |
| Combined | 4 | -2.534 | 1.288 | (-) | 1.175 | 0.459 | 3.007 |
| **Continent** |  |  |  |  |  |  |  |
| NAmer | 10 | Aliased |  |  | 7.081 | 4.495 | 11.155 |
| Europe | 7 | -1.388 | 0.305 | -- | 1.767 | 1.305 | 2.394 |
| Asia | 5 | -0.646 | 0.339 | (-) | 3.711 | 1.527 | 9.016 |
| **Smoking product (3)** |  |  |  |  |  |  |  |
| any | 5 | Aliased |  |  | 1.315 | 0.802 | 2.155 |
| cigs | 16 | 2.269 | 0.703 | ++ | 12.721 | 4.235 | 38.211 |
| cigsonly | 1 | -0.109 | 0.646 | N.S. | 1.180 | 0.272 | 5.115 |
| **Unexposed group** |  |  |  |  |  |  |  |
| nev any | 7 | Aliased |  |  | 5.905 | 3.914 | 8.909 |
| nev cig | 15 | -2.877 | 0.616 | --- | 0.332 | 0.123 | 0.897 |
| **Emp subtype** |  |  |  |  |  |  |  |
| mort | 5 | Aliased |  |  | 0.607 | 0.233 | 1.585 |
| other | 17 | 1.763 | 0.515 | ++ | 3.541 | 2.791 | 4.494 |
| **Midpoint age in RR** |  | -0.001 | 0.013 | N.S. | 2.835 | 0.523 | 15.365 |
| **RR adjusted for age** |  |  |  |  |  |  |  |
| Yes | 13 | Aliased |  |  | 3.114 | 2.646 | 3.664 |
| No | 9 | -1.730 | 0.421 | -- | 0.552 | 0.235 | 1.296 |

Table 3 - B - 6

IESCOPD - Meta-regression of current smoking, any product (or cigarettes if all product not available)

Multiple regression of data from Table 3 - B - 1 (most-adjusted RRs)

Any emphysema

Test by removing variables one at a time

|  |  | **Deviance** | **(DF)** | **Drop Dev** | **P** |  |  |
| --- | --- | --- | --- | --- | --- | --- | --- |
| **Omitting Smoking product (3)** |  | 21.898 | (11) | -8.949 | (*) |  |  |
|  |  | **Estimate** | **S.E.** | **P** | **RR** | **95%CIl** | **95%CIu** |
| **Constant** |  | 3.586 | 1.340 | + | 36.104 | 2.611 | 499.172 |
| **RR adjusted for factor other than sex, age** |  |  |  |  |  |  |  |
| Yes | 6 | Aliased |  |  | 2.620 | 1.088 | 6.313 |
| No | 16 | -0.011 | 1.270 | N.S. | 2.591 | 0.414 | 16.212 |
| **Sex(RR)** |  |  |  |  |  |  |  |
| Male | 13 | Aliased |  |  | 22.554 | 3.612 | 140.831 |
| Female | 5 | -0.335 | 0.264 | N.S. | 16.130 | 2.503 | 103.947 |
| Combined | 4 | -3.149 | 1.270 | - | 0.968 | 0.400 | 2.343 |
| **Continent** |  |  |  |  |  |  |  |
| NAmer | 10 | Aliased |  |  | 8.712 | 5.771 | 13.152 |
| Europe | 7 | -1.787 | 0.273 | --- | 1.458 | 1.130 | 1.881 |
| Asia | 5 | -0.223 | 0.344 | N.S. | 6.973 | 3.273 | 14.856 |
| **Asthma analysis type (Emp)** |  |  |  |  |  |  |  |
| inc-irresp | 19 | Aliased |  |  | 2.662 | 2.339 | 3.030 |
| excl-all | 3 | -0.883 | 0.670 | N.S. | 1.101 | 0.272 | 4.458 |
| **Unexposed group** |  |  |  |  |  |  |  |
| nev any | 7 | Aliased |  |  | 3.405 | 2.665 | 4.351 |
| nev cig | 15 | -0.936 | 0.349 | - | 1.335 | 0.773 | 2.305 |
| **Emp subtype** |  |  |  |  |  |  |  |
| mort | 5 | Aliased |  |  | 0.181 | 0.089 | 0.367 |
| other | 17 | 3.227 | 0.395 | +++ | 4.561 | 3.764 | 5.527 |
| **Midpoint age in RR** |  | -0.023 | 0.017 | N.S. | 10.370 | 1.165 | 92.310 |
| **RR adjusted for age** |  |  |  |  |  |  |  |
| Yes | 13 | Aliased |  |  | 3.331 | 2.874 | 3.860 |
| No | 9 | -2.393 | 0.357 | --- | 0.304 | 0.152 | 0.609 |
|  |  | **Deviance** | **(DF)** | **Drop Dev** | **P** |  |  |
| **Omitting Unexposed group** |  | 28.030 | (10) | -15.081 | ** |  |  |
|  |  | **Estimate** | **S.E.** | **P** | **RR** | **95%CIl** | **95%CIu** |
| **Constant** |  | 4.189 | 1.319 | ++ | 65.969 | 4.975 | 874.774 |
| **RR adjusted for factor other than sex, age** |  |  |  |  |  |  |  |
| Yes | 6 | Aliased |  |  | 2.217 | 0.890 | 5.524 |
| No | 16 | 0.507 | 1.256 | N.S. | 3.680 | 0.549 | 24.682 |
| **Sex(RR)** |  |  |  |  |  |  |  |
| Male | 13 | Aliased |  |  | 20.258 | 2.908 | 141.117 |
| Female | 5 | -0.449 | 0.260 | N.S. | 12.924 | 1.798 | 92.926 |
| Combined | 4 | -2.974 | 1.283 | - | 1.035 | 0.406 | 2.641 |
| **Continent** |  |  |  |  |  |  |  |
| NAmer | 10 | Aliased |  |  | 7.704 | 4.840 | 12.264 |
| Europe | 7 | -1.575 | 0.304 | --- | 1.596 | 1.184 | 2.151 |
| Asia | 5 | -0.353 | 0.374 | N.S. | 5.411 | 2.189 | 13.376 |
| **Asthma analysis type (Emp)** |  |  |  |  |  |  |  |
| inc-irresp | 19 | Aliased |  |  | 2.705 | 2.363 | 3.097 |
| excl-all | 3 | -1.612 | 0.619 | - | 0.540 | 0.139 | 2.096 |
| **Smoking product (3)** |  |  |  |  |  |  |  |
| any | 5 | Aliased |  |  | 2.899 | 2.186 | 3.843 |
| cigs | 16 | -0.350 | 0.365 | N.S. | 2.043 | 1.143 | 3.650 |
| cigsonly | 1 | 0.141 | 0.659 | N.S. | 3.338 | 0.791 | 14.078 |
| **Emp subtype** |  |  |  |  |  |  |  |
| mort | 5 | Aliased |  |  | 0.151 | 0.063 | 0.363 |
| other | 17 | 3.445 | 0.470 | +++ | 4.737 | 3.785 | 5.927 |
| **Midpoint age in RR** |  | -0.044 | 0.015 | - | 37.641 | 4.760 | 297.680 |
| **RR adjusted for age** |  |  |  |  |  |  |  |
| Yes | 13 | Aliased |  |  | 3.381 | 2.893 | 3.951 |
| No | 9 | -2.539 | 0.368 | --- | 0.267 | 0.126 | 0.565 |

Table 3 - B - 6

IESCOPD - Meta-regression of current smoking, any product (or cigarettes if all product not available)

Multiple regression of data from Table 3 - B - 1 (most-adjusted RRs)

Any emphysema

Test by removing variables one at a time

|  |  | **Deviance** | **(DF)** | **Drop Dev** | **P** |  |  |
| --- | --- | --- | --- | --- | --- | --- | --- |
| **Omitting Emp subtype** |  | 19.594 | (10) | -6.645 | * |  |  |
|  |  | **Estimate** | **S.E.** | **P** | **RR** | **95%CIl** | **95%CIu** |
| **Constant** |  | 1.950 | 1.337 | N.S. | 7.029 | 0.511 | 96.678 |
| **RR adjusted for factor other than sex, age** |  |  |  |  |  |  |  |
| Yes | 6 | Aliased |  |  | 4.055 | 1.623 | 10.129 |
| No | 16 | -1.366 | 1.260 | N.S. | 1.035 | 0.153 | 6.980 |
| **Sex(RR)** |  |  |  |  |  |  |  |
| Male | 13 | Aliased |  |  | 10.113 | 1.481 | 69.079 |
| Female | 5 | -0.192 | 0.263 | N.S. | 8.346 | 1.180 | 59.007 |
| Combined | 4 | -1.980 | 1.270 | N.S. | 1.397 | 0.552 | 3.531 |
| **Continent** |  |  |  |  |  |  |  |
| NAmer | 10 | Aliased |  |  | 6.329 | 4.016 | 9.974 |
| Europe | 7 | -1.090 | 0.284 | -- | 2.127 | 1.640 | 2.759 |
| Asia | 5 | -1.283 | 0.304 | -- | 1.754 | 0.877 | 3.509 |
| **Asthma analysis type (Emp)** |  |  |  |  |  |  |  |
| inc-irresp | 19 | Aliased |  |  | 2.533 | 2.213 | 2.899 |
| excl-all | 3 | 1.369 | 0.605 | + | 9.956 | 2.642 | 37.513 |
| **Smoking product (3)** |  |  |  |  |  |  |  |
| any | 5 | Aliased |  |  | 0.802 | 0.561 | 1.146 |
| cigs | 16 | 3.914 | 0.487 | +++ | 40.173 | 18.658 | 86.500 |
| cigsonly | 1 | -0.414 | 0.654 | N.S. | 0.530 | 0.127 | 2.212 |
| **Unexposed group** |  |  |  |  |  |  |  |
| nev any | 7 | Aliased |  |  | 8.921 | 6.150 | 12.940 |
| nev cig | 15 | -4.332 | 0.550 | --- | 0.117 | 0.048 | 0.285 |
| **Midpoint age in RR** |  | 0.030 | 0.016 | (+) | 0.436 | 0.051 | 3.752 |
| **RR adjusted for age** |  |  |  |  |  |  |  |
| Yes | 13 | Aliased |  |  | 2.920 | 2.504 | 3.406 |
| No | 9 | -1.101 | 0.350 | - | 0.971 | 0.476 | 1.981 |
|  |  | **Deviance** | **(DF)** | **Drop Dev** | **P** |  |  |
| **Omitting Midpoint age in RR** |  | 12.955 | (10) | -0.006 | N.S. |  |  |
|  |  | **Estimate** | **S.E.** | **P** | **RR** | **95%CIl** | **95%CIu** |
| **Constant** |  | 2.778 | 1.231 | + | 16.083 | 1.441 | 179.455 |
| **RR adjusted for factor other than sex, age** |  |  |  |  |  |  |  |
| Yes | 6 | Aliased |  |  | 3.177 | 1.319 | 7.655 |
| No | 16 | -0.609 | 1.209 | N.S. | 1.728 | 0.277 | 10.793 |
| **Sex(RR)** |  |  |  |  |  |  |  |
| Male | 13 | Aliased |  |  | 14.707 | 2.110 | 102.493 |
| Female | 5 | -0.264 | 0.265 | N.S. | 11.293 | 1.582 | 80.599 |
| Combined | 4 | -2.525 | 1.283 | (-) | 1.178 | 0.462 | 3.002 |
| **Continent** |  |  |  |  |  |  |  |
| NAmer | 10 | Aliased |  |  | 7.132 | 4.492 | 11.323 |
| Europe | 7 | -1.394 | 0.307 | -- | 1.769 | 1.307 | 2.393 |
| Asia | 5 | -0.669 | 0.349 | (-) | 3.655 | 1.504 | 8.879 |
| **Asthma analysis type (Emp)** |  |  |  |  |  |  |  |
| inc-irresp | 19 | Aliased |  |  | 2.605 | 2.278 | 2.978 |
| excl-all | 3 | 0.105 | 0.497 | N.S. | 2.892 | 0.970 | 8.619 |
| **Smoking product (3)** |  |  |  |  |  |  |  |
| any | 5 | Aliased |  |  | 1.298 | 0.787 | 2.143 |
| cigs | 16 | 2.312 | 0.714 | ++ | 13.102 | 4.289 | 40.028 |
| cigsonly | 1 | -0.125 | 0.642 | N.S. | 1.146 | 0.267 | 4.926 |
| **Unexposed group** |  |  |  |  |  |  |  |
| nev any | 7 | Aliased |  |  | 5.992 | 3.996 | 8.985 |
| nev cig | 15 | -2.929 | 0.606 | --- | 0.320 | 0.121 | 0.850 |
| **Emp subtype** |  |  |  |  |  |  |  |
| mort | 5 | Aliased |  |  | 0.633 | 0.232 | 1.727 |
| other | 17 | 1.713 | 0.539 | ++ | 3.511 | 2.745 | 4.491 |
| **RR adjusted for age** |  |  |  |  |  |  |  |
| Yes | 13 | Aliased |  |  | 3.111 | 2.643 | 3.662 |
| No | 9 | -1.721 | 0.424 | -- | 0.556 | 0.235 | 1.314 |

Table 3 - B - 6

IESCOPD - Meta-regression of current smoking, any product (or cigarettes if all product not available)

Multiple regression of data from Table 3 - B - 1 (most-adjusted RRs)

Any emphysema

Test by removing variables one at a time

|  |  | **Deviance** | **(DF)** | **Drop Dev** | **P** |  |  |
| --- | --- | --- | --- | --- | --- | --- | --- |
| **Omitting RR adjusted for age** |  | 29.395 | (10) | -16.446 | ** |  |  |
|  |  | **Estimate** | **S.E.** | **P** | **RR** | **95%CIl** | **95%CIu** |
| **Constant** |  | 2.214 | 1.365 | N.S. | 9.149 | 0.630 | 132.906 |
| **RR adjusted for factor other than sex, age** |  |  |  |  |  |  |  |
| Yes | 6 | Aliased |  |  | 3.021 | 1.184 | 7.707 |
| No | 16 | -0.452 | 1.290 | N.S. | 1.922 | 0.272 | 13.559 |
| **Sex(RR)** |  |  |  |  |  |  |  |
| Male | 13 | Aliased |  |  | 5.273 | 0.817 | 34.038 |
| Female | 5 | -0.234 | 0.265 | N.S. | 4.174 | 0.627 | 27.785 |
| Combined | 4 | -1.010 | 1.232 | N.S. | 1.921 | 0.781 | 4.724 |
| **Continent** |  |  |  |  |  |  |  |
| NAmer | 10 | Aliased |  |  | 5.489 | 3.522 | 8.555 |
| Europe | 7 | -1.016 | 0.293 | -- | 1.987 | 1.476 | 2.677 |
| Asia | 5 | -0.571 | 0.382 | N.S. | 3.102 | 1.224 | 7.864 |
| **Asthma analysis type (Emp)** |  |  |  |  |  |  |  |
| inc-irresp | 19 | Aliased |  |  | 2.581 | 2.251 | 2.960 |
| excl-all | 3 | 0.521 | 0.762 | N.S. | 4.346 | 0.820 | 23.038 |
| **Smoking product (3)** |  |  |  |  |  |  |  |
| any | 5 | Aliased |  |  | 0.770 | 0.484 | 1.225 |
| cigs | 16 | 4.034 | 0.661 | +++ | 43.475 | 15.407 | 122.671 |
| cigsonly | 1 | 0.044 | 0.661 | N.S. | 0.804 | 0.169 | 3.822 |
| **Unexposed group** |  |  |  |  |  |  |  |
| nev any | 7 | Aliased |  |  | 9.367 | 6.036 | 14.538 |
| nev cig | 15 | -4.505 | 0.663 | --- | 0.104 | 0.036 | 0.301 |
| **Emp subtype** |  |  |  |  |  |  |  |
| mort | 5 | Aliased |  |  | 2.245 | 0.824 | 6.113 |
| other | 17 | 0.182 | 0.538 | N.S. | 2.694 | 2.107 | 3.445 |
| **Midpoint age in RR** |  | 0.004 | 0.019 | N.S. | 2.079 | 0.154 | 28.123 |
|  |  | **Deviance** | **(DF)** | **Drop Dev** | **P** |  |  |
| **Omitting RR adjusted for factor other than sex, age** |  | 13.199 | (10) | -0.250 | N.S. |  |  |
|  |  | **Estimate** | **S.E.** | **P** | **RR** | **95%CIl** | **95%CIu** |
| **Constant** |  | 2.270 | 1.016 | + | 9.678 | 1.321 | 70.930 |
| **RR adjusted for age** |  |  |  |  |  |  |  |
| Yes | 13 | Aliased |  |  | 3.108 | 2.640 | 3.659 |
| No | 9 | -1.713 | 0.424 | -- | 0.561 | 0.237 | 1.324 |
| **Sex(RR)** |  |  |  |  |  |  |  |
| Male | 13 | Aliased |  |  | 9.988 | 4.273 | 23.344 |
| Female | 5 | -0.267 | 0.265 | N.S. | 7.648 | 3.186 | 18.359 |
| Combined | 4 | -1.951 | 0.547 | -- | 1.419 | 0.943 | 2.135 |
| **Continent** |  |  |  |  |  |  |  |
| NAmer | 10 | Aliased |  |  | 7.125 | 4.468 | 11.362 |
| Europe | 7 | -1.396 | 0.307 | -- | 1.764 | 1.302 | 2.391 |
| Asia | 5 | -0.653 | 0.379 | N.S. | 3.710 | 1.468 | 9.377 |
| **Asthma analysis type (Emp)** |  |  |  |  |  |  |  |
| inc-irresp | 19 | Aliased |  |  | 2.609 | 2.276 | 2.990 |
| excl-all | 3 | 0.038 | 0.734 | N.S. | 2.710 | 0.544 | 13.506 |
| **Smoking product (3)** |  |  |  |  |  |  |  |
| any | 5 | Aliased |  |  | 1.313 | 0.767 | 2.248 |
| cigs | 16 | 2.274 | 0.772 | + | 12.765 | 3.813 | 42.735 |
| cigsonly | 1 | -0.109 | 0.660 | N.S. | 1.177 | 0.249 | 5.576 |
| **Unexposed group** |  |  |  |  |  |  |  |
| nev any | 7 | Aliased |  |  | 5.906 | 3.624 | 9.625 |
| nev cig | 15 | -2.878 | 0.743 | -- | 0.332 | 0.101 | 1.097 |
| **Emp subtype** |  |  |  |  |  |  |  |
| mort | 5 | Aliased |  |  | 0.611 | 0.187 | 1.998 |
| other | 17 | 1.755 | 0.638 | + | 3.536 | 2.675 | 4.674 |
| **Midpoint age in RR** |  | -0.002 | 0.018 | N.S. | 2.920 | 0.254 | 33.550 |

Table 3 - B - 6

IESCOPD - Meta-regression of current smoking, any product (or cigarettes if all product not available)

Multiple regression of data from Table 3 - B - 1 (most-adjusted RRs)

Any emphysema

Test reduction to 2-level product

|  |  | **Deviance** | **(DF)** | **Drop Dev** | **P** |  |  |
| --- | --- | --- | --- | --- | --- | --- | --- |
| **Reducing Emp subtype to 2 levels** |  | 18.686 | (10) | -5.738 | (*) |  |  |
|  |  | **Estimate** | **S.E.** | **P** | **RR** | **95%CIl** | **95%CIu** |
| **Constant** |  | 3.352 | 1.346 | + | 28.547 | 2.039 | 399.667 |
| **Sex(RR)** |  |  |  |  |  |  |  |
| Male | 13 | Aliased |  |  | 18.121 | 2.603 | 126.132 |
| Female | 5 | -0.307 | 0.264 | N.S. | 13.329 | 1.862 | 95.434 |
| Combined | 4 | -2.828 | 1.282 | (-) | 1.071 | 0.420 | 2.731 |
| **Continent** |  |  |  |  |  |  |  |
| NAmer | 10 | Aliased |  |  | 7.618 | 4.794 | 12.105 |
| Europe | 7 | -1.571 | 0.298 | --- | 1.583 | 1.189 | 2.107 |
| Asia | 5 | -0.284 | 0.346 | N.S. | 5.732 | 2.500 | 13.145 |
| **Asthma analysis type (Emp)** |  |  |  |  |  |  |  |
| inc-irresp | 19 | Aliased |  |  | 2.653 | 2.316 | 3.039 |
| excl-all | 3 | -0.723 | 0.676 | N.S. | 1.287 | 0.293 | 5.653 |
| **Smoking product (2)** |  |  |  |  |  |  |  |
| any | 5 | Aliased |  |  | 1.971 | 1.357 | 2.864 |
| cigs | 17 | 0.899 | 0.502 | N.S. | 4.846 | 2.219 | 10.580 |
| **Unexposed group** |  |  |  |  |  |  |  |
| nev any | 7 | Aliased |  |  | 4.126 | 2.905 | 5.861 |
| nev cig | 15 | -1.614 | 0.514 | - | 0.822 | 0.358 | 1.889 |
| **Emp subtype** |  |  |  |  |  |  |  |
| mort | 5 | Aliased |  |  | 0.263 | 0.110 | 0.632 |
| other | 17 | 2.774 | 0.469 | +++ | 4.217 | 3.372 | 5.275 |
| **Midpoint age in RR** |  | -0.020 | 0.017 | N.S. | 8.846 | 0.886 | 88.360 |
| **RR adjusted for age** |  |  |  |  |  |  |  |
| Yes | 13 | Aliased |  |  | 3.229 | 2.753 | 3.787 |
| No | 9 | -2.088 | 0.396 | --- | 0.400 | 0.179 | 0.893 |
| **RR adjusted for factor other than sex, age** |  |  |  |  |  |  |  |
| Yes | 6 | Aliased |  |  | 2.696 | 1.071 | 6.785 |
| No | 16 | -0.100 | 1.270 | N.S. | 2.440 | 0.356 | 16.723 |

Table 3 - B - 6

IESCOPD - Meta-regression of current smoking, any product (or cigarettes if all product not available)

Multiple regression of data from Table 3 - B - 1 (most-adjusted RRs)

Any emphysema

Test by adding extra variables one at a time

|  |  | **Deviance** | **(DF)** | **Drop Dev** | **P** |  |  |
| --- | --- | --- | --- | --- | --- | --- | --- |
| **Increasing Emp subtype to 3 levels** |  | 12.839 | (8) | 0.110 | N.S. |  |  |
|  |  | **Estimate** | **S.E.** | **P** | **RR** | **95%CIl** | **95%CIu** |
| **Constant** |  | 2.695 | 1.375 | (+) | 14.805 | 0.999 | 219.326 |
| **Sex(RR)** |  |  |  |  |  |  |  |
| Male | 13 | Aliased |  |  | 16.327 | 1.651 | 161.465 |
| Female | 5 | -0.262 | 0.265 | N.S. | 12.569 | 1.232 | 128.225 |
| Combined | 4 | -2.680 | 1.361 | (-) | 1.120 | 0.371 | 3.380 |
| **Continent** |  |  |  |  |  |  |  |
| NAmer | 10 | Aliased |  |  | 7.103 | 4.214 | 11.971 |
| Europe | 7 | -1.406 | 0.309 | -- | 1.741 | 1.212 | 2.501 |
| Asia | 5 | -0.579 | 0.491 | N.S. | 3.979 | 1.127 | 14.043 |
| **Asthma analysis type (Emp)** |  |  |  |  |  |  |  |
| inc-irresp | 19 | Aliased |  |  | 2.608 | 2.237 | 3.040 |
| excl-all | 3 | 0.053 | 0.822 | N.S. | 2.750 | 0.371 | 20.364 |
| **Smoking product (3)** |  |  |  |  |  |  |  |
| any | 5 | Aliased |  |  | 1.330 | 0.694 | 2.550 |
| cigs | 16 | 2.232 | 0.843 | + | 12.401 | 2.859 | 53.798 |
| cigsonly | 1 | -0.151 | 0.664 | N.S. | 1.144 | 0.198 | 6.603 |
| **Unexposed group** |  |  |  |  |  |  |  |
| nev any | 7 | Aliased |  |  | 6.093 | 3.482 | 10.661 |
| nev cig | 15 | -2.988 | 0.767 | -- | 0.307 | 0.078 | 1.210 |
|  |  | **Estimate** |  |  |  |  |  |
| **Emp subtype** |  |  |  |  |  |  |  |
| mort | 5 | Aliased |  |  |  |  |  |
| other | 17 | Aliased |  |  |  |  |  |
| **Midpoint age in RR** |  | 0.003 | 0.020 | N.S. | 2.167 | 0.109 | 42.887 |
| **RR adjusted for age** |  |  |  |  |  |  |  |
| Yes | 13 | Aliased |  |  | 3.120 | 2.598 | 3.746 |
| No | 9 | -1.750 | 0.433 | -- | 0.542 | 0.204 | 1.441 |
| **RR adjusted for factor other than sex, age** |  |  |  |  |  |  |  |
| Yes | 6 | Aliased |  |  | 3.267 | 1.143 | 9.341 |
| No | 16 | -0.696 | 1.300 | N.S. | 1.630 | 0.182 | 14.585 |
| **Emp subtype (3)** |  |  |  |  |  |  |  |
| mort | 5 | Aliased |  |  | 0.592 | 0.131 | 2.682 |
| oth-prev | 14 | 1.785 | 0.721 | + | 3.529 | 2.548 | 4.888 |
| oth-inc | 3 | 1.967 | 1.074 | N.S. | 4.234 | 0.970 | 18.492 |

Table 3 - B - 6

IESCOPD - Meta-regression of current smoking, any product (or cigarettes if all product not available)

Multiple regression of data from Table 3 - B - 1 (most-adjusted RRs)

Any emphysema

Fixed model, test by adding extra variables one at a time

|  |  | **Deviance** | **(DF)** | **Drop Dev** | **P** |  |  |
| --- | --- | --- | --- | --- | --- | --- | --- |
| **Adding National cigarette tobacco type** |  | 9.809 | (8) | 3.140 | N.S. |  |  |
|  |  | **Estimate** | **S.E.** | **P** | **RR** | **95%CIl** | **95%CIu** |
| **Constant** |  | 2.891 | 1.374 | (+) | 18.005 | 1.218 | 266.183 |
| **Sex(RR)** |  |  |  |  |  |  |  |
| Male | 13 | Aliased |  |  | 14.640 | 2.196 | 97.586 |
| Female | 5 | -0.363 | 0.271 | N.S. | 10.183 | 1.484 | 69.850 |
| Combined | 4 | -2.505 | 1.288 | (-) | 1.196 | 0.479 | 2.985 |
| **Continent** |  |  |  |  |  |  |  |
| NAmer | 10 | Aliased |  |  | 6.635 | 4.174 | 10.546 |
| Europe | 7 | -1.305 | 0.311 | -- | 1.799 | 1.337 | 2.421 |
| Asia | 5 | -0.566 | 0.389 | N.S. | 3.766 | 1.516 | 9.359 |
| **Asthma analysis type (Emp)** |  |  |  |  |  |  |  |
| inc-irresp | 19 | Aliased |  |  | 2.617 | 2.290 | 2.990 |
| excl-all | 3 | -0.102 | 0.781 | N.S. | 2.363 | 0.448 | 12.457 |
| **Smoking product (3)** |  |  |  |  |  |  |  |
| any | 5 | Aliased |  |  | 1.288 | 0.758 | 2.187 |
| cigs | 16 | 2.335 | 0.782 | + | 13.302 | 4.043 | 43.760 |
| cigsonly | 1 | Aliased |  |  | 1.288 | 0.758 | 2.187 |
| **Unexposed group** |  |  |  |  |  |  |  |
| nev any | 7 | Aliased |  |  | 5.857 | 3.592 | 9.551 |
| nev cig | 15 | -2.849 | 0.767 | -- | 0.339 | 0.102 | 1.125 |
| **Emp subtype** |  |  |  |  |  |  |  |
| mort | 5 | Aliased |  |  | 0.647 | 0.199 | 2.106 |
| other | 17 | 1.686 | 0.653 | + | 3.494 | 2.650 | 4.608 |
| **Midpoint age in RR** |  | -0.003 | 0.020 | N.S. | 3.212 | 0.248 | 41.636 |
| **RR adjusted for age** |  |  |  |  |  |  |  |
| Yes | 13 | Aliased |  |  | 3.097 | 2.643 | 3.631 |
| No | 9 | -1.679 | 0.425 | -- | 0.578 | 0.250 | 1.336 |
| **RR adjusted for factor other than sex, age** |  |  |  |  |  |  |  |
| Yes | 6 | Aliased |  |  | 3.089 | 1.239 | 7.700 |
| No | 16 | -0.522 | 1.293 | N.S. | 1.833 | 0.273 | 12.320 |
| **National cigarette tobacco type** |  |  |  |  |  |  |  |
| bl | 20 | Aliased |  |  | 2.651 | 2.328 | 3.019 |
| vir | 1 | -0.108 | 0.663 | N.S. | 2.380 | 0.569 | 9.958 |
| m/u | 1 | -0.944 | 0.533 | N.S. | 1.031 | 0.328 | 3.246 |
|  |  | **Deviance** | **(DF)** | **Drop Dev** | **P** |  |  |
| **Adding Publication year** |  | 11.998 | (6) | 0.951 | N.S. |  |  |
|  |  | **Estimate** | **S.E.** | **P** | **RR** | **95%CIl** | **95%CIu** |
| **Constant** |  | 2.191 | 3.298 | N.S. | 8.942 | 0.014 | 5732.397 |
| **Sex(RR)** |  |  |  |  |  |  |  |
| Male | 13 | Aliased |  |  | 14.484 | 0.857 | 244.920 |
| Female | 5 | -0.297 | 0.269 | N.S. | 10.764 | 0.626 | 185.185 |
| Combined | 4 | -2.498 | 1.505 | N.S. | 1.192 | 0.306 | 4.646 |

Table 3 - B - 6

IESCOPD - Meta-regression of current smoking, any product (or cigarettes if all product not available)

Multiple regression of data from Table 3 - B - 1 (most-adjusted RRs)

Any emphysema

Test by adding extra variables one at a time

|  |  | **Estimate** | **S.E.** | **P** | **RR** | **95%CIl** | **95%CIu** |
| --- | --- | --- | --- | --- | --- | --- | --- |
| **Continent** |  |  |  |  |  |  |  |
| NAmer | 10 | Aliased |  |  | 7.696 | 1.903 | 31.131 |
| Europe | 7 | -1.502 | 0.754 | (-) | 1.714 | 0.812 | 3.620 |
| Asia | 5 | -0.711 | 0.441 | N.S. | 3.780 | 0.673 | 21.228 |
| **Asthma analysis type (Emp)** |  |  |  |  |  |  |  |
| inc-irresp | 19 | Aliased |  |  | 2.599 | 2.180 | 3.098 |
| excl-all | 3 | 0.205 | 1.043 | N.S. | 3.191 | 0.188 | 54.095 |
| **Smoking product (3)** |  |  |  |  |  |  |  |
| any | 5 | Aliased |  |  | 1.412 | 0.658 | 3.033 |
| cigs | 16 | 2.036 | 0.891 | (+) | 10.817 | 1.916 | 61.078 |
| cigsonly | 1 | -0.187 | 0.729 | N.S. | 1.172 | 0.132 | 10.409 |
| **Unexposed group** |  |  |  |  |  |  |  |
| nev any | 7 | Aliased |  |  | 5.408 | 2.159 | 13.546 |
| nev cig | 15 | -2.568 | 1.149 | (-) | 0.415 | 0.042 | 4.087 |
| **Emp subtype** |  |  |  |  |  |  |  |
| mort | 5 | Aliased |  |  | 0.508 | 0.082 | 3.151 |
| other | 17 | 1.980 | 0.793 | + | 3.676 | 2.431 | 5.560 |
| **Midpoint age in RR** |  | 0.009 | 0.055 | N.S. | 1.541 | 0.000 | 13845.616 |
| **RR adjusted for age** |  |  |  |  |  |  |  |
| Yes | 13 | Aliased |  |  | 3.160 | 2.291 | 4.357 |
| No | 9 | -1.874 | 0.980 | N.S. | 0.485 | 0.042 | 5.592 |
| **RR adjusted for factor other than sex, age** |  |  |  |  |  |  |  |
| Yes | 6 | Aliased |  |  | 3.080 | 0.917 | 10.338 |
| No | 16 | -0.512 | 1.343 | N.S. | 1.845 | 0.147 | 23.104 |
| **Publication year** |  |  |  |  |  |  |  |
| <1980 | 7 | Aliased |  |  | 3.018 | 0.943 | 9.660 |
| 1980-89 | 6 | -0.100 | 0.453 | N.S. | 2.732 | 1.721 | 4.335 |
| 1990-99 | 3 | -0.421 | 1.478 | N.S. | 1.981 | 0.057 | 68.292 |
| 2000+ | 6 | -0.443 | 0.503 | N.S. | 1.938 | 0.684 | 5.489 |
|  |  | **Deviance** | **(DF)** | **Drop Dev** | **P** |  |  |
| **Adding Study type** |  | 12.839 | (8) | 0.110 | N.S. |  |  |
|  |  | **Estimate** | **S.E.** | **P** | **RR** | **95%CIl** | **95%CIu** |
| **Constant** |  | 2.695 | 1.375 | (+) | 14.805 | 0.999 | 219.326 |
| **Sex(RR)** |  |  |  |  |  |  |  |
| Male | 13 | Aliased |  |  | 16.327 | 1.651 | 161.465 |
| Female | 5 | -0.262 | 0.265 | N.S. | 12.569 | 1.232 | 128.225 |
| Combined | 4 | -2.680 | 1.361 | (-) | 1.120 | 0.371 | 3.380 |
| **Continent** |  |  |  |  |  |  |  |
| NAmer | 10 | Aliased |  |  | 7.103 | 4.214 | 11.971 |
| Europe | 7 | -1.406 | 0.309 | -- | 1.741 | 1.212 | 2.501 |
| Asia | 5 | -0.579 | 0.491 | N.S. | 3.979 | 1.127 | 14.043 |
| **Asthma analysis type (Emp)** |  |  |  |  |  |  |  |
| inc-irresp | 19 | Aliased |  |  | 2.608 | 2.237 | 3.040 |
| excl-all | 3 | 0.053 | 0.822 | N.S. | 2.750 | 0.371 | 20.364 |
| **Smoking product (3)** |  |  |  |  |  |  |  |
| any | 5 | Aliased |  |  | 1.330 | 0.694 | 2.550 |
| cigs | 16 | 2.232 | 0.843 | + | 12.401 | 2.859 | 53.798 |
| cigsonly | 1 | -0.151 | 0.664 | N.S. | 1.144 | 0.198 | 6.603 |
| **Unexposed group** |  |  |  |  |  |  |  |
| nev any | 7 | Aliased |  |  | 6.093 | 3.482 | 10.661 |
| nev cig | 15 | -2.988 | 0.767 | -- | 0.307 | 0.078 | 1.210 |
| **Emp subtype** |  |  |  |  |  |  |  |
| mort | 5 | Aliased |  |  | 0.513 | 0.056 | 4.682 |
| other | 17 | 1.967 | 1.074 | N.S. | 3.669 | 2.261 | 5.952 |
| **Midpoint age in RR** |  | 0.003 | 0.020 | N.S. | 2.167 | 0.109 | 42.887 |
| **RR adjusted for age** |  |  |  |  |  |  |  |
| Yes | 13 | Aliased |  |  | 3.120 | 2.598 | 3.746 |
| No | 9 | -1.750 | 0.433 | -- | 0.542 | 0.204 | 1.441 |

Table 3 - B - 6

IESCOPD - Meta-regression of current smoking, any product (or cigarettes if all product not available)

Multiple regression of data from Table 3 - B - 1 (most-adjusted RRs)

Any emphysema

Test by adding extra variables one at a time

|  |  | **Estimate** | **S.E.** | **P** | **RR** | **95%CIl** | **95%CIu** |
| --- | --- | --- | --- | --- | --- | --- | --- |
| **RR adjusted for factor other than sex, age** |  |  |  |  |  |  |  |
| Yes | 6 | Aliased |  |  | 3.267 | 1.143 | 9.341 |
| No | 16 | -0.696 | 1.300 | N.S. | 1.630 | 0.182 | 14.585 |
| **Study type** |  |  |  |  |  |  |  |
| Pr | 8 | Aliased |  |  | 3.014 | 1.020 | 8.905 |
| CS | 14 | -0.182 | 0.549 | N.S. | 2.512 | 1.816 | 3.474 |
|  |  | **Deviance** | **(DF)** | **Drop Dev** | **P** |  |  |
| **Adding Study weakness** |  | 12.535 | (8) | 0.414 | N.S. |  |  |
|  |  | **Estimate** | **S.E.** | **P** | **RR** | **95%CIl** | **95%CIu** |
| **Constant** |  | 1.746 | 2.054 | N.S. | 5.732 | 0.102 | 320.838 |
| **Sex(RR)** |  |  |  |  |  |  |  |
| Male | 13 | Aliased |  |  | 15.116 | 1.768 | 129.233 |
| Female | 5 | -0.263 | 0.265 | N.S. | 11.626 | 1.323 | 102.144 |
| Combined | 4 | -2.566 | 1.289 | (-) | 1.162 | 0.413 | 3.269 |
| **Continent** |  |  |  |  |  |  |  |
| NAmer | 10 | Aliased |  |  | 7.252 | 4.327 | 12.156 |
| Europe | 7 | -1.426 | 0.311 | -- | 1.742 | 1.239 | 2.449 |
| Asia | 5 | -0.636 | 0.390 | N.S. | 3.841 | 1.340 | 11.003 |
| **Asthma analysis type (Emp)** |  |  |  |  |  |  |  |
| inc-irresp | 19 | Aliased |  |  | 2.550 | 2.154 | 3.020 |
| excl-all | 3 | 1.061 | 1.610 | N.S. | 7.372 | 0.155 | 351.611 |
| **Smoking product (3)** |  |  |  |  |  |  |  |
| any | 5 | Aliased |  |  | 1.332 | 0.723 | 2.457 |
| cigs | 16 | 2.226 | 0.801 | + | 12.345 | 3.111 | 48.989 |
| cigsonly | 1 | -0.123 | 0.663 | N.S. | 1.179 | 0.208 | 6.689 |
| **Unexposed group** |  |  |  |  |  |  |  |
| nev any | 7 | Aliased |  |  | 5.897 | 3.368 | 10.323 |
| nev cig | 15 | -2.873 | 0.777 | -- | 0.333 | 0.084 | 1.317 |
| **Emp subtype** |  |  |  |  |  |  |  |
| mort | 5 | Aliased |  |  | 0.591 | 0.149 | 2.348 |
| other | 17 | 1.796 | 0.676 | + | 3.562 | 2.583 | 4.912 |
| **Midpoint age in RR** |  | -0.001 | 0.020 | N.S. | 2.725 | 0.148 | 50.136 |
| **RR adjusted for age** |  |  |  |  |  |  |  |
| Yes | 13 | Aliased |  |  | 3.125 | 2.609 | 3.742 |
| No | 9 | -1.765 | 0.430 | -- | 0.535 | 0.205 | 1.394 |
| **RR adjusted for factor other than sex, age** |  |  |  |  |  |  |  |
| Yes | 6 | Aliased |  |  | 3.164 | 1.126 | 8.888 |
| No | 16 | -0.596 | 1.293 | N.S. | 1.743 | 0.202 | 15.029 |
| **Study weakness** |  |  |  |  |  |  |  |
| Yes | 2 | Aliased |  |  | 0.923 | 0.018 | 48.687 |
| No | 20 | 1.062 | 1.649 | N.S. | 2.668 | 2.258 | 3.154 |
|  |  | **Deviance** | **(DF)** | **Drop Dev** | **P** |  |  |
| **Adding Number of cases (Emp)** |  | 10.920 | (6) | 2.029 | N.S. |  |  |
|  |  | **Estimate** | **S.E.** | **P** | **RR** | **95%CIl** | **95%CIu** |
| **Constant** |  | 3.217 | 1.687 | N.S. | 24.948 | 0.915 | 680.219 |
| **Sex(RR)** |  |  |  |  |  |  |  |
| Male | 13 | Aliased |  |  | 34.718 | 1.127 | 1069.446 |
| Female | 5 | -0.325 | 0.270 | N.S. | 25.073 | 0.848 | 740.925 |
| Combined | 4 | -3.789 | 1.912 | (-) | 0.785 | 0.152 | 4.057 |
| **Continent** |  |  |  |  |  |  |  |
| NAmer | 10 | Aliased |  |  | 10.535 | 2.779 | 39.934 |
| Europe | 7 | -2.137 | 0.910 | (-) | 1.243 | 0.408 | 3.789 |
| Asia | 5 | 0.082 | 0.896 | N.S. | 11.433 | 0.337 | 387.457 |
| **Asthma analysis type (Emp)** |  |  |  |  |  |  |  |
| inc-irresp | 19 | Aliased |  |  | 2.546 | 2.151 | 3.014 |
| excl-all | 3 | 1.133 | 1.094 | N.S. | 7.903 | 0.465 | 134.390 |

Table 3 - B - 6

IESCOPD - Meta-regression of current smoking, any product (or cigarettes if all product not available)

Multiple regression of data from Table 3 - B - 1 (most-adjusted RRs)

Any emphysema

Test by adding extra variables one at a time

|  |  | **Estimate** | **S.E.** | **P** | **RR** | **95%CIl** | **95%CIu** |
| --- | --- | --- | --- | --- | --- | --- | --- |
| **Smoking product (3)** |  |  |  |  |  |  |  |
| any | 5 | Aliased |  |  | 2.167 | 0.263 | 17.836 |
| cigs | 16 | 0.615 | 2.647 | N.S. | 4.008 | 0.030 | 537.383 |
| cigsonly | 1 | 0.012 | 1.032 | N.S. | 2.194 | 0.029 | 167.353 |
| **Unexposed group** |  |  |  |  |  |  |  |
| nev any | 7 | Aliased |  |  | 4.243 | 0.732 | 24.607 |
| nev cig | 15 | -1.712 | 2.335 | N.S. | 0.766 | 0.009 | 63.971 |
| **Emp subtype** |  |  |  |  |  |  |  |
| mort | 5 | Aliased |  |  | 0.156 | 0.001 | 47.400 |
| other | 17 | 3.410 | 2.614 | N.S. | 4.708 | 1.410 | 15.715 |
| **Midpoint age in RR** |  | -0.013 | 0.036 | N.S. | 5.856 | 0.019 | 1808.811 |
| **RR adjusted for age** |  |  |  |  |  |  |  |
| Yes | 13 | Aliased |  |  | 3.399 | 2.194 | 5.265 |
| No | 9 | -2.591 | 1.519 | N.S. | 0.255 | 0.007 | 9.431 |
| **RR adjusted for factor other than sex, age** |  |  |  |  |  |  |  |
| Yes | 6 | Aliased |  |  | 3.509 | 1.073 | 11.476 |
| No | 16 | -0.918 | 1.378 | N.S. | 1.402 | 0.118 | 16.637 |
| **Number of cases (Emp)** |  |  |  |  |  |  |  |
| 1-50 | 8 | Aliased |  |  | 1.491 | 0.452 | 4.917 |
| 51-100 | 6 | 0.578 | 0.513 | N.S. | 2.658 | 0.665 | 10.630 |
| 101-200 | 1 | -2.428 | 3.295 | N.S. | 0.132 | 0.000 | 1917.807 |
| 201+ | 7 | 0.634 | 0.533 | N.S. | 2.812 | 2.074 | 3.812 |
|  |  | **Deviance** | **(DF)** | **Drop Dev** | **P** |  |  |
| **Adding Smoking results reported in study (Emp)** |  | 12.913 | (8) | 0.035 | N.S. |  |  |
|  |  | **Estimate** | **S.E.** | **P** | **RR** | **95%CIl** | **95%CIu** |
| **Constant** |  | 2.647 | 1.441 | N.S. | 14.105 | 0.837 | 237.698 |
| **Sex(RR)** |  |  |  |  |  |  |  |
| Male | 13 | Aliased |  |  | 14.083 | 1.450 | 136.743 |
| Female | 5 | -0.262 | 0.265 | N.S. | 10.834 | 1.093 | 107.419 |
| Combined | 4 | -2.461 | 1.346 | N.S. | 1.202 | 0.402 | 3.592 |
| **Continent** |  |  |  |  |  |  |  |
| NAmer | 10 | Aliased |  |  | 6.667 | 2.313 | 19.217 |
| Europe | 7 | -1.309 | 0.551 | - | 1.800 | 1.202 | 2.697 |
| Asia | 5 | -0.582 | 0.651 | N.S. | 3.725 | 1.224 | 11.332 |
| **Asthma analysis type (Emp)** |  |  |  |  |  |  |  |
| inc-irresp | 19 | Aliased |  |  | 2.608 | 2.231 | 3.049 |
| excl-all | 3 | 0.042 | 0.962 | N.S. | 2.720 | 0.260 | 28.407 |
| **Smoking product (3)** |  |  |  |  |  |  |  |
| any | 5 | Aliased |  |  | 1.184 | 0.330 | 4.248 |
| cigs | 16 | 2.618 | 1.683 | N.S. | 16.219 | 0.870 | 302.375 |
| cigsonly | 1 | -0.131 | 0.664 | N.S. | 1.039 | 0.136 | 7.946 |
| **Unexposed group** |  |  |  |  |  |  |  |
| nev any | 7 | Aliased |  |  | 6.388 | 2.593 | 15.737 |
| nev cig | 15 | -3.155 | 1.260 | - | 0.272 | 0.029 | 2.589 |
| **Emp subtype** |  |  |  |  |  |  |  |
| mort | 5 | Aliased |  |  | 0.712 | 0.114 | 4.459 |
| other | 17 | 1.571 | 0.888 | N.S. | 3.426 | 2.274 | 5.160 |
| **Midpoint age in RR** |  | -0.001 | 0.023 | N.S. | 2.758 | 0.083 | 91.223 |
| **RR adjusted for age** |  |  |  |  |  |  |  |
| Yes | 13 | Aliased |  |  | 3.089 | 2.519 | 3.788 |
| No | 9 | -1.652 | 0.557 | - | 0.592 | 0.169 | 2.077 |
| **RR adjusted for factor other than sex, age** |  |  |  |  |  |  |  |
| Yes | 6 | Aliased |  |  | 3.157 | 1.080 | 9.230 |
| No | 16 | -0.590 | 1.324 | N.S. | 1.751 | 0.187 | 16.418 |
| **Smoking results reported in study (Emp)** |  |  |  |  |  |  |  |
| Current Only | 5 | Aliased |  |  | 2.239 | 0.293 | 17.120 |
| Both | 17 | 0.175 | 0.931 | N.S. | 2.668 | 1.930 | 3.690 |

Table 3 - B - 6

IESCOPD - Meta-regression of current smoking, any product (or cigarettes if all product not available)

Multiple regression of data from Table 3 - B - 1 (most-adjusted RRs)

Any emphysema

Test by adding extra variables one at a time

|  |  | **Deviance** | **(DF)** | **Drop Dev** | **P** |  |  |
| --- | --- | --- | --- | --- | --- | --- | --- |
| **Adding Derivation of RR/CI** |  | 11.160 | (8) | 1.789 | N.S. |  |  |
|  |  | **Estimate** | **S.E.** | **P** | **RR** | **95%CIl** | **95%CIu** |
| **Constant** |  | 1.417 | 1.686 | N.S. | 4.125 | 0.151 | 112.420 |
| **Sex(RR)** |  |  |  |  |  |  |  |
| Male | 13 | Aliased |  |  | 10.009 | 1.185 | 84.523 |
| Female | 5 | -0.327 | 0.269 | N.S. | 7.219 | 0.804 | 64.791 |
| Combined | 4 | -1.946 | 1.361 | N.S. | 1.429 | 0.509 | 4.015 |
| **Continent** |  |  |  |  |  |  |  |
| NAmer | 10 | Aliased |  |  | 8.846 | 4.811 | 16.265 |
| Europe | 7 | -1.716 | 0.390 | -- | 1.590 | 1.102 | 2.295 |
| Asia | 5 | -0.698 | 0.384 | N.S. | 4.402 | 1.576 | 12.296 |
| **Asthma analysis type (Emp)** |  |  |  |  |  |  |  |
| inc-irresp | 19 | Aliased |  |  | 2.609 | 2.263 | 3.007 |
| excl-all | 3 | 0.037 | 0.772 | N.S. | 2.707 | 0.468 | 15.642 |
| **Smoking product (3)** |  |  |  |  |  |  |  |
| any | 5 | Aliased |  |  | 1.342 | 0.760 | 2.372 |
| cigs | 16 | 2.204 | 0.789 | + | 12.159 | 3.379 | 43.754 |
| cigsonly | 1 | -0.175 | 0.663 | N.S. | 1.126 | 0.221 | 5.743 |
| **Unexposed group** |  |  |  |  |  |  |  |
| nev any | 7 | Aliased |  |  | 5.758 | 3.400 | 9.753 |
| nev cig | 15 | -2.789 | 0.775 | -- | 0.354 | 0.097 | 1.290 |
| **Emp subtype** |  |  |  |  |  |  |  |
| mort | 5 | Aliased |  |  | 0.580 | 0.162 | 2.072 |
| other | 17 | 1.819 | 0.661 | + | 3.575 | 2.655 | 4.816 |
| **Midpoint age in RR** |  | 0.008 | 0.020 | N.S. | 1.613 | 0.099 | 26.192 |
| **RR adjusted for age** |  |  |  |  |  |  |  |
| Yes | 13 | Aliased |  |  | 3.034 | 2.548 | 3.613 |
| No | 9 | -1.476 | 0.462 | - | 0.694 | 0.263 | 1.830 |
| **RR adjusted for factor other than sex, age** |  |  |  |  |  |  |  |
| Yes | 6 | Aliased |  |  | 2.826 | 1.042 | 7.667 |
| No | 16 | -0.246 | 1.325 | N.S. | 2.210 | 0.275 | 17.738 |
| **Derivation of RR/CI** |  |  |  |  |  |  |  |
| Orig/2x2 | 6 | Aliased |  |  | 1.636 | 0.720 | 3.716 |
| Other | 16 | 0.544 | 0.407 | N.S. | 2.818 | 2.330 | 3.408 |
|  |  | **Deviance** | **(DF)** | **Drop Dev** | **P** |  |  |
| **Adding Analysis type** |  | 12.839 | (8) | 0.110 | N.S. |  |  |
|  |  | **Estimate** | **S.E.** | **P** | **RR** | **95%CIl** | **95%CIu** |
| **Constant** |  | 2.513 | 1.519 | N.S. | 12.340 | 0.628 | 242.464 |
| **Sex(RR)** |  |  |  |  |  |  |  |
| Male | 13 | Aliased |  |  | 16.327 | 1.651 | 161.465 |
| Female | 5 | -0.262 | 0.265 | N.S. | 12.569 | 1.232 | 128.225 |
| Combined | 4 | -2.680 | 1.361 | (-) | 1.120 | 0.371 | 3.380 |
| **Continent** |  |  |  |  |  |  |  |
| NAmer | 10 | Aliased |  |  | 7.103 | 4.214 | 11.971 |
| Europe | 7 | -1.406 | 0.309 | -- | 1.741 | 1.212 | 2.501 |
| Asia | 5 | -0.579 | 0.491 | N.S. | 3.979 | 1.127 | 14.043 |
| **Asthma analysis type (Emp)** |  |  |  |  |  |  |  |
| inc-irresp | 19 | Aliased |  |  | 2.608 | 2.237 | 3.040 |
| excl-all | 3 | 0.053 | 0.822 | N.S. | 2.750 | 0.371 | 20.364 |
| **Smoking product (3)** |  |  |  |  |  |  |  |
| any | 5 | Aliased |  |  | 1.330 | 0.694 | 2.550 |
| cigs | 16 | 2.232 | 0.843 | + | 12.401 | 2.859 | 53.798 |
| cigsonly | 1 | -0.151 | 0.664 | N.S. | 1.144 | 0.198 | 6.603 |
| **Unexposed group** |  |  |  |  |  |  |  |
| nev any | 7 | Aliased |  |  | 6.093 | 3.482 | 10.661 |
| nev cig | 15 | -2.988 | 0.767 | -- | 0.307 | 0.078 | 1.210 |
| **Emp subtype** |  |  |  |  |  |  |  |
| mort | 5 | Aliased |  |  | 0.513 | 0.056 | 4.682 |
| other | 17 | 1.967 | 1.074 | N.S. | 3.669 | 2.261 | 5.952 |

Table 3 - B - 6

IESCOPD - Meta-regression of current smoking, any product (or cigarettes if all product not available)

Multiple regression of data from Table 3 - B - 1 (most-adjusted RRs)

Any emphysema

Test by adding extra variables one at a time

|  |  | **Estimate** | **S.E.** | **P** | **RR** | **95%CIl** | **95%CIu** |
| --- | --- | --- | --- | --- | --- | --- | --- |
| **Midpoint age in RR** |  | 0.003 | 0.020 | N.S. | 2.167 | 0.109 | 42.887 |
| **RR adjusted for age** |  |  |  |  |  |  |  |
| Yes | 13 | Aliased |  |  | 3.120 | 2.598 | 3.746 |
| No | 9 | -1.750 | 0.433 | -- | 0.542 | 0.204 | 1.441 |
| **RR adjusted for factor other than sex, age** |  |  |  |  |  |  |  |
| Yes | 6 | Aliased |  |  | 3.267 | 1.143 | 9.341 |
| No | 16 | -0.696 | 1.300 | N.S. | 1.630 | 0.182 | 14.585 |
| **Analysis type** |  |  |  |  |  |  |  |
| prevlnce | 14 | Aliased |  |  | 2.512 | 1.816 | 3.474 |
| onset | 8 | 0.182 | 0.549 | N.S. | 3.014 | 1.020 | 8.905 |

Table 3 - B - 6

IESCOPD - Meta-regression of current smoking, any product (or cigarettes if all product not available)

Multiple regression of data from Table 3 - B - 1 (most-adjusted RRs)

Any emphysema

Fitted values and residuals

|  | **Study Ref** | **NRR** | **SEX** | **LOGRR** | **FITVAL** | **SEFITV** | **STDRES** |
| --- | --- | --- | --- | --- | --- | --- | --- |
|  |  |  |  |  |  |  |  |
| #3555 | WEN | 18 | m | 0.113 | 0.877 | 0.562 | -1.358 |
| #3063 | HUHTI1 | 142 | f | -0.650 | 0.469 | 1.065 | -1.052 |
| #2868 | HARDIE | 16 | f | 1.278 | 1.610 | 0.534 | -0.621 |
| #2628 | WEISS | 14 | m | 1.616 | 2.139 | 1.100 | -0.476 |
| #1734 | ENRIGH | 10 | f | 1.123 | 1.281 | 0.363 | -0.435 |
| #2182 | HIRAYA | 1 | m | 0.798 | 0.880 | 0.302 | -0.274 |
| #1691 | DONTA2 | 11 | m | 0.144 | 0.258 | 0.598 | -0.191 |
| #1993 | MILLER | 7 | m | 1.975 | 2.152 | 1.434 | -0.123 |
| #1524 | LAVECC | 28 | b | 0.565 | 0.572 | 0.090 | -0.076 |
| #336 | OMORI | 4 | m | 2.674 | 2.692 | 0.583 | -0.031 |
| #3603 | BEST | 18 | m | 2.044 | 2.044 | 0.575 | 0.000 |
| #1513 | AUERBA | 2 | m | 6.193 | 6.193 | 0.464 | 0.000 |
| #2847 | LEBOWI | 34 | b | 1.340 | 1.335 | 0.609 | 0.008 |
| #3243 | KAHN2 | 46 | m | 2.207 | 2.172 | 0.283 | 0.124 |
| #1996 | MILLER | 10 | f | 2.205 | 1.888 | 1.897 | 0.167 |
| #3574 | SILVA | 7 | b | 1.411 | 1.341 | 0.409 | 0.170 |
| #1731 | ENRIGH | 7 | m | 1.624 | 1.545 | 0.337 | 0.234 |
| #2924 | HO | 9 | b | 0.833 | 0.698 | 0.512 | 0.262 |
| #2644 | VIKGRE | 1 | m | 1.185 | 0.273 | 1.520 | 0.600 |
| #2865 | HARDIE | 13 | m | 2.711 | 1.873 | 0.816 | 1.027 |
| #2183 | HIRAYA | 2 | f | 1.001 | 0.616 | 0.361 | 1.065 |
| #2957 | HUHTI1 | 67 | m | 2.946 | 0.733 | 1.043 | 2.121 |
