# Supplementary material for: Systematic review with meta-analysis of the epidemiological evidence relating smoking to COPD, chronic bronchitis and emphysema
Source: BMC Pulm Med. 2011 Jun 14;11:36. doi: 10.1186/1471-2466-11-36 (PMC3128042; doi:10.1186/1471-2466-11-36)
Supplement: Additional file 13 — DoseNotMetaData. .DOC file summarizing the results of the dose-related data that could not be included in the dose-related meta-analyses. [file 1471-2466-11-36-S13.DOC]

**Systematic review with meta-analysis of the epidemiological evidence relating smoking to COPD, chronic bronchitis and emphysema**

Barbara A Forey, Alison J Thornton and Peter N Lee

**Additional File 13 : DoseNotMeta**

Contents

[Introduction 2](#__RefHeading___Toc275851190)

[Table 1E COPD – amount smoked 3](#__RefHeading___Toc275851191)

[Table 1F COPD – Age started 5](#__RefHeading___Toc275851192)

[Table 1G COPD, Pack years 6](#__RefHeading___Toc275851193)

[Table 1H COPD – Duration of smoking 9](#__RefHeading___Toc275851194)

[Table 1I COPD, Years since quit 10](#__RefHeading___Toc275851195)

[Table 2E CB, Amount smoked 11](#__RefHeading___Toc275851196)

[Table 2F CB, Age started 13](#__RefHeading___Toc275851197)

[Table 2G CB, Pack years 14](#__RefHeading___Toc275851198)

[Table 2H CB, Duration of smoking 15](#__RefHeading___Toc275851199)

[Table 2I CB, Years since quit 15](#__RefHeading___Toc275851200)

[Table 3E Emphysema, amount smoked 16](#__RefHeading___Toc275851201)

[Table 3F Emphysema, age start – no results 17](#__RefHeading___Toc275851202)

[Table 3G Emphysema, pack years 17](#__RefHeading___Toc275851203)

[Table 3H Emphysema, duration – no results 17](#__RefHeading___Toc275851204)

[Table 3I Emphysema, years quit 17](#__RefHeading___Toc275851205)

## Introduction

This file summarises the results for dose-related risk which were not included in the meta-analyses of the categorical data described in Additional File 1.

Each table consists of the following sections as required:

- Results presented from a multiple logistic regression analysis (MLR), or similar.
- Results presented as comparisons of mean or median exposure levels between the cases and non-cases, or comparison of mean disease severity grade by exposure level.
- Categorical data qualifying for meta-analysis except that the RR/CI were missing or incomplete.
- Categorical data not qualifying for the meta-analysis because the set of categories was not complete.
- Categorical data not qualifying for meta-analysis because the base (comparison) group was unsuitable, or for another reason.

## Table 1E COPD – amount smoked

| Type of analysis | Study | Sex | Smoking status, product | Adjust-ment | Results |
| --- | --- | --- | --- | --- | --- |
| **Multiple regression** | | | | | |
|  | DETORR | b | ever cigarettes | 3 | Not significant in MLR (p>0.05) with pack-years included in the model |
| **Comparison of means or medians between cases and non-cases** | | | | | |
|  | AMIGO | m | ever cigarettes | 0 | Median (quartiles) was 20 (10-30) in cases and 5 (1-20) in controls |
|  | AMIGO | f | ever cigarettes | 0 | Median (quartiles) was 10 (6-20) in cases and 4 (2-10) in controls |
|  | MUELLE | f | current cigarettes | 0 | As there are only three smoking cases, all in the 15-24 cigarette category, it is not possible to estimate dose-response RRs for females |
|  | SHAHAB | b | current cigarettes | 0 | Mean (SD) cigarettes/day was 16.3 (10.4) in cases and 14.8 (9.5) in non-cases, 0.01 < p <0.02 |
|  | TODD | f | current cigarettes only | 0 | There was only one female CNSLD death in a current cigarette only smoker, and that was in the <10 cigs per day category |
| **Categorical data qualifying for meta-analysis except RR/CIs missing/incomplete** | | | | | |
|  | HAWTHO | m | current cigarettes vs 6-17 cigarettes | 1 | RR 0.63 for 18-22 and 0.53 for 23+ (no CIs). Stated to be unreliable because based on <5 deaths in the 23+ cigs/day category. Not significant in overall chisq test (3 levels). Smokers of <5 cigs per day excluded. |
|  | KAHN | m | current cigarettes vs never | 2 | 4.84 for 1-9 11.23 for 10-20 17.45 for 21-39 21.98 for 40+ Refers to 16 year follow-up only |
|  | KULLER | m | current cigarettes vs 1-15 | 0 | 4.5 for 16-25 4.0 for 26-35 4.3 for 36-45 1.1 for 46+ (estimated as ratios of rates read from graph) |
| **Categorical data, but not qualifying for meta-analysis (incomplete categories)** | | | | | |
|  | KHOURY | b | current cigarettes – 20+ vs never cigarettes | 9 | 4.06 (2.67-6.17) |
|  | NIEPSU | b | cigarettes - 21+ vs never cigarettes | 0 | 5.06 (1.84-13.88) (Not clear whether this refers to current or ever smokers) |
|  | NIHLEN | b | current, any product - 15+ vs never | 0 | 5.70 (no CI) |
| **Table 1E (continued)** | | | | | |
| **Categorical data, but not qualifying for meta-analysis (non-qualifying base)** | | | | | |
|  | KULLER | m | current cigarettes vs non cigarettes | 0 | RR 1.2 for 1-15 5.4 for 16-25 4.9 for 26-35 5.2 for 36-45 1.3 for 46+ (estimated as ratios of rates read from graph) |
|  | LIU | m | current cigarettes vs non cigarettes | 3 | 1.30 (1.24-1.37) for 1-19 1.64 (1.54-1.75) for 20 2.26 (1.95-2.61) for 21+ Refers to current smokers in 6-8 years before death. It is stated that there were few ex-smokers in China at that time, so that the base for comparison is close to never smoking |

## Table 1F COPD – Age started

| Type of analysis | Study | Sex | Smoking status, product | Adjust-ment | Results |
| --- | --- | --- | --- | --- | --- |
| **Multiple regression** | | | | | |
|  | DETORR | b | ever cigarettes | 3 | Not significant in MLR (p>0.05) with pack-years included in the model |
|  | JAENDI | b | ever cigarettes | 0 | 0.95 (0.89-1.00) |
|  | JAENDI | b | ever cigarettes | 6 | 1.02 (0.95-1.09) |
| **Comparison of mean or median age started between cases and non-cases** | | | | | |
|  | AMIGO | m | ever | 0 | Median (quartiles) was 17 (14-20) in cases and 17 (15-20) in controls |
|  | AMIGO | f | ever | 0 | Median (quartiles) was 17 (13-21) in cases and 20 (15-25) in controls |
|  | TODD | f | current cigarettes only | 0 | There was only one female CNSLD death in a current cigarette smoker, and that was in the started at age 20+ category |
| **Categorical data, but not qualifying for meta-analysis (RR/CIs missing)** | | | | | |
|  | HAWTHO | m | current cigarettes vs started >20 | 3 | RR 3.33 for 15-19 and 3.42 for ≤14 (no CIs). Not significant in overall chisq test (3 levels). Smokers of <5 cigs per day excluded, adjustment includes number of cigarettes.. |
| **Categorical data, but not qualifying for meta-analysis (incomplete categories)** | | | | | |
|  |  |  |  |  | Note that results for CHEN3 were included in meta-analysis, although they exclude subjects who started before age 13 or after age 22, and include ex-smokers who gave up <5 years ago |
| **Categorical data, but not qualifying for meta-analysis (non-qualifying base)** | | | | | |
|  | LIU1 | m | current cigarettes vs non cigarettes | 3 | 1.31 (1.21-1.42) for 25+ 1.49 (1.41-1.58) for 20-24 1.81 (1.69-1.94) for ≤19 Refers to current smokers in 6-8 years before death. It is stated that there were few ex-smokers in China at that time, so that the base for comparison is close to never smoking |

## Table 1G COPD, Pack years

| Type of analysis | Study | Sex | Smoking status, product | | | Adjust-ment | | Results |
| --- | --- | --- | --- | --- | --- | --- | --- | --- |
| **Multiple regression** | | | | | | | | |
|  | BEDNAR | b | Ever, any product | | | 3 | | Significant (p<0.02) |
|  | DETORR | b | Ever, cigarettes | | | 2 | | 1.02 (1.01-1.03 |
|  | JAENDI | b | Ever, cigarettes | | | 0 | | 1.06 (1.04-1.07) |
|  | JAENDI | b | Ever, cigarettes | | | 6 | | 1.05 (1.02-1.06) |
|  | TAGER | b | Ever, cigarettes | | | 0 | | In a MLR, the regression coefficient (SE) was given as 2.323 (1.065) per log10(LTC), where LTC is lifetime cigarette consumption. This is equivalent to RR (CI) of 10.21 (1.27-82.30). (Equivalence between log10(LTC) and pack years would be 4.9 =10pkyrs, 5.6 =50pkyrs, and 5.9 =100pkyrs) |
|  | VIEGEI2 | m | Ever, cigarettes | | | 6 | | 1.03 (1.02-1.04) |
|  | VIEGEI2 | f | Ever, cigarettes | | | 6 | | 1.04 (1.01-1.07) |
|  |  |  |  | | |  | | Note re VIEGI2 results, M and F: OR/CI as given originally was described as being for pack-years expressed in decades (i.e. per 10 pack-years), and we have converted to per pack-year based on this definition. However elsewhere in the paper, it is stated that pack-years (cigs x years/20) was multiplied by 10, which would imply that result was originally per tenth of a pack-year |
| **Comparison of mean or median pack-years between cases and non-cases** | | | | | | | | |
|  | BROGGE | b | Ever, cigarettes | | | 0 | | Mean (SD) pack years was 28.8 (15.1) for cases and 19.1 (13.4) for controls, p<0.001, smokers of <2.5 pack-years excluded |
|  | COCCI | b | Ever, cigarettes | | | 0 | | Mean (SD; range) pack-years was 41 (24; 5-110) for cases and 12 (11; 3-40) for controls |
|  | ITABAS | b | Ever, cigarettes | | | 0 | | Brinkman's Index (cigs/day x years) was 649 +/- 108 in COPD cases and 421 +/- 130 in normal subjects, and was stated not to differ significantly (p>0.10) in a test between 3 groups (COPD, normal and bronchial asthma) |
|  | MADOR | m | Ever, cigarettes | | | 0 | | Mean pack-years (SE) were 77 (11) for severe COPD, 59 (9) for mild-moderate COPD and 23 (7) for controls. Difference between each COPD group and control is significant |
|  | MARAN1 | b | Ever, cigarettes | | | 0 | | Mean (SD) pack-years was 32.8 (26.4) for COPD cases and 24.7 (23.8) for non-COPD subjects, stated to be p<0.001 with an unpaired t-test |
|  | MARAN2 | b | Ever, cigarettes | | | 0 | | Mean (SD) pack-years was 26.9 (27.4) for COPD cases and 24.1 (22.1) for non-COPD subjects, stated to be p=0.388 on an unpaired t-test |
| **Table 1G (continued)** | | | | | | | | |
|  | MATHES | b | Current, cigarettes | | | 0 | | Median (interquartile range) was 36.0 (18.5, 42.5) for cases and 32.5 (6.3, 36) for controls, p=0.02 |
|  | MATHES | b | Ex, cigarettes | | | 0 | | Pack-years was higher in cases than controls, p=0.0001 |
|  | MATHES | b | Ever, cigarettes | | | 0 | | Mean (inter-quartile range) was 27.3 (10.3, 44.3) in cases and 9.60 (4.2, 16.2) in controls, p<0.0001 |
|  | SARGEA | m | Ever, cigarettes | | | 0 | | Median (interquartile range) of pack-years was 20 (10-35) in self-reported OAD cases, 30 (17.5-40) in undiagnosed OAD cases and 20 (10-30) in subjects without OAD |
|  | SARGEA | f | | Ever, cigarettes | 0 | | | Median (interquartile range) of pack-years was 17.5 (7.5-25) in self-reported OAD cases, 22.5 (12.5-30.8) in undiagnosed OAD cases and 11.5 (5-20.8) in subjects without OAD |
|  | TSUSHI | b | | Current, cigarettes | 0 | | | Mean (SD) pack-years was 38.8 (17.1) in cases and 30.0 (17.0) in non-cases |
|  | TSUSHI | b | | Ex, cigarettes | 0 | | | Mean (SD) pack-years was 40.9 (18.6) in cases and 22.1 (18.0) in non-cases |
|  | ZIETKO | b | | Current, cigarettes | 0 | | | Mean (SD) pack years was 36 (13) in COPD cases and 20 (7) in controls, p<0.0001, (but note that mean (SD) age was 60.2 (9.4) in COPD cases and 53.2 (8.8) in controls |
|  | ZIETKO | b | | Ex, cigarettes | 0 | | | Mean (SD) pack years was 28 (12) in COPD cases, and there were no ex-smoking controls for comparison |
| **Categorical data qualifying for meta-analysis except RR/CIs missing/incomplete** | | | | | | | | |
|  | LIU2 | b | | current cigarettes vs 1-9 cigarettes | 0 | | | 0.83 for 10-20 1.14 for 21+ (estimated as ratios of rates read from graph). Levels were defined as 10-20 and >=20, so it is unclear in which level 20 should be included |
| **Categorical data, but not qualifying for meta-analysis (incomplete categories)** | | | | | | | | |
|  | AMIGO | m | | Ever cigarettes – 20+ pack-years vs never cigarettes | | | 1 | 18.01 (4.22-76.83) |
|  |  |  | |  |  |  | 6 | 14.97 (3.02-74.10) |
|  |  | f | | Ever cigarettes – 10+ pack-years vs never cigarettes | | | 1 | 11.40 (3.90-32.80) |
|  |  |  | |  |  |  | 6 | 13.65 (4.20-44.90) |
| **Table 1G (continued /2)** | | | | | | | | |
| **Categorical data, but not qualifying for meta-analysis (non-qualifying base or other reason)** | | | | | | | | |
|  | CHENG | b | | Ever cigarettes vs 15-19 pack-years | | | 0 | 1.36 (1.12-1.66) for 20-24py 1.66 (1.34-2.05) for 25-29py 1.53 (1.22-1.93) for 30-34py 2.44 (1.92-3.10) for 35-39py 2.52 (.195-3.26) for 40-44py 3.21 (2.61-3.95) for 45+py |
|  | HEDMAN | b | | Ever cigarettes vs never cigarettes or 1-9 pack-years | | | 1 | 2.19 (1.38-3.47) for 10-29py 3.34 (1.84-6.05) for 30+py |
|  | KHOURY | b | | Current cigarettes vs never | | | 9 | 2.46 (1.51-4.02) for <30py vs never,  6.34 (4.03-9.97) for >30py vs never, and 2.61 (1.72-3.94) for >30py vs <30py,  these results being restricted to current smokers of 20+ cigarettes per day |
|  | | | | | | | | |
|  | KOTAN2 | m | | Ever cigarettes vs never cigarettes or 1-20 pack-years | | | 0 | 4.26 (2.03-8.92) for 21-40py 25.06 (10.13-61.98 for 41+py |
|  |  | f | |  |  |  | 0 | 6.35 (1.59-25.35) for 21-40py 29.64 (1.74-505.31) for 41+py |
|  | NIEPSU | b | | Ever cigarettes – 41+ pack-years vs never cigarettes | | | 0 | 8.50 (3.62-19.96) |
|  | WATSON | b | | Ever cigarettes vs 10-30 pack-years | | | 0 | 1.54 (0.87-2.73) for 31-50py 1.82 (0.99-3.37) for 51+py  (recalculated from 2×2 tables, result given by original author for 51+py was significant p<0.05) |
|  |  |  | |  |  |  | 3 | 1.73 (0.86-3.45) for 31-50py 1.29 (0.62-2.72) for 51+py |

## Table 1H COPD – Duration of smoking

| Type of analysis | Study | Sex | Smoking status, product | Adjust-ment | Results |
| --- | --- | --- | --- | --- | --- |
| **Multiple regression** | | | | | |
|  | DETORR | b | ever cigarettes | 3 | Not significant in MLR (p>0.05) with pack-years included in the model |
| **Comparison of means or medians between cases and non-cases** | | | | | |
|  | AMIGO | m | ever cigarettes | 0 | Median (quartiles) was 43 (35-52) in cases and 36 (25-47) in controls |
|  | AMIGO | f | ever cigarettes | 0 | Median (quartiles) was 40 (31-53) in cases and 26 (15-41) in controls |
|  | VESTBO | b | ever any product | 0 | Mean duration of smoking (years) was 26.9 for no COPD and 30.1 for GOLD 0 (implying 27.1 for none/0 combined), and 33.2, 34.4, 38.6 for stages 1-3 respectively (implying 34.1 combined) |
|  | ZIETKO | b | current cigarettes | 0 | Mean (SD) years smoking was 32 (14) in COPD cases and 22 (11) in controls (but note that mean (SD) age was 60.2 (9.4) in cases and 53.2 (8.8) in controls) |

## Table 1I COPD, Years since quit

| Type of analysis | Study | Sex | Smoking status, product | Adjust-ment | Results |
| --- | --- | --- | --- | --- | --- |
| **Comparison of means or medians between cases and non-cases** | | | | | |
|  | ZIETKO | b | ex cigarettes | 0 | Mean (SD) years since quit was 9 (5) in COPD cases, and there were no ex-smoking controls for comparison |
| **Categorical data, but not qualifying for meta-analysis (incomplete categories)** | | | | | |
|  | VINEIS | b | ex any product | 0 | 6.68 (2.32-19.24) for 10+ vs never |
|  | WALD | m | ex cigarettes only | 0 | 4.46 (0.28-71.32) for 21+ vs never any or 0.14 (0.02-1.06) vs current cigarettes. The authors omitted the age-adjusted results for this smoking group because they considered there were too few deaths to give reliable estimates |

## Table 2E CB, Amount smoked

| Type of analysis | Study | Sex | Smoking status, product | Adjust-ment | Results |
| --- | --- | --- | --- | --- | --- |
| **Multiple regression** | | | | | |
|  | SAWICK | m | current any product | 0 | Regression coefficient given as 0.0234. Adjustment is for duration of smoking. Includes 1g pipe tobacco = 1 cigarette |
|  | SAWICK | m | current | 1 | Regression coefficient given as 0.0185. Adjustment is for duration of smoking. Includes 1g pipe tobacco = 1 cigarette |
|  | SAWICK | f | current | 0 | Regression coefficient given as 0.0373. Adjustment is for duration of smoking. Includes 1g pipe tobacco = 1 cigarette |
|  | SAWICK | f | current | 1 | Regression coefficient given as 0.0293. Adjustment is for duration of smoking. Includes 1g pipe tobacco = 1 cigarette |
| **Comparison of means or medians between cases and non-cases** | | | | | |
|  | FINKLE | m | current cigarette | 0 | Mean prevalence of chronic bronchitis was 8.0% in smokers of <1/2 pack/day, 10.5% in smokers of 1/2 to 1 pack/day, and 28.1% in smokers of >1 pack/day |
|  | HIGGI3 | f | current any product | 0 | There was only one female current smoker, who smoked 1-14 g/day |
|  | JOUSI1 | m | current | 0 | Mean number of cigarettes smoked per day averaged 15.40 in disease-free group, compared to 18.60 in subjects with grade two symptoms |
|  | JOUSI1 | f | current | 0 | Mean number of cigarettes smoked per day averaged 9.73 in disease-free group, compared to 12.10 in subjects with grade two symptoms |
|  | MELLST | m | ever any product | 0 | Risk of chronic bronchitis increased monotonically with the amount of tobacco consumed. Products were converted to grams per day as follows: 1 cigarette/1g pipe tobacco = 1g; 1 cigarillo = 2g; 1 cigar = 5g |
|  | OSWAL1 | b | current cigarette | 0 | Mean daily number of cigarettes smoked was 18.2 for cases and 16.7 for controls |
|  | SUADIC | m | current any product | 0 | Mean (SD) amount smoked per day was 17.2 (8.0) in chronic bronchitis cases and 14.9 (7.8) in disease-free subjects, counting 1 cigarette as 1g, 1 cheroot as 3g and 1 cigar as 4g |
| **Categorical data qualifying for meta-analysis except RR/CIs missing/incomplete** | | | | | |
|  | HAWTHO | m | current cigarettes vs 6-17 cigarettes | 1 | RR 1.08 for 18-22 and 1.52 for 23+ (no CIs). Significant (p < 0.001) in overall chisq test (3 levels). Smokers of <5 cigs per day excluded. |
| **Table 2E (continued)** | | | | | |
|  | HAWTHO | f | current cigarettes vs 6-17 cigarettes | 1 | RR 1.46 for 18-22 and 3.45 for 23+ (no CIs). Significant (p < 0.001) in overall chisq test (3 levels). Smokers of <5 cigs per day excluded. |
| **Categorical data, but not qualifying for meta-analysis (incomplete categories)** | | | | | |
|  | DOLL2 | f | current any product vs 1-14 cigarettes | 1 | 5.40 for 15-24 cigarettes. There was only one mixed smoker and no pure pipe/cigar smokers. Refers to 11 year follow-up only. There were no CB deaths in smokers of 25+ |
| **Categorical data, but not qualifying for meta-analysis (non-qualifying base or other reason)** | | | | | |
|  | FLETCH | m | current any product vs non | 0 | 2.10 (0.78-5.62) for 1-14 2.62 (0.91-7.66) for 15+ Counting 1 cigarette as 1g tobacco |
|  | FLETCH | f | current any product vs non | 0 | 1.63 (0.75-3.56) for 1-14 4.67 (1.71-12.72) for 15+  Counting 1 cigarette as 1g tobacco |
|  | HRUBEC | m | current cigarettes vs non | 0 | 1.85 (0.99-3.48) for 1-19 8.17 (5.54-12.04) for 20+ Includes ex-smokers who gave up less than 3 years ago |
|  | HRUBEC | m | current cigarettes vs non | 1 | 1.94 (1.00-3.76) for 1-19 7.90 (5.31-11.74) for 20+  Includes ex-smokers who gave up less than 3 years ago |
|  | RIMING | m | current cigarettes only vs non cigarettes | 0 | 1.82 (1.50-2.20) for 1-9 2.21 (1.95-2.49) for 10-19 3.08 (2.77-3.42) for 20+ |
|  | RIMING | m | current cigarettes only vs non cigarettes | 1 | 1.48 (1.22-1.78) for 1-9 1.94 (1.72-2.18) for 10-19 2.81 (2.54-3.11) for 20+ |
|  | SHARP | m | current cigarettes vs non cigarettes | 1 | 2.15 (1.34-3.47) for 1-19 4.08 (2.64-6.29) for 20+ |
|  | WIG | m | current any product vs non | 0 | 2.62 (0.75-9.19) for 1-9 4.69 (1.55-14.22) for 10 19.02 (7.09-51.03) for 11+ Light exposure was classified by author as <10 cigarettes, <15 bidis or hookah <5 times per day, while heavy exposure was classified as >10 cigarettes, >15 bidis or hookah >5 times per day. Presumably medium exposure consisted of precisely 10 cigarettes, 15 bidis or hookah 5 times per day, although this was not specified and appears unlikely |
|  | WIG | m | current any product vs non | 1 | 3.25 (0.90-11.77) for 1-9 3.83 (1.25-11.67) for 10 15.17 (5.64-40.82) for 11+ (see note in row above) |

## Table 2F CB, Age started

| Type of analysis | Study | Sex | Smoking status, product | Adjust-ment | Results |
| --- | --- | --- | --- | --- | --- |
| **Categorical data qualifying for meta-analysis except RR/CIs missing/incomplete** | | | | | |
|  | HAWTHO | m | current cigarettes vs started age 20+ | 3 | 1.34 for 15-19 2.18 for ≤14 Significant (p < 0.001) in overall chisq test (3 levels). Adjustment includes amount smoked. Smokers of <5 cigs per day excluded. |
|  | HAWTHO | f | current cigarettes vs started age 20+ | 3 | 1.72 for 15-19 2.21 for ≤14 Significant (p < 0.001) in overall chisq test (3 levels). Adjustment includes amount smoked. Smokers of <5 cigs per day excluded. |

## Table 2G CB, Pack years

| Type of analysis | Study | Sex | Smoking status, product | Adjust-ment | Results |
| --- | --- | --- | --- | --- | --- |
| **Multiple regression** | | | | | |
|  | LANGHA | m | ever any product | 2 | 1.015 (1.011-1.020). Categorical data available in a graph, but too small to be read accurately |
|  | LANGHA | f | ever any product | 2 | 1.030 (1.023-1.036). Categorical data available in a graph, but too small to be read accurately |
|  | SCHWAR | b | ever cigarettes | 7 | 1.12 (1.09-1.16) per 10 pack-years of exposure |
| **Comparison of means or medians between cases and non-cases** | | | | | |
|  | JENSEN | b | current any product | 0 | Mean pack-years of consumption in subjects with bronchitis was 47.4 (range 13-76) compared to 42.3 (range 20.3-63) in healthy subjects. Smoking products transformed into 'standard cigarettes' as follows: 1 light filter cigarette = 2/3 standard cigarette; 1 ordinary filter cigarette or 1g pipe tobacco = 1 standard cigarette; 1 unfiltered cigarette = 1 1/3 standard cigarettes; 1 small cigar = 3 standard cigarettes; 1 cigar = 5 standard cigarettes |
|  | MOLLER | b | ever cigarettes | 0 | Mean (SD) pack-years of smoking was 40 (+/-25) in cases compared to 49 (+/-18) in controls |
|  | NEJJAR | m | ever cigarettes | 0 | Mean (+/- SD) pack-years of smoking was 37 (+/-29) in the cases compared to 30 (+/-32) in the controls, p = 0.0001 |
|  | NEJJAR | f | ever cigarettes | 0 | Mean (+/- SD) pack-years of smoking was 27 (+/-49) in the cases compared to 14 (+/-16) in the controls, p = 0.0001 |
| **Categorical data, but not qualifying for meta-analysis (incomplete categories)** | | | | | |
|  | KUBIK | m | current cigarettes vs never cigarettes or up to 0.15 pack years | 0 | 7.40 (6.63-8.25) for 14+. Not clear whether smoking status is ever or current |
|  | KUBIK | f | current cigarettes vs never cigarettes or up to 0.15 pack years | 0 | 8.05 (6.05-10.70) for 14+. Not clear whether smoking status is ever or current |

## Table 2H CB, Duration of smoking

| Type of analysis | Study | Sex | Smoking status, product | Adjust-ment | Results |
| --- | --- | --- | --- | --- | --- |
| **Multiple regression** | | | | | |
|  | SAWICK | m | current any product | 1 | Regression coefficient given as 0.0312. Adjustment is for amount smoked |
|  | SAWICK | f | current any product | 1 | Regression coefficient given as 0.0284. Adjustment is for amount smoked |
| **Comparison of means or medians between cases and non-cases** | | | | | |
|  | HUCHON | b | current any product | 0 | Mean duration of smoking in years was 25.6 (+/- 14.7) in CB cases compared to 22.3 (+/- 13.2) in disease-free |
|  | HUCHON | b | ex any product | 0 | Mean duration of smoking in years was 25.5 (+/- 13.9) in CB cases compared to 19.0 (+/- 12.6) in disease-free |
|  | HUCHON | b | ever any product | 0 | Mean duration of smoking in years was 25.6 (+/- 14.4) in CB cases compared to 20.4 (+/- 12.9) in disease-free |

## Table 2I CB, Years since quit

| Type of analysis | Study | Sex | Smoking status, product | Adjust-ment | Results |
| --- | --- | --- | --- | --- | --- |
| **Categorical data, but not qualifying for meta-analysis (incomplete categories)** | | | | | |
|  | DEAN2 | m | ex any product | 1 | RR/CI for ex-smokers who gave up <= 4 years ago (vs never) was 2.48 (1.52-4.02). No results were presented for quitting >4 years ago as prevalence rates for all symptoms were stated to hardly differ from those of never smokers |
|  | DEAN2 | f | ex any product | 1 | RR/CI for ex-smokers who gave up <= 4 years ago (vs never) was 0.51 (0.16-1.65). No results were presented for quitting >4 years ago as prevalence rates for all symptoms were stated to 'hardly differ' from those of never smokers |
|  | DEAN2 | m | ex cigarettes only | 1 | RR/CI for ex-smokers who gave up <= 4 years ago (vs never) was 2.69 (1.55-4.02). No results were presented for quitting >4 years ago as prevalence rates for all symptoms were stated to hardly differ from those of never smokers |

## Table 3E Emphysema, amount smoked

| Type of analysis | Study | Sex | Smoking status, product | Adjust-ment | Results |
| --- | --- | --- | --- | --- | --- |
| **Comparison of means or medians between cases and non-cases** | | | | | |
|  | AUERBA | f | current cigarettes | 1 | Mean emphysema grade in women was 0.05 in subjects who never smoked regularly, 1.37 in current cigarette smokers of <1 pack per day and 1.70 in current cigarette smokers of 1+ packs per day, standardized for age |
|  | AUERBA | m | ex cigarettes | 1 | Mean emphysema grade in men quitting 10+ years ago was 0.24 for those formerly smoking <1 pack per day and 0.70 for those formerly smoking 1+ packs per day. In men who stopped smoking <10 years ago, mean grades were 1.08 for those formerly smoking <1 pack per day and 1.69 in those formerly smoking 1+ packs per day, after standardising for age |
| **Categorical data qualifying for meta-analysis except RR/CIs missing/incomplete** | | | | | |
|  | HIRAYA | m | current cigarettes vs never cigarettes | 1 | 1.8 for 1-9 2.3 for 10-19 2.5 for 20-29 1.9 for 30+ |
| **Categorical data, but not qualifying for meta-analysis (non-qualifying base or other reason)** | | | | | |
|  | ANDER2 | b | ever cigarettes only | 0 | Results available only for grade 4+ emphysema (which is not the selected emphysema outcome for this study) Numbers too small for reliable estimation of age-adjusted RR/CIs. Unadjusted RR/CIs relative to never smokers are  2.23 (0.19-26.81) for <1pack/day,  9.26 (2.02-42.32) for 1-2 packs/day, and  8.91 (1.44-54.93) for 2+ packs per day.  Relative to <1pack/day, they are  4.16 (0.50-34.68) for 1-2 packs/day, and  4.00 (0.38-41.74) for 2+ packs/day.  Unclear into which group smokers of 20 and 40 cigarettes per day have been put or whether there were any mixed cigarette and pipe/cigar smokers |

## Table 3F Emphysema, age start – no results

## Table 3G Emphysema, pack years

| Type of analysis | Study | Sex | Smoking status, product | Adjust-ment | Results |
| --- | --- | --- | --- | --- | --- |
| **Multiple regression** | | | | | |
|  | WANG2 | b | ever cigarettes | 2 | Smoking index (assumed to be pack-years) was a significant risk factor (p < 0.01) for emphysema |
| **Comparison of means or medians between cases and non-cases** | | | | | |
|  | ANDER2 | b | ever cigarettes only | 0 | The mean grade of emphysema was 2.1 in subjects smoking <40 pack-years and 3.2 in subjects smoking 40+ pack-years, 0.02<p<0.05 |

## Table 3H Emphysema, duration – no results

## Table 3I Emphysema, years quit

| Type of analysis | Study | Sex | Smoking status, product | Adjust-ment | Results |
| --- | --- | --- | --- | --- | --- |
| **Comparison of means or medians between cases and non-cases** | | | | | |
|  | AUERBA | m | ex cigarettes | 1 | Mean emphysema grade in men quitting 10+ years ago was 0.24 for those formerly smoking <1 pack per day and 0.70 for those formerly smoking 1+ packs per day. In men who stopped smoking <10 years ago, mean grades were 1.08 for those formerly smoking <1 pack per day and 1.69 in those formerly smoking 1+ packs per day, after standardising for age |
|  | VIKGRE | m | ex cigarettes | 0 | Incidence of emphysematous lesions was 2 in continuous smokers and 0 in quitters during follow-up, compared to 0 in never smokers |
